# The Distribution of Genes Associated With Regulated Cell Death Is Decoupled From the Mitochondrial Phenotypes Within Unicellular Eukaryotic Hosts

**DOI:** 10.3389/fcell.2020.536389

**Published:** 2020-09-23

**Authors:** Jérôme Teulière, Guillaume Bernard, Eric Bapteste

**Affiliations:** Institut de Systématique, Evolution, Biodiversité (ISYEB), Sorbonne Université, CNRS, Museum National d’Histoire Naturelle, EPHE, Université des Antilles, Paris, France

**Keywords:** regulated cell death, unicellular eukaryotes, mitochondria, mitochondrion-related organelle, apoptosis, autophagy

## Abstract

Genetically regulated cell death (RCD) occurs in all domains of life. In eukaryotes, the evolutionary origin of the mitochondrion and of certain forms of RCD, in particular apoptosis, are thought to coincide, suggesting a central general role for mitochondria in cellular suicide. We tested this mitochondrial centrality hypothesis across a dataset of 67 species of protists, presenting 5 classes of mitochondrial phenotypes, including functional mitochondria, metabolically diversified mitochondria, functionally reduced mitochondria (Mitochondrion Related Organelle or MRO) and even complete absence of mitochondria. We investigated the distribution of genes associated with various forms of RCD. No homologs for described mammalian regulators of regulated necrosis could be identified in our set of 67 unicellular taxa. Protists with MRO and the secondarily a mitochondriate *Monocercomonoides exilis* display heterogeneous reductions of apoptosis gene sets with respect to typical mitochondriate protists. Remarkably, despite the total lack of mitochondria in *M. exilis*, apoptosis-associated genes could still be identified. These same species of protists with MRO and *M. exilis* harbored non-reduced autophagic cell death gene sets. Moreover, transiently multicellular protist taxa appeared enriched in apoptotic and autophagy associated genes compared to free-living protists. This analysis suggests that genes associated with apoptosis in animals and the presence of the mitochondria are significant yet non-essential biological components for RCD in protists. More generally, our results support the hypothesis of a selection for RCD, including both apoptosis and autophagy, as a developmental mechanism linked to multicellularity.

## Introduction

Regulated cell death (RCD) is a central, ancient, and widespread phenotype of life, displayed by prokaryotes and eukaryotes, be they multicellular or unicellular (Ameisen, 2002; Koonin and Aravind, 2002; Van Hautegem et al., 2015; Bidle, 2016; Gonçalves et al., 2017; Peeters and de Jonge, 2018; Durand and Ramsey, 2019). Yet, the origins and evolution of RCD and of its associated molecular mechanisms remain poorly understood. A broader evolutionary explanation for the evolution of RCD, including, but not limited to apoptosis, is still lacking. This is partly because the focus of the research on RCD has indeed largely been restricted to animal apoptosis. When broader taxonomic scales are considered, two main hypotheses are commonly discussed to explain the evolutionary origin of apoptosis. These hypotheses give distinct roles to mitochondria. The “Original Sin” hypothesis proposes that apoptosis emerged as a consequence of the evolution of multicellularity, providing a social control of cell survival (Ameisen, 1996), hence possibly independently from the mitochondrial acquisition. By contrast, the endosymbiotic hypothesis posits that the evolution of apoptosis was a consequence of the mitochondrial acquisition (Ameisen, 1996; Kroemer, 1997; Kaczanowski, 2016), and gives a central role to this organelle in the origin of apoptosis. Moreover, many hypotheses have been proposed to explain why RCD is maintained in unicellular eukaryotes despite its high cost on individual fitness. Thus, kin-selected altruistic death could provide an evolutionary advantage to a population of protists as (i) a mechanism of immunity against viruses, (ii) a response to starvation and stress, (iii) a developmental process in transiently multicellular species, or (iv) as a way to control parasite density in an infected host (Durand and Ramsey, 2019). These hypotheses suggest that a selective pressure exists to drive the evolution of RCD, but they do not specify whether mitochondria are required for various forms of RCD to occur.

Beyond apoptosis, many forms of RCD, such as autophagic cell death, parthanatos and several types of regulated necrosis such as ferroptosis, pyroptosis, necroptosis, or Mitochondrial Permeability Transition (MPT)-driven necrosis have been characterized in metazoans and, for some forms, proposed in some protists (Calvo-Garrido et al., 2010; van Zandbergen et al., 2010; Reece et al., 2011; Galluzzi et al., 2018; Tang et al., 2019). Mitochondria have been consistently implicated in apoptosis, parthanatos and MPT-driven necrosis. In the context of mammalian apoptosis in particular, mitochondria are thought to be central for the integration of cell death signals and the cytoplasmic release of apoptogenic factors (Galluzzi et al., 2018). By contrast, mitochondria are not directly implicated in autophagic cell death or in other forms of regulated necrosis (Galluzzi et al., 2016; Wang et al., 2017). Whether the distribution of genes associated to autophagy is constrained by the presence of mitochondria is therefore an open question, especially because autophagy-associated genes can be involved in cellular maintenance and their expression need not necessarily result in cell death.

Importantly, the current biological connection between the mitochondrial phenotypes of protists and the distribution of genes associated with various forms of RCD can be tested, because these unicellular eukaryotes undergo RCD and exhibit a range of diversified mitochondria. Indeed, whereas many protists harbor “typical” mitochondria, several independent anaerobic protists lineages exhibit various degrees of mitochondrial metabolic diversification and electron-transport chain (ETC) reduction. Despite heterogeneity in metabolic functions associated to these organelles, mitochondrial phenotypes have been classified into five types depending on common characteristics in their energy metabolism: first, fully aerobic mitochondria; second, anaerobic mitochondria (ETC using an endogenous electron acceptor instead of O_2_); third, hydrogen-producing mitochondria (with a reduced ETC); fourth, the more functionally reduced hydrogenosomes (lacking ETC) and mitosomes (only retaining Fe/S cluster assembly function), that retained the mitochondrial membranes but lost the mtDNA (Muller et al., 2012; Roger et al., 2017). The three latter classes of mitochondria-derived organelles are referred to as Mitochondrion-Related Organelles (MRO). Furthermore, in one secondarily amitochondriate lineage, the *Monocercomonoides* spp., mitochondria were entirely lost (Karnkowska et al., 2016), defining a fifth mitochondrial phenotype (i.e., complete secondary loss). Former studies, conducted in protists with MRO before the sequencing of *Monocercomonoides exilis*, started to question the connection between apoptosis-like RCD and the presence of a mitochondrion (Chose et al., 2003; Sarde and Roseto, 2008; Taylor-Brown and Hurd, 2013). These studies concluded that RCD could be performed by mitochondria-independent apoptosis-like death, resulting from extensive mitochondrial gene transfer to the nuclear genome (Taylor-Brown and Hurd, 2013).

Here, we further investigated the presence of characteristic genes associated with various forms of RCD, as defined in multicellular organisms, across a broad taxonomic diversity of mitochondriate protists, protists with MRO and *M. exilis*, with publicly available whole-genome sequencing data.

## Methods

### Proteome Data Mining

Whole-proteome assemblies (Supplementary Table S1) were searched for proteins or domains defined as RCD-associated genes (see Definition of RCD-associated genes). To detect RCD-associated domains in non-homologous proteins and additional remote homologs of RCD genes and domains in protists for each type of RCD, searches were either performed solely by iterative homology search using Diamond Blastp (Buchfink et al., 2015) with an *E*-value <E^–5^, 30% identity and 80% coverage as cutoff thresholds, by homology searches combined with manual parsing of proteome scans for presence or conserved associations of the death-associated PFAM domains (PfamScan^1^ (Mistry et al., 2007)], or solely by domain search. Retrieved protein sequences are available as fasta files (Supplementary Files S1, S2).

### Orthology Assignment

For each putative gene family, maximum likelihood phylogenetic trees were reconstructed. First, multiple alignments were performed on retrieved protein sequences using Clustal Omega with default parameters.^2^ Alignments were cleaned using Trimal with default parameters,^3^ then phylogenetic reconstruction was performed using iqtree^4^ using the following parameters: -s < input alignment > -m MFP (automatic model selection with ModelFinder) -bb 1000 (ultrafast bootstrap). The resulting Newick tree files are available as Supplementary Materials.^5^ Although, in principle, gene trees allow to identify in-paralogs (duplicated homologs within a species), out-paralogs (duplication that predated some speciation events), and orthologs (single copy genes originating from the same ancestral gene copy in a group of species), a single gene family contains too little phylogenetic information to robustly resolve the eukaryotic tree of life. Therefore, the backbone of these trees was statistically weakly resolved (Supplementary File S3).

As an alternative approach, orthology relationships between retrieved sequences were assigned using the recently developed Broccoli pipeline, based on Diamond and FastTree2 (Price et al., 2009, 2010; Buchfink et al., 2015; Derelle et al., 2020). The 67 proteomes listed in Supplementary Table S1 were passed as arguments and Broccoli was run using default parameters. Summarized and detailed results of orthology assignment by Broccoli are available as Supplementary Tables S2, S3, respectively.

### Definition of RCD-Associated Genes

Previous studies have identified common apoptosis regulators in metazoans, plants, fungi and protists, and inferred the existence of an ancestral apoptosis-like pathway in the last eukaryotic common ancestor (van der Biezen and Jones, 1998; Koonin and Aravind, 2002; Leipe et al., 2004; Nedelcu, 2009; Sundström et al., 2009; Klim et al., 2018). From these studies, we defined the following ancestral conserved apoptosis-associated genes:

– putative homologs of Zinnia endonuclease 1 (ZEN1) / Endonuclease G (ENDOG) / Nuclease 1 (NUC1) (PF02265 / PF01223 / PF03265), Apoptosis-Inducing Factor (AIF)/AIF-homologous mitochondrion-associated inducer of death (AMID) (by homology only), HTRA-like protease (PF13365 + PF00595 / PF12812 / PF13180 / PF17815 / PF17820), metacaspase (PF00656), metacaspase substrate Tudor Staphylococcal Nuclease (TSN: PF00565 + PF00567).
– proteins (homologs and non-homologs) containing either the Bax-inhibitor (PF01027), Apoptosis Inhibitor 5 (API5: PF05918), Defender Against Death (DAD: PF02109), IAP repeat, Baculovirus Inhibitor of apoptosis protein Repeat (BIR: PF00653) or Nucleotide-Binding adaptor – APAF-1, R gene products, CED-4 (NB-ARC: PF00931) / NAIP, CIIA, HET-E and TP1 (NACHT: PF05729) domains.

Inputs for AIF/AMID, caspase-family and OMI/HTRA Diamond searches were taken from Klim et al. (2018).

The presence of domains specific for conserved autophagy-associated genes (ATG) that act in the following pathways in yeast and metazoans was also investigated (Anding and Baehrecke, 2015):

– Induction: ATG1 (PF12063), ATG13 (PF10033), ATG101 (PF07855) domains and Tor (PF08771) homologs.
– Cargo selection: ATG11 (PF10377), Beclin/ATG6 (PF04111/PF17675) domains.
– Vesicle expansion: ATG3/10 (PF03986/PF03987/PF10381), ATG4 (PF03416), ATG5 (PF04106), ATG7 (PF16420), ATG8 (PF02991), ATG12 (PF04110) domains.

Homologs of the following described necrosis regulators in mammals were searched using Diamond inputs corresponding to human sequences and the corresponding PFAM domains: RIPK1/3 (PF12721/PF00531), Gasdermin D (PF04598/PF17708), or MLKL (by homology only, no specific PFAM domain).

### Life Strategies and Taxonomic Categories

For each species of protist, life strategies were classified either as parasitic (P), (ecto)symbiotic (S), free-living (F) or transiently multicellular (M). Included in the latter category were protists associating in colonies, aggregating or forming multicellular or coenocytic hyphae.

Taxonomic information was summarized by adding superphylum information for each taxa according to Lax et al. (2018).

### Hierarchical Clustering and Scoring

Presence/absence heatmaps were generated using custom python scripts and hierarchical clustering was performed using the Ward variance minimization algorithm from the scipy library.^6^ For each dataset, the appropriate number of clusters was determined using the R package NbClust (Charrad et al., 2014). Average numbers or genes by groups are indicated in the text +/− standard deviation. The Mann-Whitney U-test (two-tailed) was used to compare groups.

## Results and Discussion

Many forms of RCD have been defined in mammals by the Nomenclature Committee on *Cell Death* (*NCCD*) (Galluzzi et al., 2018). Given that these RCD forms have been discovered and described in the context of metazoan biology, their evolutionary scope and significance is unknown in all other eukaryotic phyla, including unicellular taxa.

### Distribution of Genes Associated With Regulated Necrosis

Although experimental data are sparse on necrosis in protists, rupture of the plasma membrane associated to necrosis had already been reported in *Dictyostelium* (Laporte et al., 2007). Yet, it is unclear whether regulated necrosis generally occurs in unicellular eukaryotes since secondary necrosis can be a consequence of apoptotic death in the absence of phagocytes (Carmona-Gutierrez et al., 2018; Galluzzi et al., 2018). We thus attempted to identify homologs of genes associated with regulated necrosis in 67 species of protists, spanning a diversity of mitochondrial phenotypes. No homologs for described mammalian regulators of necroptosis and pyroptosis could be identified in our set of unicellular taxa (data not shown), indicating that these two RCD pathways are likely exclusive to vertebrates, in agreement with previous studies (Dondelinger et al., 2016). The lack of distinctive genes for ferroptosis precluded us to test the previously formulated hypothesis that this form of RCD may be widely conserved (Distéfano et al., 2017). Similarly, the possibility of conservation of Mitochondrial Permeability Transition (MPT)-driven necrosis could not be unambiguously investigated due to the ubiquity and high duplication of cyclophilins and the uncertainty on the composition of the MPT Pore complex besides Cyclophilin-D (Baines and Gutiérrez-Aguilar, 2018). Therefore, the existence of regulated necrosis in protists remains to be demonstrated.

### Distribution of Genes and Domains Associated With Apoptosis

**FIGURE 1:**
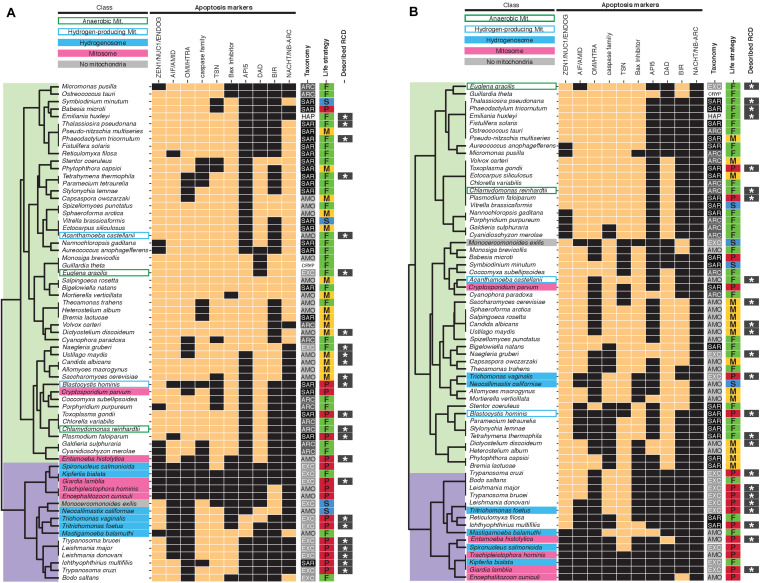
Presence/absence heatmaps of apoptosis-associated genes in protists with diverse mitochondrial phenotypes. Presence is indicated by copper rectangles; absence by black rectangles. Hierarchical clustering of putative homologs **(A)** and orthologs (from the largest ortholog group found by Broccoli for each set of putative homologs retrieved in this study) **(B)** groups protists in two main clusters (overlaid in light green and purple). **(B)** For multiple categories such as ZEN1/NUC1/ENDOG and NACHT/NB_ARC, presence of at least one ortholog is indicated. References to previous reports of RCD (indicated by dark gray rectangles with white stars) in specific taxa are listed in Supplementary Table S1. AMO, Amorphea; SAR, Stramenopiles-Alveolates-Rhizaria; ARC, Archaeplastida; EXC, Excavata; HAP, Haptophyta; CRYP, Cryptista; P, parasitic; S, symbiotic; F, free-living; M, transiently multicellular; Mit., Mitochondria.

Previous studies have identified ancestral conserved apoptosis-associated genes and protein domains in eukaryotes (Koonin and Aravind, 2002; Leipe et al., 2004; Nedelcu, 2009; Sundström et al., 2009; Klim et al., 2018). We performed hierarchical clustering of the species based on the presence/absence of these apoptosis-associated genes (Figure 1A). Two main clusters emerged, separating most protists with hydrogenosomes/mitosomes (with the exception of *Entamoeba* and *Cryptosporidium*) and the mitochondrial *Monocercomonoides*, from typical mitochondriate protists. Protists with diverged mitochondria displayed reduced numbers of apoptosis-associated genes with respect to protists with fully aerobic mitochondria (3.8 +/− 2.8, *n* = 16 versus 6.9 +/− 1.5, *n* = 51, respectively, *P* < 0.001) and protists with functionally reduced MRO in particular displayed the lowest numbers of apoptosis-associated genes (fully aerobic mitochondria: 6.9 +/− 1.5, *n* = 51, anaerobic mitochondria: 8.0 +/− 1.4, *n* = 2, hydrogen-producing mitochondria: 5.5 +/− 3.5, *n* = 2 versus hydrogenosome: 3.3 +/− 2.3, *n* = 6, mitosomes: 2.0 +/− 2.0, *n* = 5). We also performed hierarchical clustering of the species based on the presence/absence of at least one ortholog from the largest ortholog group found by the tool Broccoli (Derelle et al., 2020) for each category of apoptosis-associated genes (Figure 1B). Although this clustering does not separate as clearly protists with functionally diversified or reduced mitochondria from typical mitochondriate protists, similar results are observed when considering the average number of genes by mitochondrial phenotype (fully aerobic mitochondria: 6.0 +/− 1.4, *n* = 51, anaerobic mitochondria: 7.0 +/− 0.7, *n* = 2, hydrogen-producing mitochondria: 5.0 +/− 2.8, *n* = 2 versus hydrogenosome: 3.0 +/− 1.9, *n* = 6, mitosomes: 2.0 +/− 2.1, *n* = 5). This result is compatible with a significant role for mitochondria in apoptosis in protists. Yet, the correlation between the number of apoptosis-associated genes and mitochondrial metabolic diversification or reduction is not strictly linear. Surprisingly, despite the total lack of mitochondria in *Monocercomonoides*, we detected apoptosis-associated genes such as a NUC1 nuclease, two metacaspases and a BIR domain-containing protein. Experimental data are required to test whether *Monocercomonoides* protists are able to undergo RCD in the absence of mitochondria, and whether the predicted apoptosis-associated genes contribute to RCD.

### Distribution of Genes Associated With Autophagy

**FIGURE 2:**
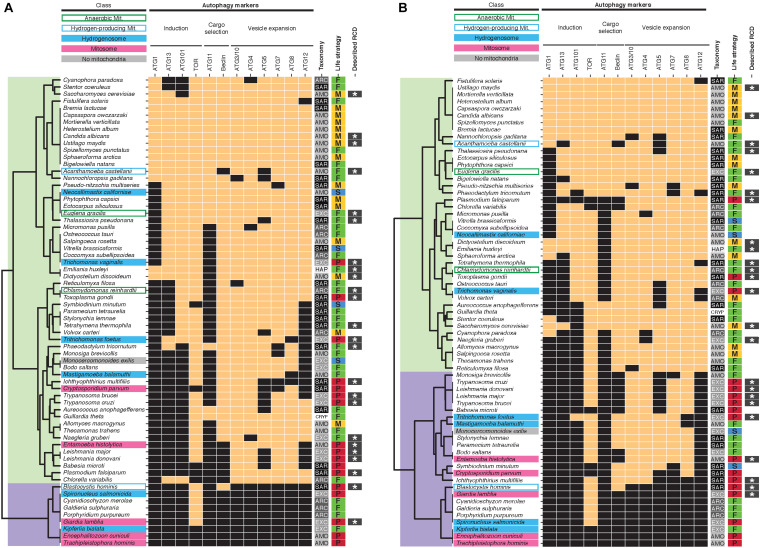
Presence/absence heatmaps of autophagy-associated genes in protists with diverse mitochondrial phenotypes. See Figure 1 legend for the description of symbols and abbreviations. Hierarchical clustering of putative homologs **(A)** and orthologs (from the largest ortholog group found by Broccoli for each set of putative homologs retrieved in this study) **(B)** groups protists in two main clusters (overlaid in light green and purple).

A more controversial form of RCD, autophagic cell death, has been proposed to occur in animals and plants. High levels of autophagy may deplete mitochondria (mitophagy) and possibly lead to a metabolic catastrophe, causing cell death. In this model, autophagic cell death would depend on the maintenance of mitochondria in eukaryotic cells. Alternatively, autophagy could indirectly promote cell death by selective degradation of pro-survival factors (Nelson and Baehrecke, 2014). With regard to unicellular eukaryotes, autophagic-like cell death features have been described in *Dictyostelium* and *Toxoplasma* (Calvo-Garrido et al., 2010; Ghosh et al., 2012) and suspected in *Plasmodium*, *Giardia* and the dinoflagellate *Amphidinium carterae* (Franklin and Berges, 2004; Bagchi et al., 2012; Eickel et al., 2013). This mode of RCD requires autophagic machinery gene sets, which are generally conserved in unicellular eukaryotes except in Rhodophyta (*Porphyridium*, *Cyanidioschyzon*, *Galdieria*), Fornicata (*Spironucleus*, *Kipferlia*, *Giardia*) and microsporidia (*Trachipleistophora*, *Encephalitozoon*) (Rigden et al., 2009; Brennand et al., 2011; Duszenko et al., 2011; Shemi et al., 2015). Consistently, in our analysis these three lineages clustered together as well as with *Blastocystis*, whereas other protists were grouped in a larger cluster (Figure 2A). In contrast to apoptosis-associated genes, autophagy-associated genes did not cluster protists with MRO together, but rather distributed them in two main clusters. Similar results were obtained when clustering identified orthologs found by Broccoli (Figure 2B). Of note, our results differ from previously published results on autophagy gene sets present in *M. exilis*, *T. vaginalis* and *N. gruberi* (Karnkowska et al., 2019). Karnkowska et al. reported ATG1 homologs in *T. vaginalis* and *N. gruberi* by homology searches whereas we did not by using the presence of PFAM domain PF12063 as a criteria. We also did not recover putative ATG1 orthologs for these taxa using Broccoli. Similarly, unlike Karnkowska et al., we did not identify homologs for ATG11 in *T. vaginalis* and *N. gruberi* but have found a putative homolog for ATG13 in *T. vaginalis*, based on the presence of specific PFAM domains. Broccoli did not identify ortholog groups for ATG11 in *T. vaginalis*, *N. gruberi*, and *M. exilis* either but confirmed our findings for ATG13, identifying a small ortholog group (ID 25622) comprised of *T. vaginalis* and *T. foetus* sequences (Figure 2B and Supplementary Table S3). Experimental autophagy assays in these taxa are needed to properly assess the functions of these putative homologs and resolve the discrepancies between our predictions and those of Karnkowska et al.

Since we and others (Karnkowska et al., 2019) also identified a putative autophagic vesicle expansion pathway in *M. exilis*, this analysis indicates that the evolutionary conservation of a canonical autophagy pathway does not depend on the presence of mitochondrial organelles. Thus, autophagic cell death could occur in absence of mitochondria, which could explain the observations of RCD in amitochondriate protists such as *Giardia.* This hypothesis could be tested experimentally by knocking down the conserved autophagy-associated genes we identified and evaluating their requirement during RCD in *Giardia* and other amitochondriate protists.

### Distribution of RCD-Associated Genes Correlates With Protists Lifestyle

The clustering of protist taxa based on shared RCD-associated genes is also decoupled from taxonomy, suggesting diverse convergent evolution of RCD (Figure 1). Whereas RCD has been extensively described in parasites, we detected fewer apoptosis and autophagy-associated genes in parasitic taxa than non-parasitic taxa (apoptosis: 3.8 +/− 2.0, *n* = 17, versus 6.9 +/− 1.8, *n* = 50, respectively, autophagy: 4.6 +/− 3.0, *n* = 17, versus 8.9 +/− 3.0, *n* = 50, respectively, *P* < 0.001 in each case). Similar results were obtained when considering orthologs only (apoptosis: 3.0 +/− 2.1, *n* = 17, versus 6.0 +/− 1.5, *n* = 50, respectively, autophagy: 4.0 +/− 2.6, *n* = 17, versus 8.0 +/− 3.0, *n* = 50, respectively, *P* < 0.001 in each case). It is thus likely that the observed gene reduction is linked to genome simplification in parasites rather than a loss of RCD. In contrast, transiently multicellular taxa tend to be enriched in apoptosis and autophagy-associated genes compared to free-living taxa (apoptosis: 8.0 +/− 1.0, *n* = 15, versus 6.6 +/− 1.9, *n* = 31, respectively, *P* < 0.01, autophagy: 10.9 +/− 1.5, *n* = 15, versus 7.9 +/− 3.2, *n* = 31, respectively, *P* < 0.001), even when considering orthologs only (apoptosis: 6.0 +/− 1.2, *n* = 15, versus 5.0 +/− 1.6, *n* = 31, respectively, *P* < 0.05, autophagy: 11.0 +/− 1.7, *n* = 15, versus 8.0 +/− 3.2, *n* = 31, respectively, *P* < 0.001). This result is consistent with the hypothesis of a selection for RCD as a developmental mechanism linked to the establishment of multicellularity. However, since the reference organisms used to define canonical RCD pathways (*Dictyostelium*, *Saccharomyces*, metazoans) and 75% of transiently multicellular species in our dataset belong to Amorphea (Supplementary Table S1), our analysis may be biased toward conservation of RCD-associated genes in these phyla. Interestingly, another argument for the selection of RCD as a developmental mechanism in transiently multicellular taxa stresses a hidden connection between mitochondrial phenotypes and RCD. None of the transiently multicellular protists harbors divergent mitochondria or MRO, suggesting that mitochondrial metabolic diversification/reduction and the establishment of transient multicellularity may be incompatible processes. However, this incompatibility does not concern the maintenance of animal multicellularity since metazoan multicellulars with diverged mitochondrial organelles as an adaptation to hypoxia have been found. For instance, *Ascaris* nematodes possess anaerobic mitochondria and loriciferans evolved hydrogenosome-like organelles (Danovaro et al., 2010; Muller et al., 2012). Whole-genome sequencing of additional transiently multicellular and free-living protists distant from Amorphea are therefore required to test the hypothesis of a biological connection between RCD-associated genes maintenance and the establishment of transient multicellularity.

## Conclusion

Our test of the conservation of genes associated with RCD depending on the mitochondrial status of their host taxa supports a coupling between the presence of aerobic mitochondria and the conservation of apoptosis-associated genes, in agreement with an endosymbiotic origin of this pathway, and a possibly pleiotropic function of the corresponding genes in aerobiosis (Klim et al., 2018). Indeed, in protists carrying hydrogenosomes, mitosomes or even having lost mitochondria completely, those apoptosis genes tend to be lost, but not entirely. Strikingly, despite various degrees of apoptosis gene loss, RCD, especially if mediated by autophagy, appears maintained in protists, irrespective of their mitochondrial phenotypes. This is logical because RCD is a broader biological phenomenon than apoptosis, likely affected by diverse selective pressures, related to protists lifestyle.

## Data Availability Statement

All datasets generated for this study are included in the article/Supplementary Material.

## Author Contributions

JT and EB designed the study. JT collected, analyzed and interpreted the unicellular eukaryotes proteome data. GB analyzed gene orthology in retrieved gene sets. JT and EB contributed equally in writing the manuscript, read and approved the final manuscript. All authors contributed to the article and approved the submitted version.

## Conflict of Interest

The authors declare that the research was conducted in the absence of any commercial or financial relationships that could be construed as a potential conflict of interest.

## Footnotes

1 https://www.ebi.ac.uk/Tools/pfa/pfamscan/

2 https://www.ebi.ac.uk/Tools/msa/clustalo/

3 http://trimal.cgenomics.org/

4 http://www.iqtree.org/doc/

5 https://github.com/TeamAIRE/Supplementary_Materials_Teuliere_et_al_2020

6 https://www.scipy.org/

## Abbreviations

RCD: regulated cell death
MRO: mitochondrion-related organelle
AMO: Amorphea
SAR: Stramenopiles-Alveolates-Rhizaria
ARC: Archaeplastida
EXC: Excavata
HAP: Haptophyta
CRYP: Cryptista
P: parasitic
S: (ecto)symbiotic
F: free-living
M: transiently multicellular
Mit.: mitochondria.
