# Supplementary material for: The Distribution of Genes Associated With Regulated Cell Death Is Decoupled From the Mitochondrial Phenotypes Within Unicellular Eukaryotic Hosts
Source: Front Cell Dev Biol. 2020 Sep 23;8:536389. doi: 10.3389/fcell.2020.536389 (PMC7539657; doi:10.3389/fcell.2020.536389)
Supplement: Supplementary file 1 [file Data_Sheet_1.PDF]

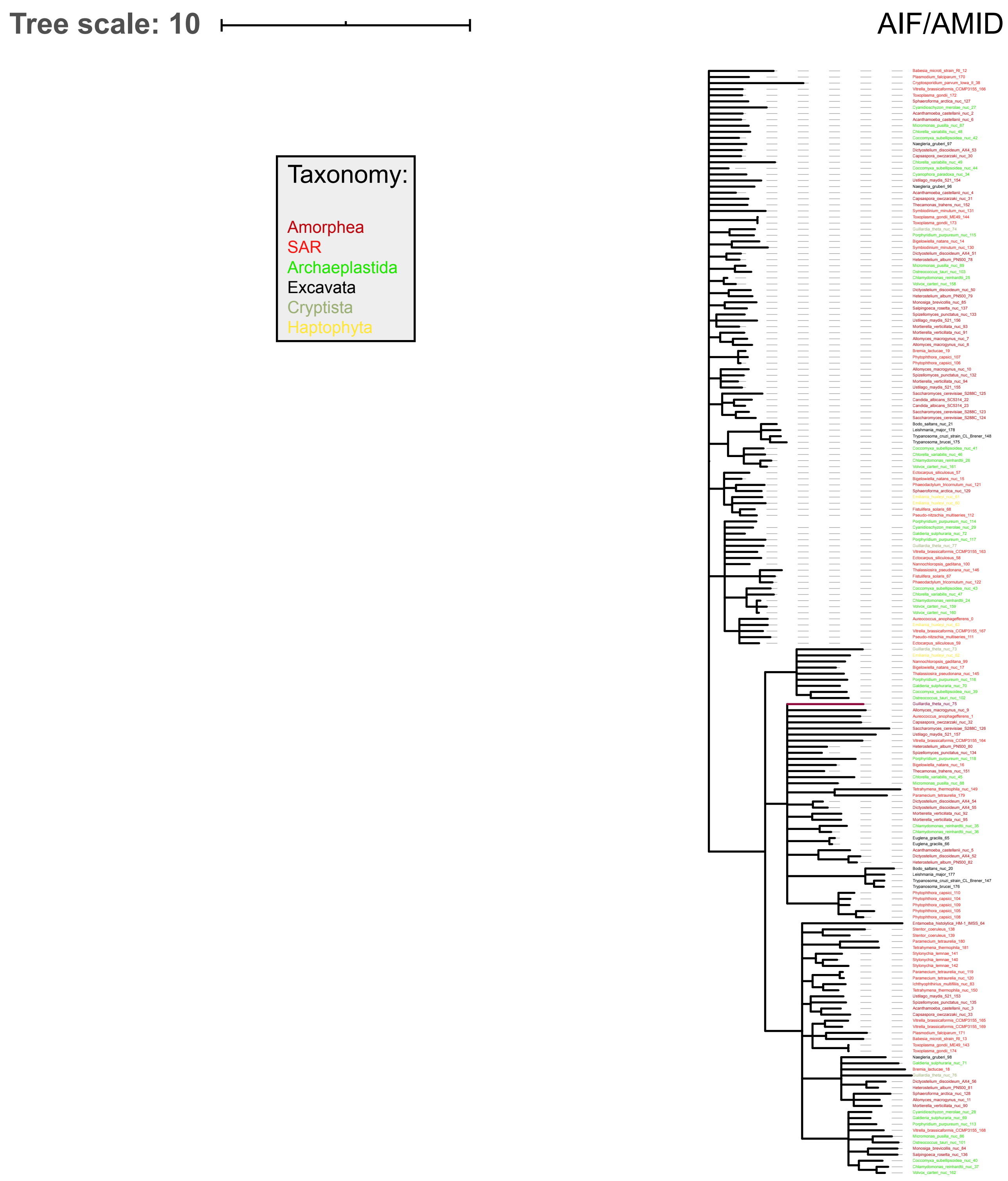
Tree scale: 10
AIF/AMID
Taxonomy:
Amorphea
SAR
Archaeplastida
Excavata
Cryptista
Haptophyta

API5

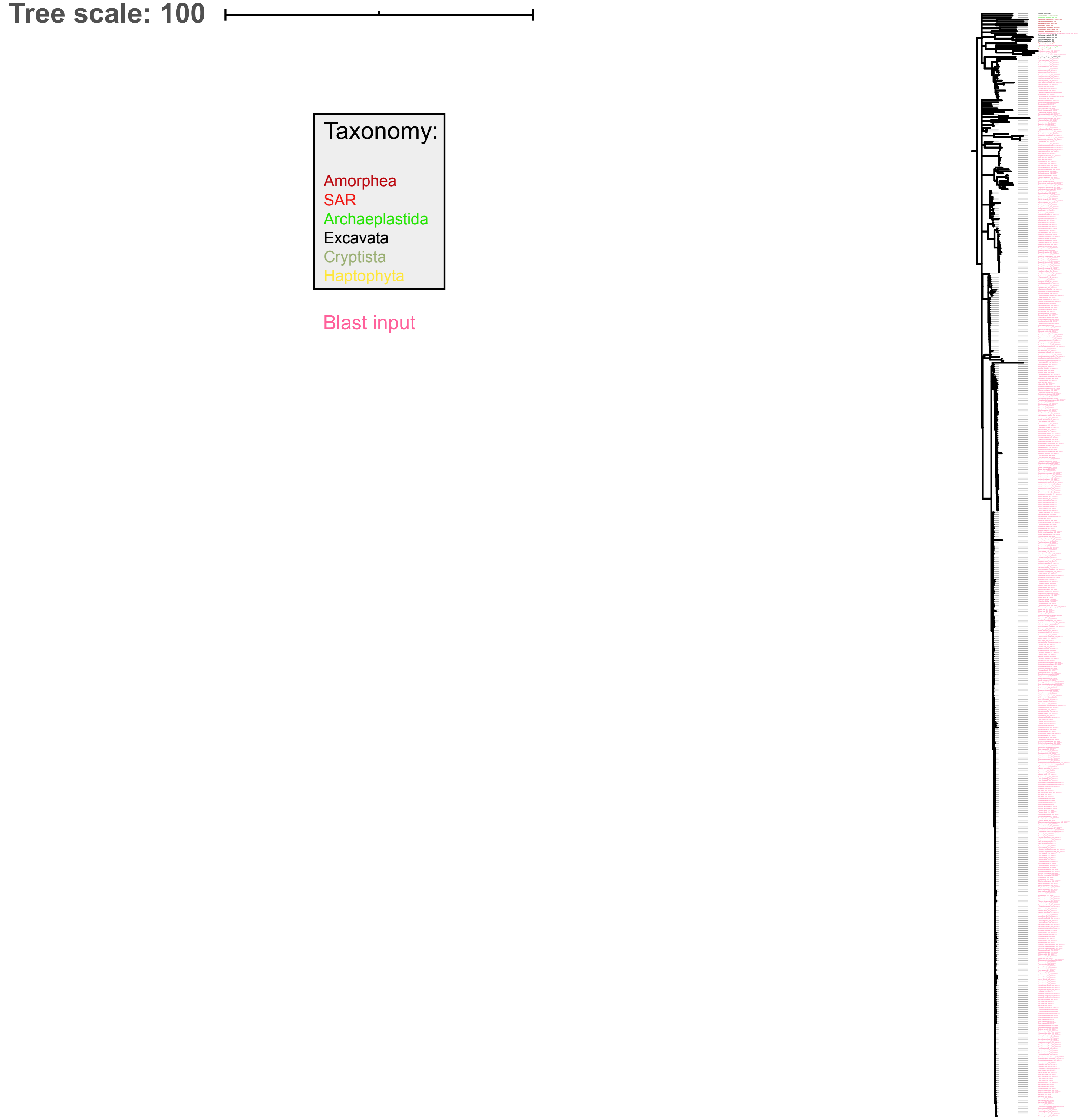

Tree scale: 100
Taxonomy:
Amorphea
SAR
Archaeplastida
Excavata
Cryptista
Haptophyta
Blast input

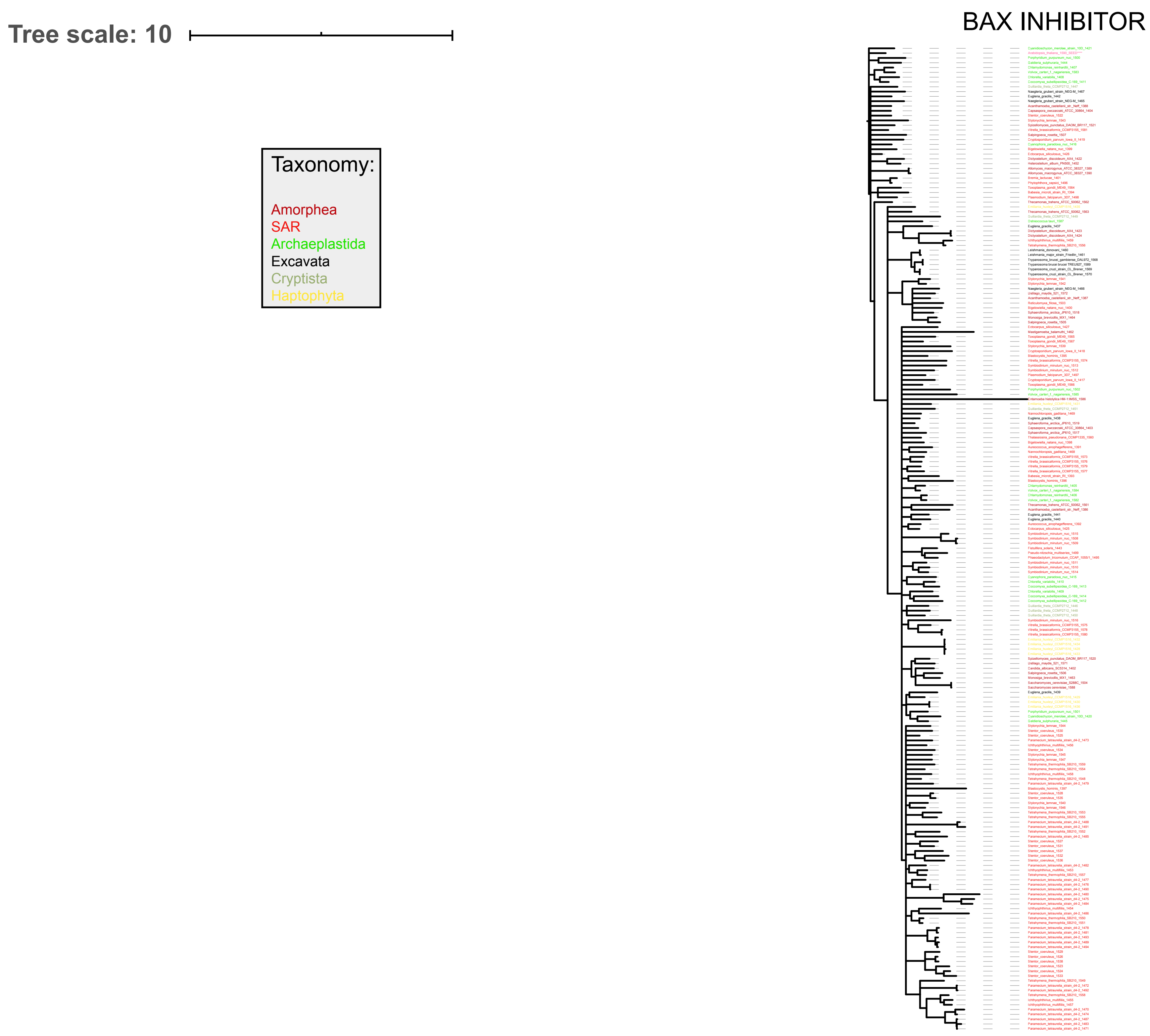
Tree scale: 10
BAX INHIBITOR
Taxonomy:
Amorphea
SAR
Archaeplastida
Excavata
Cryptista
Haptophyta

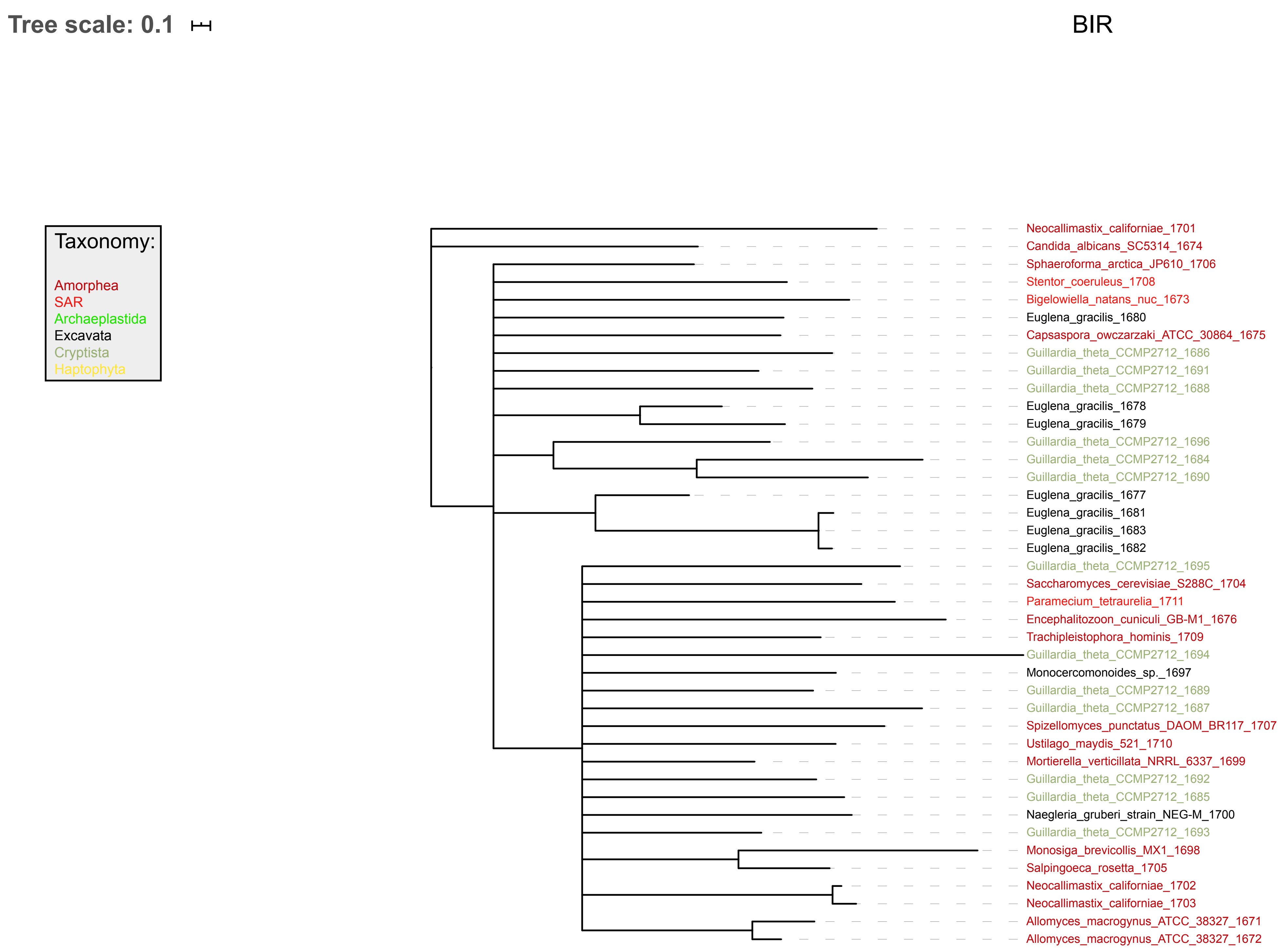
Tree scale: 0.1
BIR
Taxonomy:
Amorphea
SAR
Archaeplastida
Excavata
Cryptista
Haptophyta
Neocallimastix_californiae_1701
Candida_albicans_SC5314_1674
Sphaeroforma_arctica_JP610_1706
Stentor_coeruleus_1708
Bigelowiella_natans_nuc_1673
Euglena_gracilis_1680
Capsaspora_owczarzaki_ATCC_30864_1675
Guillardia_theta_CCMP2712_1686
Guillardia_theta_CCMP2712_1691
Guillardia_theta_CCMP2712_1688
Euglena_gracilis_1678
Euglena_gracilis_1679
Guillardia_theta_CCMP2712_1696
Guillardia_theta_CCMP2712_1684
Guillardia_theta_CCMP2712_1690
Euglena_gracilis_1677
Euglena_gracilis_1681
Euglena_gracilis_1683
Euglena_gracilis_1682
Guillardia_theta_CCMP2712_1695
Saccharomyces_cerevisiae_S288C_1704
Paramecium_tetraurelia_1711
Encephalitozoon_cuniculi_GB-M1_1676
Trachipleistophora_hominis_1709
Guillardia_theta_CCMP2712_1694
Monocercomonoides_sp._1697
Guillardia_theta_CCMP2712_1689
Guillardia_theta_CCMP2712_1687
Spizellomyces_punctatus_DAOM_BR117_1707
Ustilago_maydis_521_1710
Mortierella_verticillata_NRRL_6337_1699
Guillardia_theta_CCMP2712_1692
Guillardia_theta_CCMP2712_1685
Naegleria_gruberi_strain_NEG-M_1700
Guillardia_theta_CCMP2712_1693
Monosiga_brevicollis_MX1_1698
Salpingoeca_rosetta_1705
Neocallimastix_californiae_1702
Neocallimastix_californiae_1703
Allomyces_macrogynus_ATCC_38327_1671
Allomyces_macrogynus_ATCC_38327_1672

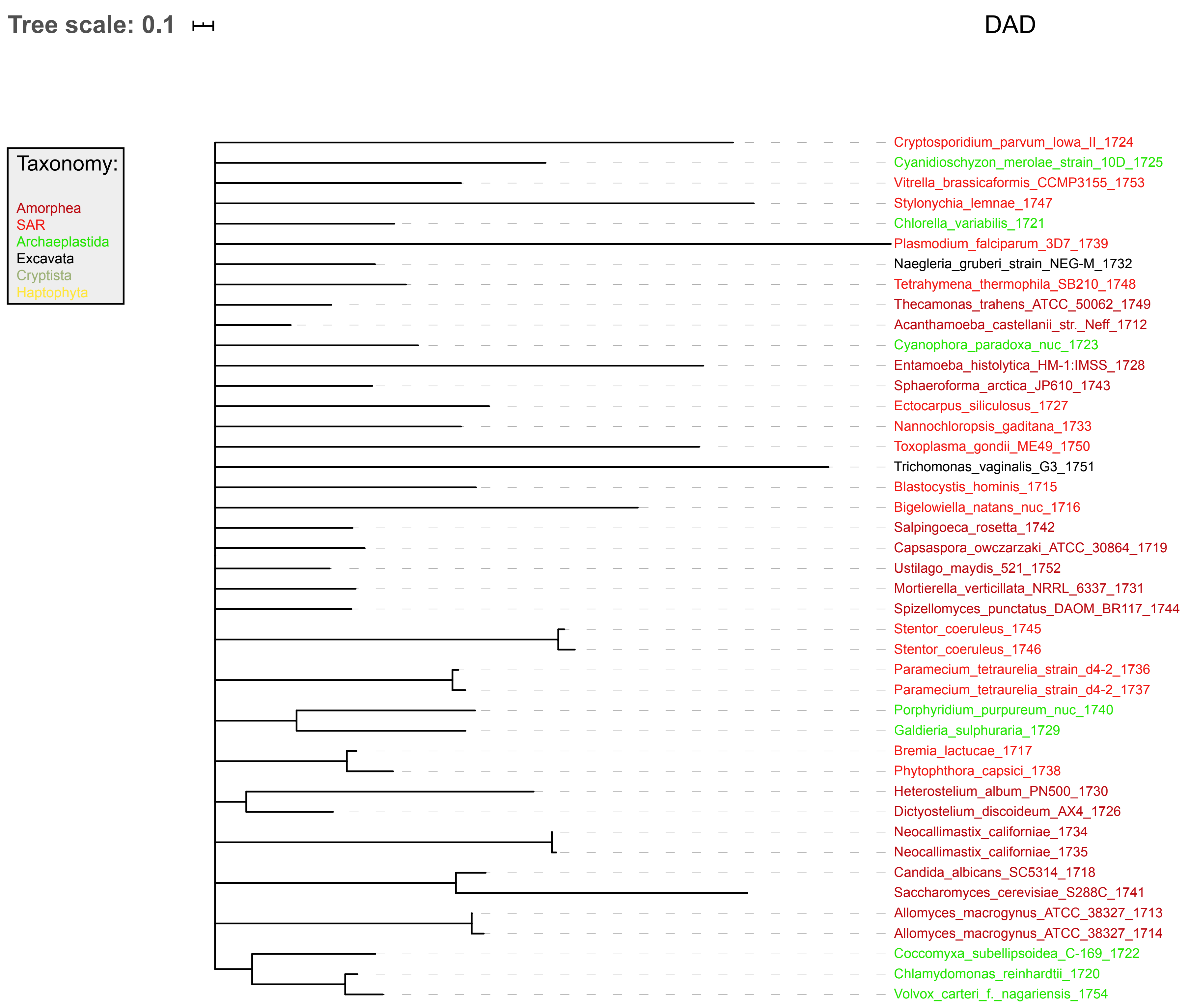

Tree scale: 0.1
DAD
Taxonomy:
Amorphea
SAR
Archaeplastida
Excavata
Cryptista
Haptophyta
Cryptosporidium_parvum_Iowa_II_1724
Cyanidioschyzon_merolae_strain_10D_1725
Vitrella_brassicaformis_CCMP3155_1753
Stylonychia_lemnae_1747
Chlorella_variabilis_1721
Plasmodium_falciparum_3D7_1739
Naegleria_gruberi_strain_NEG-M_1732
Tetrahymena_thermophila_SB210_1748
Thecamonas_trahens_ATCC_50062_1749
Acanthamoeba_castellanii_str._Neff_1712
Cyanophora_paradoxa_nuc_1723
Entamoeba_histolytica_HM-1:IMSS_1728
Sphaeroforma_arctica_JP610_1743
Ectocarpus_siliculosus_1727
Nannochloropsis_gaditana_1733
Toxoplasma_gondii_ME49_1750
Trichomonas_vaginalis_G3_1751
Blastocystis_hominis_1715
Bigelowiella_natans_nuc_1716
Salpingoeca_rosetta_1742
Capsaspora_owczarzaki_ATCC_30864_1719
Ustilago_maydis_521_1752
Mortierella_verticillata_NRRL_6337_1731
Spizellomyces_punctatus_DAOM_BR117_1744
Stentor_coeruleus_1745
Stentor_coeruleus_1746
Paramecium_tetraurelia_strain_d4-2_1736
Paramecium_tetraurelia_strain_d4-2_1737
Porphyridium_purpureum_nuc_1740
Galdieria_sulphuraria_1729
Bremia_lactucae_1717
Phytophthora_capsici_1738
Heterostelium_album_PN500_1730
Dictyostelium_discoideum_AX4_1726
Neocallimastix_californiae_1734
Neocallimastix_californiae_1735
Candida_albicans_SC5314_1718
Saccharomyces_cerevisiae_S288C_1741
Allomyces_macrogynus_ATCC_38327_1713
Allomyces_macrogynus_ATCC_38327_1714
Coccomyxa_subellipsoidea_C-169_1722
Chlamydomonas_reinhardtii_1720
Volvox_carteri_f._nagariensis_1754

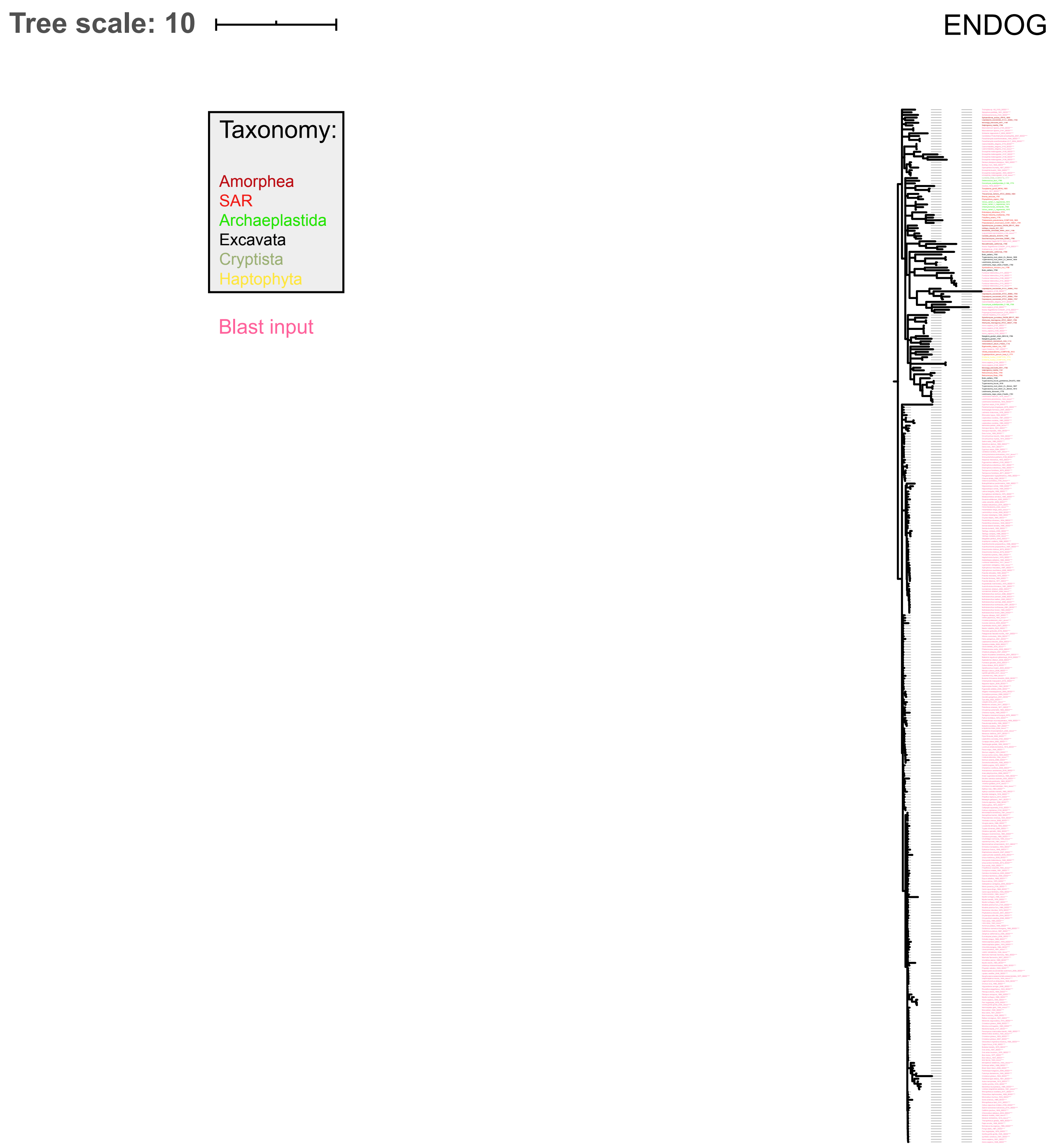
Tree scale: 10
ENDOG
Taxonomy:
Amorphea
SAR
Archaeplastida
Excavata
Cryptista
Haptophyta
Blast input

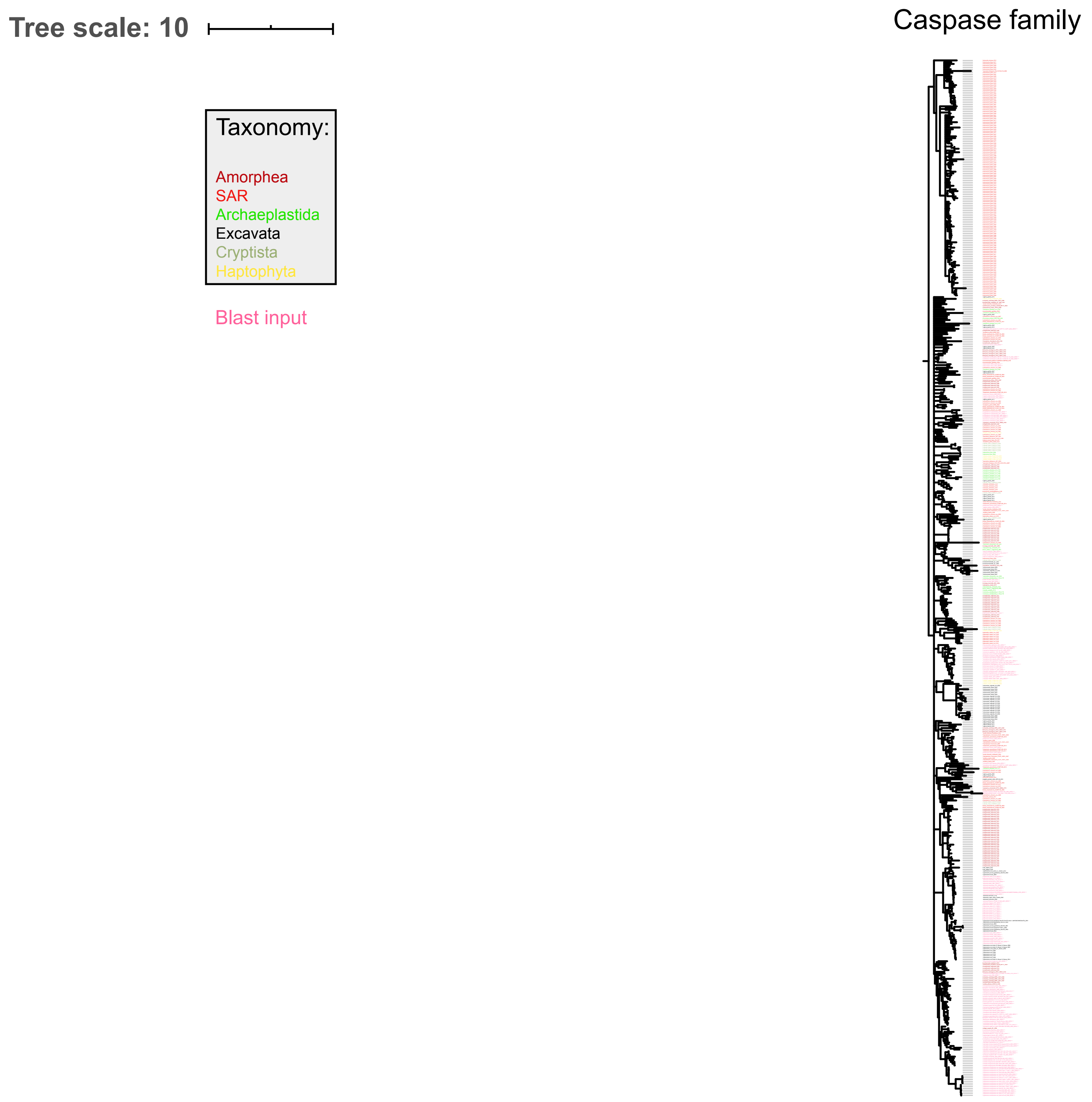
Tree scale: 10
Caspase family
Taxonomy:
Amorphea
SAR
Archaeplastida
Excavata
Cryptista
Haptophyta
Blast input

Tree scale: 10

NACHT

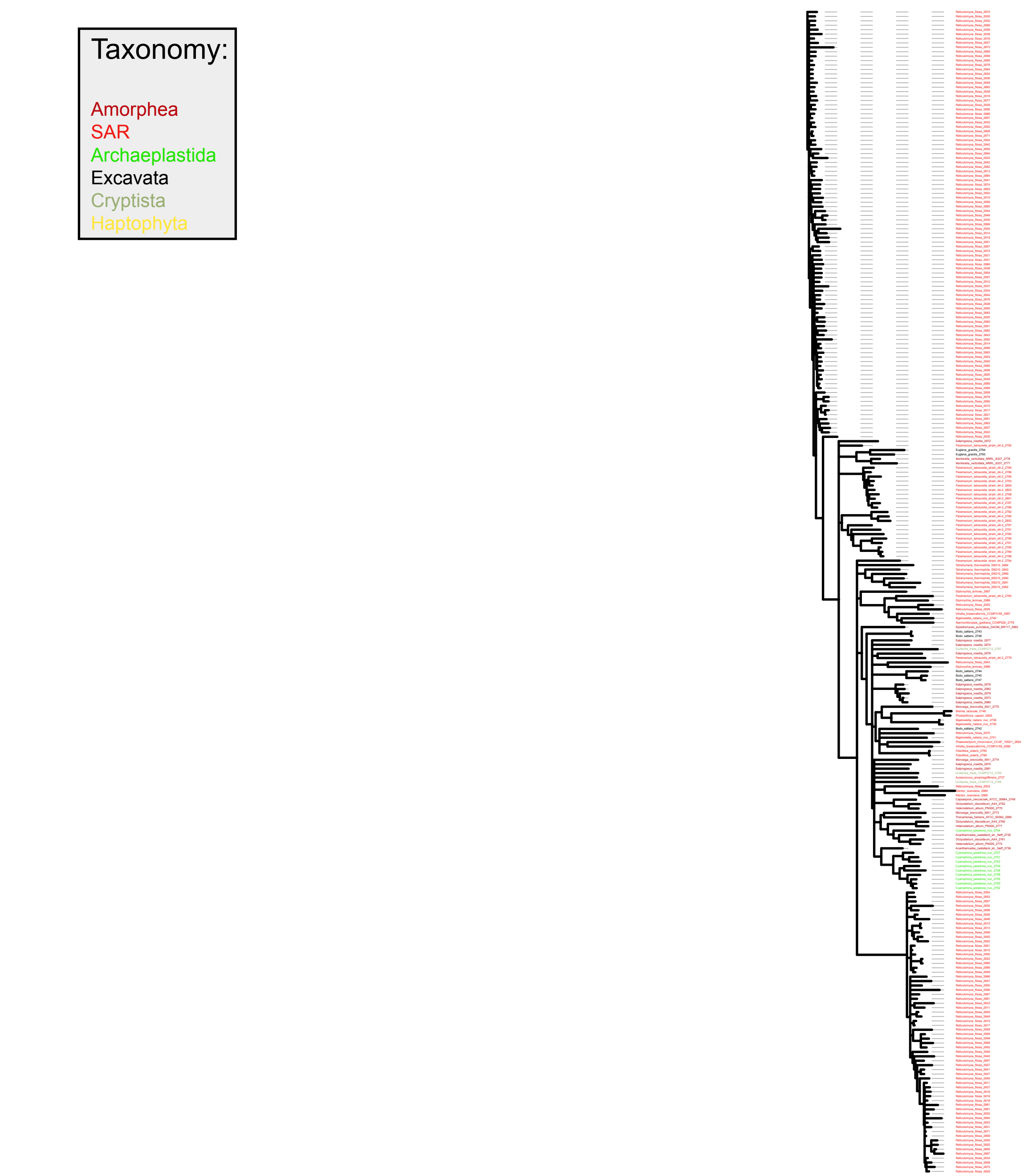

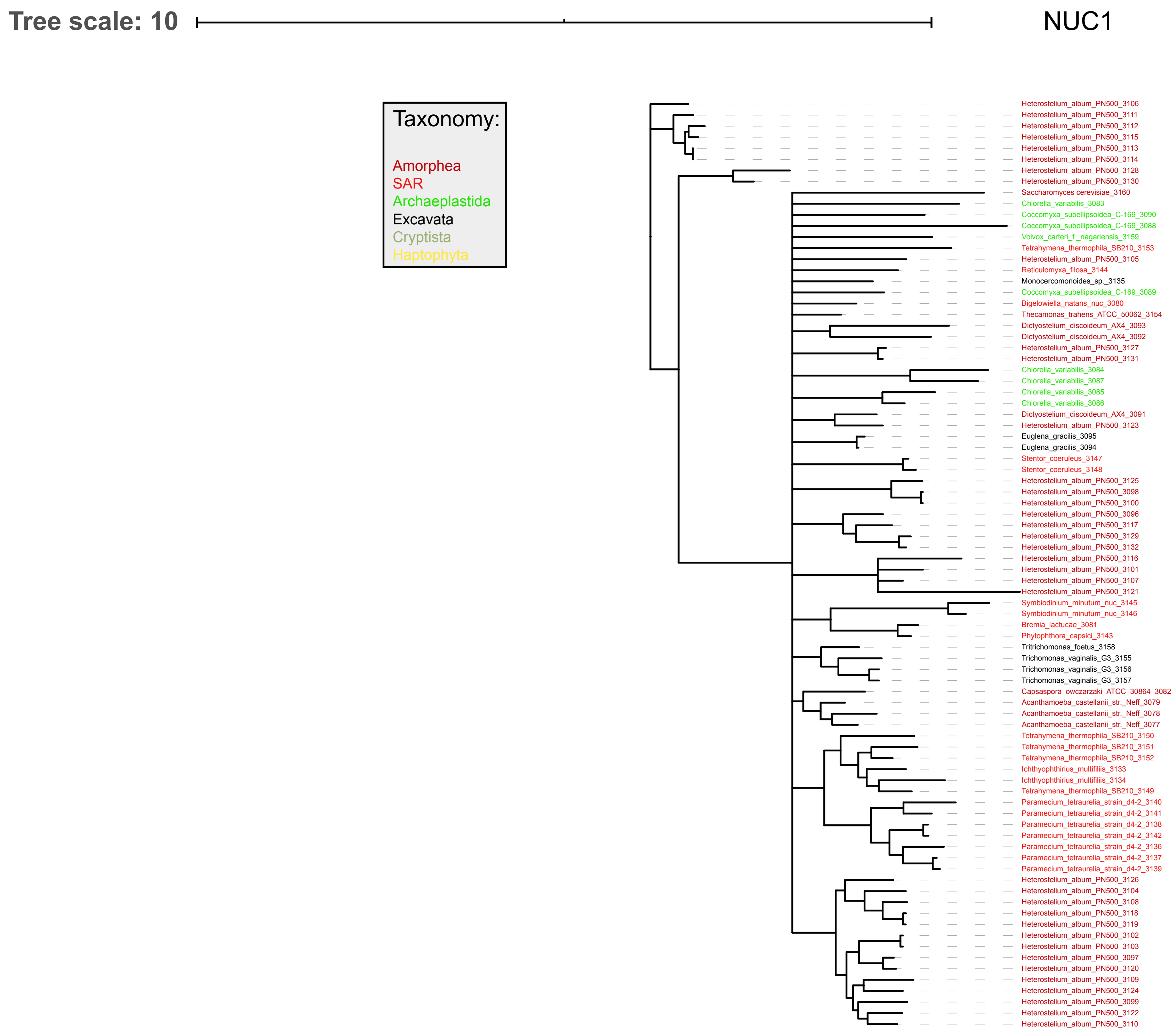

Tree scale: 10
NUC1
Taxonomy:
Amorphea
SAR
Archaeplastida
Excavata
Cryptista
Haptophyta
Heterostelium_album_PN500_3106
Heterostelium_album_PN500_3111
Heterostelium_album_PN500_3112
Heterostelium_album_PN500_3115
Heterostelium_album_PN500_3113
Heterostelium_album_PN500_3114
Heterostelium_album_PN500_3128
Heterostelium_album_PN500_3130
Saccharomyces cerevisiae_3160
Chlorella_variabilis_3083
Coccomyxa_subellipsoidea_C-169_3090
Coccomyxa_subellipsoidea_C-169_3088
Volvox_carteri_f._nagariensis_3159
Tetrahymena_thermophila_SB210_3153
Heterostelium_album_PN500_3105
Reticulomyxa_filosa_3144
Monocercomonoides_sp._3135
Coccomyxa_subellipsoidea_C-169_3089
Bigelowiella_natans_nuc_3080
Thecamonas_trahens_ATCC_50062_3154
Dictyostelium_discoideum_AX4_3093
Dictyostelium_discoideum_AX4_3092
Heterostelium_album_PN500_3127
Heterostelium_album_PN500_3131
Chlorella_variabilis_3084
Chlorella_variabilis_3087
Chlorella_variabilis_3085
Chlorella_variabilis_3086
Dictyostelium_discoideum_AX4_3091
Heterostelium_album_PN500_3123
Euglena_gracilis_3095
Euglena_gracilis_3094
Stentor_coeruleus_3147
Stentor_coeruleus_3148
Heterostelium_album_PN500_3125
Heterostelium_album_PN500_3098
Heterostelium_album_PN500_3100
Heterostelium_album_PN500_3096
Heterostelium_album_PN500_3117
Heterostelium_album_PN500_3129
Heterostelium_album_PN500_3132
Heterostelium_album_PN500_3116
Heterostelium_album_PN500_3101
Heterostelium_album_PN500_3107
Heterostelium_album_PN500_3121
Symbiodinium_minutum_nuc_3145
Symbiodinium_minutum_nuc_3146
Bremia_lactucae_3081
Phytophthora_capsici_3143
Tritrichomonas_foetus_3158
Trichomonas_vaginalis_G3_3155
Trichomonas_vaginalis_G3_3156
Trichomonas_vaginalis_G3_3157
Capsaspora_owczarzaki_ATCC_30864_3082
Acanthamoeba_castellanii_str._Neff_3079
Acanthamoeba_castellanii_str._Neff_3078
Acanthamoeba_castellanii_str._Neff_3077
Tetrahymena_thermophila_SB210_3150
Tetrahymena_thermophila_SB210_3151
Tetrahymena_thermophila_SB210_3152
Ichthyophthirius_multifiliis_3133
Ichthyophthirius_multifiliis_3134
Tetrahymena_thermophila_SB210_3149
Paramecium_tetraurelia_strain_d4-2_3140
Paramecium_tetraurelia_strain_d4-2_3141
Paramecium_tetraurelia_strain_d4-2_3138
Paramecium_tetraurelia_strain_d4-2_3142
Paramecium_tetraurelia_strain_d4-2_3136
Paramecium_tetraurelia_strain_d4-2_3137
Paramecium_tetraurelia_strain_d4-2_3139
Heterostelium_album_PN500_3126
Heterostelium_album_PN500_3104
Heterostelium_album_PN500_3108
Heterostelium_album_PN500_3118
Heterostelium_album_PN500_3119
Heterostelium_album_PN500_3102
Heterostelium_album_PN500_3103
Heterostelium_album_PN500_3097
Heterostelium_album_PN500_3120
Heterostelium_album_PN500_3109
Heterostelium_album_PN500_3124
Heterostelium_album_PN500_3099
Heterostelium_album_PN500_3122
Heterostelium_album_PN500_3110

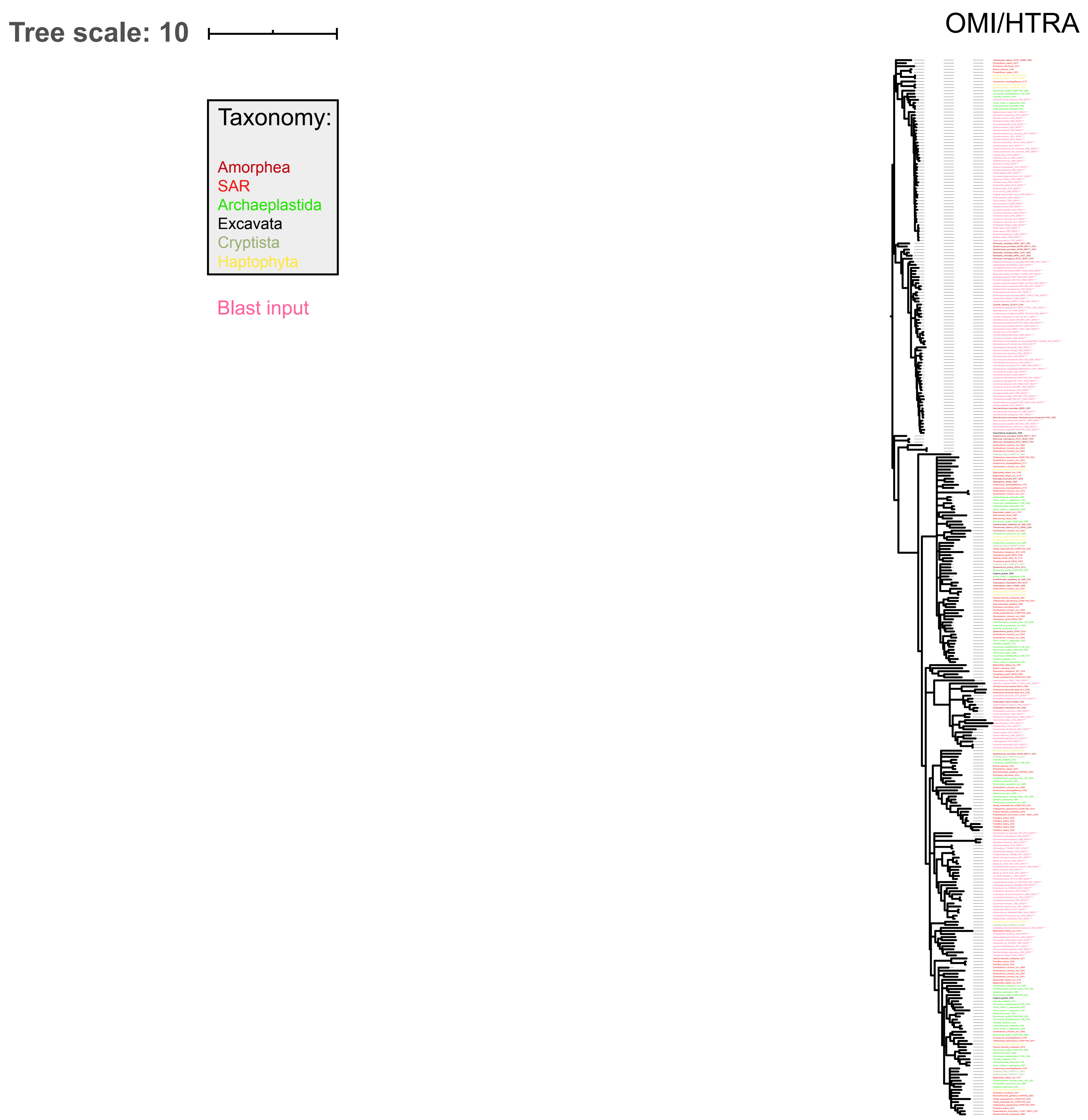
OMI/HTRA
Tree scale: 10
Taxonomy:
Amorphea
SAR
Archaeplastida
Excavata
Cryptista
Haptophyta
Blast input

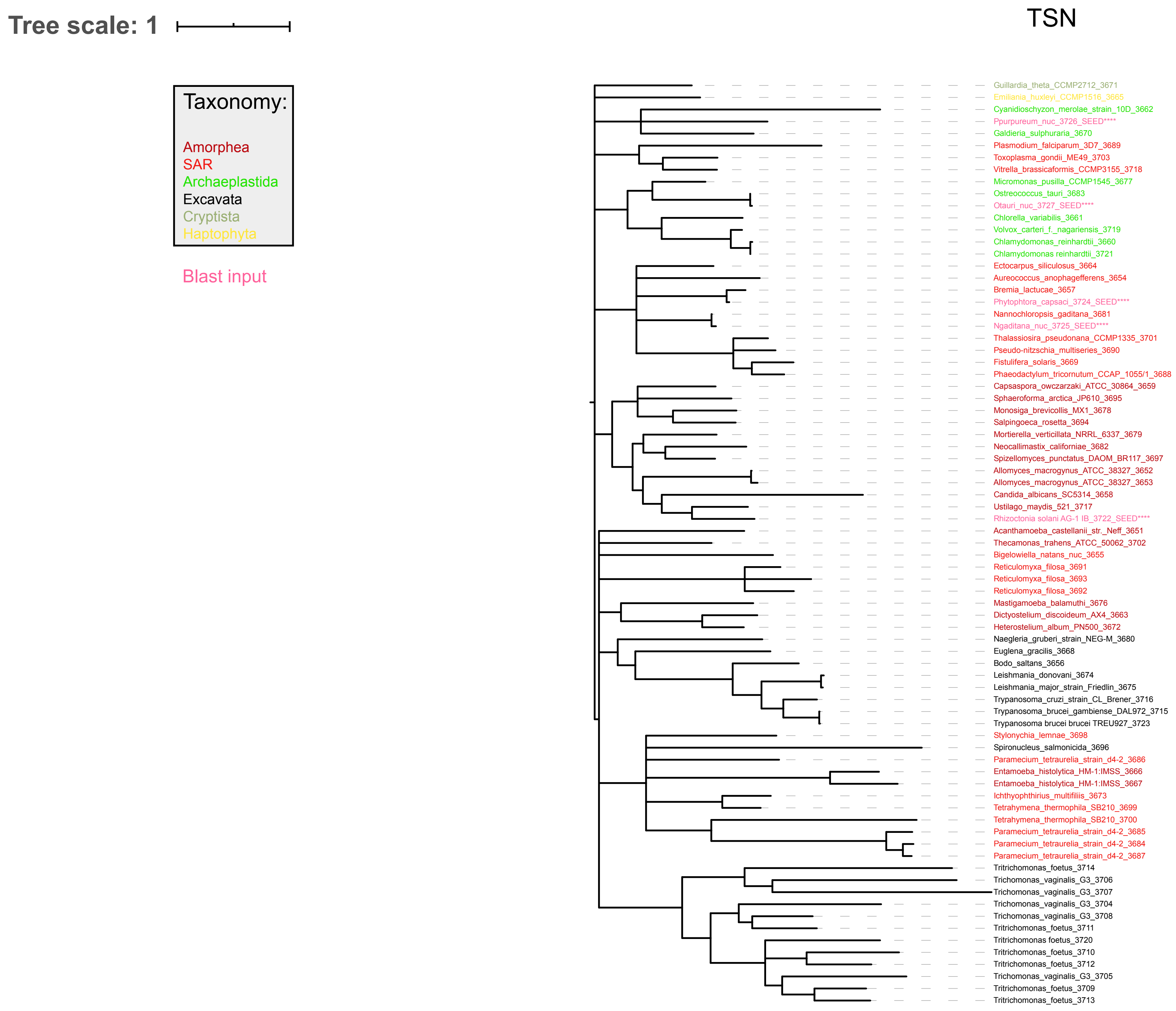
Tree scale: 1
TSN
Taxonomy:
Amorphea
SAR
Archaeplastida
Excavata
Cryptista
Haptophyta
Blast input
Guillardia_theta_CCMP2712_3671
Emiliania_huxleyi_CCMP1516_3665
Cyanidioschyzon_merolae_strain_10D_3662
Ppurpureum_nuc_3726_SEED****
Galdieria_sulphuraria_3670
Plasmodium_falciparum_3D7_3689
Toxoplasma_gondii_ME49_3703
Vitrella_brassicaformis_CCMP3155_3718
Micromonas_pusilla_CCMP1545_3677
Ostreococcus_tauri_3683
Otauri_nuc_3727_SEED****
Chlorella_variabilis_3661
Volvox_carteri_f._nagariensis_3719
Chlamydomonas_reinhardtii_3660
Chlamydomonas reinhardtii_3721
Ectocarpus_siliculosus_3664
Aureococcus_anophagefferens_3654
Bremia_lactucae_3657
Phytophtora_capsaci_3724_SEED****
Nannochloropsis_gaditana_3681
Ngaditana_nuc_3725_SEED****
Thalassiosira_pseudonana_CCMP1335_3701
Pseudo-nitzschia_multiseries_3690
Fistulifera_solaris_3669
Phaeodactylum_tricornutum_CCAP_1055/1_3688
Capsaspora_owczarzaki_ATCC_30864_3659
Sphaeroforma_arctica_JP610_3695
Monosiga_brevicollis_MX1_3678
Salpingoeca_rosetta_3694
Mortierella_verticillata_NRRL_6337_3679
Neocallimastix_californiae_3682
Spizellomyces_punctatus_DAOM_BR117_3697
Allomyces_macrogynus_ATCC_38327_3652
Allomyces_macrogynus_ATCC_38327_3653
Candida_albicans_SC5314_3658
Ustilago_maydis_521_3717
Rhizoctonia solani AG-1 IB_3722_SEED****
Acanthamoeba_castellanii_str._Neff_3651
Thecamonas_trahens_ATCC_50062_3702
Bigelowiella_natans_nuc_3655
Reticulomyxa_filosa_3691
Reticulomyxa_filosa_3693
Reticulomyxa_filosa_3692
Mastigamoeba_balamuthi_3676
Dictyostelium_discoideum_AX4_3663
Heterostelium_album_PN500_3672
Naegleria_gruberi_strain_NEG-M_3680
Euglena_gracilis_3668
Bodo_saltans_3656
Leishmania_donovani_3674
Leishmania_major_strain_Friedlin_3675
Trypanosoma_cruzi_strain_CL_Brener_3716
Trypanosoma_brucei_gambiense_DAL972_3715
Trypanosoma brucei brucei TREU927_3723
Stylonychia_lemnae_3698
Spironucleus_salmonicida_3696
Paramecium_tetraurelia_strain_d4-2_3686
Entamoeba_histolytica_HM-1:IMSS_3666
Entamoeba_histolytica_HM-1:IMSS_3667
Ichthyophthirius_multifiliis_3673
Tetrahymena_thermophila_SB210_3699
Tetrahymena_thermophila_SB210_3700
Paramecium_tetraurelia_strain_d4-2_3685
Paramecium_tetraurelia_strain_d4-2_3684
Paramecium_tetraurelia_strain_d4-2_3687
Tritrichomonas_foetus_3714
Trichomonas_vaginalis_G3_3706
Trichomonas_vaginalis_G3_3707
Trichomonas_vaginalis_G3_3704
Trichomonas_vaginalis_G3_3708
Tritrichomonas_foetus_3711
Tritrichomonas foetus_3720
Tritrichomonas_foetus_3710
Tritrichomonas_foetus_3712
Trichomonas_vaginalis_G3_3705
Tritrichomonas_foetus_3709
Tritrichomonas_foetus_3713

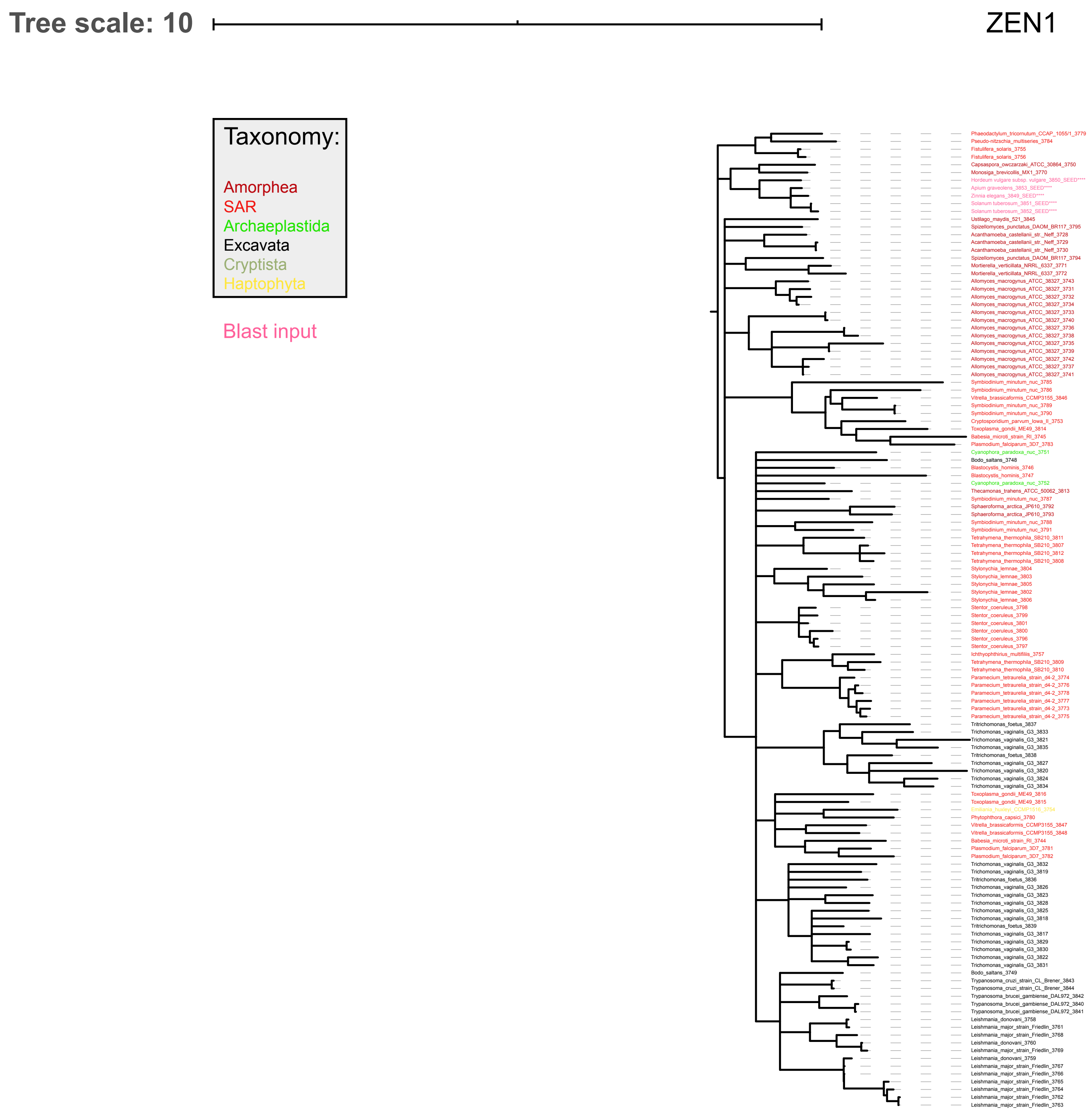
Tree scale: 10
ZEN1
Taxonomy:
Amorphea
SAR
Archaeplastida
Excavata
Cryptista
Haptophyta
Blast input
Phaeodactylum_tricornutum_CCAP_1055/1_3779
Pseudo-nitzschia_multiseries_3784
Fistulifera_solaris_3755
Fistulifera_solaris_3756
Capsaspora_owczarzaki_ATCC_30864_3750
Monosiga_brevicollis_MX1_3770
Hordeum vulgare subsp. vulgare_3850_SEED****
Apium graveolens_3853_SEED****
Zinnia elegans_3849_SEED****
Solanum tuberosum_3851_SEED****
Solanum tuberosum_3852_SEED****
Ustilago_maydis_521_3845
Spizellomyces_punctatus_DAOM_BR117_3795
Acanthamoeba_castellanii_str._Neff_3728
Acanthamoeba_castellanii_str._Neff_3729
Acanthamoeba_castellanii_str._Neff_3730
Spizellomyces_punctatus_DAOM_BR117_3794
Mortierella_verticillata_NRRL_6337_3771
Mortierella_verticillata_NRRL_6337_3772
Allomyces_macrogynus_ATCC_38327_3743
Allomyces_macrogynus_ATCC_38327_3731
Allomyces_macrogynus_ATCC_38327_3732
Allomyces_macrogynus_ATCC_38327_3734
Allomyces_macrogynus_ATCC_38327_3733
Allomyces_macrogynus_ATCC_38327_3740
Allomyces_macrogynus_ATCC_38327_3736
Allomyces_macrogynus_ATCC_38327_3738
Allomyces_macrogynus_ATCC_38327_3735
Allomyces_macrogynus_ATCC_38327_3739
Allomyces_macrogynus_ATCC_38327_3742
Allomyces_macrogynus_ATCC_38327_3737
Allomyces_macrogynus_ATCC_38327_3741
Symbiodinium_minutum_nuc_3785
Symbiodinium_minutum_nuc_3786
Vitrella_brassicaformis_CCMP3155_3846
Symbiodinium_minutum_nuc_3789
Symbiodinium_minutum_nuc_3790
Cryptosporidium_parvum_Iowa_II_3753
Toxoplasma_gondii_ME49_3814
Babesia_microti_strain_RI_3745
Plasmodium_falciparum_3D7_3783
Cyanophora_paradoxa_nuc_3751
Bodo_saltans_3748
Blastocystis_hominis_3746
Blastocystis_hominis_3747
Cyanophora_paradoxa_nuc_3752
Thecamonas_trahens_ATCC_50062_3813
Symbiodinium_minutum_nuc_3787
Sphaeroforma_arctica_JP610_3792
Sphaeroforma_arctica_JP610_3793
Symbiodinium_minutum_nuc_3788
Symbiodinium_minutum_nuc_3791
Tetrahymena_thermophila_SB210_3811
Tetrahymena_thermophila_SB210_3807
Tetrahymena_thermophila_SB210_3812
Tetrahymena_thermophila_SB210_3808
Stylonychia_lemnae_3804
Stylonychia_lemnae_3803
Stylonychia_lemnae_3805
Stylonychia_lemnae_3802
Stylonychia_lemnae_3806
Stentor_coeruleus_3798
Stentor_coeruleus_3799
Stentor_coeruleus_3801
Stentor_coeruleus_3800
Stentor_coeruleus_3796
Stentor_coeruleus_3797
Ichthyophthirius_multifiliis_3757
Tetrahymena_thermophila_SB210_3809
Tetrahymena_thermophila_SB210_3810
Paramecium_tetraurelia_strain_d4-2_3774
Paramecium_tetraurelia_strain_d4-2_3776
Paramecium_tetraurelia_strain_d4-2_3778
Paramecium_tetraurelia_strain_d4-2_3777
Paramecium_tetraurelia_strain_d4-2_3773
Paramecium_tetraurelia_strain_d4-2_3775
Tritrichomonas_foetus_3837
Trichomonas_vaginalis_G3_3833
Trichomonas_vaginalis_G3_3821
Trichomonas_vaginalis_G3_3835
Tritrichomonas_foetus_3838
Trichomonas_vaginalis_G3_3827
Trichomonas_vaginalis_G3_3820
Trichomonas_vaginalis_G3_3824
Trichomonas_vaginalis_G3_3834
Toxoplasma_gondii_ME49_3816
Toxoplasma_gondii_ME49_3815
Emiliania_huxleyi_CCMP1516_3754
Phytophthora_capsici_3780
Vitrella_brassicaformis_CCMP3155_3847
Vitrella_brassicaformis_CCMP3155_3848
Babesia_microti_strain_RI_3744
Plasmodium_falciparum_3D7_3781
Plasmodium_falciparum_3D7_3782
Trichomonas_vaginalis_G3_3832
Trichomonas_vaginalis_G3_3819
Tritrichomonas_foetus_3836
Trichomonas_vaginalis_G3_3826
Trichomonas_vaginalis_G3_3823
Trichomonas_vaginalis_G3_3828
Trichomonas_vaginalis_G3_3825
Trichomonas_vaginalis_G3_3818
Tritrichomonas_foetus_3839
Trichomonas_vaginalis_G3_3817
Trichomonas_vaginalis_G3_3829
Trichomonas_vaginalis_G3_3830
Trichomonas_vaginalis_G3_3822
Trichomonas_vaginalis_G3_3831
Bodo_saltans_3749
Trypanosoma_cruzi_strain_CL_Brener_3843
Trypanosoma_cruzi_strain_CL_Brener_3844
Trypanosoma_brucei_gambiense_DAL972_3842
Trypanosoma_brucei_gambiense_DAL972_3840
Trypanosoma_brucei_gambiense_DAL972_3841
Leishmania_donovani_3758
Leishmania_major_strain_Friedlin_3761
Leishmania_major_strain_Friedlin_3768
Leishmania_donovani_3760
Leishmania_major_strain_Friedlin_3769
Leishmania_donovani_3759
Leishmania_major_strain_Friedlin_3767
Leishmania_major_strain_Friedlin_3766
Leishmania_major_strain_Friedlin_3765
Leishmania_major_strain_Friedlin_3764
Leishmania_major_strain_Friedlin_3762
Leishmania_major_strain_Friedlin_3763

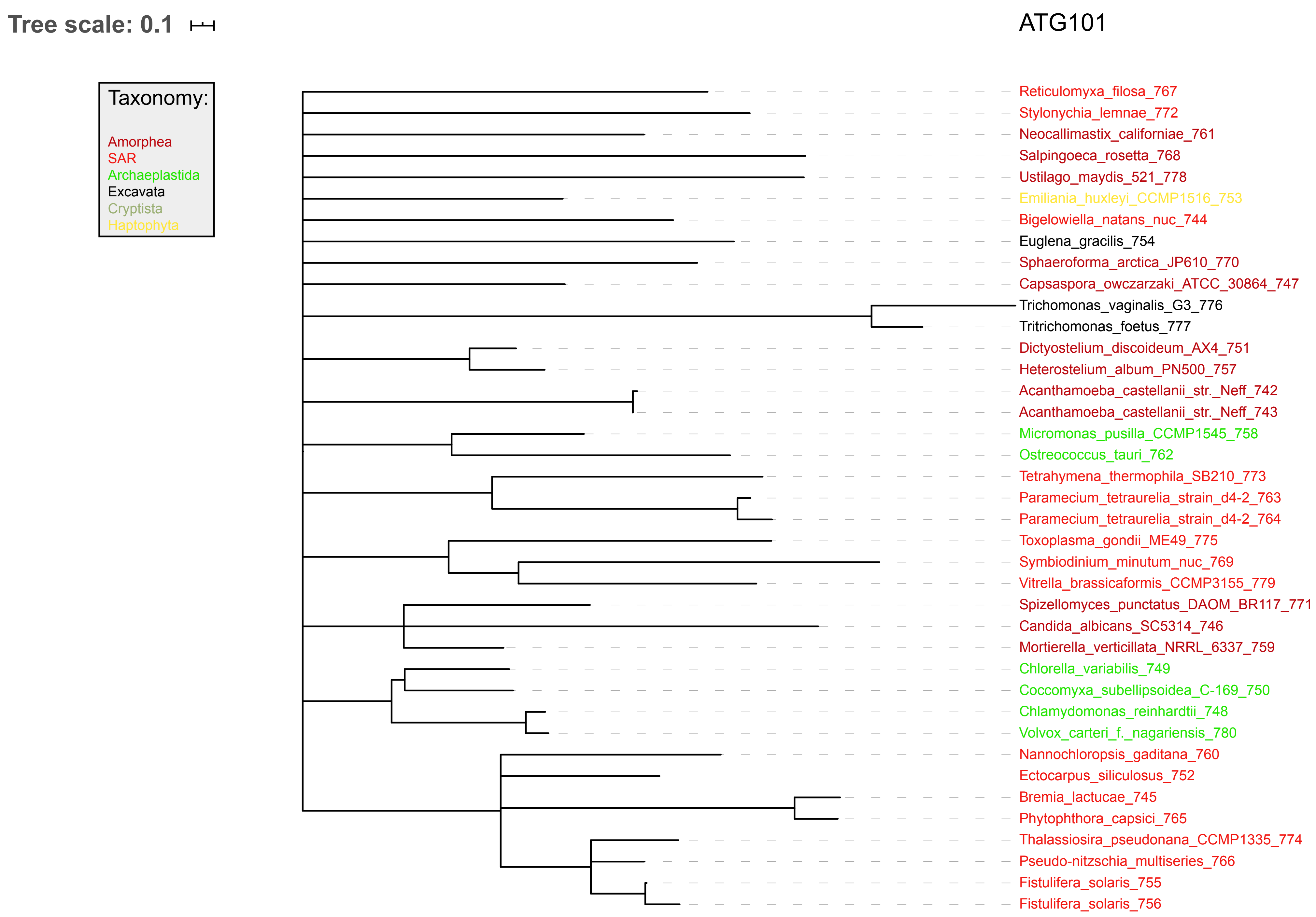

Tree scale: 0.1
ATG101
Taxonomy:
Amorphea
SAR
Archaeplastida
Excavata
Cryptista
Haptophyta
Reticulomyxa_filosa_767
Stylonychia_lemnae_772
Neocallimastix_californiae_761
Salpingoeca_rosetta_768
Ustilago_maydis_521_778
Emiliania_huxleyi_CCMP1516_753
Bigelowiella_natans_nuc_744
Euglena_gracilis_754
Sphaeroforma_arctica_JP610_770
Capsaspora_owczarzaki_ATCC_30864_747
Trichomonas_vaginalis_G3_776
Tritrichomonas_foetus_777
Dictyostelium_discoideum_AX4_751
Heterostelium_album_PN500_757
Acanthamoeba_castellanii_str._Neff_742
Acanthamoeba_castellanii_str._Neff_743
Micromonas_pusilla_CCMP1545_758
Ostreococcus_tauri_762
Tetrahymena_thermophila_SB210_773
Paramecium_tetraurelia_strain_d4-2_763
Paramecium_tetraurelia_strain_d4-2_764
Toxoplasma_gondii_ME49_775
Symbiodinium_minutum_nuc_769
Vitrella_brassicaformis_CCMP3155_779
Spizellomyces_punctatus_DAOM_BR117_771
Candida_albicans_SC5314_746
Mortierella_verticillata_NRRL_6337_759
Chlorella_variabilis_749
Coccomyxa_subellipsoidea_C-169_750
Chlamydomonas_reinhardtii_748
Volvox_carteri_f._nagariensis_780
Nannochloropsis_gaditana_760
Ectocarpus_siliculosus_752
Bremia_lactucae_745
Phytophthora_capsici_765
Thalassiosira_pseudonana_CCMP1335_774
Pseudo-nitzschia_multiseries_766
Fistulifera_solaris_755
Fistulifera_solaris_756

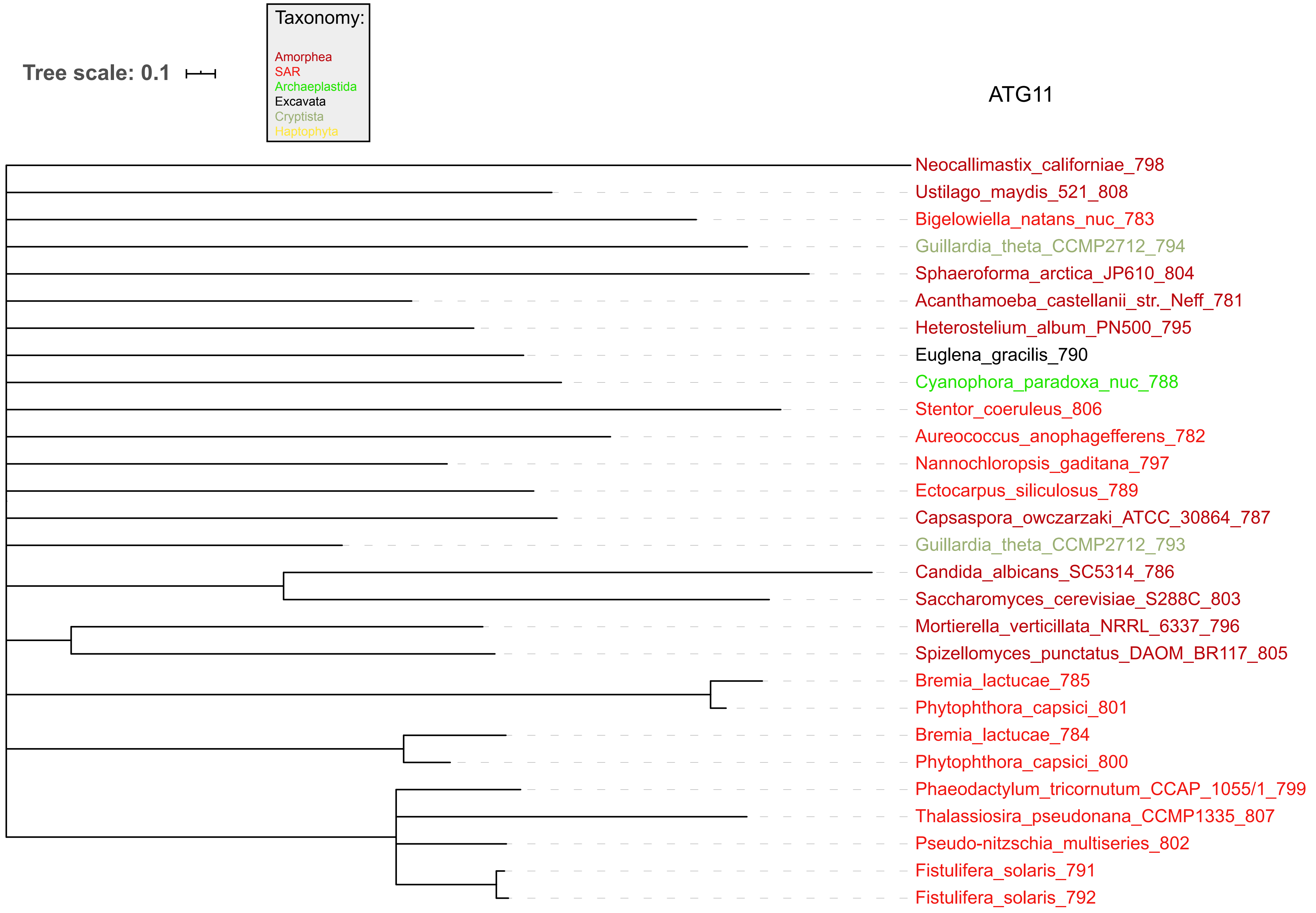
Tree scale: 0.1
Taxonomy:
Amorphea
SAR
Archaeplastida
Excavata
Cryptista
Haptophyta
ATG11
Neocallimastix_californiae_798
Ustilago_maydis_521_808
Bigelowiella_natans_nuc_783
Guillardia_theta_CCMP2712_794
Sphaeroforma_arctica_JP610_804
Acanthamoeba_castellanii_str._Neff_781
Heterostelium_album_PN500_795
Euglena_gracilis_790
Cyanophora_paradoxa_nuc_788
Stentor_coeruleus_806
Aureococcus_anophagefferens_782
Nannochloropsis_gaditana_797
Ectocarpus_siliculosus_789
Capsaspora_owczarzaki_ATCC_30864_787
Guillardia_theta_CCMP2712_793
Candida_albicans_SC5314_786
Saccharomyces_cerevisiae_S288C_803
Mortierella_verticillata_NRRL_6337_796
Spizellomyces_punctatus_DAOM_BR117_805
Bremia_lactucae_785
Phytophthora_capsici_801
Bremia_lactucae_784
Phytophthora_capsici_800
Phaeodactylum_tricornutum_CCAP_1055/1_799
Thalassiosira_pseudonana_CCMP1335_807
Pseudo-nitzschia_multiseries_802
Fistulifera_solaris_791
Fistulifera_solaris_792

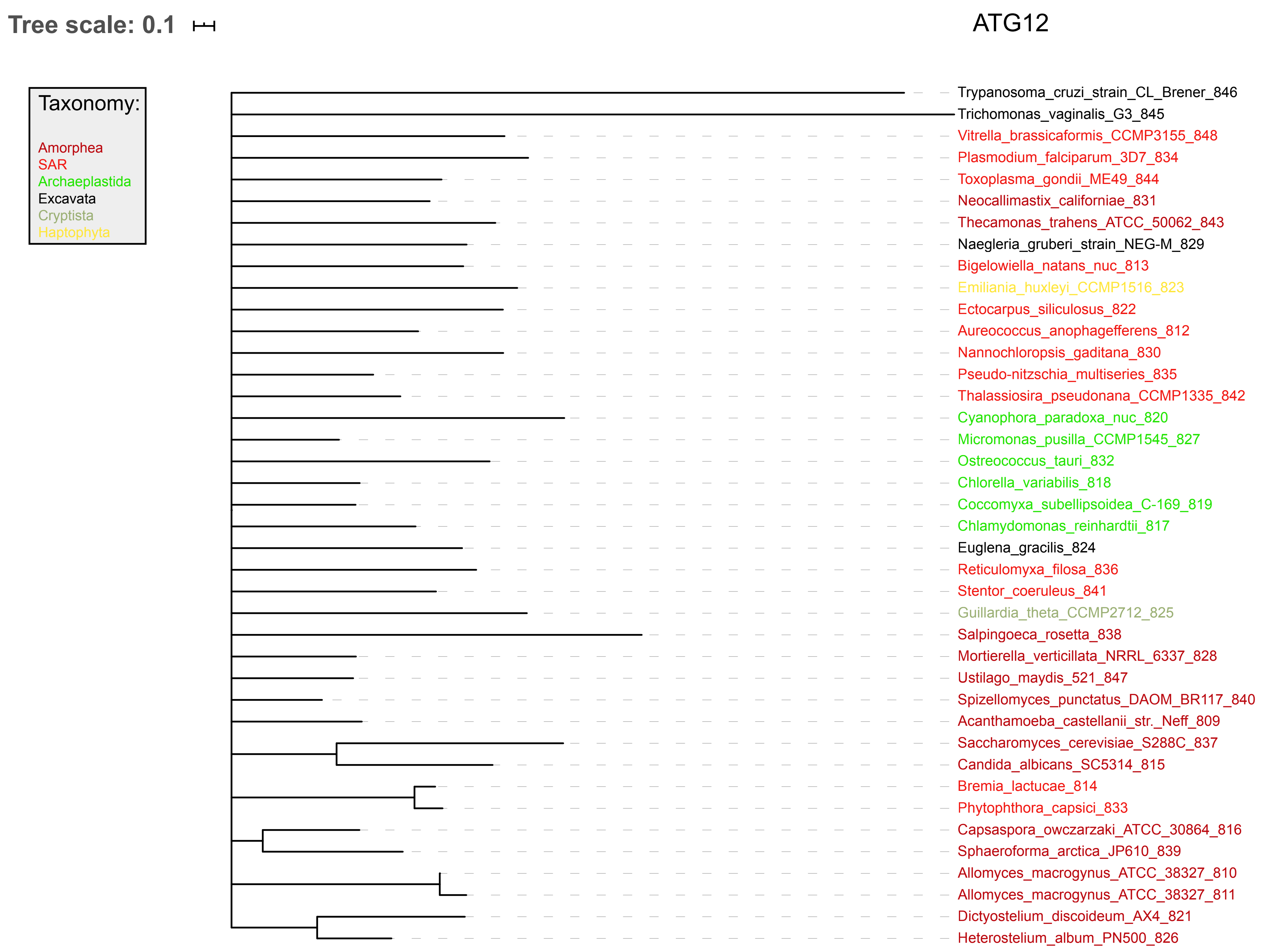

Tree scale: 0.1
ATG12
Taxonomy:
Amorphea
SAR
Archaeplastida
Excavata
Cryptista
Haptophyta
Trypanosoma_cruzi_strain_CL_Brener_846
Trichomonas_vaginalis_G3_845
Vitrella_brassicaformis_CCMP3155_848
Plasmodium_falciparum_3D7_834
Toxoplasma_gondii_ME49_844
Neocallimastix_californiae_831
Thecamonas_trahens_ATCC_50062_843
Naegleria_gruberi_strain_NEG-M_829
Bigelowiella_natans_nuc_813
Emiliania_huxleyi_CCMP1516_823
Ectocarpus_siliculosus_822
Aureococcus_anophagefferens_812
Nannochloropsis_gaditana_830
Pseudo-nitzschia_multiseries_835
Thalassiosira_pseudonana_CCMP1335_842
Cyanophora_paradoxa_nuc_820
Micromonas_pusilla_CCMP1545_827
Ostreococcus_tauri_832
Chlorella_variabilis_818
Coccomyxa_subellipsoidea_C-169_819
Chlamydomonas_reinhardtii_817
Euglena_gracilis_824
Reticulomyxa_filosa_836
Stentor_coeruleus_841
Guillardia_theta_CCMP2712_825
Salpingoeca_rosetta_838
Mortierella_verticillata_NRRL_6337_828
Ustilago_maydis_521_847
Spizellomyces_punctatus_DAOM_BR117_840
Acanthamoeba_castellanii_str._Neff_809
Saccharomyces_cerevisiae_S288C_837
Candida_albicans_SC5314_815
Bremia_lactucae_814
Phytophthora_capsici_833
Capsaspora_owczarzaki_ATCC_30864_816
Sphaeroforma_arctica_JP610_839
Allomyces_macrogynus_ATCC_38327_810
Allomyces_macrogynus_ATCC_38327_811
Dictyostelium_discoideum_AX4_821
Heterostelium_album_PN500_826

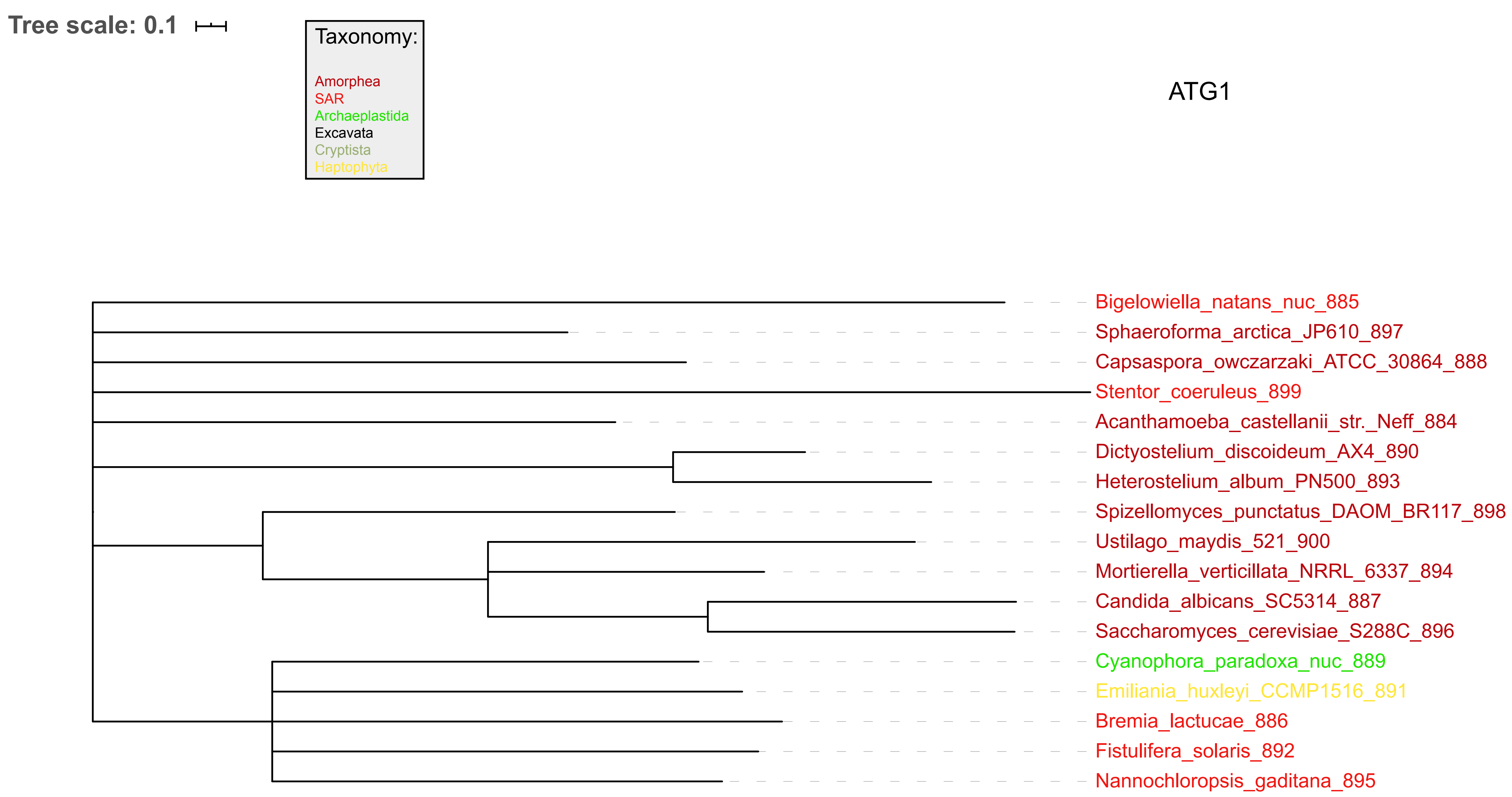

Tree scale: 0.1
Taxonomy:
Amorphea
SAR
Archaeplastida
Excavata
Cryptista
Haptophyta
ATG1
Bigelowiella_natans_nuc_885
Sphaeroforma_arctica_JP610_897
Capsaspora_owczarzaki_ATCC_30864_888
Stentor_coeruleus_899
Acanthamoeba_castellanii_str._Neff_884
Dictyostelium_discoideum_AX4_890
Heterostelium_album_PN500_893
Spizellomyces_punctatus_DAOM_BR117_898
Ustilago_maydis_521_900
Mortierella_verticillata_NRRL_6337_894
Candida_albicans_SC5314_887
Saccharomyces_cerevisiae_S288C_896
Cyanophora_paradoxa_nuc_889
Emiliania_huxleyi_CCMP1516_891
Bremia_lactucae_886
Fistulifera_solaris_892
Nannochloropsis_gaditana_895

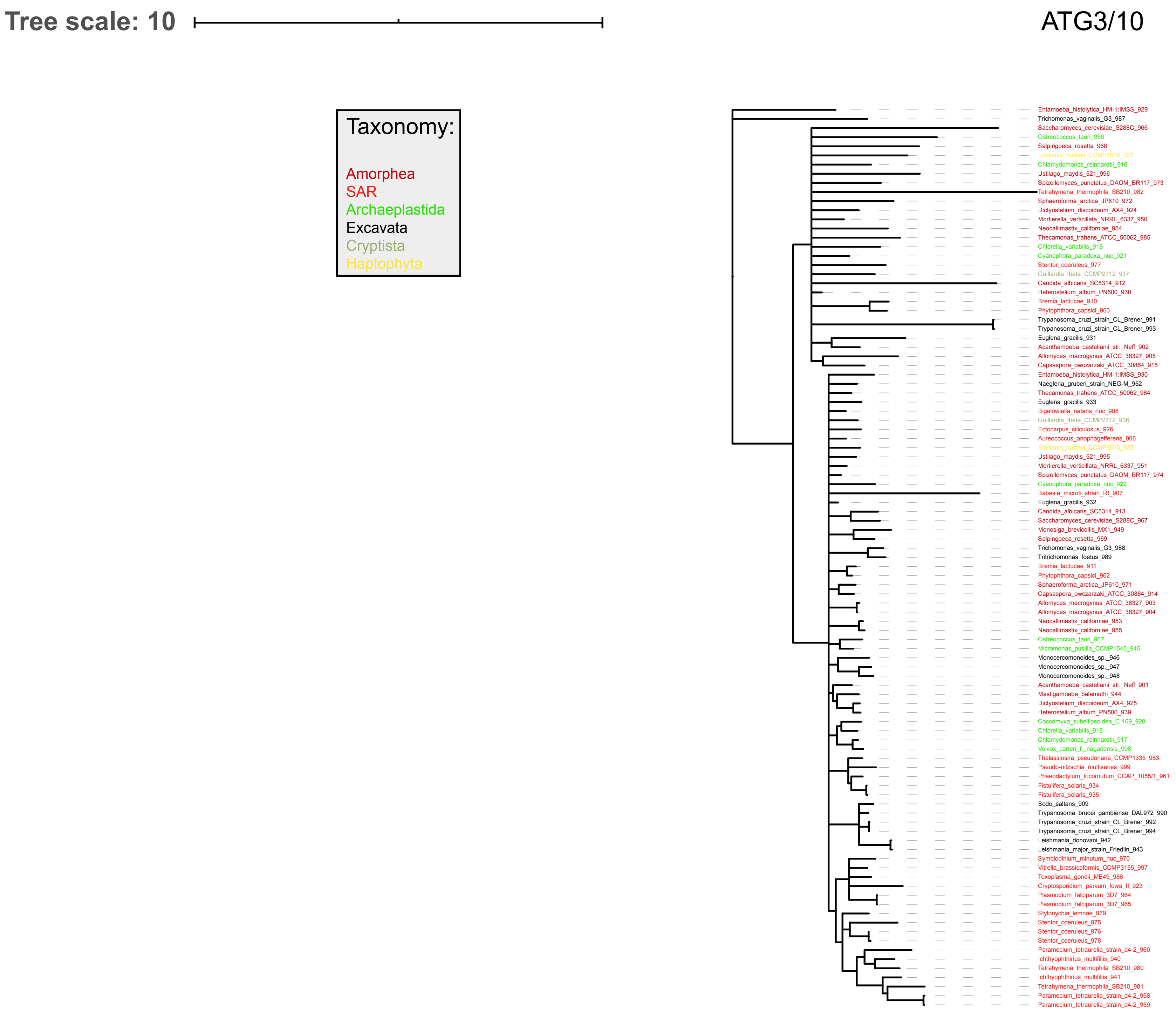
Tree scale: 10
ATG3/10
Taxonomy:
Amorphea
SAR
Archaeplastida
Excavata
Cryptista
Haptophyta
Entamoeba_histolytica_HM-1:IMSS_929
Trichomonas_vaginalis_G3_987
Saccharomyces_cerevisiae_S288C_966
Ostreococcus_tauri_956
Salpingoeca_rosetta_968
Emiliania_huxleyi_CCMP1516_927
Chlamydomonas_reinhardtii_916
Ustilago_maydis_521_996
Spizellomyces_punctatus_DAOM_BR117_973
Tetrahymena_thermophila_SB210_982
Sphaeroforma_arctica_JP610_972
Dictyostelium_discoideum_AX4_924
Mortierella_verticillata_NRRL_6337_950
Neocallimastix_californiae_954
Thecamonas_trahens_ATCC_50062_985
Chlorella_variabilis_918
Cyanophora_paradoxa_nuc_921
Stentor_coeruleus_977
Guillardia_theta_CCMP2712_937
Candida_albicans_SC5314_912
Heterostelium_album_PN500_938
Bremia_lactucae_910
Phytophthora_capsici_963
Trypanosoma_cruzi_strain_CL_Brener_991
Trypanosoma_cruzi_strain_CL_Brener_993
Euglena_gracilis_931
Acanthamoeba_castellanii_str._Neff_902
Allomyces_macrogynus_ATCC_38327_905
Capsaspora_owczarzaki_ATCC_30864_915
Entamoeba_histolytica_HM-1:IMSS_930
Naegleria_gruberi_strain_NEG-M_952
Thecamonas_trahens_ATCC_50062_984
Euglena_gracilis_933
Bigelowiella_natans_nuc_908
Guillardia_theta_CCMP2712_936
Ectocarpus_siliculosus_926
Aureococcus_anophagefferens_906
Emiliania_huxleyi_CCMP1516_928
Ustilago_maydis_521_995
Mortierella_verticillata_NRRL_6337_951
Spizellomyces_punctatus_DAOM_BR117_974
Cyanophora_paradoxa_nuc_922
Babesia_microti_strain_RI_907
Euglena_gracilis_932
Candida_albicans_SC5314_913
Saccharomyces_cerevisiae_S288C_967
Monosiga_brevicollis_MX1_949
Salpingoeca_rosetta_969
Trichomonas_vaginalis_G3_988
Tritrichomonas_foetus_989
Bremia_lactucae_911
Phytophthora_capsici_962
Sphaeroforma_arctica_JP610_971
Capsaspora_owczarzaki_ATCC_30864_914
Allomyces_macrogynus_ATCC_38327_903
Allomyces_macrogynus_ATCC_38327_904
Neocallimastix_californiae_953
Neocallimastix_californiae_955
Ostreococcus_tauri_957
Micromonas_pusilla_CCMP1545_945
Monocercomonoides_sp._946
Monocercomonoides_sp._947
Monocercomonoides_sp._948
Acanthamoeba_castellanii_str._Neff_901
Mastigamoeba_balamuthi_944
Dictyostelium_discoideum_AX4_925
Heterostelium_album_PN500_939
Coccomyxa_subellipsoidea_C-169_920
Chlorella_variabilis_919
Chlamydomonas_reinhardtii_917
Volvox_carteri_f._nagariensis_998
Thalassiosira_pseudonana_CCMP1335_983
Pseudo-nitzschia_multiseries_999
Phaeodactylum_tricornutum_CCAP_1055/1_961
Fistulifera_solaris_934
Fistulifera_solaris_935
Bodo_saltans_909
Trypanosoma_brucei_gambiense_DAL972_990
Trypanosoma_cruzi_strain_CL_Brener_992
Trypanosoma_cruzi_strain_CL_Brener_994
Leishmania_donovani_942
Leishmania_major_strain_Friedlin_943
Symbiodinium_minutum_nuc_970
Vitrella_brassicaformis_CCMP3155_997
Toxoplasma_gondii_ME49_986
Cryptosporidium_parvum_Iowa_II_923
Plasmodium_falciparum_3D7_964
Plasmodium_falciparum_3D7_965
Stylonychia_lemnae_979
Stentor_coeruleus_975
Stentor_coeruleus_976
Stentor_coeruleus_978
Paramecium_tetraurelia_strain_d4-2_960
Ichthyophthirius_multifiliis_940
Tetrahymena_thermophila_SB210_980
Ichthyophthirius_multifiliis_941
Tetrahymena_thermophila_SB210_981
Paramecium_tetraurelia_strain_d4-2_958
Paramecium_tetraurelia_strain_d4-2_959

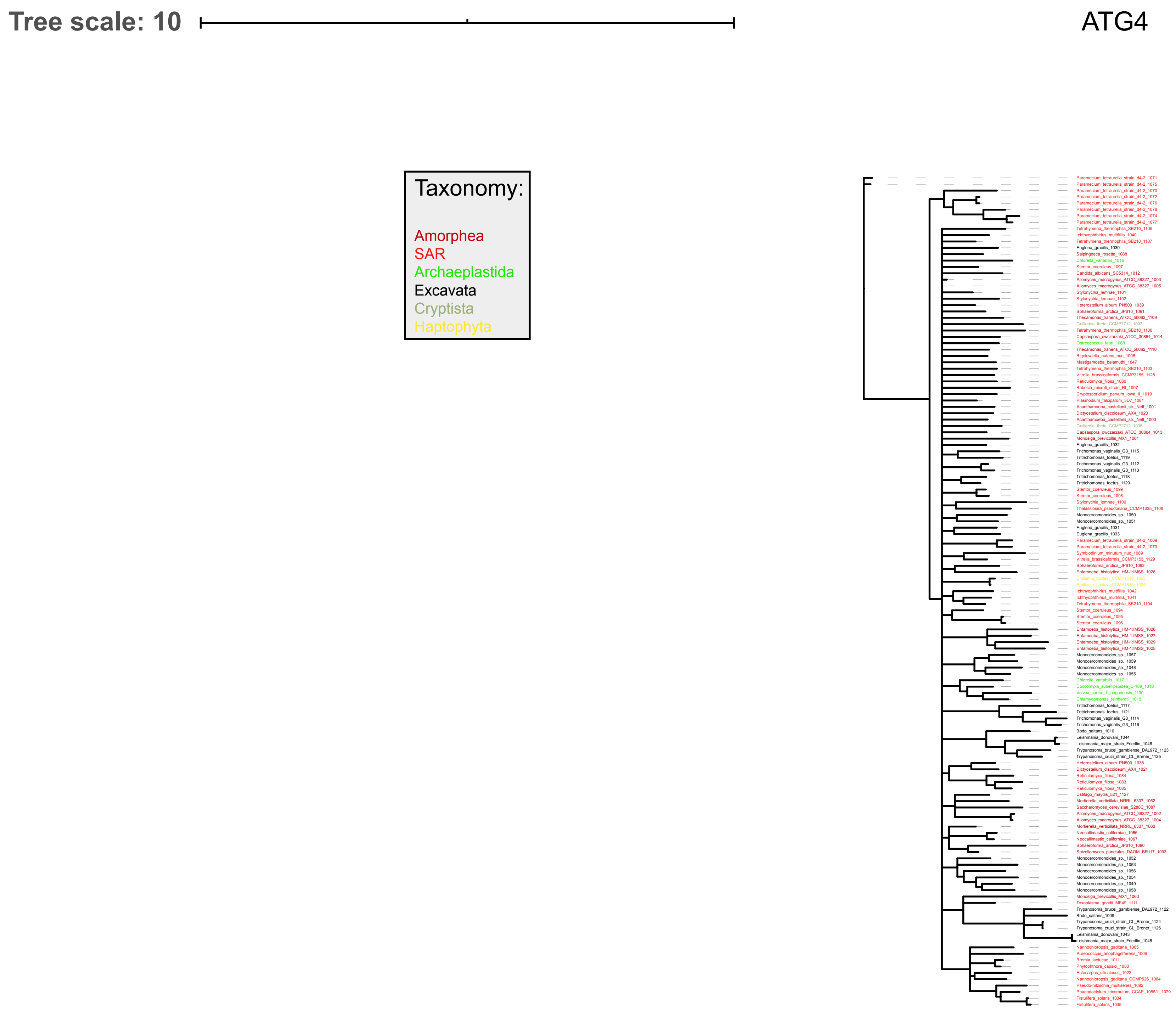
Tree scale: 10
ATG4
Taxonomy:
Amorphea
SAR
Archaeplastida
Excavata
Cryptista
Haptophyta

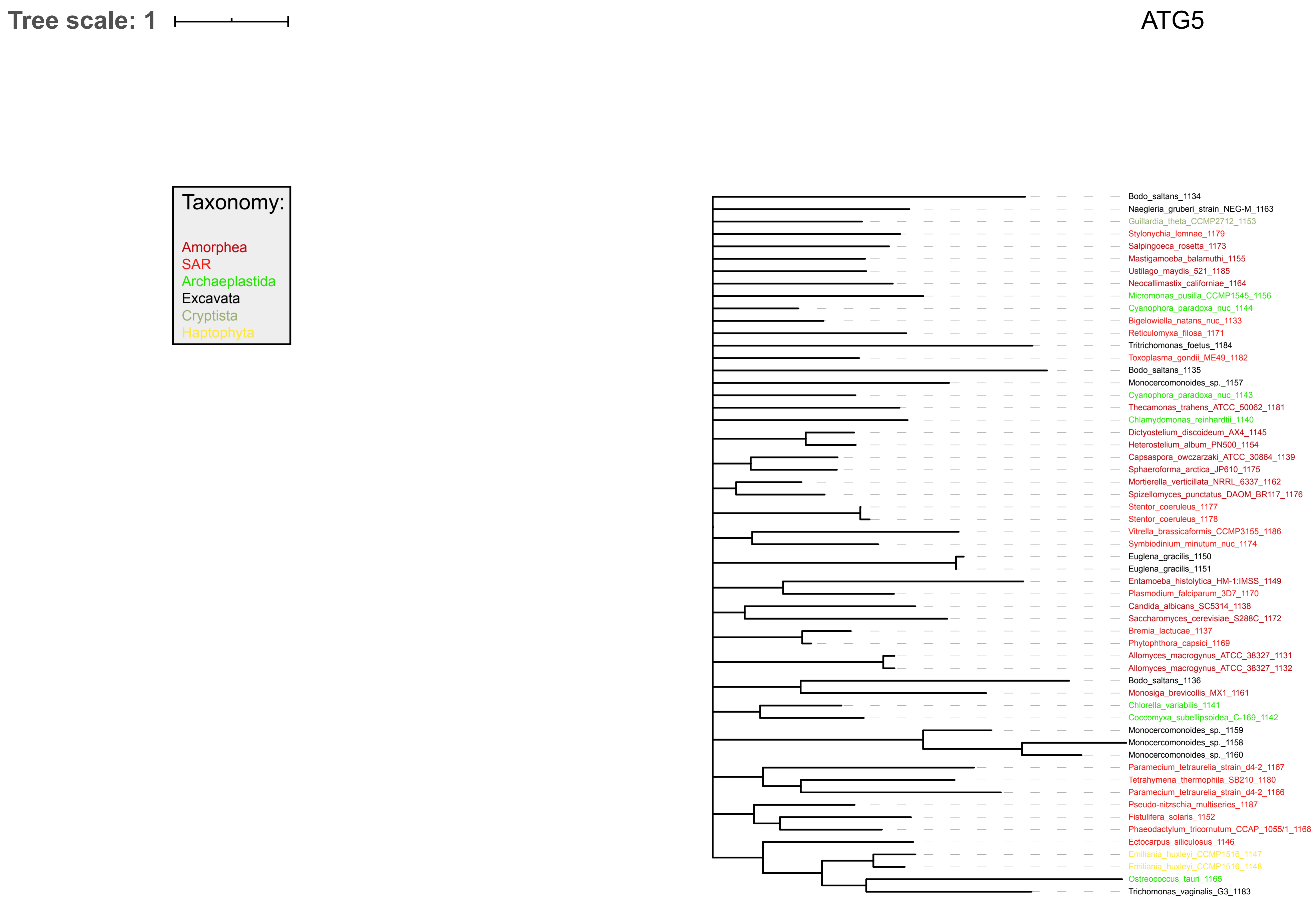
Tree scale: 1
ATG5
Taxonomy:
Amorphea
SAR
Archaeplastida
Excavata
Cryptista
Haptophyta
Bodo_saltans_1134
Naegleria_gruberi_strain_NEG-M_1163
Guillardia_theta_CCMP2712_1153
Stylonychia_lemnae_1179
Salpingoeca_rosetta_1173
Mastigamoeba_balamuthi_1155
Ustilago_maydis_521_1185
Neocallimastix_californiae_1164
Micromonas_pusilla_CCMP1545_1156
Cyanophora_paradoxa_nuc_1144
Bigelowiella_natans_nuc_1133
Reticulomyxa_filosa_1171
Tritrichomonas_foetus_1184
Toxoplasma_gondii_ME49_1182
Bodo_saltans_1135
Monocercomonoides_sp._1157
Cyanophora_paradoxa_nuc_1143
Thecamonas_trahens_ATCC_50062_1181
Chlamydomonas_reinhardtii_1140
Dictyostelium_discoideum_AX4_1145
Heterostelium_album_PN500_1154
Capsaspora_owczarzaki_ATCC_30864_1139
Sphaeroforma_arctica_JP610_1175
Mortierella_verticillata_NRRL_6337_1162
Spizellomyces_punctatus_DAOM_BR117_1176
Stentor_coeruleus_1177
Stentor_coeruleus_1178
Vitrella_brassicaformis_CCMP3155_1186
Symbiodinium_minutum_nuc_1174
Euglena_gracilis_1150
Euglena_gracilis_1151
Entamoeba_histolytica_HM-1:IMSS_1149
Plasmodium_falciparum_3D7_1170
Candida_albicans_SC5314_1138
Saccharomyces_cerevisiae_S288C_1172
Bremia_lactucae_1137
Phytophthora_capsici_1169
Allomyces_macrogynus_ATCC_38327_1131
Allomyces_macrogynus_ATCC_38327_1132
Bodo_saltans_1136
Monosiga_brevicollis_MX1_1161
Chlorella_variabilis_1141
Coccomyxa_subellipsoidea_C-169_1142
Monocercomonoides_sp._1159
Monocercomonoides_sp._1158
Monocercomonoides_sp._1160
Paramecium_tetraurelia_strain_d4-2_1167
Tetrahymena_thermophila_SB210_1180
Paramecium_tetraurelia_strain_d4-2_1166
Pseudo-nitzschia_multiseries_1187
Fistulifera_solaris_1152
Phaeodactylum_tricornutum_CCAP_1055/1_1168
Ectocarpus_siliculosus_1146
Emiliania_huxleyi_CCMP1516_1147
Emiliania_huxleyi_CCMP1516_1148
Ostreococcus_tauri_1165
Trichomonas_vaginalis_G3_1183

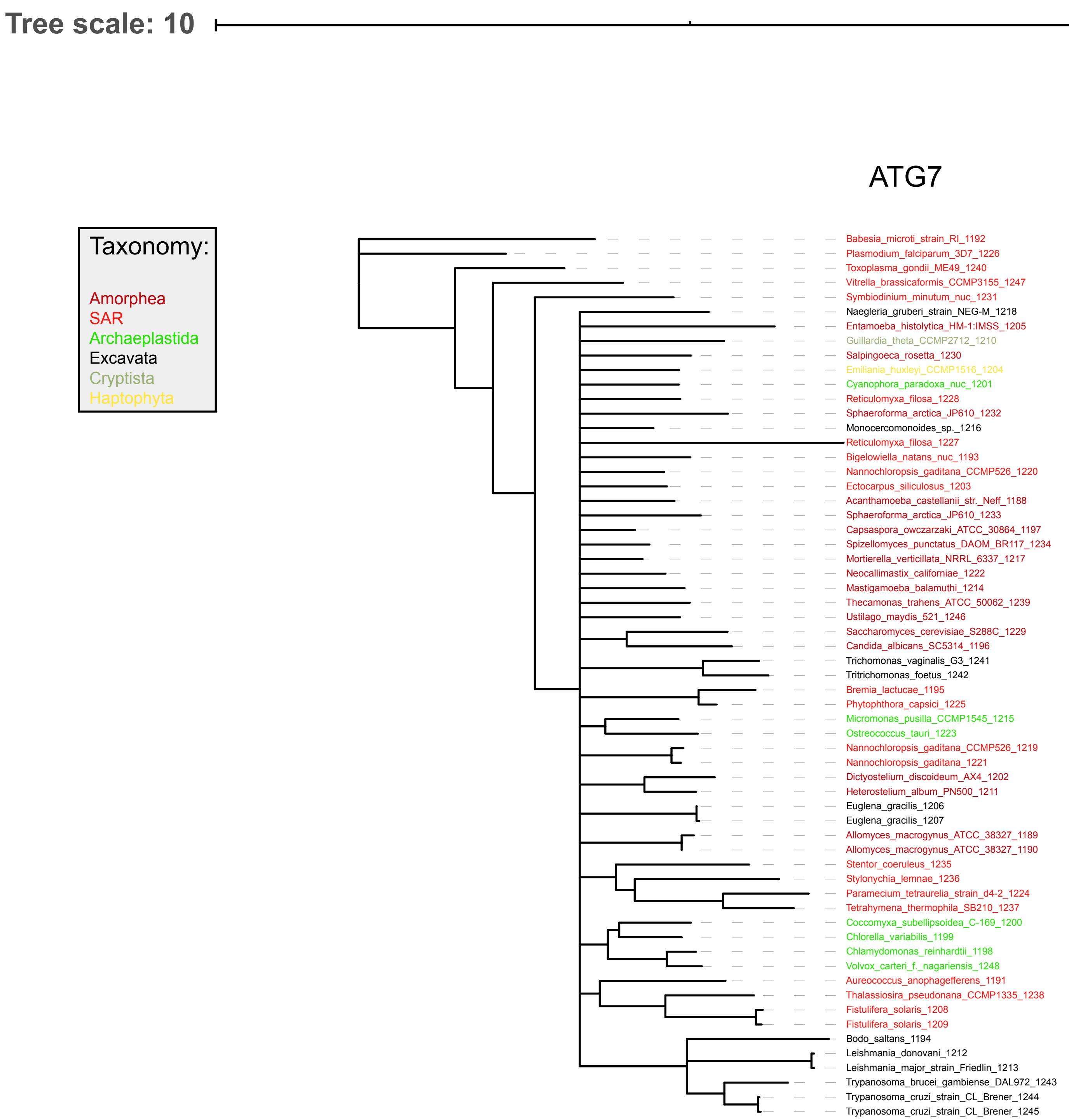

Tree scale: 10
ATG7
Taxonomy:
Amorphea
SAR
Archaeplastida
Excavata
Cryptista
Haptophyta
Babesia_microti_strain_RI_1192
Plasmodium_falciparum_3D7_1226
Toxoplasma_gondii_ME49_1240
Vitrella_brassicaformis_CCMP3155_1247
Symbiodinium_minutum_nuc_1231
Naegleria_gruberi_strain_NEG-M_1218
Entamoeba_histolytica_HM-1:IMSS_1205
Guillardia_theta_CCMP2712_1210
Salpingoeca_rosetta_1230
Emiliania_huxleyi_CCMP1516_1204
Cyanophora_paradoxa_nuc_1201
Reticulomyxa_filosa_1228
Sphaeroforma_arctica_JP610_1232
Monocercomonoides_sp._1216
Reticulomyxa_filosa_1227
Bigelowiella_natans_nuc_1193
Nannochloropsis_gaditana_CCMP526_1220
Ectocarpus_siliculosus_1203
Acanthamoeba_castellanii_str._Neff_1188
Sphaeroforma_arctica_JP610_1233
Capsaspora_owczarzaki_ATCC_30864_1197
Spizellomyces_punctatus_DAOM_BR117_1234
Mortierella_verticillata_NRRL_6337_1217
Neocallimastix_californiae_1222
Mastigamoeba_balamuthi_1214
Thecamonas_trahens_ATCC_50062_1239
Ustilago_maydis_521_1246
Saccharomyces_cerevisiae_S288C_1229
Candida_albicans_SC5314_1196
Trichomonas_vaginalis_G3_1241
Tritrichomonas_foetus_1242
Bremia_lactucae_1195
Phytophthora_capsici_1225
Micromonas_pusilla_CCMP1545_1215
Ostreococcus_tauri_1223
Nannochloropsis_gaditana_CCMP526_1219
Nannochloropsis_gaditana_1221
Dictyostelium_discoideum_AX4_1202
Heterostelium_album_PN500_1211
Euglena_gracilis_1206
Euglena_gracilis_1207
Allomyces_macrogynus_ATCC_38327_1189
Allomyces_macrogynus_ATCC_38327_1190
Stentor_coeruleus_1235
Stylonychia_lemnae_1236
Paramecium_tetraurelia_strain_d4-2_1224
Tetrahymena_thermophila_SB210_1237
Coccomyxa_subellipsoidea_C-169_1200
Chlorella_variabilis_1199
Chlamydomonas_reinhardtii_1198
Volvox_carteri_f._nagariensis_1248
Aureococcus_anophagefferens_1191
Thalassiosira_pseudonana_CCMP1335_1238
Fistulifera_solaris_1208
Fistulifera_solaris_1209
Bodo_saltans_1194
Leishmania_donovani_1212
Leishmania_major_strain_Friedlin_1213
Trypanosoma_brucei_gambiense_DAL972_1243
Trypanosoma_cruzi_strain_CL_Brener_1244
Trypanosoma_cruzi_strain_CL_Brener_1245

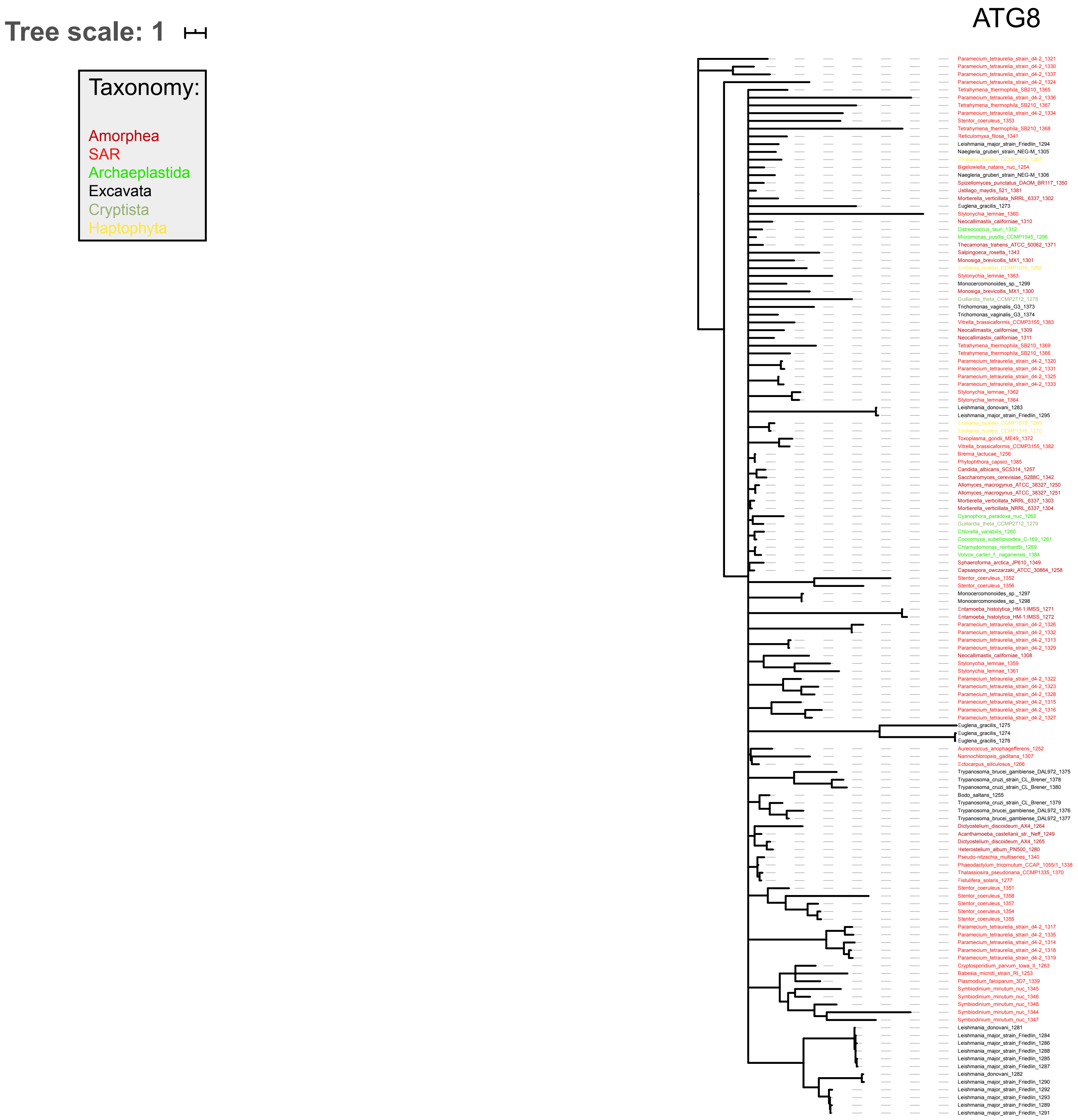

Tree scale: 1
Taxonomy:
Amorphea
SAR
Archaeplastida
Excavata
Cryptista
Haptophyta
ATG8
Paramecium_tetraurelia_strain_d4-2_1321
Paramecium_tetraurelia_strain_d4-2_1330
Paramecium_tetraurelia_strain_d4-2_1337
Paramecium_tetraurelia_strain_d4-2_1324
Tetrahymena_thermophila_SB210_1365
Paramecium_tetraurelia_strain_d4-2_1336
Tetrahymena_thermophila_SB210_1367
Paramecium_tetraurelia_strain_d4-2_1334
Stentor_coeruleus_1353
Tetrahymena_thermophila_SB210_1368
Reticulomyxa_filosa_1341
Leishmania_major_strain_Friedlin_1294
Naegleria_gruberi_strain_NEG-M_1305
Emiliania_huxleyi_CCMP1516_1267
Bigelowiella_natans_nuc_1254
Naegleria_gruberi_strain_NEG-M_1306
Spizellomyces_punctatus_DAOM_BR117_1350
Ustilago_maydis_521_1381
Mortierella_verticillata_NRRL_6337_1302
Euglena_gracilis_1273
Stylonychia_lemnae_1360
Neocallimastix_californiae_1310
Ostreococcus_tauri_1312
Micromonas_pusilla_CCMP1545_1296
Thecamonas_trahens_ATCC_50062_1371
Salpingoeca_rosetta_1343
Monosiga_brevicollis_MX1_1301
Emiliania_huxleyi_CCMP1516_1268
Stylonychia_lemnae_1363
Monocercomonoides_sp._1299
Monosiga_brevicollis_MX1_1300
Guillardia_theta_CCMP2712_1278
Trichomonas_vaginalis_G3_1373
Trichomonas_vaginalis_G3_1374
Vitrella_brassicaformis_CCMP3155_1383
Neocallimastix_californiae_1309
Neocallimastix_californiae_1311
Tetrahymena_thermophila_SB210_1369
Tetrahymena_thermophila_SB210_1366
Paramecium_tetraurelia_strain_d4-2_1320
Paramecium_tetraurelia_strain_d4-2_1331
Paramecium_tetraurelia_strain_d4-2_1325
Paramecium_tetraurelia_strain_d4-2_1333
Stylonychia_lemnae_1362
Stylonychia_lemnae_1364
Leishmania_donovani_1283
Leishmania_major_strain_Friedlin_1295
Emiliania_huxleyi_CCMP1516_1269
Emiliania_huxleyi_CCMP1516_1270
Toxoplasma_gondii_ME49_1372
Vitrella_brassicaformis_CCMP3155_1382
Bremia_lactucae_1256
Phytophthora_capsici_1385
Candida_albicans_SC5314_1257
Saccharomyces_cerevisiae_S288C_1342
Allomyces_macrogynus_ATCC_38327_1250
Allomyces_macrogynus_ATCC_38327_1251
Mortierella_verticillata_NRRL_6337_1303
Mortierella_verticillata_NRRL_6337_1304
Cyanophora_paradoxa_nuc_1262
Guillardia_theta_CCMP2712_1279
Chlorella_variabilis_1260
Coccomyxa_subellipsoidea_C-169_1261
Chlamydomonas_reinhardtii_1259
Volvox_carteri_f._nagariensis_1384
Sphaeroforma_arctica_JP610_1349
Capsaspora_owczarzaki_ATCC_30864_1258
Stentor_coeruleus_1352
Stentor_coeruleus_1356
Monocercomonoides_sp._1297
Monocercomonoides_sp._1298
Entamoeba_histolytica_HM-1:IMSS_1271
Entamoeba_histolytica_HM-1:IMSS_1272
Paramecium_tetraurelia_strain_d4-2_1326
Paramecium_tetraurelia_strain_d4-2_1332
Paramecium_tetraurelia_strain_d4-2_1313
Paramecium_tetraurelia_strain_d4-2_1329
Neocallimastix_californiae_1308
Stylonychia_lemnae_1359
Stylonychia_lemnae_1361
Paramecium_tetraurelia_strain_d4-2_1322
Paramecium_tetraurelia_strain_d4-2_1323
Paramecium_tetraurelia_strain_d4-2_1328
Paramecium_tetraurelia_strain_d4-2_1315
Paramecium_tetraurelia_strain_d4-2_1316
Paramecium_tetraurelia_strain_d4-2_1327
Euglena_gracilis_1275
Euglena_gracilis_1274
Euglena_gracilis_1276
Aureococcus_anophagefferens_1252
Nannochloropsis_gaditana_1307
Ectocarpus_siliculosus_1266
Trypanosoma_brucei_gambiense_DAL972_1375
Trypanosoma_cruzi_strain_CL_Brener_1378
Trypanosoma_cruzi_strain_CL_Brener_1380
Bodo_saltans_1255
Trypanosoma_cruzi_strain_CL_Brener_1379
Trypanosoma_brucei_gambiense_DAL972_1376
Trypanosoma_brucei_gambiense_DAL972_1377
Dictyostelium_discoideum_AX4_1264
Acanthamoeba_castellanii_str._Neff_1249
Dictyostelium_discoideum_AX4_1265
Heterostelium_album_PN500_1280
Pseudo-nitzschia_multiseries_1340
Phaeodactylum_tricornutum_CCAP_1055/1_1338
Thalassiosira_pseudonana_CCMP1335_1370
Fistulifera_solaris_1277
Stentor_coeruleus_1351
Stentor_coeruleus_1358
Stentor_coeruleus_1357
Stentor_coeruleus_1354
Stentor_coeruleus_1355
Paramecium_tetraurelia_strain_d4-2_1317
Paramecium_tetraurelia_strain_d4-2_1335
Paramecium_tetraurelia_strain_d4-2_1314
Paramecium_tetraurelia_strain_d4-2_1318
Paramecium_tetraurelia_strain_d4-2_1319
Cryptosporidium_parvum_Iowa_II_1263
Babesia_microti_strain_RI_1253
Plasmodium_falciparum_3D7_1339
Symbiodinium_minutum_nuc_1345
Symbiodinium_minutum_nuc_1346
Symbiodinium_minutum_nuc_1348
Symbiodinium_minutum_nuc_1344
Symbiodinium_minutum_nuc_1347
Leishmania_donovani_1281
Leishmania_major_strain_Friedlin_1284
Leishmania_major_strain_Friedlin_1286
Leishmania_major_strain_Friedlin_1288
Leishmania_major_strain_Friedlin_1285
Leishmania_major_strain_Friedlin_1287
Leishmania_donovani_1282
Leishmania_major_strain_Friedlin_1290
Leishmania_major_strain_Friedlin_1292
Leishmania_major_strain_Friedlin_1293
Leishmania_major_strain_Friedlin_1289
Leishmania_major_strain_Friedlin_1291

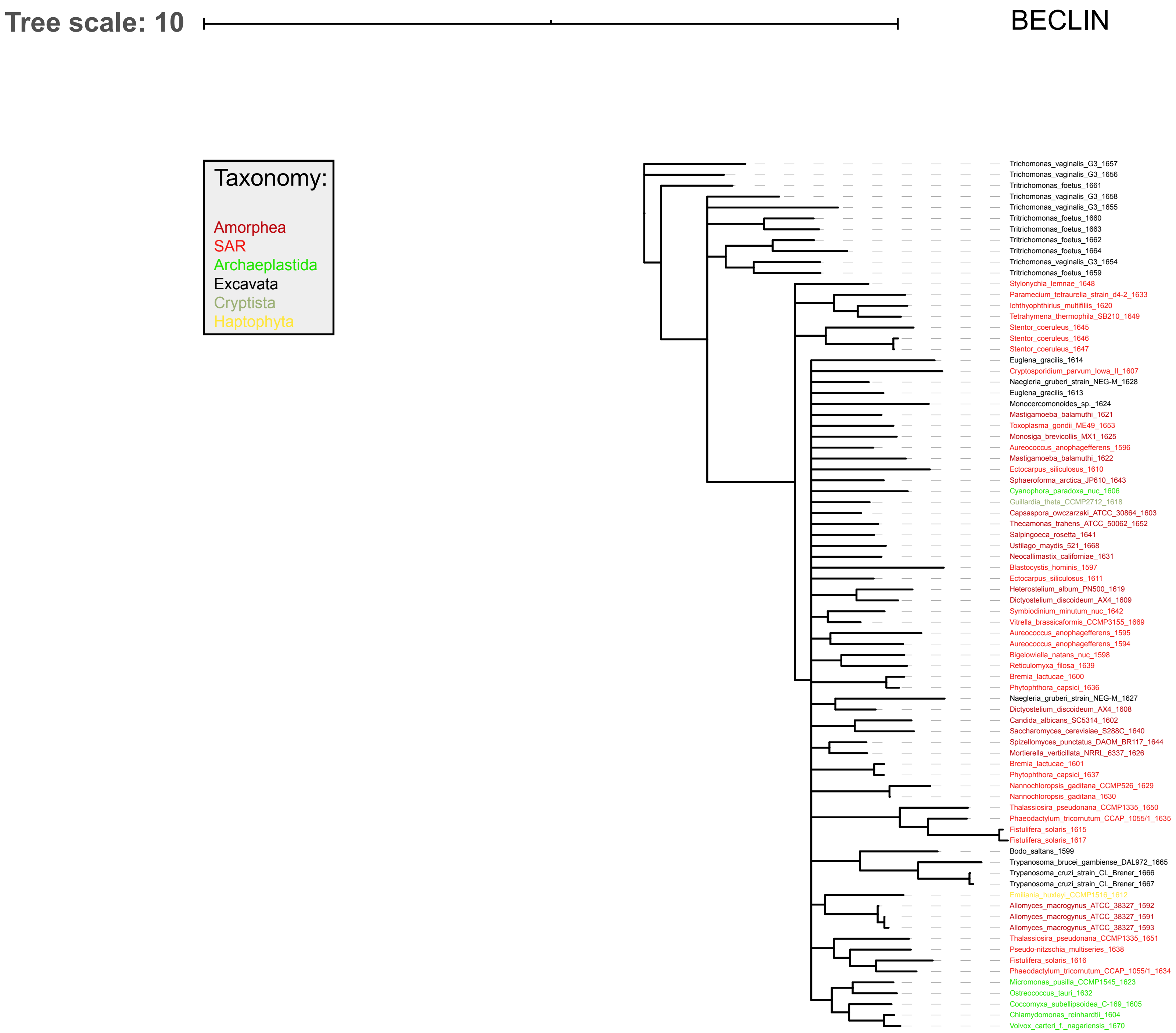

Tree scale: 10
BECLIN
Taxonomy:
Amorphea
SAR
Archaeplastida
Excavata
Cryptista
Haptophyta
Trichomonas_vaginalis_G3_1657
Trichomonas_vaginalis_G3_1656
Tritrichomonas_foetus_1661
Trichomonas_vaginalis_G3_1658
Trichomonas_vaginalis_G3_1655
Tritrichomonas_foetus_1660
Tritrichomonas_foetus_1663
Tritrichomonas_foetus_1662
Tritrichomonas_foetus_1664
Trichomonas_vaginalis_G3_1654
Tritrichomonas_foetus_1659
Stylonychia_lemnae_1648
Paramecium_tetraurelia_strain_d4-2_1633
Ichthyophthirius_multifiliis_1620
Tetrahymena_thermophila_SB210_1649
Stentor_coeruleus_1645
Stentor_coeruleus_1646
Stentor_coeruleus_1647
Euglena_gracilis_1614
Cryptosporidium_parvum_Iowa_II_1607
Naegleria_gruberi_strain_NEG-M_1628
Euglena_gracilis_1613
Monocercomonoides_sp._1624
Mastigamoeba_balamuthi_1621
Toxoplasma_gondii_ME49_1653
Monosiga_brevicollis_MX1_1625
Aureococcus_anophagefferens_1596
Mastigamoeba_balamuthi_1622
Ectocarpus_siliculosus_1610
Sphaeroforma_arctica_JP610_1643
Cyanophora_paradoxa_nuc_1606
Guillardia_theta_CCMP2712_1618
Capsaspora_owczarzaki_ATCC_30864_1603
Thecamonas_trahens_ATCC_50062_1652
Salpingoeca_rosetta_1641
Ustilago_maydis_521_1668
Neocallimastix_californiae_1631
Blastocystis_hominis_1597
Ectocarpus_siliculosus_1611
Heterostelium_album_PN500_1619
Dictyostelium_discoideum_AX4_1609
Symbiodinium_minutum_nuc_1642
Vitrella_brassicaformis_CCMP3155_1669
Aureococcus_anophagefferens_1595
Aureococcus_anophagefferens_1594
Bigelowiella_natans_nuc_1598
Reticulomyxa_filosa_1639
Bremia_lactucae_1600
Phytophthora_capsici_1636
Naegleria_gruberi_strain_NEG-M_1627
Dictyostelium_discoideum_AX4_1608
Candida_albicans_SC5314_1602
Saccharomyces_cerevisiae_S288C_1640
Spizellomyces_punctatus_DAOM_BR117_1644
Mortierella_verticillata_NRRL_6337_1626
Bremia_lactucae_1601
Phytophthora_capsici_1637
Nannochloropsis_gaditana_CCMP526_1629
Nannochloropsis_gaditana_1630
Thalassiosira_pseudonana_CCMP1335_1650
Phaeodactylum_tricornutum_CCAP_1055/1_1635
Fistulifera_solaris_1615
Fistulifera_solaris_1617
Bodo_saltans_1599
Trypanosoma_brucei_gambiense_DAL972_1665
Trypanosoma_cruzi_strain_CL_Brener_1666
Trypanosoma_cruzi_strain_CL_Brener_1667
Emiliania_huxleyi_CCMP1516_1612
Allomyces_macrogynus_ATCC_38327_1592
Allomyces_macrogynus_ATCC_38327_1591
Allomyces_macrogynus_ATCC_38327_1593
Thalassiosira_pseudonana_CCMP1335_1651
Pseudo-nitzschia_multiseries_1638
Fistulifera_solaris_1616
Phaeodactylum_tricornutum_CCAP_1055/1_1634
Micromonas_pusilla_CCMP1545_1623
Ostreococcus_tauri_1632
Coccomyxa_subellipsoidea_C-169_1605
Chlamydomonas_reinhardtii_1604
Volvox_carteri_f._nagariensis_1670

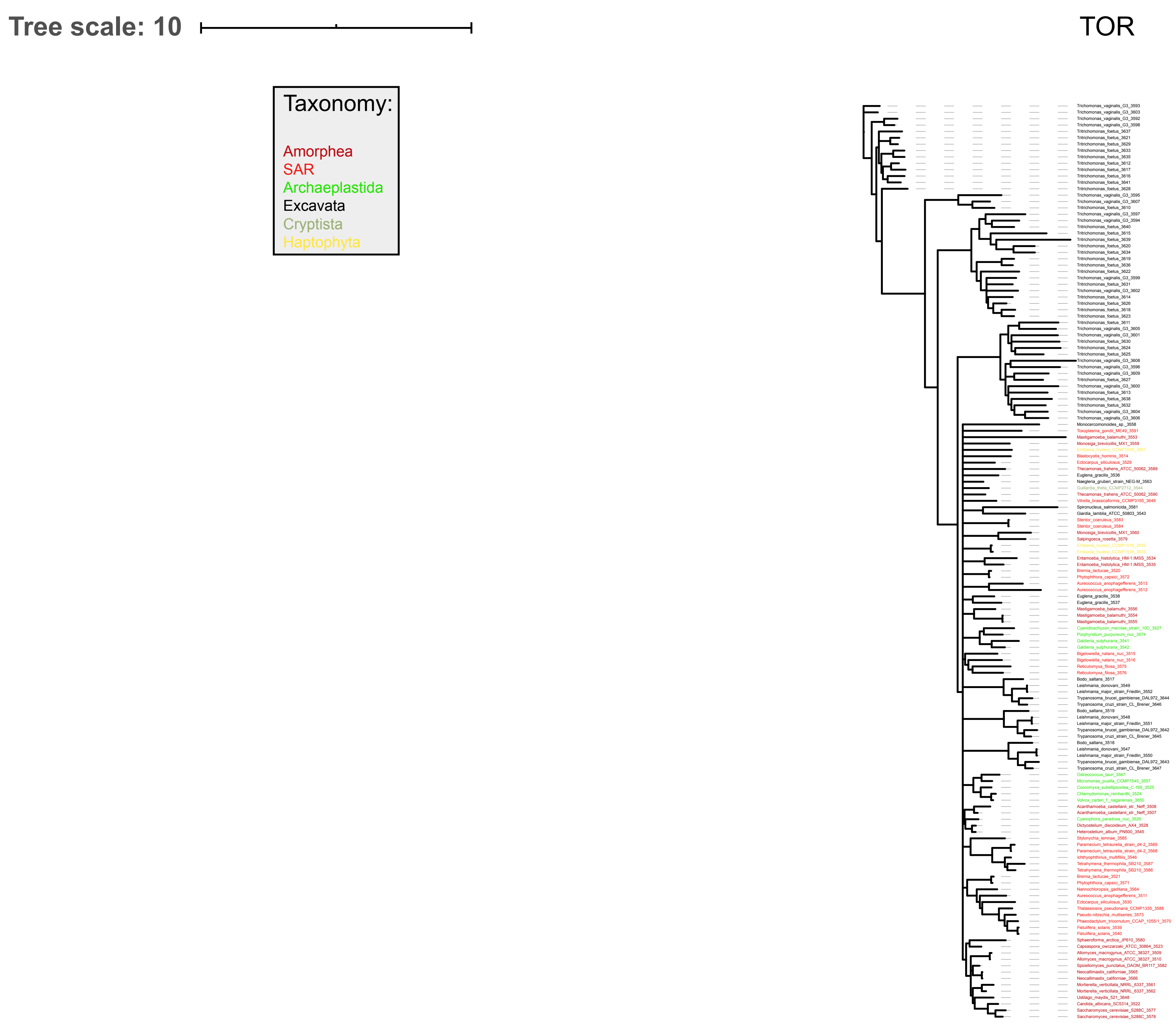
Tree scale: 10
TOR
Taxonomy:
Amorphea
SAR
Archaeplastida
Excavata
Cryptista
Haptophyta
