# Supplementary material for: The Distribution of Genes Associated With Regulated Cell Death Is Decoupled From the Mitochondrial Phenotypes Within Unicellular Eukaryotic Hosts
Source: Front Cell Dev Biol. 2020 Sep 23;8:536389. doi: 10.3389/fcell.2020.536389 (PMC7539657; doi:10.3389/fcell.2020.536389)
Supplement: Supplementary file 2 [file Data_Sheet_2.DOCX]

**Table S1. Organisms and proteomes used in this study**

The taxonomic position, proteomes, mitochondrial phenotypes, lifestyle and published reports of RCD are indicated for each taxon. Supplementary references are below the table.

**Table S2. Summary of orthology assignment by Broccoli**

For each group of putative homologs, the number of orthology groups determined by Broccoli is indicated, as well as the Broccoli group ID and group size (number of sequences) for each ortholog group. Null groups are composed of sequences discarded by the Broccoli algorithm.

**Table S3. Details of orthology assignment by Broccoli**

For each gene set, information on species, protein sequence ID and ortholog group is provided. Sequences discarded by the Broccoli algorithm are indicated (‘not used’). An arbitrary numbered sequence ID has been used to link this information to the fasta protein sequences in supplementary files S1 and S2.

**File S1. Sequences of the retrieved proteins used as apoptosis-associated genes in Fasta format.**

Information on numeric arbitrary sequence IDs is provided in Table S3.

**File S2. Sequences of the retrieved proteins used as autophagy-associated genes in Fasta format.**

Information on numeric arbitrary sequence IDs is provided in Table S3.

**File S3. Phylogenetic trees reconstructed for each gene set retrieved in this study.**

Branches with bootstrap values < 0.95 have been collapsed. Complete trees in Newick format are provided as supplementary materials at <https://github.com/TeamAIRE/Supplementary_Materials_Teuliere_et_al_2020>
