# Supplementary material for: The Distribution of Genes Associated With Regulated Cell Death Is Decoupled From the Mitochondrial Phenotypes Within Unicellular Eukaryotic Hosts
Source: Front Cell Dev Biol. 2020 Sep 23;8:536389. doi: 10.3389/fcell.2020.536389 (PMC7539657; doi:10.3389/fcell.2020.536389)
Supplement: Supplementary file 3 [file Data_Sheet_3.DOCX]

>0

VVVLGSGWGAAALVSALGESYGGGVTVVSPRNYFLFTPMLAGASVGTVEYRSICEPLRSINGAVDYLEATATRIDVERKVVVCEAVVCEGSQCSIDEFEVPYDVVVCATGATTNTFGVPGVREHCLFLKQIADADALRQGLGNCFERANLPTLSDAERRRALSFAVVGAGPTGVEFCGELLDFLESEALAFYPKLVGEASVTLLEATTTVLGAFDASLRDVAVGELEKSRNGGGIKGVDIRLGAAVTEVNGTHVLLGGDDPLPYGLCVWATGNGPTRVVTDTLKALGADGAQGDAQAWARGRFGVDAWLRVLGAPPGEVFAIGDCAADVVDFAAETKATLPATAQVAAQQGEYLARLLKLGPDYDLAKPEPSRPRGAADDDRRLDELFCDERNGHLVARPFQFLNLGILAYVGDGKALAQVALGDGDLGVKAAGRAAFGLWRSVYISKQVSPRNRLLVIGDWLRTRVFGRDIT

>1

MDTIFEFLCGTGAGDAPAGERKKLLIIGGSFGGLVTLRCLKKNGGTKLLDVTLVDAQDYWDYCLASPRCLVDPSQFEAQQFGMPLEGICDHLGATFKQGKVETLTKESATLAGGDVVPFDYCVVATGGSYGAGAIWTARPDEPTAAARKAGFEAEHEALEGCSNVVVAGAGLVGCEIAGEIKAAYPDKNVTLIGNELCPSITRAQAARRAKALEKLGVVVKEGAGRITTEPIDGKITTDKGTTVDCDKLYAATGFKFDPAFAKDLLALDDRGRIKTRGSLQAEGVDNVFVAGDVVAVPPGKVAHIAGGQFAEATCPIVAANLKSLATGSTKLKAYEWPSGPGPVGPFGTNMGRKVCVLDGIPIPAFLRDKMGVAFKNETFWLDTGFDVKGLGRGTTWGK

>2

MLRSSNKVASTRGMQRACFASRTTISHGSKQLQVPSGGRLYSSTTSTGSARWPVVAAITAVGVGGGLYLLQGHVFAAESQPKPKLVILGSGWGALSVVRELDTSKYDVTIVSPRNYFLFTPLLPSVTVGTLEPKAIIEPIRKYCRRSHADVDYFEAVATDVDPTNKTVSCHVSTPGLDDSARDFTLPYDKLVVAVGAINNTFGGPTGVEAAAELRDFVQSNVHKWFPKLEPHVSITLVELMDHILSTYDAKISTYTTSHFKNTNIDIRTKSRVVAVKPGDVIIQRTDTKETQHIPYGLCIWSTGIGTSPLINKIREKLPQDIQTNRRALLTDQFLRVKGADGIYALGDCATIAQEAMLGKLNDLFKEADLNKDNHLQIEEFRSLIDNQEGQYLGKLLNRVANKSVELDTGFHYKHLGSFCFIGSEHAVAEFAEGLVLEGFGAWWLWRSVYLSKQYSLRNKLYVGVNWLKTWIFGRDITRA

>3

MEDTNQIVEAVVAKVGDLKNGEMREVALGDAGKVLLVKEDNQFKAIGNKCTHYGAPLKDGTLCNGRVRCPWHGACFNTSTGDIEDSPGLDSVHSFKVRVEGEDVIVAAPVGELKVWKRTPRVGSCSAADTRVFVIIGGGAAGQSAAETLRDEGFTGRIVLIGKETDLPYDRIKLSKAMTASVDSILLRPESFYQERNIELLLGKEVTELDAEKKTVAIAGGETFKYDGCLVATGGIPRRLPIPGGDLGNVYCLRVPEEAHAIASKAEGKNLVVIGSSFIGMEVAAALVAKAKSVTVVGMEKVPFERVLGAEVGAVLQKLHESKGVKFYLSSTTKEFKGEGSVSAVVLGDGTVLEADVVVVGAGVIPATGFIKGNVDLGRDQSLIVDEFLKAADGLWAAGDVARYPYHLTGEAVRVEHWGMAQTQGRAVARHFLGKQTRQFDIVPFFWTVQFGKSVRYAGHATSFDEVIIQGNPDELKFAAYYVRQGKVLAVAALGMDPLASAAADLLAHDKMPTAAELKAGSADLKALL

>4

MRSRVTTSTARTSTSTLLAVAYRPAAHTATALPLSGPRFVRALATTTTVDGGKQRKKLVVLGNGWAGYRLILDVDISKYELSVISPRNYFLFTPLLTSTTVGTLEFRGVIEPVRTARPGLNYIQAGATSVDTTNKVVTFESVYEERETDEEVPVHPAASIKYDELVIAVGAAPNTFGVPGVEKYCYFLKSVADARNIRQRIIECFERASSPTTTEAERSRLLHFVIVGGGPTSVEFSAELHDFLRKDVHKIYPDLEKQVQITLIEAGKTLLSTFDQRLSDYTMRTFRKRNIDVRTSVSVKQVKRHEMVLSDGAVIPFGLGVWSTGLSPIPFIKGLPFPKDRSGRLLVDEYLHVKAPGVEGVYAVGDCAAFETNPLPATAQGAEQEGKYLAQALNAKARGEEPKKFQYHHKGMLAYVGGYRALIDSPLIKRSGFLTWIMWNAAYITKLVSIKNKMMIPMYWFKSFVFGRDISRF

>5

MALFVWVVLQCAAHYGFVLIFSLFACLLRLWGLAAGLTKRKRSQREREVVIVGGGFAGAYVAKALEDCFRVTLVDNKDYFEFTPSVLRTIVEPNHVNSIQIRHREYLNLKRSRVVLDSVTDVRADHEIGFDYLVLCLGSTYSTPFKASSVIISNRGETLSGCFQDLSAAESVLIIGGGIVGVELAAEVAEHFPHKDIVLVHSGPHLMNGRGTVPPKASAYARRWLESKGVRIMCNERVVEFGTKDCPRFVTDKGTTIEASLAFLSTGIVPNSSFLRDGLLAPYLDPKGFIMVNSHLQLRHHPNIFVCGDVIAVDEEKLAQTAEKHAAIVAKNIHLLARTEDTGAATVAASSKEKVDVDPTDAKLLGKAKDVEATSNKGDLIAATRPLGLAVYDPADLPILISLGKYAGALTWRGWALTGFLPAVMKEFVEWKVMSAYRR

>6

GRGGWRGVGTVAGLVVAAGALGAVALEGLQDDENDSLLSRTPFVSAVRAEEKTTPNAKAPPSIVSQNPPVDRAVQREQQGGADKPMRTKKRVVILGSGWAAVGVLRELDNEAYEVVVVSPRNYFLFTPLLPSVTVGTLDSRSVVESIRRTFKRAGASDVQFLNAECTAINHQSNSITCNDVSGDGAVRSFDLEYDQLIVAVGCDNTTFGTPGVEKYCHFLKELNDARRIRQQITQNFEVAGLPGQPEEEIKRLLHFVVVGGGPTGVEFAAELHDLLVEDLEKWFPRSLTQHVRITIIQSAAHILNTYDAKISDYAEKRFGRDDINVKPLCRVLSVDEKTLSYNDKQTNKTETLPYGMCVWATGTLHGPLFLRGIGPRPLVKKFCSTIKEQTNRRAIVTDSHLRVLGTTNVYAIGDCGTVEQRRLLSKFVDLFDQADENKDGVVSFDELSALVVKNKDEYPQLLIYAAKMQELFETVDLDKNQVLDREEFKALLTEVDKNLVELPATAQVASQEGKYLGQALNALARGQEVEQFHYKPLGSLAYIGARESVLELPGGFSFGGFTTWFAWRSAYLAKQVSWRNKFMVAMDWMKELLFGRDISKC

>7

MLSTTMSTYRVLATSRPAGSHAAITMAALARARRPAVGVVVPASVAHAVSTRRTMATTVTKPKGRLVIIGSGWAGYKLLRQIDTNQFDVFCVSPRNYFVFTPLLAGSSVGTLEFRAIMEPVRQFNKPMQFLQAHCTNIDFDKRQITCTTNLEDSHDTLTLDYDTLVIAPGASTNTFNVPGVREHALFLKDISDARKIRARVIECFEHASQPHVTHDEIDRLLHFQTVGGGPTGVEFSAELHDFITEDLARLYPQLMPRVRMTLYDVAPRILGAFDAKLAEYATKRFVRSGVQIRTGTQVRNVEKDKIVLNDGEEVPYGLLVWATGITQTELVRGLRGRVAPDPRGAHRIVTDRRLRVLDPQGEPMENVYALGDCATISDYDLPATAQVANQKALYLGKQLNKTDEKAIVPFAYRHLGSMAYVGGWRAVVDMPNDIKPSGYSAWIFWRSSYLSMSVSWRNKCLIPLYWFAAYVFGRDVSRIN

>8

MYSISRALRRPVAHPLASPFSQLQMALPNESNSTTVVVYTRPTARTMQLVPAPRLTPWSRHLPWSKQFNSRPRAVMHGKGTKERIVIIGSGWAGYKLVRNIDTERYDVVCVSPRNYFVMTPLLAASAVGTLEFRSIVEPVRKFHEPIAYHQARCDAIDFTSKTITCTSMLGANLDPATAPATSSARPAESVHAQGDVFTLAYDKIVIAPGAATNTFGVPGVKEHAHFLKDVTDARKIRSRVIDCFEKAMRPGVTVDEQRRLLHFATVGGGPTGVELSAELHDLITEDLATVYPTLLPLVRMTLYDVAPRILGAFDADLAEFATKKFMRAGVQIRLGTQVRRVTETSLFVNDGEEVPYGLLVWATGITQTPLVRSLADRVQPDYRGTHRLCTDEYLRVKGKDGQPMRDVFALGDCAFIEGNELPPTAQVANQQAIHLSKQLNKFDESAVVPFSFRNRGSMAYIGGWKAIVDLPTDVHKPSGYSAWVLWRSAYLNLSVSWRNKCLIAMYWALAAVMGRDVSRIR

>9

MSSLPRADPLRVAVLGAGPGGIQTFQGLAKAFASHPTPVDLVLVSERDYFFNNISTPRALADPTGTPVTRLMYPLAQLVKGDGKASGVTKRVLVGRVVSVDTDRQVTVVPSAGRNSLEWIQAASLGVVEGDGSFSADFLVVALGSQAAFPAKAPVGGISSAAMVEEFRGTTAKLADAKVKKVVVIGGGLVGIEMAGEIKSAYPTKDVTIVAPELLPGFPTALIAQASAALARQSITVIQRVRVADVPELTATSSFRPIFRPNFTVPLTPTDSRAPAPASITADAVFVATGVAPNSGPLDPRAWPITDRGYLRVTPDLAVEGVKGVFAVGDIIQYPGKPVGKLAMLAGLQSKVAAANVARTVRGELLKPWKPIPFMAVSMIGRDAGIGYMPGIGTKFGIGAAVAKMLKGKDGGIGLVNYLKETLPALDRA

>10

MLTHASPYCSSAGRPFRADPKTMSLVALVASTTRARAATAAASRTALIRRGVRMYTTEAPVQPAAAEAPRPSKWARRLKWTAYAAGAVAVVGGVLAYRHHPMPQQEPDPTKKTLLVLGTGWGAISLLRRLDTSEYNTIVVSPTNFFLFTPLLPSVTTGSLESRSIITPIKYFLRFKPFGIKFVEAESTQIDPASKTVEILDSGEVHGAITKTRLPYDYLVIGVGAESQTFGIKGVREFGLFLKEIRDARKIRTRLMDCIETAAFPGQPDDEIRRLLHMVVVGGGPTGVEYAGELHDFLRGEVDKWYPEIAPHLKITLIEALPHVLPMFSREMIEYTEKSFADMNIDLLANTAVQEVRAKELVVKDTKSGATREVPYGMLVWATGNTMRPVIKDLQARFADKGQSNRRGLVVDEYLRVAGMPDVWALGDCTATAFAPTAQVAAQQGTYLARQLNDLARTNRTGNDILATCPPFDYKHRATLAYIGDERGVSDVSLPFWSNMVSFAGYQSYLFWRSAYLSNLYTLRNRALVAFDWTKEKLFGRDISRE

>11

MATQLLLRATRARVAAAPAIRAAVPRATPARAIARRFASSESHHQSQKSSSDGSNALWAIASALFFVPAFLAITKPPEKYYAHRSEAVGPAPAHAALAPEVEDKEEETVEETEKAVVVPDRFQYVLIGGGTASYSAMLGIQEVDPTAEILIIAGERYGPYQRPPLSKELWFSEDPEVTSHLQFTNWEGKQTSLFYQDANEYEQVDAADLHPTGSGVKLVTGHRAVALDVDAQTVTLDNGTTVPYGKVLLATGGTPRTLADQKALPEAAQKRVVTYRSVDDFKKLAAVAAQGKHVAVIGGGFLGSELSVALAKKTKVTQVFPEDGNMALVFPRYLARWTTQQVQKEGVAVLSKSKVTALKYDAESDQIVIETNGVQHKFDHVVVAVGIEPNVELARQAGLEIDPVRGGIVVNAELEARRNVFCAGDNVSFHDVALGRRRVEHYDAAVLGGRWAGRSMAGKPKAFTHQSMFWSDLGPKIGYEAMGILDSSLVTVGVWGQQPAADVPAAPAAAAEGEDKAAVPAAPAKPKDDFKKGVVFYVRDDTIVGCLMFNIHGKVDVARRVLLGKHKPAEANELPSSSTSLRKLWAREDAERVRQESKELKA

>12

MYAVLGRHGMLNREINCLYHICKRFTGQNSIKNNVNGKSLPKVVIAGSGWAAIHFAKQLNRIKFDTYIVSPKNFFTFTPLLPFVSSGKILPEACTESLHYLFNGTQPKLIFSEGFDVDFDGKSLICHNISANNDSVEVTKIPYDYLVIAVGAVTNTFNIPNVDKYAYFLKDISDAKAIYNRICSNCEYASYPNLPVQKVEDLCRIMIVGGGPTGVETAACINETIVKSLSIQFPHLKQYLKIYLVESGSALLVTFSPKISKYTLKTFENNDIMVKLNTRMERVEQDYCEFVDNVTGTKTRIGHGIVIWVSGLTGRPFTKKLIEKLSKSGMQNQRNSLSVDQYFRVRGADDVFALGDCAQMIPDKMSDQAEAIANLLGNKLTAKKLNSFRNVLLNKYPQMSKLKWKGPYNNDNLSLDEFKKLLIGIDSGFRGPFPTAQNAKQEGIYLANVFNQFLTHNSCGLNTYELLGQNSLYVKPFCEVWKGSIAYVGMNRTVFKLPFIELTGKLLLQTLWKFITIDMLFTYRSKTALLLSWMLDRMFGKARANLQHSKPHYK

>13

MRIGQIIAVRGCNHPLIGYKLSRLHGISIKFVTQNHYSRTYDAKPFCVRHFGSSNSKFPPLFGCKPKICISKKLGVFLFGSASLYIAYNIVNECLCESSSENCKNGSCTVGETKRILVGKVNEFDNGGIYEIKTGDTGEKVLLHNIKGKFYCTGAYCPHFSAEFTQGICTDELLVCPWHNAKFKISTGECISGPILDDVPTYPVEIVEDQLYIRVPKNTGSGNGNSAVKKKCNPSVGENSTFVIIGGGAAAHSAAETLRDEGFSGTIKMYSADAYEPYYRPALSKSIKTSDPKKLHQEHILKDPGFYNARKIEFYPNTPVKHVDSTTKKIILSNGTAVPFDKVLIATGMEAANLKIQSRSSANNILTLRGLKDVENISKFAKKGKRIVIVGANFIGSELASSLINSGAHVTVITDMEYPMENVFGRRVGSAILTLFDQNNVKFIAKSLVKNYHLNGNSCNAVELNSGQKVAADCVIEGVGSIPNRPSIDGNNKGFIRVDSLQRVNGNPDVYAAGDVVEFPYFVTGDYINVQHWNVAMQQGRVAAINMLGRVTTNTQIPFFWSTIFRKNLRYSGFVKDFDDVIIEGSVKDLKFVAYYVKDQKVLATLAVGNDEFGAAASEAIKHGLMPTKAELMLGAKNSRDVIQTLQNRRK

>14

MSSSSLSSLLAIPRSLRRLGFGAATLVGGSYAISLYINDDFDDVIPPKWLRVPTAHGDVPKQKLVVLGSGWGAMSLIRKLNLEDVDLTVVSPRPYFFYTPLLAGSATGTVSHSNIQESVRHAVVKNGEERGRYVQATCENVDFRNKTVKCSLGGDTDSGVEIPYDKLVIAVGAQPATFGIKGVKENALFLKELEDGVKVRSKVLSLIEKASALQNISSAKSKEVPENAERENEIDRLLHFVVVGGGPTGTELCAELSDFLRKDVARGFPEIASRVRVTLVEGLPRILSMFDEQLASYAQSHLQKSAIDIKTNKFVAEVGPTTATIKDAKSKELHTLDYGMLVWAAGITTRPVVSHLIAAVGKEGGQNSRRGLVVDKHLRVKGTKEGDVYAIGDCAVSGLPPTAQVAAQQVAEGVVKPTTRFESNNINKTLGKYLGRILRKEFQQRRQKGAIVTEKDDDDNSAQVAAYDEGAQFVVPEFTYNDKGKMAYIGSGEGVVEIGSIDSMCLLQGTSSSSSSSSSSKKGGDPTQAIDYNFWRSIHSAVGEPRIVGQSGFMIWRSVYFSMLLSARNRMVE

>15

MNAIHKELKTPFPDKTLIFLPTQFRKLTRLRVVLPALQRRNLATAGGTQGKPRVVVLGSGWGGFHVAKNLDKDKYDVRVISPANHFLFTPLLPSTAVGTLEFRCIQEPVRTIPGLGEYYQAKARTIDFDTREISCSDIFKEKEFKIQYDFLVIATGAKTNTFNTPGVDEREGDEVMFLKHLYHSRKIRNRILECFERASVPSNTAVKDRDRLLSFVVVGGGPTSCEFTGELHDFVREDCTRWFPDLKEHIKITLVEAGPALLGGFEKSLSKYVLSDLKKRNVDIRLGAQGRPGVQAILADGTKLPFGMMVWSAGLKQVKFVDRLNLAHGRTGRLVIDEYLQVPQHQGRVFALGDCAANEENPLAPLAKVAQQHGEYLAKILNTHPDPQQPVDPASVSKPFRFFNPGSMAQLGLFKGVLDLSNVGPDKGEVHGGPTITGFAAWLTWRSAYWGKQVSLRNKILIPMHWFKSFVFGRDICRF

>16

MGSRGSKEVKDKPVVLIVGGGYGGVAIAKMLDKDAAFNVVIIDRKNYFLHNMALLRATCVEGWEQKCIIPYTKVLKYGSVVQGEVEAIAKDGKSIKVYGSSFILSFKGREEKEIQCDYMVVCTGSSYAFPAKVAEVDATSVPAKYSKMRQDVNAAENITVIGGGAVGLELVGELSHYYSKKSITLIHTGETVLNNTALGDKMKAKTLAAVQALPNVKSCNHKVMLGERVEFSELQSEIKESSVSYVEGKRELTTSKGNKVGTDLVFFCTGTKTNTKSFKDNLPVDEEGRIKVNEHLQIEGCKTAFALGDCAAIGPKSVYDNDGSGFFIRVAIHIILNCINAATADLSISLPLKALEKKKGMSAYKKGPTMMVLPLGPNGGNSQLPMGIVVGNFLTRTIKSGDLFSSNTWGELNQDIANPQLKKGGLNRRNSLQSLEVHGALKIDEEKAKKILEEGSGKEKKKNKHKTHKRAESASQLPIKSYAVIMSSYYTYDFMQYDSGGARPSADLRMIFG

>17

NSHRIAVLGGGFGGLYTALKLAELKWEEGAKPEVTLVDVSDRFLFKPLMYELITGEATLDQVAPLFRDLIGISGVRFVQHEVKSVTLSDTANMLAGQVKLDDDSTIDYDYLVLGVGAEANLELATGAREHALAFNTLDDVRKLEQRLQDIEDAFADKRGDTVSVAVVGGGPSGVELAATVSDRLQKSLGREAVRVTLYVAGADLMETFAPEARDVARETLASKNIEVLYNHRVEEVGKATVPSPSSSGSKTIACDMVLWTAGAKPIGVMGIPQEDGANVPITIEKTLKIKGRDREFALGDVAGMGLPATAQVAMQQSDYCAWNIFASVNAAVGDDDTKLLNFRYQHLGSLMALGSLSGTATLNLPGVGDLTLKGPLASALRKMAYVYRMPTNAQRLKVGSEWLQ

>18

MWRSWIRSPFPAAALAAGISTLSLSSSNDPVPSPSDVPLEGPDALSPPGPPSAGGEKLHLARRFTRRKQTKHHSYVIVGSGTAANAAIEAIRQEDASADILVLSDENALPRLDFGVGNDNGDEGGNERPLAPALLESYNEWRRHLSARFEEEVASSAGPPPLTLLLDKRPLEFDVEKNCVFLGDGTEVRYDRCLVASAGRPRHFYVLDSDRVSYALKDRVNTCTTRADFVRLDQLADGASEQNDSALQSVLVIGAGFLGCEVACALATDQRNNHLKIKLVFLEQLPAARTLPPYLAEELARRLNCAGVDVVPNRLVTSLRPRLDDDENESDSGVTVGLLAKDKPEAALDADYVVLASTHTDPATTLRMGRATRTGGGLERDEKNGGLVVNAQLEAISGLFVAGNAASYYDPYLGRRRVDRYDHAVNSGLTAGRNMAKSLRGAGKMKTYRHQPLFRSHLPGVSLRMEGIGEVDSSFRTVGVWVQPPCIGVDRNGSQRSSYERGVVYYLKGNKIMGILLWNASDVLESARQLMLSRPEIRDNVVEELKHTISLAPNDWLHVVSTS

>19

MFKVADHCLSDLQHFLYTPLLASTTVGTLGFRSIIEPLRDSWLSHESDFHFANVQDIDPEKKILKVTSAINAASRSPTYDIRYDTLVLGCGSRPLTFGLPGVEEHAFFLKEIQDSQKIRNRILENFEAATQPGIAPEEKQRLLHFVVVGGGPTGIEFCAELYDLVQQDLVHKYPETSKHLQVTLVDSGEILNGFDKHLRAVALRKIQKRSTMKLIKKNCIEVSDRGVTLEGGEIVPSGLVMWTAGVGPNELTRSLTVFEKSKRGNILTNQYCQVLGAPEVEEKAPWGLPRRSSVFSIGDCAEILDYPLPATAQRAQSQANYIAKLLRGKHTTPAIPYAFHSKGMMAYLGSYEGVFEAHPRDDEKITLTGWKAWFLWRSAYFTKLGSWRLRLQVPLDWLKAMLVGRDISKF

>20

MRWLLAAIGVFGGSTMASCRSVTYAAEAPRLDDVMDPTKDVKPMNCVVVGGGYAGGKLAYQFDSIFSTTLIDSKNYEEITADIIPILTTPWCEKNDEACRKLHVLHRYYLKRANVLTAKATAVKDDCVVLEDGRTIPYDVLMVTTGEEKSFPFTTTQKTISGRVAELKAFNQFLSTTKKIAIVGGGPMGVSLAARFAEDRPEIDVHLFHAQDSLLPALPDVASQYANNSFVDKIYVSVHLCSNIVDLKSNAVPVQQTFLDKLLRRPAVETVGDKFSVFVERLQYHPVAPQSVLSQAYFGRRKQAQTSDVISSKWLSDFDYVFNVGGDTPRPISYKPECILAKHLTPDGHYRVSSLMQLFGLPHVFASGRCTNLPGARTLGSSDLESRTIFRMLNGIINSNQEKTLRSSDGIRVDRLEVPRLLLQLGSMDACGSTPWSGALTGLSALREFIQDRGHFQREFSFPVFYKQQDPQRVRSRIDQWKSVEMTDITDFSHGNA

>21

MRRGFTVRFSASPLSAHSVTMQRRWSTGSKPTVVVIGTGWSGAYFVRDLDPNQCNMTVLSQRNHMVFTPLLPQTCSGTLEFRSVCEPIQRVQPALAKLPNRFFRTLVFGVDFQKQVVNCVGVGVLGAGNDENVPVQSFDVHYDKLVFAHGARPNTFNIPGVEDHAFFLREISEARGIRRRIVQNLMTADLPMTDIEEVKRLLHIVVVGGGPTGVEFAADLADFLHQDIPKIDPALLKYCKVTLVEAGEILGSFDLTLRNYGAKKLTKMGVHLRKAVVAGVTEKAVLLTDGEVLTCGLVVWSTGVGMSTLSKELAVDRNKQGRICVDDELQVLHEGKPVPNVYAVGDCAANVKAPLPTLAAVASRQGTFLAKKMNRLLRGQRDTSLFKFKSLGSMVSLGGKDALIELKQPNSFDITGLKAMYLWKSAYFSMLGSYRSKLYVLVNWCGSKIFGRDITYIAELSETKTWRMLAREEASRSQARLKAMKKLKAGEVAAAIPTVTSETTPASSAPENEVKK

>22

MRSAIKPIIIRNLGWRTHAQTHFHTLVGFRFCYIRWAYFEFQLLLFDFLLPSKGPQNLNIINTDYKKKSFLSTSFSLLLSYRRITFSNKKNTMSSKTVPRFPIIRKFFKYTLSGVALTVVGGTSFIAYKVYQESQPVDQIKQSPYFPNGQPKKSIVILGSGWGAVSLLKNIDTSLYNVSVVSPRNYFLFTPLLPSVPTGTVDMRSIIEPIRSMIRRCRGEVNYYEAEAIGIDPVNNKLTIQQSTTVHSGHSGDDTSSNDPKIHQEHKMEHITTELNYDYLVVGVGAQPSTFGIPGVAEHSTFVKEVRDSIKIKKKIIDLIEAANLLPVGDPDRKRLLHIVVCGGGPTGVEAAGEIQDYIDQDLKKWMPQIAKDMKVSLVESQPVVLHTFSSELVEYTNHIFKDTNINLVTNSRIVKVDDTHVDVMRKSDKSIDKVPYGMLIWATGNSVRGFTKIIMDKFSEQQTSPRGLLVDDQLKLKGSDNIYALGDCTFTKYAPTAQVAFQQGIYLAHYFEKLQKVEKLRYKIKQDPSISEVYVHRLQRLENSLPKFVYNYRGSLAYIGSEKAVADLAVGSWSNLSSGGNLTFLFWRSAYIMMCLSIKNQVLVCFDWIKVYLFGRDCSRE

>23

MFTRSLIKGGGRLATTRSLVNNSTSLVLKNQFKKYSTSTPPKVAKSKSSTIGKIFRYTFYTAVISVIGSAGLIGYKIYEESQPVDQVKQTPLFPNGEKKKTLVILGSGWGAISLLKNLDTTLYNVVIVSPRNYFLFTPLLPSVPTGTVELRSIIEPVRSVTRRCPGQVIYLEAEATNINPKTNELTLKQSTTVVSGHSGKDTSSSKSTVAEYTGVEEITTTLNYDYLVVGVGAQPSTFGIPGVAENSTFLKEVSDASAIRRKLMDVIEAANILPKDDPERKRLLSIVVCGGGPTGVEAAGEIQDYIDQDLKKWVPEVADELKVSLVEALPNVLNTFNKKLIDYTKEVFKDTNINLMTNTMIKKVNDKSLIANHKNPDGSTESIEIPYGLLIWATGNAPRDFTRDLIAKVDEQKNARRGLLVDERLKVDGTDNIFALGDCTFTKYPPTAQVAFQEGEYLANYFDKLHAVESLKYTIANPTPKDNVEKLSRKLARLEKNLPHFIYNYQGSLAYIGSEKAVADLVWGDWSNISSGGNLTFLFWRSAYIYMCLSVKNQVLVVLDWAKVYFFGRDCSKE

>24

MRLRASTKPVVLVLGSGWGAHSLIKVIDTDTYDVVVVSPRNHFLFTPMLPSTAVGTVEFRSLLEPIRTSNPCVTYLEAECDSLDPHTKVAVCTSSFAYDDGRRPQFEIQYDKAVVAVGEQPATFGVKGVKEHCFFMKEVSDAVALRKRIAEKFELASLPGTSEEDRKAALHFVVVGGGPTGVEFAGTLSDFVREDLKKKYPALMKYVKVTLLQSAQQILTQFDAGLGQRATEALESSGVEVRTGVRVVEITINKVMLVSDAQVLLKGGEELPYGVCVWSAGNAPRPLVTQIASQVAEQAAAAEAPPNAKLCVDSFLRVVGATDLMALGDCSLVLGNRLPSTAQVAGQQGAYLAHLINSQYQLGVGGYTQPPPFQIVKRNKLQVWAGAGGTGAYALQAAAEENAVLSWLTTAMMGGKTRIQASKEVADAMFRMDAPPWVRVHSESLTKATPLDKEAPPGGAKAVCDISAVMAAAAAAGGADPVAVARASADCLRSLEEEELKAREGAEATEVRYYDRPFEFLSLGIMAYVGNDKALTQVEAFDVINLKLYGSVAFLLWKSVYITKQVSFRNRVLILFDWMKTRVFGRDISLF

>25

MNMLLQQQKLAAGCKQRSVAQPSRGCVAAHTGLRSGRVASRQRSVTTAVMTPPAKSESSSPVYTTMSLDGQNLKTAKPRLVVLGSGWGAMSFLKALPTSISSTYELIVVSPRNYFLYTPLLPAVATGTMEERSIVEPVRNFIVGKGEFYEALCKDIDPVAKELVCCFPEDAGLDSACFKMSYDVLVMAVGSVNNTFGIKGVDQYCFYFKSIEDANRLRSRVSECFERAALPATPEEERKKLLTFVVVGGGPTGVEVAAELYDMIEEDLSKLYPNLVSIQVVELMDHVLSTYDRAISLYTAEQFKRAGIKLVLNSRVASVEDGVVRVVNKANESVDIKFGACVWATGIAMNPLVRQLQEKLPGQSHFRSVLTDDCMRVKGSDGSIWALGDAATIDQPKALDYAEQLFEQADTNRDGRLSLEELRVLLNTASKEFSHLEEHARFLDSQTGVKRFGGLVAKSLSPADAAAAAASNSSQPFAVLLDGNTEISKEQFKDILGKVDKGLRALPATAQVANQQGKYLAAVFAGNRVTGAPELDAALADKIKPFRYFHKGSAAYVGSDKAVFDLPKFGPLTGTGAGFVWKSYETMSQFSFRNQCLVAADWLRTKIFGRDISRV

>26

MAASALLATVASSYIRRSSGVGTSLLGAFSNDHRPCGTAVYQHLRRLAAQAQASKKTASSDQLPLKTGRQRLVVLGSGWAAARLLHDIDPNLYDLTVISPRNHMVFTPLLASTTVGTLEPRSVAVHLHEIQPCLSRPSSSVFIADAHAVDAASRTVTCRSVDGLDFSVQYDKLAICTGSQGSTFGIPGVLENAHFLRDVKQADAIRQKLIENIALAGVPGRQQDEFSRLLHIVIVGGGPTGVEVAGELTDFISHEVCVDVRVTLVEARELLGSFDASLREYAARKLIQGGVLLRKGIVHEVTPREVVLKDGTVLPYGLCIWSTGVGPTPFSLSLPFAKTAVGRIAVDKYMRSDTPSTAGLAPVPHVYALGDVCANPDKPLPALAQVAEQQGRYLARVLNELARGPPHVGGRQGRGGGVPQHTEFVYRQLGAMATVGGHSAVLELGDAGRRHLSLAGFLSWVAWRSAYLTRLGSIPKRLAVAFDWSITMIFGRDLSRW

>27

MLHAVRLGLYTIGAGTALWLGYRSVQVLNPPQPAGPPSLRPSARPRLVVCGTGWASHALLRSLDPRLCDVVLISDRNHFVYTPLLPSASVGSVELRSIVVPARELLARLQRRWWHWPQLLTESGQAVPATEWSFINARVEDVDPLTKQVHCSAVHGGARFSVPYDVAVLAVGSGTNDGGFPAVRSCCHALRSAEDARAIRSALNDALEGAAEPSTSAEERHRLLQFVVVGAGPSGCEIAAELHDFLHEDARRLFPRSLLDDVCVTIVQSGATVLNGFEKRIAEYATEKFRRDGIQLLLNHRVVEVTSDALTVMDKFSQEAQTLAFGVCIWTAGLAMHPLIRRVAERLGAQAQSNRYALVVNHHLGVVGDPHRALYAAGDCSTLQSAATQSHLDKLFQLADIDGDGRIHVYEFLRFIRIVRDEYPQLAEFVSMIEPDFHEGQSSINREQFQKWLVKVDEHSTALPPTAQVAFQQGRYLGRLLNERFRCWPAGFDELEAGLYPPFEWRNLGAAAYLGNSVSVLQFPFMDPLYGNVAFWLWYGYSLLHLFSWRSRFLVMIDFVKTRVLGRDISKF

>28

MLEVHRLVTRSHLSSFVCFLEKLGNHQRLFGKGVSAPKVKGSTRLSSVCKRCLVMSATSVKSYEYVILGGGNAAGYAARQFVEKHGLSGHKLAVISRESVAPYERPALSKAYLTANPPTRLPAFHTCVAGGGAPQTPDWYAKNGIDLLLSTEIVDCDLNSKCLTAKDGSKYGYGKLLIATGSDALHLDELGMQGAHLGGIHYLREIAEADKLYEAMKACAGKHAVVVGGGYIGLECTAALVINGVRVTMVFPEPHVMARLFTPEIAAHYERIYAQKGVNFIKGTVVDSFADENGSGQVKYVRLKNGPVLEADLVVVGVGAKPRTTLLEGALAMEARGIKVDGHLRTSHADVFGAGDVITFPLKMYGNRMARVEHVGHARQSAMHAVDVMMGATTEPYDYLPFFYSRVFHLSWKFWGDTPAQAKTIVVGEMNPKLVAVWIDQDGHVVGTFIESGTEHDENKLKELARTRPKANVARLEEAAAANDVDGFLNAL

>29

MPLLYGASCSDMDEGYLITGFVSSVHALGRQRQVPLVSRKPNTPNFRPQRSARVVQRTHKLRGLRLCEGERLVKTDEKPAVQPDTQPLKPGDGKESPAPPSPPPPVETRGRRRTYLGLVWDRTLDTFEDALLHLRRQFFWQGEFLSSSGRPKPRLVILGTGWVGHAMVKIIDIDKYEVIVISPRNYFLFQPMLPSSALGIVEFRSCCEPILRANPFIIYYEAEAVGVDIQRRVVKCRAKVRRRGALSVGSESDVGAPSLAETSQDVHALQPRFSTKSNTDEIIGIREFEVPYTYCVVGVGSAVNTFNTPGAKENCFFLKEIPDARKIRSEVVRIFEEANLPETSDEERSRLLHFVVVGGGPTGVEFAGELHDFLVEDAVKYYKKLLKYVQVTLLQSGQSILTQFDKSLQDRALQNLRDAEINVRTGSRVVRITETEIYLQDGAVIPYGMCVWAAGVGPQKLVTDLIESIPAQTTFKKRQLVVDDWLRVIGAEGVFAAGDCATNLHEPLPATAQVAGQQGAYLARLLNREYCLDCDIPERTEYTRTWIDRARFAKPFQFLSFGLLAYIGRERAMAQIEMGDTSVKLSGTLTYLIWRSVYAVKQVSMRNRILITFDWIKAAIFGRDISQF

>30

MSERAILMRCDAILYSPALGRRSAHSIASSQNDQNSNNNQNHNHNQSNQSSQGSFRGAAAIALATVAVAGTMGLFAVVTAAEADIDSRRRKPRLVILGSGWAAVGVIKGLVPGEYDVTVVSPRSAFVFTPLLPSACVGSVESRSLVESMRKMCANAQAHFVQAGATDVDFGRKTVVCKDEHDQLFELPYDRLVVAVGAHNNTFNTPGVEKNCHFLKQVQDARDIRAKIMDNFEQAALPTTPVDEKRRLLHFLIVGGGPTGVEVAAEIADLVRDDLVHLFPELCQKYVSVSLVQSADHILNTYDESISLYAEKKFKMQNINVITRARVLQVNPTSVEYTERIDGKDVPKTLNYGMCVWSTGIKQVPLVETIATHLDKSQNHRRALVTDSRLRVIGAGGDMFAIGDCATMAMPHLLTNVKDVFSEADENNDGVISYEEFEHMCNRAVERYPQMEMHVRQLKKLFSQYDADDNRSLDLAEFGKFLADIDKQLKAFPATAQVASQQGKYIARQLNYLALQDRRTFLAQQEGGPASTATAASEKAAAEILHQTAKRQAATGIEGNAVAVAAVLPEGSRVVTTAQDQERLARQATALAYKRPYKDFEPFHYHHMGSLAYIGHEDAAIDFGGGITGSGTAAFFLWRGAYLSNSVTVRVRVAIALDWLKLALFGRDFSRF

>31

MHSAARDAPRAVAAAEEGVANPTRSGSDDAHPGYVPEPTEPGDRRHRLVIVGSGWGGFSLLKYADAKRVHVSMVSARPFFLFTPLLASTCVGTLEFRSIQEPVRNMRFPNEGDFHQAIVTGVDTSKQLLLCQSALDASYKYSVHYDTLVLGVGMRPNTFNIEGVTKYGHFLKELADARAIRVHLLRNLELACEPGVSAEERQRLLTVVIAGGGATGVEFGAELHDFLVQDLPKLYPHLQDHIRIVLVEPNDILGAFDSRLRTFAERKIRQRRDMTIVRKFIVDVTEKNVHFKDGTTHPFGVLVWVTGLAPSPLAVSLSQFPKNKQHQFVVDQQLRVTGIPNVYALGDCAAMTPALPCTAQVAERQGRYLASVLSNLGSDSAAVASAPPFVFKSMGMMAYVGDHDAISDLPVTKLSGIMAWWLWFSAYLTRLGAWRLRLQVPFDWFRSRLFGRDISRF

>32

MSATQKQQHHVVIVGMQFAGSAVLKALIKNPQVRITIVDSKDYFDLNLATPRVLVQPAIAEATLLPHATWIANLAPQFAGRVSFVHARMTRVASTAITVQLVATQALQDIAFDFLVLATGLGADYTNSLFKATRLDETSAKRVAALQSHNARLLPAKKILVVGGGAVGVEVAAEIATDYPDKTVTLVHSGSELVKLDPKSSKADTHARKFLTSHNVQLVLSDRIDRDAANQAAALASHETPQTLKTEKGAEITADLVIVALPPKAAGVSGALSESFPGAIDEQGLLKVDQYLRVASAGNNNIFAAGDVTNADDKFAHRATAAGAVVAANILATIKKPAQPSLKTYSRLASPVFAISMGRTYGFGRLPLLGYSHGWLVTSVKSKQMFVNAVPSTFKI

>33

MSKLEVVVGTLSDLKDGEMKEVAVGEGKALLVNQKGVYSAIGHACSHYGAPLKNGVLHNGHVRCPWHGACFNVTTGDIEDFPGLDGVKKFDVRIEGENVVLSTTEEEIKSGSKRTHTMCPRDQADARVVLILGGGPAGASAVESLRQSGFAGRIVLISKETHLPYDRTKLSKTPGVAADSIALRSKDFYAQHGIELLLGVSVTELAADAKTATLSDGQVLKYDFAIVATGSSPRILNVPGSTSKGVHVLRSPEDSAAFAADAENKNVVIVGSSFIGMEAAAAIAKKAASVTVLGLEAPFSRVFGEKVGAAVAKLHTDNNIKIVTSAAGVKSINTGADGGITGVELADGTVLPGQVVLFGVGVQLNTQFVKAGAGVTVNPDGSISVDKFGHAGNGLYVAGDVARFPYKYSASGSAAVRIEHFQVAERLGSIVGKNIAGKATEIDTVPFFWTAHYGKSVRYAGYADKPDEIIIHGDLSAPKFAAFYVKDGAIVAAASLAADPVVARFASLLSTQQLPTPAQVRDRAAGAEWTFTF

>34

MAFIGSAPPAQPVQAVAAPSASSTTSSSGRKKQRIVVLGTGWAALPFVKAIDDSLYEVVMVSPRNFVFTPLLAGTCVGTLDFRIIEPIRAYKQKVKYYEAACTSINFERKVIYCTNERQQHCFALDYDKLVVAVGVTTSTFGTPGVEEHTLYLKEVSHAQAIRQRIIECFESASFPGVTEEDRLRALHFIIVGGGPTGVEFAELYDFVNEDVRRLFPDLVKDVKITLLQSGKKLLTQFDDSLGDYALKNFKRQGIDVRTGARVVKVTEREVFMNNGEVIPYGMVVWSAGNGPRDLVRELIQGIGTKLKKELLGKGTSVSGSGGVLEGARLITDASLKVKFVDDIYALGDCCVQEDLALPPTAQVAQQQGIFLAKEFNRAARSGEPIQKPFKYMHLGTLAYVGSNKALADQPLGTPIYLKGALTWLFWRSVYMAKLFSMKSRFMVGIDWMRTAVFGRDVSGIMGDYDVTKRSRILLSDETLSSAARTAAEMESVKKAISGVDNEEA

>35

MPADKPRLLILGGGPAGVILAQRCCSSFVVTLVDPKEYFEITWATPRGLMDPRVAAAAAINYWDIPDLGRVIQARVTQLTSQSALLSSGDTISFDFAAVCSGSSTSELFKSAAATSRGQRLAEMKGEIRSAKSVLVVGGGPSGVEMAAEIVDAFAGKAVTLVMLGRRVESKPPPDDPRPAAAFMAGGELAGCLDERGAVKVLPSLQVEGHPHMFALGDVNNVPETKLGFLAAKQAELAAASLQALARAKAAGGPAPKLQRWKPNGGTLAVMMVTLGRDDGVMRAGGLVFSGCVPALIKSRGLFVQKYRKLLKVNAPGPAPGAAGAAGASGLGRGAVGVAAVAGAPVGAVQAAAEVAQAVAAGCDGGSGSR

>36

MAPKPRVLIIGGGFAGVTLAKKASAFADVTLVDSKSYFELTWTTVRGIVDPEVASRSAISYKDIPGMGRFVQATVTSLSAKSAVLSNGETLSFDYAALATGSSYSDTAFKSTASSSREQRLAELKALTEDIKASKSIVVVGGGPAGVEVAAEIVEAHAGKQVTLVHPGAQLLNGTPPKAGAAAKKWLESHRVTVLLNTSVQGKPEGRGPVSLTLDGKEGRTLAADVVLWCAGARPNTAFLQGGELAGCLDERGAVKVLPSLQVEGHPHMFALGDVNNVPEAKLGYLATEHGKLVAVSLKALISAKPGASPKLGAWKPGMGNQVMIVSLGRGDGVCRMNGNVCGGCLPASIKSKGLFVDDYRKQLGV

>37

MPATYKTVILGGGNASGYAAKAFVEAGIEKDSLAIITEEPFVAYERPALSKGYLLGAARLPGFHTCVGGGGERQAPEWYAEKGITYLTNSRVTKADLASRTLILASGDTVSYSQSLIIATGARAVKLTEFGVPGADLSGIHYLRDVKDAESLVAAVAAAKEASGKAVIVGGGYIGMECAAGLASTGLAGATTIVMPEDRLMARLLTPQLAAVYERLYGDKGVTMVKGAKVTAFTGTDGKVRRRPLDASLVVVGVGARANSDLFTGQLEMAAGGIKVDRMMATSVPGVYAVGDVAAFPLTSVATGQESHVRQEHVTHCRSSAAQAVKAITSTSAPPPAYEYLPFFYSRVFNLSWVFYGEAAADATPVHFGDLNEAKVFGCLWLGAGGRLVGAFLEGGSGEEAAVLKSAVAAGVKGLDAGLDTASGSGTVAAIKAKL

>38

MSNSEKNTSNIRTCSSVFAEDEKKNVSNTFSTITNKATSLTKSIFSIFRLCSPFKTDASLGRPKVLILGTGWGFMKLAKGLDVNSNDIKVISPNKYFCFTPLLTQIVSNRLPREVCEIPINELTYRGNKEVIKYIQGLALDIDKENKEVIYFDSEKKKQERIPYDYLIINVGNEDSNIVPGIKEYALYLRNVEDSIKMRDAVVNCIKEVNANWDKMSDDEKRKKLTFIVAGGGPTGVEVSGAFAELTKNFLSKNEYKKLAPFINIKIIEMANKLLPTAGDKVSGYTKYVLSSLAGIEVLLETKLKSVSKDYVVIQKEGGDEELVPYGVFVWASGASPNSLTKQICDKVEEQSFFKKAIVVDERLQVHGIPNAYALGDCALVRPRKLAERSKEIYQNALKSSFGPTVKYLRDNFSIKAFPQMFNLSKVADLPKNEEILTEEDFKNLLEKLDSMYHSPPPTAQGASQQGEYLVKLFNDYPSDKEKQECPAFIYYWNGSTCYIYDDNIAFYTPFGSMLGGIHTKYIWRLAYTTLNPSSRSRSLLSKNWFNPFGMFAGGYKNDEIYKMNVSH

>39

MHGPRVCILGGGFGGLYTALRLDSLLWPQGSKPQVTLIDRSSRFVFKPLLYELLNGGATQDEVAPPFSQLLAPYSINFIQVFTVPLQTASLRQAVKARGPGAGGRVMLENGSAVEYDWLVLALGSDSVFFGIEGVKELCLPFNTYSDAMRVSVRLRMLEQLPGVADVVVVGGGYCGIELATTVAERMQGSGRVHLITGGEDILESSPLGQREAARRTLQDQGIDIIANAFLPFPANNKGAVRTDATLRVVDHPRVFALGDVSGCDCEASTSAPTLAPTAQAPQVAFQQADYVAWNLWASINRRPLLPFKYQHLGEMMSLGRARGAVTLPVPLAPPLRQALNGGGVTVEGPLAGAMRRAAYLYRQPTAEQRMAVGASWLQQAATEGASLAQRMLAGRPTRSPPGSSR

>40

MSASSFKYVVLGGGNSSGYAAREFVQRGIGNGELAIITEEPYVAYERPALSKAYLFPEGAARLPGFYATVGGGGEKQTPEWYAEKGIDYKTNTSITAVDVSAKTLTAASGDTISYEKLIVATGARPIYLTDFGTEGADLKNIFYLRNVVDADKIVAAIADAKTKSNKATIVGGGYIGMETAACLSKNGLEVTLVFPEKHLMERLFTAEMAAFYEKVYTDKGIKLLPGSLAASFEGKDGHVTTTVLKNGDKIESDIVLVGVGARPNVEMFKGQLDLLEDRPGGIKVDGNLRTSNPDVYAVGDIAAFPLKKYGITTRQEHVANCRASATHAVASIMDPSTGDYDYLPYFYSRIFDLSWQLYGINEMTTPTLFGDTSSGKFGTYFVRDGKVVGAFLKGGSPEEQELMKKVAIEQPAAPEDLAAQGIAFASKL

>41

MLPRRVLGGLAASRAEGVDGTCRALVSLQNALCKPSDTGTVQFSSPWQQHQTRGQKAAPTPASELPLQTGRARMVVLGTGWAAARLIRDINPKLFDFTVISPRNHMVFTPLLASTCVGTLEPRSVALPLTDIQPQLKQLQNKYYAADAVAIDKDKQVVTCTEDGVEEFDVKFDMLAIATGSQGSTFGIPGVEQHAHFLRDVSNATHIRNHLIANWNKANLPTRTQKERSRLLQIVVVGGGPTGVEFAGELSSFISTRARDIRISLVEGAQLLGSFDVRLREYAARKLHNQGIHLIKVMVKEVKETELILQNGDVIPYGLCVWSTGVGPTDFTTSLPFAKTARGRIAVDDCLHAGDKSSNDDFEPLHNIFALGDCCANKEHALPALAQVAEQQGMYLAKQLNAAAKARVGKEEAPQWKPFEYHHLGSMALVGKGSAIVELGDHSKGRGLSLTGFKSWLAWRSAYLTRLGNVRNRLYVMLDWTIALLFGRDVSAW

>42

MSFFRSPEKPFHCKDKPTIVILGTGWGAISFLRALKPLHSNQYNVQIVSPRNYFLYTPLLPASATGTVDTHSIVDPIRSHLDARCNYYEAECLNIDAKEKILTCGYTKPFREASDAGQKDHTFQMKYDVLIVAIGAVTNTFGVPGVDENCFYMKSAEDAKALRERINACFELANLPDTTDEERKRLLSFVIVGGGPTGTELAAEMNDLVIILEDMLRYFPRITRSQVTIKQIDSHDHILSAFDRTIAEYATEHFRRSGIDLVLACRVKAVEPGAVVVQKGKETDRIPFGTCIWTTGIRMHPLAERLADGQEHWRSLMVDNNLRVKGSDSIFALGDAATIEQERVLRHAEELFEQGDANHDGMLSSDELQQLLLLNVKKYPQLAEIAARVPKNTVLSKEAFLKHLEELDKSLRSVPATAQAAHQEGHYLGKLFRKYKIDPATKEVVPEDAPEFEYKPLGTIAYIGHDKAVLDPGPSAPFLRYIRGWLMGLGWKSAEVFMQISYKNMWLVSRDFLKAKIFGRDISDV

>43

MPETFLDLSSGSSLGTMSTVMRHFCPTPAVWLPSRARLQRPCRQRCRTVAAQKSVAEKKLAGPSFQTSDNLGKVTSSPGGSPPPDPEDDPRAPHFEQWPAQRSFSEVLKDKTSDTIGDLLLIARRTLRKLPRRSRIRAQLRRMGNKGKGSDDAPVALKCDKPIILVLGSGWGAHSLIKVIDTDKFEAICVSPRNHFIFTPMLPSSAVGTVEFRSLLEPIRISNPFVTYIEAECEVLDVKRKLALCSSTFAYENGRRPQFEVAYDAVVIAIGEQTATFGVPGVMEHCYFLKEISDAVGLRRRIGQCFELAALPGTPEEDRKRALRFIVVGGGPTGVEFAGTLRDFVRGDLARKYPELMGDVEVVLLQSAQSILTQFSAGLQQRALDTFRKTGVSVRTGVRVVAITQDQARLYYRPFSQHLQGVVLEGGERLDYGVCVWSTGNAARPLVQAVAGAVPVQREALAGRNPAAAKLTVDPFLRIAGVRDAIALGDCSRLSGAPLPATAQVAGQQGAYVARMINKGYRLGTGGLDKAFPARWKEGSASEEVEYFEKPFAFLSLGLMAYVGSDQAITQLEAGKASFSLAGYLSFLLWRSVYITKQVSTRNRILILFDWVKTRVFGRDLSIY

>44

MPVYYGRGGKYSGKPRVVVLGSGWGAMSFIKSLSRRDSENLEVTIVSPRNYFLYTPLLPACATGTVEERSIIEPVRKVLGTKGTFFEAVCQEIDPVEKTIKACIPSDPEDSCFKVPYDILVLAVGSVNNTFGIKGVAEHTTFFKSIDDAHNLRRKVSECFERASLPAVSQEERERLLSFVIVGGGPTGVEVAAELHDMVVDDLRRIYPSLVSLVRIRVIELQDHVLSTYDREISTYTASEFSRRGRCLEGIDLVLNSRVASVAPNKVIVVNSQTNSTNEIPFGACVWATGVAMHPLIKQLQERLPEGSQTHFRSIVTDQYLRVLGSGGSIYAIGDAATIQQACTHCLPLESKALSHSEELFDQADVSKDGKLQLSEVRDILRKSSEDYSHFAEHARFLDGKYGGLKRWNSMVGKLVKKRTDGTPVSALGEDTELDKDAFREIIGKIDQGLRALPATAQVAKQQGEYVAKLLSKGKGTPGKPITGFKGFRYGHKGSLAYVGRDKAVMDVPAIGPVFGYTAGVMWKGFETYSQISLRNILLVSSDWVRTKLFGRDISRV

>45

MLLIPTCSFFEIVPCAAHALVSPASASKSLIDFPASSTWSQKQGLISGETLDFDYALLCTGSSYPSGVKPDMTKLQDRAITAAKTVVVVGGGSVGVEVASEVADAFPDKKVTIVASGDLLDRMAPSAQQYAEEWMKKHNVEVLTGERISDWGGLDDNMPAAATLKTSSGRTLAADLAFKCVGVTPATGTYAASLSSEQLGPRGAIEVYPTLQVKGWRNVFAAGDCNSIAEEKTAAMAGLSALAAAGNIIALDSGKELKPYFERIFGGVKPPVCGGTSLGSHEGVMQMGPLNVQIGKAPATVKGFITWMFGRIVGGSRFWGFLYRRMQNLMASGMAKEARRVAAAAATVSVAPASG

>46

MQRSRQLLLRLRLQAQAEGLVDGAWSQQQQRLVILGTGWGGARVARDIDTSKYDITIISPRNHMVFTPLLASTCVGTIESRSVTVPIVDIQPKLQQPQNFYYAASCKGIHPEDRLVECCSGKLPAAQALPLAGTLAPNQTRGHTRQAHAWMNEDGLRFFVEYDKLAISTGSQGSTFGIPGVEQYTHFLRDASHSTAIRSTLVDNWNKANIPGRSPLDRDRLLHVVVVGGGPTGVEFAGELADFINRDLRKIDPSRARDMRITLIEANELLGSFDARLREYTARKLVKEGVQLVKGVVKEVTEGELELQDGSRIPFGLCVWSTGVGPTPFTVSLPFAKTPRGRLAIDDKLRVLMAPRLQPDGHVQADADRGPGPQQVSEVHMRQDEEDASLHKDWKPVGNVYALGDCCANPDTPLPALAQVAEQQGKYLARCLNEEAGKLEAPQLPPFVYKHLGSMASIGGASAVIELGEAKQRKLSWAGFSSWVAWRSAYLTRLGTMKHRMYVAGDWALTLLFGRDISRW

>47

MQGPKKKLVPGVDRPLTELDVMALRPKTKPHSMQWPMHRSWLAIAWDQAVESAEDIGRHISRAIRELPSTKASTPGPRAFDKLIGKDGKLRLQVDKPVVLVLGSGWGAHSLMKVIDTDTYEVVVVSPRNYFLFTPMLPSTSVGTVEFRSLLEPVRVSNPFVNFFEAVCDRIDLEEKVAHCTGKTPYKDGRLPQFEIPYDVLVVSVGEQPATFGTPGVEEHCFFMKEIPDSVRLRERIQSQFELATLPGSQEGEMATALHFVVVGGGPTGVEFAGTMSDFLREDLKKKYPELMPYVRVTLLNSQGTILSAFDEKMQKHALDNFKRVGVDVRTGVRVTEVTNDTITLKGGEEIKYGVCVWSAGNAPRPLVQQLAEQIPEQAQYQPGGRPSKLAVDPFLRVIGARDVLAIGDCSLVVAGQQGAYAAHMINRGFMPLPPPSKLADYLFPGQQLFATMGSTLAYDDEGGEEGEEGEGAPRLIYYKKPFEFLNLGIMAYLGDDRALTEIQLPFTKVKLSGSLAFLVCVPDQARLKAQVFGRDLSNF

>48

MSPGHAALEAHCPVRVRGADASCTSAKTRVVVLGSGWGAISFIKNLDPAAFGEDGPYELVLVSPRNYMVYTPLLPSAMGGVVSETSIVESVRNLMSGKGTYYEARTTDIDPASRTLTCVKEFCEVCAARKGPSEHTEADHTFTLQYDILLCSVGAVNATFGIQGVQQHCWFLKSMEDAKKLRRHASKSLEHAALPHVSPEERRRLLSFVVVGGGPTGVEVAAELRDLVEEDVTRQMPHIKVGAGSPGAAAALPQAAAALVTEVHEGEVEVEHKDGAKERVPFGTCIWATGIAMHPLVAALKAKLPPELQDSRRGLVVDSHLRVLGTQGTIFCLGDAAVTAASPQAALPPTAQVARQEGEYLARLLSGAKLGLVPEAEAEAAGGGGELVPLPEAAKPFRYMHLGSLAYLWGQKGVMDLPFKLPFLKTLRGYLGGHTWRGLETWMQVSNRTRWLVAHDWFRTAVFGRNTSDV

>49

MAALTSQRLQHCRQGGLVAARPPRLVAAPRRLPRAARKHQLQCNAVAEVEAPSSQQSGAKAAAGLPAYDKSASGGSGKPRIVVLGTGWASMSFVRAFDEAMRDKYELIMVSPRNYFVYTPLLPAMCAGTVEERSIVESVRAVLGGKGKFFEAQCTDILPQEKAIVACFPEDAGFPEACFKISYDYLVLGVGTHFRSAVTDEWLRVKGSNGTMFALGDAATIEQNKAVEKASELFDKYAATHSDGRLTLEELQQLMREASQEFPHLREHATFLDGKVGSQRFGGLVFNAFLQANQTMSTMYKGVGLVDPQSTLSREQFSELLTRIDSSLRALPATAQVARQQGEYLAEAFKLADGDLEELPRAAPGFKYFHKGSMAYVGGGRGWQLQASTAK

>50

MGLKNLIIRGRNYISDHLTKSSLEKAIIRRQKRGKVIENEKLIILGCGWGSYSFLKNLNSIKYDITVISPRNHFLFTPLLTSSAVGTLEFRSIAEPVRTTRDINEFKYIQASVTSINPENNSVLVKSTFHNEKPFEMKYDKLVIGVGSRNNTFGIKGVEENANFLKELHHAREIRQKIIECFERASLPDVSTEERERLLSFVIVGGGATGIEFTSELNDFFSEDLSRLFPFVPVNEVKIILLEASGKILSTFDQKLVKKALINFRNSGIDVRTHSSVKEVLKDYVILDNGDRIPYGLLVWSTGIGQHPLVKNSSFEKDSHDRIIVDDHLRVKNYSNVFSFGDCANVENKNYPPTAQVASQSAVYLAKEFNNLEKLNPNPPKPFAFKFLGLLAYTGKKSGILQTDFFDLSGFIGFITWRSAYLTRLGSLRSKIQVPFDWMRTLIFGRDISSF

>51

MISRAFSKVNKNQLIKTVSTNRIVACKNNQTQFIQRFSTSTDNSNNNEQNQQQQQQKEQEPKKKMNKFAFWGGLAVAGLGGFWIIDMVVNDDFDSVTDKFRTRLPESERKKRPKVVILGTGWGSLCFLRKLHTDLFDVTIISPRNYFLFTPLLVGGTTGTVEVRSIMEPIRKYCKRADAEDATFYEAECLSVDPVSKKVKCYDNSAVKGEVSEFELEYDHLIVGVGADNQTFGIPGVKENACFLKEINDTRNIRDKIIDCLETASYPGQPEKEIDRLLNFVVVGGGPSGVEFTAELNDFLQSDLLKTYPLAKRINVTLVEALPHILTIFDKKIIDHVEKRLQSSNNTKIWTKTAVVGVREKEITVKNTTTKEESIHPYGLLVWATGNTPRKITTQIMQSIGPNIQNNRRGLVVDDYFRVAGTDGIWSIGDASINPSKPLAQTAQVASQQGRYLGRLFNQLAEEMNNDLIKKRENPDAHKEEKEKQQEKLNLFNSITGSNKSFEEAVKEKPLFKYKHMGTLAYVGDHQAVAEFKGDHSTTVSEGYITYYLWRSVYFTKLLSVRNRALVSFDWLKSSVFGRDISRG

>52

MIALLLLQIIFFVGYYVMSITFGLFKIFLTLLKFKNLAISTLCNRIPIEDRKKVVIVGGGFSGSIVAQKLENDYQVTLIDTKDFFEFTPSILRTIVEPQHVKKIQILHSHYLKHTNVIQKEVLGVQSREVILDDRSVEFDYLVINSGSSYNSPFKESSVVSSARANTLRENYYHIRKLKRILIIGGGIVGVELAAEIVDHFKGKEVTIVHSQSKLMNRFPKKTIRYTEEFLQKRGVKLIYNERVVAHRGQTFITDQGSEIIAEQAFLCTGIAPNSNFIKNSYPDAISENGYIKANDQLQMAGTTFYRNIFVSGDVLHVREEKLAQTAECTASIVVNNINAMESRCEHKMQSYKPFAKPVLISLGKYSAIFVYKDYSITGFLPALLKQAVEFKTMVRY

>53

MQKFISKEASKLVINSNINYLKLLSNKSNNEKSFFLPNILFSNNNKNITNINFNSNENNNNENNNENNNDNNNENDSDKNKYKNFLTYGGITATILAISTGAIVSEERPNDNNQIPQLQLPKDPNNKRERIIVLGTGWASLSFIQEIDLNKYEIVVVSPRNYFLFTPMLTEATVGSVEVRSIIEPIRRVLSRLTSRPTTYIEAECTNIDYVNNCIEIETHDGSEAKAKIQYDRLVVAVGSVPQCFGTKGVEEHCIYLKEAMDAHKIRQKIMDCFERANFPGTSEEEKKRLLSFLVVGGGPTSIEGSSALYDYIKEDLSKMFPHLSKYPKITLVQSADHLLNTFDLKISNYTEKQFERIGIEVLTNTRAVEVKKDHLVVLKKAHARPPGEPINATEKPSKGPEVSIPTEIPFGMCIWSTGVGPRKITQKLCDSIESQKNNRAITTDSTLKVLGIPNGNVYAAGDCSTISQTLLMNRINEIFKEADTNNDNQLSFEEIQVLFKKHATDYPQLSPYSKGFAEFFNEYDINKDGFLQLNEFKRLMEKVDSNLTALPSTAQCASQQAKYLAETLNDQYGKDPSTFQPHNFSYKHLGSFAYIGSHTAIADIPQTFTGGGFGVWWMWKAVYLKKQFSLKNKFLVSIDWVKTTLFGRDISRI

>54

MTSEKKRVLIIGGGYGGCEVAKQLDSKFNVTVVERKQTFFHSVGSVRAVVEPELVKKIYIPYDKLLKNGKFIFGTVIEISPTLAKLEDGQELTFDYLVIATGSNSLAPFKAPLEKKSSSEILNYFQNFSQQIKQAKSILIVGGGAVACELVSEIVEKYPVKDSELVKKITIVHSGSKLVNPKMNDKFTNVVSKAMKKRNVEVILNDRITMPDEIKANLLNQTSPNIQISSQNYTTEKGVPIQADLIIWTVGIKTNSESYQSHFSNVINESGQLKVNLSCQVQGYNNVFAIGDCTDFDEFKTAYNAGYHAAIAAKAIDALSKGKSNDKLAKHKVSGPILSLSLGPQDGITQISPTMCLGSFATKMIKSKSLFIDRYISQLNNPKPLIQ

>55

MNYLYNISESLKKIFNYFSSITRDCEKKRVLIIGCGFGGSQVAKLLDSNFEVTVVERKQTFFNSIASIRAIVEPELAKKIYIPYDKLLKNGKFIYGTVIEISPTLVKLEDGKELTFDYLVIATGSNSLAPFKAPLEKISGTEIFNYYKDISEQIKQAKSILIVGGGSVGCEVVGEIINKYPIKNKELAKKITIVHSGNKLVSSKTNNKFNNLINESMKKRNVSVILNDRIEIPDDIKQCFINQTSPNFQVSLKTYKTKNGLSIESDFVIWTIGIKLNSESYKTNFSNEINEIGQIKVNQSCQVQGYDNIFAIGDITDFDELKTTYNALSHGNIVAKVIKDLSNGKNKNQLAKHKLLPPIISLSLGPKDGLTQINSNLNFGSFISRILKSNNLLINRFQTHFNNPEPLK

>56

MIRNLTKLTKFTIGNRFYQSSSKGRFSGKNGNNAFKSIVGVSVGVSALFAGCVFLDQEKEPESTPSIDVKEKKSQPPKTKEDYQKKMDEEYDIEQFKYVIIGGGTAAYHAIDKILENDKEATILLISKEYEVPYQRPPLTKSLWATKDDNVVNTLNFSDWSGKKQNLLYEQESAYGNEILQFIRTKKVIDLHIDEKLVLLNDGKLIRYDKCLIATGGEPRQLKFTSTNDKKISTYRTVEDFRKLYEVVKDGGKHVTVLGGGFLGSELTCAINSNFQDKNIKIDQIFPESGVLSTLFPDYLSKYATEEIIKSGVNVHTGTLIKDVVDNSENGRLTVTLNNGKTFETDHVVVAAGIIPNTNVVKSTTLEIDPINGGYVVNPELQARTDLYVAGDVASYYDFSLGVRRRVEHHDHARATGEMAGSNMSTKDTPAPYTYQPFFWSDLTPGVGFEAVGNTSSKLKTFSVWEKPSSDETKQSYTKGNIYYLNDNNNVVGVLCYGNYGKMDTARDLILKRRTIEDLNQLQHAIDFDEHH

>57

MRLGTRPSSSSAAGGGKTKLVILGTGWGGFRVAREVDKKKYDVTVISPRNHFLFTPLLPSTTVGTLEFRCIQEPVRTIKGLQYLQASVLSVDFKSKTLRCQEVFKGTEHEVDYDSLVIATGAQNNTFGVPGVSEENHVFFLKQLGDARNIRNRLLECFERAASPFISEEERSRLLSFVVVGGGPTSIEYAAELHDFLRTDVKRWYPDLEHKVSVHLVEASDHIMGSFDEKLISYTTRLLENRKVEVLLNTSVASVGPTECTLGDGRKLPFGLIVWSTGLAPTELVSSMEGVEKERGRINIDGRLRVPGMDGVFAMGDAAANPENPLGPLAQVADQQGKYLAKCFSKSSXX

>58

MHSRSRRGVGRGGLKMSVQTPPKPKEPEFGEGRVADEELLSVIELAGRKAAAAGFLPVLGAKAKEEQDIMWKLKVSPEDVLAFADRAIDTAEDVFMHATRAFKPAARMNLPLQDGEDAPPLTATKKERIVVLGTGWGGHAISKVIDSDKYEVIYVSPRNYFVFTPMLAAASVGTVDVRSITEPIRMANPCVKYVSGEVIDIKPGDKKVVVALPSPQEKRPPQMPVSAASVAPNVPPLVHSKSPAEGATAATGGGALSRAGEGSLSQEPLASLDDAKPLMELSYDKLVYAVGTKTGTFGVPGVRENCYMLKEANDARQLRAAIVNVLEEACLPGVTDEEKRKLLSFVVIGAGPTGVEFTGELTDLIGNDVPRLFPELVGLINLTVVSSGKVLPMFEEVLQDRGLNLLQSQGIEILLGSAASEVTKEEVVLKNGKRIPYGLCFWAGGTEARPLTQSLIETIGPEQTDASGSKRGQITVDGYMRALGTNGTILALGDASSIQGVKMPTTGQVAAQEGAYVARLLNRGYDTSVEAAPTMTGYDNSTAGQMEKAVDFFRLRGRLSASPFHFINLGVLAYIGMGQAVAEVKVGKDTPVLDAAGKAGFFLWRSTYVVKQVSPRNRINVAVDWLKVRFFGRDITRL

>59

MQTVNGLLAFLILWHGANGFSSTGAHRHVSAGASRRLRNNSLSPLRVTSTKTEEELREKLARNNEDLSEVDSKVLENFGGLEDAQFLAEIKKERPYFAVLAEKASETVDSILKTSKSERPDSSSSTGVPKPRVVVLGTGWAAHALLKEIDASKFEVTTVSPRNFFLFTPMLAASAVGTVEYRSITEPIRKVNPEANYLEATCTGIDVAQKTITCENVVCEGTTCTIEDFELPYDYLVVSVGATTNTFNTPGVMEHCIFLKQVQDAQKLRKAIGNCFERANLPTVTEEQRIAALTFAIVGAGPTGVECCAELRDFIEEEGPRFYPHLLKYVRIKLIEASDKVLSVFDGALQKAAVSSLTERSTKLIDDGFIETEMTEVLLKVGVKAVTGTQLELSDGSNIPYGLAVWAAGNGPLPLVLDLIQGVEEQKEKAAWGRGRLVTDDWLRLLGAPSVFALGDCAVINDKPLPQTAQVASQQGTYLARLFSRGFEFSATVPQKNTDNEGVEAAAGGSAASSDGSDTPLGEDGGEKVPLSEKLGLSIVKGKFAKPFQFLNLGILAYTGAGGALAQVQVGKESVKSTGATGYLLWRSIYLSKQVSWRNRLLVGTDWVKTKIFGRDITRL

>60

MIDGERVTYEYAGPAAAAPAYHTHSPSMDGTLRPIGPSGFSDSMLRVRQLGPLGLGRHFRGAAGTSASTTRPRVLILGSGWGGAKLARGLDKDKYDAKARTLDLEKQYVSCEGIFTGRSFDVSFDYLVVACGNKTNTFNTPGVAEREGEEVFFLKHLHHARQIRNRIIECIERAANPTLTDAERARLLSFVVVGAGPTSCEFTSALSDFLRDDVAKWYPEEAKQAKVTLVEAGPRILGPFDAALAEYYRGHLVARGVEMRTATSVTEVWHGDDREGNHTTHARLSDGGTLPFGAMVWSAGLALVRFVERLDALPKQELGVSSLDGSQRLLTDSSLRVPGTRGRVFAIGDCAGVEGAQLPPTASVAEQQAAYLTHCFNQHYCAFDPSTDADLPPPGPVRPAASPVSLAFFLDYLFTPTPAFRYVEPGSLASLGSRAGVMDGSKSELGLPSVTGWAAIVAWRGACWTKQLSWSNMLLIPMFWFKSAVLGRDISRSLIFSIYLFFSV

>61

MIVRGQACRGLRAAGSACRRALSTPSFAPAGSLAWPKKPRVLILGSGWGGNKVARNLDGNKFDVRLVSPSNHFLFTSFLPATAVGTLEFRAVQEPVRTNPNLSQYYQAKATRLYPKEKKVVCTDIFKEKEFELEYDFLIIAAGCKTNTFRTPGVDEREGIEVFFLKHLYHARQIRSRMLECFERAAMPARVKPGEVPTAESAAKRQEEIDRLLSFVVVGGGPTNCEFATELKDFLTKDVARWYPDLVDRVKVTIVEAGPKILGMFSEHLIEYYLAGLQRKEVDVRVNTLLAKVLPFGMMVWSAGLAPVKFLTENNNFEKGMMGRIKVDDYLRVPDTGGCVYAIGDCAVTPEPLPPIASSAEQQGQYLADCFNTYYYKPEFQSSSDEDLPLPGPVPAPVGLPFPRFMYPNSAKFSYINVGGMVSMGFGDGIVDMSRADVPGFEFGDRVYKPSMRGYFAMAAWRGGYLLKQLSYRNMMLIPMYWFKSIIFGRDISNGY

>62

MWAHGMMHFGLGILPAALKPALRVGPAAARAGGVMRASVESLGRPDVVVVGGGFGGLYTALRLANLGWEEAEPPRVTLVDRSDRFVFLPMLYEVTTGQASCWEVAPRFEELLAGSGVEFVQGEAISLDAEAQLLSVRRPEEATAGEGGAANSSGTTAGGPLALPYDAAVLAPGAVPSFGGVKGAAEHALPFYSLDDALALRETLLPLSRAPAGAKISVAVIGGSFVGAGAGSQRVCCWQQPRTERYLSVDGLPHVHCLGDAACSADSKGPAPPSTAQAAMQQADYAAWNTRAALLGSPPLPFRYTNLGEMLSLGGEDGAVSALAGLVQLRGPLASLSRKAVYAARMPTPAQAARVGASWALDAALGAAALVARGAGGAAGK

>63

MKAPEAELRKNLEARNAVTEEPGRRGRFSKAWSTLEKDADWLGFVGGRCLDALDDSLFRGGYLSSGSPPQGQRPRVVVLGSGWGANAVLSQLKNAACDVTVVSPRNYFLFTPMLAGAALGTLEPRSIIEPIREANPTATYFEAEATAIDTVSKVVTCESVVCEGVSCELRDFEVPYDLLVVAVGASTNTFGVKGVKEHCLFMKQLSDAIAFREQLGYAFEQASLPGLSEEQRREKLTFVVIGAGPTGVELCGELRDFVAQDVPRLYEDLQRFVRVVLLEASDKVLMAFDGDLQEAALQKLRSDEQGIAIDVRLSAGVREVTEEEVLLSDGSSIRYALSLWAAGIGTLDFVRRTAEALPAQAAHADEARGRLAVDSWLRVAGAPGVFAFGDCAHVVGSPYPATAQVASQAGTYLGRLVADGYDFGGGDGPPKLTPGAPGERTLRHGLSSLARSKEGLAPPFTFLNLGILAYVGKSEALVQIAVGGDEGGKKVKGAGEAGFALWRSVYLSKQYGLRNRILVAVDWAKARVFGRDLSRL

>64

MNINVQYRCDICGNTQPKGDKCQCCGSESLKKEECVKENCQPPLSSNLEVVIIGGGIASLSVIRCLIEKGIHSITLICKEKHFPYYRTAIPKILFDPPFCSNPQFFIEQKKFYQKNNINILLGFTVKSIDLKKSIVITEKDLVRQEVHFSKLVLAVGGEPNKPPFIPQSYSNDIIPIHSAADMEAINSLFETRIIKQIAIIGAGLSGIEISNALRRKSAKVTVTLIEIADRILPRQLSKTSSKIFMDVLLKNGIQLKLNSQIKAILCDESTKIIEFSNGERMACDLICYSCGIKSSVQLAQSIGCKINHGIIVNEYMQTNISNVFACGDCCEFNGRCYGNWTDAMKQGIVCGKAIAGEFTKYIYTPMPYFVFSFIGVYSVGIIEGESILKQNGNEVMEIFLKSGCIVGGNLLGKIITKVQRELVTSIERKTEGKEAQLLIEKWKTYF

>65

MGGVMNVEKRLKDKPVVVIVGGGYGGAALAKLLDNEVNVLLVERKTMFYHNVGALRASVEGDATRILIPYTNLLKHGHVIQGEVTTIDPANNRLMLNNHPSPVPYDYLVLATGTSYCFPMKVAAADAVEVAKLIQGFAADLRRAQSVLIVGGGPVGCELAGEIRHVYPEKPITLLHSRPDLIPGNTPPTFKADIRKRLEGLGVTVCTNDRLVIPDAARLTPALAAPHNPGDEEPAVGLRYLVGGGPYAGQSGKTFDADIVIFATGGQTNSNAYERVASIPQTPEKQIVVDEYLRVKGQPKIFALGDCADIEAKLAYLAIEQAKAVAVNVLAAVQGKDLKPYKKTSRTLRLVNLGPAEGCGVMGNITIGKTITLLIKGKDLFTSKMRSELGVKAPTTDVHQATEVDFARLASRLNISEVEARQLVHNSTPIKHGPDATHI

>66

MGGLMNVEKRLKDKPVVVIVGGGYAGVALAKLLDNEVNVLLVERKSMFYHNIGALRASVEGDATRILIPYTNLLKYGHVIQGEVTTVDPAKNTLMLNNHPSPVPYDYLVLATGTSYCFPMKVAAADAAEVAKRIEGFAADLRRAQSVLIVGGGPVGCELAGEIRHVYPEKPITLLHSGPDLIPGNTPPTFKAEVRKRLEGLKVTVCTNERLVIPDAARLTPAAAAHPDDEEPAVGLRYLIGGGPYASRDGKQYPADIVIFATGAQTNSNVYERVAGIPQTAEKRIVVDEYLRVKGQPKIFALGDCADIETKLGYLAGIQAKAAAANVLAAIEGKDLKPYKKASGAMMMVPLGPAEGCGVMGGMAIGKTITGLIKGKDLFTSKQRSALGVKAPTTDVQQAPEVDLARLASRLNISEVEARQLVHNSTPIHHGPDATHI

>67

MNLKHLLLLLSVSYVSPFVVLNRERSRTSTFTLRSSTKLPPVKNETKTSIDDNLLSSPSGKLDFQEPSFPQSLETTKVKSGRKELWFDENSGRFYEAGGNEKIPIATLFERTLDTIEDAAIHARRIPYDKGWIDPPELEQMSRKTVVVLGSGWASHALMKVADTYKIRLIVVSPTNHFVFTPMLASASVGTIEYRSMTEAVRASNPLMDNYIEGKAIDVDVENKLLTVQLKDLLKDTRKGEAPIQQIPYDHLVVAVGCRVANTIVPGAKEYSYKLKTCEDARKLRLAVGECLEYASRPNVAPDKFLPEAIALAREQDRRKRLTWVIVGGGPTGVELAGELSDFVRDITRDRVGPYHRLLGEIQIILVHGGDRLVQAFEEELSTHALSSLEKQGVQVRLNTRVKEVGDGWIRLISKDSGDEEEIPISLSVWAAGIAPVPFIETLLSKLPPEARGANGRVSVDKWLRCPTYSPETFGSILVVGDAAAFTRNDDESDLLPQTAQVAGQQGAYVARMLDRGYDLSITPPVIPSHHKHEVLVDAWLRARGLQEAAGFSFLNLGLLAYLGQGQALSQVQLGDVPLFSYAGSVSFILWRSVYLVKQVATRNRVLVLFDWFKCKIFGRDITRF

>68

MMILTRTPIRTARIEPFARTIATSPLFSTTAAFLSTSTKKTKKPRVVILGSGWAGNTLARRLDKQKYDVRLISPANHFLFTSLLPSTAVGTLEFRAIQEPVRTIAGLGEYYQAKALQLGVTDEGKTVVHCADLFKGHEFPVRYDYLVVAAGNKTNTFQTPGIAEREGKEVFFLKHLYHARQIRNRVLECFERASNPNITDFERDRLLSFVIVGGGPTSCEFTTELYDFLQQDVVQWYPDLMSHVKVTLVEAGPGLLGSFHQSLADYYLKKLKEKKIDVRLSMAVIGVELRPYAKANTTSDESPNSDVTNDPNHPHRSSAMGQYTVAQFADGTELPFGTMVWSAGLAPVRFVKDSGFQLQRGRIVVDEYLRVPDHENVFALGDCAALVSGPLPPTASVAEQQAYYLADCFNKYYYEAATRKEPLPLPGPVAPALMPWKLLEFLNPILCKSQPEFTYKNRGMMAGMGFGGGVTDLTNTDLPSPKTTMSGTAAFVTWRTTYLTKQLSWKNMMLIPMYWFKAMVFGRDISRF

>69

MAAKDYRYVVLGGGVAAGYAARAFVEKGLGKGELAIISEESVAPYERPALSKGFLMGNPPARLPGFHTCVGSGGERLSPEWYTEHGIDLLLSKTVTQVDPATKTLKLVSGETVQYDKLFVATGSSAVTFSDLGFSGADYRGIYCLRNIQDAQKLYDAIQAHKGKEAVVIGGGYIGMEVAAALVQNQVSCTMVFPEAHMMERLFTPEIAQFYEDFYRRQGVKILKGPSCKSFVGNENGHVTGVVLTNGTELKSELVVVGIGAKPNTKLLEPFLKMEQRGFLVNGQLQTSDSNIFAIGDVATFPLKMYDNRLARVEHVGNARQMAMHAVDVVFGSQKAYDYLPFFYSRVFDKSWKFYGDTPKDATCLVFGEMNPKLFAVWVRTNGQVVGTFTESATPEEEKKIERIARERPTVDISKLKACHTAEEGLNFFS

>70

MLLAYVHGICSRKSSFNCFRRTHFLSSTRFLFPSKRRQVRAPSLHLSCLYTTREKSVNSSKPILHPKITILGGGFGGLYTALTLSRYPWTRLTKPKITLVDRSDRFVFLPMLYEVAFGQLDKWQVAPTFSQLLQGTDVEFVLGQVEKVDVQKSTCEIFSTKYGQKEFYHDRLVIAIGTEPSLSSVPGADKYALPFRTLEHAEQLKQKLVNLTKMRRQQNRKPVIFVIGGSYSGVELASNVAEYFRGEARVCIVDRGNRLLDAASEHNRNVALQTLRSLNVESLLDMEVSCVTENAVSLKSIQESTQESEKKFDADLVLWTAGFKPSSWLQFVALEKDPTGRILTSSTLQATRHDNIFVLGDAAAVTDVNGQRCKATAQVAIQQAECAAWNIWASLCNKKPVPFRYEHLGELMTLGKYNGTAEVFGIPLSGTSAQFTRRLAYLFRMPTNLHRLKVGQNWVFKPVSCFFENFSFPL

>71

MAVGWTKVFSRCTLAALLGTSMTWVHYQVRAEEQQDTTVLKQRAAIVLSKARESLSLEALYKQKAAQIIEKYYQRQGRSKSDTLETVVAEPLSSGTDEQQGIPASAFFRELSNTSVPLCVPFVIVGGGTAAWSALEVIYEKNPSAQVLVVTEEPYAPYNRTPLSKEMWSIPTVSVPNQWPNARAELEYKYHLPGQPETYRASILMNVSVTQLYPKENKIKLSNGMEVFYQKLLLATGGKPNVSELINNSSIDPSIHDSIITFRSISDFERLHNSFINPKYRNITILGGGFLGSELACSLQTRLNQEHERNKQVTLLCPEAGVLGKFLPRYLSDYITKQMRQHNVDVRPGVSVFDIVPTTSTDSSDETRIQLQLFGWKDKEHITDQVVIAVGITPRQELAEEAGLEMDPVAGGIRVNAAMQVEGNIYAAGDVASFWDRALGRRRVEHWDHAVVTGRIAGENMSGGHATYEWESMFWCDLVGLNISFEATGMIDASLRTIGVWNLKNTFRRGEFSDQDLNEGVVWYLRGNQVVGALLWNREGKGLDVARQVIGTKFQISSVQDLTNLIPVQDESGKQAPFVIDSSVLGSHQTASP

>72

MACFVSYSYTSFHWKTNSLIRKTEICRKKGTGKVYSSRDLVAALPKTPTPQKDPVQEKNQTKRSNETSVNEDSFFSLILNRVVDTVDDAMVHFNRTFFPPKIDPQAKKPTLVILGTGWAAHSLIKVIDTVKYDVRVVSPRNYFLFTPMLPSTAVGTVEFRSIVETFRTANPFVDYFEAHCVDVDLQKQVAVCESNIPGEKRKFQIFYDYLVIAVGAATNTFGTPGVQEHCYFLKEISDARGLRRAIVERFELASFPDISKEEKCRLLSFVVVGGGPTGCEFAAELHDFLVQDLKKYYPKLFGDVQVLLLQSGDSILTQFDRTLQEKALENFRQSNIQVITKARVTEVTSTHIRLVDGKEIPYGLAVWAAGNGTQPLTRLLLSKIPEQKVDEARGRLLVDSWLRVKGALNVFAVGDCAAMEPVPLPATAQVAGQQGAYLARLFNRDYCLSCPVPESEEKSTAPLAKWRPGGSPEVAKPFQFLSLGILAYIGRERAMAQIETGLEKIKMAGVLTYLLWQSVYITKQVSFRNRVLVLFDWFKTRVFGRDMSQF

>73

MAGTSFFMVSNVLVLVLVLLLLVLLLVLMMIEQWERNWATLIVMSNPSFSSGFIASFNPSFTSLNWSEKRICIVGGGFGGLYTALNLAKYLEAKGKSKETSVTLISDSERFVYSPFLYELVTGELQDWEVAPVYTDVLKGTGVKFLQGKAQSVDKINKTISVDLASLSGGGKQEVSYDKLILATGGNSKDEKEGEPGVFGFRSLEDAKKLRARLAGLKFANKKPIKIAVVGGGYPGIELSCSLAANMKKNVDITIFQRGDKILPRANLYNRIVATQRLAELGVKKFLRTEVVEIKDDSVSWKQNNGAVAEDKFDIIIRASSSRPSVLNGLDVQEEDGRIHVNDMLKVKEEEDLYAVGDIARCIDSSGSPVPSTAQIAMQQAEVAAWNVVADLTGGVPLTFRRQDLGEMLSLGGYTASLSSKAFGLQLDDKLAHLLRREWNEGAASSC

>74

MSKKWSWKARAAVLAAGGFTTYAVVDYGLHPRPSSEVVDDLLLTKPATERKERLIVLGSGWGAVALLDKIDPFKYEVICISPRNHFVMTPLLPSVTVGTIETRTVVESIRSICPHVKFIEAECTGLNPQGKTLTFTSSKRPSSSREVQDSAKTRPEFQMAYDKLVVAVGAENNTFNTPGVEQHAHFLKEIIDARRIRAAIVDAFESACNPAQTEEERKRLLNFVVVGGGPTGVEFAAELADLLHEDLTKSFPKLKNDVKIRLIEATDKVLGMFDSKVSAFTAQTFEKEGIEVLANTFVKEVKQKEVLVQKKGSKEIESIPSSVVVWATGIRSRPITNKIRECIGVKEQTNPRALLTDGFLRVRGADGVYAMGDCATIDGKPLPATAQVASQEGKYLSKYLNGLPTAHEDSSVLNAVRKMYWKVAGGFTSEPFEYAHRGSLAYTGGDSAAADFKGAMNGFFDSIGMSVMTGKATNILWRSFYMSEQLSMRTKALLAVDWAKAKVFGRDFSRY

>75

MSNPSGQGQACKSTETSIGRILLRACANVCMIARITTFKLACLCSRVVLEVLDVLLYMMQGVKRLCARWGTSKRARKSVCIIGGSFGGLSCARNLMDDFNVTVIDQRDFFEYTPGVLQLLVKPSMFKDLCFPLSLLEGVNFCHGTAVDVHDGSVDFLPHGSGEAQRVKFDFLILACGSNYSEGIKPDPREFGMQQREEGWRARAEEVAKASSVLVVGGGPVGVELAAEIVEKFPSKSVTLVDAHQSLCETFASSSSKRYMLEWLTRRNVRVLLGHRCIPQGSDGAVRTFLVGEETVRADRVYWCLGGRPMTGFLKDSKMRFTLKDDGSILVSDHLLVYNKSNIFAVGDAISIDGLPDEKLGHTAEIQAKLVCNNIRSSLTGSRMHHYIPSLESRMYCLSLGKYSGSVRLGHFTLNGFVAALLKWGIEW

>76

MWGDKDSMQANSLAGPLEDVRLSRFNTTHSGNFSEPLPVIPTKIFKYIIIAHPKAVVAQSALAVLTQNDPEAEILVVSDRYNFDDQQGSRLYSGTFVGKETGRQMITSFTASSGKSDLLPKLSSQPLGKNVEICTTAHVVSLDVENKVVTLSDGRVVSFGKCLLATGCKEAKLPGVPDHLKKHVTNLRKVKDFERVQKMVRNGEVKKIVVVGGGFLGCEVASKLKTEGSSKQVEIIHAYVEPGALYRYVPVYFSDYMTELLRAMGVQERPYHMVLQICEPVDKKHFNLNLVLQGFEKTQLDCDHVVFAPTHLEGNVELAEASGLEIDSKSKGVMVNSEMLARTDIYVAGDTASYPDQVLGRRRMQSYDHSFYSGALAARNMSLNGREAYNHLSVVSSHGGPLGLDVVVLGDIDSTMEMYSIAQTSGRFDPNGLVAQESDTDAKNKDDAKPWGLWEKGIVYYLRDRRVVGAMLLNLNERTDDVRNLIRSGLRYQHRMSEDSGSQRILELEDAIELGLESPICRHSSARGQGIMRNSSNKERMGNQA

>77

MIPMGPYGDLPDTSVENTETRMRLAEILKDDSIIDIAFHRLYDTALDIGLHLSREASSMAHMNSGQGERKPRVVIAGSGWGAHAMLKIIDTSVLDVVCVSPRSYFIFTPMLASASVGTVEYRSITEPMRSANPCVTYHEASITSIDADRKTIRCKPVFEGFDREFDLSYDYLVLGLGMKINTFGTPGVKEHCFFLKDINDAKRLRSAIIDKFESASLPNLTDEERRELLSFVVVGGGPTGVEFSGEFFDFLNEDLKRYYPQLVPFVRTELIQAGDALLNQFDETMQAMALRSLLSQGVKVLLNARVEEVSAKEIRYNIRNPDKTTTTITTKYSLCVWAAGNSPIELSKEFQKKNQPWKQDKRGRIVTDDWLRVVGINDGSVFALGDCSESESVVLPQTAQVAAQQGAYLARIFNRQFRGPDKGFLPEQTYGAALALSLRARARDGDELAKTIIDEHRVFVRPFQFLSLGLLAYVGGRSAIAQVEVGMDSPHKTGTQLLRLSKQSGLAGWILWRSVYLTKQVAFRNRVLVLFDWMKSRVFGRDIACL

>78

MFRVLNRSTLVGSRNLLLNQRSTTSSFISRNYCSTTTTTTENNNNNSNNNNENGNNNKNSQKKKKRGLLFWGGAAATAVASLTLLDLLVNDDLDIITDKFRHRLTKEEMKDRPNLVILGTGWASLCLLRKLYTDRYNVTIVSPRNYFLFTPLLPGTTTGTTESRSIMEPIRKYCRRSDADDVTFIEAECLQVDPVKKTVKCYDNSAVKGEVSEFELPYDQLVMGVGAESATFGIPGVKENACFLKEISDTRSIRDRMIDCFETAGYPGQPDAEIDRLLHFVIVGGGPTGVEFCAELNDFITNDVKKAFPKHLTDRCRVTLVEALPHILTVFDKNIIDHVEKKLQSSPTTKIWTQTAVTGVKEREMIVRDAEKKERSVPYGMLVWATGNAPRPVTQKLIQSIGPEVQNVRRGLVVDEYFRVKGADGIWAIGDCSVTPLAPTAQVASQQGRYLGRLFNDISEDLHQKKQGQMNDQEFTADLKKKPLFKYRHMGTLAYVGDKSAVFQIKDADNKTTTSEGLATFLLWRSAYLSKCLSIRNRVLVAFDWTKASIFGRDVSRG

>79

MFKLIQKTTTTSNNQIRNRIINTTTLLNNSNNINNAIISKRNYLHIGKLEQRGVKPKVVILGCGWSSYAFLKKLNGDNFDITLVSPRNHFLFTPLLASTSVGTLEFRSVAQPVRNAKDDFNYLQAECTKINHEEKSIECLSTLHHQTPFKIDYDYLIIGVGARNNTFNIPGVEKNSFFLKELHQARSIRQRIIYCFEMASLPDVTPAERRKLLSFVVCGGGPTGVEFCGELNDLVSEDISRWFPNVPMNEVKITLLEASKSILSAFDQNLVKKALENFKASGVDVRTNSPVKEVHEEKVILSDGTEIPYGMLVWSTGVAPQKFINSLPFPKDKQGRLQVDQYLCLAGQKNIFAFGDCSNVNETNLPATAQVAQQQGIYLAEQFNNSIKELESKPFVYHYFGILAYIGRKSSLFQTNAVQASGLWAWIAWRSAYLTRLGSLRSFNMELNKKQLYMLTRISKD

>80

MSAAQKKKVVIVGGGFVGLQIAMKLESKFEVVLIEKRQTFFHCVGSMRSMVEPEFATQCFLTYDKVLKKSTIIHSYATEVHPDRVVLDNGDQVTFDYLVIATGSYNLSPFKAPRDTSNIIQYYRSIRDKINQATKILVVGGGAVGVELAGEIGTDFKGKNVTLINRGDRLVSQKVNDKFSKTVADKLKKLKVNIMFNTSIDIPNEVTEAKNQESYFQFPEVEMKTYHTSQGDVEADLVFWTTGNKLNNEMLRGFPLDGQGQVRVNESFQVDGFPNVFAAGDICNTSELKTLVNAKKHIPLVASNIEALSKQKKLATYKPEEGVMIGVSIGRKDGAGLMPNGMMLPSFVIKMLKSKNMMAPTTQSLLNKSFANIGQ

>81

MFGSIVRNTLTKSKNVVNLTKFNQNNGRSSLSSSSIKYYSSNNQNRLNNNNNFNGNNNSNKNNNRNSSWMFGAGAAAVAATALLGSILSLEEQNKDTTTTEKKEKIARPEDDLNIEKFKYVIIGGGTAAYYAVDKILEHDKQATILMISKEYEVPYQRPPLSKNLWANKDENVKETLEYSDWSGAKSKLLYEPESVYGNEVLQFIRNKRVVDIHLDGKVILLNDGTLVAYEKCLIATGGEPRKFNYSASDDPRITTYRTVDDFRKLHDVVHDDKVKHVTVIGGGFLGSEITCAINDNLKDKVKITQVFPENGVLPLIFPDYLSKYATDKVKASGVDVLEGRLVKDISKNNDKLKVQLDNGSSIDTDHVVVAVGIIPNTDIAKSTSLEVDPVNGGYVVNAELQARSNVYVAGDVASFYDYNLGVRRRVEHHDHAKATGELAGKNMAGSADPYTYLPFFWSDLTDHIGFEAVGNTDAKLKTYAVWEKQKTDDADKFSKGIIYYLNDKSKVVGVLTFRNYGKMDKARELITKGKPITNLEDLQHAISLEDEHH

>82

MKIIYATIFFFYHYALYFAFSFLHIFNFAWKLLLSIKSTVTNRVPVEDRKRVVIVGGGFSGSMVAQKLENDFQVTLVDTKDYFEFTPSILRTIVEPTHIRSIQVLHSHYLKHTNVVQKEVIGVHPREVVTDDRTIPFDYLVINSGSSYNSPFKESSVVASARANTLRENYYHIRKLKKILIIGGGIVGVELAAEIVSHFKGKEVTLIHSQSKLMNRFPKKAIKYSEQYLVDHGVRIVHNERVIAHKGNIFITDQGSEIIADQAFLCTGIVPNSDMMKASFPDVISEFGYIKSNEYLQMAGTTFYRNIYVSGDVLNVREEKLAQTAENTADIVVNNIYAMEARKESSMKQYKSFSKPILISLGKYCAIFVYKDWVFTGFIPALLKEAVEWKTMIRYW

>83

MFSFVFPANLKEGEKIEVQLDPEDPDSTVLVCKVDETYFCVSNKCPIFGAPLSKGHLFKDKIVCPQHNTQFSVKSGYVEGGPVFDGLQRFQITEKDGKLRIKVPKNKLNIPKNMKMVKYDINNKQKYVIIGGGPAGLSAAETLRQCGFSGKIIIVNKEKYLAYDRTVLSKNVFFSRIKDLQFRQKDFLDNYGIEILNEEEVVEINSERKFVETKNKNHIHFDKLLISSGSEPVVFECFQELFQQNKEINNLVTLREYQDVVKIRKQILVQNEKNKKPKIQQKKNILIVGASFNGMESASSIKEFLKENANITIIDINQQPYERLLGKEVGNAIKENFKQNGVNFLMNCTIKDFKEEDNKIQTVTLSTGEVLKPDLVLLGTGTLPNTKFVGQEVEKDEFGAIKTDSFLQSSNESIYAAGDVACYPYHFTGERIRSEHINSSVYQGYIAALNMYGKLTPVQQIPFFWTGFFGKQLHYTGFVKNYDEVYIQGDLKKLEFLAWYLKNDKVLAVASVNQGPVGILINEAMRLNLLPSAMDIKKGRVNLDDIQKKVNEAARQCKCKRQSKCKI

>84

MSSATHYANIVLGGGTAAGYVARAFAQAGATNASNLAIVSREAVLPYERPALSKGFLNKTQPARLPGFHTSVGDGGDRQDAEWYKTHNIDFLGKSNVTQVDVQDRALTLEGGQRLTYDKLIVATGADPIRPNLGDRPGDIHYFRSIVDAENLVETMKKFEGRSARAIVIGGGYIGTEVGAQLLNNGIKVSFVFPEDRLMARIFTPRLANMYRETFESKGAELVHGMANKVVYGDNNEIRGLELKDGTVVSGDLIVAGIGARPVVELFKDQLDMEAGGLKVSEHLQTSDPNIYAIGDVAAYPLKLEGGKYQRQEHVVNARRSAEHVVAELTGQSKGGYDYLPYFYSRIFDFNWKLYGINEGDVVHFGHFEEGKQYGAIWIRDGQVVGILAEKPTDEQVSRMQEVARSRPAAKGEDNVRSFVQGIFGSDIPVHFD

>85

MSWSWGPVGVLTASYVRDVDHSKYKVTVVSPRDHMLFTPLLASTTVGTLEHRSIIEPVRPQAAKNGWRYLQAEATNLDLQQQRITCRMSSLHVSGVQKDTVIDYNHLVVAIGAQPHTLNVPGVDESRVFFLKETEHARNIRSHIHDCLEAASNTTLSPEVRRRLTTFCVVGGGPTGVEFAAELSDFLEQDAARLYPELTMLPQVIIFEAGTSILGSFDQALSEYGLMRMKRQHVDIRLQTQVKEVKDQSLVLSTGEEVNTSTIVWSTGVAPRSLVQQLDAKHKSNGSIGVDECLQIQEAQNAYALGDCASLERRLPTVAQVAEQQGAYLARHFNQNFSSAKPFAFASKGMLAYLGSYGGVKLSGFKAWLVWRGGYLTKLGTWRSRLQVPFDWAKTMFFGRDPARF

>86

MSAIAKKIVLVGGGNAAGYFARAVVAAGRGAELTMIAAENVLPYERPALTKAFLHAESPARLPGFHTSVGGGGERQTAEWYATHGVEVILGTRVVDANLEEKTVVTDAGKSYSYDKLVVAIGCTALKLPSAIGGDLPGVHRVRDVADALALCDAMDGCAKGSVVIGGGYVGLEVAAALATRGLSPRVVMMEPHIMSRLWTREIAEKYEKLYEAKGTTFHRGAKVAKIIAGDDGRAAGVELDGGATLECDVVVVGVGAGAPIEPFARLAAAPAPTGGIAVDGTFAASGEGIEPKSVYAIGDVAAFPLKRAGGALRRVLLHTGPHTTARMEHVAHARASAAHAAKAVLDPSSAETYDYLPYFYSRVFEHAGSERKVAWVFYGAQPEGAEVVVVGELRPKLFAAWIDPSGAFYISQTDGTTLVGAMLESGDGEEVDVVKSAAERCPKVDVDALKKCATVEDALALVAAA

>87

MAFRRAALRLGAAAFVGGGAVAFSPHPSYFDPTGTYDTSRVLPVARADASGGMNTPNSETSNDPREKLVILGSGWGAVALVKNIDPNLYDVSVVSPRNFFLNTPLLPGVTVGTVEARSLIEPVRRLLPGKPGQSRFYEAAANAVDVRAKTVTCVDESEIKAANPGFTLSYDKLVVAIGAPPNTFNTPGVRRGVVNFLKEIDDARDVRRKLADLFETASLPGVSEEEQRRMLSVVVVGGGPTGVEFAAELHDFLRDDVPKLYPGLAEKARITVVQSADHILNTYDARISEYAESKFARDGIELVTNARVTEVRPNEASVMDKKTKKVTKIPFGVCVWSTGLGTHALARELKRQAGQNMRRRAIAVDKYLQVRGVRRTTGKPEMRGTVYALGDCADVKSKAATGTELLDRADELFALADADGNGTVDKDEFRAVMKSLEDKYPHLATFTKGGSDSRLTDIMDKFDVSKDAALNRTEFRAAMEEADALLTSHPSTAQVANQQGEFLARELNAQARAKKNGETVVYRPFEYTHLGSFANLGANKAALDLPGDFVSKGYGTMLLWYGVYFSNCVSWRNKFLVVGDWFKKSFWGRDSSRV

>88

MPARASAKAPKVVVIGGQFAGRKAARLLQRDFDVTLVDAKGVWEYTPGILRCLVEPGTSRHMVLAQPPGTLTACATGFEIEEVDDGGEVTGVELSDGSKLPADFVILATGSSYASPVKTSQLEASSVEKRREELARGNATLEAASSVLVVGGGTVGVELAAEIVGKYRAAKKVTLVTPADRLLERMPEQAGKLALKWLKSNGVRVILKDRVSDWGGAPVDDSVLAPGGGAYVVKTAGGKTIEADVVYPCVGGAPAAAPAKKSIGSAMGIKGDVHVDSAMRITGMTNVFAAGDCADTHVGEERTAFTADLNAIAAAANVKNLRRGRSLQAYPNVVTGWSRVPVIAVVSLYKWYAVMQFNRVVIGGKFPAVVKWFLETMQIAVARGTWGAAFVWDAIEKVTVALGRFLFTKEDESGSVPGLELMKSISKNPPVAPA

>89

MSHAAIGAASSALLARSPSLRSKSTRASRAKSSSKTAIVAPTRAAIAEPETTRPPSARASGRKRVVVLGSGWGAISFVKSLSASAPYDVVLVSPRNYFLYTPLLPGAATGAVEERSIVEPIRRPIAEKGYKYFEAACVGVDAETKTITCRAADATFDATVPFSDLATRTEANAMACPWHTFDVEYDYLVTAVGAVPNTFGVKGVEENCLFFKEIADASRFRREVSERFERATLPDVPEERIREILTFVVIGAGPTGVELAAELYDMVYQDIAKMYPSRLIPLVSIKIVDLQEKILSAYDRRIAEYATDFFQRANIDCLLNKQVNEVKENAVVLTDNVTKVTEEVPFGMAVWCTGIKLNPLCEKIMNALPEGSQENRRSLATDKNLRVKGSGGSIFALGDCATIERPRSIGKAVDLFRSAAKCSVDGVCDSSLSKEETKACLQSGVSEFPHLEEVINNIDDAFAKYADKDTGRCAFEGFQAMLTEVDNGLRALPATAQVAKQEGEHLAAFFNAADGDAAALASDDSQFNYVHKGSLAYIGKDAAVADIPGFTIVKGLAAGIIWKSFETISQVSVRNIFLVAADMVRTKLFGRDISRFN

>90

MNSNAFRAALRSTTRLGQHRAAMAAPRQTMISLRQTARSYASSHGTTPAPHTNSDAAWVIGSLLVFGPMIYKLTAPPPKKKAGESDHHESHKKAEEDAEEAEEAEETKTASVVASTASPKHEKDFYPYILIGAGTASFAAMEAIREKDPTAAILIIGNEDIGPYMRPPLSKEMWFNKSEETTENLSFKDWQGKERNIVLQGKEKIDTLNLEDLEKIMSAESGVKLLTGVIVSDLNVAEQSITLNNGKVLKYGKVLLATGGVPKTLPVLKGANADKVSTFRSVEDFKHLHSLVKSSDKKIVVIGGGFLGSELAVAISHYGKDKNIKVTQIFPEDGNMGLVFPRYLSKWTTSQVEQEGVVVKANSRVASTKTVNEGKQVELTLENGEKVVADHVVVAVGIEANVELAKKAGLELDDIRGGVVVNAELQARRNVYCAGDMTSFHDVTLGRRRVEHYDNAILGGRVAGQNMASDKLHKTYKHQSMFWSDLGPHIGYEAVGILDSKLSTVSIWTKKPTVTAEGDKKETTSTPSETSTAERATATPLPAEAVKVEDKEKYNKGLVLYLKDKKIVGLLMWNNFGKVEDARKILGQVYDTEKVEALVKPFGIHEE

>91

MSRLHHVSHSIWMPTRQYTVPTRLYSHLSRRTTTASTHRLALFPSSLHLISPSTTTSSSSKVFLTPFQQRGIRSVGKTQGRVMILGSGWGGFKLLRDMNKDAYQIVAVSPRNHFLFTPLLASTSVGTLEFRCITEPVRGYTRNVEYHQAWCDSVDLEHSKIDCTSNLSSVPKSITADTQGNSKKLQLDYDHLIIAVGSYSNTFNIPGVKEHAFFLKEVSDARKIRSRVIECFERAEMSRSDEEKNGLLHFAVVGGGPTGVEFSSELHDFIREDCSRLFPQLMNHVTLAVYDVAPTILANFDHSLSEYCMNKFKRSGIEVRTGTVVDKVEDGRLVLKDGKVIPFGCLVWSTGLTENPLTASLEGKVLKSRSKRILTDPYLRVLDTEGNIIPNVYALGDCATIKDHELPQTAQVANQQAIYLRKALNKLAKYPERSFTDVAEPFSFKNLGSMAYIGNWEAVVDMTKLNENAKESGKLAWIFWRSSYLTMSVSMRNKMIIPMYWMLTWVFGRDVSSFQEYDRRKRLMNAEREG

>92

MGSFRALVNPSYAEQSWIPYTNLFPEGSKHQIVQGKIAEVHHHHVVLASGVSIPFDYLVLCTGSLSPSPAKFNLDSSAEALAITKKAREDLVKSKNVVVVGGGACGVELAGEIKTAYHGKNVTLIHATSKLVDYPGYSDTMKSGALTHLESLGVSVVLNEKVAIEGLTFENAIQVAPRSIRTSSGKVIESDIQFLSVGIRVDTGYLSTLKPANNAAFDSSRLVNADTHTIKVRKTLQIDQEGLTHIFAVGDCSDFSKVPTAAACKFSAPAAVKNILALAENENSKKASKLANGNAPPAMMCLATGPTTGVMSLPLVGMRFSNFFSKLLKSKDLMLGGILADMNQVK

>93

MLRLARPQGRLLNRPASAFRPTFQRSILTSRGYSTKGELPPPPPPSSPQPRSRLYRAGKTFLLIFAGLPVAGGLAWYTYDSLFEKKGDVLHQKRPLRIIGGPKNLVMTHGCSGSVLKAEEIHQNDPRQRLVILGSGWGAVSVISQLDPRKFHVTVVSPTNYFLFTPLLPSATVGTLELRSLIEPIRRLLARLGGYYLEGTAEDIDFENQLVEVAGCHDSLGRKFYVPYDKLVIAVGSESVTYGVEGLEHCCFLKSIVDARDIRRKVMENFEKASLPTTTDEERRQLLSFVICGGGPTGVEFAAELYDFLKEDLIGYFPAIPPEEVQVTIIQSADHILNTYDLRISEMTEAKFKRENINVVTNSRVVKVNPTSVVYKDKSTNEVNTASFGVCLWSTGVGMTPLVKSLVAKLPHGSQANKHAIETDAYMRVLGTPEGTVYAIGDCATIPQPQFVEKVMQYLQENDVNGDNVLSYQEFKSLAKSITAKHSVLKVFLAHLDEVFDRYDKDHNGTLDLEEIRAFLLDAEKCITALPATAQVANQQGKFVGTRINTLHAIESDPEKVKELPPFVYTHLGSLAYIGGNDAVLDLGKGMVYGGIGSEYLWRSVYFSEQVSVRTRMLLLMDWSKRALFGRDISKF

>94

MSFARAAQMARVAAPRVTPRSMLQARFNSTNSGASIAAAASKTTPSPPPQPKKRWSRLRALKRVVQIGTVGALGYGGYEIYNKRHPVEQKPFDPKKRTIVVLGSGWGAVSFLKSINTDDYNVMVVSPRNYFLFTPLLPSCTVGTIELRSIMEPIRFITRHKSRNVKFYESECTDIDPESKTISITDVSDIKGAVTNTTIPYDYLVMAIGADNQTFGMAGVREYACFLKEIWDAQKIRTRLMDCVESAGFASQAPEEVDRLLHMVVVGGGPTGVEYAAELHDFLKDDLETWYPELADKFRITLVEALPNVLPMFSSQLIQYTESTFKQNKIEVLTKTMVKEVGDKSIKVMDANKNMVSIPYGLLVWATGNCPRPLTRKLMARIKDSQNSPRGLLVDDYMRVAGAPDMYAIGDVTFSKYAPTAQVATQQGLYLASVFENLARIEEGKNKKITPFEYSHQGSLAYIGSDKAIADLPFLNGNVSVGGVATYVFWRSVYISNMFSFRNRFLVVGDWMKSKVFGRDVSRE

>95

MKHIVVVGGSYGGVACVKELQKHISKDANVAITLIEKRDARYHCVASYRALVQADFAKNLWIPYTNLFPPGSPHKVVRGTVTEVHGDHVVMDLDGSIHKVSFDYLVLATGSYIPAPAKLKAQSSAEGIALMDRIRADLQLSQSIVIVGGGACGTEFAGEIKYAYPDKYVALIHDQPSLVDYPRFPQNFKDKAREYLEKRGVEVILNERVEIEGLSRDHPCQRAHRTISLKNSHRVIESDMQFFSIGIEVDTNYMHTLQPATAAQTKRDSGIPFDVGSIIDTKTSAIHVKSTLQLDHDDFPNIFAIGDISNADPVPTAYAAATAGEVAARNIIVLLKHEMQNASNVQENTGITRCRKSSHETKLEDYAPVNALMVLAMNPTGGVSNLPVVGTLLGGLAAWAVKSGDLFSGRFWKEMNMPRP

>96

MIIGKSSSSLMQKKSLILMMVNSFSRTSLKYNKKQFSANLLSLTTTSNQSVSNSTSSSVENTLSKEIREKIMENQKVIASQSANKKKQNLVILGSGWAGFRLIKKIDLEKYNVNVVTPRNHFLFTPLLPGSACGTVELRSIIEPVRRAVHHEDYHYYEGKAVAVDTENQRVICKPNYENDPNFTLPYDKLVVAVGCDVNDFGIKGVKDYTFPLKEISHARTIRQQITQCFERASNPSTPVHLRETLLHFVIVGAGATGVEFAAECHDLIRDLSRNFPPEIMEEVSMTVIEAGSTVLSAFDSSLQKYTQKFFRRNHIKIRTNQQVKEVLSPNSLKLQDGSIIECGMIVWSAEISQGRQLPIDPKTKKIIVDDHLHVKGFDNIWALGDISLIETVPLAATAQVAQQQGLYVARHLNGEIEESKPFVYHHMGQLAYIGNYRAISQVGAVKSGGFLSWLFWRSAYMTRLVSIRNKFNVLLNWTSTFWFGRDISRF

>97

MQSLFRILSAKAIINKKKLLLTTAAATITTSAYFLLNNNNNQVKFAPNEDNHDVDQHHRSVLANNLIIQAEEKSSPTNNSPYQPKKKLVILGSGWASVGLIQSIDLDLYDVYVVSPRNYFLFTPMLPAALAGTVSMQSITEPIRSVINRVRKDKSLIEYYEAECYDVDYERGVIKCKDISNYVIHHQNGSDIANDFELKYDKLVIAVGSQPNSFGVKGVDQYSVPMKQPEHAVKIREKLLDVLESACMPNLTDEERQKALSVVVVGGGHAGIETLGYLVDFVKEDISKLFPKDIVEKLKITVIHSSDHILNTYDCKISEMCEKEFIFNNVDLKTNARVVEVRENDLVVVFKDQQKKSEPVSLPFGVCIWTTGVAQVPLVKKLAENIYKQKNEKSLVVDAHLQVVGLNNVYAIGDCSKIDQPKLVQKYESFFEQADINKDGVISFTEMESLIKAKEKEYPNFATINQKLKKLFTQADVNGDNVLSKDEFKSLIQRIDNEYYAPLPQTAQVASKQGSYLGNCLNDIEKGITYVPPFTYKNLGSFAYIGNNHAVADLSGTTVTSWQAFYLYRAAYLSKQVSWKNRFSLASDWVKTAIFGRDVSRF

>98

MFSRASSSARSLFQRSSLFFKENNAGKNFEKVTKRFKSSGEREKYFNEYGTRPEYLQNKRKTSFMPLFFLAGFVPLVLTPMVIDWWIRSDRQNGDENLKEYLQQKAVKEEIKKDKTNHHEDVTVASAKKPEVSKYKYVLIGGGTASYAALKAIRKNDPSANVLIVTVEEYAPYERPPLSKELWDSPKSEELKFVNWLGQETSVEYEPVSAYEKDENITLLTKTKVVQLDTHNQTILASNGKHYTYEKCLIATGGTPRELPGSDQFPDKVTTFRTISDYKKLAEVANKDNAHILIVGGSFLGTELSYAVANKVNKKKGSKVTQVYLEPDVLARWLPRYLSERVRQTLISAGVNLKPNTNVVSVEPSKGSDRVIVSLDNGEKLEADFVVTTTGIYPNTYVAEEAGLEIDPQNSGIVTNSQLEAVQNVFVAGDVLSYYDVVLGRRRSEHHEHAEATGRHAGTNMSTSAKKPFHYISMFWSDVGNVHFQAVGEVNSSLDTYAVWNDVVIKQNGSSWTTSASPVVASNFGSGAVYYVRDKKVVGILLWNLPDKLGHARKVILEKKQYTSLEELKDKIKLN

>99

MEDAAMNADTDVGKGLKPLGRRGRTHSRAGACTSFLASVVAFLLADSGTAFMPLRVIKSHTRAVTTPSICMSIESSSSKVKPQVVVAGGGFGGLYSALRLSELSRPSRYAPPSIDITLVDRHDRFVFLPLLYELAMGDANEEEVAPRFESLLASTGIRFVQGEVEAIDLAGKCVQVQSQGEATEVSESAVEAPSSGSSKSLAYDKLVLALGSEPMLPPAASLPLSEGSLSPSPSSVMPFYTLRDAHALRRQLMRIDALSSPTLFRTVVVGGGYSGVELAANLATRLGRDKGRVTLVERGYQILGNSPMPVRASAGKRLEEEGVEVMLGVDVVEVDGARVVVRDRAAETQGKEGAEGAGGGGREGEVVLPADLVVWTAGARVSPVVGAMEGLEKDGRGKLVTDGGLRALGSGGDVYVVGDNAAVMGYRGGGPPGSVGEKEQVLPSTAQVAFQESEVAAWNLWSSLTPGAPGPLRFQYTPLGEMLTLGPADATISGLQGMFNLEGPSASLARRLVYIMRMPTGSQRLKAAKAWAKGRTAKVKGAFQEAFAEGNVWQK

>100

MTASVFGAAYTAAFGQHRKNLLLLCLTIVTVLKTHAFLPVRTLGHANRGSLPSTAQVRHLVAPLQAAPAPSEEPPFWSLVFSDLFLLFDRTLDTVEDIGVHLRRATERDMDRRFRRDRLTTKAGTKKRVMILGTGWGGHAVTKVVDTGLYEVVIVSPRNFFLFTPMLAGSSVGTVDYRSIIEPIRAANPLADYYEAQALAIYPNNQTVRIRSEIPNEVGEYEEFLAPYDILVYGCGAQSGTFGTPGVREHAFFLKEISDAVKLRQALVDRFERANMPSVSMEEKKRILSFVVVGGGPTGVEFSGEFSDFLNRDLAKYYPALVDLVSFKIIQAGSRILPVFDAALQEQGLEVLKAQGIEVMLNRKVLKVEEKHIELDGGEILPYGLCVWAAGTAPRDITKSLIAAIPEQSASTAGQRGRLSVDRWLRVQGTNGSILALGDAVEVEGLPLPATGQVAAQHGAFLGRLLNREYDLSTPNPTFDLEKVNAFGKVANVLRLRGRLEAQAFSFLNLGLLAYVGQANALAQVQTGNLKFGEYTARAGNLLWRSVYLVKQVSTRNRVLVLNDWLRTRVFGRDISRF

>101

MSRPIVVVGAGNAAGYLVRALVAADPALGAKTLVLGAEDVAPYERPALTKAFLHEQTPPRLPGFHTCVGGGFDRQTPEWYAESGVELKLNSTVTSADFKAKTVTTAGGESFAYETLVVATGCGVIRLPESIGGGLRGVHYVRNNSDALALTEAMSKAKKCVVIGGGYIGLEVAASCATRGLNPEIIMMEPHCMARLWNGDIAKYYEALYEAKGARFHRESKVKRILADDATGAARGVELESGVVIDCDLVVVGIGATAPLPFAGLDAPEGRLGGVKVDSRFRASGADIAPGSVYAVGDIAAFPLKMTNEIVRMEHVKHARDSATLVGNLIAGKTDAEYDYTPFFYSRVFEHPGTERAVSWVFHGLQRGEIITVGDFNPKLAAFWVENSKCVGVMLESGAPEQNSALAAATRSGKSIDVDALRAAASADDAIALIL

>102

MTSATMDDGASTPRVVVLGGGFGGLYTALRLERLDWTRTTKPEVVVVDRGDAFAFKPLLYELVNETMTRDEVAPTFEELLRPTGVRHVRGTVRGFEPDGLSETRDGTPCSSSGGTCTLADGTSLTYDYLVLALGTATNDGGVEGARERAIALNGAEDAMKISSALGEAAAAGRRARVAVVGGGLSGVELASVVAERLNASPSGGSVDVITPNGRVMSSAPVGQREAATRVLEKAGVNVVSGRVLRLSDVNDIDAVSTAASVRLADEIGEERDEVYDIVCWTIGQRAETPKEWPVATTGARKIKTDATLRVNGHSRVYAVGDASSSSAEVMNASWNELPSTAQVAFQQADYAAWNIWASMNGRTALPFRYQHLGDMMVLGELDAAVAFPVGDITLEGPAAAALRRLAYLYRMPTDEQRMKIGSKWVQQGVEKLQSDPVGFFEDVRKVLPNPFAL

>103

AMTLSAIARFGAMRLAPAGASRLNTRARTASSTSGRAVHARQYVQAAIADEVEEEARVTAATPGKKRVVVLGSGWGAISFVKSLEQSAPYDVTLVSPRNYFLYTPWLPGPPTGAVEDRSIVESIRRPIASKGYRYFEANALSVDPVRKTVRCRGSDHTFQDEDDLAKSQAWKEFDLEYDYLVTAVGAVPNTFGVPGVQEHCMFFKEIEHAARFRREVNERFECATLPGVPRERIQQLLKFVVIGAGPTGVELAAELYDYVYQDVAKTFPSRLLKDVSIEIIDLQEKILSTYDRRIAEYATEFFQRANIKCILGAAVKEVKDGAVVIADKDGSNQREVPFGIAVWCTGIKLNPFCEKLMDSLPEGAQENKRSLATDKNLRVKGSNGTIFALGDCATIERPRSLAKAEDLYREAARCTPDGDCEIDLSKEGVKKALRLGFDEFPHLEEICARIDEEFPKFTQGSDRMMYPEFRNMLEEVDKGLRALPATAQVAKQQGQYLASFFNESAADDERLQRGVARFDYVHKGSLAYVGKDAAVADIPGFGILKGIAAGLIWKSFETISQVSPRNVLLVAADMLRTKIFGRDISRLS

>104

MTRIVIIGGGPAGINTAQALARTLTETDNTQVVLIEKSAFYYHAVGAPRAYVDASYTAKMFIPYGNAFPASAAKFVRIVRGVVVSISADTNEVSYNSIDTDDKKSEGAEKLQFDYLILAMGSSYSVPIKQDNNDYARSATEAKLQEVRSHIEGSEKIVVVGGGAVGCEVAAEIKSKYPTKTVTIVEAHNQLLAGNQLTSKFYKRLNASLEKLDVKVILGERLTERLSGNGLEKRTLRTDKGTEIESDIQLLCGGFSPVAKLVEDMDASLVTERGVVKVNGQLQLESEKYGHIFALGDVCNHPSPKMAFIAGEQGKFIGGQLIAVIRKQQLGFTKPFKAPGTPMIVLPLGPAGGVSQLPLFGGFVVGNWFTRMVKSKDYMAGMMWGNLSAAVLK*

>105

MVSPSSSSTGLKKKAAICREVIVFEKSKYYYHAVGTPRAVVDAEYTKKLFIPYDNAIPVEARSFVKIQRAIVTRIAPGNEVEYTPIGNDDEMVPGPVQRLSYDYLVMATGSTYTVPLKQPKNNFKRSTTEIMMAELRTQIEKANSILIVGGGATGAGVAGEIKAKFPKKNVTIIESKDKLLGSDNVREKFRARLLKFLERLDVNVVLGERLTERLNGNSFERRTLRTDKGRELASDIQLLCGGFSPTTELIKSLDETLVTPQGLIKVNSKLQLDDSRYANIYALGDANNNSAPKHMLFASQQGTHLGSELALVVRKTQANVSKDFPKVEVAPAMIPLGPNGGVSQLPIFGGVVYGNFVTRTFKSKEYFASFAWKNLNAEVPH*

>106

MWARQLATVTKTARSTSTTWWKRASSSLDVSQDDIKKPENFQLVIVGTGWAGYQMFTQCRKHLTDIEENIGRPVDLVVVSKRNHFLYTPLLASTTVGTLEFRSIIEPLRDSMFSHESDFHFARVDDIDPEKKLLHVESAISAENSHRKYDVKYDALVLACGSRPLTFGLPGVEEHAFFLKEIHHAQRIRNRILENFEAATQPGITPEEKQRLLHFVVVGGGPTGIEFCAELYDLVLQDLVHKYPQTSKYLGVTLVDSGEILNGFDKHLRAVALRKIQKRNTMDIVKKNCIEVTAEGVTVEGGEKIPAGLVVWTAGVGPNELTKSLTVLEKSKRGNILTNQYCQVLGAAEVEEKAPWGMPRRSNVFSIGDCAEILDYPLPATAQKAQSQANYLTSLLRGKNPAPAKPYAFQSKGMMAYLGSYEGLFEARPRDDDRITLSGWKAWFLWRSAYLTKLGSWRLRLQVPLDWLKAILVGRDVSKF*

>107

MFTECSKHLADIEKNVGGRDVDIVVVSMRNHFLYTPLLASTTVGTLEFQSITEPIRDGMFRHEGHFHLASVKEIDPEKKELFVKSALGSRREYPIKYDTLVLACGSRPLTFGLPGVEEHAFFLKEIHHAQKIRNKILENFEVATQPGVTPEEKARLLHFVVVGGGPTGIEFCAELYDLVLQDLRHMYPDVSKYLEVTLLDSGEILSGFDKQLRTVAISKIESRSSMQIIKKNCIEVTADGVTLEGGEKLPAGLVVWTAGVGPNALTKSLTVFEKSKRGNILTNQYCQVLGAAEIEEEAPFGMPRRSNVFSIGDCAEILDYPLPATAQKAQTQADYLTALLRGKNAAPAKPYMFRSKGMMAYLGSYQGLFEARPHEDNKITLSGWQAWFLWRSAYLTKLGSWRLRLQVPLDWLKAILVGRDVSRF*

>108

MPRILIVGGGPAGIAVAQTLATDLTAKDDTEVIVLEKSKYFYHAVGTPRAVVDAGYTKKLFVPYDSVIPPSAKEFVKIQRAIVTRIVPGANEIEYASIGEDDDLLAGPVKSMSYDYLVVATGSTYTVPIKQPKNNFKRSTTEAKLAEVREQVKAASSVLIVGGGAVGVEVAGEIKAKYPSKTVTILEGKDKLVASDDVRDKFRTKLSTYLKRLGVKVVLGERLTERLSGNSFEKRTLRTDKGTEIESDVQLLCGGFSPTTELIQKLDASLVTPEGFIKVNSKLQLDSDQYANIYALGDASSNPAPKRMYYAGLQGKHLGAELALVARKTQTNVSKAFPKVEIVGTMLPLGPNGGVSQLPVMGGVVMGNLITRSIKSKDYFAGMTWKGLGAVVPN*

>109

MFIPYDNAIPKHAKKFVRIVRGVATRISVDPNEVSYHSIGSDDKESTTVKTLTFDYLVLATGSSYAVPIKPASHDYARSATEKKLEEVRGHIERAEKIVVVGGGSVGCEVATEIKSKYPSKSVTIVDANTQLVSGNNLRDKFYSYLNASLEKLEVKVILGERLTERLSGNGFERRVLRTDKGTEIESDIQLLCGGFRPVATLVQELDGSLVTERGFVRVNDKLQLEGEKYSHIFALGDMCNHPAPKMIFIASEQGKFLAGEIAAVIHKKQTDFTRPYEAPAVAAMILPLGPSGGVSQLPVWGGVVLGDWFTWLLKSRDYFAGRMWASIGASVPS*

>110

MMRIVIVGGGQAGLSCAQNLAKTLTEKDNTEVVVLEKSGHFYHTLGAARACVDPDYAKNMFVPYDNAIPKTSSSFVRIEHAVATGISAGKKEISFQTISSDDKKSTKTEKLKFDYLVLATGSTYTVPIKQDPNDYRRATTEAKLQEVRSEIQNAGKILIVGGGAVGCEMAGQIKAKYPDKTVTILEAHSQLISRNRLSDNFYSKLHAALNAMNVNVVLGERLVERLPGNSFEKRTLRTDKGTEIESDIQLLCGGFHPVSELVCDMDPSLITEQGSIKVNELLQLDNEKYANIFALGDSSNHETPKMAFWAADQGKFLAAQLAAVVQKKQDDFNKPYPKVSTEAVILPVGTGGVSQLPIMGGVVVGDCVTWMIKAKDFMAGRTWGTLGATPPK*

>111

MNTEKGAPSVVGSKDESSGMSDEVKRLERLIRKRAYPLFLAEKGVEIIEDVVDSLVGKGKNDNISGAQREKIVVLGTGWGAAAFLKGINANHYDVTVISPRNYFVFTPMLAGASVGTVDYRSITEPIREINRKANFLEGVATEVDVENKILTCESVVCDGNSCEINDFSVSYDRLVVTIGAQTNTFGIPGVREYCNFLKQVEDARRIRTAIVNCFERANLPGLTDEDRINNLTFAVIGAGPTGIEFAAELRDFVEQDGPKYYPNLLRYVRIKVIEASSTVLAPFDKSLQEEAIKQMNRNVEFNDPNIYRLLPEQFKLVELLLDSSVKEVTAKQILLNDGQTIDYGLSVWAAGNGPLPLTLQIVDTLGEKQAAEQNVARGRIVTDAWMRAKGSNGSILAFGDCSCTTKGPDGPLPATGQVAAQQGEFLAKLINSGYDLSPAVDNEDGDIESPPVRSSDYQSTLSESIASFSTGTKDYAKPFQFLNLGILAYTGGGSALAQVSTAPNAEPIKGTGKVGNALWKSVYISKQVSWRNRLMVINDWVRRQIFGRDITRL

>112

MAHRTTGNSYSRWMSSVATTSNNTSEFNPVAAARRWFGGSTTTTTTTTTAPASPQSNTNTSCGNNKTKNKLERNNNKPRVVVLGTGWGGHTLAKRLDKTKFDVRVISPANHFLFTPLLPSTAVGTLEFRAIQEPVRTIEGLGEYYQAKARGLDTDNKVVLCEDLFKKHPFPVRYDYLLVAAGNKTNTFGIPGVEEHEGTAVLFLKHLYHARLIRNRILECFERASNNNVPPEERSRLLNFVVVGGGPTSCEFTTELYDFIHDDVSKWYPDLREEIRVTLVEAGPGLLGNFHKSLSDYYLANLRTRNVDVRLSTAVTGITERRFARADKREHENENENDGREGRTHTVATFDDGTELPFGMMVWSAGLAPVKFVKESGLPLERGRIKVDDYLRVPGSKGRIFALGDCALVGTSEKGAGLPPTATVAEQQAYYLSDSFNNYYHEFDPVDPDNDTKDVPLPGNVVPALLPWGGFPFESINRLLTNPSPQFQYLNRGAMAGMGFGGGVSDLTKTELPLPKMATSGAAAFLIWRSVYLTKQLSYTNMILIPMYWLKQLVFGRDISRF

>113

MGADGSYKYVVIGGGNAAGYAAWEFVKQGVGKNEVCIVSREEVAPYERPALSKAFLFKDVRLPGFHTCVGGGGERQQPEWYAEHGIDLMLNTEVAKANLTAGTKTIETKDGKSISAENVILATGASPIFLTKLDGADLNGIFYLRENADGLKLAEALKANAGKTVVVVGGGYIGMEVGAAAQLMGLKVKLVFPEEHVMPRLFTPALAQYYEKFYQSKGIELLKNGRLCSAFLPDAENKGVRGILVCKDDTKEEISADMVVVGVGARPNTQLVGDQLEMENGGVKVDGNMQSSVPGVYAVGDIASFGVKQFAGKRTRMEHVEHARQSAAQAVKAAMGKSDAEYDYLPFFYSRVFDLSWVFYGDNVGECVVGGSGAPQIFAVWLEGGKVMGAFAESAADDEVKRLKAAAKGFPKAVDEAKLTQLLADKSTPLTSLLDVLEA

>114

MSLCWCAGVVPQTGNALVRGLGSADAARWTPYRTPAARPCRRARVWRCVQEQEQKEAAPEADGKGRSAAPVAGETKAKANGNGRAASDTDGKPAAGIRTLERDVTKKIAVPEPIQFEAPRSSTLEMIRYRTVDTINDVVLHYSRKFKRNETLAKLAQATKPVVCVLGFGWGGHAFVKTIDTDKYDVVVISPRNHFLFTPMLASTAVGTVEFRSILEPVRVSNEFVTYFEATCENIDQENQVLLCKNLRGNTFEQHYDYLVLGVGAPTNTFNTPGVEEYAHFMKEVSDAKEIRKGIIDQFEAANLPNVSAEERERLLTFAVVGGGPTGCEFSAELSDFLRSDLKKYYPRLMVDTKVKLLNSGSSILLQFDEALQAKALDNFRKTQIDVVTDARVVAMDDTTITLKNGSKIPYGLCVWAAGIGTNPLVSSLIARVPQQKDAKGRLVIDEWQRVKGMNNTFAFGDCAVAEKRPLPATGQVAAQQGSYVSRLLNKNVCLACEVPVILPRELNNTISGQALESLREKGQAFKNARPFEFLSLGILAYVGDSKAIADVEAGDLKLGGASGTLAFLLWRSVYLTKQVSFRNRVLVLFDWLRSQIFGRDTSQMGLIPFALFLALEMTEKASL

>115

MSGWMRGLGARVAAQKNGARRWMSVDAGAPPAPPKKRASWFKRLVYGTLAGTGAYVGYRAYLFQQRVKLEQADDLVGVLPEGRKRVVVLGTGWGAMPVLRGIDSFKYEVVCVSPRNYFLMTPLLPSVAVGTVETRTVCESLRSMLVGKKIKFYEAECQEIDPKRKVIVCGENHKVHGDLGTMRESDKDVSKVVAKSSSRAIADSTRTRPSFELEYDYLIVAVGAENQTFNTPGVKEHAHFLKELPDARRIRAAISDAFESAAMPGVSADERQRLLHFVVVGGGPTGVEFAAELNDLVNEDLNLVHKKLIDDVKITLVEALPGVLTMYDKSIAEYTMKHFSREHIDVMANTFVKGVTAQSVKIQKKGEDISEIPCALVVWATGIKPRPLVDDLRKAIGEAVQSNRRALVTNQYLEVKGADGIMALGDCATIDFPKMVESVKILFEEADTVKDDRLSLAEFQALVKQKAHAYPQLEFFTDAVERHFKEADLNADGYLSMEEFHALLDKIDKKIKILPATAQVANQQGMYLAAYLNSIDPNAEDPHQFFSPFQYKHMGSLAYIGSDEAIIDLTGTKLFNLALGGRNAFYLWRSFYFTEMFTMRTKMLLFSDWMRAKLYGRDISRV

>116

MPWSRLQKPQITLIDRNQQFVFLPLLYDLIAGEMDEWQVAPKFNDLLRGTGIRHYHSEFLGFDADKKTLSVEQAATRVIDTMPYDCLVFALGSNSTLSMVPGAQENALPIMSLRDALRIKERAQMLLADTAEAPAPVYVVGGNHSGVEIACTLADMLHPRRIPVKIVFPSEELLSTAREPNRTAAQQRLRELGVQVLSCTKVTEVMSDGVELQGMRGTETMPASIVIWSAGAMPASQPIRDSSSGNFDVEYNGRGQLRVPRTARLTEGVFALGDVAEMTDKFSRKPLKATAQVAMQQAECVAGNVFKELSSAPRNRMPKSNRLREFEYMDLGEMLYLGRRNATLASIAGVSVQGPAAHAARRAAYLARMPTNVQRALVGAAMISDPVFDALDAVQAGADVAKSASSRVRPSWDF

>117

MAFIGAPWTCLSGGTHASFPVCRRSGPRPAAVLVCSSASSSEEYLKTLQASAPAKHSSSASMPQSPSRPGAPGTGANGVPSGSSAQDYLNLLQASAPRSTPGANGSESKDAAPRSKGTANAVEKPSSGVAAASSSSVLAAPEITIPEKREFLHLVAGRTIETIEDGLRAIRNKFSRLDPRLDALKPRVVVLGSGWAAHAMIKTLDTTKFDLIVVSPRSFFVFTPMLASSAVGTVEFRSVTEPIRQANDLMTYYEAECREIRPSTKEIVCVATDRDADNVGETFLVPYSFLVIAIGARVNTFGIPGVQEYMHFLKEVRDASRLRASILNRFERANLPDTSPSERKRLVSFVVIGAGPTGVELTGEMSDLIRRDLARFFPKIVDLVQLTLIEAGPAILMPFHEPLREQALKNLERQGVKVLLQTMVTKVDADTIYTKDGSELKYGLGVWAAGVGPQPLVSQLLGTLPEQQGVRGRLKVDPFLRVAGMEDVFAVGDCAQTPEALPMTGQVAAQQGAYLARLFNKDYNLSCELPMISMSSSTTPSNGTSSDAEAGEDAQVVVPTGMSKLAHPFEFLSLGILAYTGGNKAISQVQAGDLGVAYVSGRVSFWIWRSVYLTKQVSTRNRVSVAFDWMRSHLFGRM

>118

MDKQVVVIGGGSGGAVLAHELTKAGFKNVTLVDRKDYFEVTYASNRVLVDPALGERQRMHYAKLLKCKFQHGEVAELEEKSVKLKDGSRVPFDFAIVATGASYKDLAYAKGWKTTTIVERKAEVSAEHERLHAAKEVLIIGGGPTGVELAGEIAFKFPDKSVTLAHGTDRLLPGLSPKASAVALKNLQALNVNVLLSKRLSKQDEAYKKADVVYTCVGYLPNTELMQASFASKLDKDGRITVDDKLRIYGYEHLFAIGDCANVAEGKQGYSAMMQAKTCTKNMVAVARGKPLKTYKTHPPGGIVSTGPKTGVAQTPLGITSVPFFINVKNKDLFIGMTYKSFGVAR

>119

MRKILSSAIAITLRRTPLVVPQRTQRIAATLISLPLIRYGFYTLNLNDAYEVSIEDNLNEGEMREVQVGPKKEDAVLVCKVDGQIYCVSNSCPHVGAPLSAGFLVGDKVKCPFHNASFSVKDGVHEEGPMFRGLQTFPVKQENGQLVIRVEKQLLNAPRTLNMVTKGDDPTHVVIVGGGVSGQSAAETLRQAGFRGKITIITAEDSLPYDRTPMSKMTFLVKQQGLQIRPQQFYEQYGIDVLTNTTVESIDINNQDVVVGKEKIHYDKLLLATGGTARRPQLDGVNLGNVHTLRQFNDLESIRDKAKTAKNIVVVGASFIGMETASAIKKEFKDQVNITVVDSTTVPFERVLGKEVGGSLQKLHEANGVEFELNAGVKRIGGVGQVQRVDLLNGKSLQADLVILGTGIQPNNKLVKDQLKISPNGGIETDVFLKAAKNVYASGDISSYPYWATGEHVRIEHQNEAVRQGYVAALNILGKPTPLTDVPFFWTRQWDRTLAYSGVGQGFDEVIVDGDLTQQKFVAYYARKGRVVASASMNTPNAQMIISEALRLNVMPSVEDLKEKKVSLDDIKKIVLSKGSSCHCKRAGQCQAQL

>120

MRKILSSALAVSLRRTPIMTPRLTQRLAITLVSLPLIRYSFQTLNLNDAYEISIQDDLNEGEMREVQVGPKKEDAVLVCKVDGQIYCVSNSCPHVGAPLSAGFLVGDKVKCPFHNASFSVKDGAHEEGPMFRGLQTFPVRQENGQLIIRVEKELLNAPRTLNMATKGDDPTHIVIVGGGVSGQSAAETLRQAGFRGRVTIITAEDALPYDRTPMSKVSFLVKLQGLQIRQQSFYEQYGIDVVTNKSVDSIDINNQEVVIGKEKIHYDKLLLATGGQARKPQLDGVNLKNVHTFRQINDLLQIREKAKTAKNIVIVGASFIGMETASAIKKELKDQVNITVVDNSSVPFERVLGTEVGASLQKLHQANGIEFELSAGVKRIAGEDSVSRVDLLNGKSLLADLVILGTGIQPNNKLAKDQLKVSPNGGIETDVFLKAAKNVYASGDIASYPYWVTGEYVRIEHQNEAIRQGFVAALNILGRPTPLTDVPFFWTRQWDRTLAYSGVGQGFDEVIIDGDLNQQKFIAYYAKKGRIVASASMNTPNAQMIISEALRLNVMPSAQELKDNKTTLDEIKKVVLSKGTSCHCKRSGQCQAQL

>121

RVVVLGSGWGGFQLALNLDKSIPLTVVSPRNHFVFTPLLPSASVGTLECRCIQEPVRTILGSNGSYLQAKARTLDTANKRILCESIHNELFEVEYDKLVIAVGVKTNTFGIESIKQAASAHDDVFFLKHLAHARAIRTNIIDSFEQAAIPTVTDAERRRLLSFLVVGGGPTSCEFTAELHDFIKKDVTRLYRELLPHVSITIVEAGPALLGPFDKALQDYAQGLFKKRDIDVRLGTAVVGVEDFEGPGYRFPAKRALFSDGTKHEFGTMVWSAGLAPRTFTEELGDNIARHPRTHRILVDEFLRVKGHEGSIWAIGDAAINETGEPIPQLAQVARQQGIYLGKVFNGKYREDEKPFQFFSLGSMAFMGESKGIYDGSTAGPLRDPNNKSVHHWTPPALRGILAVLLWRFAYWGRQTSVANKIMIPIHWLKAYIFGRDISRY

>122

VVVLGSGWAAHALLKVADTYKIRLICVSPTNHFVFTPMLASAAVGTVEYRSMTEAVRSANPMIESYVEGKAVDIDVQNKRLTIQLEDLLDSVRVGKASTIHLDYDKLIVAVGCRVNDQMVPGAAEYCLRLKTCEDARRLRVAIGESLEYASRPDVADAPNLAAPDKEARQQERRRRATFCIVGGGPTGVELAGELADFVKDCTKPRKGSYQRLKDDIRIILIQGADSLVPQFDRDLRDHALKTLQKQNIEVRLNTRVNEVGDGYIKLAEKGGGVEETINNGVTVWAAGTSPVPFIDTLLSKLPEEARAVGGRVKVDKWLRCPTPTADTFGSILVLGDAAAAERDDSFLPQTAQVAGQQGAYVARLFNRDYDLTQTPPVYYDDKEAIDKAWLNVRGLKEAPGFDFLNLGLLAYVGDKQALSQVQLGDFPIASYAGSISFVLWRSVYLVKQVATRNRVLVSFDWLKSNLFGRDITRL

>123

MIRQSLMKTVWANSSRFSLQSKSGLVKYAKNRSFHAARNLLEDKKVILQKVAPTTGVVAKQSFFKRTGKFTLKALLYSALAGTAYVSYSLYREANPSTQVPQSDTFPNGSKRKTLVILGSGWGSVSLLKNLDTTLYNVVVVSPRNYFLFTPLLPSTPVGTIELKSIVEPVRTIARRSHGEVHYYEAEAYDVDPENKTIKVKSSAKNNDYDLDLKYDYLVVGVGAQPNTFGTPGVYEYSSFLKEISDAQEIRLKIMSSIEKAASLSPKDPERARLLSFVVVGGGPTGVEFAAELRDYVDQDLRKWMPELSKEIKVTLVEALPNILNMFDKYLVDYAQDLFKEEKIDLRLKTMVKKVDATTITAKTGDGDIENIPYGVLVWATGNAPREVSKNLMTKLEEQDSRRGLLIDNKLQLLGAKGSIFAIGDCTFHPGLFPTAQVAHQEGEYLAQYFKKAYKIDQLNWKMTHAKDDSEVARLKNQIVKTQSQIEDFKYNHKGALAYIGSDKAIADLAVGEAKYRLAGSFTFLFWKSAYLAMCLSFRNRVLVAMDWAKVYFLGRDSSI

>124

MLPRLGFARTARSIHRFKMTQISKPFFHSTEVGKPGPQQKLSKSYTAVFKKWFVRGLKLTFYTTLAGTLYVSYELYKESNPPKQVPQSTAFANGLKKKELVILGTGWGAISLLKKLDTSLYNVTVVSPRSFFLFTPLLPSTPVGTIEMKSIVEPVRSIARRTPGEVHYIEAEALDVDPKAKKVMVQSVSEDEYFVSSLSYDYLVVSVGAKTTTFNIPGVYGNANFLKEIEDAQNIRMKLMKTIEQASSFPVNDPERKRLLTFVVVGGGPTGVEFAAELQDYINQDLRKWMPDLSKEMKVILIEALPNILNMFDKTLIKYAEDLFARDEIDLQVNTAVKVVEPTYIRTLQNGQTNTDIEYGMLVWATGNEPIDFSKTLMSRIPEQTNRRGLLINDKLELLGSENSIYAIGDCTAHTGFFPTAQVAHQEGEYLAKILDKKLQIEQLEWDMLNSTDETEVSRLQKEVNLRKSKLDKFNYKHMGALAYIGSETAIADLHMGDSSYQLKGMFAFLFWKSAYLAMCLSIRNRILIAMDWTKVYFLGRDSSV

>125

MLSKNLYSNKRLLTSTNTLVRFASTRSTGVENSGAGPTSFKTMKVIDPQHSDKPNVLILGSGWGAISFLKHIDTKKYNVSIISPRSYFLFTPLLPSAPVGTVDEKSIIEPIVNFALKKKGNVTYYEAEATSINPDRNTVTIKSLSAVSQLYQPENHLGLHQAEPAEIKYDYLISAVGAEPNTFGIPGVTDYGHFLKEIPNSLEIRRTFAANLEKANLLPKGDPERRRLLSIVVVGGGPTGVEAAGELQDYVHQDLRKFLPALAEEVQIHLVEALPIVLNMFEKKLSSYAQSHLENTSIKVHLRTAVAKVEEKQLLAKTKHEDGKITEETIPYGTLIWATGNKARPVITDLFKKIPEQNSSKRGLAVNDFLQVKGSNNIFAIGDNAFAGLPPTAQVAHQEAEYLAKNFDKMAQIPNFQKNLSSRKDKIDLLFEENNFKPFKYNDLGALAYLGSERAIATIRSGKRTFYTGGGLMTFYLWRILYLSMILSARSRLKVFFDWIKLAFFKRDFFKGL

>126

MTINTKNIVVVGAGVFGVSVANHLYRELGGTYAIKLVTASNYVYFLPSAVRLTVSKDYTKSILPLKNVLDSGIEVIKDTAASFDDKEVVLGSDRAIKFDILVLATGSKWADPIGSTYTFGDNYKEYFEREASRISDADHILFLGGGFVNCELAGELLFKYLEEIRSGKKRISIIHNSDKLLPDSGLYNDTLRKNVTDYLSKNGITLYLNTVGASLDTSPKRIFLGEGSSKYIDADLIYRGVGISPNVPVNSISDLCDKKGFIQVEKNFRVKAVEAGNVFAIGDVTNFRYHGLVKRDNWVDVLTRNVISSLQEGTEASLVDADCLETGHAPSGVSLGPNAGFGQFPLPLLGTINIPSFLISRAKSKNLFSDKMEPLFKK

>127

MKFPQCAVDTRCIIEPIREYCRRSNAKHAIFYEAECLHIDEKNNKIKCKRTPQGVYSEKGPWNEFELDYDYLVVATGTQSATFGIPGVKENALFMKVATDGRKLRGRIIDSLESANIPGATDLQSLLHFVVVGGGPTGVECAAELHDFVANEVKEFFPDLADKVSITLIEALPHVLSMFSKSLVEYAEQKFHRDSIDVLTRTLVTKVDETHVYVKNLESGETSKMPYGTLLWASGIATRPVIAELIKNLGEEAGQKSPRALSVDGHMKVLGTRNIFALGDCAFMNLPPTAQVAAQQVACCGFTGKYLGRLIRKGRNAMIGDTKNGTHEFDSYVENAEEFNYNHMGAFAYLGDNAAIADFSTKADAAGGILGTSAGTTTYFLWRSVYFSKLLSFRNRVLLGVNWVFTEIFGRDTSRR

>128

MSWLSVRTRIGPSLGRSLSKATRTHITPRRALSSRVPQGPIGNQMILVGGVSVALAAAAISSSPNSWFGLPKVVKEVAEEVIAILPEDQSENDAKVEVEEPTQSNKTVASSQNITNPYVLVGAGTASFSALQTILEKDVNAKVVMIGDEDELPYMRPPLSKELWYGPSESGDLKFKDIGHNDRSVYLQHTQDTQYVDSVEALQAKEGQGVAFIKGVKVVGLDAKEKRLTLSNGDTVVYEKLLLATGGSPRTVSELENAEEEVKSHVTLFRKIEDFRKLKEISDGAESLVVLGGGFLGSELAIALAHKGATSNMKVTQVFPEHGNMGFLLPQYLSTATTEFMRNENVNVIPSASVESASMSNGKVILKLTNGSEISTDHVVVAAGIEPNVELAQQARLEIDPKFGGVLVNSELAARSDIYAAGDCASFHDITLGRRLVEHHDHAVVSGHLAGENMANSTQRKSFEHQSMFWGDLGPKLGYEAIGIIDSELETVGVWAKAKSSTAAQPSGENSGTITLAERQKQDEDSEVFNKGVVFYLRNKKVVGVLLWNLFDKIPTARRVVKDAKQVDDFSELAKVFNVHQ

>129

SAVGTLEFRAIQEPIRTLPNVYQFYQAKARDIDFDTQEVVCDSIYGSGSFSIKYDKLVMAMGVKTNTFGTKNLVEREGQEVFFLKHLWHARSIRNRTIEVFEIAALPQVGVQEKKRLLSFLIVGGGPTSCEYAAELYDFLSEDMANLYPDLVEHTTLTLVEAADAILGPFDNHLRAYVERLFNKRNIKIRTKTAVTGVELCHVEGFHHESTKAIMSDGTEHRFGTLVWSAGLQPVKFTDKVTSKGIQRTEAGRIIIDEYLRVKGHEGKVWAIGDCAECETMPLPLLAQVAQQQSGYMAKVLTKSIREDEKAFHFYSLGSMLSVGKWKGIYDGQSLGDPYGWRAKVTNISGFAAFITYRTAYWGKQVSWTNKLLIPMYWFKSWAFGRDICRF

>130

MAFWQLRGENNLLDVYGPQWKLYFGCPRPTVNRTERAVCGTSASMFRRVARRTLRLGAVAGTAVVGVKLGHMWINDDLDDIKYEVDRLFYHFARKEQEKKKVVVLGSGWGALSFVRKLDPSAFDVTVVSPRPFFFYTPLLVGSTTGVVSPGAIIEPIRDNVPNCDFLRVHCKDVDLENKKVFCEGNLELDYDHLLVAVGAQPNTFGIPGVDKYGRFLKEIEHGRQLRKEMLDIIEQAEVANANGDISKVRQLLNFVVVGGGPTGVEFCGELSDFIKQDLKRRYPKIAEYFQVTLVEALPGLLTMFDKSVGQYVQSHLMNQGVTIKLNAMVKEVEPEKVHLKTQEGVVDMDYGVLVWVAGVGMRPFTRALCEKIGKEHGQTDRRGLLVDECLRVKGTKPGEVFAIGDCAVSGKPPTAQVAYQQGKYLGRMFRLGREHQIADPQAAPFKYCHQGSMAYIGDSQGAAEIDPNAFIKLGRSSVTDHFWWRSLYGDTDLPNRVSVRRDQLRVMGPAGFVVWRSTYFSKLFSSRNRWSVASDWLRTGMFGRPASSSAQGTADV

>131

MWTRSLRAFTRAPSRGLGLRAFADEVKTGAAATTTTSPPKTARTSGLPMTTPGAPGAIRPKVVIIGTGWASFRVLADIDTKKNDVTVISPRNHLLFTPMLASSALGTVNQRSICQPVRPLTAKKNATYYESSVVGVEKEKKLVTCQTLGGQRYSLPYDKLVVGIGFQPNDFNIPGVKENALFMKETADATRFKDHVLEKLEEAAYCHALDNDLTLSEEEKWKIQELLTFIVVGGGPTGVELAGELTDFLYNEVANLYKNLKPYIRVHMFTYDLLNTFDQKLQDYALTHLRKKQNVQVHLGAFVQKVEPNVVHVKLGESAMSIRYGTLVWCAGIKPHPFVSDFGFAMNDKGSQILVDEFLKVKDESGIYAIGDCATIDNYWLPQTAQVANQQGQYLAKALSMDEAKIKPFEFHNKGTMAYLGGLTAIMANLPGVSKITGFVAFLGWRFTYWFLQLSMRNRFMLGTDWLRTFIFGRDLTRFGP

>132

MNADIGLRMMRGLSIPLRTVATAHARREMATAVPGGQPTIKKSFLRRYGGKVILSVVAVGAGLFTYEVYNARHPPKQFTWDHSKKTIAILGSGWAATSLIKDLDTENYNVVVVSPRNYFLFTPLLPSCTVGTVELRSIMMPMRYITRFKPRQIAFVEGGCNQIDVENKVLTVEDNSEIVGEVSSQKIPYDYLVVACGAENATFGIPGVREYSCFLKEAWDAKKIRTRLMDCLETAAFPGQVESEVDRLLHMVVVGGGPTGVEYAAELYDFLKEDLADWYPELAPKIKITLVEALPHVLPSFSKELITYTEKHFAEAKVKILSNTMVKEVKQKELIVQNPQKQIEKIPYGLLVWATGNTARQVVADLIKKLPSNLQNQRRGLVVDDYLKVKGSDNIYALGDASATKWPPTAQVASRQGHYLAATFNQLARIDADRDKLLAAGEDPSAIAGQVVKPFNYKDLGALAYIGSDKAIADLPGNVHVGGALTFWFWRSAYLSNLFSLRNRVLVAFDWAKKSLFGRDISRE

>133

MTNAARYLRAVCRPLNHARSKPAWPPLVARLSTAPTKNAVLVKTAKMHKVILGAVAFGTGAAILLGAESPSFISAESLTDSSPLIAAPITPPGLPVAADTSYPPTMTIYDKELPVVTVFGDDTSDLGKPRLVILGSGWGATSLLKDLEPNGYYTVVISPTNYFLFTPLLPEATTGTVEARSLLESIRYICRTPRAHFCEAEAYDVHLKEKVVEVVGEDGRHFLVPYDRLVVAVGAQNNTLGVPGVQEHTRFLKTIADARRLRVELMTNFELAALPTTSELERKRLLSFVIAGGGPTGVEYASELYDFLHEDLINYFPDILEKDVSVTIIQSADHILNTFSMAISAIAEEKLRKHKINVITNARVTEVDSAAITYRKKNVPKGEPNTFVLPFGICLWSTGIGMRDFTRRLVGKLQQQENTRALEVDSRLKLKGDPNIYAIGDCATIENPRMLEIVKERFQKAGMDRLTYQQFQSVIGSIVDQYPQTAVHLSKLRSLFDTYDIDKNNVLDLDEITKLLDDVDKKLTSLPATAQVARQQGAYLARKFNSLAFTPSDLRPVVEQTLPPFYYKHLGNFSYIGEKTAVIDLGEGRTGGGFGVFLLWRGAYLARQVSMRTRVLLAFDWIKSKLFGRDVSRF

>134

MLSTKKVVVIGGRYAGVAAAMRLNAGLGHQENVDLTLITKEDYFQHNLGSFRALVEPSFAEKLFIPYSKIFTSSNGKIVQGTVVAIHPNHVVLAGGSNVPFDYLVIATGSSYPSPSKSSKDARTESIAELQEIATAIKEAKSVLIVGGGPVGIEIAGEVATDYPEKKVTLVHASTTLMNGPYSDKFKARLLSTLQQRKVNVVLGEKVEGLKALFPDPTQKGWHVGETFVKTNKGREINADVILLATGNSLFNSHLVQSLSEDLIDAKGQLKVRPTLQLDNDAFPHIFAAGDVTNADMKLAYLAKPQAELAADNILKLIKSGPDAKLAAYKPSTSVLAVVSIGRNGGVAQLLGVWGDWVVKMLKSKDLFVKRYWADLGLAKEFPQ

>135

MLRPHHRIKQILHRPHTFRTVVYRLLSFQSLSSKTMSTNPGEVSTPNDLQNGQMKAVDVGDKQKVLLSKVQGTIYATSAKCTHYGAPLEKGVLTSDGRLTCPWHGACFNIKTGDIEDAPAMDCLKTFNVRVVGDQILINVSAEELKKNRSPPNRGLPVRRPDTAVIIGNGSGGQAAAEALREHGFDGRIIVYGKEPYLPIDRTKLSKSLDVSIDKAALRPDEFYKERGIELNTSKEVKSVNIDGRKVELTDGTVQEYKYLVLASGSQPRTLDIEGNHLKNVHYLRSLIDATALHSAIKSFEKPNVVIIGTGFIGMEVASVLAKDDKANVTIVARSEVPLKSVLGEEVGKFVRRWHEDHGVKFKANVNPKKFTPSDVDPTKVASVLLSTGEVLPADVVLVAIGATPVTTYLPSVLLNDDSSVTVTPNLSVPNHPELYAVGDIARFPTPMNPKTTRIEHWNVARNTGRLVARNIALSSRSEPLQTFNKAPYFWTAQYGKSLRYVSTAPFETVFVQGDLQSQKGEDTAFVAWYANGDNIVGCASLNKDPIVSHVSELLRLGRCPTLSEIKAGKDVLKVELGPANRL

>136

MMTRSRMPFLFRSCFRHTSAASTQPKRMLSTASHMDKFKYVVLGGGTSAGYVAKAFADKGRGKDELALISRDTAPPFERPALSKGFLNASKPARLPGFHTTVGTGGEPQDEAWYVEHGITWLGEQDVTSVDFDNHVMSTARGHSISFEKLIIATGVESSHLPADKVDDRGDILYLRSLADAERLSQAMASHRGGHALMIGGGYIGTEVTAKLIENGLQVTMVFPEDRLMNRLFSPQLAEVYAKAFADRGVSFAKGTLKSLQHDAQGNVTGAILNDGSTVQCDLVVAGIGARPDTSLFDGKLETTAGGLKVNGQLHTTAQDVYAIGDIAAFPLVLEGGKHVRQEHVQNARETARHIVDVLLAEEDGRPAPAYDYTPYFYSRCLNFNWKFYGVNEGDIMHFGVLAEGEKYGAVWVRDGQIVGTFLDNGTPDEHERFKHVARERPAVTGKGDVQRIVEGVVGQELASTLQ

>137

MAGPGLLLAARCSALVPSASLSRASLSPLCLAPLSSTLTTRTMRAVHVHCVRARQHHQHRSASSSTAQQPPSASRQLPHVVVLGTGWASHRFVRDIDHNKYHVTVVSPRDHMLFTPLLTSTAVGTLEHRSIIESIRATASERHFDFQQAQVTDIDFDNNKVMCQSAVYSNDEEPERFPIPYDFLVVGIGAVPNTFGVPGVKEHAFFLKEASDARDVRRRIHDCFEAASFPMKTAQEIEDLLTFVVVGGGPTGVEFAAELTDFLREDCTRLYPHIQHRPRVILLEASGAVLSAFDSSLRQYALRRLERQDCHVRLGRSVKEVKRHEVVLDNGEVINTHCIVWSTGVGPRALVKSLDERYLTENKQHIRVDRGLKIANTQNAFAYGDCARIDGYILPAVAQVAEQQGKFLADEFNRATPQREVGCDTFKFASSGMLAYLGHYGGVAKIAVPTPDDVTNVKLSGLTAWLVWRMGYLTKLGRWRNRLQVPFDWLKTMIFGRDPTKF

>138

MIRFISRVFGVEVNAGKAATLTKNTMRAVEFNGEKNAICVYNDGNAFYATGGKCSHYGAPLIMGGFSSGKVYCPWHCACFDVKSGMKVTSPAVQDIGSYKTSVNSDGDLIVSLPEDVKEPAAYINKGLVKRDPSNTQQFIIIGGGAAGLTCAESLRKNNFTGSIKILTNEDINPYDRVTLSKNFRMNALKTIIRADNFYTDFDIEILKQTPAKGINIKEKTVLTENGSINYDKLLIASGCRARILKGFEGLSNVCTIRSVSDHIKIKPLVESSKNIVIVGGSFLGLEAANAIKNTWADKEVTIIESEKVPLERVMGRTIGGLLKRRLEDKGVKFLTDKIASNIQKHDNKAVSIDYNSGSIPCDMVLLASGSEINTSFIPKELLNADNSVRVNALMQTDDPNIFAAGDIANYYSIYSGTSIRSEHWTVAQDQGITAGLNMLGLGKPHISVPYFWSNQAVNMQFVGTSGEPGFTESKDLGGKDEGHITYFFNGNKTVGVGIANWFGASWIFNSLFEKGLVPNKSQILGGKKFEDLKEEFKKYA

>139

MFYSRLLKNAFSLASRLRVPKFERVDYSQFLKLGAISTGLAAYSFSTVMCNDDPVREIEVGNTADIPEGSMRKMQIGENQDNFILVSKVCGKIYATGGKCSHYGAPLQMGYLDGFSVICPWHAAAFDVRTGEIFHAPGLDSIPTYKVTESNGKVIVHIPESKINSVSGSFRSKKMVKRDPNDNRVFVVVGGGAAGSTGVETLRKEGYTGRIIFVTSENILPYDRVVISKNFKVKAEELVFRSQEFYQEFDIEMKTGTEVVDIDADHKVLKLKNGQEIHYDKVLLATGCSARVPGPFKKYTSNFSNVFTLRSAADHDKIRQSIAKANKITIIGAGFLGLEAAKNIRTTWPEKEVTIIDLEQKPLANILGEEISEQILTTQKINGVHLITGQQIGSFEGKDGVITGINLPTHASYGVSENSTLVETDYVIFATGAQVNTAYIPGNLTNIDGSIRVNSHFQSENPYIYAAGDIAQFPSLLSESRERVEHWAVAEQQGRIAALNMLGKGNNYLDVPFFWSNQFLNVSFAGFSSGHDSTFTESNGENDPSKTARITYFFKGERCIGVSSVNWPGSVLRLKIALHRGLMPSKKEIINGSVRFSSIIERVKNSNPCGANCCRN

>140

MQNQLRRYISKRLLPQQAALRNIQLLNQRVATYSQYTNQSVNFQFVQIQFLQNQQKQQSNFSAYFLLLGGAAALTYAAHNGYTNQFMKKQLVEAEGETNMLEIEIDFASDLQDGQMKDLKVGPKDEDKVLISRYQGKLHAVGNYCTHFGAPLHTGQMFDDKVICPWHAAAFSVVTGALEGAPGLDGLPKFDIIERDGKFFVQVPSNLPRSHTQPLAKRDPNDTRRYVIIGGGPAGLFCAETLRQSNFTGEITIISDDKIVPYDRTLLSKVLATGDASKFKLRNEDFLANADINVRLGSRAESVNANEKTVTLSDGTKISYDKLCVATGASPFRPPIPGINLENVLVLRNGSDQEAIKQRAANSKKVVILGGGFIGSESASALKLQYKEAQEVHLVYIESTPVERAFGKEIGGYIAAEHEKHGVKVHPGRKVTEIRGDGQNASSVVLDDGTVIEADLVICGAGVLPATKFLQGSGVQLDERGGIITNPFLQTSNKDIFAAGDNASYPYWVTGKHQRVEHYINAMDQGSYAAFNMLGKLVPFSGVPFFWTRHYNKSIQYAGYAAEFDEVHIQGSLADSKFVAFYIKDNKVRAVAGQMQSAAVLTYYEAMNQNVMPTADLIKSGAENVDSVKAKLKQNKGAGKCKREHCCSKKPVATA

>141

MITPNLKKIVITKFQDRLYAVGGTCPHQGASLSSGMIFQDKIICPWHGATFDIKSGALEQYPSVDGIPSFEIVTKNDKHYAKIPSNFSDRKIPTFVKRDVTNNERYVIIGGGPAGLSCAETLRQSGFTGEIMIVSAEDVLTYDRTQLTKNLTRLDVNDIFIRTQEYLDKADINFKLGLKVNKLDIGEKTIYLSNQEAIKYDKICIATGANPFKPPIPGINLENVLVLRTNKDQEEIKKRLASGQIKNLVIMGTGFIGSEAAASLKTKYEDEMNIEVISMESVPLERQFGKVVGQVIYDQHIKHDVKMHMNRKVVNIEGDGKNANQIVLDDGTIIDCDLILIGAGVFPSTNFLKDTEVKMDRFGGIICDPFLESSVKGVYSAGDAASFPYWPTGQRVRMEHWGTAQDQGSHAAFNMLNKSVPYGNIPFLWTFHYGHYIQYVGYAFEFDEVYVQGNLEDQDFLAYYIKDDRILAVAGESRPLEIVTYLEAMQQNIMPSASDIKSGKETVETIRKKLQAIPGSRCSRESCCNKQPSTL

>142

MRPLKVGAKENDTVLVAKFEDNIYSIGNYCSHFGAPLHTGAIVGDRVFCPWHSASFNLKSGAIEGGPALDGVPQYNIISKNGKSFVQVPEVLTQTKSQQLAKRDLNDQRHFIVIGGGPAGLNCAEALRQSGFTGQISLVSADSVAPYDRTSLSKAVATGDVSQMKLRSEEYLQNDAQINLILGTRAERIIAKDNLVQLSNNQTLRYDKLCLAVGTSAVLPKIKGINLKNVHALRDHYHQAAIRDQAAKSKNIVILGGGFIGSECASALKLQFGDKVNVHLVHGDRVPMERQFGFEVGKVILQDHKQAGVQLHMQKLVTEIGGTGDSVQHVILNDGTKLEADLVIVGAGVTPSTQFLKDSGIQLNESGALVCNNKLQTNIDDIFAAGDAVSYQTGDNSHIRIEHYITSQDQGSNAAFNMLGQSQVFNRVPFFWTRNYNKSIQYVGYAQSYDEVFIQGDPLMQQL

>143

MASLRSQAWRRVAQRAFSHLSSSSPLLSSGLQGLPVAVSVSPTQTREQKSERMFWTKGLCAAALAAGAVSVAVGEGNSRTEGRKLGGSPVFSRAFGRCPRVAACAEKRTATDLGAAEDFQRGGLYELAVNGGKDKVLLSRTADGTFYCTGASCSHYSASLSKGVLTAKRTVTCPLHDAEFDLETGKCVNGPALSAIPTYPVEIKDGRVVAQIPDEVPVRARGVYAKEKRGQNTETFVLVGGGAAAATAAETLRAEGFDGRIVMICEESVPPYDRPVLTKNLNAKLDNILLRPLKALQEDLGVQVLLNSRAVGVDLKTKTVRLEGNAPDVKFDKLLLCTGSEARRLTGLPNGTARGIFTVRGKNDLQELSAFLEENKKFNSDPRVAIIGSSFVGVELAAAFHRRGCKNVTVIGQETVPFERVLGSRVGGSIKQLICSKGVRFYPQSKVVGFTSSRDRVTGVELASGEIIQADVVIVGIGSVPATKFLADQSEFALARDGAIVTDPLLRLPANPDVFVAGDIAAYPYVKTGEQIRVEHWAVAMQQGRVAALNMLGHHVPFTQIPFFWSMIFGKGMRFAGWIGSGFDEVIIEGDIDKQQFVAYYVKDDRVTAVCTMGRDPVAVAAVELLEQNLMPSPGELRQGLKNSQDVLAIAKETAATKTVKRLV

>144

MAGQWLRLLAGASVPMLSLPARCDSPASPSSPSSPKERVSALLAQPPRPFSAFSPVSTWMSTKWTSFRLRTGLLSPAAVAASAVASASPAAREAPARRQKVVVVGSGWAAVSFLADLDMTRYEPVVISPRDYFTFTPLLPSVCVGTLPASACMTGVRELLVRGGVPCGSFYEGRVAEICPTEKKVRCQSTHGKAQDAREWEESYDYLVVAAGADVNTFGVPGVKENAFFVKELEDARRLRSALFDVIETASVPGVSEEEKKKLLHFVVVGAGPTGVEVAAEIDDFFQAEGATHFPQLRPLVRITVVEMLPTVLAAYNNDVQAFAKRLLEENPRVDLCLQSQVVGVGPDSVKVRTKRADGQVEEKEMPCGLLVWASGIKSPKVCLDLARKTAELREAQQQSPVILVDQQMKVRGCEGVYALGDCCRLSPPPLVQHADTLYEAATANGAASTDWLEREAPKLSTVFPQLASSKYDFSQKPRQTQMTKEQFVKLLADIDAAYRAPAPTAQNAKQAGRYLAQTFNAFPSVEEKRRAPAFVNQTRGALVYLGHGQAAADIEGWRTFLGGAATLLLWKAAYLQMQLTLHNAVACLGGWLRTSLVGRAVCREHLDGETVYGDRRK

>145

QPHVVILGGGFGGINTALTLPSLPWNSHSVSSGKQETSCIQPRITLIDKSERFVFLPLLYELCVEDASLDEVAPTFKTLLESTQVEGIDVNNQQVVIYKSTTNTIETIDYDALVIATGAEISLDAIPGATEYALPFYTVEQCLELKRRLALLDSYLDERAKMEEMQQKVNVVVVGGGYSGVELALNLVARLGGGDDDGDVKVSLVHRGEQVLEYATEYNRKAGMERLVEAGVNVLTSTSVVEVTPWEEETQHSSSALTKQQCMVKLSTSGVSNDETSLLPTTILLWTAGATPTSKVNAGVRNSILPRDVMGRILTSPTLNVPEYPNVFAIGDCSRPKKVPYPGTAQVAMQMATVAAWNIYATLSNDSNAGKARAGSNRETVKLLPFSFLNLGEMMTLGSNDATITTLGGRVGLSGPAASWLRRLIYAARMPT

>146

MPDNVSLLEPEQPHSIWYKRYLEYNSNKIPTNYDFVLTDKELIESDPALNLLSSEIGVKEYFTKKALPEAKKEGLPISVLLERTFDTVEDVWEHLRRFPLENGWAQMSSDEEMTRKTVVVLGSGWGAHAFMKVANCNKLRVIVVSPSNHFVFTPMLASAATGTVEYRSMTESVRSANGMIEQYIEGKAVGLDLQNRKVKVKLNSLLEDFREEDSPEIDLEYDHLLVAVGCKVDSKGVPGADKSLRLKSCDDARRLRTATGEVFEYASRPDVAGVDHVEERTKRATFLIVGGGPTGVELAGELYDLGEDITRPHKGTYPRLKGNVRVILVHSGSELVPQFEKPLRAEALKSLEKKGVQVILNTRVTEIGNGFATLSTKTVDDTGYEIGREESTLPLGLSVWCAGTAPVSFVSQLLDQLPTEAKSKDGRIQVDRWLRPPMKDPSLLGSVLVIGDAAAAIEDDEYLPQTAQVAGQQGAYIARMLSRGYDLEVTPPALPCTPSSDCDVFYDPQLTEWLKIRGLDIASKFSFLNLGLLAYLGGGEALSQVQVGDFPLFAYSGSVAFVLWRSVYLVKQVATKNRVLVTFDWLKSALFGRDMTRF

>147

MLWRLFLGTVVGGVVSACRSNIRTTLAATAPTLELILDPSKDVFPVRCVIVGGGYTGSKLAYMFDSMFDVTLIDEKNYFELTNDIIPIVANPWSELNEEACRRLFILHRYYLKRSNVLTGTVDGVDEEAVTLRDGRRVSYDLLFIAPGERKPFPFATKQRTIAGRVQELRHFNQFLGTCKKVAVLGGGPVGTSLAMDLARNRPEMQVHLYHSKSELIPALPTTSRKYAVKALQKCKNLSLHLCTRVTDVDGCDSNGKRIDLNSSSVLTRLLGFVRPPAEPARFMVRYDKLHFEPAPQQSIVSQAYFGRREPHLTGDKVESSGEEEFDYVFSTIGDVPRCIQAGKGKCNILQEHEAPDGHYRVSTLMQLYKRPNIWAPGRCNNLPWVRSYGSSDVQVRTIFRGLNSVVYNPTERFLSSRDGVQPQRMAIPRLLVRLGNNDAVGATPWSGGMVGLSAFHEFMQDRNHLVKEFQQPIFYKQQDPAKVKQRISNWASHETTDIVDFSHL

>148

MLRPTHAVLRPNVVVVGTGWAGAYFTRNLNCKLANLQVLSVRNHCVFTPLLPQTTTGTLEFRAVCEPISRIQPALATLPNRFYRCVVYGVNFDEKEVNCVGVGVVDTNFNATVQTFNIKYDKLILAHGARPNTFNVPGVMDNAFFLREVNEARGIRKRLVQNIMVADLPTTDLEEAKRLLHVVVVGGGPTGVEFAATVADFFRDDVRKINHKLVEFCKVTVLEAGEVFGMFDLRVRNWGKRRLDALGVRIVKGAVVAVNNKEVVTKDGIVIRTGLVVWSTGVGPSSLTKDLDVDRTSRGRISIDDHLRVLRKGAPIPDVFAIGDCAANEKLPLPTLAAVASRQGVYLAKKVNGELSNKPIMAPFEYRSLGSMVSLGDNAALVELNVPTKFDFVGLKALFFWRSAYLSILGSWRNKLYVLVNWVGSAVFGRDTTFIGDLSEDRVWRTLAAEEVSREYARKKAFAKLKMMETKTTITAETLEMGAEMGYIVGQSDKAEDSSTAEAKDTPNTKPQ

>149

MGQSNSTKKTVLIIGGSFGGLTAAKILAKKFNVIVVDKKTFFEFTPSFHYALQNPDYIDRITADIQNYANKNNFKFIRASVTKLDSNQATLQESKDNFQTVLFDYCIIATGSNYASSVKSTEEIQTLQQRKEQMKQLIDKFNKSKKVLVVGGGAVGVEIAGLVKDQFKHLQVELWTKPQELLPQFPKRARRLADSALKKLGIKIEYGKAIEKLPESQYDYIFDCRGNIYSPSFMMNEVFKQYVDSKGRIVVDQFMRLENHKHIFCIGDACITPNDEPKMSYNASIQGQFAAQNIIKTENNDTSLKRLVDSSNIYNITTSKKQAILCLGEKIAFESITVYYTKMMIEIITCQSFKGSSFYGVVGDMQKSLLIFMAYVFSKINRKKYQNQNLDSSTHYQKLK

>150

MFARGITKATQLTLRNTKQFSTMAGFSGLKSYSQFFTKSNMNLKIGLLGLIGVGIAGYSTSQSYHSDEYYEKVIPANLQEGQKIDVQVGPKEEDSVLVAKVDGKYYCVSNKCPHFGFSLSKGVMFYDKVVCPLHNAAFSVKDGAAEGGPVVNGLENFEIVEKDGNLHIKVPKAKLNVPKPFDMVKRDPNDKRKFVIVGGGPAGISAAETLRQSGYTGQITILSKEKFIPYDRTILSKALFFADINKLQYRSKEFLESYGIEVVNEVEVTEIDTERHFVQTKNQDHIHFDKLLLATGGSPNRIPVEGVNSKNVFTLREFSDLESLKNNFQSSKKVVVIGASFIGLETAASVKDFLKDKVDITVVDQSKVPYQRVLGSEVGAAIKKLHADNGIKFNLGVGIKSIESQNGVAKRVVLSDGTSLEADMVLIGAGVSPNTRFVGEKLEKDNYGALKTDVFLQTSHPDVFAAGDIANYPYHYTGQRARFEHINSSIYQGSIAALNMVGQKTPCGEIPFFWTRFWNKSLHYTGYAPNYDEVHIQGSLDKLEFVAWYIKNDRVVAVSAMNKGPVAMVVNEAMKQNVMPSGTEIKNNIVKYEDIQKRIADRGRNCKCSRGANCCSARKLQQNQASQSAKI

>151

MGASSSKIKARLEGAPVVVVVGAGYGGLAVAKALDKTFNVVLIDRKAYFLHNIGALRVEDGYEHKICIPYSNLLDYGHVVQAEVTRISPGGVYVYGRDEPIDFDYVVIATGTSYAFPCKIAEPEMEDATGQYTGVRERIAEAQRITVVGGGPTGVELCGEIKETHPDKDVTLIHSRGALVPGPLRDAFKSKLLEKVQSMGVEVMLNERVNLKESFSEPEAVSANFVPGTRQLTLESGATMETDLVFFCMGAHVNSKSYEADFGGVMNDDGRLRVNENLQVEGFTNVFALGDCNDVAEIKLGYAAQNQATVVAKNVAALRASKALKAYTPGHHVMIVTAGRNGGVAQLPVPGGKVMGSRFSAMVKSKGLFTANQWKLLRQSLKDAAGPSGGDEDVEPDAVPRLAQAMQISEEDAEELLRGLPVGDHSGESFT

>152

MVILGTGWASYALAKSVDRELFDVTVVSPRNHFLFTPLLASTCTGTLEHRSIIEHVRRIGFRSGADFHLAEATDVDPEAKRVMCEPVVGTGEPYTLGYDVLVMGIGSDPNTFGLEGVQEHALYLKELAHARTIRTRVLDNLEEATGAMAGRLSAARRDELLNVVVVGGGPTGVEFASEFYDMAVTDVVKYYPSVADSLKVKLVTGDGLLSTFHASLRAYTEKAITSRANMELVKHNVTAVKANGVVLDNGEVIPSACIVWAAGIGERPLTTHLEATRGFPLLRRRFKVNGQLQLDGFDDIYALGDCAAIEDNVHPQTAQVAETQAYYLADHLKNVVYGRHDASQPYHFQSKGALAYIGSYRGVADFSMAQLNAKPKPASSESSASVGALSGFHAWLVWRSAYLTKLGSWRARMQVPIDWMKTFIFGRDLSRF

>153

MSEQRIRVGSANDVAQGKMKEFTFSGEGDDAVKVLVSNVKGQLHATSSKCTHYGAPLVNGVLTGDGRIICPWHGACFHAKDGEIEDAPALDSLLSLKLEVEDGDLFVTADPEKLKGKPGIAPSCKGGAQSVAKGKGVVIVGGGAGAINCVEELRKSGYQGSITIVSNEQAIIDRTKLSKALIADADKVTWRSKSHLNNVLGVELHNTSVTKVNANAKSVTLENGSTIEYEKLVLATGGTPKRIPIPGSDFKNVLVLRQISDTKAINQAVGNEKGDESKKNKNVVVIGSSFIGMEAAIALIKRANVSVVGMEKVPFERVLGQEVGQGLMQAQVKNGLKFYMEAGVEKIEGDKSTGPTAVVIKNNQGKQESIAADVVILGVGVSPATNFLKASGFKLEKDGGIAVDSKLRVQGYQDIFAIGDIAAAPTRASEHARIEHWNVASNHGRAVAKTLAGTETEYDKVAIFWSALGSQLRYCGSGGPQFDNVYVDGKPEELKFAAYYAKGDEVVAVATMGVDPLMVQCSELIRIRAMPKLSEIKNGKNPLDVELSTPSAKI

>154

MTRSRWMHVRRYGTQTQSAQADASARKQRLVVLGTGWGGYAFLKSLSYASLRRFDVKVISPTTSFSFTPLLAQASCATLDFRSVVEPIHSNRWMEYHHAWCDAVDLKANRIELTSAFNPQFRLADPLLDANPASKDESNKDESKRVTYSLQYDYLVIGVGSYNATFGTKGVKENALFLKDVSDARAIRWRILGLFESANAKQRQYTHQGQVAISAEQEHQLRRLLSFVVVGGGPTGSEFAAELHDLINDELSRLYPNVCAYATVRLLDAGSTILSSFDARLAEYAINKFARDGIQVQLNAKIRRVERDAVVLDSAGGHQERIAAGMVIWSTGITTSPLIQAFRGVAKQDRTGKLLTNHTLNLVIHPSHPNPGANVLNPAADDSHMGSPSQPPTPLDNVFALGDCSASPDALPATAQVASQQGTYLAHLFNSHLASASPSSRSSQPKPFVFHDKGSMASIGSRSALIDSPVKKDSGTLAWLLWRSAYTIMAMSWRNRFLVPANWASNLLFGRDVGRF

>155

MLSPAIRAGSLRAAVLPSLALQASRLAVPSTSASAAAAAAAAAAVAPSSLFASQQQTRTIFWTRSRKNDKTVSVLQAAQNDAQEQLPPPNSTGQNKRGFFRTARRTFYVIALGSLGTYAYFVYQGRHPPDQLPQDPSKKTIVVLGSGWGATSLLKNIDTEEYNVVVISPHNYFLFTPLLPSVTVGTLDGRSIVQPTRHTTRFKTREVKVYEADCEYVDPINKTVTFEDRSEVKGSVSKVTIPYDYLVYSVGTENQTFGIEGVKKHACFLKELSDAEKIRARLIDCVESAAIKGQSEEEIDRLLHMVVVGGGPTGIEYAAELRDFVESDLIRWYPEVANKLRVTLVEALPNILPMFSQTLIKYTESTFKENSIDILTKHMVKDVDDRDVLVKTPSGEEKKIPYGLLVWAAGNTARPLTRQLMAALPESQKNRRGLDVDDHMRLKGAEDSIFALGDATATQFAPTAQAASQQGAYLARVFNQLARLHILEDKLEAAKKANADASELSGLERQIEKAAKIRPFKYSHQGSLAYIGSERAIADIPLLGNNQIASGGVVTFMFWRSAYMSMLFSLRNRSLVAADWFKVFLFGRDVSRE

>156

MVVARALSTLPPRARVRVSNSSVRSFASSAATRSNPTPLNQAAQTAQAAQAAQAALPKKAGLLRKTLRFTGYTIGSIVFGITATTVIILAHDALTYREAHADKVPLHPLALSPERGGPKNLPILSSYAEDEQDEISKKLANKERLVIVGGGWAAVGLLKSLDPEKYNVTLISPNNYYLFNPLLPSAAVGTVEPRSLIEPIRKLLARVHGHYIQGFATDVVMGEDKPVYHGGAQRLLEVNVISGDDWDGEALCAGGFTNNERKETKGKSIYVPYDRLIIAVGSVTANHGVPGLENCFHLKTIGDARKIRSHILDNLEVASLPTTTEEERKRLLSFVVCGGGPTGVETAAEISDMINEDVFDYFPKVLRAQAQVHLIQSREHILNTYSEKISEYAEAKFARDAVDVIVNARVKRVEPDRVLYTVKDPKTAKVQELSVPSGFTLWSTGIAMSPFTKRVTEILPNQSHLKALQIDSHLRVKGAPLGSMYALGDASTIDTRLIDQLYDFVDRYDKDKDGKLSYSEFETFAQAIRRKFPIASKHFIKLREVFDQYDVDQDGQLNLNEIANVLIETGNKMTALPATAQVAAQQGHYLGNKLNKLANHRDQGADMHPHTLEEVQDVDEEVYKPFTYRNFGSLAYIGNAAAFDLPIPGGSFAGGLIAMYAWRSFYLSESVSMRTRALLLGDYIKRGIWGRDLSRI

>157

MAINRPPTVAIVGGSYVGMNLAKSLLPALPATHRVVVVEANSHFHHLFSLPRFAVLPRGGEEKALIPYTYALDAVEGQAKILHAKALAIHTSEQDPSKGWLKLDRCTDEGDTLDFDYLAIATGTQLQRPWSLASKQSDAATAKRQAVETLQSYQDAVKHAHKIVIVGGGAVGVQVACDIAELYPAQKSITLIHSRQQLMNKFHPDLHKIVTTRFDQRGVQTVLGSRVVIPPLGFPSFVRAQTFDVELQNGSKVTADLVLMCTGQTPRSELLASFAPEAISPDGFINVRPTLQIASSKCNNVFALGDIANSRAGKTVRAAFGQVEIVKCNILHLINEQESELQHFIPGPSGIHLSLGLYESIKFGNPPKQGDPPMNKGIERDLSLDMGIEGMWKRWNVPEGTPWHL

>158

MDSSTKTSRPRLVVLGSGWGAMSFIKSLPANISEMYEVVVVSPRNYFLYTPLLPAVATGTMEERSIVEPVRNFIIGKEGGRGRRLLCSCSRACPLRRCCFPAAAAAATAAAAAAVATAAAAAVGSVNNTFGIRGVDQYCFYFKSIEDAGRLRARVSECFERAALPATPEEERKKLLTFVVVGGGPTGVEVAAELYDMIECDLSKLYPNIVSIQIVELMDHVLSTYDRAISIYTAEQFKRAGIKLVLNSRVAAVEDGCVRVVNKANEVTEIKFGACVWATGIAMNPLIRQLQEKLPGQSHFRSVLTDECLRVKGSDGSIWALGDAATIDQPKVSGWVANQQGKYLAKVFSSTKITGNPEGGGLRVRVRARARARGGWYFHKGSAAYVGSDKAVFDLPRLGPLTGTGAGFVWKSYETMSQFSFRNQCLVAADWLRTKIFGRDISRV

>159

MRLRSSTKPVVLVLGSGWGAHSLIKVIDTDMYDVVVVSPRNHFVFTPMLPSTAVGTVEFRSLLEPIRTSNPCVTYLEAQCETLDPEGEGGGGEGLLVQSTHLLQLSKPWQMQIQYDKAVVAVGEQPATFGVPGVKEHCFFMKEVTDAVALRKKIAEKFELACLPGTSEQQRRAALNFVVVGGGPTGVEFAGTLSDFLREDLRKKYPALMPYVRVTLLQSVSSILTQFDERLQRNALSNLTSSGVEVRTNVRVVGVNKDKVLLKGGEELDYGVCVWSAGNAPRPLVTQIASEASRLSPGSKLCVDSFLRVVGASDLLALGDCSLVLGNRLPATAQVAGQQGAYLAHLLNSGYNLGVGGYTQPPPFQVVPRCTLQVRRTKAQRMCLYVP

>160

MLDVRLWRKVAEPVQAPSSAAASSPQELLFPLPGDPQWPPQRAFAAVALDNLLDSVVDIGRHLRRMNVESDLPAQQEQLVPGGNGRMRLRSSTKPVVLVLGSGWGAHSLIKVIDTDMYDVVVVSPRNHFVFTPMLPSTAVGTVEFRSLLEPIRTSNPCVTYLEAQCETLDPEAKVAVCTSSFAYDDGRRPQFEIQYDKAVVAVGEQPATFGVPGVKEHCFFMKEISDAVALRSRIAEKFELASLPGTSEADRRAALNFVVVGGGPTGVEFAGTLSDFLREDLRKKYPALMPYVRVTLLQSAQSILTQFDEGLGQRALEALTSSGVEVRTGVRVVQVTANKVVLKDGEEIFCGVCVWSAGNAPRPLVTQIASEVPQQAMAAEASRLSPGSKLCVDSFLRVVGASDLLALGDCSLVAGQQGAYLAHLLNSGYNLGVGGYTQPPPFQVVKRNKLQTLTEQSAALQWLANAMMGGKNRIEVAGEVSDALFRMDAPPWIRVHSEALTAAPPLDQPSRAAAVCDIASVREAAAASGGASPEALARAAQECARSFAAEERAAREAAEATEVRYWDRPFEFLSLGIMAYVGSDKALTQVEAFDVINLKLYGSVAFLLWKSVYITKQVSFRNRVLILFDWMKARVFGRDISLF

>161

RPRVVILGSGWAAARLVHDIDPKLYDITVISPRNHMVFTPLLASTTVGTLEPRSVAVHMNDIQPALSSPSNALYIAEAQSVDPTSHTVTCQSADGMSFAVSYDKLAICTGSQGSTFGIPGVLEHAHFLRDVKQAEAIRQRLIENLALAGIPGRPLDEWQRLLHVVIVGGGPTGVEVAGELTDFISNELRKLYPERSRAMRVTLVEARELLGSFDASLREYAARKLIRRGVVLRKASLRGYRTVRPVQDGTVLPYGLCIWSTGVGPTPFTLSLPFAKTAVGRIAVDKFMRPVPHVYALGDCCANPDNPLPALAQVAEQQGRYLARVLNAAAKGPVYGETTAVQQLAPEFRYRHLGSMATVGGHSAVLELGDAQRRQLSLAGFLSWVAWRSAYLTRLGSLPKRLAVAFDWTVTMLFGRDLSRW

>162

MSSYKIIFLGGGNAAGYAARAFVENCLKAGELAIITEEPYVAYERPALSKGYLLGEFGRWCAARLPGFHTCVGGGGERQAPEWYSEKGITYLTNSRVVKADLANKALTLASGEVLSYEKLIIGTGARPTRLTEFGVPGADLGGLFYLRDVKDGDVLVAAVAATKEAGGKAVVIGGGYIGMEVAAGLSSSGLSVTMVFPEDRILSRLLTPQLAAVYERLYDAKGIKMVKGAKVTGFDGVDGKVSWRCGQSLDAGLVVVGVGARPNVELFQGQLEIAAGGIKVDGQMATSVPDVYAVGDVAAFPLTSVASGEVSYARQEHVTHCRLSAAQAAKAILGLSPPPYDYLPFFYSRVFALSWVFYGEAPADATAVHFGDMPEAKCFGCLWLGAGGKLVGAFLEGGSADDAAVLKAAVAGRLTIPTEEGGLGAAAGSGAAVVAQLKAKL

>163

MVWRTTRTASILAALACIVSSSDAVSKSPSFITQHVTQRSGCHKGLKGIFSRRRRAPVHMQEIKTRPEVTNIRVPDERGRGLDTAEDNRRRRQQSVMALVDEVGTSLLDTVEDASLFWSRQLRSEDELGDEQGPRKHRIVVIGSGWSSHAFIKSIDTHKFDVVIISPRNYFLFTPLLAAASVGTVEYRSITEPMRKANPYASYFEGEATDIFPENRTVRMRTRMQTNDGSKIDLNVPYDTLVFSPGVMSSSFGVKGVYENCYFLKEVDDARRLRSAVQDRFERANIPEVTEEAKRRILTFVVCGGGPTGVEFCGELYDLLSTEFKTLYPKLKSLARVVMIQSGPTILPVFEESLRAVGLNVITTAGVEVALETRVKAVGPYSMTLHNGTELPYGLAVWTAGTGPRKITERLIEKVAVQAEMTSRARKLTIDPWLRVYGTNGSILAMGDCTKMDPILPQTAQVASQQGDYLARMLNRGYDLSTPDDQPPVQPDDKNGDNGDNGAVRKAFNLLRTLGKKEADPFRFFNYGMLAYLGNDKGLAHIQANDLDLIKASGQAGFFLWRSVYFVKQVSLRNRVLVLFDWMKSRMFGRDLSRI

>164

MGGFCSQPQRSAKHVVIVGGGLGGKSMAGLADTAFAVTLVEKRGAMIYKIGMPRGLVDTSFATDCLVPYDKLMPNGGTVRTGVAVTRVDGSASKVHLDNGSDLSYDYLILAVGARSHSPCEPPPNACDETSTAGIIEYFKKVCEEIKTASRVVLVGGGPVGVELAGEIRQAFGAEKSITVCHSGPAPCHNQQGIDTPAAFQKKLLDACKAQNIDLKLNVKADLSTPAVQEQLAQKGYATGDLPITLSDGEVIQADMVISCIGATPGGQTLEGVTLDGKGAIKVNEHLQVEGFDAGNVFAIGDCTNTQELRLSGTAAGKYKMGMMGATGNADVVFDNIKAIEKGKKPSAKIVQPPKTAMMIVPVGTHDKKMGAVVAPVPNMRHGTLMGFKQKDYFLKIQRDIMNAPLSAKK

>165

MMRQPAMQTGRSLALIRTVAASRLPAARCVATSSQAFAEDNDKRYRLWGSAGGVAAAFTAAGLGYYYYANGKKVAVCETDKSKEITAIALGTGPVERVDLGSEAEFDEAELYDVKVFGGKSSVLVTKAGGQFYCTGASCTHYSAPLVKGVLAPDHYGSGPSYHVSCPWHDAEFDLKTGACVNGPSLTAIPTYPVEVKNGRVVATLPKDMKETVEPPVAKRDPNNKAVYAVIGGGPASMAAMETLRQEGFTGRIVFITKEEFPPYDRPVLSKNLYADIDHIQLRKADFMDKLEIESKFKTTVTKLDAKNSTLHFDDGSTMKYDKMLCCAGAEPRRIPVPGHEAKNIFILRRPDHATGIQEYAMPGKKVCVVGTSFIGMEVACTLAKKGAKVALVGMEYVPFERVLGKQVGQVFKNVLDKNKLDFYGPAIVNKYDLDENGMVKGVELKDGKGYIECDAVVLGAGVAPTCHKFVEGVQVHSRDGSIICDPFMKARDGPDNFWAAGDCVTFPWYKSGHDTRIEHWDVAYQQGRVAAQNMCGKHVPFANIPFFWTMIFGKSLRYAGHCRGFDELHVEGDLDKGEFVAYYIKGDNIEAVATCNRDPIAVATAELMKLNHMPTGTEVKTGKIDADGLVKKLKEYNKKRLPAQKDSAVKN

>166

MDFGGDVYGSNRKARQGQFDEKRERVGVDKLAQGVDDPEWDVGKMMDNEDPTKALPRPGDTMSERSLVQDSLEEAKPLPPSQQLEPPSSMPRPRPIKRRARMVILGTGWAAMNFVKDLDKELYDVTVVSPRNYFTFTPLLPSVCSGTLTPFSCIEPFRHFCRNPKTGKVDVTFYEAYAEDISFVQQRVTCKSAAQQVKARSKPKPSAHPLPFPSEYIPLEEYDTLFHIPYDYLVVAVGAQNNTYNIPGVEEYSYFLKEIEHARDIRKRIMNNFEKAYLPGVSYEEKERLLHFVVVGGGPTGVEAAAEFMDFIRDDMSKYFPEELMLMAKVTLIEMSPRLLPMFSGAVSKHTRKVFKKLGISVMSEYAVSEIKEKTMTVKNLRTPRPYDEDEAIGKDRPAQGETKEMPYGFVLWAAGVGQVELSQKVLQKLREQKGRSRMAVDGQLRLLGTENVYCLGDCAEIVPKSIAEAAPDLWKCMAPHHGFFLPWDDRHKIVTQKTQTFAMAVQWLISNQAQLAADFPQMSPTKYDFREAIERKWDKKQFDYQQLKNFLASIDENYRSPAPTAQNAAQQGTYLADTFTKFRTKASKFEAPVFIEKWKGTLAYVGDSQAVADLPPDLSALTYLMPGGTILKVENDVQPPPNPSNSNDTEKIEENKTENAVQDASAGEEEDDLRVMPSGAKKDDKFICILGGNFSSLFWRAVYLQEQMTWRNRIITTFDWLKSHYLGRDVGRDHIHYS

>167

MASYAAAVLIFALQMGGFAAGVISAHQRCGVISSHQRSSPSFIPLLPALRQRPSDGRLHLARRSASTKLDEEKLRNELASRNVDAAESALGEVDVGGGLLKDLVKPRPYAAVVTQKFIENVDDFLVSLRQRPSARGAEKGKRERIVVLGTGWGSHAFLSTVDASKYEVIVISPRNFFLFTPMLAGAALGTVEYRSITDPIRNVNPLVDYYEATCTDIDPVKKTVACQSVVCEGTSCTIEQFEVPYDRLLIGVGAQTATYNIPGVKEHCQFLKQVGDAKKLRRAIGNVFERANIPGLSDAQRQAILTFVVVGAGPTGVELMSELLDFVEQDVPRYYPHLLQHVRVKLVEATDTVLMAFDESLRQKAAERLLSRPDRLVARGLLDASADPLTEIKLQAGVKEITDTEVVLTNGDRVAYGLVVWAAGNGPLPLVLSAIDKFSEQKAAQSKARGRLVVDPWLRVKGADDIFAIGDCTLMDETPLPATAQVASQQGAFLGRLLNHDVDLRAHPIPKRLGRRGSLSEVLIDGSNDYPKAFQFLNLGILAYTGDNSALAQVQFDQNVIKQAGTVGWLLWRGVYLSKQVSWRNRFLVGVDWLKTRMFGRDICRDY

>168

MGGVLSNCFDRERVLRTVEEAKSAAPAVGDLSTEMGGADKIFETVIVGGGVAAGYAAREFVRLGAAKGAVAMISREAVYPYERPALSKGFIMNKAKVPGFNVCVGSGGENQGEQWYVENGIETYLSTSVTSIAFDSKTLTTDKAGTIGYKNLILAMGARPVTLTDMKMDDAAKLKGIYTLREVEDTQPLLDALIENKGKKALCVGGGYIGLECAAAMTVMGLHVTMVFPEPHVMQRLFTPEMAALYEEVYAQKGIKMIKGTVASGFVGDSNGKVKEAHLKNGEKVPCDVVVVGVGAKPNVELCQDKLDMEARAVKVNGRFQSSIPDVYAVGDLATFPNAFLGGVSSRVEHVDHARRSAMHCVNVILGKETSDYAYLPYFYSREFDLSWKFYGQQSNETLTVRDPGANHMAAFWMEGSTVVGAFVESGTAEEESMIQALARAKPTIDVAALKAAKTVADAFAVLKPHCSM

>169

MAAAGFAWWSGDEGRRRGAAQCAARRGEEDVDIGADNEYEDGELYEVKVKSDKKVLVTRIKGQLFAVGALCSHYNAPLKKGVLGVDHITCAWHDAEFDLKTGKCVNGPGLKAIPTYPIRVNRGRVLVTLPSDMQDHVEPTLAKRDPKDKRVYAIVGAGAAGMAAAETLRQEGFTGRILMFGREDHPPYDRAVLSKNLHADVKKIVLRDHDFMKSAHIEYLNNSIVTHVDAKAKRIQLDDGDTYSYDKVLIATGAEPRKLFVPGHDCQNVFGLRRPEDARQIANFAKRGMRVVIVGSSFIGMEIAATLARKGCSVSVVGMETVPFERVLGLKVGAMFKKLLEEQGVEFWGNAIVKRFRGRHKAEWTRGEDMKGTPVEGVELTNGEVLACDAVVVGAGVIPNATLVEGVSMAKDGSILVDALLQSRDEPSLFAAGDVATFPYYKTGLDTRIEHWDVALQQGRTAAKNMMDKHQPFSTVPFFWTMIFGKSIRYAGHVKEFDELVVEGDLSKYQFVAYYIKDDKIEAVATCGRDPAAVGIAEAMRLNIMPTGSEVALGFCNAEMILARLKEYHKKPVKTRKR

>170

MLVKFRKCGQANIFRSISNVRKIYNVAKNNLKNNKDIERKEKIIILGSGWGGFNFLLNIDFKKYDVTLISPRNYFTFTPLLPCLCSGTLSVNVCTESIRNFLRKKNGYCGNYLQLECTDVFYEDKYINCIDIENNKVKLFYDYLIIAVGAKTNTFNINGVDKYAYFVKDIDDALKIRKKFLDILEKCTLPNISNEEKKKMLHVAVVGGGPTGVEVTAEFADFINKEVKINYKDIFNFISISIIEGGNNLLPTFTQNISDFTKENFHNLNINVLTNYYVIDVDKHSFHIQSSLNKNEKKKLSYGLLIWASGLAQTTLIQKFLKTIPVQANNAILKVDEKLRVIGIPSNNIYAIGDCKKIQPKLLHEHTNEIIKILTGNKLTSEALKLKQSELTKTFPQLSISKWDYEKNKKGEMTPQQFHDYLFEIDKNYKSPTPTAQNAKQEAYYLSNVFNNFIHTNQKFNIPSFIEKWKGSLAYIGNHQVVADLPYYELKGGRFSSTFWKVVYIQLLLSWKSRFHFFIDFIKTKWYGRPFIK

>171

MRRNLLSLNGKISTIIKSDKLKTRGISSYNILKNNINVSLERKNKNRFFFSSLKGHQKIGGGVLNSYDIFLIISLLAVPTILNNKFGKMSNSIANCSNVEKVFLIKSNELQDGEMKEIKVHEEKDTVLLVRVNNKYYCLGPKCPHYSAPLKSGVLTNEYITCPWHDAKFDIKTGECINGPSFDDIPKYEVVIEGNEVYALLPKKLEIFEKKRICECKGSCEKKNILIVGGGAATLGALETFLKLGYNGKLIICSKDAYKPYDRPTLSKNVSNCNNCDELYEEIKLKEDSYYNKSNIIYKNNVYVEKVDTENKKAHLNNGEIINFDKILITTGISPSPSPMKNMNLDNLFTLHNLSDNIKIGEYAKEGSKCVIIGSSFIACELSSALKKKNVNVTLISKDDVPFYGSFGEKIGNIVLNILKEKNIKFYPSMHPTEYIIDKRFFSRKSGNIIHGVRLNNGEVINCDYVIEALGCIPNSDFLDEKYKNVNNFIEVDKHFKVKNSDNMYAAGDVCTFPYFLTDEMVNICHWNVAIQQGRIAAHNMLRDDKKEFNFIPFFNTNIFGKNFRYSGYVKNYDKIIYEGDLLKHNFIGYFVKNDKVASIITLGNNKMASLNECMAKNKVPKVYELEGGLKNSDSMIASLKI

>172

MAMLFSSSAAGSLPSRRSRSVAAKLAPFASPIFSSPLLKQGSDTAFGLRSPTPGLSVHPRFASRGPLTRSEEAGKAPFGFSTARGEQTATETNAPRFGTSLCSSFPLSATQKSHGSREKGLTNERAFSTFSFQGFMKNVKSRNAKPYTGPPQKVVVLGTGWASVNFFRHLDPNIYDVTVISPRNYFTFTPLLPSVCAGTLSPLSCIEPVRSLTYRNGRKVADFYEAHCTDVDFKNRIVACDSRQGGHFKVKYDYLVIAVGSESNTFGIKDVAANAFFLKEVEHAMAIRKKVMNNFELAALPQTSEKERDRLLHFVVVGGGPTGVESAAEFADFIKEDMSKYFPQLIPHVSISLIEGGSRLLGTYPPDISAFAEKTLTEELHVKLLLRSTVVGVDATSVRYVSNEPGASKEPKELLHGFVLWASGVGEVPLVKKIIAENFPNVEGKPRLRGLPVDAQLRLLNQPNVYALGDCAAIAPPRLADAAQELFSKAGAAEPTPQWLGRHAPTLAQQFPQLSPLKFNFAKLQSNEHLPADQFESFLAEIDAAYRPPAPTAQNARQEGIYLAKVFNECPHPEEKADAPAFQETWNGSLAYVGSGQAVAHLPYFNIKGGFLSLPFWKAVYTQMQITWRSRTICLFDWLKTFFAGRDVGRDHEYYNH

>173

MSTKWTSFRLRTGLLSPAAVAASAVASASPAAREAPARRQKVVVVGSGWAAVSFLADLDMTRYEPVVISPRDYFTFTPLLPSVCVGTLPASACMTGVRELLVRGGVPCGSFYEGRVAEICPTEKKVRCQSTHGKAQDAREWEESYDYLVVAAGADVNTFGVPGVKENAFFVKELEDARRLRSALFDVIETASVPGVSEEEKKKLLHFVVVGAGPTGVEVAAEIDDFFQAEGATHFPQLRPLVRITVVEMLPTVLAAYNNDVQAFAKRLLEENPRVDLCLQSQVVGVGPDSVKVRTKRADGQVEEKEMPCGLLVWASGIKSPKVCLDLARKTAELREAQQQSPVILVDQQMKVRGCEGVYALGDCCRLSPPPLVQHADTLYEAATAKGAASTDWLEREAPKLSTVFPQLASSKYDFSQKPRQTQMTKEQFVKLLADIDAAYRAPAPTAQNAKQAGRYLAQTFNAFPSVEEKRRAPAFVNQTRGALVYLGHGQAAADIEGWRTFLGGAATLLLWKAAYLQMQLTLHNAVACLGGWLRTSLVGRAVCREHLDGETVYGDRRK

>174

MFWTKGLCAAALAAGAVSVAVGEGNSRTEGRKLGGSPVFSRAFGRCPRVAACAEKRTATDLGAVEEFQRGGLYELAVNGGKDKVLLSRTADGTFYCTGASCSHYSASLSKGVLTAKRTVTCPLHDAEFDLETGKCVNGPALSAIPTYPVEIKDGRVVAQIPDEIPVRARGVYAKEKRGQNTETFVLVGGGAAAATAAETLRAEGFDGRIVMICEESVPPYDRPVLTKNLNAKLDNILLRPLKALQEDLGVQVLLNSRAVGVDLKTKTVRLEGNAPDVKFDKLLLCTGSEARRLTGLPNGTARGIFTVRGKNDLQELSAFLEENKKFNSDPRVAIIGSSFVGVELAAAFHRRGCKNVTVIGQETVPFERVLGSRVGGSIKQLICSKGVRFYPQSKVVGFTSSRDRVTGVELASGEIIQADVVIVGIGSVPATKFLADQSEFALARDGAIVTDPLLRLPANPDVFVAGDIAAYPYVKTGEQIRVEHWAVAMQQGRVAALNMLGRHVPFTQIPFFWSMIFGKGMRFAGWIGSGFDEVIIEGDIDKQQFVAYYVKDDRVTAVCTMGRDPVAVAAVELLEQNLMPSPGELRQGLKNSQDVLAIAKETAATKTVKRLV

>175

MICRTSFLRKPKVVVVGTGWAGCYFVRDTKPQLAELHVLSTRNHHVLTPLLPQTTTGTLEFRSICEPITRIQPALAHLPNRFSRCFVYDINFEQKRVDCISVDNTSVGPHALVNTFDVQYDKLVLAHGAQPNTFNVPGAVERACFLREVNEARTIRKRLVQNIMTANLPVTSVEEKKRLLHTVVVGGGPTGVEFSADLAEFLRDDVKNINPELVQFCKVTVLEAGEVFSTFDLRVREWGKRRLDALGVRIVKGNVVAVQEKEVITKSGEVFSTGLVVWSTGVGPSPLTKELKVDRTRQGRISVDEHLRVLRDGVPIPDVYAIGDCATNESNPLPTLAAVASRQGVYLAKKINAELAGKPFATPFKYESLGSMVSLGTSSAVVELNGPRKLDFVGLKALFFWRSAYLSIVGSWRNRLYVIVNWLGSAIFGRDLTLINDYNDERTWLSLASEGAAREKVSRMNKVKTDGDGSNGNETTARSKVDLPATKKQNE

>176

MLWKLFTSSMLAGAVSAYRSTSSSSLAISPPTLDVVMDPSKDVTPVRCVVVGGGYTGSKLAYMLDSMFNVTFIDEKNYFELTNDIIPIIANPWSELNEEACRRLLVLHRYYLKQANVLTGTVHGVDENTVTLRDGRTVPYDLLFITVGERKPYPFATKQRTVSGRVQELKNFNEFIGTCKKVAVLGGGPVGVSLAVDLARNRKDLKVHLYHSKPELLPALPTTSQRYALETVEKCDNITVNLCSRVTDVTGYDALGRRVNETSTSMLSSLLKPLTGWLTWGTSPDEPSTFTVRYEKMHFAPRPRQSIVNQAYFGTRQPQLTSNTVESIGEEEGYDYVFSTIGDVPRPIVSGKGCTNILSEHEMPDGHYRVSTLMQLYCRPNIWAVGRCNNIPRVRGYGLSDVEARTVFRALNSVAHNPTERFMHSRDGLDLRRLNIPRMLVRLGSDDAVGSTPWSGAMVGLAAVHEFMQDRNFLVKEFQKPIFYKRQDAAKVKQRISNWAAHEITDIVDFSHC

>177

MFWKFLVSGLVGSACSAYHRGPTNVSLALDPPTLSIIMDPSKDIVPIRAVIVGGGYAGSKTAYQLDSMFAVTHIDEKNFYELTNDIIPIITNPWKEDVNPKACRRMMVLHRYYLKRSNVVTGTVIGVDAKQVYLRDGRTVPYDLLFLATGERKPFPFQTRERTISGRVQELKRFNEFLQSCKKVAVVGGGPVGTSLAHDLASTRPDLQVHLFHQRAELLPRLPGVCRRHAQEKLLSNPNLHLHLLTRVTDIDGVVLPSNGDKAASSSSPARHSCAGQLVNPPTSNAAVAESSLTLATTNAPWWRSLLNTVWPRRGVVPDQYSVHYDTLQSKVQQQPSILQQVYYGKRDEVAECGVVQARGVEEGFDYVFALTGDTPRPIQCDELEGRSRKLPNILREHETRDGHYRVSTLMQFLDHPNIFGVGRCTNLPVMRGYGSSDIETRTLFRELNSVISNPTTMFLHSRDGVQLAHMRIPRIHVRLGVDDAVGCTPWSGGMTGVSSVHEFMQDRSYLLREFQKPVFYKQQDQAKVKQRMSQWMEEEITDIVDFSHC

>178

MLRSTLRRLTKPNVVVLGTGWAGSYAAHHVDPNLCNIHVISTRNHMVFTPLLPQTTTGTLEFRSVCEPITNIQPALAKPPHRFLRSVIYDVDFDEKQVKCVGVGVVGGSENVPVNTFSVPYDYLIMAYGARPNTFNIPGVEDKAFFLREVNEARGIRKRLVQNIMTANLPTTSIAEAKRLLHTVVVGGGPTGIEFAANLAEFFREDIKNVNTSLLPYCKVTVLEAGEVLGSFDNALRRYGQLRLNQLGVEIRKTAVVGVTDEEVFTKSGEVLPTGLVVWSTGVGSGPVTKALKCDKTNRGRISIDDHLRVLRDGKPIPNVFAAGDCAANNERPLPTLAAVASRQGRYIGKETNNLLKGKQMSKPFVYRSLGSMASIGNRTAIVSLGDKFKFDLNGCAALWVWKSAYLTILGSIRSKLYVIVNWAGSQIFGRDITYIGDLAEDRMYSALAVEEVSKEMNRKKTPEMPHGVNPESSYTSAVEKEGAHKGFLPRKLAESTPLAPHGQAIEGSQVAHAAAAATSAEPAKKM

>179

MRNSKKTLLVVGGGFAGMTIITQTYKQFNIVLLDQKSYFEFVPSVFNAFIHPESIFDLTLQFKQSKFGVIFIQGRLTHIEENIAYYEGGKIEFDYCAITIGSNYTYPIKSAIPKLSDRFIELKKTQQKIIDSQTILIIGGGTVGVELACEIKASYKQKTVALITRGKILSTMPKSASDYTKKRMLDLGVEIQENYKGPSLDSNFDLVYNCKGNTYDSVRLNDNFEMFDPKKQILVDDFQRTRTNQNVYCAGDICITSQNETKTAFSAEMQGEIIAYNLKHPNKQIKSYWIPNTYIISLGGWKAVFVFESFSFGGFLPYLMKLFIEVVVVNDFRGIIGFNTLHQIMNYFVYVMLYIYMILQLLFAIAPLGSKIKQDQRVELKRIQQEIEEFKKQ

>180

MRRRILKLIPIFLFACKGIRSTCKLQSDLTEEQKKGIIQTAQMLDYNNTQLITYTSFKKPKEGELMPFVIQNGDEDYQIILTRYQGKIYAFGSLCPYDLETDLSEGICFGDKLYCPKHGCQFDITNGMVEGPPSIDNLPKFGLKENEDSIEVYAPLIVPKKIIPQYHFRDYNDQRKVIIYGGGAAAFACLTTLREFGYTGELSYISNDNYMPYDKTKMSKRIKATKPEDFFFRKDNWYSAIAIDAHLGRKISYVNNKYSNTYVELDDSNKIAYDTILIATGTDPVHPPIKGIENQEVFYFSSLDSHQQLKEKLKTINDLSIIGINTMSLEVAQTIRQEYPNIKINIIDPNDESQFQVQYGPELTNLVLDLHVEKGINIYENIKIKKFEKDSIVFKGKKKLKSDAVILFPSTVSPKTEFAEVSDHQFEFDNAGRVKVDYFQRTDIKRIFAAGSCAHTYYYTNGTGHIGDQWQACYNQGMTAAYNMLALNVPWHQIPFTYTEQFGKVLQQASSWPQFDEVHIEGDLKKWDFIIYYGMNDFVVGAVGTPSKQNRVAIVNEGIRCKNIPFLSDLKNGKYSTKDIEAAVRKIKKSGCYKSTLYKFRYDVVPEYNLWTFRDNMSTFYFPYDNAEISAAPKMKGDE

>181

MRTSARYFTNFLKNKATNSFNAFNRQQFRNNFAKQVILLTGIHLTWKYAAQNLNCEEEAKSQEELEKELKQKFEEEMRKKRVDSMKNLQRYEVCNSVDIQEGQIYPFQVKDGENGTFEVVIVRYNGKLYCVGGVDTYDGKTKLKDGICFGNKLYSPMNGSAFNIENGHPELAPAIDELPRFFVEEKNGKVILYAPKIVPKRLVPAFASRDINDVRKVVVIGAGPAALGAIESLRLNGFTGEIIMVTKGTKMPYDKTKLTKSFKYLNYDNLTLRDEDWFDSHGVNYMLGREVTFVDKTHNSPHILLEDGLKLEYDSLIIATGVKPEVRNINGIQEKDNVSFLYNIDHHKKVKQYLEKAKTITVLGNNMRAMECVSTIRREYPHIKIYVIDENEDPVIKTEFGEDIYKKILDTALDNKVKFVMKNPVDKIIGDDNLAKKIQFRSGLQIETDFVLLMPNNFKADNDFLLNNDLDQFEFDDIGRVRCDYDLRTDYKRMFAPGSGGVATYFAANDRYPHMQWNTAYHQGMTAGYNTLALNIPWHQIPFEQYEIFGKVLQHIGYANVENEVYIQGDVQNWDFVAFHAWEGEILAVTGTPSQKKTINILNEAIRLELMPHLIELKKGYKTIEQIEKEIRESKRSTCFKNLVYELRDKPNPHDVIWFHRERQSRYFNFWEEGVMPDQNYAPPTQDPVV

>182

MSADAFYEAYSNIQAAIAEGKIENGKGEYLKIIGFTKSADIKNLVTPIIAKFAGHFPDLLDKAIDALLDLCEDDSSAAIRMDAIRGFRALTEHAPQCTARVTMLLTQLLVLDEEAELQVLSKSLAAIFAADIKQASSSLFHHITNGKAGNDLLRSKIVEFFAEQVKKCKSQISANMELQQHLLDSILAGVGNAPDLTENDLALLFRTLSSFEVFKDNKHPSLLNRVKETVEAAAAANGIMDTDEGASKFNQFLIMGSVLAQKKQDTSELLRVFFSKLEIAMATLEDSTITKLMRTSCRFANSFVEATAEKILPDIYGIFAANASLPEEKISLTTAEICLYLIHQIGKKNPDIVKKTLGLKPDEPGAAETKQTLQGYVESLTNAKSKKATEAKAEVTELAKKKKTLKGDELKEASRNLEKHYIVGDFPENLCVTVLGKPGYYVSKVNAGCEDRSGKAIPQSDLIPRKYYYDGVILSFRPRRGKLKTIMKEEKQKKQGAKRKRGESSDSKMSGVNKLNMSLEGIAQARKEAQKKEAQKKEAKAAKGRGGGGRGRGRNGGAKGGRGQGRGKGKGQNQAGGGGGKNKRSQSQPPSKRRKSIDAKKNNNNQNSGKGRGAGKKGRGGRGRGGGAGGGRGGGRGGGNKGQGGQRQGQKRKRNQNQQNQQQQQQKQGRGGRGGRGRRNAQGRGKNNKRKGGRGRGNRN

>183

MAEDMLTELYAWDSEIEDVSALAADDPKRDEDKAEDAYEKIIHAASGADANDVVLSLAAQLLHKHFFRFPHVQLNVVDVLLKLCGPKRSQAVRIHTLRALLQIVKTPPDTAVSTLTSANSIVRDNSRMWMLRIDEAVSHILESEKSSVILRQVTPLRQALEERLQPNVIQEKSNSAIGNDQRDNQALILECSRKQPRDEKDITEGNDGVAVSERDTKKPKLDDEINKFQNKPAEAKFVQLKRDWSSSRGNGNNDIVSRKSESSNGRIVESEGRRPKVNAFSPRNCPPCPYLFLGSVPRHTPSGEIVEFLSPVWPEIDNMSVQIKQPDHNATAYAFVSMPTIEHARLAIHYVNENKFRGRAFLNANFARGPPVDTILFVERTGDNVSMEDKDAVRDFNFDKYDPEVWDVMCQQLERFGPLSFAEKGCVRFRSAEHAKAAIRKQLFTVMGHDIFPVYDIKEQFAIDSTRRGSNVQPKQGFALKSGRLVGGDAESYSSKDYHSRKEIGPNDGLTGRHGHDRVLSLNYGERSNALSRSPLRSDRASVLDRSRSRSPKSRYSGGRYDLPIVGSMKEHRMPRSRSRSPLSLVKHVGRHKSGYGTSADKENFRELRSKFVHRSPSPIDVRSYEKDILDEECRGRQYGRKSSGVDLRLVENFRDRRELAGEGQSHKIIPRVDSPTSSSSRRYRKNEIGSHHFNEQRHNLKASRHPYRREELMEDVRGRMPPSPPGGVLHLPRSLSRSPPRFAKNGGSIKSRVRSRSRSPHVDVRAKSRANQRPGGYHRHEDLSAGSQLSEDRPAFQVERPRYADDRSIAEVARDEQHERQRFFQQQSHGRRDFIDADLSRYGSGRVAGRGGCRGDKYDNKQPSRGDRNPGLYHQEVVPMARRSRSPLPLITHHRSVSPLPQPHRRSLSPFPPTQQKRETYGREHREEFKTRGRNSRFSPPQSPLLPSVFIPGHAGSSDIHASKSATDDLGNRLEQRYHSRHDKSRDDKSYHHQFREKRPSHLERSLTPSPARRERPSGSVGRKASVPSPHRSVNRRNEGDNILEEVSAKEISSRPKDGEDAVLQQKEIKSGGARMAREELFAGMDDLTVDYEEDDDVLDRSRSRSPKSRYSGGRYDLPIVGSMKEHRMPRSRSRSPLSLVKHVGRHKSGYGTSADKENFRELRSKFVHRSPSPIDVRSYEKDILDEECRGRQYGRKSSGVDLRLVENFRDRRELAGEGQSHKIIPRVDSPTSSSSRRYRKNEIGSHHFNEQRHNLKASRHPYRREELMEDVRGRMPPSPPGGVLHLPRSLSRSPPRFAKNGGSIKSRVRSRSRSPHVDVRAKSRANQRPGGYHRHEDLSAGSQLSEDRPAFQVERPRYADDRSIAEVARDEQHERQRFFQQQSHGRRDFIDADLSRYGSGRVAGRGGCRGDKYDNKQPSRGDRNPGLYHQEVVPMARRSRSPLPLITHHRSVSPLPQPHRRSLSPFPPTQQKRETYGREHREEFKTRGRNSRFSPPQSPLLPSVFIPGHAGSSDIHASKSATDDLGNRLEQRYHSRHDKSRDDKSYHHQFREKRPSHLERSLTPSPARRERPSGSVGRKASVPSPHRSVNRRNEGDNILEEVSAKEISSRPKDGEDAVLQQKEIKSGGARMAREELFAGMDDLTVDYEEDDE

>184

MAEAADKEFDELDAKVQQLSDAVKAGTISTLESTYVDIIGGVKKASVKCKKLAAVNIPLFFKHFQKQSDAAINGQLDLCEEQDDVTRMTAIKGLPALCEDTPEQTPKIADFLGQLLMTNKAEQRLVHSGLDRLLSRDPKTLGAMFNLIKTSEDALRERTIKFVREKLTKDSLSTLAKAHEGLEDLIGEQIKEVLRDVDGAEFQNFYAILQQLSKFDVKKGGKEAAEELLKVLVDQADLSKDFDPAEKDTIERLKGTLKLAEPLFNEGASAGNFLEYLMGKVLPKLASVPAEADRVQLLVFLADLGPRASPESAKQALEATYELIKAHLPAKPAAEGEEEPAINFTLGETLLFCLHLFGSKAPETAKTLLGLQKKKPSAYLALRANNFNKEQSTQWIAEQKKIMEARKAATSPEEKKTVEQSDKSFKERKNKWSQTFDNIKKLNMNIAKKELLDEKQLPKLVHSLRSEAKGGQGGRQGQGQGAGQQQRQQQQAQAQAGTKRAAPAKPAIDPEVVAKRQARFGPSTSPPPR

>185

METTTTTTTAAATKGTNDAMETSSSTTATPTTATVVPQVVRNDEYINTFYDIANKLDTNPSFPDDDKLFSKIIELSSKTKQTKKLSPQFISKYFKRFPTLQEKAIDCLIDLFDSSDEDVIMRVNALKAIPTICRDNPDHIAKLVDILSQLLNTDSKVEAEHTKNSLIELYKLNSVTTLNSFLTFLESEESSMEDQPASSTLLSFLKESIIPLVRTEYSKSSIETQTFFRVRILKLIAKCTSTTELDLLFQLLECFTQYKVQETITDLETNVLPVIESQSLDTIRKKLINFTKILLFKSKKPHTEINSNKLFDLYLNKIFPKVNELDETNKTELVSVFSQVTPHMTQEISMQFLEPVYNLFKATVPSKTTTPVADVDLQFTIVEALLFALSSIGSKSTSSLCKLCGFKLVTGQPSDMNADPVKYEDFLGRHRFLDEKCRETLGKAKKAIPNLGNPKDKAQLKLAQKTLLSTQNILTIMQNLLKSPPVTNINNLVISSTIFKGKQNLIHNVSPVFKPQQHQHQHQQQQVQQKQYQKYQAQQQQQQNIPIQRNQQQQQQQKSNRYQPYVTPGRRHQDLDKPKQDGVEVHYYSEKHNKPSSFKGRGPKSY

>186

MEKKTAGGETLYVSTLDASESELLDKIYKFSDDMVGVKDYAEFTEGFRSLLEAAAGSVRVKSLIATLIPKYAAHFASQAAEAVDVMVTLADDVESTVRLHSIKVLPQVVKDKSGAPLPEAVAKVTDFLVRALTNKEREGDWEHLLTAFEAVAKVGIKDVCDRLLTFLEAPEGDEQDALRESACRLLREKICSNAKELIRPSEEVEELLKTRIEKVLEVASEAEFKTMVAVLKELHIFEVGQKYGPQGLLDIVAAQALLQEPLEATAVDELQRVLECVNIALPFLQKGTVDTKMSLNIVEKVLPIFAKLEPVLQLVVLQRLTDLAPFVPKEVANASVAPTFDLLLAHLAETPEAPASPEAPASPEAPASPEAPASLEAPASPEAPASPEAPASPEAPASPEAPASLEAPAAPEAPAAQEVPAAPEATEGNGEAASPENAGDQPLPEVAFTTVEALLFLLHQYGLKDLEQVTVTSGIPLTEVAEPNVEVYKAFKDRLLYTYEVCETVAKKVQPAIPKAKAAKTPEEQERKRVLEQGVASIKNITDMCRPLVTAKPSCLQSLPSGRLSWQSAPAKGTKRVAPPPAAAEGKPAKKPVAAAYVPPSRRGAATEAK

>187

MSSEDAALSAIYKAADALLDNASHFQCMMDNAKVGPKCKQLALNVLPKYVSKFPEMADSALDILIDLFEESDKYHLKAIQGFRVIASSASSPVISKLVGVLGQIILSEDEKEVEAVNDALVQALERDVKATLSALFEQMSSDEQLRSKTTSFIQRELIPRANSLLNSSDEVQSFVSENLKRIMGSGINSKEFNLFMKVLFCMNKYKNGEEGANELLDFVHTSIDLNSDFSPNSDQADKFILCAGSAKLIFQHGASPYKLFSYISKKVLPKFEEMSETHQISLLKTVAEISPFALGSSARELDEDFSAKLNQLHTSLSLMLSESKKFAAKLREVQKKLNEENPTTDNAKKELQTKKESCLMSLKSCTNIIAMANSLKELNLKDPKFLAEKAAVNLHKRPMAEGDQCAEAALVLGSQDAGEEDNSEGKQAAEEDEAAEAGEVDEDRE

>188

MDKMDTAPSTTTTTTTTTSSSTTATSSGGSEAVNQLYDIAHQLDANPSFADDSKLFLQILELSKVSKQTKQLSSQFLSKYFKRFPALQERAVDGLIDLIEEDDIPIRVGALKSIPSICRDNPDYITKLVDILGQLLNTDYKSETEAQSFFRERILKLVTVSTDAESEVLLELFQCFPEYSIKNVVDYLDYVIPLFESKPFVQYKKKLVFLIKQLIKRSKIFPSINELDEADKTEMLTLFAQISHLANVNDSSPLFEKIFGLIKDNIPSPVPEQYEFKFQILEALLLSFSNLGNKSPALLRSLCGYKTNTGQPSDMAIDLDPKVFEEFVARLRALNEKIGVTDAKLKKAKEVIPMSDPERPKIDQTFAITQNIIVLIKNLSKNPPILHANITPTFSIKQQNNKQNKQNKQNKQQQQHQQPFKNNNNNNNNNQNQNNKSNNNNNNNSGAGAGKQTRDQPKTRYSPYVPPSMRGGGGGQRKQQQPQQQGSDDFLSVGMRKSDSSGSKIGVYIYIWFVLKNARFTERNMDVIWRIHILYSNTIDISDTECLYRNNKEVYLKSIPFLVFVSMVIALVLRIIAGFTAYHYDSDAIHQDRASMAFYAFAVQFMCIEFFCISLLWIKMALVFYAKDDLPLNRVRIIDIIVYANIAISLLFYTVFVILQAVLTKELTPRWWDAVWRIYFLVNVAVLASSLAYFGLKILIQLNSKRAMDEILIKKIKGIVGLTIILGSLIWVLNVLYLTIGIEKAPQDWLNLGIFIAENINVYCVSIILGRSYLCTRIRILLGFPEIIESNSTTSNELTVTAQNSRKTVEVSQTLDNSFIDVSVDQQ

>189

GDSVQILESAPGRYSLSERSFDLSCARKPHGMSDDSNKAHDDSAEVERVYEISKLLNEPAVTPEAEDALIEELTSVGRRGNKANRLAAQVMSQQLTRTPRAADRVVDALFDLCESTDSDLRVCAAKALTPVARASPDHAPRITSVLVQLLGSEAHLEVNNATRELGTISTLYPAAVVAALLSTVREGDDVQRARAADYFAKNIRALAGASGSDSEKLVCEALRSSASSPDASPEEFRMALGTLESLLPYAPSAGKGAELVALVAAQMKAGGTSESAADAAARTSSCLAIAARICVAHKLGVGAASELLDPVCANAAALLAPAAEGVRDSDRLGLVRALADLADTATMVDARTLMPHALALLYSAVPLPQKADEKKPQQEAAAASEQPAPTATAAAAAPDAVQVNWSLVEALLVLFCKLALRAPAQLRGLCGVEVVTAQPEEMAVAVDATAAQRFAELLKEISNSVPALQGSKTASSQPDNVAAIAVRTAQCVRAVVLKMTRFAPFVAQSKSRVTVDDLKPSWHAAPEQKKRETSAGTQQQAQPQQKRQRTASPGANDAPAAAQQKKRKHRGGKGSKQRRDAMRK*

>190

MSGAAASLYGAYDTLQSLEASNEAVEQALEDLIQASENEDVRVRRLVAGFLAQNGARSEAKQEAVATAFERLLNDANSGVASEARSQLRLIRGQHHVSLAMLPRLLRQTPLPTDSVLAVAKGHPQRFFVELASLMPQCEDEARRTELLTLTCQFASTQHDLLADAPAAESAAATCLFKLLEDVSREEFEKLFAVLKSLKIYQQQTKAEPGPPPSQRSLFTLLMQRVCLLPPYQELLTYLFDLSEANNVDPTNEESLDFFLPCLQQATDLLERGAVGTAFFHVLETKLLPKYRDLPDAFRVPLLKTTAEACSTPGLIPEAAGTLGPVVLGLAVAALPAVSASEAGDGDEQSVEATKGTEQESEQEHEQEAAPKASSPPAAAEDINYTELECLLSMLHSCNLKRFDLDGADKQDVLNGLDVVIARTTAHLKGVSQTLRMKEALPDKTEADQRQIQQLRTALGTVKNIRLLRAALDDPEKVTQPILLSWTAAARKAVRPVLGKRPNNNRPRVRGRGGDASQGVLARQGGKSRANAGKRSAHQAGFYEAPRGKVEAEADSKRAKSGGRNRRRNNKGARNGRGARQPNGGGNGSSGRIVMRQ

>191

MADLDAIYNAYNEIMDAKENATQHPDAYLTIISASRGSEKAKTMAARYIPAFFKHFPDLHLKAIDGFFDLCEDESQTIRQQAIKLLPMLCKDGPQHTIKIADVLCQLLQLDDDDLATVQGALETLMLQSPREVLAVIFRQGVKGKELREVSLDFITNNVIATKDTLFKDAEVELFFIQEMQKAMESVPDEDFATFAKIIMNSGPYKSGKLDLADLLKTYIAHITSEKPFNAGDDESIKRVLSTGKYSMPIFKRTISADPLLEFYAQNILPVAEFRKIDNKKKSSLLRLYSDAITSGYPSATVVQQAGGLVIGLLNDIVPEKEGDAFSLDFKLAECFATILNFISPKHPEIVEDAAVITRFRNVYRACQNEMSRLRRESDESKKKSAEDICKNVLVMIQEFMKPRQARTRLSIIPSWTPKPPVVPPQGVVKAQETTVKPAPSKTAKPTKTPLKPVGKATNVKAGSKPGSQQQQQQQQSQAQGSGSKRKAEQESQPKAKKPKILRRSISNSIDMSPRGNSPGQHGNKHQQQHHQHHQHQQHQGKPQSKQQGRPPQGQRRPSPPSRVRTPSGSQREGKGRISFLSR

>192

MSATTTTKGGETLYVHVNAESDKEIDFLYKNEEELSNALKAAIQEQGTQVPNLEASAKSHYEAILNIVSPNHSKKAKELACQFIPKYAANFPSLSSESMSKQLDLCEEEDKSIRINATKGLPKLCINNTVNISQVTSILTQLLKEEDPVEAKVVRQSLNELMDIDPKETFKSLLDLVATPAVQDSPKETELLREKTVEFISEIMFKKRKAIQAKLSGEDTEKEIITVIEKILRLFASSSFSNKEKEAEHMRLFLSLVARLKSYEKDSFKFMQFLRNHIPVDLTKPLDLADEEDRWKLRKLISFLRAAKKGMVHLKDNAPYFSYLMNQIFPSLNNLNTSVPEEDKLRVDTLKAITEIALFTSENHSRAILPLIYQTLKNEIPEKDAANPKLNLTNIECFLFIFHQLAFKAQGSLNEICGIKIITGQPTDNYGDFKQLRQDMLQRLGNLEAAVSPYVPRLEESIKVLKAEKKEENKEKVRMETQILNCAKNILELTRALKKNRPDFISTLTTKPSWITYRKKVKNTKRKDRDGKDNNQGKNKVQKRDNNQNKNNQRRIDSSSNNRDSGSNNRGGRGRGRGGNSNRGGRRF

>193

MDFYTAYDIVDGFTEGDRGAAVDRLVQLSKANTTQRHQALPLLCTAIGKSMAAAAAAATTTTTTTAAAATTAAQSPADTDAGGTDASSSATTDGVIRHAQAVFDLVRQEVLQHTTAEAAQGEEGTGGKQGVLKASSTGQHAPQHLDLGVVARCLAPLKDVSALAASWVKLVLDMALSTTSADVKRVGQELLQGAPASFAADVVVAAMNVAVQDEVSDGARQGSLAVIEKLVNTHATSMPPESPAVEIMAAAILEILDDVTKEEFEVLVPTLRKLPIYTNAAPCQEVVDFLFNLSELGELGESLSDAAYDMFAPCFEQALAFAQPGVSLDAFVSAIMSKVLPHLSFLAAGRIRVLHLLVAAIDNTSPDASDLLAALHPLLLEYQRRAEESQQYNWALCEALLALLDTALRKTPSYMTSLEKDDQDELIECLDILPEEDRQTKAIIHQHMGLIHVTATNNDSERRKKLMQALGIMTTLQRLASSIAACKSGRAPSTRIRFSWMDAARKRRPLLSASLDKRATPTKAATATSSTRTTASARTAASTASTASTPATASPSSRGTGPSPKRRSAPSSAEKRPKFQARYQPPVGRFSEGLTAPQAAPMDAVPQIGGTRARRPSVPQAAPAPATTKRGRGGRGGRGGRGGRGGRGGRGGGRKKNNRGGKKNKKKNNAVVRRVVISSNQ

>194

MSLVIPLIPKFFYLTADLQFTASLNAYLDLCESKWPSIRSAAIARLPGFISHSSASSLSLLPEGRELPSVMVSILLQLMLSELPAEIAAVDAALADLVTVAPVQATTAIATAMALDAATPLPAPHVERATAAVAAGLAAVTRETVVSLLAPRPDPWSAALHVVDPLLAELDPSGSGPLLSVESVGAVLDAVAAGLPAAFEPRHRVRALLAQALGQATAQPAAQSANVRSLLLAALEATSGPRPRLSTVGAVATCLTAALAADPASAGDWLGLAQQAADAAAACPPPPPSAAAACLGKLRTALDAAAAPASGDVEWRVVETLVYAYAHVAQAGGPRSDEAAAWEAATLAPLETNAAAYTVSVKAKLERLSAALVAEPVSRVARAQLQQTSSLLVDALAVLDRVACILARILAPSRPEEQYLTVGANVAPSWAPPVPPAPPPASAKRLRDEAAPSAGPLPRLTADSPRLKRPKPLGNGKPAAKPKTRLPVKSRLSVKARLG

>195

MSNITELQELLQKLEKDEAATEEDFRTFMGGSSGDEEMKKLIAQNIYKFIAKFPECQRRALHTQMKFVKDESEQVQAFGVRNLFDLVVCNPSKVIGTLVKLHSEAQGSVLTMVKSNFRSHLSNTNRVFLRNFIDVAKSEETTKEDKLAMINIIKEYITFGKDTKEFVCDVIDFAYDADPVIAVRFIMKYSELMTDHQRDDRLNKFFNNIYTKLNTDDVEQYTSTIQHSILPSISNIPFKEGKAGQRLFSIAAELIFPKLSNTDEKWQIFTLRVFADAARFANENSATELINSLYEHLFTKIGSLFSSMLVVEPILFLMSTLIKKVPEHSNEIFGITFVQTGEKKEVDEAKQAQLDLVIPYLKTEAARKIEENEKEFEGLSEEEKGKKQKSYDELKIAATNCLNFAEYLSSEETWNAKKPKNPSWAFANKERKPMKNAPKKGKPKFHHGPDDRKGPRNDGFPPRGDRPPRGDKPPRGDRPPRGDRFQKNE

>196

MPPKAKVTIPTKDEFHALNEKLQSKPVEADYKQILAGAKGDDEIKVMTARIIPQYYKEFPKSQKDALEALKTLAGDANADVQIWAVRGLKDNFVANEDDVAKVVYTVLGSENATVSDAAKKIVSDAFSNEEFAKTFTSQIKDQTPAAQAKMIAIATEKITFTEETVEQLLSVIESALNCAVEEGLILMSKNKKIIPEEKRVALVNQLLDNLDASLDSQFDEVVDSLLVTILKFGHSFGQLGRLYNIVADKVLLKFEQVPIDKKIRIVQLVADNAQNAEDAKILEQLYNNVYLKFPVEVSEDTKINFSLLEATLYAFYNLAKKFPRKASELNGILLFITGQPGENDGISEDAEKKNQFRARLNGIDTVAKSFVDHYKAKKASLEEHADIKTNTELKEERRQTKIAIRTGNNVLHFCRILKEDNYIHQKPPADVSWRAPKLKKFNKKGPKGPKGNQKNKKNFNKGGNRRNERRGGNRGDNRRRK

>197

MPPKAAAVTKESVDEAVKRLSESENPSEADFKILLNAAKAGDEEKAIAATYIPSFFDKFSKQQKPALTAVLDLAKSDSIRVRSQAIRNLKKFYEVDKSQIANALFAALGDEDERIVASAIPDVVRLLKSDEEEFRNIFFEGLPNQKPESQRQLVLIVRDEIKFTEENVEQLMNVLNVSFRTCVVEGLQLYRRNRTLIKKEQFEPLAEQLLDRLDNSLQTEFRAVCENLLIPLFKFTKTLGTESTTRLLAIIAKHVIPRFEELSDAANLQISIIRKIADVSRYAEGDEMLKELYNHLFLKIPIAGPVNFSIIEATLFAFIRLAQKSHITASNLIGTVLCYTGQPGEADASNEDAGKRVEFTRRLEYLQTICPDFVTQCIGKIEMYKNSSATTDDEKRERAENIRNAVAAKRTGNNVRHLSRILLSENPLSGKMPKGPSWKRLKQDQKFKGKRGAPGKFNRNDRGSNSRRPNTGRNNSNSNRPSRGFNGNRNNSRFNNNRSNNNNNNRSRPPRRFQRR

>198

MPPKAAAKVTVDELYECESRITHSDSPSEADYQTILSAVKSDQKERILCAMFIPRFFDKFQKLEKQSVTAMINLIKDSEPEVRVKAISDIMKLLDLDRSAITQALINSLGDSESTVSEAAIKVASRYLSLDSEFKNEFLNLIPTQAPEVQAQMVGLVRDEVTFTEETLPKLIEIISSALKHCTTEGLRLYGKNRKLVPDDQSAPLIEELLTRVDNSLDSDYEGTLSNLLIPMLPFTKTIGDSATTRFLSIIGEKVMPKIDELPIDQKLSILQKIAESARLVQNEKVLTEFYNNVFLKLPKEGEVNMLLIEVSLWIFIKLAGTFPRVASKLSGTVLVYTGQPGDADDETEDEEKKKEFKERLDYFEKVAPDFVNTCSAKLKELNSQSPEEEAEKEEKRKNITFQRKAMKAGNNVRHLVRNLQGNNPLKAKLPSAVSWSKAAPGAKGNKGKKDRGNGRRRNDNRRSNSRRDDDRRRDDDRRRGDDRRGSRRDDDRRGSRRDDDRRRDDRRRRDDDRRRH

>199

MSGDSAFECSPTIGSKRRRAAIGDGDAPATKKSFTDAEIAAEVAHSLHIREQRRAEVRAIYLNYGTLLKGSESGSEALAFQGLLDCANGSLGARRLAARLVPRFLPRFPSHVEQAFRLLSALYGHGQPFPESRGLPNGAASAPDLPDDCVMLEEAIRRDALRGLGNVLEAAVRKAERNVPVVMELVLFLMSTSGGGCASDGLPLAKTTSSAQPSAQPSTQPSTQPAQPQPMQPQPVPAPPQGHHEASAASQGHGLKHRYRRNAEAAANAEANGRSAQPSPQPPPAPPQALPQHSARNDAGTDGCGKISSGADGTGGTAVKISGGDEADERSGNGPSAAAGTRIDLAASPEPDSDAGVIRSLLFNAFCLFPRVVLAACFQPFRLPPKQRGPDDCQACELMLKLLLLRTGTTVLSAVEPPVMVPPTAARNVLADSGGMDGNGGGGVGLASRGGRDGNVDGSSVGDGSGGGSGPEPGAVGGQADDGGCLAAQVLAHVPETQAWLRLLAAAMAQGKTQHMLPASVQAQLERLLLYCTATAAPAAAAPAAAGKVSPRASPQGATAAAAPRDGSGNVTPARQGGACKPDGGAANTTTVAAQSMVGSDDNSRSVPMDLDHSISTAVRPEGSGAATAVNAAVGAGAASGTVALAGPPDTAATPAPPPPPLPQHSYHQHQHQHPQHEASSAAGLAGPAAPGTGAVAASALPPPLPPPPPPPPPLPLLSYRGSDSSGLRGGPVCRAEACLYIGGLPPGLTEAALVSDLSRLGSVESVHPFDQQQVLQQHPAHGGGLGGPQVPLGEEVYVVFVSLRDAAICYEAVTRTCRFGGTRPLVVEFCSAFPADSPAARRCVAAAAAAAAAAGGGGGSTGGGPSSTASEFVWVSLSAGTGVTPEAVVAALQAAALPVPQQILQVAGRAPGMLLHMASAAIVPQVAACLQAHFTPPPLPLPPAATSALSAAAPSAAGVSTSGLPPPPPMLAGTGGAPLGLSSSAQSGPGGHYGTALHMHHPGGGGGGSAAGPMPELSPINCRTIWIGQLHESVRDEELLSMCRQHGGDVVGHRILRGSHCAFVDFASQAGAEAAKRALHGARLGPQHIRVEWKLDSGPPPRAPPTQRHANVPLPGAVVSGPVGGGLYGLGPSGPGLAAAAAAGPQGMGALLGVAAHGNVTAATGHGMAQQPTAVVSAGIGLMGQAMTPQAVAAANLAAAAAAAAARGAFPGSRSGADRWAPVVIPPPAPTGSTPATTGMGLGQGAGGLTSGGAGFLNSGQAAMQGAAQQQTASTPQQSAHLQVGPLGTGVMNPAVAAVARQAAPILLAHQQLVAGLLSGGGGGGGSGGVMHTPHNSLATPQQGPTGFAAQPQPQHLHPHSHPHTSGSSGGGALPHQTLSSSQAHAQTSPLLGHLHNARAYPGPPPLHPQQHTSQQQGSMGLPGHSSTPLPLHQPHALGMGGPSPQQPQQQPHALSHHQTPPQQPQQLRPGDWPPALSHGHSHGMAGAHLGASPGLQRPGGSHAQVPRFEPGSGTGPPLPNSPPLPHDSTVAAAAAGVSGGCAGPPHGQPQQQLQQQAGSKLGVSQSQQHLNPHVHTLSQNQQQLQQQQQQQQQPPLQQPQQQQPHPHPQQQQQLSSGGSQGGVTWQGALAKSGMHMCTLLCTTGGASAASGATPGEREPVTWPATLDVKLRVDLSYVVHSLYSHTAPHARALRRLVTSGGPEQRNKLNDFLSYLADKNRAGVIKLEAAAGLPPRTLYLVPPSEQVCAALGAEWSTGEPFLLALVVPTAGGTAGGNKGG

>200

MAATVEELYRNYGILADAKEDLSKVILDGVKGGPKEKRLAAQFIPKFFSSFPELADAAINAQLDLCEDEDVSIRRQAIKELPRFASGENLPRVADILTQLLQTDDSAEFNQVNTALISIFKIDAKGTLGGLFSQILQGEDIVRERAIKFLSTKLKTMPDDAMTKEVEDYIFIETKKVLEDVTGEEFVLLMRILSGLKTMQTVSGRQQLVELVVEQAFLEQALNPADADSVDRLLQCTRQALPLFSKNVHSTRFVTYFCEFVLPNLSLLTSPVAELDIQLEVLKLLAEMSPYCGDMDKLEVNLNMLFEKLLEFMPLPPEEENGENAANEEPKLQFSYVECLLFSFHQLGKKLPDFLIDKISAEKLKDFKIRLQYFARGLQVYIRQLRVALQGKTGDALKTEENKIKVVALKITNNINVLIKDLFHNPPSYKSTVTLSWKPVQKTEAAAAAAAIGQKRQSGEDIGATVTTKKLPTNLPRRDARQIYNPPSGKYSASIGNFSYGFPLYTCNFWSHRLETAVPVKIEPGLPALGRTELEASIVSRTEGEMNDSNHRTFPNGAARDVTGFVIHHAQPAVDMESFNVVGTLQPAERALFWVGALTTASLALWLLYKIITGFRIWVLGNGDLLSPKLGKWAVVTGATDGIGKSYAEELARRGFSMMLISRSQEKLDDVAKSLESTYNVETKTIAVDYGQNDIYPKIEKGLAGLEIGVLVNNVGISYPYPEFFLHIPDLENFITNMINVNITSVCQAFVDFFSRGLQAEYKCKGIIIQSVLPFFVATKMTKIRKPTLDKPTPERYVAAELTTVGLQDQTNGYFPHAVMGWVTTVLAPIKLVLYLGLRMNKAQRGGYLRRRKLR

>201

MAATVEELYRNYGILADAKEDLSTHKDAYQVILDGVKGGAKEKRLAAQFIPKFFSSFPELADAAINAQLDLCEDEDVSIRRQAIKELPRFASGENLPRVADILTQLLQTDDSAEFNQVNTALISIFKIDAKGTLGGLFSQILQGEDIVRERAIKFLSTKLKTMPEDIMTKEVEDYIFVETKKVLEDVTGEEFVLLMRILSGLKSMQTVSGRQQLVELVVEQAFLEQALNPADTDSVDRLLQCTRQALPLFSKNVHSTRFVTYFCEFVLPNLSQLTSPVAELDIQLEVLKLLAEMSPYCGDMDKLEVNLNMLFEKLLEFMPLPPEEENGENAANEEPKLQFSYVECLLFSFHQLGKKLPDFLIDKVSAEKLKDFKIRLQYFARGLQVYIRQLRVALQGKTGDALKTDENKIKVVALKITNNINVLIKDLFHNPPSYKSTVTLSWKPVQKTEAAAAIGQKRRSGEDIGATATTKKHPTNLPRRDARQIYNPPSGKYSASIGNFSYEQRGGFRGGRGRGFGGRGNRSRGRIY

>202

MPTVEELYRNYGILADATETAGQHKDAYQVILDGVKGGAKEKRLAAQFIPKFFKHFPELADSAINAQLDLCEDEDVSIRRQAIKELPQFATGDNLPRVADILTQLLQSDDSAEFNLVNNALLSIFKMDAKGTLGGLFSQILQGEDIVRERAIKFLSTKLKTLPEEVLTKEVEEFILAESKKVLEDVTGEEFVLFMKILSGLKSLQTVSGRQQLVELVAEQADLEQTFNPSDPDCVDRLLQCTRQAVPLFSKNVHSTKFVTYFCEHVLPNLSALTTPVEGLDIQLEVLKLLAEMSSFCGDMEKLESNLKKLFDKLLEYMPLPPEEAENGENAGGEEPKLQFSYVECLLYSFHQLGRKLPDFLTAKLNAEKLKDFKIRLQYFARGLQVYIRQLRLALQGKTGEALKTEENKIKVVALKITNNINVLIKDLFHIPPSYKSTVTLSWKPVQKADANQKRTSEDTTSSSPPKKASAGPKRDARQIYNPPSGKYSSNLGSFSYEQRGGFRGGRGRGWGGRGNRSRGRIY

>203

MPTVEELYRNYGILADATEQVGQHKDAYQVILDGVKGGTKEKRLAAQFIPKFFKHFPELADSAINAQLDLCEDEDVSIRRQAIKELPQFATGENLPRVADILTQLLQTDDSAEFNLVNNALLSIFKMDAKGTLGGLFSQILQGEDIVRERAIKFLSTKLKTLPDEVLTKEVEELILTESKKVLEDVTGEEFVLFMKILSGLKSLQTVSGRQQLVELVAEQADLEQTFSPSDPDCVDRLLQCTRQAVPLFSKNVHSTRFVTYFCEQVLPNLSTLTTPVEGLDIQLEVLKLLAEMSSFCGDMEKLETNLRKLFDKLLEYMPLPPEEAENGENAGNEEPKLQFSYVECLLYSFHQLGRKLPDFLTAKLNAEKLKDFKIRLQYFARGLQVYIRQLRLALQGKTGEALKTEENKIKVVALKITNNINVLIKDLFHIPPSYKSTVTLSWKPVQKVEIGQKRTSEDTSSGSPPKKSPGGPKRDARQIYNPPSGKYSSNLGNFNYERSLQGK

>204

MPTVEELYRNYGILADATEQVGQHKDAYQVILDGVKGGTKEKRLAAQFIPKFFKHFPELADSAINAQLDLCEDEDVSIRRQAIKELPQFATGENLPRVADILTQLLQTDDSAEFNLVNNALLSIFKMDAKGTLGGLFSQILQGEDIVRERAIKFLSTKLKTLPDEVLTKEVEELILTESKKVLEDVTGEEFVLFMKILSGLKSLQTVSGRQQLVELVAEQADLEQTFNPSDPDCVDRLLQCTRQAVPLFSKNVHSTRFVTYFCEQVLPNLSSLTTPVEGLDIQLEVLKLLAEMSSFCGDMEKLETNLRKLFDKLLEYMPLPPEEAENGENAGNEEPKLQFSYVECLLYSFHQLGRKLPDFLTAKLNAEKLKDFKIRLQYFARGLQVYIRQLRLALQGKTGEALKTDENKIKVVALKITNNINVLIKDLFHIPPSYKSTVTLSWKPVQKVELGQKRATEDTTSGSPPKKSSAGPKRDARQIYNPPSGKYSSNLSNFNYERSLQGK

>205

MPTVEELYRNYGILADATEQVGQHKDAYQVILDGVKGGTKEKRLAAQFIPKFFKHFPELADSAINAQLDLCEDEDVSIRRQAIKELPQFATGENLPRVADILTQLLQTDDSAEFNLVNNALLSIFKMDAKGTLGGLFSQILQGEDIVRERAIKFLSTKLKTLPDEVLTKEVEELILTESKKVLEDVTGEEFVLFMKILSGLKSLQTVSGRQQLVELVAEQADLEQTFNPSDPDCVDRLLQCTRQAVPLFSKNVHSTRFVTYFCEQVLPNLGTLTTPVEGLDIQLEVLKLLAEMSSFCGDMEKLETNLRKLFDKLLEYMPLPPEEAENGENAGNEEPKLQFSYVECLLYSFHQLGRKLPDFLTAKLNAEKLKDFKIRLQYFARGLQVYIRQLRLALQGKTGEALKTEENKIKVVALKITNNINVLIKDLFHIPPSYKSTVTLSWKPVQKVEIGQKRASEDTTSGSPPKKSSAGPKRDARQIYNPPSGKYSSNLGNFNYEQRGAFRGSRGGRGWGTRGNRSRGRLY

>206

MAVTIEELYRNYGILADAKPEDLSQHKDAYQGILDGVKGGPKEKRLAAQFIPKFFSSFPELADAAINAQLDLCEDEDVSIRRQAIKELPRFAAGENIVRVADILTQLLQTDDSAEFNQVNTALVSIFKMDAKATLGGLFSQILQGEDIVRERAIKFLSIKLKTMPEDAMTKEVEDYIFTETKKVLEDVTGEEFVLLMRILMVLKGLQTMSGRQQLVELVVEQAFLEQALNPADPDTVDRLLQCTRQALPLFSKNVHSTRFVTYFCDHVLPNLSSLTSPVAELDIQLEVLKLLAEMSPFCGDMEKVETNLTMLFEKLLELMPLPPEAEGENGENTLSDEPKLQFSYVECLLFSFHQLGKKLPDFLIDKINAERLKDFKIRLQYFARGLQVYIRQLRVALQGKTGDALKTEENKIKVVALKITNNINVLIKDLFHNPPSYKSTVTLSWKPVQKAEAVALKRPSGEDMGAGSTMKKQLSPPLPRRDARQIYNPPSGKYSATIGNFSNEQRGGFRGGRGRGFGGRGGRSRGRIY

>207

MPTVEELYRNYGILADATEQVSQHKDAYQVILDGVKGGTKEKRLAAQFIPKFFKHFPELADSAINAQLDLCEDEDVSIRRQAIKELPQFATGENLPRVADILTQLLQTDDSAEFNLVNNALLSIFKMDAKGTLGGLFSQILQGEDIVRERAIKFLSTKLKTLPDEVLTKEVEELILTESKKVLEDVTGEEFVLFMKILSGLKSLQTVSGRQQLVELVAEQADLEQTFNPSDPDCVDRLLQCTRQAVPLFSKNVHSTRFVTYFCEQVLPNLSTLTTPVEGLDIQLEVLKLLAEMSSFCGDMEKLETNLRKLFDKLLEYMPLPPEEAENGENAGNEEPKLQFSYVECLLYSFHQLGRKLPDFLTAKLNAEKLKDFKIRLQYFARGLQVYIRQLRLALQGKTGEALKTEENKIKVVALKITNNINVLIKDLFHIPPSYKSTVTLSWKPVQKVEMGQKRTSEDTTSSSPPKKSPAGPKRDARQIYNPPSGKYSSNLGNFNYEQRGAFRGSRGGRGWGTRGNRSRGRLY

>208

MAVTIEDLYRNYGVLADAKDNLSQHKDAYQVILAGVKGGPKEKRLAAQFIPKFFNSFPELADAAINAQLDLCEDEDVSIRRQAIKELPRFATGENIFRVADILTQLLQTDDTAEFNQVNVALISIFKMDAKGTLGGLFSQILQGEDIVRERAIKFLSTKLKTLPDDIMTKEVEEYVFAETKKVLEDVTGEEFVLLMRVASGLRVLQTVNGRQQLVELVVEQADLDQALNPADPDAVDRLLQCTRQALPLFSKNVHSTRFVTYFCEHVLPNLSTLTSPVAELDIQLEVLKLMAEMSPYCGDMEKLEANLTILFTKLLEYMPLPPEEVENGENSASEEPKLQFSYVECLLFSFHQLGKKLPDFLLDKVDGERLKDFKIRLQYFARGLQVYIRQLRVALQGKTGDALKTEENKIKVVALKITNNINILIKDLFHNPPSFKSTVTLSWKPVQKTEAVAAKRLSSEEMGSGAATKKQITPLPRRDARQIYNPPSGKYSSSIGNFNYEQRGGFRGGRGRGFGARGNRSRGRIY

>209

MALPPLVLGNGGHPTGNSLKLGSSHRLPDSDTGLGVWPEIPSVSPLRSASDTSWDSSPISRDPPGPTMPGAAEENSSRTSYHGMFRMYLDSPNHRGGENQGLPKQAASTRNSKEQGARRLARDLRTRSHHCSPGTGAQAEPPFASSILEGYQTTNLAIHRDTDAFLPYPGQPIPQELICKWTDSKGHPSRQPCSRIFSTMYELVLHVTMEHVGGPEQTNHICDWKGCAREKKPFKAKYKLINHIRVHTGERPFLCPFPGCEKVFARAENLKIHKRIHTGEKPFGCEFAGCDRRFANSSDRKKHTHVHSSDKPYRCKVRGCEKSYTHPSSLRKHLKTHRGLGPALSTATTKPRAAGEPTGAQEPGAGACQSSASPAACLTRRSAKPGSDPPARKPARTGFNQLGAKRFLSVENASSWLSLARSRRARFEPVTVTFAAS

>210

MAVTIEDLYRSYGILADAKDNLSQHKDAYQVILDGVKGGPKEKRLAAQFIPKFFSSFPELADAAINAQLDLCEDEDVSIRRQAIKELPRFATGENIFRVADILTQLLQTDDTAEFNQVNVALVSIFKIDAKGTLGGMFSQILQGEDVVRERAIKFLSTKLKTLPEDVMSKEVEEYVFAETKKVLEDVTGEEFVLLMRVVSGLQALQTVNGRQQLVELVVEQAFLEQALNPADPDTVDRLLQCTRQALPLFSKNVHSTRFVTYFCEHVLPNLSALTSPVAEIDIQLEVLKLLAEMSPFCGDMEKLEANLNMLFTKLVEFMPLPPAEAENGENSTSEEPKLQFSYVECLLFGFHQLGRKLPDFLLDKVDAERLKDFKIRLQYFARGLQVYIRQLRVALQGKSGDALKTEENKIKVVALKITNNINVLIKDLFHNPPSYKSTVTLSWKPVQKAEAVAVKRPSSEGMGSGESTKKPISLLPRRDARQIYNPPSGKYSASIGNFNYEQRGGFRGGRGRGFGARGGRSRGRIY

>211

MAVTIEDLYRNYGVLADAKDNLSQHKDAYQVILDGVKGGPKEKRLAAQFIPKFFSSFPELADAAINAQLDLCEDEDVSIRRQAIKELPRFATGENILRVADILTQLLQTDDTAEFNQVNTALLSIFKMDAKGTLGGLFSQILQGEDIVRERAIKFLSAKLKTLPEDVMTKEVEDYIFAETKKVLEDVTGEEFVLLMRVVSGLRVLQTVNGRQQLVELVVEQAFLEQALNPADPDTVDRLLQCTRQALPLFSKNVHSTRFVTYFCEHVLPNLSTLTSPVAELDIQLEVLKLLAEMSPFCGDMEKLEANLNMLFTKLVEYMPLPPEEVENGENSASEEPKLQFSYVECLLFGFHQLGKKLPDFLLDKVDAERLKDFKIRLQYFARGLQVYIRQLRVALQGKTGDALKTDENKIKVVALKITNNINVLIKDLFHNPPSFKSTVTLSWKPVQKTEAVAPKRPSGEEMGSGGGTKKQIPSLPRRDARQIYNPPSGKYSASIGNFNYGEQRGGFRGGRGRGFGTRGTRSRGRMY

>212

MSADNIEKLYQNYGILADAKDDISKHEKEYLEILSAVKGSDKEKRLASQFIAKFFHSFPNLAEQAIEAQFDLCEDDDVAIRKQAIKDLPVLCKDNKENTQRIADILAQLLQSEDTTEINVVTNSLLAIVKSDPKGALTGIFSQIHQSADTEVTNEIVRERCIKFLATKVKQLGREVINKEAEDLIIAECRKILEDVVAEEFEHIIELLTWSRLGKTPAGKKELVQLIAALAFAPEDWHPEDPEYVDRILQCTQHALPLFSPQVDSNQFINFFSEHVLSRWDDITTPEGTDPKLELLKMYAEITEHCGEVEKLQDKINTIYDILMKYLPEAPLENEENKAEKSEEESKKEETTAVPSLQFSHVECALYALHSLCRKAPDALSADAARLKALRLRLQYTARLTQGYIKKLKEVTQNKKGEDANSEENKLKVVALKTTSNINTLIRDIFRTPPSFKSKVQLSFQSKKAEKEEKQLSNSEVKEKQPHAGQKRHQPITFDNGEEKGSPEKRARSGDRNLKMYTPPSGKYSSRLNNTGRFSGPNPGRGRGGYGRRDFRNSGAPFRRRSNY

>213

MSTDSIEKLYKNFGILADAKDKLVQHEKEYLEILTAVKGSPKEKRLASQFIARFFKHFPKLADQAIDAHLDLCEDEDMAIRKQAIKDLPALCKDNKEHTARIADILAQLLQAEDSSELAVVHNSIMSLMKSDPKGTLSGFFSQILNGDDGTRERCIKFLATKLKAIGHDIITKEPEDLLIAECKKVLQDVTADEFHSIMEILAWTRLGSTVTGQQELVDITIEQAELSVPFKHTNVEQWNRLVQCVKHALPFFSSQIDSSKFVSYICVQVLPHLSLMTSPDGRDIQLELLKLLAELTVFCGIIEKPEDKVQQLYNTLITYMPLPPATEITDVPKLQFSHVECLMYAFHKLCKQTPEFLIKDPEQLKEFRLRLQYFARGIQGYIKKLREAISGKTEEELKSEENQLKVVALKTTNNINTLIKDLFHSPPSFKSIIHLSWKTPCNDKKNEKNSAQKRHTPITFGNDNSPNKRSKEDKNNKREIYTPPSGKYSSNISNYGRGRFKGNRPRGRGAFRTRGRGPWRKNFY

>214

MEAEDNIQKMYKYFGILADAKENIAEKEPEYLEILSAVKGSTKEKRLASQFITRFFKHFPNLAAQALEAQLDLCEDEDICIRKQAIKDLPVLCKESKEYLTKIADILAQLLQADDPQELLTAQNSLLSLFKIDAKGALTGIFSQMQSNEEVVRERSMKFILNKVMALGKEIIKRDVEDLIIAECKKVMQNITCEEFETLMTILSSTHLINTPDGQKELVELLASTAELDQFFNPKDLDQVNRFITCLDFAIPFFSAHVESTKFIVYICELLNRYTLIKDNDKQFIILKSLAESVPFCGKLMNPEAVVGQVYQALLDLVTVPENDSNKKVEDMDLHRVEALLYTFHKLGKQCPDFLSKDPERQKDFKKKLLYVGTCTQTFVKIVRQDLKEKEEEDVKNDPVVEKKLEGLRLACNINTLIKELFNIPPRFKATVILSWISGATKSKLAQIIQEGKKHEPIIVDGKSKRIDGSNNSQQIYQPPKDKFSAKFSNNTTNNNRRGGGTGWNTKRSFDTNNGNRRSWRPY

>215

MAGDNIEKLYKNYDILAAAKDEISQHEKEYLEILAAVKGSDKEKRLASQFIAKFFNSFPTLSEQAIEAQFDLCEDDDVAIRKQAIKDLPVLCKEHKEHTQRIADILAQLLQSEDSTEINVVTNSLVTILKSDPKGALSGIFSQIHQNTDGELANEIVRERCIKFLSSKIQQLGREIINKEAEELIITECKKILEDVVAEEFEHIMELLTWSRLGKTPLGKKELVQIVAALAFSPDDWHPEDPEYIDRLIQCTQHAVPLFSPQVDSTQFINFFCDHVLPKWKDIVVADGASDSKLELLKIFAEITEYSGDLENAQQRIDTVFEVLMDYLPEAPVEDETQKTEEDKVEETKTAPSLQFSHVECALFALHSLCRKSPNSLTSDAAKLKMLRLRLQYTARLTQGYIKKLKEVTQSQKTEDENSEESKLKVAALKTTSNINTLIRDIFRTPPSFKSKVQLSFKSKKSEKEEKQTASTSDATKDSPQKRHRPITFDNGAEKESPEKRSRSSDRNLKMYTPPSGKYSSRINSSRFSGSSGRGRGYGRRDYRNNGASFRNRSNY

>216

MSDNLEKLYEKYNILSDAKDKVTQHSREYVDSIEGIKGNENEKMLAAQILSKFFKYFPSLQDKALNGLLDLCEDEETKIRACAMRYLVSICKDVKEHITKVTDILAQMMQLEEQRDYTTASWCLLQLWKEDSTNVLRTMYNHIRSLSNAPARVKCLQFINKKLIRPIDSQPPEIESIVVEESKNFLQDDISSDEIIMIISCLKHSKYGKTAAGQQELLDFISEIMELDRDFDPLEDCIVDKIIICTTHALPLFSAKNESTKFVAYYCDQIFPQWEKIGTLEQGELFQLQLLRHLAELSMYCGKFENPSLHVVQIFDKIKLYMPSPPENADVYKMPNLEFSFVECLLYAFHRLARQCPDFLTHDPQILKDFRARLVYFSRGVQGCNKVLSTKPIKALDPTNAAKVKIAPSLLNNINVLIKDLFYQPPMYKCNVTLSFKSEALEKELKKTSVASVHKRHVPITFDNGPTNSKQSRPSRSGDNVKLYTPP

>217

MAVTIEELYRNYGILADAKENLSQHKDAYQAILDGVRGGPKEKRLAAQFIPKFFSSFPELADAAINAQLDLCEDEDVSIRRQAIKELPRFATGENIFRVADILTQLLQTDDTAEFNQVNVALISIFKMDAKGTLGGLFSQILQGEDIVRERAIKFLATKLKTLPEDVMAKEVEEYIFSETKKVLEDVTGEEFVLLMRVVSALRVLQSVNGRQQLVELVVEQADLEQALNPADPDAVDRLLQCTRQALPLFSKNVHSTRFVTYFCEHVLPNLSTLTSPVAELDIQLEVLKLLAEMSPFCGDMEKLEANLNTLFTKLVEFMPLPPDEVENGENSASEEPKLQFSYVECLLFSFHQLGKKLPDFLVDKVDAERLKDFKIRLQYFARGLQVYIRQLRMALQGKTGDALKTEENKIKVVALKITNNINVLIKDLFHNPPSYKSTVTLSWKPVQKPEAAAPKRPSGEEMGSGASTKKQITPLPRRDARQIYNPPSGKYSASIGNFTYEQRGGYRGGRGRGFWARGSRSRGRIY

>218

MEAEDNIQKMYKYFGILADAKENIAEKEPEYLEILSAVKGSTKEKRLASQFITRFFKHFPNLAAQALEAQLDLCEDEDICIRKQAIKDLPVLCKESKEYLTKIADILAQLLQADDPQELLTAQNSLLSLFKIDAKGALTGIFSQMQSNEEVVRERSMKFISNKVMGLGKDIIKRDVEDLIIAECKKVMQNITCEEFETLMTILSLTHLINTPDGQKELVELLASTAELDQFFNPKDLDQVNRFITCLDFAIPFFSAHVESTKFIVYICELLNRYTLIKDNDKQFIILKLLAESVPYCGKLMNPEAVVGQVYQALLDLVTVPENDSNKKVEDMDLHRVEALLYTFHKLGKQCPDFLSKDPERQKDFKKKLLYVGTCTQTFVKIVRQDLKEKGEEDVKNNPIVEKKLEGLKLAWNINTLIKELFNIPPRFKATINLSWLEGATKTKLAQIIQEGKKHEPITVDGKSKRIDGSNNSQQIYQPPKDKFSAKFSNNTTNNNRRGGGTGWNTKRSFDTNNTNRRSWRPY

>219

MPTVEELYRNYGILADATETAGQHKDAYQVILDGVKGGAKEKRLAAQFIPKFFKHFPELADSAINAQLDLCEDEDVSIRRQAIKELPQFATGDNLPRVADILTQLLQSDDSAEFNLVNNALLSIFKMDAKGTLGGLFSQILQGEDIVRERAIKFLSTKLKTLPEEVMTKEVEEFILTESKKVLEDVTGEEFVLFMKILSGLKSLQTVSGRQQLVELVAEQADLEQTFNPSDPDCVDRLLQCTRQAVPLFSKNVHSTKFVTYFCEHVLPNLSSLTTPVEGLDIQLEVLKLLAEMSSFCGDMEKLESNLKKLFDKLLEYMPLPPEEAENGENAGNEEPKLQFSYVECLLYSFHQLGRKLPDFLTAKLNAEKLKDFKIRLQYFARGLQVYIRQLRLALQGKTGEALKTEENKIKVVALKITNNINVLIKDLFHIPPSYKSTVTLSWKPVQKADASQKRASEDTTSSSPPKKASAGPKRDARQIYNPPSGKYSSNLGSFSYEQRGGFRGGRGRGWGGRGNRSRGRIY

>220

MPTVEELYRNYGILADATETAGQHKDAYQVILDGVKGGAKEKRLAAQFIPKFFKHFPELADSAINAQLDLCEDEDVSIRRQAIKELPQFATGDNLPRVADILTQLLQSDDSAEFNLVNNALLSIFKMDAKGTLGGLFSQILQGEDIVRERAIKFLSTKLKTLPEEVLTKEVEELILTESKKVLEDVTGEEFVLFMKILSGLKSLQTVSGRQQLVELVAEQADLEQTFNPSDPDCVDRLLQCTRQAVPLFSKNVHSTRFVTYFCEHVLPNLSSLTTPVEGLDIQLEVLKLLAEMSSFCGDMEKLESNLKKLFDKLLEYMPLPPEEAENGENAGNEEPKLQFSYVECLLYSFHQLGRKLPDFLTSKLNAEKLKDFKIRLQYFARGLQVYIRQLRLALQGKTGEALKTEENKIKVVALKITNNINVLIKDLFHIPPSYKSTVTLSWKPVQKADAGQKRTSEDTTSSSPPKKAPAGPKRDARQIYNPPSGKYSSNLGSFSYEQRGGFRGGRGRGWGGRGNRSRGRIY

>221

MPTVEELYRNYGILADATEQVGQHKDAYQVILDGVKGGTKEKRLAAQFIPKFFKHFPELADSAINAQLDLCEDEDVSIRRQAIKELPQFATGENLPRVADILTQLLQTDDSAEFNLVNNALLSIFKMDAKGTLGGLFSQILQGEDIVRERAIKFLSAKLRTLPDEVLTKEVEELILTESKKVLEDVTGEEFVLFMKILSGLKSLQTVSGRQQLVELVAEQADLEQTFNPSDPDCVDRLLQCTRQAVPLFSKNVHSTRFVTYFCEQVLPNLSTLTTPVEGLDIQLEVLKLLAEMSSFCGDMEKLETNLRKLFDKLLEYMPLPPEEAENGENAGNEEPKLQFSYVECLLYSFHQLGRKLPDFLTAKLNAEKLKDFKIRLQYFARGLQVYIRQLRLALQGKTGEALKTEENKIKVVALKITNNINVLIKDLFHIPPSYKSTVTLSWKPVQKVEVGQKRANEDTTSGSPPKKSTAGPKRDARQIYNPPSGKYSSNLGNFNYEQRGAFRGSRGGRGWGARGNRSRGRLY

>222

MMSTAECIIVSEGEDESHRMMGTSLFLAPGVRCTTDTSCETGLRRGGRPPRQAPDCFPGGLDHKDAYQAILDAVKGGTKEKRLAAQFIPKFFKHFPELADSAINAQLDLCEDEDVSIRRQAIKELPQFATGDNLPRVADILTQLLQSDDSAEFNLVNNALLSIFKMDAKGTLGGLFSQILQGEDIVRERAIKFLSTKLKTLPEEVMTKEVEEFILTESKKVLEDVTGEEFVLFMKILSGLKSLQTVSGRQQLVELVAEQADLEQTFNPSDPDCVDRLLQCTRQAVPLFSKNVHSTKFVTYFCEHVLPNLSSLTTPVEGLDIQLEVLKLLAEMSSFCGDMEKLESNLKKLFDKLLEYMPLPPEEAENGENAGNEEPKLQFSYVECLLYSFHQLGRKLPDFLTAKLNAEKLKDFKIRLQYFARGLQVYIRQLRLALQGKTGEALKTEENKIKVVALKITNNINVLIKDLFHIPPSYKSTVTLSWKPVQKADASQKRASEDTTSSSPPKKASAGPKRDARQIYNPPSGKYSSNLGSFSYEQRGGFRGGRGRGWGGRGNRSRGRIY

>223

RRGGRSPRQAPDCFPGRLDHKDAYQAILDAVKGGTKEKRLAAQFIPKFFKHFPELADSAINAQLDLCEDEDVSIRRQAIKELPQFATGDNLPRVADILTQLLQSDDSAEFNLVNNALLSIFKMDAKGTLGGLFSQILQGEDIVRERAIKFLSTKLKTLPEEVMTKEVEEFILTESKKVLEDVTGEEFVLFMKILSGLKSLQTVSGRQQLVELVAEQADLEQTFNPSDPDCVDRLLQCTRQAVPLFSKNVHSTKFVTYFCEHVLPNLSSLTTPVEGLDIQLEVLKLLAEMSSFCGDMEKLESNLKKLFDKLLEYMPLPPEEAENGENAGNEEPKLQFSYVECLLYSFHQLGRKLPDFLTAKLNAEKLKDFKIRLQYFARGLQVYIRQLRLALQGKTGEALKTEENKIKVVALKITNNINVLIKDLFHIPPSYKSTVTLSWKPVQKADASQKRASEDTTSSSPPKKASAGPKRDARQIYNPPSGKYSSNLGSFSYEQRGGFRGGRGRGWGGRGNRSRGRIY

>224

MPTVEELYRNYGILADATETAGQHKDAYQVILDGVKGGAKEKRLAAQFIPKFFKHFPELADSAINAQLDLCEDEDVSIRRQAIKELPQFATGDNLPRVADILTQLLQSDDSAEFNLVNNALLSIFKMDAKGTLGGLFSQILQGEDIVRERAIKFLSTKLKTLPEEVMTKEVEEFILTESKKVLEDVTGEEFNLFMKILSGLKSLQTVSGRQQLVELVAEQADLEQTFNPSDPDCVDRLQQCTRQAVPLFSKNVHSTKFVTYFCEHVLPNLSSLTTSVEGLDIQLEVLKLLAEMSSFCGDMEKLESNLKKLFDKLLEYMPLPPEEAENGENAGNEEPKLQFSHVECLLYSFHQLGRKLPDFLTAKLNAEKLKDFKIRLQYFARGLQVYIRQLRLALQGKTGEALKTEENKIKVVALKITNNINVLIKDLFHIPPSYKSTITLSWKPVQKADASQKRASEDTTSSSPPKKASAGPKRDARQIYNPPSGKYSSNLGSFSYEQRGGFRGGRGRGWGGRGNRSRGRIY

>225

MPTVEELYRNYGILADATETAGQHKDAYQAILDGVKGGAKEKRLAAQFIPKFFKHFPELADSAINAQLDLCEDEDVSIRRQAIKELPQFATGDNLPRVADILTQLLQSDDSAEFNLVNNALLSIFKMDAKGTLGGLFSQILQGEDIVRERAIKFLSTKLKTLPEEVLTKEVEEFILTESKKVLEDVTGEEFVLFMKILSGLKSLQTVSGRQQLVELVAEQADLEQTFNPTDPDCVDRLLQCTRQAVPLFSKNVHSTKFVTYFCEHVLPNLGSLTTPVEGVDIQLEVLKLLAEMSSFCGDMEKLESNLKKLFDKLLEYMPLPPEEAENGENASSEEPKLQFSYVECLLYSFHQLGRKLPDFLTAKLNAEKLKDFKIRLQYFARGLQVYIRQLRLALQGKTGEALKTEENKIKVVALKITNNINVLIKDLFHIPPSYKSTVTLSWKPVQKADASQKRTSEDTTSSSPPKKASAGPKRDARQIYNPPSGKYSSNLGSFSYEQRGGFRGGRGRGWGGRGNRSRGRIY

>226

MRVPNRDPRTVTVQYEYMYRATPNHRSAECCRTACFGSKSLAALCISHMFIDVLSLNLQHKDAYQVILDGVKGGPKEKRLAAQFIPKFFSSFPELADAAINAQLDLCEDEDVSIRRQAIKELPRFATGENILRVADILTQLLQTDDTAEFNQVNAALISIFKIDAKGTLGGLFSQILQGEDIVRERAIKFLSAKLKTLPEDVMTKEVEEYVFAETKKVLEDVTGEEFVLLMRVVSGLRVLQTVNGRQQLVELVVEQAFLEQALNPADPDTVDRLLQCTRQALPLFSKNVHSTRFVTYFCEHVLPNLSTLTSPVAELDIQLEVLKLLAEMSPFCGDMEKLEANLNMLFTKLLEFMPLPPEEVENGENSANEEPKLQFSYVECLLFGFHQLGKKLPDFLLDKVDAERLKDFKIRLQYFARGLQVYIRQLRVALQGKTGDALKTEENKIKVVALKITNNINVLIKDLFHNPPSFKSTVTLSWKPVQKTEAVAAKRPSGEEMGSGGSTKKQISPQPRRDARQIYNPPSGKYSATIGNFNYEQRGGFRGGRGRGFGARGNRSRGRIY

>227

MAATVEELYRNYGILADAKEDLSQHKDAYQVILDGVKGGPKEKRLAAQFIPKFFSSFPDLADAAINAQLDLCEDEDVSIRRQAIKELPRFAAGENLPRVADILTQLLQTDDAAEFNQVNSALISIFKIDARGTLGGLFSQILQGEDVVRERAIKFLSTKLKTMPDETLTKDVEDFIFVETKKVLEDVTGEEFVLLMRVLSGMKSLQTVSGRQQLVELVVEQAYLEQALNPADADSVDRLLQCTRQALPLFSKNVHSTRFVTYFCEHVLPNISMLTSPVAELDIQLEVLKLLAEMSPYCGDMDKLESNLMMLFEKLLEFMPLPPEEENGENAGNEEPKLQFSYVECLLYSFHQLGKKLPDFLIDKVNAEKLKDFKIRLQYFARGLQVYIRQLRVALQGKSGDALKTEENKIKVVALKITNNINILIKDLFHNPPSYKSTVTLSWKPVQKSEAATAAVVGQKRPSGEDVPTGTIGKKVSPLPRRDARQIYNPPSGKYSASIGNFSYEQRGGFRGGRGRGFGRGNRSRGRIY

>228

MAATVEELYRNYGILADAKEDLSKHKDAYQVILDGVKGGPKEKRLAAQFIPKFFSSFPEMADAAINAQLDLCEDEDVSIRRQAIKELPRFAAGENLPRVADILTQLLQTDDSAEFNQVNGALISIFKIDSRGTLGGLFSQILQGEDVVRERAIKFLSTKMKTMPDETMTKEVEEYIFIETKKVLEDVTGEEFVLLMRVLSGLKCLQTVSGRQQLVELVVEQAFLEQALNPTDTDGVDRLLQCTRQALPLFSKNVHSTRFVTYFCEHVLPNLSMLTCPVAQLDIQLEVLKLLAEMSPFCGDMDKLEANLMMLFEKLLEFMPLPPEEENGENAVSEEPKLQFSYVECLLFSFHQLGKKLPDFLIDKINAERLKDFKIRLQYFARGLQVYIRQLRVALQGKTGDALKTEENKIKVVALKITNNINVLIKDLFHNPPSYKSSVTLSWKPVQKTEAPVVLGQKRPSGEDMPTGKKVSPLPRRDARQIYNPPSGKYSATIGNFSYEQRGGFRGGRTRGFGGRGNRSRGRIY

>229

MSTDSIEKLYKNFGILADAKDKLGQHQKEYLEILTAVKGSPKEKRLASQFIARFFKHFPTLADQAIDAHLDLCEDEDMAIRKQAIKDLPALCKDNKEHTARIADILAQLLQAEDSSELAVVHNSIMSLMKSDPKGTLNGFFSQIINGDDGTRERCIKFLATKLKAIGHDVITKEPEDLLIAECKKVLQDVTADEFHNIMDILAWTRLGSTVTGQQELVDITIEQAELSVPFKHTNIEQWNRLVQCIKHALPFFSSQTDSSKFVSYICVQVLPHLSLMTSPDGRDIQLELLKLLAELAVFCGTIEKPDEKVQQLYNTLITYMPLPPVAEVTDVPKLQFSHVECLMYAFHKLCKQTPEFLIKDPEQLKEFRLRLQYFARGIQGYIKKLREAISGKTEEELKSEENQLKVVALKTTNNINTLIKDLFHSPPSFKSVIHLSWKTPSNDKKNEKSSAQKRHTPITFGNGNGSNKRMKEDRSSKREIYTPPSGKYSSNISNYGRGRFKNNRPGGRGGFRSRGRGPWRKNFY

>230

MAATVEELYRNYGILADAKEDLSQHKDAYQVILDGVKGGPKEKRLAAQFIPKFFSSFPDLADAAINAQLDLCEDEDVSIRRQAIKELPRFAAGENLPRVADILTQLLQTDDAAEFNQVNSSLISIFKIDARGTLGGLFSQILQGEDVVRERAIKFLSTKLKTMPEETLTKEVEDYILTETKKVLEDVTGEEFVLLMRVLSGMKSLQTVSGRQQLVELVVEQAYLEQALNPADADSVDRLLQCTRQALPLFSKNVHSTRFVTYFCEHVLPNLSMLTSPVAELDIQLEVLKLLAEMSPFCGDMDKLESNLMMLFEKLLEFMPLPPEEENGENAGNDEPKLQFSYVECLLFSFHQLGKKLPDFLIDKVNAEKLKDFKIRLQYFARGLQVYIRQLRVALQGKTGDALKTEENKIKVVALKITNNINVLIKDLFHNPPSYKSTVTLSWKPVQKTEAAAVVGQKRSSGEDVPTGTIGKKVSPLPRRDARQIYNPPSGKYSASIGNFSYEQRGGFRGGRGRGFGRGNRSRGRIY

>231

MSADNIEKLYQNYGILADAKDDISKHEKEYLEILAAVKGSDKEKRLASQFIAKFFSSFPNLAEQAIEAQFDLCEDDDVAIRKQAIKDLPSLCKDNKEHTQRIADILAQLLQSDDATEINVVTNALLAILKSDPKGSLVGLFSQIHQSTDSEVTNEVVRERCIKFLATKVKQLGREVINKEAEDLIITECKKILEDVVAEEFEHIMELLTWSRLGKTPAGKKELVQLIAALALSPDDWHPEDPENVDRIIQCTQHALPLFSTLVDSTQFVSFFCEHVLTSWKEIASPDGGSDSKLELLKIFAEITEYCGEIQNVQDKINAVYDLLMNYLPEAPVESEPNSENKEKTDTTTEDSKSLPSLQFSHVECALFALHSLCRKAPDAITADAARLKDLRLRLQYTARLTQGYIKKLKEVTQSKKADDANSEENKLKIAALKTTSNINTLIRDIFRTPPSFKSRVQLSFQSNRKIDKEEKLADQKSEDKNQSSAGQKRHRPITFDNGDSKGSPEKRSRSGDRNLKMYTPPSGKYSSRLNNSGRFSGPNSGRGRGGGYGRRDFRNNGAPYRRRSNY

>232

MSADNIEKLYQNYGILADAKDDISKHEKEYLEILAAVKGSDKEKRLASQFIAKFFNSFPTLADQAIEAQFDLCEDDDVAIRKQAIKDLPTLCKDHKEHTQRIADILAQLLQSEDTTEINVVSNSLLAILKIDPKGALTGMFSQIHQNTDSEVTNEIVRERCIKFIATKVKQLGREVINKEAEELIIAECKKLLEIQDVVAEELEHIMDLLTWSKLGKTPAGKKELVQILAALAFTPDDWHPEDPEYVDRVVQCSQHALPLFSAQVDSTQFVNFFCDHVLNGWDSVTTTEGTDSKLELLKLFAELTEHCGELENAQQKIDSVYQLLMKYLPEAPLESEENAGEKTEEKPEESKTTPSLQFSHVECALFGLHSLCRKAPDALGADATKLKALRLRLQYTARLTQGYIKKLKEVTQSQKGEDANSEENKLKIAALKTTSNINTLIRDLFRTPPSFKSKVQLSFQSKKTEKEEKLTPNSDETKQSPPAQKRHRPITFDNENEKESPEKRSRSGDRNLKMYTPPSGKYSSRLNNNGRFSGNNTGGRGGRGGYGRRDFRNNGAPFRRRNNY

>233

MDQAIEALFDLCEDEDVSVRKQAIKDLPSLCKENNEYTPKVTDILGQLLQTNDTTELSVVQSSIMTLCKNDIKGALKGMFNLIEISNGPVREKCLRFLVPKIKMLGRDIMTKEIEEYTIDQCKKILRNVTGDEFMQIMDLLAWSRLSQSLAGQKELMEIIKHHAELSKSFDPSNPEDVDRSIYCSLHCVPYLCSAIPSTPFVSYLCEQVLPKLDQIPETKDEAGGLDNQLEALKLLAELSPFYVVHEDTPQHITSIVDCLFTYLPTPPPIEESSEDTEPEFKYSRIEALLYTLHHIVKHDVELICKDVDRLKEFKKRLQYMARWSMAYTKRLREVLKSVEGDAAKAAENKVKLAALKTMANINTMVKEFFHNPPSFKSSITLSFKPLNEKSNALEENGGSHGNKRRAPISFGAGDKDGLSAAKSRPGVDSVKNRPIYTPPSGKFSTNLSYQQ

>234

MSVDVEQLYKSFGVLADAKDNAKEHPEAYKTILKGVEGTSAAKRLSAQFTSRFFKHFPDLSESAINALLDLCEDDDPSIRKAAIKELPNLCKSSKEHVSRLSDVLVQLLVSEESSEVSAVNSALNGLFTLDSKGTLTGVLSQILSGDDEVREKAITYLCTRLKSCSEADMSKETEEYVLEQLKKVLEDVTGGEFQKLMQALSGLHHLQTIQGRQQLVNIIADQLKLDQDFDPKDTDSLNRISQCISMALPLCSRNVHSSPFINYMCQKVLPQLSLIGCSEAVNGDSKPEVNQPDLKLEILKELSDLCAHCGATEIQDSIQPLFQSLVDFMPLPPTDESEDGSSAPKPKLHFSYVECLMHSFHQLGRHNSDFFTSEEAAAKLKDFRIRLQYFARGVQVYIKELKTSLAGKSPTELRTEKENQIKLVALKTCNNINTLIRDLFHNPPAYKASINVSWKKTTAAPKASPAKPQKRPSTGGNDNDDKKKERTLYRTPSGKYSSNISPGRGGYSNKRGGRKWGGGRGQYY

>235

MAAADADAAEVERLYELGERLSSAKDKSEHAADYEAIIAAVKGQSVKAKQLAAQLIPRFFRSFPALATRAMSAMFDLVEMEELAIRIQAIRGFPLLGKDTEFVSKIADILGQLLTSEENVERDAVHKALMSLIRQDVKNSLQPLFKHVESGSEIREKIICFLRDKVFPLKAELLKPQAEMERFITDLIKKSVQDVTGSEFELFMGFLRSLSIFGDSAPRESVQELIEIIQAQADLDSQFNVSDIDHIERWSSCMYMAIPIFMRGASSSKFLNYFVKQIVPAFEKIPEEKKLDLLKTIAASSPYAAAQDSRQLLPSVVQLLKKYMPGKKVEDINHNYVECLLYTFHHLAHKTPNTTNSLCGYKIVTGQPSDRLGEDFSEHYKDFTERLTGTEETVRAASKRLTQGMADFNKAISSAKTEEEKTKIKSDQQTATRTMRSYNNILAMTQPLHMKSPSFIGDKKIILSWMEQPKKPAATTAGVKRSQPATNGNGPASKKGRGGMQNQLVNRAFEGLSYGGRGSGRGRGRGGRGRGRGWGGYR

>236

MAAADADAAEVERLYELGERLSSAKDKSEHAADYEAIIAAVKGQSVKAKQLAAQLIPRFFRSFPALATRAMSAMFDLVEMEELATTPERSRSHAPCRPPALVLCPEGEEHPRQLPKSKWTSRRPRVASATQNASCEEEREVCTTPPWRGRRSALRRPCAAGAR

>237

MFLRCCIFLQIRIQAIRGFPLLGKDTEFVSKIADILGQLLTNEENVERDAVHKALMSLIRQDVKNVHWFLLFICTLYWPDSLQPLFKHVESGSEIREKIICFLRDKVFPLKAELLKPQAEMERFITDLIKKSVQDVTGSEFELFMGFLRSLSIFGDSAPRESFQELIEIIQAQADLDSQFNHSYPSFQVSDIDHIERWSSCMYMALPIFMRGASSSKFLNYFVKQIVPAFEKIPEEKKLDLLKTIAASSPYAVAQDSRQLLPSVVQLLKKYMPGKKAEDINHNYVECLLYTFHHLAHKTPNTTNSLCGYKIVTGQPSDRLGEDFSEHYKDFTERLTGTEETVRAASKRLTQGMADFNKAISSAKTEEEKTKIKSDQQTATRTMRSYNNILAMTHPLHMKSPSFIGDKKITLSWMEQPKKPAATTAGLKRSQPATNGNGPASKKGRGGMQNQLVNRAFEGLSHGGRGSGRGRGRGGRGRGRGWGGYR

>238

MWEFDNNVIMSMDSIEKLYKNFGILADAKDKLAEHEKEYLEILTAVKGSPKEKRLASQFIARFFKYFPKLADQAIDAHLDLCEDEDMAIRKQAIKDLPSLCKDNKEHTPRIADILAQLLQAQDSSELAVVHNSVMSLMKTDPRGTISGFFSQIINGDDGTRERCIKFLATKLKAIGHDVITREPEDLLILECKKVLQDVTADEFHSIMEILAWTRLGSTVSGQQELVDITVEQAELSEPFKPTNVEQWNRLVQCIKHALPFFSSQIDSSRFVSYICMQVLPHLSLITSPDGRDVQLELLKLLAELAVFCGTIDKPEEKVQELYNTLTTYMPLPPATEITEVPKLQFSHVECLMYAFHKLCKQTPEFLMKDPEQLKEFRLRLQYFARGIQGYIKKLREAISGKTEEELKSEENQLKVVALKTTNNINTLIKDLFHSPPSFKSVIHLSWKTMVNDKKSEKHSTAQKRHTPITFGNDSNSNKRSKEDKSKRELYTPPSGKYSSNISSNYGRGRFRGNRSGGRGGYRPRGRGTWRKNFY

>239

MPTVEELYRNYGILADAKDTAPEHKDAYQVILDGVKGGAKEKRLAAQFIPKFFKHFPELADSAINAQLDLCEDEDVSIRRQAIKELPQFAMGDNLPRVADILTQLLQSDDSAEFNLVNNALLSIFKMDAKGTLGGLFSQILQGEDIVRERAIKFLSTKLKILPEEVLTKEVEELILTESKKVLEDVTGEEFVLFMKILSGLKSLQTVSGRQQLVELVAEQADLEQTFNPSDPDCVDRLLQCTRQAVPLFSKNVHSTRFVTYFCEHVLPNLSSLTTPVEGLDIQLEVLKLLAEMSSFCGDMEKLESNLKKLFDKLLEYMPLPPEEAENGENAGNEEPKLQFSYVECLLYSFHQLGRKLTDFLTAKLNTDKVKDFKIRLQYFARGLQVYIRQLRLALQGKTGEALKTEENKIKVVALKITNNINVLIKDLFHIPPSYKSTVTLSWKPVQKSEVGQKRASEDTSPDLPAKKSQAGPKRDARQIYNPPSGKYSSNLGNFSYEQRGGFRGGRGRGWGGRGNRNRGRIY

>240

MPTVEELYRNYGILADAKDTAPEHKDAYQVILDGVKGGAKEKRLAAQFIPKFFKHFPELADSAINAQLDLCEDEDVSIRRQAIKELPQFAMGDNLPRVADILTQLLQSDDSAEFNLVNNALLSIFKMDAKGTLGGLFSQILQGEDIVRERAIKFLSTKLKILPEEVLTKEVEELILTESKKVLEDVTGEEFVLFMKILSGLKSLQTVSGRQQLVELVAEQADLEQTFNPSDPDCVDRLLQCTRQAVPLFSKNVHSTRFVTYFCEHVLPNLSSLTTPVEGLDIQLEVLKLLAEMSSFCGDMEKLESNLKKLFDKLLEYMPLPPEEAENGENAGNEEPKLQFSYVECLLYSFHQLGRKLTDFLTAKLNTDKVKDFKIRLQYFARGLQVYIRQLRLALQGKTGEALKTEENKIKVVALKITNNINVLIKDLFHIPPSYKSTVTLSWKPVQKSEVGQKRASEDTSSDLPAKKSQAGPKRDARQIYNPPSGKYSSNLGNFSYEQRGGFRGGRGRGWGGRGNRNRGRIY

>241

MSADNIEKLYQNYGILADAKDDISKHEKEYLEILAAVKGSDKEKRLASQFIAKFFNSFPNLTDQAIEAQFDLCEDDDVAIRKQAIKDLPLMCKNNKEHTTRIADILAQLLQSEDATEINVVTNSLVSILKNDPKGTLTGLFSQIHQSTDSEVPNEVVRERCIKFLATKVKQLGRDVIDKDAEDLIISEGKKILQDSVAEEFEHIMDLLTWSRLGKTPAGKKEMTQLIAALAFTPGDWHPEDPEYVDRLIQCTQHAIPLFTNQVDSTQFVNIFCDHVLLRWNDIATTGGGTDVKLDILKTFAELTEHCGEIENAQDKINIVYDVLMNYLPEAPVENEEENAVKGEEGENKPEDTKSTTAPSLQFSHVECALFAIHSLCRKAPDALGADTARLKTLRLRLQYTARLTQGYIKKLKEVTQSKKGEDANTEENKLKIAALKTTSNINTLIRDIFRTPPSFKSKVHLSFQTKKVEKEITSSDTEDKDKSSSGSKRHRPITFDNGEQKEAPEKRARSGDRNLKMYTPPSGKYSSRLNTTGRFSGPNSGRGRGKREFWNRGTPFRKRY

>242

MSGDNIEKLYQNYGILADAKDDISQHEKEYLEILAAVKGSDKEKRLASQFIAKFFNSFPNLAEQAIEAQFDLCEDDDVAIRKQAIKDLPNLCKDHKEHTQRIADILAQLLQSDDTTEINVVTNSLITILKGDPKGALAGIFSQIHQNVDSEVANEIVRERCIKFLATKVKQLGREVINKEAEDLIIAECRKILEDVVAEEFEHIMELLTWSRLGKTPLGKKELVQIVAALAFSPDDWHPEDPEYVDRIIQCTQHALPLFSTQVDSTQFVNFFCDHVLQKWNEITTPEGNDSKLELLKIFAEVTEHCGDLEDVQKKIDTVYDVVMTYLPEAPIEGEDKGEKEANGDKTEEKSTPSLQFSHVECALFALHSLCRKAPEALGADGARLKALRLRLQYTARLTQGYIKKLKEVTQGKKEDSEENKLKIAALKTTSNINTLIRDIFRTPPSFKSKVQLSFQTQKTEKEQKPTEKTEEKDKQSPQKRHRPITFDNGDEKSSPDKRSRSGDRNIKMYTPPSGKYSSRLNNSGRFSGPSFRGRGGYGRREFRGNGAPFRRRSNY

>243

MWKFDNNFIMSMDSIEKLYKNFGILADAKDKLAEHEKEYLEILTAVKGSPKEKRLASQFIARFFKYFPKLADQAIDAHLDLCEDEDMAIRKQAIKDLPALCKDNKEHTARIADILAQLLQAEDPSELAVVHNSVMSLMKTDPRGTISGFFSQIINGDDGTRERCIKFLATKLKAIGHDIITKEPEDLLISECKKVLQDVTADEFHSIMEVLAWTRLGTTISGQQELVDITVEQAELSEPFKHTNVEQWNRLVQCIKHALPFFSSQIDSSRFVSYICIQVLPHLSLITSPDGRDVQLELLKLLAELAVFCGTIDKPEEKVQQLYNTLITYMPLPPVTEITEVPQLQFSHVECLMYAFHKLCKQTPEFLMKDPEQLKEFRLRLQYFARGIQGYIKKLREAISGKSEEELKSEENQLKVVALKTTNNINTLIKDLFHSPPSFKSVIHLSWKTTANDKKNEKHSSAQKRHTPITFGNDSNSNKRSKEDKSKRELYTPPSGKYSSNISSNYGRGRFRGNRSGGRGGYRPRGRGTWRKNFY

>244

MSTDNIEKLYKHFGVLADAKDKVSEHEDDYLEILKAVKGSDKEKRLASQFIARFFKYFPTLTDQAIEAQLDLCEDEDLAIRKQAIKDLPRLCQDLKDYTQRIADILAQLLQAQDSSELSVVHNSLMSLLKLDPKGTLTGIFSQIFNGDDASREQCLKFLTVKVKNQGTDIFDKESEDFLISEAKKVLQDVTAEEFHLLMELLNATRLGKTVSGQKDLVDIAAEQAELDQPFNPSDPENESFDRLVNCVKHALPYFSKQLESTRFVTYFCDEVLPQLHLVGGADEGSGADQQLELLNLLAELSTFCGTLEKAETRVDKVFTRLLDYMPLPPDGETTDEAEPRLDFSHVECLIYSYHRLGRQCPDALAKDAEKLKDFRLRLHYFARSVQGYMKKLQEALRGKTGEELKSEENKLKVVALKTTSNINTLIKDLFHTPPSFKSVITLSWKPANASYTSDRALEKESVRAGNKRHTPITFGDASPKHPRTVGSNPREIYTPPSGKYSNKVSSYMPQRGGVRGNRSRGRGFGGRTRGPWRRNY

>245

MAVTVEDLYRNYGVLADAKPENLSQHKDAYQVILDGVKGGPKEKRLAAQFIPKFFSSFPELADAAINAQLDLCEDEDVSIRRQAIKELPRFATGENILRVADILTQLLQTDDTAEFNQVNASLLSIFKIDAKGTLGGLFSQILQGEDIVRERAIKFLSTKLKTLPEDVMTKEVEEYVFAETKKVLEDVTGEEFVLLMRVVSGLHVLQTVNGRQQLVELVVEQAFLEQALNPADPDTVDRLLQCTRQALPLFSKNVHSTRFVTYFCEHVLPNLSSLTSPVAELDIQLEVLKLLAEMSPFCGDMEKLEANLHMLFTKLLEYMPLPPEEVENGENSASEEPKLQFSYVECLLFSFHQLGKKLPDFLLDKVDAERLKDFKIRLQYFARGLQVYIRQLRVALQGKTGDALKTEENKIKVVALKITNNINVLIKDLFHNPPSYKSTVTLSWKPVQKTEAVAPKRPSGEEMGSGTSPQKQISSLPRRDSRQIYNPPSGKYSASIGNFNYERGGFRGGRGRGFGARGSRSRGRLY

>246

MAVTIEDLYRNYGILADAKDNLSQHKDAYQVILDGVKGGPKEKRLAAQFIPKFFSSFPELADAAINAQLDLCEDEDVSIRRQAIKELPRFATGENILRVADILTQLLQTDDTAEFNQVNAALLSIFKTDAKGTLGGLFSQILQGEDIVRERAIKFLSTKLKTLPEDVMTKEVEDYVFAETKKVLEDVTGEEFVLLMRVVSGLRVLQTVSGRQQLVELVVEQAFLEQALNPADPDTVDRLLQCTRQALPLFSKNVHSTRFVTYFCDHVLPNLSTLTSPVAELDIQLEVLKLLAEMSPFCGDMEKLEANLNMLFTKLLEFMPLPPEEVENGENSASEEPKLQFSYVECLLFSFHQLGKKLPDFLLDKVDAERLKDFKIRLQYFARGLQVYIRQLRVALQGKTGDALKTEENKIKVVALKITNNINVLIKDLFHNPPSYKSTVTLSWKPVQKTEAVAPKRPSGEEMGSGGTTKKQNSPLPRRDARQIYNPPSGKYSSSIGNFTYERGGFRGGRGRGFGARGNRSRGRIY

>247

MAVTIEDLYRSYGILADAKDNLSQHKDAYQVIVDGVKGGPKEKRLAAQFIPKFFSSFPELADAAINAQLDLCEDEDVSIRRQAIKELPRFATGDNIPRVADILTQLLQTDDTAEFNQVNAALISIFKMDARGTLGGLFSQILQGEDIVRERAIKFLSTKLKTLPEDVMTKEVEEYVFAETKKVMEDVTGEEFVLLMRVVSGLRVLQTVNGRQQLVELVVEQAFLEQALNPADPDTVDRLLQCTRQALPLFSKNVHSTRFVTYFCEHVLPNLSALTSPVAELDIQLEVLKLLAEMSPFCGDMEKLEANLNMLFTKLLEFMPLPPEEVENGENSASEEPKLQFSYVECLLYSFHQLGKKLPDFLLDKVDAERLKDFKIRLQYFARGLQVYIRQLRVALQGKTGDALKTEENKIKVVALKITNNINVLIKDLFHNPPSYKSTVTLSWKPVQKAEAVALKRPSTEEMGSGGSTKKQISPQSRRDARQIYNPPSGKYSASIGNFNYEQRGGFRGGRGRGFGGRGNRSRGRIY

>248

MVRARTLPSLSFRVRLLCVRRVRLALLTSVVFFASTLRQRTRPGVNFTMSTDSIEKLYKNFGILADAKDKLVQHEKEYLEILTAVKGSPKEKRLASQFIARFFKHFPKLADQAIDAHLDLCEDEDMAIRKQAIKDLPALCKDNKEHTARIADILAQLLQAEDSSELAVVQNSIMSLMKSDPKGTLSGFFSQIINGDDGTRERCIKFLATKLKAIGHDIITKEPEDLLIAECKKVLQDVTADEFHSIMEILAWTRLGSTVAGQQELVDITIEQAELSVPFKHTNVEQWNRLVQCVKHALPFFSSQIDSSKFVSYICVQVLPHLSLMTSPDGRDIQLELLKLLAELTVFCGTIEKPEDKVQQLYNTLITYMPLPPATEITDVPKLQFSHVECLMYAFHKLCKQTPEFLIKDPEQLKEFRLRLQYFARGIQGYIKKLREAIGGKTEEELKSEENQLKVVALKTTNNINTLIKDLFHSPPSFKSIIHLSWKTPLNEKKSEKNSAQKRHTPITFGNDNSPNKRSKEDKNNKREIYTPPSGKYSSNISNYGRGRFKGNRPRGRGGFRTRGRGPWRKNFY

>249

MPTVEELYRNYGILADATETAGQHKDAYQVILDGVKGGAKEKRLAAQFIPKFFKHFPELADSAINAQLDLCEDEDVSIRRQAIKELPQFATGDNLPRVADILTQLLQSDDSAEFNLVNNALLSIFKMDAKGTLGGLFSQILQGEDIVRERAIKFLSTKLKTLPEEVMTKEVEEFILTESKKVLEDVTGEEFVLFMKILSGLKSLQTVSGRQQLVELVAEQADLEQTFNPSDPDCVDRLLQCTRQAVPLFSKNVHSTKFVTYFCEHVLPNLSSLTTPVEGLDIQLEVLKLLAEMSSFCGDMEKLESNLKKLFDKLLEYMPLPPEEAENGENAGNEEPKLQFSYVECLLYSFHQLGRKLPDFLTAKLNAEKLKDFKIRLQYFARGLQVYIRQLRLALQGKTGEALKTEENKIKVVALKITNNINVLIKDLFHIPPSYKSTVTLSWKPVQKADAGQKRTSEDTTSSSPPKKAPAGPKRDARQIYNPPSGKYSSNLGSFSYEQRGGFRGGRGRGWGGRGNRSRGRIY

>250

MVTNTAEVTPQLVTPGPDSTHKVLLHVILSEDMNWDLSSIQRELGNGKVEGRYVFLLHKDAYQAILDGVKGGAKEKRLAAQFIPKFFKHFPELADSAINAQLDLCEDEDVSIRRQAIKELPQFATGDNLPRVADILTQLLQSDDSAEFNLVNNALLSIFKMDAKGTLGGLFSQILQGEDIVRERAIKFLSTKLKTLPEEVMTKEVEEFILTESKKVLEDVTGEEFVLFMKILSGLKSLQTVSGRQQLVELVAEQADLEQTFNPSDPDCVDRLLQCTRQAVPLFSKNVHSTKFVTYFCEHVLPNLSSLTTPVEGLDIQLEVLKLLAEMSSFCGDMEKLESNLKKLFDKLLEYMPLPPEEAENGENAGNEEPKLQFSYVECLLYSFHQLGRKLPDFLTAKLNAEKLKDFKIRLQYFARGLQVYIRQLRLALQGKTGEALKTEENKIKVVALKITNNINVLIKDLFHIPPSYKSTVTLSWKPVQKADAGQKRTTEDTTSSSPPKKSPAGPKRDARQIYNPPSGKYSSNLGSFSYEQRGGFRGGRGRGWGGRGNRSRGRIY

>251

MTCVGFGAPRTCAGLIADYVMGDMTQCQPMKTFPWLVVLNVETLSLCGGADSLSSITALSLLPLTLVTGLCHSQAEGPWGGPLDGPSPPSRRARPLAPAAALDADNVRNFFKRLYKALFDLKEIGFDTLQKQTQTNLFSFPFLKIVFLLYSFFQHKDAYQVILDGVKGGTKEKRLAAQFIPKFFKHFPELADSAINAQLDLCEDEDVSIRRQAIKELPQFATGENLPRVADILTQLLQTDDSAEFNLVNNALLSIFKMDAKGTLGGLFSQILQGEDIVRERAIKFLSTKLKTLPDEVLTKEVEELILTESKKVLEDVTGEEFVLFMKILSGLKSLQTVSGRQQLVELVAEQADLEQTFNPSDPDCVDRLLQCTRQAVPLFSKNVHSTRFVTYFCEQVLPNLSSLTTPVEGLDIQLEVLKLLAEMSSFCGDMEKLETNLRKLFDKLLEYMPLPPEEAENGENAGNEEPKLQFSYVECLLYSFHQLGRKLPDFLTAKLNAEKLKDFKIRLQYFARGLQVYIRQLRLALQGKTGEALKTEENKIKVVALKITNNINVLIKDLFHIPPSYKSTVTLSWKPVQKVEIGQKRANEDTTSGSPPKKSPAGPKRDARQIYNPPSGKYSSNLGNFNYEQRGAFRGSRGGRGWGARGNRSRGRLY

>252

MHKICLAIFLIKIESTKIVTYICDQVLPQWDKIAQVNQGELLQLVILRQLAELSTYCGKLDNATHYISQIYDKLRQYMPPPPENPDLITMPFLDFSVVECLLYAFHRLARQCPEFLTQDQALLKDFRARLMYFSRGVQGCSKALANLEKKKEGLSPEETQKQKISPKLLNNINMLIKDLFYQPPMYKCNVTLSFKAEETIAKTPEKAPVGNKRHVPITFESNGASSANKNTKKSSDGMKLYTPPSGKFSNNFQSYGGRGRPRGSRGSRGRGSGRGWR

>253

MDKIDKLYQNYDILADSKNKPSEHEAEYLEIIESVKGKTNEKMLACQFIPRFLKEFPDLASAALDAQLDLVEDEDVSIRKHAVKHLPAFCKESKACVAKISDILAQMLQTEDSAELATVQNTLMTIMKIDPKATLDGIFGQIGSTDEDVIRKRAIQFLCTKFKFIPPDIATKDVEDFVLEKCKKVFPELGGEDFLNLMPL

>254

VFPELGGEDFLNLMPLLANLKIAKSVPVQQALVNLIADQAEMDGEIQVSLDYIAGFLQCVRLALPYFSPFVQSTQFVSYI

>255

MAVTIEDLYRSYGILADAKDNLSQHKDAYQVIVDGVKGGPKEKRLAAQFIPKFFSSFPELADAAINAQLDLCEDEDVSIRRQAIKELPRFATGDNIPRVADILTQLLQTDDTAEFNQVNAALISIFKMDARGTLGGLFSQILQGEDIVRERAIKFLSTKLKTLPEDVMTKEVEEYVFAETKKVMEDVTGEEFVLLMRVVSGLRVLQTVNGRQQLVELVVEQAFLEQALNPADPDTVDRLLQCTRQALPLFSKNVHSTRFVTYFCEHVLPNLSTLTSPVAELDIQLEVLKLLAEMSPFCGDMEKLEANLNMLFTKLLEFMPLPPEEVENGENSASEEPKLQFSYVECLLYSFHQLGKKLPDFLLDKVDAERLKDFKIRLQYFARGLQVYIRQLRVALQGKTGDALKTEENKIKVVALKITNNINVLIKDLFHNPPSYKSTVTLSWKPVQKAEAVAPKRPSTEEMGSGGSTKKQISPQSRRDARQIYNPPSGKYSASIGNFNYEQRGGFRGGRGRGFGGRGNRSRGRIY

>256

MSTDSIEKLYKNFGILADAKDKLAEHEKEYMEILTAVKGSPKEKRLASQFIARFFKHFPKLADRAIEAHLDLCEDEDIAIRKQAIKDLPSLCKDNKEHTARIADILAQLLLAQDPSELAVVQNSIMSLIKSDPKGAISGFFSQILNGDDGARERCIKFIATKLKAVGHDIITKEPEDLLIAECKKVLQDVTADEFHSIIEVLAWTRLGSTVSGQQELIDIIVDQAELSIPFKHTNEEQWSRLLQCIKHALPFFSSQVDSSRFVCYVCVQVLPHLSLITGPEGRDPQLELLKLLAELTAYCGTIEKPEEKVQQIYNALITYMPLPPESDLPELPKLQFSHVECLMYTFHKLGKQIPEFLTRDPDQLKEFRLRLQYFARGIQGYIKKLREAISGKSEEQLKSEENQMKVVALRTTSNINTLIKDLFHSPPSYKSIIHLSWKTSIADKKTEKSSPGQKRHTPITFGNDNSPSKRIKDDKGNKREIYTPPSGKYSSNISSNYGNRGRFRGNRSGGRGGYRSRARGWRRNTY

>257

MTEVDVEQIYKAYEEINNASDNTPEIKRSYEILIAGAHGSSNCKRLAAQFIPRFFGKFPEYYETALDALFDLCEDTDINVRLTVIKYMPNVVKEFDKFAVRIADALVQLLENETVQEIAAVKKALEQVLRLDPETIPAIFNQSLKGSPEVRQRTINFLSNDLNRIKMELSQRNSDWESKFSDEVKKALHDANATDYEMFIKMLLSLKMYEKKENLKALSESMVDAIAKENEQLDPSNESSFQKFMLCGKTLIQFFEKGISTTPILAFLVKKMLPQNIYCNLQAKQQKSVLRYLVEFIIRSPNETTLKEAAPLIKDIFVKEVPEPPSDNNEEDPKLDLNKVENIVYAIYTIASKVPAITEGQEMNSRLRHLYTFAMKCQTRVKLGLKDLQKTQNKDQQTIEKIKKAENVQTVTQNIFTMTKELMKPAVARHFTKVVISWRTSPQTTTSPPTLTSSTTGVKRPNTEGSTSNPNKKQKSAQTNTTSQASQIRQKITAPKVTKSVRTGQANKRTGEANKRTGGSGGNRNNGVRLAKIARNKNNEKNLYVPPPRRAK

>258

MSTDSIEKLYKNFGILADAKDKLVQHEKEYLEILTAVKGSPKEKRLASQFIARFFKHFPKLADQAIDAQLDLCEDEDMAIRKQAIKDLPALCKDNKEHTARIADILAQLLQAEDSSELSVVHNSIMSLMKSDPKGTLSGFFSQIINGDDGTRERCIKFLATKLKAIGHDVITKEPEDLLIGECKKVLQDVTADEFHSIMEILAWTRLGSTVTGQQELIDITIEQAELSVPFKHTNVEQWNRLVQCIKHALPFFSSQIDSSKFVSYICVQVLPHLSLMTSPDGRDIQLELLKLLAELTVFCGSIDKPEDKVQQLYNTLITYMPLPPATEITDVPKLQFSHVECLMYAFHKLCKQTPEFLIKDPEQLKEFRLRLQYFARGIQGYIKKLREAISGKTEEELKSEENQLKVVALKTTNNINTLIKDLFHSPPSFKSIIHLSWKTPCNDKKNEKNSAQKRHTPITFGNDNGSNKRSKEDKSNKREIYTPPSGKYSSNISNYGRGRFKGNRFGGRGGFRSRARGSWRKNFY

>259

MEAEDNIQKMYKYFGILADAKENIAEKEPEYLEILSAVKGSTKEKRLASQFITRFFKYFPNLAAQALEAQLDLCEDEDICIRKQAIKDLPVLCKESKEYLTKIADILAQLLQADDPQELLTAQNSLLSLFKIDAKGALTGIFSQMQSNEEVVRERSMKFILNKVMALGKEIIKKDVEDLIIAECKKVMQNITCEEFETLMTILSSTHLINTPDGQKELVELLANTAELDQFFNPKDLDQVNRFITCLDFAIPFFSAHVESTKFIVYICELLNRYTLIKDNDKQFIILKLLAESVPYCGKLMNPEAVVGQVYQALLDLVTVPENDSNKKVEDMDLHRVEALLYTFHKLGKQCPDFLSKDPERQKDFKKKLLYVGTCTQTFVKIVRQDLKEKGEEDVKNDPIVEKKLEGLKLACNINTLIKELFNIPPRFKATINLSWLGGATKSKLAQIIQEGKKHETITVEGKSKRIDGSNSSQQIYQPPKDKFSAKFSNNTTNNNRRGGGTGWNTKRSFDTSNGNRRSWRPY

>260

MSTDSIEKLYKNFGILADAKDKLAQHEKEYLEILTAVKGSPKEKRLASQFIARFFKHFPKLADQAIDAHLDLCEDEDLAIRKQAIKDLPALCKDNKEHTARIADILAQLLQAQDPTELAVVHNSIMSLIKSDPKGTISGFFSQIINGDDGTRERCIKFLATKLKAIGHDVITKEPEDLLISECKKVLQDVTADEFHSIMEILAWTRLGSTVTGQQELVYITIDQAELGIPFKHTNIEQWNRLVQCIKHALPFFSSQIDSSKFVSYICVQVLPHLSLMTSPDGRDIQLELLKLLAELTVFCGMIDKPEDKVQQLYNTLITYMPLPPAAEITDVPKLQFSHVECLMYAFHKLCKQTPEFLIKDAEQLKEFRLRLQYFARGIQGYIKKLREAIGGKTEEELKSDENQLKVVALKTTNNINTLIKDLFHSPPSFKSVIHLSWKTHCNDKKNEKNSTQKRHTPITFGNDSSTNKRIKEDKNNKREIYTPPSGKYSSNISSNYGRVRFRGNRSGGRGGYRSRGRGTWRKNFY

>261

MMSTDSIEKLYKNFGILADAKDKLVQHEKEYLEILTAVKGSPKEKRLASQFIARFFKHFPKLADQAIDAHLDLCEDEDMAIRKQAIKDLPALCKDNKEHTARIADILAQLLQAEDSSELAVVHNSIMSLMKSDPKGTLSGFFSQIINGDDGTRERCIKFLATKLKAIGHDIITKEPEDLLISECKKVLQDVTADEFHSIMEILAWTRLGSTVSGQQELVDITIEQAELSIPFKHTNIEQWNRLVQCVKHALPFFSSQIDSSKFVSYICMQVLPHLSLMTSPDGRDIQLELLKLLAELTVFSGTIEKPEDKVQQLYNTLITYMPLPPATEITDVPKLQFSHVECLMYAFHKLCKQTPEFLIKDQEQLKEFRLRLQYFARGIQGYIKKLREAISGKTEEELKSEENQLKVVALKTTNNINTLIKDLFHSPPSFKSIIHLSWKTPCNDKKSEKNSAQKRHTPITFGNDNSPNKRSKEDKNNKREIYTPPSGKYSSNISNYGRGRFKGNRPRGRGGFRSGRGRGSWRKNFY

>262

MEEEHLPEAEPKQVEDDADRRHPLQPQYSLLYLKHKDAYQVILDGVKGGAKEKRLAAQFIPKFFKHFPELADSAINAQLDLCEDEDVSIRRQAIKELPQFATGDNLPRVADILTQLLQSDDSAEFNLVNNALLSIFKMDAKGTLGGLFSQILQGEDIVRERAIKFLSTKLRTLPEEVLTKEVEELILTESKKVLEDVTGEEFVLFMKILSGLKSLQTVSGRQQLVELVAEQADLEQTFNPSDPDCVDRLLQCTRQAVPLFSKNVHSTRFVTYFCEHVLPNLSSLTTPVEGLDIQLEVLKLLAEMSSFCGDMEKLESNLKKLFDKLLEYMPLPPEEAENGENAGNEEPKLQFSYVECLLYSFHQLGRKLPDFLTSKLNAEKLKDFKIRLQYFARGLQVYIRQLRLALQGKTGEALKTEENKIKVVALKITNNINVLIKDLFHIPPSYKSTVTLSWKPVQKADAGQKRTSEDTTSSSPPKKTPAGPKRDARQIYNPPSGKYSSNLGSFSYEQRGGFRGGRGRGWGGRGNRSRGRIY

>263

MMPTVEELYRNYGILADAKETAAEHKDAYQVILDGVKGGAKEKRLAAQFIPKFFKHFPELADSAINAQLDLCEDEDVSIRRQAIKELPQFAMGDNLPRVADILTQLLQSDDSAEFNLVNNALLSIFKMDAKGTLGGLFSQILQGEDIVRERAIKFLSTKLKTLPEEVLTKEVEELILTESKKVLEDVTGEEFVLFMKILSGLKSLQTVSGRQQLVELVAEQADLEQTFNPSDPDCVDRLLQCTRQAVPLFSKNVHSTRFVTYFCEHVLPNLSSLTTPVEGLDIQLEVLKLLAEMSSFCGDMEKLESNLKKLFDKLLEYMPLPPEEAENGENAGNEEPKLQFSYVECLLYSFHQLGRKLPDFLTAKLNTDKVKDFKIRLQYFARGLQVYIRQLRLALQGKTGEALKTEENKIKVVALKITNNINVLIKDLFHIPPSYKSTVTLSWKPVQKSEVGQKRASEDTSSDLPAKKTQAGPKRDARQIYNPPSGKYSSNLGNFSYEHRGGFRGGRGRGWGGRGNRSRGRIY

>264

MTAIDKMYDNYNILVDAGDKITEHEDKYLEILNSVKGSPNEKRLASQFITKFFKHFPAHYNSAIDALLDLCEDDDINIRKQAIKDLPSICRDCKEYVPRITDILAQLLLAQDATELQIVNNSLITVCKLDPKGFLGGIFLQIEAGEDITRERVIKFLAAKIKTLPEDTWTKEDEEFILAEGRKALQDCTKDEFVCLLNLLSSLKISKTITAQQVLVDMITEHAELDAPLNPAETEQIEKFTMCVRAAIPHFSPFVPSNAFVQFICSQIIPCMTEIVSLAIENPNIEVEILQQLAELSAFIQPTNTNLPINLDECQEVVFSKLLEYMPSPSLSTDVEEKSEEPSLQLFPLESLMFTLYTLSVINPEFNSKVNEDRMKDYKLRLQYLARLIQSHIKKAKEVPSADKKVENEGDDALSKAIALKTATNINTLVKELFHPKPASKSSVTLSWKPTKTKSAAIKRTNSTADTTVAPTATKLEDATTNASNPKRKPITAPEDSESSDSKKRQRQLYAPPSGKFSYNFRGGYSNNRGRGRGTGGFRGANRGRNNRYSKRYSY

>265

MSTDSIEKLYKNFGILADAKDKLAEHEKEYLEILTAVKGSPKEKRLASQFIARFFKYFPKLADRAIDAHLDLCEDEDMAIRKQAIKDLPAICKDSKEHTARIADILAQLLQAQDTSELAVVQNSIMSLMKSDPKGAISGFFSQILNGDDGTRERCIKFLATKLKAIGHDVITKEPEDLLIAECKKVLQDVTADEFHSIMEVLAWTRLGTTVSGQQELIDITVEQAELSVPFKHTNLEQWNRLVQCIGHALPFFSSQIDSSRFVSYICVQVLPHLSLITAPDGRDAQLEILKLLAELAVFCGNIEKPEEKVQQLYNTLITYMPLPPDTEVSEVPKLQFSHVECLMYAFHKLCKQTPEFLTKDAEGLKEFRLRLQYFARGIQGYIKKLREAIGGKSEDELKSEENRLKVVALKTTNNINILIKDLFHSPPSFKSVIHLSWITPSSDNKKIEKTSPGQKRHTPITFGNDSGSSKRNKEDGKGSKREIYTPPSGKYSSNISSNYGSRGRFRGNRSGSRGGYRPRGRGGWRKSAY

>266

MAVTIEDLYRNYGILADAKDDLSKHKDAYQVILDGVKGGPKEKRLAAQFIPKFFSSFPELADAAINAQLDLCEDEDVSIRRQAIKELPRFATGDNIPRVADILTQLLQTDDSAEFNQVNGALLSIFKMDAKGTLGGLFSQILQGEDIVRERAIKFLSAKLKTLPEDVMTKEVEEYVFGETKKVLEDVTGEEFVLLMRLVSGLRVSQTVHGRQQLVECVVDQAFLDQALNPADTDTVDRLLQCTRQALPLFSKNVHSTRFVTYFCEHVLPNLSSLTSPVAELDIQLELLKLLAEMSPYCGDMDKLEANVNMLFTTLLDFMPLPPEEAENGENSSSEEPKLQFSYVECLLYSFHQLGKKLPDFLVDKVDAERLKDFKIRLQYFARGLQVYIRQLRVALQGKTGDALKTEENKIKVVALKITNNINVLIKDLFHNPPSFKSTVTLSWKPVQKTEAVAPKRPSGEELPSGVSTKKQISPLPRRDARQIYNPPSGKYSASIGNFNYERGGFRGGRGRGFGARGNRSRGRIY

>267

MASESANDGQDIEKLYVYGERLSEAKDKSQNEEDYKSIIQAATSSNVKARQLAAQLIPRYFKFFPALSGLAVDAQCDLCEAEELGIRVQAIRGLPLFCKDTPEHIPKIIDILAQLLLAEENVERDAVHKALLSLLRQDVKGSLTALFKHIESVDEPITDDNLRERTLVFIRDKVFPLKSELLKPPEQMERHITDLVKKSLQDVTGAEFKMFMDFLKSLSIFGEKAPTERVQELVEIIEGQADLDAQFSVSDGDHVDRLISCLYMALPFFVRGASSSKFLNYLNKHILPVFDKLPEERKVDLLKNLAESSPYAAPQDSRQILPSVVQLLKKYMPLRKTGEEMNFTYVECLLYTFHHLANKAPNATNSLCGYKIVTGQPSDRLGEDFTEFNKDFTERLRCVEDLTKATMKKLTQGMNSQSNTKGTTISEEEKAKIKTQKQNATTGLRTCNNILAMIQVLN

>268

MASESANDGQDIEKLYVYGERLSEAKDKSQNEEDYKSIIQAATSSNVKARQLAAQLIPRYFKFFPALSGLAVDAQCDLCEAEELGIRVQAIRGLPLFCKDTPEHIPKIIDILAQLLLAEENVERDAVHKALLSLLRQDVKGSLTALFKHIESVDEPITDDNLRERTLVFIRDKVFPLKSELLKPPEQMERHITDLVKKSLQDVTGAEFKMFMDFLKSLSIFGEKAPTERVQELVEIIEGQADLDAQFSVSDGDHVDRLISCLYMALPFFVRGASSSKFLNYLNKHILPVFDKLPEERKVDLLKNLAESSPYAAPQDSRQILPSVVQLLKKYMPLRKTGEEMNFTYVECLLYTFHHLANKAPNATNSLCGYKIVTGQPSDRLGEDFTEFNKDFTERLRCVEDLTKATMKKLTQGMNSQSNTKGTTISEEEKAKIKTQKQNATTGLRTCNNILAMIQPLHSKSPSFIGDKRINLSWNVAVKPVAPANTVGGKRPANAANGSNNQANKRGRGGSNQPVERAFESQSYGGRGGSRGGGQGRGRGGRGRGRGYYR

>269

MAIRKQAIKDLPALCKDNKEHTARIADILAQLLQAEDTSELAVVHNSIMSLMKSDPKGTLSGFFSQIINGDDGTRERCIKFLATKLKALGRDIITKEPEDLLIAECKKVLQDVTADEFHSIMEILAWTRLGSTVTGQQELIDITIEQAELSVPFKHTNVEQWNRLVQCVKHALPFFSSQIDSSKFVSYICVQVLPHLSLMTSPDGRDIQLELLKLLAELTVFCGTIEKPEDKVQQLYNTLITYMPLPPATEITDVPKLQFSHVECLMYAFHKLCKQTPEFLIKDPEQLKEFRLRLQYFARGIQGYIKKLREAISGKTEEELKSEENQLKVVALKTTNNINTLIKDLFHSPPSFKSIIHLSWKTPCNDKKSEKNSAQKRHTPITFGNDSSPNKRNKEDKNNKREIYTPPSGKYSSNISNYGRGRFKGNRPRGRGGFRTRGRGPWRKNFY

>270

MAVTIEDLYRNYGILADAKENLSQHKDAYQVILDGVKGGAKEKRLAAQFIPKFFSSFPELADAAINAQLDLCEDEDVSIRRQAIKELPRFATGENIFRVADILTQLLQTDDTAEFNQVNAALLSIFKMDAKGTLGGLFSQILQGEDIVRERAIKFLSTKLKTLPEDVMSKEVEEFVFAETKKVMEDVTGEEFVLLMRVVSGLRVLQSVNGRQQLVELVVDQADLEQALNPADPDAVDRLLQCTRQALPLFSKNVHSTRFVTYFCEHVLPNLSTLTSPVAELDIQLEVLKLLAEMSPFCGDMEKLEANLNILFTKLLEFMPLPPEEVENGENSSSEEPKLQFSYVECLIFSFHQLGKKLPDFLVDKVDAEHLKDFKIRLQYFARGLQVYIRQLRVALQGKTGDALKTEENKIKVVALKITNNINVLIKDLFHNPPSFKSTVTLSWKPVQKPEAVAAKRPSGEEMGSGGSTKKQIAPLPRRDARQIYNPPSGKYSASIGNFTYEQRGGFRGGRGRGFGARGSRSRGRVY

>271

MWKFDNKFIMSMDSIEKLYKNFGILADAKDKLAEHEKEYLEILTAVKGSPKEKRLASQFIARFFKYFPKLADQAIDAHLDLCEDEDMAIRKQAIKDLPALCKDNKEHTARIADILAQLLQAQDPSELAVVHNSVMSLMKTDPRGTISGFFSQIINGDDGTRERCIKFLATKLKAIGHDIITKEPEDLLISECKKVLQDVTADEFHSIMEVLAWTRLGSTISGQQELVDITVEQAELSEPFKHTNVEQWNRLVQCIKHALPFFSSQIDSSRFVSYICVQVLPHLSLITSPNGRDVQLELLKLLAELAVFCGTIDKPEEKVQQLYNTLITYMPLPPVTEITEVPKLQFSHVECLMYTFHKLCKQTPEFLMKDPEQLKEFRLRLQYFARGIQGYIKKLREAISGKSEEELKSEENQLKVVALKTTNNINSLIKDLFHSPPSFKSIIHLSWKTTANDKKNEKHSTAQKRHTPITFGNDSNSNKRSKEDKSKRELYTPPSGKYSSNISSNYGRGRFRGNRSGGRGGYRPRGRGTWRKNFY

>272

MAVTIEDLYRNYGVLADAKDNLSQHKDAYQVILAGVKGGPKEKRLAAQFIPKFFDNFPELADAAINAQLDLCEDEDVSIRRQAIKELPRFATGENIFRVADILTQLLQTDDTAEFNQVNAALISIFKMDAKGTLGGLFSQILQGEDIVRERAIKFLSTKLKTLPEDIMTKEVEDYVFAETKKVLEDVTGEEFVLLMRVVSGLRVLQTVNGRQQLVELVVEQADLDQALNPADPDAVDRLLQCTRQALPLFSKNVHSTRFVTYFCENVLPNLSTLTSPVAELDIQLEVLKLLAEMSPYCGDMEKLEANLTMLFTKLLEYMPLPPEEVENGENSASEEPKLQFSYVECLLFSFHQLGKKLPDFLLDKVDGERLKDFKIRLQYFARGLQVYIRQLRVALQGKTGDALKTEENKIKVVALKITNNINILIKDLFHNPPSFKSTVTLSWKPVQKAEAVAAKRLSSEEMGSGGSTKKQITPLPRRDARQIYNPPSGKYSSSIGNFNYEQRGGFRGGRGRGFGTRGNRSRGRIY

>273

MAVTIEELYRNYGILADAKPEDLSQHKDAYQVILDGVKGGPKEKRLAAQFIPKFFSSFPELADAAINAQLDLCEDEDVSIRRQAIKELPRFAAGENIVRVADILTQLLQTDDSAEFNQVNTALVSIFKMDAKATLGGLFSQILQGEDIVRERAIKFLSIKLKTMPEDAMTKEVEDYIFTETKKVLEDVTGEEFVLLMRILMVLKGLQTMSGRQQLVELVVEQAFLEQALNPADPDTVDRLLQCTRQALPLFSKNVHSTRFVTYFCDHVLPNLSSLTSPVADLDIQLEVLKLLAEMSPFCGHGKVETNLTMLFEKLLEFMPLPPEAEGENGENTLSDEPKLQFSYVECLLFSFHQLGKKLPDFLIDKINAERLKDFKIRLQYFARGLQVYIRQLRVALQGKTGDALKTEENKIKVVALKITNNINVLIKDLFHNPPSYKSTVTLSWKPVQKAEAVALKRPSGEDMGAGSTMKKQLSPPLPRRDARQIYNPPSGKYSATIGNFSNEQRGGFRGGRGRGFGGRGGRSRGRIY

>274

MAVTIEELYRNYGILADAKPEDLSQHKYAYQVILDGVKGGPKEKRLAAQFIPKFFSSFPELADAAINAQLDLCEDEDVSIRRQAIKELPRFAAGENIVRVADILTQLLQTDDSAEFSQVNAALVSIFKMDAKATLRGLFSQILQGEDIVRERAIKFLSIKLKTLPEDTMTKEVEDYVFTETKKVLEDVTGEEFVLLMRILMALKGLQTVNGXQQLVELVVEQAFLEQALNPADPDTVDRLLQCTRQALPLFSKNVHSSRFVTYFCDHVLPNLSTLTSPVAELDIQQEVLKLLAEMSLFCGDMEKVEANLLMLFEKLLEFMPLPPEAEGENGENTMSEEPKLQFSYVECLLFSFHQLGKKLPDFLIDKINAERLKDFKIRLQYFARGLQVYIRQLRVALQGKTGDALKTDENKIKVVALKITNNINVLIKDLFHNPPSYKSTVTLSWRPVQKAEAVTLKRPSGEEMGTGSTMKKLLSPPLPRRNARQIYNPPSGKYSATIGNFTNEQRGGFRGGRGRGFGGRGGRSRGRIY

>275

MPTVEELYRNYGILADATETAGQHKDAYQVILDGVKGGAKEKRLAAQFIPKFFKHFPELADSAINAQLDLCEDEDVSIRRQAIKELPQFATGDNLPRVADILTQLLQSDDSAEFNLVNNALLSIFKMDAKGTLGGLFSQILQGEDIVRERAIKFLSTKLKTLPEEVLTKEVEELILTESKKVLEDVTGEEFVLFMKILSGLKSLQTVSGRQQLVELVAEQADLEQTFNPSDPDCVDRLLQCTRQAVPLFSKNVHSTRFVTYFCEHVLPNLSSLTTPVEGLDIQLEVLKLLAEMSSFCGDMEKLESNLKKLFDKLLEYMPLPPEEAENGENAGNEEPKLQFSYVECLLYSFHQLGRKLPDFLTSKLNAEKLKDFKIRLQYFARGLQVYIRQLRLALQGKTGEALKTEENKIKVVALKITNNINVLIKDLFHIPPSYKSTVTLSWKPVQKADSGQKRTSEDTTSSSPPKKAPAGPKRDARQIYNPPSGKYSSNLGNFSYEQRGGFRGGRGRGWGGRGNRSRGRIY

>276

MAVTIEDLYRNYGVLADAKDNLSQHKDAYQVILAGVKGGPKEKRLAAQFIPKFFNSFPELADAAINAQLDLCEDEDVSIRRQAIKELPRFATGENIFRVADILTQLLQTDDTAEFNQVNVALISIFKMDAKGTLGGLFSQILQGEDIVRERAIKFLSTKLKTLPEDIMTKEVEEYVFAETKKVLEDVTGEEFVLLMRVASGLRVLQTVNGRQQLVELVVEQADLDQALNPADPDAVDRLLQCTRQALPLFSKNVHSTRFVTYFCEHVLPNLSTLTSPVAELDIQLEVLKLMAEMSPYCGDMEKLEANLTILFTKLLEFMPLPPEEVENGENSASEEPKLQFSYVECLLFSFHQLGKKLPDFLLDKVDGERLKDFKIRLQYFARGLQVYIRQLRVALQGKTGDALKTEENKIKVVALKITNNINILIKDLFHNPPSFKSTVTLSWKPVQKTEAVAAKRLSSEEMGSGAATKKQITPLPRRDARQIYNPPSGKYSSSIGNFNYEQRGGFRGGRGRGFGARGNRSRGRIY

>277

MAEVKNETYAKAKSHFLEYHPTVTGVEPLRIMHKDAYQVILDGVKGGAKEKRLAAQFIPKFFKHFPELADSAINAQLDLCEDEDVSIRRQAIKELPQFATGDNLPRVADILTQLLQSDDSAEFNLVNNALLSIFKMDAKGTLGGLFSQILQGEDIVRERAIKFLSTKLKTLPEEVMTKEVEEFILTESKKVLEDVTGEEFVLFMKILSGLKSLQTVSGRQQLVELVAEQADLEQTFNPSDTDCVDRLLQCTRQAVPLFSKNVHSTKFVTYFCEHVLPNLSSLTTLVEGLDIQLEVLKLLAEMSSFCGDMEKLESNLKKLFDKLLEYMPLPPEEAENGENAGNEEPKLQFSYVECLLYSFHQLGRKLPDFLTAKLNAEKLKDFKIRLQYFARGLQVYIRQLRLALQGKTGEALKTEENKIKVVALKITNNINVLIKDLFHIPPSYKSTVTLSWKPVQKTDVSQKRASEDTTSSSPPKKASAGPKRDARQIYNPPSGKYSSNLGSFSYEQRGGFRGGRGRGWGGRGNRSRGRIY

>278

MSTDRIEKLYKNFGILADSKDKSEHEAEYLEILTAVKGSSKEKRLASQFIARFFKHFPALADQAIEAQLDLCEDEDVAIRKQAIKDLPSLCKDNKEHTQKIADILAQLLQAEDNAELSVVHNSLMTLFKTDAKGTLGGIFSQILSGDDLIRERCIKFLTVKIKSLGHDVITKDAEDVLISECKKVLQDVTADEFHNLMELLGWTRLGHLVSGQQELVDIVAEQAEINQHFDPKDVENDNVDRLIQCVKHALPYFSSQVDSARFVAYMCEQVLPQFEEISSNEEGADPQLEILKLFAELCTHCGNLENAESKVEKVFERLIEYMPQPPESDVEKPSQEEPRLEFSYVECLMYAFHRLGRQCPDFLTKDAERMKDFRFRLQYFARGIQGYIKKLREALQGKTSDELKTDENKIKVVALKTTSNINTLIKDLFHSPPSYKSIISLSWKPTAGSAKDGPSTGQKRHTPITFSSDSSGNKHPKTDKSQREIYQPPSGKYSNKVSSYVPSQQRGRGRGRGRSFRGGRGWRRSY

>279

MAATVEELYRNYGILADAKDNLSSHKDAYQKILDGVKGGPKEKRLAAQFIPKFFSNFPELADAAINAQLDLCEDEDVSIRRQAIKELPRFAAGENLPRVADILTQLLQTDDSAEFNQVNTALISIFKMDAKGTLGGLFTQILQGEDIVRERAIKFLSTKLKTLPEEVMTKDVEDYVFMEIKKVLEDVTGEEFVLLMRILSSLKSLQTVSGRQQLVELVVEQAFLEQVLDPTDADSVDRLLQCTRQALPLFSKNVHSTRFVTYFCEHVLPNLGSLTSPVAELDIQLEVLKLLADMSRFCGDMEKLETNLTILFEKLLEFMPLPPEEGENGENAGSEEPKLQFSYVECLLFSFHQLGKKLPDFLIDKINAERLKDFKIRLQYFARGLQVYIRQLRVALQGKTGDALKTEENKIKVVALKITNNINVLIKDLFHNPPSYKSTVTLSWKPVQKTEVTGQKRPSGEETGSGPVMKKLSPLPRRDARQIYNPPSGKYSATIGNFSYEQRGGFRGGRGRGWGGRGNRSRGRIY

>280

MPTVEELYRNYGILADATEQVGQHKDSYQVILDGVKGGTKEKRLAAQFIPKFFKHFPELADSAINAQLDLCEDEDVSIRRQAIKELPQFATGENLPRVADILTQLLQTDDSAEFNLVNNALLSIFKMDAKGTLGGLFSQILQGEDIVRERAIKFLSTKLKTLPDEVLTKEVEELILTESKKVLEDVTGEEFVLFMKILSGLKSLQTVSGRQQLVELVAEQADLEQTFNPSDPDCVDRLLQCTRQAVPLFSKNVHSTRFVTYFCEQVLPNLSSLTTPVEGLDIQLEVLKLLAEMSSFCGDMEKLETNLRKLFDKLLEYMPLPPEEAENGENAGNEEPKLQFSYVECLLYSFHQLGRKLPDFLTAKLNAEKLKDFKIRLQYFARGLQVYIRQLRLALQGKTGEALKTEEVRVLAHISYFLFSKSIVLIKDLFHIPPSYKSTVTLSWKPVQKVELGQKRANEDATSGSPPKKPTAGPKRDARQIYNPPSGKYSSNLGNFNYEQRGAFRGNRGGRGWGARGNRSRGRLY

>281

IRISAMKVLPLLCKDAKEHVCRVADILAQLLQLEDQDYNTACSALIQVFKEDELNTVKAIFNHIHTTEENTSREKNIQFLYKKLIKIPEKLSSEIEEILLEEGKKIIQDASATEFWVVMPYLTSSKLAKTITGQKELVELVAERAEIDRDFDPHEEGTSDRIIMCVECILPLFNANVESTKFLIYYCDQILPHWDEIGKLNDGSTLQYQFLKQLTELSAHCGKLENPSLHVVQIFDKLKQYMPLPPDDVDIDKMPNLDFTTVECLLYSFHRLARQCPDFLTADPLVLKDFRSRLNYFSRGVGGCKKSLEKIQVKKDDVDKIKIAPAVLDNITALIKDLFYTTPVYKCNIQLSFKTIDNKIKISPAKPTGPQKRHVPITFDSSNGSSSKHIRSNKSGENVKLYTPPSGKFSNNFQNYDRPSIRGRGNRGGRSIRGRGSLRGGWRN

>282

MSADSASQSIQKLYKHFGVLADAKDKLSEHEAEYLEILKAVKGCPKEKRLASQFIARFFKNFPKLADKAIDAHLDLCEDEDISIRKQAIKDLPALCKDSKEHTARIADILAQLLLAPDPSELDVVQNSIMTLIKNDPKGAINGFFIQILNAEDGIRERVIKYLSSKLKTLGREIINKEVEEVIISECKKVLQDVTADEFHNIMELLAWTRLGSTVSGQQELVDIAVEQAELNMPYTNNFEQFSRLIQCIQHALPYFSSQVDSQRFVSYVCMEVLPHLSSIADPSGKDVQLELLKLLAELSEHCGKLEKPEEKVEQLYKTLVNYMPLPPDSDITEVPKLQFSHVECLMYAFHKICKQAPEFLIKDPEQLKEFRLRLQYFARGIQGYIKKLREAIHGKSEEDLKSKENQIKVIALKTTSNINTLIKDLFHSPPSFKSVIHPSWKTIIVQEKKIDKVTTGTKRHTPITFGNGNSKDNHDNTNNSIKRIKLDNQRNKQLYTPPSGKYSSNISQNYGNRGGRFQNQNRFQNNKMNGGRGGFRPNRGRGAWRKNNSY

>283

MSTDSIEKLYKNFGVLADAKDKLAEHEKEYLEILTAVKGSPKEKRLASQFIARFFKHFPKLADRAIDAHLDLCEDEDMAIRKQAIKDLPALCKDSKEHTPRIADILAQLLQAQDSSELAVVHNSIMTLLKNDPKGTLSGFFSQILSGDDGTRERCIKFLATKLKAIGRDVITKEPEDLLIVECKKVLQDVTADEFHNIMEVLAWTRLGSTLTGQQELVDIAIDQAELSIPFKHTNMEQWNRLVQCVKHALPFFNSHIDSSKFVSYICVQVLPHLSLITSPDGRDVQLELLKLLAELAVFCGSIEKPEEKVQQLYNTLITYMPLPPAVEVTEVPKLQFSHVECLMYAFHKLCKQIPEFLTRDADQLKEFRLRLQYFARGIQGYIKKLREAISGKTEDELKSEENQLKVIALKTTNNINTLIKDLFHSPPSFKSIIHLSWKTPTNGKKPLEKATVAPKRHTPITFGNDSTSNKRNKDDTNSARSNKREIYTPPSGKYSSNISQNYGNRGRFRGNRSGGRGGFRPRGRGAWRKNTY

>284

MTQDSIEKLYINFGVLAEAKDKLQEHEKEYLEILKAVKGSAKEKRLASQFIARFFKHFPNLAEKAIDAHLDLCEDEDISIRKQAVKDLPLLCKDNKEHSAKIADILAQLLVVKDSSELDVVHNSIMTVIKNDPKGAIRGIFSQILNSAEDNGGDVVREHCIKYLSTKLKALGRDVITKDVEDVLIAECKKVLQDVTAEEFHSIMEMLSWTRLGSTVNGQKELVEITIEQAQLSVPFKHTDLEQCSRLIQCIKHALPYFSSQVESTKFVSYISMQVLPHLSLITSPDKSDVQLELLKLLAELSEYCGKIEKPEEKIQQLYNALITFMPLPPATEIAETPKLQFSHVECLMYAFHKLCKQTPEFLIKDADQLKEFRLRLQYFARGIQGYIKKLREAINGKSEEELKTKENQIKVIALKTTNNINTLIKDLFHTPPSFKSVIHPSWKTIVSKEKKSDKITIGQKRHTPITFGNGNSITSSNSNNSNNHASNKRMKVDSVRNNKIYTPPSGKYSSNISSNYGNNRGRFQNNQRYNNNNGRVNSGRGTGGGGFRPRGRGSWRKNSY

>285

MAVTIEDLYRNYGILADAKDNLSQHKDAYQVILDGVKGGPKEKRLAAQFIPKFFSSFPELADAAINAQLDLCEDEDVSIRRQAIKELPRFATGENILRVADILTQLLQTDDTAEFNQVNASLISIFKMDAKGTLGGLFSQILQGEDIVRERAIKFLSTKLKSLPEDVMTKEVEEYVFAETKKVLEDVTGEEFVLLMRIVSGLRVLQTVNGRQQLVELVVEQAFLEQALNPADPDTVDRLLQCTRQALPLFSKNVHSTRFVTYFCEHVLPNLSTLTSPVAELDIQLEVLKLLAEMSPFCGDMEKLEANLNMLFTKLLEYMPLPPEEVENGENSASEEPKLQFSYVECLLFSFHQLGKKLPDFLLDKVDAERLKDFKIRLQYFARGLQVYIRQLRVALQGKTGDALKTEENKIKVVALKITNNINVLIKDLFHNPPSYKSTVTLSWKPVQKTEAVAPKRPSNEDMGSGGSTKKQISPQPRRDARQIYNPPSGKYSASIGNFNYEQRGGFRGGRGRGFGARGNRSRGRIY

>286

MPLYDPAVCVLPVPALERDRGFECLRNHPSSYWRGYGEKTSVPIQFRIRKHGVVNKHKDAYQAILDGVKGGAKEKRLAAQFIPKFFKHFPELADSAINAQLDLCEDEDVSIRRQAIKELPQFATGDNLPRVADILTQLLQSDDSAEFNLVNNALLSIFKMDAKGTLGGLFSQILQGEDIVRERAIKFLSTKLKTLPEEVMTKEVEEFILTESKKVLEDVTGEEFVLFMKILSGLKSLQTVSGRQQLVELVAEQADLEQTFNPSDPDCVDRLLQCTRQAVPLFSKNVHSTKFVTYFCEHVLPNLSSLTTPVEGLDIQLEVLKLLAEMSSFCGDMEKLESNLKKLFDKLLEYMPLPPEEAENGENAGNEEPKLQFSYVECLLYSFHQLGRKLPDFLTAKLNAEKLKDFKIRLQYFARGLQVYIRQLRLALQGKTGDALKTEENKIKVVALKITNNINVLIKDLFHIPPSYKSTVTLSWKPVQKADASQKRASEDTTSSSPPKKASAGPKRDARQIYNPPSGKYSSNLGSFSYEQRGGFRGGRGRGWGGRGNRSRGRIY

>287

MADSLAEMYEKYNILSDAKENISKHSKEYLQCIERAKGSDKEKQLAAQIISKFFKHFPSLQDQAIEAIYILCEDDDSLIQICTIKILHVICKDAKEHIPKIVNILAQLLQAEDKVSNAASSSLLQVFKEDPHTTVKVMLDFINNPCISSSTEDIPATEKSVEFLYKKLVKLDEKLTPEINDLLLEEGKKIILDSNATEFLTVLPFLASSKLTKTIAGQQELVNLIAERAEIDKDFDPLDEGSQNADKIMMCVEHALPFFSANVESTRFVKFYCDQILGHWKEMGTLKDGSIMQYQFLKQLAELSTHCGKLETPSFYVVQIFDKLKLYMPLPPEDCDIDKMPNLDFTSVECLLYSFHRIARQCPDFLTSDPMVLKDFRSRLNYFSRGVGGCKKSLEKIQIKNKDDEKIKIAPAVLDNITTLIKDLFYTTPVYKCNIHLSFKSLEKKIKLSPENPQAPQKRHAPIHFESSNGSTNKQIRSNKGAENKLYTPPSGKFSSNFQSFDRVNRGRGRGGRGTRGSRGSSRSWRN

>288

MGEVTFDKLYDIYNTLSQAKDNITQHTQEYLEAIEGTKGGEKEKKLAAQVISKFFKHFPSLQLQAIDAIFDICEDDQASIRIAAMKTLPILCKESKENIGKIADVLSQLLQLDDPQEYNVASNSLIQVLQIDSLLVIKSIFKQLSESDALVREKCLKFLVTKVKTLDKSIFTPEIEDLILAEFKKILNDVTADEFVLIMGYLSTSKAANTQAGQIELINICADQVEMDQDLDMFDKDANNIDRLVTCVKFVLPFFSAKIESTKFVKYYCDQTLPRWDEIKELTDGESYQLMILRQLAELSTHCGKLENPSVQVVQIYDKIKTYMPPPPEDTTLTTLPSLDFSSVEALLYAFHRLARQCPDFLTHDPQELKEFRARLMYFSRGVQGCRNAIKADKTKTLSEENEKVMKISPIMFDNINTIIKDLFYQPPMYKCNVTLSFKTEVSPLKKTTEVNVTQKRHVPITFESKNGSAPPYKQGRPNRSGDNVKLYTPPSGKFSNNFQHYDRQRGNRFGGRGGRGGLRGRGSGRGWRN

>289

MSADNIEKLYQNYGILADAKDDISKHEKEYLEILAAVKGSDKEKRLASQFIAKFFNSFPNLADQAIEAQFDLCEDDDVAIRKQAIKDLPTLCKDHKEHTQRIADILAQLLQSEDTTEINVVTSSLLTILKIDPKGALTGMFSQIHQNADSEVTNEVVRERCIKFLASKVKQLGREVITKEAEDLIVAECKKLLELQDVVAEEFEHIMELLTWSKLGKTPAGKKELVQIVAALAFTPDDWHPEDPEYVERVIQCTQHALPLFSALVDSTQFVNFFCDHILLGWDNITTPEGMDPKLELLKIFAEITEHCGELENPPKKIDAVYELLMKYLPEAPIENEVAEGEKSEDKPEDSKTAPLLQFSHVECALFALHSLCRKAPEALTTDAAKIKALRLRLQYTARLTQGYIKKLKEVTQGQKGEDANSEENKLKIAALKTTSNINTLIRDLFRTPPSFKSKVQLSFHTKKVDKEEKQSADPETTKQSPGQKRHRPITFDNDNEKESPEKRSRSGDRNVKMYTPPSGKYSSRLNNNGRFSGNNSGGRGGRGGYGRRDFRNNGAPFRRRNNY

>290

MAVTIEDLYRSYGILADAKDNLCQHKDAYQVILNGVKGGAKEKRLAAQFIPKFFSSFPELADAAINAQLDLCEDEDVSIRRQAIKELPRFATGDNILRVADILTQLLQTDDTAEFNQVTAALISIFKMDAKGTLGGLFSQILHGEDIVRERAIKFLSTKLKTLPEDVMSKEVEDYVFAETKKVLEDVTGEEFVLLMRVVSGLHVLQTVNGRQQLVELVVEQADLEQALNPSEPDAVDRLLQCTRQALPLFSKNVHSTRFVTYFCEHVLPNLSTLTSPVAELDIQLEVLKLLAEMSPFCGDMEKLEANLSMMFTKLMEFMPLPPEEAENGETSANEEPKLQFSYVECLIFSFHQLGKKLPDFLVDKVDAERLKDFKIRLQYFARGLQVYIRQLRVALQGKTGDALKTEENKIKVVALKITNNINVLIKDLFHNPPSYKSTVTLSWKPVQKPEGVATKRPSTEDTGSGGSTKKPVVSLPRRDARQIYNPPSGKYSASIGNFTYEQRGGFRGGRGRGFGTRGSRSRGRIY

>291

MAVTIEDLYRSYGILADAKDNLCQHKDAYQVILNGVKGGAKEKRLAAQFIPKFFSSFPELADAAINAQLDLCEDEDVSIRRQAIKELPRFATGDNILRVADILTQLLQTDDTAEFNQVTAALISIFKMDAKGTLGGLFSQILHGEDIVRERAIKFLSTKLKTLPEDVMSKEVEDYVFAETKKVLEDVTGEEFVLLMRVVSGLHVLQTVNGRQQLVELVVEQADLEQALNPSEPDAVDRLLQCTRQALPLFSKNVHSTRFVTYFCEHVLPNLSTLTSPVAELDIQLEVLKLLAEMSPFCGDMEKLEANLSMMFTKLMEFMPLPPEEAENGESSANEEPKLQFSYVECLIFSFHQLGKKLPDFLVDKVDAERLKDFKIRLQYFARGLQVYIRQLRVALQGKTGDALKTDENKIKVVALKITNNINVLIKDLFHNPPSYKSTVTLSWKPVQKPEGVATKRPSTEDTGSGGSTKKPVVSLPRRDARQIYNPPSGKYSASIGNFTYEQRGGFRGGRGRGFGTRGSRSRGRIY

>292

MAVTIEDLYRSYGILADAKDNLCQHKDAYQVILNGVKGGAKEKRLAAQFIPKFFSSFPELADAAINAQLDLCEDEDVSIRRQAIKELPRFATGDNILRVADILTQLLQTDDTAEFNQVNAALISIFKMDAKGTLGGLFSQILHGEDIVRERAIKFLSTKLKTLPEDVMSKEVEDYVFAETKKVLEDVTGEEFVLLMRVVSGLHVLQTVNGRQQLVELVVEQADLEQALNPSEPDAVDRLLQCTRQALPLFSKNVHSTRFITYFCEHVLPNLSTLTSPVAELDIQLEVLKLLAEMSPFCGDMEKLEANLSMMFTKLMEFMPLPPEEAENGETSANEEPKLQFSYVECLIFSFHQLGKKLPDFLVDKVDAERLKDFKIRLQYFARGLQVYIRQLRVALQGKTGDALKTEENKIKVVALKITNNINVLIKDLFHNPPSYKSTVTLSWKPVQKPEGVATKRPSTEDTGSGGSTKKPAVSLPRRDARQIYNPPSGKYSASIGNFTYEQRGGFRGGRGRGFGTRGSRSRGRIY

>293

MAVTIEDLYRSYGILADAKDNLSQHKDAYQVILDGVKGGAKEKRLAAQFIPKFFSSFPELADAAINAQLDLCEDEDVSIRRQAIKELPRFATGDNILRVADILTQLLQTDDTAEFNQVNAALISIFKMDAKGTLGGLFSQILQGEDIVRERAIKFLSTKLKSLPEDVMSKEVEEYVFAETKKVLEDVTGEEFVLLMRVVSGLHVLQSVNGRQQLVELVVEQADLEQALNPSESDAVDRLLQCTRQALPLFSKNVHSTRFVTYFCEHVLPNLSTLTSPVAELDIQLEVLKLLAEMSPFCGDMEKLEANLTMLFTKLMEFMPLPPEEVENGENSANEEPKLQFSYVECLIFSFHQLGKKLPDFLVDKVDAERLKDFKIRLQYFARGLQVYIRQLRVALQGKTGDALKTEEQNKIKVVALKITNNINVLIKDLFHNPPSYKSTVTLSWKPVQKPEGVAAKRPSTEDMGSGGSTKKPVVPLPRRDARQIYNPPSGKYSASIGNFTYEQRGGFRGGRGRGFGARGNRSRGRIY

>294

MAVTIEDLYRSYGILADAKDNLSQHKDAYQVILDGVKGGAKEKRLAAQFIPKFFSSFPELADAAINAQLDLCEDEDVSIRRQAIKELPRFATGDNILRVADILTQLLQTDDTAEFNQVNAALISIFKMDAKGTLGGLFSQILQGEDIVRERAIKFLSTKLKSLPEDVMSKEVEEYVFAETKKVLEDVTGEEFVLLMRVVSGLHVLQSVNGRQQLVELVVEQADLEQALNPSESDAVDRLLQCTRQALPLFSKNVHSTRFVTYFCEHVLPNLSTLTSPVAELDIQLEVLKLLAEMSPFCGDMEKLEANLTMLFTKLMEFMPLPPEEVENGENSANEEPKLQFSYVECLIFSFHQLGKKLPDFLVDKVDAERLKDFKIRLQYFARGLQVYIRQLRVALQGKTGDALKTEENKIKVVALKITNNINVLIKDLFHNPPSYKSTVTLSWKPVQKPEGVAAKRPSTEDMGSGGSTKKPVVPLPRRDARQIYNPPSGKYSASIGNFTYEQRGGFRGGRGRGFGARGNRSRGRIY

>295

MAATVEELYRNYGILADAKEDLSKHKEAYQVILDGVKGGPKEKRLAAQFIPKFFSSFPELADAAINAQLDLCEDEDVSIRRQAIKELPRFASGENLPRVADILTQLLQTDDSAEFNQVNTALISIFKIDAKGTLGGLFSQILQGEDVVRERAIKFLSSKLKTMPEDIMTKEVEDYIFTETKKVLEDVTGEEFVLLMRVLSGLKYLQTVSGRQQLVELVVEQAFLEQALNPADTDSVDRLLQCTRQALPLFSKNVHSTRFVTYFCEHVLPNLSMLTSPVAELDIQLEVLKLLAEMSPFCGDMDKLEANLTMLFEKLLEFMPLPPEEENGENATSEEPKLQFSYVECLLFSFHQLGKKLPDFLIDKVDAEKLKDFKIRLQYFARGLQVYIRQLRVALQGKTGDALKTEENKIKVVALKITNNINVLIKDLFHNPPSYKSTITLSWKPVQKTEAAAAVVGQKRASGEDTDAGPLGGKKVSPLPRRDARQIYNPPSGKYSATIGNFSYEQRGGFRGGRGRGFGGRGNRSRGRIY

>296

MSTFQYPPPVITSNSDYSLLREQFRRLERTPDRKSPQRRDLFKLLISLVQSPDAAKKKLVTERVKDYFPDFRDLQEAAINAIYDICEDQEQSIRIAGYNAITSISIADPTWIVRNVDVLVQLLQSDEDVEVTVVKKQLQQHVDIDPRHTLQVLCDQCIFESEGKMDTDEVNLRTRLRGLVLQFLDEKLRGCILRVVKEKDVESILLKGMIGTIPYASPSELQIIVKNILTQLPSLGGGPSEAGNSVLLALLQSASPHILSSTTLSTSPSATYLLNVLSLASDITAPSSSQLAPRSIPSAPASMRQPVMHSPADALILLRFFKERDILRKLPEGSEELTVKFVEIVFSAYESASLISGSLKAKKEAQSILGEVAESLLEALVKSLSHQSEQQKKLILCLKILQAIHQGGDISRITDPSLVDENEFSHWLSELQKTLLDQHIPKESLNVDIKEVMDSLLGLIRKILGDIALLKTKTTNLGTQSISNSRPISTIGSSEARLSDRGPVLGPKAGQVQLHAQVQRQQKALPSRRHVVDERNREQQRRLIEEQRERARTQRQVAIPQNQNRPWSQGTIPTGPRGERRLFPTNGDKTTEGHGPVSGSTLSVRGAAGRANGDDTMDVDSSVYDISNEKNNNVAMDDKPEAEGEPARKRVKSGNLSAPGSVAANGDLSGNHLTTEGPSLLSRLSPGGIPGASRSENTATRLTSRIGVPSTPSAVGMGTDNHLVREGVTSTTSSIPSLISRLSSSASSSPAHTSTRPISVNGGLERNHITNVIRPSIIVSGKGSRASNIDSNGSATRDGGEEIKIRGAAAVSSSDPKSEKASGSFGMRLMDRLQMDANAKAAGDGDGNGQRKRKLDRR

>297

MEAEDNIQKMYKYFGILADAKENIAEKEPEYLEILSAVKGSTKEKRLASQFITRFFKYFPNLAAQALEAQLDLCEDEDICIRKQAIKDLPVLCKEAKEYLTKIADILAQLLQADDPQELLVAQNSLLSLFKIDAKGALTGIFSQMQSNEEIVRERSMKFILNKVMALGKEIIKKDVEDLIIAECKKVMQNITCEEFETLMTILSSTHLINTPEGQKELVELLASTAELDQFFNPKDLDQVNRFITCLDYSIPFFSAHVESTKFIVYICELLNRYTLIKDNDKQFIILKSLAESVPYCGKLMNPEAVVGQVYQALLDLVTVPENDSNKKVEDMDLHRVEALLYTFHKLGKQCPDFLSKDPERQKDFKKKLLYVGTCTQTFVKVVRQDLKEKGEEDVKNDPTVEKKLEGLKLACNINTLIKELFNIPPRFKATVNLSWLGGATKSKLAQIIQEGKKHEPITVDGKSKRIDGSNNSQQLYQPPKDKFSAKFSNNTNNNNRRGGSNTGWNAKRSFDTNNANRRSWRPY

>298

MTTDNIEKLYQNYGVLADAKDDISKNEKEYLEILAAVKGSDKEKRLASQFIAKFFSSFPSLSEQAIEAQFDLCEDDDVAIRKQAIKELPIMCKSNKEHTQRIADILAQLMQSEDPTEINVVTNSLLAIVKIEPLGALAGIFSQIHQSTNSEVPNETVRERCIKFLATKVKQLGREVINKEAEDLIIVEGKKILEDVGAEEFEHIMDLLAWSRLGKTPAGKKELTQIIATLAFSPDDWHPEDLEYVDRLIQCSQHALPLFTAQVDSTQFVNIFCDHVLSKWSNIATTGGGTDTKLEILKIFAELTEHCGDIEDIEKKINTVYNVLLEYLPEAPVPTEDESTDKDVEKEKSEENKTVPSLQFSHVECALFALHSLCRKSPEAIGVDAARLKALRLRLQYTARLTQGYIKKLKEVTQGKKGEDSNSDENKLKIAALKTTSNINTLIRDLFRTPPSFKSKIQLSFASAKGPKEEKVVLPAEEKESGAQKRHRPITFDNGEKKESPEKRSRSGDRNIKMYTPPSGKYSSRFNTGRFSGGNSGRGRGYGRRDFQNNGASFRRRNY

>299

MAVTIEDLYRNYGILADAKDNLSQHKDAYQVILDGVKGGPKEKRLAAQFIPKFFSSFPELADAAINAQLDLCEDEDVSIRRQAIKELPRFATGENILRVADILTQLLQTDDTAEFNQVNASLISIFKMDAKGTLGGLFSQILQGEDIVRERAIKFLSTKLKTLPEDVMTKEVEEYVFAETKKVLEDVTGEEFVLLMRIVSGLRVLQTVNGRQQLVELVVEQAFLEQALNPADPDTVDRLLQCTRQALPLFSKNVHSTRFVTYFCEHVLPNLSTLTSPVAELDIQLEVLKLLAEMSPFCGDMEKLEANLNMLFTKLLEYMPLPPEEVENGENSASEEPKLQFSYVECLLFSFHQLGKKLPDFLLDKVDAERLKDFKIRLQYFARGLQVYIRQLRVALQGKTGDALKTEENKIKVVALKITNNINVLIKDLFHNPPSYKSTVTLSWKPVQKTEAVAPKRPSSEDMGSGGSTKKQISPQPRRDARQIYNPPSGKYSASIGNFNYEQRGGFRGGRGRGFGARGNRSRGRIY

>300

MNEGEGFDFTDTHKHVPSDEPQTMNSERGISSSLRAFPPLLSLLLCMTVICQLYQYYLMGGLHKDAYQVILDGVKGGTKEKRLAAQFIPKFFKHFPELADSAINAQLDLCEDEDVSIRRQAIKELPQFATGENLPRVADILTQLLQTDDSAEFNLVNNALLSIFKMDAKGTLGGLFSQILQGEDIVRERAIKFLSTKLKTLPDEVLTKEVEELILTESKKVLEDVTGEEFVLFMKILSGLKSLQTVSGRQQLVELVAEQADLEQTFNPSDPDCVLKLLAEMSSFCGDMEKLETNLRKLFDKLLEYMPLPPEEAENGENAGNEEPKLQFSYVECLLYSFHQLGRKLPDFLTAKLNAEKLKDFKIRLQYFARGLQVYIRQLRLALQGKTGEALKTEENKIKVVALKITNNINVLIKDLFHIPPSYKSTVTLSWKPVQKVEMGQKRANEDTTSGSPPKKSPSGPKRDARQIYNPPSGKYSSNLSNFSYEQRGAFRGSRGGRGWGARGNRSRGRLY

>301

MPTVEELYRNYGILADATETAGQHKDAYQVILDGVKGGAKEKRLAAQFIPKFFKHFPELADSAINAQLDLCEDEDVSIRRQAIKELPQFATGDNLPRVADILTQLLQSDDSAEFNLVNNALLSIFKMDAKGTLGGLFSQILQGEDIVRERAIKFLSTKLKTLPEEVMTKEVEEFILAESKKVLEDVTGEEFVLFMKILSGLKSLQTVSGRQQLVELVAEQADLEQTFNPSDTDCVDRLLQCTRQAVPLFSKNVHSTKFVTYFCEHVLPNLSSLTTLVEGLDIQLEVLKLLAEMSSFCGDMEKLESNLKKLFDKLLEYMPLPPEEAENGENAGNEEPKLQFSYVECLLYSFHQLGRKLPDFLTAKLNAEKLKDFKIRLQYFARGLQVYIRQLRLALQGKTGEALKTEENKIKVVALKITNNINVLIKDLFHIPPSYKSTVTLSWKPVQKADASQKRASEDTTSSSPPKKASAGPKRDARQIYNPPSGKYSSNLGSFSYEQRGGFRGGRGRGWGGRGNRSRGRIY

>302

MAGDNIEKLYKNYDILAAAKDEISQHEKEYLEILAAVKGSDKEKRLASQFIAKFFNSFPTLSEQAIEAQFDLCEDDDVAIRKQAIKDLPVLCKEHKEHTQRIADILAQLLQSEDSTEINVVTNSLVTILKSDPKGALSGIFSQIHQNTDGELANEIVRERCIKFLSSKIQQLGREIINKEAEELIITECKKILEDVVAEEFEHIMELLTWSRLGKTPLGKKELVQIVAALAFSPDDWHPEDPEYIDRLIQCTQHAVPLFSPQVDSTQFINFFCDHVLPKWKDIVVADGASDSKLELLKIFAEITEYSGDLENAQQRIDTVFEVLMDYLPEAPVEDETQKTKEDKVEETKTAPSLQFSHVECALFALHSLCRKSPNSLTSDAAKLKMLRLRLQYTARLTQGYIKKLKEVTQSQKTEDENSEESKLKVAALKTTSNINTLIRDIFRTPPSFKSKVQLSFKSKKSEKEEKQTASTSDATKDSPQKRHRPITFDNGAEKESPEKRSRSSDRNLKMYTPPSGKYSSRINSSRFSGSSGRGRGYGRRDYRNNGASFRNRSNY

>303

MSADNIEKLYQNYGILADAKDDISKHEKEYLEILAAVKGSDKEKRLASQFIAKFFNSFPTLADQAIEAQFDLCEDDDVAIRKQAIKDLPTLCKDHKEHTQRIADILAQLLQSDDTTEINVVSNSLLTILKIDPKGALTGMFSQIHQNADSEVTNEIVRERCIKFLATKVKQLGREVITKDAEELIIAECKKLLELQDVVAEEFEHIMELLTWSKLGKTPAGKKELVQIVAALAFTPDDWHPEDPEYVDRVIHCSQHALPLFSALVDSTQFVNFFCDHVLQGWNSITTPEGGTDTKLELLKIFAEITEHCGELENPQQKIDAVYDLLMKYLPEAPIEPEGTEGEKSEEKIEDSKTTPSLQFSHVECALFALHSLCRKAPDALVSDATKLKALRLRLQYTARLTQGYIKKLKEVTQSQKGEDANSEENKLKIAALKTTSNINTLIRDLFRTPPSFKSKVQLSFQSKKSEKEEKHTADPDDTKQSPVQKRHRPITFDNENDKESPEKRSRSGDRNIKMYTPPSGKYSSRLNNNGRFSGNNSGGRGGRGGYGRRDFRNNGAPFRRRNNY

>304

MAVTIEELYRNYGILADAKDNLSQHKDAYQGILDGVKGGPKEKRLAAQFIPKFFSSFPELADAAINAQLDLCEDEDVSIRRQAIKELPRFATGENIFRVADILTQLLQTDDTAEFNQVNAALISIFKMDAKGTLGGLFSQILQGEDIVRERAIKFLSTKLKTLPEDVMTKEVEEYVFTETKKVMEDVTGEEFVLLMRVVSGLRVLQSVNGRQQLVELVVEQADLEQALNPADPDVVDRLLQCTRQALPLFSKNVHSTRFVTYFCEHVLPNLSTLTSPVAELDIQLEVLKLLAEMSPFCGDMEKLEANLNTLFTKLVEFMPLPPEEVENGENSAGEEPKLQFSYVECLLFSFHQLGKKLPDFLVDKVNAERLKDFKIRLQYFARGLQVYIRQLRVALQGKTGDALKTEENKIKVVALKITNNINVLIKDLFHNPPSYKSTVTLSWKPVQKPDAAAPKRPSGEEVASGAVAKKQITPLPRRDARQIYNPPSGKYSASIGNFNYEQRGGFRGGRGRGFGARGSRSRGRIY

>305

MPLYDPAVCVLPVPALERDRGFECLRNHPSSYWRGYGEKRSGVAAGGSYGLGRLRRGEREGVPPEGLFALPDKQLAPGAVLGHKDAYQAILDGVKGGAKEKRLAAQFIPKFFKHFPELADSAINAQLDLCEDEDVSIRRQAIKELPQFATGDNLPRVADILTQLLQSDDSAEFNLVNNALLSIFKMDAKGTLGGLFSQILQGEDIVRERAIKFLSTKLKTLPEEVMTKEVEEFILTESKKVLEDVTGEEFVLFMKILSGLKSLQTVSGRQQLVELVAEQADLEQTFNPSDPDCVDRLLQCTRQAVPLFSKNVHSTKFVTYFCEHVLPNLSSLTTPVEGLDIQLEVLKLLAEMSSFCGDMEKLESNLKKLFDKLLEYMPLPPEEAENGENAGNEEPKLQFSYVECLLYSFHQLGRKLPDFLTAKLNAEKLKDFKIRLQYFARGLQVYIRQLRLALQGKTGDALKTEENKIKVVALKITNNINVLIKDLFHIPPSYKSTVTLSWKPVQKADASQKRASEDTTSSSPPKKASAGPKRDARQIYNPPSGKYSSNLGSFSYEQRGGFRGGRGRGWGGRGNRSRGRIY

>306

MDKIDKLYQNYDILADSKNKPSEHEAEYLEIIESVKGKTNEKMLACQFIPRFLKEFPDLASAALDAQLDLVEDEDVSIRKHAVKHLPAFCKESKACVAKISDILAQMLQTEDSAELATVQNTLMTIMKVDPKATLDGIFGQIGSTDEDVIRKRAIQFLCTKFKFIPPDIATKDVEDFVLEKCKKVFPELGGEDFLNLMPLLANLKIAKSVPVQQALVNLIADQAEMDGEIQVSLDYIAGFLQCVRLALPYFSPFVHSTQFVSYICLSVIPKLGEIELLDNGAELSLDIIKLLAEISTFVHEDKQLSECIDAVYKIVIEYVPLPPPADAENGTQPDEPNLQFTHIECWLYIFLHLLKHVPDFLAGADNVQRSRDLKTRLQFLARSVQSGVKTFREALVTVKGKEAKPEETKLKAIALRTMNNINSLIKDLFRTPPSFKTVMTLSWKPVSSTSTRTSIVSPSVKRPSEGESTDDRKQIKRDGKAGRELYHPPSGKYSTNLNSFSRAQRGGSNYYRGRGRRFY

>307

MAVTIEDLYRSYGVLADAKDNLSQHKDAYQVILNGVKGGPKEKRLAAQFIPKFFSQFPELSDAAINAQLDLCEDEDVSIRRQAIKELPRFATGENIPRVADILTQLLQTDDSAEFNQVNAALLSIFKVDAKGTLGGLFTQILQGEDIVRERAIKFLSTKLKTLPEDVMTKEVEEFVFSETKKVLEDVTGEEFVLLMRVVSALRVLQTVNGRQQLVELVVEQAFLEQALNPSDPDTVDRLLQCTRQALPLFSKNVHSTRFVTYFCEHVLPNLSALTSPVAGIDIQLEVLKLLAEMSPFCGDMEKLEANLHTLFNKLLEFMPLPPAEESEDGENSSSDEPKLQFSSVECLLYSFHQLGKKLPDFLLDKVDQERLKDFKIRLQYFARGLQVYIRQLRVALQGKTGDALKTEENKIKVVALKITNNINVLIKDLFHNPPSYKSSVTLSWKPVQKTEAAAATAPKRSAPEETGASAVKKSAPPAPRRDARQIYNPPSGKYSGTIGNFTYEQRGGFRGGRGRGFGNRGNRSRGRMY

>308

MWKFDNKFIMSMDSIEKLYKNFGILADAKDKLAEHEKEYLEILTAVKGSPKEKRLASQFIARFFKYFPKLADQAIDAHLDLCEDEDMAIRKQAIKDLPALCKDNKEHTARIADILAQLLQAQDPSELAVVHNSVMSLMKTDPRGTISGFFSQIINGDDGTRERCIKFLATKLKAIGHDIITKEPEDLLISECKKVLQDVTADEFHSIMEVLAWTRLGSTISGQQELVDITVEQAELSEPFKHTNVEQWNRLVQCIKHALPFFSSQIDSSRFVSYICVQVLPHLSLITSPNGRDVQLELLKLLAELAVFCGTIDKPEEKVQQLYNTLITYMPLPPVTEITEVPKLQFSHVECLMYTFHKLCKQTPEFLMKDPEQLKEFRLRLQYFARGIQGYIKKLREAISGKSEEELKSEENQLKVVALKTTNNINSLIKDLFHSPPSFKSIIHLSWKTTANDKKNEKHSSAQKRHTPITFGNDSNANKRSKEDKSKRELYTPPSGKYSSNISSNYGRGRFRGNRSGGRGGYRPRGRGTWRKNFY

>309

MPTVEELYRNYGILADAKETAAEHKDAYQVILDGVKGGAKEKRLAAQFIPKFFKYFPELADSAINAQLDLCEDEDVSIRRQAIKELPQFATGDNLPRVADILTQLLQSDDSAEFNLVNNALLSIFKMDAKGTLGGLFSQILQGEDIVRERAIKFLSTKLRTLQEEVLTKEVEELILTESKKVLEDVTGEEFVLFMKILSGLKSLQTVSGRQQLVELVAEQADLEQTFNPSDPDCVDRLLQCTRQAVPLFSKNVHSTRFVTYFCEHVLPNLSSLTTPVEGLDIQLEVLKLLAEMSSFCGDMEKLESNLKKLFDKLLEYMPLPPEEAENGENAGNEEPKLQFSYVECLLYSFHQLGRKLPDFLTTKLNTEKVKDFKIRLQYFARGLQVYIRQLRLALQGKTGEALKTEENKIKVVALKITNNINVLIKDLFHIPPSYKSTVTLSWKPVQKSDVGQKRASDDAPSDLPAKKAAAGPKRDARQIYNPPSGKYSSNLGNFSYEQRGSFRGGRGRGWGGRGNRSRGRIY

>310

MAVTIEELYRNYGILADAKDNLSQHKDAYQVILDGVKGGPKEKRLAAQFIPKFFCSFPELADAAINAQLDLCEDEDVSIRRQAIKELPRFATGENILRVADILTQLLQTDDTAEFNQVNVALISIFKMDAKGTLGGLFSQILQGEDIVRERAIKFLSTKLKTLPEDIMTKEVEEYIFTETKKVLEDVTGEEFVLLMRVVSGLRVLQTVNGRQQLVELVVEQAFLEQALNPADPDTVDRLLQCTRQALPLFSKNVHSTRFVTYFCEHVLPNLSTLTSPVADLDIQLEVLKLLAEMSPFCGDMEKLEANLNMLFTKLLEFMPLPPEEVENGENSASEEPKLQFSYVECLLFSFHQLGKKLPDFLLDKVDAEHLKDFKIRLQYFARGLQVYIRQLRVALQGKTGDALKTEENKIKVVALKITNNINVLIKDLFHNPPSYKSTVTLSWKPVQKTEAAAPKRPSGEEMGSGGSTKKQISPLPRRDTRQIYNPPSGKYSASIGNFSYERGGFRGGRGRGFGARGNRSRGRIY

>311

MAVTIEDLYRSYGILADAKEDLSQHKDAYQVILDGVKGGAKEKRLAAQFIPKFFGNFPELADAAINAQLDLCEDEDVSIRRQAIKELPRFATGDNILRVADILTQLLQTDDTAEFNQVNVALISIFKMDARGTLGGLFSQVLQGEDIVRERAIKFLSAKLKTLPEDVMTKEVEEYVFAETKKVLEDVTGEEFVLLMRVVSGLRILQTVNGRQQLVELVVEQAFLEQALNPADPDTVDRLLQCTRQALPLFSKNVHSTRFVTYFCEHVLPNLSALTSPVAELDIQLEVLKLLAEMSPFCGDMEKLESNLNMLFTKLLEYMPLPPEEVENGENPANEEPKLQFSYVECLLFGFHQLGKKLPDFLIDKVDAERLKDFKIRLQYFARGLQVYIRQLRVALQGKSGDALKTDENKIKVVALKITNNINILIKDLFHNPPSFKSTVTLSWKPVQKAEAAAAAAAAPKRPSGEEMGSGGSTKKQISPQHRRDARQIYNPPSGKYSATIGNFSYERGGFRGGRGRGFGARGNRSRGRLY

>312

MDSRDMELQMPPTAPFILLGRVFKKGPELCLAQPQIHLESGCFLHGGGEFRLANSGLEVFDTSGHPDLIELEENGRKKSFLVLSITQDFSIYVLFFQHKDAYQVILDGVKGGAKEKRLAAQFIPKFFKHFPDLADSAINAQLDLCEDEDVSIRRQAIKELPQFATGDNLPRVADILTQLLQSDDSAEFNLVNNALLSIFKMDAKGTLGGLFSQILQGEDIVRERAIKFLSTKLKTLPEEVMTKEVEEFILTESKKVLEDVTGEEFVLFMKILSGLKSLQTVSGRQQLVELVAEQADLEQTFNPSDTDCVDRLLQCTRQAVPLFSKNVHSTKFVTYFCEHVLPNLSSLTTLVEGLDIQLEVLKLLAEMSSFCGDMEKLESNLKKLFDKLLEYMPLPPEEAENGENAGNEEPKLQFSYVECLLYSFHQLGRKLPDFLTAKLNAEKLKDFKIRLQYFARGLQVYIRQLRLALQGKTGEALKTEENKIKVVALKITNNINVLIKDLFHIPPSYKSTVTLSWKPVQKADASQKRASEDTTSSSPPKKASAGPKRDARQIYNPPSGKYSSNLGSFSYEQRGGFRGGRGRGWGGRGNRSRGRIY

>313

YKDAYQAILDGVKSGAKEKRLAAQFIPKFFKHFPELADSAINAQLDLCEDEDLSIRRQAIKELPQFATGENLPRVADILTQLLQTDDSAEFNLVNNALICIFKIDAKGTLGGLFTQIIQGEDIVRERAIKFLSTKLKIVPEEVMTKEVEEYIFVESKKVLDDVTGEEFVLFMKILSSLKNLQTVSGRQQLVELVAEQADLEQVFNPADPDSVDRLLQCTRQAVPLFSKNVHSTRFVTYFCEQVLPNLSSLTSPVEGLDIQLEVLKLLGEMTPFCGDMEKLESNLKQLFEKLLEYMPLPPEEAENGENAGNEEPKLQFSYVECLLFGFHQLGKKLPDFLTAKLNADRLKDFKIRLQYFARGLQVYIRQLRLALQGKTGEALKTEENKIKVVALKITNNINVLIKDLFHNPPSYKSTVTLSWKPVQKVEVGQKRMAEENTSSPPKKQSPAQKRDTRQIYNPPSGKYSSNLGNFSYEQRGGFRGGRGRGWGGRGNRSRGRLY

>314

MPTVEELYRNYGILADATETAGQHKDAYQAILDGVKGGAKEKRLAAQFIPKFFKHFPELADSAINAQLDLCEDEDVSIRRQAIKELPQFATGDNLPRVADILTQLLQSDDSAEFNLVNNALLSIFKMDAKGTLGGLFSQILQGEDIVRERAIKFLSTKLKTLPEEVMTKEVEEFILTESKKVLEDVTGEEFVLFMKILSGLKSLQTVSGRQQLVELVAEQADLEQMFNPSDPDCVDRLLQCTRQAVPLFSKNVHSTKFVTYFCEHVLPNLSSLTTPVEGLDIQLEVLKLLAEMSSFCGDMEKLESNLKKLFDKLLEYMPLPPEEAENGENAGNEEPKLQFSYVECLLYSFHQLGRKLPDFLTAKLNAEKLKDFKIRLQYFARGLQVYIRQLRLALQGKTGDALKTEENKIKVVALKITNNINVLIKDLFHIPPSYKSTVTLSWKPVQKADASQKRASEDTTSSSPPKKASAGPKRDARQIYNPPSGKYSSNLGSFSYEQRGGFRGGRGRGWGGRGNRSRGRIY

>315

MSSDSIEKLYKNFGILADAKDKLVQHEKEYLEILTAVKGSPKEKRLASQFIARFFKHFPKLADKAMDAHLDLCEDEDMAIRKQAIKDLPTLCKDNKEHTARIADILAQLLQAEDTSELAVVHNSIMSLMKSDPKGTLSGFFSQIINGDDGTRERCIKFLATKLKAIGHDIITKEPEDFLIAECKKVLQDVTADEFHSIMEILAWTRLGSTVAGQQELVDITIEQAELTIPFKHTNVEQWNRLVQCIKHALPFFSSQIDSSKFVSYICVQVLPHLSLMTSPDGRDIQLELLKLLAELAVFCGNIEKPEEKVQQIYNTLITYMPLPPATEVTDVPKLQFSHVECLMYAFHKLCKQTPEFLVKDAEQLKEFRLRLQYFARGIQGYIKKLREAISGKTEEELKSEENKLKVVALKTTNNINILIKDLFHSPPSFKSIIHLSWKTPCSDKKTERTTTQKRHTPITFGNGSSPNKRNKEDKSNKREIYTPPSGKYSSNISNYGRGRFKGNRIGGRGGGFRSGGRGRGPWRKNFY

>316

MEERELQVLVRRAKATSDKTSTSRRSALKQLVEATRSLNVSLKIFAAKNIPDFFQDFPEAEEDAINAVYDLCEDQMSPVRMAGYNALVQMSRLEKKWVKRNADVLVQLLQSDDPNEVTMVRKALVDHLQFDSRVTLGVLCDQVVPPDDLADPEELEMRNSLRTLVLSFVIGELKKGQLIRYMAPGSEAEDTLVNGLISALPKLGETDTQVILKDILMQLQFLDTPCPRGTTLSQSVLQRAKSALFDDHMNLDAQNGTRSLNKTRPYLDMLSVLFISKRQGNLKDLFNFYTPILGKTVLSSISIEDQLIILRHFAEALHTCKTNPPSVIRLLNLTPFFFECLSKANLTQVSPQKVCVLLLRGLLLVADNVYSCQKARNVFKTISDIKNCKRPVCSLN

>317

MDAIETMYQNFGVLADAKEKAGEHEEEFLSILNAVKGSPGEKRLASQFITRFFKYFPSHAGTAINAMFDLCEDDDPMTRKQAIKDLPTLCKSAPEQVSKIASALTQLMGTDDATENHLIQSSFLTLFKFDSKGSLDGIFSQILGEDETVREKAIKFLGAKIKTLPEDTLDSHSEELLINMCKKVLEDVTKDEFIAIMEILKCQKSMTTVQGRQQLVDIVTEQAELDEPFVASDPDCVDRLTQCIKHAAPLFSKNVHSKAFVGYLCDNVLPHIDQLASPEDGVNGKLELLKLMAEISEFTGELEDLEQRLKNLYDCLISYMPLPPEEENEDESASGEPKLAFSSVECLMYAFHQLCRKQPSFLAAEENAERLKDFKIRLQYFARGVQVYIKHLRMALQGKTGTELKEEENKMKVAALKITSNINTMIKDLFHSPPSFKSVITLSWKPLVKPVEKVTPTKTVAGQKRTPITFETNGAGGKKPHKQDRSIYHPPSGKFSEKAGTFESNETRSQGQRGRGGGGGYRRGGWRGRGRGQRW

>318

MAVTIEELYRNYGILADAKPEDLSKHKDAYQVILDGVKGGPKEKRLAAQFIPKFFSSFPDLADAAINAQLDLCEDEDVSIRRQAIKELPRFAAGENIVRVADILTQLLQTDDSAEFNQVNTALVSIFKMDAKATLGGLFSQILQGEDIVRERAIKFLSIKLKTLPEDTMTKEVEDYVFTETKKVLEDVTGEEFVLLMRILMALKSLQTVSGRQQLVELVVEQAFLEQALNPADPDTVDRLLQCTRQALPLFSKNVHSTRFVTYFCDHVLPNLSSLTSPVAELDIQLEVLKLLAEMSPFCGDMVKVEANLTMLFEKLLEFMPLPPEAEGENGENTISEEPKLQFSYVECLLFSFHQLGKKLPDFLIDKINAERLKDFKIRLQYFARGLQVYIRQLRVALQGKTGDALKTEENKIKVVALKITNNINVLIKDLFHNPPSYKSTVTLSWKPVQKTEATAPKRPSGEEMGAGSTMKKQLSPLPRRDARQIYNPPSGKYSATIGNFSNEQRGGFRGGKGRGFGGRGGRSRGRIY

>319

MAIARIFCSGPHYYFDCEHDGQTGRSVREKYGKLAEAKDVSQLSDHYVSCIEGTKCGDKEKQLAAQIISKFFKHFPSLQDKALNAVLDLCEDDNSQVSNLCAMKQLPIISKDSKEHLSKIADILAQLLQLEDQDYAVACNCLVQVYKQDPLLVTKIIFNSMHSTTDYNLREKCISFLFQKLIKIPEKMSQELEEVLIEESKKSILESSPTEFSVLMSYLKTSKCGKTVQGTYEVVNLVADKAELDKDFDPLDEESNDTARVILCVESVLPFFNASVHSDKFLIYYCDQILPQLNNIIKVNDGEAIKIRLLRQLAELSFHCGKLENPSLHVVQIFDKLKDYMPPPPAEIVEMPNLDFTSVECLLYSFHRLARQCPDFLTADPSVLKDFRSRLTYFSRGVQGCKKSLEAVIGSKVPLANENAVKAKLAPSVLNNINTLIKDLFYTPPVYKCNVTLSFKGEVTEMKATVEKSVTAQKRHTPITFDASNGNTKHPRNKTSENIKLYTPPSGKFSNNFQSYDRSGGRPRGGNRGRGGRGRGTWRK

>320

MADELQEIYEKYEILSGAKDKILEHSAEYMECINIASKGGPKGKKLVAQIISQFFKHFPDLQDTALNVLLDLCEDGDSEIRICSMKVLPFLCKGSKEHIQNIASILAQLLQLDDLDYTVACNSLVQVFREDPVLSINAIFTILYQDEALREKLVTFLYKRLATIEGKNLPEVEELLVTEGKKIIQDCTSGQFMLIMPYLTQSKLGKTLHGQKELVNLVYNKIEVEGKFNLLETHTLSTDRLVLCVELILPLFNASNESTKLLIYYCTQVLPQWNNIRSLKDGEQLQLRLLRQLAIMSTYCVWNAENMTDVIENVFNTLKEYMPLPPEDVDISKVPNLDFTSVECLLYTFHKLARKNPEFLTNDSDRLKDFRLRLQYFARGVQGCKRGLENTKTKKDELSEEDQKKIQIAPAVLENINAIVKDLFYQPPLYKCNVQLSFRSARDGKKEVTTVTKSGAQKRHVPITFGSANGTDPGPKQVRNNKAGEDRKLYQPPSGKFSNNFGRTNSRGGSTRGGRGGGRNWRN

>321

MAVTIEDLYRSYGVLADAKDDVSQHKDAYQVILDGVKGGPKEKRLAAQFIPKFFSSFPELSDAAINAQLDLCEDDDVSIRRQAIKELPRFATGDNILKVADILTQLLQTDDTAEFNQVNISLISIFKIDAKGTLGGLFSQILNGEDIVRERAIKFLSTKLKTLPEDVMTKEVEEYVFTETKKVLEDVTGEEFVLLMRLVSNLRVLQTVSGRQQLVELVVEQAFLEQALNPADPDTVDRLLQCTRQALPLFSKNVHSTRFVTYFCEHVLPNLSMLTTPVAELDIQLEVLKLLAEMSPFCGDMDKLETNLNMLFTKLLEYMPLPPEEVENGEQTAGEEPKLQFSYVECLLFSFHQLGKKLPDFLIDKVDAERLKDFKIRLQYFARGLQVYIRQLRVALQGKTGQALKTDENKIKVVALKITNNINVLIKDLFHNPPSFKSAVTLSWKPVQKTEAAAVALKRPSGDDAGTGVTLKKQMAPQPRRDARQIYNPPSGKYSASLGNLSYEQRGGFRGGRGRGFGTRGNRSRGRIY

>322

MGTAGFRLPGSGLTGDFGSPAGMKRKEKTLSFLKNTPRFFLFAYSVFFFFQHKDAYQVILDGVKGGAKEKRLAAQFIPKFFKHFPELADSAINAQLDLCEDEDVSIRRQAIKELPQFATGDNLPRVADILTQLLQSDDSAEFNLVNNALLSIFKMDAKGTLGGLFSQILQGEDIVRERAIKFLSTKLKTLPEEVLNKEVEEFILAESKKVLEDVTGEEFVLFMKILSGLKSLQTVSGRQQLVELVAEQADLEQTFNPADPDCVDRLLQCTRQAVPLFSKNVHSTKFVTYFCEHVLPNLSALTTPVEGLDIQLEVLKLLAEMSSFCGDMEKLESNLKKLFDKLLEYMPLPPEEAENGENAGGEEPKLQFSYVECLLYSFHQLGRKLPDFLTAKLNAEKLKDFKIRLQYFARGLQVYIRQLRLALQGKTGEALKTEENKIKVVALKITNNINVLIKDLFHIPPSYKSTVTLSWKPVQKADANQKRTSEDTTSSSPPKKASAGPKRDARQIYNPPSGKYSSNLGSFSYEQRGGFRGGRGRGWGGRGNRSRGRIY

>323

MPTVEELYRNYGILADATETAGQHKDAYQVILDGVKGGAKEKRLAAQFIPKFFKHFPELADSAINAQLDLCEDEDVSIRRQAIKELPQFATGDNLPRVADILTQLLQSDDSAEFNLVNNALLSIFKMDAKGTLGGLFSQILQGEDIVRERAIKFLSTKLKTLPEEVLTKEVEEFILTESKKVLEDVTGEEFVLFMKILSGLKSLQTVSGRQQLVELVAEQADLEQTFNPSDPDCVDRLLQCTRQAVPLFSKNVHSTRFVTYFCEHVLPNLSSLTTPVEGLDIQLEVLKLLAEMSSFCGDMEKLESNLKKLFDKLLEYMPLPPEEAENGENAGNEEPKLQFSYVECLLYSFHQLGRKLPDFLTAKLNAEKLKDFKIRLQYFARGLQVYIRQLRLALQGKTGEALKTEENKIKVVALKITNNINVLIKDLFHIPPSYKSTVTLSWKPVQKADAGQKRASEDTTSSSPPKKAAAGPKRDARQIYNPPSGKYSSNLGSFSYEQRGGFRGGRGRGWGGRGNRSRGRIY

>324

MSRQKAETLWKNCPSSVKHKDAYQAILDGVKGGAKEKRLAAQFIPKFFKHFPELADSAINAQLDLCEDEDVSIRRQAIKELPQFATGDNLPRVADILTQLLQSDDSAEFNLVNNALLSIFKMDAKGTLGGLFSQILQGEDIVRERAIKFLSTKLKTLPDEVMTKEVEEFILTESKKVLEDVTGEEFVLFMKILSGLKSLQTVSGRQQLVELVAEQADLEQTFNPSDPDCVDRLLQCTRQAVPLFSKNVHSTKFVTYFCEHVLPNLSSLTTPVEGLDIQLEVLKLLAEMSSFCGDMEKLESNLKKLFDKLLEYMPLPPEEAENGENAGNEEPKLQFSYVECLLYSFHQLGRKLPDFLTAKLNAEKLKDFKIRLQYFARGLQVYIRQLRLALQGKTGEALKTEENKIKVVALKITNNINVLIKDLFHIPPSYKSTVTLSWKPVQKADASQKRASEDTTSSSPPKKASAGPKRDARQIYNPPSGKYSSNLGSFSYEQRGGFRGGRGRGWGGRGNRSRGRIY

>325

MPSVEELYRNYGILADATETAGQHKDAYQVILDGVKGGAKEKRLAAQFIPKFFKHFPELADSAINAQLDLCEDEDVSIRRQAIKELPQFATGDNLPRVADILTQLLQSDDSAEFNLVNNALLSIFKMDAKGTLGGLFSQILQGEDIVRERAIKFLSTKLKTLPEEVMTKEVEEFILTESKKVLEDVTGEEFVLFMKILSGLKSLQTVSGRQQLVELVAEQADLEQTFNPSDTDCVDRLLQCTRQAVPLFSKNVHSTKFVTYFCEHVLPNLSSLTTLVEGLDIQLEVLKLLAEMSSFCGDMEKLESNLKKLFDKLLEYMPLPPEEAENGENAGNEEPKLQFSYVECLLYSFHQLGRKLPDFLTAKLNAEKLKDFKIRLQYFARGLQVYIRQLRLALQGKTGEALKTEENKIKVVALKITNNINVLIKDLFHIPPSYKSTVTLSWKPVQKTDVSQKRASEDTTSSSPPKKASAGPKRDARQIYNPPSGKYSSNLGSFSYEQRGGFRGGRGRGWGGRGNRSRGRIY

>326

MSTDSIEKLYKNFGVLADAKDKIGEHEAEYLEILTAVKGTSNEKRLASQFIARFFKHFPNLVDQALEAQFDLCEDEDISIRKQAIKDLPSLCKDNKEQVPKIADILAQLLQAEDPTERSVVQSSFMTLFKVDAKSAVTGLFNQILTGDDTVREQCIKFLYSKAKTLGADFIPKEIEDELIALCKKVLQDCTADEFVMLMELLGGTKLGKTVSGHKEIIDIISEQAEIGDENFNPQDEEQVDKLVHSIRHALPYFSSQLNSSRFVEYICNHVLPQLDKISPSKEGPDSKLELLKLFAELCTHSPTLPNIETNLEKVFAALTNFMPLPPVEEETISEPKIEFSHVECLIYAFHRLSRQSPDFLTKDAERMRNFKIRLQYLARGTQGYLKKLKESVEGKSSEDLKSEECKIKVLALKTTNNINTIIKDLFHVPPSFKTNVNLSWRPTVSTTKSSAATTNGTAVKRHKPITFDSSPSAKLAKTDAKVYTPPSGKFSNTVSSYSPAQSNFRSGRRPRGRAPVRRGSSSGNSFRLRPSRGRLPSKNWRY

>327

MPTVEELYRNYGILADATETAGQHKDAYQVILDGVKGGAKEKRLAAQFIPKFFKHFPELADSAINAQLDLCEDEDVSIRRQAIKELPQFATGDNLPRVADILTQLLQSDDSAEFNLVNNALLSIFKMDAKGTLGGLFSQILQGEDIVRERAIKFLSTKLKTLPEEVMTKEVEEFILTESKKVLEDVTGEEFVLFMKILSGLKSLQTVSGRQQLVELVAEQADLEQTFNPSDTDCVDRLLQCTRQAVPLFSKNVHSTKFVTYFCEHVLPNLSSLTTLVEGLDIQLEVLKLLAEMSSFCGDMEKLESNLKKLFDKLLEYMPLPPEEAENGENAGNEEPKLQFSYVECLLYSFHQLGRKLPDFLTAKLNAEKLKDFKIRLQYFARGLQVYIRQLRLALQGKTGEALKTEENKIKVVALKITNNINVLIKDLFHIPPSYKSTVTLSWKPVQKADASQKRASEDTTSSSPPKKASAGPKRDARQIYNPPSGKYSSNLGSFSYEQRGGFRGGRGRGWGGRGNRSRGRIY

>328

MAVTVQELYRNYGILADAKDDLSQHKDAYQAILDGVKGGPKEKCLAAQFIPKFFSHFPELADAAINAQLDLCEDEDVSIRRQAIKELPRFAAGENLPRVADILTQLLQTDDSAEFNQVNTALISIFKIDAKGTLGGLFTQILQGEDIVRERAIKFLSTKLKTLPEEVMTKEVEDFIFTEIKKVLEDVTGEEFVLLMRILAGLKSLQTVSGRQQLVELVVEQAFLEQALNPADADSVDRLLQCTRQALPLFSKNVHSTRFVTYFCEHVLPNLSMLTSPVAELDIQLEVLKLLAEMSRFCGDMEKLETNLKILFEKLLEFMPLPPGEGENGEHSGNEEPRLQFSYVECLLFSFHQLGKKLPDFLIDKINTERLKDFKIRLQYFARGLQVYIRQLRVALQGKTGDALKTEENKIKVVALKITNNINVLIKDLFHNPPSYKSTITLSWKPVQKMDVVGQKRPSGEEVAPGSAMKKVSPLPRRDARQIYNPPSGKYSATIGNFSYEQRGGFRGGRGRGWGGRGNRSRGRIY

>329

MTNDDNHDLENRFDELARAVRQRECWNKYDLYGSFVDRAAVGIGTPKERRLTSVLMRRYSIYFPKSKEASLEAEIDLCNDADAEVRKQAFHDIPHFCTSEGNTARVVVALHHHLLSESKAEVLIVERALCVLLQRSPEEAIRAVFLDMERSMFTAAGKAFVRFLCVKLRPQARGGRIESALFTGFADVSDRLPFDYFVEMFQLISALRIVKTTGGRGIIVGTIIRFLRSSPESVSWCQLKTCLTLPMPHFSSVAPSTELIKLVAVDFLPNLDAKEPESLEILDLFNQMARFVEEQHEQDDTTRDVLGKVLGCIERFLSQVSKESVTGEALGPPSLPLEHIQLLLRSFATIAQRAKATVTEEDESFRSVREKLAYLNEIVQKERSMLAIVICRRKGVSSRGEARSRTLSKLAALIESWLREPLDFEPRELASPVFVDEPPASDHDYSQGTSSSTAESSPEKRPTNTRKKVIQLQGEIRILESLLEKERQRADSLLLENAELRRNR

>330

MANRLDELYGFYDELVEAEKEGKLLEKTSVYEKIISRALTGDSKEKRLVSQFIAKFFKHFPELEIRAVEAQIELCDDADINVRKQAIHDLPSFCARGHDDPYISRIAIVLCQLLIVEEKGELAIVEKSLNTLLRQSVHATVKGVFGQMTSSVCDDSMRDACIRFLCSKVNLKVLEKETEQMMLEEAVKVATLLGGEEFVLLLKLLSALKISKLVVNQQVILKMIADQLQLSSNPAFDAEHIVKLKTCLPLAIPFFTPFTPSQPYVTHLVDGFLPKMDVQSPDSLDLLKILAQISPHYRHLTDEDNADLKLEFESVLKSLFERIQEFLPQVPADDDLEKDPELPSLHLSHLECLLLAFNNIARKVDPAFTKDEALLKSIRQRLQYLNRGLQGEAKKLRGLISQGVKIDADKRAAMQLALKTIANLTAFIKDWFHNPPAFKTTVAPSWLQNESAKKPSASAQANGSNQENKRKAIVFENNDAPAKKFGRDSRKLYEPPKGNSGRGRFRSGRW

>331

MPTVEELYRNYGILADAKDDVSKHRAAYQVILDGVKGEAKEKRLAAQFIPKFFKHFPELADAAINAQLDLCEDEDVSIRRQAIKELSQFATGENLPRVADILAQLLQSDDSAEFNLVNNALLSIFKIDAKGTLGGLFSQVLHGEDIVRERAIKFLSTKLKTIPEDVMTKEVDDYIFSESKKVLDDVTGEEFVLFMKILSSLKSLQTVSGRQQLVDLVSEQAGLYQTLNPADPDSVDRLLQCMRQAVPLFSKNVHSTKFMTYFCEQVLPILSSLTSPAESIDVQLEILKLLAEMSSFCGDMDKLESNLNKLFEKLLEYMPLPPEEVENGENSSNEEPKLQFSYVECLLFSFHQLGRKNPDFLTGKEVNAEKLKDFKIRLQYFARGLQVYIRQLRLALQGKSGDALKTEENKIKVVALKITNNINVLIKDLFHNPPSYKSTVTLSWKPVQKTDIGQKRTTEDTTSSSSSPPKKPIMGPKRDARQIYNPPSGKYSGSLGNFSYEQRGGFRGGRGRGWGGTRGNRSRGRMY

>332

MSTDSIEKLYRNFGILADAKDKLVQHEKEYLEILTAVKGSPKEKRLASQFIARFFKHFPKLADQAIDAHLDLCEDEDMAIRKQAIKDLPALCKDNKEHTARIADILAQLLQAEDSSELAVVHNSIMSLMKSDPKGTLSGFFSQIISGDDGTRERCIKFLAIKLKAIGHDIITKEPEDLLIVECKKVLQDVTADEFHSIMEILAWTRLGSTITGQQELVDITIEQAELSVPFKHTNVEQWNRLVQCVKHAVPFFSSQIDSSKFVSYICVQVLPHLSLMTSPNSRDIQLELLKLLAELTVFCGTIEKPEDKVQQLYNTLITYMPLPPATEITDVPKLQFSHVECLMYAFHKLCKQTPEFLMKDAEQLKEFRLRLQYFARGIQGYIKKLREAISGKTEEELKSEENQLKVVALKTTNNINTLIKDLFHSPPSFKSIIHLSWKTPSNDKKSDKNSAQKRHTPITFGNDNSPNKRSKEDKNSKREIYTPPSGKYSSTISNYGRGRFKGNRSRGRGGFRTRGRGSWRKNFY

>333

MSTDSIEKLYRNFGILADAKDKLVQHEKEYLEILTAVKGSPKEKRLASQFIARFFKHFPKLADQAIDAHLDLCEDEDMAIRKQAIKDLPALCKDNKEHTARIADILAQLLQAEDSSELAVVHNSIMSLMKSDPKGTLSGFFSQIISGDDGTRERCIKFLAIKLKAIGHDIITKEPEDLLIVECKKVLQDVTADEFHSIMEILAWTRLGSTITGQQELVDITIEQAELSVPFKHTNVEQWNRLVQCVKHALPFFSSQIDSSKFVSYICVQVLPHLSLMTSPDGRDIQLELLKLLAELTVFCGTIEKPEDKVQQLYNTLITYMPLPPATEITDVPKLQFSHVECLMYAFHKLCKQTPEFLIKDAEQLKEFRLRLQYFARGIQGYIKKLREAISGKTEEELKSEENQLKVVALKTTNNINTLIKDLFHSPPSFKSIIHLSWKTPCNDKKSDKNSAQKRHTPITFGNDNSPNKRSKEDKNSKREIYTPPSGKYSSTISNYGRGRFKGNRPRGRGGFRTRGRGSWRKNFY

>334

MSTDSIEKLYRNFGILADAKDKLVQHEKEYLEILTAVKGSPKEKRLASQFIARFFKHFPKLADQAIDAHLDLCEDEDMAIRKQAIKDLPALCKDNKEHTARIADILAQLLQAEDPSELAVVHNSIMSLIKSDPKGTLSGFFSQIISGDDGTRERCIKFLAIKLKAIGHDIVTKEPEDLLIVECKKVLQDVTADEFHSIMEILAWTRLGSTITGQQELVDITIEQAELSVPFKHTNVEQWNRLVQCVKHALPFFSSQIDSSKFVSYICVQVLPHLSLMTSPDGRDIQLELLKLLAELTVFCGTIEKPEDKVQQLYNTLITYMPLPPATEMTDVPKLQFSHVECLMYAFHKLCKQTPEFLIKDAEQLKEFRLRLQYFARGIQGYIKKLREAISGKTEEELKSEENQLKVVALKTTNNINTLIKDLFHSPPSFKSIIHLSWKTPCNDKKSDKNSAQKRHTPITFGNDNSPNKRSKEDKNSKREIYTPPSGKYSSTISNYGRGRFKGNRPRGRGGFRTRGRGSWRKNFY

>335

MSTDSIEKLYRNFGILADAKDKLVQHEKEYLEILTAVKGSPKEKRLASQFIARFFKHFPKLADQAIDAHLDLCEDEDMAIRKQAIKDLPALCKDNKEHTARIADILAQLLQAEDSSELAVVHNSIMSLMKSDPKGTLSGFFSQIISGDDGTRERCIKFLAIKLKAIGHDIITKEPEDLLIIECKKVLQDVTADEFHSIMEILAWTRLGSTITGQQELVDITIEQAELSVPFKHTNVEQWNRLVQCVKHALPFFSSQIDSSKFVSYICVQVLPHLSLMTSPDDRDIQLELLKLLAELTVFCGTIEKPEDKVQQLYNTLITYMPLPPATEITDVPKLQFSHVECLMYAFHKLCKQTPEFLIKDAEQLKEFRLRLQYFARGIQGYIKKLREAISGKTEEELKSEENQLKVVALKTTNNINTLIKDLFHSPPSFKSIIHLSWKTPCNDKKSDKNSVQKRHTPITFGNDNSSNKRSKEDKNSKREIYTPPSGKYSSTISNYGRGRFKGNRPRGRGGFRTRGRSSWRKNFY

>336

MSTDSIEKLYRNFGILADAKDKLVQHEKEYLEILTAVKGSPKEKRLASQFIARFFKHFPKLADQAIDAHLDLCEDEDMAIRKQAIKDLPALCKDNKEHTARIADILAQLLQAEDPSELAVVHNSIMSLMKSDPKGTLSGFFSQIISGDDGTRERCIKFLAIKLKAIGHDIITKEPEDLLIVECKKVLQDVTADEFHSIMEILAWTRLGSTITGQQELVDITIEQAELSVPFKHTNVEQWNRLVQCVKHALPFFSSQIDSSKFVSYICVQVLPHLSLMTSPDGRDIQLELLKLLAELTVFCGTIEKPEDKVQQLYNTLITYMPLPPATEITDVPKLQFSHVECLMYAFHKLCKQTPEFLIKDAEQLKEFRLRLQYFARGIQGYIKKLREAISGKTEEELKSEENQLKVVALKTTNNINTLIKDLFHSPPSFKSIIHLSWKTPCNDKKSDKNSAQKRHTPITFGNDNSPNKRSKEDKNSKREIYTPPSGKYSSTISNYGRGRFKGNRPRGRGGFRTRGRGSWRKNFY

>337

MPTVEELYRNYGILADAKDDVGQHKSAYQVILDGVKGGAKEKRLAAQFIPKFFKHFPELSDAALNAQLDLCEDEEVSIRRQAIKELSQFATGENLPRVADILTQLLQSDDSAEFNLVNNALLSIFKMDAKGTLGGLFSQILQGEDIVRERAIKFLATKMKTLPDEILTKEVDDYIFSESKKVLYDVTGEEFVLFMKILSALKNLQTVSGRQQLVDLVSEQAGLHQSLNPADPDSVDRLLQCMRQAVPLFSKNVHSTKFVTYFCEQVLPILSSLTSPAEGIDVQLEVLKLLAEMSSFCGDMDKLESNLNKLFDKLLEFMPLPPEEVENGDSAANEEPKLQFSYVECLLFSFHQLGRKLPDFLIAKVDAEKLKDFKIRLQYFARGLQVYIRQLRLALQGKSGDALKTEENKIKVVALKITNNINVLIKDLFHNPPSYKSTVTLSWKPVQKTDSGQKRTSDETSSSSPPKKPIVGPKRDSRQIYNPPSGKYSSNVGAFAYEQRGGFHGSRGRGWGGRGNRSRGRIY

>338

MWKFDKKLIMSMDSIEKLYKNFGILADAKDKLAEHEKEYLEILTAVKGSPKEKRLASQFIARFFKYFPKLADQAIDAHLDLCEDEDMAIRKQAIKDLPALCKDNKEHTARIADILAQLLQAQDPSELAVVHNSVMSLMKTDPRGAISGFFSQIINGDDGTRERCIKFLATKLKAIGHDVITKEPEDLLISECKKVLQDVTADEFHSIMEVLAWTRLGSTVNGQQELVDITVEQAELSEPFKHTNVEQWNRLVQCIKHALPFFSSQIDSSRFVSYICVQVLPHLSLITSPDGRDVQLELLKLLAELAVFCGIIDKPEEKVQQLYNTLITYMPLPPATEITEVPKLQFSHVECLMYAFHKLCKQTPEFLIKDPEQLKEFRLRLQYFARGIQGYIKKLREAISGKSEEELKSEENQLKVVALKTTNNINTLIKDLFHSPPSFKSVIHLSWKTTTNDKKNEKHSTTQKRHTPITFGNDSNSNKRNKEDKSKRELYTPPSGKYSSNISSNYGRGRFRGNRAGGRGGYRPRGRGTWRKNFY

>339

MNFAKLYSSYELLSEAKDNFTELGTEYKVAINATKGSEKEKQLAAQIISKFFKHFPDYQNDAVEAIFDLCEDDDMSIRIAAIKTLPSFCKESNKYNARVADISAQLLQLEDPQEHNIASSTLLQVLKDDPTTVLKSMFKLINDKTTELQLREKMLKFILTKVKTMDKAVFTAELEELLLTEVKSTLQDCTAEEYILLMNYLVSSKYCNTITAQQELIDIACEQIEMDQKFNPLEEDSNNTDKLITCVKFILPFFSAKIESSKFILFYCDQVLSQWETIGNLELGTQYQLAIAQQLAEMSMYCGNLETPSTQIVQIFDKLKMYMPYPSEDTSSTKMPEFNFSVVESLLFTFHRLARQSPDFLTCDQSVLKDFRSRLMYFSRGVQGCLKVLNLTDKEKIFSPEEKKVREMSPKLLNNINTLIKDLFYQPPIYKCNVSLSFKLETKKTNVTKSPEIGTKRHVPIFFESNGASNNKQSKQSRNIVGEKRIYTPPSGKFSHNFHNREGNNRFWGGRGGPSRGSRGRGRGAYRN

>340

MWKFDNKFIMSMDSIEKLYKNFGILADAKDKLAEHEKEYLEILTAVKGSPKEKRLASQFIARFFKYFPKLADQAIDAHLDLCEDEDMAIRKQAIKDLPALCKDNKEHTARIADILAQLLQAQDPSELAVVHNSVMSLMKSDPRGTISGFFSQIINGDDGTRERCIKFLATKLKAIGHDIITKEPEDLLISECKKVLQDVTASEFHSIMEVLAWTRLGSTVSGQQELVDITVEQAELSEPFKHTNVEQWNRLVQCIKHALPFFSSQIDSSRFVSYICMQVLPHFSLITLPDGRDVQLELLKLLAELAVFCGIIDKPEEKVQQLYNILITYMPLPPVIEITEVPKLQFSHVECLMYAFHKLCKQTPEFLTKDPEQLKEFRLRLQYFARGIQGYIKKLREAISGKSEEELKSEENQLKVVALKTTNNINTLIKDLFHSPPSFKSVIHLSWKTTANDKKNEKHSSAQRRHTPITFGNDSNSNKRSKEDKSKRELYTPPSGKYSSNISSNYGRGRFRGNRSGGRGGYRPRGRGTWKKNFY

>341

MDSRDRLELQMPPTAPFILLGRVFKKGPELCLAQPQIHLESGCFLRGGGEFRLANSGLEVFDTSGHPDLIELEENGRKKSFLVLSITQDFSIYVLFFQHKDAYQVILDGVKGGAKEKRLAAQFIPKFFKHFPDLADSAINAQLDLCEDEDVSIRRQAIKELPQFATGDNLPRVADILTQLLQSDDSAEFNLVNNALLSIFKMDAKGTLGGLFSQILQGEDIVRERAIKFLSTKLKTLPEEVMTKEVEEFILTESKKVLEDVTGEEFVLFMKILSGLKSLQTVSGRQQLVELVAEQADLEQTFNPSDTDCVDRLLQCTRQAVPLFSKNVHSTKFVTYFCEHVLPNLSSLTTLVEGLDIQLEVLKLLAEMSSFCGDMEKLESNLKKLFDKLLEYMPLPPEEAENGENAGNEEPKLQFSYVECLLYSFHQLGRKLPDFLTAKLNAEKLKDFKIRLQYFARGLQVYIRQLRLALQGKTGEALKTEENKIKVVALKITNNINVLIKDLFHIPPSYKSTVTLSWKPVQKADASQKRASEDTTSSSPPKKASAGPKRDARQIYNPPSGKYSSNLGSFSYEQRGGFRGGRGRGWGGRGNRSRGRIY

>342

MATTVEELYRNYGILADAKEDLSQHKDAYQVILDGVKGGPKEKRLAAQFIPKFFSSFPELADAAINAQLDLCEDEDVSIRRQAIKELPRFAAGENLPRVADILTQLLQTDDSAEFNQVNSALISIFKIDPKGTLGGLFSQILQGEDVVRERAIKFLSTKLKTMPEDVMTKEVEDYVFTETKKVLEDVTGEEFVLLMRVLSGLKYLQTVSGRQQLVELVVEQAFLEQALNPADTDSVDRLLQCTRQALPLFSKNVHSTRFVTYFCEHVLPNLSMLTSPVAELDIQLEILKLLAEMSPYCGDMDKLEANLMMLFEKLLEFMPLPPEDENGENAVSEEPKLQFSYVECLLFSFHQLGKKLPDFLIDKINTEKLKDFKIRLQYFARGLQVYIRQLRVALQGKTGDALKTEENKIKVVALKITNNINVLIKDLFHNPPSYKSTVTLSWKPVQKTEAAPVVGQKRSSGEDTGTGTLGKKVSPLPRRDARQIYNPPSGKYSATIGNFSYEQRGGFRGGRGRGFGGRGNRSRGRIY

>343

MPTVEELYRNYGILADATEQVGQHKDAYQVILDGVKGGTKEKRLAAQFIPKFFKHFPELADSAINAQLDLCEDEDVSIRRQAIKELPQFATGENLPRVADILTQLLQTDDSAEFNLVNNALLSIFKMDAKGTLGGLFSQILQGEDIVRERAIKFLSTKLKTLPDEVLTKEVEELILTESKKVLEDVTGEEFVLFMKILSGLKSLQTVSGRQQLVELVAEQADLEQTFNPSDPDCVDRLLQCTRQAVPLFSKNVHSTRFVTYFCEQVLPNLGTLTTPVEGLDIQLEVLKLLAEMSSFCGDMEKLETNLRKLFDKLLEYMPLPPEEAENGENAGNEEPKLQFSYVECLLYSFHQLGRKLPDFLTAKLNAEKLKDFKIRLQYFARGLQVYIRQLRLALQGKTGEALKTEENKIKVVALKITNNINVLIKDLFHIPPSYKSTVTLSWKPVQKVEIGQKRASEDTTSGSPPKKSSAGPKRDARQIYNPPSGKYSSNLGNFNYERSLQGKL

>344

RNYGILADATEQVGQHKDAYQVILDGVKGGTKEKRLAAQFIPKFFKHFPELADSAINAQLDLCEDEDVSIRRQAIKELPQFATGENLPRVADILTQLLQTDDSAEFNLVNNALLSIFKMDAKGTLGGLFSQILQGEDIVRERAIKFLSTKLKTLPDEVLTKEVEELILTESKKVLEDVTGEEFVLFMKILSGLKSLQTVSGRQQLVELVAEQADLEQTFNPSDPDCVDRLLXXMSSFCGDMEKLETNLRKLFDKLLEYMPLPPEEAENGENAGNEEPKLQFSYVECLLYSFHQLGRKLPDFLTAKLNAEKLKDFKIRLQYFARGLQVYIRQLRLALQGKTGEALKTEENKIKVVALKITNNINVLIKDLFHIPPSYKSTVTLSWKPVQKVEIGQKRASEDTTSGSPPKKSSAGPKRDARQIYNPPSGKYSSNLGNF

>345

MAATVEELYRNYGILADAKEDLSQHKDAYQVILDGVKGGPKEKRLAAQFIPKFFSSFPDLADAAINAQLDLCEDEDVSIRRQAIKELPRFAAGENLPRVADILTQLLQTDDAAEFNQVNNALISIFKIDARGTLGGLFSQIIQGEDVVRDRAIRFLAAKLKTMPEETLTKEVEDYIFTETKKVLEDVTGEEFVLLMRVLSGLKNLQTVSGRQQLVELVVEQAFLEQALNPADADSVDRLLQCTRQALPLFSKNVHSTRFVTYFCEHVLPNLSMLTSPVAELDIQLEVLKLLAEMSPFCGDMDKLESNLMMLFEKLLEFMPLPPEEENGENAGTDEPKLQFSYVECLLFSFHQLGKKLPDFLIDKVNAEKLKDFKIRLQYFARGLQVYIRQLRVALQGKTGDALKTEENKIKVVALKITNNINVLIKDLFHNPPSYKSTVTLSWKPVQKTEAAAAAAAVVGQKRSSVEDVATGTIGKKVSPLPRRDARQIYNPPSGKYSASIGNFSYEQRGGFRGGRGRGFGRGNGSRGRIY

>346

MTEPSEDAQHIEKLYEFGERLNEAEDKSQNVKDYQGIIDAAKTSTKAKQLAAQLIPRFFKFFPSLSGPAIDAHIDLIEEEELAIRVQAIRGLPLFCKDTPENIGKIVDILVQILASEEFVERDAVHKALMALLRQDVKASLSALFKHIGSVDEPTTDEVIREKVLSFIREKVFPIKSEILKPQEEMERHITDLIKKSLEDVTGAEFRMFMDFLKSLSIFGEKAPPERLKELIGIIEGQADLDAQFNVSDADHIDRLISCLFMAIPFVVRGASSCKFLSYLNKHVIPVFEKLPEERKLDLLKALAEFSPYTTPQDSRQFLPSVVQLLKKYMPGRKTGEEMNFTYVECLLYTFHHLAHKVPNATNSLCGYKIVTGQPSDRLGEDFSDNYKDFTERLTNVEDLTRATIKKLTQGMDEHNKAMAAAKSDEAKSNIKTQQQNAKTGLRTCNNILAMGKPLHAKAPTFIGDNSINLSWKEVTKTQVPTTTSAAGGKRPAIAANGSNNMPSKKGRGSGGLQNQLVNRALEGLSYGGRGGGMRGGRGRGWGGRGRGRGRGFR

>347

MPTVEELYRNYGILADAKETAAEHKDAYQVILDGVKGGAKEKRLAAQFIPKFFKHFPELADSAINAQLDLCEDEDVSIRRQAIKELPQFATGDNLPRVADILTQLLQSDDSAEFNLVNNALLSIFKMDAKGTLGGLFSQILQGEDIVRERAIKFLSTKLKTLAEEVLTKEVEELILTESKKVLEDVTGEEFVLFMKILSGLKSLQTVSGRQQLVELVAEQADLEQTFNPSDPDCVDRLLQCTRQAVPLFSKNVHSTRFVTYFCEHVLPNLSSLTTPVEGLDIQLEVLKLLAEMSSFCGDMEKLESNLKKLFDKLLEYMPLPPEEAENGENAGSEEPKLQFSYVECLLYSFHQLGRKLPDFLTAKLNTEKVKDFKIRLQYFARGLQVYIRQLRLALQGKTGEALKTEENKIKVVALKITNNINVLIKDLFHIPPSYKSTVTLSWKPVQKAEAGQKRASEDTTSDLPAKKAPAGPKRDARQIYNPPSGKYSSNLGNFSYEQRGGFRGEEGEDGVERGNRSRGRIY

>348

MSTDSIEKLYKNFGVLADAKDKLSEHEAEYLEILKAVKGTPKEKRLASQFIARFFKHFPKLADKAIDAHLDLCEDEDMSIRKQAIKDLPTLCKDSKEHTARIADILAQLLLAPDSSELDVVHNSIMTLIKNDPKGAINGFFVQILNGEDGIRERCIKFLGSKLKTLGRDIITKEPEDVLIAECKKVLQDVTAEEFHSIMEILAWTRLGSTVNGQQELVDIAVEQAELSVPFKHTNLEQCSRLIQCIKHALPYFSSQVNSSKFVSYICMQVLPHLSLITSPDGRDVQLELLKLLAELAEYCGKIEKPEEKVQQLYNALITFMPLPPDTEITEVPKLQFSHVECLIFAFHKLCKQTPEFLIKDPEQLKEFRLRLQYFARGIQGYIKKLREVINGKSEEELKTEENQLKVIALKTTNNINTLIKDLFHSPPSFKSVIHLSWKTIITKEKKDEKVTTGIKRHTPITFGNGNSNSAAPPAKKQKEDVPRNNKQLYTPPSGKYSSNISSNYGNRGRFHNNRYNKTSGRGGFRPRGRGGNWRKNTY

>349

MTDSSDEAKQIEKLYEFGERLNEAKDKSQNVKDYEGVIDATKTSLKAKQLAAQLIPRFFKFFPNLSSRALNAHFDLIEEEDLAVRVQAIRGLPLFCKDTKEYISKIVDILGQLLTADEIVERDAVHKALMSVLRQDVKESLTALFKHIWNVEEPSQDDTIRDKVLCFIRDKVFPLKTDLLRPPEEMERHITDLIKKSLGDVTGAEFRMFMDFLKSLSIFGEKAPPERLKELIGIIEGQADLDAQFDVSDADHIDRLISCLFMAIPFFVRGAPGSKFLNYLNKHIIPVFDKLPEERKLDLLKALAEISPYTTPQDSRQVLPSVVQLLKKYMPRRKTGEETNFTYVECLLFSFHHLAHKAPNASNSLCGYKIVTGQPSDRLGEDFSEYYKDFTERLSSVEDLTRATIKKLTQGMAEHNKTMAAAKSDEAKDNIKTLKQNTTTGLRTCNNILVMTKPLHLKTPAFIGDKSVNLSWKEAIKPSALSTTTATGVKRPAAGSGSNNLAIKKGHGAGNMQNQLVNRALEGISYGGSGGRGRGRGWGRRGRGRGYR

>350

MEAEDNIQKMYKYFGILADAKENIAEKEPEYLEILSAVKGSTKEKRLASQFITRFFKHFPNLAAQALEAQLDLCEDEDICIRKQAIKDLPVLCKEAKEYLTKIADILAQLLQADDPQELLVAQNSLLSLFKIDAKGALTGIFSQMQSNEEIVRERSMKFILNKVMALGKEIIKRDVEDLIIAECKKVMQNITCEEFETLMTILSSTHLINTPDGQKELVELLASTAELDQFFNPKDLDQVNRFITCLDFSIPFFSAHVESTKFIVYICELLNRYTLIKDNDKQFIILKSLAEFVPYCGKLMNPEAVVGQVYQALLDLVTVPENDSNKKVEDMDLHRVEALLYTFHKLGKQCPDFLSKDPERQKDFKKKLLYVGTCTQTFVKVVRQDLREKGEEDVKNDPIVEKKLEGLKLACNINTLIKELFNMPPRFKATVNLSWLGGATKSKLAQIIQEGKKHEPITVDGKSKRIDGSNNSQQLYQPPKDKFSAKFSNNTNNNNRRGGSSTGWNTKRSFDTSNGNRRSWRPY

>351

MDPGARLELQMPPTASFMMLGRTLKKGPELCLVQPQIHLESGCFLQGGAEFRLANSDLGVFDTALADLEDVWQAVLLKGLLREGEICYGLKVARSPLVFSSLRQERKHKDAYQVILDGVKGGAKEKRLAAQFIPKFFKHFPELADSAINAQLDLCEDEDVSIRRQAIKELPQFATGDNLPRVADILTQLLQSDDSAEFNLVNNALLSIFKMDAKGTLGGLFSQILQGEDIVRERAIKFLSTKLKTLPEEVMTKEVEEFILTESKKVLEDVTGEEFVLFMKILSGLKSLQTVSGRQQLVELVAEQADLEQTFNPSDTDCVDRLLQCTRQAVPLFSKNVHSTKFVTYFCEHVLPNLSSLTTLVEGLDIQLEVLKLLAEMSSFCGDMEKLESNLKKLFDKLLEYMPLPPEEAENGENAGNEEPKLQFSYVECLLYSFHQLGRKLPDFLTAKLNAEKLKDFKIRLQYFARGLQVYIRQLRLALQGKTGEALKTEENKIKVVALKITNNINVLIKDLFHIPPSYKSTVTLSWKPVQKADASQKRASEDTTSSSPPKKPSAGPKRDARQIYNPPSGKYSSNLGSFSYEQRGGFRGGRGRGWGGRGNRSRGRIY

>352

NVDDLPALQLSHVECLMYVFHQLAKRTPEFLTAEGNAERLRDFKLRLQYLARGVQGYIKKLRSALQGKKAEELKSDENKLKTAALKTTNNINTLIRDLFHVPPSFKATIVLSWKPPVTSKVADPSGAPATGSSNSAKPERRRISFSEDSPPKVLRKASATAKNERELYSPP

>353

MPTVEELYRNYGILADATEQVGQHKDAYQVILDGVKGGTKEKRLAAQFIPKFFKHFPELADSAINAQLDLCEDEDVSIRRQAIKELPQFATGENLPRVADILTQLLQTDDSAEFNLVNNALLSIFKMDAKGTLGGLFSQILQGEDIVRERAIKFLSTKLKTLPDEVLTKEVEELILTESKKVLEDVTGEEFVLFMKILSGLKSLQTVSGRQQLVELVAEQADLEQTFNPSDPDCVDRLLQCTRQAVPLFSKNVHSTRFVTYFCEQVLPNLSSLTTPVEGLDIQLEVLKLLAEMSSFCGDMEKLETNLRKLFDKLLEYMPLPPEEAENGENAGNEEPKLQFSYVECLLYSFHQLGRKLPDFLTAKLNAEKLKDFKIRLQYFARGLQVYIRQLRLALQGKTGEALKTEENKIKVVALKITNNINVLIKDLFHIPPSYKSTVTLSWKPVQKVEIGQRRASEDTTSGSPPKKSPAGPKRDARQIYNPPSGKYSSNLSNFNYEQRGAFRGSRGGRGWGARGNRSRGRLY

>354

MPTVEELYRNYGILADATETAGQHKDAYQVILDGVKGGAKEKRLAAQFIPKFFKHFPELADSAINAQLDLCEDEDVSIRRQAIKELPQFATGDNLPRVADILTQLLQSDDSAEFNLVNNALLSIFKMDAKGTLGGLFSQILQGEDIVRERAIKFLSTKLKTLPEEVLTKEVEEFILTESKKVLEDVTGEEFVLFMKILSGLKSLQTVSGRQQLVELVAEQADLEQTFNPSDPDCVDRLLQCTRQAVPLFSKNVHSTRFVTYFCEHVLPNLSSLTTPVEGLDIQLEVLKLLAEMSSFCGDMEKLESNLKKLFDKLLEYMPLPPEEAENGENAGNEEPKLQFSYVECLLYSFHQLGRKLPDFLTAKLNAEKLKDFKIRLQYFARGLQVYIRQLRLALQGKTGEALKTEENKIKVVALKITNNINVLIKDLFHIPPSYKSTVTLSWKPVQKADAGQKRANEDTTSSSPPKKAAAGPKRDARQIYNPPSGKYSSNLGSFSYEQRGGFRGGRGRGWGGRGNRSRGRIY

>355

MDEVFAEKYLRSPDENDIARLLVVGEAKRIPRNIRVSRLYALKPEELLGCMSREFTIVLKVVRLSRGFVTLSIITVSLLSPSLSSESTDVDSITIGLMHAADYEAIISAVKGQSVKAKQLAAQLIPRFFRSFPALATRAMEAMFDLVDMEELATRIQAIRGFPLLAKDAEFVSKIADILGQLLASEENVERDAVHKALMSLIRQDVKSSLQPLFKHVESGSEIREKVICFLKDKVFPVKAELLKPQAQMERYITDLIKKSVLDVTGLEFKLFMDFLRSLSIFGDSAPRESFQELIEIIQAQADLDAQFNVSDNDHIERWTSCMYMALPIFMRGGSSSKFLNYFVKQIVPVFDKIPEEKKLDLLKTIAASSPYATAQDSRQLLPTVVQLLKKYMPGKKVEDINHNYVECLLYTFHHLAHKTPNTTNSLCGYKIVTGQPSDRLGEDFSEHHKDFTERLTGTEETVRAASKRQTQGMADFNKAISSAKTEEEKIKIKSDQQKSTMTMRAYNNILAMAQPLHAKSPLFIGDKKITLSWMEQPKKPAATTAGAKRPQPATNGNTSANKKGRGDGAGRNQLVNRAFEGLSRGGRGIGRGRGRGGRGRGWGYR

>356

MPTVEELYRNYGILADAKETAPEHKDAYQVILDGVKGGPKEKRLAAQFIPKFFKHFPELADSAINAQLDLCEDEDVSIRRQAIKELPQFAMGDNLPRVADILTQLLQSDDSAEFNLVNNALLSIFKMDAKGTLGGLFSQILQGEDIVRERAIKFLSTKLKTLPEEVLTKEVEELILTESKKVLEDVTGEEFVLFMKILSGLKSLQTVSGRQQLVELVAEQADLEQTFNPSDPDCVDRLLQCTRQAVPLFSKNVHSTRFVTYFCEHVLPNLSSLTTPVEGLDIQLEVLKLLAEMSSFCGDMEKLESNLKKLFDKLLEYMPLPPEEAENGENAGNEEPKLQFSYVECLLYSFHQLGRKLTDFLTAKLNTDKVKDFKIRLQYFARGLQVYIRQLRLALQGKTGEALKTEENKIKVVALKITNNINVLIKDLFHIPPSYKSTVTLSWKPVQKSEVGQKRASEDTSSDLPAKKSQAGPKRDARQIYNPPSGKYSSNLGNFSYEHRGGFRGGRGRGWGGRGNRSRGRIY

>357

MSTDSIEKLYKNFGILADAKDKLAEHEKEYMEILTAVKGTTKEKRLASQFIARFFKHFPKLADQAIDAHLDLCEDEDIAIRKQAIKDLPSLCKENKEHTARIADILAQLLLAQDPSELAVVQNSIMSLIKNDPKGAISGFFSQILNGDDGARERCIKFLATKLKAVGHDIITKEPEDLLIVECKKVLQDVTADEFHSIMEVLAWTRLGSTVTGQQELIDIIVEQAELSIPFKHTNEEQWSRLVQCIKHALPFFNSQVDSSRFVCYICVQVLPHLSLITGPEGRDPQLELLKLLAELTTYCGTIEKPEEKVQQIYNALVTYMPLPPETDLPELPKLQFSHVECLMYTFHKLGKQVPEFLTRDADQLKEFRLRLQYFARGIQGYIKKLREAISGKSEEQLKSDENQMKVVALKTTNNINTLIKDLFHSPPSYKSIIHLSWKPSTTDKKTDKASPGQKRHTPITFGNDNAPNKRSKDDNKGNKREIYTPPSGKYSSNISSNYGNRGRFRGNRSGGRGGGYRARGRGWRRSAY

>358

MSTDSIEKLYKNFGILADAKDKLAEHEKEYLEILTAVKGSPKEKRLASQFIARFFKYFPKLADQAIDAHLDLCEDEDMAIRKQAIKDLPALCKDNKEHTARIADILAQLLQAQDSSELAVVHNSVMTLMKTDPKGTISGFFSQIINGDDGTRERCIKFLATKLKAIGHDVIVKEPEDLLISECKKVLQDVTADEFHSIMEILAWTRLGSTVNGQQELVDITTEQAELSEPFKHTNVEQWNRLVQCIKHALPFFSSQIDSSRFVSYICVQVLPHLSLITAPDGRDVQLELLKLLAELTVFCGTIDKPEEKVQQLYNTLITYMPLPPATEITEVPKLEFSHVECLMYSFHKLCKQTPEFLMKDPEQLKEFRLRLQYFARGIQGYIKKLREAISGKSEEELRSEENQLKVVALKTTNNINTLIKDLFHSPPSFKSVIHLSWKTSVTDKKNEKNSSVQKRHTPITFGNDSNSNKRSKEDKSKREIYTPPSGKYSSNISSNYGRGRFRGNRSGGRGGYRPRGRGTWRKNFY

>359

MEAEDNIQKMYKYFGILADAKENIADKEPEYLEILSAVKGSTKEKRLASQFITRFFKHFPNLAAQALEAQLDLCEDEDICIRKQAIKDLPVLCKEAKEYLTKIADILAQLLQADDPQELLVAQNSLLSLFKIDAKGALTGIFSQMQSNEEIVRERSMKFILNKVMALGKEIIKRDVEDLIIAECKKVMQNITCEEFETLMTILSSTHLINTPDGQRELVELLASTAELDQFFNPKDLDQVNRFITCLDFSIPFFSAHVESTKFIVYICELLNRYTLIKDNDKQFIILKSLAESVPFCGKLMNPEAVVGQVYHALLDLVTVPENDTNKKVEDMDLHRVEALLYTFHKLGKQCPDFLSKDPERQKDFKKKLLYVGTCTQTFVKVVKQDLKEKGEEDVKNDPIVEKKLEGLKLACNINTLIKDLFSIPPRFKATVNLSWLDGVTKSKLAQIIQEGKKHEPITLDGKSKRIDGSNNSQQLYQPPKDKFSAKFSNNTNNNNRRGGSSTGWNTKRSFDTNTGNRRSWRPY

>360

MPTVEELYRNYGILADAKETAAQHKDAYQVILDGVKGSAKEKRLAAQFIPKFFKHFPELADSAINAQLDLCEDEDVSIRRQAIKELPQFATGDNLPRVADILTQLLQSDDSAEFNLVNNALLSIFKMDAKGTLGGLFSQILQGEDIVRERAIKFLSTKLKTLPEEVLTKEIEEFIFTESKKVLEDVTGEEFVLFMKILSSLKSLQTVSGRQQLVELVAEQADLEQTFNPSDPDCVDRLLQCTRQAVPLFSKNVHSTRFVTYFCEHVLPNLSSLTTPVEGLDIQLEVLKLLAEMSSFCGDMEKLESNLKKLFDKLLEYMPLPPEEAENGENAGNEEPKLQFSYVECLLYSFHQLGRKLPDFLTAKLNTEKVKDFKIRLQYFARGLQVYIRQLRLALQGKTGEALKTEENKIKVVALKITNNINVLIKDLFHIPPSYKSTVTLSWKPVQKSDAGQKRASEDTASDLPAKKAPAGPKRDARQIYNPPSGKYSSNLGNFSYEQRGGFRGGRGRGWGGRGNRSRGRIY

>361

MAVTIEELYRNYGILADAKENLSQHKDAYQAILDGVKGGPKEKRLAAQFIPKFFSSFPELADAAINAQLDLCEDEDVSIRRQAIKELPRFATGENIFRVADILTQLLQTDDTAEFNQVNTALISIFKMDAKGTLGGLFSQILQGEDIVRERAIKFLSTKLKTLPEDVMTKEVEDYVFAETKKVMEDVTGEEFVLLMRVVSGLRVLQSVNGRQQLVELVVEQADLEQALNPADPDAVDRLLQCTRQALPLFSKNVHSTRFVTYFCEHVLPNLSTLTCPVAELDIQLEVLKLLAEMSPFCGDMEKLEANLSTLFTKLVEFMPLPPEEVENGENSASEEPKLQFSYVECLLFSFHQLGKKLPDFLVDKVDAERLKDFKIRLQYFARGLQVYIRQLRVALQGKTGDALKTEENKIKVVALKITNNINVLIKDLFHNPPSYKSTVTLSWKPVQKPEAAAPKRPSGEEMGSGANTKKQIMPLPRRDARQIYNPPSGKYSASIGNFNYEQRGGFRGGRGRGWGMRGNRSRGRIY

>362

MRERAHPYVDVARKARTDRAGALRLRVVSLASLPHLPWPSRSCWEVSSRAIARAAVGPQRRVLEPSCAGSARPHGPLVRRKRAAGRGRDRGTGIAGLCASIPTLMAVTVEELYRNYGILADAKDSVAQHKDAYQVILDGVKGGPKEKRLAAQFIPKFFKSFPELADAAINAQLDLCEDEDVSIRRQAIKELPQFAAGENLPRVADILTQLLQTDDSAEFNLVNNALLSIFKMDARGTLGGLFTQILQGEDIVRERAIKFLSTKLKTLPEEVLTKEVEDYIFTESKRVLEDVTGEEFVLLMKILSGLKSLQTVSGRQQLVELVAEQAYLEQHLNPADADSVDRLLQCTRQAVPLFSKNVHSTRFVTYFCEHVMSNLSTLTSPVAELDIQLEVLKLLAEMSPFCGDMDKLEANLSMLFEKLLEYMPLPPEEAENGENAGNEEPKLQFSYVECLLYSFHQLGKKLPDFLTDKVNAERLKDFKIRLQYFARGLQVYIRQLRVALQGKTGDALKTDENKIKVVALKITNNINVLIKDLFHNPPSYKSTVTLSWKPVQKVEAAGLKRTTSDDTTSSTPSKKPSPLPKRDSRQIYNPPSGKYSGSIGNFSYEQRGGFRGGRGRGWGGRGNRSRGRIY

>363

MSTDSIEKLYKNFGILADAKDKLAEHEKEYLEILTAVKGSSKEKRLASQFIARFFKNFPKLADQAIDAHLDLCEDEDMAIRKQAIKDLPTLCKDNKEYTAKIADILAQLLQAQDPSELAVVHNSVMSLLKSDPKGTISGFFSQILNGDDGTRERCIKFLATKLRAIGHDIITKEPEDLLISECKKVLQDVTADEFHSIMEVLSWTRLGSTISGQQELIDITVEQAELSVPFKHTNVEQWSRLIQCIKHALPFFSSQIDSSRFVSYICIQVLPHLSLINTPDGRDSQLELLKLLAELTVFCGTIENPEEKVQQLYNTLITYMPLPPATEITDVPKLQFSHVECLMYAFHKLCKQTPEFLVKDPEQLKEFRLRLQYFARGIQGYIKKLREAISGKSEEELNTKENQLKVVALKTTNNINTLIKDLFHSPPSFKSVIHLSWKTTMNDKKSEKNSSSQKRHTPITFGNDSSSSKRNKEDKSNKREIYTPPSGKYSSNISSNYGSRGRFRGNRSGGRGGYRSRGRGTWRKSFY

>364

MSTDSIEKLYKHFGILADAKDKLPEHEKEYLEILKAVKGSPKEKRLASQFIARFFKHFPKLADKAIEAHLDLCEDEDIAIRKQAIKDLPALCKDNKEHTPRIADILAQLLQAEDPTELAVVHNSIMSLLKNDSKGTLNGFFSHILNDDGERDGERESCIKFLASKLKSIGRDVITKESEDLLIAECKKILNDVTASEFHGIMDVLSWTRLGSTVIGQQELVDITIEQAELSVPFKHSSIEQLDRLIQCIKHALPHFSAQIDSSKYVSYICMQVLPHLSLISAPDGRDAQLIILKLLAELMTFCGNVEKPEEKVQQLYNALIVYMPLPPDTDITEVPKLEFSHVECLMYAFHKLCKQTPEFLIKDPEQLKEFRLRLQYFARGIQGYIKELREAISGKSEKDLKSEENQLKVVALKTTNNINTLIKDLFHSPPSFKSVIHLSWKTPVVDTKKVEKTSSIQKRHTPITFGNDGSPTKRNKDDKQGGKREIYTPPSGKYSANISSGNYGNRGRFRGNRSGGRGGFRPRGRGGWRKNSY

>365

MFEVTVESLYNCFNVISSNSSSDAEKLRAYQQILQGSKGGPNEKKLSSQFIGRFFKLFQTEQENSFNCLLDLCDDEDSMTRIQAVRDFQQICKSVPTFIPRVSDVLAQLIITENVSESNGINNSLKSLLIMDPSSTLVGIFNQIMSSKSGIQRKKLMQFLMNCLKDIPGEKMTIELEEFIIEHLNHLFSETSSTEFDAVIVIMSSLKSLSTLLGRQRLVNMVSNVVMDKVPIFDPQSVQSVELIQRAGKQIVRLLSKNVSAAKFLKYMLVNVVPLVLSVKNPFQQRGILQILTNFSSHPGDTFTSVPEDCRIQLLQPLYMALLTSFPEPSTLTDEGDAPAKMSLPAFTIECFLYTLLNLLKFCPTFLCASVQNVGDAASQEGVARLHSLRHKAQYTARLIQSYKTVIVAELQASFQAEGGCSVKTADEARRALANIEKMVRCIFRSKIEPYCLQDVTLSWIEPAPPKVPTTSSLTATTTGAKRPASQQQGASQSWNKRRRFQHLYRGSRRN

>366

MIAASVESLYNCFDTISNEKSSDVEKSNAFQIILLGSKCGPNEKRLSSQLIGRFFKLFKNEQENSFNHLLDLCDDEDPIIRMQAVHDLLQICKSEPAYISRVSDVLSQMFASDDASELHVITLVMFNLLEMDPSGTIAGIFNHIISDDSGIHRENLVKFLMANLKRLPDGKISSELEEFIIQQSNKLLPIVSGSGFVQLISLISSLKSTTSLQARQSLVNKITDHVVQSIPVFNPQVVSSVTHIRDCGKQVVQLLSKNVSAGGFLRYVLVKVIPKVSLVKNPSDQRSILQLLAEFSAHPGVTFRSDEKTHTLLPLYNFLMELLPEPSSGQELMITLEDGGSKKLLVPAFAEECCIYSLLSLLRFYPKFLCTAEAGDDEAAREGCFRLQALRQKVQYTARLIRSYRASIIAELQATFRIEGLSPVMLAEEARRALENIEKMVRCLFRPRVEPEHLSDLTLSWVDATSTLKRPAPSTTLVGSHIAAKQSPPKLQRHLPVTGHRFGGFQVGRGRARGSGRRRF

>367

MPTVEELYRNYGILADATETAGQHKDAYQAILDGVKGDAKEKRLAAQFIPKFFKHFPELADSAINAQLDLCEDEDVSIRRQAIKELPQFATGDNLPRVADILTQLLQSDDSAEFNLVNNALLSIFKMDAKGTLGGLFSQILQGEDIVRERAIKFLSTKLKTLPEEVMTKEVEEFILAESKKVLEDVTGEEFVLFMKILSGLKSLQTVSGRQQLVELVAEQADLEQTFNPSDPDCVDRLLQCTRQAVPLFSKNVHSTKFVTYFCEHVLPNLSALTTPVEGLDIQLEVLKLLAEMSSFCGDMEKLESNLKKLFDKLLEYMPLPPEEAENGENAGNEEPKLQFSYVECLLYSFHQLGRKLPDFLTAKLNAEKLKDFKIRLQYFARGLQVYIRQLRLALQGKTGEALKTEENKIKVVALKITNNINVLIKDLFHIPPSYKSTVTLSWKPVQKADASQKRASEDTTSSSPPKKASAGPKRDARQIYNPPSGKYSSNLGSFSYEQRGGFRGGRGRGWGGRGNRSRGRIY

>368

MSTDSIEKLYKNFGILADAKDKLAEHEKEYLEILTAVKGSSKEKRLASQFIARFFKNFPKLADQAIDAHLDLCEDEDMAIRKQAIKDLPTLCKDNKEYTAKIADILAQLLQAQDPSELAVVHNSVMSLLKSDPKGTISGFFSQILNGDDGTRERCIKFLATKLRAIGHDIITKEPEDLLISECKKVLQDVTADEFHSIMEVLSWTRLGSTISGQQELIDITVEQAELSVPFKHTNVEQWSRLIQCIKHALPFFSSQIDSSRFVSYICIQVLPHLSLINTPDGRDSQLELLKLLAELTVFCGTIENPEEKVQQLYNTLITYMPLPPATEITDVPKLQFSHVECLMYAFHKLCKQTPEFLVKDPEQLKEFRLRLQYFARGIQGYIKKLREAISGKSEEELNTEENQLKVVALKTTNNINTLIKDLFHSPPSFKSVIHLSWKTTMNDKKSEKNSSSQKRHTPITFGNDSSSSKRNKEDKSNKREIYTPPSGKYSSNISSNYGSRGRFRGNRSGGRGGYRSRGRSTWRKSFY

>369

MSSGLSSQWHRYPECIKQLSLQFLKYDTVTRLGSHKDAYQVILDGVKGGTKEKRLAAQFIPKFFKHFPELADSAINAQLDLCEDEDVSIRRQAIKELPQFATGENLPRVADILTQLLQTDDSAEFNLVNNALLSIFKMDAKGTLGGLFSQILQGEDIVRERAIKFLSTKLKTLPDEVLTKEVEELILTESKKVLEDVTGEEFVLFMKILSGLKSLQTVSGRQQLVELVAEQADLEQTFNPSDPDCVDRLLQCTRQAVPLFSKNVHSTRFVTYFCEQVLPNLSSLTTPVEGLDIQLEVLKLLAEMSSFCGDMEKLETNLRKLFDKLLEYMPLPPEEAENGENAGNEEPKLQFSYVECLLYSFHQLGRKLPDFLTAKLNAEKLKDFKIRLQYFARGLQVYIRQLRLALQGKTGEALKTDENKIKVVALKITNNINVLIKDLFHIPPSYKSTVTLSWKPVQKVEIGQKRATEDTTSGSPPKKSPAGPKRDARQIYNPPSGKYSSNLGNFNYEQRGAFRGSRGGRGWGARGNRSRGRLY

>370

MGRALCRALVVKDRPVLSRKVSLHKDAYQVILDGVKGGTKEKRLAAQFIPKFFKHFPELADSAINAQLDLCEDEDVSIRRQAIKELPQFATGENLPRVADILTQLLQTDDSAEFNLVNNALLSIFKMDAKGTLGGLFSQILQGEDIVRERAIKFLSTKLKTLPDEVLTKEVEELILTESKKVLEDVTGEEFVLFMKILSGLKSLQTVSGRQQLVELVAEQADLEQTFNPSDPDCVDRLLQCTRQAVPLFSKNVHSTRFVTYFCEQVLPNLGSLTTPVEGLDIQLEVLKLLAEMSSFCGDMEKLETNLRKLFDKLLEYMPLPPEEAENGENAGNEEPKLQFSYVECLLYSFHQLGRKLPDFLTAKLNAEKLKDFKIRLQYFARGLQVYIRQLRLALQGKTGEALKTDENKIKVVALKITNNINVLIKDLFHIPPSYKSTVTLSWKPVQKVELGQKRASEDTTSGSPPKKSSAGPKRDARQIYNPPSGKYSSNLGNFNYEQRGAFRGSRGGRGWGARGNRSRGRLY

>371

MPTVEELYRNYGILADATETVSQHKDAYQVILDGVKGGAKEKRLAAQFIPXFFKHFPELADSAINAQLDLCEDEDVSIRRQAIKELPQFATGDNLPRVADILTQLLQSDDSAEFNLVNNALLSIFKMDAKGTLGGLFSQILQGEDIVRERAIKFLSTKLKTLPEEVMTKEVEEFILTESKKVLEDVTGEEFVLFMKILSGLKSLQTVSGRQQLVELVAEQADLEQTFNPSDPDCVDRLLQCTRQAVPLFSKNVHSTKFVTYFCEHVLPNLGSLTTPVEGLDIQLEVLKLLAEMSSFCGDMEKLESNLKKLFDKXLEYMPLPPEEAENGENAGSEEPKLQFSYVECLLYSFHQLGRKLPDFLTAKLNAEKLKDFKIRLQYFARGLQVYIRQLRLALQGKTGEALKTEENKIKVVALKITNNINVLIKDLFHIPPSYKSTVTLSWKPVQKADASQKRTSEDTTSSSPPKKSSAGPKRDARQIYNPPSGKYSSNLGSFSYEQRGGFRGGRGRGWGGRGNRSRGRIY

>372

MSAAHLEMATPTVEELYRNYGILADAKDQVEQHRDAYQVILDGVKGGAKEKRLAAQFIPKFFKHFPDLADPAINAQLDLCEDEDVSIRRQAIKELPQFATGENLPRVADILTQLLQSDDSAEFNLVNNALLCIFKMDAKGTLGGLFTQILQGEDIVRERAIKFLSTKLKTIPEEVLTKEVEEYIFTESKKVLEDVTGEEFVLFMKILSGLKSLQTVSGRQQLVELVAEQADLEQKFNPAEPDCVDRLLQCTRQAVPLFSKNVHSTKFVTYFCEHVLPSLSSLTSPVEGLDIQLEVLKLLAEMSSFCGDMEKLESNLKKLFDKLLEYMPLPPEEAENGENAGNEEPKLQFSYVECLLYSFHQLGKKLPDFLTAKLNAEKLKDFKIRLQYFARGLQVYIRQLRLALQGKTGEALKTDENKIKVVALKITNNINVLIKDLFHIPPSYKSTVTLSWKPVQKADAGQKRSSSEDTPSNSPPKKPTAGPKRDARQIYNPPSGKYSGNLGSFSYEQRGGFRGGRGRGWGGRGNRSRGRMY

>373

MSTDSIEKLYKNFGILADAKDKLSEHEKEYLEILTAVKGTAKEKRLASQFIARFFKHFPKLADQAIDAHLDLCEDEDMAIRKQAIKDLPSLCKDSKEHTPRIADILAQLLQAQDSTELAVVYSSIMTLMKNDPKGTLSGFFSQIFNGDDGTRDRCIKFLATKLKSLGHDVINKESEELLITECKKVLRDVTAEEFHNIMDILGWTKLGSTVAGQQELIEITIEQAELGVPFKHNNVEQWDKLVQCIKHALPHFSAQTDSSRYVSYICIQVLPHLSLITAPDGRDAQLEILKLLAELVAFCGTIDKPEEKVQQIYNALITYMPLPPDSDISEVPKLQFSHVECLMYAFHKLCKQTPDFLLKDAEQLKEFKLRLQYFARGIQGYIKKLREAIGGKSEEELKTEENQLKVVALKTTNNINTLIKDLFHSPPSFKSIIHLSWKTPTVDKKNEKVSIAQKRHTPITFGNGSPTKRIKDEHKGGKREIYTPPSGKYSSNISSNYGNRGRFRGNRSGGRGGFRPRGRGTWRKNLY

>374

KEAAYRAFLLGTKGGDIEKRLASQFIARFSKQFENLREESFNCILDLCDDEDVAIRKQAVHDLMQFCKRIPSFIPRVADVLVQMFQTEDASEMHVISLSLNQLLTLEPKATLAGVFNQLLTVNPENPREHVMKFLVDRLKTLPEDKLSSEVEEFVVEQVNKVLHDVSEEEFPLLISILSSLKCMSTLPGRQKLVAMITEQALQACPEFTPTDPACVAQIRESVKQAALCISKNVHARELYMYLLQKVIPKIIHIPDTLEEDKFSLLRSTAELANLHESDLSALTASEQEQCLQYAFDSLVHYLPELPNASVDAQVYPPLEGKTVPEKPVFPTGLKLSELECLLCICCQLGRVRPQYFGGYSSEQERESEKVQLGAARLRQIRPRLQYLSQVAQEYSQSIAGHLEQNGHTDENKAKIVAHRLVTNIPNLVRMFFHNPPMFQTNVTFSWLKTDPIISPGSKRPAVMANLQITDSSTRSTMPRREQPRYAPPIGRWSRGNQPQNRAPGDFSRNYRGRGRNW

>375

MAVTIEDLYRSYGILADAKDNLSQHKDAYQVIVDGVKGGPKEKRLAAQFIPKFFSSFPELADAAINAQLDLCEDEDVSIRRQAIKELPRFATGDNIPRVADILTQLLQTDDTAEFNQVNAALISIFKMDARGTLGGLFSQILQGEDIVRERAIKFLSTKLKTLPEDVMTKEVEEYVFAETKKVMEDVTGEEFVLLMRVVSGLRVLQTVNGRQQLVELVVEQAFLEQALNPADPDTVDRLLQCTRQALPLFSKNVHSTRFVTYFCEHVLPNLSALTSPVAELDIQLEVLKLLAEMSPFCGDMEKLEANLNMLFTKLLEFMPLPPEEVENGENSASEEPKLQFSYVECLLYSFHQLGKKLPDFLLDKVDSERLKDFKIRLQYFARGLQVYIRQLRVALQGKTGDALKTEENKIKVVALKITNNINVLIKDLFHNPPSYKSTVTLSWKPVQKAEAVALKRPSTEEMGSGGSTKKQISPQSRRDARQIYNPPSGKYSASIGNFNYEQRGGFRGGRGRGFGGRGNRSRGRIY

>376

MSSRYFRSWNWGASSSAGKDGSDQITGKQSPSRTRSSACNRRFPSISQSSASFRQLSFKCDVSDINRFFARVKAVFSKGAGSFAWRIRALKPLTPHLQIQRACRSGGRLAGSLAKRLAEEGNIEHGHGDYEKLWHQEKYQACPRVFQLRKVLRYEVSTSGMKHKDAYQVILDGVKGGAKEKRLAAQFIPKFFKHFPELADSAINAQLDLCEDEDVSIRRQAIKELPQFATGDNLPRVADILTQLLQSDDSAEFNLVNNALLSIFKMDAKGTLGGLFSQILQGEDIVRERAIKFLSTKLKTLPEEVMTKEVEEFILTESKKVLEDVTGEEFVLFMKILSGLKSLQTVSGRQQLVELVAEQADLEQTFNPSDTDCVDRLLQCTRQAVPLFSKNVHSTKFVTYFCEHVLPNLSSLTTLVEGLDIQLEVLKLLAEMSSFCGDMEKLESNLKKLFDKLLEYMPLPPEEAENGENAGNEEPKLQFSYVECLLYSFHQLGRKLPDFLTAKLNAEKLKDFKIRLQYFARGLQVYIRQLRLALQGKTGEALKTEENKIKVVALKITNNINVLIKDLFHIPPSYKSTVTLSWKPVQKADASQKRASEDTTSSSQRDARQIYNPPSGKYSSNLGSFSYEQRGGFRGGRGRGWGGRGNRSRGRIY

>377

MAVTIEELYRNYGILADAKPEDLSQHKYAYQVILDGVKGGPKEKRLAAQFIPKFFSSFPELADAAINAQLDLCEDEDVSIRRQAIKELPRFAAGENIVRVADILTQLLQTDDSAEFGQVNTALVSIFKMDAKATLRGLFSQILQGEDIVRERAIKFLSIKLKTLPEDTMTKEVEDYVFTETKKVLEDVTGEEFVLLMRILMALKGLQTVNGRQQLVELVVEQAFLEQALNPADPDTVDRLLQCTRQALPLFSKNVHSSRFVSYFCDHVLPNLSTLTSPVAELDIQQEVLKLLAEMSPFCGDMEKVEANLLMLFEKLLEFMPLPPEAEGENGENTMSEEPKLQFSYVECLLFSFHQLGKKLPDFLIDKINAERLKDFKIRLQYFARGLQVYIRQLRVALQGKTGDALKTDENKIKVVALKITNNINVLIKDLFHNPPSYKSTVTLSWRPVQKAEAGAQKRPSGEEMGTGSTMKKLLSPPLPRRNARQIYNPPSGKYSATIGNFTNEQRGGFRGGRGRGFGGRGGRSRGRIY

>378

MLLSSRLQYFARGLQVYIRQLRLALQGKTGEALKTEENKIKVVALKITNNINVLIKDLFHIPPSYKSTVTLSWKPVQKSEVGQKRASEDTSSDLPAKKSQAGPKRDARQIYNPPSGKYSSNLGNFSYEQRGGFRSGRGRGWGGRGNRSRGRIY

>379

MPTVEELYRNYGILADAKDTAPEHKDAYQVILDGVKGGAKEKRLAAQFIPKFFKHFPELADSAINAQLDLCEDEDVSIRRQAIKELPQFAMGDNLPRVADILTQLLQTDDSAEFNLVNNALLSIFKMDAKGTLGGLFSQILQGEDIVRERAIKFLSTKLKALPEEVLTKEVEELILTESKKVLEDVTGEEFVLFMKILSGLKNLQTVSGRQQLVELVAEQADLEQTFNPSDPDCVDRLLQCTRQAVPLFSKNVHSTRFVTYLCEHVLPNLSSLTTPVEGLDIQLEVLKLLAEMSSFCGDMEKLESNLKKLFDKLLEYMPLPPEEAENGENAGNEEPKLQFSYVECLLYSFHQLGRKLTDFLTAKLNTDKVKDFKIRLQYFARGLQVYIRQLRLALQGKTGEALKTEEVSL

>380

MDYKTMQLRFQEIPGAWNGPPLDLHKDAYQAILDGVKGGAKEKRLAAQFIPKFFKHFPELADSAINAQLDLCEDEDVSIRRQAIKELPQFATGDNLPRVADILTQLLQSDDSAEFNLVNNALLSIFKMDAKGTLGGLFSQILQGEDIVRERAIKFLSTKLKTLPEEVMTKEVEEFILTESKKVLEDVTGEEFVLFMKILSGLKSLQTVSGRQQLVELVAEQADLEQTFNPSDPDCVDRLLQCTRQAVPLFSKNVHSTKFVTYFCEHVLPNLSSLTTPVEGLDIQLEVLKLLAEMSSFCGDMEKLESNLKKLFDKLLEYMPLPPEEAENGENAGNEEPKLQFSYVECLLYSFHQLGRKLPDFLTAKLNAEKLKDFKIRLQYFARGLQVYIRQLRLALQGKTGEALKTEENKIKVVALKITNNINVLIKDLFHIPPSYKSTVTLSWKPVQKADAGQKRTSEDTTSSSPPKKAPAGPKRDARQIYNPPSGKYSSNLGSFSYEQRGGFRGGRGRGWGGRGNRSRGRIY

>381

MAVTIEDLYRSYGILADAKDNLSQHKDAYQVIVDGVKGGPKEKRLAAQFIPKFFSSFPELADAAINAQLDLCEDEDVSIRRQAIKELPRFATGDNIPRVADILTQLLQTDDSAEFNQVNAALISIFKMDARGTLGGLFSQILQGEDIVRERAIKFLSAKLKTLPEDVMTKEVEEYVFAETKKVMEDVTGEEFVLLMRVVSGLRVLQTVNGRQQLVELVVEQAFLEQALNPADPDTVDRLLQCTRQALPLFSKNVHSTRFVTYFCEHVLPNLSALTSPVAELDIQLEVLKLLAEMSPFCGDMEKLEANLNMLFTKLLEFMPLPPEEVENGENSASEEPKLQFSYVECLLYSFHQLGKKLPDFLLDKVDAERLKDFKIRLQYFARGLQVYIRQLRVALQGKTGDALKTEENKIKVVALKITNNINVLIKDLFHNPPSYKSTVTLSWKPVQKAEAVALKRPSTEEMGSGGSTKKQISPQSRRDARQIYNPPSGKYSASIGNFNYEQRGGFRGGRGRGFGGRGNRSRGRIY

>382

MATDNIEKLYQNYGVLADAKHDISKHEKEYLEILAAVKGSDKEKRLASQFIAKFFSSFPNLAEQAIEAQFDLCEDDDVAIRKQAIKDLPSLCKDHKEHTQRIADILAQLLQSDDVTEINIVTNSLLAILKTDPKGALMGLFSQIHQSTDSEVTNEIVRERCIKFLATKVKQLGREVINKEAEDLIIAECKKILEDVVAEEFEHIIDLLTWSKLGKTPAGKKELVQLVAAIGLSPDDWHPEDPEYVDRVLQCSQHALPLFSAQVDSTQFVNFFCDHVLACWKDITTPEGTDPKLELLKVFAEISEHCGDLEEAQKKIDAVYDVLMTYLPEAPIEEELKKEDEKKEENVKEETNSEENKQAPSLQFSHVECALYGLHSLCRKAPDALGADPARLKALRLRLQYTARLTQGYIKKLKEVTQGKKVDTNSEENKLKVAALKTTSNINTLIRDIFRTPPSFKSKVQLSFQSTKKVDKEERHSEAATEDKDMNSQGQKRHRPITFDNGNKESPEKRSRSGDRNMKMYTPPSGKYSSRLNNSGRFSGPNSGRGRGYGRRDFRNSGAPFRRRSNY

>383

MSTDIIEKLYQNYGILADAKEDISKHEKEYLEILAAVKGSDKEKRLASQFIAKFFHSFPNLADQAIEAQFDLCEDDDVAIRKQAIKDLPTMCKTNKEHTQRIADILAQLLQSEDATEINVVTNSLLTILKGDPKGTLAGLFSQIHQSTDSEVTNEIVRERCIKFLATKVKQLGREVINKEAEELIIVECKKILEDVVAEEFEYIMELLTWSRLGKTPAGKKELVQLVAALALTPDDWHPEDPEYIDRIIQCSQHALPLFSTQVDSTQFVNFFCEHILKNWNNVATTEGSDIKLELLKIFAEMTEFCGDLENLQNKVDTVYDVLMNYLPEAPVEGETVNDKPKADKTEDSKTNAEPSLQFSHVECTLFALHSLCRKSPEVLSTDQAKLKALRLRLQYTARLTQGYIKKLKEVTNSKKHSSEMSEENKLKITALNTTSNINSLIRDIFRTPPSFKSKVQLSFHNKKSEKEALTVRSSEKETDSAGQKRHQPITFDKGEKESPEKRARSGDRNLKMYTPPSGKYSSRFTGSYSGRGRGGYGRRDFRNNGAPFQRRSNY

>384

MSTDIIEKLYQNYGILADAKEDISKHEKEYLEILAAVKGSDKEKRLASQFIAKFFHSFPNLADQAIEAQFDLCEDDDVAIRKQAIKDLPTMCKTNKEHTQRIADILAQLLQSEDATEINVVTNSLLTILKGDPKGTLAGLFSQIHQSTDSEVTNEIVRERCIKFLATKVKQLGREVINKEAEELIIVECKKILEDVVAEEFEYIMELLTWSRLGKTPAGKKELVQLVAALALTPDDWHPEDPEYIDRVIQCSQHALPFFSTQVDSTQFVNFFCDHILKNWSNVATTEGNDIKLELLKIFAEMTEFCGDLENLQSKVDTVYDVLMNYLPEAPVEGEGNDKKEGEKTEDSKNAEPSLQFSHVECTLFALHSLCRKSPEVLSSDQNKLKALRLRLQYTARLTQGYIKKLKEVTNSKKPSTEMTEENKLKITALNTTSNINTLIRDIFRTPPSFKSKVQLSFHTKKSEKEAVTVQSDEKETDSAGQKRHQPITFDKGDKESPVKRARSGDRNIKMYTPPSGKYSSRLSNTNSGRGRGGYGRRDFRNNGAPFRRRSNY

>385

MAEHGPDPQPAPAPLGSDVTPTSGSRQPIKWRPRSRAVHKDAYQVILDGVKGGAKEKRLAAQFIPKFFKHFPELADSAINAQLDLCEDEDVSIRRQAIKELPQFATGDNLPRVADILTQLLQSDDSAEFNLVNNALLSIFKMDAKGTLGGLFSQILQGEDIVRERAIKFLSTKLKTLPEEVMTKEVEEFILTESKKVLEDVTGEEFVLFMKILSGLKSLQTVSGRQQLVELVAEQADLEQTFNPSDPDCVDRLLQCTRQAVPLFSKNVHSTKFVTYFCEHVLPNLGSLTTPVEGLDIQLEVLKLLAEMSSFCGDMEKLESNLKKLFDKLLEYMPLPPEEAENGENAGSEEPKLQFSYVECLLYSFHQLGRKLPDFLTAKLNAEKLKDFKIRLQYFARGLQVYIRQLRLALQGKTGEALKTEENKIKVVALKITNNINVLIKDLFHIPPSYKSTVTLSWKPVQKADASQKRTSEDTTSSSPPKKASAGPKRDARQIYNPPSGKYSSNLGSFSYEQRGGFRGGRGRGWGGRGNRSRGRIY

>386

MADVSDEAKDIGKLYVYGERLNEVKDKSQHAEDYENIIKAGKSSSVKARQLAAQLIPRFFKYFPGLSDKAVDAHLDLCEAEELGIRVQAIRGLPLFCKDTPQHLSKIVDILAQLLTAEENVERDAVHKALLSLLRQDVRDSLDALFKHIESVDEQIADESLRERTLLFIRDKVFPLKTELLRPPEQMERHITDLIKKSLQDVTGAEFKMFMDFLKSLSMFGQKAPPERIQELLEIIEGQADLDAQFSVTDGDHIARLISCLFTALPFFERGASNSKFFNYLNKHIFPVFDELPEERKVDLLKDLAEGSPYTSPQDSRQILPAVVLLLKKTMPRRKAEEMNFTYVECLLYSFHHLAHKTPNATNSLCGYKIVTGQPSDRLGEDFSDQYKDFSERLTCVEDLARVTIKKLTQGMAEHNKTLPAAVSDEAKASIKTQKQNTTSGLRTCNNILAMTQPLHSKTPSFIGDKRINLSWKEVVKPSTTSTSTTTATGGKRPPGAQSGGGNNPLKKVRGGGSTQHHQLPNRAFEDQPYPHGGRNSGGRGRGRGGHGRGRGRGRGRGRGRGWGFQ

>387

MAVTVEELYRNYGILADAKDNLSPHKDAYQVILDGVKGGPKEKRLAAQFIPKFFSSFPELADAAINAQLDLCEDEDVSIRRQAIKELPRFAAGENLPRVADILTQLLQTDDSAEFNQVNTALISIFKIDAKGTLGGLFSQILHGEDIVRERAIKFIAGKLKTMPEDVMTKEVEDHVYAETKKVLEDVTGEEFVLLMRILSGLKNLQTVSGRQQLVELVVEQAFLEQALNPADNDAVDRLLQCTRQALPLFSKNVHSTRFVTYFCDHVLPNLSTLTSPVPEQDIQLEVLKLLAEMSPFCGDMEKLESNLKMLFEKLLEYMPLPPEEGENGENAANEEPKLQFSYVECLLFSFHQLGKKMPDFLIDKIDADKLKDFKIRLQYFARGLQVYIRQLRVALQGKTGEALKTDENKIKVVALKITNNINVLIKDLFHNPPSYKSTVSLSWKPVQKEAAVVGQKRPSGEDDGPGAAMKKLPSTLPRRDARQIYNPPRDKYSGTIGNFPNEQRGGFRGGRGRGFGGRGNRSRGRIY

>388

MPSVEELYRNYGILADATEQVGQHKDAYQVILDGVKGGTKEKRLAAQFIPKFFKHFPELADSAINAQLDLCEDEDVSVSLQLFRFAGTLGGLFSQILQGEDIVRERAIKFLSTKLKTLPDEVLTKEVEELILTESKKVLEDVTGEEFVLFMKILSGLKSLQTVSGRQQLVELVAEQADLEQTFNPSDPDCVDRLLQCTRQAVPLFSKNVHSTRFVTYFCEQVLPNLGTLTTPVEGLDIQLEVLKLLAEMSSFCGDMEKLESNLRKLFDKLLEYMPLPPEEAENGENAGNEEPKLQFSYVECLLYSFHQLGRKLPDFLTAKLNAEKLKDFKIRLQYFARGLQVYIRQLRLALQGKTGEALKTEENKIKVVALKITNNINVLIKDLFHIPPSYKSTVTLSWKPVQKVEMGQKRATEDTTSGSPPKKSPAGPKRDARQIYNPPSGKYSSNLSNFNYEQRGAFRGSRGGRGWGARGNRSRGRLY

>389

MSSAAADPQGAQELPSLLQHPRPHKDAYQVILDGVKGGAKEKRLAAQFIPKFFKHFPELADSAINAQLDLCEDEDVSIRRQAIKELPQFATGDNLPRVADILTQLLQSDDSAEFNLVNNALLSIFKMDAKGTLGGLFSQILQGEDIVRERAIKFLSTKLKTLPEEVMTKEVEEFILAESKKVLEDVTGEEFVLFMKILSGLKSLQTVSGRQQLVELVAEQADLEQTFNPSDTDCVDRLLQCTRQAVPLFSKNVHSTKFVTYFCEHVLPNLSSLTTLVEGLDIQLEVLKLLAEMSSFCGDMEKLESNLKKLFDKLLEYMPLPPEEAENGENAGNEEPKLQFSYVECLLYSFHQLGRKLPDFLTAKLNAEKLKDFKIRLQYFARGLQVYIRQLRLALQGKTGEALKTEENKIKVVALKITNNINVLIKDLFHIPPSYKSTVTLSWKPVQKADASQKRASEDTTSSSPPKKASAGPKRDARQIYNPPSGKYSSNLGSFSYEQRGGFRGGRGRGWGGRGNRSRGRIY

>390

MTDSSDEAKQIEKLYEFGERLNEAKDKSQNVKDYEGVIDATKTSLKAKQLAAQLIPRFFKFFPNLSSRALNAHFDLIEEEDLAVRVQAIRGLPLFCKDTKEYISKIVDILGQLLTADEIVERDAVHKALMSVLRQDVKESLTALFKHIWNVEEPSQDDTIRDKVLCFIRDKVFPLKAELLRPPEEMERHITDLIKKSLGDVTGAEFRMFMDFLKSLSIFGEKAPPERLKELIGIIEGQADLDAQFDVSDADHIDRLISCLFMAIPFFVRGAPGSKFLNYLNKHIIPVFDKLPEERKLDLLKALAEISPYTTPQDSRQVLPSVVQLLKKYMPRRKTGEETNFTYVECLLFSFHHLAHKAPNASNSLCGYKIVTGQPSDRLGEDFSEYYKDFTERLSSVEDLTRATIKKLTQGMAEHNKAMAAAKSDEAKDNIKTLKQNTTTGLRTCNNILVMTKPLHLKTPAFIGDKSVNLSWKEAIKPSALSTTTATGVKRPAAGSGSNNLAIKKGRGAGNMQNQLVNRALEGISYGGSGGRGRGRGWGRRGRGRGYR

>391

EMSSFCGDMDKLESNLNKLFEKLLEYMPLPPEEVENGENSANEEPKLQFSYVECLLFSFHQLGRKNPDFLTGDKVNAEKLKDFKIRLQYFARGLQVYIRQLRLALQGKSGEALKTEENKIKVVA

>392

MSTDSIEKLYKNFGILADAKDKLVQHEKEYLEILTAVKGSPKEKRLASQFIARFFKHFPKLADEAINAHLDLCEDEDMAIRKQAIKDLPSLCKDNKEHTARIADILAQLLQAEDSSELAVVHNSIMSLMKSDPKGTLSGFFSQIINGDDATRERCIKFLATKLKAIGHDVITKEPEDLLIAECKKVLQDVTADEFHSIIEILAWTRLGSTVTGQQELVDIIIEQAELSIPFKHTNVEQWNRLVQCIKHALPFFSSQTDSSKFVSYICVQVLPHLSLIISPDGRDIQLELLKLLAELTVFCGTIDKPEDKVQQLYNTLTTYMPLPPATEITDVPKLQFSHVECLMYAFHKLCKQTPEFLIKDPEQLKEFRLRLQYFARGIQGYIKKLREAISGKTEEELKSDENQLKVVALKTTNNINTLIKDLFHSPPSFKSVIHLSWKTPCTDKKVEKNSAQKRHTPITFGNDSNSNKRNKEDKSSKREIYTPPSGKYSSNISNYGRGRFKGNRSGGRGGFRSRGRGPWRKNFY

>393

MSTDSIEKLYKNFGILADAKDKLAEHEKEYLEILTAVKGSPKEKRLASQFIARFFKYFPKLADQAIDAHLDLCEDEDMAIRKQAIKDLPALCKDNKEHTARIADILAQLLQAQDPSELAVVHNSVMSLMKTDPRGTISGFFSQIINGDDGTRERCIKFLATKLKAIGHDIITKEPEDLLISECKKVLQDVTADEFHSIMEVLAWTRLGSTVSGQQELVDITVEQAELSEPFKHTNVEQWNRLVQCIKHALPFFSSQIDSSRFVSYICVQVLPHLSLITSPDGRDVQLELLKLLAELAVFCGTIDKPDEKVQQLYNTLITYMPLPPATEITEVPKLQFSHVECLMYAFHKLCKQTPEFLIKDPEQLKEFRLRLQYFARGIQGYIKKLREAISGKTEEELKSEENQLKVVALKTTNNINTLIKDLFHSPPSFKSVIYLSWKTSSSDKKSEKHSSAQKRHTPITFGNDSSSNKRSKEDKSKRELYTPPSGKYSSNISSNYGRGRFRGNRSGGRGGYRSRGRGTWRKNFY

>394

MRPAVTPRSYTVRSASQRLEFNCASRAHLASRSPRAGHTMSTDSIEKLYKNFGILADAKDKLVQHEKEYLEILTAVKGSPKEKRLASQFIARFFKHFPKLADQAIDAHLDLCEDEDMAIRKQAIKDLPALCKDNKEHTARIADILAQLLQAEDSSELAVVHNSIMSLMKSDPKGTLSGFFSQIINGDDGTRERCIKFLATKLKAIGHDIITKEPEDLLIAECKKVLQDVTADEFHSIIEILAWTRLGSTVTGQQELIDITIEQAELSVPFKHTNIEQWNRLVQCVKHALPFFSSQIDSSKFVSYICVQVLPHLSLMTSPDGRDIQLELLKLLAELTVFCGTIEKPEDKVQQLYNTLITYMPLPPATDITDVPKLQFSHVECLMYAFHKLCKQTPEFLIKDPEQLKEFRLRLQYFARGIQGYIKKLREAISGKTEEELKSEENQLKVVALKTTNNINTLIKDLFHSPPSFKSVIHLSWKTSCNDKKSDKNSTQKRHTPITFGNDSSPNKRSKEDKNNKREIYTPPSGKYSSNISNYGRGRFKGNRPRGRGGFRTRGRGPWRKNFY

>395

HEKEYLEILTAVKGSPKEKRLASQFIARFFKHFPKLADQAIDAHLDLCEDEDMAIRKQAIKDLPTLCKDNKEHTARIADILAQLLQAEDSSELAVVHNSIMSLMKSDPKGTLSGFFSQIISGDDGTRERCIKFLATKLKAIGHDIITKEPEDLLIAECKKVLQDVTADEFHSIMEILAWTRLGSTVTGQQELIDITIEQAELSVPFKHTNIEQWNRLVQCVKHALPFFSSQIDSSKFVSYICVQVLPHLSLMTSPDGRDIQLELLKLLAELTVFCGTIEKPEDKVQQLYNTLITYMPLPPATEITDVPKLQFSHVECLMYAFHKLCKQTPEFLIKDPEQLKEFRLRLQYFARGIQGYIKKLREAISGKTEEELKSEENQLKVVALKTTNNINTLIKDLFHSPPSFKSIIHLSWKTPCNDKKSEKNSAQKRHTPITFGNDNSSNKRSKEDKNNKREIYTPPSGKYSSSISNYGRGRFKGNRPRGRGGFRTRGGRGSWRKNFY

>396

MVSIEQLYKDYEVLAEAGADAGQHESSYASILSAVGGDSPQKQLAGQFIPKFFKYFPSLAEQAINAQLDLCEDDVSMIRRQAIKELPNLCRDSADHLLRIADVLTQLLQSDDLPELNIVRSALMALYNIDSKGTLGGLFNQILSGDDKVRDRAIKFLNNKIQTLPPDRIPKEVEEFLVEKTKEVLVDVTGEEFVIFMKLLTGLSSMQTLLGRKQLLDLVTEQADLSSDFQHTDSDSVDRLMQCIRQAMPFFSKNVQSTAFVSYICERVLPNLTSLASAPDENGASGEEKKDKEKDSNSGGAKLEMLKLLAEMAANCGDLPAEPHIANLFSTLVVSRFSCYGSQNWCVCVFKSPLPSIYENFPNRLLKLS

>397

MTELSEDAQHIEKLYEFGERLNEAEDKSQNVKDYQGIIDAAKTSTKAKQLAAQLIPRFFKFFPSLSGPAIDAHIDLIEEEELAIRVQAIRGLPLFCKDTPENIGKIVDILVQILASEEFVERDAVHKALMALLRQDVKASLSALFKHIGSVDEPTTDEVIREKVLSFIREKVFPIKSEILKPQEEMERHITDLIKKSLEDVTGAEFRMFMDFLKSLSIFGEKAPPERLKELIGIIEGQADLDAQFNVSDADHIDRLISCLFMAIPFVVRGASSCKFLSYLNKHVIPVFEKLPEERKLDLLKALAEFSPYTTPQDSRQFLPSVVQLLKKYMPGRKTGEEMNFTYVECLLYTFHHLAHKVPNATNSLCGYKIVTGQPSDRLGEDFSDNYKDFTERLTNVEDLTRATIKKLTQGMDEHNKAMAAAKSDEAKSNIKTQQQNAKTGLRTCNNILAMGKPLHAKAPTFIGDNSINLSWKEVTKTQVPTTTSAAGGKRPAIAANGSNNMPSKKGRGSGGLQNQLVNRALEGLSYGGRGGGMRGGRGRGWGGRGRGRGRGFR

>398

MPTVGVLSRIYSILADATEQVDQHKDAYQVILDGMKGGTKEKRLAAPFIPKFLKHFPELADSAINAYLMRGKSLAQDIESFDKMTPLNWGSSVLHSDKRDIKYSLSPFTSYTLCKFKKRSDNNDKSFSYLNKNSHIRTLNNIKGVVFVLVQSILLLKRF

>399

MRRKVFLKTSELSVLGAEVHKDAYQVILDGVKGGTKEKRLAAQFIPKFFKHFPELADSAINAQLDLCEDEDVSIRRQAIKELPQFATGENLPRVADILTQLLQTDDSAEFNLVNNALLSIFKMDAKGTLGGLFSQILQGEDIVRERAIKFLSTKLKTLPDEVLTKEVEELILTESKKVLEDVTGEEFVLFMKILSGLKSLQTVSGRQQLVELVAEQADLEQTFSPSDPDCVDRLLQCTRQAVPLFSKNVHSTRFVTYFCEQVLPNLSTLTTPVEGLDIQLEVLKLLAEMSSFCGDMEKLETNLRKLFDKLLEYMPLPPEEAENGENASNEEPKLQFSYVECLLYSFHQLGRKLPDFLTAKLNAEKLKDFKIRLQYFARGLQVYIRQLRLALQGKTGEALKTEENKIKVVALKITNNINVLIKDLFHIPPSYKSTVTLSWKPVQKVEIGQKRANEDTSSGSPPKKSPGGPKRDARQIYNPPSGKYSSNLGNFNYGERFRLGTSSPRD

>400

MSADNIEKLYQNYGILADAKDDIAKHEKEYLEILAAVKGSDKEKRLASQFIAKFFNSFPNLAEQALEAQFDLCEDDDVAIRKQAIKDLPSLCKDHKEHTQRISDILAQLLQSEDATEITMVTNSLLGILKSDPKGTLTGLFSQIHQSTDNEVVRERCIKFLATKVKQVGREVINKEAEDLIIAECKKILEDVVAEEFEHIMDLLTWSRLGKTPAGKKELVQLVGAIAFSPDDWQPEDPEYVDRILQCSMHALPLFSAQVESTQFVNFFCEHVLPRWKDIASPEGSADCKLELLKIFAEVSEHCGELEKAQEKIDTVYDLLMTYLPEAPIVTEEVKKAEESEEAKPDESKATPSLQFSHVECALFALHSLCRKVPEALAADSARLKALRIRLQYTASLTQGYIKKLKEVTQGKKGDNANSEENKLKVAALKTTSNINTLIRDIFRTPPSFKSKVELSFQVKRAEIKEKPSPKSEEKESQVAGQKRHQPIKFDNGEEKSSPEKRARPGDKNVKMYTPPSGKYSSRLTGNGNTGGGGGARDSGNGGRYRGGGGYRRGNAPFKRRNNY

>401

MAVTIEDLYRNYGILADAKDSLSQHKDAYQVILDGVKGGPKEKRLAAQFIPKFFSSFPDMADTAINAQLDLCEDEDVSIRRQAIKELPRFATGDNILRVSDILTQLLQTDDSAEFNQVTSALISIFKIDAKGTLGGLFSQILQGEDIVRERAIKFLAGKLKTLPEDVMTKEVEEYVFAETKKVLEDVTGEEFVLLMRVVSGLRVLQTVSGRQQLVELVVEQAFLEQALNPADPDTVDRLLQCTRQALPLFSKNVHSTRFITYFCEHVLPNLSELTSPAAELDIQLEVLKLLAEMSPFCGDMDKLESNLNMLFIKLLEFMPLPPEEAENGENTGNEEPKLQFSYVECLLFSFHQLGKKLPDFLIDKVDAEQLKDFKIRLQYFARGLQVYIRQLRVALQGKTGDALKTDENKIKVVALKITNNINVLIKDLFHNPPSFKSTVTLSWKPVQKAEALVAKRPSTDTMNPGATAKKPIPAQPRRDARQIYNPPSGKYSATIGNFNYEQRGGFRGGRGRGFGSRGGRSRGRVY

>402

MSTDSIEKLYKNFGVLADAKDKLSEHEQEYLEILKAVKGSPKEKRLASQFIARFFKHFPKLADKAIDAHLDLCEDEDMSIRKQAIKDLPTLCKDSKEHTARIADILAQLLLAQDSSELDVVHNSIMTLIKNDPKGAISGFFVQILNGDDGIRERCIKFLANKLKTLGRDTITKEPEDVLITECKKVLQDVTADEFHSIMEILAWTRLGSTVHGQQELVDIAIEQAELSVPFKYTNLEQCSRLIQCIKHALPFFSSQANSSKFVSYISMQVLPHLSLITSPDGRDVQLELLKLLAELAEYCGKIEKPEEKVQQLYNALITFMPLPPDTEVTEVPKLQFSHVECLIFAFHKLCKQTPEFLIKDAEQLKEFRLRLQYFARGIQGYIKKLREAINGKSEEELKTEENQLKVIALKTTNNINTLIKDLFHSPPSFKSIIHLSWKTIITKQKKDEKVSTGQKRHTPITFGNGNATTHSSNKRMKEDISKNNKQLYTPPSGKYSSNISSNYGNRGRFQNRYNKIGGRGGFRPRGRSSWRKNIY

>403

MAADPSDEAKHIEKLYEFGERLNESKDKSQNVEDYQGIISAAKTSMKAKQLAAQLIPRFFKFFPALSSPAIDTHLDLIEEEELGVRVQAIRGLPLFCKDTPEHISKIVDILVQLLHADEFVERDAVHKALMALLRQDVKASLTALFKHIGSVDEPSQDDIIREKVLSFIRDKVFPIKAEVLKPQEEMERHITDLIKKSLEDVTGAEFRMFMDFLKGLSIFGDKALKERMVELIGIIEGQADLDAQFNVSDADHIDRLISCLFMALPFIVRGASSSKFLNYLNKHILPVSDKLPDERRLDLLKALAEVAPFTTPQDSRQILPSVVQLLKKNMPRRKTGEEFNFTYVECLLYTFHHLAHKVPNATNSLCGYKIVTGQPSDRLGEDFTEQYKEFTERLSYVEELTRATMKKLTQGMAEKNKAMAAAKSDEAKDTIKTEKQNTTTGLRTCNNILAMTKALHSKAPSFIGDKSINLSWKEVPKPAVTSSTPATGGKRPGSAANGSGNPSKKGRGAGGIQHQLVNRAFEGLSHGGRSGGTRGRGRGWGGRGRGRGYNR

>404

MSTDSIEKLYKHFGILADAKDKLSEHEKEYLEILKAVKGSPKEKRLASQFIARFFKYFPKLADKAIEAHLDLCEDEDIAIRKQAIKDLPALCKDNKEHTPRIADILAQLLQAEDSTELAVVDNSIMSLLKNDPKGTLNGFFSHILNDDGERDGERESCIKFLASKLKSLGRDIITKESEDLLIAECKKILNDVTASEFHGIMDVLSWTRLGSTVIGQQELVDITIEQAELSVPFKHSSVEQLDRLIQCIKHALPHFSAQIDSSKYVSYICMQVLPHLSLISAPDGRDAQLIILKLLAELMTFCGNIEKPEEKVQQLYNALIVYMPLPPDTDITEVPKLEFSHVECLMYSFHKLCKQIPEFLIKDPEQLKEFRLRLQYFARGIQGYIKKLREAISGKSEEDLKTEENQLKVVALKTTNNINTLIKDLFHSPPSFKSVIHLSWKTPVVDTKKVEKNSSQKRHTPITFGNDGSPTKRNKEDKQGGKREIYTPPSGKYSGNISSGNYGNRGRFRGNRSGGRGGFRPRGRGGWRKNSY

>405

MSSNAGGGDLLTDGVTDRPSRGLAGPLMEASEYLLKNPPFFFVVLPDDTAEFNQVTGSLIAIFKMDAKGTLGGLFSQILQGEDIVRERAIKFLSTKLKTLTEEVMTKEVEDHVFAETKKVLEDVTGEEFVLLMRVVSNLRVLQTVNGRQQLVELVVEQAFLDQALNTADPDLVDRLLQCTRQALPLFSKNVHSTRFITYFCEHVLPNLSALTSPVPELDIQLEVLKLLAEMSPYCGDMDKLEPNLSMLFTKLVEFMPLPPDEAENGESSAMEEPKLQFSYVECLLFGFHQLGKKLPDFLIEKVDAESLKDFKIRLQYFARGLQVYIRQLRVALQGKTGDALKTDENKMKVVALKITNNINVLIKDLFHNPPSFKSSVTLSWKPVQKSEAAAPKRPSGEELGPGGSTKKQISPLPRRDARQIYNPPSGKYSATIGNFTNGEEHSVALRGLSNPWFSMSICTLGHFTLTF

>406

MHQHEEIEKKEERKILADGQAALGERDGHKHTTGQAVAPPRHLLDLTAATSVRTLRPHQGMQEPHEHACGQHKDAYQAILDGVKGGAKEKRLAAQFIPKFFKHFPELADSAINAQLDLCEDEDVSIRRQAIKELPQFATGDNLPRVADILTQLLQSDDSAEFNLVNNALLSIFKMDAKGTLGGLFSQILQGEDIVRERAIKFLSTKLKTLPDEVMTKEVEEFILTESKKVLEDVTGEEFVLFMKILSGLKSLQTVSGRQQLVELVAEQADLEQTFNPSDPDCVDRLLQCTRQAVPLFSKNVHSTKFVTYFCEHVLPNLSSLTTPVEGLDIQLEVLKLLAEMSSFCGDMEKLESNLKKLFDKLLEYMPLPPEEAENGENAGNEEPKLQFSYVECLLYSFHQLGRKLPDFLTAKLNAEKLKDFKIRLQYFARGLQVYIRQLRLALQGKTGEALKTEENKIKVVALKITNNINVLIKDLFHIPPSYKSTVTLSWKPVQKADASQKRASEDTTSSSPPKKASAGPKRDARQIYNPPSGKYSSNLGSFSYEQRGGFRGGRGRGWGGRGNRSRGRIY

>407

AGMEGRYSRLKEVFTCKILARSAHKDAYQVILDGVKGGAKEKRLAAQFIPKFFKHFPELADSAINAQLDLCEDEDVSIRRQAIKELPQFATGDNLPRVADILTQLLQSDDSAEFNLVNNALLSIFKMDAKGTLGGLFSQILQGEDIVRERAIKFLSTKLKTLPEEVMTKEVEEFILTESKKVLEDVTGEEFVLFMKILSGLKSLQTVSGRQQLVELVAEQADLEQTFNPSDPDCVDRLLQCTRQAVPLFSKNVHSTKFVTYFCEHVLPNLSSLTTPVEGLDIQLEVLKLLAEMSSFCGDMEKLESNLKKLFDKLLEYMPLPPEEAENGENAGNEEPKLQFSYVECLLYSFHQLGRKLPDFLTAKLNAEKLKDFKIRLQYFARGLQVYIRQLRLALQGKTGEALKTEENKIKVVALKITNNINVLIKDLFHIPPSYKSTVTLSWKPVQKADASQKRASEDTTSSSPPKKASAGPKRDARQIYNPPSGKYSSNLGSFSYEQRGGFRGGRGRGWGGRGNRSRGRIY

>408

MERLPADCVILMYEYFYSYFIPLHKDAYQAILDGVKGGAKEKRLAAQFIPKFFKHFPELADSAINAQLDLCEDEDVSIRRQAIKELPQFATGDNLPRVADILTQLLQSDDSAEFNLVNNALLSIFKMDAKGTLGGLFSQILQGEDIVRERAIKFLSTKLKTLPEEVMTKEVEEFILTESKKVLEDVTGEEFVLFMKILSGLKSLQTVSGRQQLVELVAEQADLEQTFNPSDPDCVDRLLQCTRQAVPLFSKNVHSTKFVTYFCEHVLPNLSSLTTPVEGLDIQLEVLKLLAEMSSFCGDMEKLESNLKKLFDKLLEYMPLPPEEAENGENAGNEEPKLQFSYVECLLYSFHQLGRKLPDFLTGKLNAEKLKDFKIRLQYFARGLQVYIRQLRLALQGKTGEALKTEENKIKVVALKITNNINVLIKDLFHIPPSYKSTVTLSWKPVQKADAGQKRTTEDTTSSSPPKKSPAGPKRDARQIYNPPSGKYSSNLGSFSYEQRGGFRGGRGRGWGGRGNRSRGRIY

>409

HLPPPSALSSALLKNVAWRILTSYELESERSLHGGREFRLATSGVKDDNGSHPDLIRRSCSSGELLAGSPAHKDAYQAILDGVKGGAKEKRLAAQFIPKFFKHFPELADSAINAQLDLCEDEDVSIRRQAIKELPQFATGDNLPRVADILTQLLQSDDSAEFNLVNNALLSIFKMDAKGTLGGLFSQILQGEDIVRERAIKFLSTKLKTLPEEVMTKEVEEFILTESKKVLEDVTGEEFVLFMKILSGLKSLQTVSGRQQLVELVAEQADLEQTFNPSDPDCVDRLLQCTRQAVPLFSKNVHSTKFVTYFCEHVLPNLSSLTTPVEGLDIQLEVLKLLAEMSSFCGDMEKLESNLKKLFDKLLEYMPLPPEEAENGENAGNEEPKLQFSYVECLLYSFHQLGRKLPDFLTAKLNAEKLKDFKIRLQYFARGLQVYIRQLRLALQGKTGEALKTEENKIKVVALKITNNINVLIKDLFHIPPSYKSTVTLSWKPVQKADASQKRASEDTTSSSPPKKASAGPKRDARQIYNPPSGKYSSNLGSFSYEQRGGFRGGRGRGWGGRGNRSRGRIY

>410

MPTVEELYRNYGILADATETAGQHKDAYQAILDGVKGGAKEKRLAAQFIPKFFKHFPELADSAINAQLDLCEDEDVSIRRQAIKELPQFATGDNLPRVADILTQLLQSDDSAEFNLVNNALLSIFKMDAKGTLGGLFSQILQGEDIVRERAIKFLSTKLKTLPEEVMTKEVEEFILTESKKVLEDVTGEEFVLFMKILSGLKSLQTVSGRQQLVELVAEQADLEQTFNPSDPDCVDRLLQCTRQAVPLFSKNVHSTKFVTYFCEHVLPNLSSLTTPVEGLDIQLEVLKLLAEMSSFCGDMEKLESNLKKLFDKLLEYMPLPPEEAENGENAGSEEPKLQFSYVECLLYSFHQLGRKLPDFLTAKLNAEKLKDFKIRLQYFARGLQVYIRQLRLALQGKTGEALKTEENKIKVVALKITNNINVLIKDLFHIPPSYKSTVTLSWKPVQKADASQKRASEDTTSSSPPKKASAGPKRDARQIYNPPSGKYSSNLGSFSYEQRGGFRGGRGRGWGGRGNRSRGRIY

>411

MPPVTGVSSRCVRTSEGIVTLSQKGNSDHEEEHKDAYQAILDGVKGGAKEKRLAAQFIPKFFKHFPELADSAINAQLDLCEDEDVSIRRQAIKELPQFATGDNLPRVADILTQLLQSDDSAEFNLVNNALLSIFKMDAKGTLGGLFSQILQGEDIVRERAIKFLSTKLKTLPEEVMTKEVEEFILTESKKVLEDVTGEEFVLFMKILSGLKSLQTVSGRQQLVELVAEQADLEQTFNPSDPDCVDRLLQCTRQAVPLFSKNVHSTKFVTYFCEHVLPNLSSLTTPVEGLDIQLEVLKLLAEMSSFCGDMEKLESNLKKLFDKLLEYMPLPPEEAENGENAGNEEPKLQFSYVECLLYSFHQLGRKLPDFLTAKLNAEKLKDFKIRLQYFARGLQVYIRQLRLALQGKTGEALKTEENKIKVVALKITNNINVLIKDLFHIPPSYKSTVTLSWKPVQKADASQKRASEDTTSSSPPKKASAGPKRDARQIYNPPSGKYSSNLGSFSYEQRGGFRGGRGRGWGGRGNRSRGRIY

>412

HKDAYQAILDGVKGGAKEKRLAAQFIPKFFKHFPELADSAINAQLDLCEDEDVSIRRQAIKELPQFATGDNLPRVADILTQLLQSDDSAEFNLVNNALLSIFKMDAKGTLGGLFSQILQGEDIVRERAIKFLSTKLKTLPEEVMTKEVEEFILTESKKVLEDVTGEEFVLFMKILSGLKSLQTVNGRQQLVELVAEQADLEQTFNPSDPDCVDRLLQCTRQAVPLFSKNVHSTKFVTYFCEHVLPNLSSLTTPVEGLDIQLEVLKLLAEMSSFCGDMEKLESNLKKLFDKLLEYMPLPPEEAENGENAGNEEPKLQFSYVECLLYSFHQLGRKLPDFLTAKLNAEKLKDFKIRLQYFARGLQVYIRQLRLALQGKTGEALKTEENKIKVVALKITNNINVLIKDLFHIPPSYKSTVTLSWKPVQKADASQKRASEDTTSSSPPKKASAGPKRDARQIYNPPSGKYSSNLGSFSYEQRGGFRGGRGRGWGGRGNRSRGRIY

>413

MGCGDERHAAVPQGREQLVALAASSSRPDGSSPSKCGIAHKDAYQAILDGVKGGAKEKRLAAQFIPKFFRHFPELADSAINAQLDLCEDEDVSIRRQAIKELPQFATGDNLPRVADILTQLLQSDDSAEFNLVNNALLSIFKMDAKGTLGGLFSQILQGEDIVRERAIKFLSTKLKTLPEEVMTKEVEEFILFESKKVLEDVTGEEFVLFMKILSGLKSLQTVSGRQQLVELVAEQADLEQTFNPSDPDCVDRLLQCTRQAVPLFSKNVHSTHFVTYFCEHVLPNLGSLTTPVEGLDIQLEVLKLLAEMSSFCGDMEKLESNLRKLFDELLEYMPLPPEEAENGENAGNEEPKLQFSYVECLLYSFHQLGRKLPDFLTAKLNAEKLKDFKIRLQYFARGLQVYIRQLRLALQGKTGEALKTEENKIKVVALKITNNINVLIKDLFHIPPSYKSTVTLSWKPVQKADASQKRTSEDTTSSSLPKKASTGPKRDARQIYNPPSGKYSSNLGSFSYEQRGGFRGGRGRGWGGRGNHSRGRIY

>414

HKDAYQAILDGVKGGTKEKRLAAQFIPKFFKHFPELADSAINAQLDLCEDEDVSIRRQAIKELPQFATGDNLPRVADILTQLLQSDDSAEFNLVNNALLSIFKMDAKGTLGGLFSQILQGEDIVRERAIKFLSTKLKTLPEEVMTKEVEEFILTESKKVLEDVTGEEFVLFMKILSGLKSLQTVSGRQQLVELVAEQADLEQTFNPSDPDCVDRLLQCTRQAVPLFSKNVHSTKFVTYFCEHVLPNLGSLTTPVEGLDIQLEVLKLLAEMSSFCGDMEKLESNLKKLFDKLLEYMPLPPEEAENGENAGNEEPKLQFSYVECLLYSFHQLGRKLPDFLTAKLNAEKLKDFKIRLQYFARGLQVYIRQLRLALQGKTGEALKTEENKIKVVALKITNNINVLIKDLFHIPPSYKSTVTLSWKPVQKADASQKRASEDTTSSSPPKKASTGPKRDARQIYNPPSGKYSSNLGSFSYEQRGGFRGGRGRGWGGRGNRSRGRIY

>415

MASELQTKVSITNKFTNHFKGDYNTSTHCSRLNLELSPLGTASTPRFFFRRERRTDQHRWKSVSSRPGAALVLAAGQDSRLARAVLRVGPLATRAGGGLGRDELRACGSAKTLVAFFLCIQYFQHKDAYQAILDGVKGGAKEKRLAAQFIPKFFKHFPELADSAINAQLDLCEDEDVSIRRQAIKELPQFATGDNLPRVADILTQLLQSDDSAEFNLVNNALLSIFKMDAKGTLGGLFSQILQGEDIVRERAIKFLSTKLKTLPEEVMTKEVEDFILTESKKVLEDVTGEEFVLFMKILSGLKSLQTVSGRQQLVELVAEQADLEQTFNPSDPDCVDRLLQCTRQAVPLFSKNVHSTKFVTYFCEHVLPNLSSLSTPVEGLDIQLEVLKLLAEMSSFCGDMEKLESNLKKLFDKLLEYMPLPPEEAENGENAGNEEPKLQFSYVECLLYSFHQLGRKLPDFLTAKLNAEKLKDFKIRLQYFARGLQVYIRQLRLALQGKTGEALKTEENKIKVVALKITNNINVLIKDLFHIPPSYKSTVTLSWKPVQKADASQKRASEDTTSSSPPKKASAGPKRDARQIYNPPSGKYSSNLGSFSYEQRGGFRGGRGRGWGGRGNRSRGRIY

>416

HKDAYQAILDGVKGGAKEKRLAAQFIPKFFKHFPELADSAINAQLDLCEDEDVSIRRQAIKELPQFATGDNLPRVADILTQLLQSDDSAEFNLVNNALLSIFKMDAKGTLGGLFSQILQGEDIVRERAIKFLSTKLKTLPEEVMTKEVEEFILTESKKVLEDVTGEEFVLFMKILSGLKSLQTVSGRQQLVELVAEQADLEQTFNPSDPDCVDRLLQCTRQAVPLFSKNVHSTKFVTYFCEHVLPNLGSLTTPVEGLDIQLEVLKLLAEMSSFCGDMEKLESNLKKLFDKLLEYMPLPPEEAENGENAGNEEPKLQFSYVECLLYSFHQLGRKLPDFLTAKLNAEKLKDFKIRLQYFARGLQVYIRQLRLALQGKTGEALKTEENKIKVVALKITNNINVLIKDLFHIPPSYKSTVTLSWKPVQKADASQKRASEDTTSSSPPKKASAGPKRDARQIYNPPSGKYSSNLGSFSYEQRGGFRGGRGRGWGGRGNRSRGRIY

>417

HKDAYQAILDGVKGGAKEKRLAAQFIPKFFKHFPELADSAINAQLDLCEDEDVSIRRQAIKELPQFATGDNLPRVADILTQLLQSDDSAEFNLVNNALLSIFKMDAKGTLGGLFSQILQGEDIVRERAIKFLSTKLKTLPEEVMTKEVEEFILTESKKVLEDVTGEEFVLFMKILSGLKSLQTVSGRQQLVELVAEQADLEQTFNPSDPDCVDRLLQCTRQAVPLFSKNVHSTKFVTYFCEHVLPNLSSLTTPVEGLDIQLEVLKLLAEMSSFCGDMEKLESNLKKLFDKLLEYMPLPPEEAENGENAGNEEPKLQFSYVECLLYSFHQLGRKLPDFLTAKLNAEKLKDFKIRLQYFARGLQVYIRQLRLALQGKTGEALKTEENKIKVVALKITNNINVLIKDLFHIPPSYKSTVTLSWKPVQKADASQKRASEDTTSGSPPKKASAGPKRDARQIYNPPSGKYSSNLGSFSYEQRGGFRGGRGRGWGGRGNRSRGRIY

>418

SMPQSSSESGTTHTYPQGVGSVEGASHKDAYQVILDGVKGGAKEKRLAAQFIPKFFKHFPELADSAINAQLDLCEDEDVSIRRQAIKELPQFATGDNLPRVADILTQLLQSDDSAEFNLVNNALLSIFKMDAKGTLGGLFSQILQGEDIVRERAIKFLSTKLKTLPEEVMTKEVEEFILTESKKVLEDVTGEEFVLFMKILSGLKSLQTVSGRQQLVELVAEQADLEQTFNPSDPDCVDRLLQCTRQAVPLFSKNVHSTKFVTYFCEHVLPNLGSLTTPVEGLDIQLEVLKLLAEMSSFCGDMEKLESNLKKLFDKLLEYMPLPPEEAENGENAGSEEPKLQFSYVECLLYSFHQLGRKLPDFLTAKLNAEKLKDFKIRLQYFARGLQVYIRQLRLALQGKTGEALKTEENKIKVVALKITNNINVLIKDLFHIPPSYKSTVTLSWKPVQKADASQKRTSEDTTSSSPPKKASAGPKRDARQIYNPPSGKYSSNLGSFSYEQRGGFRGGRGRGWGGRGNRSRGRIY

>419

MAAGGFGWGRIQALKKTPVVIQHKDAYQAILDGVKGGAKEKRLAAQFIPKFFKHFPELADSAINAQLDLCEDEDVSIRRQAIKELPQFATGDNLPRVADILTQLLQSDDSAEFNLVNNALLSIFKMDAKGTLGGLFSQILQGEDIVRERAIKFLSTKLKTLPEEVMTKEVEEFILTESKKVLEDVTGEEFVLFMKILSGLKSLQTVSGRQQLVELVAEQADLEQTFNPADPDCVDRLLQCTRQAVPLFSKNVHSTKFVTYFCEHVLPNLSSLTTPVEGLDIQLEVLKLLAEMSSFCGDMEKLESNLKKLFDKLLEYMPLPPEEAENGENAGNEEPKLQFSYVECLLYSFHQLGRKLPDFLTAKLNAEKLKDFKIRLQYFARGLQVYIRQLRLALQGKTGEALKTEENKIKVVALKITNNINVLIKDLFHIPPSYKSTVTLSWKPVQKADASQKRASEDTTSSSPPKKASAGPKRDARQIYNPPSGKYSSNLGSFSYEQRGGFRGGRGRGWGGRGNRSRGRIY

>420

MAVQGSAQSLPPVQAPEFLRVPGAVSRNGYHCIGLQYLDEHNGRPTNLDADGLETVLTDNAMEPLSRPAQWHKDAYQVILDGVKGGAKEKRLAAQFIPKFFKHFPELADSAINAQLDLCEDEDVSIRRQAIKELPQFATGDNLPRVADILTQLLQSDDSAEFNLVNNALLSIFKMDAKGTLGGLFSQILQGEDIVRERAIKFLSTKLKTLPEEVMTKEVEEFILSESKKVLEDVTGEEFVLFMKILSGLKSLQTVSGRQQLVELVAEQADLEQTFNPSDPDCVDRLLQCTRQAVPLFSKNVHSTRFVTYFCEHVLPNLSSLTTPVEGLDIQLEVLKLLAEMSSFCGDMEKLESNLKKLFDKLLEYMPLPPEEAENGENAGNEEPKLQFSYVECLLYSFHQLGRKLPDFLTSKLNAEKLKDFKIRLQYFARGLQVYIRQLRLALQGKTGEALKTEENKIKVVALKITNNINVLIKDLFHIPPSYKSTVTLSWKPVQKADASQKRASEDTTSSSPPKKASAGPKRDARQIYNPPSGKYSSNLGSFSYEQRGGFRGGRGRGWGGRGNRSRGRIY

>421

MPTVEELYRNYGILADATETAGQHKDAYQAILDGVKGGAKEKRLAAQFIPKFFKHFPELADSAINAQLDLCEDEDVSIRRQAIKELPQFATGDNLPRVADILTQLLQSDDSAEFNLVNNALLSIFKMDAKGTLGGLFSQILQGEDIVRERAIKFLSTKLKTLPEEVMTKEVEEFILTESKKVLEDVTGEEFVLFMKILSGLKSLQTVSGRQQLVELVAEQADLEQTFNPSDPDCVDRLLQCTRQAVPLFSKNVHSTKFVTYFCEHVLPNLSSLTTPVEGLDIQLEVLKLLAEMSSFCGDMEKLESNLKKLFDKLLEYMPLPPEEAENGENAGSEEPKLQFSYVECLLYSFHQLGRKLPDFLTAKLNAEKLKDFKIRLQYFARGLQVYIRQLRLALQGKTGEALKTEENKIKVVALKITNNINVLIKDLFHIPPSYKSTVTLSWKPVQKADASQKRASEDTTSSSPPKKAXXXXSAKKIYNPPSGKYSSNLGSFSYEQRGGFRGGRGRGWGGRGNRSRGRIY

>422

MFNIFVDDTNSGTECTLSRFANNTKLCGAVDMLEGIPSGGTWTGLREREPGSAGGSRAGSDRQHLRGQSAACCRCGCGASAKGPGLRGLHCGAWHKDAYQAILDGVKGGAKEKRLAAQFIPKFFKHFPELADSAINAQLDLCEDEDVSIRRQAIKELPQFATGDNLPRVADILTQLLQSDDSAEFNLVNNALLSIFKMDAKGTLGGLFSQILQGEDIVRERAIKFLSTKLKTLPEEVMTKEVEEFILTESKKVLEDVTGEEFVLFMKILSGLKSLQTVSGRQQLVELVAEQADLEQTFNPSDPDCVDRLLQCTRQAVPLFSKNVHSTKFVTYFCEHVLPNLSSLTTPVEGLDIQLEVLKLLAEMSSFCGDMEKLESNLKKLFDKLLEYMPLPPEEAENGENAGSEEPKLQFSYVECLLYSFHQLGRKLPDFLTAKLNAEKLKDFKIRLQYFARGLQVYIRQLRLALQGKTGEALKTEENKIKVVALKITNNINVLIKDLFHIPPSYKSTVTLSWKPVQKADASQKRASEDTTSSSPPKKASAGPKRDARQIYNPPSGKYSSNLGSFSYEQRGGFRGGRGRGWGGRGNRSRGRIY

>423

TLGGLFSQILQGEDIVRERAIKFLSTKLKTLPEEVMTKEVEEFILTESKKVLEDVTGEEFVLFMKILSGLKSLQTVSGRQQLVELVAEQADLEQTFNPSDPDCVDRLLQCTRQAVPLFSKNVHSTKFVTYFCEHVLPNLGSLTTPVEGLDIQLEVLKLLAEMSSFCGDMEKLESNLKKLFDKLLEYMPLPPDEAENGENAGGEEPKLQFSYVECLLYSFHQLGRKLPDFLTAKLNAEKLKDFKIRLQYFARGLQVYIRQLRLALQGKTGEALKTEENKIKVVALKITNNINVLIKDLFHIPPSYKSTVTLSWKPVQKADASQKRASEDTTSSSPPKKASAGPKRDARQIYNPPSGKYSSNLGSFSYEQRGGFRGGRGRGWGGRGNRSRGRIY

>424

AIASLVAFAAASSFRPVGSSPFKCGVADVNLLLDSGRPLGGGEFRLANSALEEGTGSHPDLIQRTCDQCLNPVQNLATWGAISKHKDAYQAILDGVKGGAKEKRLAAQFIPKFFKHFPELADSAINAQLDLCEDEDVSIRRQAIKELPQFATGDNLPRVADILTQLLQSDDSAEFNLVNNALLSIFKMDAKGTLGGLFSQILQGEDIVRERAIKFLSTKLKTLPEEVMTKEVEEFILTESKKVLEDVTGEEFVLFMKILSGLKSLQTVSGRQQLVELVAEQADLEQTFNPSDPDCVDRLLQCTRQAVPLFSKNVHSTKFVTYFCEHVLPNLSSLTTPVEGLDIQLEVLKLLAEMSSFCGDMEKLESNLKKLFDKLLEYMPLPPEEAENGENAGNEEPKLQFSYVECLLYSFHQLGRKLPDFLTAKLNAEKLKDFKIRLQYFARGLQVYIRQLRLALQGKTGEALKTEENKIKVVALKITNNINVLIKDLFHIPPSYKSTVTLSWKPVQKADASQKRASEDTTSSSPPKKASAGPKRDARQIYNPPSGKYSSNLGSFSYEQRGGFRGGRGRGWGGRGNRSRGRIY

>425

MAGDNTLPFRTITGEQHNQHKDAYQAILDGVKGGAKEKRLAAQFIPKFFKHFPELADSAINAQLDLCEDEDVSIRRQAIKELPQFATGDNLPRVADILTQLLQSDDSAEFNLVNNALLSIFKMDAKGTLGGLFSQILQGEDIVRERAIKFLSTKLKTLPEEVMTKEVEEFILTESKKVLEDVTGEEFVLFMKILSGLKSLQTVSGRQQLVELVAEQADLEQTFNPSDPDCVDRLLQCTRQAVPLFSKNVHSTKFVTYFCEHVLPNLGSLTSPVEGLDIQLEVLKLLAEMSSFCGDMEKLESNLKKLFDKLLEYMPLPPEEAENGENAGSEEPKLQFSYVECLLYSFHQLGRKLPDFLTAKLNAEKLKDFKIRLQYFARGLQVYIRQLRLALQGKTGEALKTEENKIKVVALKITNNINVLIKDLFHIPPSYKSTVTLSWKPVQKADASQKRASDDTTSSSPPKKASAGPKRDARQIYNPPSGKYSSNLGSFSYEQRGGFRGGRGRGWGGRGNRSRGRIY

>426

MNADGLETVVIPVLLGAHKVVFNEKRGFLPITIADINMFVTLPLMNIATKFHEFNKHKDAYQAILDGVKGGTKEKRLAAQFIPKFFKHFPELADSAINAQLDLCEDEDVSIRRQAIKELPQFATGDNLPRVADILTQLLQSDDSAEFNLVNNALLSIFKMDAKGTLGGLFSQILQGEDIVRERAIKFLSTKLKTLPEEVMTKEVEEFILTESKKVLEDVTGEEFVLFMKILSGLKSLQTVSGRQQLVELVAEQADLEQTFNPSDPDCVDRLLQCTRQAVPLFSKNVHSTKFVTYFCEHVLPNLSSLTTPVEGLDIQLEVLKLLAEMSSFCGDMEKLESNLKKLFDKLLEYMPLPPEEAENGENAGNEEPKLQFSYVECLLYSFHQLGRKLPDFLTAKLNAEKLKDFKIRLQYFARGLQVYIRQLRLALQGKTGEALKTEENKIKVVALKITNNXXLRLQDLFHIPPSYKSTVTLSWKPVQKADAGQKRTSEDTTSSSPPKKAPAGPKRDARQIYNPPSGKYSSNLGSFSYEQRGGFRGGRGRGWGGRGNRSRGRIY

>427

GTVVGRGNLYVRDNKRADRRQKLLEKLPKFSEGDHHKDAYQAILDGVKGGAKEKRLAAQFIPKFFKHFPELADSAINAQLDLCEDEDVSIRRQAIKELPQFATGDNLPRVADILTQLLQSDDSAEFNLVNNALLSIFKMDAKGTLGGLFSQILQGEDIVRERAIKFLSTKLKTLPEEVMTKEVEEFILTESKKVLEDVTGEEFVLFMKILSGLKSLQTVSGRQQLVELVAEQADLEQTFNPSDPDCVDRLLQCTRQAVPLFSKNVHSTKFVTYFCEHVLPNLSSLTTPVEGLDIQLEVLKLLAEMSSFCGDMEKLESNLKKLFDKLLEYMPLPPEEAENGENASNEEPKLQFSYVECLLYSFHQLGRKLPDFLTAKLNAEKLKDFKIRLQYFARGLQVYIRQLRLALQGKTGEALKTEENKIKVVALKITNNINVLIKDLFHIPPSYKSTVTLSWKPVQKADASQKRASEDTTSSSPPKKASAGPKRDARQIYNPPSGKYSSNLGSFSYEQRGGFRGGRGRGWGGRGNRSRGRIY

>428

MRAPSSGCQMVNTASTAVLVGMEVAVNAEKLQSGLRAQETGHQDALRRAFSAGEDESHQIIANPLHLAPGALHMTDASSTSRSFCIQESKSSTETSNGGKREARKVRSVKKSQGRGAGSLTGRGNGHTSSSRLQLGKSSSLSQSPPPALCAVRREVVRAIRPALRAGQNSGLKRWPWGSTSSSPHKDAYQAILDGVKGGAKEKRLAAQFIPKFFKHFPDLADSAINAQLDLCEDEDVSIRRQAIKELPQFATGDNLPRVADILTQLLQSDDSAEFNLVNNALLSIFKMDAKGTLGGLFSQILQGEDIVRERAIKFLSTKLKTLPEDVMTKEVEEFILTESKKVLEDVTGEEFVLFMKILSGLKSLQTVSGRQQLVELVAEQADLEQTFNPSDPDCVDRLLQCTRQAVPLFSKNVHSTKFVTYFCEHVLPNLSSLTTPVEGLDIQLEVLKLLAEMSSFCGDMEKLESNLKKLFDKLLEYMPLPPEEAENGENAGNEEPKLQFSYVECLLYSFHQLGRKLPDFLTAKLDAEKLKDFKIRLQYFARGLQVYIRQLRLALQGKTGEALKTEENKIKVVALKITNNINVLIKDLFHIPPSYKSTVTLSWKPVQKADASQKRASEDTTSSSPPKKASAGPKRDARQIYNPPSGKYSSNLGSFSYEQRGGFRGGRGRGWGGRGSRSRGRIY

>429

MHQKSFMIHSSGTVVGRGNLYLRDNRRAERRQKLLEKLPKFTEGDHHKDAYQVILDGVKGGAKEKRLAAQFIPKFFKHFPELADSAINAQLDLCEDEDVSIRRQAIKELPQFATGDNLPRVADILTQLLQSDDSAEFNLVNNALLSIFKMDAKGTLGGLFSQILQGEDIVRERAIKFLSTKLKTLPEEVMTKEVEEFILAESKKVLEDVTGEEFVLFMKILSGLKSLQTVSGRQQLVELVAEQADLEQTFNPSDPDCVDRLLQCTRQAVPLFSKNVHSTKFVTYFCEHVLPNLSSLTTPVEGLDIQLEVLKLLAEMSSFCGDMEKLESNLKKLFDKLLEYMPLPPDEAENGENAGSEEPKLQFSYVECLLYSFHQLGRKLPDFLTAKLNAEKLKDFKIRLQYFARGLQVYIRQLRLALQGKTGEALKTEENKIKVVALKITNNINVLIKDLFHIPPSYKSTVTLSWKPVQKTDASQKRASEDTTSSSPPKKASAGPKRDARQIYTTPSGKDILGSFSYEQRGGFRGGRGRGWGGRGNRSRGRIY

>430

MVSALLDLTAATSCRTLRHRQSVQEPHESTCGQHKDAYQAILDGVKGGAKEKRLAAQFIPKFFKHFPELADSAINAQLDLCEDEDVSIRRQAIKELPQFATGDNLPRVADILTQLLQSDDSAEFNLVNNALLSIFKMDAKGTLGGLFSQILQGEDIVRERAIKFLSTKLKTLPEEVMTKEVEEFILTESKKVLEDVTGEEFVLFMKILSGLKSLQTVSGRQQLVELVAEQADLEQTFNPSDPDCVDRLLQCTRQAVPLFSKNVHSTKFVTYFCEHVLPNLSSLTTPVEGLDIQLEVLKLLAEMSSFCGDMEKLESNLKKLFDKLLEYMPLPPEEAENGENAGNEEPKLQFSYVECLLYSFHQLGRKLPDFLTAKLNAEKLKDFKIRLQYFARGLQVYIRQLRLALQGKTGEALKTEENKIKVVALKITNNINVLIKDLFHIPPSYKSTVTLSWKPVQKADASQKRASEDTTSSSPPKKASAGPKRDARQIYNPPSGKYSSNLGSFSYEQRGGFRGGRGRGWGGRGNRSRGRIY

>431

HKDAYQVILDGVKGGAKEKRLAAQFIPKFFKHFPELADSAINAQLDLCEDEDVSIRRQAIKELPQFATGDNLPRVADILTQLLQSDDSAEFNLVNNALLSIFKMDAKGTLGGLFSQILQGEDIVRERAIKFLSTKLKTLPEEVMTKEVEEFILTESKKVLEDVTGEEFVLFMKILSGLKSLQTVSGRQQLVELVAEQADLEQTFNPSDPDCVDRLLQCTRQAVPLFSKNVHSTKFVTYFCEHVLPNLSSLTTPVEGLDIQLEVLKLLAEMSSFCGDMEKLESNLKKLFDKLLEYMPLPPEEAENGENAGNEEPKLQFSYVECLLYSFHQLGRKLPDFLTAKLNAEKLKDFKIRLQYFARGLQVYIRQLRLALQGKTGEALKTEENKIKVVALKITNNINVLIKDLFHIPPSYKSTVTLSWKPVQKADASQKRASEDTTSSSPPKKSSAGPKRDARQIYNPPSGKYSSNLGSFSYEQRGGFRGGRGRGWGGRGNRSRGRIY

>432

MEIPNVDKLYECGSALSSAKPADFAKYESQYKTILRGVKGDNNTKKLASQFISRFFAKFPALANDALDAILDLCEDDDVDIRKQAIKDLPTLCREMKEFLPKIADVLSQLLQTEDKNEIVVIQNSLMSLFRRDAKGTLIGLFSQVRNGGDVVRDRALKFLHLKIKTEGRDLLSTKDTEAVLLEEIKHSIQECTADEFKMFMTMLAATSLQKTISGQSMIVELISHSCQLDKGAFDIHDEEAIDRFLHCTNAALPYFSSQVKSTKFADFITGKVLPQFGSLEVEVQTQMLKLLAEVCMFVGTIQAPLEATQHVYKLLMDAVPHQKEASAENDDANFEFTKLECLLFAFHTVAQQAPKFLAEDPALLKDFKARMQYFALAIQGYIRKLDEFIRGKSKAELSSEENQVKIVAHRSAKNISAIVKDLFHTPPSYKTKIVVSWRPPTDKVAMAASTTSSNSGGALKRKSISFVDSPSSQPKKALTNAGSTFGGHKKRPLGGNKVYAPPQGKYSSNLGRVVDDDDDTGKSGKRPFNKGFRGARGFRGSRGRGGRRY

>433

MIAVSVESLYNCFDTISNEKSSDVEKSNAFQIILLGSKCGPNEKRLSSQLIGRFFKLFKNEQESSFNHLLDLCDDEDPIIRMQAVHDLLQICKSEPAYISRVSDVLSQMFASDDASELHVITLVMFNLLEMDPSGTLAGIFNHIISDDSGIHRENLVKFLMANLKRLPDGKISSELEEFIIQQSNKLLPIVSGSGFVQLISLISSLKSTTSLQARQSLVNKITDHVVQSIPVFNPQVVSSVTHIRDCGKQVVQLLSKNVSAGGFLRYVLVKVIPKVSLVKNPSDQRSILQLLAEFSAHPGVTFRSDEKTHTLLPLYNFLMELLPEPSSGQELIITLEDSGSKKLLVPAFAEECCIYSLLSLLRFNPKFLCTTEAGDDEAAREGCFRLQALRQKVQYTARLIRSYRASIIAELQATFRIEGLSPVMLAEEARRALENIEKMVRCLFRPRVEPEHLSDLTLSWVDTTSTLKRPAPSTTLVGSHSAAKQSPPKLQRHFPVTGRRFGGFQVGRGRARGSGRRRF

>434

MATSSLLPQEAQTSSVYIRHHSLAPPNYRHHFNEGLRFHLFGSGRSCSLLLTLARLRGTAPPSPFFWTCVIQHKDAYQVILDGVKGGTKEKRLAAQFIPKFFKHFPELADSAINAQLDLCEDEDVSIRRQAIKELPQFATGENLPRVADILTQLLQTDDSAEFNLVNNALLSIFKMDAKGTLGGLFSQILQGEDIVRERAIKFLSTKLKTLPDEVLTKEVEELILTESKKVLEDVTGEEFVLFMKILSGLKSLQTVSGRQQLVELVAEQADLEQTFNPSDPDCVDRLLQCTRQAVPLFSKNVHSTRFVTYFCEQVLPNLSSLTTPVEGLDIQLEVLKLLAEMSSFCGDMEKLETNLRKLFDKLLEYMPLPPEEAENGENAGNEEPKLQFSYVECLLYSFHQLGRKLPDFLTAKLNAEKLKDFKIRLQYFARGLQVYIRQLRLALQGKTGEALKTEENKIKVVALKITNNINVLIKDLFHIPPSYKSTVTLSWKPVQKVEIGQKRANEDTTSSSPPKKSPAGPKRDARQIYNPPSGKYSSNLSNFNYEQRGAFRGSRGGRGWGARGNRSRGRLY

>435

MAVTIEDLYRNYGVLADAKDNLSQHKDAYQVILDGVKGGPKEKRLAAQFIPKFFSSFPELADAAINAQLDLCEDEDVSIRRQAIKELPRFATGENILRVADILTQLLQTDDTAEFNQVNASLISIFKMDAKGTLGGLFSQILQGEDIVRERAIKFLSTKLKTLPEDVMTKEVEEYVFAETKKVLEDVTGEEFVLLMRIVSGLRVLQTVNGRQQLVELVVEQAFLEQALNPADPDTVDRLLQCTRQALPLFSKNVHSTRFVTYFCEHVLPNLSALTSPVAELDIQLEVLKLLAEMSPFCGDMEKLEANLNMLFTKLLEFMPLPPEEVENGENSASEEPKLQFSYVECLLFGFHQLGKKLPDFLLDKVDAERLKDFKIRLQYFARGLQVYIRQLRVALQGKTGDALKTDENKIKVVALKITNNINVLIKDLFHNPPSYKSTVTLSWKPVQKAEAVAPKRPSSEDMGSGGSTKKQISPQPRRDARQIYNPPSGKYSASIGNFNYEQRGGFRGGRGRGFGARGSRSRGRIY

>436

MPMVEELYRNYGILADATEQVGQHKDAYQVILDGVKGGTKEKRLAAQFIPKFFKHFPELADSAINAQLDLCEDEDVSIRRQAIKELPQFATGENLPRVADILTQLLQTDDSAEFNLVNNALLSIFKMDAKGTLGGLFSQILQGEDIVRERAIKFLSTKLKTLPDEVLTKEVEELILTESKKVLEDVTGEEFVLFMKILSGLKSLQTVSGRQQLVELVAEQADLEQTFNPSDPDCVDRLLQCTRQAVPLFSKNVHSTRFVTYFCEQVLPNLGTLTTPVEGLDIQLEVLKLLAEMSSFCGDMEKLETNLRKLFDKLLEYMPLPPEEAENGENAGNEEPKLQFSYVECLLYSFHQLGRKLPDFLTAKLNAEKLKDFKIRLQYFARGLQVYIRQLRLALQGKTGEALKTEENKIKVVALKITNNINVLIKDLFHIPPSYKSTVTLSWKPVQKVEIGQKRASEDTTSGSPPKKSSAGPKRDARQIYNPPSGKYSSNLGNFNYEQRGAFRGSRGGRGWGTRGNRSRGRLY

>437

MTTVEELYRNYGILADATDNVGQYKDSYQAILDGVKSGAKEKRLAAQFIPKFFKHFPELADSAINAQLDLCEDEDLSIRRQAIKELPQFATGENLPRVADILTQLLQTDDNAEFNLVNNALISIFKIDAKGTLGGLFTQIMQGEDIVRERAIKFLSTKLKALPEEVMTKEVEEYIFVESKKVLDDVTGEEFVLFMKILSGLKNLQTVSGRQQLVELVAEQADLEQVFNSSDPDSVDRLLQCTRQAVPLFSKNVHSTKFVTYFCEQVLPNLSSLTSPVEGLDIQLEVLKLLAEMTPFCGDMEKLESNLKQLFEKLLEYMPLPPEEAENGENAGNEEPKLQFSYVECLLFGFHQLGKKLPDFLTAKLNADRLKDFKIRLQYFARGLQVYIRQLRLALQGKTGEALKTEENKIKVVALKITNNINVLIKDLFHNPPSYKSTITLSWKPVQKVEGGQKRMASEENASSSPPKKQSPIQKRDTRQIYNPPSGKYSGNLGNFPYEQRGGFRGGRGRGWGGRGNRSRGRLY

>438

MASVEQLYKDFGVLADAKDKANEHTKEYESILSAVKGGTNEKRLAAQFIPRFFKYFPTLAEQAIESQLDLCEDDDVSIRRQAIKELPHLCKDSTEHIPRISDVLTQLLQTEDSSELSIVNTALLTLFKLDAKGTLGGLFSQIISGEESIREHAIKFMCVKMRTVSAELFTKEGEEYLIQKSKEVLVDVTGDEFVSFIKILSGLSSMQTVLGRQQLVDIVVEQADLESEFQPSDADCVDRLMQCVKQALPFFSKNVMSTKIVTYMCDQVIPSLSLLTNPEEDGDIQLEMLHLFAELCHFSGELEDETRVQKVFEKLIEYMPLPPEGEDGENDQTSDEPKLQFSYVECLMYSFHELGRKHADFLTSDEARLKDFRIRLQYFARGCQMYMKQLRLAVQGKKGEELKSEENKIKVVALKVTSNINTLIKDLFHNPPSYKSLITLSWKPIQKPTEDDRQKRPGITPITFDDASHPKKQRGSSMKSRGARQMYQPPSGKFSTKAGSAPTFGDPDYGTGFGNRRGAWRGGGGGRRQRGGWRGGRGNYRY

>439

MAGINEVDLRALMRRAKSSPDKTGTTRRNTLQHLIEATHSPSSSVKIYAAGNMAELFQDFPDLEEEAINAIYDLCEDQDSKVRIEGYRTLALLSRAENKWIKRNADVLVQLLQSDEPNEVNVVRKALVEHLQMDARVTLGVFCDQIVPPDQVMDEEEQAMRDRLRSLVLDFVTNEVKKDQWKKVATPEAEDVLISGLMVALPKAGRTETQIIVQDILLQLQSLNTRSTSAAGLCQCILAKARDARSTDLSVAEKIAPLNHTRPYLELLNILFLQRKLGNLEDLFGFYQPLIAKSVLQRLAPQDQLLIILALTETVVTAKSRDQDQKLAKDILVRAHFCFEVST

>440

MPTVEELYRNYGILADAKDTAPEHKDAYQVILDGVKGGAKEKRLAAQFIPKFFKHFPELADSAINAQLDLCEDEDVSIRRQAIKELPQFAMGDNLPRVADILTQLLQSDDSAEFNLVNNALLSIFKMDAKGTLGGLFSQILQGEDIVRERAIKFLSTKLKILPEEVLTKEVEELILTESKKVLEDVTGEEFVLFMKILSGLKSLQTVSGRQQLVELVAEQADLEQTFNPSDPDCVDRLLQCTRQAVPLFSKNVHSTRFVTYFCEHVLPNLSSLTTPVEGLDIQLEVLKLLAEMSSFCGDMEKLESNLKKLFDKLLEYMPLPPEEAENGENAGNEEPKLQFSYVECLLYSFHQLGRKLTDFLTAKLNTDKVKDFKIRLQYFARGLQVYIRQLRLALQGKTGEALKTEENKIKVVALKITNNINVLIKDLFHIPPSYKSTVTLSWKPVQKSEVGQKRTSEDTSSDLPAKKSQAGPKRDARQIYNPPSGKYSSNLGNFSYEQRGGFRGGRGRGWGGRGNRNRGRIY

>441

MPTVEELYRNYGILADATEQVGQHKDAYQVILDGVKGGTKEKRLAAQFIPKFFKHFPELADSAINAQLDLCEDEDVSIRRQAIKELPQFATGENLPRVADILTQLLQTDDSAEFNLVNNALLSIFKMEKLETNLRKLFDKLLEYMPLPPEEAENGENAGNEEPKLQFSYVECLLYSFHQLGRKLPDFLTAKLNAEKLKDFKIRLQYFARGLQVYIRQLRLALQGKTGEALKTEENKIKVVALKITNNINVLIKDLFHIPPSYKSTVTLSWKPVQKVEIGQKRASEDTTSGSPPKKSSAGPKRDARQIYNPPSGKYSSNLGNFNYERSLQGK

>442

MPTVEELYRNYGILADATEQVGQIRRQAIKELPQFATGENLPRVADILTQLLQTDDSAEFNLVNNALLSIFKMDAKGTLGGLFSQILQGEDIVRERAIKFLSTKLKTLPDEVLTKEVEELILTESKKVLEDVTGEEFVLFMKILSGLKSLQTVSGRQQLVELVAEQADLEQTFNPSDPDCVDRLLQCTRQAVPLFSKNVHSTRFVTYFCEQVLPNLGTLTTPVEGLDIQLEVLKLLAEMSSFCGDMEKLETNLRKLFDKLLEYMPLPPEEAENGENAGNEEPKLQFSYVECLLYSFHQLGRKLPDFLTAKLNAEKLKDFKIRLQYFARGLQVYIRQLRLALQGKTGEALKTEENKIKVVALKITNNINVLIKDLFHIPPSYKSTVTLSWKPVQKVEIGQKRASEDTTSGSPPKKSSAGPKRDARQIYNPPSGKYSSNLGNFNYERSLQGK

>443

MPTVEELYRNYGILADATEQVGQHKDAYQVILDGVKGGTKEKRLAAQFIPKFFKHFPELADSAINAQLDLCEDEDVSIRRQAIKELPQFATGENLPRVADILTQLLQTDDSAEFNLVNNALLSIFKMDAKGTLGGLFSQILQGEDIVRERAIKFLSTKLKTLPDEVLTKEVEELILTESKKVLEDVTGEEFVLFMKILSGLKSLQTVSGRQQLVELVAEQADLEQAFSPSDPDCVDRLLQCTRQAVPLFSKNVHSTRFVTYFCEQVLPNLSTLTTPVEGLDIQLEVLKLLAEMSSFCGDMEKLETNLRKLFDKLLEYMPLPPEEAENGENAGNEEPKLQFSYVECLLYSFHQLGRKLPDFLTAKLNAEKLKDFKIRLQYFARGLQVYIRQLRLALQGKTGEALKTEENKIKVVALKITNNINVLIKDLFHIPPSYKSTVTLSWKPVQKVEIGQKRTSEDTSSGSPPKKSPGGPKRDARQIYNPPSGKYSSNLSNFNYERSLQGK

>444

MPTVEELYRNYGILADATEQVGQHKDAYQVILDGVKGGTKEKRLAAQFIPKFFKHFPELADSAINAQLDLCEDEDVSIRRQAIKELPQFATGENLPRVADILTQLLQTDDSAEFNLVNNALLSIFKMDAKGTLGGLFSQILQGEDIVRERAIKFLSTKLKTLPDEVLTKEVEELILTESKKVLEDVTGEEFVLFMKILSGLKSLQTVSGRQQLVELVAEQADLEQAFSPSDPDCVDRLLQCTRQAVPLFSKNVHSTRFVTYFCEQVLPNLSTLTTPVEGLDIQLEVLKLLAEMSSFCGDMEKLETNLRKLFDKLLEYMPLPPEEAENGENAGNEEPKLQFSYVECLLYSFHQLGRKLPDFLTAKLNAEKLKDFKIRLQYFARGLQVYIRQLRLALQGKTGEALKTEENKIKVVALKITNNINVLIKDLFHIPPSYKSTVTLSWKPVQKVEIGQKRTSEDTSSGSPPKKSPGGPKRDARQIYNPPSGKYSSNLSNFNYEQRGAFRGNRGGRGWGTRGNRSRGRLY

>445

MPTVEELYRNYGILADATEQVGQHKDAYQVILDGVKGGTKEKRLAAQFIPKFFKHFPELADSAINAQLDLCEDEDVSIRRQAIKELPQFATGENLPRVADILTQLLQTDDSAEFNLVNNALLSIFKMDAKGTLGGLFSQILQGEDIVRERAIKFLSTKLKTLPDEVLTKEVEELILTESKKVLEDVTGEEFVLFMKILSGLKSLQTVSGRQQLVELVAEQADLEQAFSPSDPDCVDRLLQCTRQAVPLFSKNVHSTRFVTYFCEQVLPNLSTLTTPVEGLDIQLEVLKLLAEMSSFCGDMEKLETNLRKLFDKLLEYMPLPPEEAENGENAGNEEPKLQFSYVECLLYSFHQLGRKLPDFLTAKLNAEKLKDFKIRLQYFARGLQVYIRQLRLALQGKTGEALKTEENKIKVVALKITNNINVLIKDLFHIPPSYKSTVTLSWKPVQKVEIGQKRTSEDTSSGSPPKKSPGGPKRDARQIYNPPSGKYSSNLSNFNYGERFRLGTSSSRD

>446

MPTVEELYRNYGILADATEQVGQHKDAYQVILDGVKGGTKEKRLAAQFIPKFFKHFPELADSAINAQLDLCEDEDVSIRRQAIKELPQFATGENLPRVADILTQLLQTDDSAEFNLVNNALLSIFKMDAKGTLGGLFSQILQGEDIVRERAIKFLSTKLKTLPDEVLTKEVEELILTESKKVLEDVTGEEFVLFMKILSGLKSLQTVSGRQQLVELVAEQADLEQTFNPSDPDCVDRLLQCTRQAVPLFSKNVHSTRFVTYFCEQVLPNLSSLTTPVEGLDIQLEVLKLLAEMSSFCGDMEKLETNLRKLFDKLLEYMPLPPEEAENGENAGNEEPKLQFSYVECLLYSFHQLGRKLPDFLTAKLNAEKLKDFKIRLQYFARGLQVYIRQLRLALQGKTGEALKTDENKIKVVALKITNNINVLIKDLFHIPPSYKSTVTLSWKPVQKVELGQKRATEDTTSGSPPKKSSAGPKRDARQIYNPPSGKYSSNLSNFNYEQRGAFRGSRGGRGWGARGNRSRGRVY

>447

MKQRWRENGIGKHKDAYQVILDGVKGGTKEKRLAAQFIPKFFKHFPELADSAINAQLDLCEDEDVSIRRQAIKELPQFATGENLPRVADILTQLLQTDDSAEFNLVNNALLSIFKMDAKGTLGGLFSQILQGEDIVRERAIKFLSTKLKTLPDEVLTKEVEELILTESKKVLEDVTGEEFVLFMKILSGLKSLQTVSGRQQLVELVAEQADLEQTFNPSDPDCVDRLLQCTRQAVPLFSKNVHSTRFVTYFCEQVLPNLGTLTTPVEGLDIQLEVLKLLAEMSSFCGDMEKLETNLRKLFDKLLEYMPLPPEEAENGENAGNEEPKLQFSYVECLLYSFHQLGRKLPDFLTAKLNAEKLKDFKIRLQYFARGLQVYIRQLRLALQGKTGEALKTEENKIKVVALKITNNINVLIKDLFHIPPSYKSTVTLSWKPVQKVEIGQKRASEDTTSGSPPKKSSAGPKRDARQIYNPPSGKYSSNLGNFNYERSLQGK

>448

MKQRWRENGIGKHKDAYQVILDGVKGGTKEKRLAAQFIPKFFKHFPELADSAINAQLDLCEDEDVSIRRQAIKELPQFATGENLPRVADILTQLLQTDDSAEFNLVNNALLSIFKMDAKGTLGGLFSQILQGEDIVRERAIKFLSTKLKTLPDEVLTKEVEELILTESKKVLEDVTGEEFVLFMKILSGLKSLQTVSGRQQLVELVAEQADLEQTFNPSDPDCVDRLLQCTRQAVPLFSKNVHSTRFVTYFCEQVLPNLGTLTTPVEGLDIQLEVLKLLAEMSSFCGDMEKLETNLRKLFDKLLEYMPLPPEEAENGENAGNEEPKLQFSYVECLLYSFHQLGRKLPDFLTAKLNAEKLKDFKIRLQYFARGLQVYIRQLRLALQGKTGEALKTEENKIKVVALKITNNINVLIKDLFHIPPSYKSTVTLSWKPVQKVEIGQKRASEDTTSGSPPKKSSAGPKRDARQIYNPPSGKYSSNLGNFNYERGVKKDIKTSP

>449

MPTVEELYRNYGILADATEQVGQHKDAYQVILDGVKGGTKEKRLAAQFIPKFFKHFPELADSAINAQLDLCEDEDVSIRRQAIKELPQFATGENLPRVADILTQLLQTDDSAEFNLVNNALLSIFKMDAKGTLGGLFSQILQGEDIVRERAIKFLSTKLKTLPDEVLTKEVEELILTESKKVLEDVTGEEFVLFMKILSGLKSLQTVSGRQQLVELVAEQADLEQTFNPSDPDCVDRLLQCTRQAVPLFSKNVHSTRFVTYFCEQVLPNLGTLTTPVEGLDIQLEVLKLLAEMSSFCGDMEKLETNLRKLFDKLLEYMPLPPEEAENGENAGNEEPKLQFSYVECLLYSFHQLGRKLPDFLTAKLNAEKLKDFKIRLQYFARGLQVYIRQLRLALQGKTGEALKTEENKIKVVALKITNNINVLIKDLFHIPPSYKSTVTLSWKPVQKVEIGQKRASEDTTSGSPPKKSSAGPKRDARQIYNPPSGKYSSNLGNFNYERGVKKDIKTSP

>450

MPTVEELYRNYGILADATEQVGQHKDAYQVILDGVKGGTKEKRLAAQFIPKFFKHFPELADSAINAQLDLCEDEDVSIRRQAIKELPQFATGENLPRVADILTQLLQTDDSAEFNLVNNALLSIFKMDAKGTLGGLFSQILQGEDIVRERAIKFLSTKLKTLPDEVLTKEVEELILTESKKVLEDVTGEEFVLFMKILSGLKSLQTVSGRQQLVELVAEQADLEQTFSPSDPDCVDRLLQCTRQAVPLFSKNVHSTRFVTYFCEQVLPNLSTLTTPVEGLDIQLEVLKLLAEMSSFCGDMEKLETNLRKLFDKLLEYMPLPPEEAENGENAGNEEPKLQFSYVECLLYSFHQLGRKLPDFLTAKLNAEKLKDFKIRLQYFARGLQVYIRQLRLALQGKTGEALKTEENKIKVVALKITNNINVLIKDLFHIPPSYKSTVTLSWKPVQKVEIGQKRTSEDTSSGSPPKKSPGGPKRDARQIYNPPSGKYSSNLGNFNYEQRGAFRGSRGGRGWGTRGNRSRGRLY

>451

MSSIPVEVTIDDLYKYYDVLAEAKDNAGQHAETYLNIVKGTKAGTKEKLLASQFISRFFKYFPTHMPLAIDAIFDLCEDPDVNIRKSAIKDLASIAKDCSVEHLNRIADILTQLLQTEDVQEFNQVQLSLTSIIKLNPRVTLNEMFNQINTAELDQVRKRGIKFLCGKLPQFFSSATTDPSCSIFNKEIEDLLVKNVKRALSDCDAEEFIMFIRLLASLSSMNSLQGRQELVAIIMEQSELDKKFSSDDLERIMILLSCIQQAIPLFSKNVNSNKYVLMFVDNVLGTFDQIVDEEIKFEILKSLADLCSFFTWTAGMDKVSDKQMDTVYNILLGYLPKPPAEAQTSLCSDLTEDVVDKDKGEHVRFNFSYVECLIYTFHVLARSKQEFLSADQNKDKLKDFRLRLQYFAKGTKNYIKELRNTLINSSLNAKTDADNEENKIRRVALKVTTNIDALIKDLFHNPPAYKTSITLSWKSSQSLEAQDSQQPNKQNKRSLSVTNDSAELEGSAKKKPERGIYQPPTGKYSTQNASNGKRRTLSFDNQKAKNSNKQM

>452

SLYPVFLEIVPPFNPEIVEHNISFDYSSCEAVLFVLHKVGGKYPAFFNDQGRVSEFRLRLQYLSRNAVAYIKTLNEDIKKLKPDDTHIDEKRRALKVLSNIQSIVIDFMKSPPSFSKTILLSFETKKEPQNELVKRKHEIVETEVPSAKREREGKGKGFYKQPYSVPGGKFSSRSDRQGFRGRFNGRRRY

>453

MTDASTDANDIEKLYVYGERLSEAKDKSQNVEDYNSIIEAARSSSIKARQLAAQLIPRFFKFFLGLSGPAVDAHLDLCEAEELGIRVQAIRGLPLFCKDTPEHISKIVDILAQLLIAEENVERDAVHKALLSLLRQDVKASLTALFKHIESVDEPVTDDNLRERTLIFIRDKVFPLKSELLKPQEQMERHITDLIKKSLQDVTGAEFKMFMDFLKSLSIFGAKAPTERVQELVEIIEGQADLDAQFSVSDGDHVDRLISCLYMALPFFIRGASSSKFLNYLNKHILPVFDKLPEERKVDLLKNLAESSPYAAPQDSRQILPSVVQLLKKYMPQKKTGEEMNFTYVECMLYTFHHLANKAPNATNSLCGYKIVTGQPSDRLGEDFTELYKDFTERLNCVEDLTKATMKKLTQGMGQQNKTTGTAISDEEKAKIKIQKQNATTGLRTCNNILAMTQPLHTKSPLFIGDKRINLSWNEAVKSVAPANTAGAKRPANAIKGPSNQPHKRGRGGSNQLVDRAFEGLSYGGRGGSRGGGGQGRGRGRQGRGRGYYR

>454

MAVTIEDLYRSYGVLADAKDNLSPHKDAYQVILDGVKGGPKEKRLAAQFIPKFFSSFPELADSAINAQLDLCEDEDVSIRRQAIKELPRFATGENILRVADILTQLLQTDDTAEFNQVNASLIAIFKIDAKGTLGGLFSQILQGEDIVRERAIKFLSTKLKTLPEDVMTKEVEDYVFAETKKVLEDVTGEEFVLLMRVVSGLRVLQTVNGRQQLVELVVEQAFLEQALNPADPDTVDRLLQCTRQALPLFSKNVHSTRFVTYFCEHVLPNLSSLTSPVAELDIQLEVLKLLAEMSPFCGDMEKLEANLNMLFTKLLEFMPLPPEEVENGENSASEEPKLQFSYVECLLFGFHQLGKKLPDFLLEKVDAERLKDFKIRLQYFARGLQVYIRQLRVALQGKTGDALKTDENKIKVVALKITNNINILIKDLFHNPPSFKSTITLSWKPVQKSEAVAPKRPSGEEIGSGGSTKKQVSPLPRRDARQIYNPPSEQRGGFRGGRGRSFGGRGNRSRGRIY

>455

MAVTIEDLYRSYGVLADAKDNLSPHKDAYQVILDGVKGGPKEKRLAAQFIPKFFSSFPELADSAINAQLDLCEDEDVSIRRQAIKELPRFATGENILRVADILTQLLQTDDTAEFNQVNASLIAIFKIDAKGTLGGLFSQILQGEDIVRERAIKFLSTKLKTLPEDVMTKEVEDYVFAETKKVLEDVTGEEFVLLMRVVSGLRVLQTVNGRQQLVELVVEQAFLEQALNPADPDTVDRLLQCTRQALPLFSKNVHSTRFVTYFCEHVLPNLSSLTSPVAELDIQLEVLKLLAEMSPFCGDMEKLEANLNMLFTKLLEFMPLPPEEVENGENSASEEPKLQFSYVECLLFGFHQLGKKLPDFLLEKVDAERLKDFKIRLQYFARGLQVYIRQLRVALQGKTGDALKTDENKIKVVALKITNNINILIKDLFHNPPSFKSTITLSWKPVQKSEAVAPKRPSGEEIGSGGSTKKQVSPLPRRDARQIYNPPSGKYSATIGNFTYEQRGGFRGGRGRSFGGRGNRSRGRIY

>456

MPTVEELYRNYGILADATEQVAQHKDAYQVILDGVKGGTKEKRLAAQFIPKFFKHFPELADSAINAQLDLCEDEDVSIRRQAIKELPQFATGENLPRVADILTQLLQTDDSAEFNLVNNALLSIFKMDAKGTLGGLFSQILQGEDIVRERAIKFLSTKLKTLPDEVLTKEVEELILTESKKVLEDVTGEEFVLFMKILSGLKSLQTVSGRQQLVELVAEQADLEQTFNPSDPDCVDRLLQCTRQAVPLFSKNVHSTRFVTYFCEQVLPNLSSLTTPVEGLDIQLEVLKLLAEMSSFCGDMEKLETNLRKLFDKLLEYMPLPPEEAENGENAGNEEPKLQFSYVECLLYSFHQLGRKLPDFLTAKLNAEKLKDFKIRLQYFARGLQVYIRQLRLALQGKTGEALKTEENKIKVVALKITNNINVLIKDLFHIPPSYKSTVTLSWKPVQKVEIGQKRANEDTTSGSPPKKSPAGPKRDARQIYNPPSGKYSSNLSNFNYERSLQGK

>457

MPTVEELYRNYGILADATEQVAQHKDAYQVILDGVKGGTKEKRLAAQFIPKFFKHFPELADSAINAQLDLCEDEDVSIRRQAIKELPQFATGENLPRVADILTQLLQTDDSAEFNLVNNALLSIFKMDAKGTLGGLFSQILQGEDIVRERAIKFLSTKLKTLPDEVLTKEVEELILTESKKVLEDVTGEEFVLFMKILSGLKSLQTVSGRQQLVELVAEQADLEQTFNPSDPDCVDRLLQCTRQAVPLFSKNVHSTRFVTYFCEQVLPNLSSLTTPVEGLDIQLEVLKLLAEMSSFCGDMEKLETNLRKLFDKLLEYMPLPPEEAENGENAGNEEPKLQFSYVECLLYSFHQLGRKLPDFLTAKLNAEKLKDFKIRLQYFARGLQVYIRQLRLALQGKTGEALKTEENKIKVVALKITNNINVLIKDLFHIPPSYKSTVTLSWKPVQKVEIGQKRANEDTTSGSPPKKSPAGPKRDARQIYNPPSGKYSSNLSNFNYEQRGAFRGSRGGRGWGARGNRSRGRLY

>458

MPTVEELYRNYGILADATEQVGQHKDAYQVILDGVKGGTKEKRLAAQFIPKFFKHFPELADSAINAQLDLCEDEDVSIRRQAIKELPQFATGENLPRVADILTQLLQTDDSAEFNLVNNALLSIFKMEKLETNLRKLFDKLLEYMPLPPEEAENGENAGNEEPKLQFSYVECLLYSFHQLGRKLPDFLTAKLNAEKLKDFKIRLQYFARGLQVYIRQLRLALQGKTGEALKTDENKIKVVALKITNNINVLIKDLFHIPPSYKSTVTLSWKPVQKVEIGQKRAAEDTTSGSPPKKSPAGPKRDARQIYNPPSGKYSSNLSNFNYERSLQGK

>459

MPTVEELYRNYGILADATEQVGQIRRQAIKELPQFATGENLPRVADILTQLLQTDDSAEFNLVNNALLSIFKMDAKGTLGGLFSQILQGEDIVRERAIKFLSTKLKTLPDEVLTKEVEELILTESKKVLEDVTGEEFVLFMKILSGLKSLQTVSGRQQLVELVAEQADLEQTFNPADPDCVDRLLQCTRQAVPLFSKNVHSTRFVTYFCEHVLPNLSSLTTPVEGLDIQLEVLKLLAEMSSFCGDMEKLETNLRKLFDKLLEYMPLPPEEAENGENAGNEEPKLQFSYVECLLYSFHQLGRKLPDFLTAKLNAEKLKDFKIRLQYFARGLQVYIRQLRLALQGKTGEALKTDENKIKVVALKITNNINVLIKDLFHIPPSYKSTVTLSWKPVQKVEIGQKRAAEDTTSGSPPKKSPAGPKRDARQIYNPPSGKYSSNLSNFNYERSLQGK

>460

MPTVEELYRNYGILADATEQVGQHKDAYQVILDGVKGGTKEKRLAAQFIPKFFKHFPELADSAINAQLDLCEDEDVSIRRQAIKELPQFATGENLPRVADILTQLLQTDDSAEFNLVNNALLSIFKMDAKGTLGGLFSQILQGEDIVRERAIKFLSTKLKTLPDEVLTKEVEELILTESKKVLEDVTGEEFVLFMKILSGLKSLQTVSGRQQLVELVAEQADLEQTFNPADPDCVDRLLQCTRQAVPLFSKNVHSTRFVTYFCEHVLPNLSSLTTPVEGLDIQLEVLKLLAEMSSFCGDMEKLETNLRKLFDKLLEYMPLPPEEAENGENAGNEEPKLQFSYVECLLYSFHQLGRKLPDFLTAKLNAEKLKDFKIRLQYFARGLQVYIRQLRLALQGKTGEALKTDENKIKVVALKITNNINVLIKDLFHIPPSYKSTVTLSWKPVQKVEIGQKRAAEDTTSGSPPKKSPAGPKRDARQIYNPPSGKYSSNLSNFNYERSLQGK

>461

MPTVEELYRNYGILADATEQVGQHKDAYQVILDGVKGGTKEKRLAAQFIPKFFKHFPELADSAINAQLDLCEDEDVSIRRQAIKELPQFATGENLPRVADILTQLLQTDDSAEFNLVNNALLSIFKMDAKGTLGGLFSQILQGEDIVRERAIKFLSTKLKTLPDEVLTKEVEELILTESKKVLEDVTGEEFVLFMKILSGLKSLQTVSGRQQLVELVAEQADLEQTFNPADPDCVDRLLQCTRQAVPLFSKNVHSTRFVTYFCEHVLPNLSSLTTPVEGLDIQLEVLKLLAEMSSFCGDMEKLETNLRKLFDKLLEYMPLPPEEAENGENAGNEEPKLQFSYVECLLYSFHQLGRKLPDFLTAKLNAEKLKDFKIRLQYFARGLQVYIRQLRLALQGKTGEALKTDENKIKVVALKITNNINVLIKDLFHIPPSYKSTVTLSWKPVQKVEIGQKRAAEDTTSGSPPKKSPAGPKRDARQIYNPPSGKYSSNLSNFNYEQRGAFRGSRSGRGWGARGNRSRGRLY

>462

MPTVEELYRNYGILADATEQVGQHKDAYQVILDGVKGGTKEKRLAAQFIPKFFKHFPELADSAINAQLDLCEDEDVSIRRQAIKELPQFATGENLPRVADILTQLLQTDDSAEFNLVNNALLSIFKMDAKGTLGGLFSQILQGEDIVRERAIKFLSTKLKTLPDEVLTKEVEELILTESKKVLEDVTGEEFVLFMKILSGLKSLQTVSGRQQLVELVAEQADLEQTFNPSDPDCVDRLLQCTRQAVPLFSKNVHSTRFVTYFCEQVLPNLSSLTTPVEGLDIQLEVLKLLAEMSSFCGDMEKLETNLRKLFDKLLEYMPLPPEEAENGENAGNEEPKLQFSYVECLLYSFHQLGRKLPDFLTAKLNAEKLKDFKIRLQYFARGLQVYIRQLRLALQGKTGEALKTEENKIKVVALKITNNINVLIKDLFHIPPSYKSTVTLSWKPVQKVEIGQKRANEDTTSGSPPKKSSAGPKRDARQIYNPPSGKYSSNLGNFNYERSLQGK

>463

MPTVEELYRNYGILADATEQVGQHKDAYQVILDGVKGGTKEKRLAAQFIPKFFKHFPELADSAINAQLDLCEDEDVSIRRQAIKELPQFATGENLPRVADILTQLLQTDDSAEFNLVNNALLSIFKMDAKGTLGGLFSQILQGEDIVRERAIKFLSTKLKTLPDEVLTKEVEELILTESKKVLEDVTGEEFVLFMKILSGLKSLQTVSGRQQLVELVAEQADLEQTFNPSDPDCVDRLLQCTRQAVPLFSKNVHSTRFVTYFCEQVLPNLSSLTTPVEGLDIQLEVLKLLAEMSSFCGDMEKLETNLRKLFDKLLEYMPLPPEEAENGENAGNEEPKLQFSYVECLLYSFHQLGRKLPDFLTAKLNAEKLKDFKIRLQYFARGLQVYIRQLRLALQGKTGEALKTEENKIKVVALKITNNINVLIKDLFHIPPSYKSTVTLSWKPVQKVEIGQKRANEDTTSGSPPKKSSAGPKRDARQIYNPPSGKYSSNLGNFNYEQRGAFRGSRGGRSWGARGNRSRGRLY

>464

MADKFEELYQKYNLLSSAKENITELSDEYLECIEHAKGTDKEKKLAAQIIAQYFKHFPQHNEKALSAVFDLCEDGDTSVINKFNS

>465

MPTVEELYRNYGILADATEQVGQHKDAYQVILDGVKGGTKEKRLAAQFIPKFFKHFPELADSAINAQLDLCEDEDVSIRRQAIKELPQFATGENLPRVADILTQLLQTDDSAEFNLVNNALLSIFKMDAKGTLGGLFSQILQGEDIVRERAIKFLSTKLKTLPDEVLTKEVEELILTESKKVLEDVTGEEFVLFMKILSGLKSLQTVSGRQQLVELVAEQADLEQTFNPSDPDCVDRLLQCTRQAVPLFSKNVHSTRFVTYFCEQVLPNLSSLTTPVEGLDIQLEVLKLLAEMSSFCGDMEKLETNLRKLFDKLLEYMPLPPEEAENGENAGNEEPKLQFSYVECLLYSFHQLGRKLPDFLTAKLNAEKLKDFKIRLQYFARGLQVYIRQLRLALQGKTGEALKTDENKIKVVALKITNNINVLIKDLFHIPPSYKSTVTLSWKPVQKVEIGQKRATEDTTSGSPPKKSPAGPKRDARQIYNPPSGKYSSNLGNFNYEH

>466

MPTVEELYRNYGILADATEQVGQHKDAYQVILDGVKGGTKEKRLAAQFIPKFFKHFPELADSAINAQLDLCEDEDVSIRRQAIKELPQFATGENLPRVADILTQLLQTDDSAEFNLVNNALLSIFKMDAKGTLGGLFSQILQGEDIVRERAIKFLSTKLKTLPDEVLTKEVEELILTESKKVLEDVTGEEFVLFMKILSGLKSLQTVSGRQQLVELVAEQADLEQTFNPSDPDCVDRLLQCTRQAVPLFSKNVHSTRFVTYFCEQVLPNLSSLTTPVEGLDIQLEVLKLLAEMSSFCGDMEKLETNLRKLFDKLLEYMPLPPEEAENGENAGNEEPKLQFSYVECLLYSFHQLGRKLPDFLTAKLNAEKLKDFKIRLQYFARGLQVYIRQLRLALQGKTGEALKTDENKIKVVALKITNNINVLIKDLFHIPPSYKSTVTLSWKPVQKVEIGQKRATEDTTSGSPPKKSPAGPKRDARQIYNPPSGKYSSNLGNFNYEANSLF

>467

MPTVEELYRNYGILADATEQVGQHKDAYQVILDGVKGGTKEKRLAAQFIPKFFKHFPELADSAINAQLDLCEDEDVSIRRQAIKELPQFATGENLPRVADILTQLLQTDDSAEFNLVNNALLSIFKMDAKGTLGGLFSQILQGEDIVRERAIKFLSTKLKTLPDEVLTKEVEELILTESKKVLEDVTGEEFVLFMKILSGLKSLQTVSGRQQLVELVAEQADLEQTFNPSDPDCVDRLLQCTRQAVPLFSKNVHSTRFVTYFCEQVLPNLSSLTTPVEGLDIQLEVLKLLAEMSSFCGDMEKLETNLRKLFDKLLEYMPLPPEEAENGENAGNEEPKLQFSYVECLLYSFHQLGRKLPDFLTAKLNAEKLKDFKIRLQYFARGLQVYIRQLRLALQGKTGEALKTDENKIKVVALKITNNINVLIKDLFHIPPSYKSTVTLSWKPVQKVEIGQKRATEDTTSGSPPKKSPAGPKRDARQIYNPPSGKYSSNLGNFNYERSLQGK

>468

MPTVEELYRNYGILADATEQVGQHKDAYQVILDGVKGGTKEKRLAAQFIPKFFKHFPELADSAINAQLDLCEDEDVSIRRQAIKELPQFATGENLPRVADILTQLLQTDDSAEFNLVNNALLSIFKMDAKGTLGGLFSQILQGEDIVRERAIKFLSTKLKTLPDEVLTKEVEELILTESKKVLEDVTGEEFVLFMKILSGLKSLQTVSGRQQLVELVAEQADLEQTFNPSDPDCVDRLLQCTRQAVPLFSKNVHSTRFVTYFCEQVLPNLSSLTTPVEGLDIQLEVLKLLAEMSSFCGDMEKLETNLRKLFDKLLEYMPLPPEEAENGENAGNEEPKLQFSYVECLLYSFHQLGRKLPDFLTAKLNAEKLKDFKIRLQYFARGLQVYIRQLRLALQGKTGEALKTDENKIKVVALKITNNINVLIKDLFHIPPSYKSTVTLSWKPVQKVEIGQKRATEDTTSGSPPKKSPAGPKRDARQIYNPPSGKYSSNLGNFNYEQRGAFRGSRGGRGWGARGNRSRGRLY

>469

MPTVEELYRNYGILADATEQVGQHKDAYQVILDGVKGGTKEKRLAAQFIPKFFKHFPELADSAINAQLDLCEDEDVSIRRQAIKELPQFATGENLPRVADILTQLLQTDDSAEFNLVNNALLSIFKMDAKGTLGGLFSQILQGEDIVRERAIKFLSTKLKTLPDEVLTKEVEELILTESKKVLEDVTGEEFVLFMKILSGLKSLQTVSGRQQLVELVAEQADLEQTFNPSDPDCVDRLLQCTRQAVPLFSKNVHSTRFVTYFCEQVLPNLSSLTTPVEGLDIQLEVLKLLAEMSSFCGDMEKLETNLRKLFDKLLEYMPLPPEEAENGENAGNEEPKLQFSYVECLLYSFHQLGRKLPDFLTAKLNAEKLKDFKIRLQYFARGLQVYIRQLRLALQGKTGEALKTEENKIKVVALKITNNINVLIKDLFHIPPSYKSTVTLSWKPVQKVEIGQKRANEDTTSGSPPKKSSAXXXXXARQIYNPPSGKYSSNLGNFNYERSLQGK

>470

MPTVEELYRNYGILADATEQVGQHKDAYQVILDGVKGGTKEKRLAAQFIPKFFKHFPELADSAINAQLDLCEDEDVSIRRQAIKELPQFATGENLPRVADILTQLLQTDDSAEFNLVNNALLSIFKMDAKGTLGGLFSQILQGEDIVRERAIKFLSTKLKTLPDEVLTKEVEELILTESKKVLEDVTGEEFVLFMKILSGLKSLQTVSGRQQLVELVAEQADLEQTFNPSDPDCVDRLLQCTRQAVPLFSKNVHSTRFVTYFCEQVLPNLSSLTTPVEGLDIQLEVLKLLAEMSSFCGDMEKLETNLRKLFDKLLEYMPLPPEEAENGENAGNEEPKLQFSYVECLLYSFHQLGRKLPDFLTAKLNAEKLKDFKIRLQYFARGLQVYIRQLRLALQGKTGEALKTEENKIKVVALKITNNINVLIKDLFHIPPSYKSTVTLSWKPVQKVEIGQKRANEDTTSGSPPKKSSAXXXXXARQIYNPPSGKYSSNLGNFNYEQRGAFRGSRGGRGWGARGNRSRGRLY

>471

MPTVEELYRNYGILADATEQVGQHKDAYQVILDGVKGGTKEKRLAAQFIPKFFKHFPELADSAINAQLDLCEDEDVSIRRQAIKELPQFATGENLPRVADILTQLLQTDDSAEFNLVNNALLSIFKMDAKGTLGGLFSQILQGEDIVRERAIKFLSTKLKTLPDEVLTKEVEELILTESKKVLEDVTGEEFVLFMKILSGLKSLQTVSGRQQLVELVAEQADLEQTFNPSDPDCVDRLLQCTRQAVPLFSKNVHSTRFVTYFCEQVLPNLSSLTTPVEGLDIQLEVLKLLAEMSSFCGDMEKLETNLRKLFDKLLEYMPLPPEEAENGENAGNEEPKLQFSYVECLLYSFHQLGRKLPDFLTAKLNAEKLKDFKIRLQYFARGLQVYIRQLRLALQGKTGEALKTEENKIKVVALKITNNINVLIKDLFHIPPSYKSTVTLSWKPVQKVEIGQKRANEDTTSGSPPKKSPAGPKRDARQIYNPPSGKYSSNLSNFNYERSLQGK

>472

MPTVEELYRNYGILADATEQVGQHKDAYQVILDGVKGGTKEKRLAAQFIPKFFKHFPELADSAINAQLDLCEDEDVSIRRQAIKELPQFATGENLPRVADILTQLLQTDDSAEFNLVNNALLSIFKMDAKGTLGGLFSQILQGEDIVRERAIKFLSTKLKTLPDEVLTKEVEELILTESKKVLEDVTGEEFVLFMKILSGLKSLQTVSGRQQLVELVAEQADLEQTFNPSDPDCVDRLLQCTRQAVPLFSKNVHSTRFVTYFCEQVLPNLSSLTTPVEGLDIQLEVLKLLAEMSSFCGDMEKLETNLRKLFDKLLEYMPLPPEEAENGENAGNEEPKLQFSYVECLLYSFHQLGRKLPDFLTAKLNAEKLKDFKIRLQYFARGLQVYIRQLRLALQGKTGEALKTEENKIKVVALKITNNINVLIKDLFHIPPSYKSTVTLSWKPVQKVEIGQKRANEDTTSGSPPKKSPAGPKRDARQIYNPPSGKYSSNLSNFNYEQRGAFRGSRGGRGWGARGNRSRGRLY

>473

MPTVEELYRNYGILADATEQVGQHKDAYQVILDGVKGGTKEKRLAAQFIPKFFKHFPELADSAINAQLDLCEDEDVSIRRQAIKELPQFATGENLPRVADILTQLLQTDDSAEFNLVNNALLSIFKMDAKGTLGGLFSQILQGEDIVRERAIKFLSTKLKTLPDEVLTKEVEELILTESKKVLEDVTGEEFVLFMKILSGLKSLQTVSGRQQLVELVAEQADLEQTFNPSDPDCVDRLLQCTRQAVPLFSKNVHSTRFVTYFCEQVLPNLSSLTTPVEGLDIQLEVLKLLAEMSSFCGDMEKLETNLRKLFDKLLEYMPLPPEEAENGENAGNEEPKLQFSYVECLLYSFHQLGRKLPDFLTAKLNAEKLKDFKIRLQYFARGLQVYIRQLRLALQGKTGEALKTDENKIKVVALKITNNINVLIKDLFHIPPSYKSTVTLSWKPVQKVELGQKRASEDTTSGSPPKKSSAGPKRDARQIYNPPSGKYSSNLSNFNYERSLQGK

>474

MPTVEELYRNYGILADATEQVGQHKDAYQVILDGVKGGTKEKRLAAQFIPKFFKHFPELADSAINAQLDLCEDEDVSIRRQAIKELPQFATGENLPRVADILTQLLQTDDSAEFNLVNNALLSIFKMDAKGTLGGLFSQILQGEDIVRERAIKFLSTKLKTLPDEVLTKEVEELILTESKKVLEDVTGEEFVLFMKILSGLKSLQTVSGRQQLVELVAEQADLEQTFNPSDPDCVDRLLQCTRQAVPLFSKNVHSTRFVTYFCEQVLPNLSSLTTPVEGLDIQLEVLKLLAEMSSFCGDMEKLETNLRKLFDKLLEYMPLPPEEAENGENAGNEEPKLQFSYVECLLYSFHQLGRKLPDFLTAKLNAEKLKDFKIRLQYFARGLQVYIRQLRLALQGKTGEALKTDENKIKVVALKITNNINVLIKDLFHIPPSYKSTVTLSWKPVQKVELGQKRASEDTTSGSPPKKSSAGPKRDARQIYNPPSGKYSSNLSNFNYEQRGAFRGSRGGRGWGARGNRSRGRLY

>475

MPTVEELYRNYGILADATEQVGQHKDAYQVILDGVKGGTKEKRLAAQFIPKFFKHFPELADSAINAQLDLCEDEDVSIRRQAIKELPQFATGENLPRVADILTQLLQTDDSAEFNLVNNALLSIFKMDAKGTLGGLFSQILQGEDIVRERAIKFLSTKLKTLPDEVLTKEVEELILTESKKVLEDVTGEEFVLFMKILSGLKSLQTVSGRQQLVELVAEQADLEQTFNPSDPDCVDRLLQCTRQAVPLFSKNVHSTRFVTYFCEQVLPNLSTLTTPVEGLDIQLEVLKLLAEMSSFCGDMEKLETNLRKLFDKLLEYMPLPPEEAENGENAGNEEPKLQFSYVECLLYSFHQLGRKLPDFLTAKLNAEKLKDFKIRLQYFARGLQVYIRQLRLALQGKTGEALKTEENKIKVVALKITNNINVLIKDLFHIPPSYKSTVTLSWKPVQKVEIGQKRANEDTTSGSPPKKSPAGPKRDARQIYNPPSGKYSSNLGNFNYERSLQGK

>476

MPTVEELYRNYGILADATEQVGQHKDAYQVILDGVKGGTKEKRLAAQFIPKFFKHFPELADSAINAQLDLCEDEDVSIRRQAIKELPQFATGENLPRVADILTQLLQTDDSAEFNLVNNALLSIFKMDAKGTLGGLFSQILQGEDIVRERAIKFLSTKLKTLPDEVLTKEVEELILTESKKVLEDVTGEEFVLFMKILSGLKSLQTVSGRQQLVELVAEQADLEQTFNPSDPDCVDRLLQCTRQAVPLFSKNVHSTRFVTYFCEQVLPNLSTLTTPVEGLDIQLEVLKLLAEMSSFCGDMEKLETNLRKLFDKLLEYMPLPPEEAENGENAGNEEPKLQFSYVECLLYSFHQLGRKLPDFLTAKLNAEKLKDFKIRLQYFARGLQVYIRQLRLALQGKTGEALKTEENKIKVVALKITNNINVLIKDLFHIPPSYKSTVTLSWKPVQKVEIGQKRANEDTTSGSPPKKSPAGPKRDARQIYNPPSGKYSSNLGNFNYEQRGAFRGSRGGRGWGTRGNRSRGRLY

>477

MPTVEELYRNYGILADATEQVGQHKDAYQVILDGVKGGTKEKRLAAQFIPKFFKHFPELADSAINAQLDLCEDEDVSIRRQAIKELPQFATGENLPRVADILTQLLQTDDSAEFNLVNNALLSIFKMDAKGTLGGLFSQILQGEDIVRERAIKFLSTKLKTLPDEVLTKEVEELILTESKKVLEDVTGEEFVLFMKILSGLKSLQTVSGRQQLVELVAEQADLEQTFNPADPDCVDRLLQCTRQAVPLFSKNVHSTRFVTYFCEHVLPNLSSLTTPVEGLDIQLEVLKLLAEMSSFCGDMEKLETNLKKLFDKLLEYMPLPPEEAENGENAGNEEPKLQFSYVECLLYSFHQLGRKLPDFLTAKLNAEKLKDFKIRLQYFARGLQVYIRQLRLALQGKTGEALKTEENKIKVVALKITNNINVLIKDLFHIPPSYKSTVTLSWKPVQKAEMGQKRTNEDTTSGSPPKKAAAGPKRDARQIYNPPSGKYSSNLGNFNYERRGLQGK

>478

MPTVEELYRNYGILADATEQVGQHKDAYQVILDGVKGGTKEKRLAAQFIPKFFKHFPELADSAINAQLDLCEDEDVSIRRQAIKELPQFATGENLPRVADILTQLLQTDDSAEFNLVNNALLSIFKMDAKGTLGGLFSQILQGEDIVRERAIKFLSTKLKTLPDEVLTKEVEELILTESKKVLEDVTGEEFVLFMKILSGLKSLQTVSGRQQLVELVAEQADLEQTFNPADPDCVDRLLQCTRQAVPLFSKNVHSTRFVTYFCEHVLPNLSSLTTPVEGLDIQLEVLKLLAEMSSFCGDMEKLETNLKKLFDKLLEYMPLPPEEAENGENAGNEEPKLQFSYVECLLYSFHQLGRKLPDFLTAKLNAEKLKDFKIRLQYFARGLQVYIRQLRLALQGKTGEALKTEENKIKVVALKITNNINVLIKDLFHIPPSYKSTVTLSWKPVQKAEMGQKRTNEDTTSGSPPKKAAAGPKRDARQIYNPPSGKYSSNLGNFNYEQRGGGFRGSRGGRGWGGRGNRSRGRLY

>479

MHKDAYQVILDGVKGGAKEKRLAAQFIPKFFKHFPELADSAINAQLDLCEDEDVSIRRQAIKELPQFATGDNLPRVADILTQLLQSDDSAEFNLVNNALLSIFKMDAKGTLGGLFSQILQGEDIVRERAIKFLSTKLKTLPEEVMTKEVEEFILTESKKVLEDVTGEEFNLFMKILSGLKSLQTVSGRQQLVELVAEQADLEQTFNPSDPDCVDRLQQCTRQAVPLFSKNVHSTKFVTYFCEHVLPNLSSLTTSVEGLDIQLEVLKLLAEMSSFCGDMEKLESNLKKLFDKLLEYMPLPPEEAENGENAGNEEPKLQFSHVECLLYSFHQLGRKLPDFLTAKLNAEKLKDFKIRLQYFARGLQVYIRQLRLALQGKTGEALKTEENKIKVVALKITNNINVLIKDLFHIPPSYKSTITLSWKPVQKADASQKRASEDTTSSSPPKKASAGPKRDARQIYNPPSGKYSSNLGSFSYEQRGGFRGGRGRGWGGRGNRSRGRIY

>480

MVMHKDAYQVILDGVKGGAKEKRLAAQFIPKFFKHFPELADSAINAQLDLCEDEDVSIRRQAIKELPQFATGDNLPRVADILTQLLQSDDSAEFNLVNNALLSIFKMDAKGTLGGLFSQILQGEDIVRERAIKFLSTKLKTLPEEVMTKEVEEFILTESKKVLEDVTGEEFNLFMKILSGLKSLQTVSGRQQLVELVAEQADLEQTFNPSDPDCVDRLQQCTRQAVPLFSKNVHSTKFVTYFCEHVLPNLSSLTTQAEDLEIQLEVLKLLAEMSSFCGDMEKLESNLKKLFDKLLEYMPLPPEEAENGENAGNEEPKLQFSHVECLLYSFHQLGRKLPDFLTAKLNAEKLKDFKIRLQYFARGLQVYIRQLRLALQGKTGEALKTEENKIKVVALKITNNINVLIKDLFHIPPSYKSTITLSWKPVQKADASQKRASEDTTSSSPPKKASAGPKRDARQIYNPPSGKYSSNLGSFSYEQRGGFRGGRGRGWGGRGNRSRGRIY

>481

MPTVEELYRNYGILADATETAGQHKDAYQVILDGVKGGAKEKRLAAQFIPKFFKHFPELADSAINAQLDLCEDEDVSIRRQAIKELPQFATGDNLPRVADILTQLLQSDDSAEFNLVNNALLSIFKMDAKGTLGGLFSQILQGEDIVRERAIKFLSTKLKTLPEEVMTKEVEEFILTESKKVLEDVTGEEFNLFMKILSGLKSLQTVSGRQQLVELVAEQADLEQTFNPSDPDCVDRLQQCTRQAVPLFSKNVHSTKFVTYFCEHVLPNLSSLTTQAEDLEIQLEVLKLLAEMSSFCGDMEKLESNLKKLFDKLLEYMPLPPEEAENGENAGNEEPKLQFSHVECLLYSFHQLGRKLPDFLTAKLNAEKLKDFKIRLQYFARGLQVYIRQLRLALQGKTGEALKTEENKIKVVALKITNNINVLIKDLFHIPPSYKSTITLSWKPVQKADASQKRASEDTTSSSPPKKASAGPKRDARQIYNPPSGKYSSNLGSFSYEQRGGFRGGRGRGWGGRGNRSRGRIY

>482

MPTVEELYRNYGILADATEQVGQHKDAYQVILDGVKGGTKEKRLAAQFIPKFFKHFPELADSAINAQLDLCEDEDVSIRRQAIKELPQFATGENLPRVADILTQLLQTDDSAEFNLVNNALLSIFKMDAKGTLGGLFSQILQGEDIVRERAIKFLSTKLKTLPDEVLTKEVEELILTESKKVLEDVTGEEFVLFMKILSGLKSLQTVSGRQQLVELVAEQADLEQTFNPSDPDCVDRLLQCTRQAVPLFSKNVHSTRFVTYFCEQVLPNLSSLTTPVEGLDIQLEVLKLLAEMSSFCGDMEKLETNLRKLFDKLLEYMPLPPEEAENGENAGNEEPKLQFSYVECLLYSFHQLGRKLPDFLTAKLNAEKLKDFKIRLQYFARGLQVYIRQLRLALQGKTGEALKTEENKIKVVALKITNNINVLIKDLFHIPPSYKSTVTLSWKPVQKVEIGQKRANEDTTSGSPPKKSPAGPKRDARQIYNPPSGKYSSNLSNFNYGN

>483

MPTVEELYRNYGILADATEQVGQHKDAYQVILDGVKGGTKEKRLAAQFIPKFFKHFPELADSAINAQLDLCEDEDVSIRRQAIKELPQFATGENLPRVADILTQLLQTDDSAEFNLVNNALLSIFKMDAKGTLGGLFSQILQGEDIVRERAIKFLSTKLKTLPDEVLTKEVEELILTESKKVLEDVTGEEFVLFMKILSGLKSLQTVSGRQQLVELVAEQADLEQTFNPSDPDCVDRLLQCTRQAVPLFSKNVHSTRFVTYFCEQVLPNLSSLTTPVEGLDIQLEVLKLLAEMSSFCGDMEKLETNLRKLFDKLLEYMPLPPEEAENGENAGNEEPKLQFSYVECLLYSFHQLGRKLPDFLTAKLNAEKLKDFKIRLQYFARGLQVYIRQLRLALQGKTGEALKTDENKIKVVALKITNNINVLIKDLFHIPPSYKSTVTLSWKPVQKVELGQKRATEDTTSGSPPKKSSAGPKRDARQIYNPPSGKYSSNLSNFNYEQRGAFRGSRGGRGWGARGNRSRGRLY

>484

MPTVEELYRNYGILADATEQVGQHKDAYQVILDGVKGGTKEKRLAAQFIPKFFKHFPELADSAINAQLDLCEDEDVSIRRQAIKELPQFATGENLPRVADILTQLLQTDDSAEFNLVNNALLSIFKMDAKGTLGGLFSQILQGEDIVRERAIKFLSTKLKTLPDEVLTKEVEELILTESKKVLEDVTGEEFVLFMKILSGLKSLQTVSGRQQLVELVAEQADLEQTFSPSDPDCVDRLLQCTRQAVPLFSKNVHSTRFVTYFCEQVLPNLSTLTTPVEGLDIQLEVLKLLAEMSSFCGDMEKLETNLRKLFDKLLEYMPLPPEEAENGENASNEEPKLQFSYVECLLYSFHQLGRKLPDFLTAKLNAEKLKDFKIRLQYFARGLQVYIRQLRLALQGKTGEALKTEENKIKVVALKITNNINVLIKDLFHIPPSYKSTVTLSWKPVQKVEIGQKRANEDTSSGSPPKKSPGGPKRDARQIYNPPSGKYSSNLGNFNYERSLQGK

>485

MPTVEELYRNYGILADATEQVGQHKDAYQVILDGVKGGTKEKRLAAQFIPKFFKHFPELADSAINAQLDLCEDEDVSIRRQAIKELPQFATGENLPRVADILTQLLQTDDSAEFNLVNNALLSIFKMDAKGTLGGLFSQILQGEDIVRERAIKFLSTKLKTLPDEVLTKEVEELILTESKKVLEDVTGEEFVLFMKILSGLKSLQTVSGRQQLVELVAEQADLEQTFSPSDPDCVDRLLQCTRQAVPLFSKNVHSTRFVTYFCEQVLPNLSTLTTPVEGLDIQLEVLKLLAEMSSFCGDMEKLETNLRKLFDKLLEYMPLPPEEAENGENASNEEPKLQFSYVECLLYSFHQLGRKLPDFLTAKLNAEKLKDFKIRLQYFARGLQVYIRQLRLALQGKTGEALKTEENKIKVVALKITNNINVLIKDLFHIPPSYKSTVTLSWKPVQKVEIGQKRANEDTSSGSPPKKSPGGPKRDARQIYNPPSGKYSSNLGNFNYGERFRLGTSSPRD

>486

MPTVEELYRNYGILADATEQVGQHKDAYQVILDGVKGGTKEKRLAAQFIPKFFKHFPELADSAINAQLDLCEDEDVSIRRQAIKELPQFATGENLPRVADILTQLLQTDDSAEFNLVNNALLSIFKMDAKGTLGGLFSQILQGEDIVRERAIKFLSTKLKTLPDEVLTKEVEELILTESKKVLEDVTGEEFVLFMKILSGLKSLQTVSGRQQLVELVAEQADLEQTFSPSDPDCVDRLLQCTRQAVPLFSKNVHSTRFVTYFCEQVLPNLSTLTTPVEGLDIQLEVLKLLAEMSSFCGDMEKLETNLRKLFDKLLEYMPLPPEEAENGENASNEEPKLQFSYVECLLYSFHQLGRKLPDFLTAKLNAEKLKDFKIRLQYFARGLQVYIRQLRLALQGKTGEALKTEENKIKVVALKITNNINVLIKDLFHIPPSYKSTVTLSWKPVQKVEIGQKRANEDTSSGSPPKKSPGGPKRDARQIYNPPSGKYSSNLGNFNYEQRGAFRGSRGGRGWGTRGNRSRGRLY

>487

MPTVEELYRNYGILADATEQVGQHKDAYQVILDGVKGGTKEKRLAAQFIPKFFKHFPELADSAINAQLDLCEDEDVSIRRQAIKELPQFATGENLPRVADILTQLLQTDDSAEFNLVNNALLSIFKMDAKGTLGGLFSQILQGEDIVRERAIKFLSTKLKTLPDEVLTKEVEELILTESKKVLEDVTGEEFVLFMKILSGLKSLQTVSGRQQLVELVAEQADLEQTFNPSDPDCVDRLLQCTRQAVPLFSKNVHSTRFVTYFCEQVLPNLSSLTTPVEGLDIQLEVLKLLAEMSSFCGDMEKLETNLRKLFDKLLEYMPLPPEEAENGENAGNEEPKLQFSYVECLLYSFHQLGRKLPDFLTAKLNAEKLKDFKIRLQYFARGLQVYIRQLRLALQGKTGEALKTEENKIKVVALKITNNINVLIKDLFHIPPSYKSTVTLSWKPVQKVEIGQKRANEDTTSGSPPKKSSAGPKRDARQIYNPPSGKYSSNLGNFNYEQRGAFRGSRGGRGWGARGNRSRGRLY

>488

MDNIERLYKCYEILSEAGDKISEHVDEYKEILKAVKGSSKEKRLASQFIGNFFKHFPDLAETAIDAQFDLCEDDDNQIRRQAIKDLPKLCQGNPDATTRVADTLAQLLILDDATELQQVNNSLLSIIKMDTKSVVTGIFQQINTGDEPTRERCFKFISTKLLTMGPTVITKEIEEYIVEEIKKALQDVTADEFHLCMTILGATKLGNTITGHAELVKLATEQAELNNTDTDIIAVDDEVVERFVQCATAAAPYFSKTIKSTAFVAHVCDKLLPIPTWNMIATAVSQDQIQLRLLKVFAEMITNTDKLENANERINNVYNVLLEYMPLPKLSEVDLVDVPPSFEFSHAECLLYALHTLGKKHPTNLSFVEDAEKLKDFRARLQYLARGTQGYIKKLEEALKGKSAEELKTEENQLKQTALKTTSNINVLIRDLFHSPPIFKHDIVLSWIVPKANKLGKRHAPITFGEKPEANGKEKDQDVEKKSRPSNDQKFYSPPSGKYSHKVNPNYGNNNRGRQRGGGGGYRNRRYNRY

>489

MDNIERLYKCYEILSEAGDKISEHVDEYTEILKAVKGTSKEKRLASQFIGNFFKHFPDLADTAIDAQFDLCEDDDTQIRRQAIKDLPKLCQGNADATIRVGDTLAQLLILDDPTELQQVNNSLLAIIKLDTKSSVTGLFQQIATGDETTRERCLKFIATKLLTMGPTVITKEIEDFIVEEIKKALQDVTADEFHLCMTILGATKLGSTITGHAELVKLATEQAELNNTDTDIIAVDDEVVERFIQCATAAAPYFSKTIKSTAFVAHVCDKLLPIKTWNMIATAVSQDQIQLRLLKVFAEMITNTDKLDNASERINAVYHVLLEYMPLPKLSDEDLGDTPPSFQFSHAECLLYALHTLGKNHPNSLSFVEDAEKLKDFRARLQYLARGTQGYIKKLEEALKGKTGEELKTEENQLKQTALKTTSNINVLIRDLFHSPPIFKHDIVLSWIVPKNSKLGKRHAPITFGEKGAANGKEKDQEPEKKARASNDQKFYSPPSGKYSNKVNQNYGNNNRTRQRGGGGGSGGGGGGGYRNRRFNKY

>490

MSPEGLLASPDRRLAPGAVVGHKDAYQAILDGVKGGAKEKRLAAQFIPKFFKHFPELADSAINAQLDLCEDEDVSIRRQAIKELPQFATGDNLPRVADILTQLLQSDDSAEFNLVNNALLSIFKMDAKGTLGGLFSQILQGEDIVRERAIKFLSTKLKTLPEEVMTKEVEEFILTESKKVLEDVTGEEFVLFMKILSGLKSLQTVSGRQQLVELVAEQADLEQTFNPTDPDCVDRLLQCTRQAVPLFSKNVHSTRFVTYFCEHVLPNLSSLTTPVEGLDIQLEVLKLLAEMSSFCGDMEKLESNLKKLFDKLLEYMPLPPEEAENGENAGNEEPKLQFSYVECLLYSFHQLGRKLPDFLTAKLNAEKLKDFKIRLQYFARGLQVYIRQLRLALQGKTGEALKTEENKIKVVALKITNNINVLIKDLFHIPPSYKSTVTLSWKPVQKADASQKRASEDTTSSSPPKKASAGPKRDARQIYNPPSGKYSSNLGSFSYEQRGGFRGGRGRGWGGRGNRSRGRIY

>491

MSPEGLLASPDRRLAPGAVVGVCFLTTGRRGHKDAYQAILDGVKGGAKEKRLAAQFIPKFFKHFPELADSAINAQLDLCEDEDVSIRRQAIKELPQFATGDNLPRVADILTQLLQSDDSAEFNLVNNALLSIFKMDAKGTLGGLFSQILQGEDIVRERAIKFLSTKLKTLPEEVMTKEVEEFILTESKKVLEDVTGEEFVLFMKILSGLKSLQTVSGRQQLVELVAEQADLEQTFNPTDPDCVDRLLQCTRQAVPLFSKNVHSTRFVTYFCEHVLPNLSSLTTPVEGLDIQLEVLKLLAEMSSFCGDMEKLESNLKKLFDKLLEYMPLPPEEAENGENAGNEEPKLQFSYVECLLYSFHQLGRKLPDFLTAKLNAEKLKDFKIRLQYFARGLQVYIRQLRLALQGKTGEALKTEENKIKVVALKITNNINVLIKDLFHIPPSYKSTVTLSWKPVQKADASQKRASEDTTSSSPPKKASAGPKRDARQIYNPPSGKYSSNLGSFSYEQRGGFRGGRGRGWGGRGNRSRGRIY

>492

MEAPSNALEENGGSHGNKRRAPISFGAGDKDGLSAAKSRPGVDSVKNRPIYTPPSGKFSTNLSYQQSREGGGRRPSGGGRGGGFRGGFRGGRRWSGPYPYTTEQDAYNPYVAFFPDFYFC

>493

MYLYRTDKKGYRNEAPGKSISQGKEKECEHRPGEHKDAYQVILDGVKGGAKEKRLAAQFIPKFFKHFPELADSAINAQLDLCEDEDVSIRRQAIKELPQFATGDNLPRVADILTQLLQSDDSAEFNLVNNALLSIFKMDAKGTLGGLFSQILQGEDIVRERAIKFLSTKLKTLPEEVMTKEVEEFILTESKKVLEDVTGEEFVLFMKILSGLKSLQTVSGRQQLVELVAEQADLEQTFNPSDTDCVDRLLQCTRQAVPLFSKNVHSTKFVTYFCEHVLPNLSSLTTLVEGLDIQLEVLKLLAEMSSFCGDMEKLESNLKKLFDKLLEYMPLPPEEAENGENAGNEEPKLQFSYVECLLYSFHQLGRKLPDFLTAKLNAEKLKDFKIRLQYFARGLQVYIRQLRLALQGKTGEALKTEENKIKVVALKITNNINVLIKDLFHIPPSYKSTVTLSWKPVQKADASQKRASEDTTSSSPPKKASAGPKRDARQIYNPPSGKYSSNLGSFSYEQRGGFRGGRGRGWGGRGNRSRGRIY

>494

MPTVEELYRNYGILADATEQVGQHKDAYQVILDGVKGGTKEKRLAAQFIPKFFKHFPELADSAINAQLDLCEDEDVSIRRQAIKELPQFATGENLPRVADILTQLLQTDDSAEFNLVNNALLSIFKMDAKGTLGGLFSQILQGEDIVRERAIKFLSTKLKTLPDEVLTKEVEELILTESKKVLEDVTGEEFVLFMKILSGLKSLQTVSGRQQLVELVAEQADLEQTFSPSDPDCVDRLLQCTRQAVPLFSKNVHSTRFVTYFCEQVLPNLSTLTTPVEGLDIQLEVLKLLAEMSSFCGDMEKLETNLRKLFDKLLEYMPLPPEEAENGENAGNEEPKLQFSYVECLLYSFHQLGRKLPDFLTAKLNAEKLKDFKIRLQYFARGLQVYIRQLRLALQGKTGEALKTEENKIKVVALKITNNINVLIKDLFHIPPSYKSTVTLSWKPVQKVEIGQKRASEDTSSGSPPKKSPGGPKRDARQIYNPPSGKYSSNLGNFNYERSLQGK

>495

MPTVEELYRNYGILADATEQVGQHKDAYQVILDGVKGGTKEKRLAAQFIPKFFKHFPELADSAINAQLDLCEDEDVSIRRQAIKELPQFATGENLPRVADILTQLLQTDDSAEFNLVNNALLSIFKMDAKGTLGGLFSQILQGEDIVRERAIKFLSTKLKTLPDEVLTKEVEELILTESKKVLEDVTGEEFVLFMKILSGLKSLQTVSGRQQLVELVAEQADLEQTFSPSDPDCVDRLLQCTRQAVPLFSKNVHSTRFVTYFCEQVLPNLSTLTTPVEGLDIQLEVLKLLAEMSSFCGDMEKLETNLRKLFDKLLEYMPLPPEEAENGENAGNEEPKLQFSYVECLLYSFHQLGRKLPDFLTAKLNAEKLKDFKIRLQYFARGLQVYIRQLRLALQGKTGEALKTEENKIKVVALKITNNINVLIKDLFHIPPSYKSTVTLSWKPVQKVEIGQKRASEDTSSGSPPKKSPGGPKRDARQIYNPPSGKYSSNLGNFNYEQRGAFRGSRGGRGWGTRGNRSRGRLY

>496

MSVDNIEKLYKYFDILESAKDKIDQHEAEYLAILEAVKGEQKEKRLASQFIARLFKHFPMYADMALGAQLNLCSDTDVAIRKQATKDLPAFCKDNKEYTQKVADILAQLLQSKDSLELIVVQNSLLAVLKSDPQGAFAGIFNQILHGEDIVRERCIKFLSTKIKQLGSDILTKDIEDQLIENCKKVLQDVTCEEFHTLMSILSSTRLGKAVSGHKQLVDIVVEQSELDGNFEVNREEDDHRERLMQCLAIAMPYFSSQVDSSRFVAFICDKVLPVFNQLTSSKDVLLNTSSTEVQLDLLKALSELMVHCHSLDIPEKRAQRVYNVLLDYMPIPPDCDTTTESPSLQFSHVECLLYTYHSLMRMASKTTAVIEESMQGEALKDFRVRLQYFARGVQAYIKILKDALKGKTDAELKSEENQLKVAALRTTSNINILIKDLFHTPPSYKSQITLSWILPGSKRQRIVEPEYNTIKSSNFKRHTPITFESSPIKRTRNVASSESRRQGSAYANGQKMYIPPSGKYSTKVTSYVPSNSFRGRGGRGNKPFEIAIFFQIDLQ

>497

MPTVEELYRNYGILADATEQVGQHKDAYQVILDGVKGGTKEKRLAAQFIPKFFKHFPELADSAINAQLDLCEDEDVSIRRQAIKELPQFATGENLPRVADILTQLLQTDDSAEFNLVNNALLSIFKMDAKGTLGGLFSQILQGEDIVRERAIKFLSTKLKTLPDEVLTKEVEELILTESKKVLEDVTGEEFVLFMKILSGLKSLQTVSGRQQLVELVAEQADLEQTFNPSDPDCVDRLLQCTRQAVPLFSKNVHSTRFVTYFCEQVLPNLSTLTTPVEGLDIQLEVLKLLAEMSSFCGDMEKLETNLRKLFDKLLEYMPLPPEEAENGENAGNEEPKLQFSYVECLLYSFHQLGRKLPDFLTAKLNAEKLKDFKIRLQYFARGLQVYIRQLRLALQGKTGEALKTEENKIKVVALKITNNINVLIKDLFHIPPSYKSTVTLSWKPVQKVEIGQKRASEDTTSGSPPKKSSAGPKRDARQIYNPPSGKYSSNLGNFNYERSLQGK

>498

MPTVEELYRNYGILADATEQVGQHKDAYQVILDGVKGGTKEKRLAAQFIPKFFKHFPELADSAINAQLDLCEDEDVSIRRQAIKELPQFATGENLPRVADILTQLLQTDDSAEFNLVNNALLSIFKMDAKGTLGGLFSQILQGEDIVRERAIKFLSTKLKTLPDEVLTKEVEELILTESKKVLEDVTGEEFVLFMKILSGLKSLQTVSGRQQLVELVAEQADLEQTFNPSDPDCVDRLLQCTRQAVPLFSKNVHSTRFVTYFCEQVLPNLSTLTTPVEGLDIQLEVLKLLAEMSSFCGDMEKLETNLRKLFDKLLEYMPLPPEEAENGENAGNEEPKLQFSYVECLLYSFHQLGRKLPDFLTAKLNAEKLKDFKIRLQYFARGLQVYIRQLRLALQGKTGEALKTEENKIKVVALKITNNINVLIKDLFHIPPSYKSTVTLSWKPVQKVEIGQKRASEDTTSGSPPKKSSAGPKRDARQIYNPPSGKYSSNLGNFNYEQRGAFRGSRGGRGWGTRGNRSRGRLY

>499

MPTVEELYRNYGILADATEQVGQHKDAYQVILDGVKGGTKEKRLAAQFIPKFFKHFPELADSAINAQLDLCEDEDVSIRRQAIKELPQFATGENLPRVADILTQLLQTDDSAEFNLVNNALLSIFKMDAKGTLGGLFSQILQGEDIVRERAIKFLSTKLKTLPDEVLTKEVEELILTESKKVLEDVTGEEFVLFMKILSGLKSLQTVSGRQQLVELVAEQADLEQTFNPSDPDCVDRLLQCTRQAVPLFSKNVHSTRFVTYFCEQVLPNLSTLTTPVEGLDIQLEVLKLLAEMSSFCGDMEKLETNLRKLFDKLLEYMPLPPEEAENGENAGNEEPKLQFSYVECLLYSFHQLGRKLPDFLTAKLNAEKLKDFKIRLQYFARGLQVYIRQLRLALQGKTGEALKTEENKIKVVALKITNNINVLIKDLFHIPPSYKSTVTLSWKPVQKVEIGQKRANEDTTSGSPPKKSPAGPKRDARQIYNPPSGKYSSNLGNFNYGERFRLGTSSMQD

>500

MIRRQAIKELPQFATGDNLPRVADILTQLLQSDDSAEFNLVNNALLSIFKMDAKGTLGGLFSQILQGEDIVRERAIKFLSTKLKTLPEEVMTKEVEEFILTESKKVLEDVTGEEFVLFMKILSGLKSLQTVSGRQQLVELVAEQADLEQTFNPSDPDCVDRLLQCTRQAVPLFSKNVHSTKFVTYFCEHVLPNLSSLTTPVEGLDIQLEVLKLLAEMSSFCGDMEKLESNLKKLFDKLLEYMPLPPEEAENGENAGNEEPKLQFSYVECLLYSFHQLGRKLPDFLTAKLNAEKLKDFKIRLQYFARGLQVYIRQLRLALQGKTGEALKTEELFYRLEWVSSCACCGPITSNVILHVPLLEEGTGKILNV

>501

MIRRQAIKELPQFATGDNLPRVADILTQLLQSDDSAEFNLVNNALLSIFKMDAKGTLGGLFSQILQGEDIVRERAIKFLSTKLKTLPEEVMTKEVEEFILTESKKVLEDVTGEEFVLFMKILSGLKSLQTVSGRQQLVELVAEQADLEQTFNPSDPDCVDRLLQCTRQAVPLFSKNVHSTKFVTYFCEHVLPNLSSLTTPVEGLDIQLEVLKLLAEMSSFCGDMEKLESNLKKLFDKLLEYMPLPPEEAENGENAGNEEPKLQFSYVECLLYSFHQLGRKLPDFLTAKLNAEKLKDFKIRLQYFARGLQVYIRQLRLALQGKTGEALKTEENKIKVVALKITNNINVLIKDLFHIPPSYKSTVTLSWKPVQKADAGQKRTSEDTTSSSPPKKAPAGPKRDARQIYNPPSGKYSSNLGSFSYEQRGGFRGGRGRGWGGRGNRSRGRIY

>502

MIRRQAIKELPQFATGDNLPRVADILTQLLQSDDSAEFNLVNNALLSIFKMDAKGTLGGLFSQILQGEDIVRERAIKFLSTKLKTLPEEVMTKEVEEFILTESKKVLEDVTGEEFVLFMKILSGLKSLQTVSGRQQLVELVAEQADLEQTFNPSDPDCVDRLLQCTRQAVPLFSKNVHSTKFVTYFCEHVLPNLSSLTTPVEGLDIQLEVLKLLAEMSSFCGDMEKLESNLKKLFDKLLEYMPLPPEEAENGENAGNEEPKLQFSYVECLLYSFHQLGRKLPDFLTAKLNAEKLKDFKIRLQYFARGLQVYIRQLRLALQGKTGEALKTEENKIKVVALKITNNINVLIKDLFHIPPSYKSTVTLSWKPVQKADAGQKRTSEDTTSSSPPKKAPAGPKRDARQIYNPPSGKYSSNLGSFSYGNGKLSTVYKYFVWYPAMCRILNLDTVKY

>503

MVTVTLDKLYDNYDVLSDAKDKISEYSKEYQEAIEGTKGDEKTKKLSAQIISKFFKFFPDLQSKAIEAIFDICEDDDSSIRIAAMKALPSFCKDSKEHVVKVADILAQLLQLDDPQEYNVAANSLLQIFKDNPILVIKCIFKQINGNDNIVRDKCIKFLVSKIKISDNNIITPEVEDIIIQETKKVLQDSTAEEYINLMPFLISTRLSSTQAGQQELVDIAIEQAELDEELDPLDKESNNVDRLATCIKFVLPFFSVSIFILPVEKMLMPKKNRVAIYEHLFKEGVMVAKKDYHAPKHPELETIPNLQVIKAMQSLKSRGFVKEQFAWRHFYWYLTNEGIEYLRTFLHLPPEIVPATLKRQARSEAARPRGAAMPRSEVSKPSEDRAGYRRTGGVGGPDKKADVGAGTAEMEKWMXPMYKCNVTLSFKAEETIAKTPEKAPVGNKRHVPITFESNGASSANKNTKKSSDGMKLYTPPSGKFSNNFQSYGGRGRPRGSRGSRGRGSGRGWR

>504

MPTVEELYRNYGILADATEQVGQHKDAYQVILDGVKGGTKEKRLAAQFIPKFFKHFPELADSAINAQLDLCEDEDVSIRRQAIKELPQFATGENLPRVADILTQLLQTDDSAEFNLVNNALLSIFKMEKLETNLRKLFDKLLEYMPLPPEEAENGENAGNEEPKLQFSYVECLLYSFHQLGRKLPDFLTAKLNAEKLKDFKIRLQYFARGLQVYIRQLRLALQGKTGEALKTEENKIKVVALKITNNINVLIKDLFHIPPSYKSTVTLSWKPVQKVEIGQKRANEDTTSGSPPKKSPAGPKRDARQIYNPPSGKYSSNLSNFNYERSLQGK

>505

MPTVEELYRNYGILADATEQVGQIRRQAIKELPQFATGENLPRVADILTQLLQTDDSAEFNLVNNALLSIFKMDAKGTLGGLFSQILQGEDIVRERAIKFLSTKLKTLPDEVLTKEVEELILTESKKVLEDVTGEEFVLFMKILSGLKSLQTVSGRQQLVELVAEQADLEQTFNPSDPDCVDRLLQCTRQAVPLFSKNVHSTRFVTYFCEQVLPNLSSLTTPVEGLDIQLEVLKLLAEMSSFCGDMEKLETNLRKLFDKLLEYMPLPPEEAENGENAGNEEPKLQFSYVECLLYSFHQLGRKLPDFLTAKLNAEKLKDFKIRLQYFARGLQVYIRQLRLALQGKTGEALKTEENKIKVVALKITNNINVLIKDLFHIPPSYKSTVTLSWKPVQKVEIGQKRANEDTTSGSPPKKSPAGPKRDARQIYNPPSGKYSSNLSNFNYERSLQGK

>506

MPTVESSTPNDGILXDATETAGQHKDAYQVILDGVKGGAKEKRLAAQFIPKFFKHFPELADSAINAQLDLCEDEDVSIRRQAIKELPQFATGDNLPRVADILTQLLQSDDSAEFNLVNNALLSIFKMDAKGTLGGLFSQILQGEDIVRERAIKFLSTKLKTLPEEVMTKEVEEFILTESKKVLEDVTGEEFNLFMKILSGLKSLQTVSGRQQLVELVAEQADLEQTFNPSDPDCVDRLQQCTRQAVPLFSKNVHSTKFVTYFCEHVLPNLSSLTTSVEGLDIQLEVLKLLAEMSSFCGDMEKLESNLKKLFDKLLEYMPLPPEEAENGENAGNEEPKLQFSHVECLLYSFHQLGRKLPDFLTAKLNAEKLKDFKIRLQYFARGLQVYIRQLRLALQGKTGEALKTEENKIKVVALKITNNINVLIKDLFHIPPSYKSTITLSWKPVQKADASQKRASEDTTSSSPPKKASAGPKRDARQIYNPPSGKYSSNLGSFSYEQRGGFRGGRGRGWGGRGNRSRGRIY

>507

MEAEDNIQKMYKYFGILADAKENIAEKEPEYLEILSAVKGSTKEKRLASQFITRFFKHFPNLAAQALEAQLDLCEDEDICIRKQAIKDLPVLCKESKEYLTKIADILAQLLQADDPQELLTAQNSLLSLFKIDAKGALTGIFSQMQSNEEIVRERSMKFILNKVMALGKEIIKKDVEDLIIGECKKVMQNITCEEFETLMTILSSTHLINTPDGQKELVELLASTAELDQFFNPKDVDQVNRFITCLDFAIPFFSVHVESTQFIVYICELLNRYTLINDVDKQFIILKSLAESVPFCGKLMNPETVVGQVYQAILDLVTVPENDASKKVEDMDLHRVEALLYTFHKLGRQCPEFLSRDPERQKEFKKKLLYVGTCTQTFVKIVRQDLKEKGEEDVKNNPVIEKKLEGLRLACNINTLIKELFNIPPRFKATITLSWLSSATKSKLAQIVQEGKKHEPIVVDGKSKRIDGSNNSQQLYQPPKDKFSAKFSNNTNNTNRRGGSGAGWNNKRSFDSSNGNRRGWRPY

>508

MEAEDNIQKMYKYFGILADAKENIAEKEPEYLEILSAVKGSTKEKRLASQFITRFFKHFPNLAAQALEAQLDLCEDEDICIRKQAIKDLPVLCKESKEYLTKIADILAQLLQADDPQELLTAQNSLLSLFKIDAKGALTGIFSQMQSNEEIVRERSMKFILNKVMALGKEIIKKDVEDLIIGECKKVMQNITCEEFETLMTILSSTHLINTPDGQKELVELLASTAELDQFFNPKDVDQVNRFITCLDFAIPFFSVHVESTQFIVYICELLNRYTLINDVDKQFIILKSLAESVPFCGKLMNPETVVGQVYQAILDLVTVPENDASKKVEDMDLHRVEALLYTFHKLGRQCPEFLSRDPERQKEFKKKLLYVGTCTQTFVKIVRQDLKEKGEEDVKNNPVIEKKLEGLRLACNINTLIKELFNIPPRFKATITLSWLSSATKSKLAQIVQEGKKHEPIVVDGKSKRIDGSNNSQQLYQPPKDKFSAKFSNNTNSNTNTTYTNRRGGSGAGWNNKRSFDSSNGNRRGWRPY

>509

MPSVEELYRNYGILADATEQVGQHKDAYQVILDGVKGGTKEKRLAAQFIPKFFKHFPELADSAINAQLDLCEDEDVSIRRQAIKELPQFATGENLPRVADILTQLLQTDDSAEFNLVNNALLSIFKMDAKGTLGGLFSQILQGEDIVRERAIKFLSTKLKTLPDEVLTKEVEELILTESKKVLEDVTGEEFVLFMKILSGLKSLQTVSGRQQLVELVAEQADLEQTFNPSDPDCVDRLLQCTRQAVPLFSKNVHSTRFVTYFCEQVLPNLSSLTTPVEGLDIQLEVLKLLAEMSSFCGDMEKLETNLRKLFDKLLEYMPLPPEEAENGENAGNEEPKLQFSYVECLLYSFHQLGRKLPDFLTAKLNAEKLKDFKIRLQYFARGLQVYIRQLRLALQGKTGEALKTEENKIKVVALKITNNINVLIKDLFHIPPSYKSTVTLSWKPVQKVEIGQKRANEDTTSGSPPKKSPAGPKRDARQIYNPPSGKYSSNLGNFNYERSLQGK

>510

MPSVEELYRNYGILADATEQVGQHKDAYQVILDGVKGGTKEKRLAAQFIPKFFKHFPELADSAINAQLDLCEDEDVSIRRQAIKELPQFATGENLPRVADILTQLLQTDDSAEFNLVNNALLSIFKMDAKGTLGGLFSQILQGEDIVRERAIKFLSTKLKTLPDEVLTKEVEELILTESKKVLEDVTGEEFVLFMKILSGLKSLQTVSGRQQLVELVAEQADLEQTFNPSDPDCVDRLLQCTRQAVPLFSKNVHSTRFVTYFCEQVLPNLSSLTTPVEGLDIQLEVLKLLAEMSSFCGDMEKLETNLRKLFDKLLEYMPLPPEEAENGENAGNEEPKLQFSYVECLLYSFHQLGRKLPDFLTAKLNAEKLKDFKIRLQYFARGLQVYIRQLRLALQGKTGEALKTEENKIKVVALKITNNINVLIKDLFHIPPSYKSTVTLSWKPVQKVEIGQKRANEDTTSGSPPKKSPAGPKRDARQIYNPPSGKYSSNLGNFNYEKKDSFKEYFSGAVRESNQ

>511

MPSVEELYRNYGILADATEQVGQHKDAYQVILDGVKGGTKEKRLAAQFIPKFFKHFPELADSAINAQLDLCEDEDVSIRRQAIKELPQFATGENLPRVADILTQLLQTDDSAEFNLVNNALLSIFKMDAKGTLGGLFSQILQGEDIVRERAIKFLSTKLKTLPDEVLTKEVEELILTESKKVLEDVTGEEFVLFMKILSGLKSLQTVSGRQQLVELVAEQADLEQTFNPSDPDCVDRLLQCTRQAVPLFSKNVHSTRFVTYFCEQVLPNLSSLTTPVEGLDIQLEVLKLLAEMSSFCGDMEKLETNLRKLFDKLLEYMPLPPEEAENGENAGNEEPKLQFSYVECLLYSFHQLGRKLPDFLTAKLNAEKLKDFKIRLQYFARGLQVYIRQLRLALQGKTGEALKTEENKIKVVALKITNNINVLIKDLFHIPPSYKSTVTLSWKPVQKVEIGQKRANEDTTSGSPPKKSPAGPKRDARQIYNPPSGKYSSNLGNFNYEQRGAFRGSRGGRGWGARGNRSRGRLY

>512

MSFFCTREKTQHALCCLLFQHKDAYQVILDGVKGGAKEKRLAAQFIPKFFKHFPELADSAINAQLDLCEDEDVSIRRQAIKELPQFATGDNLPRVADILTQLLQSDDSAEFNLVNNALLSIFKMDAKGTLGGLFSQILQGEDIVRERAIKFLSTKLKTLPEEVLTKEVEEFILTESKKVLEDVTGEEFVLFMKILSGLKSLQTVSGRQQLVELVAEQADLEQTFNPSDPDCVDRLLQCTRQAVPLFSKNVHSTRFVTYFCEHVLPNLSSLTTPVEGLDIQLEVLKLLAEMSSFCGDMEKLESNLKKLFDKLLEYMPLPPEEAENGENAGNEEPKLQFSYVECLLYSFHQLGRKLPDFLTAKLNAEKLKDFKIRLQYFARGLQVYIRQLRLALQGKTGEALKTEENKIKVVALKITNNINVLIKDLFHIPPSYKSTVTLSWKPVQKTDAGQKRANEDTTSSSPPKKAAAGPKRDARQIYNPPSGKYSSNLGSFSYEQRGGFRGGRGRGWGGRGNRSRGRIY

>513

MPTVEELYRNYGILADATETAGQHKDAYQVILDGVKGGAKEKRLAAQFIPKFFKHFPELADSAINAQLDLCEDEDVSIRRQAIKELPQFATGDNLPRVADILTQLLQSDDSAEFNLVNNALLSIFKMDAKGTLGGLFSQILQGEDIVRERAIKFLSTKLKTLPEEVLTKEVEEFILTESKKVLEDVTGEEFVLFMKILSGLKSLQTVSGRQQLVELVAEQADLEQTFNPSDPDCVDRLLQCTRQAVPLFSKNVHSTRFVTYFCEHVLPNLSSLTTPVEGLDIQLEVLKLLAEMSSFCGDMEKLESNLKKLFDKLLEYMPLPPEEAENGENAGNEEPKLQFSYVECLLYSFHQLGRKLPDFLTAKLNAEKLKDFKIRLQYFARGLQVYIRQLRLALQGKTGEALKTEENKIKVVALKITNNINVLIKDLFHIPPSYKSTVTLSWKPVQKTDAGQKRANEDTTSSSPPKKAAAGPKRDARQIYNPPSGKYSSNLGSFSYEQRGGFRGGRGRGWGGRGNRSRGRIY

>514

MPTVEELYRNYGILADATEQVGQHKDAYQVILDGVKGGTKEKRLAAQFIPKFFKHFPELADSAINAQLDLCEDEDVSIRRQAIKELPQFATGENLPRVADILTQLLQTDDSAEFNLVNNALLSIFKMDAKGTLGGLFSQILQGEDIVRERAIKFLSTKLKTLPDEVLTKEVEELILTESKKVLEDVTGEEFVLFMKILSGLKSLQTVSGRQQLVELVAEQADLEQTFNPSDPDCVDRLLQCTRQAVPLFSKNVHSTRFVTYFCEQVLPNLSSLTTPVEGLDIQLEVLKLLAEMSSFCGDMEKLETNLRKLFDKLLEYMPLPPEEAENGENAGNEEPKLQFSYVECLLYSFHQLGRKLPDFLTAKLNAEKLKDFKIRLQYFARGLQVYIRQLRLALQGKTGEALKTEENKIKVVALKITNNINVLIKDLFHIPPSYKSTVTLSWKPVQKVEIGQRRASEDTTSGSPPKKSPAGPKRDARQIYNPPSGKYSSNLGNFNYEN

>515

MPTVEELYRNYGILADATEQVGQHKDAYQVILDGVKGGTKEKRLAAQFIPKFFKHFPELADSAINAQLDLCEDEDVSIRRQAIKELPQFATGENLPRVADILTQLLQTDDSAEFNLVNNALLSIFKMDAKGTLGGLFSQILQGEDIVRERAIKFLSTKLKTLPDEVLTKEVEELILTESKKVLEDVTGEEFVLFMKILSGLKSLQTVSGRQQLVELVAEQADLEQTFNPSDPDCVDRLLQCTRQAVPLFSKNVHSTRFVTYFCEQVLPNLSSLTTPVEGLDIQLEVLKLLAEMSSFCGDMEKLETNLRKLFDKLLEYMPLPPEEAENGENAGNEEPKLQFSYVECLLYSFHQLGRKLPDFLTAKLNAEKLKDFKIRLQYFARGLQVYIRQLRLALQGKTGEALKTEENKIKVVALKITNNINVLIKDLFHIPPSYKSTVTLSWKPVQKVEIGQRRASEDTTSGSPPKKSPAGPKRDARQIYNPPSGKYSSNLGNFNYERSLQGK

>516

MPTVEELYRNYGILADATEQVGQHKDAYQVILDGVKGGTKEKRLAAQFIPKFFKHFPELADSAINAQLDLCEDEDVSIRRQAIKELPQFATGENLPRVADILTQLLQTDDSAEFNLVNNALLSIFKMDAKGTLGGLFSQILQGEDIVRERAIKFLSTKLKTLPDEVLTKEVEELILTESKKVLEDVTGEEFVLFMKILSGLKSLQTVSGRQQLVELVAEQADLEQTFNPSDPDCVDRLLQCTRQAVPLFSKNVHSTRFVTYFCEQVLPNLSSLTTPVEGLDIQLEVLKLLAEMSSFCGDMEKLETNLRKLFDKLLEYMPLPPEEAENGENAGNEEPKLQFSYVECLLYSFHQLGRKLPDFLTAKLNAEKLKDFKIRLQYFARGLQVYIRQLRLALQGKTGEALKTEENKIKVVALKITNNINVLIKDLFHIPPSYKSTVTLSWKPVQKVEIGQRRASEDTTSGSPPKKSPAGPKRDARQIYNPPSGKYSSNLGNFNYEQRGAFRGSRGGRGWGARGNRSRGRLY

>517

MPTVEELYRNYGILADATEQVGQHKDAYQVILDGVKGGTKEKRLAAQFIPKFFKHFPELADSAINAQLDLCEDEDVSIRRQAIKELPQFATGENLPRVADILTQLLQTDDSAEFNLVNNALLSIFKMDAKGTLGGLFSQILQGEDIVRERAIKFLSTKLKTLPDEVLTKEVEELILTESKKVLEDVTGEEFVLFMKILSGLKSLQTVSGRQQLVELVAEQADLEQTFNPSDPDCVDRLLQCTRQAVPLFSKNVHSTRFVTYFCEHVLPNLSSLTTPVEGLDIQLEVLKLLAEMSSFCGDMEKLETNLRKLFDKLLEYMPLPPEEAENGENAGNEEPKLQFSYVECLLYSFHQLGRKLPDFLTAKLNAEKLKDFKIRLQYFARGLQVYIRQLRLALQGKTGEALKTDENKIKVVALKITNNINVLIKDLFHIPPSYKSTVTLSWKPVQKVEIGQKRATEDTTSGSPPKKSPAGPKRDARQIYNPPSGKYSSNLGNFNYERSLQGK

>518

MPTVEELYRNYGILADATEQVGQHKDAYQVILDGVKGGTKEKRLAAQFIPKFFKHFPELADSAINAQLDLCEDEDVSIRRQAIKELPQFATGENLPRVADILTQLLQTDDSAEFNLVNNALLSIFKMDAKGTLGGLFSQILQGEDIVRERAIKFLSTKLKTLPDEVLTKEVEELILTESKKVLEDVTGEEFVLFMKILSGLKSLQTVSGRQQLVELVAEQADLEQTFNPSDPDCVDRLLQCTRQAVPLFSKNVHSTRFVTYFCEHVLPNLSSLTTPVEGLDIQLEVLKLLAEMSSFCGDMEKLETNLRKLFDKLLEYMPLPPEEAENGENAGNEEPKLQFSYVECLLYSFHQLGRKLPDFLTAKLNAEKLKDFKIRLQYFARGLQVYIRQLRLALQGKTGEALKTDENKIKVVALKITNNINVLIKDLFHIPPSYKSTVTLSWKPVQKVEIGQKRATEDTTSGSPPKKSPAGPKRDARQIYNPPSGKYSSNLGNFNYEQRGAFRGSRSGRGWGARGNRSRGRLY

>519

MDSDKIEKLYIQYEILAESKDPSQHEEVYRTILASTQGNAKEKKLVCQFIPKFFPHYPQLAKMALEAYFDLCEDNDVAIRKEAIKGLPQLCRELKQYTKRIADILAQLLQATDSGEVSIVRESLITVIKSDPVSALKGVFNQIKNGDDKVREKTIKFLATRVRRLPSSTFSPDVQDVFIAEIKETFQDATSEEIPLLFGLLLWTRLGKNTVGRKLILEIVLSYLEADKSILIVDERYSLKRFITFAITAKTLFTSDINSNKYLVFFCKEVLPKFMSITGAKDGSGSAIALLKLLAEIATYSKDLKDIDDLIDEVFTILTEFLPCPSEDEQDVYKRLQFSSVECLLYTMHHMMRTSPSYFSDKTDKLKDFRMRLLYFFRVSQAXVKTLEDVHKKKKKDDEDRVKIVALKTISNINILIKDFFHNPPSQKSSIVLSWLSTENFPAKRKATSANSNRAEKMLKGEDGQHTYQPPIGKYSQNLWPNLRLSRFDKWAFKN

>520

MTDPSDEAKNIEKLYEFGERLNESKDKSQNVADYQGIIDAAKTSIKAKQLAAQLIPRFFKFFPDLSESAIYTHIDLIEEEELGVRVQAIRGLPLFCKDTPEQIAKIVDILVQLLAAEEFVERDAVHKALMSLLRQDVKVSLTALFKHIGSVDEPSTDEFIREKVLTFIREKVFPFKSELLKPQEEMERHITDLIKKSLEDVTGAEFRMFMDFLKSLSIFGEKAPPERMKELIGIIEGQADLDAQFNVSDADHIDRLISCLFMALPFVVRGASSSKFLNYLNKHILPVSDKLPDERRLDLLKALAEVSPYTTPQDSRQILPSVVQLLKVPFHILRIIRKWTM

>521

MTGDSIEKLYKYYGILADAGDNIVKHEDLYSEIIQAAKGDQSERRLACQFIPRFFIHFPNLGETALNSLFDLCEDPEISIRKQVIKELPHICKQCTMFTKKTSYILSQLLQSKDLAELSIVSRSLLSLFQIYPEGAVAGLFNQIRNGEDTVRDKCLSFLTNKVKSLPIANLPKLEEALITETKSTFDEASPEEIPIFMDILFWTKLGKDVTGHTVIINMIAEQIESETPFNVHDDDLLERLVTLCKSVKPLFKPLTSPDRYVTYFCKEVLPNFKSISGYDECSGYALELLQLFTELTVYCNKLNEPVIVVESIFNLLIEVMPYSESGQDNFDDLKFHFSYIECLLYSLHKLFKLHPSFLSDDVELSKFRSRLQYFSRALQGYIKTLHNAIKSESLDALKKPENAIKTLALKTTLNINALIKDFFYNPPSNKATIQLSWLIPKACQNEKAIVRLKRMCNSLGVEIPAKRSNTLKPYRPIQRPLYQPPNGKYSKTFCNPKRNGPSL

>522

MATDNIEKLYQNYGILADAKDDIAKHETEYLEILAAVKGSDKEKRLASQFIAKFFDSFPNLADQSIEAQFDLCEDDDVTVSIICNNFFIGEFPIHNLPYQNISTIKT

>523

MPTVEELYRNYGILADATEQVGQHKDAYQVILDGVKGGTKEKRLAAQFIPKFFKHFPELADSAINAQLDLCEDEDVSIRRQAIKELPQFATGENLPRVADILTQLLQTDDSAEFNLVNNALLSIFKMDAKGTLGGLFSQILQGEDIVRERAIKFLSTKLKTLPDEVLTKEVEELILTESKKVLEDVTGEEFVLFMKILSGLKSLQTVSGRQQLVELVAEQADLEQTFNPSDPDCVDRLLQCTRQAVPLFSKNVHSTRFVTYFCEQVLPNLSSLTTPVEGLDIQLEVLKLLAEMSSFCGDMEKLETNLRKLFDKLLNKIKVVALKITNNINVLIKDLFHIPPSYKSTVTLSWKPVQKVEIGQKRATEDTTSGSPPKKSPAGPKRDARQIYNPPSGKYSSNLGNFNYECRSDTLPVLAWPSRSLAVLLSPVQSPEPPCKKPLKIP

>524

MPTVEELYRNYGILADATEQVGQHKDAYQVILDGVKGGTKEKRLAAQFIPKFFKHFPELADSAINAQLDLCEDEDVSIRRQAIKELPQFATGENLPRVADILTQLLQTGTLGGLFSQILQGEDIVRERAIKFLSTKLKTLPDEVLTKEVEELILTESKKVLEDVTGEEFVLFMKILSGLKSLQTVSGRQQLVELVAEQADLEQTFNPSDPDCVDRLLQCTRQAVPLFSKNVHSTRFVTYFCEQVLPNLSSLTTPVEGLDIQLEVLKLLAEMSSFCGDMEKLETNLRKLFDKLLEYMPLPPEEAENGENAGNEEPKLQFSYVECLLYSFHQLGRKLPDFLTAKLNAEKLKDFKIRLQYFARGLQVYIRQLRLALQGKTGEALKTDENKIKVVALKITNNINVLIKDLFHIPPSYKSTVTLSWKPVQKVEIGQKRATEDTTSGSPPKKSPAGPKRDARQIYNPPSGKYSSNLGNFNYECRSDTLPVLAWPSRSLAVLLSPVQSPEPPCKKPLKIP

>525

MPTVEELYRNYGILADATEQVGQHKDAYQVILDGVKGGTKEKRLAAQFIPKFFKHFPELADSAINAQLDLCEDEDVSIRRQAIKELPQFATGENLPRVADILTQLLQTDDSAEFNLVNNALLSIFKMDAKGTLGGLFSQILQGEDIVRERAIKFLSTKLKTLPDEVLTKEVEELILTESKKVLEDVTGEEFVLFMKILSGLKSLQTVSGRQQLVELVAEQADLEQTFNPSDPDCVDRLLQCTRQAVPLFSKNVHSTRFVTYFCEQVLPNLSSLTTPVEGLDIQLEVLKLLAEMSSFCGDMEKLETNLRKLFDKLLEYMPLPPEEAENGENAGNEEPKLQFSYVECLLYSFHQLGRKLPDFLTAKLNAEKLKDFKIRLQYFARGLQVYIRQLRLALQGKTGEALKTDENKIKVVALKITNNINVLIKDLFHIPPSYKSTVTLSWKPVQKVEIGQKRATEDTTSGSPPKKSPAGPKRDARQIYNPPSGKYSSNLGNFNYECRSDTLPVLAWPSRSLAVLLSPVQSPEPPCKKPLKIP

>526

MPTVEELYRNYGILADATEQVGQHKDSYQVILDGVKGGTKEKRLAAQFIPKFFKHFPELADSAINAQLDLCEDEDVSIRRQAIKELPQFATGENLPRVADILTQLLQTDDSAEFNLVNNALLSIFKMDAKGTLGGLFSQILQGEDIVRERAIKFLSTKLKTLPDEVLTKEVEELILTESKKVLEDVTGEEFVLFMKILSGLKSLQTVSGRQQLVELVAEQADLEQTFNPSDPDCVDRLLQCTRQAVPLFSKNVHSTRFVTYFCEQVLPNLSSLTTPVEGLDIQLEVLKLLAEMSSFCGDMEKLETNLRKLFDKLLEYMPLPPEEAENGENAGNEEPKLQFSYVECLLYSFHQLGRKLPDFLTAKLNAEKLKDFKIRLQYFARGLQVYIRQLRLALQGKTGEALKTEENKIKVVALKITNNINVLIKDLFHIPPSYKSTVTLSWKPVQKVELGQKRANEDTTSGSPPKKSTAGPKRDARQIYNPPSGKYSSNLGTFNYDPFKEEWTKIILTQLLILLRKTQFNNASNQTRLWLVLNTSQN

>527

MTGGGAGGCWRRNSAGRGLGELPRWWWRRLRRRQQERILGLSLTMPTVEELYRNYGILADATEQVGQHKDSYQVILDGVKGGTKEKRLAAQFIPKFFKHFPELADSAINAQLDLCEDEDVSIRRQAIKELPQFATGENLPRVADILTQLLQTDDSAEFNLVNNALLSIFKMDAKGTLGGLFSQILQGEDIVRERAIKFLSTKLKTLPDEVLTKEVEELILTESKKVLEDVTGEEFVLFMKILSGLKSLQTVSGRQQLVELVAEQADLEQTFNPSDPDCVDRLLQCTRQAVPLFSKNVHSTRFVTYFCEQVLPNLSSLTTPVEGLDIQLEVLKLLAEMSSFCGDMEKLETNLRKLFDKLLEYMPLPPEEAENGENAGNEEPKLQFSYVECLLYSFHQLGRKLPDFLTAKLNAEKLKDFKIRLQYFARGLQVYIRQLRLALQGKTGEALKTEENKIKVVALKITNNINVLIKDLFHIPPSYKSTVTLSWKPVQKVELGQKRANEDTTSGSPPKKSTAGPKRDARQIYNPPSGKYSSNLGTFNYERSLQGK

>528

MTGGGAGGCWRRNSAGRGLGELPRWWWRRLRRRQQERILGLSLTMPTVEELYRNYGILADATEQVGQHKDSYQVILDGVKGGTKEKRLAAQFIPKFFKHFPELADSAINAQLDLCEDEDVSIRRQAIKELPQFATGENLPRVADILTQLLQTDDSAEFNLVNNALLSIFKMDAKGTLGGLFSQILQGEDIVRERAIKFLSTKLKTLPDEVLTKEVEELILTESKKVLEDVTGEEFVLFMKILSGLKSLQTVSGRQQLVELVAEQADLEQTFNPSDPDCVDRLLQCTRQAVPLFSKNVHSTRFVTYFCEQVLPNLSSLTTPVEGLDIQLEVLKLLAEMSSFCGDMEKLETNLRKLFDKLLEYMPLPPEEAENGENAGNEEPKLQFSYVECLLYSFHQLGRKLPDFLTAKLNAEKLKDFKIRLQYFARGLQVYIRQLRLALQGKTGEALKTEENKIKVVALKITNNINVLIKDLFHIPPSYKSTVTLSWKPVQKVELGQKRANEDTTSGSPPKKSTAGPKRDARQIYNPPSGKYSSNLGTFNYEQRGAFRGSRGGRGWGARGNRSRGRLY

>529

MPTVEELYRNYGILADATEQVSQHKDAYQVILDGVKGGTKEKRLAAQFIPKFFKHFPELADSAINAQLDLCEDEDVSIRRQAIKELPQFATGENLPRVADILTQLLQTDDSAEFNLVNNALLSIFKMDAKGTLGGLFSQILQGEDIVRERAIKFLSTKLKTLPDEVLTKEVEELILTESKKVLEDVTGEEFVLFMKILSGLKSLQTVSGRQQLVELVAEQADLEQTFNPSDPDCVDRLLQCTRQAVPLFSKNVHSTRFVTYFCEQVLPNLGTLTTPVEGLDIQLEVLKLLAEMSSFCGDMEKLETNLRKLFDKLLEYIPLPPEEAENGENAGNEEPKLQFSYVECLLYSFHQLGRKLPDFLTAKLNAEKLKDFKIRLQYFARGLQVYIRQLRLALQGKTGEALKTEENKIKVVALKITNNINVLIKDLFHIPPSYKSAVTLSWKPVQKVEIGQKRANEDATSGSPPKKSPAGPKRDARQIYNPPSGKYSSNLGNFNYERSLQGK

>530

MPTVEELYRNYGILADATEQVSQHKDAYQVILDGVKGGTKEKRLAAQFIPKFFKHFPELADSAINAQLDLCEDEDVSIRRQAIKELPQFATGENLPRVADILTQLLQTDDSAEFNLVNNALLSIFKMDAKGTLGGLFSQILQGEDIVRERAIKFLSTKLKTLPDEVLTKEVEELILTESKKVLEDVTGEEFVLFMKILSGLKSLQTVSGRQQLVELVAEQADLEQTFNPSDPDCVDRLLQCTRQAVPLFSKNVHSTRFVTYFCEQVLPNLGTLTTPVEGLDIQLEVLKLLAEMSSFCGDMEKLETNLRKLFDKLLEYIPLPPEEAENGENAGNEEPKLQFSYVECLLYSFHQLGRKLPDFLTAKLNAEKLKDFKIRLQYFARGLQVYIRQLRLALQGKTGEALKTEENKIKVVALKITNNINVLIKDLFHIPPSYKSAVTLSWKPVQKVEIGQKRANEDATSGSPPKKSPAGPKRDARQIYNPPSGKYSSNLGNFNYEQRGAFRGSRGGRGWGARGNRSRGRLY

>531

MPTVEELYRNYGILADATEQVGQHKDAYQLILDGVKGGTKEKRLAAQFIPKFFKHFPELADSAINAQLDLCEDEDVSIRRQAIKELPQFATGENLPRVADILTQLLQTDDSAEFNLVNNALLSIFKMDAKGTLGGLFSQILQGEDIVRERAIKFLSTKLKTLPDEVLTKEVEELILTESKKVLEDVTGEEFVLFMKILSGLKSLQTVSGRQQLVELVAEQADLEQTFNPSDPDCVDRLLQCTRQAVPLFSKNVHSTRFVTYFCEQVLPNLSSLTTPVEGLDIQLEVLKLLAEMSSFCGDMEKLETNLRKLFDKLLEYMPLPPEEAENGENAGNEEPKLQFSYVECLLYSFHQLGRKLPDFLTAKLNAEKLKDFKIRLQYFARGLQVYIRQLRLALQGKTGEALKTEENKIKVVALKITNNINVLIKDLFHIPPSYKSTVTLSWKPVQKVEIGQKRASEDTTSGSPPKKSPAGPKRDARQIYNPPSGKYSSNLGNFNYERSLQGK

>532

MPTVEELYRNYGILADATEQVGQHKDAYQLILDGVKGGTKEKRLAAQFIPKFFKHFPELADSAINAQLDLCEDEDVSIRRQAIKELPQFATGENLPRVADILTQLLQTDDSAEFNLVNNALLSIFKMDAKGTLGGLFSQILQGEDIVRERAIKFLSTKLKTLPDEVLTKEVEELILTESKKVLEDVTGEEFVLFMKILSGLKSLQTVSGRQQLVELVAEQADLEQTFNPSDPDCVDRLLQCTRQAVPLFSKNVHSTRFVTYFCEQVLPNLSSLTTPVEGLDIQLEVLKLLAEMSSFCGDMEKLETNLRKLFDKLLEYMPLPPEEAENGENAGNEEPKLQFSYVECLLYSFHQLGRKLPDFLTAKLNAEKLKDFKIRLQYFARGLQVYIRQLRLALQGKTGEALKTEENKIKVVALKITNNINVLIKDLFHIPPSYKSTVTLSWKPVQKVEIGQKRASEDTTSGSPPKKSPAGPKRDARQIYNPPSGKYSSNLGNFNYEQRGAFRGSRGGRGWGARGNRSRGRLY

>533

MPTVEELYRNYGILADATEQVSQHKDAYQVILDGVKGGTKEKRLAAQFIPKFFKHFPELADSAINAQLDLCEDEDVSIRRQAIKELPQFATGENLPRVADILTQLLQTDDSAEFNLVNNALLSIFKMDAKGTLGGLFSQILQGEDIVRERAIKFLSTKLKTLPDEVLTKEVEELILTESKKVLEDVTGEEFVLFMKILSGLKSLQTVSGRQQLVELVAEQADLEQTFNPSDPDCVDRLLQCTRQAVPLFSKNVHSTRFVTYFCEQVLPNLSTLTTPVEGLDIQLEVLKLLAEMSSFCGDMEKLETNLRKLFDKLLEYMPLPPEEAENGENAGNEEPKLQFSYVECLLYSFHQLGRKLPDFLTAKLNAEKLKDFKIRLQYFARGLQVYIRQLRLALQGKTGEALKTEENKIKVVALKITNNINVLIKDLFHIPPSYKSTVTLSWKPVQKVEIGQKRANEDTTSGSPPKKSAAGPKRDARQIYNPPSGKYSSNLGNFNYEQRGAFRGSRGGRGWGARGNRSRGRLY

>534

MPTVEELYRNYGILADATEQVSQHKDAYQVILDGVKGGTKEKRLAAQFIPKFFKHFPELADSAINAQLDLCEDEDVSIRRQAIKELPQFATGENLPRVADILTQLLQTDDSAEFNLVNNALLSIFKMDAKGTLGGLFSQILQGEDIVRERAIKFLSTKLKTLPDEVLTKEVEELILTESKKVLEDVTGEEFVLFMKILSGLKSLQTVSGRQQLVELVAEQADLEQTFNPSDPDCVDRLLQCTRQAVPLFSKNVHSTRFVTYFCEQVLPNLSTLTTPVEGLDIQLEVLKLLAEMSSFCGDMEKLETNLRKLFDKLLEYMPLPPEEAENGENAGNEEPKLQFSYVECLLYSFHQLGRKLPDFLTAKLNAEKLKDFKIRLQYFARGLQVYIRQLRLALQGKTGEALKTEENKIKVVALKITNNINVLIKDLFHIPPSYKSTVTLSWKPVQKVEIGQKRANEDTTSGSPPKKSAAGPKRDARQIYNPPSGKYSSNLGNFNYERSLQGK

>535

MPTVEELYRNYGILADATEQVGQHKDAYQVILDGVKGGTKEKRLAAQFIPKFFKHFPELADSAINAQLDLCEDEDVSIRRQAIKELPQFATGENLPRVADILTQLLQTDDSAEFNLVNNALLSIFKMDAKGTLGGLFSQILQGEDIVRERAIKFLSTKLKTLPDEVLTKEVEELILTESKKVLEDVTGEEFVLFMKILSGLKSLQTVSGRQQLVELVAEQADLEQTFNPSDPDCVDRLLQCTRQAVPLFSKNVHSTRFVTYFCEQVLPNLSSLTTPVEGLDIQLEVLKLLAEMSSFCGDMEKLETNLRKLFDKLLEYMPLPPEEAENGENAGNEEPKLQFSYVECLLYSFHQLGRKLPDFLTAKLNAEKLKDFKIRLQYFARGLQVYIRQLRLALQGKTGEALKTEENKIKVVALKITNNINVLIKDLFHIPPSYKSTVTLSWKPVQKVEIGQKRPNEDATSGSPPKKSPAGPKRDARQIYNPPSGKYSSNLGNFNYERSLQGK

>536

MPTVEELYRNYGILADATEQVGQHKDAYQVILDGVKGGTKEKRLAAQFIPKFFKHFPELADSAINAQLDLCEDEDVSIRRQAIKELPQFATGENLPRVADILTQLLQTDDSAEFNLVNNALLSIFKMDAKGTLGGLFSQILQGEDIVRERAIKFLSTKLKTLPDEVLTKEVEELILTESKKVLEDVTGEEFVLFMKILSGLKSLQTVSGRQQLVELVAEQADLEQTFNPSDPDCVDRLLQCTRQAVPLFSKNVHSTRFVTYFCEQVLPNLSSLTTPVEGLDIQLEVLKLLAEMSSFCGDMEKLETNLRKLFDKLLEYMPLPPEEAENGENAGNEEPKLQFSYVECLLYSFHQLGRKLPDFLTAKLNAEKLKDFKIRLQYFARGLQVYIRQLRLALQGKTGEALKTEENKIKVVALKITNNINVLIKDLFHIPPSYKSTVTLSWKPVQKVEIGQKRPNEDATSGSPPKKSPAGPKRDARQIYNPPSGKYSSNLGNFNYEQRGAFRGSRGDRGWGARGNRSRGRLY

>537

MPTVEELYRNYGILADATEQVSQHKDAYQVILDGVKGGTKEKRLAAQFIPKFFKHFPELADSAINAQLDLCEDEDVSIRRQAIKELPQFATGENLPRVADILTQLLQTDDSAEFNLVNNALLSIFKMDAKGTLGGLFSQILQGEDIVRERAIKFLSTKLKTLPDEVLTKEVEELILTESKKVLEDVTGEEFVLFMKILSGLKSLQTVSGRQQLVELVAEQADLEQTFNPSDPDCVDRLLQCTRQAVPLFSKNVHSTRFVTYFCEQVLPNLSTLTTPVEGLDIQLEVLKLLAEMSSFCGDMEKLETNLRKLFDKLLEYMPLPPEEAENGENAGNEEPKLQFSYVECLLYSFHQLGRKLPDFLTAKLNAEKLKDFKIRLQYFARGLQVYIRQLRLALQGKTGEALKTEENKIKVVALKITNNINVLIKDLFHIPPSYKSTVTLSWKPVQKVEMGQKRTTEDTTSGSPPKKSAAGPKRDARQIYNPPSGKYSSNLSNFNYERSLQGK

>538

MPTVEELYRNYGILADATEQVSQHKDAYQVILDGVKGGTKEKRLAAQFIPKFFKHFPELADSAINAQLDLCEDEDVSIRRQAIKELPQFATGENLPRVADILTQLLQTDDSAEFNLVNNALLSIFKMDAKGTLGGLFSQILQGEDIVRERAIKFLSTKLKTLPDEVLTKEVEELILTESKKVLEDVTGEEFVLFMKILSGLKSLQTVSGRQQLVELVAEQADLEQTFNPSDPDCVDRLLQCTRQAVPLFSKNVHSTRFVTYFCEQVLPNLSTLTTPVEGLDIQLEVLKLLAEMSSFCGDMEKLETNLRKLFDKLLEYMPLPPEEAENGENAGNEEPKLQFSYVECLLYSFHQLGRKLPDFLTAKLNAEKLKDFKIRLQYFARGLQVYIRQLRLALQGKTGEALKTEENKIKVVALKITNNINVLIKDLFHIPPSYKSTVTLSWKPVQKVEMGQKRTTEDTTSGSPPKKSAAGPKRDARQIYNPPSGKYSSNLSNFNYEQRGAFRGSRGGRGWGTRGSRSRGRLY

>539

MPTVEELYRNYGILADATEQVGQHKDAYQVILDGVKGGTKEKRLAAQFIPKFFKHFPELADSAINAQLDLCEDEDVSIRRQAIKELPQFATGENLPRVADILTQLLQTDDSAEFNLVNNALLSIFKMDAKGTLGGLFSQILQGEDIVRERAIKFLSTKLKTLPDEVLTKEVEELILTESKKVLEDVTGEEFVLFMKILSGLKSLQTVSGRQQLVELVAEQADLEQTFNPADPDCVDRLLQCTRQAVPLFSKNVHSTRFVTYFCEHVLPNLSSLTTPVEGLDIQLEVLKLLAEMSSFCGDMEKLETNLKKLFDKLLEYMPLPPEEAENGENAGNEEPKLQFSYVECLLYSFHQLGRKLPDFLTAKLNAEKLKDFKIRLQYFARGLQVYIRQLRLALQGKTGEALKTEENKIKVVALKITNNINVLIKDLFHIPPSYKSTVTLSWKPVQKAELGQKRTNEDTTSGSPPKKAAAGPKRDARQIYNPPSGKYSSNLGNFNYERGLQGK

>540

MPTVEELYRNYGILADATEQVGQHKDAYQVILDGVKGGTKEKRLAAQFIPKFFKHFPELADSAINAQLDLCEDEDVSIRRQAIKELPQFATGENLPRVADILTQLLQTDDSAEFNLVNNALLSIFKMDAKGTLGGLFSQILQGEDIVRERAIKFLSTKLKTLPDEVLTKEVEELILTESKKVLEDVTGEEFVLFMKILSGLKSLQTVSGRQQLVELVAEQADLEQTFNPADPDCVDRLLQCTRQAVPLFSKNVHSTRFVTYFCEHVLPNLSSLTTPVEGLDIQLEVLKLLAEMSSFCGDMEKLETNLKKLFDKLLEYMPLPPEEAENGENAGNEEPKLQFSYVECLLYSFHQLGRKLPDFLTAKLNAEKLKDFKIRLQYFARGLQVYIRQLRLALQGKTGEALKTEENKIKVVALKITNNINVLIKDLFHIPPSYKSTVTLSWKPVQKAELGQKRTNEDTTSGSPPKKAAAGPKRDARQIYNPPSGKYSSNLGNFNYEQRGGFRGSRGGRGWGGRGNRSRGRLY

>541

MDNIEHLYKCYEILSDAGDKISEHVSEYKDILKAVKGSSKEKRLASQFIGKFFKHFPDLSDTAIDAQLDLCEDDDMQIRRQAIKDLPKLCQDTVGITAKVGDTLAQLLVLDDPLELQQVNNSLQTIIKMDCKGSLTGIFTQISNGDEPTRERCFKFIATKLFAMGPTIDTKEIEEFIIDEIKKILQDVTADEFHLCMNILGSTKLGTTITGHAELVNLAKEQAELNADIDAITIEDEIVERFIQCATHAMPYFSNTIKSTEFVVYVCDKLLPLSTWNMIATTAGQDQVQLRLLKVFAEMCTFCDVLDNATQRIDNIYQVLREYMPLPQLKDDEDISSPPPSFQFSHAECLLYAVHTLGKKHPDSLSFVNDADKLKDFRSRLQYLARGTQGYIKKLEEAVKGKSAEDLKSEENQLKLTALKTTSNISTLIRDLFHSPPSFKHDIQLSWVKRKNNKIGSKRHAPITFDGKAENGKDEEKKTKNATDQKIYSPPSGKYSAKVQNYGNNQNNRQRSRNSGGGGGGYRNRRNFKKY

>542

MAVTIEDLYRNYGILADAKDNLSQHKDAYQVILDGVKGGPKEKRLAAQFIPKFFSSFPELADAAINAQLDLCEDEDVSIRRQAIKELPRFATGENILRVADILTQLLQTDDTAEFNQVNAALISIFKIDAKGTLGGLFSQILQGEDIVRERAIKFLSTKLKTLPEDVTTKEVEEYVFAETKKVLEDVTGEEFVLLMRVVSGLRVLQTVHGRQQLVELVVEQAFLEQALNPADPDTVDRLLQCTRQALPLFSKNVHSTRFVTYFCEHVLPNLSTLTSPVAELDIQLEVLKLLAEMSPFCGDMEKLEANLNMLFTKLLEFMPLPPEEVENGENSTSEEPKLQFSYVECLLFGFHQLGKKLPDFLLDKVDAERLKDFKIRLQYFARGLQVYIRQLRVALQGKTGDALKTEENKIKVVALKITNNINVLIKDLFHNPPSYKSTVTLSWKPVQKTEAVAPKRPSGEEMGSGGSTKKPISPLPRRDARQIYNPPSERGGFRGGRGRGFGARGNRSRGRIY

>543

MAVTIEDLYRNYGILADAKDNLSQHKDAYQVILDGVKGGPKEKRLAAQFIPKFFSSFPELADAAINAQLDLCEDEDVSIRRQAIKELPRFATGENILRVADILTQLLQTDDTAEFNQVNAALISIFKIDAKGTLGGLFSQILQGEDIVRERAIKFLSTKLKTLPEDVTTKEVEEYVFAETKKVLEDVTGEEFVLLMRVVSGLRVLQTVHGRQQLVELVVEQAFLEQALNPADPDTVDRLLQCTRQALPLFSKNVHSTRFVTYFCEHVLPNLSTLTSPVAELDIQLEVLKLLAEMSPFCGDMEKLEANLNMLFTKLLEFMPLPPEEVENGENSTSEEPKLQFSYVECLLFGFHQLGKKLPDFLLDKVDAERLKDFKIRLQYFARGLQVYIRQLRVALQGKTGDALKTEENKIKVVALKITNNINVLIKDLFHNPPSYKSTVTLSWKPVQKTEAVAPKRPSGEEMGSGGSTKKPISPLPRRDARQIYNPPSGKYSASIGNFNYERGGFRGGRGRGFGARGNRSRGRIY

>544

MHKDAYQAILDGVKGDAKEKRLAAQFIPKFFKHFPELADSAINAQLDLCEDEDVSVDLFIQLFLKMTRYDSAEFNLVNNALLSIFKMDAKGTLGGLFSQILQGEDIVRERAIKFLSTKLKTLPEEVMTKEVEEFILTESKKEYMPLPPEEAENGENAGNEEPKLQFSYVECLLYSFHQLGRKLPDFLTAKLNAEKLKDFKIRLQYFARGLQVYIRQLRLALQGKTGEALKTEENKIKVVALKITNNINVLIKSKEVVSGVDEEEAGEDVVIVAEEESTKQTTKSSVALKGESESATQQVCLEYRFLRGLPSTKTDLNVLYTFVCMIYFSNGLQLVHSETTTVFLDAYSQQFWVLIFEVSSGFIQKWKRA

>545

MDNIERLYKCYEILSEAGDKITEHVAEYKEILQAVKGTSKEKRLASQFIGNFFKHFPELSDTAIDAQFDLCEDDDTQIRRQAIKDLPKLCQGNSEATVRVGDTLAQLLILDDATELQQVNNSLLSIIKLDTKNAITGLFQQITTGDETTRERCFKFIATKLLTMGPNVITKEIEDYIVEEVKKALQDVTADEFHLCMTILGATKLGSTITGHAELVKLATEQAELNTVDADTLAVDDEVVERFIQCATAAAPYFSKTIKSTQFVSYVCDKLLPINTWNLIATAVAQDQIQLRLLKVFAEMITNTDKLDNANERINAVYNVLLEYMPLPKLSDEDTADVPPSFQFSHAECLLYALHTLGKKHPSNLTFVEDAEKLKDFRARLQYLARGTQGYIKKLEEALKGKSAEELKTEENQLKQTALKTTSNINVLIRDLFHSPPIFKHDIVLSWVVPKNSKLGKRHVPITFGDKGSANGNQEQEQQQDKKSRPSNEQKFYSPPSGKYSGKVNSNYGNNNRARQRGGGGGGGGGNFRNRRYNRY

>546

MDNIERLYKCYEILSEAGEKISEHVEEYKEILKAVKGSSKEKRLASQFIGSFYKHFPDLADTAIDAQFDLCEDDDTQIRRQAIKDLPKLCQGNAEATARVGDTLAQLLILDDPTELQQVNNSLLSIIKLDTKSAVTGLFQQITTGDEPTRERCLKFIATKLLTMGPTVITKEIEEYIVEEIKKALQDVTADEFHLCMSILGATKLGNTITGHAELVKLATEQAELNAPESADNIAVDDEIVERFIQCASAAAPYFSKTIKSTPFVSYVCDKLLPLNTWKLIATAVSQDQIQLRLLKVFAEMIANTDKLDNASDRINNVYNVLLEYMPLPKLSDDDLTVGDATPPSFEFSHAECLLYALHTLGKKHPTNLTFVEDAEKLKDFRARLQYLARGTQGYIKKLEESLKGKAAAELKTEENQLKQTALKTTSNINVLIRDLFHSPPIFKHDVVLSWIVPKNNKLGKRHTPITFGDKPEEANGKEQAEEKKPRQSNEQKFYSPPSGKYSNRVNQNYGNNQRSRQRGGGGGGGGGGGGGYRNRRYNRY

>547

MATVEQLYKDYGILADAKDKAGEHEAAYNSILSAVDGGSSEKRLAAQFIPKFFKHFPSLSEKAIHAQLDLCEDEDSSIRRQAIKELPNLCRASPDNHFHIADVLTQLLQSDDPQDTSTVNSALMALFKLDAENTLGGLFNQILTGEDLVRDRAIKFLSNRIKTFPEDITTPQVEEVLIQKCKEVLSDVTGEEFISFMKILSGTKTMQSLQGRQQLVELVVEQADFQSDFIASEPDLVDRLTQCLKQAVPLFSKNVPSTQFVLYLCEKVIPVLEEIKPAVTEKKSNGAKAGETEEKEEGKKEGEEEEAKGDGDKESPEAEERRRRREKKKMRNQQQQCILEEGKEDRVVHTWKSLSTSQKWRCIVGRSLSNHTL

>548

MWEKPAMAYGLQHTPQVYMPLPPPGTEDDTEQTQENEEDPQLQFSVVECLMHAFHCLVRKQPDFLAAEENADRLKDFRIRLQYFARGVQSSMKQLRLALKGKAGAQLKTEENKIKVVALRITSNINTLIKDLMHNPPSYKSNIVLSWKPIQKQGVATSPETAKKRPHITPITFESDKKVAAGGRNQEVPRTVLQEENTATKLAEHRPGETLITATGDHPTMAITTGTKDEVEIADVSTEIVGERDTENWRQIQS

>549

MATVEQLYKDYGILADAKDKAGEHEAAYNSILSAVDGGSSEKRLAAQFIPKFFKHFPSLSEKAIHAQLDLCEDEDSSIRRQAIKELPNLCRASPDNHFHIADVLTQLLQSDDPQDTSTVNSALMALFKLDAESLIRRHWEEFISFMKILSGTKTMQSLQGRQQLVELVVEQADFQSDFIASEPDLVDRLTQCLKQAVPLFSKNVPSTQFVLYLCEKVIPVLEEIKPAVTEKKSNGAKEGETEEGKEEEKKEGEEEEAKEDGDKESPEAEEKEEEEKEKKKMRNNRQWYLQKKGKKTGVVHTWKF

>550

MYVLRFLSTVWCFSVASQVHVQAIGGLPDFCKDTPENIGKMVDILVQILGSEEFVERDAVHKALISSLRQDVKG

>551

MAVTIEDLYRNYGILADAKDNLSQHKDAYQVILDGVKGGPKEKRLAAQFIPKFFSSFPELADAAINAQLDLCEDEDVSIRRQAIKELPRFATGENILRVADILTQLLQTDDTAEFNQVNAALISIFKIDAKGTLGGLFSQILQGEDIVRERAIKFLSTKLKTLPEDVTTKEVEEYVFAETKKVLEDVTGEEFVLLMRVVSGLRVLQTVHGRQQLVELVVEQAFLEQALNPADPDTVDRLLQCTRQALPLFSKNVHSTRFVTYFCEHVLPNLSTLTSPVAELDIQLEVLKLLAEMSPFCGDMEKLEANLNMLFTKLLEFMPLPPEEVENGENSTSEEPKLQFSYVECLLFGFHQLGKKLPDFLLDKVDAERLKDFKIRLQYFARGLQVYIRQLRVALQGKTGDALKTEENKIKVVALKITNNINVLIKDLFHNPPSYKSTVTLSWKPVQKTEAVAPKRPSGEEMGSGGSTKKQISPLPRRDARQIYNPPSERGGFRGGRGRGFGARGNRSRGRIY

>552

MAVTIEDLYRNYGILADAKDNLSQHKDAYQVILDGVKGGPKEKRLAAQFIPKFFSSFPELADAAINAQLDLCEDEDVSIRRQAIKELPRFATGENILRVADILTQLLQTDDTAEFNQVNAALISIFKIDAKGTLGGLFSQILQGEDIVRERAIKFLSTKLKTLPEDVTTKEVEEYVFAETKKVLEDVTGEEFVLLMRVVSGLRVLQTVHGRQQLVELVVEQAFLEQALNPADPDTVDRLLQCTRQALPLFSKNVHSTRFVTYFCEHVLPNLSTLTSPVAELDIQLEVLKLLAEMSPFCGDMEKLEANLNMLFTKLLEFMPLPPEEVENGENSTSEEPKLQFSYVECLLFGFHQLGKKLPDFLLDKVDAERLKDFKIRLQYFARGLQVYIRQLRVALQGKTGDALKTEENKIKVVALKITNNINVLIKDLFHNPPSYKSTVTLSWKPVQKTEAVAPKRPSGEEMGSGGSTKKQISPLPRRDARQIYNPPSGKYSASIGNFNYERGGFRGGRGRGFGARGNRSRGRIY

>553

MPTVEELYRNYGILADATEQVGQHKDAYQVILDGVKGGTKEKRLAAQFIPKFFKHFPELADSAINAQLDLCEDEDVSIRRQAIKELPQFAAGENLPRVADILTQLLQTDDSAEFNLVNNALLSIFKMEKLETNLRKLFDKLLEYMPLPPEEAENGENAGNEEPKLQFSYVECLLYSFHQLGRKLPDFLTAKLNAEKLKDFKIRLQYFARGLQVYIRQLRLALQGKTGEALKTEENKIKVVALKITNNINVLIKDLFHIPPSYKSTVTLSWKPVQKVEIGQKRANEDTTSGSPPKKSSAGPKRDARQIYNPPSGKYSSNLSNFNYERSLQGK

>554

MPTVEELYRNYGILADATEQVGQIRRQAIKELPQFAAGENLPRVADILTQLLQTDDSAEFNLVNNALLSIFKMDAKGTLGGLFSQILQGEDIVRERAIKFLSTKLKTLPDEVLTKEVEELILTESKKVLEDVTGEEFVLFMKILSGLKSLQTVSGRQQLVELVAEQADLEQTFNPSDPDCVDRLLQCTRQAVPLFSKNVHSTRFVTYFCEQVLPNLSSLTTPVEGLDIQLEVLKLLAEMSSFCGDMEKLETNLRKLFDKLLEYMPLPPEEAENGENAGNEEPKLQFSYVECLLYSFHQLGRKLPDFLTAKLNAEKLKDFKIRLQYFARGLQVYIRQLRLALQGKTGEALKTEENKIKVVALKITNNINVLIKDLFHIPPSYKSTVTLSWKPVQKVEIGQKRANEDTTSGSPPKKSSAGPKRDARQIYNPPSGKYSSNLSNFNYERSLQGK

>555

MPTVEELYRNYGILADATEQVGQHKDAYQVILDGVKGGTKEKRLAAQFIPKFFKHFPELADSAINAQLDLCEDEDVSIRRQAIKELPQFAAGENLPRVADILTQLLQTDDSAEFNLVNNALLSIFKMDAKGTLGGLFSQILQGEDIVRERAIKFLSTKLKTLPDEVLTKEVEELILTESKKVLEDVTGEEFVLFMKILSGLKSLQTVSGRQQLVELVAEQADLEQTFNPSDPDCVDRLLQCTRQAVPLFSKNVHSTRFVTYFCEQVLPNLSSLTTPVEGLDIQLEVLKLLAEMSSFCGDMEKLETNLRKLFDKLLEYMPLPPEEAENGENAGNEEPKLQFSYVECLLYSFHQLGRKLPDFLTAKLNAEKLKDFKIRLQYFARGLQVYIRQLRLALQGKTGEALKTEENKIKVVALKITNNINVLIKDLFHIPPSYKSTVTLSWKPVQKVEIGQKRANEDTTSGSPPKKSSAGPKRDARQIYNPPSGKYSSNLSNFNYERSLQGK

>556

MPTVEELYRNYGILADATEQVGQHKDAYQVILDGVKGGTKEKRLAAQFIPKFFKHFPELADSAINAQLDLCEDEDVSIRRQAIKELPQFAAGENLPRVADILTQLLQTDDSAEFNLVNNALLSIFKMDAKGTLGGLFSQILQGEDIVRERAIKFLSTKLKTLPDEVLTKEVEELILTESKKVLEDVTGEEFVLFMKILSGLKSLQTVSGRQQLVELVAEQADLEQTFNPSDPDCVDRLLQCTRQAVPLFSKNVHSTRFVTYFCEQVLPNLSSLTTPVEGLDIQLEVLKLLAEMSSFCGDMEKLETNLRKLFDKLLEYMPLPPEEAENGENAGNEEPKLQFSYVECLLYSFHQLGRKLPDFLTAKLNAEKLKDFKIRLQYFARGLQVYIRQLRLALQGKTGEALKTEENKIKVVALKITNNINVLIKDLFHIPPSYKSTVTLSWKPVQKVEIGQKRANEDTTSGSPPKKSSAGPKRDARQIYNPPSGKYSSNLSNFNYEQRGAFRGSRGGRGWGARGNRSRGRLY

>557

MDNIERLYKCYEILSEAGDKISEHVDEYKEILKAVKGSSKEKRLASQFIGNFFKHFPDLAETAIDAQFDLCEDDDNQIRRQAIKDLPKLCQGNAEATTRVGDTLAQLLILDDATELQQVNNSLISIIKMDTKSAVAGIFQQISTGDEPTRERCLKFISTKLLTMGPTVITKEIEEYIVEEIKKALQDVTADEFHLCMTILGATKLGNTITGHAELVKLATEQAELNSTDADIIAVDDEVVERFVQCATAAAPYFSKTIKSTAFVAHVCDKLLPIPTWNMIATAVSQDQIQLRLLKVFAEMIANTDKLENANERINNVYNVLLEYMPLPKLSEVDLVDVPPSFEFSHAECLLYALHTLGKKHPTNLSFVEDAEKLKDFRARLQYLARGTQGYIKKLEEALKGKSAEELKTEENQLKQTALKTTSNINVLIRDLFHSPPIFKHDIVLSWIVPKANKLGKRHAPITFGEKPEANGKEKEQDVEKKSRPSNDQKFYSPPSGKYSHKVNPNYGNNNRGRQRGGGGGGYRNRRYNRY

>558

MNGNDCIEKLYRNFGVLADAKDKIAEHEKEYLEILMAVKGSEKEKRLASQFIARFFKHFPNLADQAIEAQLDLCEDEDVSIRKQATKDLPSLCKDNKEHTQRIADILAQLLQAEDKSELAVVQNSLMTLFKIDAKGSLAGLFAHILNGEDAVRDRCMKFLGGKLKALGHEVVNREAEDYLIAEAKKVLQDVTADEFHILMEVLVWTRLGQSPAGHRELVEIVAEQALGEPDFDPSDDEHVDRLVHSAKHALPYFSSQIDSSKFVVYMCELVLPRLSEVSSADENSDPQLDILKLFAELCTHCNKLPDPSASVQCVFNTLLSFMPPPPMTDGEEQEEPKLFFSYVECLMYSLHRLARLCPEFLTQDADRLKDFRLRLQYFARGIQGYIKKLREALQGKTGEELKTEENKIKVVALKTTSNINTLIKDLFHSPPSFKSTISLSWKPARLTNNTKDGSHRGGGDAMKRHTPITFGEGGAPDSKQSKQDSRGNRELYQPPGGKYSDKVSQYSPSQLQQRGGRPRGGSGGGRGARLSGGGGGFRGGGSRGWRRGY

>559

MSTDRIEKLYKNFGILADSKDKSEHEAEYLEILTAVKGSSKEKRLASQFIARFFKHFPTLADQAIEAQLDLCEDEDVAIRKQAIKDLPSLCKDNKEHTQKIADILAQLLQAEDNAELSVVHNSLMTLFKSDAKGTLGGIFSQILSGDDLIRERCIKFLTVKIKALGHDVITKDAEEHLISECKKVLQDVTADEFHNIMELLGWTRLGHSVTGQQELVDIVAEQAEINQNFDPKDVENDNVDRLIQCVKHALPYFSSQVDSARFVAYMCEQVLPQFDEISSNEDGADPQLEILKLFAELCTHCGXXXXXXXKVEKVFDRLIEYMPLPPDSDSEKTNQDEPRLEFSYVECLMYAFHRLGRQCPDFLTKDADRMKDFRFRLQYFARGIQGYIKKLREALQGKTADELKTEENKIKVVALKTTSNINTLIKDLFHSPPSYKSIISLSWKPTAGSAKDGPSAGHKRHTPITYSSDSAANKHPKTDKSQREIYQPPSGKYSNKVSSYVPPQQRGRGRGRGRSFRGVRGWRRNY

>560

MHSYDLCEDEDVSIRRQAIKELPQFATGENLPRVADILTQLLQTDDSAEFNLVNNALLSIFKMDAKGTLGGLFSQILQGEDIVRERAIKFLSTKLKTLPDEVLTKEVEELILTESKKVLEDVTGEEFVLFMKILSGLKSLQTVSGRQQLVELVAEQADLEQTFNPSDPDCVDRLLQCTRQAVPLFSKNVHSTRFVTYFCEQVLPNLGTLTTPVEGLDIQLEVLKLLAEMSSFCGDMEKLETNLRKLFDKLLEYMPLPPEEAENGENAGNEEPKLQFSYVECLLYSFHQLGRKLPDFLTAKLNAEKLKDFKIRLQYFARGLQVYIRQLRLALQGKTGEALKTEENKIKVVALKITNNINVLIKDLFHIPPSYKSTVTLSWKPVQKVEIGQKRASEDTTSGSPPKKSSAGPKRDARQIYNPPSGKYSSNLGNFNYGSQI

>561

MHSYDLCEDEDVSIRRQAIKELPQFATGENLPRVADILTQLLQTDDSAEFNLVNNALLSIFKMDAKGTLGGLFSQILQGEDIVRERAIKFLSTKLKTLPDEVLTKEVEELILTESKKVLEDVTGEEFVLFMKILSGLKSLQTVSGRQQLVELVAEQADLEQTFNPSDPDCVDRLLQCTRQAVPLFSKNVHSTRFVTYFCEQVLPNLGTLTTPVEGLDIQLEVLKLLAEMSSFCGDMEKLETNLRKLFDKLLEYMPLPPEEAENGENAGNEEPKLQFSYVECLLYSFHQLGRKLPDFLTAKLNAEKLKDFKIRLQYFARGLQVYIRQLRLALQGKTGEALKTEENKIKVVALKITNNINVLIKDLFHIPPSYKSTVTLSWKPVQKVEIGQKRASEDTTSGSPPKKSSAGPKRDARQIYNPPSGKYSSNLGNFNYERSLQGK

>562

MHSYDLCEDEDVSIRRQAIKELPQFATGENLPRVADILTQLLQTDDSAEFNLVNNALLSIFKMDAKGTLGGLFSQILQGEDIVRERAIKFLSTKLKTLPDEVLTKEVEELILTESKKVLEDVTGEEFVLFMKILSGLKSLQTVSGRQQLVELVAEQADLEQTFNPSDPDCVDRLLQCTRQAVPLFSKNVHSTRFVTYFCEQVLPNLGTLTTPVEGLDIQLEVLKLLAEMSSFCGDMEKLETNLRKLFDKLLEYMPLPPEEAENGENAGNEEPKLQFSYVECLLYSFHQLGRKLPDFLTAKLNAEKLKDFKIRLQYFARGLQVYIRQLRLALQGKTGEALKTEENKIKVVALKITNNINVLIKDLFHIPPSYKSTVTLSWKPVQKVEIGQKRASEDTTSGSPPKKSSAGPKRDARQIYNPPSGKYSSNLGNFNYEQRGAFRGSRGGRGWGTRGNRSRGRLY

>563

MDSSGDSIEKLYKNYEILTDAKDKIGEHELEYREILDAVKGSAKEKRLASQFIGKFFKHFPNLAELAIDRQLDLCEDEDAQIRKQAIKDLPQLCKDTKEHTPKIADILAQLLITEDVTELQQVHQSLLTLAKFDATGTLTGIFSQIVSGDEPTRYRNFQFILNKLIKIGPEVITKEVEDFVIAEIKKILLDVSADEFHLCMSILNQTKLSKTVTGHAELVAIAVEQADMEADLGSLASDDETVERFIQCASEAMPYFSSQVESTQFIKFMCEKLLPLNVWNLIGAGEDQHTTQLRLLKVFAEMCAFCGSLEKPAEKVEAIYNVLLEYMPLPPADADMNETPSFQFSHAECLLHALHTLGKQAAEFLTFPEDAAKLKDFRSRLQYLARGTQGYIKKLQESVKGKTTEEMKSEENQIKATALKTTSNISTLIRDLFHTPPSFKSVIHLSWLPPKSKAVVDAKTTAKRHAAITFEENGKSHDSKGPKHAKSSSGTQKVYTPPSGKYSGKVQNYSTKNGSGGGSSNGGRRSGGGGGSGGGGGFRRSNFGGRSGGGGGRRRY

>564

MPTVEELYRNYGILADATEQVGQHKDAYQVILDGVKGGTKEKRLAAQFIPKFFKHFPELADSAINAQLDLCEDEDVSIRRQAIKELPQFATGENLPRVADILTQLLQTDDSAEFNLVNNALLSIFKMDAKGTLGGLFSQILQGEDIVRERAIKFLSTKLKTLPDEVLTKEVEELILTESKKVLEDVTGEEFVLFMKILSGLKSLQTVSGRQQLVELVAEQADLEQTFNPSDPDCVDRLLQCTRQAVPLFSKNVHSTRFVTYFCEQVLPNLSSLTTPVEGLDIQLEVLKLLAEMSSFCGDMEKLETNLRKLFDKLLEYMPLPPEEAENGENAGNEEPKLQFSYVECLLYSFHQLGRKLPDFLTAKLNAEKLKDFKIRLQYFARGLQVYIRQLRLALQGKTGEALKTEENKIKVVALKITNNINVLIKDLFHIPPSYKSTVTLSWKPVQKVEIGQKRASEDTTSGSPPKKSPAGPKRDARQIYNPPSGKYSSNLSNFNYERSLQGK

>565

MPTVEELYRNYGILADATEQVGQHKDAYQVILDGVKGGTKEKRLAAQFIPKFFKHFPELADSAINAQLDLCEDEDVSIRRQAIKELPQFATGENLPRVADILTQLLQTDDSAEFNLVNNALLSIFKMDAKGTLGGLFSQILQGEDIVRERAIKFLSTKLKTLPDEVLTKEVEELILTESKKVLEDVTGEEFVLFMKILSGLKSLQTVSGRQQLVELVAEQADLEQTFNPSDPDCVDRLLQCTRQAVPLFSKNVHSTRFVTYFCEQVLPNLSSLTTPVEGLDIQLEVLKLLAEMSSFCGDMEKLETNLRKLFDKLLEYMPLPPEEAENGENAGNEEPKLQFSYVECLLYSFHQLGRKLPDFLTAKLNAEKLKDFKIRLQYFARGLQVYIRQLRLALQGKTGEALKTEENKIKVVALKITNNINVLIKDLFHIPPSYKSTVTLSWKPVQKVEIGQKRASEDTTSGSPPKKSPAGPKRDARQIYNPPSGKYSSNLSNFNYEQRGAFRGSRGGRGWGARGNRSRGRLY

>566

MPTVEELYRNYGILADATEQVGQHKDAYQVILDGVKGGTKEKRLAAQFIPKFFKHFPELADSAINAQLDLCEDEDVSIRRQAIKELPQFATGENLPRVADILTQLLQTDDSAEFNLVNNALLSIFKMDAKGTLGGLFSQILQGEDIVRERAIKFLSTKLKTLPDEVLTKEVEELILTESKKVLEDVTGEEFVLFMKILSGLKSLQTVSGRQQLVELVAEQADLEQTFNPSDPDCVDRLLQCTRQAVPLFSKNVHSTRFVTYFCEQVLPNLGTLTTPVEGLDIQLEVLKLLAEMSSFCGDMEKLETNLRKLFDKLLEYMPLPPEEAENGENAGNEEPKLQFSYVECLLYSFHQLGRKLPDFLTAKLNAEKLKDFKIRLQYFARGLQVYIRQLRLALQGKTGEALKTEENKIKVVALKITNNINVLIKDLFHIPPSYKSTVTLSWKPVQKVEIGQKRASEDTTSGSPAKKSSAGPKRDARQIYNPPSGKYSSNLGNFNYERSLQGK

>567

MPTVEELYRNYGILADATEQVGQHKDAYQVILDGVKGGTKEKRLAAQFIPKFFKHFPELADSAINAQLDLCEDEDVSIRRQAIKELPQFATGENLPRVADILTQLLQTDDSAEFNLVNNALLSIFKMDAKGTLGGLFSQILQGEDIVRERAIKFLSTKLKTLPDEVLTKEVEELILTESKKVLEDVTGEEFVLFMKILSGLKSLQTVSGRQQLVELVAEQADLEQTFNPSDPDCVDRLLQCTRQAVPLFSKNVHSTRFVTYFCEQVLPNLGTLTTPVEGLDIQLEVLKLLAEMSSFCGDMEKLETNLRKLFDKLLEYMPLPPEEAENGENAGNEEPKLQFSYVECLLYSFHQLGRKLPDFLTAKLNAEKLKDFKIRLQYFARGLQVYIRQLRLALQGKTGEALKTEENKIKVVALKITNNINVLIKDLFHIPPSYKSTVTLSWKPVQKVEIGQKRASEDTTSGSPAKKSSAGPKRDARQIYNPPSGKYSSNLGNFNYEQRGAFRGSRGGRGWGTRGNRSRGRLY

>568

MPTVEELYRNYGILADATEQVGQHKDAYQVILDGVKGGTKEKRLAAQFIPKFFKHFPELADSAINAQLDLCEDEDVSIRRQAIKELPQFATGENLPRVADILTQLLQTDDSAEFNLVNNALLSIFKMDAKGTLGGLFSQILQGEDIVRERAIKFLSTKLKALPEEVLTKEVEELVLTESKKVLEDVTGEEFVLFMKILSGLKSLQTVSGRQQLVELVAEQADLEQTFSPSDPDCVDRLLQCTRQAVPLFSKNVHSTRFVTYFCEQVLPNLSTLTTPVEGLDIQLEVLKLLAEMSSFCGDMEKLETNLRKLFDKLLEYMPLPPEEAENGENAGNEEPKLQFSYVECLLYSFHQLGRKLPDFLTAKLNAEKLKDFKIRLQYFARGLQVYIRQLRLALQGKTGEALKTEENKIKVVALKITNNINVLIKDLFHIPPSYKSTVTLSWKPVQKVEIGQKRTSEDTSSGSPPKKSPGGPKRDARQIYNPPSGKYSSNLGNFNYERSLQGK

>569

MPTVEELYRNYGILADATEQVGQHKDAYQVILDGVKGGTKEKRLAAQFIPKFFKHFPELADSAINAQLDLCEDEDVSIRRQAIKELPQFATGENLPRVADILTQLLQTDDSAEFNLVNNALLSIFKMDAKGTLGGLFSQILQGEDIVRERAIKFLSTKLKALPEEVLTKEVEELVLTESKKVLEDVTGEEFVLFMKILSGLKSLQTVSGRQQLVELVAEQADLEQTFSPSDPDCVDRLLQCTRQAVPLFSKNVHSTRFVTYFCEQVLPNLSTLTTPVEGLDIQLEVLKLLAEMSSFCGDMEKLETNLRKLFDKLLEYMPLPPEEAENGENAGNEEPKLQFSYVECLLYSFHQLGRKLPDFLTAKLNAEKLKDFKIRLQYFARGLQVYIRQLRLALQGKTGEALKTEENKIKVVALKITNNINVLIKDLFHIPPSYKSTVTLSWKPVQKVEIGQKRTSEDTSSGSPPKKSPGGPKRDARQIYNPPSGKYSSNLGNFNYEQRGAFRGSRGGRGWGTRGNRSRGRLY

>570

MRRPGYLRHKDAYQVILDGVKGGAKEKRLAAQFIPKFFKHFPELADSAINAQLDLCEDEDVSIRRQAIKELPQFATGDNLPRVADILTQLLQSDDSAEFNLVNNALLSIFKMDAKGTLGGLFSQILQGEDIVRERAIKFLSTKLKTLPEEVLTKEVEEFILAESKKVLEDVTGEEFVLFMKILSGLKSLQTVSGRQQLVELVAEQADLEQTFNPSDPDCVDRLLQCTRQAVPLFSKNVHSTKFVTYFCEHVLPSLSSLTTPVEGLDIQLEVLKLLAEMSSFCGDMEKLESNLKKLFDKLLEYMPLPPEEAENGENAGGEEPKLQFSYVECLLYSFHQLGRKLPDFLTAKLNAEKLKDFKIRLQYFARGLQVYIRQLRLALQGKTGEALKTEENKIKVVALKITNNINVLIKDLFHIPPSYKSTVTLSWKPVQKADASQKRTSEDTTSSSPPKKASAGPKRDARQIYNPPSGKYSSNLGSFSYEQRGGFRGGRGRGWGGRGNRSRGRIY

>571

MPTVEELYRNYGILADATETAGQHKDAYQVILDGVKGGAKEKRLAAQFIPKFFKHFPELADSAINAQLDLCEDEDVSIRRQAIKELPQFATGDNLPRVADILTQLLQSDDSAEFNLVNNALLSIFKMDAKGTLGGLFSQILQGEDIVRERAIKFLSTKLKTLPEEVLTKEVEEFILAESKKVLEDVTGEEFVLFMKILSGLKSLQTVSGRQQLVELVAEQADLEQTFNPSDPDCVDRLLQCTRQAVPLFSKNVHSTKFVTYFCEHVLPSLSSLTTPVEGLDIQLEVLKLLAEMSSFCGDMEKLESNLKKLFDKLLEYMPLPPEEAENGENAGGEEPKLQFSYVECLLYSFHQLGRKLPDFLTAKLNAEKLKDFKIRLQYFARGLQVYIRQLRLALQGKTGEALKTEENKIKVVALKITNNINVLIKDLFHIPPSYKSTVTLSWKPVQKADASQKRTSEDTTSSSPPKKASAGPKRDARQIYNPPSGKYSSNLGSFSYEQRGGFRGGRGRGWGGRGNRSRGRIY

>572

MPTVEELYRNYGILADATEQVGQHKDAYQVILDGVKGGTKEKRLAAQFIPKFFKHFPELADSAINAQLDLCEDEDVSIRRQAIKELPQFATGENLPRVADILTQLLQTDDSAEFNLVNNALLSIFKMDAKGTLGGLFSQILQGEDIVRERAIKFLSTKLKTLPDEVLTKEVEELILTESKKVLEDVTGEEFVLFMKILSGLKSLQTVSGRQQLVELVAEQADLEQTFNPSDPDCVDRLLQCTRQAVPLFSKNVHSTRFVTYFCEQVLPNLSTLTTPVEGLDIQLEVLKLLAEMSSFCGDMEKLETNLRKLFDKLLEYMPLPPEEAENGENAGNEEPKLQFSYVECLLYSFHQLGRKLPDFLTAKLNAEKLKDFKIRLQYFARGLQVYIRQLRLALQGKTGEALKTEENKIKVVALKITNNINVLIKDLFHIPPSYKSTVTLSWKPVQKVEIGQKRANEDTTSGSPPKKSTAGPKRDARQIYNPPSGKYSSNLSNFNYERSLQGK

>573

MPTVEELYRNYGILADATEQVGQHKDAYQVILDGVKGGTKEKRLAAQFIPKFFKHFPELADSAINAQLDLCEDEDVSIRRQAIKELPQFATGENLPRVADILTQLLQTDDSAEFNLVNNALLSIFKMDAKGTLGGLFSQILQGEDIVRERAIKFLSTKLKTLPDEVLTKEVEELILTESKKVLEDVTGEEFVLFMKILSGLKSLQTVSGRQQLVELVAEQADLEQTFNPSDPDCVDRLLQCTRQAVPLFSKNVHSTRFVTYFCEQVLPNLSTLTTPVEGLDIQLEVLKLLAEMSSFCGDMEKLETNLRKLFDKLLEYMPLPPEEAENGENAGNEEPKLQFSYVECLLYSFHQLGRKLPDFLTAKLNAEKLKDFKIRLQYFARGLQVYIRQLRLALQGKTGEALKTEENKIKVVALKITNNINVLIKDLFHIPPSYKSTVTLSWKPVQKVEIGQKRANEDTTSGSPPKKSTAGPKRDARQIYNPPSGKYSSNLSNFNYEQRGAFRGSRGGRGWGTRGNRSRGRLY

>574

MPTVEELYRNYGILADATEQVGQHKDAYQVILDGVKGGTKEKRLAAQFIPKFFKHFPELADSAINAQLDLCEDEDVSIRRQAIKELPQFATGENLPRVADILTQLLQTDDSAEFNLVNNALLSIFKMDAKGTLGGLFSQILQGEDIVRERAIKFLSTKLKTLPDEVLTKEVEELILTESKKVLEDVTGEEFVLFMKILSGLKSLQTVSGRQQLVELVAEQADLEQTFSPSDPDCVDRLLQCTRQAVPLFSKNVHSTRFVTYFCEQVLPNLSTLTTPVEGLDIQLEVLKLLAEMSSFCGDMEKLETNLRKLFDKLLEYMPLPPEEAENGENASNEEPKLQFSYVECLLYSFHQLGRKLPDFLTAKLNADKLKDFKIRLQYFARGLQVYIRQLRLALQGKTGEALKTEENKIKVVALKITNNINVLIKDLFHIPPSYKSTVTLSWKPVQKVEIGQKRANEDTSSGSPPKKSPGGPKRDARQIYNPPSGKYSSNLGNFNYGERFRLGTSSPRD

>575

MPTVEELYRNYGILADATEQVGQHKDAYQVILDGVKGGTKEKRLAAQFIPKFFKHFPELADSAINAQLDLCEDEDVSIRRQAIKELPQFATGENLPRVADILTQLLQTDDSAEFNLVNNALLSIFKMDAKGTLGGLFSQILQGEDIVRERAIKFLSTKLKTLPDEVLTKEVEELILTESKKVLEDVTGEEFVLFMKILSGLKSLQTVSGRQQLVELVAEQADLEQTFSPSDPDCVDRLLQCTRQAVPLFSKNVHSTRFVTYFCEQVLPNLSTLTTPVEGLDIQLEVLKLLAEMSSFCGDMEKLETNLRKLFDKLLEYMPLPPEEAENGENASNEEPKLQFSYVECLLYSFHQLGRKLPDFLTAKLNADKLKDFKIRLQYFARGLQVYIRQLRLALQGKTGEALKTEENKIKVVALKITNNINVLIKDLFHIPPSYKSTVTLSWKPVQKVEIGQKRANEDTSSGSPPKKSPGGPKRDARQIYNPPSGKYSSNLGNFNYERSLQGK

>576

MPTVEELYRNYGILADATEQVGQHKDAYQVILDGVKGGTKEKRLAAQFIPKFFKHFPELADSAINAQLDLCEDEDVSIRRQAIKELPQFATGENLPRVADILTQLLQTDDSAEFNLVNNALLSIFKMDAKGTLGGLFSQILQGEDIVRERAIKFLSTKLKTLPDEVLTKEVEELILTESKKVLEDVTGEEFVLFMKILSGLKSLQTVSGRQQLVELVAEQADLEQTFSPSDPDCVDRLLQCTRQAVPLFSKNVHSTRFVTYFCEQVLPNLSTLTTPVEGLDIQLEVLKLLAEMSSFCGDMEKLETNLRKLFDKLLEYMPLPPEEAENGENASNEEPKLQFSYVECLLYSFHQLGRKLPDFLTAKLNADKLKDFKIRLQYFARGLQVYIRQLRLALQGKTGEALKTEENKIKVVALKITNNINVLIKDLFHIPPSYKSTVTLSWKPVQKVEIGQKRANEDTSSGSPPKKSPGGPKRDARQIYNPPSGKYSSNLGNFNYEQRGAFRGSRGGRGWGTRGNRSRGRLY

>577

MPTVEELYRNYGILADATEQVGQHKDAYQVILGGVKGSTKEKQLAVQFIPKFFKHFPELADSAINAQLDLCEDEDVSIRRQAIKELPQFATGENLPRVADILTQLLQTDDSAEFNLVNNALLSIFKMDAKGTLGGLFSQILQGEDIVRERAIKFLSTKLKTLPDEVLTKEVEELILTESKKVLEDVTGEEFVLFMKILSGLKSLQTVSGRQQLVELVAEQADLEQTFSPSDPDCVDRLLQCTRQAVPLFSKNVHSTRFVTYFCEQVLPNLSTLTTPVEGLDIQLEVLKLLAEMSSFCGDMEKLETNLRKLFDKLLEYMPLPPEEAENGENAGNEEPKLQFSYVECLLYSFHQLGRKLPDFLTAKLNAEKLKDFKIRLQYFARGLQVYIRQLRLALQGKTGEALKTEENKIKVVALKITNNINVLIKDLFHIPPSYKSTVTLSWKPVQKVEIGQKRTSEDTSSGSPPKKSPGGPKRDARQIYNPPSGKYSSNLGNFSYERSLQGK

>578

MPTVEELYRNYGILADATEQVGQHKDAYQVILGGVKGSTKEKQLAVQFIPKFFKHFPELADSAINAQLDLCEDEDVSIRRQAIKELPQFATGENLPRVADILTQLLQTDDSAEFNLVNNALLSIFKMDAKGTLGGLFSQILQGEDIVRERAIKFLSTKLKTLPDEVLTKEVEELILTESKKVLEDVTGEEFVLFMKILSGLKSLQTVSGRQQLVELVAEQADLEQTFSPSDPDCVDRLLQCTRQAVPLFSKNVHSTRFVTYFCEQVLPNLSTLTTPVEGLDIQLEVLKLLAEMSSFCGDMEKLETNLRKLFDKLLEYMPLPPEEAENGENAGNEEPKLQFSYVECLLYSFHQLGRKLPDFLTAKLNAEKLKDFKIRLQYFARGLQVYIRQLRLALQGKTGEALKTEENKIKVVALKITNNINVLIKDLFHIPPSYKSTVTLSWKPVQKVEIGQKRTSEDTSSGSPPKKSPGGPKRDARQIYNPPSGKYSSNLGNFSYGERFRLGTSSSRD

>579

MPTVEELYRNYGILADATEQVGQHKDAYQVILGGVKGSTKEKQLAVQFIPKFFKHFPELADSAINAQLDLCEDEDVSIRRQAIKELPQFATGENLPRVADILTQLLQTDDSAEFNLVNNALLSIFKMDAKGTLGGLFSQILQGEDIVRERAIKFLSTKLKTLPDEVLTKEVEELILTESKKVLEDVTGEEFVLFMKILSGLKSLQTVSGRQQLVELVAEQADLEQTFSPSDPDCVDRLLQCTRQAVPLFSKNVHSTRFVTYFCEQVLPNLSTLTTPVEGLDIQLEVLKLLAEMSSFCGDMEKLETNLRKLFDKLLEYMPLPPEEAENGENAGNEEPKLQFSYVECLLYSFHQLGRKLPDFLTAKLNAEKLKDFKIRLQYFARGLQVYIRQLRLALQGKTGEALKTEENKIKVVALKITNNINVLIKDLFHIPPSYKSTVTLSWKPVQKVEIGQKRTSEDTSSGSPPKKSPGGPKRDARQIYNPPSGKYSSNLGNFSYEQRGAFRGNRGGRGWGTRGNRSRGRLY

>580

MPTVEELYRNYGILADATEQVGQHKDAYQVILDGVKGGTKEKRLAAQFIPKFFKHFPELADSAINAQLDLCEDEDVSIRRQAIKELPQFATGENLPRVADILTQLLQTDDSAEFNLVNNALLSIFKMEKLETNLRKLFDKLLEYMPLPPEEAENGENAGNEEPKLQFSYVECLLYSFHQLGRKLPDFLTAKLNAEKLKDFKIRLQYFARGLQVYIRQLRLALQGKTGEALKTEENKIKVVALKITNNINVLIKDLFHIPPSYKSTVTLSWKPVQKVEIGQKRTSEDTSSGSPPKKSPGGPKRDARQIYNPPSGKYSSNLGNFNYERSLQGK

>581

MPTVEELYRNYGILADATEQVGQIRRQAIKELPQFATGENLPRVADILTQLLQTDDSAEFNLVNNALLSIFKMDAKGTLGGLFSQILQGEDIVRERAIKFLSTKLKTLPDEVLTKEVEELILTESKKVLEDVTGEAFVLFMKILSGLKSLQTVSGRQQLVELVAEQADLEQTFSPSDPDCVDRLLQCTRQAVPLFSKNVHSTRFVTYFCEQVLPNLSTLTTPVEGLDIQLEVLKLLAEMSSFCGDMEKLETNLRKLFDKLLEYMPLPPEEAENGENAGNEEPKLQFSYVECLLYSFHQLGRKLPDFLTAKLNAEKLKDFKIRLQYFARGLQVYIRQLRLALQGKTGEALKTEENKIKVVALKITNNINVLIKDLFHIPPSYKSTVTLSWKPVQKVEIGQKRTSEDTSSGSPPKKSPGGPKRDARQIYNPPSGKYSSNLGNFNYERSLQGK

>582

MPTVEELYRNYGILADATEQVGQHKDAYQVILDGVKGGTKEKRLAAQFIPKFFKHFPELADSAINAQLDLCEDEDVSIRRQAIKELPQFATGENLPRVADILTQLLQTDDSAEFNLVNNALLSIFKMDAKGTLGGLFSQILQGEDIVRERAIKFLSTKLKTLPDEVLTKEVEELILTESKKVLEDVTGEAFVLFMKILSGLKSLQTVSGRQQLVELVAEQADLEQTFSPSDPDCVDRLLQCTRQAVPLFSKNVHSTRFVTYFCEQVLPNLSTLTTPVEGLDIQLEVLKLLAEMSSFCGDMEKLETNLRKLFDKLLEYMPLPPEEAENGENAGNEEPKLQFSYVECLLYSFHQLGRKLPDFLTAKLNAEKLKDFKIRLQYFARGLQVYIRQLRLALQGKTGEALKTEENKIKVVALKITNNINVLIKDLFHIPPSYKSTVTLSWKPVQKVEIGQKRTSEDTSSGSPPKKSPGGPKRDARQIYNPPSGKYSSNLGNFNYERSLQGK

>583

MPTVEELYRNYGILADATEQVGQHKDAYQVILDGVKGGTKEKRLAAQFIPKFFKHFPELADSAINAQLDLCEDEDVSIRRQAIKELPQFATGENLPRVADILTQLLQTDDSAEFNLVNNALLSIFKMDAKGTLGGLFSQILQGEDIVRERAIKFLSTKLKTLPDEVLTKEVEELILTESKKVLEDVTGEAFVLFMKILSGLKSLQTVSGRQQLVELVAEQADLEQTFSPSDPDCVDRLLQCTRQAVPLFSKNVHSTRFVTYFCEQVLPNLSTLTTPVEGLDIQLEVLKLLAEMSSFCGDMEKLETNLRKLFDKLLEYMPLPPEEAENGENAGNEEPKLQFSYVECLLYSFHQLGRKLPDFLTAKLNAEKLKDFKIRLQYFARGLQVYIRQLRLALQGKTGEALKTEENKIKVVALKITNNINVLIKDLFHIPPSYKSTVTLSWKPVQKVEIGQKRTSEDTSSGSPPKKSPGGPKRDARQIYNPPSGKYSSNLGNFNYEQRGAFRGSRGGRGWGTRGSRSRGRLY

>584

MPTVEELYRNYGILADATEQVGQHKDAYQVILDGVKGGTKEKRLAAQFIPKFFKHFPELADSAINAQLDLCEDEDVSIRRQAIKELPQFATGENLPRVADILTQLLQTDDSAEFNLVNNALLSIFKMDAKGTLGGLFSQILQGEDIVRERAIKFLSTKLKTLPDEVLTKEVEELILTESKKVLEDVTGEAFVLFMKILSGLKSLQTVSGRQQLVELVAEQADLEQTFSPSDPDCVDRLLQCTRQAVPLFSKNVHSTRFVTYFCEQVLPNLSTLTTPVEGLDIQLEVLKLLAEMSSFCGDMEKLETNLRKLFDKLLEYMPLPPEEAENGENAGNEEPKLQFSYVECLLYSFHQLGRKLPDFLTAKLNAEKLKDFKIRLQYFARGLQVYIRQLRLALQGKTGEALKTEENKIKVVALKITNNINVLIKDLFHIPPSYKSTVTLSWKPVQKVEIGQKRTSEDTSSGSPPKKSPGGPKRDARQIYNPPSGKYSSNLGNFNYGERFRLGTSSSRDWVESEQRGAFRGSRGGRGWGTRGSRSRGRLY

>585

MPTVEELYRNYGILADATEQVGQHKDAYQVILDGVKGGTKEKRLAAQFIPKFFKHFPELADSAINAQLDLCEDEDVSIRRQAIKELPQFATGENLPRVADILTQLLQTDDSAEFNLVNNALLSIFKMDAKGTLGGLFSQILQGEDIVRERAIKFLSTKLKTLPDEVLTKEVEELILTESKKVLEDVTGEEFVLFMKILSGLKSLQTVSGRQQLVELVAEQADLEQTFNPSDPDCVDRLLQCTRQAVPLFSKNVHSTRFVTYFCEQVLPNLSSLTTPVEGLDIQLEVLKLLAEMSSFCGDMEKLETNLRKLFDKLLEYMPLPPEEAENGENAGNEEPKLQFSYVECLLYSFHQLGRKLPDFLTAKLNAEKLKDFKIRLQYFARGLQVYIRQLRLALQGKTGEALKTEENKIKVVALKITNNINVLIKDLFHIPPSYKSTVTLSWKPVQKVEIGQKRANEDTTSGSPPKKPPTGPKRDARQIYNPPSGKYSSNLGNFNYERSLQGK

>586

MPTVEELYRNYGILADATEQVGQHKDAYQVILDGVKGGTKEKRLAAQFIPKFFKHFPELADSAINAQLDLCEDEDVSIRRQAIKELPQFATGENLPRVADILTQLLQTDDSAEFNLVNNALLSIFKMDAKGTLGGLFSQILQGEDIVRERAIKFLSTKLKTLPDEVLTKEVEELILTESKKVLEDVTGEEFVLFMKILSGLKSLQTVSGRQQLVELVAEQADLEQTFNPSDPDCVDRLLQCTRQAVPLFSKNVHSTRFVTYFCEQVLPNLSSLTTPVEGLDIQLEVLKLLAEMSSFCGDMEKLETNLRKLFDKLLEYMPLPPEEAENGENAGNEEPKLQFSYVECLLYSFHQLGRKLPDFLTAKLNAEKLKDFKIRLQYFARGLQVYIRQLRLALQGKTGEALKTEENKIKVVALKITNNINVLIKDLFHIPPSYKSTVTLSWKPVQKVEIGQKRANEDTTSGSPPKKPPTGPKRDARQIYNPPSGKYSSNLGNFNYEQRGAFRGSRGGRGWGARGNRSRGRLY

>587

MPTVEELYRNYGILADATEQVGQHKDAYQVILDGVKGGTKEKRLAAQFIPKFFKHFPELADSAINAQLDLCEDEDVSIRRQAIKELPQFATGENLPRVADILTQLLQTDDSAEFNLVNNALLSIFKMDAKGTLGGLFSQILQGEDIVRERAIKFLSTKLKTLPDEVLTKEVEELILTESKKVLEDVTGEEFVLFMKILSGLKSLQTVSGRQQLVELVAEQADLEQTFNPADPDCVDRLLQCTRQAVPLFSKNVHSTRFVTYFCEHVLPNLSSLTTPVEGLDIQLEVLKLLAEMSSFCGDMEKLETNLKKLFDKLLEYMPLPPEEAENGENAGNEEPKLQFSYVECLLYSFHQLGRKLPDFLTAKLNAEKLKDFKIRLQYFARGLQVYIRQLRLALQGKTGEALKTEENKIKVVALKITNNINVLIKDLFHIPPSYKSTVTLSWKPVQKAEMGQKRTNEDTTSGSPPKKAAAGPKRDARQIYNPPSGKYSSNLGNFNYERGLQGK

>588

MPTVEELYRNYGILADATEQVGQHKDAYQVILDGVKGGTKEKRLAAQFIPKFFKHFPELADSAINAQLDLCEDEDVSIRRQAIKELPQFATGENLPRVADILTQLLQTDDSAEFNLVNNALLSIFKMDAKGTLGGLFSQILQGEDIVRERAIKFLSTKLKTLPDEVLTKEVEELILTESKKVLEDVTGEEFVLFMKILSGLKSLQTVSGRQQLVELVAEQADLEQTFNPADPDCVDRLLQCTRQAVPLFSKNVHSTRFVTYFCEHVLPNLSSLTTPVEGLDIQLEVLKLLAEMSSFCGDMEKLETNLKKLFDKLLEYMPLPPEEAENGENAGNEEPKLQFSYVECLLYSFHQLGRKLPDFLTAKLNAEKLKDFKIRLQYFARGLQVYIRQLRLALQGKTGEALKTEENKIKVVALKITNNINVLIKDLFHIPPSYKSTVTLSWKPVQKAEMGQKRTNEDTTSGSPPKKAAAGPKRDARQIYNPPSGKYSSNLGNFNYEQRGGFRGSRGGRGWGGRGNRSRGRLY

>589

MDNIERLYKCYEILSEAGDKISEHVEEYKEILKAVKGSXKEKRLASQFIGNFFKHFPDLADTAIDAQFDLCEDDDTQIRRQAIKDLPKLCQGNAEXTTRVGDTLAQLLILDDPSELQQVNNSLLSIIKLDTKSSVTGLFQQITTGDETTRERCFKFIATKLLTMGPTVXTKEIEDYIVEEIKKALQDVTADEFHLCMTILGATKLGTTITGHAELVKLATEQAELNNTDTDIIAVDDEVVERFIQCATAAAPYFSKTIKSTAFVAHVCDKLLPIKTWNMIATAVSQDQIQLRLLKVFAEIIANTDKLDNASERINAVYNVLLEYMPLPKLSDEDLGDTPPSFQFSHAECLLYALHTLGKNHPTSLSFVEDAEKLKDFRARLQYLARGTQGYIKKLEEALKGKSAEELKSEENQLKQTALKTTSNINVLIRDLFHSPPIFKHDIVLSWIVPKATKLGKRHAPITFGDKAVANSKEKEQDQEKKARPSNDQKFYSPPSGKYSNKVNQNYGNNNRARQRGGGGGGGGYRNRRFNKY

>590

MPTVEELYRNYGILADATEQVGQHKDAYQVILDGVKGGTKEKRLAAQFIPKFFKHFPELADSAINAQLDLCEDEDVSIRRQAIKELPQFATGENLPRVADILTQLLQTDDSAEFNLVNNALLSIFKMDAKGTLGGLFSQILQGEDIVRERAIKFLSTKLKTLPDEVLTKEVEELILTESKKVLEDVTGEEFVLFMKILSGLKSLQTVSGRQQLVELVAEQADLEQAFSPSDPDCVDRLLQCTRQAVPLFSKNVHSTRFVTYFCEQVLPNLSSLTTPVEGLDIQLEVLKLLAEMSSFCGDMEKLETNLRKLFDKLLEYMPLPPEEAENGENAGNEEPKLQFSYVECLLYSFHQLGRKLPDFLTAKLNAEKLKDFKIRLQYFARGLQVYIRQLRLALQGKTGEALKTDENKIKVVALKITNNINVLIKDLFHIPPSYKSTVTLSWKPVQKVELGQKRTSEDTTSGSPPKKSSAGPKRDARQIYNPPSGKYSSNLSNFNYERSLQGK

>591

MPTVEELYRNYGILADATEQVGQHKDAYQVILDGVKGGTKEKRLAAQFIPKFFKHFPELADSAINAQLDLCEDEDVSIRRQAIKELPQFATGENLPRVADILTQLLQTDDSAEFNLVNNALLSIFKMDAKGTLGGLFSQILQGEDIVRERAIKFLSTKLKTLPDEVLTKEVEELILTESKKVLEDVTGEEFVLFMKILSGLKSLQTVSGRQQLVELVAEQADLEQAFSPSDPDCVDRLLQCTRQAVPLFSKNVHSTRFVTYFCEQVLPNLSSLTTPVEGLDIQLEVLKLLAEMSSFCGDMEKLETNLRKLFDKLLEYMPLPPEEAENGENAGNEEPKLQFSYVECLLYSFHQLGRKLPDFLTAKLNAEKLKDFKIRLQYFARGLQVYIRQLRLALQGKTGEALKTDENKIKVVALKITNNINVLIKDLFHIPPSYKSTVTLSWKPVQKVELGQKRTSEDTTSGSPPKKSSAGPKRDARQIYNPPSGKYSSNLSNFNYEQRGAFRGSRGGRGWGARGNRSRGRLY

>592

MPSVEELYRNYGILADATEQVGQHKDAYQVILDGVKGGTKEKRLAAQFIPKFFKHFPELADSAINAQLDLCEDEDVSIRRQAIKELPQFATGENLPRVADILTQLLQTDDSAEFNLVNNALLSIFKMDAKGTLGGLFSQILQGEDIVRERAIKFLSTKLKTLPDEVLTKEVEELILTESKKVLEDVTGEEFVLFMKILSGLKSLQTVSGRQQLVELVAEQADLEQTFNPSDPDCVDRLLQCTRQAVPLFSKNVHSTRFVTYFCEQVLPNLSTLTTPVEGLDIQLEVLKLLAEMSSFCGDMEKLETNLRKLFDKLLEYMPLPPEEAENGENAGNEEPKLQFSYVECLLYSFHQLGRKLPDFLTAKLNAEKLKDFKIRLQYFARGLQVYIRQLRLALQGKTGEALKTEENKIKVVALKITNNINVLIKDLFHIPPSYKSTVTLSWKPVQKVEMGQKRATEDTTSGSPPKKSPAGPKRDARQIYNPPSGKYSSNLSNFNYERSLQGK

>593

MPSVEELYRNYGILADATEQVGQHKDAYQVILDGVKGGTKEKRLAAQFIPKFFKHFPELADSAINAQLDLCEDEDVSIRRQAIKELPQFATGENLPRVADILTQLLQTDDSAEFNLVNNALLSIFKMDAKGTLGGLFSQILQGEDIVRERAIKFLSTKLKTLPDEVLTKEVEELILTESKKVLEDVTGEEFVLFMKILSGLKSLQTVSGRQQLVELVAEQADLEQTFNPSDPDCVDRLLQCTRQAVPLFSKNVHSTRFVTYFCEQVLPNLSTLTTPVEGLDIQLEVLKLLAEMSSFCGDMEKLETNLRKLFDKLLEYMPLPPEEAENGENAGNEEPKLQFSYVECLLYSFHQLGRKLPDFLTAKLNAEKLKDFKIRLQYFARGLQVYIRQLRLALQGKTGEALKTEENKIKVVALKITNNINVLIKDLFHIPPSYKSTVTLSWKPVQKVEMGQKRATEDTTSGSPPKKSPAGPKRDARQIYNPPSGKYSSNLSNFNYAPEPPYKKPM

>594

MPSVEELYRNYGILADATEQVGQHKDAYQVILDGVKGGTKEKRLAAQFIPKFFKHFPELADSAINAQLDLCEDEDVSIRRQAIKELPQFATGENLPRVADILTQLLQTDDSAEFNLVNNALLSIFKMDAKGTLGGLFSQILQGEDIVRERAIKFLSTKLKTLPDEVLTKEVEELILTESKKVLEDVTGEEFVLFMKILSGLKSLQTVSGRQQLVELVAEQADLEQTFNPSDPDCVDRLLQCTRQAVPLFSKNVHSTRFVTYFCEQVLPNLSTLTTPVEGLDIQLEVLKLLAEMSSFCGDMEKLETNLRKLFDKLLEYMPLPPEEAENGENAGNEEPKLQFSYVECLLYSFHQLGRKLPDFLTAKLNAEKLKDFKIRLQYFARGLQVYIRQLRLALQGKTGEALKTEENKIKVVALKITNNINVLIKDLFHIPPSYKSTVTLSWKPVQKVEMGQKRATEDTTSGSPPKKSPAGPKRDARQIYNPPSGKYSSNLSNFNYEQRGAFRGSRGGRGWGARGNRSRGRLY

>595

MPTVEELYRNYGILADATEQVSQHKDAYQVILDGVKGGTKEKRLAAQFIPKFFKHFPELADSAINAQLDLCEDEDVSIRRQAIKELPQFATGENLPRVADILTQLLQTDDSAEFNLVNNALLSIFKMDAKGTLGGLFSQILQGEDIVRERAIKFLSTKLKTLPDEVLTKEVEELILTESKKVLEDVTGEEFVLFMKILSGLKSLQTVSGRQQLVELVAEQADLEQTFNPSDPDCVDRLLQCTRQAVPLFSKNVHSTRFVTYFCEQVLPNLSTLTTPVEGLDIQLEVLKLLAEMSSFCGDMEKLETNLRKLFDKLLEYMPLPPEEAENGENAGNEEPKLQFSYVECLLYSFHQLGRKLPDFLTAKLNAEKLKDFKIRLQYFARGLQVYIRQLRLALQGKTGEALKTEENKIKVVALKITNNINVLIKDLFHIPPSYKSTVTLSWKPVQKVEMGQKRTSEDTTSSSPPKKSPAGPKRDARQIYNPPSGKYSSNLGNFNYERSLQGK

>596

MAVTIEDLYRSYGVLADAKDNLSQHKDAYQVILDGVKGGPKEKRLAAQFIPKFFSSFPELADAAINAQLDLCEDEDVSIRRQAIKELPRFATGENILRVADILTQLLQTDDTAEFNQVNVALISIFKVDAKGTLGGLFSQILQGEDIVRERAIKFLSAKLKTLPEEVMTKEVEDYVFAETKKVLEDVTGEEFVLLMRVVSGLRVLQTVHGRQQLVELVVEQAFLEQALNPTDPDTVDRLLQCTRQALPLFSKNVHSTRFVTYFCEHVLPNLSTLTSPVAELDIQLEVLKLLAEMSPFCGDMEKLEANLNMLFTKLLEFMPMPPEEVENGENSTSEEPKLQFSYVECLLFGFHQLGKKLPDFLLDKVDAERLKDFKIRLQYFARGLQVYIRQLRVALQGKTGDALKTEENKIKVVALKITNNINVLIKDLFHNPPSYKSTVTLSWKPVQKTEAVAPKRPSVEEMGSGGSTKKQISPLPRRDARQIYNPPSERGGFRGGRGRGFGTRGNRSRGRIY

>597

MAVTIEDLYRSYGVLADAKDNLSQHKDAYQVILDGVKGGPKEKRLAAQFIPKFFSSFPELADAAINAQLDLCEDEDVSIRRQAIKELPRFATGENILRVADILTQLLQTDDTAEFNQVNVALISIFKVDAKGTLGGLFSQILQGEDIVRERAIKFLSAKLKTLPEEVMTKEVEDYVFAETKKVLEDVTGEEFVLLMRVVSGLRVLQTVHGRQQLVELVVEQAFLEQALNPTDPDTVDRLLQCTRQALPLFSKNVHSTRFVTYFCEHVLPNLSTLTSPVAELDIQLEVLKLLAEMSPFCGDMEKLEANLNMLFTKLLEFMPMPPEEVENGENSTSEEPKLQFSYVECLLFGFHQLGKKLPDFLLDKVDAERLKDFKIRLQYFARGLQVYIRQLRVALQGKTGDALKTEENKIKVVALKITNNINVLIKDLFHNPPSYKSTVTLSWKPVQKTEAVAPKRPSVEEMGSGGSTKKQISPLPRRDARQIYNPPSGKYSASIGNFSYERGGFRGGRGRGFGTRGNRSRGRIY

>598

MDSSGDSIEKLYKNYEILTDAKDKIGEHELEYREILDAVKGTAKEKRLASQFIGKFFKHFPNLAELAIDRQLDLCEDEDAQIRKQAIKDLPQLCKDTKEHTPKIADILAQLLITEDVTELQQVHQSLLTLAKFDATGTLTGIFSQIVSGDEPTRYRNFQFILNKLIKIGPEVITKEVEDFVIAEIKKILLDVSADEFHLCMSILNQTKLSKTVTGHAELVAIAVEQADMEADLGTLASDDETVERFIQCASEAMPYFSSQVESTQFIKFMCEKLLPLNVWNLIGAGEEQHTTQLRLLKVFAEMCAFCGSLDKPTEKVEAIYNVLLEYMPLPPADADMNETPSFQFSHAECLLHALHTLGKQAAEFLTFPEDAAKLKDFRSRLQYLARGTQGYIKKLQEAVKGKTTEEMKSEENQIKATALKTTSNISTLIRDLFHTPPSFKSVIHLSWLPPKSKAVDTKTTAKRHAAITFEENGKSQDTKGPKHAKGSSGTQKVYTPPSGKYSGKVQNYSPKNGGGSSNGGRRPGGGGGGFRRSGFGGRSGSGGGGGRRRY

>599

MDSSGDSIEKLYKNYEILTDAKDKIGEHELEYREILDAVKGTAKEKRLASQFIGKFFKHFPNLAELAIDRQLDLCEDEDAQIRKQAIKDLPQLCKDTKEHTPKIADILAQLLITEDVTELQQVHQSLLTLAKFDATGTLTGIFSQIVSGDEPTRYRNFQFILNKLMKIGPEVITKEVEDFVIAEIKKILLDVSADEFHLCMSILNQTKLSKTVTGHAELVAIAVEQADMEADLGTLASDDETVERFIQCASEAMPYFSSQVESTQFIKFMCEKLLPLNVWNLIGAGEEQHTTQLRLLKVFAEMCAFCGSLDKPTEKVEAIYNVLLEYMPLPPADADMNETPSFQFSHAECLLHALHTLGKQAAEFLTFPEDAAKLKDFRSRLQYLARGTQGYIKKLQEAVKGKTTEEMKSEENQIKATALKTTSNISTLIRDLFHTPPSFKSVIHLSWLPPKSKAVDTKTTAKRHAAITFEENGKSQDTKGPKHAKGSSGTQKVYTPPSGKYSGKVQNYSPKNGGGSSNGGRRPGGGGGGFRRSGFGGRSGSGGGGGRRRY

>600

MDNIEHLYKCYEILSEAGDKVSEHVKEYEDILKAVKGSSKEKRLASQFIGKFFKYFPDLSNAAIDAQLDLCEDDDMQIRRQAIKDLPKLCQDTVGITSKVGDILAQLLILDDPLELQQVNNSLQTIIKMDAKGSLTGVFTQISTGDEATRERCFKFIATKLFAMGPTVVTKEIEEFLIDEIKKILQDVTADEFQLCMTILGSTKLGTTITGHAELVNLAKEQAELNADIDAITVEDEIVERFIQCATHAMPYFSNTIKSTDFVVYVCDKLLPLSTWNMIATAVAQDQVQLRLLKVFAEMCTYTDVLENAAQRIDNVYQVLREYMPLPKLSEEEDASSPPPSFQFSHAECLLYALHTLGKKHPDSLTFVTDAEKLKDFRSRLQYLARGTQGYIKKLEEAVKGKTAEELKTEENQLKVTALKTTSNISILIRDLFHSPPSFKHEIQLSWVKRKNNKIGSKRHAPITFDGKTENGKDEEKKTKNASDQKIYSPPSGKYSAKVQNYGGGGNNNRQRSRNSGGGGGGFNRNRRNFNKKY

>601

MPTVEELYRNYGILADATEQVGQHKDAYQVILDGVKGGMKEKRLAAQFIPKFFKHFPELADSAINAQLDLCEDEDVSIRRQAIKELPQFATGENLPRVADILTQLLQTDDSAEFNLVNNALLSIFKMDAKGTLGGLFSQILQGEDIVRERAIKFLSTKLKTLPDEVLTKEVEELILTESKKVLEDVTGEEFVLFMKILSGLKSLQTVSGRQQLVELVAEQADLEQTFNPSDPDCVDRLLQCTRQAVPLFSKNVHSTRFVTYFCEHVLPNLSSLTTPVEGLDIQLEVLKLLAEMSSFCGDMEKLETNLKKLFDKLLEYMPLPPEEAENGENAGNEEPKLQFSYVECLLYSFHQLGRKLPDFLTAKLNAEKLKDFKIRLQYFARGLQVYIRQLRLALQGKTGEALKTEENKIKVVALKITNNINVLIKDLFHIPPSYKSTVTLSWKPVQKVEIGQKRANEDTTSGSPPKKSPAGPKRDARQIYNPPSGKYSSNLGNFNYERSLQGK

>602

MPTVEELYRNYGILADATEQVGQHKDAYQVILDGVKGGMKEKRLAAQFIPKFFKHFPELADSAINAQLDLCEDEDVSIRRQAIKELPQFATGENLPRVADILTQLLQTDDSAEFNLVNNALLSIFKMDAKGTLGGLFSQILQGEDIVRERAIKFLSTKLKTLPDEVLTKEVEELILTESKKVLEDVTGEEFVLFMKILSGLKSLQTVSGRQQLVELVAEQADLEQTFNPSDPDCVDRLLQCTRQAVPLFSKNVHSTRFVTYFCEHVLPNLSSLTTPVEGLDIQLEVLKLLAEMSSFCGDMEKLETNLKKLFDKLLEYMPLPPEEAENGENAGNEEPKLQFSYVECLLYSFHQLGRKLPDFLTAKLNAEKLKDFKIRLQYFARGLQVYIRQLRLALQGKTGEALKTEENKIKVVALKITNNINVLIKDLFHIPPSYKSTVTLSWKPVQKVEIGQKRANEDTTSGSPPKKSPAGPKRDARQIYNPPSGKYSSNLGNFNYEQRGAFRGSRGGRGWGARGSRSRGRLY

>603

MKLKVLKRSKHKDAYQVILDGVKGGTKEKRLAAQFIPKFFKHFPELADSAINAQLDLCEDEDVSIRRQAIKELPQFATGENLPRVADILTQLLQTDDSAEFNLVNNALLSIFKMDAKGTLGGLFSQILQGEDIVRERAIKFLSTKLKTLPDEVLTKEVEELILTESKKVLEDVTGEEFVLFMKILSGLKSLQTVSGRQQLVELVAEQADLEQTFNPSDPDCVDRLLQCTRQAVPLFSKNVHSTRFVTYFCEQVLPNLSTLTTPVEGLDIQLEVLKLLAEMSSFCGDMEKLETNLRKLFDKLLEYMPLPPEEAENGENAGNEEPKLQFSYVECLLYSFHQLGRKLPDFLTAKLNAEKLKDFKIRLQYFARGLQVYIRQLRLALQGKTGEALKTEENKIKVVALKITNNINVLIKDLFHIPPSYKSTVTLSWKPVQKVEIGQKRASEDATSGSPPKKSPAGPKRDARQIYNPPSGKYSSNLGNFNYERSLQGK

>604

MKLKVLKRSKHKDAYQVILDGVKGGTKEKRLAAQFIPKFFKHFPELADSAINAQLDLCEDEDVSIRRQAIKELPQFATGENLPRVADILTQLLQTDDSAEFNLVNNALLSIFKMDAKGTLGGLFSQILQGEDIVRERAIKFLSTKLKTLPDEVLTKEVEELILTESKKVLEDVTGEEFVLFMKILSGLKSLQTVSGRQQLVELVAEQADLEQTFNPSDPDCVDRLLQCTRQAVPLFSKNVHSTRFVTYFCEQVLPNLSTLTTPVEGLDIQLEVLKLLAEMSSFCGDMEKLETNLRKLFDKLLEYMPLPPEEAENGENAGNEEPKLQFSYVECLLYSFHQLGRKLPDFLTAKLNAEKLKDFKIRLQYFARGLQVYIRQLRLALQGKTGEALKTEENKIKVVALKITNNINVLIKDLFHIPPSYKSTVTLSWKPVQKVEIGQKRASEDATSGSPPKKSPAGPKRDARQIYNPPSGKYSSNLGNFNYEQRGAFRGSRGGRGWGTRGNRSRGRLY

>605

MKLKVLKRSKHKDAYQVILDGVKGGTKEKRLAAQFIPKFFKHFPELADSAINAQLDLCEDEDVSIRRQAIKELPQFATGENLPRVADILTQLLQTDDSAEFNLVNNALLSIFKMDAKGTLGGLFSQILQGEDIVRERAIKFLSTKLKTLPDEVLTKEVEELILTESKKVLEDVTGEEFVLFMKILSGLKSLQTVSGRQQLVELVAEQADLEQTFNPSDPDCVDRLLQCTRQAVPLFSKNVHSTRFVTYFCEQVLPNLSTLTTPVEGLDIQLEVLKLLAEMSSFCGDMEKLETNLRKLFDKLLEYMPLPPEEAENGENAGNEEPKLQFSYVECLLYSFHQLGRKLPDFLTAKLNAEKLKDFKIRLQYFARGLQVYIRQLRLALQGKTGEALKTEENKIKVVALKITNNINVLIKDLFHIPPSYKSTVTLSWKPVQKVEIGQKRASEDATSGSPPKKSPAGPKRDARQIYNPPSGKYSSNLGNFNYGPCSCEAGGRTTELNPWPYNSILKIKMIQDE

>606

MAVTIEDLYRNYGILADAKDNLSQHKDAYQVILDGVKGGPKEKRLAAQFIPKFFSSFPELADAAINAQLDLCEDEDVSIRRQAIKELPRFATGENILRVADILTQLLQTDDTAEFNQVNAALISIFKIDAKGTLGGLFSQILQGEDIVRERAIKFLSAKLKTLPEDVMTKEVEEYVFAETKKVLEDVTGEEFVLLMRMVSGLRVLQTVHGRQQLVELVVEQAFLEQALNPADPDTVDRLLQCTRQALPLFSKNVHSTRFVTYFCDHVLPNLSTLTSPVAELDIQLEVLKLLAEMSPFCGDMEKLEANLNMLFTKLLEFMPLPPEEVENGENSASEEPKLQFSYVECLLYGFHQLGKKLPDFLLDKVDAERLKDFKIRLQYFARGLQVYIRQLRVALQGKTGDALKTEENKIKVVALKITNNINVLIKDLFHNPPSYKSTVTLSWKPVQKTEAVAPKRPSGEEMGSGGSTKKQISPLPRRDARQIYNPPSGKYSASIGNFNYERGGFRGGRGRGFGARGNRSRGRIY

>607

MQQKFLPLLLIALLVYALTGVQGIDVPRPRGVSLVKAPLYQPQPDGKWSCIDGSRTIPFTQINDDYCDCADGSDEPGTAACPNGQFHCLNTGHQSVDIPSSQVQDGICDCCDGSDELEESQCENTCVALGAAAAIQRRNEAELHMKGAEKRQEMINRGKQLKADRSARRSELNARIKEQEALKTEKEQLKATAEALEHEAVEAFKEQQRELDADTAQAEQEPQQMRQEASLTFHVAEYKEILQAVKGTSKEKRLASQFIGNFFKHFPELSDTAIDAQFDLCEDDDTQIRRQAIKDLPKLCQGNAEATVRVGDTLAQLLILDDATELQQVNNSLLSIIKLDTKNAIAGLFQQITTGDETTRERCFKFIATKLLTMGPNVITKEIEDYIVEEVKKALQDVTADEFHLCMTILGATKLGSTITGHAELVRLATEQAELNTVDADTLVVDDEVVERFIQCATAAAPYFSKTIKSTQFVAYVCDKLLPINTWNLIATAVAQDQIQLRLLKVFAEMITYTDKLDNANERINAVYNVLLEYMPLPKLSDEDTTDVPPSFQFSHAECLLYALHTLGKKHPGNLTFIEDAEKLKDFRARLQYLARGTQGYIKKLEEALKGKSAEELKTEENQLKQTALKTTSNINVLIRDLFHSPPIFKHDIVLSWVVPKNTKLGKRHVPITFGDKGAANGNQEQEQQDKKARPSNEQKFYSPPSGKYSGKVNSNYGNNNRTRQRGGGGGGGGGGNFRNRRYNRY

>608

MDNIERLYKCYEILSEAGDKITEHVAEYKEILQAVKGTSKEKRLASQFIGNFFKHFPELSDTAIDAQFDLCEDDDTQIRRQAIKDLPKLCQGNPEATIRVGDTLAQLLILDDATELQQVNNSLLSIIKLDTKNAITGLFQQITTGDETTRERCFKFIATKLLTMGPNVVTKEIEDYIVEEVKKALQDVTADEFHLCMTILGATKLGSTITGHAELVRLATEQAELNTVDADTLAVDDEVVERFIQCATAAAPYFSKTIKSTQFVAYVCDKLLPIKTWNLIATAVAQDQIQLRLLKVFAEMITYTDKLDNANERINAVYNVLLEYMPLPKLSDEDTTDVPPSFQFSHAECLLYALHTLGKKHPSNLTFIEDAEKLKDFRARLQYLARGTQGYIKKLEEALKGKSAEELKTEENQLKQTALKTTSNINVLIRDLFHSPPIFKHDIVLSWVVPKTNKLGKRHVPITFGDKGTANGNQEQEQQDKKARPSNEQKFYSPPSGKYSGKVNSNYGNNNRTRQRGGGGGGGGGNFRNRRYNRY

>609

MDNIERLYKCYEILSEAGDKISEHVAEYKEILQAVKGTSKEKRLASQFIGTFFKHFPELADTAIDAQFDLCEDDDTQIRRQAIKDLPKLCQGNTETTIRVGDTLAQLLILDDATELQQVNNSLLSIIKLDTKSAITGLFQQITTGDEPTRERCFKFISTKLQTMGPSVVTKEIEEYIVEEIKKALQDVTADEFHLCMTILGATKLGNTITGHAELVKLATEQAELNTADTDAIAVDDEIVERFIQCASAAAPYFSKTIKSTQFVSYVCDKLLPINTWNLIATAVSQDQIQLRLLKVFAEMITNTDKLENANERINNVYNVLLEYMPLPKLDDEDVADAAPSFHFSHAECLLYALHTLGKKHTGNLSFIEDAEKLKDFRARLQYLARGTQGYIKKLEEALKGKSGEELKTEENQLKQTALKTTSNINILIRDLFHSPPIFKHDIVLSWTVPKNKLGKRHAPITFGDKVAAANGKEESEQQDKKSRPSNEQKFYSPPSGKYSGKVNPNYGNNQRNRQRGGGGGGNFRNRRYNRY

>610

MHKDAYQVILDGVKGGAKEKRLAAQFIPKFFKHFPELADSAINAQLDLCEDEDVSIRRQAIKELPQFATGDNLPRVADILTQLLQSDDSAEFNLVNNALLSIFKMDAKGTLGGLFSQILQGEDIVRERAIKFLSTKLKTLPEEVMTKEVEEFILTESKKVLEDVTGEEFNLFMKILSGLKSLQTVSGRQQLVELVAEQADLEQTFNPSDPDCVDRLQQCTRQAVPLFSKNVHSTKFVTYFCEHVLPNLSSLTTSVEGLDIQLEVLKLLAEMSSFCGDMEKLESNLKKLFDKLLEYMPLPPEEAENGENAGNEEPKLQFSHVECLLYSFHQLGRKLPDFLTAKLNAEKLKDFKIRLQYFARGLQVYIRQLRLALQGKTGEALKTEENKIKVVALKITNNINVLIKDLFHIPPSYKSTITLSWKPVQKADASQKRASEDTTSSSPPKKVSAGPKRDARQIYNPPSGKYSSNLGSFSYEQRGGFRGGRGRGWGGRGNRSRGRIY

>611

MPTVEELYRNYGILADATETAGQHKDAYQVILDGVKGGAKEKRLAAQFIPKFFKHFPELADSAINAQLDLCEDEDVSIRRQAIKELPQFATGDNLPRVADILTQLLQSDDSAEFNLVNNALLSIFKMDAKGTLGGLFSQILQGEDIVRERAIKFLSTKLKTLPEEVMTKEVEEFILTESKKVLEDVTGEEFNLFMKILSGLKSLQTVSGRQQLVELVAEQADLEQTFNPSDPDCVDRLQQCTRQAVPLFSKNVHSTKFVTYFCEHVLPNLSSLTTSVEGLDIQLEVLKLLAEMSSFCGDMEKLESNLKKLFDKLLEYMPLPPEEAENGENAGNEEPKLQFSHVECLLYSFHQLGRKLPDFLTAKLNAEKLKDFKIRLQYFARGLQVYIRQLRLALQGKTGEALKTEENKIKVVALKITNNINVLIKDLFHIPPSYKSTITLSWKPVQKADASQKRASEDTTSSSPPKKVSAGPKRDARQIYNPPSGKYSSNLGSFSYEQRGGFRGGRGRGWGGRGNRSRGRIY

>612

MPTVEELYRNYGILADATEQVGQHKDAYQVILDGVKGGTKEKRLAAQFIPKFFKHFPELADSAINAQLDLCEDEDVSIRRQAIKELPQFATGENLPRVADILTQLLQTDDSAEFNLVNNALLSIFKMDAKGTLGGLFSQILQGEDIVRERAIKFLSTKLKTLPDEVLTKEVEELILTESKKVLEDVTGEEFVLFMKILSGLKSLQTVSGRQQLVELVAEQADLEQTFNPSDPDCVDRLLQCTRQAVPLFSKNVHSTRFVTYFCEQVLPNLSTLTTPVEGLDIQLEVLKLLAEMSSFCGDMEKLETNLRKLFDKLLEYMPLPPEEAENGENAGNEEPKLQFSYVECLLYSFHQLGRKLPDFLTAKLNAEKLKDFKIRLQYFARGLQVYIRQLRLALQGKTGEALKTEENKIKVVALKITNNINVLIKDLFHIPPSYKSTVTLSWKPVQKVEIGQKRANEDTSSGSPPKKSPGGPKRDARQIYNPPSGKYSSNLGNFNYERSLQGK

>613

MPTVEELYRNYGILADATEQVGQHKDAYQVILDGVKGGTKEKRLAAQFIPKFFKHFPELADSAINAQLDLCEDEDVSIRRQAIKELPQFATGENLPRVADILTQLLQTDDSAEFNLVNNALLSIFKMDAKGTLGGLFSQILQGEDIVRERAIKFLSTKLKTLPDEVLTKEVEELILTESKKVLEDVTGEEFVLFMKILSGLKSLQTVSGRQQLVELVAEQADLEQTFNPSDPDCVDRLLQCTRQAVPLFSKNVHSTRFVTYFCEQVLPNLSTLTTPVEGLDIQLEVLKLLAEMSSFCGDMEKLETNLRKLFDKLLEYMPLPPEEAENGENAGNEEPKLQFSYVECLLYSFHQLGRKLPDFLTAKLNAEKLKDFKIRLQYFARGLQVYIRQLRLALQGKTGEALKTEENKIKVVALKITNNINVLIKDLFHIPPSYKSTVTLSWKPVQKVEIGQKRANEDTSSGSPPKKSPGGPKRDARQIYNPPSGKYSSNLGNFNYEQRGAFRGSRGGRGWGTRGNRSRGRLY

>614

MPTVEELYRNYGILADATEQVGQHKDAYQVILDGVKGGTKEKRLAAQFIPKFFKHFPELADSAINAQLDLCEDEDVSIRRQAIKELPQFATGENLPRVADILTQLLQTDDSAEFNLVNNALLSIFKMDAKGTLGGLFSQILQGEDIVRERAIKFLSTKLKALPDEVLTKEVEELILTESKKVLEDVTGEEFVLFMKILSGLKSLQTVSGRQQLVELVAEQADLEQTFNPSDPDCVDRLLQCTRQAVPLFSKNVHSTRFVTYFCEQVLPNLSSLTTPVEGLDIQLEVLKLLAEMSSFCGDMEKLETNLRKLFDKLLEYMPLPPEEAENGENAGNEEPKLQFSYVECLLYSFHQLGRKLPDFLTAKLNAEKLKDFKIRLQYFARGLQVYIRQLRLALQGKTGDALKTEENKIKVVALKITNNINVLIKDLFHIPPSYKSTVTLSWKPVQKVEIGQKRASEDTSSSSPPKKSPAGPKRDARQIYNPPSGKYSSNLGNFNYERSLQGK

>615

MPTVEELYRNYGILADATEQVGQHKDAYQVILDGVKGGTKEKRLAAQFIPKFFKHFPELADSAINAQLDLCEDEDVSIRRQAIKELPQFATGENLPRVADILTQLLQTDDSAEFNLVNNALLSIFKMDAKGTLGGLFSQILQGEDIVRERAIKFLSTKLKALPDEVLTKEVEELILTESKKVLEDVTGEEFVLFMKILSGLKSLQTVSGRQQLVELVAEQADLEQTFNPSDPDCVDRLLQCTRQAVPLFSKNVHSTRFVTYFCEQVLPNLSSLTTPVEGLDIQLEVLKLLAEMSSFCGDMEKLETNLRKLFDKLLEYMPLPPEEAENGENAGNEEPKLQFSYVECLLYSFHQLGRKLPDFLTAKLNAEKLKDFKIRLQYFARGLQVYIRQLRLALQGKTGDALKTEENKIKVVALKITNNINVLIKDLFHIPPSYKSTVTLSWKPVQKVEIGQKRASEDTSSSSPPKKSPAGPKRDARQIYNPPSGKYSSNLGNFNYEQRGAFRGSRGGRGWGTRGNRSRGRLY

>616

MPTVEELYRNYGILADATEQVGQHKDAYQVILDGVKGGTKEKRLAAQFIPKFFKHFPELADSAINAQLDLCEDEDVSIRRQAIKELPQFATGENLPRVADILTQLLQTDDSAEFNLVNNALLSIFKMDAKGTLGGLFSQILQGEDIVRERAIKFLSTKLKTLPDEVLTKEVEELILTESKKVLEDVTGEEFVLFMKILSGLKSLQTVSGRQQLVELVAEQADLEQTFNPSDPDCVDRLLQCTRQAVPLFSKNVHSTRFVTYFCEQVLPNLSTLTTPVEGLDIQLEVLKLLAEMSSFCGDMEKLETNLRKLFDKLLEYMPLPPEEAENGENAGNEEPKLQFSYVECLLYSFHQLGRKLPDFLTAKLNAEKLKDFKIRLQYFARGLQVYIRQLRLALQGKTGDALKTEENKIKVVALKITNNINVLIKDLFHIPPSYKSTVTLSWKPVQKVEIGQKRASEDTTSGSPPKKSPAGPKRDARQIYNPPSGKYSSNLSNFNYEQRGAFRGSRGGRGWGARGNRSRGRLY

>617

MPTVEELYRNYGILADATEQVGQHKDAYQVILDGVKGGTKEKRLAAQFIPKFFKHFPELADSAINAQLDLCEDEDVSIRRQAIKELPQFATGENLPRVADILTQLLQTDDSAEFNLVNNALLSIFKMDAKGTLGGLFSQILQGEDIVRERAIKFLSTKLKTLPDEVLTKEVEELILTESKKVLEDVTGEEFVLFMKILSGLKSLQTVSGRQQLVELVAEQADLEQTFNPSDPDCVDRLLQCTRQAVPLFSKNVHSTRFVTYFCEQVLPNLSTLTTPVEGLDIQLEVLKLLAEMSSFCGDMEKLETNLRKLFDKLLEYMPLPPEEAENGENAGNEEPKLQFSYVECLLYSFHQLGRKLPDFLTAKLNAEKLKDFKIRLQYFARGLQVYIRQLRLALQGKTGDALKTEENKIKVVALKITNNINVLIKDLFHIPPSYKSTVTLSWKPVQKVEIGQKRASEDTTSGSPPKKSPAGPKRDARQIYNPPSGKYSSNLSNFNYERSLQGK

>618

MAVTIEELYRNYGILADAKENLSQHKDAYQAILDGVRGGPKEKRLAAQFIPKFFSSFPELADAAINAQLDLCEDEDVSIRRQAIKELPRFATGENIFRVADILTQLLQTDDTAEFNQVNVALISIFKMDAKGTLGGLFSQILQGEDIVRERAIKFLANKLKTLPEDVMTKEVEEYVFTETKKVLEDVTGEEFVLLMRVVSALRVLQSVNGRQQLVELVVEQADLEQALNPADPDAVDRLLQCTRQALPLFSKNVHSTRFVTYFCEHVLPNLSTLTSPVAELDIQLEVLKLLAEMSPFCGDMEKLEANLSTLFTKLVEFMPLPPDEVENGENSASEEPKLQFSYVECLLFSFHQLGKKLPDFLVDKVDAERLKDFKIRLQYFARGLQVYIRQLRMALQGKTGDALKTEENKIKVVALKITNNINVLIKDLFHNPPSYKSTVTLSWKPVQKPEAAAPKRPSGEEMGSGTSPKKQITPLPRRDARQIYNPPSEQRGGYRGGRGRGFWARGSRSRGRIY

>619

MAVTIEELYRNYGILADAKENLSQHKDAYQAILDGVRGGPKEKRLAAQFIPKFFSSFPELADAAINAQLDLCEDEDVSIRRQAIKELPRFATGENIFRVADILTQLLQTDDTAEFNQVNVALISIFKMDAKGTLGGLFSQILQGEDIVRERAIKFLANKLKTLPEDVMTKEVEEYVFTETKKVLEDVTGEEFVLLMRVVSALRVLQSVNGRQQLVELVVEQADLEQALNPADPDAVDRLLQCTRQALPLFSKNVHSTRFVTYFCEHVLPNLSTLTSPVAELDIQLEVLKLLAEMSPFCGDMEKLEANLSTLFTKLVEFMPLPPDEVENGENSASEEPKLQFSYVECLLFSFHQLGKKLPDFLVDKVDAERLKDFKIRLQYFARGLQVYIRQLRMALQGKTGDALKTEENKIKVVALKITNNINVLIKDLFHNPPSYKSTVTLSWKPVQKPEAAAPKRPSGEEMGSGTSPKKQITPLPRRDARQIYNPPSGKYSASIGNFTYEQRGGYRGGRGRGFWARGSRSRGRIY

>620

MDNIERLYKCYEILSEAGDKISEHVEEYKEIIKAVKGTSKEKRLASQFIGNFFKHFPDLADTAIDAQFDLCEDDDTQIRRQAIKDLPKLCQGNAEATTRVGDTLAQLLVLDDPSELQQVNNSLLSIIKLDTKSSVTGLFQQITSGDEPTRERCFKFIATKLLTMGPNVITKEIEDYIVDEIKKALQDVTADEFHHCMTILGATKLGTTITGHAELVKLATEQAELNSTDTDIIAVDDEVVERFIQCATAAAPYFSKTIKSTAFVAHVCDKLLPIKTWNMIATAVSQDQIQLRLLKVFAEMVTYTDTLENASERINAVYNVLLEYMPLPKLSEEDLGDTPPSFQFSHAECLLYALHTLGKKHPNSLSFVEDPEKLKDFRARLQYLARGTQGYIKKLEEALKGKSAEELKTEENQLKQTALKTTSNINVLIRDLFHSPPIFKHEIVLSWVVPKPNKLGKRHAPITFGDKAAANGKEKDQEQEKKARPSNDQKFYSPPSGKYSNKVNQNFGNNNRTRQRGGGGGGYKNRRFNKY

>621

MDNIERLYKCYEILSEAGDKISEHVEEYKEIIKAVKGSSKEKRLASQFIGNFFKHFPDLADTAIDAQFDLCEDDDTQIRRQAIKDLPKLCQGNGDATIRVGDTLAQLLILDDATELQQVNNSLLAIIKLDTKSSVTGLFQQITTGDETTRERCLKFIATKLLTMGPTVITKEIEDYIVEEIKKALQDVTADEFHLCMTILGATKLGSTITGHAELVKLATEQAELNNTDTDIIAVDDEVVERFIQCATAAAPYFSKTIKSTAFVAHVCDKLLPIKTWNMIATAVSQDQIQLRLLKVFAEMITNTDKLENASERINAVYHVLLEYMPLPKLSDEDLGDTPPSFQFSHAECLLYALHTLGKNHPNSLSFVEDAEKLKDFRARLQYLARGTQGYIKKLEEALKGKTGEELKTEENQLKQTALKTTSNINVLIRDLFHSPPIFKHDIVLSWIVPKNTKLGKRHAPITFGEKAAANGKEKDQEPEKKARASNDQKFYSPPSGKYSNKVNQNYGNNNRTRQRGGGGGGYRNRRFNKY

>622

MDNIERLYKCYEILSEAGDKISEHVDEYKEILKAVKGSSKEKRLASQFIGNFFKHFPDLADTAIDAQFDLCEDDDTQIRRQAIKDLPKLCQGNADATIRVGDTLAQLLILDDATELQQVNNSLLAVIKLDTKSAVTGLFQQITTGDETTRERCLKFIATKLLTMGPAVITKEIEDYVIEEIKKALQDVTADEFHLCMTILGATKLGSTITGHAELVKLATEQAELNNTDTDIIAVDDEVVERFIQCATAAAPYFSKTIKSTAFVAHVCDKLLPIKTWNMIATAVSQDQIQLRLLKVFAEMITNTDKLDNASERINAVYHVLLEYMPLPKLNDEDLGDTPPSFQFSHAECLLYALHTLGKNHPNSLSFVEDAEKLKDFRARLQYLARGTQGYIKKLEEALKGKTAEELKTEENQLKQTALKTTSNINVLIRDLFHSPPIFKHDIVLSWIVPKNNKLGKRHAPITFGEKAAANGKEKDQEPEKKPRPSNDQKFYSPPSGKYSNKVNQNYGNNNRTRQRGGGGGGGGGGGYRNRRFNKY

>623

MDNIERLYKCYEILSEAGDKISEHVDEYKEILKAVKGSSKEKRLASQFIGNFFKHFPDLAETAIDAQFDLCEDDDNQIRRQAIKDLPKLCQGNPDATTRVADTLAQLLILDDATELQQVNNSLLSIIKMDTKSVVTGIFQQIGTGEEPTRERCFKFISTKLLTMGPTVITKEIEEYIVEEIKKALQDVTADEFHLCMTILGATKLGNTITGHAELVKLAMEQAELKNTDTDIIAVDDEVVERFVQCATAAAPYFSTTIKSTAFVAHVCDKLLPIPTWNMIATAVSQDQIQLRLLKVFAEMITNTDKLENANNRINNVYNVLLEYMPLPKLSEVDLVDVPPSFEFSHAECLLYALHTLGKKHPTNLSFVEDAEKLKDFRARLQYLARGTQGYIKKLEEALKGKSAEELKTEENQLKQTALKTTSNINVLIRDLFHSPPIFKHDIVLSWIVPKANKLGKRHAPITFGEKPEANGKEKDQDVEKKSRPSNDQKFYSPPSGKYSHKVNPNYGNNNRGRQRGGGGGYRNRRYNRY

>624

MDNIERLYKCYEILSEAGDKISEHVEEYKEIIKAVKGTSKEKRLASQFIGNFFKHFPDLADTAIDAQFDLCEDDDTQIRRQAIKDLPKLCQGNADATIRVGDTLAQLLILDDATELQQVNNSLLAIIKLDTKSSVTGLFQQITTGDETTRERCLKFIATKLLTMGPTVITKEIEDFIVEEIKKALQDVTADEFHLCMTILGATKLGSTITGHAELVKLATEQAELNNTDTDIIAVDDEVVERFIQCATAAAPYFSKTIKSTAFVAHVCDKLLPIKTWNMIATAVSQDQIQLRLLKVFAEMITYTDKLENASERINAVYHVLLEYMPLPKLSDEDLGDTPPSFQFSHAECLLYALHTLGKNHPNSLSFVEDAEKLKDFRARLQYLARGTQGYIKKLEEALKGKTGEELKTEENQLKQTALKTTSNINVLIRDLFHSPPIFKHDIVLSWIVPKNTKLGKRHAPITFGEKAAANGKEKDQEPEKKVRASNDQKFYSPPSGKYSNKVNQNYGNNNRTRQRGGGGGGYRNRRFNKY

>625

MDNIERLYKCYEILSEAGDKISEHVEEYKEIIKAVKGSSKEKRLASQFIGNFFKHFPDLADTAIDAQFDLCEDDDTQIRRQAIKDLPKLCQGNADATIRVGDTLAQLLILDDATELQQVNNSLLAIIKLDTKSSVTGLFQQITTGDETTRERCLKFIATKLLTMGPTVVTKEIEDYIVEEIKKALQDVTADEFHLCMTILGATKLGSTITGHAELVKLATEQAELNNTDTDIIAVDDEVVERFIQCATAAAPYFSKTIKSTAFVAHVCDKLLPIKTWNMIATAVSQDQIQLRLLKVFAEMITYTDKLDNASERINAVYHVLLEYMPLPKLSDEDLGDTPPSFQFSHAECLLYALHTLGKNHPNSLSFVEDAEKLKDFRARLQYLARGTQGYIKKLEEALKGKTGEELKTEENQLKQTALKTTSNINVLIRDLFHSPPIFKHDIVLSWIVPKSTKLGKRHAPITFGEKAAANGKEKDQEPEKKARPSNDQKFYSPPSGKYSNKVNQNYGNNNRTRQRGGGGGGVQNRRFNNHRLSAGGTRCAALCRGLILASGGVLRRIVHIITHVKGRLQTLNRNLDALALVRRHCVHLLLQVVNEAPHETLLHCVRHFVSSGFSNPLDLELDKK

>626

MDNIERLYKCYEILSEAGDKISEHVDEYKEILKAVKGSSKEKRLASQFIGNFFKHFPDLADTAIDAQFDLCEDDDTQIRRQAIKDLPKLCQGNADATIRVGDTLAQLLTLDDAMELQQVNNSLLAIIKLDTKSSITGLFQQIATGDETTRERCLKFIATKLLTMGPTVITKEIEDYVVEEIKKALQDVTADEFHLCMTILGATKLGSTITGHAELVKLATEQAELNNTDTDIIAVDDEVVERFIQCATAAAPYFSKTIKSTAFVAHVCDKLLPIKTWNMIATAVSQDQIQLRLLKVFAEMITYTDKLENASERINAVYHVLLEYMPLPKLSDEDLGDTPPSFQFSHAECLLYALHTLGKNHPNSLSFVEDAEKLKDFRARLQYLARGTQGYIKKLEEALKGKTGEELKTEENQLKQTALKTTSNINVLIRDLFHSPPIFKHDIVLSWIVPKNTKLGKRHAPITFGEKAAANGKEKKDQEPEKKARASNEQKFYSPPSGKYSNKVNQNYGKNNRTIQRGGGGGGGGGGGYRNRRFNKY

>627

MDNIERLYKCYEILSEAGDKISEHVDEYKEILKAVKGSSKEKRLASQFIGNFFKHFPDLADTAIDAQFDLCEDDDTQIRRQAIKDLPKLCQGNADATIRVGDTLAQLLILDDATELQQVNNSLLAVIKLDTKSAVTGLFQQITTGDETTRERCLKFIATKLLTMGPAVITKEIEDYIVEEIKKALQDVTADEFHLCMTILGATKLGSTITGHAELVKLATEQAELNSTDTDIISVDDEVVERFIQCASAAAPYFSKTIKSTAFVAHVCDKLLPIKTWNMIATAVSQDQIQLRLLKVFAEMITNTDKLENASERINAVYHVLLEYMPLPKLSDEDLGDTPPSFQFSHAECLLYALHTLGKNHPNSLSFVEDAEKLKDFRARLQYLARGTQGYIKKLEEALKGKTGEELKTEENQLKQTALKTTSNINVLIRDLFHSPPIFKHDIVLSWIVPKNTKLGKRHAPITFGEKAAANGKEKDQEPEKKPRPSNDQKFYSPPSGKYSNKVNQNYGNNNRTRQRGGGGGGGGGNYRNRRFNKY

>628

MDNIERLYKCYEVLSEAGDKISEHVEEYKEILKAVKGSSKEKRLASQFIGNFFKHFPDLADTAIDAQFDLCEDDDTQIRRQAIKDLPKLCQGNAEATTRVGDTLAQLLILDDPSELQQVNNSLLSIIKLDPKSSVTGVFQQITTGDETTRERCFKFIATKLLTMGPTVITKDIEDFIVEEIKKALQDVTADEFHLCMTILGATKLGNTITGHAELVKLATEQAELNNTDTDIIAVDDEVVERFIQCATAAAPYFSKTIKSTAFVAHVCDKLLPIKTWNMIATAVSQDQIQLRLLKVFAEIITNTDKLENASERINAVYNVLLEYMPLPKLSDEDLGDAPPSFQFSHAECLLYALHTLGKNHPTSLSFVEDAEKLKDFRARLQYLARGTQGYIKKLEEALKGKSAEELKTEENQLKQTALKTTSNINVLIRDLFHSPPIFKHDIVLSWIVPKATKLGKRHAPITFGDKAAANGKEKEQDQEKKARPSNEQKFYSPPSGKYSNKVNQNYGNNNRARQRGGGGGGYRNRRFNKY

>629

MDNIERLYKCYEILSEAGDKISEHVEEYKEILKAVKGSSKEKRLASQFIGNFFKHFPDLADTAIDAQFDLCEDDDTQIRRQAIKDLPKLCQGNADATIRVGDTLAQLLILDDATELQQVNNSLLAVIKLDTKSAVTGLFQQITTGDETTRERCLKFIATKLLTMGPTVLTKEIEDYIVEEIKKALQDVTADEFHLCMTILGATKLGSTITGHAELVKLATEQAELNNTDTDIIAVDDEVVERFIQCATAAAPYFSKTIKSTAFVAHVCDKLLPIKTWNMIATAVSQDQIQLRLLKVFAEMITYTDKLDNASERINAVYHVLLEYMPLPKLSDEDLGDTPPSFQFSHAECLLYALHTLGKNHPNSLSFVEDAEKLKDFRARLQYLARGTQGYIKKLEEALKGKTGEELKTEENQLKQTALKTTSNINVLIRDLFHSPPIFKHDIVLSWIVPKNNKLGKRHAPITFGEKAAANGKEKDQEPEKKARPSNDQKFYSPPSGKYSNKVNQNYGNNNRTRQRGGGGGGGGGGGGGYRNRRFNKY

>630

MTDPSDEAKNIEKLYEFGERLNESKDKSQNVADYQGIIDAAKTSIKAKQLAAQLIPRFFKFFPDLSESAIYTHIDLIEEEELGVRVQAIRGLPLFCKDTPEQIAKIVDILVQLLAAEEFVERDAVHKALMSLLRQDVKVSLTALFKHIGSVDEPSTDEFIREKVLTFIREKVFPIKSELLKPQEEMERHITDLIKKSLEDVTGAEFRMFMDFLKSLSIFGEKAPPERMKELIGIIEGQADLDAQFNVSDADHIDRLISCLFMALPFVVRGASSSKFLNYLNKHILPVSDKLPDERRLDLLKALAEVSPYTTPQDSRQILPSVVQLLKKNMPRRKTGEEFNFTYVECLLYTFHHLSHKVPNATNSLCGYKIVTGQPSDRLGEDFSEQYKEFTERLSYVEELTRATMKKLTQGMAEKNKAMAAAKSDEAKDSIVS

>631

MTDPSDEAKNIEKLYEFGERLNESKDKSQNVADYQGIIDAAKTSIKAKQLAAQLIPRFFKFFPDLSESAIYTHIDLIEEEELGVRVQAIRGLPLFCKDTPEQIAKIVDILVQLLAAEEFVERDAVHKALMSLLRQDVKVSLTALFKHIGSVDEPSTDEFIREKVLTFIREKVFPIKSELLKPQEEMERHITDLIKKSLEDVTGAEFRMFMDFLKSLSIFGEKAPPERMKELIGIIEGQADLDAQFNVSDADHIDRLISCLFMALPFVVRGASSSKFLNYLNKHILPVSDKLPDERRLDLLKALAEVSPYTTPQDSRQILPSVVQLLKKNMPRRKTGEEFNFTYVECLLYTFHHLSHKVPNATNSLCGYKIVTGQPSDRLGEDFSEQYKEFTERLSYVEELTRATMKKLTQGMAEKNKAMAAAKSDEAKDSIKTEKQNTTTGLRTCNNILAMTKTLHSKTPSFIGDKSINLSWKEVTKPVVPSSTPATGTKRPANPANGPGNMTSKKGRGGGGLQNQLVNRAFEGLSHGGRSGGGGGRSRGRGWGGRGRGRGYR

>632

MAVTIEELYRNYGILADAKENLSQHKDAYQAILDGVRGGPKEKRLAAQFIPKFFSSFPELADAAINAQLDLCEDEDVSIRRQAIKELPRFATGENIFRVADILTQLLQTDDNAEFNQVNAALMSIFKMDAKGTLGGLFSQILQGDDIVRERAIKFLATKLKTLPEDVMTKEVEEYVFTETKKVLEDVTGEEFVLLMRVVSALRVLQSVNGRQQLVELVVEQADLEQALNPADPDAVDRLLQCTRQALPLFSKNVHSTRFVTYFCEHVLPNLSTLTSPVAELDIQLEVLKLLAEMSPFCGDMEKLEANLNTLFTKLVEFMPLPPDEVENGENSASEEPKLQFSYVECLLFSFHQLGKKLPDFLVDKVDAERLKDFKIRLQYFARGLQVYIRQLRMALQGKTGDALKTEENKIKVVALKITNNINVLIKDLFHNPPSYKSTVTLSWKPVQKPEAAAPKRPSGEEMGSGASTKKQITPLPRRDARQIYNPPSEQRGGYRGGRGRGFWARGSRSRGRIY

>633

MAVTIEELYRNYGILADAKENLSQHKDAYQAILDGVRGGPKEKRLAAQFIPKFFSSFPELADAAINAQLDLCEDEDVSIRRQAIKELPRFATGENIFRVADILTQLLQTDDNAEFNQVNAALMSIFKMDAKGTLGGLFSQILQGDDIVRERAIKFLATKLKTLPEDVMTKEVEEYVFTETKKVLEDVTGEEFVLLMRVVSALRVLQSVNGRQQLVELVVEQADLEQALNPADPDAVDRLLQCTRQALPLFSKNVHSTRFVTYFCEHVLPNLSTLTSPVAELDIQLEVLKLLAEMSPFCGDMEKLEANLNTLFTKLVEFMPLPPDEVENGENSASEEPKLQFSYVECLLFSFHQLGKKLPDFLVDKVDAERLKDFKIRLQYFARGLQVYIRQLRMALQGKTGDALKTEENKIKVVALKITNNINVLIKDLFHNPPSYKSTVTLSWKPVQKPEAAAPKRPSGEEMGSGASTKKQITPLPRRDARQIYNPPSGKYSASIGNFTYEQRGGYRGGRGRGFWARGSRSRGRIY

>634

MPTVAELYRNYGILADATEQVGQHKDAYQVILDGVKGGTKEKRLAAQFIPKFFSHFPELADSAINAQLDLCEDEDVSIRRQAIKELPQFATGENLPRVADILTQLLQTDDSAEFSLVNNALLSIFKMDAKGTLGGLFSQILQGEDIVRERAIKFLSTKLKTLADEVLTKEVEELILTESKKVLEDVTGEEFVLFMKILSGLKSLQTVSGRQQLVELVAEQADLEQTFNPADPDCVDRLLQCTRQAVPLFSKNVHSTRFVTYFCEHVLPNLGSLTTPVEGLDIQLEVLKLLAEMSSFCGDMEKLETNLKKLFDKLLEYMPLPPEEAENGENAGNEEPKLQFSYVECLLYSFHQLGRKLPDFLTAKLNAEKLKDFKIRLQYFARGLQVYIRQLRLALQGKTGEALKTEENKIKVVALKITNNINVLIKDLFHIPPSYKSTVTLSWKPVQKAEMGQKRTNEDTSSGSPPKKAAAGPKRDARQIYNPPSGKYSSNLGNFNYERGLQGK

>635

MPTVAELYRNYGILADATEQVGQHKDAYQVILDGVKGGTKEKRLAAQFIPKFFSHFPELADSAINAQLDLCEDEDVSIRRQAIKELPQFATGENLPRVADILTQLLQTDDSAEFSLVNNALLSIFKMDAKGTLGGLFSQILQGEDIVRERAIKFLSTKLKTLADEVLTKEVEELILTESKKVLEDVTGEEFVLFMKILSGLKSLQTVSGRQQLVELVAEQADLEQTFNPADPDCVDRLLQCTRQAVPLFSKNVHSTRFVTYFCEHVLPNLGSLTTPVEGLDIQLEVLKLLAEMSSFCGDMEKLETNLKKLFDKLLEYMPLPPEEAENGENAGNEEPKLQFSYVECLLYSFHQLGRKLPDFLTAKLNAEKLKDFKIRLQYFARGLQVYIRQLRLALQGKTGEALKTEENKIKVVALKITNNINVLIKDLFHIPPSYKSTVTLSWKPVQKAEMGQKRTNEDTSSGSPPKKAAAGPKRDARQIYNPPSGKYSSNLGNFNYEQRGGFRGSRGGRGWGGRGNRSRGRLY

>636

MAATVEELYRNYGILADAKEDLSKHKDAYQVILDGVKGGPKEKRLAAQFIPKFFSSFPELADAAINAQLDLCEDEDVSIRRQAIKELPRFAAGENLPRVADILTQLLQTDDSAEFSQVSTALISIFKIDAKGTLGGLFSQILQGEDIVRERAIKFLSTKLKTMPDDTMTKEVEDYIFTETKKVLEDVTGEEFVLLVRILSGLKSMQTVSGRQQLVELVVEQAFLEQVLNPTDADSVDRLLQCTRQALPLFSKNVHSTRFVTYFCEFVLPNLSLLTSPVAELDIQLEVLKLLAEMSPYCGDMDKLEVNLNMLFEKLLEFMPLPPEEENGENAANEEPKLQFSYVECLLFSFHQLGKKLPDFLIDKISTEKLKDFKIRLQYFARGLQVYIRQLRVALQGKTGDTLKTDENKIKVVALKITNNINVLIKDLFHNPPSYKSTVTLSWKPVQKTEAASIGQKRQSGEDIGATATTKKLPTNLPRRNARQIYNPPSGKYSASIGNFSYEQGGFRGGRGRGFGGRGNRSRGRIY

>637

MAATVEELYRNYGILADAKEDLSKHKDAYQVILDGVKGGPKEKRLAAQFIPKFFSSFPELADAAINAQLDLCEDEDVSIRRQAIKELPRFAAGENLPRVADILTQLLQTDDSAEFSQVSTALISIFKIDAKGTLGGLFSQILQGEDIVRERAIKFLSTKLKTMPDDTMTKEVEDYIFTETKKVLEDVTGEEFVLLVRILSGLKSMQTVSGRQQLVELVVEQAFLEQVLNPTDADSVDRLLQCTRQALPLFSKNVHSTRFVTYFCEFVLPNLSLLTSPVAELDIQLEVLKLLAEMSPYCGDMDKLEVNLNMLFEKLLEFMPLPPEEENGENAANEEPKLQFSYVECLLFSFHQLGKKLPDFLIDKISTEKLKDFKIRLQYFARGLQVYIRQLRVALQGKTGDTLKTDENKIKVVALKITNNINVLIKDLFHNPPSYKSTVTLSWKPVQKTEAASIGQKRQSGEDIGATATTKKLPTNLPRRNARQIYNPPSGKYSASIGNFSYEQQGGFRGGRGRGFGGRGNRSRGRIY

>638

MPTVEELYRNYGILADATEQVVQHKDAYQVILDGVKGGAKEKRLAAQFIPKFFKHFPELADSAINAQLDLCEDEDVSIRRQAIKELPQFATGDNLPRVADILTQLLQTDDSAEFNLVNNALLSIFKMDAKGTLGGLFSQILQGEDIVRERAIKFLSTKLKTLPEEVLTKEIEELILTESKKVLEDVTGEEFVLFMKILSGLKSLQTVSGRQQLVELVAEQADLEQTFNPADPDCVDRLLQCTRQAVPLFSKNVHSTRFVTYFCEHVLPNLSSLTTPVEGLDIQLEVLKLLAEMSSFCGDMEKLETNLKKLFDKLLEYMPLPPEEAENGENAGNEEPKLQFSYVECLLYSFHQLGRKLPDFLTAKLNAEKIKDFKIRLQYFARGLQVYIRQLRLALQGKTGEALKTEENKIKVVALKITNNINVLIKDLFHIPPSYKSTVTLSWKPVQKAESGQKRASEDTSSGSPPKKSPAGPKRDARQIYNPPSGKYSSNLGNFNYERGLPGK

>639

MPTVEELYRNYGILADATEQVVQHKDAYQVILDGVKGGAKEKRLAAQFIPKFFKHFPELADSAINAQLDLCEDEDVSIRRQAIKELPQFATGDNLPRVADILTQLLQTDDSAEFNLVNNALLSIFKMDAKGTLGGLFSQILQGEDIVRERAIKFLSTKLKTLPEEVLTKEIEELILTESKKVLEDVTGEEFVLFMKILSGLKSLQTVSGRQQLVELVAEQADLEQTFNPADPDCVDRLLQCTRQAVPLFSKNVHSTRFVTYFCEHVLPNLSSLTTPVEGLDIQLEVLKLLAEMSSFCGDMEKLETNLKKLFDKLLEYMPLPPEEAENGENAGNEEPKLQFSYVECLLYSFHQLGRKLPDFLTAKLNAEKIKDFKIRLQYFARGLQVYIRQLRLALQGKTGEALKTEENKIKVVALKITNNINVLIKDLFHIPPSYKSTVTLSWKPVQKAESGQKRASEDTSSGSPPKKSPAGPKRDARQIYNPPSGKYSSNLGNFNYEQRGGFRGSRGGRGWGGRGNRSRGRLY

>640

MPTVEELYRNYGILADATEQVGQHKDAYQVILDGVKGGTKEKRLAAQFIPKFFKHFPELADSAINAQLDLCEDEDVSIRRQAIKELPQFATGENLPRVADILTQLLQTDDSAEFNLVNNALLSIFKMDAKGTLGGLFSQILQGEDIVRERAIKFLSTKLKTLPEEVLTKEVEELILTESKKVLEDVTGEEFVLFMKILSGLKSLQTVSGRQQLVELVAEQADLEQTFNPSDPDCVDRLLQCTRQAVPLFSKNVHSTRFVTYFCEQVLPNLSSLTTPVEGLDIQLEVLKLLAEMSSFCGDMEKLETNLRKLFDKLLEYMPLPPEEAENGENAGNEEPKLQFSYVECLLYSFHQLGRKLPDFLTAKLNAEKLKDFKIRLQYFARGLQVYIRQLRLALQGKTGEALKTDENKIKVVALKITNNINVLIKDLFHIPPSYKSTVTLSWKPVQKVEIGQKRTTEDTTSGSPPKKSPAGPKRDARQIYNPPSGKYSSNLGNFNYERSLQGK

>641

MPTVEELYRNYGILADATEQVGQHKDAYQVILDGVKGGTKEKRLAAQFIPKFFKHFPELADSAINAQLDLCEDEDVSIRRQAIKELPQFATGENLPRVADILTQLLQTDDSAEFNLVNNALLSIFKMDAKGTLGGLFSQILQGEDIVRERAIKFLSTKLKTLPEEVLTKEVEELILTESKKVLEDVTGEEFVLFMKILSGLKSLQTVSGRQQLVELVAEQADLEQTFNPSDPDCVDRLLQCTRQAVPLFSKNVHSTRFVTYFCEQVLPNLSSLTTPVEGLDIQLEVLKLLAEMSSFCGDMEKLETNLRKLFDKLLEYMPLPPEEAENGENAGNEEPKLQFSYVECLLYSFHQLGRKLPDFLTAKLNAEKLKDFKIRLQYFARGLQVYIRQLRLALQGKTGEALKTDENKIKVVALKITNNINVLIKDLFHIPPSYKSTVTLSWKPVQKVEIGQKRTTEDTTSGSPPKKSPAGPKRDARQIYNPPSGKYSSNLGNFNYEQRGAFRGSRGGRGWGARGNRSRGRLY

>642

MPTVEELYRNYGILADATEQVSQHKDAYQVILDGVKGGTKEKRLAAQFIPKFFKHFPELADSAINAQLDLCEDEDVSIRRQAIKELPQFATGENLPRVADILTQLLQTDDSAEFNLVNNALLSIFKMEKLETNLRKLFDKLLEYMPLPPEEAENGENAGNEEPKLQFSYVECLLYSFHQLGRKLPDFLTAKLNAEKLKDFKIRLQYFARGLQVYIRQLRLALQGKTGEALKTEENKIKVVALKITNNINVLIKDLFHIPPSYKSTVTLSWKPVQKVEIGQKRASEDTSVSPPKKSPAGPKRDARQIYNPPSGKYSSNLGNFNYERSLQGK

>643

MPTVEELYRNYGILADATEQVSQIRRQAIKELPQFATGENLPRVADILTQLLQTDDSAEFNLVNNALLSIFKMDAKGTLGGLFSQILQGEDIVRERAIKFLSTKLKTLPEEVLTKEVEELILTESKKVLEDVTGEEFVLFMKILSGLKSLQTVSGRQQLVELVAEQADLEQTFNPSDPDCVDRLLQCTRQAVPLFSKNVHSTKFVTYFCEHVLPNLSSLTTPVEGLDIQLEVLKLLAEMSSFCGDMEKLETNLRKLFDKLLEYMPLPPEEAENGENAGNEEPKLQFSYVECLLYSFHQLGRKLPDFLTAKLNAEKLKDFKIRLQYFARGLQVYIRQLRLALQGKTGEALKTEENKIKVVALKITNNINVLIKDLFHIPPSYKSTVTLSWKPVQKVEIGQKRASEDTSVSPPKKSPAGPKRDARQIYNPPSGKYSSNLGNFNYERSLQGK

>644

MPTVEELYRNYGILADATEQVSQHKDAYQVILDGVKGGTKEKRLAAQFIPKFFKHFPELADSAINAQLDLCEDEDVSIRRQAIKELPQFATGENLPRVADILTQLLQTDDSAEFNLVNNALLSIFKMDAKGTLGGLFSQILQGEDIVRERAIKFLSTKLKTLPEEVLTKEVEELILTESKKVLEDVTGEEFVLFMKILSGLKSLQTVSGRQQLVELVAEQADLEQTFNPSDPDCVDRLLQCTRQAVPLFSKNVHSTKFVTYFCEHVLPNLSSLTTPVEGLDIQLEVLKLLAEMSSFCGDMEKLETNLRKLFDKLLEYMPLPPEEAENGENAGNEEPKLQFSYVECLLYSFHQLGRKLPDFLTAKLNAEKLKDFKIRLQYFARGLQVYIRQLRLALQGKTGEALKTEENKIKVVALKITNNINVLIKDLFHIPPSYKSTVTLSWKPVQKVEIGQKRASEDTSVSPPKKSPAGPKRDARQIYNPPSGKYSSNLGNFNYERSLQGK

>645

MPTVEELYRNYGILADATEQVSQHKDAYQVILDGVKGGTKEKRLAAQFIPKFFKHFPELADSAINAQLDLCEDEDVSIRRQAIKELPQFATGENLPRVADILTQLLQTDDSAEFNLVNNALLSIFKMDAKGTLGGLFSQILQGEDIVRERAIKFLSTKLKTLPEEVLTKEVEELILTESKKVLEDVTGEEFVLFMKILSGLKSLQTVSGRQQLVELVAEQADLEQTFNPSDPDCVDRLLQCTRQAVPLFSKNVHSTKFVTYFCEHVLPNLSSLTTPVEGLDIQLEVLKLLAEMSSFCGDMEKLETNLRKLFDKLLEYMPLPPEEAENGENAGNEEPKLQFSYVECLLYSFHQLGRKLPDFLTAKLNAEKLKDFKIRLQYFARGLQVYIRQLRLALQGKTGEALKTEENKIKVVALKITNNINVLIKDLFHIPPSYKSTVTLSWKPVQKVEIGQKRASEDTSVSPPKKSPAGPKRDARQIYNPPSGKYSSNLGNFNYEQRGAFRGSRGGRGWGARGNRSRGRLY

>646

MPTVEELYRNYGILADATEQVGQHKDAYQVILDGVKGGTKEKRLAAQFIPKFFKHFPELADSAINAQLDLCEDEDVSIRRQAIKELPQFATGENLPRVADILTQLLQTDDSAEFNLVNNALLSIFKMDAKGTLGGLFSQILQGEDIVRERAIKFLSTKLKTLPDEVLTKEVEELILTESKKVLEDVTGEEFVLFMKILSGLKSLQTVSGRQQLVELVAEQADLEQTFSPSDPDCVDRLLQCTRQAVPLFSKNVHSTRFVTYFCEQVLPNLSTLTTPVEGLDIQLEVLKLLAEMSSFCGDMEKLETNLRKLFDKLLEYMPLPPEEAENGENAGNEEPKLQFSYVECLLYSFHQLGRKLPDFLTAKLNAEKLKDFKIRLQYFARGLQVYIRQLRLALQGKTGEALKTEENKIKVVALKITNNINVLIKDLFHIPPSYKSTVTLSWKPVQKVEIGQKRASEDTSSGSPPKKSPGGPKRDARQIYNPPSGKYSSNLGNFNYGERFRLGTSSPRD

>647

MSDNLEKLYEKYNILSDAKDKVSQHSSEYVESFEGIKGTESEKMLAAQILSKFFKHFPSLQDKALNALLDLCEDEESKIRACAMRYLVSICKDVKEHLTKVTDILAQMMQLEEQRDFTTASWCLLQLWKQDSANVLRTMYNHIRSLSSAAARVKCLQFIHLKFIKPIESQPTEIENIVVEESKKLLQDDISSEEIVLIISCLKNSKYAKTAAGQQELLDFISEIMELDRDFDPLEDCIVDRVIICTTHALPFFSAKNESTKFVAYYCDQILPQWDKIATLEQGELFQLHLLRHLAELSIYCGKFENPSLHVVQIFDKIKLYMPTPPENADVYKMPNLEFSFVECLLFAFHRLARQCPDFLTHDPQILKDFRARLVYFSRGVQGCNKVLNSKPITGLDSVNASKAKIAPSLLNNINVLIKDLFYQPPMYKCNVTLSFKQEVVEKELRKPATSGQKRHVPITFDNGSTGTKQTRPARSGDNVKLYTPPSGKFSNNFQYDRGGSRGNRSRGRGSRGRGRNWRN

>648

MSDNLEKLYEKYNILSDAKDKVSQHSSEYVESFEGIKGTESEKMLAAQILSKFFKHFPSLQDKALNALLDLCEDEESKIRACAMRYLVSICKDVKEHLTKVTDILAQMMQLEEQRDFTTASWCLLQLWKQDSANVLRTMYNHIRSLSSAAARVKCLQFIHLKFIKPIESQPTEIENIVVEESKKLLQLQDDISSEEIVLIISCLKNSKYAKTAAGQQELLDFISEIMELDRDFDPLEDCIVDRVIICTTHALPFFSAKNESTKFVAYYCDQILPQWDKIATLEQGELFQLHLLRHLAELSIYCGKFENPSLHVVQIFDKIKLYMPTPPENADVYKMPNLEFSFVECLLFAFHRLARQCPDFLTHDPQILKDFRARLVYFSRGVQGCNKVLNSKPITGLDSVNASKAKIAPSLLNNINVLIKDLFYQPPMYKCNVTLSFKQEVVEKELRKPATSGQKRHVPITFDNGSTGTKQTRPARSGDNVKLYTPPSGKFSNNFQYDRGGSRGNRSRGRGSRGRGRNWRN

>649

MAVTIEDLYRSYGILADAKDNLCQHKDAYQVILNGVKGGAKEKRLAAQFIPKFFSSFPELADAAINAQLDLCEDEDVSIRRQAIKELPRFATGDNILRVADILTQLLQTDDTAEFNQVTAALISIFKMDAKGTLGGLFSQILHGEDIVRERAIKFLSTKLKTLPEDVMSKEVEDYVFAETKKVLEDVTGEEFVLLMRVVSGLHVLQTVNGRQQLVELVVEQADLEQALNPSEPDAVDRLLQCTRQALPLFSKNVHSTRFVTYFCEHVLPNLSTLTSPVAELDIQLEVLKLLAEMSPFCGDMEKLEANLSMMFTKLMEFMPLPPEEAENGETSANEEPKLQFSYVECLIFSFHQLGKKLPDFLVDKVDAERLKDFKIRLQYFARGLQVYIRQLRVALQGKTGDALKTEENKIKVVALKITNNINVLIKDLFHNPPSYKSTVTLSWKPVQKPEGVATKRPSTEDTGSGGSTKKPVVSLPRRDARQIYNPPSEQRGGFRGGRGRGFGTRGSRSRGRIY

>650

MKILSGLKSLQTVSGRQQLVELVAEQADLEQTFNPSDPDCVDRLLQCTRQAVPLFSKNVHSTRFVTYFCEQVLPNLSSLTTPVEGLDIQLEVLKLLAEMSSFCGDMEKLETNLRKLFDKLLEYMPLPPEEAENGENAGNEEPKLQFSYVECLLYSFHQLGRKLPDFLTAKLNAEKLKDFKIRLQYFARGLQVYIRQLRLALQGKTGEALKTDENKIKVVALKITNNINVLIKDLFHIPPSYKSTVTLSWKPVQKVEIGQKRATEDTTSGSPPKKSPAGPKRDARQIYNPPSGKYSGNLGNFNYEQRGAFRGSRGGRGWGARGNRSRGRLY

>651

MKILSGLKSLQTVSGRQQLVELVAEQADLEQTFNPSDPDCVDRLLQCTRQAVPLFSKNVHSTRFVTYFCEQVLPNLSSLTTPVEGLDIQLEVLKLLAEMSSFCGDMEKLETNLRKLFDKLLEYMPLPPEEAENGENAGNEEPKLQFSYVECLLYSFHQLGRKLPDFLTAKLNAEKLKDFKIRLQYFARGLQVYIRQLRLALQGKTGEALKTDENKIKVVALKITNNINVLIKDLFHIPPSYKSTVTLSWKPVQKVEIGQKRATEDTTSGSPPKKSPAGPKRDARQIYNPPSGKYSGNLGNFNYEANSLFCKKGFGSNQSKTSDTCQIMKYFHPQVNMQKQVGTS

>652

MPTVEELYRNYGILADATEQVGQHKDAYQVILDGVKGGTKEKRLAAQFIPKFFKHFPELADSAINAQLDLCEDEDVSIRRQAIKELPQFATGENLPRVADILTQLLQTDDSAEFNLVNNALLSIFKMDAKGTLGGLFSQILQGEDIVRERAIKFLSTKLKTLPDEVLTKEVEELILTESKKVLEDVTGEEFVLFMKILSGLKSLQTVSGRQQLVELVAEQADLEQTFNPSDPDCVDRLLQCTRQAVPLFSKNVHSTRFVTYFCEQVLPNLGSLTTPVEGLDIQLEVLKLLAEMSSFCGDMEKLETNLRKLFDKLLEYMPLPPEEAENGENAGNEEPKLQFSYVECLLYSFHQLGRKLPDFLTAKLNAEKLKDFKIRLQYFARGLQVYIRQLRLALQGKTGEALKTDENKIKVVALKITNNINVLIKDLFHIPPSYKSTVTLSWKPVQKVELGQKRASEDTTSGSPPKKSSAGPKRDARQIYNPPSGKYSSNLGNFNYGERFRLGTSSMRD

>653

MPTVEELYRNYGILADATEQVGQHKDAYQVILDGVKGGTKEKRLAAQFIPKFFKHFPELADSAINAQLDLCEDEDVSIRRQAIKELPQFATGENLPRVADILTQLLQTDDSAEFNLVNNALLSIFKMDAKGTLGGLFSQILQGEDIVRERAIKFLSTKLKTLPDEVLTKEVEELILTESKKVLEDVTGEEFVLFMKILSGLKSLQTVSGRQQLVELVAEQADLEQTFNPSDPDCVDRLLQCTRQAVPLFSKNVHSTRFVTYFCEQVLPNLGSLTTPVEGLDIQLEVLKLLAEMSSFCGDMEKLETNLRKLFDKLLEYMPLPPEEAENGENAGNEEPKLQFSYVECLLYSFHQLGRKLPDFLTAKLNAEKLKDFKIRLQYFARGLQVYIRQLRLALQGKTGEALKTDENKIKVVALKITNNINVLIKDLFHIPPSYKSTVTLSWKPVQKVELGQKRASEDTTSGSPPKKSSAGPKRDARQIYNPPSGKYSSNLGNFNYERSLQGK

>654

MPTVEELYRNYGILADATEQVGQHKDAYQVILDGVKGGTKEKRLAAQFIPKFFKHFPELADSAINAQLDLCEDEDVSIRRQAIKELPQFATGENLPRVADILTQLLQTDDSAEFNLVNNALLSIFKMDAKGTLGGLFSQILQGEDIVRERAIKFLSTKLKTLPDEVLTKEVEELILTESKKVLEDVTGEEFVLFMKILSGLKSLQTVSGRQQLVELVAEQADLEQTFNPSDPDCVDRLLQCTRQAVPLFSKNVHSTRFVTYFCEQVLPNLGSLTTPVEGLDIQLEVLKLLAEMSSFCGDMEKLETNLRKLFDKLLEYMPLPPEEAENGENAGNEEPKLQFSYVECLLYSFHQLGRKLPDFLTAKLNAEKLKDFKIRLQYFARGLQVYIRQLRLALQGKTGEALKTDENKIKVVALKITNNINVLIKDLFHIPPSYKSTVTLSWKPVQKVELGQKRASEDTTSGSPPKKSSAGPKRDARQIYNPPSGKYSSNLGNFNYEQRGAFRGSRGGRGWGARGNRSRGRLY

>655

MAVTIEELYRNYGILADAKENLSQHKDAYQAILDGVRGGPKEKRLAAQFIPKFFSSFPELADAAINAQLDLCEDEDVSIRRQAIKELPRFATGENIFRVADILTQLLQTDDNAEFNQVNVALISIFKMDAKGTLGGLFSQILQGDDIVRERAIKFLATKLKTLPEDVMTKEVEEYVFTETKKVLEDVTGEEFVLLMRVVSALRVLQSVNGRQQLVELVVEQADLEQALNPADPDAVDRLLQCTRQALPLFSKNVHSTRFVTYFCEHVLPNLSTLTCPVAELDIQLEVLKLLAEMSPFCGDMEKLEANLSTLFTKLVEFMPLPPDEVENGENSASEEPKLQFSYVECLLFSFHQLGKKLPDFLVDKVDAERLKDFKIRLQYFARGLQVYIRQLRMALQGKTGDALKTEENKIKVVALKITNNINVLIKDLFHNPPSYKSTVTLSWKPVQKPEAAAPKRPSGEEMGSGASTKKQITPLPRRDARQIYNPPSEQRGGYRGGRGRGFWARGSRSRGRIY

>656

MAVTIEELYRNYGILADAKENLSQHKDAYQAILDGVRGGPKEKRLAAQFIPKFFSSFPELADAAINAQLDLCEDEDVSIRRQAIKELPRFATGENIFRVADILTQLLQTDDNAEFNQVNVALISIFKMDAKGTLGGLFSQILQGDDIVRERAIKFLATKLKTLPEDVMTKEVEEYVFTETKKVLEDVTGEEFVLLMRVVSALRVLQSVNGRQQLVELVVEQADLEQALNPADPDAVDRLLQCTRQALPLFSKNVHSTRFVTYFCEHVLPNLSTLTCPVAELDIQLEVLKLLAEMSPFCGDMEKLEANLSTLFTKLVEFMPLPPDEVENGENSASEEPKLQFSYVECLLFSFHQLGKKLPDFLVDKVDAERLKDFKIRLQYFARGLQVYIRQLRMALQGKTGDALKTEENKIKVVALKITNNINVLIKDLFHNPPSYKSTVTLSWKPVQKPEAAAPKRPSGEEMGSGASTKKQITPLPRRDARQIYNPPSGKYSASIGNFTYEQRGGYRGGRGRGFWARGSRSRGRIY

>657

MAVTIEELYRNYGILADAKENLSQHKDAYQAILDGVRGGPKEKRLAAQFIPKFFSSFPELADAAINAQLDLCEDEDVSIRRQAIKELPRFATGENIFRVADILTQLLQTDDNAEFNQVNAALMSIFKMDAKGTLGGLFSQILQGDDIVRERAIKFLATKLKTLPEDVMTKEVEDYVFTETKKVLEDVTGEEFVLLMRVVSALRVLQSVNGRQQLVELVVEQADLEQALNPADPDAVDRLLQCTRQALPLFSKNVHSTRFVTYFCEHVLPNLSTLTSPVAELDIQLEVLKLLAEMSPFCGDMEKLEANLNTLFTKLVEFMPLPPDEVENGENSASEEPKLQFSYVECLLFSFHQLGKKLPDFLVDKVDAERLKDFKIRLQYFARGLQVYIRQLRMALQGKTGDALKTEENKIKVVALKITNNINVLIKDLFHNPPSYKSTVTLSWKPVQKPEAAAPKRPSGEEMGSGASTKKQITPLPRRDARQIYNPPSEQRGGYRGGRGRGFWARGSRSRGRIY

>658

MAVTIEELYRNYGILADAKENLSQHKDAYQAILDGVRGGPKEKRLAAQFIPKFFSSFPELADAAINAQLDLCEDEDVSIRRQAIKELPRFATGENIFRVADILTQLLQTDDNAEFNQVNAALMSIFKMDAKGTLGGLFSQILQGDDIVRERAIKFLATKLKTLPEDVMTKEVEDYVFTETKKVLEDVTGEEFVLLMRVVSALRVLQSVNGRQQLVELVVEQADLEQALNPADPDAVDRLLQCTRQALPLFSKNVHSTRFVTYFCEHVLPNLSTLTSPVAELDIQLEVLKLLAEMSPFCGDMEKLEANLNTLFTKLVEFMPLPPDEVENGENSASEEPKLQFSYVECLLFSFHQLGKKLPDFLVDKVDAERLKDFKIRLQYFARGLQVYIRQLRMALQGKTGDALKTEENKIKVVALKITNNINVLIKDLFHNPPSYKSTVTLSWKPVQKPEAAAPKRPSGEEMGSGASTKKQITPLPRRDARQIYNPPSGKYSASIGNFTYEQRGGYRGGRGRGFWARGSRSRGRIY

>659

MPTVEELYRNYGILADATEQVGQHKDAYQVILDGVKGGTKEKRLAAQFIPKFFKHFPELADSAINAQLDLCEDEDVSIRRQAIKELPQFATGENLPRVADILTQLLQTDDSAEFNLVNNALLSIFKMDAKGTLGGLFSQILQGEDIVRERAIKFLSTKLKTLPDEVLTKEVEELILTESKKVLEDVTGEEFVLFMKILSGLKSLQTVSGRQQLVELVAEQADLEQTFNPSDPDCVDRLLQCTRQAVPLFSKNVHSTRFVTYFCEQVLPNLSSLTTPVEGLDIQLEVLKLLAEMSSFCGDMEKLETNLRKLFDKLLEYMPLPPEEAENGENAGNEEPKLQFSYVECLLYSFHQLGRKLPDFLTAKLNAEKLKDFKIRLQYFARGLQVYIRQLRLALQGKTGEALKTEENKIKVVALKITNNINVLIKDLFHIPPSYKSTVTLSWKPVQKVEIGQKRASEDTTSGSPPKKSPAGPKRDARQIYNPPSGKYSSNLGNFNYERSLQGK

>660

MPTVEELYRNYGILADATEQVGQHKDAYQVILDGVKGGTKEKRLAAQFIPKFFKHFPELADSAINAQLDLCEDEDVSIRRQAIKELPQFATGENLPRVADILTQLLQTDDSAEFNLVNNALLSIFKMDAKGTLGGLFSQILQGEDIVRERAIKFLSTKLKTLPDEVLTKEVEELILTESKKVLEDVTGEEFVLFMKILSGLKSLQTVSGRQQLVELVAEQADLEQTFNPSDPDCVDRLLQCTRQAVPLFSKNVHSTRFVTYFCEQVLPNLSSLTTPVEGLDIQLEVLKLLAEMSSFCGDMEKLETNLRKLFDKLLEYMPLPPEEAENGENAGNEEPKLQFSYVECLLYSFHQLGRKLPDFLTAKLNAEKLKDFKIRLQYFARGLQVYIRQLRLALQGKTGEALKTEENKIKVVALKITNNINVLIKDLFHIPPSYKSTVTLSWKPVQKVEIGQKRASEDTTSGSPPKKSPAGPKRDARQIYNPPSGKYSSNLGNFNYEQRGAFRGSRGGRGWGARGNRSRGRLY

>661

MPSVEELYRNYGILADATEQVGQHKEAYQVILDGVKGGTKEKRLAAQFIPKFFKHFPELADSAINAQLDLCEDEDVSIRRQAIKELPQFATGENLPRVADILTQLLQTDDSAEFNLVNNALLSIFKMDAKGTLGGLFSQILQGEDIVRERAIKFLSTKLKTLPDEVLTKEVEELILTESKKVLEDVTGEEFVLFMKILSGLKSLQTVSGRQQLVELVAEQADLEQTFNPSDPDCVDRLLQCTRQAVPLFSKNVHSTRFVTYFCEQVLPNLSSLTTPVEGLDIQLEVLKLLAEMSSFCGDMEKLETNLRKLFDKLLEYMPLPPEEAENGENAGNEEPKLQFSYVECLLYSFHQLGRKLPDFLTAKLNAEKLKDFKIRLQYFARGLQVYIRQLRLALQGKTGEALKTEENKIKVVALKITNNINVLIKDLFHIPPSYKSTVTLSWKPVQKVEIGQKRANEDTTSGSPPKKSPAGPKRDARQIYNPPSGKYSSNLGNFNYERSLQGK

>662

MPSVEELYRNYGILADATEQVGQHKEAYQVILDGVKGGTKEKRLAAQFIPKFFKHFPELADSAINAQLDLCEDEDVSIRRQAIKELPQFATGENLPRVADILTQLLQTDDSAEFNLVNNALLSIFKMDAKGTLGGLFSQILQGEDIVRERAIKFLSTKLKTLPDEVLTKEVEELILTESKKVLEDVTGEEFVLFMKILSGLKSLQTVSGRQQLVELVAEQADLEQTFNPSDPDCVDRLLQCTRQAVPLFSKNVHSTRFVTYFCEQVLPNLSSLTTPVEGLDIQLEVLKLLAEMSSFCGDMEKLETNLRKLFDKLLEYMPLPPEEAENGENAGNEEPKLQFSYVECLLYSFHQLGRKLPDFLTAKLNAEKLKDFKIRLQYFARGLQVYIRQLRLALQGKTGEALKTEENKIKVVALKITNNINVLIKDLFHIPPSYKSTVTLSWKPVQKVEIGQKRANEDTTSGSPPKKSPAGPKRDARQIYNPPSGKYSSNLGNFNYEQRGAFRGSRGGRGWGARGNRSRGRLY

>663

MWRFMTTGPKFYVRPYMRKKSKHIRLCVNLTMSTDSIEKLYKNFGILADAKDKLAEHEKEYLEILTAVKGSPKEKRLASQFIARFFKHFPKLADQAIDAHLDLCEDEDLAIRKQAIKDLPALCKDNKEHTARIADILAQLLQAQDPTELAVVHNSIMSLMKSDPKGTISGFFSQIINGDDGTRERCIKFLATKLKAIGHDVITKEPEDMLISECKKVLQDVTADEFHSIMEILAWTRLGSTVSGQQELVDITIEQAELSIPFKHTNIEQWNRLVQCVKHALPFFSSQIDSSKFVSYICVQVLPHLSLITSPDGRDIQLELLKLLAELTVFCGTIEKPEDKVQQLYNTLITYMPLPPAADITDVPKLQFSHVECLMYAFHKLCKQTPEFLIKDGEQLKEFRLRLQYFARGIQGYIKKLREAISGKTEEELKSDENQLKVVALKTTNNINTLIKDLFHSPPSFKSIIHLSWKTHCNDKKNEKNSTQKRHTPITFGNDSNANKRIKEDKNNKREIYTPPSGKYSSNISSNYGRVRFRGNRSGGRGGYRSRGRGTWRKNFY

>664

MSEENKLKITALNTTSNINTLIRDIFRTPPSFKSKVQLSFHTKKSEKEALTVQSDEKETESAGQKRHQPITFDKGDNMSPEKRARSGDRNLKMYTPPSGKYSSRFTGNYSGRGRGGYGRRDFRNNGAPFRRRSNY

>665

MPTVEELYRNYGILADATEQVGQHKDAYQVILDGVKGGTKEKRLAAQFIPKFFKHFPELADSAINAQLDLCEDEDVSIRRQAIKELPQFATGENLPRVADILTQLLQTDDSAEFNLVNNALLSIFKMDAKGTLGGLFSQILQGEDIVRERAIKFLSTKLKTLPDEVLTKEVEELILTESKKVLEDVTGEEFVLFMKILSGLKSLQTVSGRQQLVELVAEQADLEQTFNPSDPDCVDRLLQCTRQAVPLFSKNVHSTRFVTYFCEQVLPNLSSLTTPVEGLDIQLEVLKLLAEMSSFCGDMEKLETNLRKLFDKLLEYMPLPPEEAENGENAGNEEPKLQFSYVECLLYSFHQLGRKLPDFLTAKLNAEKLKDFKIRLQYFARGLQVYIRQLRLALQGKTGEALKTDENKIKVVALKITNNINVLIKDLFHIPPSYKSTVTLSWKPVQKVELGQKRATEDTTSGSPPKKXAGPKRDARQIYNPPSGKYSSNLSNFNYEQRGAFRGSRGGRGWGARGNRSRGRLY

>666

MPTVEELYRNYGILADATETAGQHKDAYQVILDGVKGGAKEKRLAAQFIPKFFKHFPELADSAINAQLDLCEDEDVSIRRQAIKELPQFATGDNLPRVADILTQLLQSDDSAEFNLVNNALLSIFKMDAKGTLGGLFSQILQGEDIVRERAIKFLSTKLKTLPEEVMTKEVEEFILTESKKVLEDVTGEEFVLFMKILSGLKSLQTVSGRQQLVELVAEQADLEQTFNPSDTDCVDRLLQCTRQAVPLFSKNVHSTKFVTYFCEHVLPNLSSLTTLVEGLDIQLEVLKLLAEMSSFCGDMEKLESNLKKLFDKLLEYMPLPPEEAENGENAGNEEPKLQFSYVECLLYSFHQLGRKLPDFLTAKLNAEKLKDFKIRLQYFARGLQVYIRQLRLALQGKTGEALKTEEDLFHIPPSYKSTVTLSWKPVQKTDVSQKRASEDTTSSSPPKKASAGPKRDARQIYNPPSGKYSSNLGSFSYEQRGGFRGGRGRGWGGRGNRSRGRIY

>667

MPTVEELYRNYGILADATETAGQHKDAYQVILDGVKGGAKEKRLAAQFIPKFFKHFPELADSAINAQLDLCEDEDVSIRRQAIKELPQFATGDNLPRVADILTQLLQSDDSAEFNLVNNALLSIFKMDAKGTLGGLFSQILQGEDIVRERAIKFLSTKLKTLPEEVMTKEVEEFILTESKKVLEDVTGEEFVLFMKILSGLKSLQTVSGRQQLVELVAEQADLEQTFNPSDTDCVDRLLQCTRQAVPLFSKNVHSTKFVTYFCEHVLPNLSSLTTLVEGLDIQLEVLKLLAEMSSFCGDMEKLESNLKKLFDKLLEYMPLPPEEAENGENAGNEEPKLQFSYVECLLYSFHQLGRKLPDFLTAKLNAEKLKDFKIRLQYFARGLQVYIRQLRLALQGKTGEALKTEENKIKVVALKITNNINVLIKDLFHIPPSYKSTVTLSWKPVQKTDVSQKRASEDTTSSSPPKKASAGPKRDARQIYNPPSGKYSSNLGSFSYEQRGGFRGGRGRGWGGRGNRSRGRIY

>668

MAVTIEDLYRNYGILADAKENLSQHKDAYQGILDGVKGGAKEKRLAAQFIPKFFSSFPELADAAINAQLDLCEDEDVSIRRQAIKELPRFATGENIFRVADILTQLLQTDDTAEFNQVNAALLSIFKMDAKGTLGGLFSQILQGEDIVRERAIKFLSTKLKTLPEDVMTREVEEFVFAETKKVMEDVTGEEFVLLMRVVSGLRVLQSVNGRQQLVELVVDQADLEQALNPADPDAVDRLLQCTRQSLPLFSKNVHSTRFVTYFCENVLPNLSTLTSPVAELDIQLEVLKLLAEMSPFCGDMEKLEANLNTLFTKLLEFMPLPPEEVENGENSASEEPKLQFSYVECLIFSFHQLGKKLPDFLVDKVDAEHLKDFKIRLQYFARGLQVYIRQLRVALQGKTGDALKTDENKIKVVALKITNNINILIKDLFHNPPSFKSTVTLSWKPVQKPEAVAAKRPSGEEMGSGGSTNKQIAPLPRRDARQIYNPPSEQRGGFRGGRGRGFGARGGRSRGRVY

>669

MAVTIEDLYRNYGILADAKENLSQHKDAYQGILDGVKGGAKEKRLAAQFIPKFFSSFPELADAAINAQLDLCEDEDVSIRRQAIKELPRFATGENIFRVADILTQLLQTDDTAEFNQVNAALLSIFKMDAKGTLGGLFSQILQGEDIVRERAIKFLSTKLKTLPEDVMTREVEEFVFAETKKVMEDVTGEEFVLLMRVVSGLRVLQSVNGRQQLVELVVDQADLEQALNPADPDAVDRLLQCTRQSLPLFSKNVHSTRFVTYFCENVLPNLSTLTSPVAELDIQLEVLKLLAEMSPFCGDMEKLEANLNTLFTKLLEFMPLPPEEVENGENSASEEPKLQFSYVECLIFSFHQLGKKLPDFLVDKVDAEHLKDFKIRLQYFARGLQVYIRQLRVALQGKTGDALKTDENKIKVVALKITNNINILIKDLFHNPPSFKSTVTLSWKPVQKPEAVAAKRPSGEEMGSGGSTNKQIAPLPRRDARQIYNPPSGKYSASIGNLSYEQRGGFRGGRGRGFGARGGRSRGRVY

>670

MPTVEELYRNYGILADATEQVSQHKDAYQVILDGVKGGTKEKRLAAQFIPKFFKHFPELADSAINAQLDLCEDEDVSIRRQAIKELPQFATGENLPRVADILTQLLQTDDSAEFNLVNNALLSIFKMDAKGTLGGLFSQILQGEDIVRERAIKFLSTKLKTLPDEVLTKEVEEFILTESKKVLEDVTGEEFVLFMKILSGLKSLQTVSGRQQLVELVAEQADLEQTFNPSDPDCVDRLLQCTRQAVPLFSKNVHSTRFVTYFCEQVLPNLSTLTTPVEGLDIQLEVLKLLAEMSSFCGDMEKLETNLRKLFDKLLEYMPLPPEEAENGENAGNEEPKLQFSYVECLLYSFHQLGRKLPDFLTAKLNAEKLKDFKIRLQYFARGLQVYIRQLRLALQGKTGEALKTEENKIKVVALKITNNINVLIKDLFHIPPSYKSTVTLSWKPVQKVEIGQKRTNEDTTSGSPPKKSPAGPKRDARQIYNPPSGKYSSNLGNFNYERSLQGK

>671

MPTVEELYRNYGILADATEQVSQHKDAYQVILDGVKGGTKEKRLAAQFIPKFFKHFPELADSAINAQLDLCEDEDVSIRRQAIKELPQFATGENLPRVADILTQLLQTDDSAEFNLVNNALLSIFKMDAKGTLGGLFSQILQGEDIVRERAIKFLSTKLKTLPDEVLTKEVEEFILTESKKVLEDVTGEEFVLFMKILSGLKSLQTVSGRQQLVELVAEQADLEQTFNPSDPDCVDRLLQCTRQAVPLFSKNVHSTRFVTYFCEQVLPNLSTLTTPVEGLDIQLEVLKLLAEMSSFCGDMEKLETNLRKLFDKLLEYMPLPPEEAENGENAGNEEPKLQFSYVECLLYSFHQLGRKLPDFLTAKLNAEKLKDFKIRLQYFARGLQVYIRQLRLALQGKTGEALKTEENKIKVVALKITNNINVLIKDLFHIPPSYKSTVTLSWKPVQKVEIGQKRTNEDTTSGSPPKKSPAGPKRDARQIYNPPSGKYSSNLGNFNYEQRGAFRGSRGGRGWGARGNRSRGRLY

>672

MDNIEHLYKCYEILSEAGEKISEHVKEYEDILKAVKGSSKEKRLASQFIGKFFKHFPDLADAAIDAQLDLCEDDDMQIRRQAIKDLPKLCQDTESTTVKIGDTLAQLLVLDDPLELQQVNNSLQTIIKKDAKASLTGVFTQISTGDEATRERCFKFIATKLFAMGPTVVTKEVEEFVVDEIKKILQDVTADEFQLCMNILGSTKLGTTITGHAEMVNLAKEQAELNADIDAIAVEDEIVERFIQCATHAMPYFSNTIKSTDFVVYVCDKLLPLSTWNMIATAVAQDQVQLRLLKVFAEMCTFTDVLDNAAQRIDNVYQVLREYMPLPKLSEEEDASSPPPSFQFSHAECLLYALHTLGKKHPDMLTFVTDAEKLKDFRSRLQYLARGTQGYIKKLEEAVKGKSAEELKTEENQLKLTALKTTSNISILIRDLFHSPPSFKHEIQLSWVNRKNTKIGSKRHAPITFDGKSENGKDEEKKIKNASEQKIYSPPSGKYSGKVQNYGGNNNRQRSRNGGSGGGGGGGFRNRRNFNKKY

>673

MNGLLSDKHKDAYQAILDGVKGGAKEKRLAAQFIPKFFKHFPELADSAINAQLDLCEDEDVSIRRQAIKELPQFATGDNLPRVADILTQLLQSDDSAEFNLVNNALLSIFKMDAKGTLGGLFSQILQGEDIVRERAIKFLSTKLKTLPEEVLTKEVEEFILSESKKVLEDVTGEEFVLFMKILSGLKSLQTVSGRQQLVELVAEQADLEQTFNPSDPDCVDRLLQCTRQAVPLFSKNVHSTKFVTYFCEHVLPNLGSLTTPVEGVDIQLEVLKLLAEMSSFCGDMEKLESNLKKLFDKLLEYMPLPPEEAENGENASNEEPKLQFSYVECLLYSFHQLGRKLPDFLTAKLNAEKLKDFKIRLQYFARGLQVYIRQLRLALQGKTGEALKTEENKIKVVALKITNNINVLIKDLFHIPPSYKSTVTLSWKPVQKADASQKRTSEDTTSSSPPKKASAGPKRDARQIYNPPSGKYSSNLGSFSYEQRGGFRGGRGRGWGGRGNRSRGRIY

>674

MPGVSEPLRHKDAYQAILDGVKGGAKEKRLAAQFIPKFFKHFPELADSAINAQLDLCEDEDVSIRRQAIKELPQFATGDNLPRVADILTQLLQSDDSAEFNLVNNALLSIFKMDAKGTLGGLFSQILQGEDIVRERAIKFLSTKLKTLPEEVLTKEVEEFILSESKKVLEDVTGEEFVLFMKILSGLKSLQTVSGRQQLVELVAEQADLEQTFNPSDPDCVDRLLQCTRQAVPLFSKNVHSTKFVTYFCEHVLPNLGSLTTPVEGVDIQLEVLKLLAEMSSFCGDMEKLESNLKKLFDKLLEYMPLPPEEAENGENASNEEPKLQFSYVECLLYSFHQLGRKLPDFLTAKLNAEKLKDFKIRLQYFARGLQVYIRQLRLALQGKTGEALKTEENKIKVVALKITNNINVLIKDLFHIPPSYKSTVTLSWKPVQKADASQKRTSEDTTSSSPPKKASAGPKRDARQIYNPPSGKYSSNLGSFSYEQRGGFRGGRGRGWGGRGNRSRGRIY

>675

MPTVEELYRNYGILADATEQVGQHKDAYQVILDGVKGGTKEKRLAAQFIPKFFKHFPELADSAINAQLDLCEDEDVSIRRQAIKELPQFATGENLPRVADILTQLLQTDDSAEFNLVNNALLSIFKMDAKGTLGGLFSQILQGEDIVRERAIKFLSTKLKTLPDEVLTKEVEELILTESKKVLEDVTGEEFVLFMKILSGLKSLQTVSGRQQLVELVAEQADLEQTFNPSDPDCVDRLLQCTRQAVPLFSKNVHSTRFVTYFCEQVLPNLSSLTTPVEGLDIQLEVLKLLAEMSSFCGDMEKLETNLRKLFDKLLEYMPLPPEEAENGENAGNEEPKLQFSYVECLLYSFHQLGRKLPDFLTAKLNAEKLKDFKIRLQYFARGLQVYIRQLRLALQGKTGEALKTEENKIKVVALKITNNINVLIKDLFHIPPSYKSTVTLSWKPVQKVEIGQKRANEDTTSGSPPKKSSAGPKRDARQIYNPPSGKYSSNLSNFNYGN

>676

MPTVEELYRNYGILADATEQVGQHKDAYQVILDGVKGGTKEKRLAAQFIPKFFKHFPELADSAINAQLDLCEDEDVSIRRQAIKELPQFATGENLPRVADILTQLLQTDDSAEFNLVNNALLSIFKMDAKGTLGGLFSQILQGEDIVRERAIKFLSTKLKTLPDEVLTKEVEELILTESKKVLEDVTGEEFVLFMKILSGLKSLQTVSGRQQLVELVAEQADLEQTFNPSDPDCVDRLLQCTRQAVPLFSKNVHSTRFVTYFCEQVLPNLSSLTTPVEGLDIQLEVLKLLAEMSSFCGDMEKLETNLRKLFDKLLEYMPLPPEEAENGENAGNEEPKLQFSYVECLLYSFHQLGRKLPDFLTAKLNAEKLKDFKIRLQYFARGLQVYIRQLRLALQGKTGEALKTEENKIKVVALKITNNINVLIKDLFHIPPSYKSTVTLSWKPVQKVEIGQKRANEDTTSGSPPKKSSAGPKRDARQIYNPPSGKYSSNLSNFNYERSLQGK

>677

MPTVEELYRNYGILADATEQVGQHKDAYQVILDGVKGGTKEKRLAAQFIPKFFKHFPELADSAINAQLDLCEDEDVSIRRQAIKELPQFATGENLPRVADILTQLLQTDDSAEFNLVNNALLSIFKMDAKGTLGGLFSQILQGEDIVRERAIKFLSTKLKTLPDEVLTKEVEELILTESKKVLEDVTGEEFVLFMKILSGLKSLQTVSGRQQLVELVAEQADLEQTFNPSDPDCVDRLLQCTRQAVPLFSKNVHSTRFVTYFCEQVLPNLSSLTTPVEGLDIQLEVLKLLAEMSSFCGDMEKLETNLRKLFDKLLEYMPLPPEEAENGENAGNEEPKLQFSYVECLLYSFHQLGRKLPDFLTAKLNAEKLKDFKIRLQYFARGLQVYIRQLRLALQGKTGEALKTEENKIKVVALKITNNINVLIKDLFHIPPSYKSTVTLSWKPVQKVEIGQKRANEDTTSGSPPKKSSAGPKRDARQIYNPPSGKYSSNLSNFNYEQRGAFRGSRGGRGWGARGNRSRGRLY

>678

MPTVEELYRNYGILADATEQVGQHKDAYQVILDGVKGGTKEKRLAAQFIPKFFKHFPELADSAINAQLDLCEDEDVSIRRQAIKELPQFATGDNLPRVADILTQLLQTDDSAEFNLVNNALLSIFKMDAKGTLGGLFSQILQGEDIVRERAIKFLSTKLKTLPDEVLTKEVEELILTESKKVLEDVTGEEFVLFMKILSGLKSLQTVSGRQQLVELVAEQADLEQTFNPSDPDCVDRLLQCTRQAVPLFSKNVHSTRFVTYFCEQVLPNLSTLTTPVEGLDIQLEVLKLLAEMSSFCGDMEKLETNLRKLFDKLLEYMPLPPEEAENGENAGNEEPKLQFSYVECLLYSFHQLGRKLPDFLTAKLNAEKLKDFKIRLQYFARGLQVYIRQLRLALQGKTGEALKTEENKIKVVALKITNNINVLIKDLFHIPPSYKSTVTLSWKPVQKVEIGQKRASEDTTSSSPPKKSSAGPKRDARQIYNPPSGKYSSNLGNFNYERSLQGK

>679

MPTVEELYRNYGILADATEQVGQHKDAYQVILDGVKGGTKEKRLAAQFIPKFFKHFPELADSAINAQLDLCEDEDVSIRRQAIKELPQFATGDNLPRVADILTQLLQTDDSAEFNLVNNALLSIFKMDAKGTLGGLFSQILQGEDIVRERAIKFLSTKLKTLPDEVLTKEVEELILTESKKVLEDVTGEEFVLFMKILSGLKSLQTVSGRQQLVELVAEQADLEQTFNPSDPDCVDRLLQCTRQAVPLFSKNVHSTRFVTYFCEQVLPNLSTLTTPVEGLDIQLEVLKLLAEMSSFCGDMEKLETNLRKLFDKLLEYMPLPPEEAENGENAGNEEPKLQFSYVECLLYSFHQLGRKLPDFLTAKLNAEKLKDFKIRLQYFARGLQVYIRQLRLALQGKTGEALKTEENKIKVVALKITNNINVLIKDLFHIPPSYKSTVTLSWKPVQKVEIGQKRASEDTTSSSPPKKSSAGPKRDARQIYNPPSGKYSSNLGNFNYEQRGAFRGSRGGRGWGTRGNRSRGRLY

>680

MPSVEELYRNYGILADATEQVGQHKDSYQVILDGVKGGTKEKRLAAQFIPKFFKHFPELADSAINAQLDLCEDEDVSIRRQAIKELPQFATGENLPRVADILTQLLQTDDSAEFNLVNNALLSIFKMEKLETNLRKLFDKLLEYMPLPPEEAENGENAGNEEPKLQFSYVECLLYSFHQLGRKLPDFLTAKLNAEKLKDFKIRLQYFARGLQVYIRQLRLALQGKTGEALKTEENKIKVVALKITNNINVLIKDLFHIPPSYKSTVTLSWKPVQKVELGQRRANEDTTSGSPPKKSAAGPKRDARQIYNPPSGKYSSNLGNFNYERSLQGK

>681

MPSVEELYRNYGILADATEQVGQIRRQAIKELPQFATGENLPRVADILTQLLQTDDSAEFNLVNNALLSIFKMDAKGTLGGLFSQILQGEDIVRERAIKFLSTKLKTLPEEVLTKEVEELILTESKKVLEDVTGEEFVLFMKILSGLKSLQTVSGRQQLVELVAEQADLEQTFNPSDPDCVDRLLQCTRQAVPLFSKNVHSTRFVTYFCEHVLPNLSSLTTPVEGLDTQLEVLKLLAEMSSFCGDMEKLETNLRKLFDKLLEYMPLPPEEAENGENAGNEEPKLQFSYVECLLYSFHQLGRKLPDFLTAKLNAEKLKDFKIRLQYFARGLQVYIRQLRLALQGKTGEALKTEENKIKVVALKITNNINVLIKDLFHIPPSYKSTVTLSWKPVQKVELGQRRANEDTTSGSPPKKSAAGPKRDARQIYNPPSGKYSSNLGNFNYERSLQGK

>682

MPSVEELYRNYGILADATEQVGQHKDSYQVILDGVKGGTKEKRLAAQFIPKFFKHFPELADSAINAQLDLCEDEDVSIRRQAIKELPQFATGENLPRVADILTQLLQTDDSAEFNLVNNALLSIFKMDAKGTLGGLFSQILQGEDIVRERAIKFLSTKLKTLPEEVLTKEVEELILTESKKVLEDVTGEEFVLFMKILSGLKSLQTVSGRQQLVELVAEQADLEQTFNPSDPDCVDRLLQCTRQAVPLFSKNVHSTRFVTYFCEHVLPNLSSLTTPVEGLDTQLEVLKLLAEMSSFCGDMEKLETNLRKLFDKLLEYMPLPPEEAENGENAGNEEPKLQFSYVECLLYSFHQLGRKLPDFLTAKLNAEKLKDFKIRLQYFARGLQVYIRQLRLALQGKTGEALKTEENKIKVVALKITNNINVLIKDLFHIPPSYKSTVTLSWKPVQKVELGQRRANEDTTSGSPPKKSAAGPKRDARQIYNPPSGKYSSNLGNFNYERSLQGK

>683

MPSVEELYRNYGILADATEQVGQHKDSYQVILDGVKGGTKEKRLAAQFIPKFFKHFPELADSAINAQLDLCEDEDVSIRRQAIKELPQFATGENLPRVADILTQLLQTDDSAEFNLVNNALLSIFKMDAKGTLGGLFSQILQGEDIVRERAIKFLSTKLKTLPEEVLTKEVEELILTESKKVLEDVTGEEFVLFMKILSGLKSLQTVSGRQQLVELVAEQADLEQTFNPSDPDCVDRLLQCTRQAVPLFSKNVHSTRFVTYFCEHVLPNLSSLTTPVEGLDTQLEVLKLLAEMSSFCGDMEKLETNLRKLFDKLLEYMPLPPEEAENGENAGNEEPKLQFSYVECLLYSFHQLGRKLPDFLTAKLNAEKLKDFKIRLQYFARGLQVYIRQLRLALQGKTGEALKTEENKIKVVALKITNNINVLIKDLFHIPPSYKSTVTLSWKPVQKVELGQRRANEDTTSGSPPKKSAAGPKRDARQIYNPPSGKYSSNLGNFNYEQRGAFRGSRGGRGWGARGNRSRGRLF

>684

MPTVEELYRNYGILADATEQVGQHKDAYQVILDGVKGGTKEKRLAAQFIPKFFKHFPELADSAINAQLDLCEDEDVSIRRQAIKELPQFATGENLPRVADILTQLLQTDDSAEFNLVNNALLSIFKMDKLETNLRKLFDKLLEYMPLPPEEAENGENAGNEEPKLQFSYVECLLYSFHQLGRKLPDFLTAKLNAEKLKDFKIRLQYFARGLQVYIRQLRLALQGKTGEALKTEENKIKVVALKITNNINVLIKDLFHIPPSYKSTVTLSWKPVQKVEIGQKRASEDTTSRSPPKKPPAGPKRDARQIYNPPSGKYSSNLGNFNYERSLQGK

>685

MPTVEELYRNYGILADATEQVGQIRRQAIKELPQFATGENLPRVADILTQLLQTDDSAEFNLVNNALLSIFKMDAKGTLGGLFSQILQGEDIVRERAIKFLSTKLKTLPDEVLIKEVEELILTESKKVLEDVTGEEFVLFMKILSGLKSLQTVSGRQQLVELVAEQADLEQTFNPSDPDCVDRLLQCTRQAVPLFSKNVHSTRFVTYFCEQVLPNLSTLTTPVEGLDIQLEVLKLLAEMSSFCGDMEKLETNLRKLFDKLLEYMPLPPEEAENGENAGNEEPKLQFSYVECLLYSFHQLGRKLPDFLTAKLNAEKLKDFKIRLQYFARGLQVYIRQLRLALQGKTGEALKTEENKIKVVALKITNNINVLIKDLFHIPPSYKSTVTLSWKPVQKVEIGQKRASEDTTSRSPPKKPPAGPKRDARQIYNPPSGKYSSNLGNFNYERSLQGK

>686

MPTVEELYRNYGILADATEQVGQHKDAYQVILDGVKGGTKEKRLAAQFIPKFFKHFPELADSAINAQLDLCEDEDVSIRRQAIKELPQFATGENLPRVADILTQLLQTDDSAEFNLVNNALLSIFKMDAKGTLGGLFSQILQGEDIVRERAIKFLSTKLKTLPDEVLIKEVEELILTESKKVLEDVTGEEFVLFMKILSGLKSLQTVSGRQQLVELVAEQADLEQTFNPSDPDCVDRLLQCTRQAVPLFSKNVHSTRFVTYFCEQVLPNLSTLTTPVEGLDIQLEVLKLLAEMSSFCGDMEKLETNLRKLFDKLLEYMPLPPEEAENGENAGNEEPKLQFSYVECLLYSFHQLGRKLPDFLTAKLNAEKLKDFKIRLQYFARGLQVYIRQLRLALQGKTGEALKTEENKIKVVALKITNNINVLIKDLFHIPPSYKSTVTLSWKPVQKVEIGQKRASEDTTSRSPPKKPPAGPKRDARQIYNPPSGKYSSNLGNFNYERSLQGK

>687

MPTVEELYRNYGILADATEQVGQHKDAYQVILDGVKGGTKEKRLAAQFIPKFFKHFPELADSAINAQLDLCEDEDVSIRRQAIKELPQFATGENLPRVADILTQLLQTDDSAEFNLVNNALLSIFKMDAKGTLGGLFSQILQGEDIVRERAIKFLSTKLKTLPDEVLIKEVEELILTESKKVLEDVTGEEFVLFMKILSGLKSLQTVSGRQQLVELVAEQADLEQTFNPSDPDCVDRLLQCTRQAVPLFSKNVHSTRFVTYFCEQVLPNLSTLTTPVEGLDIQLEVLKLLAEMSSFCGDMEKLETNLRKLFDKLLEYMPLPPEEAENGENAGNEEPKLQFSYVECLLYSFHQLGRKLPDFLTAKLNAEKLKDFKIRLQYFARGLQVYIRQLRLALQGKTGEALKTEENKIKVVALKITNNINVLIKDLFHIPPSYKSTVTLSWKPVQKVEIGQKRASEDTTSRSPPKKPPAGPKRDARQIYNPPSGKYSSNLGNFNYEQRGAFRGSRGGRGWGTRGNRSRGRLY

>688

MPTVEELYRNYGILADATEQVGQHKDAYQVILDGVKGGTKEKRLAAQFIPKFFKHFPELADSAINAQLDLCEDEDVSIRRQAIKELPQFATGENLPRVADILTQLLQTDDSAEFNLVNNALLSIFKMEKLETNLRKLFDKLLEYMPLPPEEAENGENAGNEEPKLQFSYVECLLYSFHQLGRKLPDFLTAKLNAEKLKDFKIRLQYFARGLQVYIRQLRLALQGKTGEALKTEENKIKVVALKITNNINVLIKDLFHIPPSYKSTVTLSWKPVQKVEMGQKRASEDTTSGSPPKKSQGGPKRDARQIYNPPSGKYSSNLSNFNYERSLQGK

>689

MPTVEELYRNYGILADATEQVGQIRRQAIKELPQFATGENLPRVADILTQLLQTDDSAEFNLVNNALLSIFKMDAKGTLGGLFSQILQGEDIVRERAIKFLSTKLKTLPDEVLTKEVEELILTESKKVLEDVTGEEFVLFMKILSGLKSLQTVSGRQQLVELVAEQADLEQTFNPSDPDCVDRLLQCTRQAVPLFSKNVHSTKFVTYFCEHVLPNLSSLTTPVEGLDIQLEVLKLLAEMSSFCGDMEKLETNLRKLFDKLLEYMPLPPEEAENGENAGNEEPKLQFSYVECLLYSFHQLGRKLPDFLTAKLNAEKLKDFKIRLQYFARGLQVYIRQLRLALQGKTGEALKTEENKIKVVALKITNNINVLIKDLFHIPPSYKSTVTLSWKPVQKVEMGQKRASEDTTSGSPPKKSQGGPKRDARQIYNPPSGKYSSNLSNFNYERSLQGK

>690

MPTVEELYRNYGILADATEQVGQHKDAYQVILDGVKGGTKEKRLAAQFIPKFFKHFPELADSAINAQLDLCEDEDVSIRRQAIKELPQFATGENLPRVADILTQLLQTDDSAEFNLVNNALLSIFKMDAKGTLGGLFSQILQGEDIVRERAIKFLSTKLKTLPDEVLTKEVEELILTESKKVLEDVTGEEFVLFMKILSGLKSLQTVSGRQQLVELVAEQADLEQTFNPSDPDCVDRLLQCTRQAVPLFSKNVHSTKFVTYFCEHVLPNLSSLTTPVEGLDIQLEVLKLLAEMSSFCGDMEKLETNLRKLFDKLLEYMPLPPEEAENGENAGNEEPKLQFSYVECLLYSFHQLGRKLPDFLTAKLNAEKLKDFKIRLQYFARGLQVYIRQLRLALQGKTGEALKTEENKIKVVALKITNNINVLIKDLFHIPPSYKSTVTLSWKPVQKVEMGQKRASEDTTSGSPPKKSQGGPKRDARQIYNPPSGKYSSNLSNFNYERSLQGK

>691

MPTVEELYRNYGILADATEQVGQHKDAYQVILDGVKGGTKEKRLAAQFIPKFFKHFPELADSAINAQLDLCEDEDVSIRRQAIKELPQFATGENLPRVADILTQLLQTDDSAEFNLVNNALLSIFKMDAKGTLGGLFSQILQGEDIVRERAIKFLSTKLKTLPDEVLTKEVEELILTESKKVLEDVTGEEFVLFMKILSGLKSLQTVSGRQQLVELVAEQADLEQTFNPSDPDCVDRLLQCTRQAVPLFSKNVHSTKFVTYFCEHVLPNLSSLTTPVEGLDIQLEVLKLLAEMSSFCGDMEKLETNLRKLFDKLLEYMPLPPEEAENGENAGNEEPKLQFSYVECLLYSFHQLGRKLPDFLTAKLNAEKLKDFKIRLQYFARGLQVYIRQLRLALQGKTGEALKTEENKIKVVALKITNNINVLIKDLFHIPPSYKSTVTLSWKPVQKVEMGQKRASEDTTSGSPPKKSQGGPKRDARQIYNPPSGKYSSNLSNFNYEQRGAFRGSRGGRGWGARGNRSRGRLY

>692

MPTVEELYRNYGILADATEQVGQHKDAYQVILDGVKGGTKEKRLAAQFIPKFFKHFPELADSAINAQLDLCEDEDVSIRRQAIKELPQFATGENLPRVADILTQLLQTDDSAEFNLVNNALLSIFKMDKLETNLRKLFDKLLEYMPLPPEEAENGENAGNEEPKLQFSYVECLLYSFHQLGRKLPDFLTAKLNAEKLKDFKIRLQYFARGLQVYIRQLRLALQGKTGDALKTEENKIKVVALKITNNINVLIKDLFHIPPSYKSTVTLSWKPVQKVELGQKRATEDTTSGSPPKKSAAGPKRDARQIYNPPSGKYSSNLSNFNYERSLQGK

>693

MPTVEELYRNYGILADATEQVGQIRRQAIKELPQFATGENLPRVADILTQLLQTDDSAEFNLVNNALLSIFKMDAKGTLGGLFSQILQGEDIVRERAIKFLSTKLKTLPDEVLTKEVEELVLTESKKVLEDVTGEEFVLFMKILSGLKSLQTVSGRQQLVELVAEQADLEQTFNPSDADCVDRLLQCTRQAVPLFSKNVHSTRFVTYFCEQVLPNLGTLTTPVEGLDIQLEVLKLLAEMSSFCGDMEKLETNLRKLFDKLLEYMPLPPEEAENGENAGNEEPKLQFSYVECLLYSFHQLGRKLPDFLTAKLNAEKLKDFKIRLQYFARGLQVYIRQLRLALQGKTGDALKTEENKIKVVALKITNNINVLIKDLFHIPPSYKSTVTLSWKPVQKVELGQKRATEDTTSGSPPKKSAAGPKRDARQIYNPPSGKYSSNLSNFNYERSLQGK

>694

MPTVEELYRNYGILADATEQVGQHKDAYQVILDGVKGGTKEKRLAAQFIPKFFKHFPELADSAINAQLDLCEDEDVSIRRQAIKELPQFATGENLPRVADILTQLLQTDDSAEFNLVNNALLSIFKMDAKGTLGGLFSQILQGEDIVRERAIKFLSTKLKTLPDEVLTKEVEELVLTESKKVLEDVTGEEFVLFMKILSGLKSLQTVSGRQQLVELVAEQADLEQTFNPSDADCVDRLLQCTRQAVPLFSKNVHSTRFVTYFCEQVLPNLGTLTTPVEGLDIQLEVLKLLAEMSSFCGDMEKLETNLRKLFDKLLEYMPLPPEEAENGENAGNEEPKLQFSYVECLLYSFHQLGRKLPDFLTAKLNAEKLKDFKIRLQYFARGLQVYIRQLRLALQGKTGDALKTEENKIKVVALKITNNINVLIKDLFHIPPSYKSTVTLSWKPVQKVELGQKRATEDTTSGSPPKKSAAGPKRDARQIYNPPSGKYSSNLSNFNYERSLQGK

>695

MPTVEELYRNYGILADATEQVGQHKDAYQVILDGVKGGTKEKRLAAQFIPKFFKHFPELADSAINAQLDLCEDEDVSIRRQAIKELPQFATGENLPRVADILTQLLQTDDSAEFNLVNNALLSIFKMDAKGTLGGLFSQILQGEDIVRERAIKFLSTKLKTLPDEVLTKEVEELVLTESKKVLEDVTGEEFVLFMKILSGLKSLQTVSGRQQLVELVAEQADLEQTFNPSDADCVDRLLQCTRQAVPLFSKNVHSTRFVTYFCEQVLPNLGTLTTPVEGLDIQLEVLKLLAEMSSFCGDMEKLETNLRKLFDKLLEYMPLPPEEAENGENAGNEEPKLQFSYVECLLYSFHQLGRKLPDFLTAKLNAEKLKDFKIRLQYFARGLQVYIRQLRLALQGKTGDALKTEENKIKVVALKITNNINVLIKDLFHIPPSYKSTVTLSWKPVQKVELGQKRATEDTTSGSPPKKSAAGPKRDARQIYNPPSGKYSSNLSNFNYEQRGAFRGSRGGRGWGARGNRSRGRLY

>696

MPTVEELYRNYGILADATEQVGQHKDAYQVILDGVKGGTKEKRLAAQFIPKFFKHFPELADSAINAQLDLCEDEDVSIRRQAIKELPQFATGENLPRVADILTQLLQTDDSAEFNLVNNALLSIFKMDAKGTLGGLFSQILQGEDIVRERAIKFLSTKLKTLPEEVLTKEVEELILTESKKVLEDVTGEEFVLFMKILSGLKSLQTVSGRQQLVELVAEQADLEQTFNPSDPDCVDRLLQCTRQAVPLFSKNVHSTKFVTYFCEHVLPNLSSLTTPVEGLDIQLEVLKLLAEMSSFCGDMEKLETNLRKLFDKLLEYMPLPPEEAENGENAGNEEPKLQFSYVECLLYSFHQLGRKLPDFLTAKLNAEKLKDFKIRLQYFARGLQVYIRQLRLALQGKTGEALKTEENKIKVVALKITNNINVLIKDLFHIPPSYKSTVTLSWKPVQKVEIGQKRANEDTTSGSPPKKSSAGPKRDARQIYNPPSGKYSSNLGNFNYERSLQGK

>697

MPTVEELYRNYGILADATEQVGQHKDAYQVILDGVKGGTKEKRLAAQFIPKFFKHFPELADSAINAQLDLCEDEDVSIRRQAIKELPQFATGENLPRVADILTQLLQTDDSAEFNLVNNALLSIFKMDAKGTLGGLFSQILQGEDIVRERAIKFLSTKLKTLPEEVLTKEVEELILTESKKVLEDVTGEEFVLFMKILSGLKSLQTVSGRQQLVELVAEQADLEQTFNPSDPDCVDRLLQCTRQAVPLFSKNVHSTKFVTYFCEHVLPNLSSLTTPVEGLDIQLEVLKLLAEMSSFCGDMEKLETNLRKLFDKLLEYMPLPPEEAENGENAGNEEPKLQFSYVECLLYSFHQLGRKLPDFLTAKLNAEKLKDFKIRLQYFARGLQVYIRQLRLALQGKTGEALKTEENKIKVVALKITNNINVLIKDLFHIPPSYKSTVTLSWKPVQKVEIGQKRANEDTTSGSPPKKSSAGPKRDARQIYNPPSGKYSSNLGNFNYEQRGAFRGSRGGRGWGARGNRSRGRLY

>698

MPILLSIFGEKAPPERLEELIGIIEGQDDLDARFDVSDADDIDRLISCLFMALPFFVRGASGSKFLYYLNKHIIPVFDELQEERKLDLLKALAEILPYTTPQDSRQILPSIVQLLKYMLRRKTGEETNFTYVECLLFVFHHFSHKAPNATNSLWGYXIVTGQPSDRLGEDFSEYYKDFTERLSSVEDLTRVTVKKLTLGMAENNKAMAAAKSDEAKDSIKTQKQNTTTSLQICNNILAMTRPLHXKRTAFIGDKSVNLSWKEAIKPSAPSTVNPTGQMDSCYTTPAANGSNNLVTKKGRGAGGMQNQLVNRALDGISLGGRGGRRGRGYL

>699

MPTVEELYRNYGILADATEQVGQIRRQAIKELPQFATGENLPRVADILTQLLQTDDSAEFNLVNNALLSIFKMDAKGTLGGLFSQILQGEDIVRERAIKFLSTKLKTLPDEVLTKEVEELILTESKKVLEDVTGEEFVLFMKILSGLKSLQTVSGRQQLVELVAEQADLEQTFNPSDPDCVDRLLQCTRQAVPLFSKNVHSTRFVTYFCEQVLPNLSSLTTPVEGLDIQLEVLKLLAEMSSFCGDMEKLETNLRKLFDKLLEYMPLPPEEAENGENAGNEEPKLQFSYVECLLYSFHQLGRKLPDFLTAKLNAEKLKDFKIRLQYFARGLQVYIRQLRLALQGKTGEALKTEENKIKVVALKITNNINVLIKDLFHIPPSYKSTVTLSWKPVQKVEIGQKRANEDTTSGSPPKKSSAGPKRDARQIYNPPSGKYSSNLGNFNYERSLQGK

>700

MPTVEELYRNYGILADATEQVGQHKDAYQVILDGVKGGTKEKRLAAQFIPKFFKHFPELADSAINAQLDLCEDEDVSIRRQAIKELPQFATGENLPRVADILTQLLQTDDSAEFNLVNNALLSIFKMEKLETNLRKLFDKLLEYMPLPPEEAENGENAGNEEPKLQFSYVECLLYSFHQLGRKLPDFLTAKLNAEKLKDFKIRLQYFARGLQVYIRQLRLALQGKTGEALKTEENKIKVVALKITNNINVLIKDLFHIPPSYKSTVTLSWKPVQKVEIGQKRANEDTTSGSPPKKSSAGPKRDARQIYNPPSGKYSSNLGNFNYERSLQGK

>701

MSTDSIEKLYRNFGILADAKDKLVQHEKEYLEILTAVKGSPKEKRLASQFIARFFKHFPKLADQAIDAHLDLCEDEDMAIRKQAIKDLPALCKDNKEHTARIADILAQLLQAEDPSELAVVHNSIMSLMKSDPKGTLSGFFSQIISGDDGTRERCIKFLAIKLKAIGHDIITKEPEDLLIVECKKVLQDVTADEFHSIMEILAWTRLGSTITGQQELVDITIEQAELSVPFKHTNVEQWNRLVQCVKHALPFFSVCIYIYIYIYTHTHTHTHFDNIFLCVSXXSNVRILLIKIIKFYQISKSLLIIHYSLYLFXTYMPLPPATEITDVPKLQFSHVECLMYAFHKLCKQTPEFLIKDAEQLKEFRLRLQYFARGIQGYIKKLREAISGKTEEELKSEENQLKVVALKTTNNINTLIKDLFHSPPSFKSIIHLSWKTPCNDKKSDKNSAQKRHTPITFGNDNSPNKRSKEDKNSKREIYTPPSGKYSSTISNYGRGRFKSNRSRGRGGFRTRGRGSWRKNFY

>702

MPTVEELYRNYGILADATEQVGQHKDAYQVILDGVKGGTKEKRLAAQFIPKFFKHFPELADSAINAQLDLCEDEDVSIRRQAIKELPQFATGENLPRVADILTQLLQTDDSAEFNLVNNALLSIFKMDAKGTLGGLFSQILQGEDIVRERAIKFLSTKLKTLPDEVLTKEVEELILTESKKVLEDVTGEEFVLFMKILSGLKSLQTVSGRQQLVELVAEQADLEQTFNPSDPDCVDRLLQCTRQAVPLFSKNVHSTRFVTYFCEQVLPNLGTLTTPVEGLDIQLEVLKLLAEMSSFCGDMEKLETNLRKLFDKLLEYMPLPPEEAENGENAGNEEPKLQFSYVECLLYSFHQLGRKLPDFLTAKLNAEKLKDFKIRLQYFARGLQVYIRQLRLALQGKTGEALKTEENKIKVVALKITNNINVLIKDLFHIPPSYKSTVTLSWKPVQKVEIGQKRASEDTTSGSPPKKSSAGPKRDARQIYNPPSGKYSSNLGNFNYAP

>703

MPTVEELYRNYGILADATEQVGQIRRQAIKELPQFATGENLPRVADILTQLLQTDDSAEFNLVNNALLSIFKMDAKGTLGGLFSQILQGEDIVRERAIKFLSTKLKTLPDEVLTKEVEELILTESKKVLEDVTGEEFVLFMKILSGLKSLQTVSGRQQLVELVAEQADLEQTFNPSDPDCVDRLLQCTRQAVPLFSKNVHSTRFVTYFCEQVLPNLSTLTTPVEGLDIQLEVLKLLAEMSSFCGDMEKLETNLRKLFDKLLEYMPLPPEEAENGENAGNEEPKLQFSYVECLLYSFHQLGRKLPDFLTAKLNAEKLKDFKIRLQYFARGLQVYIRQLRLALQGKTGEALKTEENKIKVVALKITNNINVLIKDLFHIPPSYKSTVTLSWKPVQKVEIGQKRASEDTTSGSPPKKSSAGPKRDARQIYNPPSGKYSSNLGNFNYERSLQGK

>704

MSPGAWGQDVSQGRARSQENPSDRSVEPAAVSIIKMNHLPPTLGASFAASGKSWKEDGKNESPTAINILQFDTTLSLFTMHKDAYQVILDGVKGGTKEKRLAAQFIPKFFKHFPELADSAINAQLDLCEDEDVSIRRQAIKELPQFATGENLPRVADILTQLLQTDDSAEFNLVNNALLSIFKMDAKGTLGGLFSQILQGEDIVRERAIKFLSTKLKTLPDEVLTKEVEELILTESKKVLEDVTGEEFVLFMKILSGLKSLQTEYMPLPPEEAENGENAGNEEPKLQFSYVECLLYSFHQLGRKLPDFLTAKLNAEKLKDFKIRLQYFARGLQVYIRQLRLALQGKTGEALKTDENKIKVVALKITNNINVLIKDLFHIPPSYKSTVTLSWKPVQKVELGQKRASEDTTSGSPPKKSSAGPKRDARQIYNPPSGKYSSNLGNFNYALGSGILLNP

>705

MKHKDAYQAILDGVKGGAKEKRLAAQFIPKFFKHFPELADSAINAQLDLCEDEDVSIRRQAIKELPQFATGDNLPRVADILTQLLQSDDSAEFNLVNNALLSIFKMDAKGTLGGLFSQILQGEDIVRERAIKFLSTKLKTLPEEVMTKEVEEFILTESKKVLEDVTGEEFVLFMKILSGLKSLQTVSGRQQLVELVAEQADLEQTFNPSDPDCVDRLLQCTRQAVPLFSKNVHSTKFVTYFCEHVLPNLSSLTTPVEGLDIQLEVLKLLAEMSSFCGDMEKLESNLKKLFDKLLEYMPLPPEEAENGENAGNEEPKLQFSYVECLLYSFHQLGRKLPDFLTAKLNAEKLKDFKIRLQYFARGLQVYIRQLRLALQGKTGEALKTEENKIKVVALKITNNINVLIKDLFHIPPSYKSTVTLSWKPVQKADASQKRASEDTTSSSPPKKASAGPKRDARQIYNPPSGKYSSNLGSFSYALAPQMSHWTDQLLQMAELGHLSNDWTCAEFLLQPLLMLQT

>706

MPTVEELYRNYGILADATETAGQHKDAYQAILDGVKGGAKEKRLAAQFIPKFFKHFPELADSAINAQLDLCEDEDVSIRRQAIKELPQFATGDNLPRVADILTQLLQSDDSAEFNLVNNALLSIFKMDAKGTLGGLFSQILQGEDIVRERAIKFLSTKLKTLPEEVMTKEVEEFILTESKKVLEDVTGEEFVLFMKILSGLKSLQTVSGRQQLVELVAEQADLEQTFNPSDPDCVDRLLQCTRQAVPLFSKNVHSTKFVTYFCEHVLPNLSSLTTPVEGLDIQLEVLKLLAEMSSFCGDMEKLESNLKKLFDKLLEYMPLPPEEAENGENAGNEEPKLQFSYVECLLYSFHQLGRKLPDFLTAKLNAEKLKDFKIRLQYFARGLQVYIRQLRLALQGKTGEALKTEENKIKVVALKITNNINVLIKDLFHIPPSYKSTVTLSWKPVQKADASQKRASEDTTSSSPPKKASAGPKRDARQIYNPPSGKYSSNLGSFSYEQRGGFRGGRGRGWGGRGNRSRGRIY

>707

MPTVEELYRNYGILADATETAGQHKDAYQAILDGVKGGAKEKRLAAQFIPKFFKHFPELADSAINAQLDLCEDEDVSIRRQAIKELPQFATGDNLPRVADILTQLLQSDDSAEFNLVNNALLSIFKMDAKGTLGGLFSQILQGEDIVRERAIKFLSTKLKTLPEEVMTKEVEEFILTESKKVLEDVTGEEFVLFMKILSGLKSLQTVSGRQQLVELVAEQADLEQTFNPSDPDCVDRLLQCTRQAVPLFSKNVHSTKFVTYFCEHVLPNLSSLTTPVEGLDIQLEVLKLLAEMSSFCGDMEKLESNLKKLFDKLLEYMPLPPEEAENGENAGNEEPKLQFSYVECLLYSFHQLGRKLPDFLTAKLNAEKLKDFKIRLQYFARGLQVYIRQLRLALQGKTGEALKTEENKIKVVALKITNNINVLIKDLFHIPPSYKSTVTLSWKPVQKADASQKRASEDTTSSSPPKKASAGPKRDARQIYNPPSGKYSSNLGSFSYALAPQMSHWTDQLLQMAELGHLSNDWTCAEFLLQPLLMLQT

>708

MSTDSIEKLYRNFGILADAKDKLIQHEKEYLEILTAVKGSPKEKRLASQFIARFFKHFPKLADQAIDAHLDLCEDEDMAIRKQAIKDLPALCKDNKEHTARIADILAQLLQAEDPSELAVVHNSIMSLMKSDPKGTLSGFFSQIISGDDGTRERCIKFLAIKLKAIGHDIITKEPEDLLIVECKKVLQVIYYDFYKHTNIEQWNRLVQCIKHALPFFSSQIDSSKFVSYICVQVLPHLSLMTSPDGRDIQLELLKLLAELTVFCGTIEKPEDKVQQLYNTLITYMPLPPATEITDVPKLQFSHVECLMYAFHKLCKQTPEFLIKDAEQLKEFRLRLQYFARGIQGYIKKLREAISGKTEEELKSEENQLKVVALKTTNNINTLIKDLFHSPPSFKSIIHLSWKTPCNDKKSDKNSAQKRHTPITFGNDNSPNKRNKEDKNSKREIYTPPSGKYSSTISNYGRGRFKGNRPRGRGGFRTRGRGSWRKNFY

>709

MPTVEELYRNYGILADATEQVGQHKDAYQVILDGVKGGTKEKRLAAQFIPKFFKHFPELADSAINAQLDLCEDEDVSIRRQAIKELPQFATGENLPRVADILTQLLQTDDSAEFNLVNNALLSIFKMDAKGTLGGLFSQILQGEDIVRERAIKFLSTKLKTLPDEVLTKEVEELILTESKKVLEDVTGEEFVLFMKILSGLKSLQTVSGRQQLVELVAEQADLEQTFNPSDPDCVDRLLQCTRQAVPLFSKNVHSTRFVTYFCEQVLPNLGSLTTPVEGLDIQLEVLKLLAEMSSFCGDMEKLETNLRKLFDKLLEYMPLPPEEAENGENAGNEEPKLQFSYVECLLYSFHQLGRKLPDFLTAKLNAEKLKDFKIRLQYFARGLQVYIRQLRLALQGKTGEALKTDENKIKVVALKITNNINVLIKDLFHIPPSYKSTVTLSWKPVQKVELGQKRASEDTTSGSPPKKSSAGRDARQIYNPPSGKYSSNLGNFNYERSLQGK

>710

MPTVEELYRNYGILADATEQVGQHKDAYQVILDGVKGGTKEKRLAAQFIPKFFKHFPELADSAINAQLDLCEDEDVSIRRQAIKELPQFATGENLPRVADILTQLLQTDDSAEFNLVNNALLSIFKMDAKGTLGGLFSQILQGEDIVRERAIKFLSTKLKTLPDEVLTKEVEELILTESKKVLEDVTGEEFVLFMKILSGLKSLQTVSGRQQLVELVAEQADLEQTFNPSDPDCVDRLLQCTRQAVPLFSKNVHSTRFVTYFCEQVLPNLGSLTTPVEGLDIQLEVLKLLAEMSSFCGDMEKLETNLRKLFDKLLEYMPLPPEEAENGENAGNEEPKLQFSYVECLLYSFHQLGRKLPDFLTAKLNAEKLKDFKIRLQYFARGLQVYIRQLRLALQGKTGEALKTDENKIKVVALKITNNINVLIKDLFHIPPSYKSTVTLSWKPVQKVELGQKRASEDTTSGSPPKKSSAGRDARQIYNPPSGKYSSNLGNFNYEQRGAFRGSRGGRGWGARGNRSRGRLY

>711

MPTVEELYRNYGILADATEQVGQHKDAYQVILDGVKGGTKEKRLAAQFIPKFFKHFPELADSAINAQLDLCEDEDVSIRRQAIKELPQFATGENLPRVADILTQLLQTDDSAEFNLVNNALLSIFKMDAKGTLGGLFSQILQGEDIVRERAIKFLSTKLKTLPDEVLTKEVEELILTESKKVLEDVTGEEFVLFMKILSGLKSLQTVSGRQQLVELVAEQADLEQTFNPSDPDCVDRLLQCTRQAVPLFSKNVHSTRFVTYFCEQVLPNLGSLTTPVEGLDIQLEVLKLLAEMSSFCGDMEKLETNLRKLFDKLLEYMPLPPEEAENGENAGNEEPKLQFSYVECLLYSFHQLGRKLPDFLTAKLNAEKLKDFKIRLQYFARGLQVYIRQLRLALQGKTGEALKTDENKIKVVALKITNNINVLIKDLFHIPPSYKSTVTLSWKPVQKVELGQKRASEDTTSGSPPKKSSAXXXXXXSAGPKRDARQIYNPPSGKYSSNLGNFNYERSLQGK

>712

MPTVEELYRNYGILADATEQVGQHKDAYQVILDGVKGGTKEKRLAAQFIPKFFKHFPELADSAINAQLDLCEDEDVSIRRQAIKELPQFATGENLPRVADILTQLLQTDDSAEFNLVNNALLSIFKMDAKGTLGGLFSQILQGEDIVRERAIKFLSTKLKTLPDEVLTKEVEELILTESKKVLEDVTGEEFVLFMKILSGLKSLQTVSGRQQLVELVAEQADLEQTFNPSDPDCVDRLLQCTRQAVPLFSKNVHSTRFVTYFCEQVLPNLGSLTTPVEGLDIQLEVLKLLAEMSSFCGDMEKLETNLRKLFDKLLEYMPLPPEEAENGENAGNEEPKLQFSYVECLLYSFHQLGRKLPDFLTAKLNAEKLKDFKIRLQYFARGLQVYIRQLRLALQGKTGEALKTDENKIKVVALKITNNINVLIKDLFHIPPSYKSTVTLSWKPVQKVELGQKRASEDTTSGSPPKKSSAXXXXXXSAGPKRDARQIYNPPSGKYSSNLGNFNYEQRGAFRGSRGGRGWGARGNRSRGRLY

>713

MPTVEELYRNYGILADATETAGQHKDAYQAILDGVKGGAKEKRLAAQFIPKFFKHFPELADSAINAQLDLCEDEDVSIRRQAIKELPQFATGDNLPRVADILTQLLQSDDSAEFNLVNNALLSIFKMDAKGTLGGLFSQILQGEDIVRERAIKFLSTKLKTLPEEVMTKEVEEFILTESKKVLEDVTGEEFVLFMKILSGLKSLQTVSGRQQLVELVAEQADLEQTFNPSDPDCVDRLLQCTRQAVPLFSKNVHSTKFVTYFCEHVLPNLSSLTTPVEGLDIQLEVLKLLAEMSSFCGDMEKLESNLKKLFDKLLEYMPLPPEEAENGENAGNEEPKLQFSYVECLLYSFHQLGRKLPDFLTAKLNAEKLKDFKIRLQYFARGLQVYIRQLRLALQGKTGEALKTEENKIKVVALKITNNINVLIKDLFHIPPSYKSTVTLSWKPVQKADASQKRASEDTTSSSPPKKPSAGPKRDARQIYNPPSGKYSSNLGSFSYEQRGGFRGGRGRGWGGRGNRSRGRIY

>714

MPTVEELYRNYGILADATETAGQHKDAYQAILDGVKGGAKEKRLAAQFIPKFFKHFPELADSAINAQLDLCEDEDVSIRRQAIKELPQFATGDNLPRVADILTQLLQSDDSAEFNLVNNALLSIFKMDAKGTLGGLFSQILQGEDIVRERAIKFLSTKLKTLPEEVMTKEVEEFILTESKKVLEDVTGEEFVLFMKILSGLKSLQTVSGRQQLVELVAEQADLEQTFNPSDPDCVDRLLQCTRQAVPLFSKNVHSTKFVTYFCEHVLPNLSSLTTPVEGLDIQLEVLKLLAEMSSFCGDMEKLESNLKKLFDKLLEYMPLPPEEAENGENAGNEEPKLQFSYVECLLYSFHQLGRKLPDFLTAKLNAEKLKDFKIRLQYFARGLQVYIRQLRLALQGKTGEALKTEENKIKVVALKITNNINVLIKDLFHIPPSYKSTVTLSWKPVQKADASQKRASEDTTSSSPPKKPSAGPKRDARQIYNPPSGKYSSNLGSFSYALAPQMSHWTDQLLQMAELGHLSNNWTCAEFLLQPLLMLQT

>715

MPTVEELYRNYGILADATEQVGQHKDAYQVILDGVKGGTKEKRLAAQFIPKFFKHFPELADSAINAQLDLCEDEDVSIRRQAIKELPQFATGENLPRVADILTQLLQTDDSAEFNLVNNALLSIFKMDAKGTLGGLFSQILQGEDIVRERAIKFLSTKLKTLPDEVLTKEVEELILTESKKVLEDVTGEEFVLFMKILSGLKSLQTVSGRQQLVELVAEQADLEQTFNPSDPDCVDRLLQCTRQAVPLFSKNVHSTRFVTYFCEQVLPNLSTLTTPVEGLDIQLEVLKLLAEMSSFCGDMEKLETNLRKLFDKLLEYMPLPPEEAENGENAGNEEPKLQFSYVECLLYSFHQLGRKLPDFLTAKLNAEKLKDFKIRLQYFARGLQVYIRQLRLALQGKTGEALKTEENKIKVVALKITNNINVLIKDLFHIPPSYKSTVTLSWKPVQKVEIGQKRASEDTTSGSPPKKSSAGPKRDARQIYNPPSGKYSSNLSNFNYERSLQGK

>716

MPTVEELYRNYGILADATEQVGQHKDAYQVILDGVKGGTKEKRLAAQFIPKFFKHFPELADSAINAQLDLCEDEDVSIRRQAIKELPQFATGENLPRVADILTQLLQTDDSAEFNLVNNALLSIFKMDAKGTLGGLFSQILQGEDIVRERAIKFLSTKLKTLPDEVLTKEVEELILTESKKVLEDVTGEEFVLFMKILSGLKSLQTVSGRQQLVELVAEQADLEQTFNPSDPDCVDRLLQCTRQAVPLFSKNVHSTRFVTYFCEQVLPNLSTLTTPVEGLDIQLEVLKLLAEMSSFCGDMEKLETNLRKLFDKLLEYMPLPPEEAENGENAGNEEPKLQFSYVECLLYSFHQLGRKLPDFLTAKLNAEKLKDFKIRLQYFARGLQVYIRQLRLALQGKTGEALKTEENKIKVVALKITNNINVLIKDLFHIPPSYKSTVTLSWKPVQKVEIGQKRASEDTTSGSPPKKSSAGPKRDARQIYNPPSGKYSSNLSNFNYEQRGAFRGSRGGRGWGTRGNRSRGRLY

>717

MESQSLELKEIFTVSINRSFKNYVLSQIKLAALIDVKDALVIHSSGTVGGRGNLYLRDNKQADRRQKLLEKLPKFSEGDHHKDAYQAILDGVKGGAKEKRLAAQFIPKFFKHFPELADSAINAQLDLCEDEDVSIRRQAIKELPQFATGDNLPRVADILTQLLQSDDSAEFNLVNNALLSIFKMDAKGTLGGLFSQILQGEDIVRERAIKFLSTKLKTLPEEVMTKEVEEFILTESKKVLEDVTGEEFVLFMKILSGLKSLQTVSGRQQLVELVAEQADLEQTFNPSDPDCVDRLLQCTRQAVPLFSKNVHSTKFVTYFCEHVLPNLSSLTTPVEGLDIQLEVLKLLAEMSSFCGDMEKLESNLKKLFDKLLEYMPLPPEEAENGENAGSEEPKLQFSYVECLLYSFHQLGRKLPDFLTAKLNAEKLKDFKIRLQYFARGLQVYIRQLRLALQGKTGEALKTEENKIKVVALKITNNINVLIKDLFHIPPSYKSTVTLSWKPVQKADASQKRASEDTTSSSPPKKASAXXXXXXXXXXRQIYNPPSGKYSSNLGSFSYEQRGGFRGGRGRGWGGRGNRSRGRIY

>718

MKHKDAYQAILDGVKGGAKEKRLAAQFIPKFFKHFPELADSAINAQLDLCEDEDVSIRRQAIKELPQFATGDNLPRVADILTQLLQSDDSAEFNLVNNALLSIFKMDAKGTLGGLFSQILQGEDIVRERAIKFLSTKLKTLPEEVMTKEVEEFILTESKKVLEDVTGEEFVLFMKILSGLKSLQTVSGRQQLVELVAEQADLEQTFNPSDPDCVDRLLQCTRQAVPLFSKNVHSTKFVTYFCEHVLPNLSSLTTPVEGLDIQLEVLKLLAEMSSFCGDMEKLESNLKKLFDKLLEYMPLPPEEAENGENAGNEEPKLQFSYVECLLYSFHQLGRKLPDFLTAKLNAEKLKDFKIRLQYFARGLQVYIRQLRLALQGKTGEALKTEENKIKVVALKITNNINVLIKDLFHIPPSYKSTVTLSWKPVQKADASQKRASEDTTSSSPPKKPSAGPKRDARQIYNPPSGKYSSNLGSFSYGKAKRWFPGWTRKRLGRTWQS

>719

MKHKDAYQAILDGVKGGAKEKRLAAQFIPKFFKHFPELADSAINAQLDLCEDEDVSIRRQAIKELPQFATGDNLPRVADILTQLLQSDDSAEFNLVNNALLSIFKMDAKGTLGGLFSQILQGEDIVRERAIKFLSTKLKTLPEEVMTKEVEEFILTESKKVLEDVTGEEFVLFMKILSGLKSLQTVSGRQQLVELVAEQADLEQTFNPSDPDCVDRLLQCTRQAVPLFSKNVHSTKFVTYFCEHVLPNLSSLTTPVEGLDIQLEVLKLLAEMSSFCGDMEKLESNLKKLFDKLLEYMPLPPEEAENGENAGNEEPKLQFSYVECLLYSFHQLGRKLPDFLTAKLNAEKLKDFKIRLQYFARGLQVYIRQLRLALQGKTGEALKTEENKIKVVALKITNNINVLIKDLFHIPPSYKSTVTLSWKPVQKADASQKRASEDTTSSSPPKKPSAGPKRDARQIYNPPSGKYSSNLGSFSYEQRGGFRGGRGRGWGGRGNRSRGRIY

>720

MKHKDAYQAILDGVKGGAKEKRLAAQFIPKFFKHFPELADSAINAQLDLCEDEDVSIRRQAIKELPQFATGDNLPRVADILTQLLQSDDSAEFNLVNNALLSIFKMDAKGTLGGLFSQILQGEDIVRERAIKFLSTKLKTLPEEVMTKEVEEFILTESKKVLEDVTGEEFVLFMKILSGLKSLQTVSGRQQLVELVAEQADLEQTFNPSDPDCVDRLLQCTRQAVPLFSKNVHSTKFVTYFCEHVLPNLSSLTTPVEGLDIQLEVLKLLAEMSSFCGDMEKLESNLKKLFDKLLEYMPLPPEEAENGENAGNEEPKLQFSYVECLLYSFHQLGRKLPDFLTAKLNAEKLKDFKIRLQYFARGLQVYIRQLRLALQGKTGEALKTEENKIKVVALKITNNINVLIKDLFHIPPSYKSTVTLSWKPVQKADASQKRASEDTTSSSPPKKPSAGPKRDARQIYNPPSGKYSSNLGSFSYALAPQMSHWTDQLLQMAELGHLSNNWTCAEFLLQPLLMLQT

>721

MPPEELLAPPDNPRGCSRCRFPQDPGRVEIRSSESGNTETYQQGVGSVQGALHKDAYQAILDGVKGGAKEKRLAAQFIPKFFKHFPELADSAINAQLDLCEDEDVSIRRQAIKELPQFATGDNLPRVADILTQLLQSDDSAEFNLVNNALLSIFKMDAKGTLGGLFSQILQGEDIVRERAIKFLSTKLKTLPEEVMTKEVEEFILTESKKVLEDVTGEEFVLFMKILSGLKSLQTVSGRQQLVELVAEQADLEQTFNPSDPDCVDRLLQCTRQAVPLFSKNVHSTKFVTYFCEHVLPNLSSLTTPVEGLDIQLEVLKLLAEMSSFCGDMEKLESNLKKLFDKLLEYMPLPPEEAENGENAGNEEPKLQFSYVECLLYSFHQLGRKLPDFLTAKLNAEKLKDFKIRLQYFARGLQVYIRQLRLALQGKTGEALKTEENKIKVVALKITNNINVLIKDLFHIPPSYKSTVTLSWKPVQKADASQKRASEDTTSSSPXFLGPKRDARQIYNPPSGKYSSNLGSFSYEQRGGFRGGRGRGWGGRGNRSRGRIY

>722

ARMEGRSSRLKEICKILARSAHKDAYQAILDGVKGGAKEKRLAAQFIPKFFKHFPELADSAINAQLDLCEDEDVSIRRQAIKELPQFATGDNLPRVADILTQLLQSDDSAEFNLVNNALLSIFKMDAKGTLGGLFSQILQGEDIVRERAIKFLSTKLKTLPEEVMTKEVEEFILTESKKVLEDVTGEEFVLFMKILSGLKSLQTVSGRQQLVELVAEQADLEQTFNPSDPDCVDRLLQCHRQAPEXCAVVFLQKNVHSTKFVTYFCEHVLPNLSSLTTPVEGLDIQLEVLKLLAEMSSFCGDMEKLESNLKKLFDKLLEYMPLPPEEAENGENAGNEEPKLQFSYVECLLYSFHQLGRKLPDFLTAKLNAEKLKDFKIRLQYFARGLQVYIRQLRLALQGKTGEALKTEENKIKVVALKITNNINVLIKDLFHIPPSYKSTVTLSWKPVQKADASQKRASEDTTSSSPPKKASAGPKRDARQIYNPPSGKYSSNLGSFSYEQRGGFRGGRGRGWGGRGNRSRGRIY

>723

HKDAYQAILDGVKGGAKEKRLAAQFIPKFFKHFPELADSAINAQLDLCEDEDVSIRRQAIKELPQFATGDNLPRVADILTQLLQSDDSAEFNLVNNALLSIFKMDAKGTLGGLFSQILQGEDIVRERAIKFLSTKLKTLPEEVMTKEVEEFILTESKKVLEDVTGEEFVLFMKILSGLKSLQTVSGRQQLVELVAEQADLEQTFNPSDPDCVDRLLQCTRQAVPLFSKNVHSTKFVTYFCEHVLPNLSSLTTPVEGLDIQLEVLKLLAEMSSFCGDMEKLESNLKKLFDKLLEYMPLPPEEAENGENAGSEEPKLQFSYVECLLYSFHQLGRKLPDFLTAKLNAEKLKDFKIRLQYFARGLQVYIRQLRLALQGKTGEALKTEENKIKVVALKITNNINVLIKDLFHIPPSYKSTVTLSWKPVQKADASQKRASEDTTSSSPPKKASAXXXXXXXXGPKRDARQIYNPPSGKYSSNLGSFSYEQRGGFRGGRGRGWGGRGNRSRGRIY

>724

MPTVEELYRNYGILADATEQVGQHKDAYQVILDGVKGGTKEKRLAAQFIPKFFKHFPELADSAINAQLDLCEDEDVSIRRQAIKELPQFATGENLPRVADILTQLLQTDDSAEFNLVNNALLSIFKMDAKGTLGGLFSQILQGEDIVRERAIKFLSTKLKTLPDEVLTKEVEELILTESKKVLEDVTGEEFVLFMKILSGLKSLQTVSGRQQLVELVAEQADLEQTFNPSDPDCVDRLLQCTRQAVPLFSKNVHSTRFVTYFCEQVLPNLSTLTTPVEGLDIQLEVLKLLAEMSSFCGDMEKLETNLRKLFDKLLEYMPLPPEEAENGENAGNEEPKLQFSYVECLLYSFHQLGRKLPDFLTAKLNAEKLKDFKIRLQYFARGLQVYIRQLRLALQGKTGEALKTEENKIKVVALKITNNINVLIKDLFHIPPSYKSTVTLSWKPVQKVEIGQKRASEDTTSGSPPKKSTAGPKRDARQIYNPPSGKYSSNLSSFNYERSLQGK

>725

MPTVEELYRNYGILADATEQVGQHKDAYQVILDGVKGGTKEKRLAAQFIPKFFKHFPELADSAINAQLDLCEDEDVSIRRQAIKELPQFATGENLPRVADILTQLLQTDDSAEFNLVNNALLSIFKMDAKGGLFSQILQGEDIVRERAIKFLSTKLKTLPDEVLTKEVEELILTESKKVLEDVTGEEFVLFMKILSGLKSLQTVSGRQQLVELVAEQADLEQTFNPSDPDCVDRLLQCTRQAVPLFSKNVHSTRFVTYFCEQVLPNLSTLTTPVEGLDIQLEVLKLLAEMSSFCGDMEKLETNLRKLFDKLLEYMPLPPEEAENGENAGNEEPKLQFSYVECLLYSFHQLGRKLPDFLTAKLNAEKLKDFKIRLQYFARGLQVYIRQLRLALQGKTGEALKTEENKIKVVALKITNNINVLIKDLFHIPPSYKSTVTLSWKPVQKVEIGQKRASEDTTSGSPPKKSTAGPKRDARQIYNPPSGKYSSNLSSFNYEQRGAFRGSRGGRGWGARGNRSRGRLY

>726

MPTVEELYRNYGILADATEQVGQHKDAYQVILDGVKGGTKEKRLAAQFIPKFFKHFPELADSAINAQLDLCEDEDVSIRRQAIKELPQFATGENLPRVADILTQLLQTDDSAEFNLVNNALLSIFKMDAKGTLGGLFSQILQGEDIVRERAIKFLSTKLKTLPDEVLTKEVEELILTESKKVLEDVTGEEFVLFMKILSGLKSLQTVSGRQQLVELVAEQADLEQTFNPSDPDCVDRLLQCTRQAVPLFSKNVHSTRFVTYFCEQVLPNLSTLTTPVEGLDIQLEVLKLLAEMSSFCGDMEKLETNLRKLFDKLLEYMPLPPEEAENGENAGNEEPKLQFSYVECLLYSFHQLGRKLPDFLTAKLNAEKLKDFKIRLQYFARGLQVYIRQLRLALQGKTGEALKTEENKIKVVALKITNNINVLIKDLFHIPPSYKSTVTLSWKPVQKVEIGQKRASEDTTSGSPPKKSTAGPKRDARQIYNPPSGKYSSNLSSFNYEQRGAFRGSRGGRGWGARGNRSRGRLY

>727

MKHKDAYQAILDGVKGGAKEKRLAAQFIPKFFKHFPELADSAINAQLDLCEDEDVSIRRQAIKELPQFATGDNLPRVADILTQLLQSDDSAEFNLVNNALLSIFKMDAKGTLGGLFSQILQGEDIVRERAIKFLSTKLKTLPEEVMTKEVEEFILTESKKVLEDVTGEEFVLFMKILSGLKSLQTVSGRQQLVELVAEQADLEQTFNPADPDCVDRLLQCTRQAVPLFSKNVHSTKFVTYFCEHVLPNLSSLTTPVEGLDIQLEVLKLLAEMSSFCGDMEKLESNLKKLFDKLLEYMPLPPEEAENGENAGNEEPKLQFSYVECLLYSFHQLGRKLPDFLTAKLNAEKLKDFKIRLQYFARGLQVYIRQLRLALQGKTGEALKTEENKIKVVALKITNNINVLIKDLFHIPPSYKSTVTLSWKPVQKADPSQKRASEDTTSSSPPKKASAXXXXXXXARQIYNPPSGKYSSNLGSFSYEQRGGFRGGRGRGWGGRGNRSRGRIY

>728

MPTVEELYRNYGILADATEQVGQHKDSYQVILDGVKGGTKEKRLAAQFIPKFFKHFPELADSAINAQLDLCEDEDVSIRRQAIKELPQFATGENLPRVADILTQLLQTDDSAEFNLVNNALLSIFKMDAKETNLRKLFDKLLEYMPLPPEEAENGENAGNEEPKLQFSYVECLLYSFHQLGRKLPDFLTAKLNAEKLKDFKIRLQYFARGLQVYIRQLRLALQGKTGEALKTEENKIKVVALKITNNINVLIKDLFHIPPSYKSTVTLSWKPVQKVELGQKRANEDTTSGSPPKKSTAGPKRDARQIYNPPSGKYSSNLGNFNYERSLQGK

>729

MPTVEELYRNYGILADATEQVGQIRRQAIKELPQFATGENLPRVADILTQLLQTDDSAEFNLVNNALLSIFKMDAKGTLGGLFSQILQGEDIVRERAIKFLSTKLKTLPEEVLTKEVEELILTESKKVLEDVTGEEFVLFMKILSGLKSLQTVSGRQQLVELVAEQADLEQTFNPSDPDCVDRLLQCTRQAVPLFSKNVHSTRFVTYFCEQVLPNLSSLTTPVEGLDIQLEVLKLLAEMSSFCGDMEKLETNLRKLFDKLLEYMPLPPEEAENGENAGNEEPKLQFSYVECLLYSFHQLGRKLPDFLTAKLNAEKLKDFKIRLQYFARGLQVYIRQLRLALQGKTGEALKTEENKIKVVALKITNNINVLIKDLFHIPPSYKSTVTLSWKPVQKVELGQKRANEDTTSGSPPKKSTAGPKRDARQIYNPPSGKYSSNLGNFNYERSLQGK

>730

MPTVEELYRNYGILADATEQVGQHKDSYQVILDGVKGGTKEKRLAAQFIPKFFKHFPELADSAINAQLDLCEDEDVSIRRQAIKELPQFATGENLPRVADILTQLLQTDDSAEFNLVNNALLSIFKMDAKGTLGGLFSQILQGEDIVRERAIKFLSTKLKTLPEEVLTKEVEELILTESKKVLEDVTGEEFVLFMKILSGLKSLQTVSGRQQLVELVAEQADLEQTFNPSDPDCVDRLLQCTRQAVPLFSKNVHSTRFVTYFCEQVLPNLSSLTTPVEGLDIQLEVLKLLAEMSSFCGDMEKLETNLRKLFDKLLEYMPLPPEEAENGENAGNEEPKLQFSYVECLLYSFHQLGRKLPDFLTAKLNAEKLKDFKIRLQYFARGLQVYIRQLRLALQGKTGEALKTEENKIKVVALKITNNINVLIKDLFHIPPSYKSTVTLSWKPVQKVELGQKRANEDTTSGSPPKKSTAGPKRDARQIYNPPSGKYSSNLGNFNYERSLQGK

>731

MPTVEELYRNYGILADATEQVGQHKDSYQVILDGVKGGTKEKRLAAQFIPKFFKHFPELADSAINAQLDLCEDEDVSIRRQAIKELPQFATGENLPRVADILTQLLQTDDSAEFNLVNNALLSIFKMDAKGTLGGLFSQILQGEDIVRERAIKFLSTKLKTLPEEVLTKEVEELILTESKKVLEDVTGEEFVLFMKILSGLKSLQTVSGRQQLVELVAEQADLEQTFNPSDPDCVDRLLQCTRQAVPLFSKNVHSTRFVTYFCEQVLPNLSSLTTPVEGLDIQLEVLKLLAEMSSFCGDMEKLETNLRKLFDKLLEYMPLPPEEAENGENAGNEEPKLQFSYVECLLYSFHQLGRKLPDFLTAKLNAEKLKDFKIRLQYFARGLQVYIRQLRLALQGKTGEALKTEENKIKVVALKITNNINVLIKDLFHIPPSYKSTVTLSWKPVQKVELGQKRANEDTTSGSPPKKSTAGPKRDARQIYNPPSGKYSSNLGNFNYEQRGAFRGSRGGRGWGARGNRSRGRLY

>732

MPTVEELYRNYGILADATEQVGQHKDAYQVILDGVKGGTKEKRLAAQFIPKFFKHFPELADSAINAQLDLCEDEDVSIRRQAIKELPQFATGENLPRVADILTQLLQTDDSAEFNLVNNALLSIFKMEKLETNLRKLFDKLLEYMPLPPEEAENGENAGNEEPKLQFSYVECLLYSFHQLGRKLPDFLTAKLNAEKLKDFKIRLQYFARGLQVYIRQLRLALQGKTGEALKTDENKIKVVALKITNNINVLIKDLFHIPPSYKSTVTLSWKPVQKVEFGQKRASEDTTSGSPPKKSSAGPKRDARQIYNPPSGKYSSNLSNFNYERSLQGK

>733

MPTVEELYRNYGILADATEQVGQIRRQAIKELPQFATGENLPRVADILTQLLQTDDSAEFNLVNNALLSIFKMDAKGTLGGLFSQILQGEDIVRERAIKFLSTKLKTLPDEVLTKEVEELILTESKKVLEDVTGEEFVLFMKILSGLKSLQTVSGRQQLVELVAEQADLEQTFNPSDPDCVDRLLQCTRQAVPLFSKNVHSTRFVTYFCEQVLPNLSSLTTPVEGLDIQLEVLKLLAEMSSFCGDMEKLETNLRKLFDKLLEYMPLPPEEAENGENAGNEEPKLQFSYVECLLYSFHQLGRKLPDFLTAKLNAEKLKDFKIRLQYFARGLQVYIRQLRLALQGKTGEALKTDENKIKVVALKITNNINVLIKDLFHIPPSYKSTVTLSWKPVQKVEFGQKRASEDTTSGSPPKKSSAGPKRDARQIYNPPSGKYSSNLSNFNYERSLQGK

>734

MPTVEELYRNYGILADATEQVGQHKDAYQVILDGVKGGTKEKRLAAQFIPKFFKHFPELADSAINAQLDLCEDEDVSIRRQAIKELPQFATGENLPRVADILTQLLQTDDSAEFNLVNNALLSIFKMDAKGTLGGLFSQILQGEDIVRERAIKFLSTKLKTLPDEVLTKEVEELILTESKKVLEDVTGEEFVLFMKILSGLKSLQTVSGRQQLVELVAEQADLEQTFNPSDPDCVDRLLQCTRQAVPLFSKNVHSTRFVTYFCEQVLPNLSSLTTPVEGLDIQLEVLKLLAEMSSFCGDMEKLETNLRKLFDKLLEYMPLPPEEAENGENAGNEEPKLQFSYVECLLYSFHQLGRKLPDFLTAKLNAEKLKDFKIRLQYFARGLQVYIRQLRLALQGKTGEALKTDENKIKVVALKITNNINVLIKDLFHIPPSYKSTVTLSWKPVQKVEFGQKRASEDTTSGSPPKKSSAGPKRDARQIYNPPSGKYSSNLSNFNYERSLQGK

>735

MPTVEELYRNYGILADATEQVGQHKDAYQVILDGVKGGTKEKRLAAQFIPKFFKHFPELADSAINAQLDLCEDEDVSIRRQAIKELPQFATGENLPRVADILTQLLQTDDSAEFNLVNNALLSIFKMDAKGTLGGLFSQILQGEDIVRERAIKFLSTKLKTLPDEVLTKEVEELILTESKKVLEDVTGEEFVLFMKILSGLKSLQTVSGRQQLVELVAEQADLEQTFNPSDPDCVDRLLQCTRQAVPLFSKNVHSTRFVTYFCEQVLPNLSSLTTPVEGLDIQLEVLKLLAEMSSFCGDMEKLETNLRKLFDKLLEYMPLPPEEAENGENAGNEEPKLQFSYVECLLYSFHQLGRKLPDFLTAKLNAEKLKDFKIRLQYFARGLQVYIRQLRLALQGKTGEALKTDENKIKVVALKITNNINVLIKDLFHIPPSYKSTVTLSWKPVQKVEFGQKRASEDTTSGSPPKKSSAGPKRDARQIYNPPSGKYSSNLSNFNYEQRGAFRGNRGGRGWGARGNRSRGRLY

>736

MPTVEELYRNYGILADAKDDVGQHKSAYQVILDGVKGGPKEKRLAAQFIPKFFKHFPDLSDAALNAQLDLCEDEDVSIRRQAIKELSQFATGENLPRVADILTQLLQSDDSAEFNLVNNALLSIFKMDAKGTLGGLFSQILQGEDVVRERAIKFLATKMKTLPEDILTKEVDDYIFSESKKVLYDVTGEEFVLFMKILSALKNLQTVSGRQQLVDLVSEQAGLHQTLNPADPDSVDRLLQCMRQAVPLFSKNVHSTKFVTYFCEQVLPILSSLTSPAEGIDVQLEVLKLLAEMSSFCGDMDKLESNLNKLFDKLLEFMPLPPEEVENGDSAANEEPKLQFSYVECLLFSFHQLGRKLPDFLIAKVDAEKLKDFKIRLQYFARGLQVYIRQLRLTLQGKSGDALKTEENKIKVVALKITNNINVLIKDLFHNPPSYKSTVTLSWKPVQKTDSGQKRMSDETSSTSPPKKPVVGPKRDSRQIYNPPSGKYSGNVGAFSYEQRGGFQGGRGRGWGGRGNRSRGRIY

>737

MATVEELYRSYGILADAKDDVGQHKSAYQVIIDGVKGGAKEKRLAAQFIPKFFKHFPDLSDSALNAQLDLCEDEDVSIRRQAIKELSQFATGENLPRVADILTQLLQSDDSAEFNLVNNALLSIFKMDAKGTLGGLFSQILQGEDIVRERAIKFLATKMKTLPEETLTKEVDDYIFSESKKVLYDVTGEEFVLFMKILSALKNLQTVSGRQQLVDLVSEQAGLHQTLNPADPDSVDRLLQCMRQAVPLFSKNVHSTKFVTYFCEQVLPILSTLTSPAESIDVQLEVLKLLAEMSSFCGDMDKLESNLNKLFDKLLEFMPLPPEEVENGDTAANEEPKLQFSYVECLLFSFHQLGRKLPDFLIAKVDAEKLKDFKIRLQYFARGLQVYIRQLRLALQGKSGDALKTEENKIKVVALKITNNINVLIKDLFHNPPSYKSTVTLSWKPVQRTPDSGQKRTSDETSSTSPPKKPIVGPKRDSRQIYNPPSGKYSASVGAFSYEQRGGFQGGRGRGWGGRGNRSRGRIY

>738

MDNIERLYKCYEILSEAGDKISEHVDEYKEILKAVKGTSKEKRLASQFIGNFFKHFPDLADTAIDAQFDLCEDDDTQIRRQAIKDLPKLCQGNADATIRVGDTLAQLLILDDPTELQQVNNSLLAIIKLDTKSSIAGLFQQISTGDETTRERCLKFIATKLLTMGPTVITKEIEDYIVEEIKKALQDVTADEFHLCMTILGATKLGSTITGHAELVKLATEQAELNNTDADIIAVDDEVVERFIQCASAAAPYFSKTIKSTAFVAHVCDKLLPIKTWNMIATAVSQDQIQLRLLKVFAEMITNTDKLDNASERINAVYNVLLEYMPLPKLSDEDLGDTPPSFQFSHAECLLYALHTLGKNHPNSLSFVEDAEKLKDFRARLQYLARGTQGYIKKLEESLKGKTGEELKTEENQLKQTALKTTSNINILIRDLFHSPPIFKHDIVLSWIVPKNNKLGKRHAPITFGEKAAANGKDKDQEPEKKSRPSNDQKFYSPPSGKYSNKVNQSYGNNNRTRQRGGGGGGGSGGGYRNRRFNKY

>739

MLSRFWTFIISVAFWVFPYSKFVNAASLTSLFKHIRGVEEPSTEPSTDDVIREKVINFVRDKVFPIKAELLKPQEEMERHITDLIKTSLDDVTGVEFRMFMDFLKSLSLFGEKCPHERIKELIGVVEEQAELELEFKVADADHNDRLISCLHMALPIVVRGASSSNFMNFINKKIIPVFDQLPGERKVDLLRSLAEFSPFTTPQDSRQMLPSIVQLLKKYMTWKKTGEEMNFTYVECLLYTFHHLAHKVPNATNSLCGYKIVTGQPSDRLGEDFSEQYNDFTERLVQLVRSWLMLRKGVGSTPVVGVRTSLVGDMLKNVEEFTRATIKKLTQGMSENNKSLADAKTDEEKEKIKTKKQNATTGLRTCNNILTMTKPLHAKAPSFIGDKRINLSWKEATKTASTTTPAVGAKRPATAGNGSNNIALKKGRGSGAMQNQLVNRAIGGLSGAGRGGVSGGGRGRVSGGGRGGTRGRGWGGRGGRGSGRGRGRGYW

>740

MTDPSEEATIEKLYQYGEQLNSAKDKSQNVKDYQGIIAVAKTSVKAKQLSAQLIPKFFKFFPEQAGAALETHLDLVEADELGVCH

>741

VRVQAIRGLPLFCKDTPENIGKMVDILVQLLGTEEFVERDAVHKALMSLLRQDVKGSSEA

>1386

MESLPHAVTKIFGGSPGSGASTLHLGGRNEAVLQHKQSVDMEAADEMYGPRSDCFVFEAYPEVKKAFIRRVYQILVAQLVLTAGVIYAIRSLYNIDNTISFSGDQDATPISWQRWRSRGAALSNLFWSGFLGSMVTLTMLHFVARRHPHNLAVLFAFTFFESLLLSSALVFVPAGLLFRALLTTTAVFIGLILYTLESKADYSFLRSYLGSALSIIVVAGFFQLFWPMGSAMDTVYTWFGALVFCGFIIYDTWRLHFQLKPDEYVLAAASLYLDFINLFLRVLHLLSKKK

>1387

MRRTFAFCSSSPHFAKVASNGLALKRAARPLITLSPAATGCMRPPLTCTASSSSSSSSSFLLAHLKSSAIRPSSSSGRRQLFTATSNRTAAKTTSTTPLLASGGHRFGAATKTAIRSIRIEGRPPRSYNGLMGDVGVVAPRDNTAWLLIGGLAALGLGGIALAGYVNQETTTIPTHGDLTTHRLRATYGYMLGGLATTAASAAVLFRAGAAHRVVAMNPWLFMGASLLGTIGSLMVMQSLPPENTVARHLAWGVFNACNGLALSPVAMLGGAVVTKALVATGAVVGSLSLVAAAAPSESFLWMGGSLGVGLGVVIAASMGQMFFPASAFLTGITLYGGLGLFGMMMLYDTQRVMHKAKTDEVYDPMTQSIAIYLNTINIFVRMATIMANNQGNRRR

>1388

MTTFTDRKINWDAVKDFSPINQRLKQHLVRVYSTLAATLAMSAIGAWAHVAYNLGGLISFFATIGLIAMLHLTPAFDENVPKRTLFLFGIGFFQGCSIGPLLAYTLELDPLILVNAFVGTFAIFGCFSASALFAQRRSMLYLGAFLSSGLSLLFWVSIANIFLGTALLSNVTLYLGLLVFCGFVMFDTQLVIEKFHMGNQDYLAHSLELFLDFINIFVRLVVILSKDKKKNSRK

>1389

MTDFFSASTSMLETPVSAVLSFAPLSPVARQHVTGAYTTLAWMTAITAATSYGVLTGWLPALHPLIPFIGMMVALGVFRMSSRTKDALTTRRAVLAGFAALEGASLVGLLELVQLVSPRILPQAVVSTLLVFTSFSVAAMTSTRRSMIYLFGFLGSALSILAWMSFANVFMSSRLLFNGELYLGLLVFAGYVMFDTQVMLARVPALAVMDRVGREHAHMDSAIELYLDLINVFVRIAIIMAKNAAKGQARRNGRGDDSDDDDRRSSRRAGTRRR

>1390

MTDFFSVSTSILETPVSALLSFAPLSPIARQHVTGAYTTLAWMTAITAATSYGVLTGWLPALHPLVPFFGMMVALGVFRMSSRTKDALTTRRAALAGFAALEGASLVGLFELVQLVSPRILPQAVVSTLLVFTSFSVAAMTSTRRSMIYLFGFLGSALSILAWMSFANVFMNSRLLFNGELYLGLLVFAGYVMFDTQVMLARVPALAVMDRVRREHAHMDSAIELYLDLVNVFSMFDTSSCSARVPGARCEWTASAREHAPMASAMKLYPDLVNVFLRIAIIMAKNAAKDQARRNGGGDDSDDDDPRLSRRAGTRRR

>1391

LFGVCAAVGFVTPLALSFAPSLRRDPSSSLALFSLFAFAESAVVGVAASAYKLQSVLLALLQTGAATGALTAYAFQPNAKYDLTQVGSALLAGLMVLTVSTVAGVLLKVPMNSLAGSTVGALLFSAFIVHDTQLVVGGKKRQLNTSDYVLGAITLYLDIINLFFYLLRLFG

>1392

ADAAVHKALRLGFIRKVYGILTVQLALTAAVASALTLVDSARDFVLGTPSLLWVGMFASVGVLVALMLYKDRHPLNAQLLLAW

>1393

MAKLHESTSSASASFDPEKSDYDDTYVLTETTPTYIRHGFVRKVFAILFAQLLVTLGFSLICYFYRESVHSFISKNIWIFPTLAILSFITSLILIFSPSLSRRYPLNYAILVIETLYFSFIVGLSCAFTKSPTAIVLSVSITLGIILLVVLFTLQTKIDFTRYIIYFILFSFVTLVFGFIGIFVPFDTPLRMFYYGLGVLGYSLWMVLDLQLIIGGKTYEWTVDDYVPASLSLYTDVIGIFLNVHGMFSDR

>1394

MIGTRNRHMSFGTILDFTPLNSKQKSHLSRVYGTLGLSCLITAATVLYNLYMPQILAFILFTASFSYLFYLSSGKDLSGKRLIALVLLSVSEGVMIKDIVRYAQVLNGEIVTSALAISMAIFFCFSLSSIFATQRLTYYFYSLVSSLMTLISIVSFANIFMRSKIMLNATAIAALLMYAGFVAVDTQITLAEFDAGNRDFVVHAISLYVDIVAIFIRILQILIEKQHNECRKHDKDYD

>1395

MIVLAVFTIVEALLLASTTSLYDPSIVYMAMVGTAVIVVCLILYASSTERDFTGYYPYLWVFCLSLCLFGIFCIWSPFTYAIYSALAIFLFSAYIVCDTQLIVGGKGRAELGVDDYVFGALVLYLDIINLFLYLLSFLSATINRDCGVCYYQTIKQTTQ

>1396

MGRYLLRNMIPVSAIYPILLVGTIGIKKHPGDWIILIIFTIFTSMVLGVGCMFVKDNTLIMAVVVTLAVVFLLVIYALTTKTDFTGCGPYLFCTLSILVLCGIFGLTRLLDPNVYAYIGIALFSLYIVYDVQMCASTKLGAQYSVDDYVLASLNMWECV

>1397

MLGKVLFIVLLQLVITTLIVWLICIIEPLRVAFKRVRFPVLIVTAVGTFLIPLIMKCVKAYPADIILLSIFTLLEALILASWAMWVDYHVIVTAFGLTAGITVFLLLFAFISKSDFSGIEPYLYQIFFIAIFFGGMTILNSNWVSIFSAISMSAFCIYLVADLKHLARGKGFEKGGKTEMYALDALKVYMDVIGLYTLIFKCCKKNRSLSPVC

>1398

MLATFAISFFFMFYQPANDFVMQNQGVVFYGSFFVSIGTLLALMCFKAKYPTNVYLLAAFTAAMSCQVGVICTMYANAGLGEVVLQATVYTAAIFGALTIYAFTTRIDVRSWGPYLFVGLIVLMLWGFGAMLFGFKTNWIYSLGGALLFSAYILYDTSRLLHTYGADENWVIFTIDLYLDIINLFLFILQLLSKRD

>1399

MHYTGPSFNLKTILKTAKVSDHTQKHLTKVYSLLMATVVAAFIGAWICMNMVTLSPNFCTIGSVAGLIIIMAMGCTQKRNTRLRVSLMILFGFTEGICLSPLLKMANVVDERIIATAFLGTATIFLCFSMAALTSKRRSFLYLGGILGSTLMLLFFSSLLNIFFNSTFLFSIELYVGLAMFMGYVLFDTQVIIEAAEKGNDDFSWDAMKLFIDLVGIFVRILIILIKLNSKKGKSSSSPLDAIV

>1400

MLRLQSIALSPALRLRSRARLFSSLRKAASVGAARVRCVGRRAYRSATASSATASEGMSPVQKAGWTLVGTGALAGIGFVSFQGQYLYSETAQNDELRKRAGIPYPPYMKQRLEETYGYIVSGLGITGATAALALRSGVAHRIASLGTFGSLLVMGLCGMAPMMVCMSIDENANGGASKGTKQMAWLASMAGMGVMLSPVGFLGGQIILRAAVGTGLMVGSIASVAMTAPSESFLWMAAPLNMGLGVVVLSSFGSFFFPASPILHNIVLYGGLGLFGGYVLYDTQKLQYNAQHKKARDNFDDDDDDDDDDCGDGLLPHERGVRAVLGHNQYLYPTRTDIGDAARWWQASVNKRRRAEAQEGRHSS

>1401

MSTFGARTSPQWTMTTMMKNSGITEDVQQHLVRVYATLAACVLSAMLSSAVTLMFGPERFAFVGSSFAATLGSIWLYMEPTQNFKRRFAILMAIAASMGLTVSTLVAVVIQVDSSILVSALLLTTLVFLCFTASALIATRRSYLYLGGILSSGLSVVFLTSVIGIFKYSTFLFNLNLYGGLFLFCGYVVYDTQLIIEKASMGDKDVLAHTLHLFMDLVSIFVRVLAALLKGKKGGAPPRNYNRRT

>1402

MSSYTELPQSAPPYSEGESGSPPRQFGDNIPDDFKYSVSVASCELPLRQLFIRKVYSLLTIQLMGSVIMGFIIRSSDSFKIWAMTNTWLLILSFIGSIGFMIGAFFKARSYPINLILLGGFTICESYTLGVACAFIESGILIEAILLTLIIFIGLTIFAFQTKYDFISWQGTVGMMLWGLIGWGFVMMFIPHQQNSMMENVYSFLGALIFSIYIIIDTQHIMKTLHLDDEIIGCISLYLDIINLFLFILRILNNNRDD

>1403

MSSDKTPLTPQYSQQQPSYPPPQQYAYPPPSYPAPQQQQQQQQAPQREASYMSSSSSDHQAYDISAAPPSYASATASSDVYHSNDPDLQEWKGIAAFDDSIVRRGFIRKVYSILTLQLLVALGFIALFLFNSSVKHYVQRNQAMLITAIILTFVLILAMACVEKIRRQTPYNYIFLGLFTLAESYLLGVTASYYDVDAVLIAVGITAFVTFGLTLFAFQTKWDFTGYGGYLFGALLVLICFGFMCIFIRGEIVRIVYAALGALIFSMYLVYDTQLMLGGTHKLALSPEEWVFAALNLYLDIINLFLFILSLVGNRR

>1404

MTSLFDRPLPFEAFGQFHNIDAKVQSHLKGVYATLAASVLIAALGATLGQRFAFAFEYPYLVSFACLGFVLWLAMNPEGSSTFGTRAAMFGGYSFFQGVALAPLVWLLAEIDPSIVSTAFLGSVAIFASFSLTALYAQRRVDLWLGGLLSSGLSVLCWTNLLAFFFPSMFFFNIQLYLGLMVFCGYVIFDTQVIIFKATRGDRDVIMHSLELFLDFVNIFVRIASILGKDKKKKK

>1405

MASYYDEEAGLLGGGKESYSYEFAERTVRQGFVRKVFGILGLQLLVTAAVTAGFMFSAPLRTYVYTAQWPFWLAFGLSISLMIAMSCSESLRRSHPYNMITLAAFTLCEAFLVGTVSAAYNTQLVLLAVGITTVVVLGCAAFAMQSRVDLTLSSGAMVSLGLAFMSAMVLNLFIRANWLSVALCGLGVALFSLYLIFDVQLLMGGHKYSLSPDEYVFAALNLYLDIINIFLYILDLLSRLNRD

>1406

MASAFEHPRHAAGLDFDVEKGAALNAFAERTVRQGFVRKVFGLLAVQLALTTVIAGTFVTSTAVKTFVAAHPWVLMLGMLAGFGILLTLTLSSSARQSHPTNLILLFAFTAAEGVLVGAASSASRTDIVLLAFGLTAGITAAMTVYALTTKNDLTMSGAALYSCLWGLLLAGLVGMFVRTSAFNILLSAVGAVVFSVYIAYDVQCLLGGEHKYAVSPDEYVLGAIAIYLDIINLFMHILRLLNEANRN

>1407

MDAVERLGSMFTGRRFDGVNLNTFLKFTQLDPGVQAYLQRVYLTLSVAVAISALGCFLDIQYSIGGWLTGLMGFGCMLGLAFTSATPQTLNKRYALLGGFAFCQGAALGPLVGLAAAVSPGLVLSAFLGTAAVFACFSLASLLSPRRSFLYLGGYLSSAVMALAALRLGAWLAGGRAGFSLELYGGLLVFCGYVLLDTQIMVEKAAAGYRDHVKAALDLLVDLLAIFVRVLLHLLKSQAAKEERRRRDERNKQRRD

>1408

MDFVDRLSNLAGASATRAHAAPQKLFDFTNLSPAVRSHLQQVYLTLAVALCLSAAGVYVSAVTGFAQGLGILGFLVSVPWMMSVPSVPATLGKRRVLFGTAALSQGLLVAPLVRATLALHPGVLFTAFAGTAGVFACFSAAALLSPRRHFFYLGGLLSSVLSTFMVMRLATWFFGGGALLFQAELYLGLVVFSGYVVYDTQVIVERCEAGVVDPLKDAFNLFVDFVAIFVRLLVILLKNAESKERRERERESRRQRGARTSRL

>1409

MARPHTEHPLEGRSLRHRRQATLQQATAPKPPPQGNFDGYDQEAAQAAAYAAAFAEGQVRKGFVRKVFLLVFLQLCVTIGVASCFIFVDAVREYVRPGGDGQWVFIVSWITSLVMMIAIMCSKTLRRKHPWNLLALVVFTLVMSVLVGTICAYWQTSVVLEAFAVTGAAVAGLTLVAVFGKFDITKKGHILAMAGGVTFMVLLVTMLVGFFYVYDIQMVMGGKAYAISPDEYVFASVQIYMDVIIIFLQASHE

>1410

MGVGTDKRFWQFGGGGGQDVETGMPLYPGADSLDNALRWGFIRKVYGIIAVQLVLTTMVAATVVMNASVQHFLLQNFGIQIALLLVSILALIPLYIWRTTHPHNLIMLGIWTTLFSVTVGMTCSFYQPAIVLEALFLTAAVVLGLTLYAFHATRQGTDLTFMGPALYGCLLAMVVWSFIQLIFPPGPVGRTIFALLGAILFSFYLVFDTQLLISRFDLDDYIWAAITIYLDIINLFLYLLRLLGEQQRSS

>1411

MDFVDRFTSGSAAQRFSPDTLFKFTDLTVPVQKHLEKVYLTLSAALLIAAVGTYVNILTGLGGFVAAIGFVVCATWLTMTEPNAYNLNKRYALLAGAAFSQGLTLGPLISMVLAVHPGILFTAFLATAASFACFSGAAMLSRRRSWLYLSGTLSSAMSIMLVMRLATWMFGGRALAFQLELYGGLAVFLGYILLDTQVIIEKAYQGNKDHIRGALDLFVDFMAIFVRLLVILMQNAEKKEERRERKRR

>1412

MARLRVQDHLTSHDTCSHEDFARGQEDPVAAAAAAAAAEEKAKQQQKPAFRFPSQQEYYARVFVAPGYVPLPGARSGAWDPEAGAWQPIFGPMFTDSETRKGFVEKVLGLVLLQLLATVAACALFRYWEPARVAVNDHAWIFFLLWAVSFVAVLALASNDRARHNHPYNYITFGIFTLSFALLTGIITAFFDTELLLMALGMTAAAVAFIFIVAASSGFDFTQAGGLLYTLGFVFMIMIFVGVFVPSNIYYLVISSVAAVLFTAYLLFDLQAIMGGRAVELSPDDYVYASVQVYLDVVLLFVSILNILALAQSGGS

>1413

MSRTDNMLRWGFIKKVYGIISAQLVLTAIVAGTILAVPPVRGFVTTSLWFQITCAVLPLVGLIPLYMYSRKHPQNLIILALWTASLSVGVGTACTVYEPAVVLEALCLTAAIVLGLTTYTFHAARKGYSFQRLGPILFAALTAMVLWSIIQVAFGAYVGGPGKTVFALLGAIVFSGYIVFDTENLISRHDLDDYIMASVSLYLDIVNLFLYLLRLLGNNRN

>1414

MADTDKQAKGPGYPQYPPQQGWGQGPNPSQAYPPQQGYAGQGYQQGYPQQGPPPGYPGYSGPAAFQQQAPPPPPQQPNYDLEAAQAAKWAASFRDEAVRRGFVRKVLFIVTCMLAFTVGCSLTFFFVHPLKNYVRHNQWPFWLSWGLSLVAIIALGCSRTLRYKVPYNYLFLTAFTVIFGFQIGTVTSWWDTQAVLIALVATGGVVAGCFLVAFCTKLDFTKLGGYLAIATLVFMVMIFIGIFWTRNVTYLIIGIVGSILFSVHLIYDLQLMMSGKSVQVSPDEYISSALSIFLDIVNIFLMILAIMGGGGCNN

>1415

MVRPREVRPEPAEVDVEKGGLPDIPLYSGSSLVTSSSSSTGVTEPGDEAVAGKLTIRGASPAASGADEENQKGFEAYDLVPPGASAVVTQTRTTTTLHIFRPGDRPGGAVVDSTATPETYVSVRLFLRKVYGILSLQLLITTATIVLFMFVADIRNWVQASPTVFWIAVFLPLALFVPMWFKRHSYPTNMVLLLLWTIVEAYTIAVICTYYSAMVVLEAAGLTAGITICLTLYTLQTSRDFSFMGAGLFVVLGIFAGWGLIQLFVPLGPVTHFVFSLVGAVLFGLYIVYDTSVLVKHLKPDEYIWATISLYLDVTRLFVFIISLIGGSGRRME

>1416

MTMARPALTNPLRPVQLDNFAPMETISNFFTGNRGPRFSADAVFSTKPLTEPVRAHLMRVYSTLAACLLSAALGSMSFVKFGFGGGAAAALGSLFSMLYLAFTPYMPGNVQQQQKRTLALMVMAFFHGSNLGPLILMSLYVDPMIIVTALIATTAIFVCFSGAAIYAKRREYLYLGGVLSSALMVMCITSLLNMFFRIRIIYEMELYAGLLIFCGYILFDSQLIIEKAFAGNHDEVSQALELFLDLVQIFVRILIILLKNKEKERQEKNQRDSRRR

>1417

INFKFVIMAFEHREAVIKDLEIESNPTDSIFICNLETKLRHDFVKRVYSLLSISIAITFGIVSFFSFYETASKWLIEHYWVSVVFSICSLIFIILFSCIPSIAKSHYVGVTLLLLLSLFFGMSISGIAVCVNKFSVLLACGITILIFLALTIFSIQVKFDFTGWGPYLLIGVLIVLIYSIILIFIPRNNIAYIILGALGVMIFSFYIIYDTQLIIGGKHRQHQFCIDEYVFATISLYLDIVNVFTYILMIINSIDR

>1418

LQRSAMASTNIHSRRNEDIDLESQEFESFSKSVRHGFIRRVYMLVALQVLFDLALSLMVINVPSLKLFMLRNLSVIKMTAFAFALISSLLFFFLYNYSNLLQNHSSKMAFFCIMTISEGVLLSLLALLVNTKYLLMALAFTSIIVISLTIFSFQTKYDFTSYQAFIFYGTIAFAVFSTIYMFFPTVRIIELIISPIAIFFFSFALVQTTQSIIGNGKQMIYEDDYVLGALLIHSYIIDIFIYILRFILAVFERN

>1419

MESFFATNSRKSQFSGNFFNSSDLTSIQQTHLLKMYSSIIAGSFMTVFGVTAFINGMLRINSFVGLLAGIGVTFYLTASSSNKSSISIKRLAAYLLLCFVIGNGLGPLILFSNFVNPVIIPTALATTCIIFISLSFGVLFTKKRLSLYTTSFIFTTIAYLGLVSFFNIFTRSKFVDSLLSYAFVMVYSFYIYYDTQKTLEAIAYGERDFLLHSIQLYLDAVNLFTKIVVILIRKQQEEEEKRRKKE

>1420

MGSKSASVDIESGNYNTYEYSVKRSASRALGHADLEEYDVGVTVAACMERIRLDFLRKVYTVLAAQLGFTAVLSGAFMISGSLNQWVISASSWLIWVCFLGTLGALVGLFWARSRPKWSLPALSVFTFFEALSVAMICAIYAASGFGFIVFEACFLTALVFGGLTIYCWRSQRDFSFLGGFLGAALLVVLGAAVLNAVLGWMGHFSTTFSFVLSVVSALVFCGYILFDTSLIIHHLGPDDWSIACVSLYLDVLNLFLNLLQILTRIQASSDN

>1421

MDPFGSDRFFRPETFKNFRELTPAVRQHLALVYRTLFYAVVWAALGCFVQLNYGASLPLPTGLLTTLATFGTLFLCNSGGLSLETRQRLLMLFAALQGYGAAPVVQVALDVDEWLLLQALLATAAVFGAFSLAALHAKRRSYLYLASWLGSALSVLTFLGLWGLITGGLGDWSLAVLIYGGLMVFAGYVILDTQVIVERASNGDRDHVRHSLELWMDLFAIFVRVVIAMLRSQETRERRRREQHK

>1422

MASTSNRNFFSSNMTQIPMDEKIRIALQFNNLSQSTKQTLTKVYCALAIGILTATVGVLFSMFIYRPGFLMTLLLVIGSAILFATTPRTQDYKTQVKRFTLFNLVTFVTGMSSSGLIELYMDINSSIVLNAFMATCGIFISFTLFSLLTNKRLYIFIGSSLASLSIGIFVLALTRLFGGYSEPLDQLFILAILASSVLFIIFDTQIMVHRIENLGEKDVLFHAFILFYDFVDLFRVILKILAKKENKNNNKSRR

>1423

MLSSNFRNILKIKKNYLNLTQLPINNNIIVGEVNNNNKFFSNNNNNNNININSNNNTLLNFKNKYNLISNKNYFKNNNFLNNRNYGKTTPFGNPSFSDIIKDSNFLLLKKDYDDYFHLIKTNIGLKLFLKNVYITTAIAFAGTILSGLIFSQLMVGSPQSLPTALGLGVGGSMLSLILCNMQQPTYTTYDTIWDGPLDNSVKKPILKSVDKSGKSVIEFEEFSKSVIPITVANYSTLHKMAFVSFCVFNGISLSPAFGLLPVSTVLGVAALSVFVTGGSAFAALKMKPDSLTKYKPILYGSLFGLFGLGLVSIGTSIMMGENTFTRFAQEFDLYFGLALFTGLTALDTHNAVKTFQEGKPDYIRVSIDLFLDILNIFVRLLRARSDSNK

>1424

MIGSPQSLPTALGLGVGGSMLSLILCNMQQPTYTTYDTIWDGPLDNSVKKPILKSVDKSGKSVIEFEEFSKSVIPITVANYSTLHKMAFVSFCVFNGISLSPTFGLLPVSTFLGVAALSVFVTGGSVFAALKMKPDSLTKYKPILYGSLFGLFGLGLVSIGTSIMMGENTFTRFAQEFDLYFGLALFTGLTALDTHNAVKTFQEGKPDYIRVSIDLFLDILNIFVRLLRARSNSNK

>1425

MTSKADMYAPPEERYKEQQGGTAYKDQQQDYEILEAGLVHNVNPGGGSSAAAAAGGWGSTAEIEKALRLGFIRKVYGILSIQLLLTAAVAAVCVLNDNVRTGILGNLWTVWVGFFFSIGLLLCLMCYRDKYPLNMYLLGAWTFVEAYTVGVVCAAYASQGQGTIVVQAAGLTMAVFLGLTLFTFQTKIDFSFLGGALFASIWVLMLWGVVMSVFGFQQSYLYSLFGAIIFSLYILYDTSLLMNHLGYDEYIVASISLYLDILNLFLYILRLLSRDNR

>1426

MASSPLGNMFGREINVSALLKANDITLDVQRHLANVYAALAATVLACAFGAAADLWLHVGGLLTVVAGLGAMMWLAADQDKNNYPKRVGILLLFGLLKGLSLGPLIDMVLHVDPSILVTSLLATTTVFVCFAGTALFAKRRSYLYLGGLLSSVLSVLMVASLLNLFMRLEFLMSIQLYGGLAVFCGYVIFDTQLVVEKATLGDRDFAWHAAELFIDFVGIFVRICIILMRNKDKEGDRSSSRRNSRRSSSYYGSGSRTGRTTRR

>1427

MASLSSCQAGRLLAIAAIVVVATRPIEGFVREERGYLTASAGAPTSTAQDEPTPWSRRPLVCYRSSCAHPSTLLPFPSDFPGTLRIRGGGAKVAPKRTSGTGGGSDAASKPLLGPITRRKAADSAVEKGEDGKEDGQQEFAPQFLDFESRVGFMKKVYLTLSIQLVYTGLVCAAMRGYRDAILGVLFGHGNVPQILFFVSTLVTIISTHTIMWKNPELRQSFPRNLPFLTAYTTAWALYVGVFSLMFTKGSVIRAVFQSAFVVGSLTAYAFRTNPKHELTQFGAGLYSAGNALSLFCLMKIFFFRGHRASDLALSCLATLFFSLYLVFDTYRIIGGKHRQSSMFSVKDWAMAAMELYQDIMQIFLHLLSIFGEVQS

>1428

MLTSINNKAPAKPQLARIRSYTIRRQSSHHWTPPELYYSGPHDAATRNGFLRKVYGIVCAQVLFTTAVMHAMMRDGMARVAATAVSGVWEDRSCSPHILWPGHLTCTSVAIEPFVPLLMPAFAVSLLLTCCLDGFKNTYPYNYLLLAGFTLVEGFTVSFVCVALGIERPEVVHQTFMLTAALFVGLTAFTMQSRIRFNFLGGFLLSSLLLLFAWATINAALGLPFASTAFSLLGALLFSLYVVYDTSMICHRLGYDDCVVAAIELHLGVVNLFLFHMRLLSDDSLSSLSGPKGAGNLWLLFVRPKLLVHEAA

>1429

MATMKPAPAWPSHDALHDDDFAYGASVASCDAAVQRGFLRKVFGLVAAQLALTAALSAAFMFYAPLRSFAIHNHWMMMVSFVASLGLLVACQVYAHSHPTNLYLLFGFTLAMAWSVATTCGAFAAAGLGLIVLEALALTASVTAGLTVYTLRSKTDFSYLGAGLGAALWVLLLGGLLAMFVPGMHLALAMGGAVLFSAYIVYDVHMIANRLSPDEYIHASISLYLDIVNLFLHLLRILSELQRD

>1430

MFGQPQQQPKPPYAPGGPPGYGGATPYQAYQADPEAGWPQPPVDGEWAPNPCANLPPAVRLAFMRKVFAILTVQLAVIAASASFIMLHPDARHFVLTNHAVTLAAIFAPLGFIVALSCYQHRHPHNLLLLGGFTLCMSYSVGVVCAATYAAGLGAIVWQALLIAAVVFLALTNFMLLYKHDFSNHGVLLGGLLMVLIIVGLFVPFFGPTARLLYSGAGALLFTGYILVDLSMLMHHHGPDDYVPAAIALYLDVVNLFLYTLEMLRIFAGDR

>1431

MDDSFRRLQEAAAAGAGSLGAGSGSNDQTMEYMLLATAACYLGVFALLGVCAFTSRRERQPPELPLRAPQFQDSGEQPFNKTPVADLDMVWRKAFLRKVYAILGVQLLTTTALVATMMMKGGADLITWVQSEGRWTMWTAMIGSFVSLFGLQCVRHRTPHNMLVLGAFTLCESWMIGTICSMYYANGMGILVLEALALTSIIFAGLTVFTMQSKIDFSVMGPALFVSLLALIVWGLFARFFFASVVASQVYALCGVVLFSLFIVYDTHMVLKQYSYDEYIMGAIQLYLDIINLFLFILELLGIKPRDD

>1432

MHTHANAGAGEAPGSGSGGVVAPLTSINIDDKAPAKPQLARIRSYTIRRQSSHHWTPPELYYSGPHDAATRNGFLRKVYGIVCAQVLFTTAVMHAMMRDGMARVAATAVSGVWEDRSCSPHILWPGHLTCTSVAIEPFVPLLMPAFAVSLLLTCCLDGFKNTYPYNYVLLAGFTLVEGFTVSFVCVALGIERPEVVNQTFTLTAALFVGLTAFTMQSRIRFNFLGGFLLSSLLLLLAWATINAALGLPFASTAFSLLGALLFSLYVVYDTSMICHRLGYDDCVVAAIELHLGVVNLFLYHMRLLSDDSLSSLSVG

>1433

MHTHANAGAGEAPGSGSGGVVAPLTSINIDDKAPAKPQLARIRSYTIRRQSSHHWTPPELYYSGPHDAATRNGFLRKVYGIVCAQVLFTTAVMHAMMRDGMARVAATAVSGVWEDRSCSPHILWPGHLTCTSVAIEPFMPLLMPAFAVSLLLTCCLDGFKNTYPYNYLLLAGFTLVEGFTVSFVCVALGIERPEVVHQTFTLTAALFVGLTAFTMQSRIRFNFLGGFLLSSLLLLLAWATINAALGLPFASTAFSLLGALLFSLYVVYDTSMICHRLGYDDCVVAAIELHLGVVNLFLYHMRLLSDDSLSSLSVG

>1434

MLTSINIDDKAPAKPQLARIRSYTIRRQSSHHWTPPELYYSGPHDAATRNGFLRKVYGIVCAQVLFTTAVMHAMMRDGMARVAATAVSGVWEDRSCSPHILWPGHLTCTSVAIEPFVPLLMPAFAVSLLLTCCLDGFKNTYPHNYLLLAGFTLVEGFTVSFVCVALGIERPEVVNQTFTLTAALFVGLTAFTMQSRIRFNFLGGFLLSSLLLLLAWATINAALGLPFASTAFSLLGALLFSLYVVYDTSMICHRLGYDDCVVAAIELHLGVVNLFLFHMRLLSDDSLSSLSVG

>1435

MSFMFGVRAARLSLRCPTGPASRPSSMGHSLRCYSSRPSRFGNKKRTQVLEQEPEAARGGDWVSQSQVKEAPPTAAGAPATFPSYEQRQQQAGSQYESLSDGVVAHMRRVYATLATGIGISAAASVTAMATPLGLVHPLIPGLGAMVPLLGLMYTSKHTHSQTLRAGLFAAFTGLSGWAMAPLLLVALKVSPAIVPQALAITTGLFGTMTALSLFAKPGSMLRLGVPLGAGMLMLLGCGVAGMFVPVTSAWYPLLHSINLYGGLGIFTLYIAYDTQNMINEYEMGEDDHLKHAVDLFINFKVIFQRVLLLLMGRSDD

>1436

PAVRLAFMRKVFAILTVQLAVTAASASFIMLHPDARHFVLTNHAVTLAAIFAPLGFIVALSCYQHRHPHNLLLLGGFTLCMSYSVGVVCAAT

>1437

LTLRAGPQWPLQCRAIRVGSKPKVEVAVEERPVEEQQQQQQQQADLQQQQQSQPAWQSSGATAAKVLEGDVGLQRFLQRTALQTAAGLAITAASAAASIAGILPLFIGKSMLLPGLLGIGLLVGASMMRPTIVRDNLGFLSSQNSLLRQGLANGFFFLQGMMIAPLLAMSSPSVIIAAGLATVGIAAGMLAFALSKPQGALLSWGGPLMVGMFGLLFCSIGGMLLGGAAASSMFWVTSIGSLGIFSAFVAYDIQSAIQQYKEGYPDHLGHAINMYINLLNIFRSLLVLLGVMED

>1438

MAAHAHHHVIEVDEAKPLLAGDKAEDHESPAIGEAGPAPLSLSRHPNLRRAFLRRVYGVLSAQLLLNVLVVAACMYQPALRTFCLAHPMLITLGGLIPAIGCLIGMALYKDTYPANVLLLGGFTLCESLSVGVICAMYAASGLGGLVLEAFGITLLVFTGLTLFTLQSKVDFSFMGAYLSVGLLALIVWSLLQMVFGWHQQWLMAWFGALLFSGFILFDTWMTMDNYSYDDYILAAVNLYLDFINLFLNILQILSNNDR

>1439

MPAPAVKQSKPVGCDFDYGKHSEELQQDWNTYNVYVADTSVAVRLSFIRKVYTVLSIQLAITTVFLFAAAFIPAYKEFLVQNRWLSMVCSLLSLFMLLPLHIMKDQHPTNLYLLGGWTLSLSMMISATTVFVPPAIVCQAFFLTFAVVAGLTFYTFTTKKDFSFLQGGLVSALWVLLLVGFLRVFVPFGPAAHLAYAAGGALLFAGFLLYDTSNLIRKYSVDDWIPAVITLYLDILNLFLHILQMLSQRRD

>1440

MFSSKPVDGSGGKPMEYDLESPPYARQEDACPTPKEDAESAEYASSVDLAQSSGVHVNLRNAFLRRVYGLLTFQLVMTTAICAFCTLHEGTKGLLLGHPQAFLYGSALPTLGICIALPFLKNVHPWNAVLLFAFTLLESVSLGVVGAMYAHLGMSDVLISAAGLTMAVFLCLTTFVLLSKKDFSFLGGFLFTVLFIMIGWGLLNIIFGWKVHFIYSAMGALLFSGFILYDTSCILLKYSYDEHIVASIQLYLDIINLFLHLLQLLSSNRN

>1441

QLPAHNPTYQPPPAGPRAAPYAGGAYAHPGAHHGPHGGRPPYSPACSDPDLEAGGSDKAYAAYRPYESAGVHANLRNAFLRKVYATLCLQFLVTTIIAGICAFHYPAQRAILQSPQGFLYGSMIPGVVVLLAFGCFQHSYPLNALLLAAFTVLESVTLGTLCAIYRAIGLGDTVVTAAGLTAAIFVGLTLYVHASRTDFSFLGGFLMAGMLVMFLWGLLNLFLGWHASFFYAALGALLMVGMIL

>1442

GVWVQMKLQFDVFFCSLAAIGFLLWVYNMDNDPAVEQKRLLLLGTFGFLKGATLGPLIAQSLAFNPELVCVAFLGTAAVFACFSAAALCAKRRSYLFLIGFLSSATSLMCLLSFVNIFLRSSSIFTFQLYGGLLMFSGYVIYDTQMIVEKARGLARPDYIKDSLELFIDFAALFVRILFILMRNSERKERSRRRD

>1443
[truncated: 1,404,061 more chars]
