# Supplementary material for: The Distribution of Genes Associated With Regulated Cell Death Is Decoupled From the Mitochondrial Phenotypes Within Unicellular Eukaryotic Hosts
Source: Front Cell Dev Biol. 2020 Sep 23;8:536389. doi: 10.3389/fcell.2020.536389 (PMC7539657; doi:10.3389/fcell.2020.536389)
Supplement: Supplementary file 4 [file Data_Sheet_4.DOCX]

>742

MNVQQFSIKQLEVESKHLSDVLRCMLHTVLFNRAFGLVYPKEEIIDTLDFEYVRCDDVTVHQYVEEQIKTFRTTLAEREDRRAQLVLSFFETRSKKAWFRKVEEKICFEQWVFFIILKEDSPVETPEAREKLENELRERIIR

>743

MNVQQFSIKQLEVESKHLSDVLRCMLHTVLFNRAFGLVYPKEEIIDTLDFEYVRCDDVTVHQYVEEQIKTFRTTLAEREDRRAQLVLSFFETRSKKAWFRKVEEKICFEQWVFFIILKEDSPVETPEAREKLENELRERIMYIIQTVNEKQNHIPLKFQPTDLIPFPYEFIKTGSALHMQLTDVITIPSTSENWGVDMMWNLIKSPQPILT

>744

MNREEVLLPPVHLKRFQTDPALRCIFHTIVFNRSLHGKMLRPRDVDCDVFDVSYAMSDNKHVAQLITEGIRGFRKRMEEFKTDKGEACISFYTAKDWSFKSLFAQDDKFHWEKWIIPVKIEDPRCSKDCERDEKQTQRQNMLRECLFQIIAKLNEKTKHIPPLSSNEATKKLTDKLGCFPFEITYLKSDKSRWGQVKRTLQNPPSLM

>745

MQQVKKTRMSSATGCSINTAASWKEHILDPIPVAAEDVGDAVTCLLHTILFTRAPGPVRPSEATCQAFPNITYALCAVGDVSRKVDHSVRSFEELVVLGGSNFVMGVSSGMIPAYGQTPLTSSKVRGATSGYMVVTFFERKVKKALFGLMSNEEKTIFEKWVIPVTVTSSPAASQEERELCAGETEAALQNTLLHILTAVQSIDHIPVAMYDFEIATYSSSEGVQSGNIGGSVRLMP

>746

MEFIINVIAERSVLRESLKGIIWTIFFNRLFGPITPVTNEFMNVSYPMANNLPDLDSLIDEKVNRILNQITEVSNQAKINVQFLSKSSNKKKTGWFGNSTYNAQDDLVVWESWLINVESLPLDQVSNGESKNVQTSIQNFANNLTRIYDIADKFKEHIPPITSLDSAPFPYKIIIPEKKSHHNRDYHTGEEGWGTYIKKILD

>747

MNFEVHSLAITTDPSQTREAVSALLHTILFHREFGRARPVDVECESLDLTYVRVDSEEVAATVNEKVAQFAKMFETSGSSRSQLSLSFYEIQRKKTLFNFKDEPVNWEQWNVIVTQRPFLSEAERASYSSALTSEIHRHVSTIIFICNSKQDHIPVVKNDTSVPFPFEIILPTVQESWGFDMIKRVVLDSKPGPLWT

>748

MANCETYNLPVLELEPHQIREALRCVLHTIIFNRALGYVVPKDVDSELFDITFVKCGDPAVEARVESRISDFCAQVDKRPADLHQLQLSFYETRRKQAWFGMQDERLYWETWVLSVLVLQPDVAMQTGSGGAATTSAAGAAAGGSAAWNAGLQQQSQHAAGAVAASSVPGVAGAQGARASSRQARMQAALEEQLAFVVRCVNDRRDHIPPVLSASAVTFPFDITFSGSSARSSASASLQGSLQAVGKMLLKATPPPVLS

>749

MSNCEVFTVTPVFELEQHQVHEVLRILLHTIVFNRALGPVKPVERDSELFAVTWVECGDPEVSRKVEEKIGQFGAWVQRNPGKRGQVCLSFYEKRQRQSWFSTAEERLYWEQWVLEVAVAEPPPAEFEEQQAFAASSLRAQRRQRLQAAIEEGLTCIVRAVNDKRDHIPPVVSASAVTFPFDIQVAGDSGSVFSLGVVKRMLQSTNPPSVLH

>750

MSNCELINLPVLELEQHQVREVLRCVLHTIIFNRALGPVRPREVDSELFEITYVQCGDTLVERVIEEKIDQFYGWVEKHPGKRGQVSLAFFEKRKTQRWSLIGGKQEERLYWEQWHAKLQEAVEQVMTLIIRAVNEKKDHIPPVTSAAVLTFPFEITVAGRHDAQRHLMGVACCRRDGNIGYGLETMKRLLMHTSPPPVLS

>751

MNSKQYVLKEINLQYAQLKEVVQCILHSILFQRSLGTVKPKDTTLDCVDFSYVKSDDPSSSKHIEDKSEELLTSIMKRKAKTAQISISFYEKRTKTTIFSNNNNVCWEQWIISFQLVHSMDQKSLFSQLTDTINKIIENVNQEKSIPPITSSNEMPFPFSIQIGSENTGGVVDTIYNMFKPPQITK

>752

MNLKEHVLEELELHEYYVRDALQCLLHTILFVRAPGSLRPRESHCENFGLSFARCGARDVDVAVDGALEDFWRSLRPAGPDLSKGWIAVSFFMRREKKSFGLFLKEEKVVWEQWVVPVLVNTSPRPTEADDTSVS

>753

MNCEQIALEELHVERFQLKEVLKILLHSIIFQRALGECRLRDVDSELFDISYVRCDSRLVEARVEEHAEAFSSAIERGARVSLAFFERRARQAAFGLFRSEERVVWERWTIPLVVVPNFAQLHRHMGGAQMPPGAHGAGRSTEADSQDEESRRRQASLEAVLRRRIEFILTVASGRREHIPPADGLGGEALWFEISTDADSWSGLDLLKQLLSSPPQLGSG

>754

MNCPTISLNPLKVAESQAKDVLRCLIHTIVFQRFLAPTTPQETECEVLDVTYCKVSDVTVESRIEDSLDKLWRGLRKKEPRHAVIVAFYTMGGTPSTWFSKPEKAYWEYWMINLLVVPDSDASSVRQFGEQLERDVVDRMKAIVKHTDAAIENNHVPPLKDAAPNFEITSDHHQQSWPSSLMEWVKSGKG

>755

MNCKEHYLPELELSTAQVRSALQCILHTILFLRSPGPVTPKDVECEDFNLTYARIANNHQNQPPNKAMGNIVHSLENDIDQKVDDAIESFLRTLSQIGPELLSGCLTVSFFERRATRQLFGLVSHEERVVFEQWVIRLVVNNTPRPISNDSAALMERQRIQDTAEGMLRSVLSKIFEITGGPIDHLPPVMYEFDISCSKRADDRENVYSRVANMPPILNLTT

>756

MNCKEHYLPELELSTAQVRSALQCILHTILFLRSPGPVTPKDVECEDFNLTYARIANNHQNQPPNKAMGNIVHSLENDIDQKVDDAIESFLRTLSQIGPELLSGCLTVSFFERRATRQLFGLVSHEERVVFEQWVMRVVVNNTPRPISNDSAALMERQRIQVRQSSKSKSGSGAIFCMHTMYH

>757

MNAQQYILEINLQYSQLKEVVQCLLHSILFQRCLGTIKPKEATLDCTDFTFTKLDDQQTTKHVEEKSEELLSSIIKRKAKTAQLSVSFYEKRQKTTFFSSTENVCWEQWIVTFQLVPTLDSQALFQQLRDTIQKIIVIVNQDKNIPPITAKDQNPFPFTISIAGSENTGGVVDMFWNVFKPPALLGGK

>758

MNCEVFTLPPLRCEARQAKEALRALTHTILLARALGPCAPSDVDSALFPDLTYVTCGDADVERVVEARLGAFASWTAKRGRDVARRGGDAREIYARVCVSFFERDRGDRGGGLGGSGSGSGAWEQWRIPIEVTLESGDGGEGDDAAAARAREERSLALRDEIRGAITHALTLANERVAHVPPVTRAGAVSFPFEISLPGDGASGTLGTLRRVMLASSPPAMLT

>759

MSHYQPVTFPVEVTIERSLLKDALRAILHTILFHRVFANIKPRDIDILDITIPIIDDPEIDRLVDEKIAAFVKSVDSSQQSKGQIGLMFYEKRTKRAWFSSSSAEVCWEQWGITINIVTNTNEKDRQRSIKNMEKALSSLFLSILRTVNERKDHIPPITTSDGNPFPYQIIIPTAQDTWGSMFKQMLGTSASSSPAL

>760

MNHKEHVLGELELRPLHTKEALQCLLHSIIFLRAPNIVRPREVHCRHLRLSYPKVERPTSSSSSSSPFSVARIDEVVDSALDACLRNLTPAGPNLSRGHLVLTFYERRQTLSFFRLIQNEERVVFEEWIIPFLVDSMGSTSTLASSLSASDDGFVGVHDGELYTEPSSHSSMSTLPPASLYGGGGEDPAIAVEQQRAQARTEALLRSRLQDIYTLVNGSIDHIPPVSYLFQISHGAASRDRAQDLATKIRNSPALINNSL

>761

MAKQFTLEIETNYDLLEEVVKSILHTIFFHRIMVLVTPIEVQLKSGIHYVKVNDKKLEENINQKTKQFVNSINSNKNVKERIEVLFKKENIIWEQWNLDFVIKETNNQKIMENLENLLIKISDVSTYTNHIPQLKPNQEFLHEIIVKSNVWSKIRRMWSSP

>762

MPPTPSSARRCETFALPPLRCELALVRDVLTALVHTIAWTRALGCASARDERCERIDVRYVTCGESAVETKIRERCDAVIQWALRNRGGEADVSVSFYESERSDGASGTSRSGVKGALTAAIGGVMGESKRTAVCWERWRIGLEIFLGEELDEDERARARDALRDQVVGTQRHILSLVNEMKAHVPPVTTSDVVSFPFEITTPIAREDSPGSFGRDMLRRIASLSSTPPSMI

>763

MLDYPQFKLPEFVKLCFHNQLLDKTYLEDVICTALNLIFFHRALGPVKLNHMRCERLPKITQLRCGEPQVDKDIKKLIDNINNDPNMINCSSLQISLKFMEEQPTTLLQFFKPKLKVFEEWLLLFQFGLSSIDKTQDEFTQLLNKIIDIATANYDHFDEQFDQSQQKPLKYPYELTFNFQ

>764

MLDYPQFKLPEFVCQSFHYQLLDRTYLEDVICTALNFIFFHRALGPIELNHMRCERLPKITQLRCGEPQVDQDIKKLIDNINNDPNMIKCQSLQISLKFMEEQPKSLLQFFKPKLKVFEEWLLSFQFGLSSLDKTQDDFTLMMNNIIDVANANYDHFDEQFDQSQQKPLKYQYELTFNLQ

>765

MLSSAGIASSSTSSVNTGGVPASSWREHLLDEIPVAAEDVGDAVTCLLHTILFTRAPGPVRPSEATCQAFPNVTYALCAVGDVSRKVDHAVRSFEEKVVLGGSLAAGSNYMMGGSSMMTPYGQPSVNSMGTSNVRGANSGYIGVTFFERKLKKALFGLMSNEEKTVFEKWIIPVTVTSSPAASQEERELCAGETEAALQNALLHILTAVQSIEHIPIAMYDFEITTFNSLEDAQFSSSYGGGLGSASVRLMP*

>766

MNCKEHYLPELELATGQVREALQAILYTILFIRSPGPVSPRDVECEGFDSLTYPRIASTPSSPSSLNYDVDKKVDESIQNFLMKLSQIGPELLSGGLTLSFFERRKTQQLFGLVSNEERVVWEQWHLRVVVNNTPRPVSDDCAAMIERQRIQDTAEGMLRSVLVKAYEIAGASIDHVPPVMYEFEISCSKKVDDRENVYSRVTNMPNLINLSN

>767

MTCACEQLYEDIEASISQVTKASEQILANPSSESHSTAIEIKFYEETKTKNDGFLGSLGAFWQKEAKSYWEIWVLFFLFWLKQIIIKQHTSDVDLRVLEKEVRENMIKIIDFANKYMDHLPKLPNIETLKDSPVSYPFEIKNPDQKDNGGFGAFKMLLKTPPIKT

>768

MSSKKHEVVFTIKCELLRDSYAYEIIEAFLHTIFFFRTLEGTFKPQDASCPSLAVTYAQVPSAKLRSSIDAIATQFHEKLQRRKPNTTSSVTMNLCFMTKSKGIMQWKVEVWEKWEIVITVPDTKTAESVSEAEAKEFAANAVIEQLNEIIYNVTTWTRHLPHVSKKAEMPSAVDISIDQLPYHYDMKIEDDGKSSALQVGLNFVQRML

>769

MLVPQGQASVRALRCVLEHSRPRGCFWSLRTFAWQAPKVDCYALLGVSLSASVEDIRKAFLQRAKSAHPDVVGSGNGDMVQLNLCYEALTQKRKEYDAAKGIAGQKNSTSGTSSNSNSREAWWRSQGGFDDFQDDYYPFHFDKFTERMWRPRKKQGQEASSKRRPSWEEFAEMWDEEFETEESRGRRNPRGRPHRRSAKYAMYEWSSDEEEEQPWEQKKRETFKEDHEQEPPSPGWKQSEDVPGEMWVEISGRNNTQWASIGGCVLLCVVAMDAVEAGSHYAVLGVSHEASLEEIRKAHRFLALKWHPDKVADPSDQISVRVATQQFQLVQAAYEILSDVQQRRRYDDRLGTRSTQPGQKRSRSAVSANWAHSDAVSLRVPEDVSTIAAAVDRLSAAGGVIEVASGTYDGLVVVSKPLVKLVCKAAKAVIRGQVVFRECATGAEIRNFCIEVPGSGAGAGAGGAVDLKGVVGDITIEDCDIRNEHSAGLVLEGCSGHVHIKNCCIHDCKFDGFGLHLLKGDISSKGSVTIENSVIKGNGYDGLYLGDPRYLVVLLQSRVCQNQRYGVLVRGSNFSMEGSTVEENGEDSVRTEEFVHKQNPRGTQKVGKAREVVVDLPEGWRAFRNSEGLVYYYHMQSGTTRWLEKAFNGRPAFEKRGQPSLFMFWSQQFGDWKIAERLQDDGACLGFAEDMKGRRRPWIPHPTLRWRLWEPSARRFVPRRLNIDSSDDAPGGNEKWETGEDDEVPWSRPHWSEWSTTDLIRWCERRRIDLSGCFDREAVLDRVLQTAKEDMAEADNDNRREDTEFYTVRIASRVKTDGSYTRPPTLDRRSSLYGNRVERFHGVESDILPWLYSAGDKSRLYGIYIGSEFAYSLVWTRYKFWGRPGAKPNLAMLNVEEFLSPELRLAMCHVKEATRIILHCLVVCRTIGGADSIDPKAVVSELFDVEYVRINESQHELERTVEKFSEILENNLDRSGKAQLVFNLYTVKTRKQSLWNRIVGSDEKTVFEQWRIPVAVQPLNRYLNPADNLREEANLQASASQQVQQVLHYIIARANTKALRLQVSAVKPLFKLCLCQVDHLPSPEKDTVSYKFEVAFLSGEGKESCGMKLGANHGLSLTLCCQEIGTGSLLPQGLRRAIDDDSMTCIALISSGLGSSLSQTIRHLPYMA

>770

MESVEDRVQDFCDALDHMANMEEKRAQIRITFSEIREKTNMLMITRQESVVWEEWTIRTQLLMPRTDTERRDMKRQVEEQILKNTERIIHACNSKYNHIPRVPNSSLFPFPFEITLPKNKGRWGGGLDYYRKIVNESGGANPSGS

>771

MLQSLQKKMDCPLEVETKVKASRVGILGRACDINLRCFVDPSSGSPVFSPLFWIIHIMAPISPVVMPLELVVDRYYLAAVMKALLHSILFHRRFTSERPMEIDLDALNVTYARLEDPELERAVDEKVRMFDKALEPLSGRNKGQIAVMFFERRAKKSWFTKTEEEVCWEQWVITITIRESRTEREHIDARRALQKDLVTALTSISQMTNEQKDHIPPIVNNDPFPFQIVISSNSDGSWTSMFKQMITSGGPWLSAT

>772

MTKLSHVRLNFKEFNQKLPLIKQNVLNRNASRYANPELVNHLHEEYKKKRFEIDLLRKRRNEHSSALKQLLVIEDDRKREEQITSHHKIGKSFKTDLQNYEKDFESLELQLITEALKLPNKTHPDSPIGPEANNVELRKNSEKFKSKNNKSHLEIGEMFDLFDFQNASKLTGNKFVFLKNEAAIMELALINYAFNFVAKKPGFTPITTPDFARVHVLEACGFQPRDEAGQVYFLDNLSECLIGTSEIPMAGMHSGELLRKEALPAKYVAYSHCFRKEAGRGESSRGLYRLHQFSKVEMFGITEGDYEKSESLLNEFISIQEEIFTNLGLHYRLLDMATEELGAAAFRKYDIEAWMPSRQDFGEISSASNCTTYQSKRLNISYFDNGTDKKLAHTVNATALAVPRIIMAILENNWNEELKTEGNFNLLQDYAYGIFNTIIYHRALGHNNPVEQKCQLLDQFYMKCGDSQLERQISTRIDSMLENITKNKVDEISLMFYNQIQSSPGGIFSAPVTKNEPWEIWIVPFKFIDISDGHINLEEQSENFAKIIMDVIDKMQKKLGVLKPKVEPLLFLDWEVCLRNQPIELQDQLVDIDRYNYTQQFKMEQTKTKAHPLQGVLDKDGRAVLVPSVTTDAIAIRPKADGSGDHDILLITRGQPPHKGSLAYPGGFVDYNEDPLVCCLRELEEECGIKGNNPVLVTVEGAPGRDPRKHIISIFYKVDVDFNAEPKAGDDAATAQWYDLKDIWNRRSELAEFIIVNFNQVIYLIISQYFQGNIGKALYFGTTHILLVILLEWSVFACCFSDPGYTVEVQQENKDIVEVRQINIDQEQNNQNSNQNLLASPEDNKDMLVYAKQPLLSQNQRDHNQNTNIDLEAGTEKLIIKVNLKEAKLLSGRKKFQTTEEKKQYEINQLTNYRYCDVCKELKPPRTHHCSICSQCVMRMDHHCPWVGNCVGLKNHKYFILFLLYTAIAAMQVFLTLLFNNPQEFTLVQHISNMQRDNGIILAFIFSISFSISTLFMFLFHVFLIVRNSSTVEMEALANFNIFDRGNKANWAQVFGEDWRYWWIPLEPKRTTDGLSYPINHRMIYWNQ

>773

MELDPVTFTLNKIAISQELVSDVVSSIMQTIIYHRALGTVFVNFQQCQKLTNTTYMYCGDEQVNESIKNLISQIEKDIQSKSDKNLIGQIKLTFKNQEKTKYLSFLDKKPAKVFEKWLISLEIKKNQEAINIQRVESDIQNIVFQIVDFANKNYDHIDESYISKSKDKIVVFPFEIEWVSQQSRN

>774

MNCQVHQLPEFESATSHVREALQAILHTILFIRSPGQVAPYDVQCESFDTTYTRIATDNDPPPTLHQSRQHHNQQPTNLLKSDLDRKVDDSIETFLQTLSPIGPELMAGCLTLSFFERRAKNKLFGLVHQEEKVVWEQWVLRVVVNNTPRPMGGDEASIIERQRIQDTAEGMLKAVLFKVFDLAGSEIDHIPPVMYDFEIANTKKADDRDEVYSRVSTMPALLNLGS

>775

MQANCAEVILPSLSLPLAYIKCSLRCILHTLLFCRQKGPAVSLSDAELPQGILSCGIGMFGGSDTLQLVTAEPLSIAFFKCGDEEAEREIESKVVAFTKMLERGPCDTAQLSLSFYHLRPRERVLGLFQPPDEKVFFERWVLSVSLVLHENLGPASRFSAARKLDIRELPVCTTVRGAKTTRAEDVSRVYSADVQIPTDRQTCAGSEGLAVLSGVCTPAAHRRVGQDLSEIDGTYKPPTGSVFSSRGEASSAPISPFLMCTLPPSKVACLGTSSLPSRLTEPASSGFCSEGQSTTHSAGFLEENARESELLMAYPVPHNHDPFKQNERRQQILTLGRNSDTSNDKEEMRGVVNLTGDYIRELSHVEKQEFEQRVSPANSYPVSLSSSGSFTQGRRIARSLTGVEHSIGGTNASGAVKPIRRGESTSVLRGQISTTNGSSGSFLSEKHLAETRVGGLLSPSIASALDSPRSQVAGGSAHGRLTCFHQHSETLREEEEPQRVPECQNRAKDYVHVQIAGVSSTNMVPTDIGAEQEHDQGSGCNWFQSTAVQGDLILEGSLGERLNDTDHTRELGRRGKQTKHVSRFSSSSWMLQEERAAQRCVERAVRQVMMKVIQVTTEKQWHLPPPFHGSPCEGSMYRFEINFSGPSAVSVDQGWHINLPAALRTAYSRHMPYLT

>776

MTTKPTEHQLDPINISPDQFLDCAKAIIHTILFHRAIDTQVIPKSIIMSGVDIAYASAETPESSENIHKRLLPMQDAIFGGAQNTWIILSLSYNTPVKGWFKDVQSSQVWERWSIPFQFQTLSAKDVRFAMLHTITQITQKANSCNVAMRPSEGSTFQYSLNLPTDKGPETAELVNLMKKIVKTPAFLFQ

>777

MNSLIPTEFILDSIQISPDQFMDCAQALIHTILFHRAIDTQVTPKSIHMTGVDIAYASAETEEVTQYIASRLAPLQDAIFEDPKKECWLVLSLAFNIPKKGWFRDTYSTEIWERWCISFSFATLTAQEIREKLLHTITQITNKANASEIPKIPSSDASFKFTLSLPTDKDSDVSTELKKLIGKIVHTPTSMFA

>778

MQRLDFTHRIRCELQHLERTAQVVLHTICFHRFLGTVKPAYVDAFGMTFPAIADAQLDQLIRQRTSQLMATTTSTKTASLTVYLLHPSSQIPSAMQSGRKDAPAPKNTTSKDVTDRALGVVHTATHAASSLRAWASPRNYQYGWLAHAFSSGAGQPSGASVACQPVQAQDDLVQADFASLDEEAFERWTITFELKQSGGDAESMERELAGFVDQVLRFVGNNKAHLPGVTSAQLCPYPVQVVVRGAES

>779

MCPELRVALCHLKEALRCLLHSVVFCRSLGAHNVVTPMSIYSEQLELGYVKCYSPDLSIDNLIETRINAFATLFERELAIPTHTHTNVPPSQGTAELRIGFYVPRAKRAIWGIFKPPDEKIFFEVWRIPIRVSVPSSSPPPFTRSLRSSTTAASQQTPPPPPDPQSTAGHAAAAGAAAAAVAPSSSSSSAGDATRLLEDELAMRRDAHTEVRRVLLYIIENVNKKRDHLPPPRDQQSIYWFEVSYDIRGQDGSAAWHLPSAIRSPPGRKLPYIT

>780

MANCETHNLPVLELEPHQIREALRCVLHTIIFNRALGYVIPTDVDSELFDITYVQCGEPGVEARVESRISDFCAQVEKKPAELHQLHLSFYETRRKQAWFGTQDERLYWETCALEEQLAIVVRRVNDRRDHIPPVLSPSAVTFPFDITFSGSSTRGSLQGGLQAVGKMLMKATPPPVLG

>781

MKVYRADTGEAYDVGSSPNSTVASVKAELASLAGVEAADQILLFDRYKLDDANTLAAYHLPAEDKPVFLFNRKQLMPGTPAPKETVLGPIETRVPPETAAPNATGLVGTLLKYQHQFLGHLNFAKAVLVAVDSRIAMSRHCCAEQDMQAKALDVAVAHLNEQYRQVPPSFEAFNEYYQRQKSKHENLLESFETDLAKLREMTLHPALRNGGMQTLMDVVSEARLRKWAEDCQKGNEQLRLRLEEVEHQIVSTQREIDSLAAGFEVNLEGLKEGLKNTGPLRQEVASTKASFSKDYKSVTATIEDLHRGKGEGGSWEICDGFDSMKTVHLKQLDRLTKIDLASAKVLTACAESKHVAILEARVRDIINKFLVLREALTRQQQAFAQLFYVRYLPDAYHAALDEVVRRKAFGKQLAALFTQFAQRLARIRDDEIKQRDAFLTKHGRYIPKNLIPGLPDELPDFVSAFVGRAFPVALDRNLPAIEPQVDSAHAAPPLMNLNSELGVSPPKSSPPTELSGRLGALEAENARLVAEIQGLRREREQQKQKTPMKTPSREREREDEAAAMYRSPYSSPAASSSSSLASPSSSMTGLSGAERKRVEESYQQRILSLEEKLTETYRQASLAEQTREAAEKKVAVAEREMRMMREALALMEGKLKDWEAARKPPAAAHGADAGFGGSADVGFGGNNSSGSVDVSAERAAMKALRVECEDLRERLNKLSEENRLLVSNLHETHETLMEEKGRQKRRDEELEAAAHDGAELRERVSALERRKIALEEELEAAKRSGADSVEAHERALEECRRREAALEADVREALERLDLTARERDDLRTQIAALKDELERAQAVLVEREKAEREIVQKLAQVEAEKEALLQEQKATAQLTEEQSQQLAKSLSKARAEREARKKAAMDVLAQYDMEKGKSLSASTALAIADLASPTSSATFGLSESPTSSSTFTPGRSGIPMRRERSGPLGDEIGRQELLAEANRASQLVEEEQASMAKSISQLEERTRVLQQTLSMREKELESKIGEQVEKEAEVTHLRVALEEEKELLHRSLLEKDLQVQDKQLQITRLEDYIRAKEEEIARLTQARNEEGELQIARLEDYIRAKEEEIVRLTQALDEEREQRQAIAEQKEALEAEMAVRQHDGKTEAEELSKTLAGLHGQLDIKDRNIATLLESNKAKEFLLGDLNDRLKEVTKQMNELEESLTTNKKELEDFRNLEQTLELLRQESEQTKKALEEKIAELTASKQRMLALSDFKVDDLMCFEFDSSAGRYEAFNRGAPNYFLSEESRELFREQHRTRRDHIIGQVVFIMEFVARTPYRLPINTRFYEVTITKIETEAAQP

>782

MDPEEAEACAVVHVGCAHSGEVIPVEVWGAVGSARSSLRRLVADACGCSEASQIVLCGPPWRPLDAESAAGVLARAPPRGSAGARASELRVFAYDRAAIKAGGDGPQRHGGADAADGRVALEALVVPRTLPEARRLGLLDDDGEMRESDAMAGSPTDALSVAGSPGEGGPLQRALGDFASQFKLRLAQGRAYARAATNRAAACRGLADRAAVRSAAVAAALLNLGDHGGALSSAHGALAAKARRHGDVQRDALERFEDDLDALGRRALHPALARAVFDGDARAHTLAECVPAERVRKWAEHCARTLAAVEADGARVGGLVADVAGRVDALVAPETTRSMRDRVDGLEAAAAAVAALAEDQRRAVDDLDGDERDASRLAAGARSGVLEDSGSGEEPTSCMSTSATSPKNTPPNADLGGFFPAHRGGLLDDSDAAGSESGTFGRGSTGALDACRELQTRWESRDRSLPAIKATEKAVRDGAKGCALEAEALADGALRRVRDVADAQSRIHGARSRHLAAFGDALRDKAAHFAQLDAVRRLPRAHAALCLEVARRRAYGVAAVAAVRGAADAVAALRDAEHERRLRFARSHGQRLPRALLKAVPALLEAPATFSPTFDPETSAEPLPDVALSDLAPDGTPLSSADPADQVQEKRPRAWSCESAVGNAGAATTPGSTPPKRDPDVRMAEATATYGEDEARRAGGSVIYGDRDRDECSDDPDDCEAENRALRARCAKLEVELAALRAEALEKPRGGKPAESDSDDFDERDDDVPETLGRRGRALSEDSAEVKRDLAAAARGIGDLEAALGLEYRGAAPPVAERCAAAVRAVRAAAAAGGAPKISFRSIGVGDVALFLPTGGAEHSKAYLAFHHGAPHRYLAPQSIAAIREAHQRYPDFILGRVTRAEPRDVTNDPDSNPYRLPLGTVFHVLAVESL

>783

MSDAESEEALRRKESMRLFLADTGRSFPIQADPTDTIASLHERLELETGIPIKSQILLTAQGSTFDSKARVADYPKLTKNPDVPVFLFNRTFILEPPAQSSEGSSSDSKQGGPILFDASLVSASCFRIYEFSFENKSPLDVTHVLGEYQRHFENQLRQARAYIESHGKRHEASCTILRALDAKNHALRAAYNSLNAHLDSVLKSMSKFDQSFTRLSEENDGLLGGFETDLVRLGKIRVEPALFATATALGPLSSSARSSSSNKPRTLLDILPEARLRDWARNCSMRQQQLKEAVGGLRTSFERLMRDVKDHGSNHFHGILTKLEEKVTSSGKLCVESENLTEILGDDVNWVKQVMSHTHHMDDLRIFTSKARDKDIEHCHKLSSVRDYYDAARRPLESLYECRKEHNSFFYQRLAAISELQTKIRSCKKKLSLYKEGSESLRRMCVELKKTKALPGVYKACVQETKRRYVFQKRFLSLVSSFEKKLIKVHEEETQARTDFAKGRWEYLTPEFFPFLRYPPPKVHINVKNVGEIPNLFGPEEQDDGDEKVQEGVDDHKDGGTEGKDGRDSESMQKQIERLTEALRIAERKLLEATSKMKEGDVKKITTAAAAAAAAVAAASSSSPPSSSNDDHNMTVSTIEAEHVTPVKVMAADISVIEERPNQQQLLAKQRESEERISRLVQAKEQLQEQNQNLRDKMLEMQVSLQEAQQNNAHLEVRVNQMHQDKRGREEEMEQMGTKVALLLEDNRQLRQREDREDMWKELASARISFRSFQLNDVALFYRRGGMGQSSSSPGNGSKRGASKEISSSSSNSSNSNSSSRRGGAQQHYVAFNQGCPHRYLAPECRSVLKSNPLFVLGKIVQVVSGVVGFIGSVGRDTRNSCANVCES

>784

MDVAMTKAQQHAEAEEKNQFGILLHVGDASTGTVHIVPLTSTHVLVEEVRKDLERLSGVPTMDQILLGGPPFGRLDPRRIIEYYGIPAEDKEVFLYDRRLLSLDSAMPAPTSAASTPILIDLPSQPTTSSEGSKMLSESSHPMMRALAEYEGYFQLQTSQSEALENGTRANISLSEQGAQELQIQERAITAAIANLELFKTSMMKHFAPFWMEFQTTKSKHERLLSEFEQYVEALATVKLHPALTTDTRKTLYDCIPVQKEREWAVQCEQSHAHVEGQVLKLQQVHDDICKEVTAMLTIHANSRREYDDARLILEEMKALRDKQMTITNTLRDNLQYVLSSIEETSLIARGSNPMQASTNALDVCRRIDALYQGQQKMIPDAQGLVKNVMIHVKTIAAIKATAFERVQFTLRQISIAQSKIRDFENSLAVFREAISAQTEHFYELEHLEKLPESYAACLKEILRRHQYGRKFSDRIQLMAEELAQLREDEVQHREAFLRNFGQHLPRDFVSGLAEKPSHCEFRMRPFDQSLPMIEDDNENAVRSPSDEFVDCEDHGSAFEMTSKVDRLQERCKELELRVSELTAELVQSKKNFLFDGSESDSISRSDISKTSAHEGDSSCEFPLVVALAETAGGMTSSLEAERSNVVNQELAAVRRNIGIDNKEATIAKLESENQQFLLNAAAFEEYPFDHVVICSHVYLLLIDCGFLNRAERSRQEMQLKETQSKLQQTNADLRTSLCQILELLQLHVELPLENNHVDAFLNDNFHIVETRVKELLACAESEMSELMKRRSDHCLLESEQDPDDSFKISFRRFSVNDLALFLPTSAPGSDAQRVYLAFHLGCPHRFLSEESISSFSNDGQRYPDYVVGRIVLIDEQITTEANNPYALHLGTMFYVLTVASLHES

>785

MSSANGQTITVCGNVFRLVEMIGRGSYGTVHKAINLSSGAAVAVKMIGKEKLRRPHERQSIEKEIETMRVAVEQFENGHPHIVRLLCTKESQQHIFIVQEYCAGGDIAQLMKTTDGLTETQARLYMSQLASGLQFLRSQNVVHRDLKPANLLLSSPDFGFARELESEMMAESVVGSPLYMAPELLEYKCYDAKADLWSVGIILYEMLANEHPFLVVDKVHATNHLALRRNIYRYYECFGHVRFPKKIKVSLECEQLVAALLQVDPRKRISFEDYFRAPFLLPPAPSDTDLPFDTAGLDSSETKQPLVAEKQTEYEDWATFSDEYVMVESEYEDVGRKVLNSQDLNVDLDAKPKHHTKEYIPERDSGSTLCDGGIEIRDIVIDTSSPRHGGPEAETARSPVLNTLEVKAGMIERNANATMPKDHWLIGERRQWVTDDNRLEELLGICYKSFTCGSVALFVPTPFGDYIAFHEGCPHYYLSEESIVASKKHDRNPPYVLGHIVYIEDHETSDEKNPYCLREGTKYHVASVTPLATSSHNANGSVETADSSDASHELPLSQVLTGKAVVTGYEDAGLHQAENELGSTEHQRISFRSFQISSLALFFLKGGKLPYVAFNDGAPNYFLANESIQAATLGVGSDRTPPAYICGEIIFIDTFQATEDFNPYRLSYGTRFYVVTVVYPTRT

>786

MSEISYLSINNAHNGIIIKIPKPVRFHTLSEFKKYIQQSYSIDSVDNLFLLTTFGIKLNYNLINEIGEVFVYDKRLFTNIVDQSLIDQYTQSTFRVSEPTHSPLLKSNVGFLKQNLSSNLKINQGWARIITQDGELMDQYCRELIQQINVIFKCLNTIFQFATNFTNEIEKNFSNFFNYVKLINYKTLHKSWITNYKNLKTFPTFKIDNENIKLSDFLEVDRLQSSADYIEKFLPLIVNKLNELKQVIETVNEEKLTVDKFIETSRNESISNFKNVNISNVLSQLQTESQQLTDDIENLHYKNMDEIYRLHRDKLSISIYNNAKDIYKNLNDLQQFKNKLTKASLKAFNTIANLQMKMVGVKTEMKKITTEDETATEDSKVGDVNYKTISNVKKYEDYLSLTIDLPLIFGFSLIEKRRQFEWYDFYSKGIVNNVSEQLSTIIEHEKVFRGIWLKKFGTLLSLINDDPLTPSLPNIDVTLVGNRQNNFSILYDLKIERDDIINYISLIEATNMSKNFVTLLNKNFKDLIASTNNMKKVTKVISSLSTYTTNSADDKSKSSHEEGTEEEIDFDLNLIKGLKSRIKKLENLLHQQQFKNLNNWPVIRNVPSMTNDNRQSTIIQPTVVSPARTNPTQLLSRNPSTTKENTTTNIHNNHQQSEVLDSSVIDKHLDNIRLKKLNNELQTKNTELTNQINSKNETITQQQKEMEHMKLKTEKRVDELMKKLQEKDEECQSLKQENKIKCDEVENLTKKLELSDNHNKELEAKITEYTQKATSKTKEIADLNKTVSNLRSELGDAMHMKNDLLSNLSSKEAEFTKERNQFNNDLKALQLKLDEINEDYENLMELTQAKQKKHDLIINDLNNVIINLMNDIKKTLLSVFEYFLEYCLVLESMGLLLVKEDEIYKIKRVKGLKSKKSIGDGDMSIISNGTPSSKVIEEIENEINIVNNIPPISSILPDSYSSGTESDSVVDRYNDQSMKLISTFNQLFKFNNENENENRIDHILNTLAFKNNVQLQEDSINDTRFFLNAISKRFRDVEGFAKRQAKDNKLKEQEHRKLVHRLNSKISVNGFQEKDLVLFLPTRIDRPNGENIPSNDKIQPWAAFNIGAPHYFLKTEQTKNKEWIIGRVKKITEYKVTEENVQSLESNPFQLSVNVTWYLVEADEE

>787

MSVTGGCSDEWCVVTERNAPEDAVQPQTKVQQQPTHAQPLRGKDDHERNGRDADELEQEPVAMEDDEEAILIYLPGEARRRNTTGGAADDGDDDDDDDDDNGDDDDGDDGVETDPTISASVVLSASAATPLMTEKAARSNTGVTSRSVVVGSLAAAAGIAALQASTLTTAEAAPALQKGAPSAASILGANADSPRNSSANNANNASSSSVAAATAKAVANAAALNAADKAASVAAATIERLTAERDDYCRRYENASRLLSVITNELSDRENERRTLTPAQLIHSWVASQRQKPTFDVDSADCVGRLTAATIDMIRDRNELLRRTIETAQRNDDNRPRLAFSNLTVGDLVVFFPTTHGHYIAFSQQPRYYLSQESIKAFDGSRTSGSRRDYLLGEIIQISEHVADDSSPDGNPYKLPANVRYHTIIAALASSAS

>788

MAAHVGAPNFFLADPDERMPFLVARIVEVQARRAAPGPGGNPFGLTIGTPYYVVTAGHPDLPSAA

>789

MEEAAEFVGPAEVTLPTEPGASPSPLPPVEGSYLLKTIAGYERGSMLTLNRGQAYLESSRRRLAGGETCAERMQTMVKALDAAVSNSLDHWDPLQRAYSGLKEKLGEQALRHEKMLETFDQDVATLGTIPLHPALAAASATTTTTGGGGGRQSRAPSLSSSTASSSSRLGSGRIDYSISEEPPLDPTAGGGLMRGGRLPSVQQGEEEEEEVSGGGSGGKTLLDCIPVEREQRLLESCRTSQRRFQQEAENVAQKYAAIENGILEQRGDPVTANDAVQELLERARRLCRDQESKRAELEANYLESFNLAKENGTDDAEKQNAINRLTKLILESEKLVPMMKDNDQQTMAIVMEIARAKASLLTNLRRRLREVSRLQTDIGKIRKHQELVGMAWGQKEEQFEHLGKVARMPAAYNAFLREVLRQRRFHQDFEATVSDFCTSVAALRSSEMVSRQEFIQAHLGNLPPAFLEMAPGLRNMPPTFNPGPITPLRPGELPEVYPDPAAAGEDEDGGGESAIKEAGVAGSGARSVTPEAAAAVVVADTCAASAVVATGSAAISTQTTAAAVDGAAVSTQTAAAAAVGGAAVSTQTAAAAVDGAVVSTQTVAAAVDGAAVSTQTAAAAVDGAAVSTQTSAAAVGGAAVSTQTAAASGAAVGTQTERDGLRPRNEGESVVGDGGRGGIDDDVSRGTADVAAAESVEARGEGNAPVKRVEERTAGPEVAVDRSISALEAENARLSEELAKARSRLEELGTAAAGEPAGAPALESTPSAAELAAIEPDQRPGTTAAAAAAADGGGGGGGGGGGGGVAVVPGAVEGEDPAPMPRGVGGEFARPRRSYSDVVVSGTGRRESAPAATAAAAAAAAGGGEVRGTGGRRGSSKETKNQLAAYRAGLTMLVKLTDQHKLADQQRGGAAVGVAAAAAAEVASAVQPAMIEDPAGGTRATATARTVAAEDPASPSSDYHPSPGSDRPSPTTAVDGQATAGGGGDVGGAASPDAKEASERESTTVPERAQETHPAPAITAVKVDKDHATAKPAAAEATAPGEDRQGGARPAGEDSSSSPDHKEAVSPEREATTAVSTATGGTAVETAGGGLVARPSASLGGVERIRRAADYVATHLQYNHALAAVPRISMCSFSVQGGSPEGSSGGPVVLGFHVNWPNRYLSEDSINNAREALGASPAYILGRIILVEQHVASEEPNPYNVAPNTVYYVLTAENVG

>790

RQVVAVEASSAAYLDTLQRLAQSTHFVTDDLASPAGLAALEGRVGHGEQCRQAVLRIAERCPLPAGLAIPGPGADPSAALAALVGEVGRLKEASVALQPVHQRYRQALDALAASLALPWTAPTEPIPLSAVDDLLNAVADLREQNAWLKTLAEQRPGQRLVAVADFAFGDTAVFVQRMGPAPPTTPLHASASSVLGDSGPEGLRVFEAVHSGAPHWYLAEETTSALFRRYPEPPAFVIIRIVSVEDRVATAQPNPYGLPPGTPYHEVLGEWVYDPLRSDVTEGCSSTKP

>791

MASNHSQSVTIRVLAASTGMTYKIKLLLSELTVTNLRSHLAAAVPPQNQILLLGPPYKVPKNTTLQSEEILNALRLGDMEDDPIQEDVTKPRTLLTRSERSGAKRLFLFSKLDLSENAPDPPICHLEPTTLTLPTEAPGPSPLSLDPSGAPPLHLPLFAFERQFMLHMSQGRVLADGADLRMAACRTCVQEQAVMARSLRAAVSNLSDHYHGAARTRAEFTANFQAKTSAHGALLQRFDAILSNLATIPLHPSLVSIARAAGRNMENLLDTVPVERERAWATQCLHSHQRLLSLFGELEITFNQLASPSSREDIAQRDHDAENEINNLWSKVEDAVQNINEAQAQRLEKLTIAHRDAVQRITHAMNSQSDDEIQNVFTPLREMSNSTKDLVSQMLKDDEQLKELMASVAGAKNRMMRRMKARLREVSVAQSSIQKVLSSVGVLREALVQQAENMVHLEHVAELPESYRVFLLELRRRRAYGQAVMASATAMMDRVAAMRDDEVRAREKFLRGAGRHLMPAFYDIFVPTLATPPPLFTPQLPALVELDTLPNVGMSSAADSAADALMHQSATVAEQGGGSASTLTAESQHPQQSSNMMTSAAAAGQEPARQLEHLIVSADEQSGDEIILDPASNTAIEAEAKTLAYENFILRQTLERLGAKPPRAYIEEARAKDGAADVAGEEVVAVRKELEKTKILLEKATHQLEASNNLNDKISHSSFRIGDVGLFMPTGRGTGGKRMYLAFHTNCPHRYLSTDCIKGSPDFVLGRIIYQEELVAGEIGTDANPYGLHVGTKFWVLTVEVLKSSQ

>792

MGLLLRRVTNLRSHLAAAVPPQNQILLLGPPYKVPKNTTLQSEEILNALRLGDMEDDPIQEDVTKPRTLLTRSERSGAKRLFLFSKLDLSENAPDPPICHLEPTTLTLPTEAPGPSPLSLDPSGAPPLHLPLFAFERQFMLHMSQGRVLADGADLRMAACRSCVQEQAVMARSLRAAVSNLSDHYHGAARTRAEFTANFQAKTSAHGALLQRFDSILSNLATIPLHPSLVSIARAAGRNMDNLLDTVPVERERAWATQCLHSHQRLLSLFGELEITFNQLASPSSREDIAQRDHDAENEINNLWSKVEEAVQNINEAQAQRLDMLRNAHRDAVQRITHAMNSQSDDEIQNVFTPLREMSNSTKDLVSQMRKDDEQLKELMASVADAKNRMMRRMKARLREVSVAQSSIQKVLSSVGVLREALVQQAENMIHLEHVSELPESYRVFLSELRRRRAYGQAVMASATAMMDRVAAMRDDEVRAREKFLRGAGRHLMPAFYDIFVPTLATPPPLFTPQLPALVELDTIPNVGMSSAADSAADALMQQSATVAEQGGGSASTLTTESHHPQQSANMMTSAAVAGQEPAQQPEHLIVSADEQSGDEIILDPASNTAIEAEAKTLAYENCILRQTLERLGAKPPRAYIEEARAKDGAADVAGEEVVALRKELDKTKVLLEKATHQLEASNQLNDKISHSSFRIGDVGLFMPTGRGTGGKRMYLAFHTNCPHRYLSTDCIKGSPDFVLGRIIYQEELVAGEIGTDANPYGLHVGTKFWVLTVEVLKSSQ

>793

MCSKSKISFTSFEAGDTALFLPTSSGNFIAFHRSCPFRYLSQESLEAAKTKAGGLVDYVLGSIVEIYQHFAGEGEDGNPYSLPAGTPYYICTVIVL

>794

MLIPVCSALSGRPLSLDLPGGSLVEELYSTIKRQLEIEVENQIVMCEWEPESGMNQRKTLSSYGLPRQDNQVIFFYDKSWFKSGLAGTKTEDEIIVIKAQLPNQEKSLSNSAFMSNQDSGEPMQSAIFNFYARFAHWKATADAFLVAAKAREKACQDIRNEVKMQHRGSALARKSLMCYFSAVRKSIEKLETELESARLSTEENLKRSEVDACLEKLQMTSLHKAHCAVWRVPEGTKLISFVKDTDLLNKCVESCHSLVSVLESKVGECIVAFQELEDLVNLEMTNGDMSERSTIAEVESFLESMSSERIEIENFNSAISEDVRELEATLPDLSNGERDVGNLLDLMNKLQSKYSQFKDGFNELSTHDNSHQKLMEGCSLAKKQMTEGILDKLQRVATLQGRLLDTKKKSDALLAGAVNERKRRINQVEIVLRLPETYKAWCEEVHKRNAYYDNLVANAEHISGAVQDLVDNEEERRLEFLEKYEAYLPAPLLDLLNEGASGAAGHPPRMEIHVSRRPKQLPEIRLRDLEEGAAGNFQLLQITRERGDASGAKAEGEQEEAPRSEEPDKRVNQDMEAVKSQMEELENELSEKERLWRKANEGKAELQLKLNETIERVNMERAQREKLQNALIRIGSLLQIDLQRQTPQSSRLRKSEDRDIMDPKSLENVISSIGDLVEKSKSQRESETFKNFSELQMNAQKSEAQISKLEEELEKKNKLFSGAMQNAASLEIQNKKVGEELQLEKSNREKLLSFASKLSASLGMEVIKPLPSSSRALRGSEDRDLEVKLDVRTNMLQRISELMGKPEERKENPGQSIKISLSSFEPGDVALFMPCGKDRTDAYGNSLYVAFNVDCPRHFLHTSSLEEFFNKDKARAESYCLGRITVCYEATEDDFNEFGMTDEETFHVCYAEPVLTE

>795

MFNLKILRSDTGSLYEIPVNSSVTINTIKESLASVTNLSPSDQILLADTNLSSNNTLQYYGIIKDTEIFLFNKKILENMSYLPEEVNFSILDFQIPRLPTPKNLKELESSLNPLNKMFTLEFYLNNHVVGTQYLKELFEAKVLQCINCLNELNVQKKSIGAALHSLDDYKNKLLSNYNSFLTVFKKQSPNFESLLMSFEYDMNRLKQTKLHDSLRTPSTTTLLDCIPELEIRKWSEQCKREYDNLKGKILEIGSQIDSIRDSVDYELKKTIDINFQELNDRLIISRENTKQFQTLYQASIANSEKIKKALESSRSSKDPNHLAAIIMSFTEIKNSQDSSFSDSHKHAELLMNTLILFSKAKNFMNQYVFSELRNISKLQYDMRQLINNFSVWNEAISKQTINFFHLECIHHMPSAYEDALNETSRRKKFGNSIQSNLGKFIESLNGIREEENQKRQMFFERVYQFLPPNVFISLKDQLPPFQVHLPAFDTNLPQIEQQSKSDTDDDFAVIDSSDTTPLQFRKKPTFERTSSSSQLDQSKLIESQQKDKIRNLEQKLQSTFLMASRSEEKYKDLLEKSRTFQNDSVTSDYIRKSQVEELKKELEEKKLEISKLLSEGKTTQKNIETLQAHESELSLKIRDLELENTYKKKEIESFKERILSLEDEGSSKLEASESLRKQLDSIKSERDELQAEIKDLQEQITRHEEYIKMSISSTNESKESFENTVLEKNNTIAQLEKSKKNMEIDLQTANMKISIHSDRIAVLEKEKSELEEERNTLVEKWEDQQEKIKLLEEKISNVESISVEKSREDQEEINRLKDKILVHEGTVKSNNKQISDMNGEIQSLQSIITENQQEIDKLRETIDHNSLMIEQLKEDNEKLSKDLVNSNHNSDSNEAVLAILREQINDLKNEAQQKEAVIKSSSDQLRDNESTMFDLENSIKNYKKLLDEFNDDKEKQSGETNSMLQSKAETIIKLESQINQLDKQIRDQDATIEQLQSGNSGLTQLNSQLEQKIQERIAQIEEFKQMLTEKDESLRTNKKESDYLSRQTSELENKVISLNQQLEQTKTRVDTLEAQNVSLESDLDSSQKEQIALAEKLNQVSAASEIETENFTLSLANLEKKLKEKESQLERASNGSDELTRKYNKLQISSSETNEEMLSQITSYQNEISKAESEIEAYKQTIAKHEERISYLKDQCDTTNVNSIQQVEKLNKEISEKDTLLTTLKQRHQKNESQLSSLNGELQASSTLIQKIYELLGIAPNMDSQNALKEIKHFRENFLHLERSVQDHQKTIQTHEHSMMDLLMSQTSQSDAALSNFGYGKIAIFQYLKSDVYEAVNVNCPHHYLSPESLESFDDEVRAKKPIIASIIEVTKVDSLPNNRYGLPNGYVYYEVLAGRVDS

>796

MRLYRAETGKRVQIPQLRPNDGLDTLRVAIEKATRIPQASQILMTDTGLQLKPDMMFEAITSADKDEYTIMVYNREVLTTDISSSILTLTEQAKTEPTVQPLSPTLLQPIAIPSRPASPSANRDWGSQYQRMFEAYIAYVLAYYRVIVAHAGICERIMEELRMQSLAVQVALTNLDAHSQSVLETFEKFNAFATKELTKQARLLQSFPRDIEALRQIRVHPALLPPDSPDRYISDFIPTDKLVLWADRCRDIHEGLLRDGKDLSRSIKEVQEGTVAIRSNSGINLEQLEDAMTEILQTVEQQSQIRQRVERDQTRVRERLAEISKPSAFSSSGPTLEALGQLANVYRTDYMEAAQRADEMLRNKLGIFIAAKRSQTANLISQLMHISKLQSTIASIPPSLGSLDDSLRKRDADFSQLVYVQRIPIAYGALIVEIVRRREYSKLLLQKSQQLAEVMSRFRQLEQRRRDSFRSEVAKFVPVVVPGLDDVPPFSEINALNTRDRLPPFTREHVTEFERLISQLSTGLGQEGSVEHSLGTESNPGASSISSQEPNHDALSKLRVTLVKMTTQMDVMGGEFDRILEKSFYTERIQRLEEENSRLRADMSRIDIQQRSGTPQLAQHPFPRLSTPVGTASVGAGSAAAAVGMSVNTPTSPKLTRQPSRSGSANTSRVESDEQQQVIQQQTHQNQKLTKENADLTTKIKAYEVRIRSLEETLYQNFNFRAGSTGESSISSKDSRQLLTAGSDQPWKAPEDIQYARSLSQAKEIERKQAEERLVTLELELQDLSLKLEQTEEKLKEEVIISQQTATANDELRQEVLELKLQSATLEEASELRAFDHSGIQKRSEELEEELKGMTQKYDHLLERHEKETETQALEIVAFKDRIEELNKQRSIEKEILEGQLEQAQRTTDEASERVIELENELIVVNEELQNKVVELEEQLEAHAAEHEEVTARAQEQERQLASHRAVHTDILVQLNDHKQKASDSQEALSKVKEQYTALQEAHAKLETDSQTLQRDAHETSKSKDELAQQIAGLQKQVSELEAQREAYNVGVNLRLEEAKTMVQRAEEEWKEKSRLLEQSERATKDLAQPIKECMTSLGHESVSVEVVSLGQVRDMLQDIGRDIQQLVASQTKERTESQKANDEAISALKQEHETSKDILETTIASLNKVRRDAEAETVAVKQESEAAISQLKNELESIRVQQRASSIPVPVSTTPISGTPLTSTSPVTPMPPTLPATTKLALSKDGNQFSVQDRILLGTLALDLGIPLPLLSEVDDQDVILPTSSILLDSSERRLKSNSTILTPVPVQGGSSSTSITGSTSRSSSTTVPLSTDVLQTLDFSDLDIAKATALIKKKLFDAEHLLKRWQRECKSLKEKYNRASTEAHDKIAFRNFKVDDLTLFLPTRNSISKPWAAFNINFPHYFLQMTPTMANQLRNREWIVARITSITESIVDKRQGASTVEAEADSGASSSITPHNPFGLADGVKYYLLEATSWSSHSHGHGHGRSASSHNSSTGGSSSGKLRHHSSTSALGESSSSSSRRDTDRSREHRDRDREPFDRRKHRDSQGGLDEERSTVVTTPSKPAPVSSALVQETRSDVPSTSTSLPPTGPSPTAIPSAPGTSQRNSIGASGTLPISIPYQSPAANALRAISSSVGSTGSGGSGSISSLLSHHLGGGSASGAGTTMAASSPPRTMFLSSSPHKTGISPVVATGTTVSINHSSATGHSASHSTSLSQGGPTSGGSGSPNMGSSATGVFGTSSISSMSNMPIHPSRLSTSSNREDMLEQLAVFATDEDQELDREEEKKRIAVARTTSSTSTYHGHPA

>797

MPRAVLPIKVAETGVVVNIELHNPYQDDSVIDLKHLLASATQVAPEDQILLIGPPFKPLKRLDPPLTFSSSSATDEEVAATQSELSQAQPLSSSSSSLSTSSPSSSASFSSSASPPARSIYLYDRRTLDAPSTPSSALPAPSPHLPVSLPDIPSSPSTPASPRLQSLIHQAVGQGLLSQTLSKYERLVGGNVAKGHAYQDRAQRLVEAAVRAQEEVRIQRDAGQVALTNLRDHFRAISRDADKLSRRHEKQAEKHRKLLENFNPNLRVLEQRPLHPALGTALRDSVREERREGGREGGREGGGGTWSSSSATGSVTELSVSPLEGGSSSSRVSPSPPGVECEALRKACSASSSSITLYDTVPVGRVTEWYRECGETRERLDSWMRDVQASLRDLQAGVEAPSALRVTADMGTAAQEEVDEITRRLSEQETAVALLEGGHAEMLERIETSLGGEAAGPDALPSSSPSSSAKLVDTCQWLDGLLTRQAGVLVSLDSTDETMVGRLKAVFAQKERTARWLRGLLAEISRLQGDIQSLRVNLSLGEQAMQAERRHFYHLEHLEHLPTAYEALLLELARRRAYARLFDLKVKSAVDGIARFREEEIQARADFLRRHASHLMPVFSQIFAGMQDRPPHFQPKPLAMEARLPVVRIAELLVGDEGEKEGNEGDEEEEDELLKGYVSCLARVSREEGADREEEGKEAGKDGGKEIMPGAESTPTLEELEERCMTQDLVIAQLQTKVQSLNLHHRAPPGRKKSGDAGVGGDGEGAGARLQRVEEENSLLRSCMETVLNVMEEMRQCAASTGGSGSGCEAEREQAVTRAREGGREAGKGAGSPSPSSSPQHLVNAVDDTASFVKARLRDLQAELQASRGEGKERGKEGVVPCTNMSPSTPSLSISPAPSSPGSHAKISFREFNVGDVALFLPTSHQGENRVYLAFHTNCPHRYLSHESLESLRGGREGGGRFPDFILGKIVYVEEMVAKEGEPASNPYHLQAGVKFYVLHVSNEVV

>798

MTFIEALTGRIIHVNENTPNEVIPKEIQKETLYLPLDQIYLLNNGREIRFNEINRFSNEKIFIFSREILEKGIPQELINGIKLNNNNIQTLIDEIKNQHEIWYSLSNHLREQLIEVQEKYNNYNQSIKDYFVKFEFLLNCYDNDMEIVQELPASLINSKQITDAIPREKIDLWKTRCQQESSKLMNEYGKLKILFNNLYQNSYNELNSQSIKKPDYSNEMMSILFDKIKRIQTISNDKYIFDMDFDKLIEYMDTIDIHFNQLDYFHNIPIFWYATLLEIIRRKHYNNLIDLKFAVFKEQLYDQEQKRKNEFKKRFQQFFNLNYIELDDKTSKFTPVIYNFCDNFPLKEYIEKNEILINKIIKNYNHEEYQNFGTSLYPIIEKMKNYIQELEDAIKNENNKYINDKEKNTIPNEYYEKQIKELKLKNSQLNEKIQQMDKTYEDEKKSLLVNINKLKEEKQELTNLLNYEKENSQKLKEENEEFKKTIELLKSKLDNTKESLKNKENEIVNNSNVINSNNSMNGSSGLEDWISLCHYSIEQLMIYYKNVCKLLNQDNFNEPETEECQDLFKYVQSISSVLENDHIQKLKVLQNQKEYKLKFNSFETNDLVLFSKTNDPEYYSMFSPLSDIPYFLKTSEHKNIINSTPYLLSYIISIELRKSDEKEIPLEKDQPYYLVKVRPYS

>799

MSSNMPSTTVGPTSIRVLVAGTGVTYKLSLHPSELTVANIRGHLAAAVPTSDQILLLGPPYKVPKDSTLQSDEVLNALHLGDAEDNPIQEDTDAPRNILCSTERSGARRLFLFSKQSLSQQAPESPVCHLEPMELQIPKEAPGPSPLNLNPTMSPPLHQALAAYERQFMLYLSQGRVLADGADLRLSACINCVQEQAVVARALRAAVSNLSDHYHAASRTRAEFTTAFQSKSNAHGSLLQRFESILQNLDAIRLHPSLISTARSSGRSIESLLDTVPVERERAWAQQCQTSHQRLITLFGDLDTGFRDLGTPASRAEENRQDREAEEEIQHLWLEVDGKAKGIRNRQASRLDGLTTSYREVVKVIMNAINAGDDDDVQAAFTPLRAMSDVSKSIVPSMITDDEAMKMLMEKVAESKTRTMKRMKVRLRDVSVSQSLIQRVLSSVGVLRDALSQQVENMVHLEHVAELPDSYRDFLSEIRRRRAYGQAVTSSCAAMMEKVASIRANEVKAREKFLRGSARHLMPAFFEVFAPTLATPPPLFTPQLPPMLELDTLPDVGYDTEDPDTLMQKRSGVNEQGASSASSLTAETSMMASVAPAGQVNMTSSNAPMSQEQQQQQDHLIVSADEQSGTNFIMGTDGGAVAEAEAKALAYENAVLRQALERMGGKPPKTYVEEANLAKLGDQSNKANEAKLMKLEKELADAKSQALLAKDATRTTLADSKLSDKISHTSFSVGDFALFMPTGFGSGGKRSYVAFHTRCPHHYLSSDCVKGSPNFILGRIVYKEELVAGESGTDANPYGLPVDTKFWVLTVEVVQPKP

>800

MDVARTHTDEDEEHKARGDIVVRVGDASTGTIHAVALASANVVVEELREELERLSGVPANDQILLCGPPFARLDPRRAIEYYGLPAEDKEVFLYDRRLLSQEEAALPPTATPIAPVHVRLPSEPVASSEGSRMLSESSHPMMRALVEYEGYFQLQVSQSEALETGTRANIAVSERGTQELQVQERAIAAAVANLDLFKTSMMKHFAPFWTDFQTTSEKHERLLSQFESYLEALATVELHPALATEERKTLYDCIPVEKEREWAVQCEQSHNHVRGQVLKLQRVHDEICKEVEAMMTEHADAYREYHEASAELEQMKVLGNKQMNITSTVRHNLQYVLSSIEETSSIASGSNPMQASTNALDVCRRIDELYQGQQNTIPDAQKLLEDIVGHVHKIADRKSATFERVHSTLRQISVSQSKIRDFENSLSVFREALTAQKKHFYELEHLDKLPESYAACLKEISRRLKYGRMFSDRIQSMAEELAQLREDEVQNREEFLRSFGQHLPRDFVSGLAEKPSHCEFRMRPFDQSLPLIEDEDDQDFDPYEDKERPPSEEFVDCEDHDSSSESTSNVDILQERCKELEARVSQLTAELEQSKKNLYYDGSGSEMSKTSAREGDSSCEFPLVLALAGGMTSSSEADRSNILDRELATVRRNIGSGDENKDATIAKLESENQQFLLNAAAFEEEIQDRSKREKQLKENQIKLQETIKDLSSSVRSQRTSLAKLLQLLQLPEELQTDGDENIDAFVNTSFDAVATRVKELLANSKSEASELDQQRSELMLMGSEQDVNDSFKISFRSFNVNDLALFLPTSAPGSDAQRVYLAFHLGCPHRFLSEESISSFSNDGQRYPDYVVGRIVLIDEQTATEGNNPYALHLGTTFYVLTVTSLHES*

>801

SFQISSLALFFLKGGKLPYVAFNEGAPNYYLANESIQAAILGVGSDRTPPAYICGEIIFIDTFQATEDFNPYRLPYGTRFHVVTVANPKRT*

>802

MISFAHRSTPSSTTASPIAIRVLAASTGVTYRISLHPSELTVANIRNHLAAAVPTSDQILLLGPPYKVPRDSTLQSLEILNSLRLGDKEDEDAMNERSGARRLFLFSKRALSENAPDPPPCILKPMNVTVPLEPQGSSPLDMTQSAVASQPLHQALSGYERQFMLYLCQGRVLADAADLRLQACIQCVQEQAIIARALRAAVSNLSDHFNGAARTRSEFTSLFQSKTAAHASLLQRFESTMANLGSIRLHPALVAIARGSGRIMESLLDTVPVERERAWAQQCKTSHDRLLTLFSEVDAEFGKMGTPASREEDTRKDWLAEENIQQLSDEVQGAVQDIRDQQAQRVERLTADHGKVLKIIMDAIRSEVEEGKEDEVQNAFKPLRQISNDATGIVPDMYAADETMKVLMAKVSDAKTEAMKRMKVRLREVSVAQSSIQRFLSSVNVLKAALTQQCDNMIHLEHVAELTTSYRDFLSELRRRRAYGEAVTANSTAMMERLAVMRADEVKAREKFLRGPGRHLMPPFFEIFVPTLATPPPLFTPQMPALAELDTLPDIGLANEDAEIDSSMQEGVTDAGGGGVSSASSLTAESNNNVQQEKIPSSGATDTVVETIPSAGGPSHGHNQQGDQLIVSADENSENEDTVMDPVGGTGEAERKTLAYQNALLRQTVERLGGKTPKAYIEEARAKDVANTEIEALRKELKAAKSKAVSAEDALADALNESVANTEKKAKEDENKPKKECDKISHSSFEVGDVGLFMPTGRGSGGKRTYLAFHTKCPHRYLNTDNINGGPDYVLGRIIYQEELVAGQTGTAANPYGLHVGTKFWVLTVEVIKS

>803

MADADEYSTAPTQQEITPLQTTATIINAISGECITTNVDFFVSLDKFKQFIARKWKIPPDQLLILLPYGNKLKPSMFKELLINRSFTLNDFYVYDRRLFSLVSKPTPTNLLTSKDSNPMNSPNSNDLTETLEYLIKNSHISQYQGSDTIMIKPMPSPLEDADVDLSRLNYHSVTSLLTTNLGWLSALEIDVHYFKSLIPDIIAHIKRIFDGLTVCSQYLKLYCFDVESLYNSNVQFLNQLVDNGMTSKWEKCFNDTLSKLTALEGDSLQKFINIESLLENEKSVKILNHSINGKLNKIKREIDENASFRDIITVNIDRLRQMFTPNESKFELEDQMAESFEVLVSEMRTRSRNVLDKEEEEFNSQEFLKSMNVMLEKDKKESVKTLFTISQALYSQIGELIDLKKSLQKHAVAILGNIAFTQMEILGIKRLLLNECNKDLELYKKYEVEFAQVEDLPLIYGLYLIEKYRRLSWFQQILSFISNFNQDLELFKQNELRTRNKWVKNFGSIATVFCEDLLSSSDFKRLNEYHSHTSPPNEDEEDENENSIANYRQDLVKVSQAIDNYMTQIKETDVSEPIIDLLSKTLFETKRFHIIYSNFKNNNNNSSNGNSISPEGSIALKSDDVVKGYKTRIKKLESLLHEFQYSDIGHWPQGVLNTHLKPFRGSATSINKKKFLGASVLLEPANISEVNIDSVSQANNHQIQELESNVDDLLHQLQLLKEENNRKSMQISEMGKKISDLEVEKTAYRETLTNLNQELARLTNEEQSHRTEIFTLNASFKKQLNDIISQDNEKIEKLTGDYDDVSKSRERLQMDLDESNKKHEQEVNLLKADIERLGKQIVTSEKSYAETNSSSMEKGEKFETIPLAEDPGRENQISAYTQTLQDRIFDIISTNIFILENIGLLLTFDNNNNIQIRRVKGLKKGTAQSNILDESTQMLDAHDNSLIKSPVFQKLKDEYELIKSVANGSEKDTQQSIFLGNITQLYDNKLYEVAVIRRFKDIETLAKKLTKENKIKRTLLERFQREKVTLRNFQIGDLALFLPTRENVNSVGSMSSSTSSLSSSFSSVDLSTPPPLDAMSIQSSPSVIHSNVINQASISGRDKNKLMRPWAAFTAFEESTRYFLKDEKGLTKGKEWFVGRIVTLEHFVADSPSNNPFRLPKGSVWFQVTAVVVSYQGV

>804

MESQCTGLMAARDEVLRAGQDVYERFVRPYDKTTKESTATLNQMRIYIDMLSRVYRCHPFSLPCFSPNRTPSTGDTSYKEPFEGFVKPHRRDSKRSSEFRSSGSSGSKIGSNGSTRGMNSGSGSGEGAGGNDEIHSYSPLHFSFMTAESATRGRPVASLHVVEESNLLGMRIRVEQANMKLNNMTGRYGVAREKFPDLKKWKDQLSYSLEPMTTAVATLRERIEHVMVLMSLQNGLLQQVQGIKGIDATSLLDGCHRDLNQRESLLVAMATNDAKLHEFVRRYMDRMVHVGVEIHTYFQTIVAFNRVLRQVCSGKSLETFPLVQNHMYETLSELLFIPRQLTAVLNVLPEAVARQRYTQLHSVTAISSQLAHASYAEEIRRLEFTDAHVHIVPDALLRLLQESSPSLPLLKVVRADVKLGANSTKILDTGANGSSHEEQGEIPLFFSWDDVVRLQKELGKFGTIADDYVSLLRDCLSSMRVGAEAEDIKYPALSDTKSISVMSHRDTFVHARDTHGHPSHPYVYSGQAGHATQEAQTVGDSRGSVDMRSGLLGGMRPASGVEKDEHGHSQLKGPSYSGYGMNSQVTVDPRERVGGDRVRSDLVQPSEETHNGWDTLEQSYLDIADEIEGSPSTEIMRLLTDEHCHSPAQNQNLAKHTSANTEGSYADGKLESSMDAPNSRDVDRPGQSGGMSNQDECGTVMTQSMVKEAYLMNELCAEMVEVGVHDQGVGAATSACGAVKTNISHDAIPDEEETGEVVPLLGGPNLGFRAARGVSPRVKRVQNFPEHTSPACECERDIACTCASTRPDSIRALLSMGKHTQPDKNLSTDTNVNSQALQREVAVLLERVSSMVIVAAEREAKLKEMGEELQRSCAEVRLRDCKIALLNSELERQQAASAVIYKDVAMATGDKSSVGGTESTSDVLVASLDESSEELSSEYGSAVSSTLEGSDCGVLVSRSEYADIGGDRDIQSETGCSMRTDTGTSVELAVAEGTQTDSPLCINSCTQTFTRIYADTCTQTDRTENLGPCGEVTERQMLLAEPDSKVAYTDEVSRRHLHMLQHQLSVYCGYVAALAQTCGIECGNRKCEDERDGLGKHMDREVGSDDVYAHIGSGTHIKTDDTRTRTHPYAEPSSSHDVKRGDVEVVERNELCGRERWASIARKLYSIPLNFTLENPDKGFNGTHSVSPNKEELAYADDQSEVYNSFSRVQSKHENGFAASQTDATDTRSEVECLDALLRCADGLPLFTIEAHVRKLSEFASTQVHAITDGKYRSFGKDNKNLFSNLDEVTRAMLRTEEGKRVLIDDVSLSGDSTLSVSRGSGGPNYAEDPEFLDSNTNWSSGSAVGHKLLGVVDDAVHRECGTDSGTDKHLPTTRETMQGETHTPTSTDSFTQAPISTQAYISCENTFDGTQRQLDTKISVQAFDTDCLMLFTPLNSGHWFAFNIDQPHYYLHETCVADYQLDRKIVTMFVGRAIYLQEHRASATYNPFGLSEGTKFSEVIAIEEAKGMKVRRKSSSRHRSGEADLSNSPPNNAPKRDEIAPLTPNYAETMMLSMI

>805

MKVYQAESGILLPTRTFSVTESIDDLRQEIFSLTSTPPHAQILLTPAGVQLKPEMVSEIFANATNKDESVVYVFNRQLLDRKTVMASLMPVSPDLEPPVPLNVLIDINLSDTTTLLDRCNTYSQAFRSHVTYGQATLATLVYHVSVCERSLNEQKAQVEALNIVLTNLKGHSRSLCEAFDAFYLHAQRELAKHTNRLQSFPTDMQALHRLPIHPSIADGNKYLSDYVPEDKLITWADNCRVAHGQLVRKVTALAEAVKSIKSGTDSETSQPFDVNFRQLEAFLADVKDARQRVESKQQRLERDFSRVEDILSEMQASTSSVVSDKITSLDHLHKIHRTEYLPKITEIDTSVRQMASAFIESKVHLSQQLQTRLQSISHIQSQIASVTPTLTGLTNLLNAHIQAFTQLLHVHRMPAAWGATLVEIVRRREFVRVFLAKAKDMAEILARFRTHEEKRREAFKNEIGRYLPPDLVPGLDDRPPICEVSVSNTKDGLPDISREDIADFEKLVTSIHSSMVETEPIGPSASQTHSNHTISKLIATMVKMASQVDATPADFERILSKTGFSERLLRLEEENMRLRAQLQGAADVQPRMLISRSPSSGPSDMRDASQVSMAQMEVIALRNKMAEYEARCAVLETRSQEYDAIAARLVETEQALETERRNTTELRATVYEIGREREQWLRERQESIAMTTAITEENNSIKAEVEKSRSFYDEVREGLEACGRALHRRNEDESTGQNTDSQPDMSSTSTRANATPDEIRRALRELQDDILCQTAQLASMKASITEGDSTSESVEGEIVALLTQVGNLREDLNAAQLHVIELRDHLTATEAREAIVEADLHSTRALLKRAEEDLAMARQKLKSRETDLEVCQGRLTEKEHQALTVQVEADWLKKRVNELEDVVAGLQEDQKQLNDELASRQAELGATEADIEQTRATLAEARQQVSTLMENLHVAETSLLQRNEEMQTMEKEQQNLAGHVASLTEQLARQGEWLNEKTTEMERLVREVGDKESQLQEAVEKSTSLQNHVQEKISEVETLRTTVSQLERNLANVQLEIAEQTQMADALRKQLIDTEGDLAEQTQMVDALRKSLTAIKEDSAAVQIECTKTQGNVRQESEDKSDPIVADIPKKQDCGPVLANTATYMQAAIGQLIAYHEALYSIVTEICGSETAERVLSHASIREILSADGITADPEIPDVLDRVWLSRLSDSIKEAHEKLIQIGTEVDPANAWTQVLERWKDLMEREVPHLRETCVKAKEAGRNKITFKNFRANDLALFLPTRNPKAWAAFNVNSPHFFLSPEVSHAFAERMRNRDYILAFITDIKEHVVDPGDPTSNPFGLATKTRFRLCMARAWEPSK

>806

MNIALRYPETGREIKVTLSSGNLVDDLNSQLVISFGADSESFILLNSEGLSLKDKMSQGAFTESIYVFLKDSIRKDKQDLTINFPSQQTPLELPLQGEERGVLAIEKSLYKFYTKSIEYTDFFTSYELFTNQIENEMNIVACGKQVLELYHNKHIQDQVDKFNTLYKKNLVHYESALGELSAFENSVANLTSFKIHKQLQYENRETLADLIDTVQLMKWKESYIAETGRLQTKFQELEKNIQQLPYFYQLPSLPNSPLKVLNISEEMKIAINFGSSVYLDYRQLCEKYNSTGDAQAGKRLHEEKWDKKIAQANYFLAYVETNIEEYRKSIEDIKQQRKTANIQLFTLLKKITEYAARIRDTIKSQLSMLSSLLKRSEKRLAFIKVPKLLPEAHDSAILEISRRNHFVKAATELQNRLSELIEIEIAERISFLDKFKHVLPNNFVPQLSAGPFIKIFAPADEPDLSLPVISDSNFENIDDYFNAYMVKYCKDEKFLLEQENYKKIKALKEVESEIQSEKEKLYEQNKKLSLEIKQKEKVMMDTIAILDNFNIEFKRKDEEIESLKQKIVHEKASNEEKMQSLAQLLSSEKVKSQEKFNEMELNFKQALQDAENNTKSIIQKLNESEKMKNLLKLELELEFEKSQKEEHKKVVNQRSPEFENLCKELKIEPSYLQLKEYILALQQSESSKICFTSFNKGSLALFFPTSEGQFLAFNYNCPDHYLNIDSISGQSIEMIQSQPYIVGVITDKKKIIAQRNNPFMLPLGQEFYLLSIKE

>807

MDLGSTMGRVGNDPRGSIIGPSFIVSVDDNSGHDDVIMRTVCHEGSDARREDSNAECATLLYENATLRQALERAGGKPPLLYVNDKTKQISMDVAEKNAKISSMEVELANMKLELEKARRDTQQSRQSQKNSATTQTEVNQCDKISHTSFQVGDVGLFMPTGRGKGGKRIYLAFHSGCPHRYLSSDCITGSPDFVLGRIVYQEEFTAGALESDSNPFGLHVGTKYWVLTVETMKRG

>808

MRVIRAYDGRSIVVNQPLSQFASLDDLLDCLSLATEIPSDSIICMTSDGSQLKQELLDRIIQQTATSPSESSSAAVVEPNPSSSTHQDAHHQSQNYEFFVYNREFLYTDPTELAAELAEHTVLEPVPPMLDVELTFPPTPKSLEALVSWSAEIATLVATYEQSCHDLLERITLIARSLQVALANLRDHADNIEKGANALIDDVAQKELKRMHSLLQGYERDLCILAMVSIHPRLQSTSAQMANAAATSSTTSAQNGNGPANGQSGLTKPKHRTLGDYISKSKMSAVADACHRVYTELRERIERIQTELSLVRNDTQGLSEEVQSTTPEVADETYQRAVEAQLRTQQLYAFICSTCSPDPQGWPVADKIDQETFDEIVRSSNELLLLDEVVREAVQRLTQDRNDILERSLHLLIDISAIQSEFTETGSLLAAVDADLHSNKLDGFKHLHRLKNMLWAYGATVVEVVRRREFAKHFLGKSQSLAELMAKVSASEWKRRQFYRSDVSSLLPWEVKGMDDKPPSLEITTPRVSSEGMADLGLADLQALYELLDHIDYQLREDGIDGVDSPIPEVKAALQALAAALQDLDDEFIHLVNHQLLNRQEQEDEASDDQGDESGSDTSIRARQRLRRRQRLSAPLAAANAQELTQLRSELAAAKAAQEQAEHGLKAETETRLVRLKTEIETLRRQVQTGKADVAKLEAEKREAEARIDTLAADLELERERRLNMVDEVNQLRRDLEQSHRIEAQAKQEVIEEADRVAELEAQAHELHVELEEAKRARIDASSRIEALLSEGSSFEGELRTAQARIEELTEQLSNAHAEAAKAREAATEVETAKERQIRSYRAEADGDRAILEEKVRALKADLDAKTKLLEKQTSIATRAEQSRIPDREAIDLLRGQLRAADVAHEEIVKEMDHAKELANEAEASRREVEASRQLLLERSRGLLAKTSLLRKAVRDMPKHTSSRQALSSEAAQATDKNNANAASTPALAASSGAVVAAQRAIAASTGSLSVSELSESQRQAALDAFDAEAEGATLETTLEALRALDVSEMYDEIKGKLDSLNIVVRKWQKAWKSANEKANKANHAAREKIAFKNFQVGDLALFLPTRSSNLAYKPWAAFNISFPHFFLNAKGALAEQLRTKEWVVARITSINNKVVPTASQVKSLIQGESDADANPFRLPEGVRFFMLDVETYGGHSSAPPALRSRKSSSATVDGGNGAANDPSSHLKDASSHPRSRSEGTLTGLKGLVEEHKNAPGGGAEPSVAKPSQLQSIAAEASKSTTPKAVDDQQIVPDDEAAGRTSGFSLHSDKIEAPEQSTPPMQTPDTSPRLGSTAVLSAPSLAEKGRSKAQQDDNTAETRSAISTSALTRVMRSASSASSFRTEGGISAGGVGRWRAGSSASANAAHWPSRSITGIAEEPTARHRDGNDADTSSSTDSTSHRSTLIEHATMTPAFGRAPNKRRFEAAGRTHNESRSGRAAAAADAAARRHGESQSPETGLLDGCNARSAPASQSRSEGITIRAPQPLHSDLSDTAVSNPFSQSPGPASLPADREGMGTLLRGQTRRKSSLHITSSASVDDEVGKREAPKVQRWPSDAVSKASQRTTSSASTASAPSQASGGIRINGRSIVVDAAVLTGAHRSEASSSSVATTAVTAAHAIPGAFPSSFSSSGSLASPASPSVGQGQTEEAGLSSSTVRQAWRSPRFSGAASPSRPTLSGTQKPSFLSQTLGRFTGAAAQAVQAPPFAPIASESETARKRRVSRPSLLQAIAPMQDAGGAEAEAASGTWQQTHRGTPESTASGVSGFSSMSGNGAQGMLKRLQGQQGSLR

>809

MADASAGGEQAAQPPVVASAPAAKPVEEYMKDGKVVIMFKAVGGASALKVNKFKLQAKASFQFIVDFLRKQLRCKPTDPLFLFVNGAFQPNPEEVIADLFKCFHNSGQLVINYCTTAAWG

>810

MSAPSTPPSTSPAPPPPAGAAAAKVVLRFHAIGDAPILRRPVFRVSASQKFAAVAAFLRRELGLAPDASLFLYVNQAFAPAPDEIMHNLDRCFADAQDASGTRALRVHYCTTAAWG

>811

MEPNNSSVVLRFHAIGDAPILRRPVFRVSASQKFAAVAAFLRRELGLAPDASLFLYVNQAFAPAPDEIMHNLDRCFADAQDASGTRALRVHYCTTAAWG

>812

PPKPSKIKLHFKAVGNAPIMRKMKFHISGDEPFRSVHRFLRDRRGAGLFLYCDSAFTPSPVEPLDHLFRCFGAGGELVVNYATTGAYG

>813

MADAAHADNKESASASIARLSEDKKVTVKLRAVGGDTPILKRKKFKVKGSHQFSHIVVFLRKSLKIKQTDSLFLYCGQAFSPSPEEIIGDLFNVCKKFHFHI

>814

MEMENITKDAAVGRPTGPEKVTLQFVAVGNAPLMKRTKFTVSGHDQLRVVYQFLRKQLRLRDSDSLFVYCNSSFAPSPAQRLSSLFESFQAGDVLVLNYSLTQAWG

>815

MSRIIHSEDDDDDDVGSQSSSSLSSPSKSLQQDPIPTKIPLSTSIILEKKLPLEQHQKLSNLTEGSTAGGHVSNNSLDNKIMIRFVPIGSTPSIQPRVFKISATQTVSTLNRFLCKKLKFKGVLNLYIQNSFMPLPDEQIGSLYGLFKTNNELIISYCNTIAFG

>816

MADDDADFPSSTTNAGPAAAGATSSSSSSSAAAAAAANPNAAAGADDDNAGAAPSAPSPWTLRKPPTKDKVIVQFRAAGNAPLMRQKKFKITASEKFQTVIEFLRKQLKFKPTDPLFLYINQAFSPAPDEIVRNLYQCFEIKGQLDIQYATTPAWG

>817

MAAAEQEKVVVVFRATGGAPILQQSKVKVSLDSRLSKLVLFLRKQLKTDSVFVYLRESFIPCMDDEVALLTQAYGIEGKLHVSYALTPAWG

>818

MSGQPADSKVVILFKQTGDAPILKQNKIDGQERFAKLVDFLRKKLGRDQVFMYLKEAFSPSPEERIATLHDAFAVDGRLVVNYALTPAWG

>819

MSESKGNQVIVWLRATGDAPILKQQKVKISANEKFSKIVEVLRTKTKSEQVFVYLKESFCPSLDEKISVLYEAYGSEGRLTVNYANAPAWG

>820

MQRREALVVKDKVTVQLKATGNAPALKQTRFKINASDKFAVLIEFVRKQVQRESVADSMSAPLGGRLVDRVDSMSGSQSP

>821

MEEEEKNNNEVASPTEQNTESTTEQQPQLPVKITSESKIIVYFKNAGGAQPLKQKKFKIQANVSFQNVIDKLRGQLKLKSNESLFLFINQVFQPSPDEILGELYKCFSHNDQLIINYSLQMAWG

>822

MDLTDLTIQPGKIHLVREILRDGVTGSVRVLGSLKSYDAAADVAVVDYKGDQLTIDACLLTDFQFRIDSLYEFIGEVQGAAGAARVSRRPGLPRWGGALCSIARGLTAGAATTTAAERLSIGIMDVSVPERGETSRIPPPATPEKVKVHFKPIANAPILRKSKFQVNSAWNCSELEASLRSMLQISDTTPLFLYCNSAFEPSPDQSLSDLYKCFNVNKELLMNYSITEAWV

>823

KVTVTFKHAGDAPILKRSTFKLSASTTIAMTADQLRKQLGIAPHEPLLLYCSTAFAPPLDETLGDVAQCF

>824

MADLEGDAVSSTEPTSAPVSIPGEPGEVPDKVLVQLQPVNAPILKQRKFKLSKDKEFVYIAEFLREALKMQHEAPLFLFINSTFAPCMNETIGDLWRCFQVQGTLVVNYSPQQAWG

>825

MSAELSSKLDLASPATGAATGKKDDKVRIFAKNVGDAPILKTTEFSASAASPFSRVIDHIQKKIRTSAAHKDAVVHLFINQSFSPAPDEQLGDLYKCFQNNGKLVIDYALKEAWG

>826

MSDTIEDDVAGDQSTPQSTGVPTSPTQSTSTTAVKDSVIIYFKNAGSAPALKQKKFKLQSTINFQAVIDNLRKQLKMKPNEPLFLFVNQVFQPSPDECLGELFKCFSYNDQLVINYSNAPAWG

>827

KVVVLLKATGDAPILKQNKFKVNASDPFAKVVQFLSAQLGRKRVFVYLNSAFTPSYDESVANLYAWHGVEGKLVVNYALQQAWG

>828

MSSAGTPSSESPPPTLAEAASTEETITTTTSEPTPAGPRLTPAEVVASRKKKDVSKVVVFFQAIGNAPILKQKHFKITASHKFMVVIQFLRRELGYQPSDPLFLYVNSAFSPAPDEIVSNLFKCCNTKGQLVVNYCTSPAWG

>829

MSSSAVDQQLSSNDTDASSETLSNTNEATTPTTTTEKVESGKVMILFQAVGSAPILKKKKYTIGAASSFNTVIAFLRDKLLRITNPNQTLFLYCGQAFCPNPDDYVGDLFDHFNTNGMLVINYSLKIAWG

>830

MEEEREEDATSSGAPAASPPSVPQAQAFDEPPSMPTLRIEREDDIPSRTSQATSTALTAATPAMCSRPGGPAGEDFSMNASTSTGGTVDLLRPSASTASSPSAPTPQSTQHSASLCPGQTAPAHAARVKLQLMAVGRAPRLTQSKFFVQASEPFRALFPVLRRLLQLKESEALFVFVNSAFAPAPEDKFGDLQKCFSRGGALIINYSLDEAWG

>831

MTEKILVRFRAVGDAPLLKNPVFKVDGNREFSTIIKGLNYKLKQCSNLHFYINNSFSPSPDEIINNLYKCFGTGNELQVNYATTPAYG

>832

MSPEPDPSSEASDERKVVVLFKATGDAPILRRNVVKIRASAKFEEILAHLTKSTRCERAFAYLGAAFAPRYDATIGALCDGYGERNDEGGKLVVFYSTTPAWG

>833

MADTRPLGPTKVTLQFVAVGNAPLMKRTKFTVSGHDQLSVVYKFLRKQLRLKDTESLFVYCNSSFAPSPDQRLSSLFESFQVGDVLVLNYSLTQAWG*

>834

MTENYIQYIPVFEKKPDMREILINRRNQKIKIVFKCISGTTILKKNKVLINGNETFSSLLIFLKRIFNKNDNIYLYINNNIKPNLDDYIYDLYDLYQISGSLNISYSFTPAY

>835

KIKVHFVAVGSAPIMKKKKFQISANLRFASVHVFLRKVLKVQQGDALFLYLYSAFCPGPEELLRDLNETFSKRGELVIHYSLQQAWG

>836

MKKSNVLMTKDKNHETKTISFLSSRNVHMCKSLFEKKSNAISKSLTMTEAQEIENTEEEIPEIHDTEDKKVPLVFVPYPEKIVVHFRPVANAPILKQKKFKVGSDKQFFSLVVFLKTQLKQSPQEELVGCSKSKLLTRYILKYHIYCNQAFCPEMDQRIGDLYECFKVSTELILYYSLTPAWG

>837

MSRILESENETESDESSIISTNNGTAMERSRNNQELRSSPHTVQNRLELFSRRLSQLGLASDISVDQQVEDSSSGTYEQEETIKTNAQTSKQKSHKDEKNIQKIQIKFQPIGSIGQLKPSVCKISMSQSFAMVILFLKRRLKMDHVYCYINNSFAPSPQQNIGELWMQFKTNDELIVSYCASVAFG

>838

MADRQTPVISAPHTEVLVTFTAAAGAPQLKESKRKIKVKSHKAFQAVVQHLQKLLDKKTLFVYINQISPSLDATIGQLAAFYGHVTAKGTYRLPLQYSVTPAWG

>839

MSVQQPPIDELPSTVSGTETIIPKPKLAPKRVAAKDKVVVRFRAAGNAPIMKQAKFKITANESFQTVIDFLRRQLKFNQHDSLFLYINQAFQPSPDELVINLFKVSECAV

>840

MRGLHSRSILTSKRDTPTRLTTLDRFGCVRSPSASIVFTMSDNPTSPISSPPTPRTANPAAKVVVRFRATGNAPILKQNFYKITASQKFQTVMNFLRKELNYKAQDPLFLYVNSAFAPAPDEIVGNLYKCFGTEGNLIINYSTTAAWG

>841

MEPETKAQDPGKSIIVNLKPVANAPILKKTKFKIASDKPFGSIINFVCSQLGGKDRVFLYCNSSFSPSADSVISDLFNCFKVGNELIINYAITEAWG

>842

KSTSRVKVHFVAVGSAPILKKSKFLIGEEDEFGVATAFLRRLLRLDSAPSLFLYINAAFVPSPDERIGDLYDCFGVRGELVVHYSLQEAWG

>843

MASTGPAEGIVPQTRSITDDKVVIKLKAVGSAKLLKRDKFKVSASNAFSHVVAFLRKQLHLAPADALFVYVDKFAPPPDANVGKLFLAYGDVKYLVIHYCNNPAFG

>844

MSPETSPSGPAGLCASQLASEPHSGVWGVSQPSDAHANESSAGRRNSFSSASNVVDPSSGRSVSASHQGDLGELEALSSTSSSGEASSLLDSDSGSASLLDSGDSDSDPADDGFFKLKATLGRDSQGASVHSSEPDEDAAKGRSSASSELAFPYRDSSGERSKAVPTRRSESREQEASNTPEDPAVCGEETTSEKAEKTGQREDPREQRKFWTSVSDADFQKSGSETGRRLEVGRRAPSSACQESKEFLEAHAAADLYLSPSAARSGSSVRPQRRDGMPASSTGIRESQRGTVFPENHVLPMQASQTDASASRNVASRPEMCGPTVTALSSKNVKTRAEGDEENESEGRLQTPRRGHELRGGDNTAADSLEEDEFESPREDLDTRSEDPSVAGTQSESSLVNRRNNSHRTAFLCSPNSQPDAADLDAREAPGTPCSQENRKSLSSLTGEGWSQESEISRETNKAGVCVSASLAKSEEEVTRGNPHTADAFLFPAPSAAREVRRDARRGRQNDSSGGEDGSGGEDSAGRRLNQVGQSLETELQQQAQHEMKTKQSFSDLETPSLSENDTHGEEEWELRWVPSITPDWDVESHFFQLRDFKVHISLSNVGGAARLRVSRFKVDGYQRFDTVISFLKKALKRDHLYVYVNNFIQPQPDEFVADLFKAFGVGGSLMVSYCYTPAY

>845

MTEEKVTFLLNSASDLPQISKNKVTLSRQKSMLFLVKYVKQKLNIQGNQSIHLFFNNFAIYPDWTAGDVADHSPGQTTLDIYYSSGQVFG

>846

MTEGAAAQKITLRLTAKAGITETLPNMNCDPGETLGQVQARIKKKLKRPVLYLFVMRGSEGFIPTPDQTLESLLHAYAESDGQRELSIAVSTGIFHG

>847

MTVPSAMTPSQELDRQLGSAATISAAGSTSVAALQALEQYKKRDSSKVVVRFKAIGNAPIMKNNHFRITAFNRFQAVTVFLRKELNFKPSDSLFLYINASFSPAPDDTVGNLYRCFGTEGHLIVNYSTTAAWG

>848

MSIETVKEHFNSIRESKVVVDLRPIANAPVLKTGKFKVSGQETFADLKSRIETMVKRDTVYLYVNNSFEPSPDDYLFDLFECFKTGEQLNIGYSLTPAFH

>849

MRRERSQSLNPSSLTGRGGTSAPVSEEGADPLKPVAPSSGAAAKNTLEDIVTNTFRTFAQVIIQARSQRLGGHDPAPSSSPHHRPDVYSPEASTWLNYYLDCLWAVDEDLAHFWRDWKRNERRSAIDLTLDIFAQLPSSDPPSRSRGTSQQPPPPQQHQQQLRRDLATSQPFLLERWVLSFNAKPNGEARHNTINVKKTAMVLRSVGSVVRALPAHKLVRLRRKQPTSFSIDYALYPSACASLISVEYATNLPSFKFQALKIEPFVISDYAPSPSEQSAARGEPSTVGAPGVSPTRRKEESATSSPRGATDISDRSARDRKSLERATSLPVFLPSSSPGMQPPITSLGGHATPPVMAPALPPAGRANLPSSASIPIRGAGASGQPRGGRGGGDDDHLDIGALAMLGTSTSPPKMMSPPVFSGGGLVSPSTSAGGGGGGGLSTRGTAAVGSAGKGNANFFNFSTPPFTRDFFLQQQQQQQHFPRSSPGFPFVEDSGSSPWEGAGSSASRSLETSGSYSESIFPTLLPPSSSAEDVGEIPGLLYTKLPASQPAFSVATQEIEVSSFLESCAHPPDLKMFSQSKNMAQGTEDLETLSRSLRRLSIDLGDARKRAPGNKQSSRETTEDDPLFFDM

>850

MARLTLGRRGSEDFGASSFETQKQCRCTMQYRTVCIRLPKSSNKAKPVPRKRQRNNLVRFDIDEDQTVREKLEAAMERDPHALISIDILLHPRKNRANDEELVLLERWGIKYDVHAAGALKKATASWENVKKNILILIRSVYSYAKILPAAQLCSVYDGVSVDVVGFPFPMALSLRADIERGESEERAFEISYKISTTSELIPFGSPPRNFLFAPIETSDGKRLQIWVSHREKCSYFDFARAKRPKSINHPRIIKDYIINSDMINGTAAAATPRNPSQSPSHFASSSSSDTKRRQSPSSRHSHNTTSSPPPPSSSSKPPPPPTAPNTKAPPPPSAVRKHSRRSNGMILWPFYLGP

>851

MLSSTDSMQSTQPSRSSSPQYPGPDYRYSAQNMRNIAVPPRSSTGRAKTEQVVLEFLYKVAELIIQSRVNFHAEPDLRRGSRRARFNLDIEEVPIVRDAMAAWKEDVTLPLAIDIYWDAGSHTILLERWSVTFVADGEHAFAYLSSTQDVIQQLKEVCKRISVLLRALFSFMRQLPAHRLFTQSYPSMLSYTLHGAVASDAVHAFETQRVATSSYSFMPITTPFGNLKVTAVYRRDCDPFTELRELAAPLRILQDDFIIQDYVPSSPEFASASAPVSRSTTVETPTREHEHRTGSFVPVNGPPPTSNYDKDALSSRRSSPRSIPVTTQQPFSHLNHDMDPQREEEVQLPIGMSRPMAIPRSNNKASIAIAANRATLQHAHSYGGEDDTHLLNAAANLNVAAAPYGYGNVAIAREREHTLSPSLAFQHRQQQLWEGHAAHHDTESRLHSSPSCLDEPPIHLLDSSSHGSTAYQCLSTPPRHPKTVALLRTTSPAVTHKPFLETHAAAGSFERFALDSVPPSHVQPHRLHSRKGSFSGAFVSECSTSTGGNESLVLLNEGTTDMSSTAKTETITTYSGSHVHPSTECLPPFTSSPPFQANPCELLSKSPGYAYAKSQLCSGSSTVPMFITTDRFQRSSETAKPVKSATMSTGEHCEFLPDFGHTGVAAWGISPDSPDSLSLALGSTGNSRDEEAAVESRDNFMDDLDALILPFAIHDGQLSAATTAGDSVSSGAGSSFSRLSGASVGNFLQQLKNAPRLSKSLSTLGPSMSPDEADNCIDTRQEMKASFSMFDEELAGFRNLREELTQML

>852

MLSDFKQQQQQKHHSHNPPNSHDDTQTKLQVAKLTQVIQKFFTKAAQIILESRAYPETSTPSLYPTKEESSKINKWFNLYMTNIPDSCKDDLKLWKGVDLTTIPPMIIETYIDLRSLPADQTLVLMDDEKHPWTVAKSRGKKQEVVLERWLIEFEPNTTDATVMVEELPLSYKQAIVLFRSIYGFTRLMPAFKVKKNLQNKLPLGNKILDGNQPISSKGRIGLSKPIINTRTNESHMTQKYFQPVHTSLGTLKISVAYRMDSEFCLHENEELLSSHFHKRDEEETKKKVSSSVSPLSSGTSLKETSTSPRKSQPPIRIQPFKVGSMSTSPPVQSPSISQPGTAPIQNQPSVPSSSLERRVSITSNKSTSNASLAAFLRNARSSTPSANNIPIINANPISGTSVPRSFSSSTGHEDSIFVNPDSASNTPRFASSFGSRASRRYSSTSIRQQTPQSDLMGQTNSVDAALSGIDADDDISDFVRMIDSKSDLRLGGGGGGGNSSVHNMSINESSYHGDALNKFQSLRSQYQQLSDSVSASLILQSRHSSRKSSLNSPAGSFDSHHHQHQQQQQQQQNQQQSQSPHTNTTSSIHSHAHSYSHSRMKDARPRSEDHQQTKFSAARRSSNISPTTAVPSSIGTPSSISSRIPHVTTIISSSDVSSTGGNRTKSAATTAIVSGMATSPSIYDYRSPRYQNVFDDDDEDDNDEEEGDREGNQLHEGRNSTESSQNQSKRIMKHIKKDEEDSEDDEDLLFTMSDMNSRNF

>853

MNPQPSSSSSQQQQQQDSDGVLNVLQSFRSKAAHAVLQSRLAETLLRSQSKASPSAAAARGGSLGASSSTAVTTPAAEKQRKVWFNLEIDETDAVREAVRPWRNDVASQLCVEVYLDLSHVGTDKVVILNNNRTVFYNANARGQTNKRKIILEQWTFAVDQSRVSNSQAAQSHVVIYKKLIVLLRSIVCYLRTMPTNTLARKCARRQMSAMAIDFQITSSHVNIPENDRDGPMSNYQFRSIDTNRGRLLVDVCFRTQLEIKIAESDRLLSTRLVENYFDMPGYQPGSTPFDGHLQSQALPAASLPTQIGSTHAVVGAHAQSYTQPTQQSGFSTQPAQLQHHQQPHPHQQEQSKAPLHTHHPLPHQHSAAQLYHHQPTQLVRQPHSAADLTTHLSSALGDGYQNLLFDRISPTMFEDRYSAPTTDHDDGEAAPPLSSFQSRSTAPITAAAAAGAAGAAGAAGISEAMNISNRGSLPKNYSTAMFGTPPSYSAHYFISSPRSYEAASAMAAAAASGAIHQQQSPPGIGASTSRRASVVSSGSGTWLSSTPPFKHANPFKSMNHQSPPQPNHSQPHQHHSHHFESGDASRLGSSRAGSGALGEDTPSHLAASPARAIPMPRGRHQSVDPSLDDPHHSNGWLQSKRTSDVSDDDDLTVLMTQLQSAEALRVYSRLDASTQHSMSDMMAKFSDFKAAAANFDFPPNADQTTAPEQQPPGQG

>854

MASSGGPAAPPSLERVVGEAFVKAGQFNLEVDEVEGASKALERWRRETGLPLVLEVFLQPWGAAAGGQPDKAETLLERWVVHYYPMLPEGPSHLSRSQISRLNPSSVYKRLVITLRSLYTYVRVLPAYRMYRACKRQRGSNFTLGYRLHSTLPGAAAAAAPGARRMQSFRFGAVDTPYGQLRIGVDYQPATTVTILEQTTSPPPLPQIIADYVGGPAARGGGSGRAPLRHAMSSAAEGWGAGRGPSPPTPTHARQQRGALASPQAQQQQQQQQAGGSPGGGAERPGGMPIRRSWSASMRGASPHRAPTYPPADLPSPASPHDSPYGSTPQALRAAGGGASAAASPGGGGGGGGGAAREGGASTALIRRPSWSSRSSAFGQLAGEASQQGVGYSDVLPFALDEGSASSPLNPRSPAPPSPPPPPAAASLDQLPVSTPAGGGGSPLAAASRQPAPAAGQGAAPSCGAATAAPAPAPAPAAVPAVDGDAAVGAFVRLIQGAPPLRMQAPRLAGRASSAELSAAAGLRAAPPGTSSGSDSSAATCLSDGRGLTLQAGLRQFSRIRERLQQRGVLVGPWPAEA

>855

MQFNLECEEVECAQREIYGNVWAANEAAAAMGPSPQADSGAAGRVLLERWVLSYAPALPSDAKAGAGRPMTSRLEPAAIYKRMVIALRSLFSYVRVLPTYSLFKDCAQQQGRGCASLQYRISRGMPGTVAPRAAPLPPRPEHFGFTPVETSYGLFRMSVEFAHRDIVTMLKKTTSPGPVAQPFTTQQPALRRHSWTPHGLRTALSTMQRTPSQASPSLPATTNSASEAGRHAFSGPVAAQSPGHDRGGYMAKLATHIESDEATGATGPAQAHAWMTPPKHPGTPTSPHYAATTGAVRTWSRAAANQEALLAKTRQTEVQERMMQQASGGRPSPEHAPDQEAAAGGGPRQSSAPVCIPGASEGTHRSRIHSSGDLAAMQRRNASPRPLKTVRWTSGVIKTPASAPAASAAVSLDQPPHLQEVDLEPISARTTGFNVPLQPDRVPPGSSRSLLRKDSGQAASSGGSGRRPSFGSGGSAQPPPTPPILGGGLGAGPGSESSGSVASSFPASCSPQLPFAFTPSALSLSSLHSGQSGGLHAGAGMAASPAAVGPGALTVFRRRSVGASPRSSAFDLPPPGALGYSLSPVQGSLESSLMGASTPRYPCTLPLILPSSYPSPLLPAGRGVAASGGSFSSMVGPPRLTYPSSGSTGDPAELAALPSEEAEADVLPFALDADAVPASPQPPVPLRMSLGGTPSPPELDEEVGAFVRAIQEAPLSLTRGGVPSALAQAGPAPPPSRLTLQAGLDQLARFKARVEAS

>856

MDSRYSDLIRKTISKISQCILLSRVETSSVSRRVNKTFHLETEESDIHRQEIDRIVLKNDMRILFVIEIHFDENVGLGLNTIVESWRIIFDPREIDNSVELPKFYKNVIILIRTIYAMLRNIPCYSLFKNFSKNRSATCSVNYQLRVCDSQSITTPSFPLSTSIKTVSFTAIPTPMGILKIDLSYKENLTREISASSHIGLESQFIIKDYNNYQSGNNYDNNNNNSNNSNSNNDYTNIINNTNNININNNNNNNNDNNNNNTNDNNLSTSYNRDYPSSYPQLTRMGTLQDLLNNNNNNNNSKPTSSSPYDSNSMPFETNNNFNNYNNSNSNANYNFNNINSNNNNNNNNNNFNNFNTNNNSYNNNNNIINSNNNNYNFNENNNNNPQSNPIFIPGMQNNRQYQQQQQQQQQQYSSSFNKPPSLSSSPPFSSNSIKIGSGNANYNNQYGSSPPFGSTTTTTTTSNNINTSGGININNHPSSNPLPIRQVSFTIPNQTTGISPTSYNNQQSKTRATSAPIAIHQQAPPFQNFPKSSSSGTKSPPFHVSISPFKEPSTLDYYSGGGSTGTSGGLIGSTGASGGGLVGNNSNNNLTLLNNSNGIGGSGGGLVGNNSSGNLTLLNSNNNSNNNLIGISGNNALFNNPLYQSSSPGSTNSFGASGIDRLNNSSMKNSKISQIHGPLLLSEHHINQFTKDGSRISSPPINTLDDNDDAVFVSTLRNSKQTTHESEIADFLKLCKIAPPLKLFNNDNLSLNTTNSQHSLQIGNEIMLLSNINFNKPTSQQYQQPPQQQQQQYYQNF

>857

MSSDRSKCEQVVYELLCKVAELVVSSRVSLIQPQRREVNSKFSLHVQELAFVRNSMKAWENNLTLPLGIDIFWVEGGGDGAPRRELLERWEIMYEAQDAAEGKSAQLRHFFKRSIILLRTLYSLVRLLPAYSVHKRIQSRDVSLADSRDDTGFSLYPLRDGGSCGSGLDFSASGGEDHAGRSGRKSTSCYTFKPLKAPHGSVKVICHYRRGVDSKAYQHGQTNTVTAAPDMTTSLIPDYVTDEYERNAAASRMPATEVNHDWPKLPSTPEDGASVPDGAVDAYGQHAGNKVSKENTSMMHWPSPELGHGYAEKRTHIGPRQGPAPFLTPGPHRGRPLGRHDVMMEPLELGPNAEIHARGPPHFPAPPVRQENEDLIRVSAQTLSGASGRTESNHVDSHCSNGMIRTAEGGEDGVADRGRALMRPYRRNSISESLSTPSGSGGSRITAGHRHSTAIEYTPPFSYNTTGPDLGERNIALGQPLVSFSPPFNAVHVGQRGHDSTFSIRVHSSGPRAPVPTLLEVLPESPFALRKEGTQPPGVPGTAVASSSRPFLPRLDCSSQEPTFAATSAGIDRTELPFAAELQDDGVSASVDSSVCGVEGGLDSSAAPSTLYALCSAASSKSIFLADPSSEYAEQTAVGFLQERDSNDVPSPGAEYTSTAGSLPQEVIVRSLEEDLEGFRLFQQEVLEQRR

>858

MEAQKLQLVVRNCVQKTLELVLRARVPLGRGTPNHWFNLESEEVACVRDRLEGWKQRISQPLQLDIFIDTSSSAVRAAVGAQPNEPNLVLLERWTLRYEPAPHAPEAIGWPTFYKRFMVLLRSLAAFLRLMPAHRLASSLGRLGHASDAPALGFRVCIGGPHGGRSSEASPEAAPLDFAAGVSPQQHAFSPPDSTHGKLRIAVSYRPPSLFTRTARPAAAAVRCSSDRMMSAALIANYLPTEGARQPPAVATLDEAALGADGSNPRFGLLPRPASAPHAPSPPAGLPSS

>859

MADASSSRIATDLRGTLVLSILNARLPPGQDGPQPPLSAAQDFHLRLEVEDAVNAKVQGVLANAPETVFLSIVAPAAPTPNEKFITLELWVLHFDAQARAEPHKGATLKKAVLLVRAVHAFLRLMPAHRIVRRGRHTRLASHMSCRVADQRPASPVFPGPTTHHGFAALPTPVGRLTVAVQYADAVPEHLLATNVPRLHIIPDYATRPLRPSLPEISSSTSSTSTAPFFPEHPGHPTVSSTPPFSCRTPPSHSSSLASLTGGSPPLPFTFFAACRPTPASIPPMPAPGEDPMHFFPAVPSLSSFPSFGSLPRSPSSGPPGLPRATAAPAGLLPPAASNPATPLLRPAEPPPTPDFSLFHAFGAAEDDPKDPQDDLTDFADLCLRVPALTLTAPPASPSHAAATHFLRLRAEFDALVAQRRARAAAAPDPPPGTAWHSADPR

>860

MSNFAYPHMRAAEVNHNGPRSKCDQVIYEAIAKACEIVVSSRVTHANDAGSSRFNLHIPEISQVRSILQAWRLTLHVPIRLDVYYQHEGDRRELLERWCLEYKPTSTEHFIQTEGVVTQDPIVQLRHVCKRIVLWLRTLYCWTRLLPAQALNKKGVKSPIGFSIYVNSERKDDITDLTQNQGFRWQRQPSSVVSPYGELEWKVIYSSSVSRLVALQKPTKSVAISIPVSNTPSQSESDGRQMIRHSAPSHFVTMRAKSFNSSNDNHLTRTKNFNDQQNTYDPSSFVRPVHQSTTATGFSPHGVLKRRHTSIGQESAPTVETGNLHAPERILSGLSLAMMSMNQDDDDDDDESFNISSDEPDLPSDAVSRRHAALHQVPLHVLEQQQSHHHPVPASREYGYGYNNHIAWQNIQPSISKPVVGRSFSDLSDNNNTYSKTPTYSSSFLGSTPPSAAFLGATPPTTSSFPRPGSTMMTPPFKPRPMGFVHEPLNVSSLIPPQAGGMHIAENKSGSLQPQTSLDLLHSSPFQQQAQLTSLVSSFSGFADGTGLNSSLVDRTLWNSGAPSRSQREDDDFYEEDIPFAVEEDTVSATKHQSPGSSLMYASSAVASFAQKCTASQRLSIFESDTAADAVSPLTDNVADKLSEFRSFDASLQASSRYAGGNSSTVFSLRT

>861

MRAAEVNHNGPRSKCDQVIYEAIAKACEIVVSSRVTHASDAGSSRFNLHIPEISQVRSILQAWRLTLHVPIRLDVYYQHEGDRRELLERWCLEYKPTSTEHFIQTEGVVTQDPIVQLRHVCKRIVLWLRTLYCWTRLLPAQALNKKGVKSPIGFSIYVNSERKDDITDLTQNQGFRWQRQPSSVVSPYGELEWKVIYSSSVSRLVALQKPTKSVAISIPVSNTPSQSESDGRQMIRHSAPSHFATMRAKSFNSSNDHHLTRPRLINDQQNTYDPSSFVRPVHHSTTAAGFSPHGVLKRRHTSVGQESAPTVETGNPQAPERILSGLSLAMMPLNQDDDDDDDESFNIPSDEPELPSDAVIRRHAALHQVPLHVLEQQQSHHHPVPASREYGYGYNNHIAWQTIQPSISKPVVGRSFSDLSDNNNTYSKTPTYSSSFLGSTRPSAAFLGATPPTISSFPRPGSTMMTPPFKPRPMGFVHEPLNVSSLIPPQTGAAHSVENKSGPLQPQTSLDLLHSSPFQQQAQLTSLVSSFAAFADGTGLNSSLVDRTLWNSGAPSRSQREDDEFYEEDIPFAVEEDTVSATMNQSPGSSLMYASSAVASFAQKCTASQRLSIFESDAAADVATPLTDNVADKLSEFRSFDASLQASSRYAGGNSSAAVSLRT

>862

MDIQNANVLRDKTDELMRRSISKIVQCILSSRVELLQQFKKINKHFHIETEDSEQVQKQIDKNIFARNGIRTLFVLEIHFDEGGELLGINTLVESWRFVFEPFKDVDTSFELPKLYRNVILLVRTLYSFLRNIPCYSVYKNFTRNRSSTSKLKYQFRICDFNSILSPSFSLSIPTKSFTFTSIPTPLGVLKLDVNYREDLSKEISISSQLGLDSQFIIKDYNNNHFKSTEPQPIQSQQQQLQQQQQQKSYYYGGSYGSNSNSNNPNIPAPSQSLPIKQQQTAQQQQQQPIYYPQSYGSGGGGSGGRNMSSNQLVSSDPYKPSSVGSSDSYDYYGGTNTVGSGGGLGSYNNSFAHQYLQQQQQQQDKQTNPIGIPGTNSNNNNTNIYGSSSSNKRNNNSPGGVNLVSSPQTSTSPPFFVNANNSASPFPKPSGAGGSSPPFSSGGGTSLLHQQMQQQQQQQQSQPSYPLTVRQVTPSSGTTNISPGQHRHRSISAPITIHQQQYQQLQQQQQQQQQLQTQSNSYRSGRSPPMNIHTGFSPPNYSGTSPPLYNYTNPLLNNNNSNNNNNTNYGSYGSQQNQVSSGGAGININLNSYSNSSSNNNSGVNNNSNNNNQTTTSSSSSFKNSINSKLSQIHGPLLLTDVHKELQVKNTIEPLTTQSAIDTEEPVFASAVSSSSKVNDSDVGEFVRLCKIAPPLKLFDTNYTTESPIKLDQDVLELSKIANNNTK

>863

MATADRATQITREWFAKCANVILCSRLADDDPHAAQTPTRARDRWFNLDMDEHATHASSIKPWRRGGAASVHASTPLVIDVFVDARMRDVDVADDATAAEAVSRTLVERWVLSHDARCRGDGEEEEEVVEEEEEECTSSSSDVAVTYKRAVIMVRSLLALTRALPANRLHRARVRGGARDDASYRLSCEVRDVGTLGTNPGTDEGRHPGADAWRTYEFAPVDAAAAR

>864

MSRHSSISSYYQPNSRPESPAPFSSSTSSSTSSNTLQQQQIYHQQQTYLQQPPQHTQLSQQHLQPQQHQQQQQQQQQPAVPPTQPRNQKADQILQNFYVKVIQIVILARVTHPEPHAGVTLRKGSLRGVPLIKKTSKWFNLELEDLDIYKEDAKFWRATAITESPPTMLVELMLDTSELSHNQMLVLVDENNRKSRVDVGSSMASTPGQPRTRRNIILESWSLTLSNTPPDPVPEPPVVYKKSIIFFRSLFAYMRLLPAYQLYRRLRKQNHSLKIGFRVSRGQTPEDSMLQEPEIGMEVPLIEGETRPMISEYRFGQVETPVGAFSLKVTYRSNCDFHVDESEAVLSSRFIDMDENYFTPTIVTHSQESVKSQRRQSIDNPAQRPLAAVPLSRRPSNDSYISGNFQEHFTPVTSLRPRRNSAQSLQQKSGEYSSSQSSVSSLGTRMSRRGSSGAPGGLFAPLDQQFPSGTPPFSITHGGHAAVAKHYVDPIMESPPFRLGSSQGDNKRPLSSTFSPFKSPSLSSSPSPSHLEALPPSIPSRPSSIHLHRSPSSSSMSRIQGSQQGSSLLGTAARSGATGTSVPTTHQSLGASSGANLLSTSVKSNASSSTSAPRVFSSFGHRHDSSGRRSSSESLPPGRNRNSIIGTSSLFQFTPDDEDVNAFVKMMDSQEPLKMFGKNAGSGIGPGFGQGDSANLSASTLKSKTALDRFQQLKQINTSLSDSMTASQVISKDSKDRPPSLSPLTITTGSPKKSSFGDLEATSPTSLPFATGVAPAIPRHTQSLSHQPGTPSPLHSEIPVYPSIDSGARDQTVYAHSSSMASGETQAKKPSSWLIGSGIHDISRRSGSDSSNMANIAFRHGSLDQGKDILGMEDAMGKLGVSGSDEIAYGSYPEDTHHQHSLYSAPLKTARGDDDAELSVSPSSGVDPRPIPPFTAFAGSTSSRPGRLLRLGSGSSGTSFTLPRLRARAAEDNDSLNELNSVDGDHGDRRRERQRHHLQADDDDYDDDPRDLYEQDKNNQSLNDDDEMLFIMSELTPGSNSGQDLVQANATVDRGTMLPLTGALVGMRSQTQSPSMGLHGPLPSGAPGIGRSDSPHMRLFGRGNGGNNNLGGGSGNGYNNGSNSTGHSRGGSINGQGTHHGYHHSSGMFSSNVTGNVTGNGNSNSSNESLNHGVLPMMRRSGSMEDYLLPLRSNSTPPVLPPVGGVAHSAAGGVGASSGASSSSSVSSGGVINMNTPLPSSGAFEAMLEGRRMSRGGSDRGVNMASANGSGSGSGANSGGHNSYHSEGRGKIEGW

>865

MSGTNESIPQEWAFTMFEAMQKAAEIILRARTKDLKAGPERAYTATLPPHGHQSGSSNHGNVSSSSSRRFRLNLEVEEVSTVRRGLAAWKESLHTPLVLDLIFQGPPSPPLAGEREQVAETTGEEGSQDLLERWYIVFEPLTSSVSSPFRGSGGRQVEGGGFALQAQVKRAQKRLSVLLRTLYGFLRLMPSQEVVKQLAETGPGEERAFVFPAIHAGLANTLRQDMDTLLAGPEEGREGGAEGGVFESHRFPEVTTPFGVFRLNVAYRRSVSVFERVAAAAAAAAATCQAENGSVQSTAIYPNYVAPGEEEGREGWREGGATRAPPSTESGLAHALRDYAAEKRAAAGKKEAGRGEDKAGGQHRTPPPPASTQPASFSASGTRQREGHDRSYPPPRPSPHPSPSSNATSAPSSSSFPTSLPHASPSVFPPPPLHAAFAHEDYARHRSFSSFSSSSPFSSSPSPSQLPPSPFPPRDPSPTPPPPPAASRETYNAPPFGYPEPPPPPRPQGKARTASQEASVGKGGREGGK

>866

MNIEKSDLDKSDFIIRNFFSKFLQIIIQSRFKFSDSEPKKKDIWFNIRIPEIQQLEELSIIRNYKTIPIPNFEPLFIDIYLDVSPIISRKKVYVKNEGGLDIQEDDSTVPQNMVEIKLYSSSQCNAKMEDNINIMNANDPPITPSTRCRSREKKKIILLERWRLSLTQNSNNLNERVESFVTYKKAVVIFRTLFMQTRMMPTYKLLKRLQQQYKDQDPNQPRPHIFYKISNNKGQSSETFEEIGFDVPFNAGDMKEKLFENVETIRGTFSIYVAYRKYCHFIFKDIPIPKSEPIENHDTNEFLNIMRPRNFKLRNPFTPETSQPQISHTPQTSQPQIPLTPQTSQPQIPHTSQNPQLPRPSHSSQTPQVLQEPQSYQLHQNLPSSASPLASSKQGILRHPSLQNLKNLPFSTKSTPSTPKRLRSRTLSFTTLKNELRSEYSDLSSNSNSNTIINNKNTYDNNHNLNNNNNITQLEPDPLTLLRLPYPNNTIPAMLDSNNNVNPYSNINTRNSPFHLNTDTGVSEINSHKEDTLFSANTIKTKDNSNERIENANNTNINNNINTNGNGNGNANHLKEDLIFTKSNNKTIINSEEGKSSLLTHELESYKTNIFSKRVFLENKNDIVNNNNNKKNEKEDSHGSITAKTTTTIVMDQNSNNSQANHINNNINNNNNNNNNNNNSISTTINSNHQNNININDQEQIFDDNTNLPSPPPYSSHSYNSSINNLNTKIMLQGQPTSSSSSRPLNPLLPITQPPSQISLGTDIDTDACSYRNSKFNNNESFNQSNPNLSGNDITMTPFFHNNNNNNNTTNDVTTNFSNSSTKEFSYPESEFLNNKYLFVNKKHLSTPSIFSNSGSSINYTPPFTEIDNYKKQSKGSLSKSSSVSSLSFKYIYMQDKSYGNLVKNNNPNVQANNPLNIKDALQYFKQLNISNKKFTNNLYDSIVFSHSRSSLDEENINNYTVINPQSSSASSAHLPFQSDSVGALNNNLNNNLNGLNLNTFKFNLFSDNRNNNNNDNNNNNNNNNNNNNNNNNNNNNNNYEDNLFGDSSSIDLSNNIYSKKINNVSCPSPHSTTSTNSSFEKMFPFSDTELEPPSYTVPTFATITNKIMIIMIIEIVILIIIIIIMIIIIIEMFMIMIKI

>867

MVSRGGGGGEGRVIAIDVWFERDGSEVEASALVERWTFHAGADAGSVGGNNHFNKDLETPVVYKRAVIMVRTLVALLRTLPAHGARIRAMRHAGATLRKESGGGRFRFEIRELASGTTVQDAHPGKQAGYKTYAFADVPTSVGKLCACVYFLDEPAVKALEEKFIGVIDSTPLRSLKSETLDASVGPDVELAGGEMSLSPSPTGKGGSDRMRRDTDLAEVSRGLTFGIDGTDDSTMQRENQPPVRGGVTASSSAGSLQQMMKPPTVPLADYMSPNFQRFDNAPSSAPSVGPAPIYGFNAAPSKSSASSKSPGTLDAPISGMPKPPIRAESCLKIDAGTSMDLADVAKFEQRPKTPIRATSVAMPTLTVGAETTPEQKMRSPMAGSTPIACSPANRHRGASAARAVPSPSWGGFACPESPSPSLGTSPGAHFAQRGSSYRRSSWSPSSSLGTSLRDVVGVYPHSPGGATAVAAAFARRMSGASDDATGGSPHFTDDGANEEDFPFDLDDADGSATYVEHTPADLLHLLENPLPLRRKSTDSPLPLDAALDEDETENDNAECVSNGVNDDTRDACVSAEPRAAKFASADDASTLTLGSALDALRELNIARDALDEPSV

>868

MTSYAYSNWRNGGVDHGPRAKCDQVVYEAIAKGLEIVVHSRSSSNLSSNAATASASNASSRFNLHVNEIPAVRTIVSRWRHSLHVPIRVDVYFQHETDASRRELLERWCVEYIPTTMERFRTREPGVPSDPIVQLRHVCKRIVIWLRTLHCWSRLLPSQAFRVGGSNQIGFSVYVVSDDQDDIGSLVEQQGFLVRQNTATIATPYGELGWQVAYAPEEVVSRLLPAQVTRNLSISPRIPRVVSASRSIPIHRGGAESRICDDAHHHYSTTVDTGIPQSAPAHLAMEDNYWENQQRPASVRQNTYDPSRLHQYHRAHTEMDDTPRRTQNHGLLERRHTTVGQEYDVATKTDYGNIYSVDDENDKKPARVLSGLSLALLVDDAVRSEENPEKEKRRAALHQMPPHVLEQQSHSQPPKPAAHRAQGEYGYGYNNHIAWQAIHPSSTHPTAGRSLNRSPSSDLEARSLSTSPARLGSTPPGAAFIGSVGTTATKNMQHLIAPRNSSARNDIAVGTAVTPPFASRPAGFAHEPAPMAALTEQANSVVEAIPKNHNDVLPPLSLDLLHSSPFQQPQGSLISSLGGLPDSALHGSTDLRRSLWNPSSHHPMLPSSSSGYDITDPDEMPFAVDLPFHNPNGASTKNPGGFESSGNGNSLSASAAIASFAHQRRLQLFDSCTGRSANVDDLTSQLAELRDFGASISVGVGGPTSSAHGSEHDAASTTTPISLQT

>869

MMSSVDLSSQSRPSYTAPQQFSASGGRYNAHNPRYPPRSSTGRAKTEQVVLEFLYKAAELIVQSRVNLQAEPDVRRSSRRARFNLDIEEVPVVREAMAAWKEDVQLPLAIDIFWDADGHKVLLERWSVTFAADGDATNLNATQDVIQQLKEVCKRISVLLRALFSFMRQLPAHRLFAQAYPSMLSYSMHAAAATDAVRAFEAQHVATSAYSFIPITTPFGLLKVAAVYRRDCDQFTERLEQAVPSRLFQDNFIIQDYVPGSPDLTPASAPVPSRTTAAMADAPMRVTDLRTDSFVPVSNAEQFGGDVGVLQRRRSSPRSIPTPQQFSNAGSMGDNRADDEVVVRPQAGVSKPMAIPRVSSKAGIVGGGAATGYSEDQRDDKLIQHAHSYGGEHDLRLRGAAANPNVTAAPYGYGNVAIEQEQQNSSSPSLAFQQRQQQLWEGTSRQDTENGLHVSQSSLHDDPPTSNSNAAYHRLSTPPRHPKTVSLLRSSRTSFGLPHKPFLAGNESHPTPGSLDSFTLDSGLQPQAQPQAPRRRKSSFSGAFVPDHTESTGMTLQSQREHDSTPGDGAQVTRPIPATSEAIAIRPNGTLHPTTDDMAVFSASPPFQANPCELLSTSPGYAYSKSQLKSSSSNVPTFITTDQFQLGLHGFKRSTSAGLISARKRGFSPDFGDSGVTAWGISPDTPDAFGLAVVDGAGGSAGGRPRFLSLSGSADASSDSVGDDLDADGADMMLPFAVGDALTIGSSTTTAVDSISTGAGSAGASLNTASVGNFLQQLKNAPRLTKSGLLEEVDQTKQEAVAPSAMFDDELASFRSLRDELAQIL*

>870

MDGMLNSPPGGTGGYPFSHQQHQQHQLYPPSTTASRHSSSPSKGPRAQCDEVVFEAIAKAAEIVVASRCWIGEGSSNINSNSNSNNTSNGGSPSPSSTGSGSSILHRWKKTMHVPLRLDVYYQHPPTATTTNTNANPHDPSSGGGERELLERWCLEYAPSTTNNGMPINMNSPHSVDPIVQLRTVCKNIIVWLRTLYCHSRMLPAQALRPRFHSHSHCGISSDNNIGFSIYVVSEGRDDVSGLLQQGFDSAPTPTIPGGVPTPYGVVGWKVYMAPRDTVRKLVALEEHFGDGRSRALQLQQQQQQQQQKQLPYRTSPSATMNNDSTRYSSSATTTTTTTRSIPMRVGQPSSQHQQQQQFAPQSYAAYSSTPPTTTSSPSRPQLAVARSAPGRGLRGYRRSHSSPVGSDALHSGHDMHMNTNTNTNTNIYHHNGNDTELQMQMQMRSRTLAAPRSYHHNTTLLQRRNTEPERPFPNRSSLHAQMAPPAPSSGGAEKSPAQGGGSDGGGIGGGKNLSGLSLALMQDPQQPQQQPQQQQPTPEEIAASEKRRAALHYAPPSATGVGEYGYAYNHQAYDEATATATPPPYSSSFPRGPSTPSAGITPGTTPPGYLLSGTPSLGSYMGAGGLLPPARASASSTAHPGTSPTGGGGASLAPPFVRPLGFSGHTTTESAAKSVTSAATAAQRPATNGTSTSVATARVAGGATGQAPSGENDHKPSLDLLHSSPFHGIVGGSSYPRDGLSSYLQADENASMAAATTYIQDFYHHHYHHHHPLGGDETDGDHHGHQYLCHDPGSSVAAAGALSSPGAGGEVFDGSSDHYLYHNPDYAGGLVLDADMPFAVDGISSLSVAPNQHAGASASSMPSLQASLVASVGMAAPKRLAMFESKNKHNNNNNNNNHSAVHDPGVQQTQSQNPSHPLSNNNNNNPSDDTVVDSLADQLADFKSFGASLMATSGVGAAPAPLTASVGSGG

>871

MVAEEDIEKQVLQLIDSFFLKTTLLICSTESSRYQSSTENIFLFDDTWFEDHSELVSELPEIISKWSHYDGRKELPPLVVETYLDLRQLNSSHLVRLKDHEGHLWNVCKGTKKQEIVMERWLIELDNSSPTFKSYSEDETDVNELSKQLVLLFRYLLTLIQLLPTTELYQLLIKSYNGPQNEGSSNPITSTGPLVSIRTCVLDGSKPILSKGRIGLSKPIINTYSNALNESNLPAHLDQKKITPVWTKFGLLRVSVSYRRDWKFEINNTNDELFSARHASVSHNSQGPQNQPEQEGQSDQDIGKRQPQFQQQQQPQQQQQQQQQQQRQHQVQTQQQRQIPDRRSLSLSPCTRANSFEPQSWQKKVYPISRPVQPFKVGSIGSQSASRNPSNSSFFNQPPVHRPSMSSNYGPQMNIEGTSVGSTSKYSSSFGNIRRHSSVKTTENAEKVSKAVKSPLQPQESQEDLMDFVKLLEEKPDLTIKKTSGNNPPNINISDSLIRYQNLKPSNDLLSEDLSVSLSMDPNHTYHRGRSDSHSPLPSISPSMHYGSLNSRMSQGANASHLIARGGGNSSTSALNSRRNSLDKSSNKQGMSGLPPIFGGESTSYHHDNKIQKYNQLGVEEDDDDENDRLLNQMGNSATKFKSSISPRSIDSISSSFIKSRIPIRQPYHYSQPTTAPFQAQAKFHKPANKLIDNGNRSNSNNNNHNGNDAVGVMHNDEDDQDDDLVFFMSDMNLSKEG

>872

MRQCYRCRCVVLAVSVCEHVCVCVCVLCLDEFFYVLLSTRARTNMCVCVAQFIHFSLAHSLDGSSHLTRSSRAIMTTLHDPWSAPKEDLMAHKSQLAFFDTVCQVILGSRHTSKNTSLRTTDWRGLKYIHNPTVTDAVAKAWKDRRPTTNQVDLYVDIIHDFEGERVVLERWKIQLRPETKHVTTLSTVVFFRALHLRLRLLPLYRLSQDPAAPLLKYEVHFNAAPPLNSDTPFMASPDVFLLSLQSVTFVGFSATFHPRPHEHFVVRRSSKLRSSSAASDDAALRARARVQQRHSRSPLQAAGTNQQQQQQQQGTKGDEKDEGKYGGRERQVSEDGMRPRVQTMPVSTSRPQYPEQQPHVHQQYPQQQPPPPPQSLSHPASGYHALPSQQHLLYQQHPLYQQQQQHMQQQQQQQQQQHTPISDRLPLLPPHHQHQQHQQPPPSSLGQMDHVEYYQQQQQQQGGSGAPSRDTSNNNLVALAQQQQQQQQQQRVQAQAQTRTSSPAAAAAVEGDSQAKTAAKAKTKGKTKGDTSGSGGGGGGGDGGDGDESSGPSRAESFASDTAVLNARTNVRQNQLRLNPSPTIDTDDDSGEWTPTRRRTNVHTHMRGLVTTAKMMVVTVVRNRTAAATHGPTLHTCTHTGPVHMCTATAAAAAAVGWFRTITVVTSQFSSISHH

>873

MWFNIGMMNAGSVLSLTEPWTGPKPDLDRPMSIEVAVDASTLPTTQVLLLNGKGAEPNSRYVVLEHWSLSIDKAAG

>874

MPMQVYEESVSASDSPSWGSVIAQGALPDQECGVPYQRVMEFISKIGQVVLRARVAVRDESKVSLQQRQLQEQQRRIDLKLAATGMHRPPPVPSATRPPGHHFDSASVTHNALLTLLRHTEFWRTSMPVNVDIYLGESRILLERWVISYESSEQITSTEDSSSFVDKRREDLTDLILLVQSLYSYIRLMPLHAILTEGKVDKHDLRYCISTADGYPLSPVSDDEDDTDDHLRAESRSTASERLDYFTPARNSHGLSLPIVAFDAAAKLKVYKFRSASTGSHGKLHLSAVYDSSVAGMIAVGGRRAASPVKSPVKAGSRPESGHAMKSTSEGDLTAGKPKDDYSVGKPPRPSSEPPRGRRSSSSLGSPFPSPTMQRRVLGLAQWKLSSTERPRPVSGLGLSDSARQVTEGGLLFSGTNWAAGTPGATPELPVELSTQGPEKADHSDRSRPVGIPFAEPIRDGQSPPKNRKPDLRLQIPTITTTAPIDHLDDNNISRTATTTTLRSINIPLSSSRPSYPHLFASQSSSTTPSELCGSFVGSYEESILSGRMSTLPSKPIRFQAEIGVVGFGKCASRSLRCPPHVSVEFDAFFYEVGEDEVLTPYVGFVEIGEAKRKREKADDGGAGDANGQDEDKKRESKTKISGYRIPPKGQLQILIKNPARTAIKLFLLPYDFQDMPNNSKTFLRQKSYTVPSATSASSKSPNVQSDHHPHPHLRYAIHVPVFRTEKGRVYVGPSLRVVFSHRALDGDEKVLVVSEDLGVGDGKYVKEAAASKLDSCREKQTTEEGDKVWQSGFR

>875

MRHGSPPVRGSSPMVAASTASLSSSAGAQANFSSSSRMGSTGRAEQIIQNVYSKVAQVIVQARATEHARRSLSSGRKLNKWFNLETLDIESLKEELKYWRAHAITSPQPPPLIIDIFLDLSALTASQVLMLRDELTQRRQRIGHEHLQAVDPSTGISGRHKSVLLESWQLTLSHPIASQAPELPVVYKKSVALFRSLYSYVRLLPAYRLYRKLRRQKDGNLGIGYRLSTSRVMPVDEAGLDQLHSSGDMRRGLSEYNFGSIDTPFGVFSLHVNYRLECDFSVEDPEAVLGTRLGDMDEHYFSPRSLDRARDIGSGPLLLQQARRSSSSSSRRGVIHQNDDMTRTSSAGSLPRRTSRYGSQTSVHSQDGFHTGRDYGRGGVPPVNIPPGSLPTRVGHRHSYSTASTSSSPGVFQFQRTGTPSPSSHHSSSQQHLMDRRQSWQVSPASNPSVAAFTPPSAAFSMGGRQDFENARFNEPPPFSANTDVAEPQVVPEILTADVIPFTSHSPPFDMQLSNKTHAKSVLAHRASFSVSRSASPVSTDLARRPSVTFPTASVPFKADISHSPPSFFTPNSSSLGRPLPAGYKLSPPPLANLSSGSGSSAMELGEFFRTLELGRRSLRISDTPSGEGTAYGVDNCGPGGHEGSIMNAADSSLARLSRTKLVLARYRELNELNAAFSNSLSEFIASEELDGPSEMGESHPQAVVETISPSNADPTDLVLSPRPLDAIEDSRRLEQHDDAEYPVLDTESFAFDEAIDRPRVGRANYPNSLPNHLQPVSHQHHLDPTSSTPIAMPRDHLSGGSPFMRREHNDSHGAAHLRRRSVATLLPDHDLDKHPGRPPDRMSPDPPIASPTPRTRDWIHSTESSHHVSRPSMHLSPGWSGGQRDPAFPYGMIQQELTSTRLSNPSHESSEEDEELLFNMSGLDLCDSRECM

>876

MSHNIGVGVRANVESAKRERRSIDNQTTMSYQRNNHYQHHHGSLIRANSLPVDQNQQHRRGVQQQQQQQQQQMQQRYEVILPEQPQSQRPQLYPANSMQHQSQSHRQIMPRSQITTEPYQQVQMQTPSQQQSLYQQQQQQQQQQYNQSSYQQHPLQPQQQQQPPQQLIGPRGKCDQVIYEAMTKACEIAVRGRLLLLHDVNVGNDTLVVGGGNRRTSYGGPSSTIITSNNNSNNTGGGTSSRFNIEVDEVPAIRTTLQTWKRALNVPLRLDVYYEHYDGDRGADGNGSGEGENNIERPPPQRELLERWCIDYVPSSSSGNNMNSCLQNDKSSSISQLRQVCKRIVVLLRSLHCLTRILPAYRLKSLIMTHIASGGGVGGIIGGMNMGGREGWGSIGYSIYVCDYESEPTLPSPSFTRHCIPNVATPYGTLTILVMHDASLNPNHMVADLAEKRAEWLQLRVGLTTPTIAQPIPIQGQNTVGSAGPHASADESRGRRKYGSCPPAALMGGPSSLGAMGRSPAVSDFIISDYHSPALKPLTAPSPGSVGSRNIRVDDEKRVMSGLSLAMMGEEENSPGVEHQAVEQNTNHHYYNPTSPDEEDEHVEASILPWGSSPATRAAFHHPPPVYADIQHAQSSEMAENSGTHFFHHHGGYGYGYNGSQMQFNSEPPPPPPIASVGSPGSGADGLSISPSPLMSTPPQAMWGKPKQLSRCGRAPPSAIKEHVGNGGDEASEDDIAPPFTNPTSLQPLPSPLDTRNSVTHSALVVANGNVVPNSLGSSPHLQERVDGRSSEVQQQKRTSSSSALLPPMTSLDLLQKSPFSASRIANKSTGNDAKVADRGDGDGLMMPFAPGSYRDEMFTSSIPRMISADAKLRTSSIGGGSSSNPLITSFGGGYSSAGGYYSTGPGASGRASGSHTLQIDAEEMPFAVDDDFPSLSGTTSPFSGKGSRSLWGSTKADLLDGASLGGVAEITSSLAVSSLHHRCANEGRRLKLFESTQTLASIRDSGNAEGDDNGNADDDFATFNDQLSDFRSFGASLMVGSLHDSRSE

>877

MINSQQEGITHHTDGGKTKLTSLSRMFLIKAAECIYENRSPESLTDLEKDNPLFSLQIRKSNQLRSILSSKHFFGWFKLDIILTNPDIVIERWHLIQLPIHPDAKMQTNYNQNELKIQTYRIMCQNMRSLYSMLNTLPAKTLQLQLSQLPMNNRKIKAVCDPFQKLPAQNEEFTELETARLRFGPITTPVGRTVIFLNHRISLEEEIPRPIISTQHWYVPMELPSTQNSLLQLNEPTPSCSLMNENSLANGAPAVNFSNTPAFNSMNHDDFIAKNSPIAETPPSPTPTPPPDIIPMSIDDFVRLVQENMNDGFEDSVSLDDIKTRFTEVKNILLL

>878

MSSEKTQILGKTELQKIISLTREFLNKVIECIYYSRCPGEIADFREDDFQFSLKIPRSQSLRQLTSSHKIHGFVKLDIIIVNPDKLLERWIIEHRPLSKQESEVFTQHYSTEEKHLIYQKFSSCLRSVFCILNALPAQSIALNLKNYTAQDRKISALCGDFKMLEDQRFSLDEGHTKQVALPPVLTPVGKTIIRCQYLKEPEKELVPIINITKGTSSSSSTSDSVQPLQVEEPSSTSPASYEWYGYDPNVPVDYGSYVTSPMETEFISPDEN

>879

MDDKQEWIHIKLPKVQSIIRKVSSKHSHGWIRIDVILDNPNEVIERWYIINTPSPAKSSPSATDESKILIYHKIQTLLRSLYSILNLLPAKTLDLTLSRFTKRPRNISLSLTPLSDFPVDEPPIECPLTYNFPEIQTPYGSLLIRCVYMSKVDLLIPNLKKPLSASGSYSSQIISPRIVPEKSKDFLSDSCAHLDSIEKSISKSREDLAQSFNQTPVDISEFAKIVESTNFDDKTDDVQEIQERYKFIKVEVDSILSEWSVSG

>880

MDFNAQSVQLKKEEQKFARLALTFLYKTMEAIYSIRCPKQVDFVEEDKNSSWFNLNIPRSNSLRQKLQSSFYHGWFSIDICLTDNQDFTEIIERWYLIHIPVKQTETLPSLSTNLKDLKFHTYRRFSQILRSIYSMINALPATTLSIVLQQLTTTKRRIVANVSGFQKFPAQITSFCEEETAKIRFGPIVTPIGQSVVICHYRLDLQPLIPTPIRTAPHYKFGSEITNDNQNYEESGNQNYEMQNQNYDMECDVKYDPINDINVVFGTPIMNNLQTSGQNVNDTYGTSVPSSNLPLMTPNSHVDFQSFVPDSMVGNFEPYDGKILKDKNSNNETEEIMPVSEFIEYLESCKQQKCADILGNFENHDQLLARVRDELDVLKSE

>881

MSSSSQISIHLSLSESEQRKYLDITKAFLCKAMECIYVNRCPNELSDLTSDDQKLFTINVPKSESLRRFLISSHNFGWFRILVKLTNPEVILERWTFIHATIRESDKIPKSKDDVYRHFSRVLRSIYSMLNALPTKSLEIILSNFPACNRRITAECFHFSKDPISFPVFSENPSIDKQRFGPVITPVGKIMIACETVTDITIYIPRLISKPPRRQSEADIMKLPYSPSKNSFSMEFSYTSPVNSPSFCGELVSSGTLTQFFEKIEMMPRKIPHEFEQNIENVIERFDLAKHEYLQFIQQNC

>882

MQPSAQHVPQQHPLSRAHSSYPTSSSSIGTDHPAHHQSDVASASISAQKPSAQRKHSVSSTSSTAPTQLTKLDGVIYHFYTTTANLVIQSRLAHLRSSLLNAPIDITSPPAAAVSSHAKLSRWFGLHIPESDLFKDELRLWRSLTCLLASDAHDSDHPTSTSAVPDLIIDVLLDLSAVSANHSILLLSRRSNNLSAQSSADSSIRIPIHRHSSGRASPLSSTTPPLRIVLERWRLQFKPSHPSPPPDLATFYKRSVVHFRALFTLLCSLPSNRLAAKIEALKVANSKSRGADATFTTSSVPSLSYRNHVGSHDAQMNIGCRLSMDADSYQARPDSAEIAITQPLPAEQHLPHLNDLNPDESGTPLGTSHGHYATRNLVPVATPIGHLELSVSYRKCTDYLVEDASSSASAQDIKVDLDEDYFRAPPSSATTTPAAPSNHRQDDPPRFSSSAQQCIGGISAARPAVLAAQKSSPDGATHSDALLSQSPANQSVFSTSAPSRHAAGLSSLRRTGSTKSNLSILSPSSSSAVPSSPALAAALSAEPAFMVPSSIRRPSTGERRLRTLSGLSSGRPSPPISPVVPSFNLPEAGTSLPRSASAAYSSSRVGRSISTRTTSGFAMTAPGAMAPPSSSQPRPSLSFSPSSPSPLAQHMSLQTSRTIGGGTGLATTVSQSTTSFRSMSGSARRSSTSNSGVLLVPSLRSVFQTYVPRSQNVSNASTARTLPTSSSAFAHGSFSPSNLGTGMKGSSIRRNSSTSEAGSGSAFGSSVTSSTAAKPQMIKRYSTNFSYRQNRERTGAYGSSLGSEGSGSLGVVGAGAEPSSYPRFGGAGGSLSSAYGRSWISRMEQRQGLGTGGAFARTSSLDDATAAANALSASRHRPSSAAAPTHLGGGLTPSPRSHDDDMDDLMRLLETRPAFGASSMGKLTSLRNERRNAPGLSSTPEEGAVPRAAAYGPDVLTDSVQSNLGQDRPSPLSGSGLRSGSGLRTANPMSRSQLDDLLNRMAESVGILGSKEPLSATSAETRPDGTVYEDTKTQVSDCAAVAQLPLPLPLPLASRPLTPAQISATLGKGRGSTGERIEGVVEPGRGRCSSAAGFARGEASVRPRMTVSHQARGYGTAAGAHSVISSDPIDSAQITSGDEARAGAPSTGADAAAVVQTSMTGSLEPSRLPDYEAEGDLIFKVDGQQDYDPQDELPGEMEMMPEEQNDDAAAAAAAATATATTARSASSGISALGGGVEGGLGATLPVGAHREGRAHDWHATDRWERVRAQEAERRRADAEMSRNFGQEVGASTRGRGSHSPWRGVTQPVVTMGTGATMTAVTVGTNATAAPIGFRCSSTRPFGGAHRLIGGGGGNGGGVGIGGADFSPSSGNTGSDVRRSHGGSAPSNQFYQPRVRQPAVPASRDPVHDDGAEEDDD

>883

MSVPRKQVEGWLAEFLIKASEVILQARVIPLKTSSHPRHMSTLFQLRLAEWFDVRQEVARGIRGYHFDRPFTFSVEIYLNKTDADGRPGDQLLVERWTFKFHPNTLAEKYSDRLYNKLCLGLRTLLCFTRLLPAYHCYVDAQRGAHATSRAPGRKDGMATARLSHRVHFRDINALANYNCKEVTSSDFLNLPCSFGVLSVGVSHRLELPFPQKPLTEDAAASAFGYVDNYEVEEGYVTCNPPTHQPRGQSPSISLSPRPALGASSHHHHQHHHQHRSWGHHHHQQAERGWTMNVLTGEGRRHKSGSDDGDVPPLPSGPSDSSTGNMMAAGTPPFAQPRHLHHRPTGISPSSGPGVAPMSISPRDHGLLGRGNTPPNVTLPPPLLRDTSQTPTPTPSSAHTPWGTTQHMRTSPRVGTKASDYSPGRGEGLPPRPHTPHSRGSAPQTPTSRQGSGGYGQGVFGPLGTPPLGYQSRTTSRDCPSPVLGVSPTSEMLPSPRPDEGFGDLWMASHPAVRKQSSASLMSVGRMSRKSSGSGACSATGAGSEGGHPVIFDDGEEEGEIFMLDQDKGEEEAFEDDEDSHVLASMIGQLGEGAGAFVIKKDDTSDWPPTAPLNLPSPAPASPSSSPPTPFSPLLQRLGFRPSPSPAPSPTPIPPSTWPWGRLVSRGSSDRRSIDRERLSRPVLHLLEVLCRAVEEGSNRGVPVDGRKVSDAAMRAVQYGGTNEGGVVQQVLNQSGGGRWQGGRMLVTLDLPGATQLLQDLRTSLYGYVHHQSVSKSSTPPASMRGLPAHPHPGSMFESCMDSSGDEKMIDEDSKTKRDRFLASVGDLMRHCQQPRPLAIAQQTIPVRELFRRLEHFEKLGEKIRSRPRDTF

>884

MEQSRGFTIGGWVSDGKRIGKGAFATVYLGHHKDDPSELVAIKVVDVDRLTRSNQKLKRHLDSEISIMKSLQHDHIVTLHEVFVEAEYIYLILEYCVGGDFSDYLKKHKRLSEDTARSFLRQLASGLKYLHSRNIVHRDLKPQNLLMAAKPGRLGGDNGDDSTRWELKIADFGFARFMEPQSVASTLCGSPLYMAPEVLLCQPYDAKADLWSVGAILFEMLTGSPPFNGVSSDCMDLLQALLKKNKEERITWREFFSHPFIVHDTAALTAFKTSTSRAIPISHSGSHGGHRKRSATIGSAPTNVGYILPPSPPMAVGSPICASLPPHRMESNFISPPRSMPHAIPSTNHYYHPSFLTPSSLPHNISSSPLERGGGGFLVGSGHPGYSSPPQFAASPPTFTGSPSSPGSVMHIRTAGAGSGDLNSPPYLRSPNSSGGFGEGFGSGWGSGPRKPLTNPFKESFDLSPFPSTTIDPKEKESAGSSGADDSFELLESESSTSNSTHTTPEERRIVDALELMVRKTAVVTELADSKVDESPIDALALYIKALHIYHSISQYAKKNAQSQNLTASKRLGLVVERMRSEFKATLKKAEYLKRNLKPNDSCPPAEKSIYESALQMGRDGAVAEVLHNFSKAESLYVRSSQLLQLLTMDASHPSDKAVLESSYQLTTFHDLAMPADIASFEKRLGEVRKSQAKLDDHVEDSLNVAND

>885

MLLDFPRSEQKRNDAQANNGKGYSSPSSSSHEGDRLLRTRDTGFPRSTTATAVIHNNATAAETMTAAETMTAATKRATTAGPHAASDEHKRYMLQEVARPQQQQQQQQQQQQYTQQPTSLQPAAPPQKKQHGRSRSSVSTSREGERRGNTTIQSEPAVVGGGSGRSDENNSQGYVVIEAPQHFQRHHHHQHQQQHNHRQGYRDKSISEADDDEKYRKQQQSSSSSQCSAEIEIEPTAGMFRPPALATAAAVAAPNSLPARRRAASAFSIAGPTSAYKQHHHHQQRRKQEEEDQQQQQQTQRQQTTTPIANDNGKNSRNRPPEAVAAAAAAAAIRSFSDAAKKALLVARVGEENEKEHKFVEAVQAYVYSITLFHRLLADIQRWKKQFSSGSSNDVAATATPINTAQGAGPVSKEGGKKKEHPVSSAAAAGPKGKIDRLEKWSLQMAKVFLHKVRSLTTFLSGKESIKVSVEELIYRHAIKVAKDAAFDETFGAHALARGKYKEAMRLFELLLCSGSRDHDSSNRNDDDDGDGDEGKRDDDKEGKGRKERDSSPKCDEHDATILGKFISEFRSRIEGRVANILQDLHMMADVEFDKRVNLCAFM

>886

MSSVKSTPRQLLSARALQSVPHRLYFLSSDISGSALQERPSFLWQSAGLNQDFNLWGAMSGRASIGDYVVTSKLGSGSFAVVYKGYHKTSKIPVAIKALSLHKLNGKLLSNLEMEIAIMRQIDHPNVVKLYEIKKTDKHMYLVLEFCAGGDLQHYMRRQQQQSGSNVLTENVAQHFLRELAKGMQCLWQHNLIHRDLKPQNLLLVEDSPTSALKIADFGFARHLATASMAETLCGSPLYMAPEILKFQKYDAKADLWSVGTILFEMVAGRPPYGGANHVQLLANIERQPLRFPPTLQLSRSCHHLLVALLQRKPALRLGFADFFMNSFVDLQPLPEQTVNMEALRHSPTLATTESIREEDEEDVQESGRRGMVKTVESSALEAGDDQNGEHCLRNFRRDSGSDRPFSASQGLSASSHVANSVPSANSRPSRSGNLEVMEAAFACAGSGSGALRRSSSSRLARSRRGSSSGSGLAGANTGTSLRCSPQMSPHVLPIPSPRINPFKQLSDKPYDSRAHQQLQQQQSLLNVASSQLRHNDGTGPSTLRNITGGGHALDSSGEYVLVDESSERRVADGADEFQDGAKDRCMSCLSTQEDLMTRALTSGSGSNIPTETFSREYGQQLIDIVVLRTQAIAPLADQLWALSTASATNSSCRADPTTESVGEVNRLSQQTAERILPSSAQGEKDFNCANLSTDFFMSSSLASSIGVSHTLGGEEVGDDDEENYVKRVAECEYIYAAEALVLYVKCLRLVQRVVLYFRHVRVSRVSPDSPLAGWSNTSLKISLAFLAEHLTHFLVRAEQCKVRMAHASRFQIPIRSIASQEELLYANALRIGRQGAIREVLGQARAAHDHYLQALLLLESLLLDAPGADGSSTIALAVDDQKNINVFLRALEMRLKNVRILLDEDKSSSGASRSNHMHQFRQQLTHLPSATAAVP

>887

MIPQSNPTAQRRSGDAQNTNNVAPNSVATTTDTALTASTQSGSSSNNANKKLEYIGVYKIGPEIGKGSFATVYKCIDTTNNKAVAIKSVYRSKLKSKKLLENLEIEIQILKSMKHPHIVGLLDYKQTTSYFHLVMDYCSMGDLSYFIRRRNNLVKSHPVISSLLHCYPSPEGSHGLNEVLVLHFLRQLSSALQFLRDKSLVHRDIKPQNLLLCPPVHSKQEFIDGEFVGMWELPILKIADFGFARFLPSTSMAETLCGSPLYMAPEILRYEKYNAKADLWSVGAVLYEMTVGKPPFKAGNHIELLKNIEKANDKIKFPSAAQVPEPLKQLIRSLLKYNPTERISFNEFFNDSLITCDLDDNDQPLETSQMDENLFISEYISPIAPAERSQFFKEQKKNDSVVRSPSPTTATTATPRQDNVVQQMTKITSPVPDDFALSIARNSSEFNLKKDDMNLEKDYVVVEKRAVEVNALADELAHAGAGADAIPNSRKNSDVDQTNRLASSQQQTETASYRRSSSSGSQKRPSFSERRISLSLSPTNALTKAIGLASNRLFGLTTNSSHSNVSAIAEDDDSSTSNNDASSFSTVIPSTNNHVLLQKLNLATIGEPGTEFDLGSVSNLDEQILDKLELIANIANAVNLYADVKFSQIIPSPPSSDGIEDDTEMLPPKIIHMISQEGIGLYIKTLSLLGRAMDIAGQWWFEKYDAVHGERPSFETTVRINQIVQWIREKYNISLERLEFLKSKSDFTAEETIEDNEPNGTTRVQQAIFAAALGIARETALKELLRNSTDIECSYVTSIYMLLAILEDLEESDRQEVKKIIEKINSRLKNFMGK

>888

MRIFRRFERGTFSHQHSALRIAWQLHFVIRAIVLPAHLLTLCACLLTSERLARSPLSYKNRLSLLELSNTHILKLAQKASRIHTATSQMALQRHTRPGDPAAPTGSDAEAALKIVGRYVYHPQTDRIGRGSFATVYRARYADAPGYVAIKRIKKQKLSARLNENLDREVDILRLVKHPHIVQLYEIQASKENVYLIMEYCDGGDLSQFIRKKKLLPEELVRSYTQQIASALEALRMFNIVHRDLKPQNLMLVKRETVIKIADFGFARYLQTDTMAETLCGSPLYMAPEILESKQYDAKGDLWSVGVILYECLVGHAPFRADNYLELLRTIKTSKDRIPLPPNASIECRDVIAGLMCVDPERRIGFDDFFAHPFVALQQYKTRLRTLDPGFAKQDEEQRQLAVAKHLASASPVPADASASQRLQATQATRRPSHPTDVVDPIIAASDARQEGRPRAATHSGGDTRTHLAQAQAQAERPAQPIAQTGKSQAVQILSAVQRAATAAAATAAAAVINRVTSSPNLAANPARSTPSRGSVSRPPRAETPPGVSSLDEFTFTDGTPADNGRSPADSGQFVESQPVSRPNSAMNAFTAAPALVSTKPQPQTVQGLAGSVEDYVLVGPSQQVSVNAFADAVARNKVPSSRFVPPSTTTTTTTTTATTTTTTASSGMPVPQKSGASVAMNQAMTDDLRQHSPGTDANRGEQAEPDEEDERTLQTASPTLGERFGEHRDNSFASRVPLTNPATNFRERAKTYAFGTSPSRGSTPHTPPADEDRAVQLALATSPTNNTTPHSPTLMGTSRVILNKFADLFYSSSASSNVQPVTDAYLLGTSPSRRAMSSSSRSFDENVLFSRIESAAQHAFAVQRLAKILNSGQQSTGPLNDDGTQALLLYMRALEFYFEGINLSRQLVRNNASIPSDVNSMIQLLRSKINDCIDSVDSLRSRVPPNAPLSFDLSVEKVIYENSIMLAREAAVRELLGHFQDAQEAYWCAYFLLESLLMDRGETPLEESDKALVKRYSASVSKRIHILQQRLSTSNGSQPISLSSSVAAGSPTHQQNALAAQLTAFTTGNSLYQHGGQLFGVSPE

>889

MASGASAKSVGPYLVLKKVGTGSFASVWHARHKTSGQEVAIKISTEKLNKKLTENLDVEISIQRSLEHPNVVKLLDILRTERTIYLVNRPVYSFNLLLSSDGDDATLKIADFGFARYIDKGNLAETLCGSPLYMAPEILRFQKYDAKADLWSVGTIIFELLTGRPPFTGTNHIQLLHNIEQTAELKFPDTLPLSQSTMSLLRSLLRRNPVERCSFEDFFKHPLVGLIPSPQPPTMLLLSGSSAASGPSTASALVEMHPSPDVLSPAVAEAEFADTQLAPAETGKDHGEIRTSPANVEANNLHFDHSSFSTSHSDADRSGGAHCESSPSNDASVNRVPIRQPRTNPFKSESSAPSRRTLTDSLDKEYVMVDAVPGDIPATASCCSQSERNVGVLGKQCFDKLVVALDWKARNYKLVRELADAYSEGGRWVDATALYVRALHILQGALLIAQQFVGNFTTSAGRVSGKFQWLKERFIEVAEKAEECRAQEQSVDVVLRLVAK

>890

MKRVGDYILDKRIGWGAFAQVYKGFSIKTNEPFAIKVVDVCRLADKNSKLTENLNYEIRILKELSHTNIVRLYDVLNEETDPTFIYMIMECCEGGDFSKYIRTHKKLTEEKALYFMKQLANGLKFLRQKQIVHRDLKPQNLLLSDDSEHPILKIGDFGFAKFIDPFSLSDTFCGSPLYMAPEILHRKNYTVKADLWSVGIILYEMLVGEPAYNSGSVPDLLNQLQNKKIKLPSHISSDCQNLIYSLLQIDVEKRISWEDFFNHKWLNLNNNDSYKNNSGNYFNNNNINNNNNNNTNNNNNNISYPISINSNNTNNNNNNNNNNNNNNNNNNNNNNNNNNNNNNNNNNNYYNNNNSPPNVYHASSLPYDFNNNNNNNNSNNYNNNTNVPNSLPFAYNNNIYSSPTEAIPQPTTLNKSKSLENTGNTIRAHPFKDDKKSTTIQQPQQQQQQQQQQQQQQQQQQQQQQQQQQQNRQLSNLSTDFERDLVILDGEELESMERVFNRAVAIAELGDLRQNEPLECVPLYILALKLMKSKIPNDPSSSPDKFINTFTEYKRKLVHIFSTSNTSVKNQDHHSSFSPNRFIYENALEFGKKGAVEELYNNYPTSLQFYTDGTLLLEYLSSIVIDSDDQEIIKKYLNAFEVRTQICKKNYENSKNTVLNTNSIQNNT

>891

MSEPRCFKRVGPYDLFTTLGQGSFAIVYRGQHRISKRTVAVKAIVRARLNQKLQANLEAEISILQTLQHPHIVRLHDVQTTERHVYLVMELCPGGDLLRVIRSQGAQTEAQTRGYMLQLVRGLAYLREHNLVHRDLKPQNLLLSSHLPDATLKIADFGFARYMQQADLAETLCGSPLYMAPEILRFHKYDAKADLWSVGTIAFELLTTRPPYTGANHVQLLQHIEASEVELPGGISQECASLLRSLLRRDPTQRLSFGSLFTHPFLYPPELSGGRPAFPSKALALHVKALDLTQQAMQLAPSQAPTAAGGSEALAPRDEAHEDVMRERFSTLLERAEWIRQQLRLTASEASRGGATTACVEELLYRHALGMGREAAVDEMVGKWAASRALYQRAKLLLEQLAEEPLVGAADRAVLSKYAAGFAWRLREIEEQLAEERGGD

>892

MNHELYLPQSKVKVVAGYALQQRLGSGSFAVVYKGVRLPSANKGPDTVAVKAISRTSEKLTKKVLQNLEIEISILRTYQHANIVCMHQVQKTERHFYLLLEYCAGGDVQGLIRSRAAGRLSERLARRLMRDLKSGLQFLWGQELIHRDIKPQNLLLTGPLPLDEEDDPAQTPAMEQERRDANFPSSQFCLKIADFGFARHLQSQSLAETLCGSPIYMAPEILQHQRYDAKADLWSVGTVLFEMISGKPPFHGENHIDLLKNIKMKAVRLPPDVRVSPECVTLLRLLLNRNPISRAGFKEFFEANDRFVALGCNGASVHDPGSCRPVVGDLETIPENDGRTIAGASGDENGSSTEMTTTTAMPQSLPVAVVNVGETTGYTKPRLGPLQTSAPVMVSQAHYPSMQRTLTPLVPSPPSTAHYFKMPQGAPEPPALELNAGRIQSATPMQEMETYHRSQTTNSNLQNSTDDNSYVMVEHGSISSAKTATVAWKEPSYRPTVLGTSPILGGRRDYPTLRQARGMLSTSPGTGGTLMGMFSSGKTRQIGGGNPTSTKRAESQLQEAATMLATAEDIGRRAITVAHVGDNRAFLALRLISMNESCTSMLSATAMDDIEEESRYNDCGNVTDDSSATDVIAVTRRRRGSSMSDKSMEKADEAEEMPFAINTEPLPSLDTTIPTRRVSNSSITGSKMVKPTPSSIRSYFSDALLCYIKALSMLKGAVSAVTKVSQELEELQKHVIPTSHANHLQQLKKRLEVTTEWLAGQFRGVLERADATNAEISKVSVPTGEQVRSPSVEELIYNHAMTCGREGAVKQLLGQLENARSCYRSAGLLVETLLMEQRLGGDDKKVLENYVDGFAARINEVDQTMLQQSRMSTGGSSRRASGVISLVGQDN

>893

MIEKGEIENYILQSRLGSGAFAQVFRAVHRTSGNVVAIKMIDVYRLTERNSKLKENLNYEIKILKSVSHPNIVTLYDVLEPPPPSDSYIYMIMECCEGGDFSSFIRKHKRLTEEKALYFMRQLANGLRFLRMNDIIHRDLKPQNLLLSDNSDLPTLKIADFGFARFIDVQSLSDTFCGSPLYMAPEILYRKNYTVKADLWSQQQQQQQQQQQIFQQQTYQQQQQQQYTTNNNNNSSSNISSGSGNNKSNSDRILLNNQYVYNQQQQQQQQVVSTKNNNTTSSSSIDFEKDCVILDDEEAINGVERLGKRAVAIAELGDLRQFEPSECIPLYLKALMLIKSKLQQSAQNFSFGSPHLARYNQLMEKLRDTFKEYLNKTVRLYTNNLEMNASFSPNKLIYESALEMGRNGGVEEMYSDYNKALQYYSDGILLLEYLYSIAIDREDQDILNRYIKSFSERIITVNRKCLNK

>894

MASHGKGVPEASQVGDYIVEYEIGRGSFATVYKGHHKTTKEPVAIKSVLRSKLTKKLLENLASEINILKGIRHDHIVALVDCRETETHIHLVMEYCSQGDLSQYIKRKGDGPASLPPPPEGGLHEVVVRHFLKQLASALEFLRSKNLIHRDLKPQNLLLHPASSVKEGSGYGLPVLKIADFGFARSLPHLSLAETLCGSPLYMAPEILRYEKYDAKADLWSVGAVLYEMCTGKPPFRAQNHVELLKRIEKAQDVINFPGDVDERSGSSGASPSQTGHHFTPISDDIKDLIRQLLKRNPVERISFEEFFMNPTVSNLASQSRSRASRRSASMHETRSDSPLIEGKRSTLDPSRYIYESVPRDRNEKRPELMQTGVKDQSDKPLNPPHRPRPRSIQEVSPPSQDHRPPLQLPISSPSPHHAYVTPSQQNYNELSSQPINTSRRIKPDDVTGSSPKSVSNHPSPQPCRRGTLVGIQDVSRRDSAPEDDALLDKDYVVVEKRTVELNELADKVAASPVLSSRTRGGSNQTSTLSPPIPTSLTQYRTPPPPPFIPYPYSTNTQSPPANNLFFSTTPPFALPAPTPEEPKSTIPLRSSVGTPPVPSPISARSGSPRATSTIAKALSMASMRLFGVSSSPPSRRSNTPLIGFAAPTNRGAEINPEENTVLQSIEDTAHKAYVVFQFADSKMKSIRPLDEPDYSPPIKTHRTSRGLLTPEEQEPSRESILAEEAFFLYLRALALLQSGMDVARRYWEERRQETKPASVRLNNGVQWIRDRFNECLEKAETAKVRSGIDQRDESGVVIEKMIYERALELSKEAALKELAKEDYAESERSYQTSIWMLHAILDGAGEDGRSEAIEEDDREILNKFIRSIENRLTLLRRRIASART

>895

MSKTVNKGGVASASARASSASLAAPVSRNSTGRTSVSSYTGGPPNYRRLERLGKGSFATVYRAVHLVSNQEAAIKDILQERLTLKLQENLESEISILKNHAHRNIVRLYDIERSDKHIYIILEYCRGGDLQRFIKNQPGARLGEATAQHFLRHLAAGLKFLNDRNLVHRDIKPQNLLLTENSPHAVLKIADFGFARHLEGSSMAETLCGSPLYMAPEILGHNQYNAKADLWSVGAVLFEMVAGRPPFAAQNQAELLRVIKRPLQIPAGVDLSAPCLALLQRLLKREPLERISFEDFFTNDFIDMKGSGILLEENHYHVSAGLEGVTSQQVPLSLPGQEGSSSPQGTTSPVYTPELPPAAAAETLALRQTRISESPSPWPANVSRRAPAARPEHGPADERDCESQEALEAIWSRAGRVAAPLAAVARRAMLPAPGALPFQTEGAKTSQVPHPTPRGNPFKPLAASPPGAVALSHQGRSTSAPWHSSRRTSSPPSSLPSSSFSSGPGGTGGGDGAESVESDGFVIVHAGVPQDTSLKLVRQPPPRPSYRPPASLFHHPFRDQEHPTRQHRQQHQQEFASCASKMPPSGGLQLQLAQTHAVKRAEDGQPEPLSCAAIRSTDSVEGALRVLAYVEHSTRRAVLIASLGDAAAIAALSVRRGVLKAGSGCLASAVQTILLHRDWCSSPDRAGKESVFSSTETSSSSLHSSSGSLSTAAREGAKASALLVDALVLYLKSLSVVGTAIKALRQVVDVIQIHSDGQLDKLCAGYGQRCAALKEAGGHESGRECALSTCGDPFGASAGMLSTSSLQERISSLQGWLELHFSVILDRAQECRAQMDKQGQEPAEKPDLGRIGVVAGEESEWAASRALVTRSAEELLYKHALSLAKEGAVKELLGQWGRGRELYAQARLMMEALLAEPLLSLEDQTTLQHYQAEFCAREEELARADSAARIGGNIHIKEG

>896

MGDIKNKDHTTSVNHNLMASAGNYTAEKEIGKGSFATVYRGHLTSDKSQHVAIKEVSRAKLKNKKLLENLEIEIAILKKIKHPHIVGLIDCERTSTDFYLIMEYCALGDLTFLLKRRKELMENHPLLRTVFEKYPPPSENHNGLHRAFVLSYLQQLASALKFLRSKNLVHRDIKPQNLLLSTPLIGYHDSKSFHELGFVGIYNLPILKIADFGFARFLPNTSLAETLCGSPLYMAPEILNYQKYNAKADLWSVGTVVFEMCCGTPPFRASNHLELFKKIKRANDVITFPSYCNIEPELKELICSLLTFDPAQRIGFEEFFANKVVNEDLSSYELEDDLPELESKSKGIVESNMFVSEYLSKQPKSPNSNLAGHQSMADNPAELSDALKNSNILTAPAVKTDHTQAVDKKASNNKYHNSLVSDRSFEREYVVVEKKSVEVNSLADEVAQAGFNPNPIKHPTSTQNQNVLLNEQFSPNNQQYFQNQGENPRLLRATSSSSGGSDGSRRPSLVDRRLSISSLNPSNALSRALGIASTRLFGGANQQQQQQQITSSPPYSQTLLNSQLFHELTENIILRIDHLQHPETLKLDNTNIVSILESLAAKAFVVYSYAEVKFSQIVPLSTTLKGMANFENRRSMDSNAIAEEQDSDDAEEEDETLKKYKEDCLSTKTFGKGRTLSATSQLSATFNKLPRSEMILLCNEAIVLYMKALSILSKSMQVTSNWWYESQEKSCSLRVNVLVQWLREKFNECLEKADFLRLKINDLRFKHASEVAENQTLEEKGSSEEPVYLEKLLYDRALEISKMAAHMELKGENLYNCELAYATSLWMLETSLDDDDFTNAYGDYPFKTNIHLKSNDVEDKEKYHSVLDENDRIIIRKYIDSIANRLKILRQKMNHQN

>897

MPSSGGFKQVGAYAYRPTVDKIGQGSFATVYKAKRQKDDEIFAIKRINTTMLKPRLLDNLELEIQIMTRMSHPNIVGLYDEMESSEHMYLVMEFCEGGDLSHYIQKRNKTDKGDIQPPMGLDMVRAVAQQIGSAVIHLSKHNIIHRDIKPQNVLIQIQKDGSMCFKVADFGFARYLESELAATLCGSPLYMAPEILASQPYGWKADLWSIGTVLYECLTGRPPFTATNHIELANVIRDTWDKLTFPDGIDEDLRDLLHRLLQRDPALRMTATEFYAHPFMKLEQYEAQESVVIGDVNEADRLAASGIEDFNAGTADTVPATTLQAPLSTPQSPSTPAPIAPQAMSDSVTITTPDAQATPASDIPPHPSVPSVAPLTALERDTPQHHSSGVAGTDTVGGDSVGTDTVEGDGAVRTSTTENPVAMERATTRGPGADESASNQATDSGTRERLREKRFTRQVSFDGQVTHITDRETVSMQPHPHPHPHHVSGPDVPGLRVRLASPQPPAGSPEGSELRPNTTELGSNTTELGPNRTGLGPNVAAPPSGGVADGRGTDTRGSEADDTLTPAAEERHRNDGERYGSEQDWYNGQGGPSEGSSGSARDRHTGVNAPARRKSCGDGAGVRHLLTRTQRDGLGNTTSPATHPHPHPTLHPHRDGGSRTSHRRSLEDVREHKHGHTDTLAPAHAPYHGERQRRSSNDACLMERRGSGEHEGRRRERSALTGKGPSSGSIPIQGVRDYTPEHEHRHADAYTRYHDTAHDRPHSSSGRETSANADMRVYSEGRYASHHTQRTDQSTPHPRGQAQGTEDKDMYGRATRGDDRRRSSNESEESRRLSNENEERRRLSNESEARRRLSNENEDRRRSSNESEERRRSSNVNMSGGGGGGEMVHFGSSGGHDNYRQGDIGSFKSPIPRWAAELGLTVRQSPSSQFKGATPTQPRLNPFVSIPNTGRLGLGNTGLPQGMHALALNDGNGIDSPSGDYDKQAYDVAHLGTSGPGMLSGVHDSGSSNSSSRDETASYVIVDKDNVVMNAFADEVDGRYERSIRQTYGTSLPAQTNLYLNDARMGQRGSTGSTESIYTHQHQPQPQLQHDDLRSPAYATCQETPPKRPTPTTHTQQHTAALGVHTQPAQQHRPHSSMGRTDRSHHRVSEKGRDGRDVGRRSSGEHPHAETLTSTPYNTLQDASTHTHAYAPAHGGKGVGGSGNGSGNGSEAAPHVYKEPPRRSTMPAAITNVFNAAKGYTMNLYGGSGAGSGAGGGRSVASISEDSPLDSGILTEIQVIQDIDDYTRRGDVIVWLGGKYINKGRQSLGDSGTAPGEAGRVARLSQSTAARDRHGHLCALSLFVRGLKQYRLALDVATQKIEQARTVTVAINQAVQQLRDRYNSCLAKAEKTRAKLIDLGQMMGIGSDRMDTPNADKLIFDYTLNKLCKDAAANELLNDGENANRMYKCAVWLLLAVLTNIPPDDADGRTIVNLIQSVQVRQEVLKNKKKR

>898

MSSIPSTVSSTAVGDYIIGHEIGRGSFATVYMGKSISSGQPVAVKSVSRDKLNRKLAENLEGEIKILKGIQHEHVVALLDIMKTDKHIHLIMEYCSLGDLSHYIKRRGLVGGATVEAGAWNPLAGPWGGLNDVVVRHFLKQLASAMEFLRAHSLIHRDLKPQNVLLSPAPQDSPAVRIPNPAKPGTYVTVPPLPMLKLADFGFARALPSQSLASTLCGSPLYMAPEILRGDRYDAKADLWSFGAILYEMVTGRPPFKAQNHIDLLRKIERGEGIILFPGEDMASMVAGRRSNALSSSPSRRLGTSPSSSPRFPHMQGAQPISDDLKDLIRKLLKRNPSERMSFEAFFADPCVISNRAIGGHTVGSFNASEGLLRGTLRRSPSSSSQKGTTSIPSSIALEGDPPFALGPSSTEPLFDVTNRGHIAISEGLGLPRGDAQMPAPLYRVHTSPAKQTTVTHPSSLRQSQTQVLGMAHPNAVPAGRVEADMLASSSQYTAGSGLGHPTDVYDHQSMRHPSPSSAPMRHIQRLDMQRQTSAPVFHVKADEIVYGPHPPTYGYSDNLRLERTGSERGRMKIPGDDSDYRDEVRKEASVGFEGEKVPPFSSVVVEEDVSHARKVDMLDHTRGGVSVAIEPPFPGYANVDPSIFVHATGSVEPVPGSSAQAPGPEEDTRDGNNNSSLSSLGSLELSDADADDVVGEQETVGDPPATLSPPVARRLETSARSGSGSGSGSHGNVRASLEEYVVVEKRVVEVNWLADEVAGAVGKNAQDLGAGESGPVSPISAAAGHAVFLPPARGRSPSTGSSQGVPVPISGLVGSGTSSSASVSSGSPHSPRSLRLEPKTQGRIFGSLRESTHQFLNVSSSNSGGSGSPGGILGTTPPAAYAAAATAGGTGGRTGSPGPNIHSGLEVVQTEGYHPLLDTLNLCALRGHAVQQLGDSLLSSSSVSPTTAEEALGCYLLALRCYQLGIEVAKGVWESRSGGVNLRILSTAVQWVRERFNECLDAAETCRGKIEGSGVEEGRRCVERVVYDRALEVSRTAALNELSSQPSHSQCESMYRHAILLVEALLLMGDDCDVDAEGAMSDEDKRVLERFVASLWGRVGAVSRKAVG

>899

MGKRIGQYELKSKLGQGTFGTVYLGKHIPSKNKIAVKMINRVGLKPEQQNRLEQEILCQRSVTSDYIVSLIDVQKTENNFYLILEFCEGGDLGQYIKSHGAVSEQVAQKWMQNLAEGFKALRIKNIIHRDLKLQNILMTENSPKAILKLADFGMSRFLGDGLAQTWLGTPLYMAPEMFKNKEGYDSKADIWSLGIVMYEMLVGEPPIKAQRRDEIPSAQKNLKAMPENLSPACLDLITRLLAYDPKDRISFDEFFTHPFIRKEEVKSLLITESNKSNKSESDKPEITQTDDFVLLDDQESCTDFVFLKTDTHPAINLSEIISLINKKVQISDVICKLASKLKINKEINGAYALIERATELLEISVDKGKELIDTHGLVPNSYPLFFEQVDKVKSLFNDCKRKSEELYKEVETSAEEQKKALKYEGKASENLAENLILNYSITLCKEAAKDEYLRDYLRASEKYKEAIVLLEFLIDKKDGENSDWEKFENFVVETKRRCETVTVKLTTA

>900

MSKSSTGRDERIGDFVIENEIGKGSFAVVHKGYRLQPREPVAIKIVIRKKLTPKLLDNLEGEIAILKAIHHPNIVELKECLKTEHQIYLVMAFCASGDLAQYIKKRFDIYERAGMAEPDSLTKGFKPTYPHPVDGGLNETIVRSILTQLAAALEFMRARDIVHRDIKPQNLLLQPPDAAFLALGNPREIPQMKVADFGFARHLSVNTLAETLCGSPLYMAPEILRFEKYDAKADLWSVGAVLFEMTVGKPPFRAANHVELLKRIERGEDKIKFPDERSAGSLAREAARRQELGEAPLPPPHPVSEDVKILIRQLLRQRPVSRMSFDDFFASPVISDFKAFIRPRAQPEAVERYEDLQRSERSVIIPSSGIKHVSVSSIEASTQQPGVQPPVSTATSPPALESRSTQEASPKAITGETIAPNKTPREDARPPRTLPRAFSAKYVTGEPPQPEDLEKRVPPTMTRTPSSPGIPEGSLLSGERDEAPQATTEHFGSSKGGEDSFLGKEYVLIEKQSVEVNALADELAASPQSRLGLASRRPSRLSRLSSGPLPSAPGASPPTAPPTILSSKPIRIANNTNTASTGAFALPPGSRPSSFPRRASLSSSGSPSTRQGGQVITNMDAVASTQSNRRDGNASSFPKDEVSVLGQRLAGFGLSGSGVGGGPSSALAKAISMASLRLFGVPSGVSLRDAAALVRTRAQRRGIARATDSLDEAEMTLLSTLEDLGQKAFVLSEFADSKLAHFFPDGPHQLSQELDSSTATSGISPSRNSVQGSARRVGSISSSSSSAVDPVAAEAASAEALMLYVRSLAFLQRAITLTKRHVESRSRPGVPAVTSAELNDVVQWLRARFNEVYDKADFARSRCSELPESAQQVDKLIFDKAVEVARAAATDELENNREGSGWDPSHCLLAYETANSMLSSLLDPGEDAMSLSEGSILMIDGYVKSINKRLWTLQEQFGGGVGAVGAAGASPVGVDAEARPGVSRSRTESP

>901

MELGGDPAKAVPYLPKDKQFLITRKVPCLMRAAALEKSANEAQNQDIVIDGDEGWVETHVGFAKDEPTEIPEIPTEEEAAQASKKDSEPTGGDEEEEIPDMEEFDDGDNLEDEDPSALAKEPEDSDLILKTRTYDISITYDKYYQTPKVWLFGYDENGNGLTPNQVFMDISQDHAQKTVTIDTHPHMQIILRQAECGKESRVDQYLFLFLKFLSAVIPTIEYDYTMEMES

>902

MLSWAQVPTCAGAMEGERNNTRRVAAVSDEKTPRHQPVEGEHVLDQVASDAPIIVEDPDDCAVVVAAGHDPSASAVHVYDYFVVYSSTYQVPVLYFQASHLDGRMLTWEEVWADVPSHHRDEDMRWNFITQQEHPVTGVPCYHIHPCETKALMTSLTADASTPSPSSSNYLMAWLSAVGPVVGLHLPLTAWLSPASTNAPPYHN

>903

MSNLTSMFKGIREYWTPVLTASRFETDAVLTPDEFVAAGDYLVFKCGTWRWEAGDPAKRRDYLPADKQFLTTKNVPCLKRLHEMEYHASEKTVETDDGGDGWLATHYKGDGAPDVSDDSDLDLLDDVAAEFVEKLTMDAAGGASSSSSTSKPPARPAAAASHPPPPPPAAAAAIPDDIPDIDDMDDLAGGVSEAPDPAAVHASSSTKIVRTRTYDLAITYDKYYQTPKLALFGYDEARQPLTGAQMLQDISQDHANKTVTMEPHPHLAGVMVAAIHPCRHAEVMKRIMDKMREGGKAVRVDQYLIVFLKFMASVIPTIDYDHTMALE

>904

MSNLTSMFKGIREYWTPVLTASRFETDAVLTPDEFVAAGDYLVFKCGTWRWEAGDPAKRRDYLPADKQFLTTKNVPCLKRLHEMEYHASEKTVETDDGGDGWLATHYKGEGAPDVSDDSDLDLLDDVAAEFVEKLTMDATGTSSSTSKPPARPAASSAAAHPPPPAHHAAAAPEPDDIPDIDDMDDLAGGVSEAPDPAAVHASTSTKIVRTRTYDLAITYDKYYQTPKLALFGYDEARQPLTGAQMLQDISQDHANKTVTMEPHPHLAGVMVAAIHPCRHAEVMKRIMDKMREGGKAVRVDQYLIVFLKFMASVIPTIDYDHTMALE

>905

MTSTVITAAEYAAAARRVAELTRDADGAAGGTAWTLHDADATRPDRVYLTARRISPAPAANVGAGLECVDDDLTFQADEVDVSDNEPARPASSWLQWEIHVVYHATYACPALYFRGSALDGRVLDEVAAVQALEYMRAASHLAAPVSTAMGPFASAVAAGPHPALGTPFLYLHPCQTHDLLAPILGPPGFTARVLLAWLSTVGAVFGGLVSTAQWTAVKG

>906

DNILRTRTYDLSITYDKYWQTPRMWLFGYDEASAPLTQPQIFEDILSDYAKRTVTFERHPHLDHPHASIHPCKHPNAMKKIIDNVSK

>907

MYRFQEAVRSVIGALRFEPEDDFSSSGRFSIKDFLEAGDSLVNLDIGWEWMCAKESFEIDYLPLEKHYLSKNNIKPIKFHEQPDNLKIFSDADSKELSTYENESEADGDFLMVKQPMEISTLWTILDDSSILETDTSSPSGLYRLSITYDRFYQTCRVWVEKQNDVNNDFNVLKDLLDHIKKGGGPIEGISLAIHPFTNEFAISLHPCQQVDYVQRFIETRSNTDKISPIEGMLFCINLVLASLPITSI

>908

MSASHTIHSMFKGVAEYWMPVLQSSNFKKKGVLTPEEFVAAGDQLVYKCRTWQWASGKENMVKPYLPKDKQFLVTKNVPSLKRASDYALNDAKEEIVENDDDDGEGGWVSTHVKEKSDKKDNRAKVEEVQEIDTFDDTGGEEEQGAENKTAVNGKETEDDADEEDEDDIPDMDDFEEDNLAEADPGMLLTDNIKEDGKDTTATKSDNKNSGQSSPLTNTEGKIIRVSEPEDNIEKTRTYDISITYDKYYRTPRIFLFGYDKNRQPLSPKQIMEDISADHARKTVTVDPHPHLEVPHASIHPCRHAPVMLRICERMEEENMRTLLKAKVAEAKKQGKDTPKAESLKLTKEEAERVAIRIEQYLFLFLKFISSVIPTIEYDYTF

>909

MSKRAIYENYKKVYNQLQGVKTEGTFQKTGTLTPKEFVEAGDTLVQKIPVWQWASGEGADYLPSDKKYLIYRGAMCFDRAPAQDEKDNEAIDEDGWVSTHIDHVPKARAAPAAQKEINWEDDEDDEEAADEDLQERRCRLYDVTVVYDQYYQTPRVFITGYSESNHSLPLTKDEMMQDVYAANREKTVSVDPHPFLKAASISIHPCRHAETMKRTLESLRERLDESQEDIPEAERVPFMFPTYLALFVFLKFISSVVPTIEYDVSIDLDM

>910

MRDGSLSYEAFCAEAELLQQRSHELASRQRVGRDKCVATWDWKHGNRQHLDGNSYLISTENVRQLPFDIDLNTLTAPDIVEAQHDGVGKFLRLCLASKFLQIHVEEVQKTVHQSGKGRLVLFEFHIVYHTIYQTPVLYFRALAEDGTPLSFNCVTNDVLFPGSDEQSTFVAMDMHPVLDEPFAFLHPCETAPVMQLLQAQFKDLSSLRNCNVPHYLASWLSLVHPLTGISPLDYYFRQ

>911

MDRKAHEIASRQHEARALKKNMGDSDLNSSFGSFEYKNNSQTLFGADGLFMQSLFHGVREYLTPVLTESSFEDKGLLTPEEFVKAGDLLVYKCPTWRWESGEPNLRRAYLPADKQYLITHNVPCRRRVTSLEENYQTEEVVEGEDEWVATSSKTTGTSLAASCVTDLSEEMDTMTIKESNSSKYSKRGILEAIVDEHFMPTAVAGSTPVLRNLSSFEDDDNLIEDDEAALSTYVVAQEPDDFNDAILRTRTYDVSITYDKYYQTPRVWLFGYDERNSPLTGDEMFQDIMQDYANRTVTMELHPHRNALIHASIHPCQHGAVMKRILANLKMRPVDSIESNEDDTKRIRCDQYLFVFLKFIQSVVPTIDYDYTIEVHAKQ

>912

MITNQQFNDNIEEFISIMLANLKLKGTDFEFSVNNNYVNKWNIDKMYSFVSVSVKQLRIDFTISFDDLYGVPLLNMRLYDNDTFLTSPMAQRTLAVGICRVDLHNHHLLQQPWLQVHPCETLQTIDSHLKNTPTCKYNSVIQYLCCWFGLYGLPSIFPQFSVRPNIYINNVQSCKIK

>913

MSLRSKLSSLREYLTPINHNSNFVTTGEISPEEFVKAGDYLVYKFPTWQWGNDCPKNLQKSFLPPDKQYLVTRHVPSYQRASNYLTGEDKKGANPEEDDEEEEEEDEEGWVKSKKIHKVIDDTHDSQINKGEEINDIDDFIDENAEEQEHDQIGDHELDDDEFDDLDIINDSKNNKLRRFDLYITYSTSYRVPKLYLVGFDSNGIPLLPQQMFEDINSDYKDKTATIENLPVAHNTTSVSIHPCKHSSVMKVLMKHSKLNKKNLQQKDESLSDDLSKLSVNEKKTQDEHSQINNDDKEEEEEGIRVDHYLIIFLKFIASVTPGIEYDYTMDAL

>914

MTAFLQSKLHGIGEFLTPVLKASKFKETGVITPEEFIAAGDFLVFKCPTWSWESGEESKRRNFLPPDKQFLVTRNVPCFKRVKSMEYSENDERVVEGDDWVETHIGHESLQKTDLQEIPGDSSAITAKSHASAAKDDDDDDVPDIEDADGDDDDIPDIDEFEEDNLEACDPSALAAPSAAAGDADDNILRTRTYDLYITYDKYYQTPRLWLFGHDESRKPLSVTKMYEDFSQDHAKKTVTMEAHPNLSITMASIHPCRHSNVMKKIISNVEVAGKELGVHLYLMIFLKFVQSVIPTIEYDYTREFSF

>915

MSSTPPPSSEFLRCARALCVLSDQLKDGWTMEYSPSRRYDHIAAMTQPAPRPGSVYLVKRSVSVRAAGCSRSSSESIQAAETQDQAQPVSPANPGLEAPIEDIPDMDDVYTEEASDPGELRGSSNLTSDSASFDSWHTYEYHVVYHKTYTVPVLYFNASTLEGKRLTVDQVWNDMHNLHKDSIDVSRSSFLSQAEHPVLGKPFFFLHPCQTCDMMVALFGPRLAAHCSPDQPLFDYLLAFLSIVAPIVRLNLPLQYHAAQSKLVS

>916

MRHAEGTPRTYRHSTGPFLRPSAGSKASSTPASAGSPVHSNISSRISFVFILACRNFSERKQSRMLAAASWRQALQALRETDNLHPWQLRETQMVSAGRTGVLCCPQPPSGTHGAAAAPALLDYHICYSHSYQVPVMHLRAVSLDTGLPLAPDAVAALFPSWPQRPEQLVGFVGLQEHPLLPGPCWLALHPCNTGVALALALELGGAALVAAAAAEDGAEEEAAAAAVAAVAGAAAVEATAVEQCSGGAAAGAMATKSGAVRTGAGPGAAGGRRVAVEGGGAGGGGAGAGDGGGCRGEQGNEETAIPDVDDLLTAMAVAQSQAGAAQPLQATRAQVAEGGMGQSGVAGRSRGGISAGADGEAGGGTARDWAVVGPAGVRWPGGEVPVCPGEPAWLARYMRAWLALVAPHVGLRAGP

>917

MSNLRHTLHTLFKQTVETVTPPLTKSQFEEKRVLTPDEFVAAGDYLVHACPTWSWEGGDPKKRRTYFPPNKQFLVTRNVPCLKRATELEGYNPNSEFDVGGGEGEDAWVATHSNPAAASGSAGKGEVPSIDGAGAGGSGGAGAAGGNKDDDIPDITDLELNEADDEAAAPSGRPYLRAEEPADNIMRTRTYDLYITYDQYYQVPRFWLVGHDESRKPLLPQQVMEDVSEEHARKTITVDPHPHLAGLSAASIHPCRHADVMKKLVDNLLEAGREFKVEQYLVLFLKFIASVVPTIQYDYTMSVGGE

>918

MITAEEFAAGAQMLAAAWGRCLAGDTQWCWQASTHPFAASVRTVLPPPLPPEAAPAAAGALPPTDQPSSAQRKAGSPGPASTSSGDVGFAIQEEEEDPASVAGSPAGSSSPPQLLTLTCHVAYHPSYRVPVLYFEAVGPNGVPLGLGAEHPLLRRPFYMLHPCQTEAIMQLLTNPEDDSQLDGAPAAAAAGSREATGATALPPQAAPAAHGRMLRYLLAWLSVAGQPLGLSPPVELWLQASS

>919

MSGLRSFLHSTYKSAAEAVLPARSQSAFKEKGVLTPEEFVAAGDYLVHTCPTWSWEAGDAKKARPFLPPTKQFLITRNVPCLRRAAAVEEYGEKEEQEVEADEEGEGWLTADQSSAGRAAAGTTTTNAEGFEEIPDVEEEAGGGEGGGVADAAAASASDGGAAGPGGDEDVPDIDDLDLEDEEEEDEAALRPAPTAGGGGGEEHIVRTRTYDLLITYDKYYQVPRFWLIGYDEDRQPLTPGQVLEDVSEEHARKTVTMEPHPHGGVTGGVQAASIHPCQHANVMHKLAERVAGEEGEFSAERYLVLFLKFIASVVPTIEYDYTMAAGGGA

>920

SAMRHAIHTAFKSAAERVLPVRSESGFKTHGVLTPDEFVRSGDYLVRTCSTWSWEAGDPAKSKDWLPKDKQYLVTRKVPSNKRASELEQYGMRAEANTMVEDAEQDGWVAPPATQDTRDAEEIPSSHPESAAGPSVEARTNPEDDIPDIDDLELEDDEAEKDEAMLPQAAAITSPGADQDHIMRTRTYDLMITYDKYYQVPRFWLVGYDEKRQPLKPNQVLEDVSEEHARKTITVDPFPNTGVLAASIHPCKHASVMKKLASMAEASGKPFQVDSYLVLFLKFIASVIPTIEYDYTMAAGW

>921

MPGSAYLVRTERSLLRPAAPPPVAVCEAVGVHEEELVEEIDSSCLRLPDEPAGEAYVYEHHVVFSPSYSVPVLYFRASRSGSPLTAEEVWANIPHRFRADSAGAAVEHPVLGSPFFFMHPCGTADLMAALLRPSPGPGEGEGYGA

>922

MNALKKLAEYLPASTTSQYAQKGTHVDRGRIRARVRQPWQWSRGSKEHAKAYLPPDKQFLLLKGAPSVRRKEALALADVVGEALAAAGDEGDAWLDTNAGEAIDLTSHIREPAEDDEDVPDIEEFDGQNLATTSGPVLTATEPVDDNILKTRSYDVSITDKYYQVPHVYLFYDEARRPLQPSQLFEDVSQDHAEKTVTIENHPHHASAMKRLADIAAASGKEIRVDMYFTFLKFIACIVPTIEFDLLEG

>923

MQSITHRLADQFRSVVGNFIPIDCSNSKFESDGFLTPKEFVDSGDYLILQFPNWFWRSASEEYIVRWLPQNKQYLHIDNVPCRKRLDSSKLCISKNCLDITSDSKGDEWILPTNENVEKLGNININDDVRYYDISVTYDKFFQTPRIWLFGYNKEGYPLSTEEMVEDIISDYATKTITLDPHPFTGILCVSIHPCNHSSLLKKMAKNHPPHLSIVILLKFITTVIPSIELDNTIDIDIKFDT

>924

MLTSKDFRDQAINLIKKWNNIIDEIPWQWNQINELNNESKGYFTTKRYHKINNNNNNNNNNNIENKNNNNIENFEEIKETIDDSSTTIIKSNNNNNENNIIIFQFDIIYSKSYQVPVLYLNGFSSFDSSPLSWNEIWNNLPLSNLDKNQQSTIPYITQVEHPILGNPCYQLHPCETDNLMKLILLKEKDYNDNNDKKEYFKDYYLLSWLSIIGPMVNIKIPFDLLKNNNI

>925

MLTSFQQAVHKAYVKTVEKVTPTLSTSKFLEEGVLTPEEFVQAGDLLTDKCQTWTWESGDPSRNVSYLPKEKQFLLTRNVPCYNRVRTLENESKASKADEIQIEDDGEDSWVAPQPVGNQDDIEDEKVDISSLKIDDKKPNTTTTTTAKPTNNNNNNNDDEDEDEDGDIPDLDDFQDDNIIEEEDPAVLSKNNKTTTTTTANNNNNNNSENKVEDNDNILRTRTYDISITYDKYYQTPRVWLFGYDENRKPLKPEEIFEDISEDHAHKTVTIDSHPHLGISFAYIHPCRHAAVMKKLVDRQSENGKEPRVDQYLFLFLKFISVVIPTIEYDFTLEFDT

>926

MLSLAKRIRETVTPARKTSAFLSQGVLTPEEFIEAGEQLVFKCPTWTWEAGDPSKAKDCLPDKSRQFLVTRNVPCHRRAKDLEGEYVGDTEVRQGESASMTAAAPATGAQQNQREEQGGVYLTMRGWSPMWWQIPREEGGMGGATATMAMRRSCSWGVGRGGLLGGEGGATDQDGVAAGLGAIQLVESHFTSPAKAATSTADTAAALQEAARETSTASGAIADFSGAGEEGKGSGGGPAGAGGGVGAGGGMLEDEDCPDLDSYVEDNLMQGDEATVQAGGVSYLTAQEPDDPILPTRSYDLSITYDKHHQTPRMFLFGYDESGQPLPAEEVFEDVMQDYARQTVTMEPHPHLSSHHASVHPCKHASTMKIMLENLTKGGKEARVDQYLFIFLKFIQSVVPTIDYDYTMSVDAGGGNNNDTSEPDLDDAFVDVAETSP

>927

MLSQSEFERMARQFALLHHTAPTWAWFEPGPLSDSGAFAPPPGFLRSSPIVVQPPIAARRQGGEEEDPPVLEEPICSTADSSALDVSALDDQPQPDLLRIHICRSATYQTPQLLLLGCHGSDGRPWDVDEARAYLQRQANGSQPLPDQMLTPAEHPGLGLPCVALHACHTAELLSPMMRDAASCSREGGRPPLDLLGAWWSLVAPLLGLRAQPGPQHTVNT

>928

MSGGHSTYSGALWNMGRNAMLGTAEYLTPVLTTSQFLEKGVLTPDEFVEAGDQLVLRCPTWSWQAGEPDKARPFLPPNKQYLVTRSVPCQRRAASLASGADAEMLVDGDEGEGDEGWSLTHANHKVERQEEAPDMLADAVAHMDVAGPSGAAAGGGSEDAGGADGAAGEAELEMGGMVEADDPSALGGGSGIVKTRTYDVTITYDKYYQSGRVWLYGYSESRQPLTQAQLLEDVSADHALKTVTLESHPHIAEGAGLHMSIHPCKHASVMHKLVAELQSAGREAKPPQYLFLFLKFISSVIPTIEYDYTVAVDGL

>929

MNFNKKEFNFQAKKIVNCLNKLGKYQWKWDTRGFIIHSCELGELLQVDDNHCNEEIYEEKDDTICQIQQKRYYFIHHILYNTSYQVPQFGVSLFDKKENKYITQLEQATTLLSHLSGIEKCDNNNPLNLFITMNEHPLIEDYYMFFIHPCGTSSSLLPIIEFSLSDYLICYLSSYGPYLCCFENWLSVQHKLITENQN

>930

MSVLSTLKRKAYETYTKSVDLVKPTLTESQFIEKGVLTPEEFVNAGDFLVNKYRTWQWVGASDCKKTVDYLPADKQFLITRNIRCATRAQGGPPTKTETLIVDGEEFEVPLEEKPEEVFEEDSDDIVDADDIVDADELSEEADDTVATVDVGKTRTYDISIIYSHFDRTPKVWLLGYDEDHKPLTESQMFEDLSATHAGQTATTDTHPFLDIKEIYIHPCRHAQVMKKRVDEMIADGKTPRVDLYLMIFLKFLATVIPTMEYDYGKDF

>931

FNALCVQFWQRHGTFGWQLVDHEDEFEGRRVYLKHIQDRSIPRHGRDGLDRGAGGADCFVEAALVQDDAALASDAVSAAPKSAFVSASFEVHVVYSVPFSVPALYFLAWSLEDGRLLSLEDIYGSVPPHLGGGRGTDRGAFITQLEHPILEKPFYCIHPCRTAELLRTWTAGNPQKGVGGPSLLEVWISLMGPVVGIHLDRSTMAAVGSGHPAPGGAVDPG

>932

ETAPAAHAVVKSRTYDLTITYDKYYQTPRVWLFGYNEEGAPLTKEEVFQDIFSDYSNKTTSIETHPYQGLPCVSIHPCRHAEMMKRMIDRLNARYQEDDASDGVKMMMRPDLYLLVFLKFIQAVIPTIEYDLGAIDL

>933

MSLAYQTYSKYKQVAEFFIPVKTSSSFQEKGVLTPEEFVAAGDLLVNTHPSWAWQAPREASRLTPFLPPSKQFLIIKAVPCLRRANALNVDAEEEEVEGGDWLDTYKDHHPQDEEVTEMAMAVKAVAVQPPAPAPVVVDEDDDDPP

>934

MTSESGTVMGKLRGWREYMAPTLKKTAFLERGVLTPEEFVRAGDELVFRCPAWSWERGSNPPKSYLPEDKQFLVTRNVPCPTRVSDMEAQLGLIQQQAQNNDDDDDDWLVSTLVKQNTHSLEDEFDILDEEGEVVKSHPNEEEEKPAEEEEGDEYADMADYEDNNVLVDDDAAAPSTAGDTNLLKVRTYDLSITYDKYYQTPRVWMVGRSAEGQPLTGKEMMEDVMTDYANKTVTIEVHPHVSGPHASIHPCKHGKVMKTIVDNLMAAQQQTDGNEEAGPSIEMYLFIFLKFVSSIIPTIAYDFTMEVNTGKK

>935

MTSESGTVMGKLRGWREYMAPTLKKTAFLERGVLTPEEFVRAGDELVFRCPAWSWERGTNPPKSYLPEDKQFLVTRNVPCPTRVSDMEAQLGLIQQQAQNNDDDDDDWLVSTLVKQHPSSLEDEFDILDEEGEVVKSCPNEEEEKPAEEEGDEYADMADYEDKNVLVDDDAAAPATAGDTNLLKVRTYDLSITYDKYYQTPRVWMVGRSAEGQPLTGKEMMEDVMTDYANKTVTIEVHPHVSGPHASIHPCKHGKVMKTIVDNLMAAQQQTDGNEEAGPSIEMYLFIFLKFVSSIIPTIAYDFTMEVNTGMK

>936

MSMFDAMKHAVHSVFKETVESVTSVSHVSQFREKGVLTPEEFVAAGDMLVYKCPSWSWEAGDPSKAKDYLPKNKQYLITRNVPCHQRVAALGESVREIAMEDAEDEEWTYTQTLGAAKDGEAREIEDIDAEEEPKSMETSGTEEVKAPVTAAPESQDEDVPDLDDLELDEDDAILPDDPSVILPSPAAGAASASSGFVKTRSYNISITYDKYYMVPRMWLFGYDEDGRPLTHAQVFEDMSQDHAKRTVTIDPHPHVPGVSHASVHPCKHAPTLKRIIDQIESGDSGRQMRPDQSLFLFLKFISSIIPTIEYDFTMDFDA

>937

MKYEEFQSAAMAFMAEANDHGQEWKWVPNHPRPLQNVGADDRGYLALEEVYREPLEIDSTENRNELDDEDSCTFIDPSVIKSETAQPNMYRYHVVYVEAYQCPVLFLQGKSIDGRLLDTEYIWRDCCGSMNHEQSWLTDSCLPTPEEHPVTGEPFHFVHPCRSKDFMDAIMQAFLPSVQEPCTQSNLRYFKLWLSVYGQAVGLRLPLALHSSNPHPT

>938

MQTHLTWQQFKLQCERLIELVNQYRGSGIQQNENQDSFNWYWLETDDRGTGCLVLKSIKTINTELPQQINVSNNKDIDIESELEENDIQTINSSNERDITTSKLNQKFVHYEYHISFSVSYQVPVLYLNVYNSDSSFIKWDDIWNLLPLSSYDKSNLSSIPWISQVDHPLLGIPFYQLHPCETANLMNQVLANNNIVDDDKCSTTITTQNHENDYLLSWLSIVGPLVGIRLPKEILILNKNLK

>939

MSMFTSIQQSIHKAYVKTVEKVTPTLSTSKFLDEGVLTPEEFVQAGDLLTHMCPTWTWESGDPSRNVNFLPKEKQFLLTRNVPCYSRVKSLENESRASISDILQLEGEDDEEWVAPKSGNPDGVIEQDLQKDMSGLKVSQSNNTTTTTTTKPTTIDNTKPTSDDDDEDEDGDIPDMEDFQDDNLVEDDPSTLKSNQQTNNNNNNNNNSNNNVSLESSTGNANSNSSVGDDDNILRTRTYDISITYDKYYQTPRVWLFGYDENRKPLKPEEIFEDISEDHAHKTVTIDSHPHLGISFAYIHPCRHASVMKKLITKQSENGKEPRVDQYLFLFLKFISVVIPTIEYDFTLEFDT

>940

MDYIKNKLNNVRMGITTALIDPPTESIFFQEGQLTVNEFVQSGDRLIQSCPSWKWKNAISDKYQNNLLPSDKQYLLLERVPCNQRICELQENINIQEKLDNEDDWVINEQIQQEKKQVENKQIEEEQQLKQIYDFYYYTPRLYITGVDENNNPLTQEEIFQDIINEYANKTVTFEEHPHLGTQQASLHPCKHAKVIKHMVDTIQGNGGIIEPHMAIQIFLKFLASVIPTIEYDIANDFYL

>941

MNEQYNEVVEKNGFLTMSDNKNNTDNNNQNNNNDNNDEFIAIPCRTYDISITYDEYYHTPRMWLSGTNEDGKPLNTQQILEDIMSEYQGETVTPEEHPHLGLKQVTIHPCKHSQVLKAFIEKAKENKVNLKPNQCLLIFLKFMSSVMPTIEYDATTDLLFDQI

>942

MSIRRSLYEGFKNVHNKLHNVKTTSDFQTTGRLTPKEFVEAGDELVQKNPVWQWVGGPESVQDYLPKEKKCIVYRGAPCTERAPVDSTNASPEAVDEDDFVLTEATQVALPATALEEEKVLTWDEDDDDSGEEDVVATATDNSNLRVYDVYIVYDKYYQTPRMYLVGYASDHVTPLSMGQMKEDVYRSNYGKTVTIDPHPVLSIPCISIHPCRHAETMRSLMHRMQENYNREKANDPNAESFVFPTHLALLLFLKFISTVLPTIQYDVSSGFHLV

>943

MSLRRSLYEGFKNVHNKLHNVKTTSDFQTTGRLTPKEFVEAGDELVQKNPVWQWVGGPESVQDYLPKEKKCIVYRGAPSTERAPVDPTNASPEAVDEDDFVLTEAPKVALPATVIEEEKVLTWDEDDDDSGEDDVVATATDNSNMRVYDVYIVYDKYYQTPRMYLVGYASDHVTPLSMDQMKEDVYRSNYGKTVTIDPHPVLSIPCISIHPCRHAETMRSLIHRMQENYNREKANDPSAEPFVFPTHLALLLFLKFISTVLPAIQYDVSSGFHLV

>944

MDNIKSAIYTRYKEVAECVLPVLKHSKFLEEGVLTPEEFVQAGDLLVFKCKTWTWEAGDPHRAVPYLPDKNKQFLLTRNVKCATRCEAFEAEMEKKAPENSIRIDGDDDDWVAPIASETNTSEPVQEIGHAAKPAAACVPRPSAAEDEEDDDDDDAPAMDLETFDADDNVVVADPAALRPSAAAEDAQGQQDDAIARTRTYDISITYDYFYRTPKVWLIGYDENGKPLTPQMVFQDISADHANKTVTFEAHPHLGVPQAFIHPCRHAAVMKKIIQRHLDAGKPPRVDQYLILFIKFISSVIPTIEYDYTMEIEG*

>945

VAEQLMPAMTKSAFAEKGVVTPEEFIVAGDFLCPTWSWQGGDEKHARSHLPRDKQFLVTRNVPCAMRAAAMEEYAGKEVHLDGDDEGWVAAGESGGGEDASRDDDDADAAAKAKAGGSAAAAKRDDDDDSDGDIPDMDDFVDLGAEEEEDDASAAAPRRGIADSDAELFSAAGATTAATKNPADNILKTRTYDLSITYDKYYQTPRVWLSGYDEHRSVLPPKKTLEDVSAEHAKKTVTIDPHPHTGAPAASIHPCKHAPVMKKLMDATARAGGGTPKVEHYLLVFLKFIASVVPTIEYDYTLSI

>946

MIKNLIRPLVSTKTVSTFYTDGKLTPEEFVAAGDILCRHCPTWKWESGDPKKKLAFLPPEKQFLVARGIPCRTRAKSAYGEGDGLKVGAGSSGEAAEDEDNGFNIVTKEEGSSGTSSSSVPSSATPEDDDDEIEDDDDMPSVAESSDPSSAAPNAAPSRPKCRVYEASLTYDKLYTSPRFWLRGVDEDGTTPLTQEQIFEDISEEHAKKTVTMEKHHHLDVLYASIHPCKHAHTMKMMMEMAKEAKKEQNKLKAEASGSAPSEDDSEEPIPVDQYFIYFLKFISTICPTMDIDFTVSAKL*

>947

MSFIDKIDRAIREKGKKEARKTATVPTKSTFLQDGKVTPAEFVQAGDLLRSKCPQWHWCTGKEENILPYLPRDKQFLELKGVPCRKRASAVTGSGLEKKLDEGEDDWVAPDEDVSKPAEAASSSAAPSSSSDDEDDMFDFSDSSSAPSSAGPSFRTYDATITYDIYYYSPRLYLKGYAEDGSTPLTPEQIFEDMATEHANKTATVDEHPHLDVPTASIHPCKHAQTMKSLIDREKEAGRQIKPEQYFFTFLKFVQTAIPTIELSGTVEAE*

>948

MAAIDRAIREAGKGIYRSNPTIPTKSTFVADGKLTPAEFVAAGELLRSKCPQWHWCTGDKDRMQKWLPEDKQFLELKGIPCRMRAADLMGGSPEKLVGSGDDEWVETGDDIASKGSEQTSSSASPSEGDDEDDDYMDFSDESSAPTSTSTGFRTYDATIVYDIYYNSPRLFLKGYAEDGTSLLTKEQILEDMSEEHARKTATVETHPHLGVPTASIHPCKHALTMKAMVEHEMEEGRQVRPEQYFFYFLKFVSSAIPTIEIAGAVETE*

>949

MLLHIKKATFVAFLSLSLSLKNSLSNQFTSLKPIHISQTLSLYLSVSQTKSLSPLHLSASLSNQISLSLSLSFNQKPQCDGDGDARAHRGSGLPEKQRKHLPADKQYLQTRNVPCFPQEDREVEEMIVEGSDDEAWVATSIQGMTDTVDELPEEKTQTTQAPVDPDDLDDDDDIPTLEDEDYSGAAVVDDDDSGAAEETVRQLRTYDITIHYDGHYSTPRVWLRGYAASGEPLQGDEWESDFSVDHANKTVTLEQHPHLPDHWVSIHPCKHAEAMKNMMDTVSDGAALDVKYYMVIFLKFLQVIIPSISYDFTSSFQVVRQMNPEEMQAAAATGAPAAAAN

>950

MTSSPSARLSYTDFEIAIKAFLDQVNGHLPWTFCEYKEPRSESSRGYLALSHLQHTHKVADAENNEENGSTNTSTDLQLLDLASIGDDLEDEEDESQLSVSKDGAHDEFLSVSYHIVFSPSYQVPVLYFNAFKSNDESAPVGLEHIYQSLVPHEWRDTVRNAGLSGGISQQDHPIFNIPYYYMHPCETVSLMETVLKANQNRFDSQEAFLKSYIAAWLSFTGQAIGLSLPIEIARE

>951

MNSVQNIFFGIREYLSPVLKNSKFKETGCITPEEFEAAGDFLVYKCPTWSWEGGEPGKRREYLHPDKQFLITRNVPCLRRVKQMEYTDEDAETQIESDGADSWVMSHSSRVFGGPIEEIAASMDEDEDEEEKAEAEAQAAIRAMEKLQLRTTEEEVAVGDDDIPDMDDIPDMDDYDDMEEEADPATLAQPVSTEVDPGDKILSVRTYDVFITYDKYYQTPRMWLFGYDEHRRPLVSSQIFEDVSQDYAKKTVTIETHPHLNMSLASIHPCRHGAVMKKIIEKIASDEGKEETTVRVDQYLIIFLKFMSSVVPTIDYDNTIST

>952

MSQQQTGTKGSTSFRAFSLFKNFAEYFIDVPKESHFYERGVLTPEEFEKAGDLLVSKCPTWSWSAGEPSKRKDYLPADKQFLITRNVPCLKRCSELIEMAKDEEPVEDGEWIATHINHTKEKEKEEIGDITGGVDDLVIHSAEDDEEDDGEAIDIDNYSDSELEDTIKDKGALEANNGDSILQTRTYDFSITYDKYYQTPRVWLFGYDERGAKLESEKILEDIHADYGNKTVTIEQHPHLNTQWASIHPCRHAEVMKKMVDRLVGGGSGEKQFVRVDLYLFLFLKFISSVIPTIEYDFTTQVD

>953

MRFYTIYNNLKDKITPILKESRFFEAGVLTPEEFVQAGDFLVSKCPTWSWSGGVKEKKRNYLPEDKQYLITKNVPCVNRAKDMECAAEDEGERITEDGWIEPYYQMKKQDINQIEEIDSDMKKLSVTETSNEQEIDDIDDIDELEEIMNEDDFDKVDPDELIEDNILKTRTYNICITYDKGYQTPRIWLFGFDENHIPLTHEQIFEDISEDHAKKTVTIETHPHENYSLATVHPCHHANVTKKIVDSMLEAGHEIRPDMALLIFLKFMSSVLPTINYDFTISLEK

>954

MPIISQKDHPILEVPYYYIHPCTTGQNMNSLFNINNNADKLKNIDDNNSDNKESLDFQKDNELEKYKNYIAAWLSTTGFIKVDIKYFI

>955

MAFYTIYNNLKDKFTPLLKESRFFEAGVLTPEEFVQAGDFLVSKCPTWSWCEGVKEKKRNYLPDNKQYLITKNVPCIKRAKDMEYTNDEMERIVEGEWIETYYQMKKQNIDQVEEIDSEMKKLTVNQSNNEQEIEDLEDLDELEEAMGNDNFEIVEDPNELTEDNILKTRTYNISITYDKGYQTPRIWLFGFDENHIPLTPDQIFEDISEDHAKKTVTIEAHPHENFSLATIHPCRHANVTKKLVDSMIKTGHEIRPDMALLIFLKFMSSVLPTINYDFTISLER

>956

MIREDGTLSAEAFEVGVREILNDRSGCVRGRWVEETTSMVRATTANGYGVLESDFREETLEIVEREGDDVFDDDHDDAVVRTATRGHVREYRVVYSATFCVPVLCVRARDATTRAAWTVSRLLTSLRRENPCVRTNDDDPVLTPYSNPHERENGDWACVHPCATAGAMRLLLAADAAATSPRRYLEAWLRCVAREVSLALDDD

>957

MHRLRHAVHEAFKSSVENLTPARTSSAYESQGVLSPDEFVLAGDALVRACPTWSWARGSDEKAKKFLPREKQYLVTRRVPCAKRAKDMEAYAGSEIALGGEDEGWVKAGEGRVVDGGGGDGEIPDIGTLTLDDASKKTTNDETMKIDDIPEIGEDFEDEDDDAVVLPSATEIARAAGRVNGGTEDDDIVKTRTYDLTITYDKYYQTPRVWLNGYDENSLVLKPSKTLEDVSAEHAQKTVTIDPHPHTGVPSASIHPCKHSSVMKKLVESMRAERGESPSVETYMFVFLKFIASVIPTVEYDYTL

>958

MFQGINNFVNSFVTPKPQDFYSKGWMTPEQFIEAGDQLTMTGWQWKKAQVKKGVDPPHPEKMYLIANATSQTRIKEFLSFDFQNNQGQDGFLCVDMSQKQQQALNEQETRVYTITITYDRKYHCPRLWLQGVALNSGLPLKHQEIYEDIMSVYQNETVTVEEHPYLHYQQVTIHPCNHSTTMKAFLDKAKQNGADIKPMQALFIFLKFMQSVMPTVVYDTTIDICLGVD

>959

MFYLLLIIKVQMFQGINNFVNSFATPKVQDFYSKGWLTPEQFVEAGDQLTMTGWQWKKAQVKKGVDPPHPEKMYLIANATSQTRIKEFLSFDFQNNQGQDGFLCVDMSKKQQQALNEQETRVYTISITYDRKYHCPRLWLQGVALNSGLPLKHQEIYEDIMSVYQNETVTVEEHPYLHYQQVTIHPCNHSTTMKAFLDKAKQNGAEIKPMQALFIFLKFMQSVMPTVVYDTTIDICLGVD

>960

MFSNLGNYANNLVQAVGAALIAPPTKSVFLTKGMLTPEEFINAGDRLISNGGNWKWCKAISDQYKNKYLPNDKQFLIQENIISYKRIKDLNRGGTFTEQQEGEDVTIIRSEEQPIQEITQSQDRYYTLYITYDLYYFTPRLYLSGKVDDRQLTYQEVKEDVSGEYADKTVTEENFLELNIKLPTIHPCKHADTLKFFVDQMRDNGCPEEKIHPDNSLTIFLKFMNSVIPTIQFDFVNTIEL

>961

MMGHFWAAREYLTPTLKTSAFLEKGVLTPDEFVRAGDELVFRCPTWSWQGNSRGSGSQASATKTYLPAGKQYLVTRNVPCQARVASMETAMDLQRGEDDEGDWLISNFIQHKERCIEDEFDILDETGEIMDVPKTATLEESDGNDEYADMADFEDDNVIRDDVATAVVVDRDDNLIKTRTYDLSITYDKYYQTPRVWMMGMSAEGQPLSGQEMMEDVISDYANKTVTIEAHPHVSGPHASIHPCQHGKVMKTIVRNLMQSSTDGDEGPSVEMYIFIFLKFVSSIIPTINYDFTMDVTASTSK

>962

MQSLFHGVREYLTPVLTESSFEEKGLLTPEEFVKAGDLLVYKCPTWRWESGELSLRRSYLPENKQFLVTRNVPCRRRVTALEQNYQTEEAVEGEDEWVAASSYATEGGNANVVTDLSDEMGEVSLSDVDKPKASTASKGGILGAIVDEHFMDGSTSAAAEPELRDLSSYEEEDNLVEDDEAALGPSASSYLVASEPDDAEDAILRTRTYDLSITYDKYYQTPRVWLFGYDERNSPLSGDQMFEDIMQDYANRTVTMEPHPHRGSLVHASIHPCQHGAVMKRIIANLKARNPGETETEEQVANEIRSDQYLFLFLKFIQSVIPTIDYDYTIEVNAKQ*

>963

MRNGSLSYEQFCVEAELLERRSHEVASKQEVGIDGYVATWQWRHGNRQHLDGDSYLELGDIDELLATDEDHVMTKQPQDTQTALLEFHIVYHTIYQTPVLYFRAYAVDGTPLPASSVMHDVQFPGSNGRSTFVAMEEHPVLGKPFSFLHPCETAGAMQLLQEQLQSTKELSELQVPQYLASWLSLVQPLTGISPLEYYSV*

>964

MSDQINVKHKIGDTCRKLYSYFKTVNNTSTFIQNGTLTPSEFVDSGDFLVYKFKTWEWQEADKDRVVPYLPENKQFLITKNVPCKQRIKDLNNIVHDLKIVDNDWLLPSYEEDNNPTDIYEYLPNSEYTINDKNIYNYEEEEEDDNCDEAIDINNFYMENNLIKEHDPASINSTSCYSKNMLHDNLMKIRTYDVSITYDKYYQTPRIWLFGYNENGDPLKSEEIFEDILSDYSYKTVTYDPHPCTGVMTASIHPCKHAEAILNVVNNWISEEKEPRHDLYLLFLLKFISGVIPTIEYDFTTDIEIPRDSNAGL

>965

MSDQINVKHKIGDTCRKLYSYFKTVNNTSTFIQNGTLTPSEFVDSGDFLVYKFKTWEWQEADKDRVVPYLPENKQFLITKNVPCKQRIKDLNNIVHDLKIVDNDWLLPSYEEDNNPTDIYEYLPNSEYTINDKNVQNVQNIYNYEEEEEDDNCDEAIDINNFYMENNLIKEHDPASINSTSCYSKNMLHDNLMKIRTYDVSITYDKYYQTPRIWLFGYNENGDPLKSEEIFEDILSDYSYKTVTYDPHPCTGVMTASIHPCKHAEAILNVVNNWISEEKEPRHDLYLLFLLKFISGVIPTIEYDFTTDIEIPRDSNAGL

>966

MIPYQEWHSQLQSLYDSQIFHNWALCQDVHLNDEKDGLLLRLIPTRQLQKNTERIENKLLNHIELYLTYSKVYNEPLLLLRIWEEKSIDGIPMTKLMLPTDIESLLDVQGKFQLGLDTIINLEGSVWYSFHPCDTSCIVGDQAEFMSTYLRRWVSIFIFSWLGYEDS

>967

MIRSTLSSWREYLTPITHKSTFLTTGQITPEEFVQAGDYLCHMFPTWKWNEESSDISYRDFLPKNKQFLIIRKVPCDKRAEQCVEVEGPDVIMKGFAEDGDEDDVLEYIGSETEHVQSTPAGGTKDSSIDDIDELIQDMEIKEEDENDDTEEFNAKGGLAKDMAQERYYDLYIAYSTSYRVPKMYIVGFNSNGSPLSPEQMFEDISADYRTKTATIEKLPFYKNSVLSVSIHPCKHANVMKILLDKVRVVRQRRRKELQEEQELDGVGDWEDLQDDIDDSLRVDQYLIVFLKFITSVTPSIQHDYTMEGW

>968

MSRVCLLVASKQEATKQKMKQAAQSTHQTRNTMSGGCITLDAFVEQGQALARDDPAYTWRTVKEHPLLQHPCWQVHPCKTKDVLAQVCASPPNATADDDDDDDDGADADVLAGDDPGEDDAEATLAADHSTQQREQPSRQHSYLEAFISTIGRLVGMPQPQPQRHRPPA

>969

MSQLRTKITQKVMLTLDSLQKGITDSNFSKTGKLTAKEFVEAGDFLVANFPSWSWAAGLAENKRSHLPDDKQFLISKNVPCFPREEREIQLDELKLDDDDDGWVQTSLVGAPEACGVVPDLEDDVPVKAQEELDDDAAEADLDDVPTLDDDDDLEGAYYKDAVEDDDEAALDPSGDADNIKKLRSYDISIHYDAHYSTPRVWLFGYDPDGKPLQGDAWKADFSPEHVDKTVTFERHPHLGYHCASIHPCKHAEGMKNTVELLVGDQGAVSAKFYMVIFLKFIQSVIPNIEYDYTSKFEVSPRQAD

>970

MEHFSIAKHRLGDALRSTVNAFTSASGESKFLESGTLTPEEFVEAGDQLTFKFPTWRWEAGDANLRASYLPQEQQYLITRNVPCKDRVRALDSALDQIKTEDDWLLPPEVSGSGCKDSLQDLDDLAGPSEGKEEAVGPGLNFNADDDFLGDGAKPSNLPDFSDLDKQLQQEDGYPAAATAPSVIVAEAPDANILKTRTYDLSITYDKYYRTPRLWLFGYNENGDPLTPEEVYEDVLSDYKSRTVTVDPHPLTGTPTVSIHPCQHAQVMKKVIDDWIEQGLTPRHDLALFVFLKFISSVVPTINYDFTMEIEF

>971

MQSFLHSLGESITPVLKNSKFKETGVLTPEEFVAAGDFLVYKCPTWSWASGEETKRRQYLPADKQYLVTKNVPCYQRVKNVEDIKHDEVDVEDEGGDGWVDTFHSRVATTQLEAELKDMTLNDTVRIQPIEVADDDSDDDSDDVPDMDDFEDDNLQEDEESVVRPKEEDNILKTRTYDIYMTYDKYYQTPRMWLYGYDEHGRPLSERAMFEDFSQDHARKTVTMESHPHINLTMASIHPCKHANVMKRIIDNIAGQGREDLPVYMYLMIFLKFVASVIPTIEYDYTRQTEM

>972

MPLSWPDFVADAVCFVERSDKINDGWELIFNHGCEGGRFIKKNTRYRVASGSIKKTTSREASDEQSDDTDSELSASELELTDESDYGIIDDNDEFAVMPQSAKNTWFDMEYHIVYSLSFKVPVLYLTGRKKDGKLLRSDDILGCLPAYQVATFRNLSTPVVTQVEHPLLGVPYLMVHPCRTEDLMQIVLSAKSEFTDRVVSRTRSDIYQASRTCIENNAASSGHNYIVSWLTLVGPIFGLKVATKYLNY

>973

MKEEILLLHKPYYPKFHVQVLFKSRLLINCPLTVMTVPTTQSAFDRTCQTFLSHSKKIGDTWRWVTPGAYLAKTVWVVDNSNEETASKDEESALHEPSDPAILSPAKPGITLEFHVIYSTTYACPVLYFCGHTSAGTGLSLEDIRQTVKGHMPDLDEQKRTEYDMPVVSQKDHPILGIPFYFIHPCHTADVTAEMLEACDSDANYFVFWLSLVGQLFSPLRLVEVGYVTSETCCR

>974

MTTTFHSLFHSAREYLNPILKTSKFKETGVLTPEEFILAGDFLVYKCPTWSWAAGEPSKRRDYLPADKQYLITRNVPSLQRVKEYGEVANEDEMEVEGEGDFGWVATHSNRDGKAAGEDQLVIGEIEEGEQDDVNAEVVNDQMKHLRIDDVHKGTAAPVPDAVNAETEEIPDFDDIPDMDEEDGMGVVEEEDPAALPTSAEVRRAPENHNTGSGPNDKILKTRTYDLSITYDKYYQTPRVWLFGYDEQRRPLTSSQIFEDISQDHAQKTVTIEPHPHELVSMASIHPCKHANVMKKIIDRLVDSGAEEGGKELRVDQYLMLFLKFVASVLPTIEYDYTTSMEA

>975

MENFGHSLGNIYRGAYSLIATVPDISEYKTKGTLTPKEFEEAGDFLVHRCPTWSWQGCESRYRQNYLHPDKQYLIARGVPCYKRISKLSASIMNITEMDNYSSIPDDLKPKDDWQVADISIDPEQKYEMVDESIKEGCKECIFIDKHESQENPETIESIKDENAVPILERDGYLIVNAPDENTINCQINDSIQAIRRYNLTITYDYYYRTPRLWLEGYDENYYPLSIDKVFEDVNPDYINKTVTGEIHPCLGITQVSVHPCNHSEMMKFLLDICHTDPEDIQVNKCLILFLKFLSSIVPALNYDFTMSTEFKKYNN

>976

MENLGHKIGNMYRNVAKKFKSVPTESAFLEKGTLTPQEFVVAGEQLVHRCPTWCWSTSTPGNEQKHLPPENQYLITRGVPCRKRVRNIEDSEVTEKEIDGGWVVAESHKVEENIEEIIIKGRDEVVEEDEEILDMEEVKESLIDENAVNIFSQDGYFKAEEPEDVIIKTRVYDLSVTYDLYYQTPRLWLVGYDENRNLLTPEQMYEDIMEDYAKKTATIETHPCLGTPQISIHPCNHAKMMKHFIDILASNNSVAQVHQAIFIFLKFLSSVVPTIEYDFTVDLSLE

>977

MNPDAYQEQVEILLRIFSQFPDISCLYDTRFLKIYFSLENDWEICIVYSHAFRSPVLYFRNRIQGLTLEEVHNHIKAYTEYISPAELPYTGEPYFFLHPCRSQNLLEGFSLATWLSVVLQVLGLSLPLGFYIEFNKFIGVNKSV

>978

MYRNVAKKFKTVPTESSFLEKGTLTPQEFVIAGEQLVHRCPTWCWSTSTPGNEQKHLLPENQYLITRGVPCRKRVRNIEDSEVTEKEIEGGWIVAESNKVEENIEEIIIKGRDEVVQEDEEILDMEDVKESLVDENAVNIFSQDGYFKAEEPEDVIVRTRVYDLSVTYDLYYQTPRLWLVGYDENRNLLTPEQMYEDIMEDYAKKTATIETHPCLGTPQISIHPCNHAKMMKHFIDILASNNSVAQVHQAIFIFLKFLSSVVPTIEYDFTVDLSLE

>979

MSLATPFVGVLKDSKFLEQGVLTPQEYIIAGDQLTHKCPTWSWMPGDPKLRNKNLPEDKQFLVSYIDYLNQYQVTKHVPCSKRIKDLQGEESKEKDLDDGWVETEGPIVKQNTDGKASADIDDIGDIDMEDIGGNAQQSEAVDIDDIDNMDNKEVNLLAAKDYIVMNEPEDTCHKVRTYDLSITYDFYYQTPRLWLLGYDEDGNVLKESEVFEDIMADYAKKTVTMEPHPHLGMKQASIHPCNHAKVMKKIIDTIQSNGGTPQVHQSLFVFLKFISSVVPTIEYDFTIDLELD

>980

MNVYDNIKNKLNNVKNDIISVVYAPPTESRFFEEGKLTPQEFVTSGDALINMCPQWKWMPASAEKYKNKYLPAEKQYLLMEKVPCDQRVQELMDSIAVNEKEDEEEYIINDQKKNNDQNIIETKIGQLSIKEHFTEEKQGGEEKKEDNDDDNVVVVEAPIERRYYDLSICYDLVTYTPHLFLQGVDEDNVPLKQNQVFEDIVSHYSNKTITFEVMPQTGIVQASLHPCKHSQVIKHMVDNINQSGGSIKSHQCLFVFLKFLQSVIPTIEYDVAGDIIFDE

>981

MNAFSNFVSSFKTPSPEDFDKHGFLTPKQFLESGDQLTLMGWKWEKVDDNKKLNKSLTDPKKQFLTFQGRSLTESKRMFLMIQKRKLIVKDLLRLANKKAKKKQINRQSNAQDNQEEEEEMRIYNFSITYDTYYHVPRIWFSGVDENQKPLKKEQMFEDVMPEYRDETVTLEKHPHLGYDQMTIHPCKHSQILKSFIDQAKENGRTIKPNQALIIFLKFVGSVLPTLEIETTTDLEI

>982

MDLKQDKYYQFNFEQFRQDCQQLCKSINLEGFEISYKQIDDDFRSNENSEYLEINHTFDYQEKMYLLKGSLVYSVAFKFPTLYFHIYDMDYQKPLDLLKATELIKQRELEINGAKKNIELIQDIHPVLGFSYLTFHQCRMREVIEQIQQIQVHKNQNKNNKETEDQYFFSPTITSLMILLKEFNIYLPKEIYKQLQIQQ

>983

SAFLTRGVLTPEEFVKAGDELVYKCPTWTWESGDPAKRKKHLPADKQYLATRGVPCTARVSSLENVVAVSNHNEGCGAIGGLDDDDGDWLVSQILTTEEVIRREEKALEDEFDILDGEGEDNSENNQGGADDDQEDEYADMADFEDDNVLEDEAAVVAAPVSTNATTKGENDNNHILKVRTYDLSITYDKYYQTPRVWLLGYADDGSSRPLTGDEMMQDVISDYAHRTVTIENHPNISGAHASIHPCQHGAVMKTIVKNLTREGDGGDGESGGEGSGGPSVEMYLFIFLKFVSSMIPTINYDFTMDVSASTKK

>984

MASMFKRMRESVMPTLKETRFQEKGVLTPEEFVAAGDMLVYKCPTWSWESGSTVRAFLPEDKQFLITRNVPCRERVAAIEYFDDNDELVAMDDGDDEGDGWVATHMDMGAGGEQLAAIADAMQPVSVPAVASMECSGSDSDSDIPDMDDFDDDDNVLEDDDDATAVFGGAGPSSGMEEANILMTRTYDVSITYDKYYQTPRVWLFGYDETGQPLSQEQIFEDISQDHAKKTVTFEPHPHSGIPHASIHPCKHSNVMKKIIDNLGLSGQTLRADQYMFLFVKFISTIIPTIDYDHTFDMEG

>985

MSVEWAEPWRAIESANGLYLACGPVMRGGEGWANLTQSTDDDWFDGGEVVDGEVDEAEALPAGHAAMVLSFHMVYHPGYAVPVLFFEAANEAGGPMGLAELWPRLPLSRGVGAGADAEQIQLTYLALAEAPHLPGRAMLMLHPCETAAVLEAVVTDGSEAADEAAGEAAVEGAAAASILASHATSPLAQFGQRDMLLWLSLYGPAVGVTTGTRVGPGLGSVTFTSRRTQVAAPRGMVASSQAAATAAGLAVLQAGGNAADAAVATAAALAVTEPCSTGLGGDAFVLYYDAESGTVQALNGSGRSPAALDAEAVAAAVTAADPAAVRLPWDSVHAVTVPGAAAAWMDTLEHWGSGAVTAAEVLAPAIALAEDGYAVGDVTACGWTAGSHKLTRSGNAAGSELLTAGGCAPSAGEIMTNPGMARVLRTLASDGKAGFYSGWPAAAILDVLSAHGSYMEASDLEAHTSEIGEPISYAVDGVRLYECAPNGQGLVALVALNILASRTEELKALGHNSAGYLHMLIEALRMAFAAAGPYVAEPKAHGEFAHLLSPEWGVAAAGKIDAGKASANGAPSASSDTVYFCVVDAEGNAASVVNSNYEGFGSGLVPPGTGFTLQNRGCNFALRG

>986

MTKPQETPGDAASSSSSSSSSSSSSSSSVSPLHGKKGVEASAAERSRASAMSKLGSFGSLSGSNVTHRLADMGRNLVASFTSAPTASSFISKGMLTPSEFVDAGDLLTHKFPTWQWKGVGPTGKRASGWLPEDKQYLITKNVPCYRRVRDMDDALNTRVGHDVEGGWMLPLLNDEEREGGSSGEAPDLTQSMQNLRLNAECGHELRKPAPPTPTQTSASTASNQENSLRDDVPDLINFADIDCLVQEDDDPAAAEAPSVVRTSPDAEIVAARSYDLSITYDKYFQTPRIWLFGYSENGVPLLPEEIFEDILTDYAAKTVTVDPHPCTGIPTASIHPCRHASVMKKVVDSWVESGVRPRHDLALLILLKFVSSVIPTIEYDFTMDVDMLIHRSTKNEK

>987

MDFQTKIKNFVKTFSKDGWILKESNGKTYATNERRVLPVEINDETVFVTYYIDVDEVFQAPVLSAYFYTESGHRLTYDELLKIMPEKLDMNSVSERIHPITGIPLFFIHPCKTIEYITPIEHHGIDFMNAWIGVYGPLFLYRLPI

>988

MKNQIHQKWMSFVNKYNSVPHTSTLIKDGKLTPEEFVAAGDCLIANCPVWSWCSAPEGHEVDYLPKDKQYLINRRVVCQKRATDLSKMMQEEVDIGDGWCQAGEAAAQAALEINDDEEAVDLDEIDIDEIEPEVQEVDVIDYRTYDISICYDKFYNVAHIFLYGVNNEGVPLTLEQMYQDISADYADKTVTYENHPFTATKNLSIHPCQHGHVMVRLVERLDHPEKFCAPMYYFIFLKFIHTVIPTIDISTPTLDFEA

>989

MSVKNAIHQKFMAIVTPYLKAPHKSTLNKNGKITPEEFVIAGDSLVAVCPNWSWAPSPDPKKAVDYLPADKQFLINRHVVCQDRAQDFLKLVDKEENVPEFDDWYQTGEAAKEEVVDLDDEEVIDLDDINIDEIEPEEIENKDAPVCRTYDITICYDNYYNTAHVYLYGVNPQGVQLTLEEMYQDISADHVEKTVTFENHPYFNQKTLSIHPCQHHNVILKLIERLENPESFCAPAFFFLFLKFIHTVIPTIDISTPQIEIGENRT

>990

MNKQSLYEGFKKVYNSVVGVKTTSSFHETGTLTPMEFIQAGDELLHKMPVWSWAEGPENIQPFLPPNKKYLVYRGAPCYERAAVAGNDDADEIVEDDDDEWITTHANRVLKATTEIAAEKTINWDDDDDDDDDANNNNNVVVVDSSRKDEGDDDEDADRDQTERRRCRLYDVYMVYDQYYQTPRIFLIGYAEDHVTPLTTSEMMEDVYPVNRERTVSIDPHPFLQAACISIHPCRHAETMRRMIQHMKQRFEESSPETAKFVFPTHMALFLFLKFISSAVPSIEYDLSTGIDI

>991

MDSLTCTRGEFDEMLEDLSKRGLGWRACRRSDSFGRTLKWLEGSSIIQRDAPCGQAEQLLVSYFITYSECYSQPQLYFAPEHPMSPQEMCTWMGKVCYGAVERQEYDAPVVSMNFCEEIQMAVWGLHPCDATQLMMNTLANGVRSENLLELFLRSVGHFVGVDERLLPLHVAGQQK

>992

MNKRGLYEKYKKLYNCLNGVKTVSNFQVTGTLTPLEFVEAGDELVQKMPVWAWAEGEEGIQPFLPPRKKYLVYHGAPCYQRGPDADSLGENEMEGEDGWVTTHAERQPSKNVVMAPAKTINWDEEDDEDQDHAEDIGERKCRLYDVYMVYDQYYQTPRIFLIGYAEDHTTLLTKDEMMQDVYASNREKTVSIDPHPFLKAACISIHPCRHAETMKRLIQHMKTRYEAEGANEAEKDAFVFPTHMAFFLFLKFISSVVPTIEYDLSTSIDM

>993

MDSLTCTRGEFDEMLEDLSKRGLGWRACRRSDSFGRTLKWLEGSSIIQRDAPCGQAEQLLVSYFITYSECYSQPQLYFAPEHPMSPQEMCTWMGKVCYGAVERQEYDAPVVSMNFSEEIQMPVWGLHPCDATQLMMNNSANGVRSVNLLELFLRSVGHFVGVDERLLPLHVAGQQK

>994

MNKRGLYEKYKKLYNCLNGVKTVSNFQVTGTLTPLEFVEAGDELVQKMPVWAWAEGEEGIQPFLPPRKKYLVYHGAPCYQRAPDADSLGENEMEGEDGWVTTHAERQPSKNVVMAPAKTINWDDEDDEDQDNAEDIGERKCRLYDVYMVYDQYYQTPRIFLIGYAEDHTTLLTKDEMMQDVYASNREKTVSIDPHPFLKAACISIHPCRHAETMKRLIQHMKTRYEAEGANEAEKDAFVFPTHMAFFLFLKFISSVVPTIEYDLSTSIDI

>995

MNALQTHFWAVREYLSPVLRESKFKEHGRITPDEFVAAGDFLSYKFPTWQWCAGSSSKARDYLPKDKQFLISCGVPSLRRVSQIEKGVGVGVKDDDEKLMSFGEEGGADAPEDDQWVATHFDDQQTGSSSQVADMLDIPDIGEDQLAPEHQLTEGQQDDLAARVAGVTIGHQSDSIHASASGDGMGDIDDIPDIPDMDDETDELAAGVHEDEDPATAAPPTHNAARTGWASAGDNGKLLSVRKYDCIITYDKYYQTPRMWLVGYDEHGVPLKPAQIFEDVSSDYAQKTVTIEPFPHGHAGPDSSVTSSASAVGVATASIHPCKHASVMKKVIERMNASVIEEQRRAAACSGTASVAGEKKKKKGWSVSSAVKRVTGGATDGSSAPTAEAKEDGSTTAEAAATEAEDDVDGLRVDQYMIIFLKFMASIVPAIEIDATQAL

>996

MSIVMSEVNMTTSGWCATRCADGGSESVRIRYEEFEEYCQAYLKRRDEDMVEGEAGRDLSWLAYSGGWRWTGAVMANGTRYDCTAGMERRFGVVVDTKRLMGMDGATLADSVVEDDGDEALDRADCSQTVVLHQSILYSATWKVPVLWFSAHTRAGEPLGASDLLNLRIIHHSGSLDSSHPFLHHDTHSRPRTNMHDSLTIHDPPAPISLDHSPFPPLSISDHPRTSLPSFFLHPCNTHTALHLLLSTPPSCQPDPSTSQCPSRYMCAFVSLCASAVEMRAS

>997

MQSAHQIADLYRSVVSSFTPVPTKTQFREKGILTPQEFVEAGDQLVFKFPTWTWEPADAKREVKWLPPNKQYLVTRNVPCKARVKDLDTTLTQGEGHDRDWFLPGEKGGDVGEDVPEIKDLTLEDNAKDADKDKASGPGLVVQENFINENKDDIPDLDSFADVDNLVKEEDPASAAPSSAPAAMDGQGKYFVCAAPDADIVRTRSYDLTVTYDKYYQTPRLWLFGYDENGVPLKPEQIFEDVMTQYATKTVTVDPHPCTGIPTASIHPCRHAQVMKKVVDSWVEQGVAPRHDLALFVFLKFISSIVPTIDYDFTMDIDMALPSLTAGIGRK

>998

MNVKHTLHTLFKSAVEKVAPPLSKSQFEEKRVLTPEEFVAAGDYLVHACPTWSWEGGDPKKRKSYLSAEKQFLVTRNVPCLRRATDVAGGSSYNPMSEFALPGEEGWVATHIQPTPGGAGTGPGGDADIPSMDDDEDSPLQAGPAAAAAGADAGGGGGGGGGGKEREKEKDGEGDIPDIGDLELNEGDDEAAAPSASAAGYLRAEEPADNIVRTRTYDLYITYDQYYQVPRFWLVGFDESRKPLLPHQGIGVMEDVSEEHARKTITLDPHPHLPGLSAASIHPCRHAETMKRLADKLVEGGRDFRVDLYLVLFLKFIASVVPTIQYDYTMAVGGD

>999

MTSSMNSVLGSLRRAREWAYPQLSESAFLEKGVLTPEEFVRAGDELVFRCPTWEWSNGGKQENVKSYLPPNKQYLITRNVPCWNRVASLETNLKLRGLDEGDDAGDGEGWMIASDNHGDDDIDGEEPEDTLEDEFDILGDDGEVVEVDPVKERETEKATAASTTELEPEAEATDMDDYVDEDVLYYQVPRVWMVGRDEHDHPLTSSQMMEDVISDYANKTVTMEPHPHVGSTCRHASIHPCQHAKVMKAIVRNLMRPATDGSNSSNDDEEPLSVPSVENYLFIFLKFVSSMIPTINYDFTMDMHGALEKWNASGRWDSIWLAGVRDTDPETIFDEIVRKKEIPATIVREDDTLLVFEDLKPAAPARVLVIPKDRSGLSCLSKAAPEHTEILGCLMVAAGEIAADKSLGFGDGAQIVIKDGKKAGQEVFHLHVTT

>1000

MRKPFAFNNLFINTKSKFYNLCKPMSKTLPDFTTSPVVMLGITYRSSPRVSAKGKSTTARRQPQPQPQRTQQAAKPQLYTFFNKLSVIPWTEEIQAEPSPPAASTEILPAHIRSFLTDFRSRMWLTYRSNFPAIGETNLVTDMGWGCMLRTGQMLLAQALITHYLGRDWRIQAEENMMTYRELLRWFADEPSSRSPYSIHAIARIGLRKFNKQIGDWFEPTTISEALRLLVTEHSPNGLKMYVPKDGIIYRKEVYQLCAVQPADGPAQHSPLRVDDDGGDTDHDGDTDGLESSTDSMRHSHGNPGVPSTIEAGDYSSSHAELMSSAESECESLDDNFTELTWHPVIILVPVRLGIQCLNPIYIPTLKAFFSFPQCLGVIGGKPHSSFYFVGYQDNKVLYMDPHFVQPTVKMDDDPLFPIESYRMEIPQAMSFDDIDPSLALGFLCSSQAEFDDFCLNAVAKTVYKGSSSSSSSSSSSSPSSSTSSPSTSRSGSDSCSVLLDEDDEEYVLV

>1001

MEYIHKYHYQPAELAARGELPPLEESPIWLLGHRYDPEVGGLSSAGVCDELRVGGRGRSTTHQHHHDSPLTTQASDQGEAQGQGDEEEGDCGPPTVFTMLHGSERRWASFLTHFRSVVWCTYRAAFPRLGSDSYTSDMGWGCMLRTGQMVLAQTLTRHLLGTEWRRQSDRSSPLYAKMVQWFADDPKQPFSLHRIAHAGLKYGKNVGEWFGPSTMAQVLEELLKEFSPSGLRAYVCQDGCLYLDQLRRTATAAHWPLDEDDDEGQGKSWAPMLIMLPLRLGLDQLNEDYAPVLKETFRIPQSVGISGGKPRASLYFVGNQDDYVFYLDPHTVQPAPRFPEVGDVPASEDVYDTFHCSAPLRLPIRDIDPSLCLAFYCRNREDFDDFCARAIQLSEGPMPIFTVAERMPDYLVRPKPPKHSEKLFSDDEDDVVFI

>1002

MADQSAPQPSPPNVTPSTEQLINSRESETPQVDVPRAADASAADTNASAAAPMLPSPATPPASTSPASTVAPVATSARPAVATQVQALWSNLSSSARSLLNQVENTIHDFLSATSLTPLPADVSAADEAVLLAKSFSLDPAGITALREELARRPRLTYRSQFSPLEPATFTSDQGWGCMIRVGQSLILEGMLRVRLGRGAVRDASNPDYLEIVQWFRDGPAAPFSIHAIVKKGMEMDTAIGEWFGPTIMAQAMKKNSAAAPSIPPIFVAQDAIVFASDMDDLFRNNQGGPILLLIPLRLGIDALHAAYWPFIKQSFRSPYSIGIAGGRPSSALYMVGTVRDSLLVLDPHMTQPAFHGTASELGTIHTDVVRCLSATSLDPSLCLGFAFASPADWTRWVAWIAKLRESGIEVPFSIQAATWVTDENLALSESDSESELP

>1003

MDWSTIPLALQSTTAGAGFSSWLYRVTHHRDFDAAALRSIGPGQLVLLGEHASLDRECALPPAKASPSTSCATTPTASTSTNAPLLLANARATQLPHPPPSSSRIPMPVSALPLVPPRPAPTSTSAATASSAYTMTTLLPRRAQWTMPSAPAAAPASAARPSNGLAPAPPPSLDHPATATATATATPLGFWRAPTSTAAPAAPALGASPERLECDMVARTSLPSRVTMPLPAPTTTGTSPVYVAASYGASATKWGSHVSAAPMPIYSTRPTAREPSTPLSPTSDYEFLRRGSDACGSYGSIRSAHFIDEDDVDDMPHPRSNHHDEDDEHDDEHHPHRPDQLDESHVLIDSIYRRNSAVADTALLDVSPSMLVVPPGGRDPHQDLCDDVLAATDDEADSDTPIHITHRDGRPPVTTYLVSPNGATNGHAAPAAHFTSRSRTTGIVQRLDTVIPSPSLPPPPRPPLSGPVVPIVPGVDPATAKVLRRFLDRWQALMWFTYRTGLEGDIKSDTSWGCVHRTGQMLLARALLVACFGRDLTIHSFQGAPITPRSGLPSPTTSTSPASSSPTSSSTPAPHSPPTARNGSHALPTPPSPLAHPDTYRAILSYFEDTRAAPFSLQNICRAQSIYLGERLGSWLSPGTIAHLLARLNAERRLRQHVAGAVGMPSRDPIDFVVHVAKDRVIVTDQLVLAAETAGTPDATVGGGGATPTSSSAPKGSGIVMPRVRRRSFGLGREKVDTASTVAEGAGSPVPPAAPWKPLVLLVPVRLGSEAFHDSYAQNVVDLFSWPQFVGIAGGKKDSSLYFPGVWVQPPGTSRASSTASSPSVDVRLAGTTRSTSPLHATAAGADTPGLSPGADLVRRTHLLYLDPHYPQPVVPAVATRQGGGSAPGTPTASGLGCPVPHPSYHTAQVRSLPMGRLDPSMLLGFVLTSRADLDDFVDRVSAWNAHGGGENPCGDAAVFGG

>1004

MADQSESQSSPPNATPSTEQLVSPETEAPQTVVPRTADASSPANTNASAAAPMLPSPTSPPASTSPAYPAAPVAEPARPAVATQVQALWSNLSSSARSLLNHVENTIHDFLSATSLTPLPADVSAPDEAVLLAKSFPLDPAGITALREELARRPRLTYRGQFSPLEPATFTSDQGWGCMIRVGQSLILEGMLRVRLGRGAVRDASNLDYLEIVQWFRDDPAAPFSIHAIVKKGMEMDTAIGEWFGPTIMAQAMKKISADAPSVPPIFVAQDAIVFASDMDDLFRNSQGGPVLLLIPLRLGIDALHVAYWPFIKQSFRSPYSIGIAGGRPSSALYMVGTVRDSLLVLDPHTTQPAFHGTASELGTIHTDVVRCLAATSLDPSLCLGFAFSSEADWTRWIAWVAKLRETGIEVPFSIQAATWVTDENLALSESDSESELP

>1005

MDWSTIPLALQSTTAGAGFSSWLYRVTHHRDFDAAALRSIGPGQLVLLGEHASLDRECALPPAKVSPSTSCATTPTASSSASAPSLPANTRTTLPQPQPSSSRIPMPVSAPPMVPPRPAPTSTSATAPTAYTMTTLLPRRAHWTMPSAPAAAPASAARPGNGLAPAPPPSLDHPAAATATPLSLWRAPAPAAAPSPVLGVSPERLEYDMVARTSLPSRVTMPLPAPTTTGTSPVHVAASYGASAAKWGSHVSAAPMPIYSARPPAREPSTPLSPTSDYEFLRRGSDACGSYGSIRSAHIIDEDDVGDMPRPRPNHHDEDDEDEDEDDHHPHHPDQLDESHVLIDSIYRRNSTVTDTALLDVSPSMLVVPPGGRDPHQDLCDDVLAATDDEADSDTPIHITHRDGRPPTTHLVSPNGAANGHAAPAAHYASRSRATGVVQRLDTVIPSPPPPPPPRPPLSGPVVPIVPGVDPATAKALRRFLDRWQALMWFTYRTGLEGDIKSDTSWGCVHRTGQMLLARALLVACFGRDLTIHSFQGTPSTPRSGLPSPTTSTSPASSSPTSSTTPAPHSPPTARNGSHALPTPPSPLAHPDTYRAILSYFEDTRAAPFSLQNICRAQSIYLGERLGSWLSPGTIAHLLARLNAERRLRQHVAGAVGMPSRDPVDFVVHVAKDRVVVTDQLVLAAETAGTPDATVGGGGATPMSTPAPKGSGSVMPRVRRRSFGLGREKVDPASTAGDGAGSPVSPAAPWKPLVLLVPVRLGSEAFHESYAQNVVDLFSWPQFVGIAGGKKDSSLYFPGVWVQPPGTSRASSAASSPSVDVRLAGTTRSTSPLYASATNADAAGLSPGADLVRRTHLLYLDPHYPQPVVPAVATRQGGGSAPGAPTASGLGCPVPHPSYHTAQVRSLPMARLDPSMLLGFVLTSRADLDDFVDRVSAWTAHGGGENHAAMPLFSVVEECAWPATPDEDDPRLRDLMDAGIGDDDDLAASVTAESKDEARPWWTTKSAAGGPSPPTPTQSPPAPAPVPVPAPAPLPGPDPNDLVLGSTPDQASQSLADGWMDAAASMMVAERAARTPPRYPAAHAAAAASMSDRTGTFSPWATIHMEPRSAAVAAAAAAAAAANARAFAMPPALAPASAGSSPTSTSNGIALPAPPSALSQVNGGAAHSPSPVAMAAAARVRDRTISDASLVSSPSAGTLSVRSVAGSFTGTPPMPPSVHGGAPRFGSPGGLVMAGVGGGSSRDGGSSDDDDEDENGHAARASKSGPGADADDEEDEEEVRGPRPGLPSATVGAVRGPVDAFGADESFGILHGWDED

>1006

MSAFAGGDVVLGAPCAAGAGSAAVAELLWFTYRCGFEELAPYGFTDDAGWGCMLRSAQMLLGNALTRNGAAPRLATAALFADAPGDSAPFGLHNFAKCGLRYDVLPGEWYGPGVACHVLRDLVDWRRNAPGGPALRVAVRTPESPLSVEGACDAMTQGRRDAAAEDPAPAPEDSAPLDVDGADPLLRPPPELAARERRRSESAAAAAAARRGRLDGAWDGALLVLLPLRLGLARLEPRYAEPLRAALRLPQSAGMLGGRPRANRIFNTTSMCASSDQNLQLCFENSTRAIDPSKSGRPRAALFFPGLAARDGGADVYGLDPHTVQPALAVGDDGALGPGAAASVAPRDAKKLAADALDPSLALAFYCADRDDFLDFVGRARALPGAPLFEVVDAAPRRGGACFDDDDDDDASGGGGGDDEDDWEVV

>1007

MLYIVRYAIKYVISLFARKHKVHASAKTLTLYSQEFENFWESLPYYTYNKHIPKSTVNSDEGWFSDAGWGCCVRSTQMAVARALIQLNGKVDGLFDDFMDAPLGIQRFYQIGTNWGPTSCARTIANLSNSEPSLNIPFLCFPDGIIVTEDIESAMSKHSRLVLLVSQRLGTDHFNTTHVGTLSSLFKCPEFSGLIGGDLWGRGYMFPAANETFLVGLDPHYIQNVKKDVRFQGKKPKILFWERLSPTITLVFAIKTDTLSNIMEFFRKISNLRYPPFELTQRKPQYCSTDADIITQF

>1008

MLRTSQMMFCQGLLSRFAAHITKIAAGTNSSGTSRAQGEAAEETKWKALVIVVPVRLGVEVVNKNYMNGLIKCLKLPQSLGFVGGRPRSSLYFVGFQGSRLLYLDPHTVQKVPPFQTDLRRVSSSFHCAKLRTMKLDELDPSLALGFYCRDRAEFFHFWSSIEVCVWGGGVDICLNNFLNREGERGVGVCMGE

>1009

MELMMLAKDIMQRKQNPITYPCYILNTRVDTEEELNSELSKIFLFTYRSGFDPLPKSSCRTDKGWGCLARSCQMMIGRMLAMHFRTDLRFAYFRDVDEPDAPFSIHNLVRGMLNQSLTFQPNFWSPTQGCEAIRYAVAAAVNRKLVKTPVSVLVAEGGTVSHRDVEFRLNEMGSVMLLIPVRVGIKRHINQQTFMALEHMMQTRLSLGVIGGVPRRSYYLVGTCGQRLLYLDPHSVKPAMIRAEDVSCDVEIAKTLPAVSWDRIDTSLLFGFFLKSHDDWIEFSAHVKKNTELCGIERLFYLDVGDGRRPDASDLLNNGQRAEDAIMTWSSSEEDDR

>1010

MSKKPQGPVAVPASSSSAAASSSPPVSSSYSGQLYSYLYNGAASVSNALSGETKLADEVPCYVNSLCYKGTRTTNWCSEAVRRCCPYFTYRKNFPCLLSNGGDHDAGWGCMIRTGQMMMCRALRKLYSCDVQVTVDPVEFAMFQKMTVDSSSSNSRQSSTLLSPPISPRTNGHGNALAAKTAPNFLLNLDDDFGGACGSSALENDASGSISQWVQEFFMDTPDALFGVYRLVAEGAKIGCAVGSWFSPTVLARSLAAIGKSHQELNEKLLIIPAVEQTVSQETILQTLLVEERSILLLVPLMLGMGSVGSSYEQVILRLLELPMCIGIVGGKPQKSLYFVGHQREQVFFLDPHVVQQAFTTRASVGKLGGPRGTTPVSTLDPNLLACFFFENEEVFLQWCDEMSVINKMGEFPIITIQSGNGNKGPSSLTVTGRSSPQAVDPCDFDDEDDDSQVAPDDDDLL

>1011

MAQFAHGISALDVAPSASLGAPVWLLGQRYDGVVAADFDAYKRNFESILWFTYRREYPAMTPYDHTSDAGWGCMLRSAQMLLGQALQRRLLDRNWRLPALFEAELDTRLPETYVQLLRWFADSPDVECRYSIHHMVKLGMQYDKLPGEWYGPTTAAQVLRDLVNVHRRDFGGEIAMYVPQEGVVYSDDVARLCVSHLDEESREMETGNDAVEFFDPLLHPPASDIKRKWSTALLILIPLRLGLDQLNERYVPAIQQTFTFPQSVGIIGGKKGHSVYFVGTQQDQLHLLDPHDVHSAPELSAAFPTATHLRTVHSSRPLVMNVNTIDPSLALGFLCENIADYEDFQQRVERLHEEVKADGGMCPFSVAEHRPDYAEGGDLLMADCLLSDDMNEDELTNAGPKENDEEDYVLL

>1012

MNQPNPNKQPIVQSTSEQTSNEEVDTVLGRFTLFVKDLSNGLNGSQEVPPSQESVSEEAEVISRKIIVLGQTFDNFDNANDYIESKLWLSYRCGFEPIPKSIDGPQPIQFFPSIIFNRSTIYSNFANLKSLFDKENFTSDAGWGCMIRTSQNLLANTLLKLYPKNEPEIVKLFQDDTSSPFSIHNFIRVASLSPLHVKPGEWFGPNAASLSIKRLASELLQDQEIDGIKIPRVFISENSDLFDDEIRDVFAKEKNASVLILFPIRLGIDKVNSYYYNSIFHLLASKYSCGIAGGKPSSSFYFLGYEDTDLIYFDPHLPQVVETPINMDSYHTTNYNRLNISLLDPSMMIGILVTNIDEYIDFKTSCLDINNKIVHFHPHTLPVQQDSIINQSWEEVQDEEEEFINLNVSKIENEQQQEQGQSTDAPDEFIDIGNQSSSVVSVPSNV

>1013

MSHNTTGPLVTVNGHKFQLPADEQALEHAVRSFPWMTYRNHFAQIADSYYNTDAGWGCMLRCGQMLLARAMTVQHLGKNWAPTSRKQRHQEMARFLPLFFDTPAAPFSIHRIAERGEALGKTIGQWFGPNTVAQVLKNLVNSQRSSLIVHCAMDGVLNRTEASTQLAAALSDGKKHSLLVLVPIRLGLNQSINPVYIPALKATLELPQCLGIIGGKPNAAHFFVGTVNENVLYLDPHVVQDAAMELTPDTVESFSVAVLSKMAISDVDPSMCAAYLCSSVAELEDLGKRSKQITSQFRGYGLFDVIEDSRINSLSLDSMAVDSDDGSDEDDFDMCN

>1014

MSDGSGVGSLNGSSSSSSSGGVGVREEHHHQQQHQSAHVAPPRLTLTTPASATARADSDAAAGGGGATAGGAPTTTTTEMDNVAQAAKPTLSLTHPHQHQHQQHSHDNDMLLLEATKAGRRLGQLRRQRTGSVGDAGSLLSVDSAAGTASSSSSHQDVEDEDEDDQDLYGDDDNYDEDFVLQSSLGDGHHEVQGGHEAQGQAQGQGRAAAAALNLSLEDKHAQASGKEEHAGNTHHHKAKHGQDGAATTVASNSTSSAAAAAANPAAAPAPRDSKHLQLPIEREASCFIHLDGAHSAPDIGAHGPGHPHTKWSDHSLAFYATDEAASVAADANARSHPETRPFAHGDVKGKMLAAFYNIRHNVNNFLSSISDSPIWMLGNCYSGKELECNGHTENKHNKRSRHICKFFADFQTLVCFSYRKDFERIPGSKHTTDCGWGCTLRSAQMLVAEALVLQIFGRRWRIEDRSCPAPLSSSKEDQLRLIIRLFQDQLRLDSPFSIHNIVQHGCQLFDKRAGDWFGPASVVRVFADLINQAYAMHQSPFRAYQAIDHIIYRDLVAELCSGPDAVRDLEFSTPTSTSESVSTDETVTPSASTSQSPPVLPPPFIPLLILMPLRLGLNEINRMYIPCLKALLMCAQCVGIIGGRPRHSLYFVGYQEDNVIFADPHGCKRFVDMQQTSFPTETFHSAVPNKIPFTHMDPSMAIGFLCQNQADFDDLCNFLTSLDKKCSFISVENTCPAYSRSTFVPAPPRLTIEELRVKLASAASTSSSSAPGKAPARRRASRPRSDLTADAGLASKHQPREDGPPKESSYDEDNEDGFGEGDDDRYDSDFVMCAAATSIPPQPQRQLSSSSSVSSTKKKPASGGFRKYFSTLRGSTSEPENEQQLQLQQQQHQQQLQGHEVGAAGASVPPQQPRSADPERKSPFSFLSLRHRHQTPTSEFAASSHSSLSDQPASASTSQPNSPPVIMQHHSSADHHHPLSHHHPVITGSLTADSLKALNRAAALQDATHPGSMSAFHSLVTPSTTE

>1015

MSNGASTGTLTYTQAFSLRVAQLAFGMSQALSLRKLSELMHALEAMLGDFRSRMWCTYRKDFPALGPSLLTSDVGWGCTLRSGQMLLAEVRHGWRAGAMMRVALGRDWQRCSDNLEAVRPVVAALLDCAEAPLSIHRICDAGGPAGIVPGRWLGPWMLCKGLEALFSQLAGAQQEPLGLRLHVACGAGGGAPELHMDLIRAAMAPAAHRSQVEEDVGVACKQPEGCGCAKQEVPVRGLPADLGLEAHDCGHPGHTGTGGDRQVTASGPDAAVAAPGSAGREIASDTAPEQESASSSTAASSCCGEAEDAAAHGRRCRGVAAGTCGRGHSLLLLVPLTLGMDKINPVYIPQLQQVLSWPQSVGIVGGRPSASLYVCGVQDASFIYLDPHEAQLALGTYFCDVVRVLPSAQLDPSLAIGFVCTSSAELEDLFARLQALATQHSSAPLMTLTTGSGAAVGCGSDADFTDDVLEGGTGQQQLDEWELV

>1016

MAGEAELSAVDKLSLGLSRSYYALARALRLNKLHDLLASGASITPDAPVWLLGQCYSCPPGASEAQQEEALARMLHHYQSIPWMSYRTGFTSIAAGSAHLQSDAGWGCTLRRQAAACCARTPRRTPAWRCSGTVLAC

>1017

MILAQGLVRHVLGREWRWPEAARQQQAAAAPALAAAPAEAPPRLARLLELFWDTPAERNPFSLHSLCRAGQACGVVAGRWLGPWVMCKTLAAAAGAARRQGVDLGLTVAVLAESGGGAPLLVTSRFEPAFGAGGPPSQGQDAAAQQGQQQEREQRQRQQGTDGSAAAAAELVPVGSGMLAVEPPSLAASVSQLTGGGGGGRGLVLLVPLVLGLGKLNPRYIPQLEAVLAMPQSIGIVGGRPSSSLYFVGFQHLHQHQHAAAPAPAAAAAAPAAGAPAAVGAAPAEAAAAPLGAAPAAAPAAGPAAAGPAAVAAGGAAGREAGAGSAAATSSVIYLDPHQVQEAAACPDDWRTFWCETPRSMPLPSIDPSLALGFYCSSLGEYRDLCSRLEALERRSGGAPLVCVATQAAAARYASPHETEWEPDELSSEGEEEEEGGAGSAEEACQEEPVVLQPDVVAEAEAAVPSPPPAAARGAASAGPASPAAAAAPPAPPRSGPGSLQPAQQQQGLSPQQQQAGQQQGGAPGMSPRQMSRGWELL

>1018

MAQVLGLRRLQEYFQVLNAILSDLMSRIWMTYRRGFPPICGSGITSDVGWGCTLRSGQMLLAQALVYHLVGRQWRRKLEAAYPEEVAQVLQWFGDQACEQRPFSIHNMCTTGQTHGVKAGDWLGPSGLCHTLADMVNKVQPGGLQCRVVATFGGGAPVLCTSRLATAFEGGADRSGGEVGSSGSEESGPAGQGLLLLIPLMLGLNGKINPRYCAQLQQLLTWPQSVGIVGGRPSSSLYFIGLQDQHVLYLDPHEVQEVASEAADLDTYFCSSLRLMPLANIDPSLAIGFYCSSLSDFEDLCGRLRTLEAEAGCAPLVCMVDEDAGEPSWPAEEVLSDEGIPSDADSPAPPAGGANRDNWEML

>1019

MNFGRTIYFSISSFFREMTQKFLSAEEEIFMLKNKFAPEEKKYFLKEFHDIILFTYRNEFKNIIITRNTVQLTKNYSKNINSDVGWGCMYRVTQMSIAHGICQFMKRFLGNLNIEKILNNFQDNESAKFSIHNMVNIGLSEFGIDPTSWIGPTTSSMIANKLINDNRSIISNIQIASITYVEGTIYRDQAVKHFSEVGSDSCTFVWLCMKLGTSKFNINSYKKTVISMSNVSQFICIMGGNNYSSGALLIVAFSNSFLYCLDPHIKVLPSFSDKNFIRDDFIQKVPTRIYWGELNSSLSMVYICRNLEDFDDLCSNLTRINSDLFEVINNCDFEVKSINELDSGFLVV

>1020

MNCLYLIRGAAGNTLAQPQFSNSPIWMFDNEYRPSLAENLLKLNRLITTINNINSNIGENSSNIDPTTTTTTSTADNLEIKNEANDDIVGNENVNNNNHNNPNSNNIDPSPTITPTSINSQQENNNNNNNNNNNQNQLFIKEFLNDFTTRVLWFTYRQGFPCIDDTMYDNDCGWGCMLRSGQMLLSNVLLHNILGDEWKRSSSATHPDIISMFLDKPSAPFSIHNIAMEGQNLGKNIGEWFAPSIISQTIKILVSRNYDQCNISVFISEDGSLYIDQLLNISSKKKKTSSTEQQQKQQQIESSTDNGDDKINDEKHNENVDKNNIDDNNNNNNNNNNNNNNNNNNNNNIINDSSNVNEKQDKIDDESKDEISENNNKDNDETWEPLLILIPMRLGLDGLNSIYHSSLLEIFKFPQNLGVVGGKPRASLYFIAAQDDNLFYLDPHTVQNHIEVENGSKFPLNTFFCSTTKRTHVSEVDPSLVVAFFCKTKDDFNDFVERSKKMTSQMENPIFSIFDNEPDYDSSRDYEYEEIDETGGETSDDIDIEADYHMM

>1021

MFTKYSHHNGYQDGSHLQPFQYQQQTYNQQSYLRQQQQAPQQISYGFNQPNSPTSSSSTPSSSTAMGNSFQNQRQINLQQQQQQEQFLQEQVFYQQQLLQQQSQIKEQQRQKEQKQKTNILNIYKEGKQKIMMSFYNLYRNYPTEPPHFSPSPIWLMGRCYTSKDNNSNNNSNNNQVPQTQPTQLQQSIGIFQNNNSNSNNNNNHNNNHNNNNNNLTTDLIYRPAIESGFLSDVASMIWFSYRKDFPPIENTNITTDIGWGCMLRTGQMILARALIKHLYKENDMVPEIERKKPHSNYSQVLAWFSDYPSKEHVYGIHQIVNKKQAMEKNNRKQQILREQVISLNRGGGGSSKGKKKKEKEEEINDNVEEWLAPTRISNILRQLIKFQHLEDLEMYVPTDGVIYKDYINNLCNNSNTHNHYQIIQQQLQHLREQQNIQQNNNKNNNNNNPTTTTTTTTTATSSNNNNNQSPPSRVPNGYNNQVFDDESLFDYNTAISSIPPKWKSLIIMIPLKLGADKLNSTYIEKLKLLLKLPQSLGFIGGKPKQSFYFIGFQDDQVIYLDPHFVQESVNPNSFDYSNTYSGCIPQKMPFTQLDPSLSIGFYCRDQASFEDLCDRLSVINNCEFPIISVCQKLPDYQIECELVDDYAESETTEMLAITIANGGNNHSCIPENIVVDDEEFIVHHHIPYNPNNNQNNNQNNNNNNNKNNNNNTNQQQTPNYPPKLNTYQPDFSSDGEIDDFTMVG

>1022

MRGRKQRQRTHPRQLMIRARAAAFFDDDENDGAPAPTWIMGIPYTELREEERRLDVFSTMWITYRSGFPKMEPYGYTDDSGWGCMLRSAQMLMTQALQRHTLGRSWRVPRTLEERLRVPEYRTLVRLFADHPGEANLFSIHNMCQVGIRYDKLPGEWYGPTTAACVLRDISEIYAERLSPPASPPEPPLSSTGRLRPAEVSAAPDEPAAAGDACRGEATSDTSLEGGENVVEGGSERESGASPAANGGASGCGGGDSDACGSPSGGNGDGAVFPSSRPLRVFVSQGDVVYIDEVEAVAIRGASDTATTATTANGHGAVSGGEHREEANGSATTGDGSSSARSDAAADSTDSPAPAFFDPLLNPGSGEGENPLEKAWSSAVVLLVPLRLGLDELSTGYIPSLLETLRVPQSLGFLGGRPNHAIFFIGAQGNTLTGLDPHTTQPAADMGEGFPSERYVHSLHCQSAVSMDVHRIDPSLALAFYLPDRATFEDLIKRIGETNPPPFSVEQTRPDYEGEMGLAFMINQRDDDGTTDDDDDEYVFVKGPER

>1023

MLESSTVVWLRSAAREASYRLARFLPESSDAVQPDSPIHILGTVVEASAEATAEVQACVRACLWFTYRQHFEPIPGTVFTSDAGWGCMMRSGQMILAQALLRLSAGGGGAGASLERREAATVALFADCLAAPYSLHRITLEGQAQGLPVGRWMGPASIAQVLVRLADRDAPSCGRRGGRGGGRGGGGRGCRRRSGGAVRSGRGERLLYLDPHEVQPALRPDDPDVASCHYGRGLRTVRVGDIDPSLAFGFLCGSADEFDELCAGCARVGSSSLSAFSIAEARVLCYERAGSEAEEAEPLSSDDDDDLVVL

>1024

MLESSTVVWLRSAAREASYRLARFLPESSDAVQPDSPIHILGTVVEASAEATAEVQACVRACLWFTYRQHFEPIPGTAATVALFADCLAAPYSLHRITLEGQAQGLPVGRWMGPASIAQVLVVAAASGGGGGWRPLLLLVPLRLGLDRLNAEYVPTILGLLRLPQCVGVIGGRPRSSYYFIGSQGEPARRLLYLDPHEVQPALRPDDPDVASCHYGRGLRSVRVGDIDPSLAFGFLCGSADEFDELCAGCARVGSSSLSAFSIAEARVLCYERAGSEAEEAEPLSSDDDDDLVVL

>1025

MSMLRCIGYNIGSYFYNSVSSKRLIKLQPFTQKNVVHILGNCYYPETNENLNHLTFNDANIKIHDLIVATYRQKYSYLGNTYLSSDAGWGCAIRATQMMIVNALVIFKDQMQQIVDYNSFEHQQNKSQAKELIYDRISSLLSIHNIYIQQVIKTHNPKGTNFLPPSVCCIAISSLLQEWDKKPFNCITCLNHIPSCSCPTLYLIPRIITFTEHQLILDSLALSQSRGFVGGIGESAIFVFGCQGTTLFFLDPHYVQNAGDFGYFNPPTYQIDISLISSSVVFAFMCYEENECEQLRTIFGMGKVPIVAREFTSTKEIDGVEVLDF

>1026

MDDEQNIYKESTQNIPFLQWLNQKKSNVFSQLYNYIPNNYTSPQLYPNEFIIFLGELFPPHIVRTETSNFFDVAITHIKAYPALTSLIYFVYRSNFSALPNTSLTSDGGWGCTIRACQMLLANAIIKLFGSDNINRKTVIHWFLDFYNSECPYSIHSLFTTQIIVSGNPNGSSFLPFSSVIYALTELVNKDFNRAFECHVITNKFLLKSINKPTIVFIPFTIPDKFDQRLITIFSFNLFAGMVGGSKQKAFYFFGIHHNQLLFLDPHFVRPCASSIMKFDEKDYIAKLSDIKSLRINELERSVVFSFVIHSFQELISLQELAKNVLGIDDKQLTIKREECDGFEVLEF

>1027

MSYFQKLVQHGSYNILSKFYNQIGHEDEQKPIFVGGCSFYILGVEFKTKQMDKQLAEQPPEVYLQYSSAATFFRISNLFWMTYRSGYEKLPNSSLTTDVGWGCTIRAMQMMIANAMETIVYSGALNNTQTPYIPTKQEVMNVLIPFIDSPNSTTPLSIHHVYESRFVVEKNKSGVNYLAPSVVAKAYSGLVNSWKLCPIRCVMCSNVSIPTHELSKLPFKPTLVFLPIVLNHLIHSKLQQIYKSKLFAGIVGGMGDRAIFVFGFHALQFLYLDPHIVQPSFKSFTEIDTKSYSPIGTNRFSVHTIDPTKLDDFCTFGFLIKNLHEVDDFMKLAKDVFEISNDKELRTSHDLNGFEVLDF

>1028

MEVGSLNVIEAIALGENETEVPKKRENKKDIDDFVKHTIWITYRKNMPLIKEKTTDSGWGCMIRSLQMALAQTFLSIVLGNNWKYEDNCINTERNIFHIKSIINLFGDSTGSLFSIHRLVARASTRGVTEGQWWGPSFASDIAAEHINEMRVFRTRGYVAKLGSIIGSKIEELIKDGGGFNPCIIFVPLRLGPESPENEFKPLLKTIFDIPQCMGMIGGKPGYAHYFHTFDGTNLYFLDPHTTQNAIDMKGDWSYQSYFCKDNKSMLYSKMDPSISLVFLVKHANDYEHFKKSFENKTFSKLFTFKDETEKELNSDDFISLDDDDIDLIK

>1029

MLPPQQKKKKSFTSFVNACSYNLSSHLYNSVVKSQFKPFTFNLKYVLQGVTYTEQPTSNNNIAKHLSTLFRITYRNGFTYHLPHCSLTTDAGWGCTLRSIQMLFLNSLIRLQEPNPGFGDDAAEKVQQNFIIHSMEERREYVQLIEDTPKQEAVLSLYKMFNLKIVRQNNQKGTNYLSPSTCAIALSQLVEMWDQRPCHVIYSNTFPKEIQPNTLLMISAPLNEKTISCLDNTFVGGVVCGVDTKAIYVCGRTGNMLLLLDPHFVQKAHEDGDFDIVDYSVKPSDLRMVRLTELVFGNCIWGILVREDNIEILKNWCKTSLGVTNEEEIREMVSVGNEEGFEVLDF

>1030

MADAASGVGPAPAPGPRLSQVTALNFDDASSSAAVGNAFGFKVFRCNPFAKCYEMHGPNTALLAVSLPAALLVWVEAPPAACVTSQMAGPCRRSWGHTVHLHSLTSSRRLQTLRFPSRVIVVHLVGELLLVACDRELYVYCTRQFPLYQLHVFHTLPNPRGLLAHTLHQSTVSIAYPSNLVGDACVAVAEITQTETPAFPFGLRLRTTCALPLPAHQVTLMHLSRDATLLVVALGSAKGIQLTDTRNPQRSASVVWRGLGMAQEVVAMGLSPDNALLCVAAGSGTVSVHDVSKALQAPGEEDEEAPSFFSVSVLNSSMEFVTQLKEFVHPRAFASFDIPKRHGHPMECTASRPLCAWLDATTLAIVAGDGRLFRLEVRAGECRVREAYGLTDDHVWLLGLRYDLWLLPAEGPYSCWDDGRALMARLHSLLCFTYRADFLPIGKYKTSDAAWGCMLRTGQMILAQAFRVHHHVDVLTGPKLPPGEAARRDAGIAAWFCDLPGSPYSVHRIAQAGERRGKPVGDWFSPTLIATVLQELVETNHSLHGDRFCVYVADTATLYLNQIEELFAADPAPALLLLVPVMLGASPLANPLYFPALCRLFHLPEFLGVVGGKPGASYYFIGYQVDTSCRLVYLDPHAAVHPALTNITAQSRETLRQTPLLTMPIARLDPSLVLGFYCRDRDSLQSLVARTEEVQEQCGATMFTWQAASECGDRSFDLKACPSNMDVEWRMVQEKGVEGSPQSISDRGYSEFCNDEEWEEVGPKAEVEDPGQ

>1031

MAALRFCSPTALAEEAAVFCPLDVGFEPTFGWVLGEQYMLRSAAGEELSEQAQDMRERVGALFRFTYRRGFVPIPGSTPLITSDAGWGCMLRTGQMMLAQAFRLHYCTGPIEPGLPESILDETAYTIASWFCDRPGSPYSVHNIAMFGVRMGKPVGSWFGPTTTALVLQELVGLHSSLLHGDHLRVCVAEHATLYRDQVEGATEEEWRSLILLVPIMLGASTFANPVYLKSLLQCFHLPGFLGVVGGRPQSSLYFVGYVGDDHHARLLYLDPHLAVQPALLRVEMGSPLNGLGSLREERIDMMDPGLLDPSLLLGFYCRTRRDFEMLAAVTKAMFEDTEAPLFCWQDHNTMAQAVEDGLVDAFSSDSEEEFHLV

>1032

MLCRGSSCMDGVSYLWAYALSATLTCYYRARAAAAELLYADGIDALAAAPEVWILGQRFAPPGPEGDAEVYRSALLGALRSVPWFTYRSQFAAIPTSHLTCDSGWGCMVRTGQMLLAATLLRHCLGRSWRHDGEDAHPLYSEVMAAFCDVPAAPFSIHRICLAGQEHGALPVGRWFTPSLIAHVLQRLTHANRLALPGGPMCVYLGQHTTVYTTEVEALCCGAHSAADSVHSSAVRPSPQAGEELKGRSEAPAEAPAPPPPGG

>1033

PGGAPVEPGGWCPVLLLVPMLLGMREVNEAYYPLLLQCCQFPQFVGIVGGRARYSLYFYGTSEGALLYLDPHGDVQAAMVAVDDAPGRLHTTAVRRLALPDLDPSMAIGFYCRTRAEFADFCQRTKALNAGSPTPMFAIGDHLDVSACGGGGPEDLEQAAALSVLTRVHQSLEASTGSDGAIGPSAAAAS

>1034

MNLFGSNSTKRFDQNETELDDIRVLSFVSREREPGFDDCPEYSYDSDNENTSQNDCHETHGSDDAHGSLQAYILGKKYHPVHDYTLRREDEISLFWFTYRCDFPEIKPYRIQSDAGWGCMLRSAQMMLCQVLRLHYKSRTWRPPKSLAARRQDPFVSRLMTWFADFPSTTECFYSFQNMVAAGLNYDKLPGEWYGPGTVSFVIRDLVAIHDQQRQELDKTLFRVHVASQGTVYRDEVRRLMIQGANTAAALTKGGTASRPGAAHPLDHAAWEEDHLEKVPDWDTALLLLIPLRLGLKTFNLDYVRALAHTFSLPQSVGVLGGRPRGARYFYGAFSDGSKIFGLDPHTVQAAPQRASVSINGELSSMIDLSDENLRSIHTAYPESISLEKMDPSITLGFYCENERDLQFIFESVQQWKTENPGSPELFTTANYSPDYAANISSTMKKIMLSSSSLLDTEEDQISDEDDYVML

>1035

MTTASISCAIDRNDASIPADTYCSRKDEQRLQQTAEDRDATEYRNPLMNFFGSNNTQRLDHDEIELDDIRVLSFVSREREPEFDDCPEYSYDSDNENTSQDDYHVTHGSDEADEVKSLQTYILGKKYHPVNDYTLRREDEISLFWFTYRCDFPEMKPYRIQSDAGWGCMLRSAQMMVCQVLRLHYKSRTWRPPKSLAARRQDPFVRRLMTWFADFPSTTECFYSFQNMVAAGLNYDKLPGEWYGPGTVSFVIRDLVAMHDQQRQEFGKPLFRVHVASQGTVYRDEIRRLMIQGAVTAVLTSGQSASRPRAAHPLDHAAWEEDRLEKVPDWDTALLLLIPLRLGLKTFNLDYVRPLAHTFSLPQSVGVLGGRPRGARYFYGAFSDGSKIFGLDPHTVQAAPQRASVSINGELSSVIDLSDNYLRSIHTADPESISLEKMDPSITLGFYCENERDLQFVFESVQQWKRTHPGSPELFTTANYSPDYAANVSSTMKKIMLSSSSLIDTEDDQISDEDDYVML

>1036

MYGDRADEGLLEKIARVGRAIPGFLCNLPVRVVLRRALGALYALGRSVKSILTMEALSDVLLEHKLPHTVWLLGVRYTLAPPPMGQRGEGRETEQTVVDESQNFKLDMWSRLWFSYRYNFHPISGTELTTDTGWGCMIRSGQMLIGQALVHHHLGRDWRLSHTSKYNELPSDYRKVLEMFLDHPCAPLSIHSFVRAGQQVGKKAGTWFGPNTVCSAFSKLHAGGALGSDNNLQLLAYDGNDGDNTIYKSEALELLQAGPLFILLPTRLGVSSVDPSYIPKISHVFSFPQSLGFIGGKPSSAHYFIASQGEAVYYLDPHTPQPLINISEKESV

>1037

MAAGFVESFPSPNSRVMGSNCAPLPKFSNHETPHIPPLPVDEYQSYMHLSPDSRKGIIKQGWLHKLSYHGRGWKKRYVVITEDCELQYWSHVFPFNGGTAQWELKGIVSLDDAEFGKVQNLVVDGHEGRFPVAIKISSQRSVSPKHSQWWILRDYYFAADENELEPWMREMADVKFKCEVELVDRAYCLTSSVDASDSPRSKLDSEYMFVEPRILLPLSRQLVLNTHNNMANATQLDKRQFLDGSCAPAWVLGWKYKVGPDSPRGNATNRGTTDQQTVHISIWFFSNRTEQSYFRQRLGMHAAQWPNDAGTSIATSSLEERLAIAKGQAHSSLVIVLMLHGAISQPLCCRWFGPDTICRVLRHIWNMNEGVWPCHTAGMLFVEDHCIYRDLAESVACSRQAYSGTNCSRMAQAREPCSWRPLIVVVPVRLGARSEDQHLSRIDKHLQSLGFIGGRPRHSYYFVGVRGYNAYYLDPHITQPYQSIRKNINVASFHCAHPGKMSLAHIDPSLALGFYCDDKSDFEDLIRRVEELAAGDSHPILSVGNRAPDYLSLDDVDEDIVTFEDVN

>1038

MFLLNIENLMSRFHCLYLNIKVGYQGSYSFQHLQIQPQLEKQQQQNQQHQQQQQIIQQQKQQHQQQFKQFGQLQQQQQNGQQLQQQQNQKNEKKKNFFDFYKSGKQKIMISFYNIYRNYPSNPPSFSANSPLWLMGKSYNTHNGASKSTALIVTSGFSSCSSENMIYQQNPYQFEKQHHQQQQQQQSTSLNINSLFSSIFQSSNNSNSSSSSSSNSSSSNSSSSSSSSQQLPTPKCNNTMTPLHHQSQIITPTHYSPNNNNTINNREEANQEIDRFIADFKNILWFSYRKDFAPIENTNITTDIGWGCMVRTGQMLLARALLRHLYQNENIPEVDRTRPSSKYRKVMNWFCDLPTREHYYSIHQIVHKNKIIAKYHNSKLKDFDIETDENIDLLNVDEWFAPTKISVVLKHLLKSHGLSDITMYVPSDGVVYKDYVRKLCTDERLSFDPESSGINSGCNSYIIDGNAARVPSMQSYQYKSASSFGQNSPSNPAFSSFVPSKSPPSSMEEENYLVGENSGSFKDPLTCSDFFSSSCIPQRWKSIIILVPIKLGLDKLNEVYFREIKSMLELPQSIGLIGGKPKQSFYFVGYQDEHIIYLDPHFVHDTVSPNDINFSDSYHHCVPQKMLISQLDPSMAIGFYCHTQSDFEDFCVRIKEIEKRGFPVVSVGEQCPDYQVESDDVDLDEFEEECENSFINNLQGQVSDFIGANNNSNSNNNTKFEDSEDDLDGFTMVN

>1039

MNNNSINSNDLNSSSSNTSSEYQQQQQQLQQQQQSPFRYINNSNSISSNNLYNKSIFSNSNNNNSNGNNNNGSNSIYNNNNNINNNNNNNNSINDHLHDMKRSKSTPFLNLQQMQYIQQQQQQQLMQSSSSSSLLSPNSSSLNTNYPYTTSSLYKSNLYNSTNTTNNNNDRYNNNNDIVNRLENQIQQILNEVEELKKKIKIMEEQQGNTNNWGLKYSRRVLITSNFLLETSITSCDGTVFECHGGTQSYFVATAGGDLQCSEKESIIPNFIMNCLYIFRGAGNVLPQFSDSSIWMYGKEYKPPDPNDQKSNIDSSISSDSSNVSADGHRQQQQQHQQINDGVSKLVIRDEEDSINHSSSIMSGSFELTNIIDSSSTGNNNTTTTTTTTTNNNSNNNNNNNNNRQPSFINEFLEDFSNKIWMSYRQGFPYIGDTMFENDCGWGYWKKSGQNEYPELLYNIVRMFLDKPTAPFSIHNIALHGQNHLGKNVGEWFAPSNITHAIKSLVNKFNLQCNISVVISEDGSLYVDQMLDAALQPNGSIVGGKPRASLYFIAAQDDNLFYLDPHTVQQAIDNEVEFSLSVSVETKEDFLDFLERSKKLVSKSEFPLYNIAERVPDYQLQKTMSVEGEEEDSSDDIDFEMEYHML

>1040

MGLYDKSRLNDVSIRFKSSYKKLYCQKKIRVYKYYKLQSYFLFFLFLNFIGFLDYDDGLNKNISFFSIQKISALAYFYFDLKPQNWYDPNRICFILEKLYNFSSIKGTENLKFKYFSNHKLIFFEDLIKLMVDSQAKLCNQNIHNEQQQNLDLNNNSSQLIEDSFEVITKSSKQNTLDNLICKKCHQSDKSLLIFISCLTNTNKISNKKQQEVVISLLKNQFSIGMIGGVPGKAYYFLGIIDNDFIYLDPHYIQEAHQNEKTVQNIDTYFCKFINRVSQKKLESSLAFGFYIKNLQELEQFYQDIKNLEYFYKDDFFQVYKKKKKYQNKISVEFEIDQFGFQNVNNYDENQNYDSDQQDEQQQDIDYQEILNQLYLKFKQELKI

>1041

KVFFQGNQLSPTEDYEKNCKKLIENFKNIIWMTYRRNFFPLLHNTKDHKIQNYISDTGWGCMVRVGQMALAEGLRHHLQQKGIYDNKRIIQAFLDNDFGDDNIAPYSIQKICKIAYKEFQLVPGQWYSPVRICHVLSLLHNDKKQILDCEDLKVGVFSTDRPIIIADMIKKINPGVQNACEETNCQFIKNQKDQKIVCQQHNQSIFSLL

>1042

MFESIIWITYRRKFPPLKAPQYEYISDTGWGCMIRVGQMALAEGLKRFQIKEDEIIDLFQDKKDSLFSIQNICEAGKEEFKLEAGDWFNPIRICYILQILNEKKGFKDLKIRTISSDRILIFEDLEMEFSSEKNGLILFLVCKLGLEKTEENYLKIALKIFDYKNSIGMIGGKPKKALFFVGRIEDQLIYLDPHYVQDFNQNNVDQNSYFCKNYAVLDQKKIDSSIGNVLFFENKEELKMFFQFLDQLKEEFVQDFFLAVEKVKPDYLKQVEYENELYSSDFEVL

>1043

MLRYVQDLWSAFFPTEAVFPLQVVGRSGAAVESREELEKALTDTFLIFTYRDGFEAIPAVTRLIETDQGWGCLLRTSQMLLAHFLWVHGRPADRRLSLFFDHSAETAPFSIHNMIRSLWNRRAFKAEYWSPSQGCEAIKRTVQGAVKTEQLQTRVMVVTSANGCIYADEVQHTFKQGADVVLVLASVRVSAAAQLTQESYLQIEKLMEQPQCLGVVGGVPGRSYYFFAHNQTQLFYLDPHQRTAAALLSEGPSAAVSVTPSVADVRCVHWSRVDTSLFLAFAVTTRDEWAALEVHLSNRFMHVESQRTQRDCDQLSRARPGHADGTFLSPLQTGDNCGMGGTSISEVAPLAHRRTLRVPRKRFKEGEVGTATAAKAERNDGEDEVDTDSWEYLD

>1044

MSTNAKMAEKTPCVDLGSLFKWWLNPSKIFRTPNRKMKNTTPVVVVGSGSHSGDGTTEFVKAATKKLLYFSYRNCFPPLPNGSTTDTRWGCLVRTTQMLVGTCLLRYHCQGAYVLPEADNAELKERISRLFMDVPSAPLGIHKAEDEAHKNSVKYASMLSPTEAGMAIAAALIAFHAQGGDVPFTFCCESRNIDEPAVMAKLSEGQHVILIIPVVLGIAPMSDQYERMMLKILDMKACCGIAGGLKRASLYMFGHQGRSVFFMDPHYIQNAYTSDRTVGTLEGARGELSARRFDPCMVLGFYLHTLEDYRVFAEELAVANSLVAFPLISFGQRPREGTTPSDNGVVSVAESEEGIMPHENEKSQLSPNPLAAGGGHARSSNPISPLPS

>1045

MLRYVQDLWSAFFPTEAVFPLQVVGRSGAAVESREELEKALTDTFLIFTYRDGFEAIPAVTRLIETDQGWGCLLRTSQMLLAHFLWVHGRPADRKLSLFFDHSAETAPFSIHNMIRSVWNRRVFKAEYWSPSQGCEAIKRTVQGAVKTEQLQTRVMVVTSTNGCIYADEVQHTFKQGADVVLVLASVRVSAAAQLTQESYLQIEKLMEQPQCLGVVGGVPGRSYYFFAHNQTQLFYLDPHQRTAAALLCEGLSAAASVTPSVADVRCVHWSRVDTSLFLAFAVTTRDEWAALEVHLSNRFMHVESQQTQRDCDHLGGARPGHADSTFVSPLQMGHSCAMGGASISETAPLVHRRTLRLPRKRFKEGGVGTATAAAAERDDGEDELDTDSWEYLD

>1046

MGTNAKVAEKTPCVDSGSLFKWWLNPSKIFRTPNRKLKNTTPVVVVGSGSHSGDGTTEFVKVATKKLLYFSYRNCFPPLPSGSTTDTHWGCLVRTTQMLVGTCLLRYHCKGAYVLPEADNAELKERISRLFMDVPSAPLGIHKAEDEAHKNSVKYASMLSPTEAGMAIAAALIAFRAQGGDVPFTFCCESRHIDEPAVMAKLLEGQHVVLIIPVVLGIAPMSDQYELVMLKILDVKACCGIAGGFKQASLYMFGHQGRSVFFMDPHYVQNAYTSSRTVGTLEGSRGELRARRFDPCMVLGFYLHTPEDYRVFAEELAVANSLVVFPLISFGRRPREGTTLSEDRVVSVAESAESITLHEKEKPQRSRNPLAADGEHARSSNSISSPPS

>1047

MIFLDPRYPYLRFTRSHHRSSDTGILCRFMFGPAPFYGQRGPRRHLLAPACVMSVSRGLAHITDMLRGAIRSGGSGGDGDAAIWMSGARFAPASASPLPSPSTPPPASVPPVPASASASAPVPVPVSVSPRPLWGSEGAPPPYDEIVHIQSVLQFSRFQSAMRRTLWFTYRRDFEQIGNTGQTSDAGWGCMLRSAQMLFAQAFVAHLLGNDWKWSDRDPGPEKCLVLKEVVRMFGDSPRYPYSIHNLARVAESLGTQCCDWYGPATAAYALRSAHSKLPAYIPVHPTLAKEMGGFEVLVSQDGTVAADEAEAATRGWSAGLVLLVSLRLGAERMNPVYVPALASVFRFRQCLGFMGGRPRSSFYFVAAQGSSLYYLDPHTVQPAISHAYSSDYDISSYFTTAVKKMHAVDLDPSLTIGYYFPDRASFQAFRAQLAEMPRTEVELFVVTERSEHTSPQRPRLKQPQQPQQTQQQIERPKPVAAAHVAAAAPGGVIALDDSEFNTL*

>1048

MLVAEAIQRHLLGRNFSFNPNFFPFCPRCVWLASLFSDEMKPGTRYQHPFSIQHLMELGENYGKAQGQWFAPGTIANVYKSIITRHVGPQLGMAVYIPPAGFNTIYISDVLRLLHSPLQQPEKFPDSRFTTPSCPSCPIIGCMCSIQSREEKHRKMEEEKKKKILQKLSEKEKQETNKKESEPSSSDPSDSRIDCQKLKFPELSPSQAASNTTTPSSVTLSPRPSEQEGSKEVNEKQETKQETEISDSSKTEMENEIKNENTTESDVSSELSSPQQSPSQQQSPSSTPTSFISSHSASSEKLSSSAPYSFGSVSRSSSLPTSSGPMAASASVSSAASTTESPSRAVLILFPTRLGLTKIPLHYTRILSALFQLPSFCGIAGGHPSSAHFFFGCVCEKEKDDKRERQRKRGTESVLYLDPHVTREAEEVKMIRVEHDEFWEKEREKENKIKNFDAVSPIITSAPSPSSPSSLSSTSPSSASLPTSPLSKSMSPSSPSDPSCNSSSPNSPSANIQSTSSSSTSSTSSTASSPSSGNTAYPHSSSKCLAANSFHPPSPSAIHSMPIKAMDPSMCLGFLIKSDEELFELKKQVEFIFTVQSAIRKKEARERREKRRMAKKLEREKRREKEKEKGQGQVEESQEADDESLENDSVTDESLDPDEDIPPFIFFEGKESADDSFEQRIKVKEGKRPNKNKVGGADQEKCCKRKAPSLSEGLSQDIQGQLIDSVPSDEEMSDDGDDSQKRSRARLDGKNAENEGAIRSGAEGDMSHASELGGDDSKDSYHFEITGQPSSFAAAPYLDGCEDDEDDDAGEDSFDIIELT*

>1049

MPKKSIEDDIWIMGIRYPRASKSSKDNSKETISSFSQEKFEDNIRSKILFTYRRGYQSIGTTKLTSDRGWGCLHRTTQMMFAEALFRLESDRVPNKNYLDISSIIEQFLDNPTAPFSIHNISQTGIKLGKKVGSWFAPSDGAHALQTISQQMSNQRFPIVIVCVDTTVIVEDIQRRLSEANQRNVEKSILRVSHFPSAQKNTSTSQKRQSTSDIASTAKFPKGNQKQNYSSRHRFRELADGQGALVLHARSAPAPVESTFRFIEKTGSIFTEVDALWSNETEKMTTVLDISMENGVEAKKGETSSSSSSKDGIPQINESEMQQPLVSNKTSQNSVGSKQLRFYLPSMTHIVSAESSSPPLPLPLPLPIPVSFQTSLSEDTSHKCSSLSSFSLSSSSPSSFSSPSSSLSHSPSFSPTKPEFNQTPHSSQDKSNIQTSSTSKEQLSQEISNLSSNLSDSSLKHHQNSIDSLIAEAMKPIIATEEEYGFLSKPIVTSSAASSVEQSNDYSSFNSSSSSSSFSSSSSSSFSSSSSEALSPHSFSSSSSSSSSPSPHSYSYSPSSSSPSSPSPSPFPSSSSSSSSPSTSPSPSFHSPNLSSSHSSPHPTSSNHNRSNSNTGKSFLSKLLSHPSSYPSLSYFLPPLSLNSSQYSVTSVENTKGRGKREEEEKEKEEEKEEEKVNQKEEVREMNSNRASSSSSSSSFSLTEKISNNDSSNISSFSSSSSSSSSSSSDSSPTIHFNSNTLHFSSLKGTNSSQTDSSSLNYHSSDMEKQQNTTKELSFCEEINSSSSSSSSSSSSFDSSSSSSDSVKEIDNAASTASLTHSLPLPLPLPLPSSSSSSSSSEIAITASSSSSSSSSSSSSSSSSSSSLSSHSPSFTSIPLQNSTIMTNSIDIHQTHSIYHTEKGEGEREREENREEREQGKGERKGEKEETSEEQKSSTATSANDKISSIQQEKDHKLQNNSQNIEKTHLQTTNNEGKKNEKGKEIKSDIFNENSSEAASDDFSLKKAVQTTPGDSGSVYSFSSISSSSSSSSSSLSSHSPSPSPSPSVSSSPATASVSSSATSDLSSFDPPLPLNTESSSSSSITSSISSSSSSSSSPSLPSTSPHQTLHSNTQLPSSFASSSSSSNSSSSSSSSSSSSSSSSSSSSSSSSSSSSSSSSSSSSSSSSSSSSSGDAALSKSPSLSFSSPPLSFENHKTHKSHKTNKFFIENTNSDEQSISQSEKKKEAKMNSLYENGYERAYSIYEPSPLTSHLHTRQNSNVLGDEDAEEKAEESKEHEKTGKHSNVEVRRRTVTQELNMDNKDGINDSYATVSTEGITPLNSSSSSSSQSYLSRGYTIDAGVFRTSHSMRSGVNSSSASSSYLKEVAFNGTTSLSSSSSSSSSSSLLLSQQPHLPHLPKRCPPSFLWHPFILLIPIRLGVQRINPIYFQALKTMLTLPFSLGIVGGKPRSSYWFVGFDENFLLYLDPHTTQPATLRKEDINLRTFHQRKILRINIADIDPSMMLCFLLRSKGEFDEFCDLTATITPALFTIMKKKAEMW*

>1050

MSQEPLWLFGHCFPIPEPRLCGQSLSDSMQILEQFDRDFKALTWFTYRKNFKPIGTTNFTSDKGWGCVHRTSQMMFAESMKRIARFQNQNLNPNEEYLITQDIISQFFDEDDALFSIHNIAMRGERYDKKIGKWFAPSDVANVYASLMNGIDNSQLPIVVPCMNNTIVISDVFRTLFEVSHFRRAMRRARLSKCPVERPLDKSLELIVSKFDLSSSTMDKHFREKVKQYMLLSLISDFKYPFSPTPFHIAKHLMKSCPFTSFGSASIMSSSSALSEPSSSPFSQSSEDAAVRGNLIDPFVFEQQLAKRRLSACSDDGSSAQVYACFPPLAPPSLPSVRKEPPPSPPPSVARSLSIFSSPLHTEDCGIESKDALSKVTEFQDDKHETEVKGAEGSREEVEEQLNEGEWENEYCDVNDVSQEDVQGELNKRNSLKHQLVRKRIQRMMKRDSNKQNVSSYSNQNNSPKLSLTHSHSKSSLDSDSSDRSPSFSSESPSSTPPPSPLSHSPSVQSGNDLFDGDWEDAQASFQKFRRTYSTFSPSPSSSSSSSSSSSSSSSSSSSSSLPMFTLPCPQWMFSRSSFLPSPSSYSPQLFLPRRRRLSSVSETCCCNATFESLTSSPSDLSISPFASLQHSIASFKQKAQQNNPFAWSSCSLSAIQSACSSSSSSSSAAAASASVRFPNTASQKSAMDRLNIPIFARTSKKKTHHSLHQHPPPFPPSPLSDSFDRYTPQLAQLPFNSEHSNCSQSSSAAKAKESVCSSSPSLNSSAQPSSTQSAVPSLSSSSTSSSSFNPLSTCASSFSSSSPSSSSSSSSSPPPSSSLNSRLSSSNRKKLEKPSLFHEIPYLSSSSDLFTSPFYLSPFPPLLLLLPVRLGLTTVNIEYYDTIKLCISHPSSVGIVGGKPRCSLWFVGHRGYDLFYLDPHTTQQAVTKERVMREWMSEAQKMSDGRGEGAGDGAPLSASLLSSSVSSSSSFSPSSSFVNQNSGSFSIPSSSTPFSSSSVKKPFRELTEKEKQQQQEMRKARLLIELDLKMEIAPSSLLNSSSSSSGSSGGGGGGGESTTSDWLSANSHSTKKKSFYSPPVPVQPPLPYHSLAQQPNCVPLQAQHQTNTQTLPLLSQSSCYAPSPPPPFYAASLPAVQNPTICGAKQTPQNFSVSNTFQTMQLNNASLVNQTVMPSLNTSNPLCPSSPAAPDATTTSSSSSLSASPSNTLTATTASTSTQTPFPSTTLPTSQHSSNTNTHPVAESPPPPPRLCGLPPSIFSSFTPQRILKMPISEVDPSMLFCFLILSQTDLKSLVMFLRELKEPIISVVQADDMCESGDEIIEPAEEDIIILSEED*

>1051

MQTAIWFNGRVLVNSDEPTITKTYISCPDPMQHFSGLYKSLLQFTYRKNFQSIGQTKKTSDRGWGCVHRTTQMMLAETIKRIAVIQRGISSEKENTILDQEIVPLFLDDPNAPFSIHNISMAGEKLKKKIGSWLSPAESSIIVERLVNHHHHRSLPIVVPCRNSIICLRALAQKIKRESELRNKSKSLLAFPKVTEEGLMETLSSYYSHYSCVVSHYDIVSSTHNPKNRRKVFCLVALAETSSHSSENEFYPFYPSPFRIIHWKEYSKQTIIPKIATKLCESEVIQTARSQSTETSNLPSSEKSRRFSLNEKRKLSFPSFKHPASPPNCPFSQVSSSASSQFSASFIQKQDTPVAKLGLGILLQSQKADISSEESCGEGDETDDGVLILSDDDDAEEEYKCICYKKEKDANASQAELDVCISTKKGLENGILSEKSNSMASAQSQSRLANTPFENVKVVVQQNDVLEKKDGNDESEEESEYEDEEGEEGEVEEEEEEEEGEGEEGEEGEEYEESNEEGEESEEGEEEEDDEDEEDEDDDEEEENDKTVADKVDEEDEYDLVTIPDFEVDMANSIMLNALTSGELDLTEMYKKRRDGEKTEKKSGINNASGVSEREGKVKTDKKKGGIQPMTEKKTKCCDALDEEFEELDDEFEGAEVIEANEEDFYCNASRTDEEEEESEEEGVKGDEEECEEDEDEDELGRDFYECSEGVNDEDQLNSVSSERNRRFKINSTNNERTRSFNLVKEDSSINSFSDAADKDIPTPLQSYQSDLFLTNQSNSIATSCSPINWKISASSSSSSSSLNTLHNLPSFPALSQPPTKCLCFSSPPVLPLSSLLPFIPLSLHNSFSHRSFSYLPSAGVAAPSLASSDDARFALSSDIQPSSLAVSPAASNISSNETFSSSAISSSAVASLSSSSSSSSSSSSFASKAINIRTVSEKDTHRINRLSNLFSSKLSAKRNNSPKSSASSSSSSSSSSSSSSSSSSKSKKKRIKSAKSKRTNKTGISCEVGCPSSSQQKSANCLSNHLNPDPLQAHESVPFHSKTSTQPLSKTPTLPHPTSSSSPSSSSSSSSSSSSSSHSYIFPSPFPPVLLLIPVRLGLSRITEEYIPQIKAALSFPSSVGIVGGKPRSSFWFVGWKGDEVYYLDPHTTQKCAEPFSTKALESAISSSLNHSTTPRNSVSHSSDSMNTSQPLSFSNTPQTAKSPIPSSVDYHSPFPSSSSSSSSSSSSSPFPSSSSSSSSSSSSSSKSSLSYQPRSPSWMSNPETLFESRTSPSPDSPSSPLFSASFSNSSSCQAISRPQPPPPQPMLTSALSSHSSSFSSSSFSLPTSANHTFSLFSSPSTQHASSPLTSSISSSSSSPSSSSSSSTSFLRSSALPHSSSPSPSHMPLLPPPPPPPLPLPLPSLPPLSSLNVLQPLQPSTSFQSDYSMTPPPNQLQCTQIFPSSTSNSFSSFSSSSSSSNQLNSYASDTRNSPSPPPPVPFSIDYINSPFGTTEKKESSSHSSSSPFNSSASSTHVISNLSNPSSSSSSPRSLSFPFPLSMSHSLSTLLSEPLSSSPSLSSFYPRRVLHMPFSSLDPSMMFCFLITSEEELMDLCDYFEKIKDSVIYVKR*

>1052

MSDKIDDETLWLLGTSFLKRRPPHYKNTCAVTRNPKQKFSLSMQSLLWFTYRKHFSEIDKTGFTSDKGWGCVHRTSQMMLAEALKRIELWRKGSTCSKKQDTINRSIIMEFLDDDESTFSIHNICKIGHSIGRTIGTWLSPYDAARILQVIINKSLEPRYPVVKICMDNAIIMEDVFLALCTANEQRQKMLIEKKRKQATSASKDFIFQDNIKGAFNSKKSSGMFSQQKNTFSELNHKNLQVSTVTHRISIPSATETFSSSSLAIISPPRPLNFIEQLKAISAQETRKERVLKAKNDTNFLFEATKSSSPSPSPTSLTTDSFPISSAKFSSAVSSFVAEKNADNKIASMKGRNLEESLKFARDNEKTQNMFRNTSSSTTSTSSSSSSSISSSSSPSSSSSSSSFLKTGTKPASVPSAKGSKIQNVKSEQSEADAKSRREEEDKDRERRIKEHREAVLKKNEKNLIFLVNSITDEVDDEEDDEGSVIVCDDDEMGDDEFVVDDGLNEEEGTDTKHGDRKEEEGKAKEEREREEEEEEEEEEEEEDDDDDDSDEGEQIDVRSSKESNEKLLKRGGRGRGRRDRDRGSRRSRRRSKNERNHRKPSKLDGTKGKANAKEEEEDSKVLIVDDESGREEKRGSIRECGCGCADDSGDGSSDAAKGKRKRVEYGFEVHSWDDGDTESVTETETETEKEEEENTDKGRRKREGGRSSEDVISTEDGDYDSADEYSTITNTSVMEETGISSKRSREEKKREKEKKEKKEKEKEKRRRREKKQNDRKNRRSSGIIVDQNKGKSLIKVLTRTKNEGSSDEWEKWDELDNSNGNAKKGKNDQKGVCSLSHQHSQQHRRHKHKHKHHQCRHHHHHHHTQHKSPKKNSIPIETKQTEHEKIVPAKQNKETPIKNAYEEEDDDEITEISLSDVPCSMHPTAFDSKSNTKTNSNNNSNANSNTSTNAKAKHEIDSENEKENKSDFDSDTFSYSDSDSASTSSSASTSCSTCLSSSFSASSSSSSSSSSSSACSKLSSSLSSYSSSCSSCSSFCSTCSSCASISPSPSSHRHSHSHSHSPQVLYSSLSPLSSHHQRHSSSSSFSQHKKEEVKRSGKRAIPHNSSETSASQSSFASLPSSPCSSFSPSPSYSPSPSPSPSFSPSHSDPSTIALSNAPSPFQSLSPLSLSSRNSSVSSDRNITKDTKKRKEYKQKHNHSHRQGKGEGGGGGEGEINREEMRRGREANEREIQGNSKGIGKMTKKEDKSNQNGINVDNKESKESKERTERSEKEEKEGKKEKKDMIVSSNLVALLGAENEVRRFKHKSHMKEQMKQMKKAKEETRMKHEESRKRKREREERRERRELKEKEKEKEKEKEKENEEEEVEEEEFEGEEEEEEEGTDEGEVFIENDESTSEGETLHDYLINSVTPYLSSSSSSSSSSSSSSSSCSSSSSSSSSSASASSASSSPCPSSFISSPPSPSRPSSSLASSSSTLTSSFSLVSMSQGELITSPVPSPFSPSFTSSTPPSANLLSSANFSSSPFADTQLVKHSPPLLIRNISSTDTTSNNNTNNNKTNDNTDVSLQMKMQKEKEKVEGKENGKDKMKCEDKMIEKKHNFAFTEQSKGISTNYVDHHRHHQNKHEQKHHNHHNHRRHRNRHRHRRHSIKSAHDDDDEEEEEEEDGGGGRRLKRRKKTRRHRRSNTKDETSDSEGKAGRRRRLTKSQKNSNKKKKSRKKSDKFMSNYIKRSRLSPTRVRSMSTPPFTSLCFSPARFRIQPSLYSPICHSFPPVAHKYNQNRSYAAQGEYNSKTKEKEGEQYSSNGIISSSPSSPLLFSDIFPETPFCPFDTPLSYVLKISSQPHKHHKTKADEDDLFERRGGGGGGGSSRGRLKNDKKSAISSEESTFDEENSNLFPVSPFFSPQLPFLPFFAHSTAYPLSPAFSSRFLSCFISRSPSFFFSDPYSASSSSSYSVTSSSLFSSASATASIFSPSFENGPKTASSVSSSHSSPSSSPSSSLLSNADRFSSHSSSPSSSSSSSSSSSSSSSFHSKLPPNLLLHHRFSFIPSLPSSVLYTSLVASSFAFRPLLMLIPVRLGLNSINSEYYSAILKCLSHPLSVGIVGGKPRSSLWFVGFQKNSLYYLDPHTTQEAVTRRIVRERIEYLSSLSSSSSPSPPPSSSPSPSPPPLMRAQHPRSSLSDQPKHNAALSSAPSPLSFHAHSSSSPSSTLKAMASATSSSSSSSSSSFSHSSYPPSLSSPSSFPLSSQKTAESSHLLRNAQNASQMKGSPLKDQSHRLHLSVPSPSFSSSSLSPSSSPSNSSSPSSSLPLSMQLLSVDSARATNTPRISAETTTTSSTSSSSSSSSSSSSSSSSSSSSSSSSSFARNLSSSQPFRFPFFISNIDPSSPSPSSSSSVASSSSSSSSSHSSPPPHIPHASTSFPSKSSPSSSASNSQITNTILNATKTAIVPKHSTHPTQHQSQHTPKLTSANSLPTKLTSSSSSVALQIPSSSSFSSSSSSSSYSDSSSSSSSSSSSSPRPHVTYSSSILRSLSLLQPSMNISFPLPTTRSPSIPYSSSRNGSSSFAGSSRASYTSSSFPPIPPLPLSSSPLYTNPSPYLSSIGLSSFFCRKMKKIALSSVDPSMMLCFLVCSRAELREVKEMFMSIDSGLVSVVMKRNDMYKE*

>1053

MEVRADGAKGLIYMMGMRYTKKEDDSPDSYLKFREDEYSRFLFTYRKNFPQIHNSGKTSDKGWGCMLRTSQMLLGEALSRLYLGRGYRVPKGHKEESPRALWLLRLFLDIPDRPFSLQRISTRGQKYDKRIGSWFGPGNATYVLRDLMRAWNHEQVPLRIVAMTNQSIYTRSVEHIAKRRPSSSSSNSQVYQDIRFPFFTPTPLPSYTPPDGQPEPMIDSFFRMIAAASSSPPSQTQSPGTRGSPTSLSTSASKEESKDATSEGDANSQKTNESEDASSDRPSSSSTPSSSSSSSSSENSSSSEPESIFYPTLLLVTSMLGTNKPAPNCVRPVAHMLSLPWSVGFIGGKPNSSYYFYATREDYVYYLNPHQTQQAVSDPDLSVPFSTATYHGGGLHQKMKILKLDPSVCFGFLFQNEKEWNDFKAEMKKYPDHQILFSIEDRRPGFVQKDTGEFEP*

>1054

MERKVNKNDIWILGNKYSKTTSHLLERAEGNLSPIPHTYQDMLKSRLWITYRKDYQPIIPSKFTSDRGWGCVHRTSQMMFAEALIRLEPYRRSYTGQIDEREILQYFLDTQEAIFSIHNIAQGGMHIGKRIGQWFSPSDSARVFENITSKYNDERFPIIIRAVDCTLIADEILQKMRDAESRRFSQSFVRASDSRKHFKIDEHKASESIQNLAALQPKQNWFRRIFDGTYLDHFLPNIPLLNRIGSPSSSSKEDAQKATEKKLETITIDESDLDEWEDVSNTSCLIIRSSNPTNSKQSFSAEKSLEERNFVEIPPSPSDPPNHFIIKPHKPTPKSKTSSNSPSSSDASQKSHPQSSSSDNHNFFSILSPNKPSKSGAAPTVVSPLDSTQPNIANQIRIAQTPSTILESAMHKQGEKEKVRWKEDELAKGGMRENMKENSLSNGKSDLKYKNINKETKETKGKHANDLRSLLSMKNASNKDASQKIEQDNCLRNDGEKAAYQFQKNREETKKTNENMKTASNISTSNPSSAFQHSPNNSMSSIEFVSCSPSPPSHSQPQNSHCTSTHPSPHSSTECFELVELDVDGGLSKPLGQSFFEGASLCSLNDICLSSPLSHSDSTVKLVSAENKKVVSSQLNENSSDVAADGSSSLVDAKDDDYDIEVDLDEDCVVLSNPLKDEEKEVSNESVHMNSNETSNTVKRETLAASPSLASPSSAIYRGRKEPCGRDKEEDGDRQKKGKNAILNSETESANQSAMNVSMLSTASSASDSSDSSSSSLGSIKLSPPLSPPIDPNAPSTKRRKKRPKVNLLEAATTPFIMSDEISSPAKSPSSHKANSECASPDSSSSRSTPPLPLGFVQTSQNAHNKEEKNASSSLSNALQSPLTQPDESDLNDSCSSLLMSSPLSSPLSPPSSLSDSPSSITTSTSSPSSSTSSSPSSHSSPSSTSSSSPSPPLTVKPKIAPNLLTSFVASATVPVRWIEQQQQKRRSTEETERHNRIDEHSHSNRETGNKSNKSNKNSEEIETNRNNENNKTPSLLSPISRIGFNSPQFSPFSSFVSFNTPPSISDSQHHVKLSSSIRSFYSPQSSCSSSSLCPSSSPSSSSSPPPSSYLLASQSSLSLHTPKSSSSPSPSSSFISSTSSSLRVPASTKCHTEHSSESSLASSFSSSTSSSSSSSSSSSLSSTHYSSSSSSSSSLRPSAVSWTPLIVLIPMRLGLSSVDSSVIPSFLSLFTLPWSLGFVGGKPRRSFWFVGCSSSSLFYLDPHTTQRAAVDARNIPLETFHQNSVKEIDIKNIDPSTMACFLLPTYNDFVEFCSAVQEMNNPLFTIIDHKSSRW*

>1055

MVAFVNDYIKAPLRGMWEFFTQPKVSPKGAIAMIGKTHTNKESFFKDVCSRPFITYRRNFLKTSKGFTTDAGFGCMIRTGSMLVAEAIQRIFFGRGYCMDPTYSLFCPRSVWIASLFADEVKPNTRHLHPFSVQHLMEFGEAHAKKEGEWFAPGTIAFAYRDIISEYCEEQLRMNVYVCSSGLNTVFMPDVINLLKSPLQIPKPLPHTCEIVPYCPECYIQDCPCRAAITVNEGEVLKRQKQKTNSRLEDEEQKSDGTMFAMIPLEPKERKGFCNASNSASDKSIQDISPNSIHTPADSSSNAQTIQSEKECFLKEQQGNSPVAACPTDALLLQGEHSSLTGSGLQERQNEALCQMQCSDESSEIFGTFEFSSLDIPPITQSPPEKIASSISSPISAILVPPQHVTLPSSLSPSHSSSSSQSPSQSSSSSAADSVPHASQHRSHSKTHRVSSYSSLAPAHSPFSGACTSILLPINSTHQTYPGETGSSHSLSPFPAALPPESSHASASLPTFTPSPHLHADTPTSLSSAGSNEPSGSLSSDVSPSLSSSTPTHTPTHTPTHTPSFRSVSPRNSTSAALKRPHSISSYSAQSNHHLLILLPTRLGLTHIPKHYYRVILNLFQLPFFCGIAGGHPNSAHWFFASEGELLYYLDPHITREAEDVEMLKESIASEVQASHMEKKMKKSRIKEAEEVGRKGNEGEAQISENKGIVKSDESDEDESESGEEHCFVRVTDLDTIKKKEAGKTANKKNLQQQKKLSSPSSSSHSSPSMGNKTASGSATAAVSLSERETNKNEKQSPQFSLSISSDLSLPTNSNQTASSQQAVQTTSTVPETSTPSSSPSPSPSPSPSPSSSSSSSSSIPQSFIHTSPSFAGVSSLASSPMQVPSANNNNSVFSNTTSKTQAQSKSPSSITLDNEISQPHSTAPSSPSPSFATQNAKVLSPQSFVSTSSAAAADNDALNNPTEQPPQSLPASPSSSSSPSVSSCNSPPPPHSPPPPPTSSTSPSAFSVSAPQTQPPIITPITTQPGDSLSSDELSASSFCNITSPLENSPLASTSCCSTPKPNPFSLVSARMMDSATPSALASQMPHSSSSLSFHPGKRSDIHAMHISSLDPSMCLGFLVGSVEELGELRKLVEDMFAMNVEIIAAERKKKEDKKRGKQNKEKGKENKSKMEKEEKEKEKEKEKEKEKGKNTNKTTENKTTPKEELKTEEEKENDKFRMEEIRKAVREEVTPFLFFEDVSQAEMDKTREMETTRKTAVIKHYEKLEALEREEKREESNSNEEGKAEKESRAVANSGFAVEQGSAEKKKGKVASHIAPGQGNFSDEDDDSFAIIEL*

>1056

MIQLKENELEKSILHVKHNEYILPSVLLEPINFEKSSDEIESRCFFDDIASLANQFILPPQVPISLEKNNIPIKLPAFVKEMEKALSSKSALPHASSKSNPMNAKEHCRFLSEKQHQFGQTSTDSYTEKAKANANEVKKQTTMGNAASLFSLDASTHVKGLDAEQKKDQHSELKKERNETSLKSSCLSDAMQFDRLDFPENFDCEEGFDTQIDNFDEDSCFIPEIEENALDSKRKSEKEEEEEEEEEKDKNRIWVTRSSNSLNHLNIQENTLTDGKEDETLEYLITDYPILTEEEEEEERGRGRGRGRGRGRGRGRDEYKSEKHKEASNHFESANTQETNHVSKDKDEMPVKINMNYSLSAVKREVEDGKDGKEEKEKEENNKTNASMPVSASTDTFSCTSNNLAEEPTLVIEAHSEQRETGLFAENSNTFTTSSVCVNQSSPPLPPPPLSSSVLSFSPLSFPRPLHHPSFLSSPSSPSSSHSSSSSSSSSSSPSSRYPLRSPPFPPPPQPHRQSQSHPHLPHNQSPSSLFDEVAFISPSPLFLSPAKSAQLVPLPTKPPPSLAQKTSCSIQSTHNNEKLHLLNKQDNSRDGSVEYKVGEEAENFMSNQIKQKMNEQKEKEFSQMCRCSKDAEDNNAFLSTQQSPQLQTTSCFPSSSSYSINSDSSSPSLPHSFSHSLSHSLPHSHPHTHTSPHTHPHTVNPLVSSPTLCSVSSSTLPLNTLCEPPLIEPAAFDARSISSSDCFSLTSFVDAESDAIGAQSEHSGSEEGSIRSYSVSLADNSSSSSSSSSSSLSSSSSSSSSSSSSSSSSSSSSVSSLSADVKESLSPSSSLSSPSPDTSPSRLVSAQLSSSFESTSASASASSSASVSTSVSNEHSSSEPLSSSSSSSSSSSCSSSSSSSSSSSFSSFSSSQTLSLSDSSGNNTSSQATSSPSLPSPAAASPTLRSTFLSNGTNFSSSSSSSSASTSSSFSSSSSSSSFINSSSSSSSSSSPDLSFQFIQTPSQEDASFSSFTYISASKLQPHEPSLTNSKEKYTSFIHSNQNKRERHSFVNSNSNNHNTKTHSSRSSSMSSSSSSSSSSSHSSSSSSSSSSSSSLSSSFFSPLLLLLPVRLGLSKVSPEAAYAISYFFTIPFCVGLIGGKPRSSFWFIGVDAAVQNSEEEELKGEETEENELGQSEDEAIDEAIDAVICSANTISSANSQEDLQNLNEKERKKLLSPQCSPSEYMQDTHLFYLDPHVTQPAVLNKESINSQSSSFTQQRILSIPLCNIDPTVLVCFFVASLEEFDCLCEELKKMNNPLIYVEKAKPDFFSEN*

>1057

MTEFVQDYIKAPIRKMWQSINQPKFDPNDEIVMMGKVYHNEKQFYADAMNRMYITYRRGFHPTSKGFSSDTGFGCMIRSGSMMVGEALQRILFGRDFKIDDNLFPYCPRSVWFCSLFSEEIRPNSRHLHPFSVQHLMELGEKYGKPQGEWFASGTIAKVYSEIIESYIPPPLQVGVYVCNPGLNTIFLPEVISLMREQPKPPNPFIYNDSATKSEKKAYVPQCPTCPIPSCPCHEQERKNKAKIMSMKQKKEALKLKKRKYNTPQIKSISDSQEAELSSVHATRSEPSLLLNSDASDSPLSQSSNSTSLFFASEDISSPSLYPTVSTHILSFEGQNIPPPPATPPLSILSLSPPPPPSRPVIILLPTRLGINKIPLCLHSVIYALFSLPSFCGIAGGHPHVAHFFFAAMKVKNSEEKEKEKQSASVAQNSSSTSSSSSTSSSAVPPHPPKKSQPSKVILFYLDPHTTRKTENVECWWSDEMEQKMQTSTMDPHSAADEEQTSDESPSPSPLRLQNGSASAIQSDDGRALSAKEDSNSPSALPKETDVTEAAQYLTQSNFCGLNDSDRQLTLTNATEDTAGIKDEQNTADQTLSEKPTEQISLFELGKKKTPEPPPSQPLSPHSLVPTLEPFNTSSASSSSSQTERIPLSSSAPSATPLGMADETLSLFTPSNPEIFTSLTSSPSSTASPSSSSSSTLSTSSPSSPLMPSFSSHHVSFDIPRDTTDASEYQSDIPSPSHHSHSFSAAASAPSSPAIQPISHSTHSLPPLPPPSRYSVSLKSAHLPSLGSFPFADGAPVPKSPRATVTHTRSSFSAKPSRMFSTECTTTSVELSPLSVKSKDTSTATSNSSFGYQSQVVHSSLFSSSSSSSSSSFSSTASTPKQGNACFPPILSALSLHPSKKDEVHTMSLSSVDPSMCLCFVVRNEKELSSLQKQIERIFDMEEEKLMNELCEENGSEEEEDEEKDEEEEEKESYEGDIEEEVVENTAEIQKKIEKISSEEIPIDKQENLKKMFSEILNKNKNSSPKEESGVINVEEKRNRQLARFIHFQNIFSDRYGTCGSANAAVSNKNSSQSSRNGGDANKDEKKSVGYSFSSVNANEQDSVAKQKANDKLVIGKDNNSSSSDSSSSSSSSSAFAPSKGEDNLKMKYDDSDNFTLVDLN*

>1058

MDSFYVYGEDTIWVLGTPYPSILKLKSDPTLQNKITYQERLQSLILLTYRRNFPPLLNTKITSDRGWGCLLRTSQMMFAEALLRVEKERLPDDSVLDIDAIINQFLDTPLSPFSLHNISVVGTRFGKKVGSWFGPSDGANTLQHISLQSNNLKFPAVISCMDTIILKEVIYQRLADLNQRQVEKSLLRTSRIAPHPSVGPDGVQKMPMKFRSQKTIEHDLGIVKGKARHRFAPLDYQKQTIFIAPRSAPAPTNPGAINLSLSSSSSTSTSSSSSSPSSHSIDQSSNCIKIGSRIVREDAPCEDLEGFTSIVADENDDKLDEEQNNATASGESESQKGSDEAKEGASSEEEQSKTDNEKMQLLMKLSLSSSLPVSTYSIASVERSASASSFQQLQEAEQKDMIPPSPFKTPLSPANSSPVQSVSSNHTQLSPLQSSSSSSSSSSPTSPSASSSSSSSSAGLSPHSPDDTSSSSITVSSPLQQPANMPVILSQQHTGAMLTCPSSLARLSLSLSSFTNTSEPFYLSKPTSLSASFAHSFLRKEEDKEEAEAKHTARAASPLKGQSPVSPMLQSQAQTSQNGTLASSTSSSSSSSSSNSNNNSCSTTTLSSSSSSSTSPNIQSTCLSPSHASLSPSRTFSPSASSISSRSSTDTSSSFFTPLPAPAILRSASISPSSTCSDPPSPQVVSNALSSSSSTHQADHLSSSPSLATSLTTSLTTATETSILSGSDSSAPKLPLPSQISPISSPHSHPIPILPSLRSIQLSSGASLPSSVPSISSISSISIGASSSPSSIPTSPYQSFISSPHSTPLSVSSAISQAITEKLNISPDDSVRSSNALSSSSSSSSSSSSSSSSSSSSSSSSSSSSSSSSSSXXXXXXXXXXXXXXXXXXXXXXXXXXXXXXXXXXXSSSSSSSSSSISSSQSPSSPSSSNSSNSSSPSSSFRSPSSTAHKNLPSKEIVPSSTLISPKTSQNASESENESDDESLTNFELVGETPLNRLHSSSLSQNGSLPAAYAATPSRGMLNPSTRPSSASFSSISSTSSSSSSPASSVASSSSSSLSSSVFTEEASSLTSTSSSLQPLSLHPFPVEPPRPLQTTDDPIHTLYSTRTIDLSEIEDAPRDNSRGKEREEEEEEEEENKGEKEDGNSTDGTGKEKEDRGAYKCPSLPDISGSVSYMKSASSAPSLAASLSKSSSSSSSSSSSSSSTSSSSSKTGSSAFFRFRRAPTFASASSSPSTFESISYIRQRGRSTAVRPGSPSAWTADGKFSCSTSSLVPNTALAPPSGPWHPLVLLVPVRLGVESVNPVYYPSLRAIFTLPFTLGIVGGKPRSSYWFVGVQQNRLFYLDPHVTQPAVTKREQIEPMMHTFHQKNILSINMANIDPSMMLAFLVSSRQDWDEFCQRINEIEPPLFSLLEKKAQLWGE*

>1059

MTEFVQDYIKAPIRKMWQSITQPKFDANSEIAMMGKVYRNERKFYEDAMNRMFVTYRRGFFPTSKGLTSDTGFGCMIRSGSMMFGEALQRILFGRDFHVDEAYFTNCPRSVWLSSLFSEEMRPNTRHLHPFSIQHLMELGEKYGKAQGEWFSSGTISHVLAEIVKRYIPPPLQIGVYVCDNGLNTVFLPDALEMMHKQQIPPQPFIYGKGKFISAPTYVPHYPTCPVGSCPCHEMERMKVKKEKELQMQKEKKLQKQMENNKNEESQFPSPLSEPSPSFSSSFVPNTDSSVSTFMPSFDVSPSHNKNAEDGLSTVSSNSKNPFIENPFQAKQDSLISSDESCDESDEVSIVDPFTQQTSQLDLPSSDSANDTTRPLIILLPTRLGINRIPSCLHPVIYSLFYLPSFCGIAGGHPRIAHFFFAATKVKIQDTSNTSSDEAKGSTYQTAPFASATATPRQVNAADTSAQNAPKKKKKPKEKIILFYLDPHTTRKAEDVECWWDDAMENAMDGITDDIIQPTLAGREKMDNEGERIDVLSEENCENDKLEISSEKEVPTENNTTTNENETQKEESISNETNNSSVLIQPNEPDKSVLPLSIASESSTETKTESESAALFDDSPEERYIDTSPPLQMTYPKGNKNKTRQKTTSASSDAPSLFSSFSPVSSPLFSSSKNTSDEDNLLFDGKELESKGIEEEKNYKLQNKKQTFHNNTNQSLHPSSSSSSPSHSSAPSPSIHKASAVKASSSFVSFSSVEDSPLTILTASDPLSSSVPHNAVSSTTTSSSSNSAASSSSSSSSSSSSSSSSSSSSTPTSASSSSSSPPSPPPPSTTTPKPIAPPPTAMPIAPRPYQRVYPPIMSALSFHPSKREDVHAMYLSSVDPSLCLCFVVRDEQELQTLQNQIDRIFNMEEEELIEELKEKMGNGDEKENIEIDSDAEEVDFPEAVSQQTYQHIRQRRERAANAICEAPPSSFVTLPERNIAKRTPNKEELRKRQLTRFIHFEKQTSKEFGLIKTESDPYENGGQVENVIHMLKIRDEETLLRNQEEERLLAERKELEEPESDEEDSIDYGEIESEPEVLGANERNVDAASSADSKCQTKNLRSSGEFVLTSSLDMDNANEDYQMTRSFKEKKEKEKEKEKEKEIKSDNKNQCNISVSVRSLAESNDDDSDGFTVIDLN*

>1060

MSYSGSFQEGGASELVSAAFLDGASWSAALWLFLGLVGTAAGWQHLAPQLPVAMASFRTGFLSWLNNTRYRIGNTAEIRTGEMMFLLGAAYQVPPRPDKEAPEEEQAAAREALRMFAQQLEDDVATRIWFTYRKDFPPLPSSRRTTDVGWGCMLRCGQMILATTLMAVLQPRVHHLLKYTMENHHLKAGRFQGPSSVGSALLHQVPSALAQLNQFRDEEVKLRTYFASDTLVILDQLRPEEGQAEFEPIMLVLPLRLGIEKIGPQYHARLQLLLRQPWCMGFIGGHDKRAMYIFGYQGHQYFGLDPHRCSAAVAQSTAELRDRWVEVRDSFHTSKLSGIERDDLDPSLAVFLLARTAEELDDMLSVIGQPTSEDRPGPALVSVVQHSPVGLF

>1061

MAHAVFTYDVGDLEHEEGFADEVEHTVIILGREFKFKYTDQGSRDEIRSRASAFLWFTYRNSEYAIGDSPRHKTDRGWGCTLRVGQMIVGEALQRCHCPRDYDKLSYPSEAARMSILKEFEDRPDRVLSVHAMAMQSKFVGKRAGQWHTPTDVAHVLRLAVNEQEAMGLQVHVAMDSMVVLDDLRKLFRADRATLLFVPLRLGIDIVQAEMIPAVKRFFHSPSALGIMGGRPGAAHYFIGYMDHNLLLLDPHTTQDPLRAGSQDALVSCRCSRPMLLDLDKVDPTMCLAFLLTDEESLQRFADDYNASVEETGVRLFSMLDTKSFASSVAVASSLAEEEEFSDDDFEVINMSAMS

>1062

MNPPLTDDSRGYFAGGCEPELDERQIRQQERHLEKQQEKELEKARKAARARERDIEKEIERNQEKERAKEKARIKNIEKQEKIREKLLAKEKEREKDQLRKQHEAGIFDSKLFVSGEDRSNIGLGLQFPNDSKDTKQGVVRRMRSLSILQKVPSAMDLLPSRKTLQDSTSGRPIERPLQMRSQSISNLSALSQKSATSSTVSNSEANRSPPMSSSLKSSISVQEALAIAPKQSVLSKLAHLNLSQKKLTAQLNFDELPDIPVYKIQENNTDEASTTLSPALPLSPTSGDSRTQPSPHGTPNRRRMTISGIFSKDKSPNSSGSLATLKTLVASRSPPKSNNDTSSRMDGPPSSLGPSGPRITFGIPSTDKYDFTSRKGSVGRVQNWLERRLSSNNIANVFIASTDEPVPQMTLDVTLSPPTSVSGNSEIADPSSLTDFTSHLKEARSRKRSSLFSSSSSSTPPTPTKSALKFGNSRSPTLPQTPTMSGSEESSLNTLHPLDHGPPSRTPPYNTTWTSEVDTSFTDQKTSSPKSRHQAPVIQRGLSEGLDGESFVNVVILPPLPPESPSEAFVLLDKEAELASKASHDGEDPVAVRDDHRSGSFVQVDHVKSPERLDSDIEAVKEQSQEIFQQIAAFSSEKSEILSDLQLDWESLWPGKASKVEPTDDAGEFVHVKESQDNVDIAAEASTPKPERQRVRKTGGTYVKVKEVKETPFTPAIIAPQPKPLVPVQTKVLENSLYPTVPEQPQTVPHQPQHDAPPISMKQRALALPSLLTTNIPRQPSPNSAGSSLSRSWRAISPRSLSPRLSLPVTSHSSSTSASLHSVGADILSDNKPVYMRERRNGIKETGPNSKLHPLAHSALAKPASFDEPTPNQLILQRFMQDFQSRIWFTYRKDIARIEPSFYTSDAGWGCMMRTGQSLLAEAFVQIMLGRGWRATGAHSVDTSEKYRTILNWFADEPDRYYSIHNIAKSGLALDKRVGDWFGPSTMAHALKQLSQKHNDCPVHISVPMDNQLRASEIVQIALGGSKDYVNGATVPSPSSTTFNGLDRWKPVVLLMPVRYGLEKLTEKYSNNLKKLFELPQFLGIAGGRPGRSLYFVACQGNELFYFDPHFVKPRATQEELNQCPAVSYHCNVVRTMEMQELDPSMMLGFLIRSQDDLLDLDHRLKNDMEQAYPLVTIVNDISLLPGARPEPRPVQSKRKSSKDCVVIEVKEVAEESMQIIEPPTSQTPEPHHHDHHHEEEHVGRSHKEMKRVAKALKKEQKLMKSRANREKKKKAKQPEHFDPYVYRFTTHDGDHAHDQETLSVKSFDSDSSL

>1063

MQAVDSARDHAATEVPVTPAIVPATATSQTSEPGQKAAFSSMTPISSTDKTKAKAMTTAGEMAATCSEQKSKENSQEDTLAKDNPTSLTCSNGGSIPVSSDATSPSLPPTSHSLQGLSSGVQQSQQVQGQPDTVPNSATSRLGSRFSVNLSGVGLPAMATNLGLTQANSERAADMAQDLKKNVVNMWHAWFPAPSSSSPTDTLSSGDPQSTDVETSIRSPTSGTQSLSQHPQQRPVKAQRMNSYTMGAKSGTDIKNLALSPVQTTRAYSFDISNRPSTSPKSSDDVAQPEHDPYLVESRSSVSAPPAANNNNTTPIYLMGNFYPPSPTQWTDFQRDFTNGLIWCTYRHSYAPIKPSNFTTDVGWGCMLRSGQGLLANALAIQFMGRDWSRPVPGDANWEIYVRILSWFLDDMNAKSPFSVHRIALLGKQLGKNIGEWFGPSTTSQVTKALVHNFPESGLGVYVTTDGVVYKDQVEEAATLKKKDGFGHLLILVTIRLGIDKLNPIYNDAIKTTFEFPQSLGIAGGRPSSSYYFVGYQGDDLFYLDPHHSRCVVETKELSEYVAEDFATYHCEVVRKIDINSIDPSMLLAFYCRDRSDFDSFCERVKEMNTRPGMGSSIFTIGEKAPDYGDDEGHVLSVADEEDDDLELIL

>1064

RHVFGSNWRAPRDLDDLKSNPLYCQLLDSFVDAPGPNHVFSVHNMVQIGMSYDKLPGEWYGPTTVAYILRDLALLHRRQQQDVEEEAHALQPVGGE

>1065

MPTSLPSRPQYVPVSTCPLSTSSAHTVRPKEIVFELFLPLAPLSAARRASASSSSSPSSSVTGRWILGKRYDPEDLDGWTQHFRSIPWFTYRHTFPTMAPYPYTDDAGWGCMLRSAQMMMGEGLLRCERGWRGVHVET

>1066

MDSQINYNKKDNTSIDREEQELEGIINNNETNAQLKHKKSKNQNSVSYYYYNVVNWTSDVIIQAKERLKSRTNPVWLLGQYYPNGMEKANQLESFFFLDHNSRIWLTYRYSFEPIFPSDYTSDMGWGCMLRSGQMLLANALVYHFLGRDWRINNKEKYQFYKKLIGWFMDFNEKPFSIHKIAKLGQKYGKTIGEWFGPNIISKVLKDAMEEFSECNNCISVCISVDQSIYKDEVLKVTNNWKKALILLLPIRLGIKNLNPLYYNDLKKCFQFPQCIGIGGGKPKSSLYLIGNDDNDIIYLDPHIIKPALPRKDNYDDNDYLSYRCPTVQTINIASLDPSMVIGFYFSDEGDFNDFCKLYENSKGQYPVFVISDKKPEYLDEDIDIISVPTH

>1067

MDSQKDFNENNDARNVEEEEKKIKENSDNSKYNDEINVQLEHPVWLLGQYYPNGMEKANQLESSFFLDHNSRIWITYRYSFEPILPSNYTSDLGWGCMLRSGQMLLANALIYHFLGRDWRINIREKYETYKKLIGWFMDFPEKPFSIHKIATLGQKYDKAIGEWFGPNIISQVLKNAVEEFSECNNIISICVSVDQSIYKDEILKVTNNWKKALVLLLPIRLGIKNLNPLYYDDLKKYFQFPQCIGIGGGKPKSSLYLIGNDGIV

>1068

MVTFDDVAETRASSALEATTTSDANETRRKDDEIERGDDGDVIEPFGEKVRRFARVSRAAVAHHPRVRALGRLVTRKTLDDLPPDASLVMFGVTHWDRETSSGERSNEVGRREWERDWRSRCWMTYRRGFEALGRTKWCTDAGWGCTLRSAQMMLANALSIHSRGRHWRREVQLVAVHENETADDGSKSPAVSFLSGVVNKLKIPQSERTRAGSDAQEDILRLFADEVGAPFSIHRVCEKTTEWGAPPGRWFEPSVMCRAFEALVAEHDLGSELTVHVVSGREGEDGGVPTVDEAEVRAKSADVGKALLLFVPVVLGVGRTINARYLSQLRSMMAFKQSVGIVGGRPNSSLYLVGHSDDVFFYLDPHTVQVASSMVTMDFESYYCPTPLHVCGGDLDPTLALGFYCRDGDDVASLLVDIEALARVNATAPALAIRPADDRYKSISPRHAERASPSVPPTDIRDDEFADWTFV

>1069

MYIFGQEIQNVDSFLQLKETFIWFSYRANIQYEGRAISDQGWGCLIRVGQMIVANSLIRESTNSKPNDLKTKIICLFDDNQCFSTLAPFSIQQIIKRADLVYNIKIGDWYTGPKIMCLLEDLLQSAKTIKQLKIINFLEQCVIEKQIDLQFKQPQLLIIHAIIGNKELDQYFVAELQKHMQIPQFAGAIVGKSKKAYFLIGYQNNQGIVMDPHYVQESNLLQLNSQLKCIPLKEFSGTIALCYYISNSYDYQQLKTNLKDLKGSIFSIIDETCTCFF

>1070

MFQSLLNLKYSWQQSSKEDINNKELVDAAISILGFSIKNLPSEKKKEIIQQIYSRTIWFTYRKNFPQILNSQQTSDAGWGCMLRSGQMIWAQILRVHIRQKKQHSKDYQYKLLCAFSDDDDDEHKKMFTDNFKLCLSPYSIQKIEAISQIKFSMKPCQWYRPDQILNALSLLHQQKQLEGSEDLEITISDSLLYDRLYSEMYGLKMDCEHIVNEIKQDKNKEISKICNICQKKDPKALAIFFITRIGLDEINKEYLPFLNDLIDLPQFQGIIGGRDDKAYYILGRVNKRLIYLDPHYIQEHINRGNVVMLKDTFFCKDVKYINEEQMSPSIALGFYCQNQSELDKFFNSIEQIKKNYDNEKTFGYISRITPNSYIIGFDENDILVIKEDEI

>1071

MQQIGHSILSKWYSSNYFYRALFQYEVKEEGYIMGKLIESKEDILDVVIHTIRFTYREGFQAYQCQNSTLTTDSGWGCVIRVGQMMMAELLKRHLKCFYNVNLFQFPPLMQEVLQLFKDDDEMESLKVQGKPSKYGFSIQKIMRIAYEEWGKKPGEWYSPNQIVQAIYKILSDNNIIYSCGLSLLPFYESQIDLKVILQEMCVMENCICEQRVFFIEKFLQDLVRLEINKEEVIQVIHGNDSISDVYYEDLSQQNKQEIGMLLKKYVCQKCFVPIRAVAICLLSRIGCDEPNPDYIQAIRQFMKKKYFAGLLGGRPREANFIVGFVDDKFVVLDPHLVQQANMNPEEYVKSCFPGEALFMSDKEIDCSLGLVFYLKNEEDLIELIYDIQAHQQINFFSFATIQNWTYSKIQKEVELEKIKEMQNFLNEFLVQQQQQILNLDVSANLEGYEIGSSQQTLVNDIEYSYEQI

>1072

MDQRQENYKEVITDWIYNLKYILSVYLNNKNVPSDHPIYILGHRIDIDQFEIEDRINKIKQLVQETIWITYRRNYPPLYQSNYISDTGWGCMLRVGQMAMAQMLKKHLKNHGDKRDEDYDNIILAFADNDSQENKEFIEFQNSKDKQKAHNFICPFSIQKIAYLAKKEFNLDPGEWYRPNYILFLLELLHNTIPIRASENLKLSVFNDSCLFLDQLMNRMFEAKFETDKDLEEQLEKTQLIGKNSLAIFVLTRIGLDEPNQKYLKILDEIMELPYFQGIVGGTPKRAFYILGKINDHYLYLDPHYVQEAENKDQINENKMFNRTSYSCKNIHLLNQKHVDTSMGLSFYIRNQSELLQFWRNMKQIKQSSDDFFIFLSDSAPEYVDYSGQLEESSNKLNDDDVVFLQ

>1073

MKQKQGFGQSLMQMMTNTAFQLDKMWSEDIKIDELMYIFGQEIQNAEAFNQKKDTLIWFCYRANIQFEGKAISDQGWGCLVRVGQMMLANALMRECKILAINKTKAMIIHLFDDNQEYSTIAPFSIQQIIKRASINLNMKIGDWYTGPKIMSVIEDLNKNNMNIKQINLVNFLEQCVLESQIDLSFKKPHLLIIHAIIGDKSLGQLEIQNLQSHMQISQFAGAIIGKNNKAFFLIGFQKNNAIFMDPHYVQESNKIEMECNLKCQPLKQLNGTIALAFYISNYMEYLEFKKQVNKLQGSIFSIIEDIGYKFT

>1074

MQNLCESLLNRWYNTKFNLTQYVYDIDKQLRQQDKIIHILNETISNDADIEQRIEKVKQTCSKIIWFSYRKNIPKFQVSSLTSDTGWGCMIRVAQMALAQIIRYYNYFKKPEQLIVLIRHFIDDDDNELTDFIQQFHKNQNQYYHAPFSIQKIVHYAKVELKKEPGDWYKSDEILQTLDYLFKYSQYSLNMEIYINYDCAFILQDAIQQMFNQQEGNEIWLKERAKNNNQFDLQDHKGICIFLPTRIGLQNINKDYLEVLNQIIALPYFQGMIGGVSKRALYFVGRIQDYLIYLDPHFVQNAQNFDDLSKNQASYTCQNIQLIHNSLIDPSIVVCLCIRNALELLDLWQIFQHFKQEYQDLFFFSLLETKAEIEKSFEQIFGDDNEFINIAK

>1075

MQSIGNQILSKWYSTNYTYRALFQYEVKEEGYIMGQLIERNEDILDVVVHTIRFTYRQGFQAYQCQDSALTTDSGWGCVIRVGQMMMAELLKRHLKCFYKVDLFSFPPLLQDVLQMFKDDDDMESQKGFSKPSKYGFSIQKIMRVAYKEWGKKPGEWYSPNQIVQAIYKILQEINIPYCYGLGFVPFYESQIDLRAIFQEMCMMEDCVCQKKVFSIEQFLKSLEKLEIGKEEMVQVMHGNDSISDVCCEDQSEQNKKEIGNLLKKYICQKCFVPVRAVAVCLLSRIGCDEPNPDYLQAIRQFMKKKYFAGMLGGRPKEANFIVGFVDNKFVVLDPHLVQEAKMNPEEYIKSCFPGEALFMSDKEIDCSLGLVFYLKNLDDLIELIYDIQAHQQINFFSFAHIQHWKYTETKKEDQIRRIREMHEFLNEFLVPQQQQQILNLEISTYQEAYSNDSASFDQQPILKEIDSSYEEI

>1076

MDQRQENYKEVLIDWIYNLKYILNAYLNNKNVPPGHPIYILGNRIDIENNDIEDRINKIKQLVQDTIWITYRRNFPPLYQSNYISDTGWGCMLRVGQMAMAQMLKKHLKNHGDKRDEDYDNILLAFADNDSQECKEFIEFQNKKEKQKVHNFICPFSIQKIAYLAKKEFNLDPGEWYKPNYILFLLEELHNTIPIRASENLKLSVFNDSCLFLDQLMNRMFDIKFETDKDLEEQLEKTQLKSKNSLAIFVLTRIGLDEPNQKYLKVLDELMELPYFQGIVGGTPKRAFYILGRINDHYIYLDPHYVQEAENKGQIIENKMFNRTSYSCKYIHLLNQKHVDTSMGLSYYIRNKSELLQFWRDMKKIKQKSDDFFIFLSDTTPEYVDYSNQLEESSNKLNDDDVVFLQ

>1077

MYNLCGSLLDRWYNVKFNLIQYVYDIDKQIRQNDKVIHILNETIKNDVGIEVRNPSFILKQRIEKLKRICSSIIWFSYRKKIPQFQISSLTSDTGWGCMIRVAQMALAQVIRHYHSFTQPEQLIVLIRHFLDDDDDELINFIKQDQKNQVQYYHAPFSIQKIVYHAKVEFKKEPGDWYKPNEILETLNYLFKYSQYSLNMQIYINYQCAFILQDAIKQMFNYDKGNQEWLKECIKNNNQFISQHDKGIAIFLPARIGLQRVNQDYLEVLNILMTLPYFQGIIGGVTNRAFYIVGRIQDYLIYLDPHFVQNAQNFEDLSKTQASYTCQNIQLIHNKSIDPSIVVCLCVRNGLELLDLWHSLNHMKQEFQEFFFISILDTNVELQISESFQYLDENHELVNILK

>1078

MQYIYGTFIDGWYNLRFFLISYFQDSEKRLRQIPNNIYILNHVINDDLAVDQKMEKLKSLFEGTIWFSYRSKILQLQYSTLTSDTGWGCMLRVGQMAMCQQIKYFYNLSSSQELTELIQQFADNDEEELSKFMDRNDGDQTIQYKSPFSIQKIVVQTKLELQKSPGEWYKPNDILFVLKYLFRYSKYQKNLRMHINHENAFILSDVISLMFNKNGGDEEWLKEQIEKGQNDEFGVSIFILTRIGLDTCNQEYLKVLNDIMTYPQFQGILGGFPNKALYILGRVGNYYIYLDPHYVQNAQNYQEMENDRSSYTCQSIQLIDSNQLDPSMAISFCVKNALDLLDLWRRLKQTKSENGESFFMALTETHVYYQLAQSYYCVSDEDDFVTILH

>1079

MSIVRNSTADSSVVLVTAPGWSERSRSDGLVQVEAVRSENVDIPKTNLIKHFVESRFGGETKDGHLTEMKEMPLKVTPSPNQQTEADADFDACQQYNCETSGENEKEEASDLNGPFGTMSAFASAVMSSILLDGNNSESPQSNPSVESVSMSFVLGKTYHPLHDYSIRRDDERSLFWFTYRCDFPEIAPYNITSDAGWGCMLRSAQMMLGQALRLHFKSRDWRPPQLLARRRQDSFIRSVLTWFADYPSSSESVYSLHNMVAAGLSKYDKLPGEWYGPGTACYVMRDLVHIHEKQQALGKTRLDRRIFRVYVAPQGTVYRDTIHAFMTTEARVRIEEKKKVKEQTQPQAHPLDLEWEEELMESANTVEWDTALLLLVPLRLGLTSLNEEYVQSLAHTFSLPQSVGVLGGRPRGARWFYGAQKDGSKIFGLDPHTVQTAPGRQTARVNGQASSVVELSDDYLRSCHTTCPEMFPFCKMDPSIALGFYCRTRADLNHVLNSMGAWQKEHSSIPELFSVLDRAPDYSANVDDLLLGGDSSMMETSGFEDEASDADEYVML

>1080

MSQFAHGISALDVTPSAALSSPVWLLGKRYDDVAAADFDAYKKSFEAILWFTYRRDFPAMTPYEHTSDAGWGCMLRSAQMLLGQALQKRLLGRDWRLPALFETEMDNKLPSNYVKLLKWFADSPDLECRYSIHHMVKLGMQYDKLPGEWYGPTTAAQVLRDLVNLHRREFGGELAMYVPQEGVVYSDDVTTLCVSQIQEEEVTAKVNHNEDEGNKPEFFDPLLHPPTTEDRSDWSTALLILIPLRLGLDQVNECYVPAIQKTFAFPQSVGIIGGKKGHSVYFVGTQQDQLHLLDPHDVHPAPEINAAFPTATHLRTVHSSRPLVMNVTTIDPSLALGFLCETRADYEDFERRVRILHDEVKAEGGMCPFSVAARRPDYSASGGDLLMADCLSGDDMNEDELGGGTGGDGEDDEDDYVLL

>1081

MSKEMKNFEDNQKKIEINSTIQEGKEEENKTLDEIKNVPVAINSNKINKKDNKYIKKKNSKKTKNNFIHIKNKNVDDLYSYQNKSKYKILNYFNNFKYNFSIKRYFKILVNVSLLNYLPLNLRNISNKVYIFGLCLNLKNPDEMKIFLILCKSKILFTYRSNFLIRINDNNLYRSNNIMLVDNNVSQSNIFTYDNTTYNNSNITINNNNNNNSNITINNNNNNNNNSSNITINNNISNLCINNNNHTSTSNTTNSSDNSLPILCQEYEHIDQQDYLIYISKKKKKKKKLKKKKIYYKQRIINFTDIPEHFLKKIYFDDKECFYINFKKSSKGHNDTYTYQKQHSQEIKENYSLQNNKKKKSKEEIYGDTCTYTTSESNKTIIYLENSKKCNSVDNKEIKNKNISKDDMHYYREQKKKIIKNKSENNTCDDDLECFNNPDNDRNDPNEEMKKKKKKIKINNNNNNIESEATIKNINIIKTKKNSLKILHSRSKKHSVLKIKKKNKNNIIKNNNINIKEYLKYVNQNITKSQIYEKDKKYICMSDNGWGCMIRVIQMVLANILIHFNISNRYVYFHNVNDYILYKNYINKLTTCTNKENKIIQIEEKTMNKDKEESIYLNYKNKDSTKEMVKHEKNNQCESNECNLNKNASQNNKESDTLMIHPQSNIINGNNNNINSNNNNNNNNNNSNNNNNSNNNNNNSSSSTTFSNSNTYLVPDSFKQEYNKSSIKTDFLQSDKQITLNDTNINESQGSDIYNIDNVENITNKYNSSDLHHCENYNNFLLNNKNNNNDSNSEIMHINNSLIYSILSEFRDLEQAKYSIQNIIYEMIKYKKIDDNQIEHFVHDWLGPTSSAIIISNLINKKKVRFVKKNKMKNNFRGTNIHMDKNIYIEKEQKELTNYTNNNKQIYKLLNQKENINISKSDDNIKNDKKKYNKLLFLKEKKKKKYTFFSVAFETGVIYNNKVLKFFQIKQDIFIIIWICLKLGIDSLNVSKYKKSLLSCFLLKQFQGISSGNTNTSAHYFYSANDNGLFYLDPHIKCQNAFIDFNENISSQFFMHKVKFLPWEYLNSSLSLIFVVQSKDDYFNLIQNLKLIDSSLFEIYHEEPQYVYKNELNYDTDDSGLVVL

>1082

MKQEPLGKPEYEYFYDESNVDDDFDDTEDAIYTTVLTNNNQRQQIPFIYSLGRRYHPVHEYDERKKYERSLFWWTYRYDFPEINCISSDAGWGCMLRSAQMMLSQALRIHFKSRHWKPDPSTSKARQDDFVASLMTWFADFPSKTESIYSLHNMCAAGLAKYEVLPGDWYGPGTACYVMRDLVALHQQRQSNVFRVHVSSEGTVYNDLVYELMTKDAAKKKRQSQQIGKDEEKTVAEPLHPLDPACITEDVELDLKDVEWDASLLLLIPHRLGRDSINETYVKSVVQSFGLPQSVGILGGRPRGARWFFGAYADGSKVLGLDPHTVQPAPSRRENIKTGGKERRESSIDLTDEYLASVHSPYPDVLDLNRMDPSIALGFYCRDRKEFLNLQEALTRRKQSSDSQLVSFVDKVPNYMSSSLVNDMIENDDDLGCFDGEFDNTGKDELSDEEDYVLL

>1083

MSKFEETENSIQNNEKDSEECQLENICEIETEDNSNNSQNHEQADGSKQPLSLQFSVPSLSSLTQPLNTMFTEAKHHICTFIGSNPSSMLGRKSHTFSTKEPVWIMGSQYDMTESPSDCDMKEDGTMNGMERVVRKLSTILWITYRRGFEVIEGSKYTSDAGWGCMVRTTQMMCAQGLILFYCRKQELTESTDGSYSSLFEHVLSCFNDTYGACFSLHNMLKHSKSFQKGVGTWFGPAEACVMAVKCLQDSFLSNQIAGLVASQSNGVIYKSEITKLCCNEGKWEKAIFIFVPLRLGLNELNLNYIPSVLRCLEMKHSIGIVGGKPQRSLYFVGYQHENLLYLDPHTVFDTNKDKSNIPYNCKNIYSLNVRELDPSMALGFFVRDKHDFELFWEECQCFIKKPFPIFTIQDEMPLLDLTWEEASTNDHDSESSEGSEWENL

>1084

KEESKQHANEDKDKKRVWKKSLLLFIPLRLGLEKLNLEYMSGILHCLEMKHSIGIIGGKPQRSLYFVGYQRDHLFYLDPHYVFEYPSSLSSSSSSFSGTSDIPFTCKTIQSLHISQLDPCMALGFFIRDQLDFDLFWNQCLLFSTLHSFPIFQVGEQMPTLPDLFPTLAHNTLSYPEDPSPHSQLHPTFEDSEWEIL

>1085

MFYYLLFKHSQYLESASIFFSKKKREIRQKKQDKRNFEQIFVLLGMSSFEVIEGPPANSDATLAEEEDLSPNQIWPSCSDSTIISSADVTAARTEEEKKEEEKKESEEEKSSKSLLAFPFSISMSMSSLSDWTQPLNTLFSEAKHRIYTLLSSNSGAMFACTNPHSFNSSDTVWIMGEEYKLQQQQQQSNEKKEEEGEEGEEGEEKEEEMDKVLKKLKSIIWITYRNNFEVIKGSKYTSDAGWGCMIRTTQMMFAQGLSRYWNNNDSTRNDNNKNENKEIIDKQILSYFNDSYDSSLSLHNMLKHGECLKKSTGTWFGPTEACVMMVKSLESSPLAKELTGLVAMHSNGVIYKSEIIKLCCDEQKVWKKAIFIFVPLRLGLDQLNANYIAGILGCLQFRHSIGFVGGKPQRSLYFVGHQNQHLFYLDPHTVFDCSVPHSVPTYCHLVQSLSIRELDPSMALGFFIRDQRDFDLFWSQCLEFTQIPFPIFTVGDHVPTFDDRWEDVSVNSHDHGSSDDSEWEKL

>1086

MSTIYTYDSPIQYTPQKKVTTDLRGTYESNSSLEKTSAATEQTRVESMKSEKGLFSFSMSMPSIPGWTQPWNTLLTEAKHQMYAFLGSNSPSLFACKNVHKFSSRDVVWIMGRAFHPSENAEEEEEREDEEKKEDEDENRSVSRSKSKSKSKSKSKSKSRSTSSIKTMWRIKSEQDQEKDKRNRNGKRTGTGTGQKGEHKREMKKWENDIIMRWHELVWITYRRGFEAIRGSKYTSDAGWGCMLRTTQMMCAQGLLCHYRNEDANENANANESEDKKDDDNDNDNDNENDNDNSNDNDNGDNINGNNMNGNDTNGNETNGNNINGNDKKQQTTAMIRLKKDEHKINPQTSSLYRYVCDFFKDSYDSLFSIHNMLRHKSKWNSKHVGSWLTPTETCLIRKFLICAVNILPMITMTM

>1087

MQRWLQLWKMDLVQKVSHGVFEGSSEEPAALMNHDYIVLGEVYPERDEESGAEQCEQDCRYRGEAVSDGFLSSLFGREISSYTKEFLLDVQSRVNFTYRTRFVPIARAPDGPSPLSLNLLVRTNPISTIEDYIANPDCFNTDIGWGCMIRTGQSLLGNALQILHLGRDFRVNGNESLERESKFVNWFNDTPEAPFSLHNFVSAGTELSDKRPGEWFGPAATARSIQSLIYGFPECGIDDCIVSVSSGDIYENEVEKVFAENPNSRILFLLGVKLGINAVNESYRESICGILSSTQSVGIAGGRPSSSLYFFGYQGNEFLHFDPHIPQPAVEDSFVESCHTSKFGKLQLSEMDPSMLIGILIKGEKDWQQWKLEVAESAIINVLAKRMDDFDVSCSMDDVESVSSNSMKKDASNNENLGVLEGDYVDIGAIFPHTTNTEDVDEYDCFQDIHCKKQKIVVMGNTHTVNANLTDYEVEGVLVEKETVGIHSPIDEKC

>1088

MSSSTTKSSAHILWFEEISIEDVPVVGGKNASLGEMYQALTPKGINIPNGYAITASAYFYFLDKAGIRNEIRTIMQGLDVTDTRNLEERGAKVRKLILSKDLPADLEAKITAAYDKLSEHVGVPNTDVAVRSSATAEDLPNASFAGQQETFLNVRGHKALLESCKACIASLFTNRAIAYRENNGFDHFEVGLSVCVQKMVRSDLASSGVIFSIDTESGFDKTVFVTGAWGLGENIVQGHVNPDEFYVFKPMLDEPGCRPIISRKLGAKQFRMIYATDSSKLSVKNIRTQQQDRLRFCISDDEVIQLAKWATEEMYACADVVATAQSVHIYIVCAQARVINDICPPQSARISRRTQTILSTRAARTCHAAIISRELGVPCVVGCQTATEQVPNGEMVTVDCSGGETGHVVRGERKFVVHETDITTIPKTKTKTMLILGNPDLAFDLSFTPAQGVGLVRMEFMIMNHIKVHPLALLDMDVLTPEERVEVKQLIQGYDDPSEFFVSHLAEGIASIAAAFHPHDVILRFSDFKVSAPISTLLVLVSTRTRQEEPHTLVFRWLKHMAEGLLSAFLGGVSEPGAITVLGRRYDATQESDDLKAHIRRLVWLTYRKGYDPIHGDAQLTSDTGWGCTYRSGQMLLAQALMSNAEPSARMQRLEGVRPSTWQHEETKRAVLSMFQDSHDPAAFFSIQHMAETSFVVRKKPGQWLSPSEVALIIRRLNPPETGMRVRIVNDTLLSTRRILAGEPWMPTLLMIPLRAGLDTLQPESVPAFVAFFDWPWCVGAIGGKPGSAYYYVGIDHDRRRVLYLDPHTTRSRLDLSNQAAEKTCVPDKLKSMDMSKSCSSICVGLFLPELRDLTELVQRYKREQLSGMWSTPLFHVVNDAGLSISEEPHMDGSVVETTFSASTDMRARGPPSVPRPRRRHVGTAQPGPSTASPASSSSSSASSSSAHARPNLARRFTVAAGPLSRRMPMADAELHFASHQQQAPSPRAPLRANHTRQPQPTTATPASTTATAPAAPTHPAPPAAPPYLAQQQEFTEEEDDEDGFVVIDYAPPES

>1089

MKQIHRMIDEIRKWMPFTPSSKVVCFGITYDCSEEAAREEFLEDFRSRILLTYRSGLEPPLQLAAGGTTASDSGWGCMLRVTQMMLAQCRDWRFGEEDLQEGQVFWCNNRCRHRNLLGRNEAPPICASPVASSIPRRHSFLCIALSKWGSVSWVRSLRHGLAPPQQHKQLDISSRRPRLALVVQTS

>1090

MQGLLNHSLSSESDGLAWVLGCPYNLPCEYSDLVDDVRSRIWVSYRSGYFPIRDHNGGCFTSDQGWGCMLRCGQMILAQTFLTRELGRG

>1091

GWRWQKSSDEDPSDTTNHSTDSNSNPNSDGTENGISVPSSPHTRSNAYSPPHKYKEPHILSPRSSSENERYRQVISCFRDAPTSPYSIHAIAYEGVALDKHIGQWFGPTTVAQAIRRLCEHEQLKPLHAHAHAAEEEPVSKPIPLQHTTSIGSDSIGIAIGGKEEGLTIATYIAMDGVVGKDDMDIALYSYIERKQKAHVAATAQRRAKKAKIVLQKDGQRGVSKACRSYNATEPRARSKSREAGEAVQNAKDMGSAGNRDEIYSCGESNEVKHGLRDDWDGLDVTDTDIHNMDTHQQSHAQADRAASKDISTHTEIHYDLKNPADGYSNRHTKAQTNTGLGSAKNRIEDRYKEDIDGEGGYVLAEGVRTPEADRDAGLVEGGLVPVGNVGKDYDVISLDKSLSVNRTLQGMSISEKTDEDTDSDECIDTPVHRHGREGETEDKSRTHEEEQEELDNTPLLLMIPLRLGLDYINTCYIHQLKKVLMMPSSTGFIGGKPNSAYYFIGYKGSDVIFIDPHLTQPTVATQANGRINTE

>1092

MSQSYHCSQINVLPFSSMDPSLCLGFLLNTKAERDGFFSTFELKQRLETTPLFNVVQMANPFAQTSGADTGDVVSGSGSCEDSNSDDFEII

>1093

MEPVKAETLPTTEASQHLKQSISEEQEHTLWEKVTEGISHFFHTASGRFRTHSTTAGANNIILAAHDDNTLLTRLTARTGIDLGLGNGRNRDSGPVRLLGIEYPSCADQGFQDDFKSRVWITYRNGYPPIKPSTYTSDVGWGCMLRSGQMMLANAFLFHELGRDWRLTRCQDDKGAWDKYVTILNRFLDTPSAPFSIHRIALLGKQFDKNIGEWFGPSTISHVLKVLLDDHKDIDMGIYVATDGVMYLDDINNECTKGHEGWRGTLILVPIRLGVEGLNPVYFDALKACFSLPQCVGIAGGRPNSSLFFIGVEGNHLVYLDPHFLRPAVEVKHPASYAPEDLSTYHCETVRTMSMEAVDPSLVLGFYCSSKQDLKSFCEQAVNISHGRTPLFTIQEKAPEFQDRDGDVLSDGDDF

>1094

MESEINICILGSMDDFVVINQSTPNALLSEMLNSLYSREDSYNTGQSDRALIAKWKTNARYQMSEVPSVPIFGYKQVDLSGSFENVSLYSKNFNNAEEFLEAFQSVIWITYRYGFEPIIYKDKIFTSDTGWGCTIRVGQMLLLNTLKFHFRLSSSSQLLELIQENLTAAPFSLHKIVECGKNFNKNPGDWYSPALISHVIQALSETFEIPGMKILVCMDNTIYKNKIPENEACLILIPFMLGIENIQPEYYEVIKSFLSLEWTVGIIGGIPKSALYIVGFQGDNLLFLDPHFVQLACTSGQDLLNKINTYQCSSPKLLPLSVAESSLSVGFYFRNKSEFYNFENFVSKSSGALREILNFKDKDIDSNIQEDNSDEDFYLV

>1095

MKNSQEGYFLDLTGDASCSIQIALFDMDNEKHKKKEDFISNFKVSKVFISKYGTNIRYFLNSKLSPLNEANFELNTVLKGTKYDNSDYWIFIHEFSHIIWVTYRENFRSVSSTKLLNSDVGWGCTIRVGQMLLMSLLKRHLSNPDYDLILKIQENLESAPYSLHKITELGLEYDKKPGDWYSPSTMGYILTLLLENSPIAKLKCFISMNCALYRDQILSLAYNFSVDMIKSVCKCIPNEYEDLGIMCGGCGKRIGIAKWENAIFIQVPIMLGMRGILPEYIDTLKSILDQPSCVGIIGGKPRMALFIVGYTSDSLIVLDPHLVQPAARSEIEFKQLLSSYYCRSPMLIPFNQIEGSFNVGFYIENEEMWNKLERFLERDSRLKGVITIRDKEFSEDSEIMEIIHSGGMF

>1096

MNNSQEGFFFDLTGDGSSSIQVAMFDMDNEKHKKKEDFISGFKVSKLFISKYGTNIRYFLNSKLNPLNEVNFETNTVLKGTKYSISDYWIFIHEFSHIIWVTYRENFRSVSSTKLLNSDVGWGCTIRAGQMLLMSLLKRHLSNPDYDLILKIQENLESAPYSLHKIAKLGLEYDKKPGDWYSPSTMGYILTLLLENSPVPKLKCFISMNCALYRDQILSLAYNFSADLVKSFCRCIPNEYDDLGIMCGGCGKRIGIAKWENAIFIQVPIMLGMRGILPEYIDTLKSILDQPSCVGIIGGKPRMALFIVGYTSDSLIVLDPHLVQPAAKSEMEFKQLLSSYYCRSPMLIPFNQIEGSFNVGFYIENEDMWNGLERFLERDSRLKGIITIRDKEFSDDSEIMEILNSGVYFR

>1097

MESNWLKKFTNYVRYNIKSSRIEELFEYGTDNPIVLLGEIYETKIPGEKNNRPPVNYLGSDYIDIYSATGKTIHNLSAYNEFAEKFKSILWVTYRSNFLPILRTKKKQKYYSSDAGWGCTLRAGQMLLLNTLKKHYKKNKETSIELFHLVEESLSAPYSIHNLINLGATNKQAGDWFCSAEVCSAIVKLSEINPIPGFRCCLFTDFTIYKDQVFMLAMDETENPDRENNTESQEWKNGVLIFFPLMLGHKKLEGKFYDVLKLFLSLGPSVGIVGGRPRSALYIVGYNGDNVICLDPHIVKQLNNKLQFKLPVEDFFCSDLILTKFKELESSMTFGFYFRTAQEFYMFEKNVMANDGLLNGLMLFKDCFVDVPETQELVLENDFILM

>1098

MNLHNTPKLEGFIIIIPDFQASYYKNHHNGNLWLKKIARNFTFNLKYCFNVSLKRLYMINENSVYLMGKEYKCSPFIKKVYKELKKNIKEIVWVTYRAGIREFESENGRVYNSDSGWGCTIRVGQMLLINCLKIHYKMGNETISELLKTIEDNMENAPFSLRKMLSTDITKGPGEWFSPSEVSHMIVSVLSNHPLNDFKALVFMDSLILKDRIYSEACNISFEDIRKYCTCSIISSSESHLCETCNKPKGSFKWVNSLLIMLPLMLGIRKIQQNYVESLKKILQNKYSVGIIGGKPNSALYLAGIKNDSVIVFDPHYVQDASSSLSDFKRTLSTYFNPSLNTVEIENLESSITIGFYLRNEEDFADFSDQVVNEKGLNNIIFIKNETPDYMLDSFSAQDSMIIEFD

>1099

MDIFNKESNSHSDSLSKSDISLDITPKSEEFITIIPDFQANYYNNPHSGSIWLKKITRNLTFNLKYCFNLSLKRLYMITQNSVYLLGKEYKCKPPMKKIYKELKKNIKEIVWVTYRTGIREFEGEKGKVYNSDSGWGCTIRVGQMLLINCLKIHYKIENETISELLKTIEDNMENSPFSLRKLLSTDITKGPGEWFSPSEVSHMIVSVLSNHPLNNFKALVFMDSLILKDRLYSEACNISFEDIRKCCICSGISTSYSYICEVCNKPQLSFKWVNSLLIMLPLMLGVRKIQSNYIDSLKKIFQNKYSVGIIGGKLKSALYLAGIKNNSVIVFDPHYVQDASSSLSDFKRTLDTYFNPSLNTVEIENLESSMTVGFYFRNEEDFIDFSNQVVNEKELNNIIFIKNQTPDYLLDSFSAPDSMIIEFD

>1100

MERRIQSKKPFQSNVKRENTNGSYQVESYDFEANEEDEGNINNFQIYHALSKQITDGGQIMPQYNSRGSDDRTESPASNQVNKVRASDTNLAQVQRHQIDNQVKRLSQQPNPIIHAQEQPISISQAEYEEDEQFFDQNIERQKKRSLTDDKKFLSQPNSKDPQDQKQQKDLISQIQSKGGNSTDSTYTKINKSNQNNNKKVRIQSNQYSDFNSEDYQEKTEPITIGLMNPSKDFMVHDFYDQEFKQLKFNQDIKHQIIRKDSASKQQRNKAQKLNQSQNSGSKDGSYLQYWVNKGINYKYKLPWTSLAKVFTEFHPVSFDQKDLPLTLLDQSWVQLEQADPRQKKRRGSDELRLIKIEFEKKFSQIVLCTYRKGFNPLLSESEKLRNMVMAHAKLGESKIFTSTDVGWGCAIREDDPIIREKFRSIIRLILDNDGTKQAAFSIQNIAKMGFCHDKYPSEWYGHHAMSIMMRDLNKMYQPVHDFQICIYRDGNIYYDKLKKLAITDGQKFQIQSFQAKRYQGEARDTRCRQDIVFHEFLNQTGYFSGITLSHSKHHNMSKEFDSPKRESQISDNDMMEKEQLKIIRNEKLLEKAWRNGILIIIPTRLGLNKVNKEYFYAIQYILQFPLNVGILGGRPQQALYFDAHVGHHLDQNVMQTYQCDQAKKISLTKIDTSLAFGFFIKDFQDYQSFANFIETGKKVYKENWVFSIFKEKPSFDNFTPSMNDNFYLPKTTLGFEQISNRISKPLQSPQSNNQQIQQKQEKQDFLTINALPLNRNRSTNVQDINPIKIQPEGDEEFELI

>1101

MIELDKSQLVEPESNQHFKFISIPSDLTLSITERRKSSQNYLDDDEEDVKKQCEVINQQNEKLSKIQDSFECIEYTTEPGIINNVMLSISNTTKFSFLNKIDSFNQSVVEKLPPNCNNQNLYQMTNSSLITGQLTFQSRGLRENLMQKLTNLRYRFNDGDLIEYMAYPKVSFGDSSAFDKTAFLFNSKYNSKKSFNSSFKSIIWVSYRNNFPQLTRNISIGSKNYLQPSLNDKNQIEQKFLTSDCGWGCMIRCQQMMLANALVRLDSSYHKQFSMNNDEILELFLDIPEKYFSIHQITEEGKISMDKQPGDWYGVNSITQVIKNIFDQKSTLRPDCPQVFKKISFMVFQEGAIFMNEIKQELLLYLDEYNRAKTEELNYNKKQKFQQHSKTDQHKKFMEKRKLSSQFEMIDDNLDDVIVQEEDSFQLVGIESTSQSNTLSLNEQFSNKSDEKSLYTHDSDYPKAALVVITMRLGLNKIEEEYFDTILECFSLPQCVGILGGKPSFALYFVGHQDKQLIFLDPHYVQDSLKTKQDLQNDDLRQTYYPSGQAKKIKMDGLDPCIGVGFLIRNSEDLRTFEEAFSKTGKLSKIATLYQDRPKDQCLSRKLDEINFSSDYIDHSLL

>1102

MESSFAEIVRPAGYSDFEVIRRDDLARLMGSVLALDEMQDQDENPNLYLLNHRSIVENRKSLQSSDVEDQYSVVSNGDEEIQDFHKNHTLKIDVTIKQKLFEKLTNFGYSVQKSSSQNKVQINDKSLTYLLGFTVDKQELFDKFVADMIWMTYKKDFKPLLIEKTSIQGQKVQNLTTDCNWGCVIRSGQMLVANALVQSQFFYQVPQILELQFSICSFNDGIITMDKLLEKGCQIQKEDVAIVQNLYMSFDENVTRFSDGYSLSQNFKEPEWINEVLVIVNARLGMEGINSNYHQVILKYMKFPQFIGLMGGKPKKAYYFIGQQEYKNGDPAKLIYLDPHVVQKYSKNISKNYQQEKAKYHTTNARLIKIKNLDPCLGFGYLIKNYADYKQFYELLRKYIEQDKENSLFTVFEKFDYIDDDRRYTHNTASL

>1103

MQDKDSSFDIIELQNIQADAFKDAQCSNNQSYQDKLKSNSASKKHYQYLSQTSYTEEKNNLNPRNAVHFQEFKKEIKQMSYSERIKAFFSNATYNMKTFSKSSWKAVKINSKHPYARLFEKDFYYDDRNPEQENNNFLKYHERVIWFSYRNNFPLIRDVADDNQSVSNDYGWGCMIRCSQMLLAEALKRHYLNDQNIQIEQLSQDDEKHFYSNIIKLFLDCTSESDVLNQPGSYQDIQSKMLLNEQNLNNIYSLFGIQNICQSAILRQYQQNVKNWYTSIQVSVILQEILEESQSKLNSKLGFHILNFTDQIIFLKELEEASRKQNDRLNNILVMVHLKFGINKFEMQHKDYFIELLKIKNFVGALSGTETKGMYIIGFQEDRLIVLDPHFIQKSTEGEQGLDKDYCTYFNKTPRSISLECLSSDISLGYFIQVNEEQSINQFIDQILTLNEKHKEPLLSILNDRIETDEMEIEEHQINKEVKDQENQDSVNNISQNEEGEEQKQSSEHNQNIDQINDIYKKEIKEDKNRRVINKNIDKQSIISNESNFSHLNSNNNNNHSNINSSNINYRIQDFYISNGSAISESSNSNLDKNQSYNEQEQMSLAIPCIKKLNMSSQLDNLKIQNKDYSDSNKTIQKNVVYNDFEVIS

>1104

MEEDSRLEEQKREQTFMEKMHDLGTNFLFNVSSWSFKSSAPFKKEDTVYMQGRNMNENKETYEKNYKEVLENFYNIIWITYRKNFPALLNMIDKANLKNQKMSEYISDTGWGCMVRVGQMAFAEGLRRHLVENKKLVVKKKEDLRVIIEGFLDDDQKCIDFAPYSIQKISKIALSDFNLLPGEWYTPIRICYILGLLHNERKAIKGTEDLKVAVFSSSRPIVFQDFLERMCKVDPQRGKHAQICPNQCRIIKQDQKSKVDHDHHKDIKLEKQNSNSEILVVSEETPKLRLVCPIHHELQYSMIVYIVCLIGLDTPQPEYLELAKKMMDFKYSLGLIGGKPKKALYFVGRIEDEFIYLDPHYVQEFSNEKNFQSSSQLETYFCKKFQTYPSKNIDSSFSLMYYLKDLEQLEEFYQFMMGLKRDYNEHFFMMMEDTEPSFCLGDGKESSNLISDKNLNILADNQNKKRQKEQMQQANQNFEEVDDIDENGNNIKNSKYDHKNKKDKVEDDGDDEYEVI

>1105

MEDSKFILLGQTLSSLSQIKEAQHNLIYFSYRSGFSHQFQNHIFSDSGWGCMLRSGQMIFANGLLRHLKENPQIQNQLKIQNINDILLFIIKFFIENKDQPFSIQQIAAVALEEFKLEMGFWYSPNRIAYSLKKLLNNFQTFSEMNIVSEVMYSDRPLYFSQCVTAMTGQKIDSTLPNNILVDANKNYEKKQSLILLIICQIGLDYPEEKYLDILIKLFTHRLSIGMIGGKHSSGYYFTGLNNDKLTYLDPHIVQHADINTNEINLKTYFQEEVKQINKHALGPSVGLGFYLKDLNDLNEFWGYLVELQTKHDDQFFLMIEKDQNTNYQEQELVEDEDFIEID

>1106

MSTTQNCIQLVNEDSIFSTSKKFTLSSTERPMCQILGKIFYCGEKTNELLQEELSSLVFLSYKKNMKEFQYLSTTITTDNGWGCSLRTSQMMLAQGLKRHLYEKRVQSFIYNDKTKLDFQHLIMMFAESNSLENMDQSPFGFHSLLTQAINLFQVPLKQQYTPVQGIKALKQQFKQQKLVKSLKIVTSSTGVIFQEDIRQKMKNWEKSLLLILHFKLGTGKLNQIYVEQIKSLMDLEYFVGAIGGIKNKSLFMVGYMNDQFLSLDPHVQQVNNLKLIHTNNYTKLTFIIRMHAKIH

>1107

MVLIAVSYLLCRSTQIYRQLLEFRDIYILCKTWIIYKIQFNKSTNSVALANILEMSQSKEIDYFKSIKLSMSKAFSEDGRRVSTNNITEGIMSYGMQQIYRFNKWGTSCKTIDLKVDIPSFILGNDLTPQRVKLVQDKSRVLQDDGNQNKYRMLMLSNSSDIRVDTEPKQEEIISKETYQTQETSGQDYDDEDYQETEKQMKEKSKQILQIYQNTILFTYRKNFYPLLKDKINDPQKNQTSDAGWGCMIRAGQMIFAQTIKRHLKKTDYIEQHQLINIIIGFLEEEEVQEGGKGYIFNQQSYIQDRIRPYSIHQITNRAFCKYKIQPGQWYTPNQIAIILKELHKKNKIKGTENLKIDVHSSDKPIIFEKILQTLLGRQGKINLNCNHENQQSRNSINQDQDDSFEKIMPPNQQEIEEFSSQYEESKEDQTDNLCCKDCFKTDNKLFLLLPCRLGLDEISPIHIEILKKLLSLKQSVGMIGGKPNKAHYFLGFVGDDLLYLDPHYIKECVRKEDLMENISSYFEEDVFKMPINKISTSLVFGFYFSGVDELNKFYKFLRQLEKEYKDDFFLVIEKQTPSYAMSNISEIKKLTKQIKITQKDNQNLLEQQFENYDGSVGDDFDEDDDTSYDEQECYEDKLDEFDQYNNLEESDYSQKLKQQTNLKNYSDPFGTESHSENQLKYGKLANHELPIRNQIQKKQSEEELKNLVNLIQLQQQNQQISNLLQNQNCI

>1108

MGNSNDANGEQCSTTEGMLELDNLKFNAARNVNGHHQAGDDTYANDNDNGDYIDDGGDSSSDDEQISSSFHHAPLHQNSDSSVDQLRTYFLGHVFNPNTDQLSMLSFQKQLYWLTYRSDLTVPLRPYNGGVGLKSDAGWGCMLRSAQMMMAQTVRMHYEGSSGEGCCGSGGVKHQSEGSRGSAVGGKGVEVERIACWFADFPNHFDINDDEEDEATDDVSNQQQQRRLLGYRYGDGGLHHHWYSLHQMVAAGLGLGVLPGEWYGPTTACHVLRELNEIHCGCRERVAEVLKRRRKGGDKGDIDEHNHVGDDSQYTCDVFRVHIATEGCIYLDAISKLMTSSNQSLQTESNDAPIQHNTDSAANVIDHPLSLPEEVFDPLRAQVTTQSSDKEQILNQQWDTSLLLLLPLRLGIQSIPTPTYGSTLAKLLSFPQSVGMLGGTPRHALWFYGADEVDPPTFGDDGKALNGQECGGWYGLDPHTTQVAPRGTRTTKYGKDEVSSDDIELNNCQWQVQLNDAYLRSLHFTPTTTHANHQRSIPLSKLDPSCALGFYIRDHSDFVQFTNAIDALSKEHCRPNKLPDIVTVTEKTPNYEVDVGSVMKTLMLCGKGTTKEREGGRGDETCNDDCGGGSDNQLDGFSMDSEEGVLSEEDDDDDYVLI

>1109

MLRSGQMLLANVLVGHLLGPSWRLASPGQGRKASSRDTRLYRRILSWFADVDRPSAPYAIHHMAQLGALRHDISLGCWFGPHTTALVIAELVGLHTPRLAVYVASDSTLFRDRIRDASTPHRRPRYPFEEFDAEVADGVTIQTKRVRRVALPPDALDVRPLRSRPPLTRSAEPTRSRTVRVVPLDRSTLGLGAAWDVLGVPMVEPWDELPSRSSGTQAAASCSDNDAPSEAAGFLPLLILVPLRLGIDAVNPLYYPSLQALLAFPQSRGMMGGRPNSAFYFVASRASTRCTWIHT

>1110

MMKLVSAVVAKSNKAAALYNKMTAAKGVALDPDSPYVLLGRRYPPPAEPQPHSPPLLRVICSTPWVTYRRGFAPIEPSPQSSDVGWGCMLRVGQMALAQALVRLLLGDDWVIPPETFAARLTAARVLDDAELEAIAASAGPASLAALPDAYYDILSWFADVPSPATPYAIHHFAWLGCRLGLNIGEWFAPTTIGNCMRYLVNAHAPGNLAMVVAYDATVYLRRLVASCTAPVGESDPLDATMWLVAPGSSRASDGVPDLPHVDPAGVSSDTAGIVRATSTTPPISPAVSCEALDQAAAAGPSSLYPEMPTLSPGLDYVARSSGSAAGLPTTADNWRPLLLLVPVRLGLEQLSEVYHEPLKAVFEFPQTVGVLGGKPKGALFFVGVQGDNLIYLDPHSVQPVAHVEHGNPQSAFSYQCLTPRSMPLSKIDPSMALCFLCRDLNSLHDLLARLDALNATAGVSMFSVVPGDAPGYPPAACSSAPPPSQSLSGGNTSWEDDDFEFL

>1111

MLVSKYLASSMLGNLPSRSISKHAEETRKAAAQNSNGRRTTQPLGASETNPTPSTDNSGQVESCEPFDLRANPPDVSTEVSPQQMTLSTTCDQAPPEGGAPEEGFLRSTPCDGGRTRRSIDGDLTDLTAEETFENQVCIRKLPPHQELATVEADFSALNLLASSQLSPIPSVAFLDLFQKEGNQPAVNSDCYELGTETNCAHPHTCAGAATSDKRKLALHCFSDKTSLLCGSDLRLEKAKAALQLPMATACLGAGRVCPPSRDHESTGQMEAASRPASGPQCFTAAGTEGNRFQKELSPAEGCNNAACIRGVSMGQRENEQGPNSNPGECLQSNKPPCSAAPDSSSETCTLEGDVRTGHTGVCTPDVSGKEGDDGSPPEWPLHVQADTGDFPGAEGESSGTHPLMGASVLADELTGLNDVSGHVDGPCLSSPLAPRSDSPVEEALRLPSRDRSPFPTVSTSFSSFEKLPSLTGSSQPDGRDAVGLDGFRISNETREDACRLLNYDENTRHPPATDPPSGSSFWSLGSPQASRRSTPVSGASTPQARSMSSSASPVLVSPLYGAAASPLLTASSAPVSSWALASVRLPPPLASFASFSRLRNSASRAHAETGHACLPAPPVPPTHSEERMSEDSVYSTSSVSSTSSSVSSASTSVLSTSSSVSFTSSSVSSTSSCTSCVSSPPSEASCGNSISTAPSSVVARPHRCTHQRPKGSVAVPACSPYSEDRREELKTSTSRPSHDPPPSLLPEAASELDHGKPSESLCLGTTASSILSSPNFFKAPGASLLSLRGPSSFFGHTLAGASFNATSDATTASACAPQSLAMSLDALEKRPSLEARAGGRASIAGNSNDPLQPKPTGSSLFRRVRSLKQLQQDVTVVFSTWTHTLANWAAPWGAGPEEGGSVKAGERAVVLGRLFWVCEEVDHAEEACDRVWKECERKSGLPNSPNKALTWLGRRAQRRGGATGGGSPTALGSTGTPIWNGTGPRSGALPPRRNSACAPSAAASKDRDGGWRRTSAALSGLLGGGGRRGEATESAIAGLHASESGASEFAHSSGPCVGAQYAACSAEKLHALQEPDDEPDTSLTALSMDRLGVAVAGRSNKRRRLFRLPISLPGGDPWPAGRVGCVSSDAAEVQHKLAETVRAIARFTYRSGFAPMYKCCGEKKRRVGPGFEREWIAINSDVGWGCTVRAAQMLLMQALRRHFLADDEGELETGKRGSRVKDEGEAKAMILRRFAEAGQATETQRETGSAGEHGRERDTEGDKRGGRGSSRKVSRGVQAAEAIDEEGGKKNTGEGLRCIREECMGRRSRKTGGEDTVSSSAARCEEQMKSLSSFRGDSRFSEPQETGTWISCDCGAAPPLGSLKNRLENIHLDNSCNAAGTDDSLPNRCRCMHTASGHLPRGRRLSTTRETLPSSDEVFASLSSPPQSRQTLDLSREGDSSHTREDARTSPQRHSASPSVMPRKPAPHASGEARSTSPSVQERPREMEDLLQWFLDVPSPPGQYPFSIFSFIRAAGGGIGWARQLYGELPLEEALFGAGRDKKTWRGPTSPSKPVTSCRGQTVSSGCKESSHLSGGSRDSQGSTFPSLSWPAASSSAASLPTCELPARNGEVDEAAAFAGVSRENEGPSRLASLPNAFARTSSLQGTRPRQEAAIGGATSTDSPLGDENAEASQNAESAPDLGRMHAATHAGHREEDENPMERRGLGKFAGEWFGPATASSAVKLLVEAMPQTKDELYVYVNSDGLLYPDEILLHCRNVDASSNLSALASVSSPGRGGTATPQCPEGHRGREAFHGRTPHASVTHASTHASVQSTPRGCASCSGSGGLFGVACDLSESLRGPQKGFACSDSEAVGTGRETRGPAELGEESASKREIPSDETCVHAGHTGCRASLRRRHSQERTSRGDLPSSIGGRAVEAQKAQKGRLWHVSNHGEPSASENLWRPAGGAAQTSGQPTGNWNNAHGASALEEEWEEIPFCGCASPHVTTRVSPFASQGDFCLPPRALAVGEKVRRGDPAATPDPGEPSKEPRPLLSLAPTQQPPRGPGEKVQGKRSPPSPSNCRASARAWGEETEGSHDLKRPPWNSGKGDSGGDTGGEESFEAAEEILRRLLLDEGAPRLRGDSEDSHGDTCTLPAGAGRPRRAPPSPRVLPEEGTPLLASADPTAYTILSSPRGPRPVHLLVSRGSWSVPSSPRRVRGEETGATCGDASGAEEPQRLVRRRRWVDRADQEHESCGSRDPSRATEQTEERTRSVPRRAGGRGGAARPRGRSAAGRPPGGKSGDPGKACGLHPGSGGGATEGAATQPQADEDGWRPDMCSSGVEAEKTDTEKKADSMRARDVGGEDFGASTRVGVLRTLESACRLDMKSEAKKERRLACVPRLMAATPGRSVSVQSGPGGEAEAVAAERVAKLPSSILGVDQSEDEEQEKQALVVSSRPSWSSPHLPRLPLFRSRSLSLSASLVSRGPQKEGRDTKHIPERRLRTPLEARRIVESPLLGSPAGPSAAAVADDVRAELSNVCVNGVHARREPVATPVSDDGCGARASSAAAEAQPLDDCEERHALLTGFASATLSPHTPDRTRRSGPLVPPFVPRLRALPLSLDALGRGNGGTPREGVRPSEGLCRVASCEKARDETGTRACNDRFGENACVEAFGDSFPDARECGFREGISPMCFTASPVLNSSRSEGSAAPSDGSATARWCSVETLLPGAGEDDILLASSGLVSHRNFPPSPACRCCRAGAASSTPRCIQFAGEERGDFGRKGTSPTNRKDERVDGRRGGEAEDEARQDADDGGEKRTEGWRKEQSAETTAAVAAGLQKSVTAGAHTFSFRGEVREAERNGGTPGPGRDDTHAADSGRDAQRLGTFVGEERDDGDSAWVSFLPFCPQRGIASPGNEKREALRQRREARRPLGEYVCVCSSSQRDSHRSSGVGGREAGRPSACAPIACDAHAFGPNECDPLAFGPNECSEQPSIEEGEETDGWRERRKERRGNSDGDRETDATGAKAKAVHTCVKPEARNRWRDLHAEAYEKAATVPAAEATSVRGGEEFRAPSLVDEKDTDAKDEEAEAPSVALAASADASGASRKTRNREEDSGETGETPDEQIDETPDGAAVDCLRDDSCADVPWRRGCLLLFPLTLCSGEKINPVYVHSLLAYLELPWSLGMVAGRGQQAFYCIGTQQKALLYLDPHSGIQPPALQLPAATPSFFAGSCWKVSDVAALNPSLAVAFFVRNERQLLGLAAALKKLEEVDSFSMLQVVERRRPFSPLDLDDVLQMEEVEEGLYFGESQGAQDSSPQAAPHRSDELEARQRSAHAQQGSTAKVGEATRGARGEFVKTGGTRTEGQAKNDLEDATVGGRDDDVDPKDDPVDDGEEAETPGDAGESGDEEKCPTQATPASSLPRRGCGEGPQAFAASSSLSAFAFSSSSRRSFSARSCVDTSVAESRAQPPTATEEAAGVHREIDEEDAALGREKTRDQSVEGAAETPGSGRVRGESEQQRLVALSELSPFRDDSRLAVGVNSGDARDSGDGDQQREEERNAGGEACVNGRERKGGALVCSCDMVEEKNGERDESPRAQLCIDVSQAALDANSEHSRDREEETIVTFCYGEFDQGQGVERMWGTEAEVEGTNDEEETGEEKAKERQTETRQAIGEERQETREEKEEERQKATGEEKEEEKEEEMEVEGRGTGRSKEGTRANGDRNDVEVRLIQEECDEGESEISESFDCRGGEATRARDGAYVAAEVESGTDR

>1112

MTVILGTTIIQSDTEKLKKVVDTIPRFTYHKGFSPLAGGYTTDKNWGCCIRSGQGLLMQFVSKLYQLYGDKIKNIFPNGSKFELFFDHPQAPFGIHCICRELETFGVKAGEWVKPSMLAPVFKDLLSFFGIHVVIAENGCLSRESLREALSYGHPVLLLFTLMLGYKDFDLKYLPFLRLTLSLIYQSVGVVGGQQGKAYYLVGHQKENLLYFDPHEVYDSVTKLDNMNLLFKAQLKKMQSSQLSSSMLVGFYITSMQDAEELPMLLSASGECPIQIVDKIDPVKTTHVEDDWEVVNN

>1113

MTVILGTTIIISDTEKQRKLLETIPRFTYHKNFAPLQGGFTTDKNWGCCIRSAQGLIMQFITKLYKHLGDDIRNIFPTNSKYELFYDLPHSPFGLPHICAELQSYGVMPGEWVKPSLLAPVIKEIMNFFRIPVVIAEHGCLSREVLNEALSHNIPVLLLFTLMLGYENFELKYLPFLKLTLSLIYQSVGVVGGQQGKAYFIVGHQKEKLLYFDPHDVNESITKIDQINQLFKPPLKVMPADTLSSSMLVGFFITNLQDAEELPMLLNQSGECPIHIVDKIEEAKETHTVEGDWDIVST

>1114

MKVINSDDTNVDANQILAEIPRFCYRNNFQAIENSTLSCDSGWGCCFRSSQGLVCQYILRLHKNFPDLYNSTFGIDKNPLDLFLDIPEAPFGIQNIVTHANSLGLPIGNWAKPSIIASAYKSIFQSLHLNCIVPQDSTFIYEELESTNYPVLILIPGLFGLEKIEKPYISFIFLSLCMNSSLGFVSGHNDSAFYFIGFDSDYFYYFDPHVTKQALTGPPYDSLFELKLKSMKIENINPSVLLGFYCDDSIQELIMQLMGCIQSPIAVIEKSKLDDILQDVIEFE

>1115

MLKKISEACGLSSVPLTKGDIKKYENIPRFTYRCNFQAIQPGNITSDSGWGCCYRSAQGLIASYFLNYAPVDAEYFFTVFNEIPMFSLFEDRVEMPFSIQNLVYRSELFGVKPGTWAKPSQLAATIESIFKDLKLSVLISKDSNIIPEDVKTMRAPFLLLIPILLGMKDVEQKFIPFIKYTFQRPEFLGAVSGSSDFSYFLVGLSEDQNVVYFDPHVTKQAVASSFDHSEFFEVPPRGIKMKSLNPSFLLGFFCSSTENAISLIEDITKLKDSPITFSEIRKDLVDQVLDIDDIDL

>1116

MKVISTNGEDREIKDVIADIPRFCYRYNLSDLANSLLTTDKGWGCCFRSTQGLLCQYILKLHRKFRSLYDQVFGQNVNPLDLFLDIPSAPFGIQNLTKNAFAIGLPVGEWAKPSIMAATIKLIFDTLNLSCIISQDLTLDSNDIKHTKYPALILIPSLFGLSKMDDSYLSFLLLCLCIESSLGFVSGQNASAYYFVGFDLEDFYYFDPHVTKEAVVSPPYDSFFDLELKSMKKESINPSVLLGFYCDGSIDDLIMQLTGCIKSPISVIERENLDDILNSVIEIE

>1117

MISYLKSFWGTSKEDHTDEYKERPRFTYRSDFAAIPNTNIISDIGWGCCYRCTQGIIAQYLKIIDEINHSLILTRFSNIKNDDIISLFEDTLEAPFSIQNLVSETSKLGVAPGEWAKPSQIAIAFSNILDNFNLHNYTTLNCLIEPSKIQDDSKYPMILMVSLMCGLQQLDELHFPFLKYVFSLPETIGIVSGYSGSAYYVVKIDDSENVFYFDPHVVQPAVATPDLYETFYNQKIMMMSLSQMNPSILLCFVCRDRESTEKLVTNLSEFPGSPVSLSECVSDEILEKVLDIDDLDLS

>1118

MYLKNLAEFVMRKRSDAKTILGQPCRDANEVVAVIGMIPRFTYRRGFKALKNEQHYFPIKSDTHWGCCIRCGQSMLAQYFQKCILTFPEEYNRHFSNTSYLDHFNDTPDSPFSIHNFCKEIINLGGHEGKWVSPSKLSFAIQNLLKPFEYPVYVCQNSTIVKDEIRPLFTGDKMVLCLIPLKCGMQTFDMKCFPIVGLSVTLRSGIGFIGGVKYKAFYYVGVSKNSFFYFDPHITQTKVDECRDHLSYFVPKLKSMQIEKISPSLVLCFAWENEEEMEENLYIFNNMEHLPFSIIETKVNLDFHDAEGVFNADDSWNIVQTETPQNQETSQNQETSRNNDMSQENDEVQLNEIHNSEINSSWEIVSSYANPSNIHKISDFKIYQSNSSDISIEEDSGFDKVVYTSHLSANSSNEIEKFETYSNGNDSNDNLPKASDDH

>1119

MSNFFRILFTKPDQLTEPYRKIPRFTYRTNFEEIPNTKISSDSGWGCCYRCCQSIAGNYLRILMSLDPKSILENDKLPKEENILLFFKDCLSAPFSLQNLVQETIQFNDKPGTWAQPSHVASAMKNLFQKHSLSCEVNLNVPVDFNLIDNLRFPCLYMFSLRLGLDKFEYKDYSEFLKAVFTFDETIGLISGRSGSAFYIVKIDDNGDVFYFDPHVTQSAMVESGHVLTLFNQQIMKMKMKNLNQSILLCFACKNNEETKTMFLKLSKLPNSPIAIGETVFDVEI

>1120

MTTILGVECNDQDDQLALISTIPRFTYRKGFRALQNENHSFTITSDTHWGCCIRCGQSLVAQYVKKLLMKFPKYYNSHFPKRESFLSLFNDTPDAPFSIHNICKEVIKNDGHEGEWVSVSKLSKSLENLMKPDFPIYVCENSTIVIEEIKPLFTGESSVLCLIPLMCGFKKFDSRCFQTVVLSVTLPEGIGFISGHKGKAHYFVGVSSKFFYFFDPHTTKKYVQQGSDQAILFDPKLKSMKINDISPSLLLGFAWDSLDEMMSTIECMRKLKFCPISIVDVKSETVISANDDWDIVEEGEVIPSFEASFQEVSLLSGTMRLRKLSDIKNEIKNMKSSNESESEEPMGLAFETDFNKSASIPSEEK

>1121

MRVVGCRSTLSSASDPKKKFEHVPRFCYRKCFSPILNTKITSDKGWGCCFRCTQGIIGQFIYRLHNQYKTDYDRVFGQDCDPSSLFFDTPEAPFSIHNLVKNSIEFGLEPGTWAKPSIAAAAIQRIFGELHLGCVLCHDFCITKSEMDISFPAIFLVPGLFGLDSLDLSFIPFLHAAICIQGSLGFVSGKRNSAYYIAGLDSDKFIYFDPHTTMPALLRVEDKSSLFSIKPRTIRFRAINPSILIGFMCTSTSDLDNVIETMMSINSSPIDVNEPSDAAMAQVLDIDDLDLDGE

>1122

MERLKNFVHSVVGCDAHIEYPCCVLGTIQREPQQLDEHLENSFYLFTYRRYFDPLPYSTLTSDKGWGCLARATQMLLACSLRRHSAQDCKLQYFADLDDEQVAPFSLHCMVRHILKQGESLRPVYWAPSQGCEAISGCVKRATERGILSSPLSVVITVAGAVPAEEVSCHLKESRNVLILAPLRCGASRCMSQKMFLSLEHLLLAPESVGMVGGVPNRGYYIIGTGAQELLLYLDPHCKTQDALLSGEPGETGVVKPTSSNLRSVPYGQVDTSFFLGFFVDSQSRWESLQKRIEGLSKQKLHPIVSVYRGGPHTPVDLLVMEWPTEP

>1123

MIGFVYNIVCMVRCRSRDVVEEEDAYVVGSGTYCGGGTAEMVKLAACKLLYFSYRCQFEPLRNGSTTDIGWGCTIRAGQMMLAHALMRYKNGGGASFEDSIVPSLKQATQHLFHDDPSAPFGIHAITNKGVQHGAPCGSWFGPTHVAVVMGALMEDYLSSGGQGPDVLVLRDRQVMEDEVRKILLLSKHVLLLIPVMLGPHHISEGYAKLLKRCLRMESTVGAVGGKEGSAFFFMGYQGGNLIVLDPHYAQSAFTCSDTQGKISGEWYTLPLTSCSTSVLLGFYIHSPDSFSQFTGDIKDANSSLIFPLIEVTTSDCVGHIFSEDDPDVCSLVSFGDEESSGASQPVT

>1124

MEWLKIALVAFSGRGNDVDYPCSILGRVANNDKELVNILRNGFFLLTYRMNFSPLPHSSVTSDKGWGCLVRSSQMLLAHALWRYSANDCRLDHFRDMDTEDSTPFSLHKMVRAVMKKADVFRPEYWTPSQGCEAIRCCVNNAVDRKLIPPIRVVVCSQGCLLAREICSNLEFGTVLILAPMRCGASRRMTQMMFFSLEHLLHSSACIGVVGGVPQRSYYILGTSGQRLLYLDPHCMTQEALVSSHAEKAGVVTVTASLVKSVRWDCVDTSCFLGFLVDSFAEWLELRTCLEELQRRGMEQLLCVDDGVAVGLVDEEIAGWPSEEDVAE

>1125

MQGTMTEAADDREAGAAPFCDSADAIKAVYNCMFRLRAHKLLLADGVETYIIGSGMYNGAETMKWADKATEALLYFSYRNRIVPLMNGATTDLFWGCMIRTGQMMLAHAFMRYFNGGGPHIGSERLQELRARTQTLFCDVPSAPFGIHAVTSEGTKHGVNCGEWFGPTPIAKTLSALMASYLAAGGEGPVVLAFPERQIFLEEVKELLRQSTHVVLLIPVMLGIRVISEKYSQLMKRCLEMESSIGILGGKSRSALFLFGHQDDDVFFLDPHCVQPAFTSSGSPGELTCARRVLPTTSYDTSMTLGFYISSLDSLALFERDMASANDGLTFPLISLLPGRPASGTAVHLNLEDTFSS

>1126

MEWLKIALVAFSGRGNDVDYPCSILGRVANNDKELVNILRNGFFLLTYRMNFSPLPHSSVTSDKGWGCLVRSSQMLLAHALWRYSANDCRLDHFCDMDTEDSTPFSLHKMVRAVMKKADVFRPEYWTPSQGCEAIRCCVNNAVDRKLIPPIRVVVCSQGCLLAREICSNLEFGTVLILAPMRCGASRRMTQMMFFSLEHLLHSSACIGVVGGVPQRSYYILGTSGQRLLYLDPHCMTQEALVSGHAEKAGVVTVTASLVKSVRWDCVDTSCFLGFLVDSFAEWLELRTCLEELQRRGMEQLLCVDDGDAVGLVDEEIAGWPSEDDVAE

>1127

MTDSSSHAVDRHHSNLGLHNVAIPDSSLDPASSPDFPLAASGSTKQALPKKGFRVRVNSFGAAVMLNSKATSSSDPAPLAQQPKSSKFKMRARSATVNNLQMPTSHSVQADNFNPASSSCAPLSPTPHHHSPPPHSPQTRDPTLLSPAASKQSELSVTAESGNGFNRRKFFGRSNSIRSLLGGNSATSSNTTKAGSSHVSEPNPGTITTTTTTPTMGSGLLRRMSSKSFLNLNQPKTGAKAANNSSSDDLSSTTSVESHASRRFSDSLKGMRSRRGNSISKDTTKSRSLSSRSSSEARTARLTHKEILPPNSSRRSTDSTHSSLSVSSAEPHPPSSEADHSGNMAQPAREAQDSFTNAPRRLTGWLYNMVSSDNTPSSVEVGAPLSPVREADAIDFSPSSPTTHRQNPRILSHQTPAQSLAASSQVDASQPVSTTSSASTRSKAGALFQSLSNASPNRAVRMTNAATGDNNGSGSVPGTTSHTISSNHGGASSNSTSNATTGGANTGSWVPGGVNFDRAFKFFMDHDPSSKDDEGIWLLGVWHGPRQAGKVNSSEASVLPPDLSTSTSPCAPEMTASTTSNARNIDRPRASSPSSSLSASSPRQSPSELVHASLASLPPLHDRASIPTTRNESRVGSSISTAATASTSNSIRTKSSTTSFSVVNQASIPAAVDRQFAFQPDFASRIWCTYRNHFAPISRDGTISDQAASAAENMGVAQESVLQDTSASSTCSAVPSSSEATRSSSPPASTGRGWLGRKTAESNAAQDAALQANSPLGLGAALGAGYANASSTLGERMGITNLWSRATAAAQAAGFSRAGLTTDSGWGCMLRTGQSLLANALLNVHLGRSWLREAPPMRQMDFLEQLASLSLDSSVEMQSLQEWREKRARHAAYIKILSWFLDDPSPACPFGVHRMAREGKRLGKEVGEWFGPSTAAGAIKQLVTEFPDAGIAVELAHDGVFYLDEVRLAAGARSALQSGKGRQGDAAVTWRRPVVILIGIRLGLDSVNPIYYESVKETFSFPHSVGIAGGRPSSSYYFMGHQGNSLFYLDPHNVRPAVALRYPPSTFPTAVPHQLDVAHRFALEDKDDELEWWSHAYTEAQTSTFHCEKVRRMPIKSLDPSMLLGFLVKDEEDLMDLCTRIKGLPKTIFSFAESAPKWVDDDDFDPSMESISEPSVDEESEVDGEDKQDVQATHEGEPMQRTRASANAEDTSPASSKGTDARSSDKMFRESQQRTAAWLGQGQRLNSSREASHASSVSGGGIAFPSLDLLTPQSEGHTLQRPSRSDVRQVSTSSATTTRPARRALETVPAAGSKVGRELTVSQSTASPYTEQTPPWRSPGKLGTNESSSTVHLSDSEVGSGWEEVSDGGTIAPSGYAGASVLRTSSPFRVSLATKSPTTGVQGTADGGQVVDVAASDLIEGDLVSLDMNPPEEAQGGATHLLSHTSKSSATTDTVTASSASDSAATAQDESYFGLTSAHKPLALPRRRSQSKGSPIELATAVPTDDAPPVPVVPAAFLAELGQSKNKSNAAPDLNDSDDDF

>1128

MQPETGVASLVEHPAHIPAQPAQPDECVAPPPPPAPPIDPSPSPSPTTDPESSPVDTFEICDALESDSSAQADRQQQRDGPAEAEGEGVGGQESTSAATAAALREQFLRRRDLSRSYEDLGKPSALNESDSCGNMEFSSAAMWSSAIISSSIEEFEHLEPQPQPQPQPQPQPPAQQPQQPQQPPPPPPPPLPSPNDHSYSFLDDHEGCDNDHQQPAASDAPPAAAAESVGGRSGSGGSFVSGAGALRMLWNMQAGQWVGGTSAAASASGATEGNGEGEEGMGIGLLRSLTGSSNKPQGSSPRVVTQHSDLKHLLHRLNGAYREALAHALPHHLPKTGSQSGQVYLLGNCLSSDHLEERSFRRLFGDIEHFTYRTGLSPMLRCEVEPEHHMKVLTSDVGWGCAVRSGQMMIMQVLRRHLGCEPYPSDASASAAQPTPHHGKHEKVKKSLLEKFLDITDKQKAPLSIYSLLNIRSSDPSHFQSSSSSTSGSGSSRSRYRFPPWNPGCWFTTAQVCGMIVECLQRPPISASLLAHVADCGVLDDKALMDECMGTDDGTDGNGGWEAVGEPVDGDSDNGENGDGERGLVLLVPRVTGSSKIEEKFLPDLLKLVACRWSVGMLGGRARQAYYIVGRQGDSLFYLDPHGVKPAVTRVSEAREALYSAPPLKMHARELNPGLALAFYFKDVADFREFLEFLSFLDGSILRRASAQRPHASSSHSLPPSPCTDPSPVRCMSDMPPDDMEVLDLPSHRTHPTHAHDSQHPSISEWIDVAEPVAHTRPPGGGHHGGEGRKDGLWSWWKGVGVPDWSLTTVIW

>1129

MLACEYDLSSPESNKQFLDDFCSRTLFTYRDQFTPISQDQDMIEMDASPPPHGFSAVHREAGGILSDAGWGCMIRVTQMALVQCLIYHTLGRDWRFNTRADLTPGATFWQLIALFMDTPSAPFSLHNIVREGLKLGKRPSEWFGPTSGALAVKSLMDQHGAQSCGLRCVTFPEGIIYTNEVLEAFSGVPRKEAADKAAGASSSSGGPSAPAGPSSSPSECPTADEPQAAQQAKRESSGPAEDVSGVVIWLCLRLGVDSFNVDKYQSPIQACFSIPQFQGLAGGGPANSAYFFVAANRENLYFLDPHSKCQAAFTEIPSPTAPEQLAAFACQVHPSEPRQLAWSSLNPSMALGFVCQTVDQYEDLCARLKAVDADLFEILPTRPEYNYEGGLKTDEEDPDLVLL

>1130

MELLMAGLLKSSFWIQRSISRVGVIELSSYVLCGAPLRDGIAQQLGPAPDTGGVSLPAAGVLDGDDYDRRSCNHSSQTGSSGTDTSTDRDSRSRQQVPEELSGEMAPGQRVAKVTHGLPVNAVTELKKGSAEGSTGCDGPDISPARRSGSACGSREAPRTSGGNRARDSAFGAAGCRGAGAQGYQHGLCGGDVAAGPSGRPAASAPLLLLIPLTLGMDKINPVYLPQLQRILTWPQSVGIVGGRPSASLYLCGVQDSSFLFLDPHEAQPTVRWGIAGDAGHTKEAGNGGSAVVLPASSLATYFCDTVRLMPATALDPSMAIGFLCMGAADLEDLFTRLDALAKEHSLAPLMTLTSGTAQAGVGLEDDFGEPEVGGAQHGGQQQQLEEWELV

>1131

MATTDLASPSSAAPAPAALPAALSRTSSAVAVDSVNAPASFPVPAVDDAVIETVFHAAVPLAFHVLDAQGNMTASPPLYVLAPRQSYLHAVFARVPHAVAGLLPRPSTPPGDATGPPASSAADLQRRLVSTSFLCDATGTTLPWHVPIGVLYDQSQHPALRTHDDASWWSPTDGIPPWRIRVARTTDAPTATAAFPFTGLAQLESLYMHAVKEAEYMAFGSTRKVMALSKSLSEQLWRGVMDNNLSQVEAVTKDLFHNEPVARAWPVRVHIRDLAGTVATFQPLLTPTSAPEIGHVVATAGVEESDTSVLLVQGIALTCDTPMAWVHRHLVGCDGWVHVVVIKQGGADDAKTEVRTTTD

>1132

MASIDLASPSSAAPAPPALPAALSRTSSAAAVDGGNAPALFPVPAVDDAVIETVFHAAVPLAFHVLDASGNMTASPPLYVLAPRQSYLHAVFARVPHAVAGLLPRPSTPPGDATGSPASSAADLQRRLASTSFLCDATGTTLPWHVPIGILYDQYQHPALRTHDGASWWSPTDGIPPWRIRVARTTDSPMATAFPFTGLAQLESLYMHAVKEAEYMAFGSARKVMALSKSLSEQLWRGVMDNNLSQVEAVTKDLFHNEPVARAWPVRVHVRDPAGNVATFQPLLTPIVAPELGHVVAAAGAEEPDTHALLVQGIALTRDTPMAWVHRHFVGCDGWVHIVVIMQGGGRAVEEPAMERTATD

>1133

MASTKDKVWGGSVPVKFSLASREVTAMQAPKPIYKLLPRMSYLPLVTTSVRDHFVNYAPAIRGDLWFDFDGRPLKWHYPIGVLFDLVHNCGDKNSSATRLPLEITVHFTGYPSELLPCKTLSTVNEHFFHSLKEGCYLKYGTSSIIMNMQKSLHLKMEQSLVTGDYRLYQQASRNWAISDSKCQKIPIRIMIGDGFNDKTIKNRLIQPSLEMKDLEGKPFTLRSALKSTFPSLDLKFIRAVVQGIKVPTETSLFYLAENMCHADQFLYICLSLHTTPQNPSEDADGKIERQDIEGKEEGVDEDVREGAIQKTKAKANTDRSQEQNEVDSNLESAT

>1134

MADSIGCVPLVIGLCPRDNHREDAVPEPLVLLLPRSSYLMSVAHLVREAFAAFVPTIGKPLAVWLCRGGGGNNNNSGVERPLPWHYPIGVLFDLLSPSSSSNFNSQSSSSPQTARPLFLTARMTPVVEESPSDVPLCVIGTGRLDPHSGTEDNASSPAFSVDDSKREKREVQHAVSVFIRQCLKASLASSFGTTRHYFTLDPASSEALSNALVATDEEFLLEGGLTRFRKSREALLKQATVTHPTKYLIAVYDAQLDSIAPKMTTVSAAQVHSTDVSSSGSGGGGHGDQKDVFTFAHLLAMAAANLGGSDVFFSLATITTSVASVGSPSSTEGHLPLLSRGEVDLMLDEFHDSRARRMEKDGGRLLILVDGIEIPMKAPASLVLKHFLGYDYRLHIVLAKKPIE

>1135

MSTKLELSREGTFEKSEQITEKDIDFVVPDPLLEQQWSCVPVLCTLSESESLDGRPKRCQFIVPRQSYLFHYTYTQTITSALCNFARGSGCRWFSYRSMVFPKSEVFFPLPIQYPVHVLCDMIASERAFSGIPAFRAPLQLRLHFTDPKKEEFEAMCRSVPAVQQNLLPSWEIFQQRYQAVLRLDNARSGYKWHRHILKQSLVARFGTTHALQMIERDLPKHGGELIQSVVFNQCSSFCKAKCCIEQCGEMKTTKDEHHLIMIHHGLNHTQLVVCKNSTQKLGLFLKEHISAFAQLGRDQVHTNLPCNVVFVQGLNPLLSTPLDALFDLFTSCDLAVHIILKSEVSIAQYLRNEH

>1136

MLLPVHCHTLAKHINKKRALMGFKNAHVDAITEAQWMGAFACKFVPHEATRLEYQLNVARKFHCKIPRLSYLPLITPAVVEHYFERPEEVANLRISYELVVPSQTSSSGGDGGEQAVWAPEYYPLGVLVDSLCNHDYGVVTFSFRVTGGAPDSNVLALPIGTSDKGLFLNLHQQQKATCVSLFGSNQAMMKLEPQLMNQLDDAVRQNDCRAFYHPRNIVFQSGSQDSLRSSISSAALVGDMATFMIHKDGLHTMRLVSIRSAPTLGLVLKCCLQCFEGLDVDGIDNQPADSQKYAAVLLTGVEPALSTPTEFLRDNLACADFAIHLTVQQAATSTLPRGLFPQVDWKALSRNASMSSPLM

>1137

MTKAPASPRNLLKQRVWAGRLPVVFSLDPNEVTTLHAPRPFYAMVPRMSYLVTQTRDVVEYFRDAAPPVPAMQGSSIWFEAKGVPLQWHLPFGLLRDVLCGIGATEDSTDLPWALTVHFLNYPKEHLLPCDNEQSIEMHFMHTLKQATFLRMGTTKAVMALPEAQQTCIWSSVFNADYASYRQATHELHLDGGVDASALRYLPLRVHLDTAPAIQMPIVPHQNGLGRDKTLIQVLNYLLPDLFPSGIAEDTDRHTFRFVVHGVLIPGNVSIVELYQHFAYGDGFLYVAVRTISPEPSH

>1138

MNDIDNLAEIKKKLWNGSINVKILLNIEDQIIEYLLTIPRNSYFPTVFPQLIRYFQNFITTIELSKVPIWLEFEEVPLKWNLPVGVLYDYLYLPALLNDHDLGCWTISMKYEPVYPIEYIIPFNEKLAGDGQIDYMKTMNRILMNQLKQSCFVLNGTAKPIMQLSEANTNQLWKSLISRNLGDFNVLNKKIIKTIDRIPVKIYIAGSPIVVQAPISKDQTLQEILSLHTPNLSSSSSSMSHPYIQGIDVTSLMNQSIREIWQLFKHLDNFLYITLIIL

>1139

MADDREIERQIWEGKVPIVFNLAQSEVTGDEPPEPYYLLAPRCSYLPLVTTKVKKHFQSTGTDTEDEMWFDYAGQALKWHYPIGVLFDFYGSPSQLPWSVTVHFQGFPEDEVLRCPDKETVETHMIATIKEADYLKNGSTKKLLAMLKKDQKQLWMGLKTFKFDQFWSVNKRLTQRDETDPFKNVPFRIYVPDQPVLQEPFPPFRQDGSEYTLGDLLATVLPELFPAERVSPFLVAFNAAAAEARAAEGGAPEDVPVAPPSSLGKQVELPQIVLHGISPAFETPLLWLGEHCCYPDNFLHICILPSELTA

>1140

MAAASAAAGAVSQVQEQTWETKIPVHLSLAPDNISSPAAVRPIYLMAPRQGYLHALAAQAWPHLQHVLPSVPGRSTPRPWFDCGGVPLKWYLPCGVLYDLLSEGGRLPWRLTVHYTHPPDSLVGWETGANAMAQFMNCLKESSYVCRGPDGAGAVMRTGSSQQEALWGGAGCSQGGTAAASRTRPQHPRRRSSGLGRRSWRWRWRWRGCRGRRRLHHCTQLCACGSSWLGLRLL

>1141

MQQADGQAVTEECWRAAVPLQVHLAENEVSSLEQPPVLYFLVPRQSYLHSVVPRALALLQHLLPPGELAPWFEHGHLPLKWGVPAGVLYDLVAAPGGELPWRLTIHFRGFPDRQLAAYGGESALRGAFFGSLKEAACIARGSAQREDLWCQVLAGSLQPYSQILASLQLAPVAQRGGRRPAVPLRLYVRRDAGGYLSSYEDIAATSRPVEAEAADGGSISLREVLLPLLAECLAAGGGAAAGKAAPAAQSLPSTPVGRSSSAAGDGGASGSEPGAGTGAEGLVAPTAAEEAAGAAEAEPGQDAPDQQSSGAQAAAEEADGAVQAAAEPDGTAAAASWLEAAAAERRLLVGGVTPPLDAPLAWLHAHLHAADYFLYIVVHAAGA

>1142

MRQVPVGVLYDLLTSDHEQPWRLTVHFRAFPSDVLLRWDGDSSLRAAYFNSLKEAAYICQGSARGVMDMALQAQNDMWNAVTAGDAEQYASVCSSLLMVPKARGERRPSVPLRLYIRQSHGGYMASLDTATQYTSRPVETVTETGALRSLGEVLRELLSALCAKPEATNSTAGIVSSSEQRDKLDGNAADAEGEVKGEIVEAVSSTNEKPEGGSATMQEKDLLVNGRPAEALINGVSPDLDAPLAWCHANLHSADFFLYIVVAVRE

>1143

VREEAQGEAARPEGDLAVPGLQPAREASAAAAAAAAVPAEHGQFTVTGIRPALTTGLVWLSEALAHPDGFTYLVVARR

>1144

MSRLSAATWSGAIPCLVTLAEHELVSLNPPPPLTLMLNRLSYLPLYEAQIRAHFAEHVPPIEDQLWFDFAGRPLHVPIGVLFDLMAGTEDLPWKLQAHFTQFPADTLLRCAGEETARQYYMNSMKEAVYLRLGTVRPVIGMTKQDQEALWESIKAGARNHERFVAAYRQLTAEGAKMPLKHLPIMPTDES

>1145

MSSFDEDIKRSIWEGKIPIVFTLSPDDLTSHLSPSPYTLMAPRNSYFPLITSLVKDYFSSSTLVLLDEMWLEYRGIPLKWHLPIGVLYDTIVGNNNCNNSNNNNNNNNNNNNNNNNNNNNNNNNNNNNNNNNNNNNNNNNNNNNNNNIIMEQPYWNIVVHFQSYPDRILLRCPNIESVRTYYKNVLKEANFIKQGDITKINNLNINQSNDLWDGLKSHDYDKFWSVNKKLIPNSNKEYKNIPIRLIINYKPPIQELIPVFDENLVELTLENLFSRIPYESFSNFLNYNNNNNNNNINNNSPPLSPNSNNNNNNNNVDNSIENSLNQTNVESAEPEFSNLLQYIKATNAEYKIQGIQPSLKSSAVWLYEHFGHPDNFLYIVLIDPSQNNNNNNNNSNNY

>1146

MSIVEGAERSGVWPVVETERHQYMVLDMRSRTTELDWGSVKFDQVSEGEGSRRMTSSRHFMCKVFLDSDAESYCSKLADMLTYMRRLALPPTAPSGLARLSETPARRLVVSQTKCTCGGELLPEADVNATLVTEIRQGSVMDALLQDVRQEAWGGMIPTELSMDSSEVTSLQRPLPLYLLLPRMGFLPCVAEAISHHFGEVAPDAQRTLWLEETRSGEPLRWHVPTGVLFDLIAGVGQEEGGGAKHGEEVPWSGPGRLGLLPWRITVHFQGCPRRQVFPLEKEADIRRHYTNALKQALFLQSGSSRAGMTLAKENQNRLWQAIKSNDTKVFHEMDALLGGDRGTVRLVPVRLLTGGAPPSQLPIPAQRPDGTPFTLRDCLRWFCPGVPWDAEEGAEEVPLSQQQQQQAGIAAAQPGKGDFGRRDSAEEGTVNTVDTQGNAAERSGVEERLETDTRVIGGQQAGMLAVSSDDAGAMSLPVVLVQGVEVPLDAPITQLWGALRHPDHFLYIVVRKQG

>1147

MEGQMEAAEARELAWAGCIPAVFSLAQDEVTTLEPPPCYHANLPRQSWLPLVTGEVVEHFLPFAPPMARASSMWMQCRGEPLRWQVPVGVLFDLLTSGDDQPGNGDMLPWQLTVRFQARPTDQLRGSVDDAKALVRSALKESAFLRCGSAQPVMDLSPLDDARIFEALCSASPIEAGSPAPAPSSGGSPPPLPPPSCLPPASPVSSQAAAGQAASQQTPAGKQGQPSASDAPASSPYEVYCQARTPLEASLLRSLGSRGERAVPTRAFVAQREWRQLPVAPLLPSGDPTTLADALALLLPRAFAAASDAESSGGEGQRPTAIVQGVVVELNTPVSWLYEACSHPDGFLYVVCLMPQSQRTRG

>1148

MPDFSKACVRAALCADRAAFVAQREWRQLPVAPLLPSGDPTTRVHVLAASTRGVAASDAESSGGEGQRPTAIVQGVVVELNTPVSWLYEACSHPDGFLYVQCQLPRRTLRADE

>1149

MQVAEYSSLIDSTIKRKVIEGTIPLIVRIPFKNEEKFTLYNASRNEYLPFLLKSSLTEFLEEVEQPISSVTYTVNEQNVKWYFPIGVIFDALHNGSLPMEITIGISQNQSTFQPYENEETIKNYHIQQLKESVYLRYGSIQTIRQLAVESITPLWEFHAQNKLEEYEQLLNPILITGENWQNIPIHWYFGSQRRYTDCIPVIINDHLTTLIEGLQVFLKERDINDNDPPSLQLLKTNSFEQCPNLCIVIQGITIPLDTPLLWLALNLSHSDLFLHVIIRKTDELLIVI

>1150

MGGSATDEAVQREIWKGEVPVEYTLADSEVAVIGKPPPFYQLIPRMSYLPFYLPDVRNHFAPAAVKLGQDKARMWLSHNNAPVKWHLPLGVIYDMIHQDSDPAAGPVLQLTVHFDGFPEGKVLSLQTEEDVGSFFLHSMKEAACIRFDSAKAVITLTTKDKQELCEAVRDCYSRFSSYQKVREKIEQGEKACDKLAVRLVVRSAVDAPGQFRLLLRTARRDQTVGQLLHATLPAHFPTVPEGAAASPSATAVLHGVTPLAAMPLRFVAKCSHPDLWIYLVVLLRPTDPEAGVGVTP

>1151

SDKLAVRLVVRSTVDAPGQFQLLLRTARRDQTVGQLLHATLPAHFPSVPEGAAASPSATAVLHGVTPLAAMPLRFVAKCSHPDLWIYLVVLLRPTDPEAGVGVPP

>1152

MNPSQESPHLFDSETVKRINNDGRIPVVVTLAPTSLSAPTGPPPLHFLVSRQTYLHLGLSTAIRRLHPFAPIAFQGLKWQEPDPGELKEEETDTKEPIQPTDNSKSDLPDCWWEDEATQAPLRWQLFTGVLYDMHANHTLPWRLRLHFNNFPSQLLPFSIQALRSHFQNSIKQAIALRAGSGRAALQWMTKEAHDELWNNLQEAKCGSRHEWRSRITEDVTNQRIPVRVLLSSNSLPCLQKSCLVDRQLRLGGLLRDWLPDLGFDRTMDTDQDSLADFSSAGWKIQGINPSLDLSVLDLWECLCHPDSYLYIVVSMT

>1153

MAEEERAVGEGEGLRGAWREAEHLLPLDEVVRKEVWEGSIPIRVELARQDVSTFEAPMPFYVQAPRMGYFPLVLGQAKAYFQNYVPAVCCNDREMWLEHKGNALKWHLPTGVLFDLHAHGEDLPWHLTLHFSGVETKGLLPYEGDVSLRRHFRQTLKQATSLRYGSCKRVNNLSVAQMEQLWSSLQENKFESFTSVNSELLKLSTPGPSGLTEIKRLPLRIYLPHLPSPIQQPFTPFKDDGHLTTLHDVLLQILPGLFETTRLSFAKTQEEEGEDAGDDQRPTVLVQGISPPLQTPVLWLCQQLAGADTFLYICVIPKSKDQEVFAS

>1154

MSFFDEDVKKSIWEGKIPIMFTLSPNDLTSHIPPSSLSFVIVMMMMMMMLTFANQKTKKMFVPRNSYFPLITGVVKDHFSYSTLVISDSELWLEYKGIPLKWHIPIGVLFDMLVATPDTTASQIWNITVHFQSFPEKVLLRCSNEDAIRTYYKNVLKEANYIKQGDINKLNNLSINQTNELWDGLKLHDYEKFWSINKMFYPGNGYKHIPYRIIQNNKAPLQDLISPINENGQETTLESLFSKIAFNNSNNSSSNNNNNNNNSNNNSNNNNNIDTSNETQQSTTTLSSSSTNSTTLLQVLNSGSIRYSIQGIEPPLNSSLIWLGDYFSHPDNFLYIVLA

>1155

MADDDIRQASWEGSIPIAFALARADTLSFAPPAPVYVMASRNSYLPVVAQAARERFTRISAITPGSEDEVWFETRDGVALKWHYPIGVLFDLYGDGTELPWEITLHLRGFPGGEIPRPATPDACRSVHMSSVKESACLRRGDVTQIDNLTPREAQDLWEGVATNDMRRWAAVNALLVAGAPPRHVPVRVCRPGAPTSQDLVPVGATLREALAAVAPGALEGGGARAVVQGVEPPLDAPVAWLCATCASADNWLYVVLRQ*

>1156

MPSTDELLQSELWRGAVPCEIRLARDEVAEMSPPPPLYALVPRGAFLPLWHARDGRCATHFARSLPPGVGEMRLRLLPIRYDPVRVRGERHAATATPWFDHEGTPLRWGMTAGVLRDVTCGASDETHGTLPWKLTIHYRAYGARGGGGGGGGGGEDAKENDADGLIRCDDEESVRARFFNDLKEAMCVARGSAAAVMSMTRAAQADLWRSVVDGERRLAWRAEEALGSRRGGAESSSGGGSGKRSDARRRVPVRFYLLAGDARREVKCLGEVKIVSAPVFVVMREEEEAEDPGGERERERTTTTTRTSTVRSALAGLGVLAPTRAVCQGVALELDTELERAHATLKGSDHLLHVVVWCEA

>1157

MHVIEKMKTERIEKKEERRRQREAERERRKAEKRMLKKESEHGNKEQAGVQSVESAKDKDDENANQNSESVKDESAGISSADVSTASSSEAKEEGQSVSSASASASASASASASSSSVSEDSEKQKQENERNEESSKEGKAKGRKKVSKGRRPKDPDEKKIYQKEYDYLQKYSPFYTLYHYLHTVLPELYNREPEEVFPELNEESDSEYLSDEEDDDEEDEEGDVEEDVEEDEGKGEKKSEDDKNTKKESESSQTQSESNASESATPANSASSSSTDLPSPQNPSLQLPSDSSALPAANSSSSENEVIRPDAQKTALSPSSSPSDSSPIESSASPAGSTDPASSSQSLSASSSSSSAHLSASDASDIASSSSTPSAALTSSLSSASPSSDASPDASPDADPEKDEISYPEALMTPPLLSSRDLQPQRPCSPSLQFPPVYCHSVRVPLDTPLMFLLDHFSYPDYFLYFVIGLDRSGEKENERENEKEKDNEKEKEKEKEKGKEKEKENANN*

>1158

MCSLQAKLWNLKIIVSFKLDPSVISGKYIPISLPIKIPFSSYFPLFMPTVYRFFSPFVSDMDYRSLWLSFDNKPLEWRHPVGVLYDIYVSSQNRNLITTKRKLTWEVIVHCGNFPIDSVMQLHNKELVKFWYRSTCKEALQLRYRVNDDVNLACDEDLKEMFDSAANANYANFKRFTTKSFLMKASRIRASQLHKKASPAIRVFFTHNSSVYYEKVVEREEFDWLDEAGEDREGGERSDALRRLSDGSNSSVSTGFGFGFGYGFSFDGGGSGAEDEGEKGERRGGGRGSERGNERRSEKGKETSQKGRKNSKRSATEQKAAKARKSSKSEKEEEKAAAEEEEKEEYSEANRTFFSITTNADVPGSDCDNIAGRASYSPSSQSSQSSQSPRSFQSNQSIQSVQSFQSIHSIHSVHSVPSAQPTHSAHSVHCSQQNQPFQPFQSPQSPQALQPSFARVMLLDYLNFCFPELFPLSDEEKALRCAQRSFARSSTTGTVSSGCSRSATPASFCSSSSSSSSSSSSSSSSSSSSPSSSSSSSSSSSPFPTSSSSSSSSILLSPPQSPSYSPVATSPSVHSSPFSPSSSFSSSSSSFSSTPNTEQSDPNSPFQFNALPPSSSPSFSSSPSSLSSSSPHSSSSSFSSSSSSSFSPSNYSSTHSICTSPTAPSHPAASFSSIRRPTLSPPSPQFVVTLSPVIDHRSSFASFNAFNTSYLNNASNPLNSSYQLNSMASAKPVTTLASLTSLASISSLNAYGFDATTASVSASSVASPIVISTTSSSSSSSSSSSSSSSSSPSSSSSSLSLSSSEYFTLNDMHPLQHYPSCQTSFSSNSSQPETNEEQNMDGTKTNTNSDNNNTNTNTNTNTNTNTNNTYCNCNNNGNDDNDVNININNNDNDNNKININCRKELLSSSMRKRGHSTPELTLSTEKMKKADVLCNCAKDQVNEKENADSLTLTSDEMKLSSSSSFHDSSSSSFPSKTSNSTLPSTELSSSSPPPSSPSPSSPSSSSQTKQPQHNLQYQLYQHYAQTDVSSGASLISNAISSHLCSLSDSLKTAPVDSSYSSTLSSSSSSSSSYSSSSPPFPSISSVSSSSSTQRFSSSPSTSSSSSSSSSSSSSSPSSSKHQKGSRSLSVTHSPSHSLSEHKINPKNISSFSCVNYSSVVESEPNNISSLSSSSSLSSSLSSSNSNANSSSSSSSSSSSFPDSSFNLIAHEDIDEDIQMSFTFISHSPPPSLLSPLSLVSLPSSSSLPSSFPPLSSQSNCEPQKIPASSADTPNPEITSGNSENISPSPISPLANSPSSDHSLLSAKSHSGVSEQQNLSISSDSLQSNSTSYQLNGVINQTTAKNEENISSSIALAAASADGVNPFSLASTEISYPIMLSSPSSSSSSSSSSFSSSSSSSSLSEHTRTSSRSSLSSCHSHSSSHSGSSLKQFSSGKANGLNKRDSLSNSSLEDEKEKEKEEKKEVKKEEEEEEEEEEEEEEEEGEEEEEEEELDYVGDFVDVTRDDVKRLNAAQVMFIEGKEKADKKDGNHVELAVGDSIHSLSSSSSPSPPPPSPHSSMSSELPTALPSISSIAFPPPPSPPPFSSPSPSSSSSPHPFVRNPLLVENASSISKVDLIPFRPHSVSEPISAKSSASPAFSATSQANDKIQAFLSPQPHSNSLCSSSSPSPSSSSSFTTLSSSSSSSSSSNSLSLHVHSQSIDSSAPSSSASLVSNLKKKKNFSSSTVTIERDSSTSSLENGKLVGKVGKLLENNEISSDSIKMEFSSVDKKASVGLQQHESSISSSSSSSSSSSSPPPSPSPSPSPSPSSFSSSSSSASSSPSSPLSPSFSGYTDQTTTHSNPLISHCYPPVYCHSMRIPLDIPLSVLLAHFQYPDYFVYLVIDDTRTAHSQEDGAVEQG*

>1159

MSKIQKELWKSAILVHFIIDPSTKCYQRKPLPLSILIPFSSYLPLYLKKVQTYFEQYIPHIVLDQIWFSYDGKPVEWQHPVGVLFDVYVISKTHLSQSTINGKSFPWVINVHFENFPTDSLLRLASEKDVRFMYRSACKEALQMKARVTDDVNLANDNDLEKMFQAAIHANLKRFFKFFSSKFLAKASKMMLMNKKNSPPIRVFFTGNATSYVQKRCTREEFEWIREFSFTSFSTFKMNGGESEDEGERGDERDGYYEGAGGNMYQTSSTADGSQMLSLEGRREEADAMDKEGGKEKMREREREAGEGVYSLDRVNTNRDDPEAVAEPAEIQQYFSIVDAKDKDGAAMYIPSPASSPSSSSSATSVHSKDGSSKKVGKEGNRKEKEKEKEKAKEKEKRKGREEKEKLKKKKKKKKKTLKESEKKKKEGKNGEDGDKDNKGEEDEDEEGDDQFDRPLPIDEWLFYDPTITLHHYLNFCFPSLFPNPPPLASSERNYFGEEEFESDANKQDEAEGAATGEEEEGAEGKGRKMGMTGEGDEEGGEGGEGGREEAESKRAATGSFSQREERGRGRESERDGTTATSGDADDQLSVSSSSHLLQSKSTSEQGAYQKNKHHDSSSSSSSYLRSHKHATSSAPAKPPSPPPPNDFVYSVIAASLMDNLFEPATAAHPSSSSSSVSSLASSSSASASSSSLIAAEPAGSPTTSMLSSQSIDSLHPFDLFRSASTSSSSSSSSSSSSSLRSPLHRKGASTDSSSSSSSSSPSSFSSPSPSPAIRRAPPAPPPSIASYNSPSDSVLSSASSFASLNGLSGSFSHFHRTSTAGSAASSSSALSVDTLPFSSSYSFSANSSTESAPLPKSHIRIHSSSPLSSSPSPSRSDSDSATPTIDSPAVSPPPAAALAGASSSVSQLSALSAGEGDVVSSFANPFQNQEVQSSLSGREEGAVEDGEQLNSQKGEKDEKDEKEETTEYEEYESEYDEEEEEGEEEEEGEEEEEEEGEEGGQTGGRIRERRRGTENEDEHIRKMMKMAIQGEEMIYDRGLSLLHDSMRQQLDEPDFKEKEKEKEKEKEKEKEKEKEKDTESVNVNNEKNDNTKTDDVQSHPLKNAVEENAQNRNKENNKDAKEAEDKNENEEDEGEGEGEEGEEEWEEYSEEYNESEEEGEGEGEGEGEGEGEGEGEGEGEGEGEGEGNIKKGKEIIQCNKIEEREEKKKIVYEQKVKHEFDGKDDEEDENSKEITEQKEEETKEKEKEEKIKEDENTENEVQQKEEKKIDTTEERPVIFEENIKENESNQKELDNKAESLTDDKIESYKEEEKTEKGKTTNGNEESSNASKEKNECNIQETNTISDEQKKSETQNELNKNGIIETEAEAKTEAKAEKKETFSLDTAKELSPDAKGEACETSEEDHSIFRVPDELKQTTKEEELNGMENEAKDESKKEILKENVSDTKEEEKTTEGKPSLCSQTVGELASSDESTKQIPKDKEQAEEQNIKETSVSEELSNSQSIYRSIYNMSEASSHSPQSQTEHIPNSLSQSDLTLAKRNLSSNDSLASPISSSSSSSTSSSSSYSSAKLADQISAPSFSSEDARASSPSPSLSPSPSPSFSSSPESSPQPSPFNTFISAQPLHPISLNDLSSSAQIQNDSSSFASSSSSSSFPSSSSTFSSSTDDDTPVVLDNTPHLPRVFCHSIQIPLSTPLSFLLSVFSSPDYFLYLVIAPD*

>1160

MHQLQNKLWTLKVRVLFCIDSSLITGKYRPLPLPMKLSFSSYFPLVMPTVHKYLQQFVPDADIKSVWLSYNGKPLEWRHPIGVLYDVHVSSSQNKKDFKEGSPPLWHVTVHCGSFPHDDVMSLFDKQTMMFWYRSSCKEALHLRYRVTDDVNMAKDSDLNEMFEAATSASLTNFRRYFSKSFLSRASKISPAFLHKKASPAIRIFVMTNSSSHSNEANLSENASEKADETSNERKMDGKYYQFAIDREKFDWMGAKEEEKEEEKTESSEEEEEGEEEDEEEEEREGNIVGKKANYEVIYDGGINSIRDDKYEGRENTKGRERERERGRERGREEGEKGGEGEKEEEEEEEGEEKEKEKEKKEKKKKKKKIITNM*

>1161

MARQHAVRHVVVGDIKGRVAFREVFSQAVVIGVKRLQQYQGINTMAAPPPTSDLSREDLEAQVAQQGSLVRELKKSKAPTEEIKAAVAELLRRKEAMAAFDAANARPQFDKQGFEDLMKRRFFFAPSFEIYGGVAGLYDYGPTGCAIMANMIAEWRQHFVLEENMLEVNCTAMTPHYVLEASGHAARFADIMVRDVKTGDCHRADHLLEDVMHNIATAPDTSAELKKECEKIGRLADSYSKAELWDLYKRFDIKSPTTGNDLSEPVDFNMMFQTQIGPTGHLAGFLRPETAQGIFLAFKRLYEFNNFKLPFAGAQIGTAFRNEISPKAGLLRVREFQMAEIEHFLDPTDKSHARFDRVKDVCPPLYTANAQQAGEKITRMSIGDAVAQGIIANETLGYFVGRIFQFMVHIGVDQERIRFRQHLPTEMAHYACDCWDCELETSYGWIECVGCADRSCYDLEQHAKAAKQPILASVPLKEPLIKDVVVVEANKGLVGKAFRQNAKVVFAHFEAMSNEEAEQFEKDLQNGPVTLKLEAGEFEIQPAWITIKRKQVKEHERKFVPSVIEPSFGLGRIIYALLEHSFECREGDAQRTWLRLAPLVAPVKCSVLPLSNNAEFNPFLDELVLALTRAGVSTRRDDSAASIGKRYARTDEIGIPFGVTVDFDTIKDRTVTFRFRDTMEQIRLSIWRGQLPVRFTYVNASDDAIEPCHVRIRPRIHFRFQLNRLTQRTDLMELLRLTQMLVPRLAYLPFCLEQIAEHYRSIPLINSLAVLRQSSDVAAVLANDYERFNQINASLTGTEGDGAWFKHVPYVVYLTQGDSSNAAVTFETTTEPFPTIDENGVPWCLQDLLTWLLPDQFPPQTKESTGEELAEQVLIQGIQLPLNTPLQWLAQHMCHADNFVHIIVSQATNATNA

>1162

MPPQPGQGRTPATASTSNTSTTLAPHNPVAKAIWDGSIPIQFSWDQAEATAVGSQAVESFFLEAPRCSYFSLLTSRVRRHFVDMALHFIGDDAEVWYDYEGTPLKWHYPIGLLYDIHGLQASSGKGMNGSLLPWKITVHFQNFPADKLIKSQAVDSSQDYFMSMIKEADYLRNGSIKKIMNMSKSDQTQLWEGLSANDYDKFWGMNQRLTFNDGTLARNLPVRIYLPENCPVIQEPMSPVDENGQPKTLRQVLNVVLPDLFPLVETGPPVAAAMVHGIIPSLETSAIWASQNMSYPDNFLHLVIIFK

>1163

MHPFEGYAKLCGQDVEYYLQKLNVSLGRTRVPDPQVIDPKSLAEEQTVDANQSLNSTLSTTGHLQPSIETLQRVLWEQDVDIPLGTNKNISRLHAKIIYNAITKFFELHVISKNGVKVNGIYYPPQPHSQPIPLHNGYELQLGDCFLTFMLPIKKITQSEMYQMVNLKKRDMQNADRADRDNSSTNDQDRQGNDMMMTDESHTSTMNDEVSDALSKNFLREIEPMDDPNGEYGSFFLNEGKKNIFFRNLGNGLAGGTPISSEQANVLARSLTTSIGPSLTAAVTQTLSGIYLENHSDFIKSFLSKSNAPSPHTEMVLEYLYNSPDIENAESPEAVSMLARLMTDMDNNIGSNFSNMSKNTKKLLHIVLNAALATEQQDIFSSRVNLPSIDSQPPPANNFTAITSSSQPLQMATTTSSLTSLIPPPILSSSQQQQTQLTDVPPSSQSTTQLPPDDDLDALDEFDELMAFVNAPMDTNSEQWRLLNQNSNVPPVTTTTSSIPPNDTTVNSSATTSNVTAPTTEKKKKTTKKKKDEKETTTKKKETKKKKDDKKEDKKEDKKEEKKEEKEKKKKKKKEEPSTPKDTVKQEEPAVSTPKDPNLKKPDISYSTMIAHALMSAANNMMPLKSIYSWISTTYPFYDLAETGWKSSVRHNLSTNSKYFRRVSPEEWRQLTGEEKKSKSKKQGSSDKKMAFSVYALVTEHCDEVLGTKKAKSDKKASSSTSSLTTLSNATTAPVSSSSTLPANSTLSSSTNATTDQTAITSEVKEVKEKETKKKKSSTSSATKKRSRKKSESTDLDLNDGLLNFDEIAPSMSLDKPEKKKKKKTSKKSETPTASTTPAAESSTPVTQTNNNNNTNVTTPQLSHSSSNQNFQHSTPTNQISITQNIMPNTSQPLAPKQPFPLSNQIPNQIPPNQMPNQMPNQMPNQMPNQIPNQIPRQSTNNLLEMFVQNGMLNAQEPSFLKILLDGNGDSSANNPLSALMGQGGQNMNPNQGLNTAPLGNMVDSQGKQIDLINLLSMVTGMPQQQNMSQSGMSQPPMHQNFLDPNNRLVTQGRPPNWPPGAGLPNQLQGGRPSMGSVVGMQNPGQFPVQNPNMNPLGHPGFNQFPFPMMNYSQTLLNFDPSSILQQQGNGGIPPNNNNNKYNNNSDDASIRKQLWEGSIPSVINIKSEHITSFQQPFPYFTMIPRHSYLSLLIDKVKSHYSEFSGFDSDQLWFSFNTIAMQWHVPAGVLYDLMYILDPNTLKLPWSLTANFGAFPADTVIRCSNEEDAQWNYLNNIKECMYIRFNSTSAMMTFSNEDQKELWSAVKSQNEQVFCKLFEKIKLGAKIASIDDMVYAIRIYTPLKKDSNSSVNLTDDSKKKDVLKYKKNMICLKRDQAKQLDLQTFLNNHVIPQLTKGGYFGDCETKIDRVVIQGVEPSKETEMLWLFENCAHPDGFLYMCCK

>1164

MESENTTESEENIYPFENVREKVFNGILPVKFVIAENDKSKIPESINYEPVYYANIPRCSYFPIYFKQIAKYFIDRHIIENEEDIWFSYNDIPLKFHYPVGLLYDIYDNTNINQSQNSDKLGLPWEITIHFSNFPKEKLIKIKDLDTIKDTYFTTLKESDYMRYGNANKVMCLSLDDQKILWDSLCQSKFKDFWNINIKLLNIMPWSNISARLYINDTVIQEKYANISDENVTLRDLINDWCKEIFSDKNLEDINYQYILHGINVSLDTPLLWLGQYLSYPDNWLHIVLKQKEV

>1165

MSHVDACVGTVAITLEHKEKSLLLNVPRTASLGGFLARTEIRERLRLILDVPERFTAIEVRARDGKGEWERFDARFAHASALADCASTLDGVCGIRLEVKGDDDDAMRVDSGSGWDDLERECRQSFFNGVKEATYATHASASKAMTMTRESADAMWAAAQRGDYEGLRRAESSSAELREGRREVLPVRVYEVRDGSFARAVVVSAPASVERRDITVGDALRQFGVDLRDVRTVISQGIEIDLDFDLAKTHEALRHVDGFLYLVTHCGTSS

>1166

MDNQPLEEMSLEDIRKQINEQSFLGKFIIQDVEDIIPFVCIVYNNKYPGFYFESIYNHFIRYSNKARSSLWLSFNDQPIQWHIPFFVQIDRINEEVEMVVHFKKRPENVLPISENIQVDIKNRYFWNLKQACMIRYGHKWERQIFQTITIEQQYKMFQNYSDHNYRPQFYEFFDKIWDLTMLKKEQQLSIPIRIFIDGNMIQRSHVLKDAKETLQNVLQPIFEDSLNYNELNVNVLGLQVNLDFPVRLLVSTFINPDGFCYIIIK

>1167

MNSQQYQQEIWDMRVAVRVELQDSKIPPMYLMIYRQHYLLFYYQQIFDHFHSFTPQVNKINQITFQCNEVQLPYQIPFGVLVDLHPSEDIFMPIELKLIYTKETHFVDLEDEIKNQIKFNLKQACFGRYNHEGYGTPLKNLTQYMSISDEKQALKQIKSFENKEDLYRYYKKIFESPKTGRIQLPIRIYFKTGGHLQTIIELKDQQLTIGQALSTKLQGATFIFNGAHLNMSIPAQDFYDQLYSIDGFCYFVCE

>1168

MVEDEGRRTQAANWHGSIPVVLTLSSRSLSSPTMPPPIHVLVPRNTYLHVGLQTAVNRLHRFAPTTLSFVSGMIQKEPDPGASTFAEDDENDQADTNTASQTPSDSAIGRAADERYPVCWFEDEDTQFALRWHLFAGVLYDTKRAKVPSASSLPWKIRLHFTAYPTSQILPLEADRVLVQIQNFYKNSVKQALCLQYGNSKAAMNLTKESHFRLWDAVLTTTYPLHRQVNDDLPTQTQETISQIPVRVLVNAARPPIQRACRDSSVQLGALLSQWLPDYFETVNDTAQAKDQVVQWRVGGIQPGLELPVFQLWKALCHPDYFLYVIVLTN

>1169

MSSPSSSSGALLKQHVWAGRIPVVFSLDPNEVTTLHAPRPFYAMVPRMGYLVSQTRDVVEYFQDAAPPMSAMQGASIWFEAKGVPLHWHLPFGLLRDLLCGPTEDGGADLPWAITVHFLNFPKEILLPCENEQSVESHFMHSLKQATFLRMGSTKAVMALPEAQQTQIWTSIAQNDYESYREATHALHLDGGVDASALRHLPLRVHLDNTPAIQMPIAPLHDGREKTLLEVLSYLLPDLFPPDVQTHKSKLVVHGISVPADVSIVELYRNFSYADNFLYVAVLS*

>1170

MEIGYMEVPNIKDINSNIEKSGLVLCVSLNQKESESLISPSYYYIYVHRYMYLSNIIPKCLEFFKSFILPFYGNKFGVYFECIKKEQKKSSNNNNNNNNNITYTSTNNYEEKIVLDWRLPIGVLFDIYCDLDKSQKEYIKTYEKFNDTNINMNNKKNITICSNNELFLNHINIVKLKGTDKNYDKKSNERQNQKNIYDANRNISTNTNNSSNVNHQQNCDRTDDDTLHKGDDILHKGDDILHKGDDILHKGDDILHKGDDILYKGNDILHKGDDILHKGDDILHTDAFINEKETKEGTDKKKQINRNIKNEERERGKEVKNIIVENYYEENYMKEKKIIVENGKGSTKNDVEENKKDINTHYDDIKLNNVDIYDDIKLNNVDIYDDTADFQYIQFIQNEKINNEWYNKQFVNISNKNIPWMLIVHFKGEEEYPLSIINKKYDEKNTHFKGDINILPYNNYIPLYKGFNNFEEYIINQLKKANCILNKNNRALEILPQRIQKDILYSLKHFHIEKICSLYREYIDYNMLNFINYFNNSYIKKVQQVCDLNRHKDNSIQINGKSSGKQNEENLCLLKMDLHEEESPVKITHCKDILNNDNENYDNENNNNENNNEHNCHRNSYDVHLNENFINDQSQKNHISNIRTSYNNDHVSDSLQTDIQNDFSSIILENTKEQGDHINFKNNLHNNEKYPNVYDILKDEKVLKDCPIILHIYGPPYNQILTKYPFLKLIHSNNNNNNNNNDNKDNNNNSNKYDGCKKYIQNVHLNTLGDFLHEQLPSFVRKIINKDEKNETSHILNKYDKDKYLNSETIYYFIEDDYLIFSPYMFIIVNGIQIPLNTPLYWLAANFSQFDHFLHITIRIPPY

>1171

MITFVFFLPKNLRVKNCFFFVENNLKSNLNNNNNNTLLNNCNNPNGTNPNKFKTVKKKKKKGNKKFIKKKKKMNRSNGDHTKNYSFDLNFALFSSNATNVQTKLDMDFRDRIWNGKVAVLFSLAEHEVVSKKSPAPVLSLLPRMSYLPLVTDHIRNHFIPHAPALQDGMWFECETTHHVLKWHVPIGVLYDICVPLHYSTPWHIIVHFQSYPDRPDVGRCDHIDVVKRTYFNALKQALLLGYNSVNMLVNLKKEDQDQLWSSVVQARYDLYFSIQNLMFDPTKEDQSSKKLLKYPFRIILQGFDRDSLDGVPIQRPVEVPSEKDGRYKTLEQVLKPILPGILEIVESEKTVALAEKSVFIKIQGLNVPLGSQMSWLVKHMCHPDYFLYVVIVKISKK

>1172

MNDIKQLLWNGELNVLVSIDPSFLMKGSPREIAVLRIRVPRETYLVNYMPLIWNKIKSFLSFDPLTDSEKYFWFEHNKTPIPWNYPVGVLFDCLAGKSATFTTSFENQVKDVLTFLRIHLVMGDSLPPTIIPIASSKTQAEKFWFHQWKQVCFILNGSSKAIMSLSVNEARKFWGSVITRNFQDFIEISNKISSSRPRHIPLIIQTSRTSGTFRISQPTISMTGVNPTLKDIEGDILDVKEGINGNDVMVICQGIEIPWHMLLYDLYSKLRSFDGFLYITLVPIKGGDKASSEL

>1173

MDEAVERQLWTNRVPIAFKLSEADEPRHGASSKPRRTDTTCYMMVPRMAYLPFASEALASFFSIDMLEIIGPDGSHQPSSLQASELEAQVKEQQPKVLWFEFKGYPLKWHVPVGVLFDALTGDIDDVGRPWEITVHTKNFPAKALLPYSTIEDLQRHFLCRLKEACFIKQGTLNLDELGDVQTLKRLWSGLLYDNYNDFQDVNQHLMSTPDNNWFKQFPFCLYMTARTGDTGPWEITQMVREPFGVADDEGMPLTLSDLLSVLLGGGFTYTNATATTDVDADADDDDDNGGDDGDDDDDGTEDKDKKVVMTAGEDNDGGEKQQVSVIVQGVEPPLDTPLQWLAAHMSHADNFCHVIVRLPSTP

>1174

MVEAMSIRDVPLQKLVESQETRRQLCGGKLLLRIWLAPDEVASLNEPDPLFSLVPRVAYLPFLFSDVLEHFKKSISRMGQPYELWFDYNNIALKWHFPVGVLCDSLVGMEVPVPWDLTVHFRGNSSLTKDLLPFSGMADLQRSVMNAFKQATFLEIGSASRFMRLEKKEHITLWNAILRSDLDTFSQVKEKLMCTSLSECKSLAVRLHLWVSTVSTGSAPSSNSSNCDVLLHKAPPLTEDGKASTVRDFLGLVMPPLLKDGELTEGVDILLQGLQVPLETPLFWLALHASYLDHFLHLVARVPDKCFGDVLSP

>1175

MGEDKEISRQVWDGRIPICFTLAEDEHTPGQDPPPPYYVLAPRISYLTLVTDACQRHFLNSKREPPNFDDEFWCDLNGMPLKWNYPIGVLFDLYGSPDMLPWNITVHFQVYPEDDILHCAEKSVVEEHMLSCLKEADCIRNGSAKRTLNLLKKDVKQLWDGFRSGNYERYWQINKRLAPHDGESAKNIPFRIYRRQGGVVMGPFNPINEQGEFQTLGDLLSTCLPDLYPPATSYASETDNLQHSLKPTPIVIIQGVQPSLETPVQWLSDSCSHPDNFLHIVIRMSDEEVEIADDSVQPPM

>1176

MQRIDTAVTHGIWDGQLPVRISIDPTDAKTLASGAILHSHYTFMHRCSYLPLVTESVREVFVKSGVAFTGHDADIWYEYRGVPLKWHYPIGLLHDLLTSQYRGSSPLPWHIIIHFTNFPTDKLIRINAANIIDTPHDSFMSMIKEADYIRNGSIKKLMSLSKQDQTNLWQGLSSHDYDKFWNVNARLVTNDNTIPKYIPLRVYLLDQPVIQEPVAPVDDNLDCEYTLRDILGRMLPEIFPPSLDAPTTEGADNTRKSDASEAVAILHGIVIPIETPILWLSQNCSYPDNFLHIVICR

>1177

MTELEEQVLDEVWSGFVPLKISLSKYDISFSGTPLDYYICVPRCSYLPLIVENIRKHFDQYTSTDYIQDEIWFSYKSVPLKWHYPIGFLLDIYSSILYNSVNLTIPFEIEVNFRRFPADILLPYKGLKSLYTIFQNSLKESCAMLLGTSSPVLNLSREQEDKVWNCAKTGNYKGFAESTRDFSGLSQENCKLIPVKIYYQKLGNGIMLHPFQASEQKNVLDAVKEVVEEFQGKIVAQGIEIPGDSDLHWLWKCLAHPDNFLYITLLDS

>1178

MTELEVQVLDEVWSGFVPLKISLSKHDISFSGTPLDYYICVPRCSYLPLIVENISKHFDQYTSTDYVQDEIWFSYKSIPLKWHYPIGFLLDIYSSILYNTVILNIPFEIEVNFRRFPADTLLPYKGLKSLYTIFQNSLKESCAMLLGTSAPVLNLSREQEDKVWNCAKTGNYKGFAESTRDFSGLPQENCKLIPVKIYYQKLGNGIMLHPFQASEQKNVLDAVKEVVGEFQGKIVAQGIEIPGESDLHWLWKCLAHPDNFLYITLLDS

>1179

MQKSQINQSQYINTQQIPQKNQVPADAVNMELRRRLWDGQLPIKVDLALNDTISIEKPRSLYIMAPRENYFFYILNEVKSLFDPYVSSDRKDSYDNMWFDFNNTPLKWNVPIGVQFDTIVGIKNKQKDLPWCLTFHYKDFPEEINIHLQGLNFLKYHYINSLKESHNLRMGQGSEILNIQRKDEERMIQGITQHNFENFWEVNQPLCDKNVAELKKYAIRVFCNKHHTYLQPNIEVNSTVDPDKNPLTVTLGDVLAETFPKLFEPQLNDKGDIEIVKKREFDVIVQGVEAEFSTPMYWLSLNLAYLDNFIYISFHMS

>1180

MAEEQKQTDFERDQKEIRSLIWQNKIPVKIKLDPEQNSNNEEPFPLYMFFQRVHLPVFYYEQIHEYFEKFAPVPRNQDDIWLEWNNKPIAWNLPFGVTFDLNYQDPSMKGELGDDDSFDLGIQEIPPPFIPQNIILHYGPCTISNDYTPIPSIPGKTVLDIAKKSFRFALKESLHIRFDSDTILMQMEAEKQNELWESYKNLNQNTFFSILDQIFQLKNHLQNNKAIKLPVRVYIEGYIGRIQLSHQIDSENDTLGDFLTKYFPKILKQTDTAFEIINDNYKNFKVICNGIVCNLNMPLLYFYKYFSHYDGFTYLCIQK

>1181

MWVTAAVKAATGGSAGAAGVDGVWELVVHFTDFPHDAEDDEADTANMMDMTTMVEAEEVKAWYMNRLKEAEYVKHGSSKGIMLMSKANHAQLFRAVATHDYDRYWAVNRTLSADSVASPAKLKHVPLRLAFRNTPTMADLILPIHPTTGELTTLGNALDALLPDKPPGTIVLVHGVELDPDVTLHDLTNSMAYADNFVYVAIAYPPHSLPA

>1182

MSALRSRNSGTDCPSRKEGLHAGTASETGPKREEEGTCDGVYVSIRDQGDPRDQLLSPRETIKDQEPDDRRGDAQRSRVSQPLFRENRGEKLLHSASPPLFSSSTSVGSSPQPFLLPPAAPLASLRPASGRSGARSSSSRGNADKLRSRQTARNVIEAPSFPGVPDQRSRGRQAENAREGGRVRLARQSSLGRLHSSLRQGLLRSGLPVRIALETSEVVSLQPPPPLFLFLPRSSYLPQIVNECVTRFRFFLRPTQTRGVAGPERPPSPSFSCLGFPLDWRLPLGVSVDLLAGQHLPCGVRPPLWRDAGDAGDPAGSDAGDGAVLPTHERETQDAGDAGRRGPTAKARSGRKRASFRRTVSSVPAVDRDALDLVAVCPPEACGAAVPLPWPLTFHFHSSPTTLARSASAGASPGEHGDERGCEKAGGGEGQTVRCPPVLLPPASSPSYEGWAAFESMFLNSLRQASYLLTGSAAAFHRLSKADELALLAAFRSADLAGFLEAIAPLQGGDPERIRGEAAGGGSERRRRGDRGMSGRHDQAAVHRLPVRLHFVTRGVEGCERESNVSWGQAVDGAEGRWGSATQAMGGSKNAHAVPLVMSMLMAAPVFAETPAGAQGDVRGLAGSGAGGGGEQDVAAASLEGERKKEGGREGDKAEEERQRRQATGETGPMQAGQAASVSLRKGDTVETSKSARRACIDEFDIREFYTLGDLLHSTLPQLFPQKVWHCLESMSRARRRSEFSADREAGAPAERRRDRSHRTLSPSLAERGGREAPADVRGALPRREEEAGGKRESSEEAWFSGSEEADAAGGSDYSEDQRESANSEWEDEEGDDAHVDTTREIVAVRSQFRSRGCRVLVQGIEPMLETPLYWLWKNASCMDLFLHVVVVLPPTVVAPVMRGNRT

>1183

MDRQGKVTNHFLPGDGENTLKDAINADKAIIQGIEVDGNTPIKTLIPTLLYPDGFLYVVV

>1184

MSEETINTIRKHVFESVVPLRISIAESPVPLCFNAPRNSTLGLFVYSKISVFLPEDTRNIWFSYDFKPVKWTLPIGVLYDVLIPSSDEFTPLRIEVKTADFPEGNIIRCESQEIASYYFCHSFKESLFLTDKNMDLTQRNAGLHQNIVKAVDQHDYETYKELFSLRFDNPISQWDKWPIRIVKSDLTIVQAYLQVEPGQTVSTILNARNLDSNEVIINGVSIKKDAPLEEVVPILLGADGFIYIVLK

>1185

MSQVPVASSQQRSVSAGNHPSSSSTSSSFAVPFSSTSATSTPLLAQGSLHAGSPTSNHLSSSPYAAAGSLPAAATPASLSHQHSSSLVGTPAILATTSFRKLVWDGTIPICVSVDPAELPPGSDATIDSTYLVVPRISYLPLIIADVRRNLLELVLEQPALNVLNEKELWFEYEGQPLRWHWQIGLLYDYHTSNPARTAIAYQSSSTNTTGLGSLRPNTPLIPQGGSDTEFSSAAQPSRLPWNIRLRLSKLPVERLHSNSGLESCKTSFMSMIKEADFVRYGSTKKVVNLRKQEQDTLWDSVVSHDYELFWSIANKLVPNAGVSGSDAAAGNGLALGRSPIAARTMSLNLGGTQEDRTNASAQRSLTTLQNESQASLAPSTLSTLTSTTSSDPTSATNSTPTSSAGNSVRSIPIRFFLPDNAPIVQEPVPPTLDDGRANTLAAVLSALFPLLFPPPPSFSSFQAPAPPLAYALVQGVRMPLDTEIAWLGSALVGPDGWVSVVVGLVS

>1186

MTALPDEAVNRGCILVQVRLHETDLSSLRVPTPYFKLVSRLSYLPVLYGDIYDHYKSYILPDSLSHNGRNLWLEHDNIPLQWHLPVGVLFDALFGGHPRVPWQLTVHFSSGTPEQHIEHLPEHIRRHVPKPIPMTGSSAVNSFKIRFLNTLKQSLFLLHGTSVRFASLSKSDQTDLMDAVVEGRVDDFWSIARRVDLTCSAIADIKSVSIRLHVYGPPHLQLLRAFEPASSSLTSVRCLLRSVLPQEVADMCEDSHDDTLPRAQVLVHGVCASLDCSVRWLALYGLYCDQCLHIVVRLPGALT

>1187

MVTADAIRRANFDGKIPIRLALAQSSLSSATPPHPQHALVSRHTYLHIGLESAVRALHEYAPLALSGHRKIVVREGGDGGEDEDENGKEADNGNDNDKSKNNNNNENNNKNQSDSDAGEGTPPMTMTTTPPYPVCWFEDVATQQPLRWQYFAGVLFDSLHAPSNHRHRQKIPWELRLHFRSYPSDTILELIDPNHGHGEGVLETIKRTYKNSLKQGLVIHHGNAREAVNMSKQSHTVLWEEGIRKNNYEAIRPILFPESSDGAGNQATRANPSLAMIPVRLSLDPTKPMLQKRIEGGSSADSMTLGSLLREWTPQRLFQTDESAQSVADRFIWTVSGISPPLCTPLIDLWKTLRHADNFLYISVTPRNDNVHNSNSNSNSSNNNKTSRAKLRSKTTIVAAKLSSAQQRRRRNT

>1188

METPTTSSLPGQAAAAKGKGDGGQLLQFEGWIVESSRSSSSPRPPVPLAAHYPTGHHQQPTTTTAAATAAAAVAAPVLPPRLLLEASAFDAPPHRRTPKYHFEVPGELLNTNTIEAFHALDKKKQLDLVAAKLDAGRGWLRRVGVWTDIESGEALREPWRLCRFLMLAFSDLKKYRFHYMLAFPALLPARPFTALPALALTDALAPAEVESLRDGYEKLRGLHSTPGEESNPAAAAAQADEAADRCSGHAFFLARRTRTTLESSTDGDQPSTTSITASTTTCIEVAPLHAHDHFWKDVPHADRLVGFADPSSRRTHPGWPLRNFLLLLVRWVCRDMTSVPVICYRQLPGKRNIDSSLVIPVLLPDPAGEALIDGRNLKVAGWEKDSEGKLRPRVVSLADSMDPQKLAGTAVELNLQLMRWRLLPSLDLARVAATRCLLLGAGTLGCNVARNLLGWGVRNITLVDNGLVSYSNPVRQSLFTFEDSLHGGKPKAAAAAHRLQQIFPGVVLPPSPPQHQHQSSACNAVGHRFSIPMPGHAVGEGQREQLVINAALGFDSYVVMRHGLWDRTAAAASPAAAPPLGCYFCNDVVAPQDSLSDRTLDQQCTVTRPGVSYLAGCLAVELLVSLLHHPLGAKAGADVDKDVSAPTESPLGLVPHQVRGYLSHYKNMLVVGHAYDKCTACSDTVVGAYQQYVFNNPTHLEDITGLTQLKQATVDFSGDWEEFDDDNETGDDI

>1189

MSTRLRTSRRSTNPLCLRALPTRFWQSTTSGTALTDPTELVRFLLVAYADLKKHKFYYWFGFPALTALADAQVVRRSTLVEEFGAEKTAAISSIFRDHIAEFAFLLTVDDAKNVHYAPFKDITTLARDGRKFLVAFVDPSSLADSPGWPLRNLLILLAHLQLHHVTILCGRNDLRTSRTPDGVGRQSLVLDVALGSLDIPASVSTPPAAVGWERHPSGKLAPRVIDLSHMMDPQRLAESAVHLNLKLMKWRVLPELDLDAVSGLKCLLLGAGTLGCYVARTLLAWGVRHITLVDNGKVSFSNPVRQPLFTFDDCLNGGVDKAPAAARHLKQIFPGVNATGHVMSIPMPGHPVLNEAEFRTTLVQLEEMVKAHDVVFLLMDSRESRWLPTLLGAVHDKIVINAALGFDSYVVMRHSRQLGCYYCNDVVAPTDSLSFRTLDQMCTVTRPGLAAIAGAHAVELLASLVNHPDRQNTPATATGTLGKVPHQIRGFLAPLETMLISGQAYHQCTACSERVRSVYTAQGPEFVRDVCNNPKALEAITGLDRLREAVDDLAWSEGEDGDADADADDF

>1190

MQFQTLTSVVEPTFWHALAELKLDVLKLDDSTVPLAATLEPGTLVQTSEAAYSSMPACISFDRASLLRGLERADAAASPSARACRVPGLLKNVNTIEDFKALDKPALFASVANTIWQSTTSGTALTDPTELVRFLLVAYADLKKHKFYYWFGFPALTALSDAQIVRRATLAGEFGAEKTAAISSIFRDHIAEFAFLLTVDDAKNVHYAPFKDITILAREGRKFLVAFVDPSSLADSPGWPLRNLLTFLAHLQLHHVTILCGRNDLRTSRTPDGVGQQSLVLDVALGSLDIPASVSTPPAVGWERHPSGKLAPRVIDLSHMMDPQRLAESAVHLNLKLMKWRVLPELDLDAVSGLKCLLLGAGTLGCYVARTLLAWGVRHITLVDNGKVSFSNPVRQPLFTFDDCLNGGVDKAPAAARHLKQIFPGVNATGHVMSIPMPGHPVLNEAEFRTTLVQLEEMVKAHDVVFLLMDSRESRWLPTLLGAVHDKIVINAALGFDSYVVMRHSRQLGCYYCNDVVAPTDSLSFRTLDQMCTVTRPGLAAIAGAHAVELLASVVNHPDRQNAPATATGTLGKVPHQIRGFLAPLETILISGQAYHQCTACSESVRSVYASQGPEFVRDVCNNPKALEVITGLDKLRDAVDDLAWSEGEEGDADADDF

>1191

MTEFETLRFAPFSSAPKVAFWQAVGDAKLRDWRLDDGARAITGAYWPGSSSGGGARLELGASSVSGGAGPEGALAAPGRLTLVNTVEAFRSRDKNAFLADAAAALADAMDSGAAWERPEVLAGFEVLAFADLKTQKFVYWFAFPALALDPAARLAKSEPLASLGGDVADRLVAEALASVAFAFDPATNTILTLAAAAAVDDAPRTLVFCVADPATAQPGWPARNLVALVSRRFRATAARVACIRATAAESVVLDVALPEGCGRGAESRGVGWEPNAAGRMGPRSVDLAHLSDPAELATAAATLNLELMRWRLLPDLDVDKLKATKCLLLGAGTLGCAVSRNLVAWGVAHVTFVDSGKVSYSNPARQSLYEVDDAAKHKDKAVAAADALQRIAPGTSSKPARFEGRVMTIAMPGHSLAGDANAEQDLQDLSAMVDAHDVVFLLTDTREARWLPTVLAAAHDTLLINVALGLDTFVVSRHGLAGQGPGKLGCYFCNDVMAPSDSSKDRTLDQQCTVSRPGLAPVASALAVELLRVDAKARPLGILPHSVRGFLTHFQTVLPTTRAFDKCSACSDAVVGAFRDRGFAFVAAVAADPAHLEAVSGLTGMKADVGDWEDDDDDDF

>1192

MDEPIKCKIRNFECQIDDSFFVELYKRRLRSYKTAEVALCITANITNGVLQLNEASFQNTFGCTGLLVCFNTFAEFSGSCDFEMVESQYSILTHPLEVDFDNNDLNYYTSKLSKFAIFTYVDLIKLVCHYRIIPLGNFNFMGYTSQKDGIDVITTAMEKFWGFEKIFLLKNKKIKSLNSLNILTNPSKLDATIQLLVISDTESVNSMSLHLANVLSYVCNRCDLTQLYVYFANSNGVEFVKLIVKYSERWEIVNSIICGTRVLNNNNGIDMSDLASNLHFELALWRIIPELDRDKMGNLSIAILGMGTLGCAIATQLLNWGIRHITLVDSGRVSYFNIGRQSLYTLDDAKNGKLKVYAASDNLRKICPNLNVKGYNLDIPMLGYSIYLSDFNKLERSVNLLCDIVCGNDVLFMVTDSRESRWLPTLLISHRNWGNSPVGVTNKGTLGIVASIGFDSCLVSRQSFRGFNGSCYFCGDFVAPTNTQITGLSDEICTVTRPGIAPICASLAVELVAALTQSHLSFSAMHGDSSAKSCLGSTPQTIRFNLSNYSASTYCNERNPKCICCSSAVVEAYDEGKLDFLKQVIAKPDLLVALTHLDKMDICDDVITL

>1193

MASESPPPSIARFQPLASCVDVSFWHTLSQKKLEEFKLSTESVPIVGTFGPAPPVVKSSASVPARISLGPNAFKPEELLQGGEYPFWGQLFNANTVEEFKAFNKKRMIDDEGKALWKDIESGACTEDPSRLNRFLLISYANLKTHRYYYWFAFPALNPAKVLTVEKPMQPLSGRFSPEVIGQIFKQHARKADSHFVVEVPSEKSARAREGAVKLLDLKAFSRMPPSARKGPSSPIYLFAFADPATLKEYPGWPLRNYLMYLRYCFPDLDVIEVCIVRLRDGPAKSAAAAAAAAAAAAAAGGATNYGEYTTTGWEPNAKGRLGARLVDLKSVFDPKTLARTSADLNLKLMRWRSLPSLDTEMLKRTSCLLLGSGTLGCNVARTLIGSFKKSSSRRDRIFSPRLTTTTTSVGGGVAAVVVFIVAYCFSAFVWFFSVCPNQPYPYFPVSRMQESRWLPSLIASAHNKLLINAALGFDTFVVMRHGTRREEEKKREGRRLGCYFCNDVVAPTDSLSDRTLDQQCTVTRPGLAPIASALSAEMAISLLHNTSVGEGGGGGEGELGAMPHQIRGSLRQFSLLSITGNAYDKCTACSDVVVEWYRKKDWDFVVDALNKPAHLEEVTGLAKMKKEAEDFDVDIEWDDDEDDDA

>1194

MTDDKNTLKYKDLSLHIAADVWHELERVKLHDWKLEEPDAPVRLFIPNAPIVGGAAAAGQQASSKKLSMASFVPEGLRNAEATLENHHSIPGILKNFNTVNQLTDLDATQRQALLTAAAQKLLLKDLVDRNSPWWTKPKPATTTTADKDDPFYFSPVVSVVFTFADLKTHEFHYCAAFPTIDLRSIKSPVTVASRLTCSVGIAKAIGGDNTTTAVLGHVKSVRLNLRAPAGAPFVIDTKNGAILPFHSSTVTPADLASGQHVVAFLDSVGSGEYPGWAARNIITAIRLTHPATTTFQLLALRGPLAEDLGDDSIMFSCTCNPLAESVLSRLAPNSEDPLPSIGWVEKSITKINMGGVMDSVQLATSSAKLNLSLMKWRMLPELQLEPLQGCKALLLGSGTLGCNIGRHLLMWGVTHLTFVDRGNV

>1195

MTDTKWQMLKFQPWNSAPDVSFWQTLASLKLDKFQLDDQAQKITGYFTPGRSVDVPARFTIDKSSFRQAETPFENHRYILYSKNIHEKSYLKPNNALKPSDLDRARYEWHAPGLLYNTNTLEAFKKLDKTKILNDAGARILNLVLHTTDLSIEYLNHFVLLTFADLKNHSFVYWFGFPALSPITSFQYHLPALPVTSVFSKTEQGTILQHLLNLREYNEETQVHTTSFPSFFIVERHVQSPQSSTIHVVPVKTWTPHDTSTTHLNTFFGFIDPCPLTTNPGWPLRNYLAYLTAHTNLDLSHPLQILSFREHMPPFKTIPQDFEWTKSLVFQVQSTNSFMANNRSRESVKIVGWESNPRGQLGPREMQLQRFLDPIRLMETSVDLNLKLMRWRQVPSLNLSILAQTKCLLLGAGTLGCYTARSLLSWGFRHITLVDQSMVSYSNPIRQPLFEFQDIGKPKAICAANALQRIYPLVKAHGVVLTIPMAGHASTNLPDMTTTLKTLQDLITMHDIVFLGTDSRESRWLPTVIAASTKTLVVNTALGFDSYVVMRHGVYTDLNQIALGCYFCNDIVSPRDSLTDRTLDQMCTVTRPGLAPIAAALAVELVVAVLHAPEGKYVNAMKPNEPMAPMAFIPHQLRGFLNAFHHISITGEAFAQCIACSRLVVQAYETNAMELLSHACNTTAYLEKLTGVQDLTNQVDAMTFEVEDSEEDEEMM

>1196

MSNHSDQIVKKYILNQSFVESSFFTKLSELKLEKYKLDSSYIAIHGFQTHPTKLNKFNDTPVLNLDQSSFDDTLNDSRINIPGELFNVNTIEEFKSLDKLKLLNTWGQNVYSEVTNATSFDYKLFNKFYILTYSDLKKYKFYYWVAYPTLSNTWTVESESQETDTTITQLVETELDNEYGQFFQYYGGKLHKSVQADKEHTFVFIDTCLSKDRKPSSQLKNYLYFIAYKGIKEIDLVTYRNNNLSFTQHLKLDAFTDSPKISGWERTNQGKLGPKLADLGSLINPLQLAEQAVELNLKLMKWRIAPDIDLEIIKKQKVLLLGAGTLGSYVARALLGWGVRSITFVDSGRISFSNPVRQPLFNFEDCFSDSGQGEYKALRAAENLKRVFPGVDAKGICLAVPMVGHPVTDEHKERENYETLVKLFEEHDVIFLLMDSRESRWLPTLIGAANEKIVINAALGFDSYLVMRHGVTNQKDRLGCYYCNDVVAPNDSLSDRTLDQMCTVTRPGGALMASSLAVELLVAILQHPERNLAPHDAETKFGNIPHQIRGFLHNFQQTKLFAPSYVHCSACSPRVITEFKQEGWEFVRKCLDDSQYLEDISGLTKVQQEAELAAKQLLEDLSLDDDVSNDIDEDSEWLS

>1197

MAPLQFAPFSSAVDASFWHMLSRQKLDVYKLSDEPHPVLGYYTMGEHPDMPARVCVNQAAFAPESAAASSSPSSSSAKAAAPTDLEAFFAQLASAQVASLPIPTQSFPALGTLFNANTADAFKEFDKKAMLEDMAKKMWGCITSGAAIKHPALLNRFLLLCFADLKKYHFYYWFAFPALYVGELESVAEPAPLFDTFSASQMASFQASHAELSRTHPDQAAFFLVSKVSDDKLQAHALADWAAAFPAEATDDITIGFADPCALAKNPGWPLRNLLLLLSHWKGYLNRNVRIICYREVSRGGELDLSQSIVLTVRLTPLSAPDLAEGPKVVGWEKNAKQKLGPRIVDLSSSMDPTRLAETAVDLNLKLMRWRLLPSLELEKISSTKCLLFGAGTLGCNVARALMGWGVRHITFVDNSRVSFSNPVRQTLFQFEDCLDGGKPKAAAAAAALKRIFPSMVSEGHNLSIPMPGHSVEGEEPIRTAKETVAKLEALIDEHDVIFLLMDTRESRWLPTLLAASRHKLVLNTALGFDTFVVMRHGIVPSPDHPATHKQLGCYFCNDVMAPSNSLKDRTLDQQCTVARPGMSFLAASLAVELLVSLLHHPLGASAPAASTTDRASHNTEQVSTELGLLPHQIRGFLSQFDNVLPIGHAFNRCTACSNTVVDAYRKEGFSFLQKAFNSPTFLEELTGLKKLHEETEAALADWDEDDIEPVDDNDM

>1198

SDLQHAALQSVLDVSFLAELTDLKLNVLKLSEEPVEVVGYFSPNRYDSVPARLTLDVSSLTPAASSRLDCHAAPGRLVLYNTIEGFRGADKPALMRRVAAEVWADICSGAAESEPWRLTRFLVLMHGDLKHYKFNYWFAFPALKPPAPFTSPELPPTRLADALPPAAVEAVSGAAGMGQRRTLGVRGMRQHVAMGRARRTPHTLTRRLPPLRAGGRHVLVVVSDGSHLPDCPAWQLRNLLLMAAVRWRVPELRVCVLCLRESSRGGGRLDPHRSLLLHVCLPSLPSPAPTPSQAPVPPPQAPTPCPDAVGWEPDAAGALRPRFLDLGPHLRPEAQAEQAVDLNLRLMRWRAAPELDVGAMAATKCLLLGAGTLGCAVARTLQAWGVRHVTLVDSGRVAFSNPVRQSLFNFEDCLGGGRPKAQAAAEALQRIFPSAVTRGVDLSIPMPGHPPAGAAQEEAMREAAAQLDGLVSSHDAVFLLTDTRESRWLPALLAAAHGKLAITAAVGFDSFLVMRHGAPPGAANAPAAAAVGGGGGGGGSSSAAATASGGRRLGCYFCNDVVAPANSTRDRSLDQQCTVARPGLAPVAGALATELLAAVVQQREG

>1199

MQSKVDVTFWSALGDLKLHRLKLEEGPEPLRASWSPSNHAELPGMLTVSAASLAGQPGAEGSAAAAGAPLAGAFPATGSLYVLNTMERLASFDRKAAVAEVCVGMWAAIHSGAAERKPSLLLGTVLLAYCDLKHYKYRYWFAVPALQPPQPFTLTATPTSMQQALGEQAADLAAAACSDHLAAVGQPAWLVAVGGGGEVEAAPLTDWHRLQQGGVSGGQRRLYLTVADSSSLAEHPGWPLRNLLLLAAARWGCRQLGVLCLRERRGRFDAAASLALAVALPDLPPGFCPAPLGGWESNERGKLGPRGADLGPAMDPRLLAESAVDLNLRLMRWRAAPSLDVGAIAATRCLLLGAGTLGCSVARTLLGWGVRHITLVDNSRVAYSNPVRQSLYCFEDCLEGGKPKAAAAADALRRIFPGAVARGVQLSIPMPGHPIADGELAQARRDIAALEALVGGHDVVFLLMDTRESRWLPTLLCAALGRLAINAALGFDGFMVMRHGAPVPPEAEQLPGGGGSGVPAAAPRPAPPPPSVVGSRLGCYFCNDVVAPINSTVDRTLDQQCTVARPGLSAIAGSLAAELMAAVVQQPAGAGAPAAGSPPAAAQQAAAGGEAPPLGHAPHMIRGQLGGFTQMCLTGQAFRQCTACSEAVVREYRRRGHDFLVQGLLDPKHLEDLTGLTELHRASEAALEAWASSDDEEGELGGVYAGAGAGSSLGEQRGAPTGAQPGAAAAEGEEDWEEL

>1200

MIRGSSLQQRAAPSDAESVLMSASQETILQFGQLQSSVDVAFWAELGNLKLDKMRLSEDPQQITGAHYAKLPSILNELLLDSSGPLVDMTRCGVPGELRNVNTLERFKALQEQRGALLQEAAGQIWQGIYSGAAEADPSLLSRLLLISYADLKLFRFYYWFAFPAIKPPQPIMVRAVQPLQQALGMELSEGVAESCNEWLGTGGRPLFWLMSVSDDNSASSGKHVVLAYMDHSNLQSNPGLLLRNALLMAAVRWRVGHLDVACLRLRRGKVDATASLLLSVSLPAIPEGWGKEAAPAAIGWEPNARGKTGPRVADLGPTMDPRRLASAAVDLNLRLMRWRAAPALDTAKLAATKCLLLGAGTLGCAVARVLLGWGVRHVTFLDSGRVAFSNPVRQSLYEFADCLDGGKPKAAAAAAALQRIFPDVKSEGVQLCIPMPGHPLSPGEVKQAEEDVRQLEELIEAHDVIFLLMDTRESRWLPTLLGAAKNKLVINAALGFESFLVMRHGAGPDAEDAAASSTAQSGSKGWQRLGCYFCNDVVAPLDSTVDRTLEQQCTVARPGLAGIAGSLAVEMCCGVIQHPQGVRAPAACHDPDASSSSADSALPLGPVPHMVRQLGGGFAQHCLVGQAFKQCIACSGAVVTQYREKGWLFILEALQDPHALEDLTGLTELHSSAAALAMMYSDEDSEDEVERLGPDKTDSGEQGDVKDDWLEL

>1201

MQVAPWSSFVDVGFWSELAKRKLERYRLNDEARPLVATFTGNAGKGANAPALICLSAESLDTELDGKDAVRPPYQFAAPGTLRNYNSHEEFKAADKAAPDAPPALLVERGRGDQVWEDIHSGAALAEPSRLNRFAAIAYADLKNWRFHYWFCFPALLHLPDAAYFADP

>1202

MTNTLQFKEFSSFVNISFWHELSNKKLDELKLSEESIPLNGHYTFSPSQQLDPFLCLEFNAFLRNNVTNSTENQYVLPPRSYLSHGTLYNYNTVDDFKQSPKIKLFNDASKRIWNDINNGNIDKDTSLLNRFILLTYADIKNHQFYYMFGIPALLPSQPIQQFTEKPESINIESLKSFSNQILPQYFCLKQQQQESSTTTTTSFELIGSIEEKGNQYLNECLENDLIPLVGFCDPCSLPLNPGWPLRNFLIYLSIKYPMLKKIKVLCYRGNGSTSNSILLSLELPSMGEQLIKKQQEEDAGEWSGKSVGWEKDSNGKIAPRFVSLASTMDPLKLAEQSVDLNLKLMRWRVMPSLELEKIKTTSCLLLGSGTLGCNVARSLMSWGVRNITFVDSSKVSYSNPVRQSLFTFADCSPKAKEKSIAAADALKKIFPAINANAHVFSIPMPGHSVPQSEYQSIRNTIELLENLIKQHDVIYLLTDSRESRWLPTMLSRAHGKLCINAALGFDSYLVIRHGIKDQCQNELNPSISSKLGYQGSDLGCYFCNDVIAPTDTLKDRTLDQMCTVTRPGLSMMASSIAVELLISTIHHPYGGRAKGETETDVYVQGSTPLGIIPHQLRGFISHYQTLPLFSNPYKHCTACSDYIIDEYNSKGFDFIINVMNDSSCLTKICGIDDLKNTEVNIDWDIDISDDDDDNNNNNNKEKNDDF

>1203

MTSTTGPGDALKFVAFSSLVKVEFWTELATKKLDTYRLNDDAQPIYGFYGSGHDARTPCRLNLLGESSFDSPDDEGRRAAGPGARFEECRAIGYVKNVNTKEAFKELDKPAALAAIARETWADAVESDRAVAEPELLLRFLLLTFADLKKSAFLHWFAFPTLGSQALFRLVSSRPASAAAPGVLLGGPADAASVVRGLAGLWARSVEGTGRPHCPPFFVVVKDPRPGGRDDGDGEEAVAAGLRVLSLLEFERERTGGEVGDDTVVFGFVDPCSEPGGMPGWPLRNFLVLLSARQEHITRPSPVRHPRHAGGAAAHTTGGGPSLLGRSVVLDIDLSAAPSVSGGPGEAAAAVKAAWLPARVGSLGWEPNAAGRPGPRMSDLSSVLDPARLAENSVRLNIKLMRWRALPELDVELLAETKCLLLGAGTLGCAVARCLMGWGIQHITFADNGRVAYSNPVRQSLFAFEDCKGGGRFKAEAAAAALSAVYPGARSSGHVLTIPMPGHPLTTPAEASRARRDAETLEELVSSHDVVFVLTDSRESRWLPTLLAAKHDKICVNAALGLDSFLVVRHGGSPDDGEGGETLKEDNGRKRQAEAPAVGAAASPSQMAEKVESGKPQASAASAAAASSPEARKPESEPTAPSCPPGTDATTVATAAAAASAAVRRATPSRLGCYFCTDVVAPENSSLNRTLDQQCTVTRPGLAPMAAATAVEMTVGLLHHPLRQRAPADDSAGRARGLGAAQAAAAAAAGQGGGHPLGALPHQVRGFIASFTTVTPSALAFDRCTACSKPVVAAHRSQGWGFVERACQSPSVLEEVSGLEEMRKGLDALDVDLDWGVGDEWEEEDDDNDDDDVDKKEA

>1204

MADRADTLRFLPLSGAIDVSFLTELGRRKLHEFKLSDAPVEIRGTFAPSNHHEVASPLCVSADAFDSGGAAGVPPSLCVVPGTLINANTLEDFKEWDKAELLRKAAAAIWQDMCSGAALAEPHRLCRFLLLTFADLKTHKYYYWFAFPAFALDPPPRATPPAPLASLLDASQLDALRRGYAALPASAHGRPAFFAVRLSCGSAGEPVQVAEVGPLSRWLEWRGGAGGREQAWIALCDPSPQHAGWPARNLLLAAAAALREASPAAAAGLPSEVRLTLLCFREPVGGGGPPPAAAGEGPREPKSAAASTLYNVTMDVRGLFGGGGGGGGGTAAPPSAVGWEKNSSGRPGARLMDLSAQMDPAATAAAAVDLNIRLMRWRLMPALEPEKVAAQRCLLIGAGTLGCAVARCLLGWGVKAITLVDSGNVSYSNPVRQSLFAHKDCIGGKTKKAPAAAEELRRIFPGAEARGVVMGIPMPGHPVGAAEVDGVRADAEALDELVQQHDIVFLLTDTRESRWLPTLLAAARRKLAVTAALGFDTFLVMRHGLPPPAEEAAALGAGGLPAGYEQAAPAEAAAPPPPPPPRPRLGCYFCSDVVAPANSMTRRTLDQQCTVSRPGLAMVASALAVELAVTVLHHQHGAEADADVPAEAARAATCDESPLGVVPHQIRGALSFFRTDCMLGRAFGKCTACSATVTDAYAARGFDFLLDAFNGGDYLEELTGLAQMHREAEAALEDVGFFEDDEDEAMSE

>1205

MTAVKTLPLDLQIDVTFWHEFTKRKLEVFKLSEKAIPIYGSVEAGSNIIRLTHASFERQESCIEGELLNYNTLISFKESDKKAIFTEFSERCMKLYEENYSIVAKFILITYGDLKKYDFHFIGGCPVPKQHKVIGEIVNINNEESNDVLNKFKEKNCMVLNNQFEPLKKGDNEAYILDLSPVKETPGWTVRTLIHHKLDIIHCIRPNNSFTLKLTHLEEPLKGSSGWFTVKSTGKIATQIHHLAESMNPEMLASQAVDLNLQLMKWQLFRNLDLPAIQATKCLLIGAGTLGCNVSRVLMGWGVQNITFVDNGVISYSNPVRQSLYKFEDCIDKKYKAQRAAEMVKEVFPGMKSKGIVMSIPMPGHPIGEKEIESTKKDILLLDQLVQENDVVFLLGDSRECRWLPSMLCSVYNKICITVGLGFDSFVVMRHGDSSLDKEHKPSCYFCADIVAPTDSLSRRTLDQQCTVTRPGISYIASALAVEILISMIHHPLHSKAPTSGEGYIPHQLRGYLNTWKIEEGVGSAYSKCIACSEAIKEAYTKNGVEMVLDAINDPKTLENIVGIPQEVENDIEILTDSEDI

>1206

LNTNTLEELKRSDRQGLIAAAGAEVWAAIQEGAWLQRPGLLNRFVLLCYADLKQHRFHHCAGFPVLLPAQPFTRAGPVAPVGDHFTLEQQHALHAGVQQLRSTAGPDGSSAFLVSVLSDSGTVQVRPLAAWAVETASADRAVWVAVADPCAQPTVPGWPLRNLLLALTHHFRLEGVVHVLCYREVPGRPDVSHSVTVDVAVSAARLLREGAAQPDCAGWPQDNKLRLVDLSPLMDEEKLSSSSANLNLSLMKWRMLPSLDLARLENCRCLLIGAGTLGCNVARTLMMWGATQLTFLDYGTVSYSNPVRQTLFEFRDAKEGRPKAQAAADACRRIFPACRAEAVSLVVPMPGHPVDAARREAVQAAAARLEELVRAADAVFLLTDSRESRWLPTVLCATHSKLCINAALGFSSYMVMRHGVKGQPADQRLGCYYCSTITAPRDSLSDRTLDQQCTVTRPGISAIASALAVELLAATLQHPLGAAAPADTEQDAAASLLGLVPHQIRGQVDTFQQSLLHGRAYDKCTACSDVVLEAYAAQGFGFVETVLNNPEVLEDMTGLKQVKEEVDAMLAKMEEEAVADNGSDEEWVM

>1207

STLLQFEPLACRVEVPFWFELERRKLHEYKDGVFPVTVWGSYSPAGHQPVSSPVAVAAEGFAAPPATPVGFPIQGTLLNTNTLEELKRSDRQGLIAAAGAEVWAAIQEGAWLQRPGLLNRFVLLCYADLKQHRFHHCAGFPVLLPAQPFARAGPVAPVGDHFTLEQQHALHAGVQQLRSTAGPDGSSAFLVSLLADSGTVQVRPLAAWAAETVSADRAVWVAVADPCAQPTVPGWPLRNLLLAL

>1208

METFQQVDKNDLLNKHFLPHFFSDEKRLQTVLLITFADLKQHKVLYWYGVPALMPAQGKSITASKQEWWGWSGEEKALFWENLHLMRKNQGRLDPCFIATKTKCVSLAEFVSVRGENDLLFAFFDFSEKVEDLFKGPASMGWTMRNLVAYLCFHLNLGGQSVSILSFRGQQLRRWDVLTKPQEYDATLDHSIVVQIQVPTKDDYDWRFNSTQVNSPPEYRSTGWELNVRGKPGPRWMNLQPLLDPTHLAIQAADLNLKLMKWRMIPELQVEKLQSTRVLLLGAGTLGCNVARVLLGWGVRHFDFVDYGKVSYSNPVRQSLFTLEDCHDGGRPKAEAAADALRQIAADVVSRGHQLSIPMPGHAAVEEDAVDRLDQLIQECDVVYLLTDTRESRWLPTVMAAAYNKVMINAALGLDSWLVMRHGGGFGCYFCNDIVAPENSMKNRTLDQQCTVTRPGLAPIASSMAVELMVSLLHHPDGINASAPKSGLNPFSPTVDSESPLGLIPHQIRGSLVSYTMMTPTVPAFTYCTACSKSVIETYLNDKSATVLQACKDGDYLERLSGLQTFRAEAASKFEDTWDEDDVLDEE

>1209

METFQQVDKNDLLNQHFLPHFFSDEKRLQTILLITFADLKQHKILYWYGIPALLPVPGKSITASKQEEWGWTGQEKALFWENLHLMRKNQGRLDPCFIVTKTKCVPLADYESVRGENDLLFGFLDFSEKLDDVTQGPASMGWTMRNLVAYLCFHLDLGGKSVSILSFRGQQLRRWDVHTKPHDYDASLDHSIVVQIQIPTKDNYNWRLKTAQADSLPAYRTTGWELNVRGKPGPRWMNLQPLLDPTHLAIQAADLNLKLMKWRMIPELQVDKLQSTRVLLLGAGTLGCNVARVLLGWGVRHFDFVDYGKVSYSNPVRQSLFTLEDCHDGGSPKAEAAADALRQIAADVVSRGHQLSIPMPGHAAVEEDAVENLDQLIQECDVVYLLTDTRESRWLPTVMAAAHNKVMINAALGLDSWLVMRHGGGFGCYFCNDIVAPENSMKNRTLDQQCTVTRPGLAPIASSMAVELMVSLLHHPDGINASAPKSGLNPFSPTVDSESPLGLIPHQIRGSLVSYTMMTPTVPAFTYCTACSRSVIEAYLNDKNATVLQACKDGDYLERLSGLQTFRAEAASKFEDTWDEDDVLDED

>1210

MALRHPAPASSLDASFLSRIASLKLHDLRLSLRPLPFTASFCLPHRVPQGSSSSPSQPSQERLVVAADALDLDASAIKRAVLTTGEVTIFNTIESFKELDKKQFLHNIATEMCRQIDSGEVEENPSLLVRCDVIVFSDLKDYKHYYWFAFPALCFPDPPTLSDSILSMEDVMSSDMIHQLHIGYNGLLESSGNGIPPFFIVVEDKDANTVSVKPLSHYPACAQSGKQVLFGFVDHSPLQSNPGWPLRNLLYFLNRRWSLSDVTVLCYRDFSENMQRESQAEWHSRQGRRADSDRVKETLSWELNQKGKLGPRCADLAPFMDPKRRAIESADLNLKLMRWRFLPNLDTESLSHKRCLLLGAGTLGCNVARSLTSWGFRKITFVDYGKVSYSNPTRQWLFEFEDCLSLSRSDVSLQAAADRLSRIVPNMEAEGFELSIPMPGHPVAEELQGKVLADVAKLADLIQHADLIFLLTDTRESRWLPTLLCACKSKPCINVALGFDTFVVMRHGVPDPANATDPQQIPSCNLGCYFCNDVVAPMNSTRNRTLDQQCTATRPGLSPIASSIAVEIAVSLLHHPKGCLAQADTEEEQEGTDLGILPHQIRGFLSSFSNVVVQGRPFKHCTACSAVILNQYATRGNEFLLRAFNEPNFLEEASGLTDMLREGEALMEDWEDDEVEELEP

>1211

MSNNEEILQFKEFSSFVNISFWHELAQMKLDVFKLSDKEVPINAYYSYSQAAQLDPYLCLEYNAFQPSSLDIKDLSLDKEQRPLLFKAPPKSLISNGILYNFNTKEDFKNTSKAKKLFEDLTKTIYADILSGAAEEDPSKLCQFLLMTFADIKNHNFYYMFGIPALSFSTPITTVGACQPLGSFFNENQLQSFKSGVQSLISSTSGVFVVKKSTSDQSTVELGKLNEWNKFYPNVADDAEVPIVAFCDPCNLPSNPGWPLRNLLYLLAVRHKVSKLNVICLRDQKGSLVDNSIVLTVHLPTETSTAGVADPNIIPKSVGWEKDANGKILPKSVSLASTMDPLKLAEQSVDLNLKLMRWRILPSLDLELIKSTKCLLLGAGTLGCNVARCLMGWGVRTITLVDSGKVSYSNPVRQTLFNFQDCVGAKGKDKATAASESLKSIFPAVDASGVVLSIPMPGHTVAEHLVDQTKATYEQLRQLVADHDVIFLLTDSRESRWLPTILGRQLNKIVINTALGFDTFLVSRHGQNVAHANENSSGDAKTTGSDLGCYFCNDIIAPTDTLKDRTLDQQCTVTRPGLSYMASGMAVELMVSLLHHPIQGRAPAETSTDIHSGSSTPLSILPHQLRGFLSHFNTLPLFGHAFKNCTACSQPLLDQLNSRGFDFVLEVLNDSSCLIKYAGISEQLATEGVSIDWDEDLLESSSDDE

>1212

MSAVSGKPHLTFSDLQLSIDVGFWEQLRQLKLTEWRLEEPHAALAGVIRANVSDRVFLSPANLVHLSAGSLHPSTLAQAAAENAADVVSVQVQGTVRSFNFAEELNALNLRNALISIAAKTLLGPAVALYASAEEEADAWGNMPFATLCMFTYIDAKAYRFFHWEAFPCTAIESAVLVDRLVLGASPALPFSAEAAQAMHRHGISLLRQKPERACNPFLAVCANDSVEFISFSPTAFVTATAAKGDSVVVCLFDFSDAVGSVSLPVRNVITCLRLAVPSLNTLRLYALRSGGTEKSVFVKLAFDALEESLVTSLRERLTGASFADLTRVQWKEEFPSLKASGWRKKKIESLDLGAFVNPVQRADSDSRFNLELMKWRVLPSLALDQIARCKALLLGTGTLGCNVARNLLMWGVRNLTLVDRGRVSFSNLARQSLFTFEAAKDGKTKVDAAAEAVRAIIPSAVVRPVPLTIHMPGHRIDEANADKALGEIRRLEELIAESDVVFLLTDSREARWVPTIIAAATGTPVINVALGFDTYVVMRHGVPAQTSRSNAVGEDDCRDTLHTPLGCYFCSDIIAPTDSLSFRALDEQCTVTRPAVSSIASAIAVELLAELYQHPSGFRCPAYREAATGESDQGRCRLGVIPQQIRGSVFSHTMHHLCGERNPFCTACADALLRAYREGGSEFLLQCVNSPSFIEEVCGVKALKAKWEAGMDATGWSSDEELAD

>1213

MSAVSGKPHLTFSDLQLSIDVGFWEQLRQLKLTEWRLEEPHAALAGVIRANVSDRVFLSPANLVHLSAGSLHPSTLTQAAAENAADVVSVQVQGTVKSFNFAEELNALNLRNALLSIAAKTLLGPAVALYASAEEEADAWGNMPFATLCMLTYIDAKTYRFFHWEAFPCIAIESAVLVDCLVLGASPALPFSAEAAQAMYRHGTSLLRQKPERACNPFLAVYANNSVEFISFSPTAFVSATATKGDNVVVCLFDFSDTVGSVSLPVRNVITCLRLAVSSLTTLRLYALRSGGTEKSVFVKLAFDALEESLVTSLRERLTGASFADLARVQWKEEFPSLKASGWRKKKIECLDLGAFINPVQRADNDSRFNLELMKWRVLPSLKLDQIARCKALLLGTGTLGCNVARNLLMWGVRDLTLVDRGRVSFSNLARQSLFTFEAAKDGKTKVDAAAEAVRAIIPSAVVRPVPLTIHMPGHRIDEARADKALGEIRRLEELIAESDVVFLLTDSREARWVPTIIAAATGTPVINVALGFDTYVVMRHGVPGQTSRSNAVGEDDCRDTLHTPLGCYFCSDIIAPTDSLSFRSLDEQCTVTRPAVSSIASAIAVELLAELYQHPSGFRCPAYREAATGESDQGRCRLGVIPQQIRGSVFSHTMHHLCGERNPFCTACADALLRAYREGGSEFLLRCVNSPSFIEEVCGVKALKAKWEAGMDAMGWSSDEEWAD

>1214

HPTTGEHGRGTAAALQCCTIVASRPAHTAAVPWPASAVDVQIFPQNPRGAFSAPRFHVSRNRRPLGTMSALVQFAPLKTAISVAFWHALARRKLDEYGLSDAAVPVRALWRSGSALLRLDYDAFDPAAPAPPAHHSAPGALFNTNTLDDYKKMNAKRMLEGEAEKVWRAVASGEWESDPGCLTRVLVACHADLKRHEFRYIAACPALRWASGDVVAAGPPRPLSSALSPERAAELRAVLEGCPVAVVPAEGPVVRMAPGAWRPGAALAVADACEEREHPGWPLRNVLLAMAATHGLRRATVLCVRPSGGLVLDVELPEAPQPSGAAPQAVGWELQDGRLQSRVVSLAHTMDPLRLAETAVDLNLKLMRWRMFPALDLDAISATRCLLLGAGTLGCNVARALLGWGVRSFTFVDNARVSFSNPVRQSLFTFEDCLDGGKFKALAAAERLKAIFPSVNAEGRVMTIPMPGHYVGKDDEARARAATEELDELVRSHDAVFLLSDSRECRWLPTLLGTLHSKLVLTVGLGFESYVVMRHGVPAAPDRVGCYFCSDVVAPRD

>1215

MTRETTADVDDDDLGAAEVLRFEPWQSAVDPGFFAELARRKLDSIGLSEAPLRVTATYAPAQHALVSSPASMARASFAEDGDDAAARAADARAATRALMPGTLHNVNTFERFKTFDRARVLADAAGALWSQIASGAAEEDPSLLNRFAVVAFADLKRWSFYYWFAFPAMKLTDPVKVLNPGVRSLTAAWGEDVAVKAAAACDAWLKGGGAFAWLYSRSTGACYSLTAWKALTGGNAASADDVALAFADACCAKTHPGWALRNLALLAAARWNVERLRVVAARSPAGRISADACLHMTLALPAIAADAGALPPGPAVGWELNAKGRAGPRCADLGASMDPTRLATQAVDLNLKLMRWRLLPELDADALASTRCLLLGAGTLGCAVARCLLGWGVRAITLLDSGKVSNPARQSLFEFDDCLDGGAPKASTAAARLKKIFPGVDARGVRASIPMPGHHACDGDDEETKRVLKDVDDIDALIETHDVVFLLTDTRESRWLPTLMCAAKNKLLINAALGFDSYLVMRHGAGRRLGCYFCNDVMAPGNSTRDRTLDQQCTVTRPGLAPIAGALAVEMMVALRHAPPGPDPGGGGAPTATGIRLPASTAPPPLGADPPTPLGIVPHQVRGQLAEYAQRLFAAPAYPKCTACSRVVVNAYRGGGGAGVGEMKTEAEAHPTFLEDATGLTEMRAAADDAEWVGSDSDADDF

>1216

MSDKLLFQPFTSSPDILFWKVLSEKKLDVLKLSDDAVGIVGHFCSGTEFERAHSLFFLDENAFEASKAPPEHHYAVPGSLINTNTKDAFLKLNFKQILEDAGKKILKGIASKEILEHPEELSRFTLCSFADLKEYSYMYWFAFPVLNHPLLKMTSKFESISNFYKEDEIEKLLNQVNGISSCYDPSTLEIFPISSLPMLIESVKKGERKTIGIAVKDPGSSNAYPGWNSRNYLYALSYYGWESGLKEVPVEIAFIRECDPRKATNITAVASSSATPSSESDESAKNSPTFLVRGVIPALESDPCAEGAAIQCIGWEKNQHGKFKARMVNLREQFDSRSIADTSVDLNLKLMRWRLVESLDLEALKETRCLLLGSGTLGCYVGRALAAWGVRHITFVDRGKVSFSNPVRQPLFQFEHCLGGGQPKAETAAEEMKKILPTIDTKGISMTIHMPGHPVQPGKEEEECRRDVLQLERLIRSHDVVFMLTDSREARWLPTLLCSYYGKVAINSALGFDTLVTMRHGHVRGAERITVSPLEKEEEGAQKCTVNPERLHRKRRPHLGCYFCSDVVAPRNSLRERTLDQQCTVTRPGMAPIASAVSVEMMVCLLHSRKKNMAEAGDAQEDEDEIAKKLEELKISKESDEAKKEGEDEELVGDEDIIAEEDKEELKRKEILKAADQDEDEGTMKFVPHQFRMFMNKFQTGLYHFSSFEMCTACSSKVRAEYAKRGFDFLLQAFNDPEYLERLVGISQMMNDDVQDDDEDSVREVNDDGFEIV*

>1217

MLLQFHPWASAVEAPFWQVLTQRKMDVFKLDDSQKSVMGYYSTGQQVKIPGKETTVALPARLCLGPAAFPENSPIESSGYKNPAYTFPAPGTLKNTNTIEDFKAIDKTSLFQSVVQQIWSDITSGQAAESPDVLNRFLLITFADLKKFKFYYWFAFPALVAAPAWTSDAMQDVSQQFSTAQVDSLRGNLEQYRQTYPSNQHGFFLIRQHDVAVARLQDWETIYAGCNHDQIIIGFADPSSLDSNPGWPLRNLLALLQFRWKTTNVRVFCLREVPGKSDISSSKLFTASLEDYVPVTEIPKAVGWEKNAQGKLGPRLADLAPLMDPARLADTAVDLNLKLMRWRLLPGLQLEKVQGTRCLLLGAGTLGCYVARSLLGWGVRNITFLDNSKVSFSNPVRQPLFDFEDCLEGGKEKAATAAANLRRVYPGVNATGISMSIPMPGHPVSTQGYEAAQKDVTRLEQLFDEHDAIFLLMDSRESRWLPTVLGAKKKKIVINAALGFDTFLVMRHGVGQDKETNYGAGSPLGCYFCNDVVAPTDSLSDRTLDQQCTVTRPGLSAIASAMAVEMLVSLLHHPSGIRAPADRADTDDQGAPDESALESSTLGLLPHQIRGFLSRFKNLLISGQAYDRCTACSDSILEAYDSEGFAFLLKVFQDAKHLEDVTGLTRMKEESENLDMDWDEDDDEMFSEDD

>1218

NGTLYNANTIEEFNNYDKKSILRNVGLETIYNKIVNQEWLKNPNELTHFLLLTFADLKQHAFHYWFAFPSLSLSDMEIKYEKSELLNEEIQLKLQNELLTFKSNHSKEEHGFYLIIENENSYQIKSLSEFENVINGNEKYYLGFSDPSALNEYPSMILRNYLLASYLTFKKDLFNVIAFRQDQSLLLKNVKITTSIENIENIQELKDKISVIGWEKNTKNKLGPRFTNMGSTMDPIKLAESSVTLNLQLMKWRMFPTLNLDKLGKTKCLLIGSGTLGCHVARNLMAWGIFNITFVDRTRVSFSNPVRQPLYEYEDCLNGGKDKASCAAEHLRRIYPNVNVQSHSLDIPMPGHFITDRQKTLESYKKLHDLIEEHDVIYLLTDTRESRWLPSLIGIRKKKIIINAALGFESYLVMRYGVYGDNSNDNNNNDSTNENNDEFKEMTSKRLGCYFCNDVVAPVNSTKDRTLDQQCTVTRPGVSAIAGSLAVELLVSLTHHPEYKYASAYNPTSQNNDGHVESDLGVVPHQIRGSISNYQTNLLYGSSYNQCTCCSPIVLQNYAKDDFNFLERCFNDSKYLEELTGLKQLHEQTLKLLQDFDYSSEEE

>1219

MRRPFWRCVQRTLAFKVEREVELSPATQYIFGWHPHGILLLSRFAIYGGLWEKLFPGVHFKTLAASPLFWIPPIREGSKEIYTTNPYTPETTLVLKIRKGFIRMALRYGCPLVCPLDAYEKMDRFHGEQQQVMFGYVDPGTVPDAPGWPLRNFLLLLANRWGLCEATVVCYRGRVTGPTHDPEAGGNGSHLNPLGGAQVMRGSFVLHVSLAPLASSPSSSPPGALMTTGWEMHKGKYMPRRVDLSATMDPIRLAEQSVDLNLKLMRWRLLPALDIAALARTKCLLLGAGTLGCSVARGLLGWGVRTITLVDNGRVALSNPVRQSLFEYTDCADGGKPKALAAATALRRIFPGVVAEGHVMTIPMPGHPVSSPAEEASARREVELLDKLVAEHDVIFLLTDTRESRWLPTVLGAVHDKILVNAALGFDTFMVMRHGAQPNPPLTSVAPSCSGGEDGGTARESKARTGTGKGSPGPADGDGIGCYFCNDVVAPDDSTKDRTLDQQCTVTRPGLAPVASALAVELMVALLHHPLRHRAPADGGEASGLGHRFLDDPDRPLGILPQQIRGYLAHFTTILPVTRAFQHCTGCAPAVIEAYKREGAGLVLRVCNEQGVLEEMTGLAQMRRETDALELSWSEDEDEEEEEELGGGGKEKEVEMGEKEQKGREKPTQEWNVKEEEKNSGGLDLALEELAIEHAGIRRTGGNCE

>1220

GVVHNFNTDEAFRAFDKRAWMDRLGLQVWRDIMEPGRDAAVLRDPGRLARFGTLCFADIKRNVFIYWFAFPAVVSAPPFRHLKAPAPLAEAGQGEGNPFFSGTECSLLYQGLLAYRQRRFQETGDASCPPFFLILRSTTPP

>1221

MLRGHQTECFHLLVRLSHGGQCPALPALEGARPFGRGWARGRKPIFFRDRVLSSVPGLAGVPSTSLPGDGRRVLSSLLPHPPFHDPPPQTADDREGGSSSLSLEVCPLDAYEKMDRSHGEQPQVMFGYVDPGTVPDAPGWPLRNFLLLLANRWGLCEATVVCYRGRVTGPTHDPEAGGNGSHLNPLGGAQVMRGSFVLHVSLAPLASSPSSSPPGALTTTGWEMHKGKYMPRRVDLSATMDPIRLAEQSVDLNLKLMRWRLLPALDIAALARTKCLLLGAGTLGCSVARGLLGWGVRTITLVDNGRVALSNPVRQSLFEYTDCADGGKPKALAAAAALRRIFPGVVAEGHVMTIPMPGHPVSSPAEEASARREVELLDKLVAEHDVIFLLTDTRESRWLPTVLGAVHDKILVNAALGFDTFMVMRHGAQPNPPLTSVAPSCSGGEDGGTAGESKARTGKGSPGPADGDGIGCYFCNDVVAPDDSTKDRTLDQQCTVTRPGLAPVASALAVELMVALLHHPLRHRAPADGGEASGLGHRFLDDPDRPLGILPQQIRGYLAHFTTILPVTRAFQHCTGCAPAVIEAYKREGAGLVVRVCNEQGVLEEMTGLAQMRRETDALELSWSEDEDEEEEEELGGGGKEKEVEMGEKEQKGREKPTQEWDVKEEEKKSGGLDLALEDLAIEHAGIRRTGGNCE

>1222

MKGLLKNTNTIEDFQKLDRKALFEEVSKKIWESILSGEAIKCPALLNQFLLISFSDLKKYKYYYWFAFPALIPKNSYYLNNVTPLHEYLNENENKTIIAFADPCVLPNNPGWMLRNLLMLIKKKWKISEVTIMCYREIAGENTDLSQCKLMDIEIPEEELTDDLPQSVGWERNVKGQFGPRLANLGPTMDPNKLIESAVDLNLKLMRWRIMPSLDLEKISSTKCLLLGAGTLGSYVARSLMAWGIRHITFIDNGSISYSNPVRQPLYEFKDCLNGGKPKAETAAESLKRIFPGIHSKGYKFTIPMPGHNEDKKITKENVELLEKLFDEHDAIFLLMDSRESRWLPTVLGAVKEKIVINAALGFDSFLVMRHGAKSIDDDSLPTNHGSKLGCYFCNDIVAPIDSLKDRTLDQQCTVTRPGLSAIASANAVELLVSIINSPDGINSKADSSSSPFDETPTPLGLIPHQIRGFLTHFNNLLVIGQKFSQCTACSKTVIDEYKKDSVEFVQKVISQPDYLEHLTGLDELHKEGELLNCDWDDDDIDSL

>1223

MTSADARARALAFAPHASAPDGGFWARVAKMKLSETMLRESGISCACRVRAANRAEISGAHALDHESFDGLNASDGGDGGGGWVARGDARLLNTREGVTTFDREAYLREIGEEILRDIESGDAERDPSRLMRFGMIAYACLKSWSFTYWFAFPAATSAEFTITSTSVEKMRAEDEGDAMRAQACDNWIASGGAFAWLLDEDGASARPLADYSNIVAEGRRPTLAFADTCGSATHPGWALRNLAVLASASWEASELDVVCVRTRKGRVVPEACVKFTMFLPKFDKDAVKFVGWERNTRGKMGPRTVDLGTSMDPTQLASQAVDLNLKLMRWRLLPELDQDKLASTKCLLIGAGTLGCAVARTLMGWGVRHITLLDSGRVSYSNPVRQTLFEFKDCFDGGAPKAEAAANKLADIFPGVNSRGITMSIPMPGHSVSNDLKESVFRDIDALETLIDEHDAVYVLTDTRESRWLPTVICASKDKLCINTALGFNTYVVMRHGCGVDADSDESRLGCYFCNDVMAPANSTRDRTLDQQCTVTRPGLAPIASALAVELMVALLHTKDGSKTAPPTRNADDSEPSPLGVVPHQIRGSVAGFTQTMFDAPAFPRCTACSRAIVNRYRADREGFLISVFDDPKTLEDATGLTELLGAVDADDAEWLDDDDDF

>1224

MQFIPFSPLIDIGFWSQLSKNKIEIYKLDDGERSLLVKTKINPYPEKTSQLYLDIYSFQDEITINKSGPFEVYSRIQFQNYNTIEAYQEFDHLNYVQQTFKKMIESFSKEEKPNLFPARMSIFADLKKYLFYFKLVVPQFQVENIQNIIQKNLTDYLGDQMPQFQQQLSLIIQQQQKEISNTSFVVLRKDNLQYVQFEDYYKNKSEVVFLYFDSFNQAQINGQFNNFIAFLLTNNSFKDQLNNVKIIVIKDALTINKNQFQFKNSIYVELNLSESKITELNGQYKAFNIEGYLQEKRIDLKSFMDEQSLAKEAVDLNIKLMKWRLLPDLDLDKVQTQKVLLIGAGTLGCQLARNLIGWGIRKITFVDYGKISYSNPVRQSLYDFEDSTKGGRPKAEVAAEKLKKIFPDIESEGYQLQIPMPGHFVTELQVQQTLESFYKLSELVSTHDAVFLLTDSRESRWLPTVLSNAYGKMCFSVALGFDSFLIIRHGISLKKYNPEIHGERLACYFCNDISSPGNSMKDRTLDQQCTVTRPGLSFLASAYSSELFVSLIHSPLLDGTPASDNPDQLQQTDLGILPHFLRGQLSDFEVRIFYGRAFKHCVACSQQILDALEKNPQAFLLEALNRPDILQDISGITEELTQNKQEIHEIQDIDGDDECSVLQIVS

>1225

MADAEWQVLKFQPWNSAPDVSFWQRLTSLKLDKFQLDDQAQSITGYFTPGRSVNVPARFTIDESAFPSADVNRDTDRARYEWQAPGLLFNTNTLEAFKTLDKTNLLRDAGEKVLDLVLTDESVSIDQLNAFVLITFADLKKHSFLYWFGFPALSPPSAFQYRSPPASVSSVLSAKEQVQALRGLLKLRRVNSATGAVEGNFAPFFVLERLANSEQSVRVSEVLTWRAADHSAEDVVETLFGFVDPCPLKTNPGWPLRNFLALLTALPAEKVDCSRPFKIISFREHVHQFSEVPDDFEWKSSVVFEVKSDQSFMANGRSRADVRTTGWETNARGKMGPRVMELGGILDPIRLAETSVDLNLKLMRWRQLPYLDLELLAQTKCLLLGAGTLGCYTARSLLSWGFRNITFVDNSTVSHSNPVRQPLFEFNDVGKPKGECAANALKRIFPLVNSEAVNLTIPMAGHALSSPQLMEEAKNGLETLEQLIEAHDVVFLGTDSRESRWLPTVIASSKKKLLLNAALGFDSYLVMRHGICPESGDQTKPSLGCYFCNDIVSPRDSLKDRTLDQMCTVTRPGLAPIAAATAVELLVAVLHSPQGKYVSAAKPSDGSVPMGYIPHQLRGFLNAFENMVITGEAFDKCIACSSKVLDAYVADALGLLEKACNSTAYLEELTGLNQLTEEADNLVIDLEDSDEDGDMI*

>1226

MKKKFEEENKPSYILKHNNNEFKIDISYFTQLHEHKINIYKLQSNYVNLCSSTYVNKIKLGFKYKLLNRYLIEFAHPFIHVRTIEINKKSFLKYENFDNEDEKNNMEPNDCTKTIENERNHINNINDGNKKVQKIWYIMNNYRNNYLGVLLNFNTLEEFLKCNKDDHINYTLEPLKCYINNEKNDICKDMNLYIHDNIYDDTFWEYKENCLTVLEKINKYVILSFFDLKKYICYYSIANPIIKPKDNYYKLIKNSTRYFFYIDSKYVYINTENRHINIIDIFYLSYKIDDYFNNYKMFLNTNIFLLLKFDNIPLHTMNNQDYYDEYINKLYTNIECEEDQKSKKEFYQINSFYKLFEYLKLNDISQNSYHPMGNKSFNNHYNNNSSMLHKNYDMVILPINALSELKEDIKNSKDKILRYIKKDFFDLYICFIDINYIFNSLSWDFRNLLYCLTLKYKLYDFQIDVLAFRDISLLRQQYVGTFKSQEGFIWSYPKVVMKRGSINNPRNYNDEDKNNDNNNYDDKNNDNNNYDDSHNNNYDDKNNDNNNYDDSHNNNYEDSHNNNYDDSDLHKDIDMDKDKNNSFHYNPINNCLSHQDVSFCSVTKMCKVNYNSIKDCKNDWRDDLTNEYSHDMNPIHEDIEHSSSQYENNMSVNNTYKKDNRNIKHNHNNIYHNHLVKYILNSSLFQVTVPDKVHFIYDNGSNYVDINLNGKKDDSLNKQDIHILEKKKEGDTCIINSYLKSFSDEKKNDCIDVSSNLGFSINIRKEDNHFTTRVKYKDEEMDVLHISEGDENENNMNNATNNNINNNIIKNYKTFCCDNKVYDILCGWKYYEDKKKEKKSIISIINLNDFINKDTIQRISLELNIKLIKWKILKDLKFEHIKKLKILIIGLGTLGCMVARNCVSWGIQHYTFVDNSRVSFSNISRQYLYTLEDAEKYGNIGEYKCVAAKKNLLKICPDLNITAKVMDIPMPGHLNYLNENLEDTINELDNLINNHDVVFLLTDSKESRYFPCLMIAEKQYNSLKELQESVNHNNNNNNNNNSSSSSSGSNKFRKGDNVLCEEENMITHEYIENIKCTKIMDKSLNNILLYEQNNNIYKSLNNIHMYDRYQEIFYNNILTSVKRLCKMPPLGITVAISFDSFVVLRHSYLYFKGACYFCNDMHCPSDSLSYRTLDEKCTVTRCGISNISSSIATELLLALTQHPLYFFAPHIDRDQYIYNYDNDMNQKKNSDISNIFTSCLGATPHIMNFNLANFTIKKIFCEPFEKCMCCSERVILKYQEDKMDFIRNVIRDSSILERITNMDQLKVEENDVIILE

>1227

TGSIVQNWKEFMEKTRQSACLLQRRDCTFHSLSKWKDIFGSQNTSDSGSTKTTDEITSSASISSIGSLSSSDLQSEGITDKNRNRNDNNNNNNNNNNKNIQSSPVLLCYVDGGSLSHYPSWHCRNLLLMARMIFNVTCIDILCIRNVPNQLCDSIVLQVTITGGDDIMNYDLIHNSQDIQKIRAVGWERNDKNKLGPRKLDLTQQLDPSSLAQESVDLNLKLMRWRMVPELELEKLFGLKCLLLGAGTLGCNVARNLLVLHCCLSNTRYIVMFLPPFESDGCRQGIAQNFPCCGMCVRILYTFICCNCTECNNNNNNNNTNDNNSNNDNNNNNNNNNRMHVASSEELKSTQEAVNALEKLIQEHDAIFLLTDSREARWLPTLLAITHGKDNNNNYNNQQIKICINAALGFDTFVVMRHGNILPDKLNVLKKVVEKVTVINSQEGIHQSEQKAVERKKKRHSNI

>1228

MLLFFFLYIFICQKKQTCTLSKKKKKKLSGSDQSDKKDNKELRSGEATSPTALAENDNPNQLRFQPLSSVVSPQFWTAFARLKKDILKLSEEPIDIIGTFTSGYYHKQAKLPPRIFIDEKSLGSNGSGQSDIPRFYFTCPGRLINCNTYEAFKNMDKKKLLLEESAKILKDIQTQKVLEDPSLLQRFLMLSFADLKKHTYAYWLCFPAIVPDNLEMQKHRVHAFADYFTVKE

>1229

MSSERVLSYAPAFKSFLDTSFFQELSRLKLDVLKLDSTCQPLTVNLDLHNIPKSADQVPLFLTNRSFEKHNNKRTNEVPLQGSIFNFNVLDEFKNLDKQLFLHQRALECWEDGIKDINKCVSFVIISFADLKKYRFYYWLGVPCFQRPSSTVLHVRPEPSLKGLFSKCQKWFDVNYSKWVCILDADDEIVNYDKCIIRKTKVLAIRDTSTMENVPSALTKNFLSVLQYDVPDLIDFKLLIIRQNEGSFALNATFASIDPQSSSSNPDMKVSGWERNVQGKLAPRVVDLSSLLDPLKIADQSVDLNLKLMKWRILPDLNLDIIKNTKVLLLGAGTLGCYVSRALIAWGVRKITFVDNGTVSYSNPVRQALYNFEDCGKPKAELAAASLKRIFPLMDATGVKLSIPMIGHKLVNEEAQHKDFDRLRALIKEHDIIFLLVDSRESRWLPSLLSNIENKTVINAALGFDSYLVMRHGNRDEQSSKQLGCYFCHDVVAPTDSLTDRTLDQMCTVTRPGVAMMASSLAVELMTSLLQTKYSGSETTVLGDIPHQIRGFLHNFSILKLETPAYEHCPACSPKVIEAFTDLGWEFVKKALEHPLYLEEISGLSVIKQEVERLGNDVFEWEDDESDEIA

>1230

MTSTPVLKFAPFSSSADTAFWHSLSNRKLTDYRLSKEAQPIFASFATGHRPDMPARAAVSAESFERDPAAARVGQVYLMRGDLFNTNTLDEFKKADIAAMTQTCAGEIARAITSGDVLSDNSQLHRFVLLTFSDLKKYKFYYWFAFPALVPTDTPLTQLALQPIADSLTEEQMSAIAAGVTALCASTPTLSAFIVDMRGSDEDDGPAAAVLPLTDMAPEMFDDERVLFAVMDASGVAAHTSKHVKGVEKDSKGRLVPRVCDLAGTMDPTRLATTAVDLNLKLMRWRLMPSVDLERIAATKCLLLGAGTLGCNVARALLGWGVRHITFVDNGRVSFSNPVRQSLFQFEDCLDGGKPKAETAALRLRQIFPGVTTRGIVMSIPMAGHPVSPAAFPQTMANTRKLQSLISEHDVVFLLTDTREARWLPTVIAAAENKVCMTAALGFDTYVAMRHGHPSGPSGNMTTGCYFCNDGTAPQNSTRDRTLDQQCTVSRPGVSMIAAATVVELLISLMHHPQGVTVGLTDPSDDDASPMGAVPQQIRGSVSGFSSMLFAGHPFSHCIACSRKVVDLWQSDPERLLRDAVNDPKLLEDLTGITEMKALVDNLADIDIDIDISDDEAEAN

>1231

MAPKDGGSQDSARPRNRAFESFYWANLVETGLVKESEWETFLDSLSKPLPVTLWITPTDRDADTVRRALRQYKIAAEEANTGDTEQKVSVQTLPWMSEEMGWRVDIPKTVLRKDARFKPLHQMLIDYTAKGTINRMEEVSMLPVAVLDIQKGHRCLDTCASPGSKTAQMLAMLANANFKKWGRGLESISSEEVKKCQPFLKGRIDYSSDEGCVVANEISAERAGMLVHQIARHQSLYPLVVFTSHDARYFPSIRQADGQTEVLFDRILCDVMCSSDGTLRKSPHLWREWGTKLSMELHADQLAVALRAVRLLRQGGRMVYSTCSMSPVENEAVVCEILRAAPCLALVDVREQLALRTAPGLEQWKVAHNGQLFESHAAAATAGVKLHRGFFPPEEQNVLSQLSKCLRVLPHQNDTGGFFVAVFEKTDLNPANCGYDSDRDEDGEEVERRKQLERLEQVAAAAGSAQERAKAEARRERARTSGSLARELARYRCLASTSEQAAALRSFYGLHPTFPEQLLFSRHHLQLSAEGELLQTHQGEANQLLLIARAAAEILQCGTGDHAKRKLKIIAGGLRVFEKDRFDVPERCTSFRFAQEGIELLLPYVGQRISVLKDVADTRRLLNQRDSAIAQLVSKGKEHLESLGPGGCVLLLGTMGEGDLLAVSALRTQKAVNLFVNDITMPSVPVAKDFEPVQAAPDVGFWQELSQRKLDVWRLDSSNVPVSAFYEASQASGVPAKCFLQKDAFDVAKVPAGAAKVVGELKNFNTEEEFRAFLGNAEARQSCLAEAVKHISDDIASGDALKEPARLRRLLMCSFADLKKYNYSYLLTLPALKTPRPWQRAKEPVEKLGQDVLVQVAKGLREEHFDGLCLLVRSSATGWALQPLAELPSVQVDSEDDLVLVFVDPSSEDAPAWPLQNALLLLARYRPGPRLVLAFRDPQLASGTARGYGSGGPQLRSQLLRYDVAFDMITSECRPGWSKISTVDLTRFLESKTVAANAVDLNIKLMKWRVLPGLEPERLKELRVLLLGAGTLGCGVARALMGWGCRRMTFVDSGKVSFSNPVRQSLFTHKDAAEGRKKATAAKEAVEAILPDAEVVDVVMDIPMPGHPHQSAEVLHSNVAKLQKLIDSHDVICMLTDSRESRWLPSLLVAAAQDRESPPLGLTVALGFDSFLVSRQSYRNSPAACYFCNDVTAPSDSLAFRTGCTDTACQPGLV

>1232

MPSLLFLPFASNAEVGFWKNVSEKKLQSIKLSEDPLPLQGHYTTTASSSTLSIGSSEVITSTHTAVNTKTTQSLKDTGERNVNNPLKLTAQEEAVILPPRFTLGDRGLVSTQPTESA

>1233

MCTQTSQFALLLSHTPLVPTVPVQGVLKLMNQLDHFQSVDKQQLLETEGYKVWRDIVSGRALEHPELLNRSLLLAYIDLKKFLYYFWFAFPALSLKYEMDRPGIPLSAHRSQTYVDHMLATHVRQGRPAFFCIVDTDGLDPDADGEVVELKAWEAAVDKPNRRLTVGFLDPSTHPQHPGWPLRNLLALVRFHTHARVDVIDVVCLRKNFRNGQASTERSIAFKLGHQVAVQMAMPKIVGWERNVKGKLGPRVVNLGASMDPRTLAESAIDLNLSLMKWRILPGLDLDVIKDTKCLLLGAGTLGCNVARCLMGWGVRHITFVDNGYVSYSNPARQSLFSFKDCIGGGTEKAIAAAANLAEIFPGMVTEGHHMSIPMPGHSVAQSEEAEIRQTVDKLSDLIAAHDVVYLLMDTRESRWLPTLLCQSMNKLALNAALGFDQYMVMRHGTQKSQDDVRLGCYFCNDVVAPANSTKDRTLDQQCTVTRPGLSFMASALATELMVSVLQHPLK

>1234

MTATPDTPQPILQFEPFISAVEPTFWHTLTRNKIDLYRLDDAPRELMGYYSTGQIVSARDSTVQPSVAPAKLCVSSGSFDGSCPPFTFPAPGTLINTNTIEEFKKIDKMKIFRDTAEQIWSDIVSGRAVTDPSLLCRFLVLTFADLKQYRFHYWFGFPALLPPEPTLVKRTTPIAEALSPSQIASLRVEYDALKGGDKAAVAPAFFVLHKRDNGVKIGNLSELDAGGDKAPGGEIMVGFADPSGLSSHPGWPLRNFLILLKKRWNIHTIRVLCYRESLGKGDTSASIVLDIEIPGEFKVDECPKSVGWEKNAAGKLGSRLADLAPLMDPKRLADTAVDLNLKLMRWRIMPSLHLEKIAATKCLLLGSGTLGCYVARSLMAWGIRHISFVDNGRVSFSNPVRQPLFTFEDSLDGGRPKAVVAAENLRKVFPGVKATGYNMSIPMPGHHLESADKTKADIETLRGLIDSHDAVFLLMDSRESRWLPTLLGASLGKIVINSALGFDSFLVMRHGMRVQVPMTTANSKTTNIRLGCYFCNDVVAPADSLSDRTLDQQCTVTRPGLSAIASASAVELLISILNHPDGPAAPPDTTLLPSEPTSQPLGLVPHQIRGFLTHFANLLVVGHAYDKCTACSDMVLSLYQKEGDDFLLRALASPAYLEEVTGLKQMHKETEEAAVEWDIDVDAEGW

>1235

MGKLKFHNISSAIDLSFWHILAKKKLEEMKLEDQILNINGYYSTNNNPDIAPVFRIISDSFNENAAIPSNSIRIRGLLKVFNTKEDFISSSKPPLLQKAAEQITSENLVSFILLVYADLKNFVFTYWVGFPVFNFNPVEYEELPIQEVNLQHLKVSLAEKVDQNSLKSIFGINKTGEYEENWGESDIICYLDPASSTDFPSSVLRNILGLMSQGKQFGSKKFLAIKNGLSSNPQYFTLERSKLYNIEIPETTKDFIGWEANSKGKIQAKTIDLRPQLDPHNLATSAVDLNLKLMRWRMSPELNLDILASTKCLLLGSGTLGSQLSRNLLAWGMKNITFVDSGKVSYSNPVRQSLYEFEDAKNAVPKALRAAKKLQEIYPGVQSSGYQIEIPMPGHSISSTEDHARENYLQLESLIKEHDVVFLLTDSRESRWLPTVITSALDKICLSVALGFDSFLVVRHGGCPSDDRLGCYFCNDVVAPRNSMTDRTLDQQCTVTRPGLSYMASSVAVELLVSLLQHPLRHKAKHGDSTHLGILPHHLRGSFSTFGIASYVSSAFSKCTACCVKVIDQYLNNGFEFVKMACNDPDYLEEVCELKELEANEKLLADELIEIEDFD

>1236

MKLDIWRLQNPMVQINGLVSLPMNQKNPQNISIDENSLKQQKRKVIGGLLTQELQGVFHHTNTIEEFQAGSKFESIFLESTKGILDENDFQNLNSIIIYSFGDLKNYNYFYKFALPELEGTNFPMIKSNKLKTIMNQDQMTNLSQKLLEYIKGVEQTQIVITVSQNEDNAFSVSDDIKASLQPNGYLCFFDASPLLPSILLKNILTKLNHYFQKNSSDLSPGQEKIIRIISIRDKISKLSEQVNLNESFYIEVKFVKSEDQITDLKVKPIDFSQLRTVTLTLKEQMDPNMISEQACDLNLKLMKWRMIPGIDLDILKEQKCLLLGSGSLGCQVARNLLSWGYRNITFVDYGKVSYSNPVRQCLFTFEDSHKPDNEKASIAAMRLQEIFPSVNTSGHKLRIPMPGHAVGNNKEALDELYSDLNQLETLIQEHDVVFLLTDSRESRWLPTVIAAAHKKICLTIALGFETFLVMRHGLSTQHHDNTVNGDRLGCYFCNDIVAPRNSVADRTLDQQCTVSRPALCCMSSALGVELLTSILNHPLKQGAVARENPHQCDRTELGILPQQIRGDLSSFNINVMYGEAFDKCIGCSHKIIEAYQNDRDAFILRACNEPDYLEELTGITAMLAQVRMDDIECFDFDEEMD

>1237

MDKKFEIIPFSPMADIGFWSTLAKKKLEEWKLNSDPQDIFVKFKISNFTSKKAFLNLDVYSFQQWELQLQGPVEIVIQTQLKNYNTIEEFKQINYHDLFKDLTQKQISAIDNFLSGKDENLNAFIMKLVTFADLKKHNYSYKLCSPSIKVDDFNLLEKSSFKTAFNDEQKQKAFDESLKQFLKSGKISPFFYCKEVDGGKFVFGTLREYLSELETGKTNDLYGVFFDPYNQNTGTHAYFTNLLALVLKLAEQKGIKNILSNLKFILLKDSLIYNFANKYDMKNSLVVSVDLTNAKIDAESYTGCDPNQIPPSIDLKSSLDEATLATDAVDLNIKLMKWRVLPTLDLELLKSTKVLMLGAGTLGCQLSRNLIGWGIKHITFVDYGKISYSNPVRQSLYEFEDTINGGKPKAETAAEKLKKIFPDIVSKGYQIKIPMPGHYLASVEHAIETLKDVDLLEELVKEHDVLYLMTDSRESRWLPTILANKYNKICITVGLGFDSFVIVRHGLSPKVHNPEINGERLSCYFCNDVISPGNTMKDRTLDQQCTVTRPGLSFVSSAYASELLISLLHHPLKNGAPAADEIEKLPQTDLGILPQHIRGTMGEFETRVMYGRAFEHCVACSEFVLDEYLKDRDNFLQRVINDPDYLQQVTKLSELLRDLDGTDAFIEVDD

>1238

LVRFQPFRSVTEPSFWLDHSARKLNELRLSEEGVPLWGFFGVTAAATTTANNASLGMRFDVSDPSVRRNESIRTYGHLFSLNTKESLRTIDKNKVLSDANLPSLLACCGVEDTDEDIDPNIALVPSTCIAYCDLKANAVVYWFAFPALAPKPGCGCRKLSKSFHKFRVLQEEESVKAESVVWCPPFFMLLRGDEVDGNSMQCLPLNKTQYESLTDEEREKAIFGFVDPNSTSNDIATTDFAVGWTLRNLVAYLSLRFGLGGSNATFLSYRPSLLRRIVSLLLDIKLPHATDYQWPSNDTTTNDNHSAYKCAGWEPNKSGKAGARSINLKPLVSPSHLARQANDLNLRLMKWRALPALDIERLSQLKVVLLGAGTLGCGVARTLLGWGIRNITFVDSGRVSYSNPVRQSLFGIDDCKEGGKYKAKAAAESLLAIAGPGVNSEGVVLTIPMPCHSFGGGKDGAAEIKSVRQDVERLHALIDQSDAVFLLTDTRESRWLPTVMALATNTPLINSALGLDSWLVMRHGSLSTTTSSNNTHQRLGCYFCSDVVAPENSTRDRTLDQQCTVTRPGLAPIAASMATELMIAMFHHPLGHYINGSGAYAPVDSGTSASSSALGLLPHSIRGTVVTYTMMTPTVPAFPSCTACSELVVKQYQKDRFDFVKSVCCDADGSYLGRVSGLASFREEAAKKIDDCMDWDDEDEMD

>1239

MAVQFYPLRLVVEASFWYALAAEKIDRLQLATEPVPVAARFSTGAKAPVDGRLFLSGDSLQDPLPPRPHHHHVHGALTLVNTAADFKALDKKSLFDAAAAQLWADIASGAWLDAPELLLPFLVLTYADLKKHIFRYQAAFPAIKLSPAVQSPAPASLLVLDDGGKALAAAVDAKFGTDSRPLVWLVRLSPGCEPAVEEVSEWAAFWAADPETAPLIAVADPVSSPDHPGWALRNVAAAVATTFGLSTFTLLAYRESLAAADADAPLASSLVVPVEVAPEALAAAAENRPSAVGWEKNKRGKIGSKVVDLGASMDPNQLMETSVDLNLKLMRWRLLPELDLDTIAGTRVLLLGAGTLGCNVARALMGWGVRTLTFVDSGKVSYSNPVRQSLFTFADAAAGEYKAPAAAAAIRAIFPGMNASGHVLSIPMPGHGASGDAQASVDKLVELYNEADAVFLLTDTRESRWLPTLLGAVMDKLVINTAMGFDTFVCMRHGPTDAASRAAGSRLGCYFCTDIVAPTDSLTQRSLDQACTVVRPGNSYLSSSLAVELLIALLHHPARHHAPADEPVAVGVPTEHRLGLIPHQIRGFMTHYAHKLVVAHAYDKCTACSSAVVDAYVAEGWAFVERVLTDPEVLEEVTGLAAMKAELEAGTSWDIDDFSDDGSAGASGAPGGGDRSDDSFELI

>1240

METKKENKPDADAECAAPAGFGGHEASRPEDEETENKKSKVSGTESARACPTSPLPRDEREPSTSESSCFYPPAVSACSSIHSRCASTAAVSSSTWYSSAAARPVLKFQPFTLSIDISFWQKLEEKKLREWRLQTPWVPLLAVSTPKHCMVRAPRRAESLPEGGQEWRREKAVSAATGSLSPALLRLDQTAFDSPSLCSVLPSSCASSAFASPPCFTGDVPLLGFLYNCNSLEQFKAFNRQAAISQVLQAPLLSTDACPPSASCSRSSPSSRPPSSSVVRQDTRLREEAETASACRASLGLLQLPVYEDSFWKFPPACSLPQPSVSPAADPASAPCQSSSSLASPSSTASPSSSAFPAPPESLHAPWRAESMYSALPPVALACRFLLITFIDLKAFVAFYSVALPAVRPGADFTLLRPPRSVLSCSICRKERNKETQGNSALEASEGTEAKDERRRQTRREKGEEMRENVRERGEEARKKREEEQKGDTSDEEREGVHTRPASLPSMTEDRLNRARGEDKERNRIHSEGDKSGETRSDQDGDESCFTFDEMVSLSRCIYTLDSGTALWLRGGVFLLLKKANSEKREEREEAARGESEGEREEASERPYLLLPLTALELLGPLLPDISACESGNASASRLLLSSSHLSALLSYLPGRDSGSSPSWAKSRTPASLLSPGVCTLYLCYLDPSGAPDAVGWPLRNLLLILSVRFSLYNKVLSLLSFRDLLLHRMLASSESLMPHKESGRPASSATAVPLFADDHLSKTHAAPSLQTLVFHVTIPPQRAFVGALSDPLHPSMPLCSSWSSSSPCSSSPSSDSVSSVSPVRGVDRQVAGGPESEGGRAGSPSCLQEERQTRLCPTVVSGWLRLSSASPSEATHRVASSPASSTVFGVALRRYLDGKVIQRDAVELNVQLIRWRILPSFEPRRIQDLRVLLLGAGTLGCGVARLLVAWGVREFTFVDSSCVALSNPARQSLYTYEDAVSQGTEAGNSGGVKKVDAACRRLLAIRPDLRCRGVDLEIPMPGHPRFGDEAALPGRRSLEEAHDLLSSLIDEHDVVMMLTDSKESRWLPSLLVADKSLEARRGAPLGMAVGLGFDSMVVMRQGCGGNELGCYFCNAISAPADSMSNRSLDQQCTVTRPGISCLACSVAVELLAALTQHPQGFAAPHIPSDPLASSHSSSFLSSSSQSSSSSSVAAADAGSSTSRVGGARQGKETRPSEAKRQADGFSCMGATPHTVRGYFSSFRMLSLVSERSPQCVCCSDAILARYRANRLQFLQEVIASSSVLEKYSGLEDMQRQIEARGEDADVICLSDEEETEKEEGSDKEEDAAETK

>1241

MSFQNISSLIEPSFWYELNKVKLNDKMLDETPFDIISYFQAGRSAGVKAFAFINEDSFKPKKEVHDVHFLNVLGTFPITFYLTNTKPSFKKLDRNGIMASLKAEMINNINSGEWINNPSILLKSALTVFGDLKHWQYTYCFAFPNPKLDNIKIVSKEVTPEIEYLSQQTNYSNWIYVLGPENSLLPLTEAKSDSTFVLIDPSTNQDLGWPAKILSLAIARKFNTKTIKIARLSYDSALFTVNVEDFNLNDAPFTGWNLTPKKTAHFVDLSATMDPMQLFTAATSLNLRLMKWRLCPQLDVQKLQAQKCLLIGCGTLGCNVARYLLGWGVRKFVLIDYGKVSFSNPPRQSLFTFADCIDGGRSKCEAAAKELKRICPDVEAEYYEMPIPMPGHPLGKNEYEKTRKNVELLDKLIKECDCTWLLTDTRESRWLPTLLATANEKLCISVALGFDTFSVVRCGCHGLGCYFCNDVIAPTDTMTDRTLDMQCTVTRPGIAPMASSYGVELWASIVQTKEGVNAEADADSVLGTVPHQLRCFLHSWQLLPMAGKPFKNCVACSEPIIKKWKEEGWNFILRALTEVGYLEEVSGINAMKAEMADVDCEWEEIDEDQ

>1242

MATFQQISAIIEPSFWHALSQKKLNDMMLDERPFESVAYFQCGRSQGISSFLFLNEESLSETQQDKLYSHYLNYTAKLPIQITLVNTKNNFLSLDRAGILKSLSDSIFQAIETRSWLEQPSLLLKSALTVFGDLKKWHFLFCFAFPTPSIKSITIAEKEEFQDEFENYPDWAFALNSEGKPIKLSEANSESTFVIIDPSTCDDLGWPALIFSLAVAKTFNLSKFKLIRLNYKPFKCEVNVPELLTADTKFTGWKLNKKKPFFVDLSSTMDPLSLFSSASYLNLRLMKWRMSPRLDVDQIRLKKALLIGCGTLGCNVARDLLGWGVRNFTLIDYGKVSYSNPPRQPLFCFNDCLNGGRPKSEAAADELKRVCPDVSVQFDSFEIPMPGHHTTQESFPKLKENVEKLDKLIQEHDVTFLLTDTRESRWLPTVIGVARHKLCISIALGYDTFSVVRSGSKNVGCYFCNDIVAPVDSMTDRTLDMQCTVTRPGIAPLASAVGVELWAAVTQHPDGLDAHGDTESILGGVPHQIRGFLHSWQVLPMTGDSFKCCVGCSEKVVNGYLEQGAEFVMRAVNEPNFLEDVSGITEMKATMVDEDCEWIDDD

>1243

MERLKYLMHDFRIEISFWYELEKRKLHEWRLSEPIIRSPVFGVTSSSSSGIYRSLVSLRAESLSASRVNVTRRLRDETSVSEGDPIDDHSGGTGTSLRCVHTLHDGFIQNFNTAKQLYQLPRRSALWDILKKSLLLPLYTCCKNKCEENVTECPSAEELAWEDVNFSIMALYTYADLKSHRFLYSVAFPVFDLGSPVFVQRRVKGGYTAAGSEFKGVYFPNQFAVDRVHAHLLEKLQKRPENGPNPFIVVRSSAADVKGKGNNDDGCVIFLPFSPKSMEAASNHSMPLIAFLDYSSEGSPGWAVRNIVSALRLAQPLITSFALYCVRNNDVSESVLFHCMCEPLSYTLEEVVEGVSPAKVVGWVDPESDGGAPSVHTIDLGPLMSPDKLADASAGLNLTLMKWRALPELNLDRLAQCKALLLGTGTLGCNVARQLLMWGVRHITLVDRGKVSFSNPVRQTLFELSDVNNPREEERNKAIAAAKALKRILPGVNARGVPLTIHMPGHRVDKAREEEVKAEIEALDELIRSHDVVFLLTDSREARWLPTLMATAHCKPTVNVALAFDTYVVMRHGLDPPEGSDKGVKYVRLGCYFCSDSVAPRDSITARTLDQQCTVTRPGLSAIASAIAVELLAQLYNHPLGFACPPYVQSERDATPGVSDSVAGGAVCPLGTIPQQIRGSVAHHNVYTLHGCRYEQCTACSDPIVRSYKDEGSAFVLRCINDPMYIEEVSGVKAFKESCALDSCDGWDDDVDNQ

>1244

MTSTATTPAGSRLKFKEYSPHIEVSFWYELERRKLHEWRLQEPVVPLTLFGTVNSAYTATPANIVSARRESLGNDTDAIVAGLPQNPLVGVAAATDTNAPFPIPVLLSGELQNFNTLEQLCKLPRREAIWRVLKEKLLIPLFSGKGSKTIDVNEDRKAAEILVDKVWEGINFALAALFTYADLKSHRFHYMMAFPVLDLGSPVDVKHRVNGGYAALATDHGVSYFSSRKAVDRIHAHLLDQLRRHPERGPNPFIVTCQASGHGENEEVIFYPFTFSTINKMKELSFLVVMADVSTMEDFPGWPARNVIGALRLARPSITAFALYCIRHNEVERSVVFECTCDPLSYTFEDVMTEGTEGKQNAFARAVGWTERKSADSPVSCIDLGAMMDPERLAESSARLNLSLMKWRMLPELSLDGLASCRALVLGSGTLGCNVARHLLMWGVTKITLVDRGNVSFSNPVRQTLFEMSDVINPRLEERNKAVAAAKALKRILPTVEAHGVPLTIHMPGHRIDKQREPEVIAEVERLDTLIQEHDVVFLLTDSRESRWLPTLMATTHNKPLINAALGFDTYVVMRHGLEPKEGSGGSRLGCYFCSDVVAPRDSMTARSLDQQCTVTRPGLSAIASATAVELLAQLYNHPLGFACPPYTETEMQEVHLQQPGDSKATNPTDTNGAVCVLGKIPHQIRGSVLTHYIYTLYGYRYESCTACSDSVVGAYRRERQSFVLRCVNDPLYIEEVCGVKAFKESFNLEDLDEWDVDSHSD

>1245

MTSTATTPAGSRLKFKEYSPHIEVSFWYELERRKLHEWRLQEPVVPLTLFGTVNSAYTATPANIVSARRESLGNDTDAIAAGLPQNPPAGVAAATDTNAPFSIPVLLSGELQNFNTLEQLCKLPRREAIWRVLKEKLLIPLFSGKRSKTIDVNEDRKAAEILVDNAWEGINFALTALFTYADLKSHRFHYMMAFPVLDLGSPVDVKHRVNGGYAALATDHGVSYFSSRKAVDRIHAHLLDQLRRHPERGPNPFIVTCQASGHGKNEEVIFYPFTPSTINKMKELSFLVVMADVSTMEDFPGWPARNVIGALRLARPSITAFALYCIRHNDVERSVVFECTCEPLSYTFEDVMTEGTEGKQNAFARAVGWTERKSADSPVSCIDLGAMMDPERLAESSARLNLGLMKWRMLPELSLDGLASCRALVLGSGTLGCNVARHLLMWGVTKITLVDRGNVSFSNPVRQTLFEMSDVINPRLEERNKAVAAAKTLKRILPTVEAHGVQLTIHMPGHRIDKQREPEAIAEIERLDTLIQEHDVVFLLTDSRESRWLPTLMATTHNKPLINAALGFDTYVVMRHGLEPKEGSGASRLGCYFCSDVVAPRDSMTARSLDQQCTVTRPGVSAIASATAVELLAQLYNHPLGFACPPYTETEMQQVHLQQAGDTKATNPTDTNGAVCVLGKIPHQIRGSVLTHYIYTLYGYRYELCTACSDSVVGAYRRERQFFVLRCVNDPLYIEEVCGVKAFKESFNLEDLDEWDVDSHSD

>1246

MAILKFVPFSTNIHPTFWQELSSLKIDKLQLSDDAVAIHAHYTAGKVVLDRQTGESVSLGSQISLDAASLQSDMSHDSISFRSTSCSAVTSLSAVQQSCPGIAARGFLQNFNTIESFRNADKQFIFDNTVRGIWKGLTDEQQDPEVFLTTFLALTFADLKKFKFYYWFAYPALTTNPPWEVIDDAEGVDKVWKPISTQLNEVDYSVIASWVQQQSVRRGFFLFKAAVGKVGQCGRISTYASFFDGVAESERYVGFVDPSGTSQTPGWPLRNLLAYLHARFGVEEAQVICWKDEIANAASGLTFGQTRSVFGKVRLPVSNTTDGVSTMGLELDGRRTRLTCKQNNVQLPNGVGWERNAQGKLAPKVADLGPLMDPRKLADQAVDLNLKLMRWRIMPEIKLETIQNTRCLLLGAGTLGCYVARSLLGWGIRQITLVDSAKVSFSNPVRQPLFDFEDCLEGGQPKAECAARRLKRIYPGIDAQGISLSIPMPGHPVAPIAEPQVREDVGRLEKLVDEHDVIYLLMDSRESRWLPTLLGAAKSKLVINAALGFDSYLVMRHGAPPQSAESVTGVAPDASKASKGKDAKQSWHGRLGCYFCNDVVAPSDSLTDRTLDQMCTVTRPGLAAIAGASAVELMVSVLQHELGLHAPATAGESLRQGIEDAQAIADATTVLGIVPHQLRGFLAQFNTLRVVGQAYDRCTGCSAAVVDAYRTEGFEMILRAMNEDKYLEHLTGLDKMYQEADEMEAAVDWDVQDDEDEGF

>1247

MAHPLSFEPPRVVVDISFWQELAAQKLDRMKLTTPRVPIVATMERPAPHSPASKDASRTSGQLRLTRSSFTHTSSTGEGGVATNDESGSSLLHEEWRGTLINVNTLEEFRSFDRKQLTDTVAAAIANGGAEGAAESDDYSSRRAAGDTAGGGGVRVRGLREMAPCGAGRVLVMLTFIDLKKYRVYYSIAFPVISPNPSYQHIDHQHLDQAMTAQDISAMMRQLKADHTTLKDGLFDVHVGGGNAGGAADGPWLGYVDSSTSAEAMGWGLRNVLFLLASKRRLYGRTIRILAFRDLRKVITHQADNEYRPSYQSSVYTVRFPPADAFTLPPPLSHDSRNGSSSARATCLGGWVRWPVADGHGQAPGHQQHKSTTVQQVNLCDYLDFTTIQANAVDLNVKLIKWRMVPQFDPERFNSMRFLLLGAGTLGCAIARALIAWGAKHITFVDSGNVAMSNPVRQSLFKHQDAAAEGGSGRPKVDAACEALREIRPDVDARGVVFEIPMPGHFDASAASAPSPSGSGAAASTSASIERSLHTLDELISEHDVVFMGTDSRESRWLPSFLIANKSWRAVRRQQQQQQQQTAGGGAAVGSEGEGGCGGVPLGITVALGFDSFLVLRHGYKDQSLACYFCNDLSAPRDTTSNRTLDQQCTVTRPGVSPMASALAVELLVTLSQHPHGFVADPSEKDTGSSDDSCLGACPHSIRGFIGGFRLVVGSSPQSPYCTCCSPAVHEAFEQNPIGMLTQVMHDPGHLESVSGLRVFMDKMSRVEEDVIQIDDEELCLL

>1248

SPSQSVVDVTFLSELSEFKLNVWKLSEDPVEIVGEQRQGSKSLKSARLPFLDTTAQLRSLSPCPDCHAAPGQLLLFNTIEGFRGSDKQSLMRQVSGQIWSDILCGAAEAEPWRLCRFLLLMHGDLKHYKFHYWFAFPALKPPAPFTLAAPPAPLASALPPTAIELVRRYSSTAHATTPPPTRCVLLSSAELPLLHACPGDGAGGGESPKRRHVVLVFSDGSHQAEYPGWQLRNVLLMAAVRWRVRELRVLCLRESSRGGGRLDPQRTVILDVRLPDIDTRSWPPPPPPPAAAAVGWEANAQGKLLPRFLDLGPHMRPEAQAEQAVDLNLRLMRWRAVPELEVGTMATTKCLLLGAGTLGCAVARTLQGWGVRHITLVDSGRVAFSNPVRQSLFNFEDCLGGGRPKAEAAAEALCRIFPSAVTRGVQLSIPMPGHPPADPAEEQALQQATQQLDDLVSEHDAVFLLTDTRESRWLPALLAAARRRIAITAAVGFDSFLVMRHGAPPGAANAPAASAGGQRRLGCYFCNDVFAPANSTRDRSLDQQCTVARPGLAPVAGALAVELLAALVQRGYAGEAALCAPPPSLAGMQPDASAPLGPAAHMVRGQLSAFSQVLMEGWAFGQCTACSAAVVEAYLARGWGLVREALQSPAALEALTGLSELHAAAAAAMMEEEEVEEEDEEAGEEEGKEERERGEGKGREVGGEEAKRGEGKEEEDEDDWTEL

>1249

MQKPFKEEHSLDKRREVADRIRAKYPDRIPVIVEKAPKSDAPDIDKKKFLVPADITVGKFVYEIRKHMKLSPEKAIFLFVNNVLPPTAELMSHIYAKYKDEDGFLYITYSGENTFGQ

>1250

MRSQFKDEHPFEKRKDEANRIRQKYPDRIPVICEKVEKSDIATIDKKKYLVPADLTVGQFVYVIRKRIRLSPEKALFIFVNNGTLPPSAALMSLIYDQHKDEDGFLYITYSGENTFGDDARAVEDVDE

>1251

MRSQFKDEHPFEKRKDEANRIRQKYPDRIPVICEKVEKSDIATIDKKKYLVPADLTVGQFVYVIRKRIRLSPEKALFIFVNNGTLPPSAALMSLIYDQHKDEDGFLYITYSGENTFGDGDARALEVVE

>1252

MSFKFDHPLDRRKSEAERIRAKYPDRIPVICEKADRSDIPDIAKKKRAPAPGASRERARAGRARRFIYVIRKRIKLPPERAIFIFVDNVIPPTAALMSTVYEVQKDEDGFLYITYSGENTFGDLLEELPEADL

>1253

MPSCHRDLPFGMLDIRQSEATNIKKRYPNRIPVICERYPRTNLPILDKKKYLVPCNMMVGEFKYIIHKHLCMASAMHGSKTSFDKTVYLFAGGQVPKAGAIMQDVYEELKDPDGFLYMQYSAENILGGRGSNTTHTIAIQTIL

>1254

MSDYKTENKFEKRKNEADKIRAKYPDRIPVICEKAKKSDIVDIDKKKYLVPADLTVGQFVYVIRKRIKLTPEKAIFIFVKNTIPPTGAVMSQIYKEHKDEDGFLYVTYSGESTFGH

>1255

MANKKFQSKYKAAHTFEYRKSEADKVRERHPDRLPVICEKVENSNITDLDKNKFLVPSDLTVGQFVLVVRKRVMLEPEKAVFLFIGESVPPNAAQMSDLYAKYKDEDGFLYVKYSGENTFGASA

>1256

MSSFKKEHPFEKRQAEAQRIRSKYPDRIPVICEKADRSDIPDIDKKKYLVPADLTVGQFVYVIRKRIKLSPEKAIFIFINNVLPPTAALMSNIYEEQKDVDGFLYITYSGENTFGQ

>1257

MRSQFKDEHPFEKRQAEAARIAQRFKDRVPVICEKVENSDIPEIDKRKYLVPVDLTVGQFVYVIRKRIKLPSEKAIFIFVNDILPPTAALISTIYEEHKDEDGFLYVLYSGENTFGEKLAIDISSLDFSDIPDYV

>1258

MKSSFKGEHDFEKRRTEAQRIRQKYPDRIPVIVERDDKSDISDIDKKKYLVPADLTVGQFVYVIRKRIKLSPEKAIFIFVKNVLPPTSAAMATIYEEHKDDDGFLYITYSGENTFGQ

>1259

MVGSRPPTFKQEHSLDKRKAEAARIKEKYPDRIPVIVEKAERSDIPDIDKKKYLVPSDLTVGQFVYVIRKRIKLSPEKAIFIFVKNVLPPTAALMSSIYEDHKDEDGFLYITYSGENTFGAGEQLQLPVEELAL

>1260

MARTKGFKEEHPFEKRQAEAARIREKYPDRIPVIVERADKTNIPDIDKKKYLVPADLTVGQFVYVIRKRIRVSPEQAIFMFVRNVLPPTAALMADVYADHKDEDNFLYITYSGENTFG

>1261

MTARLKSFKEEHPLEKRQAEAARIRDKYPDRIPVIVEKAEKSDIPDIDKKKYLVPADLTVGQFVYVIRKRIKVSPEKAIFMFVKNVLPPTAALMSDVYEDHKDEDGFLYITYSGENTFGC

>1262

MAKSNTPAFKQEHPLEKRKEESRRIREKYADRIPVIVERSEAGKTDVPDIDKKKYLVPADLTVGQFVYVIRKRIKLSEKAAIFIFCNGTIPPTAALMSTVYDEQKDEDGFLYIDFGLPCFHPALASCEICGGTGVFFLLTLLAFQAP

>1263

SIRAKYPNRIPVICERSSRSDLPEIEKKKFLVPMNMLVGEFKYIIHKHITQSSNNQNSGIKIPVPYEKTIYLFVNNTIPKAGSLMQEVYEQYVSDDGFLYVEYSSENAFGGYHSII

>1264

MALLSKSFKQEYSLEKRKLISSKIRNRYKDRLPIIVERAANSDVPDINKKKFLAPSNMVITNFIMEIRKHLDDSDHNEQKAIFLFVNKNNLPPSSQLLSSIYDAHKDEDGFLYICYSGENTFGSDI

>1265

MVHVSSFKNDHPLDKRREVAERIRSKYLDRIPVIVEKAPRSDAPDIDKKKYLVPADITVGKFVYEIRKHMTKVSAEKAIYLFVNNTIPPTAALISQIYERYKDEDGFLYITYSGENTFGSDL

>1266

MVSFKQEHSADKRKSEAERIRAKYPDRIPVICEKADRSDIPDIDKKKYLVPADLSTGQFIYVIRKRIKLDPEKAIFIFVNDTIPQTSALMSQVYEHQKDEDGFLYITYSGENTFG

>1267

MSYRDAHSFESRRTQALAIRAKHPDRIPVIVEKRPRDATLPLIDKKKFLVPADLTVGQFVYVIRKRIALKPEQAIFLFVSKGAWHARCSWRRRTLPPTVATCQAVYDSHKDEDGFLYMTYSGENTFGTV

>1268

MSYRDAHSFESRRTQALAIRAKHPDRIPVIVEKRPRDATLPLIDKKKFLVPADLTVGQFVYVIRKRIALKPEQAIFLFVMVKVKIEIWLMVKVYVGMLAVARGRQGRAKAAKRAAAAAEPSSASEDAVAEAELATVMASLRRFTVASASSDSDFPRTPLVPLVLGNQQPSTGLSTGSSGGCSSAEAAAMRRSGDAFLRWFDDSNTRLFGQSSDVPLPESS

>1269

MVVAWASGAGVWGRGEDHPFEKRQAEAARIREKYPDRIPVICEKEPRSDIPPVDKRKYLIPMDLTVGQFVYVIRKRISIPPEKAIFIFVNNTLPPTAALMSTVYENHRDPDGFMYMMYGGENTFGELELRPLSEVMKAVRVPRGTYFKHAVGTTGLDERQRGAIMSYNASLTQCPQRSCGNYLEDGHVGAHVQIEGHSGMWIIPTCKDCNTEAKNIADVNVYNTNEWVDLTQGDSEY

>1270

MPRCADFAYKKDHPFEKRQAEAARIREKYPDRIPVICEKEPRSDIPPVDKRKYLIPMDLTVGQFVYVIRKRISIPPEKAIFIFVNNTLPPTAALMSTVYENHRDPDGFMYMMYGGENTFGELELRPLSELLEELDR

>1271

MESQPKLPFKQRVSFEERKAFAIQLRQKKPNYVPLVVESDGTSNAIELKKDRFFIPEDSKVSDFVKVLVDKYIETDGETPISTVSVKIQTPSKAIQPSNEDTIGSLYAMYQEEDGYLYFIVYRESVFGN

>1272

MDPTFPINQPKLPFKQRVSFEERKAFAIQLRQKKPNYVPLVVESDGTSSAINLKKDRFFIPEGSKISDFVKVLVDKYIETDGETPISTVSVKIQTPSKAIQPSNEDTIGSLYAMYQEEDGYLYFIVYRESVFGN

>1273

MESGSDLRHGGMDGVVAWQARQEVRWWTTMSFKKKSAEERAAECRKVCHRFPGRHPVVLQKAPFSALNHVPKSRYLVPENALLTALVPSIRSKVLPESASRAGEAAGSGIMLLCRGGPLPLTANVQEVYAQHHDADGFLYLMYATAEETEDPLLRSALHLRCGARDLFQLVLQHRWHAWQRRPAPDTEPAAPAAPELPSPTSLPPLLPAPSEGSWPESQGSDTKTPPTSSSRSVPDLLKPSGTAVEEAAAAVQGLVGRAAEMQLQSPVEVTPWEEYREQLEEIAAADIVAKADRAAYAQYLESRSRRLADHHTEYLRRLRHQWHALQDRQASEQLLAADQQSVLQSVAASLQDQLISLEGQEAELRSRQEERSIAEYSPAVGDLAAKQQRMAAALQRATASVLEAMELWEDYAQCLAANAAARAEERRLLAAQRDAERRWEEVRRHQTDVRATKEALEAKLADLHRQTTSRLQAMHETEQQLLQHWQAAYQEQCALRDREMEALARSIREKKAWLDRLEEDLRRKEAALEEGPPWLSTAALVADVVDLERQRDELQRRVASRLQALTGWVDQQTAQQEEFAEMLRRCVRARRSAKAPTASPVDASPPEESLALSVPAPAEQAIDVVEVPEPAVVASVDTAEPAPQPPALNLDPEVPPEASQDVAGEELGRQDESPPPQPDEQTVALTTTPTQRSAPGEEPAAAPMCESLQFSDAGSPEDTSESAAATSSGVGPEDDPEGDVGEAAARPQPDRGTPAGGLQDLDDFSGDDTDDEFEEIFSEPLR

>1274

KLLSATQGSQYQLKRVEHFANCLAEVESRAEQERDQAMATRQQLQAAEDELQRLRAAELQAAEAEAATRRQLERSAAEAQRLEAARHQLQAEVERLAADNRRQNADLEEATCRTYELEAQLLELEACRDRDRSLAARLRAEHERLEGELQGASDAEGQWEEKARAMQTQIAELSSALADASHSVCTLTRRLTAVEDERQGWLTQQHDSKAEHTRALHAATQQLQQTLAARTAEWQEKERALQAQLLSLRATSQEREGQAGAQQRLIEAQLTAAQQRIADLTAQVERCQAEGAARAAELERQLQAARLQLAGHEEEAAVLALGHQQEVERLEAEAAALQNKREVLENALVAMKGALATSTAQRDDASRQGKKLVSQAKAEGLQQVKEVEARLQGVIHQLQEQLREKSRSIWAHSRQMAELRDSTGAAKAELADVRAELDRQAADLKTAWAAAERKSEEAITFQQRLAAAEEAAKANAARLDAAQADAARLRSQLAAAQAQASLCDEAQDRQAQLTQQVAELQAALEEAGSAHKTLAAEHAAEAEDWHQNRAALAASEERLRAEADQLRAEGARAAGELAAARAALVAAAAEADTGARSLQALQQEREALRLQSKLDAETFKRESAEKDLMIASAELLADERLQEVEEARKELERVRERLDVKAARLAAAERALAELKAPAEKRAAAAAAAAAAKPTKPNADIVAVLAEHPNSVPLRCTRDPASADLPPLDKSRFVAPKTMTLSGFTNHVRQKLHLAAGTPL

>1275

MWLWGSSTSKAEEHGESSTQDANIQEFDDDEFDRLSNCSGESGGFFVVTPSDDVKKLQMELSGLKSDLKTTNVQLENMKRLHCHEIEFKDRLVQQKENQMQALKKEMAEKEKKFRSEIRALEERLDVVSKEAQDQAKDNRTIQNEVADLRRFRQDAERTCQSLQSTNDLLTEQLRMRNEEFQQLEKLNSEMKSRLADQERQTHALEAAAAAQDAMMKDVRTLEESQHQQDLIELARVKRQQTDLLQANEILKEELQRAKGEAAAKSEELNRQAIRMNDFENLKSKYMSMRDELKTELVQAHEASAKMAKQLEDLQASSRTQSQEVARLRQLEMDRTSEIKRLSDELRGLSEALEGKTLQLNAAKEEARSSKAELEDARQALQATKQELERQTGRLERMQQGVEEVTRQVDSTVISSAEVNRIQGKYRAEVARIMKENPNCIPIRCVRAPNCDAAYPLLEKKKFAAPKDMTLSRFNEHIRQKMKLDQDRPLSVYIDSVVPLPSSELLEDIYSFCKGIDGFLHLKYSHVSEEAP

>1276

QASLCDEAQDRQAQLTQQVAELQAVLEEAGSAHKTLAAEHAAEAEGWHQNRAALAASEERLRAEADQLRAEGARAAGELAAARAALAAAAAEADTGARSLRALQQEREALRLQSKLDAETFKRESAEKDLMIASAELLADERLQEVEEARKELERVRERLDTKGARLAAAERALAELKAPAEKRAAAVAAAAAAAKPTKPNADIVAVLAEHPNSVPLRCTRDPASADLPPLDKSRFVAPKTMTLSGFTNHVRQKLHLAAGTPLTITVESDVMPLVSMRLGDIYEQCKKADGYLHVRYGHQ

>1277

MNKSFKEEHPLEKRKSEAERIRAKYPDRVPVICEKADRSDIPDIDKKKYLVPADLTVGQFHYVIRKRIKLAPEKALFLFCSNSIPPNAALMSTVYEEQKDEDGFLYIQYSGESTFGAME

>1278

MQVTATSPFKHEHALDYRICESSKIMKSSPNKVPVILDSENELPGLLPLRNRVKLSCETTAGQVSKAIASRIELDRNHRVVLFCGNETVQKSASMGSLYKERKDEDGFLYIRY

>1279

MVKAHKSKFREEFPLERRKAEASRMREKYPDRIPVIVEKAAGGDLPDIDKKKYLVPTDLTVGQFVHVIRKRIKLAPEKAIFIFVNNVLPPTAALMSTIYEEQKDEDGFLYITYNGESVFGA

>1280

MTHSSGFKNDHPFEKRKEVAERIRSKYQDRIPVIVEKAPRSDAPDIDKKKYLVPADITVGKFVYEIRKHMPKINAEKAIYLFVNNTLPPTAAFISQIYERYKDEDGFLYITYSGENTFGQ

>1281

MSAYVLSTPLEARVAKCASLRAANAVPVVVEEAQARGGKAHFSALARETTVAQLVAAVRGFRGVDAKKPVALTVAGCSVSPSATLGELHDACRRADDGMLYVAYTAERCMGAAVCTPCGSCAWDGSTADDDVII

>1282

MSMYQSLIPSDARRAECERVCREHPEQLPVVVESANSSHVRFLAVPRDATVADLEAEVRQTLGTTNKKVALAIEGCSPAATTVVGDIFDACKQVDGFLYVSCAREPSMGAKDFCCFGNTGKYFADIENNPNLLGSL

>1283

MHAPPKPPPPPRTHYQYMHSFECRCLLSKKMLRLYGAGTVPVIVEPTESHLRLSPSPPSLHGDSKSSAGSAGSFGGYARNGQLSSSGRSGAGVTASSAASAFTSPSAKSTLKCILPRSKSVAEVILTLRGRLALDSCQSLFLSVGENDVLVPGNSLLGDLYERYRNPDGFLYLGYLLENTFGGDVRSAAVEASVHTVR

>1284

MSAYVLSTPLEARVAKCASLRAANAVPVVVEEAQARGGKAYFSALARETTVAQLVAAVRGFRGVDAKKPVALTVAGCSVSPSATLGELHDACRQADDGMLYVAYTAERCMGAAVCTPCGSCAWDGSSADDDVII

>1285

MSAYVLSTPLEARVAKCASLRAANAVPVVVEEAQARGGKAYFSALVRETTVAQLVAAVRGFRGVDAKKPVALTVAGCSVSPSATLGELHDACRQADDGMLYVAYTAERCMGAAVCTPCGSCSWN

>1286

MSAYVLSTPLEARVAKCASLRAANAVPVVVEEAQARGGKAYFSALARETTVAQLVAAVRGFRGVDAKKPVALTVAGCSVSPSATLGELHDACRQADDGMLYVAYTAERCMGAAVCMSCGSCALN

>1287

MSAYVLSTPLEARVAKCASLRAANAVPVVVEEAQARGGKAYFSALVRETTVAQLVAAVRGFRGVDAKKPVALTVAGCSVSPSATLGELHDTCRQADDGMLYVAYTAERCMGTAVCTPCGSCSWN

>1288

MSAYVLSTPLEARVAKCASLRAANAVPVVVEEAQARGGKAYFSALARETTVAQLVAAVRGFRGVDAKKPVALTVAGCSVSPSATLGELHDACRQADDGMLYVAYTAERCMGAAVCMPCDSCAWDGSSADDDVII

>1289

MSAYHSSNPVEARRAECARLQAKYPGHAAVVVEAAEKAGSKVHFLALPRDATVAELEAAVRQALGTSAKKVTLAIEGSTPAVTATVGDIADACKRDDGFLYVSVRTEQAMGAFASPCLSTY

>1290

MSMYQSLIPADARRAECERVRREHPEQLPVVVESANSSHVRFLAVQRDATVADLEAEVRQALGRTNKKVALAIEGCSPAATTVMGDIFDACKQVDGFLHVSCARESSMGAKDLCCFGNTGKYFADIENNPDLLGSL

>1291

MSAYHSSNPVEARRAECARLQAKYPGHAAVVVEAAEKAGSKVHFLALPRDATVAELEAAVRQALGTSAKEVTLAIEGSTPAVTATVGDIADACKRDDGFLYVSVRTEQAMGAFASPCFSVA

>1292

MSAYHSSNPVEARRAECARLQAKYPGHVAVVVEAAEKAGSKVHFLALPRDATVAELEAAVRQALGTSAKKVTLAIEGSTPAVTATVGDIADACKRDDGFLYVSVRTEQAMGGIAGLCFASDSGGI

>1293

MSAYHSSNPVEARRAECARLQAKYPGHVAVVVEAAEKAGSKVHFLALPRDATVAELEAAVRQALGTSAKKVTLAIEGSTPAVTATVGDIADACKRDDGFLYVSVRTEQAMGAFASPCFSVA

>1294

MSSRVAGSYKKAHTLEARLRDAEKVRERAPDRILVICEKAENSPVPDLDKSKFLVPPDATVGGFLVSIRRRITMEAEKALFLFVGDSVPANSTLMSDLFNRYKDEDGFLYVTYSGENTYGGQGLH

>1295

MHAPPQPPPPPRTHYQYMHSFEHRCLLSKKMLRLYGASTVPVIVEPTESHLRLSPSPPSLHGESKSSAGSAGGFGGYARHGQLSSSGRAGAGVTASSLASAFTSPSAKSTLKCILLRSKSVAEVILTLRGRLALDSCQSLFLSVGENDVLVPGNSLLGDLYERYRNADGFLYLGYLLENTFGGDVRSAAGEAASHTVRRPVQR

>1296

MSARSTFKQEHALDKRQAEAQRIRDKYPDRIPVIVEKAEKSDIPDLDKKKYLVPADLTVGQFVYVIRKRIKLSPEKAIFVFVNNVLPPTAALMSAVYDDHRDDDGFLYIAYSGENTFGSALDLDLIALEEDE

>1297

MSSRPVKFKERFPFDVRKQQSEKLLREHPERIPVIVEKYEKSDCPDIDKKKFLVQKEMKMAQFICVIRRRIALQENQSLFLFVDNKPPSMAATLASIYDKFRKKHKKDDDGFLYVVYADQETFGQ*

>1298

MSRPVKFKERFPFDVRKQQSEKLLREHPERIPVIVEKYEKSDCPDIDKKKFLVQKEMKMAQFICVIRRRIALQENQSLFLFVDNKPPSMAATLASIYDKFRKKHKKDDDGFLYVVYADQETFGTSSSTNKAE*

>1299

MSQKKFLVQESITVGEFLTIIRKNIKLNASESIFLFVDNTIPSTGSPLSVVYEDYKSKHPKDDGFLYLTYAGEATFGNL*

>1300

MMRSGKETNPYKQKHDFAARSAEARRIRKKYSEQYPVIVLRGKRCKLPPLDKNKFIVSGEMTLAQFQQIIRSRSNLAASQSIFVYVENDGKFAIPETSKAIRQLYQDFGDDDGFLYLTYHHDDAFGASALEM

>1301

MSKSTAFKAQHAFETRLRESEKILQKYPDKVPVIIEPAKRCTLSEIDKKKYLVPEDLTVAQFQFVIRKRMNIKPDKAVYIAAEVTEGLTVKHEMLMTTQAMSVIYEHYKDEDGFLYLKYSGDNAFGAVLSAC

>1302

MSHHQPEYYEKVIVRDETKQSSRFGERAISAFQQKHPFETRKAESARIRQKYPDRVPIICEKADKADITTIDKNKYLVPADLTVGQFMYVIRKRMELSPERGIFLFVGEVLPLAAALISSLYEEYRDDDGFLYFTYAGESTFGHPTTST

>1303

MVRSAFQQEHSFEKRKAEAERIRQKYPDRIPVICEKVDQSDIETIDKKKYLVPADLTVGQFVYVIRKRIKLPSEKAIFIFVNEVLPPTAALMSSIYEEHQDKDGFLYITYSGENTFGVEQL

>1304

MVRSAFQQEHAFEKRKAEAERIRQKYPDRIPVICEKVEKSDITTIDKKKYLVPADLTVGQFVYVIRKRIKLSPEKAIFIFVNEVLPPTAALMSSIYEEHQDKDGFLYITYSGENTFGADAE

>1305

MDPNLIPSVKPITKTGKIFKEQKTLSERMEESKKVREKYSDRIPVICEKSSVEKDLPSIDKNKYLVPADLTVSQFVMVVRNRLSLNETTSLFFFVGRDVLLLQTDVMSNVYERYRDEDGFLYVTYSGQNTLG

>1306

MKYKEDPENTFEKRQAEASRIRQRYSDRIPVICEKADKSNFPDIDKKKYLVPADLTVAQFVYIIRKRIKIDSTTNIFIFVNNVLPPSATLMSQLYEEQKDDDGFLYVTYNGENTFGEI

>1307

MSAHRCCVPPLCRLVLFVGATADLATFSGVGELLRTGLTLGTKQQHTATIEAATKLCVGFVVVVLREKPSTLHLRTIPAPSKMSSFKAEHPLGKKGEEAFVGGYDEGGIPTTCHPSHVLSFFAFRFPLLATPFCCFLAADKRKGEAEKILSKYPDRIPVICEKADRSEIPDIDKKKYLVPADLTVGQFTYVIRKRIRLPPEKAIFIFVNNYIPPTCTDPFPPTLPPFLPSFLPPSFLPLQVYGRCSRARIGRSPSERFTA

>1308

MFYGNKKKNSDFEFNFQKENPFEKRQEESRNILNNYPGRIPIIVERFPNSTSVPELAKKKFLCPSDITFSQFSESIRKKLKIESTQAIFFFSPDLISNEIVISELYEKKKNADGFLYISYSSENTFG

>1309

MSFRDTHTFEERKKEAMKIREKYVDRIPCIVEKAERSQIQDIDKNKFLVPSSLTIGQFMYVIRKRLKLQQEDAIFLFVNGVIPPVANIMSVVYEEHKDEDVILNFYLSDLII

>1310

MSKFKEEHSFEERKKESEKMRKKYPDRIPCIVEKSEKSDIADIDKVRFLVPSELNIGQFVYVIRKRIELNSDKAIYIFINGTLPPTAAQMSLIYDEHKDEDGFLYVKYAGENGFGIPNFF

>1311

RKRIKLQPDEALFVYIDGVNVPAANLMSTIYEEHKDEDGFLYMIYSGESTFGSL

>1312

MATKFAFKTEHAFDKRSSESARIRTKYGDRVPVIVERAEKSDIPALDKKKYLVPADLTVGQFVYVIRKRIKLSAEKAIFIFVNNVLPPTGTLMSAIYEEHKDEDGFLYITYSGENTFGFNKDTE

>1313

MFSIQNKYTINKITNNNMQKESFKKQYSKEQRLKESENILKHYQDRVPIIIEKDKNSRLPDLDVQKYLFLSNFRVFQLNTLIRSKLNLNKAEAVYLFVNNKVALRGGTIHIKEVYDKYQDDDKFLYIQYCEYNTFGL

>1314

MCDNLKISDNFIEKYPDRVPILLQISVRSRLQFQDGTKIKKYLMSKSDHFYHFLQIIRETLHIQQQESLYLFINNSGLVKAESQVDEVYNKFKSPDGFLRIQLTEYPSFGQ

>1315

MKFQFKTQNSFKQRQEQSFRLIERYEKMIPIILEIYIQDAEKKQIPKADLQIKKILVHEQISVQQIFDKFNQRFSQYIGKKESLFLFYGGNKLITNSFSQTIQKLYQQEKDIDDWLYLELRIQETSG

>1316

MKFKYKIQNSYEQRTQQSFNLIEKHGQMIPVIIELVLKSRWLKEIFNKHKQFQKMIVRETISVQDLFNTLKYKYYPQMTPTFGIFIYVGGNKLLLHNSFPKSLKEFYEQNKDSDGWLYLALTFQECYG

>1317

MKSKIKKQPPQYALKLTKLNQPLDSKNLKVLNLQGLQLQQIDIKVDNLLTLILDSNNLKNVDIISQFPNLNTLSIAHNQIEEFDVPSTLRILNISNNLLKTIHLRQLQQLDASSNQLQILKQDQQNNLVQFKIDWLKIINQNDVWEHLTIKQFANQQQITFQQFLDKAIDLLIQYGFKKENNCFNQQIIHTSILQNDKYYFDLILPYYKDNRHLFDNSETPLSLAIKKLKMNFIGDLMSSIPIKYEIDAFHESIKQGQVSLVKQFLELGIDCNGYSQKGLTPLTNAVLNITQMNMEMIIHLLLQSQANPNKLNQNGQSLIQMCIIKSNLTALRFIANLNKKKQTRLKFKMNIKNTNGDYPLHLAVNSVSILQFLLNNQIGNPLQVNYQNLTAKQMPFSQNRPLVYKLLQKEERVQVAKQLAKNDITFRVLNKCQNTQKDYSSDSSCPELSDDEMPNKPKINRKYLTEIDQLSEDIRQLSEDEECQHNCSSESEIQYPKDNLKSLKLVQLNYQNYQLYKLNSHVRQSSILINKINKKRLNADQFNYYSRQGQNFKEKTTSLVCLRQELLFLGQIRQYQDLICSDFGFIHQQTQSGYTQLRVGVSKGFAQRLYEFLISLIFNMSYFSDININYFMQSIQESIIDKYPDRVPILVETIDKSKLRFSDGSKFKKYLVEKSDHFYHFFLILRNTLKLSKQESIYLFVNNTGLIKPESQVGEVYSKYRSKDGFLRIILSEYATFGNC

>1318

MSENFVEKYPDRVQFLMITSPILLQISLRSRLRFQDQTTIKKYLVSKSDHFYHFLQVIRETLHLQKQESLYLFINNSGLIKAESQVDEIYQKFKSSDGFLRINLTEYPSFGNE

>1319

MSENFVEKYPDRVQFLIINSPILLQISQRSRLRFQDKTSIKKYLVSKSDHFYHFLQVIRETLHLQKQESLYLFINNAGLIKAESQVDEIYQKFKSSDGFLRINLTEYPSFGNF

>1320

MQKQKTQTYKDSHTLEDRKKRVQEQLAKYPEMIPIIVEKIPGCKLPQLQKVKFLVNSSFSFNEFKNTIKKKLNLDEKTSTLFMYCGKNLMNERTSYNQQLDDKLKNIYDQYKDPEDGFLYLHYADAETFGF

>1321

MQTSLTYSLGLKNDSNYQFLTKHTLAKRKELSDKYNNGQQVAVICEPHRLYRQAWAGAGPQLPTLVCVFSKKDPVSSIFQTLKAKLKINQETTLYLLCNNYILQYEQSIGQVYDKYKNHDDGLLYIKYSSQETFGN

>1322

MFQASVNFKSYKELNSLEERQMRCKQKLSQHPEMIPVILEKHPKSKMPQLNKSLQVRSQSY

>1323

MYSIQERQVKFKEKLNQNPEMIPIILEKHPRSKIQGLKNQLYIYLYNLIQFRDTLKCTLQISPKQSLYFHIGNQLLPEGLADIYQKKKDPDDGFLYITYSDLEVFGFISK

>1324

MNAQTKSSFKERHTLEERIKLYQTKQLAYPSKILIILEQFIDSSSTSQLQQQHIIKRCAADPEQLMVTFEGDISLLFKKKIPKAQHISLFFFINNVLPQKDQTMGQLQKKMQDQEDGFLYIQVKSLETLGSL

>1325

MNSQALEATKTQSVKDYKAQHKLEDRKKRVQQYKEKYPEMTPMVVQKHPKAKIMSLTRPQFLVNQTVKFSEFKNQIRTKLQLSPQQTLFFYCGNNIISDDISLQELYNKYKDREDEFLYLNYSDCEVFGNQID

>1326

MKQILSFQNTRSFDERKNESDNCKYLYPNDILVVVEQLNTSKLPRQNFIKFKMPNTMTIINVLQYIKSKIKLSQYDSINLYCGKILLRIDQTLKELYQQFKDLDGFLYISYIEMNSFGIQF

>1327

MKFSYKIKNSYEQRTQQSFNLIEKHGQMIPVIIELVLKMRYHKEIPNKLQQFQKMMVRETISVQELFNILKQQYSPYMTPQDGIFIFVGGNKLLTNSFSKSFKEVYQQNKDSDGWLYLEVRSKDHSG

>1328

MFQTKTKQKSFRELHTVQERQAIFKDKISHYPDMIPVILEKHPKSKIQHLNKQLYFLPQNIDIYFRMIQFRDTIKSTLQISSKQSLYFHIGNQVLPEDIKLADIYEKKKDSEDGFLYITFSDLEVFGFNSN

>1329

MQKESFKKQYSKEQRLKESENILKHYQDRVPIIIEKDKNSRLPDLDVQKYLFLSNFRVFQLNTLIRSKLNLNKAEAVYLFVNSKVALRGDMSIKEVYDKYQDDDKFLYIQYCEYNTFGL

>1330

MFQKKINVMGVANDNEFEFQKKFTLAERQLKYQNVINAVGEQKALVVVEKHKKSNIQSNTQVQQPWKIFAIDKTKNLAEFLHCIKLNAAINKQTSIFLYCNNTLLMLRGMLQLYLDDQTVGQIFDSQKNKEDNILYIKYSDFETFGF

>1331

MQKQKTQTYKESHNLEERKKRVQEQLAKYPEMIPIIVEKIPGCKLPQLQKVKFLVNSSFCFNEFKNTIKKKLNLDEKTSTLFMYCGKTLMNEHEKLKKIYDQYKDPEDGFLYLQYADAETFGF

>1332

MKQTLNFQNTRSFEERKNESDNCKQLYPNDVLVVVEQSSKSKLPKQNFIKFKMPNTMTIMNVLQYIKSKIKLSQYDSINLYCGKTLLRIDQTLKELYQSFRDQDGFLYINYIEMNSFGTQF

>1333

MNSQQLDITKNQSVKDYKAQHKLEDRKKRVQQYREKYPEMTPMVVQKHPKAKIMSLTRPQFLVNQTVKFSDFKNQIRAKLSLSPQQTLFFYCGNNIISDDITLQELYNKYKDKEDEFLYLNYSDCEVFGK

>1334

MQNKSNTYKTYKDVIPLEERKEKYNYYKQKYPKCVPIILQRQNNCNLIFLDRPEVLLDEEKTGNQLIQYLKDQLKEKSLNNSFYVYFTTTDNEGNSQDTMLQMEDKIKQIAEKYKDKEDGFLYLKYDYQQTFG

>1335

MQSIQVSLADKYPDRVPILLEITDKSKIRFSDGSQCKKYLVSKSDHFYHFFQILRNSLKLSKKEAIYLFVNNSGLIKPESQVGEIYSKHRSSDGFLRIILSEYATFG

>1336

MDKVNAEEEGLINRKQNKEFSACKNYIPVVVEPYPQTKLKKIDDNRWLFNKDSTLYPVMYKIHESLNGAKNMYFYVKRQNGRYALLKHGMINLLNLENKLQLVFKKYKSNDGFLHVYYHNDMINRSKLLDYCFYFAQIVIAFYLVLFIYGYLKMHEYI

>1337

MGMVNDNEFEYKKAYTNEQRLEKYEKVIKHTGEERILVVLERHRKAKIQKQNDKLSQFQLFAINKNKTLVELMQYVKQNAGIDVSTSIFLYCNNQLLMMKSDITVGALYDTYQNKEDKHLYLKYADFETFG

>1338

MSKSFKEEHQLEKRKSEAERIRAKYPDRVPVICEKADRSDIPDIDKKKYLVPADLTVGQFHYVIRKRIKLAPEKALFLFCSNTIPPNAALMSTVYEEQKDEDGFLYIQYSGESTFGDVDAEEEE

>1339

MPSLKDEVSFENRVAETHKIRSKYPNRIPVVCERANRSNLPIIEKKKFLVPMNMLVGEFKFILHQHINQSAYGSNMKLFRERTIYLFVNNIVPKTGLLMQDLYEMYKDEDGYLYMEYSCESCLG

>1340

MSKPFKEEHSLEKRKLEASRIRAKYPDRVPVICEKADRTDIPDIDKKKYLVPADLTVGQFHYVIRKRIKLAPEKALFLFCGTVMPPNAALMSTVYEEQKDEDGFLYIQYSGESTFGTITEETEDRSEKNNQLCA

>1341

MTSTTFEFEKEHMLAERKDKAHRITAKYPDRIPVICEKDPKSKLNEMEKAKILVPKDLSVAQFIKRIKLKPEETIFLFINNKLVHNGALMADVYENNKREDGFLYVRYSSENYFG

>1342

MKSTFKSEYPFEKRKAESERIADRFKNRIPVICEKAEKSDIPEIDKRKYLVPADLTVGQFVYVIRKRIMLPPEKAIFIFVNDTLPPTAALMSAIYQEHKDKDGFLYVTYSGENTFGR

>1343

MASFKEQFTLQQRIEQANRIKQKYSDRIPRPASPRQSSPAVTRTMGEIYKEHQDEDGFLYMIYANDEVYGGCEQQQDK

>1344

MEIPFEQFPGLGYDEYPGFPVDECPALPDLSRHCSVCTDILKAHPEIYEDLKDCKTSKGVTLARCIKTGIDNCGHPAFKSLGAVAGDAESYDTFAPLFDKLLERRHGEVPSLNSKCYGRRSLTTESAEPFGGYVMSCKVAAWRSLDVRFPPAASLQEREEVERILVDXDFKGQYYPLTGSNSFPSKPGGMSSEERKMLADNQLFDRPDAPNVLSTGAGRHWPHGRGVFTNERKSLTLWINEEEHLKATSQQTGGNVQAAYLELSNALDQISSKLPAGFAYSKRLGYLTSNPGNLGTALRALQKLPFNDDAIQKHLDKQAGESQEDGADIEESHEELTPESVVSPTECLQQEPFPSDASASGDLSRRIQGAAATLGGATGMLLAGPVSGAVLGAAALYASTREDFSGAVARKAGSLYLKVSDRACDEGVRVMDKGVEKAGAVLDKGCRRLSQSQSVPAPIRAGFQQLTGATHGSAGYSGHPTVGPEEAKRIREKHPDRIPILCERSAYSQLPQLVKNKFAVPGEMSAGEFKYLVQKELKKGRRIWGCFNPVEEKLPGRPPEQTIYIFVNGVAPRCSARMAELYEQHCREDGFLWVKYSAEQTLGGMLHGYIANKSQCQCKNSISQTLPGRFEALVCCSRFGDRVCGT

>1345

MSGLASKTCWMTFFQNSRLGAQRLIVSTRHKDALWQVWQVIVTRNVANREHSTVGRATEVWRKLTRAPFARALPESRLIESCSLGGVDNFYRDTDVRHCVLAICCRASGPAILRVQCRGSSFRVDRLVGAGRPIDLQIYSMSVSLQQSVPFEKRFAEANKILAKYPDRIPVICERAPHSTLPEIERKKFLVPSTMQCYEFKYIILNNIQHAQEGDHASDQVIYLLANNKATKTGAPMAELYEAYKAEDGFLYMTYTAENTLG

>1346

MSGLKETVPFEKRAAEAQRILSKYPDRVPVICEKAQRSNLPEIEKKKFLVPGSMLCGEFKYIVHKHIQQAANGTGLAADQVIYLFTSSGRSTATVKTGEMMSEIYDRYKSDDGFLYIAYSAENTLG

>1347

MLPSLKPLTSCCCCGVCTGTGFIVIIHFLACVAVIVNSIFHFVLRKETYGSVWSPEGLLLFTMFAVMGIPVTVGGFFGSWLKVEQYVRAYLLYLLATFLEDVALLDGHLLYLQLSLGIFLALRGRGPAALQGLALSAQHVECALQKSRLTHVLALLRSVLCKPVALSDPKDVQHLSVVILEAFLHAAFRNMRHENLLPRPGMDQGKSWPEVLRFEWFCGHDTDWSWTHHGLVLDTPGMARPVKAQGATEDLRLVLFDASLEEWLRPSEGPTWGMESYTVDTYLSSSTPVDALRSLLKAGVQVLCCQKLVDPWLMDYLSSKGVSILWRLSIRHVEFVRRLSGATPVTALAVLPPWQDVIGAVGTVELKRICNRDYVILTPPVSAGASRAKVSTMLISAPDDHALEELKKPGYKFLKVTQEKLQKEMAMLTTSCALPLGKRPEWRCAAGTEIHLAAELDHLAQLHMTESSGTAVALRAVATCFKDAAASLTVSGAEHQCPSASAAGTDMIDEATLVLQRWTESDSLPSGMVFDAEFPKRSCIMGAMNLAGILCTEPILKMAPKVRKAAHVEKVEQLVAAPMEVNEDPEKARCIEGDQVEDVQPGERSTTFLQRTPSATNQAASDLANDRRLMGAAATIGGAAGTLLMGPVSGVALGAAALYAATREDCTGSVARKAGTVYLNVADTAIDEGIRAVDRGVKAFGTAVDRGCRQLETSASVPTPIRVSLQQWRRQSEDKTPKTGEAADEAEKIRSKYPDRVPVICEKSARSTLPELPKKKFVVHGNMLCGEFKYMVHKQIVDATPERLSVDQTIYLFINGITPKTSTPMSQLYDQFRQSDGFLYIRFGAENTLG

>1348

MFDWGGKDETCEQVKKLLKKYPDRIPVICTKAAKTSLPEIEKKKFLVPANMLCGEFKYIIHKHIAEAQQSGLAPDQ

>1349

MGKDSFKEVNSFEKRKMEAQKIRCKYPDRVPVIVERAGKSDIEEIDKKKFLVPMDLTVGQFVYVIRKRIKLSPEKAIFIFVNNVLPPTSSALATIYEEHKDEDGFLYVTYSGENTFGFEL

>1350

MPVRSQFKDEHPFEKRKQEAEKIRQKYPDRIPCIVEKAEKSDIAPIDKKKYLVPCDLTMCQFVYVIRKRIKLSPEKAIFIFVNNVLPPGSSLLSEVYQEHKDEDGFLYIVYASENTFGDA

>1351

MFKFKVDTPLEKRIEEVQKITSKWPGRIPIILEKSKNSQLEDIPKSKFLCPSDYTVQQFLGCIRKKVKMRRDTALFIFVNGKELVTGDASMLAIYEQKKDEDGFLYMIYSDQDVMGAHYNQ

>1352

MSKSEIKEFKEKTPIEERKAKAAQMLQDNTDKFPIVLLRSKTCKYSLPAHKFIIDGNMTVGGFICAVRKYLWLKPTEGLYLYLQDTLPMLNVKLSDLYRTYSDPDGFLYMVYTNQEDKGF

>1353

MSTFDMTIFEDRVQQANRFKSRFPNSVPVVTCKESKSTLPGKINNQLFLVPRNLMAAEFTALIRKKVNLPRTQALMLFINSKVLVTADAVISELYDKYSSPDGFLYVLCTDHEAYGR

>1354

MWKFKIDHTLDERKEEAGKIGRFWPDKIPMVLEKFKTSKLEDIPKAKLLCPRTYSFFQFISCLRTKLKLDSKQALYVFVNEKQLVTGDKLILELYEECKDEDGFLYLKYCEHETLGCDYSL

>1355

MWKFKEDHTLDERKKEVGKIARFWPDKIPMVLEKFKNSRLDNIPKAKLLCPRTYSFFQFISCLRTKLKLDSKQALYVFANEKQLVTGDKSILELYEECKDEDGFLYLKYCEHETLGCNYNLSSE

>1356

MSKELRILSFEARREKSANLLSENPGKIPVVMLKGKNSKLELEKHLYLVSNQLTLAEFLYSIRKSIRIDKSEGFYLFVSDRLPPLNLHMLQLYQRSSEDDGFLYITYTTQEDKGCDLNLVL

>1357

MWKFKQDNTLEYRKEESSKIMRYWPDKIPIILEKHGISKLSEIPKAKFLCPRNYSFFQFNSCLRAKLKLESNQAIYVFANEKKLVTGDKSLLELYEESKDQDGFLYLMYCEHETLGCDYS

>1358

MSKYQKNIDLEKRKIESSTVRRCWPEKIPLIIEKDEKYNIKEIKPKCLCPIDYNVGMFLQYLREKIELNRSDALYVATENGQILSGDRMMYDIYQNDANEDGFLYLKYGMHSTYGNR

>1359

MSKTRDDFNFQFKDEIAFERRAIESEKIRLKYDGRIPVIIEKSSTDQVLCDLEQTKFLIPGDFTFQQFQMVIRKRLQLPKTHSLYIFFHNNKLHANEKSLSQIYSECKDHDGFLYCKYASENFTG

>1360

MKQVQTFSLNQSLNMTQNRPGQLPPLYQSQESTEIFQIDKFISEGVDHYRNLFSFMIESINQQVIDNQKIIGKFFYRFHVSDDLTIGELSEIIKFKINSKIREEEEKLSQKERIIFFNNRLSCSETLELKEVYDKMRESDGWLYMDFLIEVING

>1361

MLKKQQDAFEFDFKNDIPFEYRASECQKILQRQSNRIPLIIQRAQRETDIEEIKQQKYLVPTDFTYQQFLEFIRQKLNVSKNTGIYIFFSNNKFYQGEKAMTQVYEECKDEDGFLYCKYAGTEVFG

>1362

MNYKKSHPDQDMRKKESEKIREKYPDRIPVICEKSQTSKLPDIDKSKYLVPNDLTSYHFNYIIRKRIKLPEKESLYFFVNGKYLLKGDTLMAHAYEQKKDADGFLYITYTEESTLGAFLEENNIQLD

>1363

MSDSNNSARLSETFDSSQSTLFKQRNPLDKRVKWAEQALEQYPHKYPLIIERAADQSKIIELTNPKFLMPKVFKVSEVQTIIRKKLQLTREQNIFLLANGKHIMKQDQTLMQVYDRHRDDDGFLYIQYALEQTMG

>1364

MNYKKAHPDFGISNSHLYNVILEQRRSESERIRERYPDRIPVICEKSETSKLPDIDKTKYLVPSDLTAYHFNYIIRKRIKLPEKDSLYFFVNGKYLLKGDTLMAHAYEKRKDNDGFLYITYTDETTLGAELQ

>1365

MDAQNYKPFKEKFSLAERQKELEKQKSKYPNMIPVIVEINKKAKTVRVLEKQKFLVSKDVKLHEFQQTVRKKLDITNEADALFFFVGNKKIEKINRTMSEIYQESKDKDDGFLYITYSDQEVFGSI

>1366

MDSNKRYKDLLSEDQRKSECENIRKNYPDKIPLIIEKHPKSKLEQIDRTKFLILEKLRVYQVNTIIRRKMSLNKVDALYLFVNDNILLRGDQCLKDVYEKYKDSDGFIYLSYCEYSSFGKNI

>1367

MFKICKKRKTKKSSKSLLESMQPKTIEELKAEEVIIAQYQKEFTFEERYNDSQECKKKHPECVPLIIERHPDHKDIKEMQTPKFLFHCQSQTANAMVRTQKDVQAKKMWFFVHMPDNQLKLIYPQSKLGVLYQRYKRSDGNLYIFYSDKNLVQQTSTFMNVMQIICVILLIFYGYVYFFGVKDILWLFGYDLAKLQQEYIQSQTNSTLTNSTHVNSTFSNTTNSNAEKIEL

>1368

MNQTAESQLDQKICIEILQESEQNFKKTHSFEERFIEYQIIRCNYPGFIPIACELENITDVDVSKHINFRFYVSDDIFVGHLKNYLKELFQKRIKTMKKSHRLHLLIEDDVIIKHDIPIKEVYTKKVEPDGWLYLKLSIDY

>1369

MMQRLFEIIQQNKSQNQDLTHILYKYITPVNQRIKECKQILDQYPDYVPLIIEKHPNSKLDDFKKLKLVIQGKMDILKVQSVIRNNIKLNENEAVSIFVHNGFMEQLKLLNLGSRLSDLYKSYKSTDGFLYITYCEFSSFG

>1370

MSKSFKEEHPLEKRKSEAERIRAKYPDRVPVICEKADRSDIPDIDKKKYLVPADLTVGQFHYVIRKRIQLAPEKALFLFCSNTIPPNAALMSTVYEEQKDEDGFLYVQYSGESTFGGEELELVEE

>1371

MSANSFKAEHSLEKRRSEADRIRAKYSDRIPVICEKAENSGDIPQIDKKKYLVPADLTVGQFVYVIRKRIRLSPEKAIFIFVNGTIPATASLMSSVYEEHRDEDGFLYATYSGENTFGADE

>1372

MPSIRDEVSFEKRTAEAHRIRAKYPNRIPVICEKAPRSDLPVIDKKKFLVPMNMLVGEFKYIIHKHINQCAQNSGLPLTHEKTIYLFVENTAPKAGALMQEVYEQHVSDDGFLYVEYSSENTLG

>1373

MELQSLDRSNNIMTMLNKYPDHIPVIVERDPHCTTLPDIKNKKFFVTKTLSVGNFVYCVRNRLDINEKDAIFLFVDNTLPNPSDNLGAIYEKHKSEDQMLHCTYSSDSAYGFL

>1374

MFSSKNESRYKREKSFADRCQESAGIIRKFKSHVPVIVDKDPKCTLPDIERQKFLVPSELSIGQFIYVVRKRINLQSAEAIFLFVNKKLPPPNSTMGALYEENRDEDGFMYCLYSSDNSFGSN

>1375

MPSHYRYQYTRSFAERAKETESARLRYPKHIPILCEPTSAASASTPRDVRLFSTRQQVQRELDCNKFLLPETATVMEFMMALRQRLLLEEGQAVFVFIGNELPPNSACLGDIYARAKDPDGFLYVSYGVENTFG

>1376

MKYNFKDSHSLVKRLNESTKVRKSHPNHFPVICEKVYNSDIGELDRCKFLVPSDLTVGQFVSVLRKRVQLEAESALFVYTNDTVLPSSAQMADIYSKYKDEDGFLYMKYSGEAAFG

>1377

MSKKDSKYKMSHTFESRQSDAAKVRERHPDRLPIICEKVYNSDIGELDRCKFLVPSDLTVGQFVSVLRKRVQLEAESALFVYTNDTVLPSSAQMADIYSKYKDEDGFLYMKYSGEATFGC

>1378

MLAAEWQRYVFFLFFSFAVHVKVFFFYYFVFPLPPSHYFHVFHINVFVCVCALFSFLSSHESIGMWGREERASLSPFLLFFSHICTHTHTEMFLHRRSLLVFLLLFFLFFFFFLSFSLPALVVSSRCGRRELLLLAAYATIDLSFHFCTGKYITAHLYYYTAFLPVVSIYICLVRCTPCSLRIYISIYMPRKYRYQRTHTFAERQKEVATIRGRFPQHVPVVCEPASIDTVLSAGPISDRCRLLRDLDCSKFLVPGDASMQQLMVLLRSRLVLDAEQAIFLFIDDAVLPNSACVGDLYAQRKDADGFLYMTYSIESAFGGAAVRRN

>1379

MAPKKLESKYKKTHTLESRQKEAAKVRERHPDRLPIICEKVDDSDIGDLDKGKFLVPSDLTVGQFVLVLRKRVRVDADEAIFLFVNGAVPPTTAQMSDLYAHHKDEDGFLYIKYSGEATFGSW

>1380

MCLCVFCFLFSPATRVLTCGGGSKERFSLFFFCFSLIYKHTHKRRCFLIVGPCLFSYYYFSFFFFFFFFPSFSLPALVVGNRCGRRELLLLAAYATIDLPFHFCTGIHTCTLILLYSLFTGSVHLYLFGPLHTLQFTYNIYIYIYIYIYIYMPRKYRYQRTHTFAERQKEVATIRGRFPQHVPVVCEPASIDTVLSAGPISDRCRLLRDLDCSKFLVPGDASMQQLMVLLRSRLVLDAEQAIFLFIDDAVLPNSACVGDLYAQRKDADGFLYMTYSIESAFGGAAVRRN

>1381

MRSAFKNEHSFEKRKAEAERIRQKYPDRIPVICEKADRTDIPTIDKKKYLVPSDLTVGQFVYVIRKRIKLAPEKAIFIFVDEVLPATAALMSAIYEEHKDEDGFLYVSYSGENTFGQL

>1382

MATMSLREKVPFEKRTEEAHHIRAKYPNRIPVICEKAVRSDLPQIDKKKFLVPVNMLVGEFKYIIHKHINQCSDNRGSNFPAEKTIYLFVNNMVPKAGALISEIYEQHKDDDGFLYMEYSAENTLGGGEYH

>1383

MASYKEKEPFEKRCQESARILEKYPNRIPVIVEKGKRTSLPQISKTKFLVPKEMTIAEFCAIIQKHINDQGDESPTAQPDNAAGRTSPATPDASHRRRAHQQTLYLLCKGVWTERGSKMSEVFAKYKDDDGFLYFTYMAETVFGC

>1384

MVNTRPLTFKQEHSIDKRKAEAARIKEKYPDRIPVIVEKAERSDIPDIDKKKYLVPSDLTVGQFVYVIRKRIKLSPEKAIFIFVKNVLPPTAALMSSIYEDHKDEDGFLYITYSGENTFGGVAALGEGEEQEGNEQLLLQG

>1385

MSSFKKEHAFEKRQAEAQRIRSKYPDRIPVICEKADRSDIPDIDKKKYLVPADLTVGQFVYVIRKRIKLSPEKAIFIFINNVLPPTAALMSNIYEEQKDVDGFLYITYSGENTFGQ*

>1591

MVASASSAGDTLQSRIDCATVLADRLHADLADLEEELRCYQQFAPPPTASTDSGDNLAELLALEADLAAQLAAAETEEAALELTLADLAADAAALDAAECDFWHASHAFQASLQAYQAERDALNTKYDAASRHLDKLKRTNVYNDVARLGHDGTYPTINGLRLGRGVSGNGAPPVPWHEMNAAFGQVCLMVDALARKAKVEFVGYRLLPMGSFSKIQKLVPAPANGASSSSSSSSRSSRRVVPSDTASARRGSTASTDPTVQQWVVYATYDLHGGPGEFTTLNRLLLNSKFEQGLEALLACVKQLAEYCAAMDPVEFNMPYPIDKDKVGGYSIRPRTSSDAWVRACKCLTSDLRWIMAFVTLVAARAEAGDAARSGKGAVPAVAAPGVGRKV

>1592

MSARVATIERLHALATNSNVPLPLCSDCATVLADRLHADLADLEEELRCYQQFAPPPTASTDSGDNLAELLALEADLAAQLAAAETEEAALELTLADLAADAAALDAAECDFWHASHAFQASLQAYQAERDALNTKYDAASRHLDKLKRTNVYNDVARLGHDGTYPTINGLRLGRGVSGNGAPPVPWHEMNAAFGKCV

>1593

MNAAFGQVCLMVDALARKAKVEFVGYRLLPMGSFSKIQKLVPAPANGASSSSSSSSRSSRRVVLSDTASMRRGSTASTDPAGQQWVVYATYDLHGGPGEFTTLNRLLLNSKFEQGLEALLACVKQLAEYCAIMDPVEFNMPYPIDKDKVGGYSIRPRTSSDAWVRACKCLTSDLRWIMAFVTLVAARAEAGDAARSGKGASAAAVTPGVGRKV

>1594

MHRVEIIQSGKHCLRLRRVRTYVFALRQRWLYENQRALLQIHRLHEQRDSNQALLRWCSAKQSQLSTVNVLNDCFHIWHSGPFATINGFRLGRNHTTQVDWNEINAALGDILLLLATIDYNFSRYTLSLI

>1595

MGSCWLGPVRRCQLWVLKYPVWAMRDGYIPVYTVGMVNTRYIPVFLEYPDPSTVSRLRVEPPRAAPVPPRHALGDAARMLRWAYYSLLARATAGWGAVVGLGVRGRRARARAAASRARLAQVAPVPGDGRGVGVGVVVAEGDAVLVWGAGLPTHVVVVRSSQQCTRLAAAEPGSRGVPVYGSPKSKIPGMGKRARVYTGIYRYFTIPVYTLRNTRAPILPVQLQFRQSDQDYGNGGYMHPRGRLRGTEGRQKHASPRVPCGELQPREFLGEAPKFLQLEVNLTCVELAREAYTSTKEEAPNSNRSKFMLESLLGKRRQVNGSRLAHLNWTCKTIEKMNRVEALNDCFHIWHSCSCATINGFRLGRVAAGQVSWHEINAALGEIVLLLQSINFANTSIGLTPRGNYSKISKGIGQILFLGLRVAYADGDPQIQYHLYTDDSFSLLPKRNFNLAMLSLEIRGVLVSGAAVTVAVAARPTERPSNDRPRKYVPEMHQVALKSPGSGAISVQGARRALYKAGLYPDATRAALRVDSPQFAQRCLPDDPETIINYAAIAFKEGQYDEARGKYIEAMNTLGYQADLACNVALCYYKQKQYAAALKSLAEIIERGVREHPELSVGSNTDGIDVRSVGNSSVLQETCLVEAFNLKSAIEYQMGNLDAAKEALSDMPPRQEEELDAVTLHNQALVHMDEDPSTGFRKLNFLLSNPPFPPETFGNLLLLHCKFGYYDLAADILAENTHLTYKFLSNELFEYLDASIMVQTSPEEAYRKYDDLTNKHIDQLRKLTKAIQDARISRDNEAIKQSLKFYDEALERYIPVLMAMARIYWDKENYPMVERLFRQSAEFCSEHEVWKLNVAHVFFMQESKFKEAIRYYDPIVKKKSENILDVPAIVLANLCVSYIMTSQNEEAEELMRKIEKEEERLAYTEPDRQCYHLCIVNLVIGTLYCAKGNFEFGISRIIKSLEPYDKKLGPDTWYYSKRCFLALAENMAKHMLMLKDASVHEIINFLEACDSHGANITTVITPTVDPDGNHPAEVTHNVSYEARQLKKIFLKLRD

>1596

VLILFYKQELGNFVETKDPALRYPYLIQNDKIDSSSVMHTSNEGKWTKAMKFLLTDLKWAVAWIVKHCDGHLKP

>1597

MVSLYRTHYQNVVIHPLGSFSEVIDRDSRTSFKLYFNPKSGNRKLFSQALQLFLQAVACLMKHCTERFKMEPRFAVDAENGKVGNEDFLFNDVDSWLRALRYLAIDVKQMIVYAYTASVYIVTHSCDVCLKEENRGNFCTRF

>1598

MAASGELEQVFVCQECQRALKIVERKGVKDSTLQALKTGELPTYLDESHKKKEGDAIKRLAESFVVLSESKVVKRPPENVVGNNTNFHAQVQALSKIFEVVSDKCQVDCPLCDQCSIDLLRRLRDKIRDVEEECKSFQRCIDLLKKEDLAKQHNSEKEKEEEEREKLLSMEVRRQIEEIRKERQRMRKQTKQLEEDSKKLDECERLFWEEYQEYQLELAVLQDTQGALQQKIEITQQQLEQLKYTNVYDDAFYISSKGHFGCINGFKLGRTSSEPVGWDEINAALGQVVLLLSSIAKQVQYEFQTVILQPLGSYSRIADRKSSPEIFNELYNYSSSEFFIRQGTSSSSAGKRFNTALSLLLRAVKELGDYGSRIDSSFKLPKTISGDKIEGFSIQAVAAEGDWTKVFAAQRIWVEDKMMIRSGYVVKRRFVVLMEVWGRIRNTEESGTVERRVRASRPPPIHAAHFGILLRMQRKGRKRKSVDASSTKAESQVRRSSRRTTRKDTCKRGEDKGKSGDDKGNDSVKVEVVPRIVLKLKDWRNYMSETREVAKKLAKEGKIAILQKGKVLDPSETFKGPIRLRVASKSWAQRIVSRAAKESSIFAAATHKNTAHSIEYEMGCSAAATTRVSRMNVHWECGSLT

>1599

MSILGEAHVALDGSCLQCNKCRRMFAVDCSNASVSVEFLTGDAPTSKAEAIRKKIAAAKRSLDQRGPSESFVAGPSSMFDEVRAAPWLAAPAGGEDGDACYQEAPLTLSTMPLTLAPNVHLSTLIGERAKAQPLLRVDTAMLEPIQEVSHSRVLTEDDSGASPASVSMRDALQRRSAFVAAEDSTPAVRPRGGLQASATAADASQYGPYLQRALLLHAAWDLLEQISEEALINVPLCSGCWKEVLGDLQHRVDREQSLVSASQAYLHPTSENVAMCALIARDPGEQDNGSTPTASSPSEHPPSQTLAYSRLLDAVGSESVRELSELDDKLHELRNVQSELDEEEMLINEEEAIVESLRNSVELALFCNNDDRDAIVGTRQQAIQALQFAESQSTLRELFVIELPSGCPSIGVINGVRVGKETPPVQPQQAAATSSQLLSHSFVVLGKSVAPPAPGGSPQAPSSPRAHEQRQGQHRREFPVRDIARRTGGEPYHIFTNYGSYAVSVPEINGGCGFLIAALDAVVQRSGKGRLDSCVLRPNGWESMIDVPIPNKKPVKYDDLKFYIVDKLFSWKTFGQAWVAYCLGLREMIEFLQGKLSTRSGLDLHDASSFPPFPIDKEGRVGGFSPKHGSVSDEMWGRAVRCAMANLDWCLAAQTVVEEHVAALLLLQNSCNATGSDAPVTTSTA

>1600

MRKCQQCQKSLENELLSTPVLGESYVLLPTASASQSMLLLQKLPTNAITPLEMDESGVFGDDLYASARYNLSLQQHYDQEQKRQQVQRRQELQHRQRRQQQRFKNSFAVSGKYFEDRASYPSRDEENIGTENSMVAVPTYADLSHHVQLLTTQLAHCGGLPVSTPLCKECVDGMVTMIDGQAERARYEKRCHSGFLHTTAFINIKSNVIDQIHEKIRFYESELNVLNENLQLMDNERKVISLQQKAIDHEEKALDHEEEELWKLLNESQLQESVYREIRDAGTAQIDAMEHKVKHLNILADMFVIGNDGEFGTINHFRMGQSASDTLEWNEINAAFGECALLLQTLANMVGLEFTDFKIVPLGSFSKMIRTSNLRMEYCLHGSDQQNFAESHFNLGLGAWISCLGQLMAYVQARDSSIKLPYKVAKHSIGGHSVIFLKKKQKEWTKALKYALTDLKWLLTWVSTRAYNSSTSPSTVIATSRKAALASTANSSILRNNQSVIIMEHYM

>1601

MSGNSSLQPPVIITSLPRPPATTGAYRVIPTSSLSSSPSSVHASFSPYAKSPVPRTRRVAMEGKSLFTCHQCGALLKFQYSPISIEEKIMKLRTIPYEHLTELCEQRVSNEDGEENLMPRLTEGTDNGEATDADEEEDVAQTARNSFNQQQQKISAVIPELVWERQQLDSSSGIGVWVSAELDLMDGIATFSSCNRGDIGQNIADVVPARLLNKPHLWLSDWEVDHSLLDCDEGGWVYAASNAHFGSLEGLVRTDDNETNLKTPEQIEGERLVRRRRLIRKRRIDGSDLSWFQELLDCSTLLLKKRLPTCHDCEASLLSQFTQVLNELQTDCNEYDRFIAELTDAERSYKRNYIDISHDVGDEWQDDPELRALVEEERKLKLQLEAIEDETVQVTVKRRELWSTGMDLEQLIHGTFAEGAFLQHLLGLSRDERQSVSVFAVHASDMLRRLQRYNVCNDVFHIWHDGLFGTINGLRLGRLPSKPVEWVEINAALGQAVLLLATIADRANFEFSRNRLVPRGSFSRVVNMYGKEYSLYSDGGMFRRRGFNQAMILFLECVEDAGRRAMKEEPSLKFPYKVERGKIGGLPISLGNDEQWTRALKYMLTHLKWLLAWISKRY

>1602

MSNNQVFECQKCDTPLNLDQSLLNLNESQLKLLVSQKKHKLFRKANSQILKDLDPQDYIPQDRLEMYQGITNQEPINHRNYFESEDDEDDEYEDEEQNNQQQKLHDSGFTSDSNSYLVLNESDEFKENIKNSHDGDDTEDRSTDERNDFRMSTRIKTLNKIFAILSNNQEIDHPLSEDCAKLLIENYQLKFDQSQKEKETYLSFLRKLKDKDSQLNLYNEDDENNEIVNPDLLHQDLDQKLYQSIQEFQKLSVQEKENLKELKKLEQTKTELETQLSNYEQELNNLRENGLNSILKLKNKLQLELNEKNKKLEQSKAAYDVQLDHIDKLRNLNIYTRIFHISCDSQDKFATINGFRLGHKIIWPEINAALGQIVLLLVFLIKRLKLDLKNYKLVPMGSQSQIIKFSAKDAVDGTTKSKTILNLYSSDEFSLGKLFNFNKLDVAMIALLDIVSLIEAKVLSIDQEIELPYKIKNDTIGGKSIRVTSNSEWTSSCKFLLTNLNWILTFVSAHTNSEETL

>1603

MSASGLAISNASAGGGNPNSVAGSSPPNLGSMSPSRAQAQAQAQAQAQAQAQAQAQAQAQAQAQLQAYQMPPSAMVPALSLPQAPIPTATMPSSTASSLQTQQSMDGNQVLGATTHVSFVCQKCSLPLKLDASLADMDQETCARMAQQLLQPPSQGDAADGDGRRASLTPGVGASQGPATGGSSTLTPSSSFAAVDLATSGSSTPTNKKNSSLKYRIRAASSLFDLMSSMGEVDHPLCKECSDQLVESLEDDLLDAEQELNYYREFLARSQEEDADPRDSALEREELQKLRFEEAGLQQRVFQLETDREIASQELASLTVQQAEVDRDSEVYWKEYSEFQRQLREFLEEHDCIEMRLQNASASLSRLNKTNIYNDTFHIWFEGHFGTINGFRLGRLQNSPVDWAEINAAWGQTALLLQSMAERLKFTFNKYRIVPLGSYTRIENVEDETRFELYSTGASKLFNFGQSSFDSAMIAFLDCLQQLTLHVESRDPQFHLPYPVVKDKIGEQSIRFVNSKLETWTKALKNLLTDLKWCLAWVSKMIPQ

>1604

MEGAYTFTCQSCRARLTLVGPVEAAPGTDSTWPITRGLPGQLQASVFDNASVDESFIVLDGKRQGASVGSHPGSGQAGGPPALRAGPSRTALEAGPALGTTTTSTTRQSPPQPVPLPQQQQQQQQLMQARGLEESFVMLGAASALAQLFELASTNTQVDHPLCMDCVGQLKEEMEAQVRSAWDRCRTLAWACTLGNVRTLIPTQGAGADEEYYVLEEISRLEQERDQERARAEVLGSELAAVAAELSSLAAASADLDSLEERYWHDVADFELLLRAHTDERGALLAKIDRAGQRLQLLKNTSVLYDAFKIWHDGPFGTISGFRLGRTPEVPVEWDEVNAAWGQAVLLLHTMAQMVKLQFSQYRLMPMGSHPRVADKRATYDLFGPVSKLWSANYDRAMTAYLACLREFGEFARRKDLQDGKVMPFNFPFAIDGDKVNNATIKLTLNKDARWTKALKFMLANLKIALQWTVKQDQAGDAPPLPHLAQEGPQLLPEL

>1605

MTGPSEQAFCCQSCRCRLNITDLDTLDQAGPSGKQSVLMNGSLFGGRNLDESFVMLAGSASVLHQGHGGHLRASNHGVPLQALDDHFAQLARTFEIASGETAVDQPLCLECATRVREEMDAAVTEMEAECEAYEAALQNLAVEDAKPLSEKEFAVEMAAAEEGERAEALRAESAEAALAVARREMEAARRRAAELGEMEARYWHDFNDFQLQLRAHVDERDVLLRKIDRTSAHLDRLRRTNVINDAFHIWHDGPFATISGFRLGRTSAVPVEWDEINAAWGQAACKLVFSSCQLQPMGSYPRVCDKKGSHDLFGPASKIYCADFDRAVCLYLACLKEFAEFAGARDASEAIPGRTPLELPYAIDGDRIGGLTVKLTFNKDTRWTKAMKYMLTNLKWCTSWMITRQEGGLALHELPGAMSHPERTLGASLASSTLATPRTVTKDDWTGR

>1606

MERVAEHLTTLKGYTLLSCTNVLNDLFYITYNDKVGMGTINSLRMGRRPNVPVLGRRANFTFRPYRPVPLGNVSRMVCDEPKRREALELHCEEGSSINFNRVSKQSKFDAAQVAFLQCVKALGDWALSLDPRGVQLPYIDDDKINGASIRYHGRQFGEKGDEQAPSRAPPSSPPLSLTAPPQWTKALKYTLTHLKMLMAWVAKRQQ

>1607

MFLLGLGSKKIEKLEDDPKVEENMIRSCSRCGSFFEVVNDSDDELDPSEIINTVESVHDMFIDYLSLEKRNPNNFEDFILQFMEVSLEKDNMTLSNKTNVEESLCVDCMNCSISQFSMALNKEISLLDKYKEISDSLISSECYDEDQMEDSEVNFEPTSKEYLEIMNIYNEFIKMEQYNQEKTIINDENDNDEIDCTETDKDIKEGYGKCRFQVINTIEVDDIVELRQNQNLESNLKHELIQYELKREGMKNHLDFLRGYLERLKRSDFLNLSFYIQVIEGGACINGLRPALFEADFDNWNEVNAALGVSAMLLYTILERHKLPLSIYPNGSYSTIKDSESSIWPLHGNTLCNSDYNECTNFDKGILSFVFLIDTVYNIIPGTNETLPYSVDQRNGTIGGISLNLLFNERESWNRAMSMNLINLKWLLVRSSESIRNKLNNES

>1608

MDLYCQKCYKQLELDDSLLDIDQSLFLKTNIHEIIEELEELSLKPVPKERETLILKQQQQQLLQQQEQAAAAAAIQSPQTVSKMSKLKSGLFGKRKELTQSNSTTPTTPATPTATPTSSSLSLPTPPPLPQQQQQQQQQQQQTFNDQSKLTATTPTQPTPIEEKKQQSFKTFYPAAHSNNSSNGSDHGGNVNTTGVSPSSPGGGAHSFGSLSQHRLSSSLSLKSISSGGGNSSSNLVADINNNNNSVNKDNNTTISSSTVTTSNSISESNNQDSKLLVATTTTTTTTTTTTILATIPTTTTTSTPTTPSTVGGTTPSPPSSSSSSSSSSSVITSPISRISPSNITSPSKLSQQSTPRQRPLTIAPTKLNISQVSSPQKPRLKYLRTSSKSLSMASLPAVGPDGTIIVDGGASSGTESTSMTNHHSSFLNQSTLPLGSSPVQTTPPLLSNSMNNSTNNLQSLQQQQQQQQQQQQQPISENNYYKYMKGLSLFKIATDLINYDLPLCLECTKLTIGELEDEGSILDGEVSIYSAYLKQLEKGKTEEDLEELGKEMTLLCEEEEQLRLMIENTHQERKEVEQLTLQLQDRIATLKSLEDSYWSCFSEFHYETFRNKDERDQTTVQIQWVNDHLESLKQTNILNDAFHLWHDGHFGTINSLRLGKLPSQPVEWNEINAAWGLAVSLLDAMAKKLKFKFQQFTLVPNGSCSRVDKRDVDPPLAYELYGTNDISLGRLFWYRRFDNGMIAFLQCIKELCEHITEKDPEFSVPYKIDKEYIGGMCIKLQFTNDDTWTKSLKFMLTNLKWILIWIVKNETLTLFNNQQFQQSKLNNNQNNNNINNNNNNNINNNNNNNVNKRN

>1609

MKKSDSSSSLVFSGIQIEINNNYGSGSGSNNNNNNNNNNNNINNNNNPNNNPNNNGPPLRPSSEWVLLSSSQPTIATTTNLTTNAPLSDSMIMNYVGNDNLNTPITPSTSSPYLKRNNSNLNSIIENSNNSSPSNAITRNNSFNMDPNNNNNNNNNNNNNNNNNNNNGEYMNSSIVFDNNVNNNNNNPNNNPNNNVHSNSHNTNNSITHSGSITSSNSNNNNSNNNNNNNSNNNNNINNSVNSVNSLMMNSDKNCNHQFPSDDVLISNEHDIIDLALNKEFLNQYRIQTNSIFINSQHVINQDVQSYQDYESLLSSYNTILNQLLLITSSNKNNNNNNNPNNNNNNVTSPNSLLSPPSTSQLQQQQQQQQQSPSPPLNNNNNNNNNNNNNNNNNNNNNNNNNNNNNNNNYYLMSTSTMSSTTTNLSTSTNSNINTNIPTNYNMSTSITATTTTTTATNTIAIGTTTTAAITTAATNSTNSNNLNLSSTNLGLDNNFLYQRILPEILSLLEKLTVFSRNNDTEIFNRLGNNVKLIFDHLNLIKNDILEANFSHLSGIFTIYNHTIISSDKTTKQFVFRFLHSLAPMTVPPAIVVTPELKKVVQKIHFSKPTFTPLCIEWKEICKYIDVLKSVSLFIAQVGQYCFLNQSIIINDELLFIPSIYVLFDNIQNLSMKEERFHIEYILHNLKFIVSKIPKQTREFTKTEIERLYSYIRGNPSYVKEILPKLLKNIYIDPYFYHLWNNHCIHLLNNNINNNNNNNTNNSSNNSNNNNNTNINIGKDIGNDLTLLLVSNKNSFLLSFDERHFSIFYSSISNSTTTTITPTITSPNNNSNRINNNNNNNNNNNNNNNNNNNINVNNNVNNNNVNNNNNNNNNNSNQYIVNYFDNTTSLLNFIESNQFKYIILSNKEVETIKFFKSVLPVTLPLPQPSSRQAILQQQQQQNYKPRILCGPCLSRFKTILDEFDNEESIENQIYSNYLNNINNNENIDPNLIDGNENKINGNDNNNNHDDDEKTNNNNNNNNNNNNNNNNNIYNNNNIEDYNNNNDNNYNFENEINDKENEFRELEKEINEIEKEEQELIKEYLELENRNQINLDIKQKLEDILKEIKLEEKTHYDLVNNYYQEYFDLKQEDEIYTNQINAIRDQLERVSQIDINKITFKIELPNTGVNGGIVGYNESSNTTISSINGFRLGTLSNLKVDWEEINSAWGETSLLLYVLASQLEFNFQNYKLIPMSSKSIIQSKNDKMSYTLYGGDNIQFSRSFLWFGASDQRFDSGMEAFLSCVNDIATFVQSKKPSINFNYRISKDKIGDSNRLLSIKIAGNSELNWTMALRFMLSNLKKILDNLDSIVESKQKQQQLQRQQSLNNL

>1610

MQPSLLSLPSSPVDPLDWERVIGPSCCVCGQAYIPAAEVDDEHIIGGGGGGGGGNGRKHSSPSSRQRGGGGARNGEESYVVLPDSKPVLSEDYFTLARNQHHAAGGVAAAVASSSLAEGARLAGARSQQASGPGIGRSVGGGSSLPPGSELLESGFSAYGGSVAGFGGGWTGSGFGAGSSMLESIVQISHAGLSDNVRRESLLYDMSQRPARRFRAGEQKLPASKDGASAAEVDGGDDERGGRLSQRQRADSEGAEGQEESDDPTLCCHCYASLLEQIDEDSRLADREALAYRDFVEVLSGIIVSQDNGLESAGPTAPGRRRKAEGFPAAAAAAHRSPPATTLPGEGSADSMRREGSDRQEHDEDDDGSAAGPAASPFAQALRMAQETSVLLRRELRSLETQRGALCARGSEAWAALSELAYARGVLGDECRELLQASREVAENAAHLSERSALSKLFSIRHVDGFPSINGFRLGRAPDDKRRLHWNEINAAWGELASGDNSGGLSQEDGAGRRGHGPEPAAAATSVVVVCAHDLFSTGGGGGRGGGRSGSGEENLRHLAAVRAFLACSAEVMAFLERRVLERRLEGEVSAGAACVAWSDVGVPYKQTEDAVGGVSIGALKGQGSWKPWLCQLVKNLEWAVGAASSLCVA

>1611

MAYRELQGRLQLAEQQVRMYREAMEHLIAAPPAACPAACHPRASPISTTAGVAQESLSNVSAGGENDPGATKPAPGAVPDVATTGGAKAEAVADGDDDSVLDHGRGGTKKGVPRLSSGDAEGAETSAGSTAGKGPNIWGFIGGAVGWIGEGTAGWMAQSEAKREHHRLLAEVGKVRRRRAAVWRERELLANTRAELARCRADLLSRRIALDSRRSDVAEARQNCRSRKALYSEQLVSAMRMGALNDCFHIWHQGPFGTINGFRVGRLNTHVVDWQEVNAGVGQAAIVLVTVAMAMGVEFTKYLIAPCGSFTKIARSEDGRVLYSLHTDESFSLFPRRNFNFALKAFLHCLGEVGDHVQEHDPTLRLPYHIKEKEGTVGGLPVALGSSYEDWTRALKYMLTDLKWIAAWAAKYLPPC

>1612

MDCQRCRQPLLEASPGPGGRGLAPLGESYVVLPQSRRASALHASLNESLSQPVGGGVLQASSIHDQLQTVDRLVGLAERCASLPCGVPLCEDCASGVVRDLNARLRDAREERDRLQAAFAELEAGDGGPSHGALSDAEFAAEEAARELEVSELRAALASARRERSALADEAVRLRAQRAEQEAEEEAWHAVINGNTLRAQAEQEEATRERQLVQFCRRELARLGRVDVVSDVFRISSTGSFGTLNSLRLGARTPRGSSGRLPGVSVALLLVAVARESGARFSSHALLPMGSYSKAYKLDEPRALYELHGSGSPHLGRIFGSSRFDKGLTMLLACASDLLAFAGARPRAGVAPAPPHPIEADAVGGFCIRLQASGGEERWTRALRCLLEDLSWLLTWRRAGAAAAATGTALHAA

>1613

MSKKPALSPTKFPNEPRSPTESFTSFSCQKCRKHLAVTSRVSADSFASAASLVQDENAWMGVSPFRSVQNSLGSNWMAVTNQFLAFVSEVTQHDQPLCFECTDLLVADLETRLRASEAERDHYVGLLQDLEAEEQSAAGAERQLAKELPALEREEEQLLQELKRIEEEQREVRRQAELTDQQQADLEAQVEDYWRRYNTCLVETVVVSERKSSLDQHLRMEMEELTQLNRTNILNEAFHIWFDGHFGTINNFRLGKTASEQVEWNEINAAWGHAALLLHTIARLRRVQWTKHTVVPMGSFSTVEPGRYELYGGAAVFWAASRFDKAMAGFLHCLNEVLQKERQTAQEPVPYEIQDDKIGGLTIKLMGNEPEKWTRACKFLLTDLKWLVVVLSTNPPLQDGP

>1614

MQDRRHRRGEGRCVSCGKAAKGSTLEDSGEDDVSLFTFAPNDPLYGSYIDIIPSQNSSLVLKQEQAQAVRSGEVTGPACVSCFEEWVEVAMQQEKEALINHLRRCKEFELAAAQDQAEYPTPEALEADIVQLGVLEQGLLQELATLEGERRRAKAELDAAVTEGDKLQAALERCEAAEIAREVGAFHSREAEAAASAKTEHVREQLHALKQLSLLPTLFDIQFAGPGIRQATVTINGLHLAWTNELKVELEEVSAAWGMAALCLQATAEGCRRPFSMFRPVPNGSTSVMEDIRVGGVRHNLYLKDMNRQAVASFDRAVSAFVVCVYELAERTAMSNALPCSMQGDLVMGTSVYLMGNSLERWSQSVQYLFINLHRLLEHVSATHISPC

>1615

MDASFVHLNVPLEEESRVGKNSVCSEWTRQTDKWTRETPQSCCWDCLSRLQQALEKDTERLEYESNVYLNHAQSSRLRQKQWQQKLDGELTREQLVEQAKESYTREIDEITLACEQLEAEFKELKMVRRELAAKAKELDQATEDIHRARNDIELAATSIEQDQENLYRAVASAHEQIDCLSSTKIQLYSQSFDLQVDQERGLRYPLINEHRLAYRPKGDVHWEEIQTAWSLAAQLLLVVASTFQRPSKTPYHITWDTQKLTTAVPFWHGMLCCIA

>1616

MNMNGESKHPDGTRTSSIDDLLRELEKIQRRRRQVKELRLQLYSEKKQALQEQLNHATLMQEQALLDYRSALYLNQKNRNLWQLAGQWSSWTDCFQISHRGAYGTINGLRLGAEALALQSPSAEEMEVSEELATNNAPNTGKTPAPRLPFSFLLPDTNNNNNNNDNNVASNASSTARPTIKVPWVEINAALGHVALLLVTMQERLQSIDAEFVFRHVIKPQGSTSQIGIRRNSNQVSSWYPLYSDDNSFPFVIMGKRNFQLALQYLVECVVDAAHAIEKYDRTIVLPYPMEQQQHQWTVGGSSVQYNFNSGEQHHHNDAMEWTRTMKYLLTNLKQLMTFRAFHLYGDT

>1617

MTIECTCNLRYGVEEASSPLTMEASFVHLNVPLEEESRVRKKSVCSEWTRQTEKWTCETPQSCCWECLSRLQQALEKDTERLEYESNAYVNHAQAARLRQKQWQQKVDGELTREQLVEQAKESYTREIDEITLACEQLEAEFNELRMARRELAAKAKELDKATEEIFRARNDIELAAANIEQDQANLNGAVASAHEQIDSLSSTRIQLYSQSFDLQVDQERGLRYPLINEHRLAYRPKGDVHWEEIQTAWSLAAQLLLVVASTFRKQFQIRFHIIWDTRKLTMAVPFWHGMLCYIA

>1618

MLNKTNVINDAFHISHNDVYGTINGFRLGSIQTQPVEWDEINAAWGQTTLLLQTLASNWDFTFPHFRLVPMGSFSRIIKRDDEKCVYDLFCASDIGLSRIFGFGSFDKGMAAFLECVRALQEHVKSIDPTFSPPYRIVEHKIEELSVKLQFNTYDKWTKALKYMLTNIKWLVASSLSRRS

>1619

MSNTNTNINNNNNNNNNTTNNNNNNSSNKNSNSSNSNYNNNIESDKDGIQQPISDWVLVRNSISSTATTNNTNLNANSNTNNTLTNTTTTTTTSFVPLNSSTPTTSLTSSFANIQIQQQQQQINKDDNNNILNNNSFKPISSNPINIPGLPGRKNSFKSGMETFLVGSHTVTPTVSPTRSPTHNQYQYTHSHSVPNFNTTGGGYGTTAKSTTGSLGESSTALYNQQQLQQQQLQQQQQTLSTSPTTSSLNTSHDKKCYYCAKDLDRNQHQYEHEIVDIAQNIERLQQYKIQQNFLHSITPFLSPPTVLFTDVLKKYAKISITKQKNTPLGCEWRIILIFTKSLLEIIKFCDPIKAITLLVYHSSSQTPQYFHVKNKPIIINEEKQFIGSVNHFCSDLQPLLQKEEKFYLVYLVNNIKHLLSVIPKQTRELSKLEIDRLQILMKKYPNTYLKEVIPFFIKNLYVDPLFFNLWNQNCIHFINTKDGISGVGGGIVLSNREVQPIQYFNSILPSTLPTQPITTPHHITLCGPCISTLKEYLERMNQELQRDYTTYTQFIQDHNINIKQDNNNNDNNNNNSNNTNNITTFNSNNTNKQSFTPSIHKSKEWEDLSNLSNEELDAQIKLIELEEIELMNEYLQLERNNNDVIDIRNKLEDIRGEIKSEEKQHYNNVNHFYQEYFNLLGQKKSLESQIATMKDDLDSLSDLNIVKEVFQITFEKIDEHTIVKINDLRLGTLPKNKVEWDEINGAMGQIVLLVHTIAKQLNYTFTKHKLIPMGNKPLIQSKSDKEAYPLYGGDDIYFRSFIWSREHKFDIGMEAFLGCINEMCSLFKNVVFPFKIEKDKIGGNNGFQSIRVTGNSEVNWTKALKYMVTNIKCILTNLNLFGQQITNSPTTTTTTTTTTTTPTK

>1620

KKEEEEESIKLNCNSCKEEKTQEQLLFFTINYEKQYYCFECKQKLEQGTKEIEQLEKQINEIQINLTEINSKISHVTHEINLIKTSEQKNETEIEQEYKQLEQEEEELNQKIFELKEKILTYEQQENEYWDEVNIFEKNLYQVLEKKQIAENQEKHYNEQLIKLNTYNTLNQVFNIQCDEQAGTINNIIIGKKLNQEIVWDETNAGLGQIAMLFSYLVKKYGYEMKFIKDINLCANESQICEIEKNQILQLYGPLNYQSEYSRFDQAIKLLLKEFYCFYEFLINQVKIGDYVEEVKLPYNIDIQKCEIDYVEISIMKNIDLWTSGMKKFLINFKYIIYINELYYSKKEIEYLEQEN

>1621

MSSGSSPPPAVLYSTPVFVCQLCKKLLVFDGSLGRVDLSLLSQGERSPEKLLRPEASADTSPWLMPDTSTPAQPRRVQSRGPAEAPTTPPDALAGSARLSYGALSMRARSDAMASAAASVVVGSVPSRRSRLQQPPLAAAPGTPQQQQRAQRHAASRSALRSTTSGARGELCDASDGDDPRASGGCGGPSIMPAAALVHHSDLFAVASDASGIDSPLCADCARAELEKLGQRAQSARLEAQRYAELLARLEARAEAARAGPASDELLARLREQERELSERLRVVAQAKRRVMHEEVLAKRHARTLDAVEERYWALFGEHTRRAREFDEALRCARAARRHDKQRLWSLCRANALNDSFHVQHDGHFGTINGYRLGRLRSVPVSWSEVNVALGFAAQLLVTCARILRFDFAQFQIVPLGSSSWIQRKDTGATYELSGDTSFWRRRLDAALVAFLSCVREIVEQCQRSDPFFPPPHKIDKDRISELSVRSQFNDDDKWTRALKFLLADLKWLVAWIAHQPNPGLVFLQPQAGAAPSPTLTYNSS*

>1622

TAQPAQPRMATTIATPVCEQCRRALPEYFVYVGRGGGRQERGPGDPEPRTEQVRLREIADADPARVLGVPHLCNACADRVVHRIKAHMREEEAYRQAYTTAIDDLQRIAPAVTADIPVLRSRLSSLEEEERELQKAFAQAEAERASAREEEEEIAGEEQALCDAEERHWSELNRFFAHARQSEDEASAYDAEAAFCRAQSRVMARVDVYREVFVIALSPQSIAMINACRLGRLPAARVEWDEINAAWGHAALLLYVLSKKLRVGSASQRYRVQPFAGRSKIQIVPQQQPSSSQSAVELPLYGSDEVRFAKAAPGAGLWPFGSGGGSQHGGAQAAAAPGSQLAFDDAIEAFMSCLWDLVLRCRELCPGVAIPCVIDRDRIRYKDKEYVTVRTLSSTEESWTKAMQYVLASMNRVLVNLPRLLQAPGLGRHRQNSLSMPCSPCTSPPPPSSL*

>1623

MDGHGAVAAASAEGRGGRGATHFRCQGCMATLDVSGASDVENDDPGASTGDLTTTTTTTKGEGAASDAASTRRVDESFVVLAEDRDRARGAAAADAMTSSSSSSSSSSHPAAAPPASMAESFVVLAEHARRSTGDADRAGLNARFAAMSKISDMASDPSRARVPICVECAARTREEMDARARALEEECDAYERCLKELDAADAAATWRPDDVAAANGVGNASATSATTTTAASEEDVEKEAARLEAEARDAIAAAAAARDELARLRATRDALTREHAALDDEERAYWHEHNAFKRELAAHVNARDALTVNIEQTTRQLARLRRVNVLNDAFRVWHDGPFGTVNGFRLGKLPSIPVEWDEINAGFGMACSLLHTIARLRRVKFTAYTLRPVGSFSKVEDAKGHAYELYGPVNILSSHRYDKAMVGFITCLKEFAAHCAECDVRNGVTPPFELPYTIGASVPRFQSPPSTHLDAFQLLHLTPFNSTPRAADGDKVHGMKIGFPFNRYERWTMALKLMLTDLKTCLAWVVNSEDGGGGGGGGRRERGGEEGGRA

>1624

MNVSDPSLTADEISAEVARLEEESIKIGFDFPMCPTCQYNEFQRLQKQIEQIQSETQMIEKRSTELDCQLKAVHQDEEIKRLKEEEIALNEELSQIDKEIEEAQKLKEEFAETEEILDYIESRSWELRNEYLMEMESASEEIQQLEAKIHRTQKHIELLKSANPLCDAFVIRCEENSINGLKLSSQLDARETNAALGMCCLLIHLLQKKFQIDGIRIIPLGSFSWVQYDAPSSTTTAAASSSSSHQSSSLASASQSITNSGSSSVPSSSSSSSSSPSASAPSSSGLAPGATKNPGGSGKKKQIRPLFFSTTPKDHMQLFEEGLAALARAMDELTQYLSNRGYQKPSSKLTMSEFQDKEEVKKPLKWKFIDGWGRDFLLWLDHLAHFASHTPAAP*

>1625

MQYQGFNRFARLDFLARCCPPLQIPALTLAQELSATLSSPELYESACARLKVACEAQGVPLPANMPTEAEMATRREQALMAEAQSDQPLAPHEQLGDRATLLRKSHIEYERGDLTAAARYLARVPHYCKTPEDMLQYSLESARLAAEQGMWAHVITLVNKERLSKILPDHPKAEAQFNCLEGLATFMIGKLDKSVQLLASAAYTENGTACELMTSRDLSLMCGLSALASLDRPELVLLLQNTELKQHMESCPEIRELLRAMVKAQYRSLLDILQKVKPLAMADMYINKSWARLYENIIARAMIQHVTPYVALDLHAMAATFNMELEALEEQLVTLISEGRIAARIDNQHKVLHATQGNVRAAVIEDSVQTARDLVDNTMQEAHEQEFVRLHVDLAGTMDDLLIGLEAEAEAVDVTASTPGDKPLEPAAASAGASSPPADQQTPVIDVELLPYAGRAIFPLFRLSNLLDCAGSHDWAFLLTYLTPTACSSSGENTNMEALEQALGAMQNPKLLQELFDHLSSNSEIDHPLCEECTNSLLNNLDRELRDLGERRREMNDFLAASQSHSDHAPPEEELLDQLRLVDEEIETLSKQLTDLDAAEADLRSAIGDKEATIQELKNEEQRHLQSLHEAQLDLQDVDDERAELEIQQQELSDQLEFLRRTNVLNDAFHIWFDNHFGIINGFRLGRLPVVRVDADEINAAWGQALLLLHVMSLRLNVHFSKYQLVPNGSFSRIEEVATKAKLPLYMSGSRSMFGYSSADFDRAMVAFLHCLDEYRSQIQANDPHFDLPYTIQDDGLLDRRQEKLSVRLKSAEETWTRALKLMLTDLKWCLAWLCKQMV

>1626

MSASSRQFLCQKCNQPLGIDESLQDINSAAFDLLLGPLSEQSSGSSTSGHDSAAHAHSHQGSPGQTHSQKQGQRPGSSASNDSRPEAVFTRTIPPQNSSSTRANQDQGRSDASESFVMLPKGQPSDTLGSHSYLARGGSIPPLGNYPGGKAASTLSSVNGTGVAQGANGARKPGYSGGVSGDHLDQLSNRTAKANRLRTTGKLFDLMSAKSDVDHPLCQECADMLLDALAKQLRDVSRERDCYIDFLRTVNSNIASDAEMEALENDIKLIQEDENTSIHALRDIEEQQKKVREEIALLELESLELDKEEERYWQECNEFQLALQNFHNERDSINLKYDYDSRQLEKLHKTNVYNDTFCIGHDGHFATINGFRLGRLPTQHVDWPEINAAWGQTLLLLHTIANKLNFEFKTYRLVPLGSFSRIDKIEGDRASYELYGSGEFGFGRMFLNRRFDNAMVAFLNCLQQLGDYAEQQGPRLELPYKINKDRIGDASIKLQFSQDEIWTKALKYTLTNTKWILAYASSSAASNMTSYTPSAPSLPISRRSSPAVRSEPQS

>1627

MNVKHKYPCTHCKSEVYFQPSSTMASNSAALDDGFVFITSNDGGGGQTPTNNTTSLNIECNHSGKIERIEDIEYLTEDILSSITIGEDLKKNTKLSTLINDLYLCNTCMTDLFGGLSEEIEQLELECKLYDEKLKKSKKFDTNGMYKKYMKEMDELRNEELVLREQVNVIQNDRHRIGRNITSLQTEYNTQQESEKNYWKIYFQFIHNNQFLAEENRRKSQLIDKEDKIRRKSPLAGMFHLWYEGSYGTINGLRMGRLRHDPVSWEEINAAWGLAVLLLDLTAQQLNNFKFTKYKLVPKASCSSIKELESGDKYDLFFSSTRNIQSFNEGMKCFLFCLEELCQHIGIVVPGSSIGTRLIVKADEGKIQDLSMLYSVEREYEWTKALKYLIINLKFVQKNVIK

>1628

MRKNKGSLSSTRSVPNLSALRTNNNTPQVLTNNISNAILGSSTTTSTGSSNSTSQQATNGTSGVSNINAATTALSYYYQHTPLPPHHQNLLQSISSSEATPQLSADDVQQLSEMLYFPKYKKAVHSAVCYRCKHQVVQIMFETNGDEENPPKRAFLKVYDPSFYNLNYDQNMSSFEESENSDGQPTDNSFTPTSYMGTYESSPLTSLNESSGYLPTNSDQPSAEILSSSLSKNVSFALPRKTDAVKDPRPTTPNNPLQKLTQQDKKRFSFNPNAKKKEQQQLRQLQLEKEQQQMKLQQRMNQLNEDVDFLDKCVISNEEYEMNVEYVKQLYQYLSDVTQLDSPLCIECMEQVSSQLNQQLEKTKEEEAIYTNFKSKIIEESSGMKNEADLQNELDLLEEEEKLLRKELDLLQKEEEELSLREKHQTEKEDKFTNLQESYWKEYNDFHNELLLYNEEREAIQQRIIHVTKEMEKLNSTNVFNDAFHIWYEGHFGTINNFRLGRLPTQNVEWNEINAAWGQAALLLFSLSKHKHFAFSKYKIIPMGSFSRIETLDNKNSYDLFGGGSNGGLFWQSKFDKAMVAFLHCLKEIGTFAEKQDTYFELPYK

>1629

MPSEGDGPEAHILHLREKLKAVEARRITLHRRRALVEKKRRRLQGLLDLWQSLNNSAAEALLDLSESHQATHGSELSAGARLEALTQLNVANDCFHVWHSGPFATMNGCSIGRLPAFAAVEWTDINAGLGQAILLLDTLVKQTGFRFRRLELVPFGSFSKILRSPCDLYGRGDEVDAEQSSNHRQQCHEFLLRRGQPGVFPSSEHECGAGCIPAVRGGVRIPRGGARPHDPPPAQGAGQQDRRSTHRLDARRRGHMEPSSQVPIDRPEMALGLECSTCTAV

>1630

MPSEGDGPEAHILHLREKLKAVEARRITLHRRRALVEKKRRRLQGLLDLWQSLNNSAAEALLDLSESHQATHGSELSAGARLEALTQLNVANDCFHVWHSGPFATMNGCSIGRLPAFAAVEWTDINAGLGQAILLLDTLVKQTGFRFRRLELVPFGSFSKILRSPCDLSDVETKLMPNSPRITASNAMSFYYDEANLAFFPRRNMNAALVAFLQCVEEFGSHVEGLDPTIRLPHRVQGNKIGGLPIVLTPGEEDTWNRALKYLLTDLKWLSAWSARHVLL

>1631

MSNIKQNSYVNFFCQECRKPIKLEPSLLEGTSERLFALLPDKSMDDEGSRNLKSNLKTGDPLTLIEKPTESFVVLSTTSTSNVLTAESVNRASLSFRLKVVKKIFDIISGKNEIDHPLCNDCAKILNEKLKQNLDKIKKEIVSYQSYYDQLRLNIRNSPEETETEIDINAEEEEINKLKSKQKSNSETLEMLEKQLKELEIESTKLDQESKELDEREQELLKKANLFQQIMDEYNDNKESVEINLKQMEEKLVQLRKANAYNDTFRIEYDGAFGTINGFRLGRLPSYQIDLCEINAAFGQTLLLLYSLAKKTDFSFKNYRLIPNGSYSRIEKIDDNSSYDFFIRENAFGRIFKSWRNEKALLVFLECLKQFSEYLENNNNTDRAFQLTYKIEQDKIGDVSVKSKEDEIWTKALKYILIDLRNIAIHVT

>1632

MTSSNPSSPSRARARVSASPDVEHARTSVRVRTPSAFACAACAKTLEFVNVPERSRNAPMEESFVILAPELALRARKTLGEEAWRRHALGGDAVSREGGMEASFVVLPERAEKSGIEPSVMARVFDVASELSERDHPLCDACSSLALTEVERRTREVEAECAAYEEALERLREAETEEGSGAGVSDVGGTSKAISTAVMDMEKAERDAEETLRELELELESTRAARSKLAKKAAALDEAEDTYWREFHAFKRNLNSHLEKRDSILTRTEQAQAHLDRLEKTNVFNDSFHIWTDGAFGTINGFRLGRLPNVMVEWDEINAAFGLACLLLHSMARICKFTFTQYTLKPMGSFPKVSDANGGVFELFGPVSIISSHKYDKAVLGFLTCLSELSEFMKARDVRQGVNPPFQLPFTISNDKVDGKKMSFTFNRDENWTYALKLMLTDLKLMLAWLSHKD

>1633

MEEQEECSDCKIIIAKKEMYCEKCLQQQMKKEEEEVKQLENEINELQIQLKQVRDEMYKMETNQIEKKNDKIELDKKNLDKEIYQLEQDEQQQKKELIKLKNSILEQEHNEDNLWEHINKFTRKLYSLFESNQIADNKLSQLNNELDRLSKLDVLNDLFKISVQDEVATINGLQIGKKGEQPVDWDDISAGVGHLTLLLVYLMKKFMYTYQKIESIELNGSFSKIKIKDEYQNTLCTKTLNLYLNNKTSQKDEEEFSDAFLQLYFEFQQFCKYLQDGDILRKRKINLNLPFTMNGMSVDNKILSLKQPAAQKWQEWTNSVKKFLANVKALIVANAQFDLSL

>1634

MDDRTLDCEVDLQLERTAAHIRRTSVAEPRAAIAGSDRFQDDLATCSDEESLRRKLVSVRRRRLQCHRLLEQAEAQQNALDEQLLAVIRQRDEAVLDYRSVRIAIATQQRQLQISRQWNVTNDTFHIWHSGPFVTINGLRLGAEAPSMEVGSSADTDRQPNGPIEQPRRYLGFGAQPAPSTVNPSSNSNEVRNQEGKNTIRIHWTEINSALGQVCLLLSTLEQKQCCGIRYRHEIVAQGSTSKIGIRSINNQHTAYYNLYSDDSFQLFGKRNFNTALQALVQCVVDAADAVQARDPTITLPHALEKTNTRSGDYVVGGLSVAYGTDGVEWTRAMKYLLTDIKQLMMFKPFALRHARS

>1635

MNQSLETHSSPASCECGQHIRLEHGLHDTVALLDDEPQKSFPLHAHTAALDASFVHLPSSILRDVACCPASSEPGGREFRDTLHDICLLEQALHDSDNPPHLCLECIQRVEQALLADTERLKYETKRYDEAVRDERDRQRSLHQALNAVSFAGLGDNFQPEFMSQSADADSSSPLLQRAEEAFRNEIEALQQCCDQQEDEIGHLRSLVKEQELIKQELQATELLVAAERNSLEVEARAFDNDHEQLCDHLLSIQKEVETLSSSQIRWPAFFLDVRIDTRGLRYPLINDLRLAYRAKGDLSWNEIQTAWSLAAQLLLIVGTIFEFASQEWKIVPLSHCAKLIQYPGGKKQPPTVYNIGCSGKQTSLALLAWNALLHEIIKDTSEKLNQAHKDGLLDLSVLPPLPFAMAKTTIGDLNLLRLSPEDDVRWSQAIQYTATNLQWLSVCTSAHTMQKSLTLYLIAHVRQKHVGPFIRPSSTFRILNKLVVNDKL

>1636

MENEMEAIAQQQEVMNETEKALIYEEADLWGQFNGLQLQEAIFQEIRDGGTAQIDAMERKVASATHLNILTDVFVIGYDGEFGTINQFRLGQSASFAVEWNEINAAFGECALLLQTLATMVGLDFSDFKIVPLGSFSKIIRTSNLRMEYYQQNFAESHFNLGLGAWITCLGQLMAFVRARDPSIRLPYKVAKHSIGGYSILFLRNKHKEWTKALKYALTNLKWLLTWVSARGYSISAANTATSDTVSTSSKAALAFAADPPVMRNNGPKQSVVIMEHFS*

>1637

MSSDGSLQPPLIITALPRPPATTGALSAASVSSLSPAPSSASASFSPYAKSPAPSGRRVAMEGKSLFTCHQCGALLKFQYNPVSIEEKIMKLRPVVYEHQTERCQQQDSKGDDEEDQMPRLTEGTDNGETTDADDEEEAVQAARSSMSQQQQQKTPAVVPEIVWERQRLDNSGDVGVWVPAEFDLMNGVAAFTSCNRGDIGQNIVDVVPGRLLNKPHLWLSDWEVDHSLPSSDDEGWVYATTYADFGRMEEPLERLVRRRRLIRKRRMDGSDLSWFQELLDCRCVAYTLLLKKRLPTCHDCEKTVLSRFTQCLEDDHDQYEQFMTEFTSVERASARAISEDGDYETDEWHDDPEVRALEEEERRLKAELEAIEDETLQMTANRRELWSTGKDLEQFVHDTFAEGAFLQHLLGLSRDERQSVSVFAVHASDMLRRLQRYNVCNDVFHIWHDGLFGTINGLRLGRLPSKPVEWVEINAALGQAVLLLATIADRANFEFSRNRLVPRGSYSRVVNMYGREYNLFSDGGMFRRRGFNQAMILFLECVEDAGRRAMKEEPSLKFPYKVERGKIGGLPISLGNDEQWTRALKYMLTHLKWLLAWISKRY*

>1638

MERNGPRPINDKEQEQELHQLRKRLESIRKRRRRIQKLARIDCVEQQKVLLERLNARRRDRNQMVDEYASTLKGRDSASLFLECARRWNVLNDCFYIWIDGKHAFATINGCRLGGEAVPLPTELLVSARDNNNNNNTRKGKWVSPTKKHTATNTNTNTNTNTNTNNSHGTRGSSPPRRRLLGLFGASNSNDTNTNTNTNSNAVGASSAFRRKLFSPAPTITEPTRIPWLEINAALGHACLLLKILQESSSKNAGTGMKFTHELHPMGSTSKIGIRFGTPDSGTGVLAAAAGFGSIILSSSSSSGNSNGNGTNSIANGTTPIVYNLFFEEASGFSFFKNNVRDFNWALQAFLQCIAEAAAQQTDKTIAIPHAIRHEKAASEYHAGIKNGPRTYTNDNSANFLNGGEWTIGGLSICHPSQTAAAGNVTGDQGGGRVGGTTVGRTAGISAVNSAALEWTKACRYVLTDLKWLVAYSAKHVDR

>1639

LESGTKELQLRLQSLRDERQHLTKELARLDEESHKLEELEQMFFEDANDFRYALENVSDQHAAVRQKIREVKHHLEVMKSTNVFDDAFHIYNDGHFGTINGLRLGRLTSVEVGWEEINAALGQCVLLLDVLTKRVKKFTLKGFELYPLGICSEIHETKKGTTGGLKKSIHQMYGSQKLFGYSGCDKALECFLECMNQFCRWIQTQDKKFKMRYQFWITFTFHFFFLHIFHKQN

>1640

MKCQTCHLPLQLDPSLEGLSLTQRNLLLSNNSIITATNENVISNKGIEAADNCGPQIPKERLRRLGEIQNIKDLNLKDDKLITDSFVFLNHDDDDNANITSNSREDQRYGNANGNDNKKANSDTSDGTSTFRDHDEEEQEATDEDENQQIQLNSKTLSTQVNAMTNVFNILSSQTNIDFPICQDCCNILINRLKSEYDDAIKERDTYAQFLSKLESQNKEISESNKEKQYSHNLSEKENLKKEEERLLDQLLRLEMTDDDLDGELVRLQEKKVQLENEKLQKLSDQNLMDLNNIQFNKNLQSLKLQYELSLNQLDKLRKINIFNATFKISHSGPFATINGLRLGSIPESVVPWKEINAALGQLILLLATINKNLKINLVDYELQPMGSFSKIKKRMVNSVEYNNSTTNAPGDWLILPVYYDENFNLGRIFRKETKFDKSLETTLEIISEITRQLSTIASSYSSQTLTTSQDESSMNNANDVENSTSILELPYIMNKDKINGLSVKLHGSSPNLEWTTAMKFLLTNVKWLLAFSSNLLSKSITLSPTVNYNDKTISGN

>1641

MTSTSATTPQTTMVGFCCQACRAPLKLDADFSDVTAGRRETFARELSSVKAPAEPTVGDADDENFTDIVDDKYVRPNFGNLDDNSSQNTKEQQNQSAMALFDFLSSRTNLDHPLCQACTDSLLDQLDDELQHAHKEKQDYEALWDELSSLKVTTSVEEIEAEIAELEKQEKEALAEIEEQEKQRAEIAQMKAAQEAELKVLEEEEEKYWREYNDYQRQLIEFEERQDSVEHQYHQASQHLEALKKTNVFNDTFHIWYDSHFGTINGFRLGRLPSVPVSWGEVNAGWGHTVLLLYIMAQRLGITFKGRQLLPNGSSSRIRVERSDTEPAEDLPLFGSGSRFFSDPKFDSGMKHFLECVRQFKDNVDSHDPHFKLPYAVNADGTISDGKQNLSICMQNNTEENWTKALKFMLTNLKWCLAWMCKQMAS

>1642

MHAFLCHVQEVVSFLRRSTSLQLPFKIEKDKVGGFSVKGSHFDQERWTKALKFMLLDLKYLIAVVESRDFASSRTACVIRVFFPVRFVSSPQQVALNILGQRLPPPPLSLKLAQLRPWLSPGCFSLLCRCSSLLWLRTKDTVRSATPSLVATPKWEASPNLRTQLWVLGATCTVGLVLSVGWMIKTTPGKDQ

>1643

MSDNVPIADFTCQYCSRPINLDESFASIHPDVIEQSHLASSSDDDSYSDDGEITPTRTPHESPALSPIHSRTRTPTAHSESTHSPHTTDKASVGTSAHIHAPAPVAARGDADSDSKPNTQGPTVSGGTGAPKIGPTGASDPPRAKVQLNANATVHVNADDRSSEPIRVVVRSPPRSPDAKRDHGAPLRDGVCALSPAMRARAGGSADVSANASAQYSYVVLPEHEHTDLSDEGTPGQHPRQSQTDANTTIATAVHRKTWGSADGDPGTQTNSRPTPNTYTHMRPAGGEHVRGNVSGIEKGLGVYGASGDGDRVGERVGARGYDVRERLDVMVLSDLFDYASGESNIDHPMCAECADQLVRVLEDQLDEAKAEEKMYTDFVDSHYSDEQEIIQQAQHDKEQLAIYAAEEAELIAQLEVIENERAETRADVIRLSAERDELKTDEEMFWKEVNEVQRQVTDFVQTSAQVHTNHQHVLHTLERLKRTNIYNDAFHIWADVRGFGTINELRLGRSAHVQVPWEEINAAWGQAVLLLDTLASHIGLTFSLFRLHPQGSRSRLERLDNGQHLDLWLSSGLQVFKTASYDQAMVAYLNCLHDLSKRIEVMDQRFKLPYEQTPSQKDKIGGASIKRGLPNTDEKWTKACKFTLTNLKWCLTWICKCVPDTQDR

>1644

MLVAQHGAIRRSTMERWGPSDFMLASYFCCSCSQPIKLDPSLLENIPYEEAVATVVENGSQISPQIMEGSTPSLNHGTRSFHPTPSSVNRKGSGNKLSIPIQESFVMLSKSQVSPLLVAPQRGSKGGTAEDQQRGSLSHRLKTAGKLFDLISHVSQVDHPLCQDCADELIIKLEKRLSEVRKEKEAYEAYLATLVADEEAGEGRRVTEADVVMLKEQERNALDVLKDLEKEKVALKEELEVAKTELEEVDALKLNYWQEINSLQDELQKYYNERDSVNLNYEYVSNQLEKLKKTNVYNDTFRIWHDGPFGTINGFRMGRLPNQPVDWSEINAAMGQALLLLDTMASKLNFTFKGYRLVPMGSFSRIEKTDGDKGVHELYGSGDLKGMLFWNRRFDNALVSFLNCLQQIGDYAEQRDPKFRLPYRISKDKIGDTSIRLQFNQDEAWTKALKYMLIDLKWILAFCCTRMNTRASSSSAI

>1645

MEINSCLVCSEALNNENTEQNVESENMEVSFYEDMKKTQILNNICLTNEGAILCKACKHELIQKYSEKILVLETTNKNLTQSLEDIKDQLGFINTNKHKNPNFLYESLKACDDTEKSLQEQIKNLEISLEEADKNIEKLDQEDLIIQAKMLETLDPYLESNTRKHVIEEEIKYLTNEDIMLKIFVIDSQDKIGTINGLRLGRLDHILVHWDEINAAWGFCALLLLGLYQKNNLKSSKISLYPLGSASRISYMKIDKERFELFFSDYDYTGKIKRFNKAQCMFLELLKEYEDIIQDKGVPYNIVLGSIGGISIEFNPTQKTSWTNALRFMLQNLNYFLNKL

>1646

MEDKPRCYYCKIQVSLVDSDQPLIPSSLLQNLLPYNSEKIVEIDRVLLLASKNYPSQQYVCLNCLENLFSPIEKETNLATMNKKSLEEHLNLFKKELEQQTLDNSDNSLIFDLEQKLQNLKNESQILANELLKHENTLSELKKEEQGYWIDANKTEMDLLLFEEGHSEVTQRLKIAEDELKYLSSINVLNELFYISTCNQFGTISGLRLGRLDSDMVSWDEINAAWGHCVLLLSTLFRMKGFVSAQCILYPLGNCSRISQVHDNEKKYDLFMTETSFSKNLSKYNLAQSLFLDVIYELSLLLEAECPRLLLPVKIENRIFKVNTMEIKLDAGNKEAWTRGLKYLLQDLKYLIYICVKEDYLH

>1647

MEDKSRCYYCKIQVSLVDSDQPLISSNLLQDLLPYNSEKIVEIDRVLLLASKSHPSQQYVCLNCLENLFAPIEKETDLATMNKKSLEEHLNLFKKELEQQTPDNSDNSLILALEQKLQNLKNESEILAEELLKHENTLSELKKEEQGYWIDANKTEMDLLLFEEGHSEVTQRLKIAEDELKYLSSINVLNELFYISTCNQFGTISGLRLGRLDSDMVTWDEINAAWGHCVLLLSTLFRMKGFVSAQCILYPLGNCSRISQVHDTEKKYDLFITDPSFSKNLSKYNLAQSLFLDVIYELSLLLEAECPRLSLPVKIENRMFKVNTVEIKLDAGNKEAWTRGLKYMLQDLKYLIYICVKEDYA

>1648

MSDNLAILKDFDNDTAIQLIESAYTYLLDKNSLPLQKLTFNVRAPFSAIYAFLRKQPAKQINKLEQLSLSKEIKQAIKDKLYLMYAQNYQKVGLLTNYKLECMNPKAIEVQTESDLVQTHKNSPSVLDYASCQWKINVILSTNYLNKVLRPEVVIEIQTAQNEKVLMTVSVEKFEELRRQVAYLIRYTQQIECIRYLNQTQSSILRESFDDLNRDLQQHDIQHNIYQHDLYFFGMSGDAADNSNQVDKELKIKDMVECGPGQQLQLKYPVCFECFDSIIKKLEEKITGEEEERDLYIKEIKKIEGKLAKIANQKESDLEKELKSLENEEAEMDKVLAQLEQEEKNNEEEVQRLSKVKESLQAEEKAFWRDVNNYEKNLAGFQESLSQTDYLIQNLDWQFKRLRNTNFINEVFYISTLDEFGTISGFRMGRLPTTDVKWEEINTAIGQSLYLLTVLAHRFNYKFELYDIQLCGAFSKISLKSNPKMKFELFMPSNEDRFNNGLVCLLNALNGLCQFVQQNYSQQRQNILPSQDKSIQQERQKVVFKIQSDTIHGISIKYNSNDLPQWTRACKYYLTNLQYLIYISVIRDQYEQGQSQQNF

>1649

MSNKIILEEIDENRFCEKCKVGERKDLNVNLYLVDFYQFPFYLCEPCLKIQLKNEFQDIEIIGQEITETQKKLQLVDENMISIKNKIEENEKLINQSQQQKQKLEIEELKMKEQQLNQEFEKLKKNVLDFEIRESKYWVEVNSFEKRLQTLLNKKQIADNLEQYYNDKLNNKLNKYNTINSVFFIEVSEQVGLINNLMIGKKLNEDINWDETNAGLGQVILLFLYLMNKFGYQSSYIKDIQVCGNESYICEANKSEQLYLRGPFSNKGDEAKFNRAMKMILDEFSLFVDYLQQTYLQNIQLPFKIDQSNSSIDGLLLTTQTAANAQYKGLDNWTQAFKKLLINFKFLISFNAQYDSKKEIELLDRE

>1650

MSCSICSLPSNNSFHRQGSEASSRPSQKSLPSPANNVSWAMPLRRRRVGLLPTMTNVSFDASSVNVSASDGNVGGSRELYDCDIHLASQRMDSFLKATLDMLGDDQNTQNTVCKSCLDRISSALDTHSEMLLDECAAYDEAAASEEERVSSVRRALSSVVKMNSNNNIYEEIELNALSSACNEQEQELRILQNLMYDQMCRSKAISDEEERVFHALNALEIDARNFVEESHLVTNMYHSVSEEVEAISHVKLMSVPFNVVISQDTGRYPTINNLRLAYRVNEKASLSYDEVIAAWVQAAQLVAFTCGLHPGFVSPHLRIIPLSHPCAKIVATFADGQSVHNLGWDVVSGADRSKHIDPPSILCFLALLSQLAEHIVNATKSDPPPFSMTLISIDSVDHGRQLCIASQ

>1651

MADDLDKSRRYGEITDVNPTPSIRASSNGWHSDFQAEQSQVVDEPPPHAPLHLNMTQDTSHKPSNQLHRIQRESSSFNESGSIATKELDGELSGLQAAAVKTALMVDGKVVREKPLHGVVTFEEDDEDGKDQSYHWIFHKHVGFSSNTKHLPPNYHGHHHLSLDSDAHSTEGSSTPNQEVVTADTLSTYQNEKNHLIKQLASLRSQRNHVQRNETTQLHRHLNEWNYRLSSAQQSHSKALSTLEEVSRQRICSEEENQQTSKWHVLGDVFFIHHRGPFGTINGVRLGRSAITAVGLVTKCAGKGGSGSGARGNATTSSESAGPVSPASNGVSSFFSWGNVDNASSDNKNVKAQLNDAQPGNTKRNIINRQTTSPERVVVPWNEINSALGQIVLLLYTLRHTPHGGIDFTKHILQPIGSSSKIGFLKKHATSNKATSTSPHVTERRRITALSAYYTPDTSGQTAAHEKTTSSTQALPPHEVTWYNLFHYEENGSVLSMGYYARRNFNAALEGLLYCIAEACLVVEKRDMALAAPYVMSVSGLVVGKDVHGTGIVVGSTKHGGSGSGEATIGGLPLSYDPADGERWTTICKYILTNLKWLIAYAAKHVDR

>1652

MASVARDSSDGQGDGVAIADHRRPAVGVSVPTTGGEPGGGPGVAGSVAAVAAPSPASVLGSWGTPVSHSPSAGVHWTDTPSPGGTSGLAGSAFGASAAPPRVDVFACQHCKRPVHVHDSLLSLELVPRPGVSREDEASETAGASSGELPKLSLLAVLDDDIEEYVVVDDEVACAVDHNDARPLLAPVASFADKLGRVGRIFELASLQSDDEHPMCRECAGGVVARLQMQIDAAGRTRDAYAGYLRRELAAAADSKDEAAYAAELAALEVEEAELERELAAAEAEAAAVAGETAVVEAQVSELDGLEASFWLEYNAFQESVLEFEARRLSVENATEVAYAALKELQMTNVYNDTFHIWFEGQFATINTFRLGRLPSVPVSWNEINAAWGQAALLLWTLAGKTSFTFTAYTLKPMGSFSRIYRNDKPNHSGFELFGSTSGFSKRLLGNRSFDKAMAAFLVCLKELGDHIESIDPNFRVPYQIEGATIDSLSIKLATFSAVESWTKACKKTLTNLKWMLAWVAKRE

>1653

MMVPRSLHRCIDCAAPLFVVHDAVPPACLLPEECSAHGFPGETPRETRRLSSSSSSSPAASGTSVSQIALGMPAAFSVAANSEDRNEGGDSVKVPPHAPQLTSASSGVPTPHAEPRDTAQVQTPEAVSGFSRAPVSLSSPRAPSGESASSPSLQDSFVLLSPLPGTLAPAVGHREEAVSASSPVAATAHSADGSVGPSAFSQALAPEGLSRHSSEETHRQRFVARLAEGENWTQFVLDDAALCSKCFSSAVDQLEKQLEDERALLRQYTRALERLKKLRRAEAARRCRQERRERVDKGQALSQLDSRGGRKGEETNTADAPSTRGNDCGSSCVQEDERTALDTTEQSPLRGDQGDHEQVGRNDTPQEGRLRLAEDLRAAQQEYEEAEDEEERLLEELLSLNLLQRELWHLSSARQGQIARHEEATAAMLRQREYVTGQLERLKRLNVMNDAFHIWTDSALPSINSCRIGRVSSPATPSWAEINSGWGHMCLLLDVLFRKVYVHPTHYRLVPRGPFSCLIRRKDDIVLPLQGGGKETGLSRFFYRNRHFDEATVAFLECVQELHEALVHFARQPWPPYASPGVQTPWEPPDLPFAIEGDRVGGLSIRLHLSQDERWTKAVKYLLIDLKWLLSYVEKVCVVCPPAS

>1654

MDTDGKENKQSNSSEENNENELPQVNALFVCPICGGLTLILSDSWTQSMNGAPDLSPTPKTRRPAELISYSKSFSYKKNDMNWTHYKKFQEFNEFFDSTKLSVFPYCNSCSSNILQSIRKKSGFLNYGESLFLRLDITDKFIFANTLKEEIEKIINEKNAFLEAKTEYEKETNRLKTQGGKRRNSSYLTKGYVEGEINSSALVPKTSGPPRAFSSLTSCMIFHISYNRFYGTINTLRLGQNISREVPYEEINNGFTMLFHLISSMCRIGNIDCSNLTNGIPLTIDGTVLNAADTTYRKGVVVFNQAITKLFAFCSSLFMNSIVGTHSLTPPFIINTEENTISQESFLFDAKNPDGWTRAMKLLLFNFKFIQMKMLKGTRIIPSV

>1655

MTEEQELSHIGALFICKCCGKLGIIPDFLKKENQSENLTISTSPSKLRIKLIQKSVSIDTSINLQDLDQYQNFLQLVQETDCEETHSLPLCPHCLHRKLVQINSMIHQIKLQTQVLREFTKEEFDSLKETAQKQIQIYKTNTIAMKKYIDLPNKQMISLPIKKSDLELDKNVLKPYEDIKIDSKYSLFNHLFDIDLKEQMGHINGLRLSIFHQEFIPLSELNSALMVICFLIQNFSRILNRNQVNIQFGATTYVELPLKPNSPPEIIPFSLQGYDNKSIRLFNVAIHVLMVMTYGLYYKDEAFDPYFHPPCEINLQKHTIDGFNYDVNPHSFSDWNLPMKLLLHALKFIQSQLILYNQLNL

>1656

MSETLFFVCPSCHSLSAMDTTLTDDGIVNKLGSVINLSDQSQSMLMKELYELNKQSGNEEPRRHLICVKCIDKFLAYFKNTNELFQKFDVQGKSLDNIHFLIPDQEPQEEPKKPIRKISDSENQIIPQTSPKKQANPSQPDPSRCFTIHTVFSISFDGQYGTINSCRVGSLKSIPVPLAEIQTGLLLICKYLKILLQKSMIDSSDLIIDSKIILIVNGKQHELLYPEKKANVPEFNIALSRMMFLFEQLFSVLSKRAISPPHRIDSSKETINLVSYALNLNDPFNFVLSMKRLLVNLKSVQMLETFI

>1657

MNIPENSIITQCTKCGALVVITADTYKDMEKYINKPIPNQFKSKLKTMENLPFYEQFMNSMKRSRQYRLSLFPLCMKCIDSLSKFEEKYDKIISETISAVNGSTHQIYQCRAYMNDVQNADMPEIPTRIIYQNDIDERSGSFEKKPVPRTEQPKMSPYFALLFFAYKISVSDFFGVINSMRVGTLEQYPVPVEEIQNGLYCIIRILMNLFSSLRLPTSYITLSKNIVFHIQNQNLEFNYPTSRKEYKPFNVCLSKMMEMFELAFDFLSKKGTIGPNLIVSAEKKIGDIDYTLHSNDTVGFTLAMRALLIDIKTVQVYQYKSHFR

>1658

MEESPQKHLPNALFVCSRCRKLCIATELNWNAAFRNHRRQTFSTKTPRRIQLHCGKDFKWEYYDQFLEDKEIFQKREQADFPICNKCANLKLEQINCQRDYLKYESDPLSKNMPNVSLDIIKQLKHKTEKLKKEIQVYREVFESSSAGSDVRIETLSPSIPRDESFRSKAPDTLMLRQNPNENLKVNTFVACTTFVISSNGHYGTINELRLGTQTPTPVPLEEINSGLMLLCQILNYYLSITKAYIFEIHLGPEMTFKIHDHEYTLTAFDLKHKKSVKRFNEAMDIIMSVFSHVFVFFDSGQPRPPFLIKPKDKTIGGESYEYELKHPEKWTLAMKYLLANLKSAQVQGLRCSLIQYQKSQNQK

>1659

MADVGESPFERDPYILFVCRKCGALCLTFPEYLFNEEEDFSIVHPFESPDNNIPDILTYSSSASHQQSSTSLEYFKKLQDKDFSFPVCNICSMEIIQKISSQIKFLNTAESFYSRLDIKDKSIFQETLEQEIEELNQELVSVQNNLNSSSDSPNQENNVNLDDTKNNHDLPIDKRKRRPSKLPRSGLFKSIMLSVTFHIHFYRHYVTINSMKIGQNEHHTIKDEEINIGLLLLGHLIKCLADIVQIDSSPIVIGANICMKLANGKVIPLNATDMKKRKTVLQFNLALDVLFSVAARIFESPMIKSDSMMPPYLISVSDHTISNESYLFNDRNPNAWIHPMKLLLTNFKFMLAYGIRNSVLSFR

>1660

MEIFTTCKQCGKLLFIPKSIWDVISSNPNSDKNSNAIISQPPLKRNCDVTILVDPQQTESYLNFVEETKTMTFNKNNVYFPLCPDCSTTYIQHLKNYRKLFEKTSELIDKKFTNIPKDVFNIQYQNAISPSETRGLLKPQQNTTSSPKKKLKRRQLKQIKQNLHSSPKDKICPDPLDFTNPRHRILPLLMDISSKPKTFCPMESCYVFKISANRHYGTINDLRIGFFKYYRNTILENNIGLSFILHLIYHLKRAFNSDVIRIRLHPHPAISLQDGPFYKLIVPENKKHQKLDEINNAIHSLFVAFNIINDASMVIPNIKYSMPPYEINTEIRTVGDISYDFNWKTIEEWSVAMRLFLVDLKMIQFRSLRTAFLH

>1661

MSEKGVVVFVCSMCGEIRVMDSAVWQSLEQQKTQPLPPREPSPEILEPFDDIENWEHIAEFKNLLQLTKNPNLSSFPLCDHCAEISCEHTNRLNEMMAQFFDNADAIERYGQEFSQRILNNVQVLSPFAITQNKKDLSTESENVSNENPNSNPNPKPPSGQPSTKANNSAFDDQTPIQRRITSRFAQLSAFKLTIDGMFAKINGLRLGKLKSIPVTQNEVQNALLFLCQFLKYQMRIVDVDSSNVNVSHIITFTTSKGSQEMKFPERSREVSPFNAALDEMMQSFDRVFNSKALNLMRPSNLIDTKKHVIASETYYYSESDPSKFTRAMRKLIVNLKTIQSFQTLFSI

>1662

MSVNINTKKMNKSFIRKIPNAIFICKSCQLPCIAPLQSWSETLPSGMATKYIPSTKAQNILVSSKDPNNWAFFKSYTELLEEIKNYHFNKIPLCHLCTNLTLQIYKGQISFCNSMSSYIIKHVDSTNSQIIPGLIDDVKFLYEENKQFLAAQEFQPTCIQYSSKLQRDVPPKMEKKEIQGFNRNANITIGFSSCVLCSTFRISHNRHYGTINDQRLGSHSPDSVPHDEIDCALTFLGQIIVCIGNMINVDVNDIKVTEFLELRQDDGKFIPIHFPDFKKHKKMTKFNKHIEHLFTICARIFEAPVIADASFKPPYVISREKKTISHLSYHLDKKDPWRFTNAMKWLLFDFKAVQFIALQYCVFHK

>1663

MQFHKIHSSSTSKLASMKPQIIAKCTRCGKLIVFQNDVFMKSKVFSNETLPQIIPYSIKKNVNIYLHCGQVVTQYTKRFNLVRNLLTFNNNKYFPLCQDCTTALNSQIRMYTTLVRNATDEYANKYGKIPVRKFKNTFNMTMAKAKDNKAISIQNNNDNQKKNRTDDETKPITALSTLSTSLYNDDEILEAHVKKMPLPSDFCLDPISCQNKRHQILPILMNIQNRTKESVFCPLLSCYAFKIGFDNHYGTINDVRIGFFKYSPNSVIENNAAFFFICHLIYHFRNAFNIPQIRVKLYPRPAIAIGDQSKFLNVEFPEKKKKSAINEFNQALTMIFQAFSLINEITSGFKQYSMPPFLVDNESKTIGDIPYLYRSRNVEEWSIAMRFLLVDLKMIQYRSIRSSFHH

>1664

MRNRTSMRAIPHAIFICKSCQKSTYALFKQWNVDVTESKPKIITKTIPIHYKHYKVRIYIKDSQWTLSENYKIALESFHNLYFDYFPLCSDCMKEYIYLFTDITQFYDSFSSYIIKHVYNKNNIVPSKFLDEVYLSHHRLNDFKKAVSSIKVIKPLLGKEPIRKIKYYTQPKSPTKNIQMKKNLKQKRKTKDNSSIISSIILVKSFQISFNKHYGTINEMRIGNLTPDEVPIEEIERGLYFLGHLIVTIGAFINIDVSEIRLSEFLEFKVDNSKKYDPIFTSDIKSMKLLDSFNEKITQLFAVSNKLFSSQQICDNNIKLPYIIDISSKTINGNFYSYDRREPWQFTLSMKLLLYDFKTIQFASYQMAIDTL

>1665

MGDGSHPGGSVCALFVCCVCRRSVPLAGQSTVCTMNIPGSAKRIYKHKNPEILSRAQTNTDEDVKSAKECCAAFPVISQRNERHQQGKVRLHPAVKGSRTTATGKYGALSPSPFTPWSVDGNSREASQYTESDCGGASEASTTYGNVKKVAIGRAQITATLPETFAHQYAWFPKEKNDAYISDVWSSVQEGSTVPSSGALNPSTALASAPCSNILQDRLPIPLASLILSTPFPSLNISTPHIAGIVEAESPGRSGGGAADGEACNRSNAANLEECDDSNEYYSMAGSHLSFTFAQMGGGLQSNESHALRTPSPRPQRSGNAPTNVNAELNDVSCSRIGLARTLTLERTVSCGTISPPPMTDVYKALLTQFSERLMRQVVHGSTTDLPLCISCWISALAGQKDATTIAMKDLAKLMEVAEIPDELEVQPSGYHVDKSNCKEQQDRQSGVFDLQQQESLERLSIEVDEAESHLAHISWAHLEVADEMLPDLKAQREETSRKIIEVSIRTTAYQTKAFYEVAETAAALEKSIQNMSQSYTLARSTRATSLAFPINTKGSIGTIARLRLGKVAPNTPKQTQVVATPATNKEAVPAISEDKEDSNDRSDVSCCGAGEFTALTTMQQQVCLHFTRGLLDKRQGFVSTTEINNACGYLLLLLQHLIEWHSINTSSVILHPNGERSTIELKKMRSAPPDAPNITVDFFIKDNFFSRKTFDKACFGVASCVREITVWMGRRLQQLRECVERSERGDRSETAANADTRAEVVKHGWVTPSSPPFAIKGEKVGGLSVRYGEVSDGEWTAAMRNLLDVVHWCVVVSCEVNDLQQMLFERDESSSS

>1666

MNNVRSEGGNPMRSSITTPGDEAYAPVCTFFACCVCRRTVSLDGQKMTSQKNLPVPSANRLSLSGLSSPSSTGGFLMSSQTTRYLDDEAKERRRRLRDDQMSVLRKLEEEFQQLEEQEPKTSSQGKADLEDDWEERETLRRQKLHPAIKGSLLTAAGKYAPLSPSPLSPWLKERYAVLRKNMSLVRSGEPQTVTSNKKRTTIAQDRMVTRLAPFFIYQYEWFAPEEPVVYMQELWRMFHNGPVPPPQPSSKLSAQQTEFRSEQPLPRATATPDASAMQMSATHRRASTQTASELPVPHADVLVSPLCPFTCVEPSSVASGEETFAFSPQLSVAAGHVLPPRISQITAQATANLPHSARILNMHKIKSMVPHAGGKYASMIPTAAQKAGTTSVASSLTTASSPGQPCVPTFSRETFLFEFSRHLMRLVVHKSRTNVPLCVRCWKDSLEAQQKETTRIMEDIIALSAVSASPDAKSFVLCFCPRRKRDVASDEPHFGITGGVGITDRNEKTSDRRQLSDSDNRYTNADTTENDVISQESLYRLQIEVWEVEANSRLMAKACQALHDELTVLSKQRTAVKLEWDEAIARYNARKVASFFDIEDAASGVSLRMQLAACSCTWMSKLHAMSLAFPIDTSGTVATIAGFRLGKCPTTAPSRRRSFAAQQRRAASAPPSKNIFAAEMSRSLESTMLMNQLHMQKEYARSLLGSRNDTVSIAEINQACGYLLRLLETIIGHHRISLSTFVLRPNGEQSTVEFVPARGSSMQITRRSSDFFISEKFFSWRTFGAACVIVASCVREIATWMKERLISLREAVYRRFIVEQGTMHSSWVSSATAVLESIESVDAPYDIVHDKVDGFPVRHGDVSENLWTLGMKKLLEVVQWCVMVSTNVDELQRLLEET

>1667

MNNVRSEGGNPMRTSITTPGDEAYAPVCTFFACCVCRRTVSLDGQKMTSQKNLPVPSAKRLSLSGSSSPSSIGGFLMSSRTTRHLDDEAKERRRRLRDDQMSVLRKIEEEFQQLEEQKQKTFSQEKADLEDDWEERETLRRQKLHPAIKGSLLTAAGKYAPLSPSPLSPWLNERYAVLRKNMSLVRSGEPQTVTSNTKRTTIAQDRMVTRLAPFFVYQYEWFAPEEPAVYMQELWRMFHNGPVPPPQPSSKLSAQQTEFRSEQPLPRATATPDAAAMQMSATHRRASTQTASELPVPPADVLVSPLCPFTCVEPSSVASGEETFALSPHMSVAAGNVLPPRISQITAQATANSPHSARILNMQKINSMVPHAGGKYATMIPTAAQKAGTTSVDSSLTTASSPGQPCIPNFSREAFLFEFSRHLMRLVVHKSRTNVPLCVRCWKDSLEAQQKETTRIMEDIIALSAVSASPDAKSFILCFCSRRKRDVASDEAQVSITGRVDITDRNEKTSDRRQLSDSDNRHTNADTTENDVISQESLYRLQIEVWEVEANSRLMAKACQALHDELTVLSKQRTAVKLEWDEAVAKYNARKVASFFDIEDAASGVSLRMQLAACSCTWMSKLHAMSLAFPIDTSGTVATIAGFRLGKCPTAAPSRRSSFAAQQRRAASAPPSKTIFAAEMSRSLESTMLMNQLHMQKEYARSLLGSRNDSVSIAEINQACGYLLRLLETIIGHHRISLPTFVLRPNGEQSTVEFVPARGSSMQITRRSSDFFISEKFFSWRTFGAACVIVASCVREIATWMKERLISLREAVYRRLIVEQGAMQSAWVSSATAVLESIESVDAPYDIVHDKVDGFPVRHGDVSENLWTLGMKKLLEVVQWCVLVSTNVDELQRVLEET

>1668

MSWSCQRCRQPLLLHPSVSSEIDLNQSAYDLVQDSFIAPRHASSPSTSKALLPSKESSSEAAPTTASSSSSAGLSTNDPNSLSARLAASSALFDLLSHPPRTKTGRTSPSASRSGKKALRLRTPNNSSTVIDHPLCKACTDTLLEIMDTQMSEVRSQRDSYLAFEAELRKYKLLPQIQRRVSSSSSPSVATEKDMTHDLTAIRQQQLEECKSLQHEIAQLLSDESFALSELKEAEAARLSIEAQLSTIAEQEAALQQEEERFWSQYSQHSLTLSRLEEDKASLAMAVAHDRELLLRLQSTNVYTDAFCIGHSGGIATINGLRLGRLPGQSVEWNEINAAWGQTALLLDVVARKLGVAFRGYRLIPKGSFSVVYRYEDARSQHYSSASSTSTLFAASNDATETEGEETAGEKTVYELYGSSDWQIGRLLQSRRFDHAQTGFLACLKQVVEFAASADPTFQAPHAINKDKIGEASIRLQFGSDETWTRALRHVLVNCNRVLMWVSEREKRQVQQADGRRAMSNGQEADTRLGQDITA

>1669

MIAPSSQPSGASSSSSSRQQLNVINYCSKCNVPLAIVDDIPSIDTVDLHKSFATMLESFVVLAGAASTLEASKMGAGGGNGGYHDAISRVERILDLASGQADATHPLCVDCVDQVLEEAQKQAQEAEEEKKAYQAALQALENEQNQHKQHGSSAGRQLEDEIHRLEEEERHLLREIESVDHQLAALHREESTLQAEMKQLHEDEHSFWTSFSDYQSQVVCHEEEVAATNAAIHYATRQLQRLKTMNVINDTFHIWQDGPFGTINGFRLGKLLHTPVSWDEINAAWGQVCLLLDVVTKKCRLQLQKYRLLPRGSYSAIIRKHDKATLELYGSEGGLSRFFSGRRFDQAMVAFLDCVQEVANMIHRRGDPTARIPFKIDGDKIEGFPIRLQFNQEERWTKALKFLLIDLKYIIALEAASR

>1670

LASLSAAAADLDSLEERYWNDVNDLELLLRAHTEERGALLAKIDRAGQRLQLLKNTSVLYDAFKIWHDGPFGTISGFRLGRTPEVPVEWDEINAAWGQAVLLLHTMAQVMLMMIQHRLLPMGSHPRVADKRATYDLFGPVSKLWSANYDRAMVAYLACLREFGEHAKKKDLQGGKPTQFNFPFPIDGDKVNNHTIKLTLNKDVRWTKALKFMLANLKVRHHVVPRTVRQRRWRRSRGAACNLGHPQQLGRQSSFTG

>3507

MADKLKKGGNGDGDGAWSDAVLNKLLSELKNKNPEIRAKAAKKLKNHPSVLSNLLYLSPVATQAREMSSEHFTRFMNDLTNKHIFELVNSQVVHEKIGGIMAIDKLIDIEHDENAKTTRLANYLRLGLQSNDPTVMTMAAKALGRLAQASGTLTAVFVVEFEVKRALEWLQDARHESRRYAAVLVLKELAENAPTLFYVHVASSALHDNNVSIREGAVDALHAALALISERENRLRLQWYHKIYEEAQKGLRQNTVPTIHGSLITLGELLRNTGEFMNSRFKEVCDTILKHKDHKEKIIKRTVISLLPRLAAFASEEFVLNYLNVCMQTLLAVLKKDGKNEERASAFIALGEIAHSVGTNIKPYLEQLIGILKSALNVKNKSYCLQSLTCVSMISPVVGVAMQKDMHEILDLMFSGGLNPTLTDALTELAIHIPSMLPMIQEKLMDQLSIVLAGRPFTHPGNRTTKLRKSISVQPSITVGSQPIYADKPAAIQLALKTLGSFNLQEKLLTEFVREVVVGFLDDDDPSIRKAAALTCSSLLVRSNTHAPTRGQLAVVIGEVLEKLLIVGIADPDSSIRKTVLSCLDSRFDHHLAQAENLRSLFIALNDEVFEIRELAITTIGRLTIRNPAYVMPSLRKTLIQLLTELEFSGDSRNKEESARLLGHLIRSSQRLIKPYVEPILNALLPKLKDTNPRVASCVLATLGELATVGGEDMTPHIPQLLPLIIDTLQDQSSVVKREVALRTLGQLAESSGYVIEPFVKYPKLLEILINEIKTEQGASIRREVVKVLGILGALDPYKHKTIQIEVRKEKSEETSTGPANQLPTDSLLGMGSEEYYLTHHTTVIQAVMLIFKSLGLKCIPFLPQIMPPFLQCMRTKETGFLKFLFKQLGLLVAIVKQHIRDYLDEIFSLIKEYWETSLLDQIITLVEVLSQALNDEFKVYLPELIPQMLNVLHTDRTKERDATQNILHALELVEQADVIDSQVRILATQTLGRLGNKLNFSDYASRIIHPLARILDGDDLEIKHVDHGDALGSDYAIFIPMVNKVLVKHRIQHSPTRLSLLKNQPLVLEGAEAIEAQRKHAESNASSEMFAMGTGSKLKMNEPKLRKAWEASNRSTKDDWHDWMRRFSVELLQQSPSPALRSCLSMAQVYPSLARELFNAGFVSCWTELHEQYQDQCVYSLEMALNAPSIPPEILQKLLNLAEFMEHDDKPLPIDIKTLGALADKCAAFAKALHYKEIEFQTSPATTCEALISINNQLDLPEAAVGILLYAQQNYDIELKESWYEKLQRWDQAKEVYERKQKARPDDIELTMGRMRCHHALAEWEELTKLAEGIWPKADKSTRQRMAPLAAGAAWNLRLWKNIEDYIGVMPDNTVPTIILIIIIIIIIITFIIIAFIIIAFIIIAFKKTHQPDGTFFKAILNLHKENYEEAHKYINRTRELLDTELRALVGESYNRAYKVVVKVQQLSEMEEVINYKQSVDAEDRKAMIRTIWTKRLKGCQRNVEVWQDMLAVRSMVIPPIEDMDNWLKFSSLCRKSGHMRLSYKTLVNLLGFDPAPTSLDKFASHPYPGVTFHYLKHMYDAGDKKDAFERLRVFTRNLQDDAKLLARCHLTLGQWESELHDNLSESTIPHILASFKAATEYDDHWYKAWHSWALSNFEVITHYQKLAQPDKITPHLVPAVAGFFRSIALAPQGKSLQDTLRLLTLWFKYADKKKVEEALIEGFNTVSIDTWLQVVPQLIARIHSPVPSVACMVHDLLTNVGREHPQALVYPLSVASKSQASARVSAANSILDKMRKHYNSLVEAALLVSKELIRVAILWHEMWHEGLNEASRLYFSNRDVEGMFKKLQPLHEMLDKGPETLREVSFQQAYGRDLQEALEWCKKYSRSNKVSDLNQAWELYYHVFRRLDKQLLQMTTFELQYVSPNLLEAHDLELAVPGTYEAHTDEVVKIASFAPSLSVIMSKQRPRKLSMFGSDGKEYTFLLKGHEDLRQDERVMQLFGLINTLLANDRETANRHLSIQRYAIIPLSPNSGLIGWVPNHDTLHALIKDYRESRKILINLELKLMVQMANASTCDALSLIQKVEVFEHALENTQGSDLDKVLWLKSPNSEVWLDRRTNYTRSLAVMSMVGYILGLGDRHPSNLMLDRHTGKITHIDFGDCFEPKMRQRSSSEQDDGDLLESPSRGETPEIFSPPSGGSPHTGTYRSKSILSRAVANAELASGTENGLNERALKVIDRVSAKLRGKDFSKEQSLDVPQQVQRLIDQATSHENLCQCWTGWCPFW

>3508

MADKSKKGGGGAGGEGDQPWIDLVLNKLLFELKNKNPDVRTKAAQKLKTYVMAQSREMSSELFARFMNDLTNNHIAELAHSQLVHERIGGIMAVAELINIEHDDNAHRMRLADYLQEGLQTNDPGAMKMAANALGRLAQASGTLTAELVQQEVKRALDWLQGKHESRRYAAVLVLKELAVNSPTRFYVHVSSFFDLVWSALHDNNVSIREGAVDALHAALALISERENRLRTQWYHKIYEEAQKGLRQNTGPTIHGSLITLGELLLNTGNFMSMNSRFKDVCDTILKYRDHKERIIRRTVINLLPRLAAFAPEEFAAKYLDVCMQALLGVLKKDGKSEERASAFLVLGQIAHSVGPGIEPYRPQLTALIKNALDVRNRGYCLQSLTCWSMVSSALGAAVQQDMPEILDLMFKSGLSPELTEALTELAAHSPSTLPNIQEKLMNQISLVLAGKTFVYPGNRTNIFSHMQQDTQSNFIQPTLIQLLTELEFSGDSRNKEESARLLGHLISSSQRLIKPYVEPILNALLPKLQDTNPRVASCVLATLGELATVGGADMTPHIPQLLPLIIETLQDQSSAVKREVALRTLGQLAESAGYVIEPFIRYPKLLEILINEIKTEQGASIRREVVKVLGTLGALDPYKHKTIQIEVRQEKNQVAGAGDGVGGSAHQPPHEVSGMGSEEYYLTVSVGALMKILRDPSLAQRHSTVIQAVMLMFESLGLKCVPFLPQIMPPFLACMRTKEKKRLEPLFKQLCLLVSIAKQHIKDYLDEIFSLIKEYWNTGLLDQIITLVEALSQALNDEFKDLMFKSGLSPELTEALTELAAHSPSTLPNIQEKLMNQISLVLAGKTFVYPGNRTNIFSHMQQDTQSNFIQPTYMERNAAIQLALETLGTFNLQEKLLVEFVREVVVGFLDDDSPAIRKAAALTCSSLLVRANRNAPVRGQLSVVIGEVLEKLLIVGIADPNPAIRETVLSRLDSRFDYHLAQAENLRSLFIALNDEVFEIRELAITTIGRLTIRNPAYVMPSLRKTLIQLLTELEFSGDSRNKEESARLLGHLISSSQRLIKPYVEPILNALLPKLQDTNPRVASCVLATLGELATVGGADMTPHIPQLLPLIIETLQDQSSAVKREVALRTLGQLAESAGYVIEPFIRYPKLLEILINEIKTEQGASIRREVVKVLGTLGALDPYKHKTIQIEVRQEKNQVAGAGDGVGGSAHQPPHEVSGMGSEEYYLTVSVGALMKILRDPSLAQRHSTVIQAVMLMFESLGLKCVPFLPQIMPPFLACMRTKEKKRLEPLFKQLCLLVSIAKQHIKDYLDEIFSLIKEYWNTGLLDQIITLDMLAIHSMVIPPIEDVDNWLRFSSLCRKSGRLRLSYKTLVNLLGSDPAHHPLDLAASHPGVTFNYLKHMYAAGDKKEAFERLRVFTEGLPGDDAALQARCYLTMGQWESELNDNLSETNILALFKAAIEYDDTWYKAWHSWALSNFEVISHYQKLNMPEKISPHLVPAVAAFFRSIALAPQGKSLQDTLRLLTLWFKYAAEKQVEDALVEGFTTVSIDTWLQVVPQLIARIHSPVPSVARMVHDLLTNVGKEHPQALVYPLSVASKSHASARMSAANSVLDKMRKHYNSMVEAAQLVSVELIRVAILWHEMWHEGLEDASRLYFSNRDVEGMLKKLETLHEILDKGPETLREVAFQQAYGRQLQEALDWCKKYTRSNRVADLTQAWDLYYQVFKRINNQLKPGQMKTLELQYVSPNLLAARDLELAVPGTYEARTDEVVNIASFAPILSVFSSKQRPRKLSMLGSNGKEYTFLLKGHEDLRQDERVMQLFGLINTLLANDRETANRHLSIQRYAIIPLSPNSGLIGWVPNHDTLHDLIKGYRGPRKIDLNHEHKLMMQVTSKFDELSLIQKVEVFEHALENTKGSDLDRVLWLKSPNSEVWLERRTNYTRSLAVMSMVGYILGLGDRHPSNLMLDQHTGKITHIDFGDCFEVAMHRDKYPEKIPFRLTRMLINAMEVSGIEGNFRFTCERVMRVLRDNRESVMAVLEAFVYDPLINWRLLQPTSPDNANETSSAKMVRETTNTGEQDDAGGMETPEIFSPSGDDDGSPHTGTFRSKSVLSRVANAELAAGTENGLNERALMVIDRVSAKLRGKDFSDKQSLDVPRQVRRLIDQATSHENLCQCYTGWCPFW

>3509

MTLVEALALSLEADFKVHLPVLLPHILGIFESDTSDQRRAAFKVLDVLCIVGSGLEEYMFLVLRPLMRLIEHTLVDVSIRKYAVHALAHIIQHVNLAHHASRIIHPLVRCLAVADLRPHVMDALVALVMQMRTEFMIFVPIVQTAYVKHKLAHPAYERCLAKMLRNEPMPEFVVPLPSGAVPATTAATAMALDPIKKLPVNQQQLKKVWEASQQSTKDDWLEWMWRLSIELLKESPSPALRACASLASVQTPLARSLFNASFVSCWSELHDQYQDGLVQALETALTSPNIPPELLQALLNLAEFMEHDDKPLPIDLRTLGKYATKCQAYSKALDSSGRFGLSHKTLTNLLKFEPDESNLLNGPPPVVDAYLKHMWETGRGLDALKLLSAFTGRLAELVASNPAALAAIAAPLVPVAPSAAGGPGAGSGLLAVLGSIGAGSGAAAATSAVAAADVIQPSNSSWYMGPDPVSQARLLARCYFWLAEWQTQQLDHWPDDPTVRHEILQAYRLATHYDKAWYRAWHAWALANLQVVNHHERALAAAPPDEYAALVSHHVVPALQGFVHSISLSRGGDSLQDTLRLLTLWFKHGHLNEVHDSISKGFSHVTIDTWLQVIPQLIAWVHTPSPNVRRLIHQLLCDVGKEHPQALIYSLMVASKSSNKPQKTRAALSILDKMRMPRADLVDQGLLVSKELIRVAILWHEMWHEGLEEASRFYFGEGNADAMIAVLEPLHQMMHKGPETLREISFHQAFGRDLAEAHEWVQKFLVTREKDDINQAWKLYSQVFKKIAKQLTQLPTLKLLNISPALLAAKDLQLAESRKILLNIEHRLMVQMVPDYENLTLIQKMEVFECALENTTGQDLAKVLWLKSKNSEAWLDRRTNYARSLATMSMVGYILGLGDRHPSNLMLDRYTGKVVHIDFGDCFEVAMLREKFPEKIPFRLTRMLVNAMEVSGIEGSFRITCEHVMRVLRENKDSVMAVLEAFVYDPLINWRLMTNVPSPRADKKRKEAELEAGAGGRRGQAAPTAALQPPPAGDEDDDDLVDLDDDDDDTQYKPEALNARAVQVVHRVSNKLTGRDFKPNQVLDVPQQVDKLIQQATSLENLCLCYIGWCAFW

>3510

MASYEALDHIIARLWSRNDELKAIAAEDLKQHVLAVSRELASDVLAKFLTDVNQRIFELILGSEVHEKVGGIMAIEKLIEVDGEENATKITRYGNYLAMALNNGDPQVMVLAARALGKLVSPRIGGAFTAEFVECEVKKAIDTLHLGEKHDGKKHGAVLVLKELAQNAPTLIYAHVPTILESLWNPLRESKLVVREAAAEAMSVCLELVYQRETPSRTMPWFSKVADEAMKSLKAPQVEHVHGALLVVRELLLHTGRFMEPKYREICELLLKLKDHKDALIRRTVISVIPTAASFNPPAFELYLSRFMSHLITQLKRERDRSAALIAVGRIALAMESSMKDFLEDIIKVIRESLLMKAKSRNEAPAYQCVSMLATAVGQALTKYLQDVLDLFFANGISQPLVQALGDISTNIPPLLPKIQERLLNLVSLILSNQPFRPPGSPARTSVSAVTSMGGTSSEARQTESIVLALQTVGTFEFGQSLNEFVSQIVTQYLEDDVADVRLAAALTCCRVLSSDPVRFQTSAHALEQVNSVLERLLTVAVADPDPKIRLAVLSSLGDQFDHYLAKAENVHSVFLATNDEVFANREVAMQIVGRLALCNPAHVLPFLRRMLIQLLTEFEYANVSRTREECARLLTHLVGSCRYLVKPYVDPILTVLLPKVKDPSPGVASATLGAIGALSLVSGKNMLPHMTTLLPHVIESLADATSQAKRVASLQALGQITSSTGYVIQPYLDHPTLLDTIISLLKTESSPPVRQETMKLLGTLGALDPYKHKQINRQVTDAVQGDGNTIDPATMLTMGMGPSHEDYYPSVAIAALMKMCRDFALATHHTAAIKAVMYICKTLGLKCVPFLPQIMPPFLGMLKTSQPPMLEFFFQELAQLVQVVGAHMTPYLPDMIALIEQHARSLELLVMTLVEALALSLEADFKVHLPVLLPHILGIFESDTSDQRRATFKVLDVLCIVGSGLEEYMFLVLRPLMRLIEHSLVDVSIRKYAVHALAYIIQHVNLAHHSSRIIHPLVRCLAVPDLRPHVMDALVALVMQMRAEFMIFVPIVQKAYVKHKLAHPAYERCLAKMLRNEPLPEFVVPLPSGAVPSTAAATAMALDPIKKLPVNQQQLKKVWEASQQSTKDDWLEWMRRLSIELLKESPSPALRACASLASVQTPLARSLFNASFVSCWSELYDQYQDGLVQALETALTSPNIPPELLQALLNLAEFMEHDDKPLPIDLRTLGKYATKCQAYAKALHYKELEFVSEPTTPAIESLISINTNLQQPDAAIGILTFAQHHHQVRLRENWYLKLERWEDGLLAYEHKAVEDPHNFAITYGMMKCLHKLGEWDRLSKLARDKWPNAQPDERKMMAPFAAAAEWGLGHWEAMDEYIHVLKPDSPDGSFFRAILAVHRNLFPQAHVYIEKTRALLDTEFMALVGESYSRSYDVVVRVQMLAELEEIIQYKRNHDYPQAQEMIRVTWKNRLMGTQRNVEIWDRMLKVRSVVLAPQEDMEMWIRFSSLCRKSGRFGLSHKTLTNLLKFEPDENNLLNGPPPVVYAYLKHMWETGRGLDALKLLSAFTGRLAEVVASNPAALAAIAAPLVPVASSAAARLLARCYFRLAEWQAQQLDHWPDDPTVRHEILQAYRLATHYDKAWYRAWHAWALANLQVVNHHERALAAAPPDEYAALVSHHVVPALQGFVHSISLSRGGDSLQDTLRLLTLWFKHGHLNEVHETISEGFNHVTIDTWLQVIPQLIARIHAPSPNVRRLIHQLLCDVGKEHPQALIYSLMVASKSSNKPQKTRAALAILDKMRMSRADLVDQGLLVSKELIRVAILWHEMWHEGLEEASRLYFGEGNADAMIAVLEPLHQMMHKGPETLREISFHQAFGRDLAEAHEWSQKFLVTREKDDINQAWELYFQVFKKIAKQLTQLPTLELQYISPALLAAKDLQLAVPGTYRSGAPLVKIRRFAPTLQMFMTKQRPRRMTIFGDDGREYHYLLKGHEDIRQDERVMQLFGLVNQLLEGDAETFRRHLHITRYAVIPLSPNSGLIGWVPHCDTLHALIKEYRESRKILLNIEHRLMLQMAPDYENLTLIQKMEVFEYALENTTGQDLAKVLWLKSKNSEAWLDRRTNYARSLATMSMVGYILGLGDRHPSNLMLDRYTGKVVHIDFGDCFEVAMLREKFPEKIPFRLTRMLINAMEVSGIEGSFRITCEHVMRVLRENKDSVMAVLEAFVYDPLINWRLMTNVPSPRTDKKRKEAELEAGAGGRRGQAAPTAAPQPPPAADEDDDDLVDLDDDDDTQYKPEALNARAVQVVHRVSNKLTGRDFKPNQVLDVPQQVDKLIQQATSLENLCLCYVGWCAFW

>3511

MDAFEREALLTLLRASHESERTLAAYQLRRLVSRAAREMSGETFGRFEDELYSTLFRMVHHGGDVEERLGGVAAIEALVGAPSAEPETKGIKFANFLGSPEGQRQFVTRTAAALGRLARRGPASSSDHVEFEVGRALEWLQRPAGADGHLGGAKAAADAPGGAQRRLAACLVLRELAKHAPTLFYARVRDFFERVWPALMDARSPDVREAAAAALGAALEIVARRPTAQHSHFYCAIYAKAHAALAPHATGHGALLAVGALLRHAGGFMMPRFREACDAAIALREHRSRAIRKAVTDLLPRLAQYCPDAFARAYLKGTTKHLLAMAVHRTSGELRDAAYEAMGRLALAVKHHLVPALPEIVAAPASPEEKRRRGVVVDCVADVFEALGEKIPPPHADALLDALFANGLCDPLIRALGVVAKALPSRRPAVRARLLDALTSVLDFDASPYAPPGWARPLPRPRRRRADSAPRTRLEVDARDEDLILLSLRTLGSFSMEGVCLLPLARDCASRYLDASSAAVRSAASAGRTAKQPGAAPLGAWYFAGPSAVVVDDVLRRLLAVALADDDPGPRRAVVRALRADARFDGHLARSAHVDALALLLHDEDTELQLSALALLGRLAARNPAAVLSRVRAALARALDTLRCPASDAPAKERAARLAAGALRAESPRARKAVWPLAAEVVAALPLGYRDLAVPTRLACAALDALGECVLVLGPRSRVVVYKPRVLAPLFDALLDRSSSRKRELALRVLGRLASSAGCVVAPYLEHAPLLPRMLAAGDRGGAPWSLCREALRALGLLGALDPYKFELDGKKRAKGKDRAARAATPAAGEDYYARVAIDALVRVLRDGSLAAHHAAVTQALMFIFQSLGLRCVPFLGNIVPHLLDVARTCEPGLRESVLLQLAALAAIVRHHLRDWLPRIFELVVDYWAEHLEQVVPLLEEIATSVSEHLAALPPLLTKAKALTAPAALSETRLALILRATMLLRGALSDYLFLVARVVRCLRVVVGRRALARRRDLGARIARSLCRLLERSDVVAADAAPAKAAHGEALRDAARDALEVVRDQLGEELFAPFVGLATRCLSLGPRGAASPAALAWAKRDARTRAGDDDDDRAADGDGGEGHAFLSRSQHIGEKYRASSIDHAGPSRQRLHVNQPNLQRAWDVSQRLTADDWNDWLRRLSLELLRESPSAALRSCATVAHAYPRLSRQLFQSAFVSCWLELDDAYRDSLVRTLETAFRSDELQAVAPDALQLLLELAEFMERDVDALPIDIRVLADLATKCRAYAKALHYKELEYRTPELARDRHAAAEALIAINRKLAQPEAALGVLHATRKRSERRRRTRHHRLGSYEEALALYRRRLEGDGGDVEAILGAMKCLDALGEWHEACGLLVRSWPRLQVALGDNTRLLMKAANVGARSAWALGRWGEMTNFVAAMEDDDASKPFYRAILALRSRERLEPARIDEAVDLVDDARRLLHGSFAALVAESYKRAYGTMVTVQQLAELEEIVGLRRVELAARGESRRSGDARRGADAVSDHRAALVARWRRRLAGCPHDVAVWQRVLNVRALVLSYEDDPDSWLRYASLCRHSGNAALSESVLTRQLGFDPASVPRASDGLPDHRLRYAYAKHCHAAGKRTDALRRVEELADALGDAPANLAFDRLRARCLLRLGDWRSRGPDGRASPAPGSPAGDDDALCLGPYELATRLDAASYKAWHAWALVNYRAVQRAARSARADRKSTRALVAAAGGFVRAIALGRRRWAASVQQDLLNLLNLWFRFARQPEVEAQLLPDEDDRGGVGGAPLDAWLGVLPQLIARIGAADASPKSALHRLLARLGARHPQALVYPLSLQLKSPRDERRLAAEGIMQGPRTVAPRLVDQALLVSEELIRVAILWHEKWHEGLEEASRLYFGDGDVDAMLQILRPLHAELSAGPTTLREAAFQQAFGRDLGEAAACLDDYERHARAEDRLGRGGDRGDGDARRRLREDADAALNQAWDLYYTVFRRVNKQLPQLTTLELRYVSPALLGARSLDLAVPGTYRVDGAGARISRFSPSVHVITSKQRPRRLAMKGEDGREYGFLLKGHEDLRQDERAMQLFGLANALLAKDRRTREHGHLSIQRYAVTPLSHNCGVVGWVPACDTLHALVRDFRDARKIVLNVEHRVMLQLAPDYDALSLPQKVEVFDAALANTAGHDLSKVLWLKSSHSEQWLERRTHYARSLAAMSMVGHILGLGDRHPSNLMLDRRTGKVLHIDFGDCFEVAMHRDKFPERVPFRLTRMLVNAMEVSGVEGTYRATCERVMRVLRDDPRDNRDSLLSMLEAFIHDPLISWRLLGAAAEPDAASPSPAPGRARRPPPSSPPPPMHVRMQRMAASLTGNSTRHSALAASQRGGRAGSVANSRLDHRSARESLLVKAHGAEGADAPIEALNEKALAVMARVQDKLSGCDFRDHQGPLAVADQVDRLIAQSTDVQNLCQSFVGWCPFW

>3512

MTRVRLGRAPWPNISVHGCQDHSCYVIESICGFLRSIELFDINREARNDTVIIQAMLMTISLWFKYGSWSSAIYALKQGLAKSSVGIWLGVLPQLIAHLDHAALEPRLLLTHLLTRIGHVHPQALIYPLTISGKSPVVSRRDASFFILCNLQACLYASQANKTLVAEAGLVACELHRAAITWHEAWFQGIENAANLQFGECDLRTTLEQLGRLHSLGDAARILAFSHAHGCEVELARRHLHQFLLSRRDLDLHQAWDVYSILHRRLKQRISSVGCEILELCHIAPMLLSVRNLSLAVPGTSLSAGDKSRTLPICGMQQCGIIHITSFSLKANIICSKQRPRRIKIHGSDGESYDFLLKGNEDLRQDERVMQLCGLINCLLAQAYSKCEPALSAQGLMQITRYAVMPLSNNSGLLEWVSNCCTVHSLIAEHRNRFSIQTDAESSCSQIVAPAYSELCSPGKLEAFSYSLARTKGIDLARMLWLNSEAPCKWLSIRQRFTLTLGLNSMVGFIIGLGDRHLSNIMVNHLTGDVVHIDFGDCFDVASQRAKFPEYVPFRLTRQLISVMEVDGVKGIFRLVCNHAIRVMRAECLSLTAVLEALVHDPLIGLSVILPASPRRSQCFEGTLEVPSQEAAIRVVALIQDKLTGRVVRSGDVTAVEDQVLNLIHQARSRSNICQMFFGWCPFW

>3513

MRLADLCYGSGHFLRCGNVLAFLDSVPQIPSVNVILRACHLLWALGRRALAYDVLSRVTESLIFSHQKNSWDLKDKPGDEILIASILTRSEWCGILIRASKFTRRSRYFNQCLSIVIRLLQRVTKLVPLQPRAWHSWALANYEACEQLERRELHKLGNSGPVLASSPGTVSVFHHVIQSIGGFFRSLQLFDAVVLQDTLRLLTIWFKHGTRSQVSAKMLEGFGHTSVDVWLGVIPQLIARVDYPDEDVRHLVADLLVQVGRAHPQALIYPITVSAQSASESRRVASVGILGGLQQFLVAEANLVSRELNRSAITWHEAWYQGIEAAANLYFGSADVQATIHGLIEMHHSSHDTTIVTTRLAAFAHVYSPDLLAAESFLSRFCRLRNHADMHQAWDIYSAIHKWVKQSIFTDKLSHLDLGQVAPSLMCAGSMHVAIPGTEGGRNLPVRILSISPQLRVLASKQRPRCLRISGSDGKEYQFLLKGHEDLRQDERVMQLFGLINALFNKTYNAGHGHKEPLHVQQYVVMPLSNNSGLIGWLKCCSTIHQLIVNHRSHSGILVDIERRFTEAIACDYHKSPFMYKLGDFKYVLNRTYNRDLANVFLVTSGDVSIWLMRRTRYARSLAVTSVVGYVLGLGDRHLSNILVSDKSGEVIHIDFGDCFEVAMKRKRFPEKIPFRLTKLLVNAMEVSGVEGSYRLTCNHTMRVLRNDFHSLMAMLEAFVHDPLVDWKLLSTNNSHFRNVLKQTLVITDRVQQKLTGSTLHDPRFCVSPIHQIDGLIREAQCAENICQMYGGWCPYW

>3514

MESFKQLILANLNDKDIHIREAAGKALSACLLLMQQRENQKRNDFYHKFLEPVLSLPNDNRSESIHGFLIAIDSFLKNSMEFSSMYIDPIYPMIRSYFNSKEVYIKKAVIHLIPVIFRVMKLYAISFLTDEYRDEAFKYLMACTARDTYRGDALKAVGELAMVLPEDAYLRINEIVDVIKENLVSKRSNSIEVLIARRGDNVKDALDCFTHMSCALKEQFTDKISLLCKDLFAAGISRTLIEALREVSSHTENSLNLVQKHLLEELGRVLGGSSHTEHSLPSFNFQSQQKRMLSAPESRLSSSTPPSNVLLALGALEIFDFCDMNLFSFVTAHVMPHVDSPSEEIRCAAVRVACKLLLPKHPRIVNHNQFQQAYNQVQKLISVGLSDREVSVRSCLLNALDKRFDPYLIQSENVSFLLMALHDEKYEIQRAVLKIISRLSPAIPGVVTNAVRQLILDLLRALEYSQETSTIIESVKLLGLSIHSTSSLIEAYVNPVFELLVNHLKTIQHGECLEWKLRIDNIKIAFLETLSLLAEVGSPELAKHNDVLMPIIIEALKQSNELREAGLVLLEKYSFCTGYVIEPYLQYSSLLDLLLNQYNRSGNASYTLNESVMRCLGGLGAIDPFIYKKNSKQTSKSDLQVVSYDDLQEDQRPLLCPCEERDLIITLEAIMSAFQDATYSEYRFTAFDALTTVITKPNSSSYQYLSLVVPPFIRMMQSKQCDSASFRRTVYFLNDLVKTVKEHIRSYLPDILRSMEPFWDTNQFEILIFIRVCSSCFHSEFKLYLVYVMPRMLSILSESAPEKRRAATEIVHCLHCIVPSLDNYLNVVFPGLMRFIENNEHVEASEREGVKCLKRIVNELDVSLYTSQIIMPLLRVLSKDYCCVTDDIMDVICSVMCQKQQEAYIYLSAINKVVSEKNINYPDFQKISNILYRGGVLAPRDYSFDQKDDSVVHPEEQVAPRHHNGDMTALQTLWRQAVNLTTKDEWIEWSRRFTITLIKESPDFSIRSCHALAQTSQIFANELFNAAFNSLWNELPAAYRADLASCFKQAFNSKQPVPPEITMKILSLAEFMEHDEEIVLQMSKDIVLPLDIGILGDLAMNNNAYAKALHYKEMEYETTPDSCIETLIQINNQLHYPDAAVGVLRYSQKYYEDITVMDELYEKLGRWEEALEAYESKQVNRPLETELTMGRIRCLNALGESEMVLRVIQRAEDKLGDCGQAETAAVYGAKAAFDLGDWELLRMFITNASAHSTEISYYQVALYIHDREYEKAEKLISETRNAISRILAPIISEGYDRIYSHVTQLEELKELEEICSLRKNYAPESPDYFPAARHLVTIFNKRLDGVQRDVSVWHGLLSLRKLFVEQKNPRSPEPKRSYELEPLNEDDRSSYDALCSYHHWLKFISLARKSNRPALALRTLESLGIDIKSCPRLGANPNDENDAYAEVRYAYHKFLYDQGLTKEAIRRLRKLVDEDSPKGSKLLYQSVDQEEQMTVRVRMRLRLAQWILDENHRNLSHEVVSDIATIIDECSQFNQDHKAYHEIAMLHMTCAEYYNRLNTEEAHNEVTDHLKGAIGSFFDAISLSKDKNSSLVLQDILRLITLWFTYGNREKVINAINCGFNIISIDTWLYVIPQLVARIHIKESGAKRLLINLLVQLTKAHPQALVYPLTRSTRSETKSRQRAALEVLSHLRRDNSVLVNEADLVSSELIRVAVLWTEKWRRGIEEASTQYYEFRNVHKMLLIFDDLYRTIGVPSETRIEDYFHRMYERQIQSTREYLLRYIETKNVTDIESAWKDLNEIYKQMTRSIKLVTELELDSVSPKLLNIKNLMIAVPGTYSPDKKSEIVRIKQFCRILSVLPSKQRPRKITIFGNDGQKYEFLLKGNEDLRQDELVMQLFTMVNRLLAANPETCQQELFVRTYSVTPFSDSCGMVSWVDRHDTLHALIKMYRQCKRADLGAERNLIAKYAPMYEEMMPVRRVDVFNTLMNETIGDDLKSVLWLRSKSSEKWLLRRTNYTRSLAVMSMVGYILGLGDRHPSNIMINRATGSICHIDFGDCFEVTMVREKFPERVPFRLTRMLVKAMEVGGVEGTFRSTCENVMSVIRQNEDSLMAILEAFVHDPLMNWKLTEKTPENTASEQDGSEMIKLEANTKATTVISRVRDKLTGNDFYQDEKISVQEQVRRLITQATSRYNLCQSYLGWWENVMMGMMCRCPFY

>3515

MSAPKEFWELEKCLTKLRSDDQKQQHLAAMHLKAHAIKEARIRTSQEFNIYMEEICKNIHDFCESYNRNERMGGILLIRNLVDVVYAEHDLHLQKLGRYLQKIIDNGTNIRVSVSDNINEIAAETLGKLVQANPTSADIFEREVKQAFKWLASGNSSSSRNEYNVYMAMLIIRELAKAAPTLFNEYVPQFLESVWVGITDYKAEIRETSKLALRQVLVLIGGKRAVQAYHKLFSTAKQKIVQQQQSKSSGQYHVIHGAILVIGELLSQDKKQQPTYIIKVYGEICQLFFAHKDARLPLLKETIITLMPKLAHFNPILFSKRYLESCVRHIIQEHKLQTVSPQVTYSSLGGVALAVKHNIMPMVPPIITVLDEGLTRTRKGRSSPAISHSTVATEALRCIALLAKAVGPLLGPHATDLVDKMIKSDGLTAPHGAELVRALTALVDHVKDQRLLSDIQKRLLAAIFQILTPSAALPTSILGGANASAALSGGSISSNYLSTNSAASADAVSAAASQYLIPSDIGIMADVLKILRSSSSPSSSFTAADHQHDSKIKDPAVVRLALNTLGTFNFDNIRLLWFVCQCLIRYLDHPKVAVREAAAVACSNLLLRPNDPYLKANLAKTHYRAMIYRVLEQLLTVGVADPMPRIRQTVINALHPRFDAFLAQAENLHALFVALNDENFPIRAAAISVIGRLALKNPAYVMPSLRKSLIHLLTQIQYGVDSVSQEQSCRLLGHLVESSEMLTKPYLPAILKVLLPSLKKMGPTTTAGAATADPRVVSCVVETLGKLEKVGGLELRAYLPDLLPFLLGHLKNRGQAHSIEREVVLKALGKLIRATGNVILPYVQYPELLPTLLRILSTEVKWEARLEALKVLGILGALDPYQHKINEAKLRDAAIEAANRKGQSATTMGPHRALLKMYYYKNKNTTAAGGTRTAKAGGGNDNERAGRVSVNPTRVTDDRYLLPSMVGSLDEYYSTVAMNALMRVLADPTLSSQHNSVMKSATIIPTLLAVLRNTADEQNIALQKQVLERLQELVSIVGKHMRPYLHPIFEVIYTRWTRSRTYDGALAHEVLALVETISLALREEFKFYLPDLIPHFLRALHGSPPPAPSGAAALLVATADDKKKKNIGVGGGGGAAGGVAIATGAASVTMTNSSNLSSSSTNAAATSGGNLFESKHHHHLEQRILETMQTFGSNLEEYLHLVIPTLMKLAESQDVAQSVGETAVQTVGKLCTNLDFRDYISCIVHPTARILAGARDSNHRDKVMAMLLAVIRQVGVDFLLFVPLINKALEVQTRNAQLMRDQKEKARLAACVDSYDLLKDIIMRGETPTTFDLNKALSLFNGQKHLSWDRERKIVREMAAREKPTIFERSTKLTIDVNALSKAWECQNKLSKEDWQQWMTNFMVKLLEFSPSTSLRPCVTLAKRDRSLARDLFNAAFLSVWSELRDRSKDDLIRSLETALRPSTPPEVLQVLLNLAEFMEREGRALPLLQDESKDQPTLGRLAERCHAFAKALHYKEIEFKSAPQDTIESLISINNDLQQPDAAVGILKCAQDMAGMELQPTWYEKLQRWEDALEEYEVLQLRDQASIKHTLGRMRCLDALGLWSRMSNMCDHIWKTRLPPDDNDGDDNTGWHAAAPSTPSSSRVSNLMMMSEKEVLSVAKFGASAAWELRDWEGMIKYVKRMPDGTLDTCIFKCVLAIQREEYESALNLISIARKAVFPELRSLVAESYNRAYGHMVKVQVLAELEEVVAYKKARRDEERVHIQQLWRNRLGGTQRNIMVWRHILAARKLVLNPEEQIDTIVDFSALARKSQRLALSLQALAPFLLESSSASMAAVSAVVAARLGKQLHEEKKKKFNRLLYHHEVHAEYDRSGGYYALLRSDPRSVRLPFAYPKLCLAYLVHLWDAGYSCEALERLEALVEDTRPQYHHHHHGGGSASSTPVFLPPMSPRSPRDFVAASTSASPAAAAAADDSAAQQTTRMECYLTLGEWRRKLGGDSPSNLERVLQAYRLATRICKDSYDAWHAYASVHFRLVSLLDAEAKRIPPAAGGGGGGDSKKGEDADGGGGAAAATTASASSRKIYQHVIAAIEGFFRCIALAGPNRSTLQDVLRLLTLWFSHGGSEHPQVNAALSRGFNTTSIHTWLTVIPQIIARIYTVDPGVRKLVEQLLRKIGKEHPQALIYPLTVASKSQIAARRKAAEGVMNYMRKYSKKLVDQAELISSELIRVAILWHEMWHDALEEASKFWFGKRNIEGMLQVVEPLHQLMAKGPQTMREVAFQQRYGRELIEAKEWLDRYKVSSNESDLNQAWDAYCNVWRRINKNKERLVTVDLHHASPKLKAVRHLELAVPGTYEKVVSAANLIRPHALATAAAAAGTAASRGNKRMQPSSKLRGGGSDNTSSSSTNINTVFQNQYHQHQDSVRAADAAAAAAVGYPIMGEGVAMLMKGNDEQHYYQHLHSKGGAAAAAAAAARVDQDQHLQHFGGGSKEYGNSVDEGMEGKITRIAWFEPRLKVIESKQRPRKLTIIGSDGLEYSFLLKGHEDLRQDERVMQLFGLVNTLLATDDQTTQKNLNIHRYSVIPLAPNSGLIQWLENCDTLHSLIKEYRDARAIMIQVETMLIKAFSNRVYEQLTVIEKIEVFEYALSRTSGLDLYHVLWLKSQDSETWLDRRTNYTRSLAVMCMVGYILGLGDRHPCNLMLDQFSGKIIHIDFGDCFEVAMQREKFPEKIPFRLTRMLINAMEVSGIEGNFRSTCEAVMRVLRGHKESVMAVLEAFVYDPLINWRLLQTKVSRLHENPEQKEEIEEDDNDDDEKGSGLSESSAALLGREEEAAATENNNNREGVGGTGVLNQKALKVIGRIADKLDGKDFGDGEPLNVPQQVEKLIRQATSHANLSQCYIGWCPFW

>3516

MEMGQVGDLLSSGNPLNALSSTFNALASKSQETRSAAAERLSSYMEAKSRQLTADKFEELASNITSKITDLVNGLDIYGKMGGIMAISMISEHFSAEKNIFENIILRIAACFHRVFEQIKEPVVLEMAARALGHLTQHGSGFFSEIVNDEVVVALSWLENPKYAARRLAAVLLLREFAINAPTLFNIHVQKFLTHIWVALTDKLVAVRYRARDALSACMDDIAKRKSRWRQYCHRRIYERAREGLKTPDRTPKVHGSLLALGELLGKAREFMIVSGQFTETSELIMRTMVVKSNTIRQTIISLLPPLATISPRRFARQYLRGSIKFIMDGLKQSSSWTLPSSYLALGELAIAVGPAILPFLDTVISLVKEGLQMRKNNYSEAALSCLSMLARAQGVELEHYADDIVEKMFSHCPFSKEMVQDRLLSTLSSILSAKDQKSFNTLINNGGIYTSPALEQSSSKKSPLLHPLSPVSFLASIPKGSTTTGPSSMSKFSLGPSMLQKSDPVNQQSAARKAAALQTLGTFNFRNLKEINLLRFASTTVVPFLNHERATLRKEAGVALCKLILGMLSEIEEGRHIAQIIFDILKRVASVAISDSERTIRLALLQSFKVPLDKYACQVGILSILFPALNDEALENRHQAITLLGRLSQHNPAYVLPQLRKMLLQLLDQLDQFDDGPELEESAFLLGHLLVACPLLVKPYVLRISNMGAASASTHYDDDDDDDDDDDIVFESGGSLKSSKIWNRNLFHSHAYYKSPLASSMLPAHSSYAGSLTAPATSSSSTHQQPMSGFATSSISSSSSSSSSRMLLSWGAGGHLIWAIGELASSFVASNADFTKHLDKLLPLIIDALQDDTISSGKRMVAVRALGKLVQGTAYAIHPYVKYPKLLPTMLAAVRNEHNWITRREVIRTLGILGARDPLLCKNELDKKEDDLGGGGGGNTSGKSRTAAAEGGGGGGGGAMRGRDGRRDKKDEEGGGTHDDDSTKNSTMAENANNNKGGAAAAAAKRRRDRRRKNMKKKKKLCEDDGDDDDDDNSMEGATSITANTTNSSISEMVLGVSSHGCVGTEGFYAKKATTSLFDILRNSKLKQHHAKVIQAVMFIFQSLGSRCVGFMPHTLPLFSDVLWTCDSANRMMFFSQLNGLIRIVGHHISPYMDEIVSVVLEFWPNTSLQSSILILIEDLSDALNEDQFIPHLPSIIPSILAVLHEDRSPSRERSFRALKALETLALNGNLNSSLSTVIPSLTAAFNRGGGGGGGGGGKGKESSSSDVTQILKTPLTIFHRIIQILGILCETGEIQDHACRVLQPLSRLVAKYPSLRGVILEAICTLVSTLGMSFMIYAPVISGIVNKCRDVKDRSLLRRYRSIVSSLLRHIQERRKENLALSGCHVVGQDTFQEAVDKLASEIESAISSSGGGGVNQKNLKDAWNSTKCHRREDWVQWMQGFSIELLKESPSPTLRACSELARKYQPLARELFNPAFVSCWIALDDSLQDELIYHLELALQAANIPHEIVQLLLNLVVLIDCGKDDRGRVEFMEKQGRPLLIDVRLLGTLAERFHAYAKALHYREQHFRTSPSSAIEKLISINNHLQEPETAQGLLKSYISICIYLTNQYMTWICCCAMMFLRRYEKLEHWNDALEAYELKQLKDPASVSCMLARARCLDALGEWDRAVTLCRKLWQVKNDELKSVVAPLAAAGAWNLGQWKWFESLVKRMNKDDEEEGSFFWALHAVHKNEFDEAARCIERARRTLDVELRALLGEGYIRSYRVIVKVQQLAELEEVIVYKKAAMAGRNFVIENIQKTWARRLQGVQKKVQVWQHILSIRQLVMSPRDDIEVWSRFANLCRKSKRLELSARVLKMLLGYDPLRLALQPTIYTCLLMTMYVSMDGSFAFRAPTSDDATVAAATPGGHVSSSSSSSSSSSSSMENGSIRRLQSRCHLKRGQWEMEYFEIKTNSPTAAGSVDDDYDEDDDEDDDMVYVNSGGGGNTKRTSRTRGKLYATTVSRVIDSYQMATICDPKNQRAWHAWALMNYQCPNVLEFISYPSQVIAHYQKNNRPDLASERVVPAIQGFFTSIQLQRKGKRLQDILRLLHLWFEHGSQPEVQRTLVNRFETISIDVWLDVIPQLIARIQTANSQNRKLVHGLLVRIGKAHPQALIYPLAVASKSTSMRRRRAAENIFDKMRQHYPVLVDQSLLVSRELIRVAILWHELWQEAIDEASRLWFGERNFHAMYYTTLLPLHKRLALGPSTIREQRFVSMFGQCLDMANERCRMYAVHQRQDDIDRAWDYYITVFKKISKELQKMRELQLREVSPKLLSAKNMVLAVPGTYEVGRQVVRISRFVSTLKVIDSKQHPRKLKVKGSDGKLYSFLLKGHEDLRQDERVMQLFGLVNSFLAQDRETGHRDLSIRRYAVIPLSVQSGLIEWVPHCDTVHLLVKEYRDREKILLNIEQRIMVQMAPDYQTLSLIQKVEVFQRALADTTGEDLSKIFRLQSNSAEAWLARRSTYTRSLAVMSMVGYILGLGDRHPCNLMISRRSGKIVHIDFGDCFEVAMHREKFPEKIPFRLTRMLVKAMEVCGIEGIFRFTSEAVMRVLRANKESVMAVLEAFVYDPLINWRLLDRRKSRRGKTGSSAGEGDSDESNSSETEDDEDEGDHKEEGRVGKTTTTSPQSKHRGGGGGGGKGKGWKAPPKKKDDRKQRRNTPGLLKPELDDNLARGSIVRAMIGLNERRSLNKQALSVLSRVENKLTGRDFAMSGHTILDVRTQVQKLIVQATSNKNLCQCYIGWCPFW

>3517

MSSSSAADPSSTGGGAHVAQHPLTDQLLPIFEELIANPVVTVPLSSSATAAGAAAAAAASATQLINSGATKSALLRLQNLVDEEADSLFHRSNAVNAQTKLLSQFIDGQLTKLLSQPQTRLSGIHVIEILRAVDYVEIGTRLKTFHDMLIKVVTDSSHSSKEAARVWGLLVDVAPGAATEMVDAQLVKCLVDLFGAESLGASKRFVGALMLQEVALRVAPQSVITKIDDLMRGIWMALRDTQDVTRAAASVALRRVLRIALQSAETSVGDSIMDNTLGQVVKSLMQKQREVGHGGLLALNAWFTASVGSQRSHGHHAIGELRIKEVYGLIVQLLQKPSTFAEIRLETVGCIPLMAQYDRTRFVDVCLAPFVIWAGTCYRNLTGPDERGPMFLAIGRLAGVLQDKVPLFVDRVMVFIEGSLVKQPKRDRCPEAGTCFAMIAAADPKTSRRFLRTVLHPLFQCTPTAEFAADIASICRSFPELRTFCMEKILEVTKAQFSNIKQRSALSAVSEDDTRILLNGLHVVSSLDTNGYSVLPFMTEMVLRFIQDPHEQVRRAVIAMCLKLAHSGCCAPGSPCRIDDHQGTMIHQGHQHIRLLLQVMLALINSAVADPESDIRLSTLESLTEAYDHVLSLQDCIRALFPALNDKHNNRIAVIRILGRLAKRNPACVDTMLRRVLLQSITEIQYFAQSKKQEQSTWVLAAVVEAAPGMVRPYIPALLKSTVDRLCDKGSPQAVQTALLACIGRLVRHSEGGDVDVMLLRTVRSIAVLHVMDSSCTPKKQEAIRALCEIVRATRDVAVYEQHPELLRVLLSALHGGFKETWPLRQDVLRLLGIMGAVDPVRVKNFYRMENDGKGSSNNSNGIGDANPNHHTAPVAGLRLRPEEAAAHSTVRAVMNVIELPSTHDTQCVEAMQALVNICTSRDMQLPSMISFFPKIIPAIIRHIHAHAKQREPLFRQLAILVGVAKHHIRGHLDDVVDVAKQYILCDDPEVLSQILLLLTELRRTLHEEFKPYLRVVLPLLAYAATEDVARSASKVFECFVAFGKLMDGHLHIVLPCIVEIATSVGAPVHTRVSAVSAIFSFSKHLMTISDHAARCVHCLCPILQARTQSPAEARDLVHAASTALLTLAQNLGPGFAKFGPIIKPIVAQRYGENSQDYAKFVHTIDEAVRSQTRYILPEMPPKAIDESRFTQPQTNRRREEPQHRFRAVRQHLLPIERTGEEEWKQWLRTLAIELLRASPCNAHGFALQLANIHEPFARELFHPAFAACFGEMDSNCRQEVIACLTTVLKSPKLPSEVLQELLNLSEYMERVDGGVGTSSSSIGASSSSSSSSRGRGGLFEISTVIDRSETCSLHAKALHYLEAQFVELTREYEQGLSHGRYNPLTDENWRQLLAVTEKSIYLCNLLGQRESANGALKYIQDHYSRLTNTAHKSSFSSQAPTTTSTTAADDVAGAQNNAGGFDILDAELLQKLQWWSQSLRAFQERLKTDPNNVANVRGVLQALDAQGEYSKLLEVYKAFARRASKKESAELATTGARAAWILQSWEDMEQFTSLVKSEGYQGTTAVFYNAVIAVHKREFRKAESLIGRCRRELDGALAALVAESYDRAYELLVGNQQLCELEEIIMALREPKSISHWKGLWERRLGNMAYEGWQGTLATHSLVLPHTEELDMWIQFVSLCRSNGRHRVSMEVLQKLLGDRKVADAFTHPTDIPRPIVALAAVTHMYESGKQANALTLLKSYVERYSTTEATTLDVLNDQADYARCRARLGEWTAAQNNAALCYNKALTDDVLHNLQLATVLDPSNGSTWHTWARVHHNIVIKGQSSLLDSQAMDHHVISALQGYLKSIESQQNLQDVLGFLSLWFVHGTRPAVMASGVFDEVMQINSNVWLRVLPQIIARLHTPNAILNEQVQDLLRNIGKTHPQALLYTLNVSSGVAVIGGDTSEDVLQRRKAAQRVLTRIAELHHNGQQLVQEAALVCRELVRCAVLWAEMWFDELDRAWWCWGREKDAAVVWGMIEPLMQLLANPESTMEHHFIQDFGVHIESAASLIRDAAMLPNHGVAQVEDAWGKFRTIVERLDKQIQSMSSLALQVVSPTLVRNGRNLLVAVPGQYREHGDFPRIASFHSTLKVMSSKQHPRRISIYGNDGVTYKFLLKGHEDLRLDERVMQLLGLVNTVLTANPSTNGKDCLVQTYSVTPLSDNAGLVGWVDHCDTIHQIIKDHRVHSKNISAELNAMKMFYEELPRLTVMQHVEPFEFALESTEGADIARSLWLRAPSAEHWLDRRTTYVRTLATMSMVGHILGLGDRHPSNLMIHSFSGRVVHIDFGDCFEVAQTRSTFPEKVPFRLTRMLVKAMEIGGIEGLFRHGCVGVMTVLREERNTLLSILEAFLHDPLVTWWRDDNDDAAADQPQTATTQQQPQRPQQQQGPAAAGFSLQRGHGEGLDVGSVSSLALPSLRRRTMASVRGPALNHNQQTKKAQKVMQRITEKLNGKEFISVDIQRSAFAAREALPVEEQVNRLILEATSHENLCQHFSGWCPFW

>3518

MGQISDIMNHIGTSPTRQRVADTCATLLDTLVKHSREIADNTTIQTHLLGCIVEVSSNADNGVGKAIAALETVLDASVMETNAKRNHCTLICTRILEHESVVHDSASCELLIGMIRSLILESSFTAPEVMTSICECAIALVRDQSRQTVRTQTRAAAFQVLTHIVTNHAIQLKTVKQITESMRALSPSYLCRETINAENPWLRLWSSKLLAEIIRHSVFEISSENAESLVREVETQLVNSEDDANAFLGVVALVEAFSSVKTLSQAYSWNANKWRQCLLETDLLAATKEVQDAVVIGIVAITSMRISDIVSAAIVALCQQHMTRDFSGASRALENIIRRHKKQISEAERVEMLERVLQMLLDKDTTVGDVAIPQEAGQLLASLLEASGFETTESYTRAQDRVFDVASQFCAESRLLLGASLLQSVDTALFRGGNPKYLQMLEGATVPWASLTANDIQGASRLASIVRTGRALSFAIDALTHEQAGVRLEAAKSAIVTCEHLIVSARSGDDDDAHSRRLDNVVQNAVESLLDAAVADIDCTVRLTIMAGFSSPFDPYLSMPDNLQSIQMAKNDTNHEVRVTALELLCRLLPSHPAFVHPVLNRLQDYALKEIEARDSTVSEILSASHMLRTCAAHRALLPQPKQVQQIVLDRIAKQPFASRMLSHEFLQLIKCVLDTYGPLHHVDCASLVHTLKPIICDGHTALLRSLALETLASTVRTLGVSLETQLFSEVYNMCGGILVNESKEDDEPRKSAALVLSVLGAMNPVKIRAVTRAFDAEPTPNAAAEEGTDLLPSTKPHHRSHPHIAEKYPSIVLFLLIKTMQTASDPRQQKDALRCAYETLQASPPNHKSHLMTQLVPQLRRWLRDPEKSFLHGGILKIFTDLVGLLQQFKETIPAHIGTDILKSVQTLCHNPQASQPPLNILVVELLDQLARALPMPDIRDHRWAVEFVHQRLLRDRREAKLVISVVKALESFASFLQEKDKRHVLPHVLDCLDIPPEVNREGTTGANVLQAIQDVRLVNSACYDFIAMLTESHTTLVKDFSAQAVHKLMQLIDLAWDEEDRGNAIHTAANLVATVGRPANRFIPHVQRLCDARGLPRDCFKLMVDAIRNGVQKVKLPPVKQEDVPSLDCPLAVISNNPSLSRADFERELRAICRFEESEFEVLGVSHPGGQTVVHFQFAKHLPQAESRLVYFMRKANEAQSTLRRTLGIISVEQKPQNSIRLNSDFVTQLAALPEAQARVRRKKEFSWMNWMQVACLNFLRNSPFQPFRVLSTLTPTNMWLVKEVFPFAVGAVLSQVETQQRANILQTFNIVISKAPNDIRQILFSLAEYLESERGEKKAVVSKVAKTVSCTVERDAPDQKFGINYDQDNRGIIVTKLAPDGPGMRAGVPVLGVLQTINQQKVHTVNEIPGLIRGATKIHLVFTVMEEVRRVPETKPLMGLADVAKAAAESEFHAKEVYFSEVLLQQLYKNLGAGKNRKDAEAQQVLQVVESLMKCYNRLGLPMEAKGLVNMIAAKFTENIIAPENFGFDEADTLEQLHWWTDALRLYRSRFNDDSSVSSLVGMLRCYDAMGEIEMMQKTVESYWPGLDEESRVQVAPHRAAAALALGDWGTFDEAAAFSPESLDTVERGVYLLRNGQREALTKFIRDERDARFDQFSETFDDSYLRCIDILAELQHLTHLEEIVQYQFSASDERRSTLRGVWRRRAAQLTRQPRVWKTMVTLNSLVLTPEEDLLNRVDCINVCSKQGWVSYAEHLLQQFVGGNPISIENLSVMDPNVTYVYLKHLYNTNRKMEAYTTMMEVLQSAHVSPSDTHSEVWGRCWFLFGEWTIQLNSSDVPIAIDALSKATELSPRSSSAFHSLGILHYERSRDVSQPAESRHDSCVAAVNALINSVQLSSDGKNSVMQDMLRILTIWFSHGGVRIVNEAVESGMQVIPDHVWLRVIPQLIARMGINSHRARNMLVDLLIRVGTAYPQTCIYPITVSEKSTEVVRKRMAEQILAGIRTKCDQLVKEASLISNELVRIAILWSERWHAQIQAAAHKQDDAAAILHILQPLFDELDHGTTPNEKNFEKSFGQTLKRAKTSLSSKALDQAWQLLKQVYSSLTKLIAERKLVMNDVSPVLDNVHKSIVAVPGTFDPTKPVIGISRFQNKIIVMSSKQKPRRFGMEGSDGKLYRFLLKGHEDLRQDERVMQFLDLINTIFSGDSASATLELFVPRYAVIPLTDNVGLIGWVEHTETIYRMLETRRQDFNISVYDEVNLIIQKGHLRHIEDYHKLAKPDRKNLLQVVMNGTPSDELSRIIWDKNDACEQWLEYRRLYGHTLAIMSMVGYVLGLGDRHLNNLMLQQGGAVVHIDFGDCFEVAMHRAMYAEAVPFRLTRILVNALGVTGVDGVYRHTCEHVMKLLRRHKENLLSVLEAFIYDPLINWKLAPAAAAAPPATPHQSSGQPTTASAAPAPPVPTDEHGVPKIIADLDAPAQAKQPALSCTVRGRTSMGRSVAAEYEGEQELRNQQGDAALLRVHAKLSGQDFETLATSSASGSPGSATFSNHIDRAAMNDFFVGESVKDSVAGPLLATYLASQQVLPGTHVLDVSQQVERLIQEATSLDNLAEAFITGWAPFW

>3519

MATQPSDAQRFVFAVRNVVGRAETKHNFIVDTVSKAKDLQRTAQQEYEAHLLILFKALDLYQLTGGSPSEMHCALTTIEILLQLPLSSSQLFQCNSSIRVCLLSAGDVATMLEAARVLGLSIATSGPNDSVRKACDDSMIWIGLTDARSEARRMGGLAIVDEVLQRVPVLFLKYLSDFFDRIWGPLSDPTTEVRDFAIHVFDRAMKLLSTRDADSRLTTFSQIKLKVRSMLQSKNIEQNIGALSVLDSFVAGLPVDTKQQAAAAYDDLVTMLQPLFVVSSGTNCNPKVRQLLFHAYSALCKFNTVSFSNKDLKNAVNYGLEFTKKEVERPYAFRMLRDVITLVRTDAFMPYQGQTILAIKQVILNQKRQGKPPVWESLECLATICKVCPSSEVEAHVKSCIEHIFSYGLSTQLIDCMRDIVAASSTMARLSLEESMLDLISITLCGLPFRQQQGGSKSSIDGLITAATEPTEEQIVIALDAFLKFGFSNSEQLGDFLRDSVLPFVDSDKMSIRCAAIKTIATLLLPTESSAPTSPGSASANTLLPTSSSSQAMIAGTQHNLVAAQSVLECLTFSRRMCVDFVLSRLLVIGTSDNEPSIRGLVLSSLNAASYFPFLCEPQFLTLVCTALGDEDASCRLAAIDLICGMLPCSPSLLLPLLRREMVKVLQTLSSTERPQYIEQGLRCLSRIARGAPLFVRPFFNNILQVLVPRYRVVTARDVTFQPLLEALVTVSHSNMFAKRHSLADAFESAFSPLISKTLEVLQSIPMDTKSKQQLRLLCIQLLTGLLRPSFDGVSPYQHFPALYHTLSSIVRNKDECADCRLESLRCLGKIGALDPHKFAELEIAEGIATSQSGGTGSAGPLKEKMCTTIVLEALAVALAPRSARVAAGGSEPLLRTTLKTALMISERSSACSEIATLFQPLCGLILDLPRQSRIFYTALHELGNLISVCGRRSLKEAHHFRALIRSVWETPRARFLIVRLCSMLVEVAKHNFDSQSEIALRDNLGLLLPKILDELVASEQLSYNIAAMNFILQHCQAATSLTQRICFALLELLQSSHWSVDHAGHVLVVLTKVATVSANRDLVPPLVRGCLAKLTTFVTTTVPVPSESNPFVAKALGLLRTLASEKYLEFVPFAADVLRSLKTLRLNNQELTFLCTQACRGSQTCPPALMQRSKQDLDAHVNVLLETTFLQSYAAALAEVGEIWGDTKTQPGLANLSETFANPDAAVAAPDALLVIGEARIVNKSTKVTTLQTKDDWLRWYDEFCKVLIAEAPYHVFRCVSASQGVNSASLVEKLPDFVQDIVNVAFRSIHTNGSFHLKTVLSDMIQKIAAMGGQSIPDDVVVGLLGIAEHMDMCGFALPVPHLQLAELAQNKGMLAKSLYWQEQGYRTTAVNHSKESSQSLIATYASLGHVDSAAGLLHAEETTKHEPSVHTSGQSLSHHHHVRASSVGVVPEFGIVIGALGGASSPKAFQGDGGVHNNSVANNSSSDLMGLDWQDHGSPTAQTSYQQQQVLMRLGKFEEALEMMNKHSMMASPNGNMSLQLSMFDGDSIFKNSYGVASPTPHRSRHTRTRSYSTVSDSADPYPLPQDTELTRKVEADGGKIWCLSEVGDMQGTLSEWRLLLPHLRSLRQQNNGEEEENTIQCMIAPFVADAAVRLNSWEDLSDALTWVPKDSLTFGTSTAALQISQRALGSAKESITQARELLLEEITGLLHESYARAYDHIVSAQQLVEFEEIIGTLGVTEPLHRAQQSDDVSLLWQERICSSAHSVSSWKRMMGHRGLLIHPSKDISTQILFVKLCREYGGSRRLERFTLEQMLGSSSPSYDQLIDPSMNPRVVFEYITYLSSCGQLSKYASHGDERSILEKLIEIHGGVPENKTLEARMHLRRGIAADPATAAECYKLATDCDPTWFRAWRSWADANVLYLEGNANHQKTDEPFMNAIDGFVKAIELAPTTSNKLQDVLKLLSLWAKHANSPKRLSELERRVFDVSLSVWQLVIPQLIARLDSGSDECCALVAKVICAVAMQHPQSLIYPLNVCTASSIKRRKLWANDILAKMQSRFPQLVAQGTMVTTELIRIAALIHEQWHEALEGAASAFFGRRDNVEMLEAVLPAHEKLKKAPETIVEVQFFTKFHRQLEEARHWVRSFSLTGKTADLHSAWHIYHAIYKQIDEQLKSGSTLSMQYCSPRLWEAKNLALAIPDARPKEGKETVRIAAFDSKLTVIASKQRPKRIAFLGTDGAPHKFLLKGHEDLRLDERVMQLFKLVNTLLLSNARTSKEAGFQIQRYSVTPLWDTVGLIGWVDECDTIHELIHSYRRERDITPELELKIMHNILPTEYQKVYDFLPVMSKIEVLEYLWDHTSGQDIRKAMWASSLTCETWLERRRTFTSSLATMSVVGYILGLGDRHPNNIMIQRSTGRVVHIDFGDCFEVAMTRDRFPEKIPFRLTRMLQNAMEVSGVDGNFRSSAETVMTALRDNKDSLITMLAAFVQDPLISWRLVKRTGNEMLMSNDANKAIDESHLEVAIGEEADEVVDSPLMTLVPFSEDDDLAALEMTRRQRAETSAAEEDRKASEEEVAHQGVAILNRLASKLRGQEFGYSKKLNNGRGGRRSLDPKSQVSKLITEATEISNIAQSWSGWYPFW

>3520

MTESLTRYLSGLRSTSSSVRHRAAQSLRLYIESESRDLSHGLYMKWVTDISARLMLLCNSNENADRMGGIAAMDELVELFIAERNDQTIIEFAHSLTKVFEKIPSADPPMLRVASKALGHIVSTGGTSLIEFVEDYHVKPALEWLKNETFHVRRHAAVMILKELSINAPSTSFRYMEKYFDFIWSAFWDSKLVVRVSASESLQSCFRLIQQRESNRKTSWYNRALEEAENAFKRNSSDATHGALLILNELLRNTGDFMHSHYARACRLVFSHQDYKSATVRSAVISLFPRLAKFNTSVFVEKCYRPCMNHLLEVLFSATTTTRPDALLSIGKLSLAIGPLLARDEQALMAIMNGIKGGLQVRKKDFDTHREALACLRMLAETVGPALIRVDLESMVGPLFQNDLDSSLVEALATIVKKITPMKPIIQQKLFERLSSILRTRHNEGTGSATTPTSRRLKTTSISVTGGMLSNLFLSATGAKINAASENNAPLTEVSSAIAMQALALETLANFDFQGNRLIPIMSFVHETVVKFLDHEVATIRKSAALTCCKLLLPPGEDRRENRVMTSDDFARPVTSVLERLLTVGIADTEASIRLRVLASLDSRFDPLLALNDNLRCLFIALNDEVFSIRQTAMSTLGRLTHHNPSTILPSLRQTLVQLLAELEFSGDSRGKEEGALLIGCLLRSASQLAQPYVLPILRVLMKNLREDSERRSQSRSTVSVSKAILATLGDLAEVGAQELTPYLGQLLPDVIDEMRDSSNPQILQVAIKTLGQLVSSTGYVILPYHEYPELLDLLCQALQKSGDAFESLRVEAGRTLGVLGALDPYNLRLFHLQKQGKLTDAKKKEFRFSANAVVASEVFNLMAIEAKPLENLTTNALNFGQSVANGSVVGIRIGNGNGGSGALDGSGEEEAQRLGRSLHLTPARKVAESDKQTSGKLTNIILEVPPDDLLPSMIPDSSQYFPTVAINALIRILLEPRNSVHYQGTFMAIMYICKSQQKRMRQHLDKIIPAFLYALEKVNRSLRKFLFEQLCDLVQIVEEQIQPHLDHVALLSIVNSYWDEHLEEVLNLVKKLANSLGENFRAYLPDLVPQMLRVIRTERDNHVRPRTLLVLKTAVSLGRLLDGYLHLIIPALVALIQSDADVNARKQGLGSLGSLVKKLNVSVYASKIIHMLARVISSQPEMVYLAMDCLCCMVYTMGDDYAIFVPVISQVLGRHTSRSNDIFDRYDLLVSKILKYQPLPVASWATDPLKSRVDASRDRKDSSSSAQDETKSLPCDQKNLMKAWEASQRSTKEDWNEWILAFSVELLRESPSPVLRACKELASVYQPLARELFNASFVSVWPHLSSLTQDNLIRSLELAFQSPNLPSEILQTLLNLAEFMEHDDQPLPIDIRLLGSLAEKCHSFAKALHYKELEFNTNPSTDGIQALISINSKLNQPEAARGILKYALEKLPGIEVKASWHEKLLRWDDALATHDRVLQGNPNNVEAIFGKMRCLWAIGEWQKLNDHVQETWSKIYGERQDLDGEQVEGEKLLDVAPALKRELCSSGARVAFSLQNWDSISKYINSDMDATESHLFKAVVCIRRMELDDALSSITECRKEMDPTLRSLVSESYARAYIPAIVNLQMLTELEEIVAYLKTFAYKNNGELTLLSAPTSSPSSTPTSTAVSRRKLSISSLNFVSSSSFTSYGHEKKVALQKLQTIWTRRMLGVERNIQVWQSLMLVRSLVFDPKEDVDIWLKYARLCLKSGHLNLAASALWRVGAQPFIRSVERDPNVPIPINLGGNAGASQGFANGLLSLADSAAQDPRVAFSYLRHLWAENREDVALKQMDYFIEALEQHGDPKNEDLRKLRVQVYIQLGEWQMSLTEPHKQAYDYVLECLETATTLDPTNDRAWHEWALMNFRALEATVKESDEGDPTRYAVRAIQGFFRSISFGHTSYDVTKDVLRLLTLWFTQGNRSDVHMAMVEGFHEASIDTWLDVIPQLIARIDTPHPKTSELLHDLLSRIGQAHPQALIYPITVASKALNPTRKQAAEGILAAVRRHSSQLVYEADMVSRELIRVAILWNELWHGALEEASKHFFNNRDVTSMIAELAPLHDQMERINIEEVPTLREVAFYQAFARDLAYAKEWTNVYESTKSLDDLNQAWDIYYSVFSKIRKQLANLSTLELANVGPKLLSVQNLTLAVPGTYKAGAPIVRIQSFDRKVTVLTSKQRPRKVSINGSDGKAYPFLLKGHEDLRQDERVMQLFGVVNTLLANDSDTSKRNLAIQRYSVLPLSHTSGLIGWVPNCDTLHQLIRDYREARKIQLNVEHRLMVQMAPDYDKLPLMQKVEAFKYALGETTGQDLYRVLWLKSRDSEIWLDRRRNFTRSLAVMSMAGYILGLGDRHPSNLMLDRVSGKLVHIDFGDCFEVAMERDKYPEKIPFRLTRMLTQAMEVSGIEGNFRYTCEASMRVLRDNRDSLMAVLEAFVYDPLINWRLLKKDAVPSHAQPEDDRAGNVERGDGSDANIERSSVRAENEIEREIDAIGEGDATNVGDLEEDKLAAGIIKSSNSSAPVTPRRRRHSSSEAAMLGYNMDMLDSTIARNSMADVQRDEFGSSVVAAEHPQLNEKALSVIERVKKKLAGRDFDDGSRVLSVDAQVDRLIHQATSHENLCQLYYGWCPFW

>3521

MQIQDREHVETLLFSLVQRLDVSDAATRIKRVSELYAAVASLSRQLSAERFAKAIGQLTPQLYALLHQGTTESAQLGALLAIEHLIDVSNEDQFIRFVNYLRYFLQQPQTSRRALLAASSAMGKLASSSVSGTLVASFVDFEAKRAFEWLQEAQANTHAARNEALVSQRRLAACFVLRELAQAAPTLFHVNLTTFFQSIWGAVRDTKVEIREAATCALAACLQLITKRQTRHRVQWYCKVYDQVFEGLALRINAISSDSLPLSSGHALTSWESAHGSLLVIGELLANTGRFMVPRFREVCDTVLKYKDAKDKLVSRAVSRLLPQLAEFCPGAFVQYYLDVCVAHLTRRILEYSAPSVERGVAFFAIGRLSLAVGDALVPHLPVILPLVKESLAPHAGTKRKHNRNKLFCVQTLTCVANMSRALGPRFEPFLFQGGVLETMMIGGLSDELIDALAGVVASVPSALPYVQERLLNEISGILRGTPFSRGSAYGVIHPPGGSSGSALSSGDSSDLTLDPTSYDVTTETPPLKQTAVQALFSSMKRGFSSAEDDPSSGKVSIMIKGDTGDEIDTILLSLRTLSCFDFSGSFCLMPFVRDCVALYLKNPDARIRKQAVITGSKLLLPSLDTSNSAVMSPWRHVKKRGPTGRVIDLVLTQLLQVGISDMDVGVRRSVVESLDPRFDELLVQETHLRLVFLFLNDECAIIRESAMQLLERLAPRNPAFVMPSLRRVIIQLITELQHTSDMRMREESTRLLGHLVRGAQHLVDPYVVRILEVLLPILVRGNAPLTMAVLVTLGELALVSRTQIAAYERYLFPLIIHTLQDHSSIEKRQVALQTLGKLAGSTGCVVRPYLASPKLLEIFLSLLHHNVGSPWSLRREAMRTIGILGALDPYKYKLCISRASLRQANEAETSTTREKLGKNADSVPLGLSKPAATTMVSRYPMLKIDLQALGLTMNLESFDRKATNDALDEKQQIELQLFTAAPIGIRKNDSSTVAGTLDAGSQLSHPIKSAMEVPTEMSSSPFAISRTKRQVGLNGMSLSDDSFDFESSLMDSELEPDPTELSPASEAYFPTVAIHALLRILREQSLNAHHHGVIQAIMFIFKSLSTQCVPFLKYILPPFLRVLSRGEPQLRESLFLQLTALISIVNAHMKPFFPPIMVLAQIFWGSHLPQIVRLVEKISQAAPHEFRTQYFPKLLPKLLEVLQPQFGVGGGTAEELSGANAGIKGSTGLTKGSGDFVVNVGGGSKDHHGQGSESSSGGKIEHVASTYDVVQKGGADGTTAVAVAAIQVQVVQSLIVFGDAVEDYVFLLVPVLVHLMESLDTPLEVKITIVYALARFCVVANFEQYAGPRLLLPLARVASQVSTKGAFFFSRSSNVTVNSNVSGSAGNGPTNTAAARIATSKSEASRFSEVMLYALGALIIQLQAEILNFEQVMHQIIASLPFSHGGSLATAGKDTKGSIPSSFPQLMVQQIAQALESLRRGTRVPRTFLLDPVFMSPTLRKFSTELQAQTAASMTLAASPPTNARLHVNQQNLRRAWEASQRSTKEDWLEWMRRFSIELLRESPSAALRSCCALAQAYNPLARALFNPAFVSCWNELYEQYQDYLVRALETAFQSDTIPAEILQTLLNLAEFMEHDVEALPIDIRELGELAQKCHAYAKALHYKELEFHTSPSTCIEALISINNQLGQPEAAVGILKYAQAHHSRVIQVKETWFEKLQDWRAALALYDRRLQFTQQQSGENEGFDIELCIGKMRCLEALGKWEELSVLAAKVWAALRSSKHSASQTSALPPRSRQSTATRNLSSSSTGINSGRGNSSNLSRGGSSHDSSLSSVSSSDPPLSSSHQLGDDRAEEAAALRRVAMLGARASWCLSQWDSMAQYVTECAAQTDKTKVNTTILSRAASGNNHGYNYGHTHGHGIFGADEDVTELSLYESVLAVHQGSFDQAAKLIDATRKTLDTKLGALVGESYNRAYRSMVTLQQLSELEEIVTYKKLCAQMSSGEEAARYKQHLMRMWRDRLTGCKRVVEVWQQVMVVRALILSPHEDIDTHLQFASLCRQSGNLLLSLKVFTNALHVHGGVGTQGSGISEMLSVTPSNSGESTLFGFGEKDRHRVAFAYLKHLWAAGQKQDALVDLHRLVARISSMARHRAQGSGGINTMASPSFEAIPVRTDEKELLVKCHLKMAEWQLAVHDQQIENVPVENVLSSLRLCTELEPRSYKAWHAWALMNFQVVEHSTHSQHISAHATTSAVAASSSVAPFIAPAIEGFFRSVALGRSRWAANVQQDILRVLTLWFAHGHCSDVHTALEKGFRSVSIETWLIVIPQLIARIHTPYPRIQKQLHRLLVAVGQQHPHALIYPLSVALKSSVPERQQAAEAIMSTMRTNYVELVDEALLVSRELIRVAILWHEMWHEGLEEASRLYFGEHNVDGMAEVLRPLHAMMERGPETLREVSFHGAFGRDLREANEWLQRFLSNRRNESDLNRAWDLYYHVFRRINKQLPQITTLELQVVSPNLLSARNLQLAVPGTYRAGHAIVKIGSFLPTVAVITSKQRPRRITIVGSNGLEYMFLLKGHEDLRQDERVTQLFGLVNALLINDRHTSKKDLKIHRYPVIPLSDNAGIVGWVPHCDTLHQLIRDYREARKILLNIEHRLMLQMAPDYDALTLLEKVEVFQYALENTAGQDLYKVLWLKSENSEVWLDRRTNYTRSLAVMSMVGYVLGLGDRHPSNLMLHRFTGTIVHIDFGDCFEVAMDREKYPEKIPFRLTRMLTNAMEVSGIEGNFRFSCESVMAVLRENRHSLMAMLEAFVHDPLINWRLLSSTNVLRPSHASNSVASSVTSSSIGSSAISMESKSDDESSNASFHPLENAAHEQAFAHESTESSDREEQFVHHRSNSISVPGSIPKFGTTPFASCKMQAAQEGGGMEDTNNASMMSENQDKRLDDGAGDDVNSETFEAQTLLQTQQEHQGTNAPVPRELSIRLATSRPLCLDSQETEAPPLPSSLSSRPMSLSKSIPLPVRKLPPRVPAVYRLRDATIDENEESNTVEKINHTTTNLHAEISSLAASVSSVGHSSLSRSFSVTQAQLHVQPRGPAKQPQSKPPLLSSSSGPVPRMAPAASTSFVSVTATRIPPSSEAVPPSVSSNDLHASRSVRERELLNALGPEGIAAPRVALNEKAVAVIRRVQAKLSGRDFEGDAGEPLDVSAQVQRLISQATSHENLCQCYIGWCPFW

>3522

MSASASTSAQLQVVDGVALTEIFKGLTSTSEEERSKYAEELHNYLSSIARDLSSEQFNRYNNDINKTIFDLLHGEKTSEILGGIAALNALIEFDSGVGKENAGKTARFSNYLGSLILSNDLVIMKQAIRTLGKLATLGGNLTGDFVDFEAKRAIEWLQSDSKQHENRRHAAILIITSLADNASTLLYPLINQVLENLWTPLRDHKLIVREDAAIALEKCMHIIYDRDVNARSFWIKRMIESASKLLNENDAADGADSNSSYNITFSGQSTENIHGSLLTYRELLKYYKDPFIVSRFEQIYENTYLYKNHKVAIIRQELTNIFPLLCKVNTELFVEKYLHRTLYYYLSQLKKYKSQNNETANTDKSAIFKSIGLIALEVGNQMATYLDAILDNIREGLSYTSNAGVQSILAKAASNESPNISTTNIASLASGSKYTASRKETEPAIFDCISKLSIAVGPALTKHLQRDILDMMFANCSLSKHMQDVLQVLIENIPTLTNLINEKLLNLLSLVLSGKGFQPPGSPFGTIKVNASLARDCRLIMISRDTGMSINTILMNQERYEKLDSKILIQALEMLAFFKFENYQLNEFVRYCTITYLEHNNAKVRLTATVTSCQIFVKDPICQQVSVNALNTVNEVLGKLVSISITDPAPEIRLAGLNCLIKAGNFDPQLSQANNVRLLFIALNDEVFGVRKVAIQILGRLSCINPAYIVPSLRKTLIQLLSKLEYSTTSRKKEESAILLSLLISNSKELTRPYVKPIVDALLPKAKDLSSSVASSAIKCLGELSVVGGEDLKPFIPDLMPLILDTFQDQSSSYKRDAALRTLGQLAFSSGYVIQPLLDYPQLLGMLVAILKSETSPDIKRETVRLLGILGALDPYKHREVEQNSKNIPVEQNAPPVDVALLMQGMSPSNEEYYPKVAITNLMKILKDPSLSIHHTKVIQAVMYIFQTLGLRCVAFLPQIIPGIINVMHTCQLSMLKFYFQQLGDIVLIVKQHIRPFLDDIFKVIKEFFNAGSQLNIQVTIINVIQSVSRALDGEFKMYLPEVLTLMIGVFEEDKSAKRSPSLHVLKSFVVFGSNIEEFVDIIVPHIVKLFETGPVELRRAAIETIGRLSKNIMLNDMASRIIHPILRILGQGNIDLRESCINTLTYMLVQLGPEFTVFIPVIKKTLLQKNIHAIKFEQLVGKLIGGDPLPLHLDIYKDYDYSLYDIADTDMPSKKLPVNQASLKAAWDASQRRTKEDWQEWIGRLSKELLLQSPSHAIRACAGLASDYYPLAKDLFNASFASCWSELYSQHKEELVESFCIALSSPSNPPEIHQTILNLAEFMEHDDKPLPMSISTLGQYAQRAHAFAKALHYKELEFYDQPTTPTIESLISINNQLQQSDAAIGILKHAQLHHDLQLKETWYEKLQRWDDALKAYNEREKIEPENMEITIGKMRCLHALGEWEQLSELARSKWDNSSSEIKRSVAPLAAAAAWGLSQWDRMDACIKVMKAESPDKAFFNAILSLHRNNFDDASVHILKARDLLVTEITALVSESYNRAYGVVVRVQMLAELEEIIKYKCLPSGSEKRAVMRKTWNTRLLGCQRNVDIWQRMLKVRALVIKPKQDMDMWIKFANLCRKSGRLNLAEKSLNLLLEEGSPENPSRAPPQVVYAQLKYMWAKGQRPEALRHLVDFTTRMSQDLGLNPNDLITQPLPSEGPGIPKHVEEYTKLLARCFLKQGEWQIALNSNWRSETSEIILGAYLLATHFDNKWYKAWHNWALANFEVISLYTSQNTSANNKIEILQDERNGSTEDGHSELKRAEQQKQQQQQQQQQQQQANIIPIEAVQRHVIPSIKGFFHSIALSNSNSLQDMLRLLTLWFKFGGIPEAAKAMTEGFNMVKIDNWLEVVPQLISRIHQPNEIVSRSLFALLTDLGKAHPQALVYPLTVAITSESTSRKKAAQSIIEKMRVHSPSLVDQAELVSRELIRVAVLWHEQWHDALEDASRFFFGEHNTEKMFETLEPLHQMLQKGPETMREQAFANAFGRELTDAYEWVLNFRRTKDITNLNQAWDIYYNVFRRVSKQVQSLASLELQYVSPDLKHAQDLELAVPGTYQAGKPVIRIIKFDPTFSIISSKQRPRKLSCRGSDGKDYQYALKGHEDIRQDNLVMQLFGLVNTLLVNDPECFKRHLDIQQYPAIPLSPKVGLLGWVPNSDTFHVLIKGYRESRSIMLNIEHRLLLQMAPDYDFLTLLQKVEVFTSAMDNCKGQDLYKVLWLKSKSSEAWLDRRTTYTRSLAVMSMVGYILGLGDRHPSNLMLDRITGKVIHIDFGDCFEAAILREKYPERVPFRLTRMLNYAMEVSGIEGSFRITCEHVMRVLRDNKESLMAILEAFAYDPLINWGFDFPTKALAESTGIRVPQVNTAELLRRGQIDEKEAVRLQKQNELEIRNARAALVLKRITDKLTGNDIKRLRGLDVPTQVDKLIQQATSVENLCQHYIGWCSFW

>3523

MEGRFDEVCEIVLKHREHRSALVQRAVIVLMPALASYYKDQFIKSYLKTCMAHLISNLKKNTDRAVAFQSIGEMALAVGEHIRPYLDSILQPIKESLSVKGRKGPQERTVYTCISMLSRAVGPELEPHLQDLLEPLFGSGLNKPLTDCLIDLAGNIPTLLPAIQDRLLDNISYTLARQTFKNPGVPKLNVMRGTMPRGGGGAVAVAIDKADGTIPDAATIALALHTLGAFDFSGHVLTQFVKDCVVKYLEDENAGVRKEAALTCAQLLVRSNNAAALPRTTSLGANYRQATPRPMSTHGTSSIAVTPVEKISQAPKSIHSVVVIGEVLEKLLIVGIADPDSDIRWSVLSSLDDRFDNYLAQAENLRALFVALNDEVFAIRELAIGIIGRLTVLNPAYVMPSLRKTLIQLLTELEYSGVSRNKEESARLLSNLISNAPRLIKPYVEPILRTLLSKLRDLSPGVSTNILAALGELAQVGAQDMVHHVNDLLPLIIETLLDQSSVAKREVALRTLGKLAASTGYVIEPYLRYPKLLRILLDLLKTEQVAPVRREVIKVLGILGALDPYRHRPDSRLPTIASTPSSSSTLNKTKVARDAGDMLDAVAIGAGGPGGAVGGNHGGGNLGGGGLVAGGGLGGTGAGAGGAATAQTQLPMLLLLLLLLLMIAAMCLTMSSVVSAFPAMCWTCRRRRKSTARQCRSRRLMRILKDLSLAVHHTMVVQAIMFICKSLGLRCVPFLPQIMPPMLQVMRTREPGFREFLFQQLGALVAIVKQHIRNYLPEIFVLIREYWDFSPAIQITILSLVESISLALGGEFKAFLPELIPHILRVFMRDDAEYRSTLKVLHALEVFGSNLDDYLHLVIPPVVTLFESFETRVDVRRTAIQTLTLLCRKLSFSDYASRIIHPLTRVLDGSPELRQDAMLALTALVHQLGADYIIFIPLVSSVLARHRIQHQKYDLLVSRILENVPLPEDFADVLIIESSATRPGMLSASGLPGMAGVAPNAGAGAAPGGAAGAANAGANAANAAGTGAAGAGGAGAGGAPGAGGAGSVANAGPVLDDPDAGTIKKLHVNQKNLKKMWEVTQKSTKEDWTEWIRRFSVELLKESPSPALRSCSALSQIYTPLARELFNAAFVSCWTELYEQYQEELVQALETALLSPHIPPEIQQTLLNLAEFMEHDDKTLPIDFKTLGEYASKCHAYAKALHYKEIEFYSAPNPQTIEALITINNLLQKPEAAAGILVYAQQNPDIEITVHESWYEKLQQWDQALASYEQKQKEDPLNFQLTLGRMRCLNALGDWDELHRLCQDKWQTADEGSRRALSSMAANAAWGLGRWESMDDYLVVMPRESPDAAFFAAVLALHRNNFPSAHHFITVTRDLLDTELTALAGESYNRAYGVVVRVQMLAELEEVMQYKLYADQPDRQQIIRHTWMKRLRGCQRNVEVWQRILKVRSLVIPPHEDKETWIKFASLCRKSSKMMLGHKTLCNLLATSATIPLSASSLQHLAAASPEQLLHHVDSRKMFLSAHPRVSLAVLKYLWHTANKKEALEKLRECVGVWQVEHHQHRAQLQQQLQQQLFQVQALQHAQAQMHPQQGGSQAPNSASSMSDGMTPTSSAPGSVASGHQFQQHLQQQQQQVQHNLPLALQQQHLVQLHQQHQQQQQQHNITANRMSNMSFMSNLNELGNSGSQLPMAIPSSPSSRGPKSPGSDLTSGIVTMTVPDQAKLLARCYIKLGEWEMALMDSLNERAITKILDSYLLATTYDDTWYKAWHSWALMNFEAVSHFEKSNANQKQVRAHIAPALTGFFRSIALMQGNSLQDTLRLLTLWFKYGSHQEVHDAMVEGIKTVSIDTWLQVIPQLIARIHTPSPLVGRLISQLLISIGKEHPQALIYPVTVASKSQSPARRDAAYAIMDNIRNHAGTLVDQALLVSQELIRVAILWHEMWHEHLEEASRLYFGERNIEGMLAKLEPLHQTLERGPETLREISFHQAYGQDLMMARDWTRKFQRSGVIKDLNSAWDLYYHVFRKISKQLPQLTSLELQYVSPKLLEANDLDIAVPGTYRPGEPINRIRAFGPQLTVITSKQRPRKLAIQGSDGRDCTYLLKGHEDLRQDERVMQLFGLVNTLLGHDPETFKLRLGIQRMEVIPLGPNSGLIGWLSHCDTLHALIRDYRESRKILLNIEHRLMLQMAPDYDSLTLIQKVEVFKYALDNTTGQDLYKVLWLKSPNSEAWLERRTNYTRSLAVMSMVGYILGLGDRHPSNLMLDRFSGNVIHIDFGDCFEIAMHREKFPERIPFRLTRMLVNAMEVSGIEGNYRVTCENVMRVLRENKESLMAVLEAFVYDPLINWRLIEGNVKNKKNKTTPSKAQTGNLDNGSEIMEDAIPFSVKRARSESELLESTDGLMSSQPEMVNNRAVTVINRVQAKLTGRDFSATETLETATQVQKLISQAQSHENLCQCYVGWCPFW

>3524

MMLSGVGPVPTKPAFKAGGDTLSRHLEELCRSGAWERRHKDGDKALLEYIEAEARDLSVEAFGRLMTDVYQRIGNMLLKGNDITRRMGGVLAIDELIDVKLSGDDAAKTARLSGLLSRVLEESEDPVLSESASHTLGHLVRSGGAMTSDIVEKEIRRSLAWCDPRNEPNESRRLTALLVLTEAAESAPAVFNVHVKSFIDAVWFPLRDAKQHIREAAVRALKACLCLVEKRETRYRVQWYYKLHEQTMRGMKRDHRTGALPSPESIHGSLLALAELLQHTGEFMLARYKEVVENVFRYKDSKEKNIRRAVIHLLPRMAAFSPERFASEYLARAIAFLLIVLKNPPERGAAFAALADMAAALARVNCAAGVSEVLVQALTQVANALPELLEDIQYQLLDLLSLVLSKRPFNSSTTQPKFAALSAAIAAGELQGNALTKLALQTLGTFDLGGIQLLEFMRDHILAYTDDPDKEIRQAAADCLRALFVGMNDESVAVRGLAIRLVGRLAERNPAHVNPALRKHLLQLLHDMEFSPDNRAREESAFLLEVLITAAARLIMPYVSPIQKALAKAAALQRDLVNENNVIKTVLSTLGALAEVSGTTFRPFISEVMPLVIEAIQDNSDGRRRVVAVKTLGFIVSSCGNVMGPYLEYPQLLSVLLRMLHEGHPAQRREVIKVLGIIGPGDDGGPGGDLLPSSGLVTSSEDYYPTVAINALMRVLRDPALASQHLAVIRALAAIFRALQLSVVLPILLGVLRGGDEALREEILASLRALVGYVRQHMRRFLPDLTQLVHEFWPAAPRTCLALIADLGMALRDDIRCVLDGHSDEQLRRDALDTICAVAVCLGPEFAIFVPTIRKACQRHRLHHEWFDRLAGKSSHRVTKEDWAEWMRNFAVELLKESPSPALRACHGLAQVHPSMARELFAAGFVSCWAELEQGLQEQLVRSLEAALASPTIPPETVTALLNLAEFMEHDDKRLPLDTRTLGALAEKCHAFAKALHYKELEFQTSPQSAIEALIHINNQLRQPEAAVGVLAYAQKHLHMELKEGWYEKLCRWDEALDAYERRLLKEAPGSMEYHTLLRYNPMNITQAGNPGYGAGSGAPHVMLAFLKHLWTQGNRTEAYNRIKDLEASRLYFGESNVEGMLNTLLPLHEMLEKAGPTTLKEIAFVQSYGRELSEAYEWLMKYKASRKEAELHQAWDLYYHVFKRINKQLHSLTTLELQYVSPALVRAQKLDNPGVIAENLASFRAATEHAPNWAKAWHQWALFNVAVSAHYSVALGQAAGDRTGNLQDILRLLTLWFNFGAYAEVRAALTEGFQLVSIDTWLLVIPQIIARIHTHNTDVRQLIHHLLVKIGRHHPQALMYPLLVATKSQSPARRQAAYSVLECIRQHSAALVEQAQLGLELAVPGTYIAGEPLVTIAAFAPQLHVISSKQRPRKLTIHGGDGAEYMFLLKGHEDLRQDERVMQLFGLVNTMLAHDRITAERDLSIARYAVIPLSPNSGLIGWVPNCDTLHALIREYRCKVEVFEYALDSTSGEDLHKSRNSEVWLDRRTNYTRSAAVMSMVGYILGLGDRHPSNLMLDRYSGKLLHIDFGDCFEASMNREKFPEKVPFRLTRMMIKAMEVSGIEGNFRTTCENVMRVLRSNKESVTAMLEAFTREKELKEAFVNLGDANEVGRLINMAVNHENLCQSYIGWCPFW

>3525

MGSTGVVAGMLERRREAEHHLQDYVEAEARDLSGEPFTRFMNQIYKRIYALISRCASLNERLGGVLAIDELIDVKLLGEDAAKTSYFASYLREVFQPSTDPMTMEVAASTLGHLVKSGGALTADIVDMHHAQVRRSIEWLGDRGEARRYAAALILRELAENAPAVFNVHVRAFIDAIWSGLRDAKLLVREASVAALRACLVLVEKRETRYRVQWYYRLFEETQRGLTRYPNSVDHIHGSLLALGELLRHTGEFMLARYREVVEIVLHFRESKEKLIRRAVISLIPRLAAFAPERFAATYLSTCTDYLINVVKTPNKRGPGFVAIGDMALALASAGCAARMEPVLPRLAEQLREAIAAPRSRRGVNCPEALQCVGVLAEALRGAWKPYATALIEPMILMGLSPTLVLALQELVAALPELLGHIQGQLLDLLSLVLARRLYREGLPQLHLNALHQAVAHGELQGAALTRLALQTLVSFDFGSARLLDFVRDDVTPYLDEADVSVRRAAAAAAAAVLHRTASAAPGSRAAIHPQAHTLAVEAVVSRLLMAACADTSVSVRRTVLQSVQAPSPLDTYLAQADSLRALFVALNDESGAVRALAVGVAGRLASLNPAYIMPALRKHLMQLLSDMELSPDSKQREGSAHLLGRLINACPRLVLPYVSPILKALVAKLRLAPHLQLTAAGPSGATTKATLLARVAGPGLRVHIGEVLPLIIEAIQDTSGGNKRLVAVSTLGQVVESTGLVMSPYLEYPQLLGVLLRMLNEGEGSQHVRREVLKVLGIIGALDPHTHKLNQADLQGEGKLEREGVRPQRQLGPDGVLPGETLGALSFGFMMREEFAHAGAMGGDELTGDLLSSTGLVTSSEEYYPTVAINALMRTLRDPAMSSHHPQVVRSLFYIFQALQLQAVPYLPKVMSVLLGVLRNADEALSEFLVQQVTALVALMKAHMRKWLPDILQLIADFWSPTSPLLPHLLRLLSELAVALRDDIRAYLPGLLPKFVALFGEAERGGGYELVRPALACLEALGTALEDHLALLLPALVRLIHPSVAGTPLEIRRAALHSMRRLLPRMPLAGSSAAVLHPLLRVLDGSVEELRRDAADTICSVAIALGPDFALFVPTISRAMARHKMVHEKFARLAERVTLQEPPCMSEADDWESNNRWIDDLVPAADATSSLPALPDAASESKLPVNEAGLRRAWESSQRSTKEDWAEWMRHFSVELLKESPSPALRATHSLAQVQPAMARDLFPAGFVSCWAELEEGMQEALVRSLEAALASPSIPKDIVTTLLNLAEFMEHDEKSLPLDTRTLGALAEKCHAFAKALHYKEVEFAQTPETAVESLISINNQLRQPEAAIGILAVAQNDLHMELKESWYEKLNRWEEALEAYERKYQNTAGTPAYVDAALGRLRRDTFPSCDVGKMILCLGALAEWERLSEACRFEWRQLEPHLRPRMAYLGANAAWHMGQWDEMSTYVAALESEEGPELSTGSFMSAVLNVRHGNYAAAKSCIERSRELLGQELAALVGESYERAYQNMVRVQQLTELEETIDYSLALTLAQGDEVAAEARRALTRQMWRERLNGVQRNHEVWQSLLSVRSLVLPMHEDTHTWLKFASLCRKSGRPKQAYRTLRSLLGYDPTAIPAGQSGYGAGSGAPDLMFGFLKHLWATHSRHDALHRMEDLEHEVTVSPLPTAEEAAVAAQSIVVGGRTGPAMAILFATPAGRMAGGYRASLVARVHLRLGMWRWSISEASKEVTEDVIEAVKSNLHIAIQAAPDWAKAWHNWGLFNVNAMDHYSRSDVATANRHVAPAVAAFFKSVSLSQASGGEGSRGSNLQDILRLLTLWFTHGAAPDVEKALEEGFGHVSIDTWLVVIPQVIARIHDQQVVVRRLICSLLICIGRHHPQALMYPLLVASKSQSGNRRSAATSIIDNVRQHSATLVEQAQLVSTELIRMAILWHEMWHEALEEASRQYFGESNVEAMLATLMPLHEMMAQQGPTTLKEIAFVQAYGRELAEAYEWCQKYRMSRREAELHQAWDLYYHVFKRINKQLPSLTVLELQYVAPALVRAQGLELAVPGTYIAGEPVVTIAAFAPQLHVITSKQRPRKLTIHGSDGAEYMFLLKGHEDLRQDERVMQLFGLVNTMLAHDRTTAERDLSIARYAVIPLSPNSGLIGWVPNTDTLHALIREYRDARKIPLNVEHRLMLGMAPDYDHLTVIQKVEVFEHALDSTSGEDLHKVLWLKSRSSEVWLERRTHYTRSTAVMSMVGYLLGLGDRHPSNLMLDRYSGKLLHIDFGDCFEASMNREKFPERVPFRLTRMMVKAMEVSGVEGNFRSTCESVMRVLRSNKDSVMAMLEAFVYDPLINWRLVKTGEGETDNALAAMNSEMLPSNVTFTGKLEDVGSAEMPSPPRRETREREMLTAYGQLGDAVEVINERAVAVMKRMSDKLTGRDFVQTLLQEGLPGSTESDSIQSQVQRLIVQAYSHENLCQSYIGWCPFW

>3526

MNAMRQHSPALVEQALLVSQELIRVAILWHEMWHEGLEEASRLYFGDHNVEAMLQTLAPLHHLMDKGPETMREMSFQQRSAFGRDLIEALDWCKKYMRNGKDSDLAQCHSSATFELTSDSHATAKAWDLYYHVFRRINKQLPQLTTLDLQLVSPKLFASRNLELAVPTYRANEPVLSVISSKQRPRKLTVQGSDGQDYVFLLKGHEDLRQDERVMQLFGL

>3527

MSTTSSSSTGAVSASRAVASPLMNGKSGLADAAPVLTASRSTPPPPPPAAAAPGTSTRARAASQGAHASERWGAANMTSLPALASGSSQLLAGSTHALVALHDASIERALRRLERATGSERRYEAAQRLRMLYERKFQTMKQPEAISVLSDALAYRLQAMISSGSQRDRLAGLTGLSALIETRGEAYRTKVQRTYNGLRPILRRPPDWEAARAAADMLGRLSRLGGVLVNRFVEAEAVRALEYLNTNSVLLKGMAIMVLNALCEHVPASMYTSRVQLAQVVWRGLTDSRPILRLESASLLRSFLALLEQRASDDSARTCQMIIQRCLEMLISDERETTPPETHGSLLALHELVRNRFSATFLDEHISRIRKRLESLRLTRDNLIRAAILQLLPDLVRLDRTLIELGMSMALENEDLVALGKLAVAVGPTAALPWMEHIFVVLRNSEVAAAAKHECIALLAPIIVESEQSERLAELLEDSILTQGLSPSLVEALERVCEHVPSLRDLVHGHVLRLCAETTDIACALQALQRFTFGRRPCLDDYVRQHVLPYIYGKRSLRSAAFAASLHVLTAAVQSATPGRPLQPKIFETLQQLIVVVVADPDSRIRAEVLLHLAKPAYFPVFERYLAQPELLRALCLALHDEDATVRERALSLVGRLSTVNPAHTLPSLRRLFMNLRIILECESEPLQGSQEDALRLLLRLVQDAPQLIEPYTPTTAKLLVQRLQQNSTSAVAVDTSVIHTLHSIACLAERGVAAELQTLLHDAMPHLLRILQHTSVEADLREAVCRALSSLARAAGATTELYTQYPSLLSTLLRMLRTETNLFVRLEIERTLGTLGAVDPDRTAPLLLDRTRYPYTRLESLTEVYHQQTAPMHYAPRPTATGTGATNAFTTDTRCGLVAEEPALPGTAAAAAAATTTTTTTTLTGQALRAQLRFEAPNELETLVGRLPHPFTANEEYFPSAALDALHRILANAKLTTLHYDTVGAIVNIMSSLGMKCAPFLSVVVPRLLWMLRPRTEDTHDLAFREYVIRGLASVVLSARQYIRPYAASLVALILEYWPLQVLHRSMFALMECLRVALHDEFRPFVPVLLPLILPWMSLRVLKVLLVFGTHTSEYVVLVLPPVMRFLEDATRPLAARIEVLKRLPRLFAMLDLSDMASQLMHPLCRMLRTSEFQWHARQLLESLLPRLGLDAAYYTLLVREMLLGDAASASAAATGNDDEDDDGNDDEDDDQHPTDEASARGAAASTESASTQAGAALQSSSASALHRLARYRHERTALSRSESLDSLNGVPRTTSLGSLADLASGDALLAHSPDSLLDSTHVSLVASASMLDLQQQLQQQQQQQALLGSGASNTTTTNTNNNNNSGHNSARRHHVNEKMLQKAWQVGRRTTRDDWNDWMMALASALFRESGSPSLRSCARLAEVHPPLARELFNAAFLSCWTELSDPCRASLVRNLVLALSSESIPLDVLQVLLSLVEFMEHDEKPLPIDLRQLAAMAFRCGAYAKALRYKEAEYMQNPASAMEGDDSLIAIYEALGQREAAMGALLDAERHQVTIRHEACYERLQQWDLALSAYERQGPAHSFANRRGRMRCMAQLGELHRMEALCNDLWSEAAEQPSLRQELAEEAAQVAYQLQLWDKFTERVAYTSRDSIRGNVFRAMLAIHQGDDEAARGYIRAGRRLLDTGVTARVGEGYPRAYGDILLTQQLVELEECLMVRQRILPQRHVVEQLWNTRLYGCRFDYTTWQQTLLVRRLLLEPRHDKDVWLRFVSLCRRANRLPMANEALAMLQDEQHPDPEVTYASLKLCWSQGRHHEAYECLRRCAEQPVPSARLAARRFLKLCVWGRALRAEGTSTFVSSPSSSLRWHTLMEHARRAVVSDPSWSSGWHTWAALNADAAAAHSTGHRFGGAWPARPTAVAAAESNVLVRAYVLNAVNGFFRAIALGESYAEQAQDVLKLLTLWFRYGAVPEIEQALLNGFAETNVDIWVDVIPQIVARLHSPVPPVQAGVRALLIRIGRAHPQALVFPLAVAAKSSNARRREAAIDVLQALRLESPALVAQAELVSRELVRIAVLWPEMWHEALEEASRVYFGEHNVPGMLAILSPLHDLVEAGPSTAREASFIREFGRELAEAREWCRRYLASGRDSDLNQAWELYYSIFRRINKSLSSMTQLQLAEVSPALLEARNLALAVPGTREQVTIVSFAPVLNVISSKQRPRTLTIYGSDGHEYPFLLKGHEDLRQDERVQQFFGLVNTLLPPEMSIVTYAVLPLSQQVGLIGWVKNCDTLHALIREYREQRKIILNVEHRIMLSAAPDYDNLTLLQKVEIFEYVLANTSGNDLANIMWLRSRSAEMWLERRTNYARSLAVMSMVGYIIGLGDRHPSNMLITRDTGKVIHIDHGDCFETAMHREKYPEKVPFRLTRMLVRALGVSGVEGIFRVTCESTMEILRANKPVLMAMLEIFVHDPLLVRTLQPAAAPGATTTGLEATGLAAVAAGSPLHTPAVWPRQATGATAAATAATATAATATAAAATGQWGTVSTFPVQEARLGERAGEEQPQPASTLVQVPRAPTRMRRVDMSGAEATAAAAAAGEASLSLRLLQGTMSLRAALRLEQSNAGTVEEVAEEGIAETPSPLTTRRPGAGSLTPRTLGNGTLTTGAGAGAGAPTILPEDLLGDENRGERAVAIVQRMSQKLSGRDFDPRRVLTVAEQVERLIQQATNSENLAPAYVGWCNCW

>3528

MSQHEQPSIWADALTKLLNDLKSKKEEDRVKASKNLKSYVISQSREMTNENFTKFMNELNNNLIFELVNSSVIPEKIGGIMAIDELIDVDYDENATKITRLANYLRIGLGFNDFTVMLMASKALGRLARSSGTLTAEFVEFEVTRALEWLSGDRIEARRHAAVLVLKELAQNAPTLFYVHASSFVDLIWVALKDPKVAIREGAVEALRACLELISERESRLRLQWYQKIYDEAQKSFKQNGSPESIHGSLITVSELLRNTGDFMLSKFKDICETVLKYKDHRDKLVKKTVLALFPRLAVFCSRDFVLNYFNACMNHLLAALRNQNERPTAFIALGEIAMAVGGSIKPYLDSIVVMIKQGLMTKGKQFCPEVLTCISMLASAVGQSMYPHMQVILPQMIVSSGLTVVLTDALRDLTINLPTLIPNIQYKLLNLISQVLANKPFSEPGAPSPYRKSATPFQGGSIPQLGQNSDVDPQMIALALKTLGSFDFSKHNLLEFVRECVVNYLDDDNIEIRREAAITCAQLMVGTEEPTPTRGHSAVIVGEVLEKLLVVGIADPDPSIRKTVLSSLEARFDHYLAQAENLRSLFIALNDELFEIRELAITVIGRLTIRNPAYVMPSLRKTLIQLLTELEFSGDGRNKEESARLLGHLISASEKLIKPYVEPILKALLPKLRDSNPRVASCVLAALGELSVVGGEEMVQHIDSLLPLIIDTLQDQSSTSKREVALKTLAQLASSTGYVIKPFSKYPMLLDTLLNAIKTERIGSIRREVIKVLGILGSLDPYKHKMNELGKRREDPKANDDKNNNMTNEVITISPSNEDYYPTVALTALMKILRDPSLSSHHTSVIQAVMYIFKSLSLKSIPFLPQIMPPFLHAMNTGEPLFREFLFQQLGSLVSIVKQHIRDYLVNVFALIEKYWNSNLLIPIIKLVEEISSALNDEFKVYLPNLIPQMLNVLHTDRSPKRSPTTKVLRALEVFGTNLDDYLHLVIPAIVKLFEQVDVTTQVRTLAIQTIGRLCKKLNFSDYASRIIHPLARVLDSTESELREETLNTLCALVYQLGSDYAIFIPMVGKVLARREIQSTNYELLISKLLKNQQLMLTPGSGDDGGMGANRFGGDHNGHHLGEDHNNTSTPLDIGVKKLKANEQHLKNAWETSQRSTKEDWGEWIRRFSVELLRESPSPALRSCLSLAQDYHPLVKELFNAGFVSCWTELHEQFQEELVRSLETALLSPNIPPEILQTLLNLAEFMELHEKPLPIDIRTLGALAEKCHAYAKALHYKESEFSQSPSSTIEALISINNQLQQPEAAIGILIYAQKNHSVELKEGWYEKLRRWEDALAAYEKKQKDDPNGGTIENTMGILRCLHALGEWERLSALSSETWKSDINDHTRATIAPLAAAAAWNLVNWDKMDEYVCAMNKDTVEGSFYRAILEVHHDNFTLAHGFIDHARTLVDTELTALLGESYNRAYKVVVRLQQLSELEEIIEYKKCVDSPERRNMIKNTWKTRLRGCQHNVDIWQSILAVHSLVISPHEELDMWLKFVGLCRKGSRLGLAQKTLTMLMGKDPSTTSQFGSVLPNTHPRITFAYIKQLWSAGAKQPAFEKLRTFVQALRDTDDLPLQGRAYLKLGEWQLALGDTLSEASIPHIISSFKAATECDPNWYKAWHSWALINFEVVSHYEQNGGTPEQIGAHLLPAVHSFFKSISLGPDRSLQDTLRLLTLWFKHGAQKEVEAALMQGFNTISIDTWLHVIPQIIARIHAPVLPVRRLLHELIDTIGKEHPQALVYPLTVATKSHSPARLAAAKSLMDKMRKHSATLVDQALPVSQELVRTAILWLEMWYEGLEEASRQYFGDHNPEAMLATLAPLHQILEKGPETTSETSFLQAFGRDLQEALEWSKKYEKTRKEGDLNQAWDLYYQVFRRIYKQLPQMSSLELQYVSPKLLNSNNMELAVPGTYKASENVIRIQSFSQALSVIPSKQRPRKLTIIGSDGLEYTFLLKGHEDLRQDERVMQLFSLVNNLLSANHETAKSHLSIRRFSVIPLSPNSGLIGWVPHSDTLHTLIKDFRDSNKILLSIEHRLMLQMCSDYDNLTLLQKVEVFQYALENSNGLDLHKVLWLKSRNSEVWLDRRTNYTRSLAVMSMVGYILGLGDRHPSNLMLDRHTGHILHIDFGDCFEVAMHRDKYPEKIPFRLTRMLINAMEVSGIEGNFRLTCEAVMNVLRNNKESLMAVLEAFVHDPLINWRLLTPNENNTKHKATNIASNNSTSNSTTKIEGDLNTIDNPINKESPDHEAVAGSLKSSPVHGRQIARNQRVGVDAEQVEAEIVPEALNERALSVINRVNKKLTGRDFSSNETLDVPEQVQKLIDQATSHENLCLSYVGWCPFW

>3529

MAVAGGFRTFLFRQVIVLIYAAGPRLPPWAVSRLVQIVHQDSLEGYLQLLLPVLLRMIERRDVGLAMRHITLAVRALFEAMDTTGCTKRGDWTEWMRVVSVELLRQSPSRVLRPCAALAEAHQPVAQDLFNAAFLTVWDELFMENWEGDNTHSPVIEAIQSALSSPSLPPEIQTQLLNLAGFMELQDKSVTSVMESDFGFAAGGVGWGAAWEGVPAAVPPGGGESVAGSESGAGGPGSVSGGGGRSCSGSGPNPDTLEALISVNHKLGLDMAAAGILRQAEQQAEAGLCEFVVRPSWLEKLWRPTVMSRPAAGRQGSGDSLLGGAGGGESSAAAAAAAAAVVAAVTAAAVVAGPSPAVSRSSSAGLIPGVGPAAAAAVKGGAAGGDEHTPAFDPDRMLLGSFNSDEAFAEVGPAGAPASSAAAAAAVGMGGAGAWSGGFDGGLSTTDVVVEWYQVMLGRLRCLDALGEHRKEDWARVGVGREPAGGFSTVAAGARGPRAPKMLPPPALATKAATKAAAAASATATTATTVAALTTMTVPAALLSTTAYPSLASRSTDSMDLGGGAAAAASLASAEVMGSRAAWALGEWPALEMFVRGEHMQERRHVVEEGQGVEACLVLEAVVATQKGRLDEALTLIEEARQALAPGLAALLSESYTRSYKRMLTIMSLAELEEVVEYKRVVKDARSLGTPPVKGEGMEGSERSRRWSEVAEHRSNLRAKWTARLQWVPEDVDVWRGILAVRSLVLKPREDLGTWLKLASLARKTGRPELCANTLRLLGAQPPRPDEPSGPLTYGRSTASSGGGTGSSRPRSESSSSVGGGDNSNNNNNNNNNNNNASSSGFAATSASMSSFSSMLSSAAAQQQENLSLSMHSSRLLTSELTVPSDAAAAASGGGGGGGGGGVSTRTAAVASSPPPHPRVVYHMYKYMWATGDQERALGRLERFTSTLMLRLRRGHSMHGVTPPAGSKEAAGHGLRSLLVKCLLQACEWRLEMREMKDSDEQADGQGGGSGGSGGGGGGGLNPDGNVIPESLVNTLSWLRRAIELDPTSYDAWHAWALMNYQLTEEENARRKEIDEREREEKERGGGGEDDDAKREQAQSSTAAAAPSSSPGSDLPPPTNTTATTTISSSALSGKFKIGKKAVRWLPKWPSLSKAQQGADPPPQYRDYPSQSSLMRDASPIRSGLERSRGSVGDMSGADTLRLLTLWFAHGGFESVHREMSLGIQACGVDTWLGVVPQLIARVHAASPRVTKLLRELLVRIGRKHPQALIYPITVASKNSSRPRQEAAGAVMADMRKQYPVLVEEASLVSRAMIKVAMTWPEVWHEGLEEASRMYFGDGNVEGMLRRLQMLHDLLPWETPPASAVYSGGGGGGAGTTGGAGGGGACGDLSSSSANAAAAASGGGGGEGTSSTSSPGSDDGSASTDGPAADTVRGVAFLHLHGRDLAEAWEWVQRYQACRREADILQAWDIYYHVFRKISRQLTTLKYIELRHVAPALPQANGLQLAVPGTYRAHADVVRIRSFAPTVEVITWSKQRPRRMTIHGGDGVPYLFLLKGREDLRQDERVMQLFGLVNALLASERKTARFNLSIQRFAILPLSNNSGLIGWVPSCDTMHQLIKHYRESKEMRLNMEYKIMTSLAPDFDKLPPLNKLEVFERALAQTDGQDLARMLWLKSQSAEAWLDRRTNYSQSLSVMCMAGYILGLGDRHMSNIMVDRVSGRVVHIDFGDCFEVAMQRDKFPEKVPFRLTRMLVNAMEVSRIEGVFRSTCEMVMAVLRDHKDSLMAMLEAFVHDPLISWRLLAQPRMSASPLTDEDGEDSDDDDEATHDPTADGSEVGGHTGASRSSQYSDGGTTVRSVGSTATGGERAASGSTTSRARGRRRRRRDGRGSFGGMGMSKEPDPLAEVDRLIQLASNNENLCQLFVGWCPFW

>3530

MDPPSWDPGTTYVFDLITDEKLLAWESCLTVLRQETDRSARADAAERLTVCVERLARELSTESFEKFELQLHQRVVDMLAEPELAKKLGAVAAIYALVGTASMQAEATKKKFSLHLSYTLETSKNYELLNEVAKALGYMARTSISSSSEYVENEISQALQWLKEENWHRKLAACLVLRELAQKAPSWVYGSMHDIIPHIVKAVHGDIEKVQEKKMSRKRYSYHKRYIVFQQLREGFSKPTEANVHGSILMAGPMLEHGGNFMMPRFDEICAAVMGLKDNRSRCVKLSVTTLLPQLALYSPLDFSRKYLIDTLEYLYASCQSRCDQSASAYKALGKLTLALGVADLRKEVVALVEVVLIGDSDRDRDKEKGRGKVSCCPAAMECLANITQALGELLAPFMPKLLGPLFSNGLSERMIATLRVLSETLPSHTIQVQEDVKRLQEEENLRVLALHTLGAFKLDAFVLLPFVREILPELVKGPPGKGPKQGGVNAWQGPGEPGKTQFPRGPSPQGNRDTDGKDSWSKTSCPSGGIRVKAMFDIRLDALALLGRLAQFNPAYLLAPLSNVLKHIVVALKYGSEDIIKEQSTRMLCTFLKAPALKYLVHPYVKKVIEILPLKGHPRLATAALEALGWLSLSASELMLPFMDKLIPFMITSIQDSTNVNGREVSLRTLGRIVSATGYVIKPYLRYPTLLDSAFSILRARGNTRWSLRCEVLRTLGILGALDPYRYGQILLYLRAQRLQAEAVAAGASTSRGDISPSATGNAAGGDGDGLAARRRKKNGGGNAFAGGVGSNVPAYPRAGNLSSVGMGQPPGGGNEGMPAGSSMSVALDGLFEFDFHGNAAVNGTNGQPWAGHGLLEGDGGQGAAHDTMLSQTHDDDQAAHSVMWEQSFMSAQPNPIMEPTTLTVLQDMYNEDVAITNLMFMVAPAVMRIFASIGHRSVQFLGAILPTMFFVISRCEAGLRSSLLIQMQMLVRMLKKAMTPYLEAILTLAAQCWVTRLDPNYKEVTKGVGGLPDVHQPSINCTPLERRADKRLAMVLRAVLVMRPLLNQQLSIVLPALVNLVGVMSETHPERDDLEWHLACLRTIRGLTSGGALSHVPALAANLVHAFVKLLSVTGGGGGGGTDALAEDGGRHPAAAAAGKKMDSSSGGISLPQHLWGFIGGASLVAPWDLGVGGRSNSIRDDGSAARRERRGGVQRRRGESLGAMEDHNRNRRNSTGSRTGIGRGVGVGGDLAAKEEMARQQRRRLDSLQDAGMEVLTSVATQLGNRFFMFEGMADNFLDGAGASEQAARYRQLVKRLKADAAASTVTMAPPHGANPPTWGSANPSPNPSPGRGHDYGIRPAYTLPPNGGKSWMSGIPPPAATAGPPHQQYLSRGASQGGYGGSWGGEGGGGGGGSSGDDRSGGGGGGNDGDGGSDGGGEGSAYRPLSIALFHAAFVGCWLELSEDAQSHLVRSLKQVMRNQNVPPDILQTLLNLAEFMDHEVEGEALPIDIRLLSNLAERCHAYAKALHYKELEFATNPAICIESLIAINKKVGQPEAAMGILTYAQNRLGGEVSVEDDWLAKLGHWDEAIILYRQRQEADPTDTGAILGCMKCMDAMGEWGDLVSLCNSSWDHIHTVGGDAAVARKAATMAARATWSMGDWAHFEQFVGFTEENVVEGAYLRAVLALRKEDLEQCTRFVNHARQLLDNTFTTLIGESYKRAYNSMVMVQQLAELEEIVDIKRSVSALSKTSTFGPSGSHAPGGPAGQTPGTKGGGPNAQKLWKRLRKTWKQKLGVSVMQRILMLRGLVLTPEEDIEAWLQFASLCRVKKNYRIAKKVLDAPVGLASGNESYDNDDGLGVGGDLTGELRQQLYQPTRRSLPIKHKLMFAWVKQRKAQNKMGEALKDLSRLAAELSEEENSLKVKCLLKMGNWELSQVAPSKPLPVPVRDRVFTAFKKATELEEDNYKAWHHWAMVNFRVVELAMQEASMSRGGQRFRPGPGRGSGRLYGRQTEQERKVFTERLVSAARGFMRAIMLGKKKWSALVQQLIACMNHKDEVCRKALHKLLMRLGEKHPQALVNPLSVALKSPKEDRKAAAEKLMQHMVLNSNFLVKAREALLVSTELIRVAILWHEQWHEGLEEASRLYHGDHNIPAMLKFVEPLHKELEKGASTAKEDSFQKAYGKELSQAWASIKKYQAIMEPGHNPEGARLHGGALNHLNQAWNLYYDVFRKINKHLPQMSVLQLELVSPLLLKSRDLSLAVPGTYRVDNSCVRIHKFLPDVQVIPSKQRPRKITMQGEDGRDYVFLLKGHEDLRQDERVMQLFGQVNALLAKDRRNYSHDLSIQRYAVSPLSHNAGVVGWVPNTDTLHQLIRDYRDSRKIVLNIEHRLMTQATIPCEYDTLTLMQKVEVFEMSLQHTAGQDLYKVLWLKSRHSEAWLERRTCYTRSLAVMSMVGYILGLGDRHPSNLMLDRFTGKILHIDFGDCFEVAMNRGKFPEKIPFRLTRMLVNAMERGYYVVVRKLLPPTLDGLPPPPPGSGIQGNFPNTCELVMEGLPESRENLGTLLETFVDEPLISWRLLERKQEGAQPPGSNHHSTNTAGGAPSETPAPTDPVAAAVPPDGAQGQQQQQQQQQQQPLNAMPQQAYAMPPPQATAVPQQPPPTPRIGPMNPGQRGPGRAQHPQQQPYHHQQRQQAPQQRGSSLGPQLQQQPPLQQEQFQQPPGGSSNVGPVPPRQQQQQQHVGRLGRLLQETAGPDGAASGDPPPHPGGNAVGDPSASVRVALAPPAAGGGARDGWRTAAAPPQHVVATAAAAAGSGAGVEASGNQSGGGGGGGGGGGGDGGGSHTAASGRVSQDSYGQRYHEQQHPPQAVKQYASRVGVGGLMPPPPPPPPLPPPPSARAVSYRVGPSSLSGRPLTLQPVHELRSRYGRGGADSDTEVGSDVGTEENDETPRSPQSRTGSRAGEAGGGGRGGRGGRATDSGRDEAVRKEDPGGEWAGQRQAGYAMTGGGGGSGGGGSESVDEGGAREGLPPGGRPEELRSVPPTPSGPVVVAPGIGAAGVGGGRRVFSGIIEPPSPRNAVDGSDHGFDTSSPRQQDDANLPVLGTSNEGGIDEAAAASGGAWGTGGVPMDHGPPVGRDEEDGGEDVAGPLGGLRSPSADGTKRDGAVDGSNGNTKALPERQSAKAAVAAVGGGRDADRPRSSSVAIQSSSFRPHMHVRMQALSALRHESYLSSRAGLTVGPAGGGVDDLPAEMASIARSIAASRFSRSTRERDLQQELGPEGTSAPQEALNEKAVKVIRRVQDKLSGLEFGDGDEPPYPVKAQVDLLIKQATSNERLCQCFIGWCPFW

>3531

MRCHAALGEWEAVRRLSDKLAEQRAVLAPGEVAELARLGAAAALDLASHATAGNERHWAALGRHAALLPARSFDGAFSRAVLALHGGDWSGAQAYIDAARGVIDAEVTGLVGESYARAYNGMVRLQRLSELEEVLLNATSPTTLPRARLLELWRGRLGHAAADLSAWRELLPVRALAVPPRHDPHGMIAFAQLCSRNGQHTLAFEALRHAEPRAAASWGDAPDMQPDVWLAYTVAIGWVHLGEWTLASAAPAAGEASLLEHFRRAAELDPASCAAWHNLAMVHFDHVRCTCWRSEDELGEHHRHHPDPGATDTEERVVAALECLFRCISLRPSSSPTGHTQQDILTLLTLAFEHGETEGAAAALSRGLADTPAETWLAVVPQVIARLGSESERVRTFVLSLLSTLAERHPQGLVYPLTVAATSPLRAQATGAAHVLAHLRRGRDVLVEQAQLVAAELVRASCLWSEAWIDGLETASKAFYTEGDDAGCVRALLPLHEALERGAQTPAEGAFVREFGGALDAARAELRQWQESGERAHLHSAWALYMGLFRAVSPRQAALTSLDLASVSPRLLGATALELAVPGTYEPQAPLVTISGFRPRLSVIASKQRPRRVMLAGDDGREYSFLLKGREDLRQDERVMQLFGLVNRLLGSTAPLAGPAIRRYAVVPLSPASGLIGWVPSCDTLHQLVRHHREYRGGGELRAYNALPLLHRVEVFAAALSRTGGRDLAEALWLRSPSAEWWLERRSSYTRSVGVMCVVGYILGLGDRHLNNMLLDRVSGEVVHIDYGDCFEAAALRPRFPETVPFRLTRMLVTAMGVAGVDGVYRSTCEGAMRALRRHRSSVLAMLEAFLYDPLVHWNLDGAERGSAKRVSSRASLDGGEARGGAARGDKVMHAQALRVLRRVKSKLDGSEFGAAPIDVPTQVDRLVHEAQAHTNLAQMWGGWNPFW

>3532

MARYVAAMRPDTVETAFFKAVMATHDARLSEARAHIIAAQQLLQSELTALVGESYHRAYRATVQVQQLAELEEILLHKADPEAMPLPLLLSVWRGRLLQAERSADVWQEILSVRALVAPPARDPQTWLKFSNLCRKSGRGALGHKILSEILGEDWDAHWERMPRQAAHASVTQPTSPDHSPSTTASFLAGGAALLRSTGEMTAGGGLGGGLGGDRAYAGGTVDGAQRHAGTYGYLKQMWADGSREEAIARLATFARYEAAGTAFAAKAWRRLGGWQRAVGEDQPFSVERAATVLASLGNATDASPHSYKAWHAWAMVSFEAVQQLPPSDAAEFVDLLRLLTLWFTHGHRADVEAAVQAGCDDISADLWLLVTPQIIARIHAPNPFVRRSVNRLLAEVARTHPQGLIYPLTVASKSLLLPRRTAALRVLTEVRKANDTLVEQAALVSTELIRCSILWHEVWHAALEEASRLYFQASDVDGMLAALAPLHAQISRGPVTTEENDFLRTYGAELRAAHEHCERYRLGGRSRQELQSAWELYSTVFGWLTKQIGRMTSIELAHASPRLLQARDLELAVPGTYEATAPVVRIGSFARSMSVIGSKQRPRKLSVYGDDGSLHDFLLKGHEDLRQDERVMQLFGLVNALLASTDDGARLDLGIQRYSVVPLSPNSGLISWVAACDTLHALIKAYRDGRKAMPLNVEHRLMAFEHALAQTPGDDVARVLWLSSSNAEDWLLRRTNYTRSLAVMSMVGYILGLGDRHPSNVMLDRLTSKILHADSGACFEVRERRLLTPPAASRHIDFGDCFEVAVQREKFPEKIPFRLTRMLVAAMEVSGVEGTFCSASGGMRGTRTFRATCIAAMGVLRRNKDSAFVHDPLINWRLLIRQAVQHEALEEAPTSGGASPQADPPPDQQALLRTVSESSRSFPTQAGRPTERRRSPLGARAGGATSPFGSRASASAEAPRRSGGPVMRVGSLRRSVVLTTTSDRLQQRHRERARSSGLGEGIATIELESSDPALSHQPISRSVVSVATMMHESIAHDGGGNQDEDALNARARAVLRRVRDKLVGEDFAPEPLNVPTQVERLIAEARSNSNLCQLYVGWCAFW

>3533

MLKCVGELEHTDGKVRKAAVQTLGKLPAEALAAHGAAVAARLEDANEYVRRAAVETLGKLPAEALAAHGAAAAVAVLGKLPVEALAAHGAAVVAARLALTRGAACSTISFCTVGLPGALAKHGQIFYEITLKRVGKYPQIGRAPLARAAAPAVRSLAAALAMVVSAARDLDVSLQQLGHRSASVRQGAARRVRTMFERAGSALEILRRLSEALGSNDPARRHGGLALVNELVDVNEWSRVGTGAARFCRLLRAVLERSTASGTARKAHRRSAPDEIALAVRTLGHALRSAGAAATDATDAELSRALEWLHAETPAAASRSHRRLSSCLIVTELAMRSGPQAASADPLSETRIAAIGMLRSCLAIVSQREGGLGAWYEAALSRSLAGIQSYHPEAAAADSPSSSASSSAPRPIEEGGGSSGLDEAHGCLCALSELLSGELLSLALGPLHPPSPPLLDPPLLEPTFQLPAWRRSASASSAASILSPALLSPALLPPSAADAAPHQTLGAADAVSTHPSSASLPPPMPQCASLPRSAEHRAAFAEVERAFRLVWRLRDESPPHLRVALLAALPRLVAVAPPPLAEQLLPLVASHLLARAASHASTARAACLLREGSEKAVFKAIGDAWANFTRGFAPPPPPARGRRQRGGMDGRGGRGDGWRGEGWRGEGSPAAAALWCIGSVCEACGGAVGDASGELVERLCALPLSGALLACLAAIARHVPPLERQVRSRLLDAVSLALVREPFDVWEERCTQQQQQQPARGTRALRGDGSKGEAGRGEAGRGEAGGAEGGGWGGRGAVCGGGEPSEPAHGGRTEGGALSLVNLLPPAASPAVAPPLRAGVERAERVAQLSLALRCLSDMPVASRLLPSTLPRFMLAAMRECLDDSAVEVRTAAAVGACTVLAALAAEAETRAATAIGRPSPPPPVRTFLPRPALAASAAAPSCAAADGSVRCWPPADCEGGPPLPSLRLVVLGGCGERAGGGAACGRLLYHGAREPSDDAPLGQGLAGQGALPSVCERVELLEKVLVLAALEPARGARLATLQALSPPLDVHLQQPRLVELLLPYLEMGDYALCSAALAVLGRLAERNPGEVVPALRTFLLSLLTQMTHAHSPAARQQAARLLSRLSEARPARPQLLGSHATPIVLALTPLLGEADAGVATAALAALGELAGLAELAGAALTPYDQSSSEKRHAALRATWRLLRYTAPPPKGMQGHAGSLSQRSQLLHTLLGTLLHAEALDARRSERMAGGLSTSDAPLGLGGAAGGAASPLYYPAVALAALTRVLREPSLVAQHRATVPPIVTVLSELGTRATPLLPDVMPLLLAAASGPDPLLVEAVVRELGALVPALGLLSRPYVPPLLELVLAHLPGPHASIQAHCICLVEALCEALRAELTPHLEALLPRMLSILRTDRSDTRMPTLRVLHALGAMATSLGDAAPPVVGELLWLAQQRDAPMRPRRRAIKLLRLLFARLPLREHAAHTAHVMLALLGAADDAAELRQPALHCLHSLSDSLGDSMLSLSPNLASAVAAASASSPRATLSPGLGPHRAVGRLGALGASCRPSPLISRRSAPDDDSPLLEPAAPPRLDLTRGSAGRLDRLVDRSDSAVALPALGASPTASLDAGSFALPPAAPSVGTMAAAAAAATAALDGAEAGCEDGAGGSSTAAPAALELLRVSPAQPLRACLLPAQAYPPLAQELLNAAFLSCWSQLTPDGCGLLAAAVEAALERESMPPAVLQKMLNLADFMELHLQPLPIPVRKLGSLAERCQAYAKAAHYREMSFTHAAFRHAAVTQAPPGRDMGWTHAPGPRTAAAAGGGGGGGGGGAVGAGASGASAAAAAAAEEATATAADIESLISINNQLQLPEAAAGVLAFSRDLGVEPRVSWYEKLGRWSDALAAYDRMSQSAEGGAAGGSIAVRLGSMRCLHALGEWRRLCQLASEAWLDPALDVGTGELRAEAARLAAGGAWNLQQWGSMARYVAAMRPDTVETAFFKAVMATHDARLSEARAHIIAAQQLLQSELTALVGESYHRAYRATVQVQQLAELEEILLHKADPEAMPLPLLLSVWRGRLLQAERSADVWQEILSVRALVAPPARDPQTWLKFSNLCRKSGRGALGHKILSEILGEDWDAHWERMPRQAAHASVTQPTSPDHSPSTTASFLAGGAALLRSTGEMTSSAAAAAAAAASAHVVHADSASPPPSRGGGAGACGSGRASPPAYLRASSDGTLPTGTLPVGTLPGPPRLPPSIDGLSRTGGGLGGGLGGDRAYAGGTVDGAQRHAGTYGYLKQMWADGSREEAIARLATFARYEAAGTAFAAKAWRRLGGWQRAVGEDQPFSVERAATVLASLGNATDASPHSYKAWHAWAMVSFEAVQQLPPSDAAEFVVPAVRGFFRSIALGREAALQEPWTRSAEISRDQPHSPEMAGCDDISADLWLLVTPQIIARIHAPNPFVRRSVNRLLAEVARTHPQGLIYPLTVASKSLLLPRRTAALRVLTEVRKANDTLVEQAALVSTELIRCSILWHEVWHAALEEASRLYFQASDVDGMLAALAPLHAQISRGPVTTEENDFLRTYGAELRAAHEHCERYRLGGRSRQELQSAWELYSTVFGWLTKQIGRMTSIELAHASPRLLQARDLELAVPGTYEATAPVVRIGSFARSMSVIGSKQRPRKLSVYGDDGSLHDFLLKGHEDLRQDERVMQLFGLVNALLASTDDGARLDLGIQRYSVVPLSPNSGLISWVAACDTLHALIKAYRDGRKAMPLNVEHRLMAFEHALAQTPGDDVARVLWLSSSNAEDWLLRRTNYTRSLAVMSMVGYILGLGDRHPSNVMLDRLTSKILHADSGACFEVRERRLLTPPAASRHIDFGDCFEVAVQREKFPEKIPFRLTRMLVAAMEVSGVEGTFCSASGGMRGTRTFRATCIAAMGVLRRNKDSVMAMLEALARGKARPHPRHAFVHDPLINWRLLIRQAVQHEALEEAPTSGGASPQADPPPDQQALLRTVSESSRSFPTQAGRPTERRRSPLGARAGGAAGGGGGGGGGGGGGGGGGGGGGGGGGGGGGGGCGVGGGGGGGGGVGVSGERGAEGEAPRRSGGPVMRVGSLRRSVVLTTTSDRLQQRHRERARSSGLGEGIATIELESSDPALSHQPISRSVVSVAAVVATMMHESIAHDGGGNQDEDALNARARAVLRRVRDKLVGEDFAPEPLNVPTQVERLIAEARSNSNLCQLYVGWCAFW

>3534

MSSTDWQAKLNGFIDDLIECKTKEQRELKAEGLKSFFMTMSSEFHGEADSTFIELIKYCFKERLQNNTMTGEGPTIKVQMGMLRVIDIILKSDFGEMFCKEVCKDALNQLIKTKDKEVMKKTAKYIGKIIRYNLHDYIEQELKYISVSVGNNPKSGIGAISNSSNNSSYEELSVLAKVMVLREIIVNAPSTCYTSKKDIVSIITEGFKERDYETRKQANIALDEYLKNEKDKIRFEYLYASALNEFKRENLTESNEETNHGVVLMRITILKNFKESSDKINYYEVLSDLLYLILNCKSQTIRNSSILSLSDMCKIDPEKFSPKEAPIGFTKEGFICKSEKRNSRMMGRRTGGTSNSKELKMFIGLNENDMKFISNEIPSFYNVMKALLICFRNGIEKPSVLKVMGDFCEYIPNQIKRYFTIEGLNLINKTEFESIKKLKNEKERKDMVYGIFYCLSKIIKYLKGEEEKNGGIEKGIEETKESNNKSNEIYTIFYKVYSDIHECGFNPYLVELLKTLFESFPQTKKQYQDILIKMIRSVLFPDHTRPVSVIPIIQFNPHELTILALQTLYSFEFEEEVSQNIIPLIKETILQFIDSEDIEIRKEVACLGKVLLPKNCKEQDIHRYDIGQVVQILLTHGLSDLDSRIRYSIMSSFDERFDYYLSQAMNIQKLFIGMNDESFKVREQVICVICRLTSYNYMYIIPSFRKIVIQLVSQLRHGVEILSVEESTILIGDVIKTSGSLILPYSESIIEVLMPKLTDELLANSTSLKRNLLIAITELISLGNIEEKYIHQTLDAIVSILHQKGTSNQKTELRLTALESITKLVRSTEVAIGLYQQYPEMVEILMEIMINERSPQIKTAMVSVIGVLGALDPLQYRQHTNTEIVEVEVDESAELQIPMLSNQPDEYYAWTIISTLTKLLKDNTMMSIHVSCINTIGNTLKMMNKNQHQLFYPLVSYIFSTYVNVFKTCPTSIRPDVIKGIGTILPLGDKTIRDTYLPKLIGLIKEYWDGNILGEACAFCHEAASSIKDEFKQYLPTIIPILLSELTKLKYSKKEDDSKVKAPMILKCFLTFAERLNIDDSLYIIIPVFLELLGEQVSVGLRQSIMQYIFRFFTCVNIGEYAGRIIFTTSRLIGNEELSDSVVMGLSTLATKLGAQYFVFHPVVRAAIGEHPCKSGIVLEESIEKIRKSISISSKGGNGGINGGRGSGEQTRIISGSEGNEEINKTEEKKKENYRKIMAVWDSCTQRSKKEEWSDWVEKISLVFLKSNPSRILSRCEDIANNSNSTVARDLFNCTFYSVYIAQDQKQRNELIQKMKIALTQPTITHEAVSLILNLAEFLEHEGLIEPMIEFGDKAKAIGAYAKALHYKEIEFKGLKNKESQVRRNTLGEEETTEEQAIFEELIGLNNQLQRQDAASGLIQMAEKQNQMKLNSTWYAKLGMWHKALNKLEEESKKEEVKMTCLYEMGDWEALDEVASTFWDEENKKWKLNEKEIKKMGSMVAASSFYLDKWERLKAIIETPTFKKVEGFEGTIYNIIISIHLIDEIDKKQGIGESEKIEMREKEIEKVKRIIENNQKQLGNEFSVLATEGYERIYGALTKAEMITELEEIIEMKKKKSSINKEIVMKGWNERLMKSHKDIKTWQKILKMREIISTKHENIKSWISFTGIIDKMGEEGLAFKALNKIAGHKIEGKIEALPKDNFEVGVKYLKLMWKGTKDIKEKENMYQLLEEYKEVINKNSENKEVKSQIYSQLGEWKLNISQNKKEFNKENIEIILKHYQETIKLNKDNYKYWHRWALINFEVVNYYENTKKKSKDEINIIRRNIKEIENNTKEEHNKEKKIQIEEDKIEEIIKKTRKDEREMVTYIETTVKAFVQSLILSNNKQTLQDTLRLLTILFKFGKYYEVEHAISSGINELPIEIWLHVVPQIIARIQSEVGSVRRVTKELLTIIGKAHPQAIVYALTVASKSPNKDRKDVAISIIEKIKKESGHLVQQAMLVSEELVGAAIIPYEMWKEAIELASKEFYINKNFKQMMEVLKPLMKKLEKPNTPRDESFRLIYGKELKEAFEYCLRYEKEYKEGEEIQYIDDLAIAWEIYSRIYNRLNNTINKISRLELSLIAPRLSEAHDLDIAVPGTYKANSGIITIKSIYPILEVIPSKQRPRKLTIIGSNGKEYQYVLKGHEDLRQDERVMQLFGLVNDLLASNSETSKIHLFIHCYDVIPLSPMSGLIGWVPHSITIHQFVKEYRNGKNVQVDTEKILCNRIAPRYDNLTTLQKLEVFERVLKDTKEREMDLANAMWLKSWTSEIWLERRTNFTRSVALMSMVGYILGLGDRHPQNLMLQKFTGDVVHIDFGDCFEVAMNREKFPEKIPFRLTRMIVNAMEVSGIEGTFRMTCENVMTVLRENKDSLMAVLEAFVYDPLIVTILGGKDRTEQTPILSQDNQTTEKEIKVIEKQDEKELFSDNDDMTGAYNTKAGNVTQRVLDKLTGKDFGNEELDVHNQVDKLIQQAISHENLCQCYQGWYPYW

>3535

MTTQMKWSDKFMSLFNELVSETSQTQNDIITKKLHDFVLIVVRDLQGDGVSTFINEISSDLLAVLKKPEVNEKQHSAVINLIDFFITSDIDDSYPRTNAKDIIDCIMKSNNQNVIRNASVVLGKIIRLGPHDLVEKELDIANSYLEREPKVNGALIMQQIGLNNQVICFMNQNKIIESLLKGLYDRSINARTESQKALSILFNLFTSKESNNFDPEALYKQAVVYLKQTNSADLQHGALLLMVSLLRNCKEYMKPKYNEVLQMFLNVKEHKSMVIRKTVITSLPYFAQFDCDSFAPKNEEVIESFQKDRKSIADFAQFEHDELTRIKCEEPFNGIMNYLINSIKSFSDKSIVFTVIGDITREIKYEMYAFVEQVYAKMREIINSKEKKAEILNSIMYCMEQFIINIPVPRVFALIHKTFPTLLELGLSTPLVSLINQSCIIYNNPTYRLQTQKLLLDVICGVLLGKAFIPFGAPEHLKMFNRTIVPTVEGDDQIILALDTLHSFKFKHNIVPVVKECTMKYIDNETKKIRQEAAMLTQVLLPPIDTTKKFISTLDVTEVLEKLLDHGLSDPESEIRITIMKLLDERFDSFLAQSRNVQTLFVVLNDENLKVREYAIKIIGRLTHYNPAFVMPLFRKTIIRLLTQLQFINDLKTDEQVTLLMGQLVKSSGKLTKPYVEPLLNILLPKLEEAINKSHSTICIYILNIIGDLTTIGSDSSQYTPGLMKCIISVLQDKGNSQKKSLRRESALIALGKLVRSIGYVVQPYYDYPELLGLLIELASNERNQDIKIELIKVFGIIGAIDPYKYKLLTETEGANQNDDAETVESLLPISQAGTEEFYAMTVIGLLLNILRDNNLVSGHEQTVKTFVKVICYLNRKTLPYIPQILSLYLKLFKTCTISVRPALIKGLADIMYLIEKNIREYLPEIFTLIQECWDDTTVLPILQLVCEIAKVMHDEFKQYMPQMLSLIISELNKYVFEQNSLVFERIINSIVILAEHIDFEDYLHLVIPSISDLVNEQVHWSIVLPTLKALYKFLQYVNAEEFISRLVLPLTRLLQNEILRDATMDVFCSIADGLGKRFLVYAPTIKFALNKYCPKYNNQRYESLTIKLQDTPDGLVLPIDENEGQNVTISEDLHAQVTSFNQDTTIKPNADINKVIAVWETYRQRRSKEDWFDWFRQIAMVFLKESPSQSLCYCHLLSTDHFPLARDLFNYAFLSMWQDSTDNSQRLTNCLIKVLENNNTPHEIIQTILNLCEFMEREGIKVPIKSLGDYSKKCNAYAKALHYKEQEFLENANINLIEELIGLNNQLQNYDAAAGLIEYTKQIKQVQNNDTELNQTWYEKLGRWQQALSIYERKLQEDINNPELLVGKLNCLHELGDWESLDTTAKTLWKIGDKKAISSARSLYAAALWYLDDWNEFDSIVSEMTESNFENDFFKSINCIHNEEFEEAKALINQQRIALDAELSSLVGEVYERSYSTIAKAQMLAELEEVIACKEGLYNQDAKSILKSAWSEKLINAKADVSIWQKLLKIRSFVLTETENSESWIKFTGLCYKSGKYKLAMKTLDRLAGINIENHLEELQPSKLRIGVQYLKLQWKGAKDLEEKKRLLNVLQNFADIIEERRGDDLNLKAVVCSKLGEWNLYIAQNSNAFNYETIPNILMYYHSTIQYDPESYKYWHHWALINFEIVSYIELDENYNEETLVDYLKVSIEAFVKSLILTKNNQTLQDTLRLLTILFKYGKYQEVEEAIVEGIRALPVDIWLHVIPQIIARIQSNVPAVKRVMTDLLTTIGKKHPQALVYPLTVASKSPSYDRRKTAMSVIEKIRTDSGHLVEQALLVSEELVRIAILWHEAWHEALEEASKEFYVNHSFDGMMAILQPLYDRLEKGGETQNERGFLQSYGKDLRDAFELCIRFKNKGKRASEKDHDIEGAWEIFFRIYKRIHKSINAVAVLELPHVSPRLMEAHDLDIAVPGTYKAQNVNNIIRIKSIAPVLNIIPSKQRPRKLTIVGSNGKEYKYCLKGHEDLRQDERVMQLFGLVNDLLASNSITSTHHLFITCYDVIPLSTMSGLIGWVPHSDTLHQLIKEYRESHNIPVDFEKRLINKICPRFDDLPFLQKVEVFEKVLAESSGMDLANILWLKSSSSESWIDRRTNFTRSVALMSMVGYILGLGDRHPSNLMLQRFTGNVVHIDFGDCFEVAIHREKFPEKIPFRLTRMIVNAMDVSGVEGTFRITCENVMAVLRENKDSLMAVLEAFVYDPLIVRLLGGKDVEDEDDKMNKESQGENYVMKSKAVSVMRRVLEKLTGKDFGNEELNVHDQVDRLIREATSNENLSQSYQGWCPYW

>3536

MELIGGLLPSKSAATPPRAAAVRGARTRALQAMVDSLKVRSPALRAKASRDLLNFVLEELRGSPQNLSAMLAELNAIIHQQVNSQEQSELLAGIAAIDVLLEVELNATQMAIFGSYLRLAIINSDQGSVPAVAAVLGRLVKYGGILTADVVDREAKKALDWLESPDTKEAKKYAAVVVLRELASNAPTIFYNLLDSFTSLIWSAVRDPSQQIREAAAESVRAVLEIMAERDSRHNTIWYDHFYHKSLDGLEQKAHECVHGSLLVIAELFRAQPPLINTKYEMIGTPVLRLRDHPNRNVRQAVMELLPFMARYRVDLFCQKFGAEAVAFLLAVVQRDTERIPLAPHAYAALARLVRIIGGGFMQPWVPQLLASIHQTLLYARGKPHIPEALTLLASLCVVVPEEVSEGQVVLLLDSIFQWPLTLSLSEDLTDISATFPQLTPELQARLLNAISLALCLTPFGSGAAAYASGHDRARAMVAAPPAAGGLPPRTAAYEGLDAVRSLQDKTLLALRMLRRSRFDNHRLGEFVRDAITPFMDNESAAVRREAALTCCKLAINEPPEMDPHVFMDSVYRSPELASYPRIHFNATEDSLRRHEQSTYHRDAVLAEILERLLGAALSDADPDIRFAILASLDNRYDHSLCQPDKLQLLMAAVNDEDRRVRETAIATLGRLSPLNASGVLPVLRQTTLQLLAEVEHSVDSRHLEAAAGMLAALVQSAPAMSQMYAARIVEVLQPRLTDPSPPVVVAVLAALGNLATAVGPPMLRFLPAVAPVVIDTLNDRSSAAKRAAALRCLGQLCQSTGYVSHPYLRHPNLFTNLRAVVQQGANEGPESRQELVRVLGILGAVDPFKLMVLQDEDEEKRKSLRLPSMAAQAANANGGLKANQLRSPDYYTEAALDALVDILRDATLSPFHWAAIQAVMSICTHLGAKCAPFLGLVVPSFLRLFTAESRDNQRYVVSQLGRIVQIGRRGMAPYLTGVMDLLPAAISDAELRPGLLNLCVNVALHLGEEARPCLATTLPVLLRLFREDSSETLLTSRKVLYTLAAFDNHLCDHLPPVVTTLMRAVGSEDVPLLLRVAALKTLRRLIGVEGIEAHASSVIHPIVRLLSSPYQVLKRDAMLVLCSLVYKLGPQYTHYVQLVQRAMTLHRVSHPFYETLVATLLKGGCPLPPPGDEGADLVTLSALREILLPGDTMEADTTAEETDDSLVVVNLNLDEPENRKLVVNQAALRRVVEQAKNKTTKEDWEDWLKKFGVELLQQSPQLPLRYCSALANQYQPLAKELFNVAFVSVWNEMSDKTKEDVERSLEVALMHDSITPEAMQTLLNLAEFMEIHDMPMHLANLAALAEKSQAYAKALRWKEIEFQNSPHSCIDELVSINTQLQRPESALGLLIFAAKEYGVKERHSWNEKLGRWPEALRVFTEELDSLPEGDERAAPLVLGKMRCLAALSEWRGLLAECRRIWRYEVRQYDEEAGTVQQELAPLAVGAAWHLGEWDFMAECVDKLPAATLDSAFYKAVLCVHRQDYQAAQAQIAAARGLLYNEVAALLTESYSRAYDHIVQCQKLTELEEVMQYKLGTPDRREALRAMWLVRLKGAAANVEHWKDLLAVQSLVLRPQEDLEIWMEFVQLCQRMGDHSMERRTLLQLLGEDDDCTTFDMARLVEDEAPYPPRVAIAYLQHAWATGDKLQACLNLERFLARGGAGGADAHFSAKCHVTLGEWEAALLADGVVPPSAGKDMAATLQRYKRALELDPRWYKAWHAWALANEAVMAAKRTAGRGKCTAEVVEHIVCAARGFISSIKYGRNKSVVLQDALRLLTIWFDFGHLPEVERVLEEGLETVNTDTWLLVLPQIVARMHVKSPAIKRLIIKSMTRIGTQSPQALIPPLTVALNSTSLERRAVAAEILGTMKKLYPNLVEQASFVSQELIRCAIIWIEMWHEALEEASKLYFGQRNITGMLNTLAPLHAMMRKTETLREVSFVQSYGRDLQEALEWCRSYQRTGDEQDINQAWELYYSIFKRLNGLLFQQQSTTTLELQCVSPKLHEAKDLELAMPGLVGTHNVTHIASFHPEMTVMPSKQRPRKLTILGSDGVQYKYLLKGHEDLRLDERVMQLFGLVNALLQSDPVSSKLNLIITRYAVIPQSANAGIIGWVDNCDTMHQLVKDFREKKKVFINVEHRHINAMTVQTDADNLPLIKKVELFEYAMESTTGQDLYKVLWLQSPNSEVWLNRRTLYARSLAAMSMVGYILGLGDRHPNNLMIQRVTGKVVHIDFGDCFEVAMNRDKFPERIPFRLTRMLVNAMEVSGIEGNYRFTCEQVMRVLRDNKDSVMAMLEAFAHDPLISWRLVGKDKKADGEVQTDAAPNAIGQLVEEQDNQRLARGESIHPERQTMKCSPVEDTQDVNQRAYSVIKRIADKLSGTEFRSSTLDLPSDDTPFSVAKQVDKLIQQATSVENLCQCYIGWCPFW

>3537

LSPAQAPSAPGQSPSSNLKPSDRQFMPRLLCNLLAVAVSDVDPEIRMVVLGALEARFDPFLCSPECLRLLFMCLNDEVLPNRHAAIDILGRVNRRNPAYIHTSFRVVLLQLINALEYTQDARKEEESALLLSKVIRAAPALIPPYAARILEVLVKKLGDATVPAVTGTLLSTLGELSIVCGASLSDTPRLLPLVIEILMDQSSWVKRIHALRALGNIVVSTGNVISPYTSYPQLLPFLLKALMPDQKVDWESRREIMKLVGIVGAIDPYRHKQIQLQYHEETEQKSKDSQKSRPFGLKGGVIPFSRLTALMEEYYPTVVINCLLSILSDESLAEHHQFAMAGFLPIFRALGIPKCLPYLPPVLPRLLKIAETDNLVLREYSLGQITGLLNYVKQHAIGFMEDLFRLVQNFAHTPEESTLIPTLSLVEEMRVAFHEDFPQFLPWLIPWLVRLVKENYADTRKLSIKVFHCFEVLGEMCEHYLNFVIPCLVETLADSQIGETVAAYQCRTAACNTLCNLSRMLDTRPYVCRIIHPLFRTLVETASAAGPATPAIGIGSVASAAAANAAVGLHHVKDGLEHVMQPPGFHPPGPTAVSGPVATAMNQLHEGATSLLCHVVHGMGPSALRYCKMLQVAVKTHRLNSLVIDILISKVLNGELLDPDTVLELARPHAAVPHSPLTIKIPPVLPVSFALSSGLSNLEEASEGPPPVNATNLTAIYEFSRVTEEDWIAWNNQLALELIRQSPSTAIRHCFQLAALYLPLARELFNAAFVSCYNNLKDGDQVQLLTAVSQALDCRNVPISVLQTLLNFTEFLEHQVTLTGNGAAIFARARQRAADPEMDPDPVSRLLKKLKSLPVHPHATGGDLPAGLPSVAGVAAEDIFDIGVLARNALNCHLYAKALRYKEIQFLKLVEGHRRVHSPQPPQAAQIDPALVKATEDPHVWLQICQELIDCNHRLDLPESSIGKIRFCQNNLQPLGIASVDQFLAGAGDVLEKLNRWDESLKVYERRLEQDPKSIDNLLGVMRSLRAMGEWGRLIQVCEDKWRSADDYTRQLLAPMAGHAAWILSKWNFLAKTNEYMDQAADPIACFLSAVLSVHQGEFQKASTLVHQCRKLLAPSLAAYVSESYDRAYYSVVQLQQLSEVEEVISFKKALELADQARAEDGGLQKKATDLEERREQLRSVWGLRLKGMQLHVQYWEEVLAVQSLALTPHENMNSWLKFSNLARQAGKPKLAQTTLINLLTPELIQSFQTGAFKPSVLLTAKDALPQVVCAYFEHMWGVGEKTEAMALMAEYVTKLHLTGPPGLNALKAQGYLTLGKWQQQLQKDCCSNEGRRKLILQNLQLATQLDPQSYKAWHAWALFNQKIAERLQKKKAHPALLLQFLVCAIRGFVQSIYLCSADISMSVQDLLRLLTIWFNNGTLEEVAAEVNAGITKISVEAWLLVIPQIIARLQSPIPSVSEGVSLLLTRIGQAHPQALIYPVTVAAKDTGAGVAILESMKEHSPSLVAQASLVSDELIRIAILWLDQWCEGIEQANKAYFVGNGSTSADGMMAVLEPLHSMMQNPETETEKEFLHQFGADLMEAHNWCKQYRSSRKQTDMNKAWDLYYHVFRRLNKFIAMTTLQLASVAPRLMSCRDLDLAIPGQYRAGETPPCIRSFAPTLQVIQSKQRPRKLTIHGSDGNEYSFLLKGHEDLRQDQRVMQLFGLVNNFFLYDLETARRDLSIEKYPVVPLSSNVGIIGWLEHAETFHNLLVEYRKAKKIHLTLEHKLMTQMTADYDNLALMQKVEVFEYALENTDGQDLNRVLWLKSPSSQVWLDRRTCYTRSLATMSMVGYILGLGDRHPSNITLDRLTNKVVHIDFGDCFEVAQHREKYPERVPFRLTRMLVNAMEASGIDGTFYTTCERVMKVLRKNKDSLMAMLEAFVHDPLIAWRLAGAVGSAGDHTVANTTTAVVKPTPLTDYDFVFAGDATDLVATSELYSRTTRERMLYSASLMQSAENTNKKALEVVTRIKRKLEGTEFAPHIHLPGKRHSQPAAAPAHTQQRPTGAPE

>3538

NVHDGLSEDPEDLVLSPAVPPEVVEHYVEHAWQWGDRPGAFAQLQRFTAQLPPTNAALRARCRLKLGQWMQHQHPQSYYREDRFPPIMEHLQAATQLDPDNGKCWHAWALLNQRVARQYCLVNGSPPGGPHTIHLANAVHGFVQSVHRSSSSSVMDLDILQQDILRILTIWFDYGQEPEVEAQLRRGFNLVNIDTWLLVIPQIIARIHATQPAVREMISEVLCRIGKVHPQALIYPLTVATKTTVLARKAASQNVLENMREHSATLVQQAQLVSSELIRTAILWAEQWYEALEDASRLYFGEHNVEGMLNVLQPLHEMINHPETKQEMAFQQAFGGDLRDAQACCNAYRSSRNQPDLNQAWDLYYNVFRRISKQLQQMHSLELQFVSPALEAAHDLELCIPGQYRPNAPLVRIAGFAKTLKVIPSKQRPRRTTIMGSDGNEYKFLLKGHEDLRQDERVMQLFGLVNTLLANDRDTSYVDLSIEKYPVIPLSGNAGLIGWLDHAETFHSLIREYRENKKIKINSEHLIMMQMTSDYDHLSLIQKVEVFEHALENTDGQDLNKVLWLHSENSELWLDRRNNYIRSLAAMSMVGYILGLGDRHPSNLMLQKHSRKVVHIDFGDCFEVAMHREKYPEKIPFRLTRMLINAMEVAGVDGTFKHTSVNVMKVLRKNRDSLMALLEAFVHDPLINWRLVDADKLELASQAKRPQQKRIETAYSFDQEGKKDEQDKFSRSVKEKQIRSILGDGCDGADKEEAGKKEKAMAVIKRIRQKLEGTEFPSLLPASLPDARRAAPATPCPTSFTDKVRAEENARSAGPPSEGPPSAGQSVTGPGPGLPSLHSGASQPGGGWPEARQPSSAADDAEVIPLTVAQQVDKLIAAATSHENLCQCYIGWCPFW

>3539

MRWDVQSDFALLDAALLTQTTDTGDRQRLGSKSSVKNSYVSPLIAIHQSIRRKNPVAAAAATTPSGHNQLFMNDDLGTDDLPSSDLFDSTDPWEFTLSPSRTPKYTIKTPSEENILDSERIGDEKSIEECSCRYIGRRSSIQGGTSDICAHCGKRRTLSSGELRRHRSVSVSQQLQTHLSQLQKEAMRQDPQHTQALLQTTPQLRFICAASTDMQVAGHLSDMNLLQRSLFPLVQLIDGQADGSSSARTPGSSLMPGMSEYKDSLLRDRANHQAASELASLMWVLAHEMSQEDFFAVESQVFTEVFGLVHSSDKQLRMAGLAALDALLAAPSADEEKKSIKCANTLSAGLRAANGDFEYLSAVSLALGHMATRTANVDLVECEVSRALEWLGSERSDRRLAACLSLKQLAINAPTTFHSKTSQSTLGQGGSNEFLDQIFQAIRDPQPIVRACAADALSQCLKIIVERRHFSMTGLLCQVYFALMEGMNQDASGKKRPRQAVMATEASQHGSLLVVSTMLTFTGDFMLPRYEEVCRVVLAFRNHDKALIRLEVIRLLPRLANANPKAFGRRFLEDSLTFLIESASKPTAPRVGVDVRPSAYKAIGELILAMMDEETGHVIGSLNAPTIKMISDPKGPGSGTIVELKAQGIVYEKLADIFCLVKAGLQSSCDTPVSSQTMKKADSSKLSLSPVLHCAANLVAALGDLAKPYVPDLIDDMFRAGLSNDLIHCLQSIAQCVPDQQEVIEYRMLQEVSICLAGLRDVYDTLSSTIISGSNIPLTLQPSNGNRVNAPGDRIGKIRIQMSGEPVVVHSLVLSLQTLASFGVTIGKTKASGSAVSLLPFVENVASRYLAHPSSEVRRAAALTCCALLLPYEFAYNSRLGGHTGVIVDNVLDSLLRVAVSDPSGSVRVCVVQALDSRYDSFLCQRHHLSELFLLLQDENLAVRTAGLRLLGRLAAINPATILPVMRRFLIDSIIEMQCGIYNGRGREEATRLCVVFLKAKPLQRLVQPVLPSLIDALPFHDAAPPRLAAASLEALGELALASGVALKPWIKDLVPRVLEIMADQSSASKQRTSLRTLGQISRATGYVIKPYLDHPGLLLQATDMLPATKRAPWSLRREVIRTLGICGALDPDMQLSSALKARKGGAVGGAYFEEADFRDQGQMDQEEGYDHFAHIDLEDKQRINGSQELFIENDENLPAYLFMHEQYAMFAQPMSELIPAKRISPADEDFYPTVAVQALMRIFKDPNLTVHHSLVIQAVMYIFKSMGLGCVSYLHKVVPHMILSIRTCVSNSLRESILKQLARLSLIVREHLRPYVSDIFDVAESLWSSRHLATVFNLISNIAVGVPDAFHKFIPRLIRRLLSTFDDLQVADWTENEKRNPLNRGREGTERLHLLLFSLSSLKGVLGDYLHILIPALLKLADGLASLGLNLDAGIPEKSLSDLSVMVFRTVSILIESKNAANNRVPSTYEGHSQFDFKFRESSEKGLPSRVVQPITRLLRDKPPHSIPVCLVMVETLCVCARLIGLLTWLKLYDKEVRNALCEWQVGYRIAAMNLATFTLDVVGDDHPRCLQVYDDFLQSLKNVDIRSTENSFDFATRFTLTPNAVYISSIDNTPEAYEQQIMLPSPAGATKHKVNQRNLERAWDVSQRSSRDDWDEWMRRFSIQLLREAPAPALRSTASLAQAYQPLARELFSAAFACCWNELSNPFKANLVHALKTAFVADVSPEILQALLNLAEFMEYDSAGGLPIEIPVLADLSLKCRAYGKALHYKEREFSLNGGTSCIESLISINRKLDLHEAALGILKASSLKDARPGDTPSSSLSTRFSRHHARDMFYSVIWSTQEHQQSKTDAADMTAKEELWLARLGSWTDALAVYENKLSRNPDDPEAILGCMRCLGAVGEWRKVLDLAHENWGIMSGTYHKLSDGVEHGHTNPRSKRKALRICAQSAWRLGQWDKLEKFATQLVGSSSPQPNQPSYSSVDFDGAFYSAVLHVHRKEWGPAAEAIDGARKAMDSRLTALMAESYSRAYPSMVTAQNLAEMEEIIEFHKLEEHALSSNNQHPANRPDAERARARLLAVWRDRLAGCRMDADVHASILAVRSLVVKPEEEVEATLTLSNLSRHAQQFKFAERVLLDSLEALKADLDGPVFGLGLPPSLRTLHDFSSVPTAALPNIVDNLLAGDFRNIVASYGSTHEQWSKKLLAEAGGLQRLDIQHRLYFAYVKHLWYTERKDEAMSRLSCFCDVMEMISRCEQVADSSLRVASWLELGEWKLSQEAPPALLSSPLQLETLTIFRRATTLHNCGYKAWHTWALLNLRIVLEQRERESSGKISVAHSGRNLRNHVVAAVKGFVNAISLGTKKWSASVQQDLLNVLTCLFQYGKLEDVATVINECIGNVAIEAWLGVLPQLLARIHIVEPSIRAVLHPLLIRLGEKHPQALMYPLSVLLNSPVAERKASAESLMNSVKAHSSALVEEAQMVSSELIRVAILWLEMWHEGLEDASRLYFGEGNVTGMLDLLFPLHEMLEKGAETRKETEYLSNYGQDLSKAFAYIKEYSRLATMDGEDSSRRDGGNVRVSDEAETAMNRACVQFCVWFTGQFGYSNSSVIKDIYYIVFRKINKQLPALTKLELNQCSPSLSKARNLELGVPGTYRIDGSYIKIDRVQSEVEVITSKQRPRKITMRGSDGKNYLFLLKGHEDLRQDERVMQLFGLVNALLVRDLQTKKHDLKIQRYAISALSHNCGVVGWVPHSDTLHSLIRDYRLSKKLPLNMENREMTRIAPDYDLLTVMQKVEVFSEALRKTPGGGNDLGEILWLKSTNSEEWLDRRTKFTRSLAVMSMVGYILGLGDRHPSNLMLDKVSGRILHIDFGDCFEVAMNREKFPEKVPFRLTRMLIKAMEVSGVEGSYRSTCERTMSVLRDSRDSLVTMLEAFVYDPLISWRLADLSNSGSSHKPSTHHTGAEIGPRSLSDDVPMEMIRTGRPRVQSIRELADEDIDDDDIYGIDQQARGSFPNDPSQSNNDTPGRSQARSMQMYANIQNWAASLTTDARIASIAAERSEQASVEGSLARSRIERSMKQRELLSLLRGNDGVALEEALNEKALKVIRRVQDKLTGTDFPDCEGVPLDVADQVQRLIVQATSCENLCQLFIGWCSFW

>3540

MRWDVQSDFALLDAALLTQTSDTADRQRSSSKASVNSYVSPLIAIHQSIRRKNPVAAAAASTPSGPNQLFMNDDLRADDLSSSDLFDSTDPWESTLSPSRAPKYTIKTLSEENIVDSEGMGDEKSIEECSCRYIGRRSSIQGGTSDICAHCGKRRTMSSGELRRHRSVSVSQQLQTHLSQLQKEAMRQDPQHAQALLQTTPQLRFICPASTDMQAAGHLSDMNLLQRSLIPLVQLMDGQADGSSMARTPGSPLMPGMSEYKDSLLRDRANHQAASELASLMWVLAHEMSQEDFFAVESQVFTEVFGLVHSSDKQLRMAGLAALDALLAAPSADEEKKSIKCANTLSAGLRAANGDFEYLSAVSLALGHMATRTANVDLVECEVSRALEWLSSERSDRRLAACLSLKQLAINAPTTFHSKTSQSTLGQGGSNEFLDQIFQAIRDPQPIVRACAADALSQCLKIIVERKHFSMTGLLCQVYFALMEGLDQDFTRKKRPRQAVMAAEASQHGSLLVVSTMLTLTGDFMLPRYEEVCRAVLACRYHDKALIRLEVIRLLPRLANANPKVFGRRFLEDALTFLIESASNPTAPRVGVDVRPSAYKAIGELILAMMDEETGQVIGSLNAPTIKMISDPKGPASGTIVELKAQGIVYEKLPDIFYLVKAGLKSSYDIPVSSQTMKKGDSLKLSLSPVLHCAANLVAALGDLAKPYVPDLIDDMFRAGLSNDLIRCLQSIAQCVPDQQEVIEDRMLQEVSICLAGLRDVYDPLSSSIISGSNFPLTMQTSNGNRMSAPGKGTDKIQIQMSGEPAVVHSLVLSLQTLASFGGTIRKTKASGSAVPLLPFVENVASRYLAHPSSEVRRAAALTCCALLLPFELAYKSRLGGHTGVIVDNVLESLLRVAVSDPSGSVRICVVQALDSRYDSFLCQRHHLSELFVLLQDENFAVRTAGLRLLGRLAAINPATILPVMRRFLIDSIVEMQCGIYSGRGREEATRLCVVFLKAKPLQRLVQPVLPSLIDALPFHGAAPPRLASASLEALGELALASGVALKPWLKDLVPRVLEIMEDQSSASKQRTSLRTLGQIASATGYVIKPYLDHPGLLLQATDILPATKRAPWSLRREVIRTLGIFGALDPDMQLSAASKARKGGAVGGAYFEEADFRDQGQMDQDEGNDHLSPTNPEEKQRINGTQEYLIENDENLPAYLFMHEQYAMFAQPMSELIPAKRISPADEDFYPTVAVQALMRIFKDPNLTVHHSLVIQAVMYIFKSMGLGCVSYLHKVVPHMISSIRTCVSNSLRESILKQLARLSLIVREHLRPYVADIFDVAESLWSSRHLATVFNLISNIAVGVPDAFHKFIPRLIRRLLSTFDDLQVADWTESEKRNPLNRGKEGTERLQLLLFSLSSLKGVLSDYLHILIPALLKLADALASLGLNLDAGIPEKTLNDLSVMAFRTISILIESKNAANNRVPSTYKGHSQYDVRFRESSEKGLPSRVVQPITRLLRDKPPHSIPVCLVMVETLCVCARLIGVSTWLKLYDKEVRHALFEWQVGYRIAAMNLATFTLDVVGDDHPRCLQVYDDFLQSFKNIDDRSAANAFNFATRFTSTPNAIYISSIDDTPEAYEQQMILSSPAGATKHKVNQRNLQRAWDVSQRSSRDDWDEWMRRFSIQLLREAPAPALRSTASLAQAYQPLARELFSAAFACCWNELSNPFKANLVHALKTAFVADVSPEILQALLNLAEFMEYDSSGGLPIEIPVLADLSLKCRAYGKALHYKEREYSQGGGSSCIESLISINRKLDLHEAALGILKASTMKDVRPDDIPSTSLSTRFSRHHARDMFYSVIWSTEEHQQSKTDAADMTAKEELWLARLGSWTDALAVYENKLSRNPNDPEAILGCMRCLGAVGEWRKVLELAHENWGIMSGTLNKLGDGVGHAHTNPRSKRKALRICAQSAWRLGQWDKLEKFATQLVGSSSHQPHQPIYSSVDYDGAFYSAVLHVHRKEWGPAAEAIDGARKAMDSRLTALMAESYSRAYPSMVTAQTLAEMEEIIEFHKLEEHALSSDNQHPANRPDAERARARLLSVWRDRLAGCRMDADVHASILAVRSLVVRPEEEVEATLTLSNLSRQAQQFKYAERVLLDSLEALNADLDGPVFGLGLPPSLRTSHDYSSVPAAALPNIVDNLLAGDFKNIVLNYGSTHEQWSKKLVAEAGGLQRLDIQHRLYFAYVKHLWYTERKDEAMARLSCFCDVLEMISSCEQVTDSSLRVASWLELGEWKLSQEAPPALLSRPLQLDTLTIFRRATTLHNCGYKAWHTWALLNLRIVLEQRERESSGKLSVTHLGRNLRNHVVAAVKGFVNAISLGTKKWSASVQQDLLNVLTCLFQYGKLEDVATVINECIGNVAIEAWLGVLPQLLARIHIVDPSIRAVLHPLLIRLGEKHPQALMYPLSVLLNSPVAERKASAENLMNSVKAHSSALVEEAQMVSSELIRVAILWLEMWHEGLEDASRLYFGEGNVTGMLDLLFPLHEMLEKGAETRKEIEYLNNFGQDLSKAFSYIKEYSRLASMDGEDPSRRAGGNARVSDEAETAMNRACVQFCVWSTGQLGCSNLFIIKDIYYIVFRKINKQLPALTKLELNQCSPALSKARNLELSVPGTYRIDGSYIKIDRVQSEVEVITSKQRPRKITMRGSDGKNYLFLLKGHEDLRQDERVMQLFGLVNALLVRDLQTKKHDLKIQRYAISALSHNCGVVGWVPHSDTLHSLIRDYRLSKKLPLNMENREMVRIAPDYDLLTVMQKVEVFSEALRKTPGGGNDLGEILWLKSTNSEEWLDRRTKFTRSLAVMSMVGYILGLGDRHPSNLMLDKVSGRILHIDFGDCFEVAMNREKFPEKVPFRLTRMLIKAMEVSGVEGSYRSTCERTMSVLRDSRDSLVTMLEAFVYDPLISWRLADLSNSGSSHKPSTHHTGAEIGPGSLSDGVPLEMIRTGRPRVQSIRELADEDIDDDDVYGIDQHARGSFSNDPTQNNNDAPGRSRARSMQMYANIQNWAASLTTDARIASIAAERSEQASVEGSLARSRIERSMKQREILSLLHGNDGVALEEVLNEKALKVIRRVQDKLTGTDFPDCEGEPLDVADQVQRLIVQATSCENLCQLFIGWCSFW

>3541

MCKSENNSKASSQLLNLVRQVYQSKEPKSRWEAAVRFREALEQRIKDSRGGYSRSSSTVNLNVLEEIYPNLANLLSSNDLLERVTGVVCLNCLLDLHGEPYREKVQRTYNGLRTVIQAIDSIPLSPRTTELFLFSCSTGILRTDTKDSSEMDFMQHDLVEVDLIVTTAAALGRLARIDGLLVNRCVETEIVRCFERMTVKSHENVHSIVAKSLSIAILKEIISNCPLFFYSRRTEFIKQIWSGLWDTRSIIRLDSAVCLQVFIHKVLKRYASEMEFLVGKLVDTAIGILQSLNSEDGDERDSFSDCDSECEAAELSPEKLAKLHGCLLTLEELLKDQDTRELLREHFHLLCRFMLGFRSLTSFSIRSTLGSCLTALASLDPELFCDPSKGYLDRAVHLFKEFNELYVEVNNPGYHILLFGKLAYGVGIEHAKSWIDVVFKMITEALMQKSESTHGPNKGPLAESIFTCISLLASSIRDNDPRFVAVFEELIDLMFAHQLSPALVESLGCIAKYFPFFLVEIQDRLILAIDRIFLQLNNHEMNALPSSRVNNKSYNKKHSMVRKFDETRSWSKEELFCLELALNTLSSFEFEGHFFSPLIREKVAELMDCGVSHIRRLAMFACCKMIASWIPYCLSSSSCSPLIKRVRDSLSRDVKALTEQILTSSITDPDSSIRLAALEGLSDERFSWHVSQPEALEKLVIVLYDENIPTRKAALELCGRLSKYNPGVILPVLRRLLCQILRIVEYNGSSEVLHTLRSNACVLLASLVRETSSLVEPYVEPILTVLLWRMKHILRALSSSSVLDSRALNAQTEIYSAIGNLAVCFGQSFSLMKLLPDVISTLIMTVEDELLDAKAKVAATNALCLVVRYSKIVLTPYSSHPELLSKLMRIIQMDTNAEVRQAVERLLGTIGAIDPKEVEYTSLNETEEEETVSTLESLSMSFNHNNFRKSPLLRSSDDHSYSHQLENSPNSMRSHSKASFSDSHLSHSSGSETAFRSGLRSSSFEEGLLSPAVLEIAFSKKSLVSRLDRPYSKSDDYFYAAALDCLHRIVLDPKSAQYHREALHAMASIVKSFHSTCMWLVPVTLSKLLWLLRPAGTRIVRNTSILERNITEEVSKNKIGISPKRRTGSVESADSLSGSSPTSSPVISSAALYVTHGSSSSSSSSATHLDPHLREFVLRLLCSLIEFAKQNVRSFARDIVATVRYYWERDPSVSELKVIFEIVRLLSCILSDEFYEYIPAFAACITNTLRSDSSNDREITIHMLAMLEFFGPHLDDYPSILREILKTLLEVNLNVNTRKKILDFIVAIIPHIQVTEIGSFLMRLTVNILRGSDAKEFVSIITHIFYLVGVASPMLLSLFVDDILSILCRIEGTLDSQFLELIQEEYGMEFGHRITDEESIRRRNRNYSMHMTSSTSNSLDADPFENEDIDEFNNTSTEFNRNLYMQMNEDALKSSWEIGRRTTSEDWEEWMSKFSVALFRESPSVAIRSCSRLAEVYTPMLRELFNAAFLSCWISLQQSAQHHLARVISEAMHSENIPLDALQNLLSLIEYMEHDEKPLPFNVRSLAKMAFRCGAFAKALRYKEAEYAEVENSQSALSAVAGPHGLISIYDKLNQQDSAVGILEDVEMRFGIERRQEWYEKLQQWDEALAAYDGNLNFKETRINKFDTLNITSLSSKPLLSSQPQPFLDDPLEEPFVILSESDRLFGILRCLEELGYWRREEQLCEEMWECASESDKRKIAEQGGASVALTLQKWNEFEERIPYISNNKLLKVCCEAILQMKKKNYDMAESLIDSSRKYLDIRLKARAAEGYERAYYDVLNAERIVELEEACAFLRNPTSSMKRNLAELWSARLKGLPYNYFYWYQLIRTRSLVFEPQETMQQWIQFSKLCRKAQRYPMAANAIRFLLAYPEAMPDEQPSSWDVELAMKDSHPEVSFALLKHLFETDDRVKAFKMLRMEASSEIRDDEGNLAARRYLKLAKWARQLEEEKALLQLQGQDDEDDFDEKLDLSAIEMVQRLDELSARHISPKAVLHYATKATELSPDWFKTWHVWACINAELIETAGRSFPKKGSAYSQEQSREDDPKSTNDRSKLLVIAAITGFFRAVSLSSGSASRLQDILRLLTLWFRYGHIQEVNISVNSGVAAAEVDTWLEVIPQLIARLHVNNQAVRSAIRSLLIRIGRRHPQALVYPLHVATKSTNKIRRETAEEVVHSIRFHSPTLVEQAEMVSKELLRVAILWQELWHEGLEEASRLYFGEGNSEGMLEILEPLHEMIDNGAETIAEMEFLRDFGQDLRDAASFCERYKSSGKESDMNQAWEIYYQVFRKINKQVPQMTSLHLANVSSKLLNVRFLELAVPGTYRSEENSIEKNSIVRIAAIDPTLQVISSKQRPRRLTMYGSDGKEYTFLLKGHEDLRQDERVMQLFGLVNDLLSQNIKTNSNQCKIKRFSVIPLSPNTGLIGWVPNCYTVHSIIREYREQRKIVLNIEHRLMLQVAPDYDELTLPMKLEAFEHAISNTNGFDISKSMFLKSKNSEVWLAHRTMYIRSLATMSMVGFILGLGDRHPSNLLMEKGSGRIIHIDFGDCFEVAMLREKFPEKVPFRLTRMLVNAMEICGVEGKFRHSCEQVMQVLRNHRDSLMAVLEAFVHDPLINWRLLMDNENVPLIETVPMMKDHQGLNEKALKEAYELSMQGGVSLSASKSLLMGRTGISLSQMARVHSGESLKDEDVFLSSSKRPEYSFSLRPGETPAEVVLRDLERKKGTEAIETMNEELNKRALSVIHRVQCKLTGNDYVEYEVHSVPAQVSRLIADAMNIENLCQCYVGWCPFW

>3542

MLPVQDISGRWQSQVVSLLKQVYNSETRSARVEAARRFKETLEQKWRETVVPLGNSSKASSLHVNLAEELTTQLNNLVNSSDVRERIAAVVCLDVLMELRGESYREKIQRTHNGLRSVLQNVERCLPSAMAVEKLFASQPFIFEAQEEDVDMVSLESSTVSKHVADELELLRVSSKALGHLARTGGALAYRCVESEVSRAFERLSFRDHSDARFSLARLSAVFVLKEIAENAPAFFYGQRTSFVRLIWSALWDPKQQVRFYAALALRAFIQTVVRRDAAEMATLVGKLLQVSIDTLSPITKEEKSEFDTIEIRKLSSQEKPSTDIKTEKIHGSLLCLAELLRDEISRERLHGRFEEICEVVLYYQSTQDIFIRTEVVICLPLLASLDPTTFCHSTSDYLRRSHQVLVSFHKLYLENERLPAQHLVAYGQLAEAIGSEYISPFVNQVFCMIEDILPKKDVRKELQRNAKDVFSCIRMLAETLEFRTFSYTNHFHDLLDHMFAHGLSSAMVKALNSIGKKIPELLPQIQERLLDSVSLILSRGSPFGNEPASKANMRKKYSRTSAKDLVSMESGSLQEFNHSSILIERALETVANFDFRGRFLSSFVRENIFDYMNSSLPSWRRLAVSACCRLLAASSVYALEQQFLFRQQKLRRVYSNNLVKEIASLISRVLISSVADRDEQIRLAALEGLKDPRFSKYLAQPHLLNKLLVCLYDESLSVKRVAISLCGNLSFLNPALVFPVIRRRLAQLLITLRCEGKTFALRSSRDAAAVLLSIMVRDAPRIVWPYVVPILRVLIIRLRETLFGSNLSFVWDSGGEEAETVMYAAVGNVASNVGPNLEDLKRMLPELLNLLVGAVQDQTSEPKTKTAALNAFTLIVQNTSCVISPYNNFPSLLPSLLHAIRTETDSQVRQGVEQLLGTLGAIDPKEYKYGVSFDFNQTEHLWKESSPVGPSYIAERSIVYPGACEDYNSYAISPLEGGILGTTWEMPLSPKVSHSVAPRNYFGNENKDRLFSQRIINMALSSETLVSRLNHPYTANEEYFPSAALDALHRVISDSKLSHHHREAVNAITCIVQSLGPKCNEILPFTLSKLLWTLRPPGMGIMREMNSNLKTSSSMGDLRRGLGSRSGGKYGSCDSLESLSGIGISGSSVGSPPRGSLPYVTTSTLTSTNTASNDPNLREYVFKALAEVVHVARQHVRPYSWDIISICRYYWEREPLSSELRTIVILVERTCLALMDEFAVHLPTILPCIVATLYTDTSANRENALPVLHLLDVMGNHIEDYAFMLIPIVSKMACDGSASTSARLETLGILTKLITRVPIREVASQVIHSLLNALEQPDAKDVSLLITKIFGLIAERNTHVFSLFLDTIVHVLYSLSSPIDALLLQHLRKNRVDVSMFTEQGKVTSNLRQASLNRISSLSSLNRSGSSSSLTSLEGSTGPASSGGNLEKRRHHVNQRSLKNAWNLGRRTTAEDWEEWLNKFSNGLFRESGSPSIRSCARLAEVYTPLMQDLFNAAFLSCWTELAPNYQASLVETLLAALSSPSLPLDALQTLLSLAEFMEHDEKPLPIDVRRLATMAYRCGAYAKALRYKEAEYAQVTQPQTAKSAVAGEHGLISIYNNLLQQESAVGALKDAEYRFGIRRREEWFEKLQRWDEALIAYEKGSNAMSTNEEEKSSSEPERLLSFQKPVLSLQPQPSPDDAYEEPFAVLSEWDRKLGMIRCLNELGEWRRMESLCQELWQSVDTEKRYVLSYEGAASVAFNLDLWDEFEERVKYLQKNSFKWALYNALLAVHQKQYDEALEFVKHGRRILDGRLRARAAEGYSRAYLDIVNAERLVEIEESIKYLKNPTIAYRNQLASLWKARLQGIQSSYFYWYRILRVRCLVFHPFDSMEEWIKFTSLCRKSGRLPMSAESLRWLLSPNEALHSDDVDSWDLNEALKDAHPEIAFALLKHVYVAGRKMKAFSYLKQLAASHVSRQPKREEDEEDHLAARLYLKLAKWGKNLQDEMTPLRSRSQSVTEFFDSDNSSSDEAQVDIPEMSLHNISADSILQFAKKATEMNPNWYKTWHVWASLNAELVSSHGEVLSKRKKKHLLGTSSHFYRSDSFRDEPKELVIQAINGFFRTLSLCSETAIRLQDILRLLTLWFRYGGMTEVSASINAGIAAAEVDLWLDVIPQLFARLHSPNQAVRSTVRSLMVRIGRAHPQALVYPLHVAAKSTNKVRREAAEEILNALRLHSATLVEQAETVSKELVRVAILWHEMWHEGLEEASRLYFGEHNVEGMLEVLEPLHAMLELGPETAREAAFIKEFGRDLAEAAEWCRRFKASGKESDMNQAWDLYYHVFRRINKQLPSMTSLDLAHVSPKLLRASNLELAIPGTYSPSFESNQVSIVRIAGFSPTVQVINSKQRPRRLIVYGSDGREHAFLLKGHEDLRQDERVMQLFGLVNELLSQNASTNSKALMIKRFSVVPLSPNTGLIGWVPGCDTLHSLIREFREQRKILLNVEHRLMLQMAPDYDNLTLIQKVEVFEYALSNTTGADLSRVLWLKSRNSEMWLDKRTTYTRSLATMSMVGYVLGLGDRHPSNLMLERNTGRVIHIDFGDCFEVAMLREKFPEKIPFRLTRMLVNAMEVCGIEGYFRHTCESVMSVLRDNKDSLMAMLEAFVHDPLINWRLLGTAEDIIVGRHVGYSQESSGKGVNGLEKTKSGRSMKTFAFSMADNTSRARFIVEEEGDQGNAHLLSKNKHLLQGPYGFSLSDIARIQGEKVGTEEEEDSMWNTNSRRGFSLRPGETPVEIRHRDMERLQGSEAIENMNEAVNRRALAVIRRVHNKLTGKDFDDRQQVGWTVSSQVDRLIVEAMKVENLCQCYIGWCAFW

>3543

MSTDAITSSRFYPYLADLKSIDPNIYTRAAMAIRMQVEKDSRQMLADPRTLFISDLMSFIRNLATSSSYEDRLASLWVMRELIQSRPTISMIPLTYFEEIALELVNDPSEVIILTNCVFIGRLINAEGHGIDFVPKLFVISIDKIKSGDSTKVYTGMLLFNDLTEVETSFAIDISSEEFLEPIWHVITETRNSNIKEVGFKAINKFISYQKKATKHDVLVRLITMIRAALDGKNISEQLIALHLLLNMLNLSVKDKSIADYFQENYSSFFSLVANIFRSKTSEGRMLVASIISLFAKLDPERFCTTERLQMILTFYKKMIPTQQELFIPLGEFVIVIGEKHVKPIFNDLINLITSSFKSQNFTKAGSILPPEPLKCIQYITKGAPNLVQKHVTSILECIFSVGLCESLTSALQDLSANLHNRLLDIQGLLLYLVANELGGINLFLSNAYGVIDGSEDYYLVLTENQSQAVADGHDSGVLDSQTSIIPGTRPSTTQATTHVEDTRTNEDAIELAHDLSITGLKEESEQCDFKNSLSVYQTVTTSAGITALYKLSDRIATALGTSKLRPRRHSTSEAILLAFQALYDFDFSLFDLTIFAADYLTRYLRNDSSTLRIKAAQTILKLMSEDHFEKIRRGKHSQYKSMTIKAEDCHSWAGSTLGEAPTPSGGATIIPPAVVSNQSFATTISPKTRHIRTHSSSHLDDMSSTSFCINQDCILSQDILKSSDLGTDGFFNSGPYRHMLISDTLRQIIIIGTTDIDPEVRMSIIQALTTTNRYDIYLVHESSLRTLFIALYDDNIEIRILAVKLIGRLCSLNPALVTPEIRHLILVLLTELRLTTENNKRATSNELIVKIFKFCGSIVLPYVPMVYSAVMGLISKDCDDEYLLITSLTTLAELFTIGSTSMVHCLAEVIPLLITCIKEETSKTLRLTTTRCFVRIVEATSFVVFPWFIYHDLFTIVFGILKNDTSQEIRLEAAKLITTIGAIDPIFYQNGKPDLSALVPPTNLTCKCQSTDVFSLDYIMDESNIWVCHKGEGGAAQEASIKSHGKAVVSPITGHVVENVYIMKLDSGSEVSANVSDFELTLNVIMSTLYSILSESTLDMYHYDAVVVFRTIIQTECTAISLPMVPKIISLILENIQNCKVFMQETMLRELILIISSIGHHAHSYVSDIFDIIEVLWANQQLIELILALLEEVSIVLDLSLGNYGSQFFLKFVGLFTVLKSSQEISDSAKKLLIRACRSVVALSSLFSNHIHLIVDLLCSGIRKQKLGKELRFALLDTLSRLTICTNLSRFGASIIHPILEVIMEADPCTSVEHNKNANPEKGLNPMNIVSYKAFEVLHFICAQFGVSVTLYIPVITECLLKVGFTSGLLDSIFSLLLRREEVDPVTFYRLICEWKKKQAKKFSEYTSLASLLAMYETPVTQDTLLYMESISNKVPGDDKDAMILVVKDINFDDIGSVINYLHTSSTWFGDDMRPLPAGAIGVHSLSIQENDMLNKHVTIPQQIWRADTCKKNEEWRHWLSNLSLTLLQCSPLKCLRACHKLATSYPRVARDLFNYSFLACLSETYPSNNEHRNYIVESIRSVFLSQNCPLDVLQILLDLAEFWENRSREFSTTFSHNFLGEVAERCHAYTRSLRYKETEYAQHPIEAVGRLIAINYSLGYEETAIGTLRVECKRLNFISTNLIYDILLRCLYTGLDDVFTMMQREGGLEPGAAVNCSAESTLERIINKVVSKIVTKALTEAKKISNLNQSIPQQLVSYGSIGGIFVEGRPVMSGPEGSVLQVDYNEIGIYLSALARRMLKSKLTYQYPTFLMYEENLFQSVLKTLRYKAQGHMQDLYGPSPKWFENVCLWDKALSGYNRQIDQLKLSLKSNTSEARTLLPMLVNLTKDKIRCLFALADYSAVQEEADQLLNYLSSESKEEAGLTTVKHTLIKGFQADIATYAAWSLLEQDQEDVEIWLRVLPEDNIDGTIIRATSYIKANKMLEAHKLLCKLRLRIDPDLIGLSAESYTRAYNLCILLQTITELQNIISYKLALNGLKLDAPSTLIKEGGNPYENIFETTLISFNPPPGFADTEKERLRLMWSRKISMIRWDCESWEALLRVRRLIIFPIEDQEVWIRFSVLCLQSGRVQLARKTLETFINSSSVHHIKVSKSRGLYTLLGLTSFGTPRRYLKHGSGDVSTGGSVTPIGSALGAEMGVVQDLSAVGSSLAKYLLKDGVVKDAFTAQQHVALPTSDVLSIASHSVAKPTLQMIANASTHQGQSIEKETPLSIVGQQCDDAKRNAQRDLFNALIPPLNHVYPHVLYAYCKHLWVSSNENVLEKLVAFTHLIRLCNDVSIQKTEPKLVCRMLIRLGAWYSDLLTPLDHKSCSMIGTISPSEGYVNIPVQDTVTSCSAIWNSIVNNCDADASVCSTTYEATEHSAERLELSSASTLDKAVAKLVDNILLAYDELSNVSLEECNQYNTVGMSRIGLIALEWFQVAADRDETSNHIWKCISHTTYAIVNSFAAYIDKLNDRPGVDGGLQIVTRRSSVDATSDAVGSTSEALSQSVQDSDRPLVCPEHLGFVQNRQVLLEKAKLEYLVESIIAHFKCIETGRSIEALPTTLRLMTLWFRYGSYDEVETEIINGLAAVPINIWLDVIPQLIARLHSPQHKIRLLVHQLLVLIGTEHPQALIYPLVVASKSTILNRRVESLSIVDQIRRTNEAMILESLNINKELQRVAILWPEIAYDLITQASTAFCEEPRDLVKFFNYACQLQLLASRTPETQSESVFIQMFSSQLQAAWDSCRQFPVTNDLVCLNIVMKHYKEIYDRIHSELPFLNTIDLLQTSPLLYNISNTNLCVPGSYHPARAITTIVKFYQKVFVIRSKQRPRKVGLVASDGEYYAFLLKGHEDLRQDERVMQLFTLINNLLMNDSYCSRRGLMVMKYPITPLSQNSGLLYWVPGCDTIISLIKEFRQLKRIHLETEMQQIKYRAPAFMNMALHYKVDAFKYMLTTSDAIDLCRSLWNKASSAEDWLLKRMNYTRSIAVSSIVGYILGLGDRHPANLMIERTTGMVLHIDYGDCFEVAMHREQLPEKVPFRLTSIMMKAFESCGTEGSFRMTGEATMSILRSNKDSLVSVLETFIYDPLMSMKVLSGENTMGGKENIDGNTEFYGTRRPVQESYLEGNERNPKAVKIVQRIYDKLTGYDIKYIDRSLSVSEQIQSLIHSATNAENLCQLYIGWCPYW

>3544

MAPPTNVHASNRALPNTTEKILVDLRSRNEHTQRKAMGELRSAVEMESRQMSREEFSGFITELNKRIFDLVKSDDPCEKMGGINAMDSLIDFECEETSTLITRFANYLRLVLPCNDVGTMIMASKVLGHLAQSGGTLTADFVEFEMKRALEGLNTSKRSEHQKLASLLVCKELATNAPTLFFMHVPSYLKSIWVGIRDQKQIIREAAIESLRACMTIIAERDFVVRKKWFTAVYEEAEKSFHTNSIEGVHGSLLVMGELLSLPNIAPDIPASATLSAMWETTMAYKDHRDALIRRTVISILPTVAKIDEEEFRAAYAEDCIKYLLATLKRGNESSAAFVSLGAIVKVAGAWVIESFVDDVMNLVQDGITPTPRKPFCFEAPQCLASLTTVDSSHVVLNERMPELIEQMLAGSEGNLTPALISSLSQIGKSMPLLLPDIQMKLLDSISFTLQRTPYQPSGTPNSRRIVPLRGGVMSVHSGTAKGRQNAVIQALHALTSFDFTGNQLCEFVKDCVSQYLDDQSRMVRLTAAQTCSNLLLRPGETVPKRGRTAIVVSEILERLLVTAVSDSDPQIRVIVLECLGPRFDYFLAQSHCIDSLFVALNDEVFNVQEAAIRILTRLTLCNPAHVFPGLRKVLIQLLTALEFGGEDKKEKSTRLLQHVILAGHHLVKPYVSSILDAILPKLQDSNPTVAANVLNTVGVLSHVGSYDVLKRKDDIFYLMMEALQDQSNTNKRRVATKTLGLLVQNTGFVVEPYKTYPNLLSIMLKRLQAEQTNEIRQELLKVMGILGAVDPHILKMRQTRNSSGLPITFSAEKWRREEELPGPSTDEYYPTVAVSKLVKILNDQSLSSYHQMVIQAVMFIFRTLGLKCATLLPQMMDPILNLLRSHQQQLRDFLFQQLGLLVSIVKQHIRPYLPGIFELIHEFWNQQSLQPQMLFLIGEITSALKDEFKVYVSPLIPKLLGALNKENARSRPMTCQKVMYALELMDANLELYLDVIIPAIVAIAEQEDSKLDVRVAAIQWIGRMSLRLNISDFASKIIHPLVRILEGPIDSLYKPAIITICALMNALGQRFFLFEPMLTKIIVRKRVNDGVGRYQMLLERLRSGEVLTLDDYPTEELGTLGSNSSSLESVLDTGQVRKIAVNQPNLKRAWEASHRSTKEDWADWMRRFAVELLRESPSPALRSCAALAQVYHPLARELFNAAFLSCWMELYESYRDSLMRSLEIAFSPEKSPNIPPEVLQTLLSLAEFMDHDEKPLPIKIPKLGAVAEKCQAYAKALHYKEMEFLGSPHTAIQSLISINNFLGHTEAANGLLVHAQKCLNLEVKESWYEKLHKWEEALDAYRQRLKTEQAAEAQEGCMRCLQALGEWEQLKDMTEAAWSSALPDARSRIAPLGAAAACQIGDVEQLAMYIEAMDPKSVEGCMYKAMFLVHGDALDEAQEKLEVTRRLLDTQLTALVSESYERAYKAVVITQQVTELEEVLQFKRAKAMDALPACEHIRHMWTQRLFGAQANVEVWQSLLAIHSLVIPPREYSEAWLKYASLCRKTGRSSLCYKALTKLLNYNEQLQQLESPFSPSVDPEIGFAWLKYIWYTGQREQALVTLQEFIRAQRGSHTLQARCHLKVGMWTQELHESPQQNDFGLILSAYRNATILNKNWYKAWHHWALFNFDIVSYNENKQQRNRAGGIPYVVAAVNGFIRSIALGVSNARGHVQQDILRLLTLWFRYGSIAEVERALAEGFNILPIETWLDVIPQIIARINSPDAVIRNSIHNLLIRIGKAHPQALIYPVTVASKSHIQARRDAAKKILSEMEGFAPLLVEQAKLVSKELIRVAILWIEMWHEALEEASRLYFAEGNIDGMFAVLRPMHELLQRGAQTQRELEFQQKLGQNLDEAKEFCDKYCQSKQEKDLQKAWENYYNVFRTINKLLGRITSIELEHASPELKAAKDLELSIPGQYRPGEPIVSIQSVEETLSIITSKQRPRKCTMIGSNGKPYQYLLKGHEDLRQDERVMQLFGLVNTLLHNDRECSRRDLQIVRYSVIPLSPNSGLIEWVPDCDTLHILVREYRENRKILLNIENRLMLAMAPDFENLPVIGKVEVFQHALTSTTGQDLNKVLWLKSNNAEDWLLRRTNYTRSLALMSMVGYILGLGDRHPSNLMLHKYTGKIVHIDFGDCFEVAMQREKFPEKVPFRLTRMLVNAMEVSGIEGTFRFTCESVMKVLRANKVRMFKTERRDNGARGKQEKRRRRGQKSGGIGMEDEGG

>3545

MSSSEQPPQWADALNKILSDLKNKKEEDRIKASKNLRNYVITQSREMSNENFTKFMQDVNVIIFELVNSNGIAEKIGGILAIDELIDVDYDENAAKITKLANYLRIALSTNDQTVMQMASKALGRLARSSGTLTAECVEFEVTRALEWLSGDRYENRRHASVLVLKELAQNAPTLFYVHAANFVDLIWVALRDTKLAIREGAVEALRACLELIAERESRLRLQWYQKIYDESQKAFKQNGSPEAIHGSLITLGELLRNTGDFMYNRFKDVCDTILRYKEHRDKLVKKTVITLLPRLAIFCPKDFVHHHLNTCMAHLLGALRNQNERSTAFIALGEIALAVGGSIKPYLDRIVVMIKSALATKGKQFNPEVLTCIKPGTPSQYRKSSAPFQGVSIGYTLPNPDTDPAITALALRTLGSFDFSHHNLLEFVRDTVSTFLDDDNPDIRREAAITCAVLMVKPGEPAPTRGHTAVIVGEVLEKLLVVGIADPNPSIRKTVLSSLDIRFDHHLAQAENLRSLFIALNDEVFEIRELAISVIGRLTIRNPAYVMPSLRKTLIQLLTELEFSGDSRNKEESARLLGHLIGASEKLIKPYVEPILKALLPKLRDSNPRVASCVLAALGELSVVGGEEMSQHIDQLLPLIIDTLQDQSSTSKREVALKTLGQLASSTGYVIKPFAKYPTLLDILLNAIKTERNSNIRREVIKVLGILGALDPYKHKMNELGQRREDPKIDDKSTSGSINDLVSISPSSEDYYPTVAITALMKILRDPSLSIYHTNVIQAVMFIFKSLGLKCIPFLPQIMPPFLHVMNSCEPGFREFLFQQLVQLVPIVKQHIRDYLVDIFALIEKYWNSNLLNLINLVEEISSALNDEFKVYLPNLIPQMLNVLHTDRTLKRLPTSKVLKALEVFGTNLDDYLHLVIPAVVKLFEQVDVTPQVRILAIQTIGRLCKKLNFSDYASRIIHPLARVLDVDGTNELKDDALQTLCALVYQLGSDYAIFIPMVGKVLAKREIQCNNYEVLVSKLLKNQQLVGTGNGDGDTQGIRHPDTASEENTTTVSPEIGFKKLKANEQHLKNAWETSQRSTKEDWVEWIRRFSVELLRESPSPALRSCLSLAQDYHPLVRELFNAGFVSCWTELHEQYQDELVTSLETALLSPNIPPEILQTLLNLAEFMELHEKPLPIDIRTLGALAEKCHAYAKALHYKEIEFQQSANSTIEALISINNQLQQPEAAIGILIYAQKNHSVELKESWYEKLRRWEDALAAYEKKQKDDPNSIENTLGIMRCLHALGEWERLSQLTSDVWKNANEGTRLSIAPLASAASWNLSSWESMDEYVKAMSQDTIEGSFYRAILEVHKDNYDNAQKHIEHARSLVDTELSALLGESYNRAYKMVIRLQQLSELEEIIEYKKCGGEGNDRRQMIKNTWKTRLRGCQHNVDIWQSVLAVHSLVISPHEELDMWLKFIGLCRKSSRIGLAQKTLSMLMGKDPTTHQFGGILPNTHPRITFAYIKQLWSAGAKQPAFERLRTFVQALKDTDDLPLQGRAHLKLGEWQLELGDTLNESSIPHIIASFRSATDCDPNWYKAWHSWALINFEVVSHYEQNGGTQEQIASHLLPAIHSFFRSIALAPDQSLQDTLRLLTLWFKHGAQKDVEASLMTGFNTISIDTWLQVIPQLIARIHAPVLPVRRLLHELLDSIGKEHPQALVYPLTVATKSQSPARLAAAKAIMDKMRKHTNLVDQALPVSQELVRSAILWHEMWYEGLEDASRQYFGEHNPDAMLATLAPLHQILEKGPETTSETSFIHAFGRDLQEALEWSKKYERSRKDGDLNQAWDLYYQVFRRIYKQLPQMISLELQYISPKLINSSDMDLVVPGTYRAGEPIIRIQSFSPVLSVIPSKQRPRKLTIIGSDGLEYTFLLKGHEDLRQDERVMQLFGLVNTSLSANHETAKSHLSIRRFSVIPLSPNSGLIGWVPHSDTLHALIRDYRESSKILLNIEHRLMLQMCSDYDNLTLLQKVEVFEYALESTTGQDLHKVLWLKSRNSEIWLDRRTNYTRSLAVMSMVGYILGLGDRHPSNLMLDRHTGRILHIDFGDCFEVAMHREKYPEKIPFRLTRMLINAMEVSGIEGNFRLTCEAVMTVLRNNKESLMAVLEAFVHDPLINWRLLTPNHEKETKIKQDLIDTESPEPSLTTSPVHRQPRGHQRVDEQVEAEVVPEALNERALVVINRVNKKLTGRDFSNETLEVAEQVQKLIDQATSHEHLCQCYVGWCAFCLNNNYFKGYVDLSTKDIQTYQSN

>3546

MYNSELIKLFDQLKKARNETSERLAQELQQYYLKNSSQYDEIFQYLQKIINSSDLQEKYNGLLGLDQISYVIQENQYMNFVNKFHTNVFQQFQENADQRYLKKTAEVYGRLLKLGGTRIQNILHTYITNAIDWLFNKNFKENRKYAGLLALKELLEQAPFVTFKSIIQTEKYRENIWNLIKNKSIQLREAALMFFEVYIKLISLKESDFQIKEYTTFYNDIQQSVKQKDDDVIIGNISVLKIILSYSNPDVFQTQQFYDMCEYVTSKRSSSSEQIQKAVIEALPILSKYKKQYFIENFFQKTIEFLILKIAQNRTSRNYNLDITISYYDTLNKICQCLDGGVYTKLDEAQQNIVFKNIELVTRTIRQDLIDRKPYIPSRILCLQNILKVFNAQYQQLIIDEDSLINNILFNGLYPQSIDFLKQIQKTYFFQKDQQQNSKELTKKKIDLTQSIQYKLLLCIITVLSKQKNTIPQSLLSRKQERKEEHQNFLKALNQINEQNKQNDEKSIANAIQTLSTFNFSKYENELANFVKDNVLDYLDDKNNKIIRKAAAKAGCLLYVKKKREQQISKNIMYEILEKFMSVAISDPEDEIRQTMLSSLNENFDQYLNEPNYLKKLFLCVNDSNINVQQLALTILCRLSKHNPSDIVPFLKKTLFEFLSQLAFDEMQNEKQMINLLSSLTCLIKNGPDIVKSHSESIAHILLNFLNDPNMTNNMIPELLKAFSQLGNLGEQNMLLYIDEVVPIILQAMQDKSSTSKREAAVKSFVDIIKCTGFVVLPYYKYPNFLEIILGLMKNEVNLEMRQQCMRLIGCLGAIDNFYYKKVVDKLKNKFKLINNQDNNEIVNSIVSKQLKKKFKFFEKFKNAKQLQGSYNLTKQLHQYYENIILLENFKQKKLFLKLANIQAESNQNQTNNKNSVNGTIFAKKDSKLRDQIQIDKINNRQENDEEIKVLLQAPTVISLNDEDYYSRVTIKALLKILCDNSLSQHHDLTVNTLTCIIGVLKNRTKNFLDIIIPVFTKIISQENLRQSLLDLIQKIIQHCGVHYDQAYIDPILNIFLEYGKEVKHQKICFQILENLIDQQKINLRHKMEPIIRLINNVINKPQLDNQAEIVDFSKKMIKIYMKLAELLDSNLHLIIPFLCQFINKNHSNAHSEVKVEIVKLFKTLALYCPSTIQFLSLIVDSILNYLNDGKNDQVTNNQILDTIVVFIYKYRNQFLVYLPKVNIIIKNCNIHHNQYSRCIEIFLNNGNLDDVSSQLENEYPNIVEQIQRQPPYTTESNNGIYRKMVNEKFIKVFDTSSLTSKEDWNQWIRKTSVELLKESPNLILSPCHQLAEVYEELQTELYNISFACVWSFLRDKDKEFIINQLTKAINPQHQDADNIPINILQTILNLAEFMQHDKDGLQIDNSTLGDLAERCMAYAKALYYREHEFETANEETIEILISLYTNLGQREAANGLLNIVKNQLGMNQNMSWYERLHQWENALDDYRVRQLNQVGQNFFVPKMRCLNALSDWEKLIKSTEDEKNMENRKQVMHLAANAAMHLGKKKLVFFFFFFYFIGKWDQLELYTEQVNDDYPDKNFWNAALCIQKAQFEDARQYISESILKLDSQVSGLLLESYNRAYDSILRLQQLFEMQEIIEIKEFEDKVKQAQKENREGLTKIYLQQDLEQKKKQLQDVWIDRLNGNPKDIDTWQNILSVRQLLLSKVDNMDTQEIYLQIQNQYFFKNRWLKFCRLALKGNQMKICQKSLDELMKEQNPDPGAIEFFDYPPKVVLANLECSYQMCTAKEQQTFERMDEFLQKNQNQIDKKLTAKMFLKMGNWLRDKAEDLSQPEIIEKIERFYQSSKNFKADYYKTWHHYALLNFDAINIAHNSLGENQEEKKQQYIKNALEGFMKSISLGASQEHKSPYLFQDSLRLLSIIFEYGELEEVNNKFLEDYKQIDIRAWIEVVPQIIARISISKKDIQRLLHQLLIHIANHHPQALIYPLTVACKSKTQGRQQAANQIISDIKTHSPTLVNQAMLISAELNRTAILFKEQWHEGIKEAWESFLQGKNSHQHLIKVLLGLHEMMTMQPESLSEISFHQNFGAEVFEAEAWLQRYNTTENEICLCQAFDIYYKIYQRINHQLESLDYVYLENVSPKLLETKNCEISIPGLYKPSRPVIKISCFQPKLDVLFSKMHPRKLFIYGSDSKEYHFLLKGREDIRQDERVMQLFALVNRLLHNNPETEKKALNITRYSVIPLSINTGIIGWVHNCDTLQSLIKEYRKAYNIRENPEMSLMDQFCTNYSALPLPNKVEIFRHIIENTKGEDLKKILWLKSPNSEIWLERRTNYTRSLATMSIAGYILGLGDRHPSNIMLQRQTGKIVHIDFGDCFEVAMRREKFPERVPFRLTRMLVNAMEACGIVGNYRNTCELVMKVIRENKESLIAVLEAFVYDPLINWRLIAISGDDQGNNKIAETKGNKNQDKDQQKNSLIENDQQQNYLGSIIQQGTSLAIKKAMKQTQKNKTEDKPPLSFKKEKEQQYYEDEEKEQPQEIINKKALEVMDRIKKKLNGKDFKENEQLSYVEQVNKLINQATSHENICQAYMGWCPFW

>3547
[truncated: 242,455 more chars]
